# GRK3 deficiency elicits brain immune activation and psychosis

**DOI:** 10.1038/s41380-021-01106-0

**Published:** 2021-05-12

**Authors:** Carl M. Sellgren, Sophie Imbeault, Markus K. Larsson, Alfredo Oliveros, Ida A. K. Nilsson, Simone Codeluppi, Funda Orhan, Maria Bhat, Maximilian Tufvesson-Alm, Jessica Gracias, Magdalena E. Kegel, Yiran Zheng, Anthi Faka, Marie Svedberg, Susan B. Powell, Sorana Caldwell, Mary E. Kamenski, Marquis P. Vawter, Anton Schulmann, Michel Goiny, Camilla I. Svensson, Tomas Hökfelt, Martin Schalling, Lilly Schwieler, Simon Cervenka, Doo-Sup Choi, Mikael Landén, Göran Engberg, Sophie Erhardt

**Affiliations:** 1 Department of Physiology & Pharmacology, Karolinska Institutet, Stockholm, Sweden; 2 Centre for Psychiatry Research, Department of Clinical Neuroscience, Karolinska Institutet, Stockholm & Stockholm Health Care Services, Region Stockholm, Sweden; 3 Department of Molecular Pharmacology and Experimental Therapeutics, Mayo Clinic College of Medicine, Rochester, MN USA; 4 Translational Psychiatry, Department of Molecular Medicine and Surgery, Karolinska Institutet, Stockholm, Sweden; 5 Center for Molecular Medicine, Karolinska University Hospital, Stockholm, Sweden; 6 Department of Medical Biochemistry and Biophysics, Karolinska Institutet, Stockholm, Sweden; 7 Research and Development, Innovative Medicines, Personalised Healthcare and Biomarkers, Translational Science Centre, Science for Life Laboratory, AstraZeneca, Solna, Sweden; 8 Department of Clinical Neuroscience, Karolinska Institutet, Stockholm, Sweden; 9 Department of Psychiatry, University of California San Diego, La Jolla, CA USA; 10 Functional Genomics Laboratory, Department of Psychiatry and Human Behavior, University of California Irvine School of Medicine, Irvine, CA USA; 11 Human Genetics Branch, National Institute of Mental Health, Bethesda, MD USA; 12 Department of Neuroscience, Karolinska Institutet, Stockholm, Sweden; 13 Department of Psychiatry and Psychology, Mayo Clinic College of Medicine, Rochester, MN USA; 14 Institute of Neuroscience and Physiology, University of Gothenburg, Gothenburg, Sweden; 15 Department of Medical Epidemiology and Biostatistics, Karolinska Institutet, Stockholm, Sweden

**Keywords:** Neuroscience, Schizophrenia

## Abstract

The G protein-coupled receptor kinase (GRK) family member protein GRK3 has been linked to the pathophysiology of schizophrenia and bipolar disorder. Expression, as well as protein levels, of GRK3 are reduced in post-mortem prefrontal cortex of schizophrenia subjects. Here, we investigate functional behavior and neurotransmission related to immune activation and psychosis using mice lacking functional Grk3 and utilizing a variety of methods, including behavioral, biochemical, electrophysiological, molecular, and imaging methods. Compared to wildtype controls, the *Grk3*^−/−^ mice show a number of aberrations linked to psychosis, including elevated brain levels of IL-1β, increased turnover of kynurenic acid (KYNA), hyper-responsiveness to D-amphetamine, elevated spontaneous firing of midbrain dopamine neurons, and disruption in prepulse inhibition. Analyzing human genetic data, we observe a link between psychotic features in bipolar disorder, decreased *GRK* expression, and increased concentration of CSF KYNA. Taken together, our data suggest that *Grk3*^−/−^ mice show face and construct validity relating to the psychosis phenotype with glial activation and would be suitable for translational studies of novel immunomodulatory agents in psychotic disorders.

## Introduction

G protein-coupled receptors (GPCRs) transmit information from a variety of ligands, including neurotransmitters such as gamma-aminobutyric acid (GABA), glutamate, and monoamines. Phosphorylation of GPCRs by GPCR kinases (GRKs) is a primary regulatory mechanism accounting for receptor desensitization/turnover and is increasingly recognized to control a vast array of physiological processes. Importantly, recent data also suggest that GRKs can regulate non-GPCR targets in both a phosphorylation-dependent and -independent manner [1] as well as diverse biological processes, such as cell growth and proliferation, transcriptional processes, and immune modulation [2]. Thus, GRK dysfunction may be involved in the pathophysiology of a broad range of neurological and psychiatric diseases. The GRK family comprises a number of isoforms designated GRK1—GRK7 [1], and each protein controls signaling pathways of the brain with some degree of specificity regarding region- or cell-specific functions [1]. In particular, the large multi-domain proteins GRK2 and GRK3 serve as molecular scaffolds via direct protein-protein interactions that do not involve kinase activity [3]. Although GRK3, also known as beta-adrenergic receptor kinase 2 (ADRBK2), is the least abundant of all GRKs, it is widely expressed in the brain, including the limbic regions [4, 5]. Like other GRKs, GRK3 is involved in the desensitization of a variety of receptors, including adrenergic, muscarinic, histaminic, dopaminergic, and opioid receptors [1]. Moreover, GRK3 is linked to reward mechanisms [6–8], as well as learning and memory [9]. The serendipitous discovery in the late 1950s that blocking dopamine D2 receptors mitigates psychotic symptoms remains the cornerstone of treating psychosis in schizophrenia and bipolar disorder. However, genetic, immunohistochemical, imaging, epidemiological and biochemical studies implicate brain immune activation in the pathophysiology of both diseases [10–15]. Indeed, increased cerebrospinal fluid (CSF) concentrations of interleukin (IL)-6 is found in chronic schizophrenia [16, 17] and increased CSF IL-1β in first-episode schizophrenia [10]. Subjects with bipolar disorder [11] also display increased CSF IL-1β and the highest levels were observed in patients with a history of psychosis [18]. In line with these data, postmortem studies report upregulated mRNA levels of IL-1β, IL-6, IL-8, and tumor necrosis factor (TNF)-α in the brain of both patients with schizophrenia and bipolar disorder [12, 19]. Given the largely shared heritability [20], symptomatology [21], and overlapping immune-related biomarkers, it is likely that such immune-related pathophysiological mechanisms are at least partly shared between the disorders. GRK3 is highly expressed in immune cells and can critically regulate immune-related behavior [22–24], while decreased RNA expression and protein levels have been observed in postmortem brain tissue obtained from schizophrenia patients [25] and polymorphisms in the GRK3 promoter have been suggested to increase the risk of bipolar disorder [26–28]. We here set out to examine the functional brain impact of *Grk3* deletion in mice, hypothesizing that this model would be suitable for translational studies of novel immunomodulatory agents in psychotic disorders.

## Materials and methods

### Animals

Adult male C57Bl/6J (*Grk3*^+/+^) and *Grk3*^−/−^ mice (backbred to at least N10 prior to deposition at Jackson) were obtained from the Jackson Laboratory (Bar Harbor, ME, USA) and kept on a 12 h light-dark cycle with food and water available *ad libitum*. Experiments were approved by and performed in accordance with the guidelines of the Ethical Committee of Northern Stockholm, Sweden and the American Association for the Accreditation of Laboratory Animal Care. All efforts were made to minimize the number of animals used and their suffering. Throughout all types of experiments, mice were randomly selected from different groups to limit the impact of bias. Unless otherwise stated, the investigator was not blinded.

### Behavioral testing

Learning and memory were assessed using the continuous alternations paradigm in the Y-maze (*Grk3*^+/+^ *n* = 19, *Grk3*^−/−^ *n* = 20), novel object location memory (*Grk3*^+/+^ *n* = 12, *Grk3*^−/−^ *n* = 14), novel object recognition (*Grk3*^+/+^ *n* = 13, *Grk3*^−/−^ *n* = 13), rewarded alternations in the T-maze (*Grk3*^+/+^ *n* = 7, *Grk3*^−/−^ *n* = 8), and the Morris Water Maze (*Grk3*^+/+^ *n* = 13, *Grk3*^−/−^ *n* = 16). Anxiety was tested in the light-dark box (*Grk3*^+/+^ *n* = 13, *Grk3*^−/−^ *n* = 13) and elevated-plus maze (*Grk3*^+/+^ *n* = 12, *Grk3*^−/−^ *n* = 13). The open-field paradigm was used to assess locomotion, rearing and center activity (*Grk3*^+/+^ *n* = 20, *Grk3*^−/−^ *n* = 25). Psychosis-like phenotypes were examined using novelty-induced hypermotility (*Grk3*^+/+^ *n* = 20, *Grk3*^−/−^ *n* = 25), prepulse inhibition (PPI) (*Grk3*^+/+^ *n* = 19, *Grk3*^−/−^ *n* = 20; vehicle *n* = 8 and IL-1β *n* = 9), and D-amphetamine-induced hypermotility (*Grk3*^+/+^ sal *n* = 7, *Grk3*^+/+^ D-amph *n* = 13 *Grk3*^−/−^ sal *n* = 8, *Grk3*^−/−^ D-amph *n* = 17). For details see Supplementary Information and Supplementary Table S1.

### Analysis of striatal dopamine and hippocampal kynurenic acid (KYNA)

Probe implantation and dialysis procedures for dopamine analysis in mice were similar to those previously described [29]. Analysis of KYNA (*Grk3*^+/+^ *n* = 8, *Grk3*^−/−^ *n* = 7), by HPLC with fluorescence detection, and dopamine (*Grk3*^+/+^ *n* = 5, *Grk3*^−/−^ *n* = 5), by HPLC coupled to electrochemical detection, was performed as previously described [30, 31]. For details see Supplementary Information.

### Intracerebroventricular interleukin (IL)-1β infusion

IL-1β (0.5 ng) or vehicle was infused intracerebroventricularly (ICV) in wildtype C57Bl/6J mice over 8 min (4 μl infusion volume) plus 5 min to minimize backflow (vehicle *n* = 8 and IL-1β *n* = 9). Mice recovered in their home cage and were sacrificed 6 h post-infusion. Brain tissues were harvested and stored at −80 °C. For ICV IL-1β behavioral experiments (vehicle *n* = 8 and IL-1β *n* = 9), surgery was performed as described previously [32]. For details see Supplementary Information.

### In vivo recordings in the ventral tegmental area

Electrophysiological recordings of ventral tegmental area (VTA) dopamine neurons in mice (*Grk3*^+/+^ *n* = 7, *Grk3*^−/−^ *n* = 6) were performed as described previously [33]. For details see Supplementary Information.

### Analysis of tryptophan, kynurenine, KYNA, and quinolinic acid

Detection of tryptophan, kynurenine, quinolinic acid (QUIN), and KYNA was performed using a Waters Xevo TQ-S triple quadrupole MS in mice (*Grk3*^+/+^ *n* = 8, *Grk3*^−/−^ *n* = 7). For details see Supplementary Information.

### Membrane fractionation and western blot for P2X7R

Brain tissue (*Grk3*^+/+^ *n* = 5 and *Grk3*^−/−^ *n* = 6) was homogenized, and membrane fractionation performed using sequential centrifugation steps. Proteins from the internal and plasma membrane fractions were separated by SDS-PAGE and transferred onto nitrocellulose membranes. β-actin was used as a protein-loading control and all signal intensity measurements were made using Quantity One software (Bio-Rad Laboratories, Hercules, CA, USA) normalized to β-actin expression. For details see Supplementary Information.

### Cytokine measurements and qPCR

IL-1β, IL-6, KC/GRO (IL-8), IL-10, IL-12p70, interferon (INF)-γ, and (TNF-α were analyzed in the mouse hippocampus (*Grk3*^+/+^ *n* = 5, *Grk3*^−/−^ *n* = 5) and detected by multiplex sandwich enzyme-linked immunosorbent assay (Discovery assays, MesoScale, Gaithersburg, MD, USA) on a SECTOR® Imager 2400 instrument (http://www.mesoscale.com). For details regarding RNA isolation and quantitative PCR (*Grk3*^+/+^ *n* = 6, *Grk3*^−/−^ *n* = 5) see Supplementary Information.

### Immunohistochemistry and autoradiography

Brains were collected from anesthetized mice following transcardial perfusion. Post-fixed and sucrose cryoprotected brains were frozen with CO_2_ and cryosectioned at 14 μm thickness. Sections (*Grk3*^+/+^ *n* = 7, *Grk3*^−/−^ *n* = 8) were pretreated with 0.03 % H_2_O_2_, incubated with anti-glial fibrillary acidic protein (GFAP), anti-aldehyde dehydrogenase 1 family, member L1 (Aldh1L1), anti-ionized calcium-binding adapter 1 (IBA-1), or anti-CD11b and processed with the TSA-plus Fluorescein System (PerkinElmer Life Science, Waltham, MA, USA). The immunolabeling was analyzed on a Nikon Eclipse E600 Fluorescence microscope and quantified in micrographs using ImageJ Software (National Institutes of Health, Bethesda, MD, USA) and custom python script [34]. Autoradiography (*Grk3*^+/+^ *n* = 10, *Grk3*^−/−^ *n* = 8) was performed and quantified as described in Supplementary Information.

### Label-free neuroproteomics

Mice (*Grk3*^+/+^ *n* = 4, *Grk3*^−/−^ *n* = 4) were anesthetized with isoflurane and brains immediately removed, washed in ice-cold 1x PBS, and the prefrontal cortex (PFC) was subsequently dissected, snap-frozen on dry ice and stored at −80 °C until analysis. Tissue was prepared and label-free proteomics was performed as previously described [35, 36]. To determine the effects of *GRK3* deletion on canonical pathways and disease processes we utilized Ingenuity Pathway Analysis (IPA), see also Supplementary Information.

### Human studies

Data were collected from euthymic bipolar disorder patients enrolled in a long-term follow-up program at a bipolar outpatient unit at the Northern Stockholm psychiatric clinic. The Regional Ethical Review Boards in Stockholm approved the study. After complete description of the study, written informed consent was obtained from all subjects. The diagnostic procedure has been outlined in detail previously [18]. The general population controls were randomly selected by Statistics Sweden and underwent the same clinical evaluations as the patients. Subjects were genotyped using the Affymetrix 6.0 array (Santa Clara, CA, USA) at the Broad Institute in Boston, MA, USA. Procedures for genotyping and the quality control (QC) have been provided in prior publications [37]. Details of cerebrospinal fluid (CSF) collection and analysis have been outlined elsewhere [38]. Expression quantitative trait loci (eQTL) data was obtained from the HapMap2 sample (for details see Supplementary Information).

### Statistics

All analyses were performed using the software programs Prism® 7 for Mac OS X (GraphPad Software, Inc. La Jolla, CA, USA), or IBM SPSS Statistics 22 for Mac OS X (IBM SPSS Inc., Chicago, IL, USA). Gaussian distribution was tested using D’Agostino & Pearson normality test and parametric tests were used where appropriate. All tests were two-tailed with alpha set to 0.05. Further information about statistical tests and sample sizes are described in the figure legends. Unless otherwise stated, as no pre-specified effect sizes were available, sample sizes were chosen to reflect at least 80% power, assuming effect sizes such as in similar and previous experiments.

## Results

### Attentional deficits and psychosis-like behavioral phenotypes

**Fig. 1:**
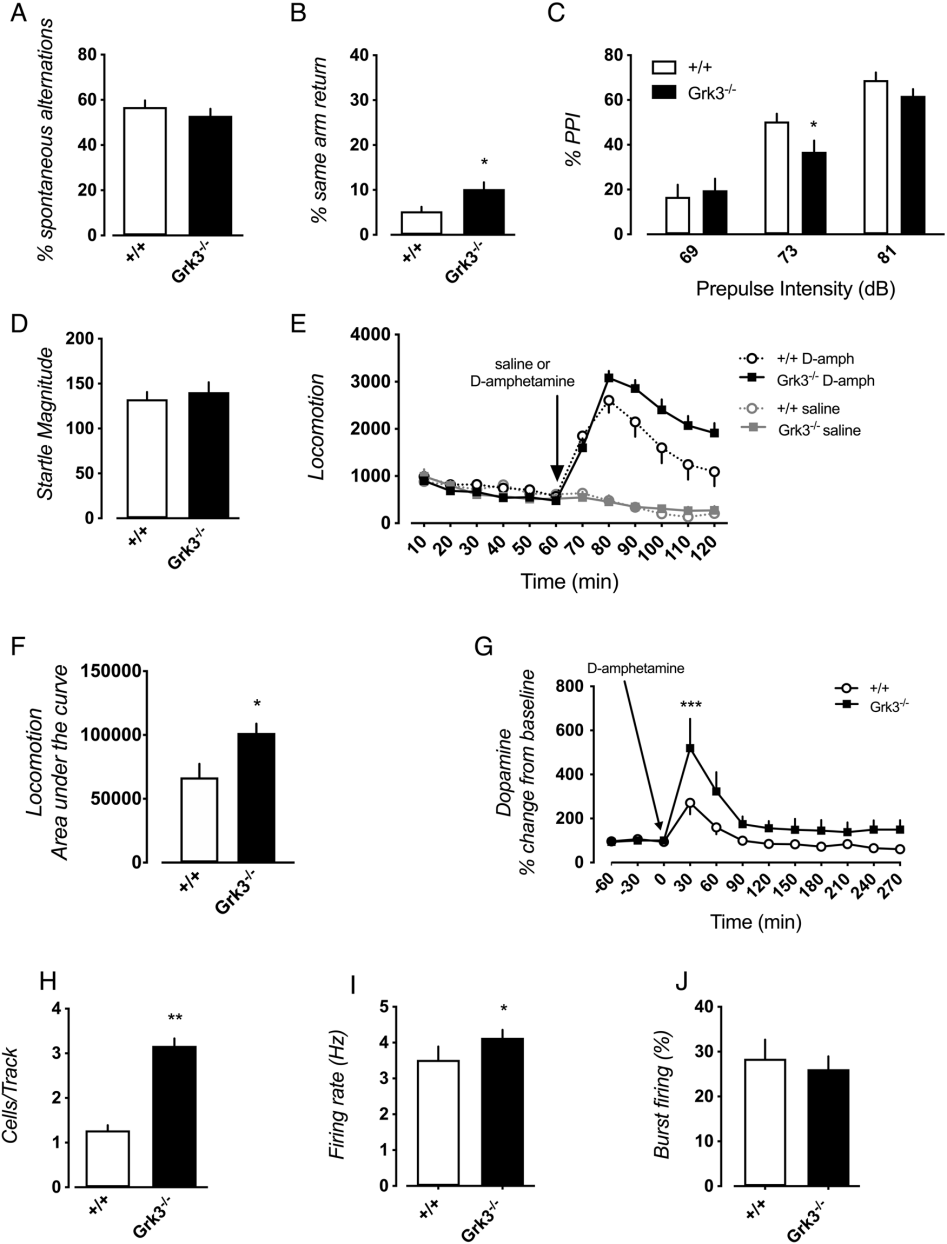
*Grk3*^−/−^ mice exhibit deficits in working memory, psychosis-like behaviors, and altered dopaminergic transmission. Working memory was assessed in *Grk3*^+/+^ (*n* = 19) and *Grk3*^−/−^ (*n* = 20) mice by calculating the (**A**) % spontaneous alternations (*P* = 0.37) and (**B**) the % same arm return (*P* = 0.011). **C** The percent prepulse inhibition was influenced by genotype (effect of: prepulse intensity F(2,74) = 134.90, *P* < 0.0010; genotype F(1,37) = 1.27, *P* = 0.27; interaction F(2,74) = 4.20, *P* = 0.019) with deficits observed in *Grk3*^−/−^ mice (*n* = 20) as compared to *Grk3*^+/+^ mice at 73 dB (*n* = 19; *P = 0.030 post hoc Fisher’s LSD), while (**D**) the startle magnitude was similar (*P* = 0.75). **E** *Grk3*^−/−^ mice (*n* = 17) showed significantly higher sensitivity to the locomotor effects of D-amphetamine (5 mg/kg) than *Grk3*^+/+^ mice (*n* = 13) (effect of: time F_(5,140)_ = 18.54, *P* < 0.0010; genotype F_(1,28)_ = 4.94, *P* = 0.034; interaction F_(5,140)_ = 2.95, *P* = 0.014). **F** Calculation of the area under the curve following injection was made in order to measure the net effect of D-amphetamine administration on locomotor response. *Grk3*^−/−^ mice (*n* = 17) showed a stronger response to amphetamine with enhanced locomotion compared to *Grk3*^+/+^ mice (*n* = 13) (Mann–Whitney U-test, *P* = 0.039). **G** Accumulation of striatal dopamine induced by amphetamine (2 mg/kg) as measured by in vivo microdialysis was increased in *Grk3*^−/−^ mice (*n* = 5) compared to *Grk3*^+/+^ mice (*n* = 5) 30 min post infusion (effect of: time F(11,88)=13.52, P < 0.0010; genotype F(1,8) = 4.08, *P* = 0.078; interaction F(11,88) = 2.22, *P* = 0.020; ***P = 0.00080 post hoc Bonferroni). **H** In vivo electrophysiology of dopaminergic cells in the VTA shows increased number of detectable cells per track in *Grk3*^−/−^ mice (*n* = 6) compared to *Grk3*^+/+^ mice (*n* = 7; *P* = 0.0012), (**I**) as well as increased firing rate (*Grk3*^+/+^ *n* = 35 cells, *Grk3*^−/−^ *n* = 78 cells; *P* = 0.043) although no significant changes in (**J**) burst firing could be detected (*Grk3*^+/+^ *n* = 35 cells, *Grk3*^−/−^ *n* = 78 cells; *P* = 0.56). Group comparisons in (**A**), (**B**), (**D**), (**G**), (**H**), and (**I**) were performed using a Mann–Whitney *U*-test. Group comparisons in (**C**) were performed using a repeated measures 2-way ANOVA followed by Fisher’s LSD post hoc test. Group comparisons in (**E**) and (**F**) were performed using a repeated measures 2-way ANOVA followed by Bonferroni’s post hoc test. All error bars represent standard error of means (SEM). All tests were two-tailed. *P < 0.05, and **P < 0.01, ***P < 0.001.

Loss of Grk3 function showed a number of behavioral aberrations in mice. In the Y-maze, *Grk3*^−/−^ mice displayed normal numbers of spontaneous alternations (Fig. 1A), but a significantly increased number of same arm returns (Fig. 1B), possibly indicating attentional deficits^39^. However, we observed no deficits in the rewarded alternations T-maze, novel object recognition, novel object location memory, or long-term memory when tested in the Morris water maze (Supplementary Fig. 1). *Grk3*^−/−^ mice did not exhibit any overt signs of anxiety using the light-dark box paradigm, elevated plus-maze, or open-field. However, a slight decline in explorative behavior was noted in the *Grk3*^−/−^ mice with decreased peripheral rearing in the open-field and increased corner time at the beginning of the open-field testing session (Supplementary Fig. 2).

*Grk3*^−/−^ mice were also tested for PPI, a murine model of sensorimotor gating deficits observed in schizophrenia patients [40]. Dependent on stimulus strength, *Grk3*^−/−^ mice displayed disrupted PPI compared to wildtype mice without changes in startle magnitude (Fig. 1C,D). Although there was no outright effect of genotype on PPI, there was a significant interaction of genotype with the prepulse intensity showing an influence of genotype on response to the prepulse. Furthermore, *Grk3*^−/−^ mice, although not showing elevated spontaneous locomotor activity (Supplementary Fig. 1), were more sensitive to the locomotor stimulatory effects of D-amphetamine (Fig. 1E,F), another rodent model relevant to psychosis [41, 42]. This is in line with a previous study reporting enhanced amphetamine-induced locomotor activity in *Grk6*^−/−^ mice [43].

### Dopamine neurotransmission

Previous studies show that GRK3 affects dopamine signaling in a phosphorylation independent manner [44]. Here we performed a series of biochemical and electrophysiological experiments to investigate dopamine-related aberrations. Microdialysis experiments in the striatum, an area chosen since administration of D-amphetamine enhanced locomotor activity in the *Grk3*^−/−^ mice, showed that dopamine release in *Grk3*^−/−^ mice following D-amphetamine administration, was markedly enhanced whereas baseline levels were similar as to wildtype mice (Fig. 1G). Such dopaminergic hyper-reactivity is suggested to reflect a decreased inhibition of midbrain dopamine firing by amphetamine [30]. In line with this, we observed a higher density of spontaneously active VTA dopamine cells in *Grk3*^−/−^ mice (Fig. 1H). Moreover, the dopaminergic cells of *Grk3*^−/−^ mice showed an increased firing (Fig. 1I), although burst firing activity was unaltered (Fig. 1J).

### Label-free neuroproteomics

To explore how *Grk3* more broadly influences the protein expression signature in the PFC, we employed a label-free LC-MS/MS neuroproteomics methodology. The PFC was selected for this analysis due to its intimate relation to cognitive dysfunctions and since our behavioral results from the Y-maze suggest possible PFC dysfunction. 4501 proteins were identified in the PFC, with 429 of these meeting focus protein significance criteria (fold change ±1.5 and significance value of *P* ≤ 0.05, in *Grk3*^−/−^ mice relative to wildtype controls, see Supplementary Table S2). Confirming *Grk3* deletion, we did not detect Grk3 protein in the PFC of *Grk3*^−/−^ mice. IPA analyses highlighted pathways involved in mitochondrial dysfunction, oxidative phosphorylation, lipopolysaccharide (LPS)/IL-1 mediated inhibition of the retinoic X receptor, acute phase response signaling, complement system activation, GABA receptor signaling, glutamate degradation, and L-glutamine biosynthesis (Supplementary Table S3). Network analyses were used to determine how Grk3 affects the data set of IPA inferred proteins. The first analysis suggested a direct association between an upregulation in interleukin associated kinase-1 (IRAK1) and the IL-1 receptor, and indirect interactions between IRAK1 and IL-1β, as well as IRAK1 and Caspase-1. Our network analysis also identified a cluster of proteins indirectly associated with vimentin and chromogranin A (both upregulated in reactive astrocytes) [45, 46], both forming part of the defined schizophrenia and bipolar disorder networks, respectively (Supplementary Table S4), as well as significantly downregulated proteins that form part of GABA receptor signaling and glutamate degradation, associating with both schizophrenia and bipolar disorder spectrum disorder (Supplementary Table S4). A secondary network analysis of significantly altered focus and reference proteins associated the IL-1 receptor with SERPINA3, GABRG2, GAD2, and IRAK1 with schizophrenia and related disorders (early-onset schizophrenia, schizoaffective disorder, and psychosis). We also identified significant upregulation of the complement component 1q (C1q) that associated in this network (Supplementary Table S4) and are involved in microglia-mediated synaptic pruning, a process indicated to be dysregulated in schizophrenia [47].

### Brain immune signaling

**Fig. 2:**
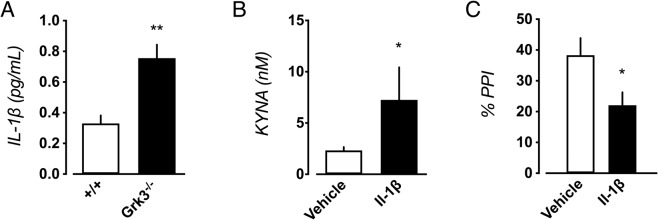
IL-1β activation in *Grk3*^−/−^ mice. **A** Increased levels of IL-1β are detected in hippocampus of *Grk3*^−/−^ mice (*n* = 5) compared to *Grk3*^−/−^ mice (*n* = 5; *P* = 0.0079). **B** In wildtype mice (vehicle control: *n* = 8, and *n* = 9 IL-1β administered mice), ICV administration of IL-1β (0.5 ng) significantly increased hippocampal KYNA levels (*P* = 0.027), and (**C**) induced PPI deficits (*P* = 0.027; vehicle control *n* = 8, and *n* = 9 IL-1β administered mice). Group comparisons in (**A**) and (**B**) were performed using Mann–Whitney *U* tests. Group comparisons in (**C**) were performed using unpaired *t* test with Welch’s correction. All error bars represent SEM. All tests were two-tailed. *P < 0.05, and **P < 0.01.

Increased levels of the pro-inflammatory cytokines IL-6, IL-1β, and IL-8 has been observed in CSF obtained from patients with schizophrenia [10, 17]. In bipolar disorder, increased CSF levels of IL-1β have also been observed among euthymic patients with a life-time history of psychotic episodes [11, 18]. Furthermore, postmortem studies indicate increased mRNA levels of IL-1β and IL-6 in schizophrenia as well as in bipolar disorder patients [12]. In *Grk3*^−/−^ mice, we measured hippocampal levels of IL-1β, IL-6, IL-8, IL-10, IL-12p70, INF-γ, and TNF-α. Increased IL-1β levels were observed in *Grk3*^−/−^ mice (Fig. 2A), while no differences in IL-6 or IL-8 concentrations were observed (data not shown), and the other cytokines were undetectable.

We have previously shown in human astrocyte culture that IL-1β induces the kynurenine pathway of tryptophan metabolism, by activating tryptophan dioxygenase 2 (TDO2), and thereby increasing the production of KYNA. To investigate if IL-1β increases hippocampal levels of KYNA in wildtype mice, we measured KYNA 6 h after ICV infusion of IL-1β (0.5 ng). This resulted in elevated hippocampal KYNA levels (Fig. 2B). Brain KYNA levels [48], as well as *TDO2* mRNA expression [49], are also increased in schizophrenia as well as bipolar disorder patients with psychotic features. To investigate if IL-1β induced KYNA increases contribute to psychosis-like phenotypes in *Grk3*^−/−^ mice, we investigated PPI following administration of IL-1β ICV (0.5 ng) in wildtype mice. IL-1β caused a disruption in PPI (Fig. 2C) with no effect on startle magnitude (data not shown). Notably, deficits in PPI were previously observed in rats with elevated brain levels of KYNA [50]. Altogether, these experiments suggest that an IL-1β-driven induction of KYNA synthesis may contribute to the disrupted PPI in *Grk3*^−/−^ mice.

**Fig. 3:**
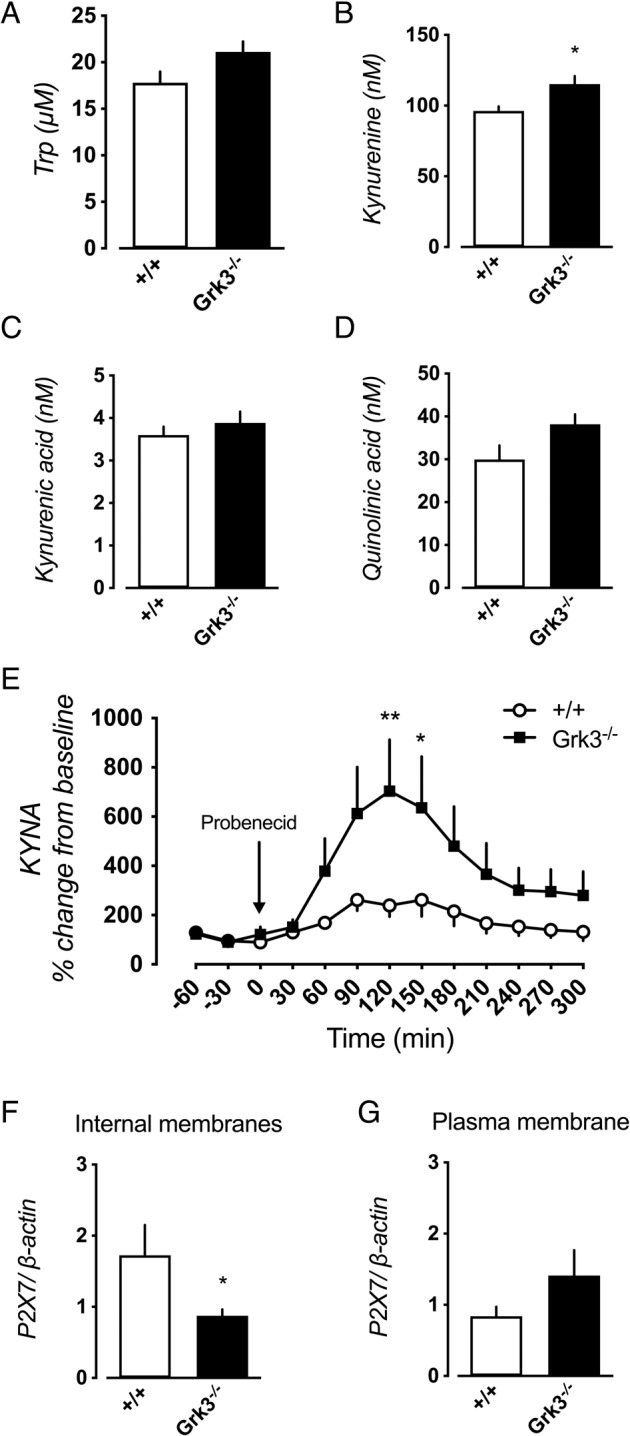
Kynurenine pathway activation and *P2RX7* upregulation in *Grk3*^−/−^ mice. Levels of (**A**) tryptophan (*P* = 0.094), **(B)** kynurenine (*P* = 0.014), (**C**) KYNA (*P* = 0.44), and (**D**) quinolinic acid (*P* = 0.12) in hippocampal tissue obtained from *Grk3*^−/−^ as compared to *Grk3*^+/+^ mice. **E** Probenecid administration (200 mg/kg) was used to monitor accumulation of hippocampal KYNA (effect of: time F(12,108) = 11.47, *P* < 0.0010; genotype F(1,9) = 3.59, *P* = 0.091; interaction F(12,108) = 3.771, *P* < 0.0010) with significantly increased accumulation of KYNA in *Grk3*^−/−^ mice at 120 as well as 150 min post infusion (Bonferroni post hoc test, *P* = 0.0028 and *P* = 0.033, respectively). **F** *Grk3*^−/−^ mice displayed decreased expression of P2X7R on internal cellular membranes obtained from homogenized brain tissue (*P* = 0.017), while (**G**) no changes was observed in plasma membranes (*P* = 0.32). All experiments in (**A**)–(**E**) were carried out using 8 *Grk3*^+/+^ mice and 7 *Grk3*^−/−^ mice. In (**F**) and (**G**) 5 *Grk3*^+/+^ and 6 *Grk3*^−/−^ were used. Data in (**A**)–(**D**) and (**F**) to (**G**) were analyzed using Mann–Whitney *U* tests, while the data in (**E**) were analyzed using a 2-way repeated measures ANOVA followed by Bonferroni post hoc test. All error bars represent SEM. All tests were two-tailed. *P < 0.05, and **P < 0.01.

To investigate directly if the kynurenine pathway is induced in *Grk3*^−/−^ mice, we examined the levels of KYNA and other kynurenine pathway metabolites in serum and hippocampal tissue. We also performed microdialysis experiments in awake, freely moving mice to measure KYNA turnover in the presence of probenecid (200 mg/kg, intraperitoneal; IP), an inhibitor of the large neutral amino acid transporter 1 [51], previously shown to cause accumulation of KYNA in brain tissue [50]. This strategy enabled us to measure turnover of KYNA by assessing the rate of accumulation. Analysis of the accumulation of a compound is a traditional approach reflecting its functional ability to affect its targeted receptors [52]. A significant elevation of kynurenine was found in the hippocampus of *Grk3*^−/−^ mice (Fig. 3A,D). Using microdialysis, we found that probenecid administration was associated with a considerably larger increase of hippocampal KYNA release in *Grk3*^−/−^ mice (Fig. 3E). Despite the basal increase in hippocampal kynurenine, no changes in hippocampal mRNA expression of critical enzymes of the kynurenine pathway [i.e., indole-dioxygenase (*IDO*), *TDO2*, kynurenine aminotransferase (*KAT*)-I-IV or kynurenine monooxygenase (*KMO*)] were detected (Supplementary Fig. 3). No differences in serum levels of tryptophan, kynurenine, KYNA and QUIN, another metabolite of the kynurenine pathway, were observed in *Grk3*^−/−^ mice (Supplementary Fig. 4).

To explore putative mechanisms underlying increased IL-1β levels in *Grk3*^−/−^ mice, we then studied the purinergic P2X7 receptor (P2X7R). In the brain, P2X7R is expressed in glial cells and the receptor is phosphorylated by GRK3 [53]. By activating caspase-1, P2X7Rs are involved in IL-1β release [54]. We found a significant decrease in P2X7R protein levels in the internal membrane fraction (Fig. 3F) while plasma membrane P2X7R content was unchanged (Fig. 3G). The decreased levels in the internal fraction suggests that internalization of P2X7R, a pivotal instrument to control receptor signaling, may be disrupted in *Grk3*^−/−^ mice.

### Glial activation

**Fig. 4:**
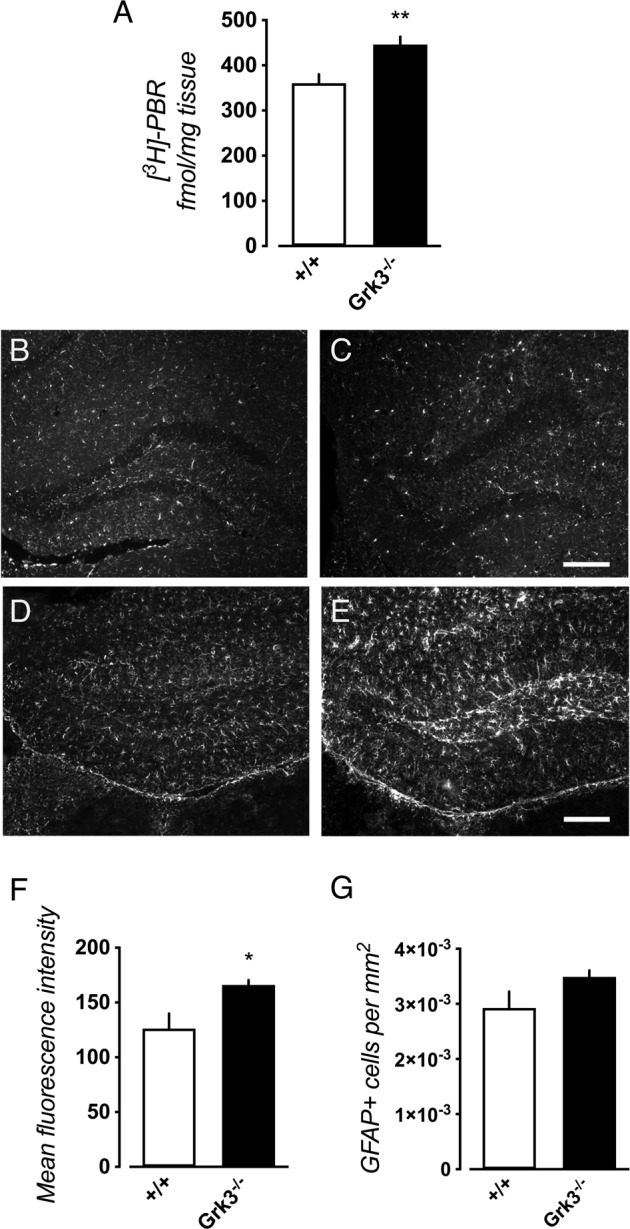
Reactive astrocytes in *Grk3*^−/−^ mice. **A** Autoradiography using the [^3^H]-PBR28 ligand for the 18 kDa translocator-protein (TSPO) in hippocampus revealed increased binding in *Grk3*^−/−^ mice (*n* = 8) as compared to *Grk3*^+/+^ mice (*n* = 10; *P* = 0.0085). Representative image of immunostaining for the microglial marker IBA-1 in hippocampus of (**B**) *Grk3*^+/+^ and (**C**) *Grk3*^−/−^ mice. Representative images of GFAP immunostaining in hippocampus of (**D**) *Grk3*^+/+^ (**E**) and *Grk3*^−/−^ mice. Quantification of the (**F**) mean fluorescence intensity in 8 *Grk3*^−/−^ mice and 7 *Grk3*^+/+^ revealed significantly increased intensity in *Grk3*^−/−^ mice (*P* = 0.044), although (**G**) not reaching significant measuring cell density (*P* = 0.13). Scale bar = 200 μm. All group comparisons were performed using Mann–Whitney *U* tests. All error bars represent SEM. All tests were two-tailed. *P < 0.05, and **P < 0.01.

Since elevated levels of hippocampal IL-1β were observed in *Grk3*^−/−^ mice, with tentatively dysfunctional P2X7R internalization, we investigated glial activation in hippocampus by autoradiography using the [^3^H]-PBR28 ligand for the 18 kDa translocator-protein (TSPO). This revealed a significantly increased binding of [^3^H]-PBR28 in *Grk3*^−/−^ mice (Fig. 4A). However, immunohistochemistry revealed no increased immunoreactivity for the microglial markers IBA-1 (Fig. 4B, 4C) or CD11b (data not shown) in *Grk3*^−/−^ mice. Instead, we observed increased immunoreactivity for the astrocyte markers GFAP (Fig. 4D–G) and Aldh1L1 (data not shown) suggesting that astrocyte activation is more prominent in *Grk3*^−/−^ mice. In line with this, brain KYNA is also mainly produced in astrocytes [55].

### Human studies

**Fig. 5:**
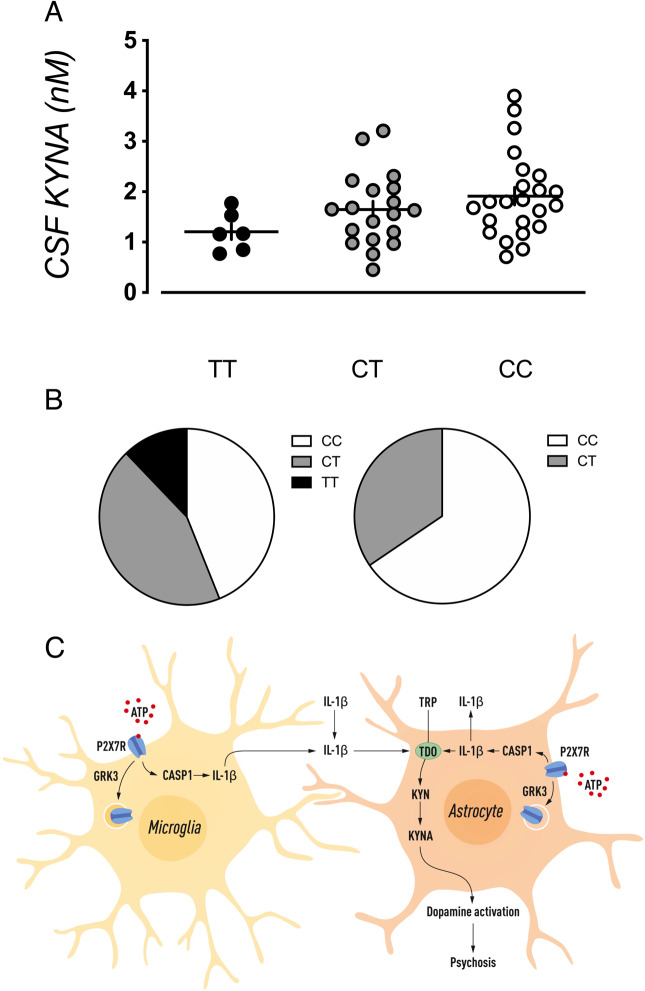
Genetically predicted *GRK3* expression, KYNA levels, and psychotic symptoms in humans. The SNP rs478655 (MAF: 0.29) was used to represent a cis acting eQTL in the *GRK3* promoter with TT genotype denoted as high *GRK3* RNA expression (black circle), CT as medium *GRK3* RNA expression (gray circle), and CC as low *GRK3* RNA expression (white circle). **A** CSF levels of KYNA, in a sample of 48 healthy individuals, as a result of genetically predicted *GRK3* RNA expression (*β* = 0.051, *P* = 0.026). **B** Distribution of subjects with high, low, and medium prediction scores for *GRK3* RNA expression in a sample of 70 bipolar disorder subjects. Subjects with a history of psychosis had higher predicted *GRK3* RNA expression (OR = 2.6; 95% CI: 1.09–6.16). **C** Lack of GRK3 prevents internalization of microglial and/or astrocytic P2X7R, thereby triggering caspase-1 to produce IL-1β. This cytokine induces the kynurenine pathway, resulting in increased production of KYNA, a neuroactive compound that facilitates dopamine neurotransmission. All tests were two-tailed.

To investigate the role of *GRK3* expression for central KYNA levels and psychosis in humans, we used eQTL data. In 48 healthy subjects [*n* = 48 (22 males and 26 females), mean age = 37 years (SD = 13)], we assessed the effect of a *cis* acting eQTL (rs478655) on CSF KYNA levels. The allele associated with decreased *GRK3* expression also associated with increased CSF KYNA levels (Fig. 5A). Utilizing a sample of 70 genotyped bipolar disorder type 1 subjects (20 males and 50 females), of whom 29 had a history of psychosis, we also observed an association between genetically predicted decrease in *GRK3* expression and a history of psychotic symptoms (Fig. 5B).

## Discussion

Numerous studies implicate GRKs as primary components of GPCR signal transduction. Our present results indicate that GRK3 displays glial modulatory actions that are possibly unrelated to such mechanisms. Deficiency of *Grk3* in mice leads to signs of disrupted internalization of the glial P2X7R, a key receptor involved in brain immune responses and leading to increased release of hippocampal IL-1β, and activation of astrocytes. This will subsequently lead to a chain of events involving activation of the kynurenine pathway in astrocytes and increased dopamine neurotransmission (Fig. 5C). Thus, induction of the kynurenine pathway, in particular increased synthesis of the endogenous NMDA receptor antagonist KYNA, plays a fundamental role in signaling brain immune activation to neuronal circuits in *Grk3*^−/−^ mice.

While our immunohistochemical experiments indicated reactive astrocytes, we observed no signs of classical microglial activation. However, cytokines secreted by activated microglia are crucial for inducing reactive astrocytes, and P2X7R dependent IL-1β release [56], as well as TSPO protein levels [57], are more pronounced in activated microglia than in astrocytes. Our proteomic data also revealed increased C1q levels in *Grk3*^−/−^ mice, a key inducer of reactive astrocytes [58] that within the brain is more or less exclusively expressed in microglia [59]. Thus, it is possible that *Grk3*^*−/−*^ mice in addition to reactive astrocytes also display reactive microglial subtypes [60] not captured by our immunohistochemical analyses.

The P2X7 ion channel activation by ATP is among the most central mechanisms initiating inflammatory responses. This receptor, launching a cascade of signaling events leading to the release of IL-1β, is as mentioned above preferably present in microglia although astrocytes to some extent also express this receptor [56]. Previous studies show that increased P2X7R signaling results in activation of microglia and astrocytes [61, 62]. Our results point to a disrupted internalization of the P2X7R with decreased protein levels in the internal membrane fraction of *Grk3*^−/−^ mice. Such a condition may induce caspase-1 [54] and contribute to the presently shown elevation of IL-1β observed in the hippocampus of *Grk3*^−/−^ mice.

KYNA, being an endogenous NMDA-receptor antagonist, acts as a messenger allowing a flow of information from immune signaling in glial cells to neuronal circuits [48]. Our finding that IL-1β elevates brain KYNA levels in wildtype mice suggests that the IL-1β -driven induction of the kynurenine pathway may serve as a core mechanism underlying functional aberrations in *Grk3*^−/−^ mice. The high KYNA turnover seen in *Grk3*^−/−^ mice could account for their hyper-reactive response to D-amphetamine, as seen by a facilitated dopamine release and increased locomotor activity. Indeed, previous studies in rodents show that elevation of brain KYNA enhances both striatal dopamine release [30] as well as the locomotor response by D-amphetamine in mice [38, 63]. In accordance, increased KYNA concentration may also account for the elevated spontaneous firing of midbrain dopamine neurons seen in *Grk3*^−/−^ mice as a large body of studies show that endogenous KYNA activates dopamine firing, likely via a disinhibition of GABAergic afferents [33, 48]. Also, disrupted PPI and attentional deficits in *Grk3*^−/−^ mice are to be expected under a condition of increased turnover of brain KYNA [50, 64, 65]. This view is strengthened by our observation that wildtype mice show disrupted PPI when IL-1β is infused in amounts similar to those used for elevating KYNA.

Despite a behavioral battery of 11 tests (summarized in Supplementary Table S1) covering a wide range of behaviors relating to learning and memory, locomotor behavior, anxiety-like- and psychosis-like behaviors, *Grk3*^−/−^ mice showed few differences from *Grk3*^+/+^ animals. These differences clustered to psychosis-like behaviors with increased locomotor response to D-amphetamine and PPI deficits. Possible attentional deficits in *Grk3*^−/−^ mice in the Y-maze were also detected, however, further testing is needed to fully determine the affected attentional process.

The biochemical and functional data obtained from *Grk3*^−/−^ mice bear striking similarities to clinical findings in patients with psychotic syndromes. In addition, our proteomic data from *Grk3*^−/−^ mice displayed enrichment of altered levels of proteins linked to schizophrenia and bipolar disorder. The disturbance in attentional behavior and PPI seen in *Grk3*^−/−^ mice are prominent features in psychotic disorders [66, 67]. For decades, dysfunctional dopamine neurotransmission has been considered to play a pathophysiological role both in schizophrenia and bipolar disorder [68]. The presently found striatal dopaminergic hyperreactivity in response to amphetamine in *Grk3* deficient mice is also observed in a large number of imaging studies of schizophrenia subjects [69–71]. Furthermore, IL-1β, a cytokine inducing the kynurenine pathway, is shown to be markedly elevated in the CSF of first-episode patients with schizophrenia or bipolar disorder with psychotic episodes [10, 11, 18]. In line with this, elevation of brain KYNA is one of the most frequently described biochemical features in psychotic disorders [18, 38, 72–75]. Altogether, our human genetic data and experimental findings suggest that dysfunctional GRK3 signaling is associated with altered CSF KYNA levels and risk of psychotic features.

To conclude, our analyses reveal an essential role of GRK3 in brain immune homeostasis, where GRK3 deficiency induces the kynurenine pathway to signal inflammatory processes to neuronal circuits of the brain. Furthermore, *Grk3*^−/−^ mice display face and construct validity relating to the psychosis phenotype with glial activation and constitutes an animal model suitable for translational studies of novel immunomodulatory agents in psychotic syndromes.

## Supplementary information

Supplemental Material

Supplementary Table 1

Supplementary Table 2

Supplementary Table 3

Supplementary Table 4

Checklist

## References

[1] Gurevich EV, Tesmer JJ, Mushegian A, Gurevich VV (2012). G protein-coupled receptor kinases: more than just kinases and not only for GPCRs. Pharm Ther.

[2] Steury MD, Lucas PC, McCabe LR, Parameswaran N (2017). G-protein-coupled receptor kinase-2 is a critical regulator of TNFalpha signaling in colon epithelial cells. Biochem J.

[3] Tesmer VM, Kawano T, Shankaranarayanan A, Kozasa T, Tesmer JJ (2005). Snapshot of activated G proteins at the membrane: the Galphaq-GRK2-Gbetagamma complex. Science.

[4] Arriza JL, Dawson TM, Simerly RB, Martin LJ, Caron MG, Snyder SH (1992). The G-protein-coupled receptor kinases beta ARK1 and beta ARK2 are widely distributed at synapses in rat brain. J Neurosci.

[5] Erdtmann-Vourliotis M, Mayer P, Ammon S, Riechert U, Hollt V (2001). Distribution of G-protein-coupled receptor kinase (GRK) isoforms 2, 3, 5 and 6 mRNA in the rat brain. Brain Res Mol Brain Res.

[6] Guindalini C, Collier D, Laranjeira R, Barrett TB, Kelsoe J, Castelo A (2007). Association analysis of GRK3 gene promoter variants in cocaine abuse. Psychiatr Genet.

[7] Dinieri JA, Nemeth CL, Parsegian A, Carle T, Gurevich VV, Gurevich E (2009). Altered sensitivity to rewarding and aversive drugs in mice with inducible disruption of cAMP response element-binding protein function within the nucleus accumbens. J Neurosci.

[8] Gluck L, Loktev A, Mouledous L, Mollereau C, Law PY, Schulz S (2014). Loss of morphine reward and dependence in mice lacking G protein-coupled receptor kinase 5. Biol Psychiatry.

[9] Loh R, Chau L, Aijaz A, Wu K, Galvez R (2017). Antagonizing the different stages of kappa opioid receptor activation selectively and independently attenuates acquisition and consolidation of associative memories. Behav Brain Res.

[10] Soderlund J, Schroder J, Nordin C, Samuelsson M, Walther-Jallow L, Karlsson H (2009). Activation of brain interleukin-1beta in schizophrenia. Mol Psychiatry.

[11] Soderlund J, Olsson SK, Samuelsson M, Walther-Jallow L, Johansson C, Erhardt S (2011). Elevation of cerebrospinal fluid interleukin-1ss in bipolar disorder. J Psychiatry Neurosci.

[12] Fillman SG, Cloonan N, Catts VS, Miller LC, Wong J, McCrossin T (2013). Increased inflammatory markers identified in the dorsolateral prefrontal cortex of individuals with schizophrenia. Mol Psychiatry.

[13] Woo JJ, Pouget JG, Zai CC, Kennedy JL (2020). The complement system in schizophrenia: where are we now and what’s next?. Mol Psychiatry.

[14] Pape K, Tamouza R, Leboyer M, Zipp F (2019). Immunoneuropsychiatry—novel perspectives on brain disorders. Nat Rev Neurol.

[15] Brown AS, Meyer U (2018). Maternal Immune Activation and Neuropsychiatric Illness: A Translational Research Perspective. Am J Psychiatry.

[16] Sasayama D, Hattori K, Wakabayashi C, Teraishi T, Hori H, Ota M (2013). Increased cerebrospinal fluid interleukin-6 levels in patients with schizophrenia and those with major depressive disorder. J Psychiatr Res.

[17] Schwieler L, Larsson MK, Skogh E, Kegel ME, Orhan F, Abdelmoaty S (2015). Increased levels of IL-6 in the cerebrospinal fluid of patients with chronic schizophrenia—significance for activation of the kynurenine pathway. J Psychiatry Neurosci.

[18] Sellgren CM, Kegel ME, Bergen SE, Ekman CJ, Olsson S, Larsson M (2016). A genome-wide association study of kynurenic acid in cerebrospinal fluid: implications for psychosis and cognitive impairment in bipolar disorder. Mol Psychiatry.

[19] Trépanier MO, Hopperton KE, Mizrahi R, Mechawar N, Bazinet RP (2016). Postmortem evidence of cerebral inflammation in schizophrenia: a systematic review. Mol Psychiatry.

[20] Lee SH, Ripke S, Neale BM, Faraone SV, Purcell SM, Perlis RH (2013). Genetic relationship between five psychiatric disorders estimated from genome-wide SNPs. Nat Genet.

[21] Hill SK, Reilly JL, Keefe RS, Gold JM, Bishop JR, Gershon ES (2013). Neuropsychological impairments in schizophrenia and psychotic bipolar disorder: findings from the Bipolar-Schizophrenia Network on Intermediate Phenotypes (B-SNIP) study. Am J Psychiatry.

[22] Tarrant TK, Billard MJ, Timoshchenko RG, McGinnis MW, Serafin DS, Foreman O (2013). G protein-coupled receptor kinase-3-deficient mice exhibit WHIM syndrome features and attenuated inflammatory responses. J Leukoc Biol.

[23] Balabanian K, Levoye A, Klemm L, Lagane B, Hermine O, Harriague J (2008). Leukocyte analysis from WHIM syndrome patients reveals a pivotal role for GRK3 in CXCR4 signaling. J Clin Invest.

[24] Steury MD, McCabe LR, Parameswaran NG (2017). Protein-Coupled Receptor Kinases in the Inflammatory Response and Signaling. Adv Immunol.

[25] Bychkov ER, Ahmed MR, Gurevich VV, Benovic JL, Gurevich EV (2011). Reduced expression of G protein-coupled receptor kinases in schizophrenia but not in schizoaffective disorder. Neurobiol Dis.

[26] Barrett TB, Hauger RL, Kennedy JL, Sadovnick AD, Remick RA, Keck PE (2003). Evidence that a single nucleotide polymorphism in the promoter of the G protein receptor kinase 3 gene is associated with bipolar disorder. Mol Psychiatry.

[27] McCarthy MJ, Barrett TB, Nissen S, Kelsoe JR, Turner EE (2010). Allele specific analysis of the ADRBK2 gene in lymphoblastoid cells from bipolar disorder patients. J Psychiatr Res.

[28] Zhou X, Barrett TB, Kelsoe JR (2008). Promoter variant in the GRK3 gene associated with bipolar disorder alters gene expression. Biol Psychiatry.

[29] Liu XC, Holtze M, Powell SB, Terrando N, Larsson MK, Persson A (2014). Behavioral disturbances in adult mice following neonatal virus infection or kynurenine treatment—role of brain kynurenic acid. Brain Behav Immun.

[30] Olsson SK, Andersson AS, Linderholm KR, Holtze M, Nilsson-Todd LK, Schwieler L (2009). Elevated levels of kynurenic acid change the dopaminergic response to amphetamine: implications for schizophrenia. Int J Neuropsychopharmacol.

[31] Larsson MK, Faka A, Bhat M, Imbeault S, Goiny M, Orhan F (2016). Repeated LPS injection induces distinct changes in the Kynurenine pathway in mice. Neurochem Res.

[32] Vinkers CH, Risbrough VB, Geyer MA, Caldwell S, Low MJ, Hauger RL (2007). Role of dopamine D1 and D2 receptors in CRF-induced disruption of sensorimotor gating. Pharm Biochem Behav.

[33] Tufvesson-Alm M, Schwieler L, Schwarcz R, Goiny M, Erhardt S, Engberg G (2018). Importance of kynurenine 3-monooxygenase for spontaneous firing and pharmacological responses of midbrain dopamine neurons: Relevance for schizophrenia. Neuropharmacology.

[34] van der Walt S, Schonberger JL, Nunez-Iglesias J, Boulogne F, Warner JD, Yager N (2014). scikit-image: image processing in Python. PeerJ.

[35] Ayers-Ringler JR, Oliveros A, Qiu Y, Lindberg DM, Hinton DJ, Moore RM (2016). Label-free proteomic analysis of protein changes in the striatum during chronic ethanol use and early withdrawal. Front Behav Neurosci.

[36] Oliveros A, Starski P, Lindberg D, Choi S, Heppelmann CJ, Dasari S (2017). Label-free neuroproteomics of the hippocampal-accumbal circuit reveals deficits in neurotransmitter and neuropeptide signaling in mice lacking ethanol-sensitive adenosine transporter. J Proteome Res.

[37] Bergen SE, O’Dushlaine CT, Ripke S, Lee PH, Ruderfer DM, Akterin S (2012). Genome-wide association study in a Swedish population yields support for greater CNV and MHC involvement in schizophrenia compared with bipolar disorder. Mol Psychiatry.

[38] Olsson SK, Sellgren C, Engberg G, Landen M, Erhardt S (2012). Cerebrospinal fluid kynurenic acid is associated with manic and psychotic features in patients with bipolar I disorder. Bipolar Disord.

[39] Wall PM, Messier C (2002). Infralimbic kappa opioid and muscarinic M1 receptor interactions in the concurrent modulation of anxiety and memory. Psychopharmacol.

[40] Powell SB, Weber M, Geyer MA (2012). Genetic models of sensorimotor gating: relevance to neuropsychiatric disorders. Curr Top Behav Neurosci.

[41] Arguello PA, Gogos JA (2006). Modeling madness in mice: one piece at a time. Neuron.

[42] van den Buuse M (2010). Modeling the positive symptoms of schizophrenia in genetically modified mice: pharmacology and methodology aspects. Schizophr Bull.

[43] Gainetdinov RR, Bohn LM, Sotnikova TD, Cyr M, Laakso A, Macrae AD (2003). Dopaminergic supersensitivity in G protein-coupled receptor kinase 6-deficient mice. Neuron.

[44] Gurevich EV, Gainetdinov RR, Gurevich VV (2016). G protein-coupled receptor kinases as regulators of dopamine receptor functions. Pharm Res.

[45] Ridet JL, Malhotra SK, Privat A, Gage FH (1997). Reactive astrocytes: cellular and molecular cues to biological function. Trends Neurosci.

[46] Paco S, Pozas E, Aguado F (2010). Secretogranin III is an astrocyte granin that is overexpressed in reactive glia. Cereb Cortex.

[47] Sellgren CM, Gracias J, Watmuff B, Biag JD, Thanos JM, Whittredge PB (2019). Increased synapse elimination by microglia in schizophrenia patient-derived models of synaptic pruning. Nat Neurosci.

[48] Erhardt S, Schwieler L, Imbeault S, Engberg G (2017). The kynurenine pathway in schizophrenia and bipolar disorder. Neuropharmacology.

[49] Miller CL, Llenos IC, Dulay JR, Barillo MM, Yolken RH, Weis S (2004). Expression of the kynurenine pathway enzyme tryptophan 2,3-dioxygenase is increased in the frontal cortex of individuals with schizophrenia. Neurobiol Dis.

[50] Erhardt S, Schwieler L, Emanuelsson C, Geyer M (2004). Endogenous kynurenic acid disrupts prepulse inhibition. Biol Psychiatry.

[51] Singh N, Scalise M, Galluccio M, Wieder M, Seidel T, Langer T et al. Discovery of potent inhibitors for the large neutral amino acid transporter 1 (LAT1) by structure-based methods. Int J Mol Sci. 2018;20:27. 10.3390/ijms20010027 PMC6337383 30577601

[52] Carlsson A, Holmin T, Lindqvist M, Siesjö BK (1977). Effect of hypercapnia and hypocapnia on tryptophan and tyrosine hydroxylation in rat brain. Acta Physiol Scand.

[53] Feng YH, Wang L, Wang Q, Li X, Zeng R, Gorodeski GI (2005). ATP stimulates GRK-3 phosphorylation and beta-arrestin-2-dependent internalization of P2X7 receptor. Am J Physiol Cell Physiol.

[54] Giuliani AL, Sarti AC, Falzoni S, Di Virgilio F (2017). The P2X7 receptor-interleukin-1 liaison. Front Pharm.

[55] Schwarcz R, Bruno JP, Muchowski PJ, Wu HQ (2012). Kynurenines in the mammalian brain: when physiology meets pathology. Nat Rev Neurosci.

[56] Savio LEB, de Andrade Mello P, da Silva CG, Coutinho-Silva R (2018). The P2X7 receptor in inflammatory diseases: angel or demon?. Front Pharm.

[57] Pannell M, Economopoulos V, Wilson TC, Kersemans V, Isenegger PG, Larkin JR (2020). Imaging of translocator protein upregulation is selective for pro-inflammatory polarized astrocytes and microglia. Glia.

[58] Liddelow SA, Guttenplan KA, Clarke LE, Bennett FC, Bohlen CJ, Schirmer L (2017). Neurotoxic reactive astrocytes are induced by activated microglia. Nature.

[59] Srinivasan R, Lu TY, Chai H, Xu J, Huang BS, Golshani P (2016). New Transgenic Mouse Lines for Selectively Targeting Astrocytes and Studying Calcium Signals in Astrocyte Processes In Situ and In Vivo. Neuron.

[60] Hammond TR, Dufort C, Dissing-Olesen L, Giera S, Young A, Wysoker A et al. Single-cell RNA sequencing of microglia throughout the mouse lifespan and in the injured brain reveals complex cell-state changes. *Immunity.* 2019; 50: 253–271 e256. 10.1016/j.immuni.2018.11.004 PMC6655561 30471926

[61] Jimenez-Mateos EM, Arribas-Blazquez M, Sanz-Rodriguez A, Concannon C, Olivos-Ore LA, Reschke CR (2015). microRNA targeting of the P2X7 purinoceptor opposes a contralateral epileptogenic focus in the hippocampus. Sci Rep..

[62] Rodrigues RJ, Tome AR, Cunha RA (2015). ATP as a multi-target danger signal in the brain. Front Neurosci.

[63] Erhardt S, Pocivavsek A, Repici M, Liu XC, Imbeault S, Maddison DC (2017). Adaptive and behavioral changes in kynurenine 3-monooxygenase knockout mice: relevance to psychotic disorders. Biol Psychiatry.

[64] Chess AC, Bucci DJ (2006). Increased concentration of cerebral kynurenic acid alters stimulus processing and conditioned responding. Behav Brain Res.

[65] Shepard PD, Joy B, Clerkin L, Schwarcz R (2003). Micromolar brain levels of kynurenic acid are associated with a disruption of auditory sensory gating in the rat. Neuropsychopharmacology.

[66] Geyer MA (2008). Developing translational animal models for symptoms of schizophrenia or bipolar mania. Neurotox Res.

[67] Barch DM, Ceaser A (2012). Cognition in schizophrenia: core psychological and neural mechanisms. Trends Cogn Sci.

[68] Kesby JP, Eyles DW, McGrath JJ, Scott JG (2018). Dopamine, psychosis and schizophrenia: the widening gap between basic and clinical neuroscience. Transl Psychiatry.

[69] Laruelle M, Abi-Dargham A, Gil R, Kegeles L, Innis R (1999). Increased dopamine transmission in schizophrenia: relationship to illness phases. Biol Psychiatry.

[70] Laruelle M, Abi-Dargham A, van Dyck CH, Gil R, D’Souza CD, Erdos J (1996). Single photon emission computerized tomography imaging of amphetamine-induced dopamine release in drug-free schizophrenic subjects. Proc Natl Acad Sci USA.

[71] Breier A, Su TP, Saunders R, Carson RE, Kolachana BS, de Bartolomeis A (1997). Schizophrenia is associated with elevated amphetamine-induced synaptic dopamine concentrations: evidence from a novel positron emission tomography method. Proc Natl Acad Sci USA.

[72] Erhardt S, Blennow K, Nordin C, Skogh E, Lindstrom LH, Engberg G (2001). Kynurenic acid levels are elevated in the cerebrospinal fluid of patients with schizophrenia. Neurosci Lett.

[73] Erhardt S, Schwieler L, Nilsson L, Linderholm K, Engberg G (2007). The kynurenic acid hypothesis of schizophrenia. Physiol Behav.

[74] Linderholm K, Powell S, Olsson E, Holtze M, Snodgrass R, Erhardt S (2010). Role of the NMDA-receptor in Prepulse Inhibition in the Rat. Int J Tryptophan Res.

[75] Schwarcz R, Rassoulpour A, Wu HQ, Medoff D, Tamminga CA, Roberts RC (2001). Increased cortical kynurenate content in schizophrenia. Biol Psychiatry.

