# Supplementary material for: GRK3 deficiency elicits brain immune activation and psychosis
Source: Mol Psychiatry. 2021 May 12;26(11):6820–32. doi: 10.1038/s41380-021-01106-0 (PMC8760053; doi:10.1038/s41380-021-01106-0)
Supplement: Supplementary file 1 — Supplemental Material [file 41380_2021_1106_MOESM1_ESM.docx]

**Supplementary Information**

**Title:** GRK3 deficiency elicits brain immune activation and psychosis

**Running title:** A new animal model of immune-induced psychosis

**Authors:** Carl M. Sellgren^1, 2^, Sophie Imbeault^1^, Markus K. Larsson^1^, Alfredo Oliveros^3^, Ida A.K. Nilsson^4, 5^, Simone Codeluppi^6^, Funda Orhan^1^, Maria Bhat^7,8^, Maximillian Tufvesson-Alm^1^, Jessica Gracias^1^, Magdalena E. Kegel^1^, Yiran Zheng^1^, Anthi Faka^1^, Marie Svedberg^8^, Susan B. Powell^9^, Sorana Caldwell^9^, Mary E. Kamenski^9^, Marquis P. Vawter^10^, Anton Schullman^11^, Michel Goiny^1^, Camilla I. Svensson^1^, Tomas Hökfelt^12^, Martin Schalling^4, 5^, Lilly Schwieler^1^, Simon Cervenka^2^, Doo-Sup Choi^3,13^, Mikael Landén^14,15^, Göran Engberg^1^, Sophie Erhardt^1^

**Affiliations**:

^1^Department of Physiology & Pharmacology, Karolinska Institutet, Stockholm, SE-17177, Sweden. ^2^Centre for Psychiatry Research, Department of Clinical Neuroscience, Karolinska Institutet, Stockholm & Stockholm Health Care Services, Region Stockholm, SE-17177, Sweden. ^3^Department of Molecular Pharmacology and Experimental Therapeutics, Mayo Clinic College of Medicine, Rochester, MN 55905, USA. ^4^Translational Psychiatry, Department of Molecular Medicine and Surgery, Karolinska Institutet, Stockholm, SE-17177, Sweden. ^5^Center for Molecular Medicine, Karolinska University Hospital, Stockholm, SE-17177, Sweden. ^6^Department of Medical Biochemistry and Biophysics, Karolinska Institutet, Stockholm, SE-17177, Sweden. ^7^Research and Development, Innovative Medicines, Personalised Healthcare and Biomarkers, Translational Science Centre, Science for Life Laboratory, AstraZeneca, Solna SE-17177, Sweden. ^8^Department of Clinical Neuroscience, Karolinska Institutet, Stockholm, SE-17177, Sweden. ^9^Department of Psychiatry, University of California San Diego, La Jolla, California, USA. ^10^Functional Genomics Laboratory, Department of Psychiatry and Human Behavior, University of California Irvine School of Medicine, Irvine, CA, USA. ^11^Human Genetics Branch, National Institute of Mental Health, Bethesda, MD, USA. ^12^Department of Neuroscience, Karolinska Institutet, SE-17177, Stockholm, Sweden. ^13^Department of Psychiatry and Psychology, Mayo Clinic College of Medicine, Rochester, MN 55905, USA. ^14^The Institute of Neuroscience and Physiology, University of Gothenburg, Gothenburg, Sweden. ^15^Department of Medical Epidemiology and Biostatistics, Karolinska Institutet, Stockholm, Sweden.

Carl M. Sellgren and Sophie Imbeault contributed equally to this study.

**Corresponding author:** Göran Engberg, Dept of Physiology & Pharmacology, Biomedicum, quarter 5C, Karolinska Institutet, 17177 Stockholm, Sweden. Tel. +46706296706. Email: [goran.engberg@ki.se](mailto:goran.engberg@ki.se)

**Table of Contents**

Supplementary Materials and Methods ………………………………………… 3

References ……………………………………………………………………… 28

Supplementary Figures 1-5……………………………………………………… 33

**Supplementary Materials and Methods**

**Drugs and chemicals**

D-amphetamine (Sigma-Aldrich, St Louis, MO, USA) was dissolved in sterile saline at a concentration of 2.5 mg/mL and injected intraperitoneally (IP) at a dose of 5 mg/kg (hyperlocomotion) or at a concentration of 4mg/mL and injected IP at a dose of 2 mg/kg (microdialysis). The following drugs and chemicals were also used: Probenecid (Sigma-Aldrich) dissolved in a minimal amount of NaOH and diluted to 16 mg/ml in Tris (Tris (hydroxymethyl) aminomethane) (pH 7.5); interleukin (IL)-1β, diluted in phosphate buffered saline (PBS) and administered intracerebroventricularly (ICV); Bupivacain (Marcain**^®^**, AstraZeneca, Södertälje, Sweden); Isoflurane (Forene**^®^**, Abbott Scandinavia AB, Solna, Sweden); Perchloric acid (PCA) (Kebo Laboratory, Stockholm, Sweden); Lysis buffer (100 mg/ml; 150 mM NaCl, 20 mM Tris pH 7.5, 1 mM EDTA, 1 mM EGTA, 1% Triton X-100); Protease inhibitor (Cat # 1 836 170, Roche, Basel, Switzerland); Phosphatase Inhibitor Cocktail 3 (Cat # P0044, Sigma); Phosphatase Inhibitor 2 (Cat # P5726, Sigma); Acetonitrile, calcium chloride, EDTA, magnesium chloride, methanol, PBS, potassium chloride, sodium acetate, sodium chloride, sodium metabisulfite, Tris, zinc acetate (all Sigma-Aldrich), apomorphine: Apoteksbolaget, Gothenburg, Sweden.

**Animals**

Male mice homozygous for a targeted mutation in the gene coding for *Grk3* on a C57Bl/6J background (*Grk3*^-/-^, B6.129-Grk3^tm1Rjl/J^) were obtained from the Jackson Laboratory (Bar Harbor, ME, USA) and subsequently bred in our animal facility. These mice had already been backbred to N10 prior to deposition at Jackson. Control mice (*Grk*^+/+^) were C57Bl6/J also bred in our animal facility. Mice were kept on a 12h light-dark cycle (lights on 06:00/off 18:00) with food and water available *ad libitum*. Mice for the IL-1β prepulse inhibition experiment were male C57Bl/6J mice obtained from the Jackson Laboratory (Bar Harbor, ME, USA) and housed on a 12h dark-light cycle at University of California San Diego (UCSD; lights off 7:00/on 19:00). Experiments were approved by and performed in accordance with the guidelines of the Ethical Committee of Northern Stockholm, Sweden (Permit number N55/14) in line with Directive 2010/63/EU and by the American Association for the Accreditation of Laboratory Animal Care. All efforts were made to minimize the number of animals used and their suffering. Throughout all types of experiments, mice were randomly selected from different groups to limit the impact of bias. Unless otherwise stated, the investigator was not blinded.

**Behavioral testing**

Testing was carried out in the Department of Physiology and Pharmacology at Karolinska Institutet unless otherwise stated. Animals were between 12-18 weeks of age at the start of testing. Unless otherwise stated, mice were habituated to transport to the testing room and to handling by the experimenter for at least 2 days prior to the start of testing. Animals were brought to the testing room 30 min prior to the beginning of the experiment. All testing occurred between 08:30 and 16:00. Some cohorts were subject to multiple testing, with a minimum of 3 days (usually 1 week) between the tests in the following order (note the change in test order for cohort 2 and 3): *cohort 1* - spatial novel object location memory, light-dark box, and elevated plus-maze, *cohort 2* – novel object recognition, T-maze, Y-maze, D-amphetamine-induced locomotor activity, *cohort 3* - novel object recognition, Y-maze, T-maze, D-amphetamine-induced locomotor activity, *cohort 4* – prepulse inhibition with a subset also used for microdialysis. Otherwise, separate cohorts were run for each test. A separate cohort of mice was run at UCSD for the experiment testing prepulse inhibition following ICV administration of IL-1β.

*Y-maze*

Working memory was assessed in the continuous alternation paradigm using the Y-maze (PanLab, Barcelona, Spain). Animals were placed in one arm of a Y-maze and allowed to explore continuously for 5 min while being recorded from above. Videos were then analyzed and the number of alternations calculated as: no. of alternations (entering into three different arms consecutively) x 100 divided by the total number of arm entries minus 2. Same arm returns were determined by: no. of same arm returns (re-entering the same arm) x 100 divided by the total number of arm entries minus 1. The total number of entries was also determined. Y-maze was cleaned with 70% EtOH between subjects. The number of subjects was *Grk*^+/+^ n=19 and *Grk3*^-/-^ n=20 with such a sample size chosen to increase the sensitivity of the test.

*Spatial Novel Object Location Memory*

Mice were habituated to the open-field arena on three consecutive days for 5 minutes prior to the test day. The open-field arena was made of Plexiglass (51 x 51 x 23 cm) and covered with 2-3 cm of bedding material. A spatial cue was present on one of the walls to provide orientation. Identical objects consisting of three stacked yellow wooden cubes designated A1 and A2 (children’s play objects “Träklossar”, Cat # 44868792, Åhlens, Stockholm Sweden; total dimensions 3 x 3 x 9 cm) were placed at 45^o^ and 20 cm from the maze corners. In a sample phase, animals were allowed to investigate both objects for 10 min. Object A2 was then moved 60^o^ from object A1 one hour following the sample trial, the animals were then put back into the maze for 5 min to investigate the shifted objects. Lighting in the arena was 40-50 Lux, the lowest allowable for recording with a handheld video camera mounted above the arena. Time spent interacting with the object (sniffing and head dips within 5 cm of the object but not jumping or digging around the object) was calculated by an observer using JWatcher 1.0 software (Blumstein, D. T., Evans, C. S. & Daniel, J. C. 2006 *JWATCHER* v. 1.0. See [www.jwatcher.ucla.edu](http://www.jwatcher.ucla.edu/)). The percent interaction time was defined as the time spent investigating the shifted object divided by the total time spent interacting with both objects (A2*100)/(A1+A2). The number of subjects was *Grk*^+/+^ n=12 and *Grk3*^-/-^ n=14.

*Novel Object Recognition*

Mice were habituated as for spatial novel object location memory in the same open-field environment. Identical objects consisting of two green-colored wooden cylinders designated A1 and A2 (children’s play objects, Träklossar, total dimensions 2.8 cm diameter x 6 cm tall) were placed at 45^o^ and 20 cm from the maze corners. In a sample phase, animals were allowed to investigate both objects for 5 min. After 1h, Object A2 was then changed to novel object B (a small blue triangle). Animals were allowed 5 min to investigate the new objects. Lighting in the arena was 40-50 Lux, the lowest allowable for recording with a handheld video camera mounted above the arena. Time spent interacting with the object (sniffing and head dips within 5cm of the object but not jumping or digging around the object) was calculated by an observer using JWatcher 1.0 software (Blumstein, D. T., Evans, C. S. & Daniel, J. C. 2006 *JWATCHER* v. 1.0. See [www.jwatcher.ucla.edu](http://www.jwatcher.ucla.edu/)). The percent interaction time was defined as the time spent investigating the novel object divided by the total time spent interacting with both objects (B*100)/(B+A). The number of subjects was *Grk*^+/+^ n=13 and *Grk3*^-/-^ n=13.

*Morris water maze*

Long-term memory was assessed using a water maze in which a hidden platform (10cm diameter, Ugo Basile, Gemonio, Italy) was placed 1—1.5cm below the water level in a target quadrant of a pool (120cm diameter, Ugo Basile) filled with opaque water (temperature 24 ± 1^o^C). Visual cues surrounded the pool, dividing the pool into quadrants. Animals were given a maximum of 60s to find the platform and performance was recorded using a video camera suspended above the pool. Each animal received 4 training trials per day on a per cage basis. On the first two days of acquisition, trials were initiated from the same quadrant. On days 3-5, trials were started from different quadrants every day. On Day 6, a probe trial lasting 60s was administered during which the platform was not in the pool and the amount of time spent in the target (platform) quadrant was calculated. On days 7-9, reversal training was carried out with 4 trials per animal with trials initiated in different quadrants on different days. Here, the platform was moved 180^o^C from the previous location. A second probe trial lasting 60s was conducted on Day 10 and the amount of time spent in the platform quadrant was calculated although the platform was not in the pool on probe trial day. The experimenter was blinded to cage identities (+/+ or -/-) and cages were used in a random fashion. The number of subjects was *Grk*^+/+^ n =13 and *Grk3*^-/-^ n=16.

*Light-Dark Box*

The light-dark box is made of Plexiglass (50 x 25 x 25 cm), and is equally divided into two compartments (one black and one white) separated by a partition with a 10 x 5 cm opening in the center. Lighting in the white compartment was 610 ± 30 Lux. The black compartment was completely dark and covered by a lid. Each mouse was placed in the center of the wall facing away from the partition in the light section and allowed to explore the box freely for 5 min while being recorded with an overhead digital video camera. Urine and feces were removed and the box cleaned with 70% EtOH between subjects. Time spent in the compartments and total number of transitions were analyzed with the help of JWatcher 1.0 software. The number of subjects was *Grk*^+/+^ n=13 and *Grk3*^-/-^ n=13.

*Elevated-Plus Maze*

The maze consists of two open and 2 enclosed runways in a ‘plus’ shape and is elevated 50 cm above the ground. Lighting conditions were the following: open arm 300-330 Lux; Center 45-50 Lux; closed arm <5 Lux. The maze was cleaned with 70% EtOH between subjects. Animals were recorded via a ceiling-mounted video camera for 5 min. Videos were analyzed off-line using TopScan Lite tracking software (Clever Systems, Inc., Reston, VA, USA). Time and number of entries into each area were determined. The number of subjects was *Grk*^+/+^ n=12 and *Grk3*^-/-^ n=13.

*T-maze rewarded alternations*

Working memory was also assessed using the rewarded alternations protocol for the T-maze as described by Deacon and Rawlings ^1^. The T-maze apparatus (T-maze, PanLab) was modified to include both visual and tactile stimuli. On day 1, animals received group habituation where up to 5 animals are placed in the T-maze for 10 min and receive unlimited rewards once the previous reward had been eaten from the food cup. On days 2-4, each animal received 10 min of training where one arm was blocked off and the animal had to consume the reward before being gently guided to the start point. Alternating left-right trials were given. On day 5, each animal received 3 trials of the test procedure where first a sample run is given (with one arm blocked off) and then a free run where the animal must choose the non-visited arm that still contains the reward. A choice was considered made when the tail-tip entered the arm. On day 6, animals received 10 trials of sample and free runs with a delay of 60s between sample phase and goal phase and an intertrial interval (ITI) of 15 min. The number of subjects was *Grk*^+/+^ n=7 and *Grk3*^-/-^ n=8.

*Open-field, Novelty and D-amphetamine-induced hyperlocomotion*

Two one-hour habituation sessions were run on consecutive days in open-field boxes made of Plexiglass (50 x 50 x 21.6 cm) and located in sound-dampened dark chambers (ADITECH, Fjärås, Sweden). The first ten minutes of the first habituation trial was used to determine novelty-induced hyperlocomotion. The first one-hour session was used to assess general open-field behavior such as locomotion, rearing, and center activity (*Grk*^+/+^ n=20 and *Grk3*^-/-^ n=25). On the third day, a one-hour baseline session was performed followed by injection of saline or D-amphetamine 5 mg/kg followed immediately by another one-hour session. The number of subjects used were 7 *Grk3*^+/+^ saline, 13 *Grk3*^+/+^ D-amphetamine, 8 *Grk3^-/-^* saline, 17 *Grk3^-/-^* D-amphetamine. The boxes were cleaned with mild soap and water and 70% EtOH between subjects. Locomotion and activity parameters were measured via photobeam breaks as described in Olsson *et al.* ^2^. Sample size was chosen in accordance with other papers examining psychostimulant-induced locomotion in the field^3-5^.

*Prepulse Inhibition*

Startle response and prepulse inhibition (PPI) testing were performed on *Grk3*^-/-^ (n=20) and *Grk3*^+/+^ (n=19) mice. The sample size was consistent with similar phenotyping studies of schizophrenia models^6-9^. Mice were tested in a dual setup of ventilated, sound-attenuating commercial startle chambers (35×33×46 cm, SR-LAB^™^ system, San Diego Instruments, CA, USA). Within each chamber a Plexiglas cylinder (3.7 cm in diameter) was mounted, into which the mouse was placed. Sudden movements by the mouse were detected by a piezoelectric accelerometer attached below the cylinder. A loudspeaker provided the broadband background noise and acoustic stimuli and a standard computer controlled the presentations of acoustic stimuli. The experimental session consisted of a 10 min acclimatization period to a 65 dB background noise (continuous throughout the session), followed by presentation of four trial types: a 40 ms, 120 dB startle pulse (PULSE ALONE), and three 20 ms prepulse+pulse combinations (69, 73 or 81 dB prepulses followed 100 ms later by a 120 dB stimulus (PREPULSE+PULSE). There were 12 presentations of the PULSE ALONE trial and 10 presentations of each PREPULSE+PULSE combination. Throughout the session, hidden NOSTIM trials (i.e. no acoustic stimulus) were presented in between each trial. Trial types were presented in a pseudo-random order with an average ITI of 15 s, not including the hidden NOSTIM trials. In addition, six PULSE ALONE trials were presented at the beginning to assess startle reactivity before appreciable habituation. Percent acoustic PPI from the prepulse intensity block of the test was calculated using the following formula: 100 – (avg startle of prepulse + pulse trial/avg startle pulse alone)*100. For IL-1β infusion experiments (vehicle n= 8 and IL-1β n=9), a brief session (20 min) was conducted every hour for 6 hours post-infusion as previously described ^10^. Briefly, a 2-min acclimation period was followed by presentation of four 120 dB pulse trials and then two blocks of prepulse+pulse trials. In the first block, prepulse trials varied by their prepulse intensity (69, 73, 77 dB above background; 10 of each); whereas, in the second block, prepulse trials had a constant prepulse of 73 dB but varied interstimulus intervals (ISI) of 20, 120, 360 ms (four trials of each).

**Microdialysis**

*Surgery*

Mice were anesthetized in a small Plexiglas chamber continuously ventilated with 4% isoflurane in air using a vaporizer (Univentor 400 Anesthesia Unit; Univentor Ltd, Zejtun, Malta) and mounted in a David Kopf stereotaxic frame (David Kopf Instruments, Tujunga, CA, USA). Anesthesia vas maintained with 2% isoflurane delivered by a nose cone. The body temperature of the animals was controlled by a thermometer and a heating pad maintained at 37°C (Homeothermic Blanket Control Unit 50-7053-F, Harvard Apparatus, Holliston, MA, USA). An ocular lubricant was then applied and 0.5 ml sterile saline was given subcutaneously to prevent dehydration. After the scalp was incised (7 – 8 mm), the skull was cleaned and a thin layer of quick-setting cyanoacrylate glue (BT AB, Stockholm, Sweden) was applied to the exposed skull, serving as an adhesive surface to the cement. A small hole was then drilled over either the striatum (A-P: Bregma +0.5 mm, M-L: -2 mm) or the hippocampus (A-P: Bregma -2.9 mm, M-L: -3.2 mm), and a guide cannula (AT4.7.IC, AgnTho’s AB, Lidingö, Sweden) containing a dummy probe (outer diameter: 0.2 mm) was implanted (D-V: -1.5 mm below the skull surface), and secured to the skull with acrylic dental cement (Dentalon® plus, Heraeus, Hanau, Germany). Stereotactic coordinates were according to the mouse brain atlas by Paxinos. The wound was then swabbed with bupivacaine (5 mg/ml), sutured and mice were allowed to recover single-housed for 48 hours.

*Experimental Procedure*

All microdialysis experiments were performed while the mice were freely moving in their home cage. On the day of experiment, mice were tethered to a liquid swivel and a microdialysis probe (AT4.7.2.PES, shaft length: 7 mm, membrane length: 2 mm, molecular cut-off: 6 kDa, AgnTho’s AB) was inserted through the guide cannula and connected to a microinfusion pump (Univentor 864, Univentor Ltd) set to a speed of 1 μl/min and perfused with Ringer solution containing 148 mM NaCl, 4 mM KCl, 0.8 mM MgCl_2_, 1.4 mM CaCl_2_. All samples were collected in plastic tubes attached to the tether throughout the experiment and immediately subjected to high-performance liquid chromatography (HPLC) analysis. The first three samples were taken for determination of basal extracellular kynurenic acid (KYNA) (injected into the same HPLC system used for KYNA brain tissue analysis, see respective section) or dopamine levels. Results for subsequent samples were calculated as percentages of basal levels. Values were not corrected for *in vitro* probe recovery (normally circa 15-20%). The mice then received an IP injection of D-amphetamine (2 mg/kg, striatal dopamine measurements, *Grk3*^+/+^ n=5, *Grk3*^-/-^ n=5) or probenecid (200 mg/kg, hippocampal KYNA analysis, *Grk3*^+/+^ n=8, *Grk3*^-/-^ n=7). Microdialysate was collected in 30 min intervals for up to 300 min. After the session, the mice were sedated with isoflurane and sacrificed with cervical dislocation and tissues were frozen for later analysis and histological verification of probe placement.

*Kynurenic acid (KYNA) detection in microdialysate*

Brain dialysate was mixed with 100 μl 70% strength PCA. This solution was then re-centrifuged (21000 × g, 5 min), and the supernatant was transferred to a new tube for HPLC analysis. To determine KYNA concentration, samples were subjected to analysis utilizing an isocratic reversed-phase HPLC system coupled to a fluorescence detector (FP-2020 Plus, Jasco Ltd., Hachioji City, Japan) with an excitation wavelength of 344 nm and an emission wavelength of 398 nm (18 nm bandwidth). A mobile phase consisting of 50 mM sodium acetate and 7% acetonitrile (pH set to 6.2 using acetic acid) was pumped through a ReproSil-Pur C18 column (4 × 150 mm, Dr. Maisch GmbH, Ammerbuch, Germany), at a flow rate of 0.5 ml/min, delivered by a LC-10AD VP (Shimadzu Corporation, Kyoto, Japan). Zinc acetate (0.5 M, not pH adjusted) was delivered post-columnar by a Pharmacia P-500 (GE Healthcare, Uppsala, Sweden) at a flow rate of 10 ml/h. Signals from the fluorescence detector were transferred to a computer for analysis with Datalys Azur (Grenoble, France).

*Dopamine detection in microdialysate*

30 μl of brain dialysate was separated by reversed-phase liquid chromatography using a 55 mM sodium acetate buffer (pH 4.1, 10 % methanol) with 1.16 mM octanesulfonic acid and 0.01 mM Na_2_EDTA. The mobile phase was delivered by an HPLC pump (Bischoff Chromatography, Leonberg, Germany) through a Agilent Eclipse XDB-C18 column (4.6×150 mm, Agilent Technologies, Inc., Santa Clara, CA, USA) at a rate of 0.7 ml/min. Samples were then quantified by sequential oxidation and reduction in a high-sensitivity analytical cell (ESA 5011; ESA Inc., Chelmsford, MA, USA) controlled by a potentiostat (Coulochem III; ESA Inc.) with an applied potential of -350 mV for detection of dopamine. The signals from the detector were transferred to a computer for analysis (Datalys Azur). The retention time of dopamine was approximately 7 min.

## **Intracerebroventricular administration of IL-1β**

Surgery (at Karolinska Institutet) was performed according to the *in vivo* microdialysis surgical procedures up to the point of skull exposure. Here, a small hole was drilled through the skull (A-P: Bregma -0.34 mm, M-L: -1 mm) and a thin glass capillary was then inserted into the ventricle (D-V: 2 mm below the skull surface). Thereafter, IL-1β (n=9) or vehicle (n=8) was infused over a time period of 8 minutes (4 μl infusion volume). The capillary was allowed to stay in for another 5 minutes to minimize backflow. The wound was then closed with a stainless steel suture clip and mice were allowed recover in their home cage. Mice were sacrificed 6 hours post-infusion by cervical dislocation under isoflurane anesthesia and brain tissues harvested and stored at -80°C. For ICV IL-1β behavioral experiments (prepulse inhibition, conducted at UCSD), surgery was performed as described previously ^11^. A 23-gauge 7 mm unilateral stainless steel guide cannula was implanted above the lateral ventricle (flat skull; A-P: Bregma -0.1 mm; M-L: +1.1 mm; D-V: 1.5 mm below dura). Mice recovered for 5 days prior to behavioral testing. IL-1β was infused through a 30-gauge 8 mm injector in a volume of 0.5 µl (via gravity infusion). At the end of the study, cannula placement was checked via dye infusion. The number of subjects was vehicle n=8 and IL-1β n=9.

***In vivo* recordings in the ventral tegmental area (VTA)**

Electrophysiology was performed as previously described ^12^. Briefly, mice (*Grk3*^+/+^ n=7, *Grk3*^-/-^ n=6) were anesthetized (chloral hydrate, 400 mg/kg, IP, Merck, Darmstadt, Germany) and mounted onto the ear bars of a stereotaxic frame (David Kopf Instruments) with the skull set in a horizontal plane and the nose and upper incisors secured using a clamp at the front of the frame. Throughout the experiments, the depth of anesthesia was monitored by hind paw pinching and additional injections of chloral hydrate were given as needed to maintain a stable level of anesthesia. Whenever additional chloral hydrate was given, the experiments were paused for at least ten minutes after injection. The body temperature was maintained at 37°C by means of a thermostatic heating pad. The skull surface was exposed and a 3 mm burr hole was drilled with its center located 3.1 mm posterior to Bregma and 0.5 mm lateral to the midline. Glass microelectrodes were prepared by pulling a glass capillary (Harvard Apparatus) through a heated vertical electrode puller (Narishige, Japan) and filled with a 0.5 M sodium acetate solution saturated with Chicago Sky Blue. The in vitro impedance was always between 6.5 and 7.5 MΩ, as measured at 135 Hz in 0.9% saline. The electrode was placed 3.2 mm posterior to Bregma and 0.5 mm lateral to the midline and lowered using a hydraulic micro drive (David Kopf Instruments) in to the VTA, according to the stereotaxic coordinates from the atlas of Paxinos and Watson (3^rd^ edition, 2008). The electrode was subsequently moved in a predetermined grid pattern between 3.2-2.8 mm posterior to Bregma and 0.5-0.6 mm lateral to the midline, with each grid separated by 100 μm. Single unit potentials were passed through a high input-impedance amplifier and filters. The impulses were discriminated from background noise and fed into a computer, while simultaneously displayed on a digital storage oscilloscope, monitored on an audio monitor and on a strip chart recorder (Gould Electronics GmbH., Eichstetten, Germany). All neurons were found 4.0-5.0 mm below the brain surface and fulfilled the neurophysiological characteristics (i.e. triphasic action potentials with a duration more than 2.0 ms, basal firing rates between 1 and 10 Hz and frequent occurrence of burst firing including progressively decreasing spike amplitude) previously described for midbrain dopamine ^13, 14^. To further confirm that recordings had been made exclusively from dopamine neurons, the inhibitory action of a single dose of the dopamine agonist apomorphine (100 μg/kg, intravenous) was verified at the end of the experiments when the experiments allowed. In order to ensure no dopamine cells were recorded more than once, the location and firing properties of each individual cell were noted and compared. The distribution of spikes was analyzed on line utilizing a Spike II software program. In order to avoid artifacts in the sampling procedure, the program was set to ignore time intervals below 20 ms. The onset of a burst was determined as an inter-spike interval shorter than 80 ms and the termination of a burst by the next interval longer than 160 ms ^13^. Cells were considered to be bursting if at least one interspike time interval of 100 recorded spikes was below 80 ms. The intervals were analyzed with regard to the number of bursts that occurred during a sampling period of 100–500 spikes along with a calculation of the percentage of spikes fired in bursts. The number of cells recorded were *Grk3*^+/+^ n=35 cells, *Grk3*^-/-^ n=78 cells.

#### **Liquid Chromatography/Mass spectrometry (LC/MS) for tryptophan, kynurenine, KYNA, and quinolinic acid**

Brain samples (*Grk3*^+/+^ n=8, *Grk3*^-/-^ n=7)were run as previously described ^15^. Briefly, the hippocampus was dissected, weighed, sonicated in 600 µl PBS buffer and ultracentrifuged at 4°C, 30 000 × g. The supernatant (50 µl) was added internal standard in 5% formic acid and filtered at 3000 × g for 60 minutes at 10°C using 10 kDa Ultracel®-10 filter plates from Millipore (MilliporeSigma, Burlington, MA, USA). After centrifugation, 7.5 µl of the filtrate was injected using a Waters Acquity HPLC system (Waters Corporation, Stockholm, Sweden) equipped with a SymmetryShield^TM^ RP18 2.1 × 100 mm, 3.5 µm particle column. Mobile phase was run at 300 µl/minute and consisted of 2.1% formic acid in MilliQ water (A phase) and 95% acetonitrile + 0.1% formic acid (B phase). Runs started with 5% B in A for 2 minutes following gradient elution, with a total run time of 10 min. Retention times for quinolinic acid, kynurenine, tryptophan, and KYNA were 1.2, 1.7, 3.6 and 3.9 min, respectively.

Serum (50 µl) were prepared as in ^16^ using solid phase extraction (Oasis® MAX, Waters Corp.). Cartridges were equilibrated, added samples and internal standard in 5% ammonium hydroxide, washed, and analytes were eluted (60% acetonitrile, 38% methanol and 2% formic acid). The organic phase was evaporated using nitrogen and analytes were redissolved in 2.5 % formic acid. After solid phase extraction, 7.5 µl of the extract was injected using a Waters Acquity HPLC system equipped with an HSST3 2.1 × 100 mm, 1.8 µm particle column. The mobile phase was run at 300 µl/minute and consisted of 2.1% formic acid in MilliQ water (A phase) and 95% acetonitrile 0.1% formic acid (B phase). Runs started with 2% B in A for 2 minutes followed by gradient elution, with a total run time of 15 minutes. Retention times for quinolinic acid, kynurenine, tryptophan, and KYNA were 1.5, 3.8, 5.9 and 6.3 min, respectively.

Detection was performed using a Waters Xevo TQ-S triple quadrupole MS, operating in positive ionization MS/ MS configuration. The MS was tuned for all analytes, and the mass spectral transitions were set at m/z 168 > 106; 209 > 146; 205>91; and190>116 and for the IS172 > 110 (13C 15N -QUIN), 213 > 150 (D -Kynurenine) 210 > 150 (D5-Tryptophan) and 194 > 120 (D5-KYNA) QUIN, tryptophan, D5-Tryptophan and kynurenine were purchased from Sigma-Aldrich and 13C 15N -QUIN from SynFine Research Inc. (Richmond Hill, Canada), D4-Kynurenine and D5-KYNA from Buchem B.V. (Apeldoorn, Netherlands), formic acid, methanol and acetonitrile were purchased as MS-grade from Sigma-Aldrich. Standards of each analyte were used to establish a linear calibration curve and plotted using the ratio of analyte peak area over IS peak area after integration by Masslynx 4.1 software (Waters Corp.)

**Preparation of membrane fractions & Western Blot**

Mice (*Grk3*^+/+^ n=5 and *Grk3*^-/-^ n=6) were anesthetized with isoflurane and following decapitation the brains were quickly removed and stored at -80°C until use. Tissues were homogenized in complete hypotonic buffer (10 μM, sodium bicarbonate, 1:1000, protease inhibitor cocktail and 1:1000, phosphatase inhibitor cocktail I and II) and centrifuged at 1200 x g for 10 min at 4°C. The resulting supernatant was centrifuged again at 21 600 x g for 10 min at 4°C. The obtained pellet contained internal membranes and was resuspended in extraction buffer (EB: 0.5% Triton X-100, 50 mM Tris-HCl pH 7.5, 150 mM NaCl, 1 mM EDTA, 1% sodium dodecyl sulfate, protease inhibitor cocktail and phosphatase inhibitor cocktail I and II). The remaining supernatant was centrifuged at 150 000 x g for 2 hours at 4°C and the pellet which contained plasma membranes was dissolved in EB. The protein content of internal and plasma membrane fractions was determined using the bicinchoninic acid (BCA) protein assay kit (Pierce/ThermoFisher Scientific, Waltham, MA, USA) according to the recommendations of the supplier. Internal and plasma membrane fractions were separated on NuPAGE 4-12% Bis-Tris gel electrophoresis (Invitrogen, Carlsbad, CA) and transferred to nitrocellulose membranes (Invitrogen). The non-specific binding sites were blocked with 5% low-fat dried milk in Tris buffer (50 mM Tris-Cl, 6 mM NaCl) containing 0.1% Tween-20 for 1 hour at room temperature. The membranes were later incubated with a primary antibody against P2RX7 (1:10 000, Cat # APR-004, Alomone Labs, Jerusalem, Israel) overnight at 4°C. Following three washes in Tris buffer containing 0.1% Tween 20 for 5 min, the blots were probed with horseradish peroxidase-conjugated secondary antibodies (Cat # 7076 and # 7074, Cell Signaling Technology, Danvers, MA, USA) for 1 h at room temperature. Specific protein bands were visualized using chemiluminescent detection reagents (Supersignal West Pico and West Femto, ThermoFisher Scientific). β-actin antibody (1:10 000, Cat # 3700, Cell Signaling Technology) was used as a protein-loading control and all signal intensity measurements were made using Quantity One software (Bio-Rad Laboratories, Hercules, CA, USA). Obtained data of P2X7R was normalized to β-actin.

**Cytokine measurements**

Mice (*Grk3*^+/+^ n=5, *Grk3*^-/-^ n=5) were euthanized as described in the previous section and their brains removed. While on ice, hippocampi were quickly dissected and stored in -80°C. Samples were homogenized in ice-cold lysis buffer, protease inhibitor, phosphatase Inhibitor Cocktail 3 and phosphatase Inhibitor 2, 2 times x 3 min at 4°C using a Bullet Blender^®^ (Next Advance, Inc. Averill Park, NY, USA). Homogenates were then centrifuged at 16 000 × g (bench top microfuge) for 10 min at 4°C. 50 μl of the supernatant was used for cytokine quantification. IL-1β, IL-6, KC/GRO (IL-8), IL-10, IL-12p70, interferon (INF)-γ, and tumor necrosis factor (TNF)-α were detected with multiplex sandwich enzyme-linked immunosorbent assay. We employed Discovery assays (Meso Scale, Gaithersburg, MD, USA) per the manufacturer's protocol on a SECTOR^®^ Imager 2400 instrument ([http://www.mesoscale.com](http://www.sciencedirect.com/science?_ob=RedirectURL&_method=externObjLink&_locator=url&_issn=00063223&_origin=article&_zone=art_page&_plusSign=%2B&_targetURL=http%253A%252F%252Fwww.mesoscale.com)). All samples were analyzed in duplicates. IL-6, KC/GRO (IL-8), IL-10, IL-12p70, INF-γ, and TNF-α had high inter-individual variation coefficients or were undetectable in both *Grk3*^+/+^ and *Grk3*^-/-^ mice. Results from these cytokines are therefore not shown. IL-1β was reliably detectable in all hippocampal samples. The lowest level of detection for IL-1β was 0.4 pg/ml and the variation coefficient was less than 13%. For statistical analysis the highest values of IL-1β were consistently chosen.

**qPCR**

RNA isolation and quantitative PCR were carried out on hippocampal tissue from *Grk3*^-/-^ (n=5) and *Grk3*^+/+^ (n=6) mice using methods and primers as previously described ^17^.

**Immunohistochemistry**

Anesthetized mice (*Grk3*^+/+^ n=7, *Grk3*^-/-^ n=8) were perfused via the ascending aorta with Tyrode’s Ca^2+^-free solution at 37°C, followed by fixation with a solution of 4% paraformaldehyde and 0.4% picric acid in 0.16 M phosphate buffer (pH 6.9 at 37^o^C) and then with identical fixative at 4°C ^18^. After decapitation, brains were rapidly excised and immersed in ice-cold para-formaldehyde with picric acid for 90 min and rinsed overnight in phosphate buffered 10% sucrose (pH 7.4). The tissue was frozen using CO_2_, cut at 14 μm thickness on a cryostat (Microm, Heidelberg, Germany) and thaw-mounted on SuperFrost plus slides (VWR, Radnor, PA, USA). In addition, some slides previously used for radiography with [^3^H]PBR28 were subsequently processed with immunohistochemistry directed against glial markers. Sections spanning approximately Bregma +0.98 to -2.30 were included in the analysis, thus including areas such as the thalamus, the hippocampus and the caudate putamen. buffer for 30 min. Sections were washed in TNT buffer and incubated in biotinyl tyramide fluorescein conjugate (PerkinElmer Life Science) diluted 1:200 in amplification diluent for 10 min at RT. Sections were analyzed with a Nikon Eclipse E600 Fluorescence microscope equipped with 10X objective (Nikon, Tokyo, Japan). The GFAP labeling in the hippocampus was quantified using the ImageJ Software (National Institutes of Health, Bethesda, MD, USA) together with a custom python script ^19^. Briefly, the selected regions of interest were binarized using the Otsu thresholding algorithm and the fluorescence intensity was quantified in the identified connected components.

For sections incubated with the astroglia marker aldehyde dehydrogenase 1 family, member L1 (Aldh1L1) antigen retrieval with sodium citrate buffer was needed in order to reveal the epitope. All sections were pretreated with 0.03 % H_2_O_2_ and subsequently incubated with antiserum against glial fibrillary acidic protein (GFAP, Cat # G9269, 1:4000, Sigma-Aldrich), Aldh1L1 (1:3000, Cat # ab87117, Abcam, Cambridge, UK), ionized calcium-binding adapter 1 (Iba1, 1:4000, Cat # 019-19741, Wako Pure Chemical Industries, Osaka, Japan), or Cd11b (1:2000, Cat # MCA275G, Serotec, Kidlington, UK) overnight at 4˚C, and then processed according to the TSA-plus Fluorescein System (PerkinElmer Life Science, Waltham, MA, USA). Briefly, sections were washed in Tris NaCl-Tween (TNT) buffer (0.1 M Tris-HCl, pH 7.5; 0.15 M NaCl; 0.05 % Tween 20; Sigma-Aldrich), incubated with TNB buffer (0.1 M Tris-HCl, pH 7.5; 0.15 M NaCl; 0.5 % blocking reagent) for 30 min at room temperature (RT), followed by anti-rabbit/mouse horseradish-peroxidase conjugated secondary antibodies (Dako A/S, Cat # P039901-2/P044701-2, Glostrup, Denmark) diluted 1:200 in TNB.

**[^3^H]PBR28 autoradiography**

Frozen sections at 14 µm were thawed at room temperature and pre-incubated for 20 min in 50 mM Tris HCl, pH 7.4 including 120 mM NaCl, 5 mM KCl, 2 mM CaCl_2_, 1 mM MgCl_2_, followed by 60 min incubation with 1.5 nM [^3^H]PBR28, specific activity 50 Ci/mmol (Karolinska Institutet, Department of Clinical Neuroscience). For the determination of non-specific binding, 10 µM PK11195 was added to parallel sections to block specific binding. After incubation, the slides were washed three times for 3 min each in ice-cold 50 mM Tris HCl, pH 7.4, followed by a brief wash in distilled water. The slides were dried and exposed to phosphor imaging plates (Fujifilm Plate BAS-TR2025, Fujifilm, Tokyo, Japan) together with micro scales standards (American Radiolabeled Chemicals Inc., St-Louis, MO, USA) for calibration and quantification of the binding density. Phosphor imaging plates were scanned and the resulting images were processed in a Fujifilm BAS-5000 phosphor imager (Fujifilm). Mean pixel values of the hippocampus were transformed into radioactivity values and to binding density (pmol/mg tissue, tissue wet weight) using the micro scale standards. Quantitative analysis was performed using Multi Gauge 3.2 phosphorimager software (Science Imaging Scandinavia, Saltsjö-Boo, Sweden). Specific binding, which is an indication of receptor density, is calculated by subtracting the level of non-specific binding from the total binding for each section. The number of subjects was *Grk3*^+/+^ n=10, *Grk3*^-/-^ n=8.

**Label-free neuroproteomics and pathway analysis**

Mice (*Grk3*^+/+^ n=4, *Grk3*^-/-^ n=4) were anesthetized with isoflurane and brains were immediately removed, washed in ice-cold 1x PBS, and the prefrontal cortex (PFC) was subsequently dissected, snap-frozen on dry ice and stored at -80°C until analysis. Tissue was prepared and label-free proteomics was performed as previously described ^20-22^. To extract total protein from PFC, an individual biological replicate was combined with 0.5 mm zirconium oxide beads and 50 µL of ice-cold lysis buffer containing CelLytic MT lysis reagent (Sigma-Aldrich), Complete protease inhibitor cocktail (Roche) and phosphatase inhibitor cocktails II and III (Sigma-Aldrich). Cortical tissue was homogenized in a Storm 24 Bullet Blender (Next Advance Inc.) for 4 min at 4°C at a speed setting of 4. Homogenates were then centrifuged at 16 400 RPM at 4°C for 15 min. Supernatants were collected and analyzed for protein concentrations using Bradford Reagent (Bio-Rad). Proteins were denatured for SDS-PAGE by heating in NuPAGE® LDS Sample Buffer (Invitrogen) for 10 min at 70°C. Cortical lysates were loaded in triplicate (15 µg per lane) and resolved on a 4-12% Bis-Tris Gel in MOPS running buffer (Invitrogen). Gels were fixed with a solution containing 50% methanol and 10% acetic acid, washed in ultra-pure water, then stained with Bio-Safe Coomassie Stain (Bio-Rad) per manufacturer’s instructions. Gel sections were destained, digested with trypsin (140ng of trypsin dissolved in 25mM Tris pH 8.2), reduced with dithiothreitol and alkylated with iodoacetamide. Protein digestion was performed overnight at 37°C. Peptides were extracted from the gel section fragments with 50% acetonitrile (ACN) in 4% trifluoroacetic acid (TFA), followed by two additional extractions with ACN. The combined extracts were evaporated to dryness on a vacuum concentrator and stored at -80°C until further analysis.

**Liquid-chromatography tandem mass spectrometry (LC-MS/MS) for neuroproteomics**

Peptide extracts from each gel section were reconstituted in 40 µL HPLC-grade water containing 0.2% formic acid (FA), 0.1% TFA, and 0.002% Zwittergent 3-16. 10 µL of the peptide extract (15 µL for the two higher molecular weight gel sections) were loaded onto a 0.25 µL bed OptiPak trap (Optimize Technologies, Oregon City, OR, USA) custom-packed with 5um, 200Å Magic C8 (Bruker-Michrom, Auburn, CA, USA) stationary phase. Loaded trap was washed for 4 minutes with an aqueous loading buffer of 0.2% FA and 0.05% TFA at 10 µL /min. Following the wash, peptides were transferred via 10-port valve onto a 35cm x 100 µm PicoFrit column 9 (NewObjective, Woburn, MA, USA), self-packed with Agilent Poroshell 120S 2.7 µm EC-C18 stationary phase, using a Dionex UltiMate® 3000 RSLC liquid chromatography system (ThermoFisher Scientific). Peptides were separated using a 400nL/min LC gradient comprised of 2%-30%B in 0-70 min, 30%-50%B from 70-100 min, 50%-95%B from 100-104 min, held at 95%B for 8 min and re-equilibrated to 2%B. Mobile phase A was 2% ACN in water with 0.2% FA and mobile phase B was ACN/isopropanol/water (80/10/10 by volume) with 0.2% FA. Eluting peptides were analyzed using a QExactive mass spectrometer (ThermoFisher Scientific). The instrument was operated in data-dependent mode by collecting MS1 data at 70,000 resolving power (measured at m/z 200) with an AGC value of 3E6 over a m/z range of 350-2000, using lock masses from background polysiloxanes at m/z 371.10123 and 445.12002. Precursors were fragmented with normalized collision energy of 27, fragments measured at 17,500 resolving power and a fixed first mass of 140. Tandem mass spectra (MS/MS) were collected on the top 15 precursor masses present in each MS1 using an AGC value of 1E5, max ion fill time of 100ms, an isolation window of 3.0 Da, isolation offset of 0.5 Da, and a dynamic exclusion time of 60s.

**Bioinformatic analysis of LC-MS/MS results**

We utilized a label-free peptide MS1 intensity-based method for finding differentially expressed proteins between experimental groups. The quality of the raw data was assessed using the quality control metrics in the Swift proteomic data processing pipeline. MaxQuant (version 1.5.1) software processed the raw data files to produce a list of protein groups and their corresponding intensities in each sample ^23^. To accomplish this, MaxQuant was configured to use a composite mouse protein sequence database containing UniProt mouse reference proteome (downloaded on 12 February 2015) and sequences of common contaminants (trypsin, keratin, cotton, wool, etc.). Reversed protein sequences were appended to the database for estimating protein identification false discovery rates (FDRs). The software was configured to use 20 ppm m/z tolerance for precursors and fragments while performing peptide-spectrum matching. The software derived semitryptic peptides from the sequence database while looking for the following variable modifications: carbamidomethylation of cysteine (+57.023 Da.), oxidation of methionine (+15.994 Da.), formation of n-terminal pyroglutamic acid (-17.023 Da.) and protein n-terminal acetylation (+42.01 Da.). MaxQuant was instructed to align the runs and match features between multiple sample runs of the same gel region. The software filtered peptide and protein identifications at 2% FDR, grouped protein identifications into groups and reported protein group intensities. An in-house script written in R programming language performed differential expression analysis using protein group intensities. First, protein group intensities of each sample were log2 transformed and normalized using Quantile method. For each protein group, the normalized intensities observed in two groups of samples were modeled using a Gaussian-linked generalized linear model. An ANOVA test was used to detect the differentially expressed protein groups between pairs of experimental groups. Differential expression P-values were FDR corrected using Benjamini-Hochberg-Yekutieli procedure. Protein groups with an FDR < 0.05 and an absolute ratio fold change of at least 0.5 were considered statistically significant in terms of differential expression and saved for pathway analysis.

**Ingenuity Pathway Analysis**

QIAGEN’s Ingenuity® Pathway Analysis (IPA®, QIAGEN Redwood City, CA, USA; www.qiagen.com/ingenuity) was used to analyze the proteins identified as significantly different by the MaxQuant bioinformatic analysis described above. Proteins were further restricted to include only those with intensity value readings from at least two technical replicates. The normalized ratios, P-values and FDRs from these resulting proteins were uploaded to IPA with their corresponding SwissProt/UniProt identifiers. IPA was then instructed to only analyze proteins with equal to or greater than a ratio fold change of 1.5, and a Gaussian corrected P-value of less than 0.05. IPA Knowledge Base was restricted to select tissues and cells primarily from the nervous and immune systems. The canonical pathways identified by IPA and reported in this study were selected based on their association to the central nervous system and immune response. Proteins that were identified in the schizophrenia and bipolar disorder network interactomes were selected based on their inferred association psychological disorders biological process. IPA used the right tailed Fisher’s exact test to determine statistical significance. Results are given with significance (P-value), ratio ([number of proteins from data set]/[total known proteins in pathway]), and/or z-score (number of standard deviations above or below the mean) when applicable.

**Human Genetic Analyses**

Data were collected from euthymic bipolar disorder patients enrolled in a long-term follow-up program at a bipolar outpatient unit at the Northern Stockholm psychiatric clinic. Age- and sex-matched healthy, population-based controls were randomly selected by Statistics Sweden and contacted by mail. The Regional Ethical Review Boards in Stockholm approved the study. After complete description of the study, written informed consent was obtained from all subjects. To assess the effect of *GRK3* RNA expression on CSF KYNA levels and occurrence of psychotic symptoms we utilized expression quantitative trait locus (eQTL) data. eQTL data from the HapMap2 sample (lymphoblastoid cell lines) was accessed through SNPexp v1.2. (<http://app3.titan.uio.no/biotools/tool.php?app=snpexp>) on 2013-12-21. We focused on *cis* acting eQTLs and searched for single nucleotide polymorphisms (SNPs) with a minor allele frequency (MAF) > 20 (to optimize power) and selected for directly genotyped SNPs in the genotyped subjects with genome-wide association study (GWAS) data that we were to evaluate. rs478655 (MAF = 0.29), located in the *GRK3* promoter, was chosen based on an additive model (β =0.051, *P* = 0.026) with the minor T allele associating with increasing *GRK3* expression (Mean T/T [n = 31] = 7.712, T/C [n = 104] = 7.584, and C/C [n = 112] = 7.575). Genotypes from a sample of 48 Swedish subjects, recruited as healthy volunteers in a large multi-center GWAS effort, were available from the Affymetrix 6.0 array (Affymetrix/ThermoFisher Scientific, Santa Clara, CA, USA) performed at the Broad Institute of MIT and Harvard (Cambridge, MA, USA). Procedures for genotyping and the quality control (QC) have been provided in prior publications ^24^. These subjects also underwent a lumbar puncture and cerebrospinal fluid (CSF) was utilized to measure KYNA levels and assess the effect of *GRK3* RNA expression, as predicted by the *cis* acting eQTL, on the CNS KYNA pool. Details regarding lumbar puncture and CSF KYNA analyses are described elsewhere ^25, 26^. Genotypes where then also collected from a sample of 70 Swedish bipolar disorder type 1 subjects, included in the same multi-center GWAS effort, to address the relationship between predicted *GRK3* RNA expression and occurrence of psychotic symptoms.

**Statistical Analyses**

All analyses were performed using the software programs Prism**^®^** 7 for Mac OS X (GraphPad Software, Inc. La Jolla, CA, USA), or IBM SPSS Statistics 22 for Mac OS X (IBM SPSS Inc., Chicago, IL, USA). Gaussian distribution was tested using D'Agostino & Pearson normality test. Parametric tests were used where appropriate. For D-amphetamine-induced hyperlocomotion, the amount of locomotion was compared in the 60 minutes following D-amphetamine administration as no differences existed between groups in the baseline session prior to treatment and to calculate the area under the curve, average locomotion in the last 30 minutes prior to D-amphetamine administration was used as the baseline. One Grk3^+/+^ mouse in the P2X7R membrane levels analysis was identified as an outlier by Grubbs´ test and was excluded. Data are reported as mean ± standard error of means (SEM). All tests were two-tailed with alpha set to 0.05. Further information about statistical tests and sample sizes are described in the figure legends. Unless otherwise stated, as no pre-specified effect sizes were available, sample sizes were chosen to reflect at least 80% power, assuming effect sizes such as in similar and previous experiments.

**References for Supplementary Materials and Methods:**

1. Deacon RM, Rawlins JN. T-maze alternation in the rodent. *Nat Protoc* 2006; **1**(1)**:** 7-12.

2. Olsson SK, Larsson MK, Erhardt S. Subchronic elevation of brain kynurenic acid augments amphetamine-induced locomotor response in mice. *J Neural Transm (Vienna)* 2012; **119**(2)**:** 155-163.

3. Gainetdinov RR, Bohn LM, Sotnikova TD, Cyr M, Laakso A, Macrae AD *et al.* Dopaminergic supersensitivity in G protein-coupled receptor kinase 6-deficient mice. *Neuron* 2003; **38**(2)**:** 291-303.

4. Gainetdinov RR, Premont RT, Bohn LM, Lefkowitz RJ, Caron MG. Desensitization of G protein-coupled receptors and neuronal functions. *Annu Rev Neurosci* 2004; **27:** 107-144.

5. Beaulieu JM, Sotnikova TD, Marion S, Lefkowitz RJ, Gainetdinov RR, Caron MG. An Akt/beta-arrestin 2/PP2A signaling complex mediates dopaminergic neurotransmission and behavior. *Cell* 2005; **122**(2)**:** 261-273.

6. Otte DM, Bilkei-Gorzó A, Filiou MD, Turck CW, Yilmaz O, Holst MI *et al.* Behavioral changes in G72/G30 transgenic mice. *Eur Neuropsychopharmacol* 2009; **19**(5)**:** 339-348.

7. Belforte JE, Zsiros V, Sklar ER, Jiang Z, Yu G, Li Y *et al.* Postnatal NMDA receptor ablation in corticolimbic interneurons confers schizophrenia-like phenotypes. *Nat Neurosci* 2010; **13**(1)**:** 76-83.

8. Clapcote SJ, Lipina TV, Millar JK, Mackie S, Christie S, Ogawa F *et al.* Behavioral phenotypes of Disc1 missense mutations in mice. *Neuron* 2007; **54**(3)**:** 387-402.

9. Chen YJ, Johnson MA, Lieberman MD, Goodchild RE, Schobel S, Lewandowski N *et al.* Type III neuregulin-1 is required for normal sensorimotor gating, memory-related behaviors, and corticostriatal circuit components. *J Neurosci* 2008; **28**(27)**:** 6872-6883.

10. Gresack JE, Risbrough VB. Corticotropin-releasing factor and noradrenergic signalling exert reciprocal control over startle reactivity. *Int J Neuropsychopharmacol* 2011; **14**(9)**:** 1179-1194.

11. Vinkers CH, Risbrough VB, Geyer MA, Caldwell S, Low MJ, Hauger RL. Role of dopamine D1 and D2 receptors in CRF-induced disruption of sensorimotor gating. *Pharmacol Biochem Behav* 2007; **86**(3)**:** 550-558.

12. Tufvesson-Alm M, Schwieler L, Schwarcz R, Goiny M, Erhardt S, Engberg G. Importance of kynurenine 3-monooxygenase for spontaneous firing and pharmacological responses of midbrain dopamine neurons: Relevance for schizophrenia. *Neuropharmacology* 2018; **138:** 130-139.

13. Grace AA, Bunney BS. The control of firing pattern in nigral dopamine neurons: burst firing. *J Neurosci* 1984; **4**(11)**:** 2877-2890.

14. Grace AA, Bunney BS. The control of firing pattern in nigral dopamine neurons: single spike firing. *J Neurosci* 1984; **4**(11)**:** 2866-2876.

15. Aeinehband S, Brenner P, Stahl S, Bhat M, Fidock MD, Khademi M *et al.* Cerebrospinal fluid kynurenines in multiple sclerosis; relation to disease course and neurocognitive symptoms. *Brain Behav Immun* 2016; **51:** 47-55.

16. Orhan F, Bhat M, Sandberg K, Stahl S, Piehl F, Karolinska Schizophrenia Project c *et al.* Tryptophan Metabolism Along the Kynurenine Pathway Downstream of Toll-like Receptor Stimulation in Peripheral Monocytes. *Scand J Immunol* 2016; **84**(5)**:** 262-271.

17. Agudelo LZ, Femenia T, Orhan F, Porsmyr-Palmertz M, Goiny M, Martinez-Redondo V *et al.* Skeletal muscle PGC-1alpha1 modulates kynurenine metabolism and mediates resilience to stress-induced depression. *Cell* 2014; **159**(1)**:** 33-45.

18. Zamboni L, Demartin.C. Buffered Picric Acid-Formaldehyde - a New Rapid Fixative for Electron Microscopy. *J Cell Biol* 1967; **35**(2p2)**:** A148-&.

19. van der Walt S, Schonberger JL, Nunez-Iglesias J, Boulogne F, Warner JD, Yager N *et al.* scikit-image: image processing in Python. *PeerJ* 2014; **2:** e453.

20. Ayers-Ringler JR, Oliveros A, Qiu Y, Lindberg DM, Hinton DJ, Moore RM *et al.* Label-Free Proteomic Analysis of Protein Changes in the Striatum during Chronic Ethanol Use and Early Withdrawal. *Front Behav Neurosci* 2016; **10:** 46.

21. Oliveros A, Starski P, Lindberg D, Choi S, Heppelmann CJ, Dasari S *et al.* Label-Free Neuroproteomics of the Hippocampal-Accumbal Circuit Reveals Deficits in Neurotransmitter and Neuropeptide Signaling in Mice Lacking Ethanol-Sensitive Adenosine Transporter. *J Proteome Res* 2017; **16**(4)**:** 1445-1459.

22. Starski P, Peyton L, Oliveros A, Heppelmann CJ, Dasari S, Choi DS. Proteomic Profile of a Chronic Binge Ethanol Exposure Model. *J Proteome Res* 2019; **18**(9)**:** 3492-3502.

23. Cox J, Hein MY, Luber CA, Paron I, Nagaraj N, Mann M. Accurate proteome-wide label-free quantification by delayed normalization and maximal peptide ratio extraction, termed MaxLFQ. *Mol Cell Proteomics* 2014; **13**(9)**:** 2513-2526.

24. Bergen SE, O'Dushlaine CT, Ripke S, Lee PH, Ruderfer DM, Akterin S *et al.* Genome-wide association study in a Swedish population yields support for greater CNV and MHC involvement in schizophrenia compared with bipolar disorder. *Mol Psychiatry* 2012; **17**(9)**:** 880-886.

25. Olsson SK, Sellgren C, Engberg G, Landen M, Erhardt S. Cerebrospinal fluid kynurenic acid is associated with manic and psychotic features in patients with bipolar I disorder. *Bipolar Disord* 2012; **14**(7)**:** 719-726.

26. Sellgren CM, Kegel ME, Bergen SE, Ekman CJ, Olsson S, Larsson M *et al.* A genome-wide association study of kynurenic acid in cerebrospinal fluid: implications for psychosis and cognitive impairment in bipolar disorder. *Mol Psychiatry* 2016; **21**(10)**:** 1342-1350.

**Supplementary Figures**

**Supplementary Figure 1. Similar cognitive function.** (**A**) % correct choices in the rewarded alternations T-maze (*Grk3*^+/+^ n=7, *Grk3*^-/-^ n=8; P=0.30). (**B**) % time interaction with a novel object (*Grk3*^+/+^ n=12, *Grk3*^-/-^ n=14, P=0.12, dotted line indicates chance performance). (**C**) % time interaction with an object moved to a new location (*Grk3*^+/+^ n=13, *Grk3*^-/-^ n=13, P=0.098, dotted line indicates chance performance). (**D**) Performance is similar during the acquisition phase (effect of: time F_(4,108)_=17.52, P<0.0010; genotype F_(1,27)_=0.18, P=0.67; interaction F_(4,108)_=0.68, P=0.61) and reversal learning phase (effect of: time F_(2,54)_=5.64, P=0.0060; genotype F_(1,27)_=1.13, P=0.30; interaction F_(2,54)_=0.12, P=0.89) of the Morris water maze. (**E**) No changes in memory retention are seen following acquisition in probe trial 1 as both genotypes spend significantly more time in the target quadrant (effect of: quadrant F_(3,108)_=86.56, P<0.0010; genotype F_(1,108)_<0.0001, P=0.99; interaction F_(3,108)_=1.72, P=0.17; ****p<0.0001 post hoc Bonferroni). (**F**) *Grk3*^-/-^ spend significantly more time in the target quadrant in the probe trial following the reversal phase showing successful learning versus Grk3^+/+^ animals who spend similar amounts of time in the right and opposite quadrant as in the target quadrant (effect of: quadrant F_(3,108)_=9.95, P<0.0010; genotype F_(1,108)_<0.0001, P=0.99; * p<0.05, ** p<0.01, ***p<0.001 post hoc Bonferroni). Morris Water Maze - *Grk3*^+/+^ n=13, *Grk3*^-/-^ n=16. Group comparisons in: (A) were performed using a Mann Whitney *U* test; (B), (C) were performed using unpaired t-test; (D) repeated measures 2-way ANOVA; (E), (F) 2-way ANOVA post hoc Bonferroni vs. target quadrant. Data are mean ± SEM. Tests were two-tailed. * P<0.05, ** P<0.01, ***p<0.001, **** p<0.0001.

**Supplementary Figure 2. A subtle anxiety phenotype is present in *Grk3*^-/-^ animals.** (**A**) The time spent in the light compartment (P=0.96) and (**B**) the number of entries into the light compartment (P=0.68) of the light-dark box test is similar between genotypes (*Grk3*^+/+^ n=13, *Grk3*^-/-^ n=13). (**C**) Time spent in the closed, center and open areas of the elevated-plus maze (effect of: area F_(2,69)_=120.40, P<0.0010; genotype F_(1,69)_=0.031, P=0.86; interaction F_(2,69)_=0.51, P=0.60) and (**D**) number of entries into the closed, center and open areas of the elevated-plus maze is also similar between genotypes (effect of: area F_(2,69)_=43.45, P<0.0010; genotype F_(1,69)_=3.14, P=0.081; interaction F_(2,69)_=0.33, P=0.72; *Grk3*^+/+^ n=12, *Grk3*^-/-^ n=13). (**E**) Total activity in the center of the open-field does not differ between genotypes (P=0.49, *Grk3*^+/+^ n=20, *Grk3*^-/-^ n=25). (**F**) Locomotion in the open-field is similar between genotypes and both habituate to the open-field over time with a decrease in forward locomotion (effect of: time F_(11,473)_=46.74, P<0.0010; genotype F_(1,43)_=2.07, P=0.16; interaction F_(11,473)_=1.13, P=0.33; *Grk3*^+/+^ n=20, *Grk3*^-/-^ n=25). (**G**) Although both genotypes habituate to the open-field (effect of time F_(11,473)_=9.73, P<0.0010), Grk3^-/-^ mice show decreased peripheral rearing compared to *Grk3*^+/+^ controls (effect of genotype _F(1,43)_=7.34, P=0.0097, interaction F_(11,473)_=1.33, P=0.20; *P=0.044 post hoc Bonferroni vs. controls, Grk3^+/+^ n=20, *Grk3*^-/-^ n=25) which is indicative of less vertical exploration. (**H**) In the first ten minutes of the open-field, *Grk3*^-/-^ mice spend more time in the corners than controls which could be indicative of a higher anxiety state – this is especially notable in the first two minutes (effect of: time F(9,387)=5.82, P<0.0010; genotype F(1,43)=2.56, P=0.12; interaction F(9,387)=3.69, P=0.00020; ****P<0.0001 post hoc Bonferroni versus control, *Grk3*^+/+^ n=20, *Grk3*^-/-^ n=25). Group comparisons in: (A),(B), (E) were performed using unpaired t-test; (C), (D) 2-way ANOVA; (F), (G), (H) repeated measures 2-way ANOVA post hoc Bonferroni. Data are mean ± SEM. Tests were two-tailed. * P<0.05, ** P<0.01, ***p<0.001, **** p<0.0001.


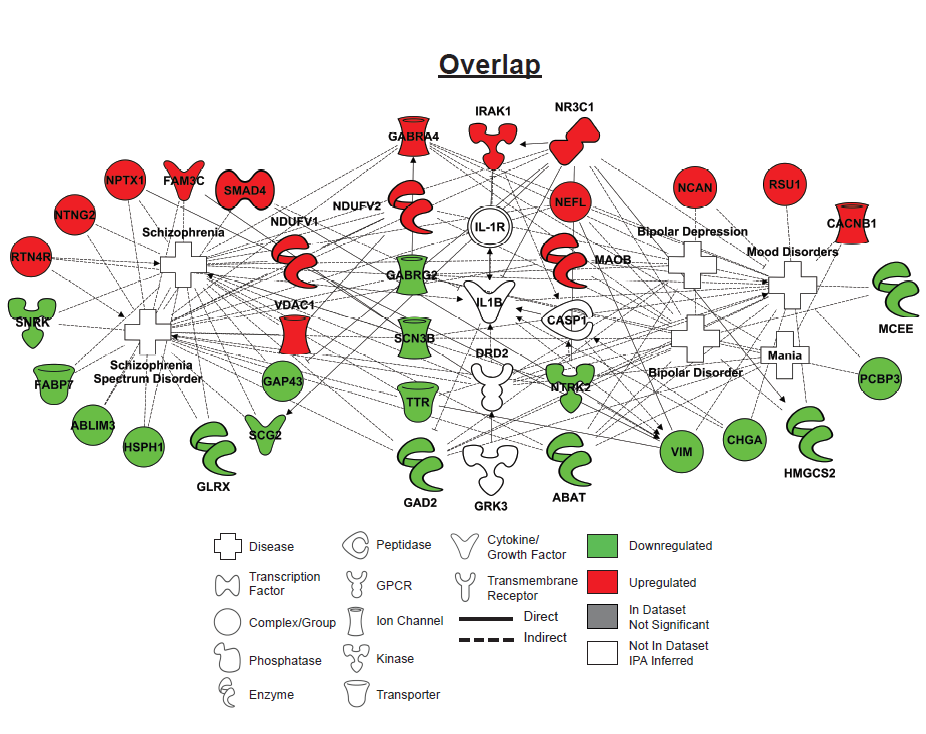


**Supplementary Figure 3. Comparative network of differentially expressed cortical proteins from *GRK3*^-/-^** **mice associated with schizophrenia and bipolar spectrum disorders.** Comparative pathway analysis of differentially expressed proteins found in our dataset or inferred by IPA to directly or indirectly interact with proteins associated with both schizophrenia-related disorders (left) and bipolar spectrum disorders (right). Proteins below “overlap” indicate proteins that associated with both schizophrenia spectrum (left node) and bipolar spectrum disorders (right node). Red and green colors indicate upregulated and downregulated protein expression, respectively, which met significance criteria for focus protein determination. Grey indicates a reference protein associated in the network that did not meet focus protein criteria. Clear proteins or disorders were inferred by Ingenuity Pathway Analysis to be associated but were not part of our data set. Solid line and dashed line indicates a direct or indirect interaction, respectively.

**Supplementary Figure 4. Transcript levels of kynurenine pathway enzymes are unchanged in hippocampus of *Grk3*^-/-^ mice**. Fold change mRNA expression compared to wildtype controls of the enzymes: kynurenine aminotransferase I-IV (KATI-IV, P=0.12, P>0.98, P>0.98, P>0.98, respectively), tryptophan 2,3-dioxygenase (TDO, P>0.98), indoleamine-2,3-dioxygenase 1 (IDO, P>0.98), and kynurenine 3-monooxygenase (KMO, P>0.98). *Grk3*^+/+^ n=6, *Grk3*^-/-^ n=5. Group comparisons were made using Mann Whitney *U* test with Bonferroni adjustment for multiple comparisons. Data are mean ± SEM. Tests were two-tailed.

A

B

C

D

**Supplementary Figure 5. Serum levels of kynurenine pathway metabolites.** (**A**) Tryptophan (P=0.19) (**B**) Kynurenine (P=0.54) (**C**) KYNA (P=0.87) and (**D**) Quinolinic acid (P=0.46). *Grk3*^+/+^ n=8, *Grk3*^-/-^ n=7. Group comparisons were made using Mann Whitney *U* test. Data are mean ± SEM. Tests were two-tailed.
