# Supplementary material for: GRK3 deficiency elicits brain immune activation and psychosis
Source: Mol Psychiatry. 2021 May 12;26(11):6820–32. doi: 10.1038/s41380-021-01106-0 (PMC8760053; doi:10.1038/s41380-021-01106-0)
Supplement: Supplementary file 2 — Supplementary Table 1 [file 41380_2021_1106_MOESM2_ESM.pdf]

Supplementary Table 1. Summary of behavioral tests performed in *Grk3*$^{-/-}$ mice

Test	Process	Schizophrenia Symptom Dimension	Grk3$^{-/-}$
Y-maze (continuous alternations)	working memory	cognitive[2]	=[1]
T-maze (rewarded alternations)	working memory	cognitive[2,3]	=
Novel Object Memory Location (spatial)	working memory	cognitive[2]	=
Novel Object Recognition (non-spatial)	working memory	cognitive[2,4]	=
Morris Water Maze (spatial)	reference memory	cognitive[2,3]	=
Light-Dark Box	anxiety		=
Elevated-Plus Maze	anxiety/blunted affect	negative[5,6]	=
Open-field (first 10 mins)			
center time	anxiety/blunted affect	negative[6]	=
corner time	anxiety/blunted affect	negative[6]	+
Open-field (60 mins)			
center time	anxiety/blunted affect	negative[6]	=
forward locomotion	general locomotion		=
horizontal activity	general locomotion		-
Novelty-induced hyperlocomotion	psychomotor agitation	positive[3]	=
Prepulse inhibition	sensorimotor gating	positive[3,7,8]	-
D-amphetamine-induced hyperlocomotion	psychostimulant sensitivity	positive[3,8]	+

[1] Increased same arm returns indicative of potential attentional deficits

[2] Amann LC, Gandal MJ, Halene TB, Ehrlichman RS, White SL, McCarren HS et al. Mouse behavioral endophenotypes for schizophrenia. *Brain Res Bull* 2010; **83**:147-161.
[3] Arguello PA, Gogos JA. Modeling madness in mice: one piece at a time. *Neuron* 2006; **52**:179-96.
[4] Rajagopal L, Massey BW, Huang M, Oyamada Y, Meltzer HY. The novel object recognition test in rodents in relation to cognitive impairment in schizophrenia. *Curr Pharm Des* 2014; **20**:5104-5114.
[5] Wallén-Mackenzie A, Nordenankar K, Fejgin K, Lagerström MC, Emilsson L, Fredriksson R et al. Restricted cortical and amygdaloid removal of vesicular glutamate transporter 2 in preadolescent mice impacts dopaminergic activity and neuronal circuitry of higher brain function. *J Neurosci* 2009; **29**:2238-2251.
[6] O'Tuathaigh CMP, Kirby BP, Moran PM, Waddington JL. Mutant mouse models: genotype-phenotype relationships to negative symptoms in schizophrenia. *Schizophr Bull* 2010; **36**: 271-288.
[7] Forrest AD, Coto CA, Siegel SJ. Animals models of psychosis: current state and future directions. *Curr Behav Neurosci Rep* 2014; **1**: 100-116.
[8] van den Buuse M. Modeling the positive symptoms of schizophrenia in genetically modified mice: pharmacology and methodology aspects. *Schizophr Bull* 2010; **36**: 246-270.
