# Supplementary material for: GRK3 deficiency elicits brain immune activation and psychosis
Source: Mol Psychiatry. 2021 May 12;26(11):6820–32. doi: 10.1038/s41380-021-01106-0 (PMC8760053; doi:10.1038/s41380-021-01106-0)
Supplement: Supplementary file 4 — Supplementary Table 3 [file 41380_2021_1106_MOESM4_ESM.pdf]

Supplementary Table 3.

Neuroproteomic Analysis of *Grk3*$^{-/-}$ Mice Identifies Significant Differential Expression of Proteins Associated With Schizophrenia and Mood Disorders

Symbol	Entrez Gene Name	UniProt/Swis	Exp Fold Chang	Exp p-value	Location	Type(s)
ABAT	4-aminobutyrate aminotransferase	P61922	-1.511	5.63E-04	Cytoplasm	enzyme
ABLIM3	actin binding LIM protein family, member 3	Q69ZX8	-3.335	8.92E-03	Cytoplasm	other
CACNB1	calcium channel, voltage-dependent, beta 1 subunit	Q8R3Z5	3.105	1.72E-04	Other	ion channel
CASP1	Caspase 1	P29452	Imputed by IPA		Cytoplasm	enzyme
CHGA	chromogranin A	P26339	-3.349	1.02E-04	Cytoplasm	other
DRD2	dopamine receptor D2	P61168	Imputed by IPA		Plasma Membrane	receptor
FABP7	fatty acid binding protein 7, brain	P51880	-2.788	7.36E-14	Cytoplasm	transporter
FAM3C	FAM3 metabolism regulating signaling molecule C	Q91VU0	1,667	6,67×10^−03	Extracellular	cytokine
GABRA4	gamma-aminobutyric acid (GABA) A receptor, alpha 4	Q9D6F4	1.697	1.07E-02	Plasma Membrane	ion channel
GABRG2	gamma-aminobutyric acid (GABA) A receptor, gamma 2	P22723-2	-2.807	2.37E-03	Plasma Membrane	
GAD2	glutamate decarboxylase 2 (pancreatic islets and brain, 65kDa)	P48320	-1.534	4.34E-03	Cytoplasm	enzyme
GAP43	growth associated protein 43	P06837	-1.697	4.98E-03	Plasma Membrane	receptor
GLRX	glutaredoxin (thioltransferase)	Q9QUH0	-1.638	3.70E-02	Cytoplasm	enzyme
GRK3	G protein-coupled receptor kinase 3	Q8BVT9	Imputed by IPA		Cytoplasm	kinase
HMGCS2	3-hydroxy-3-methylglutaryl-CoA synthase 2 (mitochondrial)	P54869	-2.637	6.51E-22	Cytoplasm	enzyme
HSPH1	heat shock 105kDa/110kDa protein 1	Q61699	-5.613	2.51E-02	Cytoplasm	other
IL-1R	interleukin 1 receptor	P13504	Imputed by IPA		Plasma Membrane	
IL1B	interleukin 1 beta	P10749	Imputed by IPA		Extracellular Space	cytokine
IRAK1	interleukin-1 receptor-associated kinase 1	P51617-4	1.940	3.58E-02	Plasma Membrane	kinase
MAOB	monoamine oxidase B	Q8BW75	1.932	9.78E-05	Cytoplasm	enzyme
MCEE	methylmalonyl CoA epimerase	Q9D1I5	-1.951	2.37E-02	Cytoplasm	enzyme
NCAN	neurocan	P55066	2.308	4.22E-08	Extracellular Space	other
NDUFV1	NADH dehydrogenase (ubiquinone) flavoprotein 1, 51kDa	Q91YT0	1.576	5.60E-06	Cytoplasm	enzyme
NDUFV2	NADH dehydrogenase (ubiquinone) flavoprotein 2, 24kDa	Q9D6J6	1.655	5.48E-03	Cytoplasm	enzyme
NEFL	neurofilament, light polypeptide	P08551	1.762	1.33E-03	Cytoplasm	other
NPTX1	neuronal pentraxin I	Q62443	1.502	2.41E-02	Extracellular Space	other
NR3C1	nuclear receptor subfamily 3, group C, member 1 (glucocorticoid receptor)	P06537-3	1.632	3.60E-06	Nucleus	ligand-dependent nuclear receptor
NTNG2	netrin G2	Q8R4F1-2	1.731	1.84E-02	Plasma Membrane	other
NTRK2	neurotrophic tyrosine kinase, receptor, type 2	P15209-2	-1.506	3.69E-02	Plasma Membrane	kinase
PCBP3	poly(rC) binding protein 3	P57722-2	-2.324	7.84E-19	Nucleus	other
RSU1	Ras suppressor protein 1	Q01730	1.800	2.60E-10	Cytoplasm	other
RTN4R	reticulon 4 receptor	Q99PI8	2.184	1.45E-04	Plasma Membrane	other
SCG2	secretogranin II	Q03517	-2.799	1.26E-04	Extracellular Space	cytokine
SCN3B	sodium channel, voltage gated, type III beta subunit	Q8BHK2	-2.148	4.38E-03	Plasma Membrane	ion channel
SLC6A11	solute carrier family 6 (neurotransmitter transporter), member 11	P31650	-1.518	1.23E-02	Plasma Membrane	transporter
SMAD4	SMAD family member 4	P97471	3.876	1.30E-18	Nucleus	transcription regulator
SNRK	SNF related kinase	Q8VDU5	-1.580	3.21E-03	Cytoplasm	kinase
TTR	transthyretin	P07309	-1.814	1.22E-07	Extracellular Space	transporter
VDAC1	voltage-dependent anion channel 1	Q60932-2	1.838	4.69E-03	Cytoplasm	ion channel
VIM	vimentin	P20152	-1.677	4.33E-03	Cytoplasm	other
