# Supplementary material for: GRK3 deficiency elicits brain immune activation and psychosis
Source: Mol Psychiatry. 2021 May 12;26(11):6820–32. doi: 10.1038/s41380-021-01106-0 (PMC8760053; doi:10.1038/s41380-021-01106-0)
Supplement: Supplementary file 5 — Supplementary Table 4 [file 41380_2021_1106_MOESM5_ESM.pdf]

Supplementary Table 4 . Neuroproteomic Analysis of *Grk3*$^{-/-}$ Mice Identifies Significant Differential Expression of Proteins Associated With Schizophrenia Spectrum Disorders

Symbol	Entrez Gene Name	UniProt/Swiss-Prot Accession	Exp Fold Change	Exp p-value	Type(s)	Location
AKAP5	A kinase (PRKA) anchor protein 5	D3YVF0	3.307	7.62E-03	other	Plasma Membrane
Ank2	ankyrin 2, brain	Q8C8R3	27.094	3.41E-02	other	Plasma Membrane
BDNF	brain derived neurotrophic factor	P21237	Imputed by IPA		growth factor	Extracellular Space
C1QA	complement C1q A chain	P98086	2.034	7.49E-04	other	Extracellular Space
C1QB	complement C1q B chain	P14106	1.779	5.18E-06	other	Extracellular Space
C1QC	complement C1q C chain	Q02105	1.874	6.85E-03	other	Extracellular Space
DRD2	dopamine receptor D2	P61168	Imputed by IPA		G-protein coupled receptor	Plasma Membrane
ENPP2	ectonucleotide pyrophosphatase/phosphodiesterase 2	Q9R1E6	-4.445	3.86E-02	enzyme	Plasma Membrane
FABP7	fatty acid binding protein 7	P51880	-2.788	7.36E-14	transporter	Cytoplasm
GABRA4	gamma-aminobutyric acid type A receptor subunit alpha4	Q9D6F4	1.697	1.07E-02	ion channel	Plasma Membrane
GABRG2	gamma-aminobutyric acid type A receptor subunit gamma2	P22723-2	-2.807	2.37E-03	ion channel	Plasma Membrane
GAD2	glutamate decarboxylase 2	P48320	-1.534	4.34E-03	enzyme	Cytoplasm
GNAL	G protein subunit alpha L	Q8CGK7	-3.440	5.65E-05	enzyme	Cytoplasm
GRK3	G protein-coupled receptor kinase 3	Q8BVT9	Imputed by IPA		kinase	Cytoplasm
HDAC3	histone deacetylase 3	O88895	-2.381	2.84E-02	transcription regulator	Nucleus
IKBKG	inhibitor of nuclear factor kappa B kinase regulatory subunit gamma	O88522	-1.919	9.03E-03	kinase	Nucleus
IL1B	interleukin 1 beta	P10749	Imputed by IPA		cytokine	Extracellular Space
IRAK1	interleukin 1 receptor associated kinase 1	P51617-4	1.940	3.58E-02	kinase	Plasma Membrane
KIF1A	kinesin family member 1A	P33173	2.628	2.19E-04	other	Cytoplasm
L1CAM	L1 cell adhesion molecule	P11627	-2.610	3.66E-02	other	Plasma Membrane
MAOB	monoamine oxidase B	Q8BW75	1.932	9.78E-05	enzyme	Cytoplasm
MARCKSL1	MARCKS like 1	P28667	-2.894	7.19E-07	other	Cytoplasm
NAT8L	N-acetyltransferase 8 like	Q3UGX3	1.939	3.89E-03	enzyme	Cytoplasm
NCAN	neurocan	P55066	2.308	4.22E-08	other	Extracellular Space
Nedd4	neural precursor cell expressed, developmentally down-regulated 4	P46935	-4.735	1.30E-02	enzyme	Cytoplasm
NKIRAS1	NFKB inhibitor interacting Ras like 1	Q8CEC5	1.510	2.83E-02	enzyme	Other
NTRK2	neurotrophic receptor tyrosine kinase 2	P15209-2	-1.506	3.69E-02	kinase	Plasma Membrane
RTN4R	reticulon 4 receptor	Q99PI8	2.184	1.45E-04	other	Plasma Membrane
S100B	S100 calcium binding protein B	P50114	-1.720	8.07E-02	other	Cytoplasm
SERPINA1	serpin family A member 1	Q00898	-1.942	1.33E-02	other	Extracellular Space
SERPINA3	serpin family A member 3	Q03734	-4.549	6.38E-05	other	Extracellular Space
SLC17A6	solute carrier family 17 member 6	Q8BLE7	-1.803	1.05E-02	transporter	Plasma Membrane
SLC32A1	solute carrier family 32 member 1	O35633-2	-1.820	3.96E-04	transporter	Plasma Membrane
SLC6A11	solute carrier family 6 member 11	P31650	-1.518	1.23E-02	transporter	Plasma Membrane
TH	tyrosine hydroxylase	P24529	-3.355	3.80E-13	enzyme	Cytoplasm
TTR	transthyretin	P07309	-1.814	1.22E-07	transporter	Extracellular Space
XPO7	exportin 7	Q9EPK7-2	-3326.592	2.66E-03	transporter	Nucleus
