# Supplementary material for: GRK3 deficiency elicits brain immune activation and psychosis
Source: Mol Psychiatry. 2021 May 12;26(11):6820–32. doi: 10.1038/s41380-021-01106-0 (PMC8760053; doi:10.1038/s41380-021-01106-0)
Supplement: Supplementary file 3 — Supplementary Table 2 [file 41380_2021_1106_MOESM3_ESM.pdf]

Supplementary Table 2.
Full list of differentially expressed proteins in GRK3-/- mice relative to GRK3+/+ mice
identified by LC-MS/MS and subjected to Ingenuity Pathway Analysis

SwissProti	Protein.Groups	Protein.Group	Gaussian.p.value	Gaussian.FDR	Intensity.lane.a	Intensity.lane.b	Intensity.lane.c	Intensity.lane.d	Intensity.lane.e	Intensity.lane.f	Grp1.Rates	Grp2.Rates	Log2.Fold.Change	Grp1.CV	Grp2.CV	FC KO/WT	Log2_KO/WT	FC WT/KO	Log2_WT/KO	
**Q3UYH7**	**sp|Q3UYH7|ARBK2_MOUSE-GRK3-Beta-adrenergic receptor kinase 2 OS=Mus musculus GN=Adrbk2 PE=2 SV=2**	**−81716905689**	**0,049**	**0**	**25,7508401370558**	**24,7331922094988**	**24,8955478776018**	**0**	**0**	**0**	**81716905689,0781**	**1**	**36,2499155245608**	**0,0217591325754411**	**NaN**	**0,0000000000122373698755**	**-36.2499155245608**	**81716905689,0781**	**36.2499155245608**	
Q8BL80-3	sp|Q8BL80-3|RHG22_MOUSE Isoform 3 of Rho GTPase-activating protein 22 OS=Mus musculus GN=Arhgap22;sp|Q8BL80-2|RHG22_MOUSE Isoform 2 of Rho GTPase-activating protein 22 OS=Mus musculus GN=Arhgap22;sp|Q8BL80|RHG22_MOUSE Rho GTPase-activating protein 22 OS	−2947409,50	0,0558505414023197	0,348700973074456	32,4047952999906	32,2182618919671	21,6695873010279	21,0597463648342	0	20,543586508048	3105558949314,06	1053657,10127585	21,4910160850216	0,213627785503425	0,866225336546409	0,000000339280985636604	-21.4910160850216	2947409,49930827	21.4910160850216	1
P52840	sp|P52840|ST1A1_MOUSE Sulfotransferase 1A1 OS=Mus musculus GN=Sult1a1 PE=2 SV=1	−1535290,39	0,0588413819231506	0,359463031694725	21,9979846941957	22,2295117621056	21,0988721291394	0	0	22,5936782818681	2863917257,66371	1865,39124693559	20,5500801282694	0,0274283658670816	1,73205080756888	0,000000651342577004936	-20.5500801282694	1535290,39142243	20.5500801282694	2
Q91W43	sp|Q91W43|GCSP_MOUSE Glycine dehydrogenase (decarboxylating), mitochondrial OS=Mus musculus GN=Gldc PE=1 SV=1	−113807,20	0,089773798073022	0,441176105282424	26,3722038170175	22,1382342375959	25,5630102279109	22,490566066182	16,6560990645485	0	52872512458,7137	464579,68701319	16,7962322824323	0,0910287922979779	0,894415952723609	0,0000087867904400413	-16.7962322824323	113807,198068934	16.7962322824323	3
Q922D4-2	sp|Q922D4-2|PP6R3_MOUSE Isoform 2 of Serine/threonine-protein phosphatase 6 regulatory subunit 3 OS=Mus musculus GN=Ppp6r3;sp|Q922D4|PP6R3_MOUSE Serine/threonine-protein phosphatase 6 regulatory subunit 3 OS=Mus musculus GN=Ppp6r3 PE=1 SV=1;sp|Q922D4-4|	−89207,71	0,0686765105651004	0,389166893202235	22,1710415493333	21,2911396120514	21,8248730561356	9,47447434158858	0	21,616411665443	2826631072,82009	31685,9507940738	16,4448807648923	0,0203686773995933	1,04554127197596	0,0000112097935591082	-16.4448807648923	89207,7088420131	16.4448807648923	4
Q9D2V7	sp|Q9D2V7|CORO7_MOUSE Coronin-7 OS=Mus musculus GN=Coro7 PE=2 SV=2	−26372,45	0,176213981328786	0,592470563889896	26,6737413947028	22,6504567909222	25,6624905516586	23,2006453878741	21,2458172816804	0	71686115053,3077	2718219,30476517	14,6867442318469	0,0837315174440962	0,868534607557222	0,0000379183514512375	-14.6867442318469	26372,4545431778	14.6867442318469	5
A6X919-2	sp|A6X919-2|D19L1_MOUSE Isoform 2 of Probable C-mannosyltransferase DPY19L1 OS=Mus musculus GN=Dpy19l1;sp|A6X919|D19L1_MOUSE Probable C-mannosyltransferase DPY19L1 OS=Mus musculus GN=Dpy19l1 PE=2 SV=1;sp|A6X919-3|D19L1_MOUSE Isoform 3 of Probable C-mann	−6397,92	0,324790252447157	0,726844368394785	26,1869977779283	26,76973984521	26,7207981765655	26,8660517354712	26,5203006373062	0	342377100604,303	53513835,7684313	12,6433866497096	0,0121707695342493	0,866079888522644	0,000156300861459421	-12.6433866497096	6397,91739253864	12.6433866497096	6

Q9EPK2-3	sp|Q9EPK2-3|XRP2_MOUSE Isoform 3 of Protein XRP2 OS=Mus musculus GN=Rp2;sp|Q9EPK2|XRP2_MOUSE Protein XRP2 OS=Mus musculus GN=Rp2 PE=1 SV=3;sp|Q9EPK2-2|XRP2_MOUSE Isoform 2 of Protein XRP2 OS=Mus musculus GN=Rp2;sp|Q9EPK2-4|XRP2_MOUSE Isoform 4 of Prote	−6188,17	0,26540909400193	0,685262729052764	25,0609738563947	23,5068458156028	24,5525942656835	23,9091561309391	0	23,0200748010908	38482572466,9183	6218734,43731305	12,5952966124227	0,0325106146422957	0,866491527432999	0,000161598719593369	-12.5952966124227	6188,16784264317	12.5952966124227	7
P97367-3	sp|P97367-3|MEIS2_MOUSE Isoform Meis2B of Homeobox protein Meis2 OS=Mus musculus GN=Meis2;sp|P97367-2|MEIS2_MOUSE Isoform Meis2A of Homeobox protein Meis2 OS=Mus musculus GN=Meis2;sp|P97367-4|MEIS2_MOUSE Isoform Meis2D of Homeobox protein Meis2 OS=Mus m	−6004,21	0,257790044669085	0,67968190098123	24,3708201043853	23,8999487036671	23,9198035652238	23,5262499539463	22,5636759051768	0	28226416293,8682	4701107,22962933	12,5517577633215	0,011067008441449	0,866591820640627	0,000166549914827501	-12.5517577633215	6004,20601256819	12.5517577633215	8
Q80SY6	sp|Q80SY6|ADAL_MOUSE Adenosine deaminase-like protein OS=Mus musculus GN=Adal PE=2 SV=1;sp|Q80SY6-3|ADAL_MOUSE Isoform 3 of Adenosine deaminase-like protein OS=Mus musculus GN=Adal;sp|Q80SY6-4|ADAL_MOUSE Isoform 4 of Adenosine deaminase-like protein OS=	−5753,78	0,230432037368989	0,654468548759245	23,5630146461116	22,8085879999257	22,8906559375229	21,7674627603317	21,5219605614353	0	10635034481,4356	1848357,1470878	12,4902935433384	0,0179282247264397	0,866067182836993	0,000173798886154462	-12.4902935433384	5753,77680563075	12.4902935433384	9
Q9EPL2-2	sp|Q9EPL2-2|CSTN1_MOUSE Isoform 2 of Calsyntenin-1 OS=Mus musculus GN=Clstn1;sp|Q9EPL2|CSTN1_MOUSE Calsyntenin-1 OS=Mus musculus GN=Clstn1 PE=1 SV=1	−5290,41	0,244077832579857	0,667793418975268	23,754344369546	23,2345232311874	22,7962312002305	0	21,334360086982	22,7297871435156	12659827149,8811	2392977,76899451	12,3691630925523	0,0206190595220413	0,867327188425785	0,000189021361876729	-12.3691630925523	5290,40733846874	12.3691630925523	10
Q8BHB9	sp|Q8BHB9|CLIC6_MOUSE Chloride intracellular channel protein 6 OS=Mus musculus GN=Clic6 PE=2 SV=1	−5211,00	0,233332526450679	0,657279264635888	23,3953782803666	22,0605658022871	23,2303027245149	0	21,3878092046391	21,6228591796111	8777132549,79026	1684348,60324989	12,3473432209	0,0317835304583111	0,866064199181293	0,00019190192169197	-12.3473432209	5210,9952374794	12.3473432209	11
Q9D394-4	sp|Q9D394-4|RUFY3_MOUSE Isoform 4 of Protein RUFY3 OS=Mus musculus GN=Rufy3;sp|Q9D394-2|RUFY3_MOUSE Isoform 2 of Protein RUFY3 OS=Mus musculus GN=Rufy3	−5119,32	0,244561747268372	0,668311722366731	22,7276309950859	23,5928619631574	23,3098271341832	0	22,1514329050445	21,8565573461608	12023232990,3996	2348600,28141392	12,3217360659837	0,0190068530181398	0,86608372435314	0,000195338498662485	-12.3217360659837	5119,31855137192	12.3217360659837	12
O35409	sp|O35409|FOLH1_MOUSE Glutamate carboxypeptidase 2 OS=Mus musculus GN=Folh1 PE=2 SV=2	−4403,23	0,25464049462036	0,676705623584201	23,5733620117965	23,1077379050397	22,6114053377269	22,5875559426552	0	21,5346685350988	10742801631,1567	2439754,94354273	12,1043464181617	0,0208273616444214	0,866764814251341	0,000227106021995869	-12.1043464181617	4403,22978321636	12.1043464181617	13
Q8R2Y9	sp|Q8R2Y9|SOSB1_MOUSE SOSS complex subunit B1 OS=Mus musculus GN=Nabp2 PE=2 SV=1	−4223,71	0,300621451567527	0,714034939123499	24,4368206009609	24,2617206040686	24,7320198340803	24,197055235212	0	24,1881015331326	42673914962,5027	10103430,61963	12,0442935128308	0,0097108748857012	0,866025448268339	0,000236758934082046	-12.0442935128308	4223,70544907701	12.0442935128308	14
Q91W40	sp|Q91W40|KLC3_MOUSE Kinesin light chain 3 OS=Mus musculus GN=Klc3 PE=1 SV=1;sp|Q6P597-2|KLC3_HUMAN Isoform 2 of Kinesin light chain 3 OS=Homo sapiens GN=KLC3;sp|Q6P597|KLC3_HUMAN Kinesin light chain 3 OS=Homo sapiens GN=KLC3 PE=1 SV=2;sp|Q6P597-3|KLC3	−4163,45	0,253257770770756	0,674868697133729	22,7962312002305	22,9159360119671	23,0551430075932	21,7796036535179	0	21,9854071197575	9017533988,71956	2165879,17930245	12,0235644741028	0,00565290077913837	0,866054129177428	0,000240185308091086	-12.0235644741028	4163,45199441078	12.0235644741028	15
Q3V3R1	sp|Q3V3R1|C1TM_MOUSE Monofunctional C1-tetrahydrofolate synthase, mitochondrial OS=Mus musculus GN=Mthfd1l PE=1 SV=2	−3837,76	0,0957050645414692	0,452812129597592	25,9600094693325	22,1096485360386	24,8222977128199	6,40719940145827	21,0533396385392	20,6734853937543	35660824588,156	9292096,9939161	11,90604822891	0,0814137586358234	0,520325860658918	0,000260568764217591	-11.90604822891	3837,7585394884	11.90604822891	16
Q9DC28	sp|Q9DC28|KC1D_MOUSE Casein kinase I isoform delta OS=Mus musculus GN=Csnk1d PE=1 SV=2;sp|Q9DC28-2|KC1D_MOUSE Isoform 2 of Casein kinase I isoform delta OS=Mus musculus GN=Csnk1d	−3782,73	0,308764460627522	0,71853230316343	25,2054719742707	23,8091764130591	24,1940816395417	24,457410707647	24,0367134446898	0	39632289285,4759	10477158,3552247	11,8852131109055	0,0295535071783353	0,866123163181039	0,000264359151189994	-11.8852131109055	3782,73267824689	11.8852131109055	17
Q9EPK7-2	sp|Q9EPK7-2|XPO7_MOUSE Isoform 2 of Exportin-7 OS=Mus musculus GN=Xpo7;sp|Q9EPK7|XPO7_MOUSE Exportin-7 OS=Mus musculus GN=Xpo7 PE=2 SV=3	−3326,59	0,00265597123849248	0,0484154757089051	24,7860991569549	20,7818141292922	22,242612719966	9,63004269562842	16,9632605816636	16,8881119694319	6555096454,75275	1970514,20070401	11,6998291473177	0,0896493848868297	0,290628261376117	0,000300607964246704	-11.6998291473177	3326,59183699909	11.6998291473177	18

Q8BX09	sp|Q8BX09|RBBP5_MOUSE Retinoblastoma-binding protein 5 OS=Mus musculus GN=Rbbp5 PE=1 SV=2;sp|Q8BX09-2|RBBP5_MOUSE Isoform 2 of Retinoblastoma-binding protein 5 OS=Mus musculus GN=Rbbp5	−2837,27	0,308814306609267	0,71853230316343	22,8859967988134	24,4487515518704	23,3041913196319	23,2593273389152	0	23,5278189761452	16828054199,1018	5931071,45434628	11,470287996645	0,0343587880362599	0,866068181695862	0,000352451411444994	-11.470287996645	2837,27052163066	11.470287996645	19
P48771	sp|P48771|CX7A2_MOUSE Cytochrome c oxidase subunit 7A2, mitochondrial OS=Mus musculus GN=Cox7a2 PE=1 SV=2	−2430,65	0,344916948672549	0,730213633407042	24,8090721097822	23,9175054815775	24,1418352095624	24,35916318947	25,1215085600284	0	35382066534,0692	14556631,9205152	11,2471260108281	0,0190928981419317	0,866333706219254	0,000411412711196467	-11.2471260108281	2430,64925507967	11.2471260108281	20
Q99LJ6	sp|Q99LJ6|GPX7_MOUSE Glutathione peroxidase 7 OS=Mus musculus GN=Gpx7 PE=2 SV=1	−2398,58	0,306565719297744	0,71837258919956	21,7584100724818	23,8296632028795	23,2674949779094	0	23,0507037791687	22,4569741763383	9286764806,76205	3871784,17217985	11,2279618492678	0,0466670078333857	0,866246496539436	0,000416914205618802	-11.2279618492678	2398,57502220572	11.2279618492678	21
Q8K3K8	sp|Q8K3K8|OPTN_MOUSE Optineurin OS=Mus musculus GN=Optn PE=1 SV=1;sp|Q96CV9-3|OPTN_HUMAN Isoform 3 of Optineurin OS=Homo sapiens GN=OPTN;sp|Q96CV9-2|OPTN_HUMAN Isoform 2 of Optineurin OS=Homo sapiens GN=OPTN;sp|Q96CV9|OPTN_HUMAN Optineurin OS=Homo sapi	−2351,12	0,293921991624233	0,711969538636193	23,750582547653	22,1779631888277	21,5621146118181	22,2047387177875	0	21,9979846941957	5892149936,92486	2506107,06584843	11,1991303768524	0,0501648580267671	0,866053823822097	0,000425329818941501	-11.1991303768524	2351,11660519983	11.1991303768524	22
Q8BH65-2	sp|Q8BH65-2|DEN6A_MOUSE Isoform 2 of Protein DENND6A OS=Mus musculus GN=Dennd6a;sp|Q8BH65|DEN6A_MOUSE Protein DENND6A OS=Mus musculus GN=Dennd6a PE=2 SV=1	−2306,82	0,309962915073159	0,719371512809526	22,47968402626	23,1968846809395	23,2867314959679	23,0297539046255	0	22,7026757793855	9626318881,56528	4172989,43577178	11,1716872311734	0,0192406612166915	0,866091848382667	0,000433497943202691	-11.1716872311734	2306,81601996074	11.1716872311734	23
Q99LG4	sp|Q99LG4|TTC5_MOUSE Tetratricopeptide repeat protein 5 OS=Mus musculus GN=Ttc5 PE=1 SV=2	−2290,50	0,323963441920593	0,726844368394785	23,393552816274	23,1763774222025	23,6863570906192	23,7519013740161	23,29481600134	0	14812873089,9525	6467105,05526333	11,1614438244752	0,0109281078132118	0,86614801413739	0,00043658681310447	-11.1614438244752	2290,49520046019	11.1614438244752	24
Q3UKJ7	sp|Q3UKJ7|SMU1_MOUSE WD40 repeat-containing protein SMU1 OS=Mus musculus GN=Smu1 PE=2 SV=2;sp|Q3UKJ7-2|SMU1_MOUSE Isoform 2 of WD40 repeat-containing protein SMU1 OS=Mus musculus GN=Smu1	−2225,34	0,475821701066886	0,79947899324683	0	22,7607929420049	23,2262234311461	0	22,8640191705498	0	4542578,94236115	2041,29506972972	11,1198111315607	0,866158457719016	1,73205080756888	0,000449369200982711	-11.1198111315607	2225,34165183802	11.1198111315607	25
Q3U3E2	sp|Q3U3E2|F117B_MOUSE Protein FAM117B OS=Mus musculus GN=Fam117b PE=1 SV=1	−1889,83	0,313048936628174	0,720917870804304	22,0698626041024	23,075311364659	22,3165440216705	0	22,5602574624914	22,2687401280658	5835579020,95021	3087891,94265927	10,8840378275752	0,0233024638168759	0,866080334952031	0,000529149195233838	-10.8840378275752	1889,82617569339	10.8840378275752	26
Q9ER41	sp|Q9ER41|TOR1B_MOUSE Torsin-1B OS=Mus musculus GN=Tor1b PE=1 SV=2	−1497,55	0,326615941370883	0,726927682919497	22,5167234850316	22,026190417643	22,1014692905163	0	22,4867335334843	22,2228918150151	4443883075,48315	2967434,84638572	10,5483887885456	0,0118917797454498	0,86607064095369	0,000667757183522002	-10.5483887885456	1497,55034416197	10.5483887885456	27
Q9WTK3	sp|Q9WTK3|GPAA1_MOUSE Glycosylphosphatidylinositol anchor attachment 1 protein OS=Mus musculus GN=Gpaa1 PE=1 SV=3;sp|O43292-2|GPAA1_HUMAN Isoform 2 of Glycosylphosphatidylinositol anchor attachment 1 protein OS=Homo sapiens GN=GPAA1;sp|O43292|GPAA1_HUMA	−1449,84	0,330223446034441	0,72953198881961	21,7962886665004	22,6114053377269	22,2626581889544	22,311282263451	0	22,5214498237658	4482517784,87846	3091738,24354391	10,5016754105642	0,0184026466551552	0,866053950469775	0,00068973251014725	-10.5016754105642	1449,83741564757	10.5016754105642	28
Q80X50-2	sp|Q80X50-2|UBP2L_MOUSE Isoform 2 of Ubiquitin-associated protein 2-like OS=Mus musculus GN=Ubap2l;sp|Q80X50-3|UBP2L_MOUSE Isoform 3 of Ubiquitin-associated protein 2-like OS=Mus musculus GN=Ubap2l;sp|Q80X50-4|UBP2L_MOUSE Isoform 4 of Ubiquitin-associat	−1387,19	0,337883470346345	0,729620681454069	23,0956367782024	21,7460236150581	22,0932341720107	22,502151721827	22,7276309950859	0	4895741202,32483	3529243,99048482	10,4379524085222	0,0314101482569239	0,866057687093666	0,00072088042333792	-10.4379524085222	1387,19261562086	10.4379524085222	29
Q922R1	sp|Q922R1|CP070_MOUSE UPF0183 protein C16orf70 homolog OS=Mus musculus PE=2 SV=2	−1379,60	0,36584520245375	0,739315231400715	24,2686184132209	22,451234435472	22,7620598330115	0	24,5712773674685	23,2219874868566	11442926013,0757	8294376,26573323	10,4300348880491	0,0419696507492245	0,867060163805144	0,000724847495846372	-10.4300348880491	1379,6005445702	10.4300348880491	30
Q9CXU1	sp|Q9CXU1|MED31_MOUSE Mediator of RNA polymerase II transcription subunit 31 OS=Mus musculus GN=Med31 PE=1 SV=2	−1335,83	0,318956464421104	0,722907157640623	21,8402680937608	21,5741688711869	21,504041661994	21,6429764340917	0	21,6835797836826	2499840772,13762	1871377,60715023	10,3835197943217	0,00819689450756265	0,866026544656613	0,000748598721969804	-10.3835197943217	1335,82915740048	10.3835197943217	31
Q8BSI6	sp|Q8BSI6|R3HC1_MOUSE R3H and coiled-coil domain-containing protein 1 OS=Mus musculus GN=R3hcc1 PE=2 SV=2	−1299,69	0,366849150459238	0,739315231400715	22,9874166300028	22,4725237041215	23,6723843197498	23,6274438234	0	23,9952333945482	10184249712,262	7835895,63512298	10,3439538956934	0,0261210324639999	0,866102880940758	0,000769413148392113	-10.3439538956934	1299,69185227697	10.3439538956934	32
Q0KL02-3	sp|Q0KL02-3|TRIO_MOUSE Isoform 3 of Triple functional domain protein OS=Mus musculus GN=Trio;sp|Q0KL02|TRIO_MOUSE Triple functional domain protein OS=Mus musculus GN=Trio PE=1 SV=3;sp|Q0KL02-4|TRIO_MOUSE Isoform 4 of Triple functional domain protein OS=	−1296,65	0,339864758104194	0,729620681454069	22,4379044725108	21,7767731508675	22,3236330895835	22,3038750494383	0	22,7318219676303	4289503823,41894	3308147,77352567	10,3405713817388	0,0159321786629781	0,866142693116107	0,000771219215487007	-10.3405713817388	1296,64819018873	10.3405713817388	33

Q5DQR4-5	sp|Q5DQR4-5|STB5L_MOUSE Isoform 5 of Syntaxin-binding protein 5-like OS=Mus musculus GN=Stxbp5l;sp|Q5DQR4-4|STB5L_MOUSE Isoform 4 of Syntaxin-binding protein 5-like OS=Mus musculus GN=Stxbp5l;sp|Q5DQR4-2|STB5L_MOUSE Isoform 2 of Syntaxin-binding protein	−1261,62	0,307603191309542	0,71837258919956	21,2173839147839	20,9472431782709	21,2326685501382	0	20,7310314969917	21,2458172816804	1505561689,39475	1193359,4157266	10,3010574883566	0,00759780531364433	0,866220750601011	0,000792634020998729	-10.3010574883566	1261,61629895723	10.3010574883566	34
Q9C5J4	sp|Q9C5J4|Y3210_ARATH BTB/POZ domain-containing protein At3g22104 OS=Arabidopsis thaliana GN=At3g22104 PE=2 SV=1	−1256,56	0,44924450674337	0,786343703431612	27,3929021595667	27,6030325495586	21,8627446203422	27,7726589193272	27,6776121109565	0	133793014731,69	106475309,096064	10,2952681621861	0,12705891061926	0,866029220490049	0,000795821136922513	-10.2952681621862	1256,56375987582	10.2952681621862	35
Q8BJ64	sp|Q8BJ64|CHDH_MOUSE Choline dehydrogenase, mitochondrial OS=Mus musculus GN=Chdh PE=1 SV=1	−1173,60	0,325364300548086	0,726927682919497	21,045356532296	21,6228591796111	21,6345534049339	21,71146161698	0	21,3878092046391	2036006997,02258	1734836,2828581	10,1967272825449	0,0157153990764057	0,866098656100634	0,000852077760732204	-10.1967272825449	1173,60180735228	10.1967272825449	36
P04186	sp|P04186|CFAB_MOUSE Complement factor B OS=Mus musculus GN=Cfb PE=1 SV=2	−1109,20	0,296157988225348	0,711969538636193	21,4618051524005	21,0597463648342	17,1235419917144	17,0367325494773	21,5741688711869	0	431034131,486423	388597,923960282	10,1153082049581	0,120567042343924	0,883783366255807	0,000901547918305681	-10.1153082049581	1109,20338197812	10.1153082049581	37
Q8BH24	sp|Q8BH24|TM9S4_MOUSE Transmembrane 9 superfamily member 4 OS=Mus musculus GN=Tm9sf4 PE=2 SV=1	−1044,11	0,355838122916397	0,732843629012038	21,9456502500876	21,616411665443	22,4365116962145	22,6290908514132	22,5167234850316	0	3583208758,75252	3431831,97073346	10,028057465109	0,0187593065756527	0,866033451364294	0,000957753846283921	-10.028057465109	1044,10961530459	10.028057465109	38
O88878	sp|O88878|ZFAN5_MOUSE AN1-type zinc finger protein 5 OS=Mus musculus GN=Zfand5 PE=1 SV=1	−877,89	0,35756431372257	0,732843629012038	22,3326153441959	21,3656840009079	20,5963942637238	20,9840740472232	0	22,9780575036453	2030533769,90241	2312972,03386171	9,77789548319485	0,0405935546887089	0,868693730275122	0,00113909557582629	-9.77789548319485	877,88946004343	9.77789548319485	39
Q9QYY0	sp|Q9QYY0|GAB1_MOUSE GRB2-associated-binding protein 1 OS=Mus musculus GN=Gab1 PE=1 SV=2	−874,06	0,375110393192269	0,746362141983349	22,502151721827	21,6488223621637	21,8769368165872	22,0488781458796	23,6595996010165	0	3618421194,47126	4139805,02368987	9,77158181483052	0,0200731359638163	0,86763703380911	0,00114409152533576	-9.77158181483052	874,055945573521	9.77158181483052	40
Q3UIK4-2	sp|Q3UIK4-2|MET14_MOUSE Isoform 2 of N6-adenosine-methyltransferase subunit METTL14 OS=Mus musculus GN=Mettl14;sp|Q3UIK4|MET14_MOUSE N6-adenosine-methyltransferase subunit METTL14 OS=Mus musculus GN=Mettl14 PE=1 SV=1	−849,33	0,381827899229565	0,749797813889279	22,9115158172942	21,7145801016844	21,8141497464129	23,355353113101	22,8515326580154	0	4151553984,23236	4888007,21766656	9,73018932752534	0,0299897914980355	0,866179830118561	0,00117739218524708	-9.73018932752534	849,334667352278	9.73018932752534	41
O54990-4	sp|O54990-4|PROM1_MOUSE Isoform 4 of Prominin-1 OS=Mus musculus GN=Prom1;sp|O54990-5|PROM1_MOUSE Isoform 5 of Prominin-1 OS=Mus musculus GN=Prom1;sp|O54990-6|PROM1_MOUSE Isoform 6 of Prominin-1 OS=Mus musculus GN=Prom1;sp|O54990-3|PROM1_MOUSE Isoform 3	−275,63	0,470108075878765	0,796715861328205	22,8927270958949	22,3703619013105	16,7517108126104	0	22,7467657754392	22,4108797662297	949646090,282619	3445392,92968546	8,10657762205193	0,164706948447064	0,866097270056886	0,00362808099242539	-8.10657762205193	275,62780491609	8.10657762205193	42
D3YZG8	sp|D3YZG8|MTD2L_MOUSE Probable bifunctional methylenetetrahydrofolate dehydrogenase/cyclohydrolase 2 OS=Mus musculus GN=Mthfd2l PE=3 SV=1	−181,45	1,16×10^−120	1,76×10^−117	22,1674770243792	22,2120966659598	21,5718826381945	16,6560990645485	16,9407271440356	16,7517108126104	3527371370,24674	19440045,0373797	7,50341810269618	0,0162594167737163	0,0086305894691809	0,00551119884947636	-7.50341810269618	181,448724190929	7.50341810269618	43
Q9D074	sp|Q9D074|MGRN1_MOUSE E3 ubiquitin-protein ligase MGRN1 OS=Mus musculus GN=Mgrn1 PE=1 SV=2;sp|Q9D074-2|MGRN1_MOUSE Isoform 2 of E3 ubiquitin-protein ligase MGRN1 OS=Mus musculus GN=Mgrn1;sp|Q9D074-4|MGRN1_MOUSE Isoform 4 of E3 ubiquitin-protein ligase M	−171,45	0,228943894872328	0,653672489647647	23,6149347963874	23,5823483342612	23,6236389235436	9,98538687940625	23,6768094780596	21,7258576307335	17880454361,1089	104289856,563358	7,42164074183379	0,000922015601825764	0,401137635848191	0,00583261781032785	-7.4216407418338	171,449601623013	7.4216407418338	44
P11589	sp|P11589|MUP2_MOUSE Major urinary protein 2 OS=Mus musculus GN=Mup2 PE=1 SV=1;sp|P04938|MUP8_MOUSE Major urinary proteins 11 and 8 (Fragment) OS=Mus musculus GN=Mup8 PE=1 SV=1;sp|P11588|MUP1_MOUSE Major urinary protein 1 OS=Mus musculus GN=Mup1 PE=1 SV	−133,23	0,10400036663211	0,465873103918229	24,2920359350875	24,2920359350875	24,6453225574227	13,5047097698342	22,451234435472	22,5971910117934	39906224154,8598	299525986,79961	7,05778879392888	0,00835607592145013	0,266830654792254	0,00750574611211704	-7.05778879392888	133,23125843354	7.05778879392888	45
Q8C5L6	sp|Q8C5L6|INP5K_MOUSE Inositol polyphosphate 5-phosphatase K OS=Mus musculus GN=Inpp5k PE=2 SV=2	−67,19	0,308846997900826	0,71853230316343	22,8515326580154	22,2332585619834	22,5936782818681	10,0906172139228	22,4365116962145	22,5286124376778	6272807345,92133	93354420,6615292	6,07024910344667	0,0137659003780216	0,389858026963278	0,0148823988229496	-6.07024910344667	67,1934687342162	6.07024910344667	46
P60989	sp|P60989|PIP_PANTR Prolactin-inducible protein homolog OS=Pan troglodytes GN=PIP PE=3 SV=1	−53,54	0,293575321628154	0,711969538636193	22,311282263451	21,2173839147839	21,0636421850033	10,0962233512701	20,3022376764705	22,252583800289	2242303347,01413	41881228,4439882	5,7425338947312	0,0315966831005747	0,37199841442433	0,018677771007102	-5.7425338947312	53,5395791938858	5.7425338947312	47

Q8N4C8-5	sp|Q8N4C8-5|MINK1_HUMAN Isoform 5 of Misshapen-like kinase 1 OS=Homo sapiens GN=MINK1;sp|Q8N4C8-2|MINK1_HUMAN Isoform 1 of Misshapen-like kinase 1 OS=Homo sapiens GN=MINK1;sp|Q9JM52-2|MINK1_MOUSE Isoform 1 of Misshapen-like kinase 1 OS=Mus musculus GN=M	−43,48	0,220538474615684	0,649593858715503	23,5572160930767	23,3393648146177	23,1333909033916	23,4971371284695	21,711461611698	13,5047097698342	13736521654,932	315952624,12841	5,44216467462816	0,00907926957708655	0,2722880618337	0,0230009191602715	-5.44216467462816	43,4765233959544	5.44216467462816	48
Q8CHY6	sp|Q8CHY6|P66A_MOUSE Transcriptional repressor p66 alpha OS=Mus musculus GN=Gatad2a PE=1 SV=2	−37,85	0,334170066660214	0,72953198881961	22,1096485360386	20,543586508048	20,7079743413186	10,0441213625493	21,6345534049339	20,7818141292922	1487560145,42889	39303716,2036574	5,24213846368762	0,0407494831755759	0,369401497249889	0,0264215980271006	-5.24213846368762	37,8478243054906	5.24213846368762	49
P28665	sp|P28665|MUG1_MOUSE Murinoglobulin-1 OS=Mus musculus GN=Mug1 PE=1 SV=3	−36,90	0,011379477679694	0,135568108105331	21,0988721291394	22,1728119761975	22,2607396283641	20,9771283438076	17,0744220795957	16,6560990645485	3067538374,7192	83127253,1927045	5,20561604102834	0,0296151528897882	0,130686530275522	0,0270990100328619	-5.20561604102835	36,9017170290479	5.20561604102835	50
Q9EPK6	sp|Q9EPK6|SIL1_MOUSE Nucleotide exchange factor SIL1 OS=Mus musculus GN=Sil1 PE=1 SV=2	−36,20	0,248020582416476	0,673304679179655	22,8225685424939	23,3482940068699	23,0200748010908	13,2874558662933	22,1461242459219	22,9902222541556	10385181627,0379	286887522,370258	5,17789741537323	0,0115141043096766	0,275991313710855	0,027624699564555	-5.17789741537323	36,1994887098462	5.17789741537323	51
Q8K479	sp|Q8K479|C1QT5_MOUSE Complement C1q tumor necrosis factor-related protein 5 OS=Mus musculus GN=C1qtnf5 PE=1 SV=1	−34,81	0,37648563326439	0,74727992066734	21,2458172816804	20,7310314969917	21,7420055571971	9,71105095397993	21,1029986109079	22,2553767996608	1675903252,25691	48148896,9912052	5,12129230750948	0,0238005945327766	0,391965160508327	0,0287301172823455	-5.12129230750948	34,8066800484137	5.12129230750948	52
Q922D8	sp|Q922D8|C1TC_MOUSE C-1-tetrahydrofolate synthase, cytoplasmic OS=Mus musculus GN=Mthfd1 PE=1 SV=4	−34,80	0,000691115911437636	0,0176234557416597	28,8979126911188	25,4204801455624	27,8216158205759	23,6328120342077	24,2198565302694	23,6385289981359	778008188203,34	22356863163,5766	5,12099565253545	0,065021216363773	0,0141538571211963	0,0287360255361907	-5.12099565253545	34,7995236411724	5.12099565253545	53
Q8BWU8	sp|Q8BWU8|AT2L1_MOUSE Ethanolamine-phosphate phospho-lyase OS=Mus musculus GN=Etnppl PE=2 SV=1	−30,75	0,190120252268925	0,616397017891892	22,4341047494528	21,6957403679723	22,1209126219968	21,0373734260528	21,4835907437214	13,4521678826468	3897440580,12063	126747906,576867	4,94249320358308	0,0167814561171541	0,241918735895467	0,0325208053776009	-4.94249320358308	30,7495459718461	4.94249320358308	54
Q9QZD4	sp|Q9QZD4|XPF_MOUSE DNA repair endonuclease XPF OS=Mus musculus GN=Ercc4 PE=2 SV=3	−25,28	0,0256357262837653	0,222062140461853	23,5108751221745	21,1739565399968	22,524642240339	20,3022376764705	16,620355218845	20,5963942637238	5364977222,80336	212190649,726673	4,66013905620192	0,0523670026472612	0,115555170995195	0,0395510811909462	-4.66013905620192	25,2837588730422	4.66013905620192	55
Q8C7X2-2	sp|Q8C7X2-2|EMC1_MOUSE Isoform 2 of ER membrane protein complex subunit 1 OS=Mus musculus GN=Emc1;sp|Q8C7X2|EMC1_MOUSE ER membrane protein complex subunit 1 OS=Mus musculus GN=Emc1 PE=1 SV=1	−21,74	0,048788143232194	0,323205843530114	23,9723375129221	22,4474378958212	23,164049627614	21,4438630713332	21,7796036535179	17,1235419917144	11838329245,8457	544658511,691014	4,44196969938859	0,0328916510422446	0,129087942592854	0,0460080557298358	-4.44196969938859	21,7353240456868	4.44196969938859	56
Q80W40	sp|Q80W40|ACSM4_MOUSE Acyl-coenzyme A synthetase ACSM4, mitochondrial OS=Mus musculus GN=Acsm4 PE=2 SV=1	−20,11	2,15×10^−14	3,90×10^−12	23,4824267429013	24,5185951445093	24,4317373548227	20,8156783455322	21,5346685350988	21,0792559200743	30599609388,5871	1521862778,37808	4,32960305017994	0,0238069653352994	0,0172034219764152	0,0497347125923018	-4.32960305017994	20,1066809855213	4.32960305017994	57
O55026	sp|O55026|ENTP2_MOUSE Ectonucleoside triphosphate diphosphohydrolase 2 OS=Mus musculus GN=Entpd2 PE=1 SV=2	−19,65	0,396748708077303	0,754338506152154	24,2371377474287	22,0698626041024	23,8100825694553	24,305821491108	23,3634269361434	13,5142251319107	14141236968,2385	719805206,172528	4,29615797451441	0,0491190846676035	0,29307361712835	0,0509011487318418	-4.29615797451441	19,645922045261	4.29615797451441	58
P63154	sp|P63154|CRNL1_MOUSE Crooked neck-like protein 1 OS=Mus musculus GN=Crnkl1 PE=1 SV=1	−16,16	0,0868195050598833	0,439814434672779	30,6668670710889	30,6114251490149	30,5898070211484	28,1173618978386	30,5053526695314	24,8971877540398	19919091250390,7	1232378848255,07	4,01463409887374	0,00129799003150614	0,101090424777788	0,0618692305167736	-4.01463409887374	16,1631232786851	4.01463409887374	59
Q8VHI5-2	sp|Q8VHI5-2|VITRN_MOUSE Isoform 2 of Vitrin OS=Mus musculus GN=Vit;sp|Q8VHI5|VITRN_MOUSE Vitrin OS=Mus musculus GN=Vit PE=1 SV=2	−15,52	0,723654168869727	0,921631098094813	20,6574696454777	13,2874558662933	16,6560990645485	0	25,3372129119629	17,0367325494773	21146991,1749863	1362250,25160948	3,9563887466792	0,218742458145884	0,9145139961033	0,0644181595545761	-3.9563887466792	15,5235729631919	3.9563887466792	60
Q99MD9-2	sp|Q99MD9-2|NASP_MOUSE Isoform 2 of Nuclear autoantigenic sperm protein OS=Mus musculus GN=Nasp;sp|Q99MD9|NASP_MOUSE Nuclear autoantigenic sperm protein OS=Mus musculus GN=Nasp PE=1 SV=2	−13,37	0,209992210836167	0,641644645910089	23,2438889303564	23,8277112897959	23,7280173231175	16,8881119694319	23,2024655836816	22,9293534018626	17753926051,7386	1327606656,40211	3,74123842020054	0,013232896139335	0,169916063055871	0,0747782013135118	-3.74123842020054	13,3728811663635	3.74123842020054	61
P25269	sp|P25269|TRBP2_ARATH Tryptophan synthase beta chain 2, chloroplastic OS=Arabidopsis thaliana GN=TSB2 PE=2 SV=2;sp|P14671|TRPB1_ARATH Tryptophan synthase beta chain 1, chloroplastic OS=Arabidopsis thaliana GN=TSB1 PE=2 SV=1	−13,25	0,236871480014704	0,660417474070481	25,3144985329333	31,6276420584925	24,9361022925742	25,1800505226162	24,6110501334689	24,3357757397456	713000264265,868	53822573538,2943	3,72761933086496	0,13772498353164	0,0174256179654508	0,0754874524397436	-3.72761933086496	13,2472347082879	3.72761933086496	62
O88705	sp|O88705|HCN3_MOUSE Potassium/sodium hyperpolarization-activated cyclic nucleotide-gated channel 3 OS=Mus musculus GN=Hcn3 PE=1 SV=1	−13,17	0,148048823981219	0,546336269959962	23,4615901922734	22,9274759558395	22,7780591977932	22,115881027351	22,376705503092	16,9407271440356	10303077239,0579	782317498,815789	3,71917724851258	0,0155863977715997	0,149720633212714	0,0759304701560524	-3.71917724851258	13,1699434751925	3.71917724851258	63
Q8BIJ6	sp|Q8BIJ6|SYIM_MOUSE Isoleucine--tRNA ligase, mitochondrial OS=Mus musculus GN=Iars2 PE=1 SV=1	−12,20	0,0102944118805874	0,125608428833296	29,8046256258292	27,0421427414941	28,8755859989111	26,2142780507656	26,9293606494445	25,0736508555744	2567921986171,58	210432306058,451	3,60917326119541	0,0491948391032957	0,0358981346619103	0,0819465338867933	-3.60917326119542	12,2030786729976	3.60917326119542	64
P54818	sp|P54818|GALC_MOUSE Galactocerebrosidase OS=Mus musculus GN=Galc PE=1 SV=2	−11,94	0,104280033835849	0,465873103918229	21,6695873010279	20,744494383141	20,543586508048	16,9407271440356	21,4780831635369	17,0984389848419	1300337128,49538	108879221,274587	3,57808514962716	0,0286173558154728	0,139163439736638	0,0837315330683291	-3.57808514962716	11,9429319320351	3.57808514962716	65
Q3UZZ6	sp|Q3UZZ6|ST1D1_MOUSE Sulfotransferase 1 family member D1 OS=Mus musculus GN=Sult1d1 PE=1 SV=1	−11,33	1,33×10^−20	4,03×10^−18	24,275977743973	24,3929941603799	24,2796892913244	21,5941058210057	21,6695873010279	22,4030699429728	36341290043,6443	3208024043,14587	3,50185277379249	0,00273537254415067	0,0204150134441947	0,0882749082185353	-3.50185277379249	11,3282474055298	3.50185277379249	66

Q61316	sp|Q61316|HSP74_MOUSE Heat shock 70 kDa protein 4 OS=Mus musculus GN=Hspa4 PE=1 SV=1	−11,17	0,00686356129634913	0,0927193592980021	30,6166732848265	27,5424060245662	29,2013315647317	26,7858549456868	26,8006206385251	26,5351193087617	4433174159810,61	396998483472,516	3,48113463930473	0,0528411359308811	0,0055867912145443	0,0895517453547271	-3.48113463930474	11,1667281976343	3.48113463930474	67
P63084	sp|P63084|S10A5_MOUSE Protein S100-A5 OS=Mus musculus GN=S100a5 PE=2 SV=1	−10,30	3,15×10^−21	1,02×10^−18	30,6563201848299	30,5147582056784	30,3160330662053	27,7120449619398	28,399855058049	28,3794989601019	17543485825533,7	1703659431181,89	3,36422656583627	0,00560544201737713	0,0138959735682988	0,097110656805861	-3.36422656583627	10,2975310114435	3.36422656583627	68
Q571E4	sp|Q571E4|GALNS_MOUSE N-acetylgalactosamine-6-sulfatase OS=Mus musculus GN=Galns PE=2 SV=2	−9,52	0,300849159511533	0,714202058066343	23,43383578992	23,5647953275257	22,5318902919992	16,620355218845	22,9406662221807	23,2107589666823	11629844930,5446	1222210330,25435	3,25026737528624	0,024264236534275	0,178238445660049	0,105092573250426	-3.25026737528625	9,51542025350445	3.25026737528625	69
Q64704-2	sp|Q64704-2|STX3_MOUSE Isoform 3B of Syntaxin-3 OS=Mus musculus GN=Stx3;sp|Q64704-3|STX3_MOUSE Isoform 3C of Syntaxin-3 OS=Mus musculus GN=Stx3;sp|Q64704-6|STX3_MOUSE Isoform 3D-2 of Syntaxin-3 OS=Mus musculus GN=Stx3;sp|Q64704-5|STX3_MOUSE Isoform 3D-	−8,39	0,191893339946814	0,618802084535046	21,6895799900045	22,8687955606518	22,3703619013105	21,4368240413319	22,0932341720107	17,0191442503345	4885703628,4975	582632676,271969	3,06790783026106	0,0265335758219217	0,136729676874089	0,119252562286744	-3.06790783026106	8,3855640568585	3.06790783026106	70
O88704	sp|O88704|HCN1_MOUSE Potassium/sodium hyperpolarization-activated cyclic nucleotide-gated channel 1 OS=Mus musculus GN=Hcn1 PE=1 SV=1	−7,44	0,344320094074138	0,730213633407042	23,2345232311874	22,8773938140701	22,8773938140701	23,2058538104204	16,7517108126104	23,0095077948973	9710144037,84425	1304419745,41305	2,89608451148593	0,00896611790464026	0,174897790952721	0,134335777134635	-2.89608451148592	7,44403331212185	2.89608451148592	71
P60041	sp|P60041|SMS_MOUSE Somatostatin OS=Mus musculus GN=Sst PE=2 SV=1	−7,07	0,232812191841407	0,657279264635888	22,8906559375229	22,0134108257316	21,8565573461608	21,9088833413978	21,9012572974974	17,0835792308148	4619449457,07979	653534984,234373	2,82138455066443	0,0250433539478789	0,13714181669437	0,141474647640697	-2.82138455066443	7,06840424540019	2.82138455066443	72
Q64288	sp|Q64288|OMP_MOUSE Olfactory marker protein OS=Mus musculus GN=Omp PE=1 SV=3	−6,53	1,71×10^−34	8,60×10^−32	32,4715364843887	32,4800692515926	32,3120570949621	30,2598956411556	30,6639825913764	30,7106727634419	120325410613382	18427225468808,4	2,70703057630659	0,00291891798212897	0,0081152716837535	0,153144920718509	-2.70703057630659	6,52976275875366	2.70703057630659	73
Q6ZPS2	sp|Q6ZPS2|CRNS1_MOUSE Carnosine synthase 1 OS=Mus musculus GN=Carns1 PE=1 SV=2;sp|Q6ZPS2-2|CRNS1_MOUSE Isoform 2 of Carnosine synthase 1 OS=Mus musculus GN=Carns1	−6,36	4,50×10^−13	7,29×10^−11	26,3678330475435	25,7861812046239	26,1149474128652	24,620547219318	24,0965663332202	24,0031450361206	214088193952,764	33677176720,54	2,66836213721662	0,0111787486946211	0,0137286415284622	0,157305155874081	-2.66836213721662	6,35707071674419	2.66836213721662	74
Q80VP5	sp|Q80VP5|CL065_MOUSE Probable peptide chain release factor C12orf65 homolog, mitochondrial OS=Mus musculus PE=1 SV=1	−6,16	0,226465846167286	0,652238880554131	22,2553767996608	21,8690502708892	21,1647787321785	17,0191442503345	21,616411665443	21,2017394076376	2828659045,82765	459553124,967462	2,62181473729201	0,0254086120927527	0,127495454959916	0,162463244075073	-2.62181473729201	6,15523840911305	2.62181473729201	75
Q8R092-2	sp|Q8R092-2|CA043_MOUSE Isoform 3 of Uncharacterized protein C1orf43 homolog OS=Mus musculus;sp|Q8R092-1|CA043_MOUSE Isoform 2 of Uncharacterized protein C1orf43 homolog OS=Mus musculus;sp|Q8R092|CA043_MOUSE Uncharacterized protein C1orf43 homolog OS=Mu	−5,97	0,324660270456749	0,726844368394785	22,1923188032946	21,8106908372461	22,0882083383777	22,0655043166933	22,0428427182337	16,620355218845	3695589775,0722	618557524,078207	2,57882494778592	0,00895448335441115	0,154979612746654	0,167377214930768	-2.57882494778592	5,97452885336651	2.57882494778592	76
Q61699	sp|Q61699|HS105_MOUSE Heat shock protein 105 kDa OS=Mus musculus GN=Hsph1 PE=1 SV=2;sp|Q61699-2|HS105_MOUSE Isoform HSP105-beta of Heat shock protein 105 kDa OS=Mus musculus GN=Hsph1	−5,61	0,0250610406313801	0,219211300821997	32,0900263529684	29,7730411693604	31,3230324235924	29,4911659736167	29,8660763732758	28,6537286050758	30907911890913	5506720202572,08	2,48871097851228	0,0379993482814239	0,0211577512180181	0,178165390855507	-2.48871097851228	5,61276236197302	2.48871097851228	77
Q3TXS7	sp|Q3TXS7|PSMD1_MOUSE 26S proteasome non-ATPase regulatory subunit 1 OS=Mus musculus GN=Psmd1 PE=1 SV=1	−5,35	0,0414887386494872	0,299624741892926	25,9760107033085	23,1361785433251	24,3979637610652	22,8906559375229	22,8505765759992	22,7369952893588	43821234198,2968	8189149917,87995	2,41984451303829	0,0580674126161806	0,0034919009994717	0,18687629565208	-2.41984451303829	5,35113346778752	2.41984451303829	78
Q9ER97	sp|Q9ER97|NGB_MOUSE Neuroglobin OS=Mus musculus GN=Ngb PE=1 SV=1	−5,27	0,0010379583100558	0,0242850142749654	23,9703394650702	24,5268886798457	24,0268686400196	21,5741688711869	22,8462665924133	23,114783803093	31545567611,411	5980319045,43012	2,39914295172361	0,0126707720783823	0,0365580480853298	0,189577157688133	-2.39914295172361	5,27489710361133	2.39914295172361	79
P56183-2	sp|P56183-2|RRP1_MOUSE Isoform 2 of Ribosomal RNA processing protein 1 homolog A OS=Mus musculus GN=Rrp1;sp|P56183-3|RRP1_MOUSE Isoform 3 of Ribosomal RNA processing protein 1 homolog A OS=Mus musculus GN=Rrp1;sp|P56183|RRP1_MOUSE Ribosomal RNA processi	−5,27	4,58×10^−11	5,94×10^−09	22,4633145269286	22,1461242459219	22,9695985789723	20,7818141292922	20,7897257103873	21,0220202458726	6068308781,12034	1151717367,6267	2,39750777617119	0,0184379598625718	0,0065401235760523	0,189792149537596	-2.39750777617119	5,26892183073098	2.39750777617119	80
Q9CZU3	sp|Q9CZU3|SK2L2_MOUSE Superkiller viralicidic activity 2-like 2 OS=Mus musculus GN=Skiv2l2 PE=1 SV=1	−5,06	0,39051769684848	0,753319093070654	21,8106908372461	27,6606396741905	22,3038750494383	22,5136465441664	21,8445984352844	22,5552010672857	24576794465,9547	4860863896,6718	2,33801211368073	0,135610228931986	0,0178803729157981	0,197782664594659	-2.33801211368073	5,05605484711931	2.33801211368073	81
Q99JX7	sp|Q99JX7|NXF1_MOUSE Nuclear RNA export factor 1 OS=Mus musculus GN=Nxf1 PE=1 SV=3	−4,91	0,00553209119118629	0,0817809931121212	22,9793957310975	23,835941088441	22,6338453533699	20,744494383141	22,2942056201138	21,6345534049339	11318760315,6133	2303628063,69991	2,29673624816668	0,0267339924696059	0,0360754265071639	0,203523000705496	-2.29673624816668	4,91344956851844	2.29673624816668	82
P46935	sp|P46935|NEDD4_MOUSE E3 ubiquitin-protein ligase NEDD4 OS=Mus musculus GN=Nedd4 PE=1 SV=3	−4,73	0,0130340765535323	0,149398165344654	26,3000797338373	24,4060266307704	25,8693686586924	24,4665538370577	23,800646758244	23,6434784339963	121740594225,086	25712069349,4144	2,24329268243517	0,0388963012852334	0,0182291460077727	0,211203744429532	-2.24329268243517	4,73476454075676	2.24329268243517	83
Q8BFR2	sp|Q8BFR2|FSTL5_MOUSE Follistatin-related protein 5 OS=Mus musculus GN=Fstl5 PE=2 SV=1	−4,72	2,38×10^−16	4,91×10^−14	24,7608480287448	24,6534719708188	24,3163006006741	23,2024655836816	22,7620598330115	23,1077379050397	47162906126,3245	9982383974,1694	2,24019631479604	0,00943819834703515	0,0100682860961078	0,211657524823256	-2.24019631479604	4,72461350398503	2.24019631479604	84

Q80ZI2	sp|Q80ZI2|RTP2_MOUSE Receptor-transporting protein 2 OS=Mus musculus GN=Rtp2 PE=1 SV=1	−4,58	7,01×10^−43	5,31×10^−40	24,3086666205643	24,5047987626687	24,4443019138267	22,9990398942442	22,7080917274642	22,9837162468125	40285432562,952	8790604252,95794	2,19622400389746	0,00411299311076469	0,0071509442889208	0,218208014503042		-2.19622400389746	4,58278309473395	2.19622400389746	85
Q03734	sp|Q03734|SPA3M_MOUSE Serine protease inhibitor A3M OS=Mus musculus GN=Serpina3m PE=1 SV=2	−4,55	6,38×10^−05	0,00258360226876699	27,7640796168976	28,8140965858009	28,0003802943019	26,9415112903228	26,8202978882426	26,2722099307776	1753881005289,22	385576853164,624	2,18546051743595	0,0195380689778034	0,0133674820421522	0,219842082787732		-2.18546051743595	4,54871964147804	2.18546051743595	86
Q9Z0G9-2	sp|Q9Z0G9-2|CLD3_MOUSE Isoform 2 of Claudin-3 OS=Mus musculus GN=Cldn3;sp|Q9Z0G9|CLD3_MOUSE Claudin-3 OS=Mus musculus GN=Cldn3 PE=1 SV=1	−4,45	2,37×10^−12	3,71×10^−10	23,4318412203999	23,0135041204645	22,9780575036453	21,6488223621637	21,9172465363468	21,3795262247439	11221913672,0452	2522538840,64275	2,15337033116974	0,0109062356479107	0,0124193306623151	0,224786869188508		-2.15337033116975	4,44865842746977	2.15337033116975	87
Q9R1E6	sp|Q9R1E6|ENPP2_MOUSE Ectonucleotide pyrophosphatase/phosphodiesterase family member 2 OS=Mus musculus GN=Enpp2 PE=1 SV=3;sp|Q9R1E6-3|ENPP2_MOUSE Isoform 3 of Ectonucleotide pyrophosphatase/phosphodiesterase family member 2 OS=Mus musculus GN=Enpp2;sp|Q	−4,45	0,0386172528096035	0,288186689063277	24,024166766318	21,8534467936578	23,1798328591074	20,8466617883996	21,9944238728401	21,740785022867	9933202855,59933	2234515421,89698	2,15229697265778	0,0475362671656616	0,0280077008908765	0,224954171819554		-2.15229697265779	4,44534987687245	2.15229697265779	88
Q5SS80	sp|Q5SS80|DHR13_MOUSE Dehydrogenase/reductase SDR family member 13 OS=Mus musculus GN=Dhrs13 PE=2 SV=1;sp|Q5SS80-2|DHR13_MOUSE Isoform 2 of Dehydrogenase/reductase SDR family member 13 OS=Mus musculus GN=Dhrs13	−4,37	0,118953806674403	0,493003989498548	24,5047987626687	23,5244400838048	23,7625704964307	23,3041913196319	23,4032259601932	20,6574696454777	24713194155,6583	5650231238,49352	2,1288996727579	0,0213669406880811	0,0693593537995081	0,228632171256578		-2.1288996727579	4,37383765593413	2.1288996727579	89
Q6P5E8	sp|Q6P5E8|DGKQ_MOUSE Diacylglycerol kinase theta OS=Mus musculus GN=Dgkq PE=2 SV=1	−4,34	0,0790799147501893	0,418825847815587	26,5575313626245	24,0558013103755	25,7421229489083	24,1341293512829	24,5806585090775	23,2373012347919	113131708700,29	26069442981,0941	2,11757168378913	0,0501292432266601	0,0285248780017775	0,230434449197241		-2.11757168378914	4,33962892043127	2.11757168378914	90
Q60673	sp|Q60673|PTPRN_MOUSE Receptor-type tyrosine-protein phosphatase-like N OS=Mus musculus GN=Ptprn PE=1 SV=2	−4,33	6,98×10^−12	9,90×10^−10	25,266100588418	25,7538649352021	25,6863781120229	24,1446543239673	24,3404652835671	23,8277112897959	127168804722,313	29400483765,8602	2,11283301321538	0,010336513414741	0,0107346375632419	0,231192577692771		-2.11283301321537	4,32539837558664	2.11283301321537	91
Q923U0	sp|Q923U0|TM1L1_MOUSE TOM1-like protein 1 OS=Mus musculus GN=Tom1l1 PE=1 SV=1;sp|Q923U0-3|TM1L1_MOUSE Isoform 3 of TOM1-like protein 1 OS=Mus musculus GN=Tom1l1	−4,29	0,000638481770345108	0,0166555675608991	24,7090851139904	25,1789507460175	24,8877355888685	23,7058662910714	22,6809000175204	24,0187780694206	66819256681,7808	15568489314,3005	2,1016349742314	0,0095152241688252	0,0298190147392517	0,232994051227533		-2.1016349742314	4,29195507237839	2.1016349742314	92
Q99KG3-2	sp|Q99KG3-2|RBM10_MOUSE Isoform 2 of RNA-binding protein 10 OS=Mus musculus GN=Rbm10;sp|Q99KG3-3|RBM10_MOUSE Isoform 3 of RNA-binding protein 10 OS=Mus musculus GN=Rbm10;sp|Q99KG3|RBM10_MOUSE RNA-binding protein 10 OS=Mus musculus GN=Rbm10 PE=1 SV=1	−4,28	0,0644421842428237	0,38006509013234	23,4079437247829	20,9771283438076	23,1374797714001	21,3795262247439	20,9183799039599	20,8620051295316	5955120531,86786	1391020869,13123	2,09798664415967	0,059190757378159	0,01348575171684	0,233583999129389		-2.09798664415967	4,2811516082944	2.09798664415967	93
Q8BIF0-2	sp|Q8BIF0-2|C99L2_MOUSE Isoform 2 of CD99 antigen-like protein 2 OS=Mus musculus GN=Cd99l2;sp|Q8BIF0|C99L2_MOUSE CD99 antigen-like protein 2 OS=Mus musculus GN=Cd99l2 PE=1 SV=1	−4,20	0,243708911041902	0,667588863741216	23,6185544152518	26,9731145224765	23,0613359467876	23,4291787490228	22,9752239618567	22,9406662221807	45958361014,965	10932687345,6851	2,07167927787746	0,086189279545587	0,0117938456834334	0,237882446289266		-2.07167927787747	4,20375700518901	2.07167927787747	94
Q9D258	sp|Q9D258|ACBD7_MOUSE Acyl-CoA-binding domain-containing protein 7 OS=Mus musculus GN=Acbd7 PE=2 SV=2	−4,13	3,03×10^−17	6,55×10^−15	25,2432823524486	25,4105254331557	25,1876434359737	23,5758934844069	24,1023003631839	23,9115536096075	95317926941,1366	23103481384,3856	2,04463731206656	0,00458869943236648	0,0111680983034321	0,242383380816214		-2.04463731206657	4,12569540301216	2.04463731206657	95
P30681	sp|P30681|HMGB2_MOUSE High mobility group protein B2 OS=Mus musculus GN=Hmgb2 PE=1 SV=3	−3,93	1,22×10^−05	0,00061056430845434	24,0493524445907	24,6380315993696	24,9998552831891	23,4032259601932	22,9115158172942	23,2631031706819	46485817780,4047	11814754665,7002	1,97620096387057	0,0195315560368016	0,010922453599985	0,254158262236284		-1.97620096387057	3,93455633195323	1.97620096387057	96
Q9WTZ0	sp|Q9WTZ0|UXT_MOUSE Protein UXT OS=Mus musculus GN=Uxt PE=2 SV=1	−3,92	0,398033511389294	0,754338506152154	21,045356532296	21,2812138014139	21,5496008959617	16,7517108126104	21,968529841547	21,0597463648342	1766130931,55309	450857705,882996	1,96984821577194	0,0118493483615933	0,139856925252846	0,255279887707149		-1.96984821577194	3,91726903745419	1.96984821577194	97
O35218	sp|O35218|CPSF2_MOUSE Cleavage and polyadenylation specificity factor subunit 2 OS=Mus musculus GN=Cpsf2 PE=1 SV=1	−3,87	0,133246989172167	0,518639007654437	27,3835409950841	27,1190534987677	24,6808051491555	25,1045144102 8	25,4719455493022	24,547663319805	290382991123,136	75046550138,6047	1,95209929313084	0,0564495091421368	0,0185838364361669	0,258439896387669		-1.95209929313084	3,86937161784017	1.95209929313084	98
P70408	sp|P70408|CAD10_MOUSE Cadherin-10 OS=Mus musculus GN=Cdh10 PE=1 SV=3	−3,74	0,0055325499730331 8	0,0817809931121212	23,9578712584091	22,6095143587135	23,3326444849489	22,3631795486139	21,4552101793533	22,1271865824891	13154240749,3188	3520464472,09819	1,90169029152924	0,0289600983263465	0,0214316472862201	0,267629621442081		-1.90169029152924	3,736507172157	1.90169029152924	99

Q8BP00-2	sp|Q8BP00-2|IQCB1_MOUSE Isoform 2 of IQ calmodulin-binding motif-containing protein 1 OS=Mus musculus GN=Iqcb1;sp|Q8BP00|IQCB1_MOUSE IQ calmodulin-binding motif-containing protein 1 OS=Mus musculus GN=Iqcb1 PE=1 SV=2	−3,71	0,366656274887335	0,739315231400715	24,9067459177116	24,6502909577635	24,8919913695741	24,5984115810658	25,2846128887524	20,6348824710516	59923989138,6679	16162651916,0201	1,89046973689087	0,00580239300744297	0,10678114559655	0,269719225110643	-1.89046973689087	3,70755922048117	1.89046973689087	100
O88809	sp|O88809|DCX_MOUSE Neuronal migration protein doublecortin OS=Mus musculus GN=Dcx PE=1 SV=1;sp|O43602-2|DCX_HUMAN Isoform 2 of Neuronal migration protein doublecortin OS=Homo sapiens GN=DCX;sp|O43602-4|DCX_HUMAN Isoform 4 of Neuronal migration protein	−3,69	4,74×10^−18	1,08×10^−15	27,5424060245662	27,2551673122194	27,6623996166401	26,0031584198129	26,3021104634812	26,2339091105729	865573085541,405	234266688538,843	1,88550381483253	0,00761320917485056	0,0059843347114801	0,270649229339556	-1.88550381483253	3,69481931443227	1.88550381483253	101
P62846	sp|P62846|RS15_CHICK 40S ribosomal protein S15 OS=Gallus gallus GN=RPS15 PE=2 SV=2;sp|P62843|RS15_MOUSE 40S ribosomal protein S15 OS=Mus musculus GN=Rps15 PE=2 SV=2	−3,65	0,00841720587436171	0,107981753720955	26,2569962681467	25,8577700523384	25,9437232703096	23,779469321038	25,0924879023731	25,30131699854	199583100471,913	54661460709,8506	1,86839364847212	0,00807558980340464	0,0333673959771217	0,27387820201512	-1.86839364847212	3,6512580871434	1.86839364847212	102
P62965	sp|P62965|RABP1_MOUSE Cellular retinoic acid-binding protein 1 OS=Mus musculus GN=Crabp1 PE=1 SV=2	−3,63	0,00436220466733717	0,0699623693401338	24,5185951445093	24,2429818835158	24,0459906620689	22,1209126219968	23,3276794305009	23,4877467946751	34671686160,5631	9540193600,21494	1,86166755127623	0,00978152562763496	0,0325183955855831	0,275158051328531	-1.86166755127623	3,63427490190366	1.86166755127623	103
Q9EPW0	sp|Q9EPW0|INP4A_MOUSE Type I inositol 3,4-bisphosphate 4-phosphatase OS=Mus musculus GN=Inpp4a PE=2 SV=1	−3,57	0,149448224771545	0,547936585006497	26,4674873848154	23,591698233046	25,2470629747378	23,9284441294148	24,2796892913244	23,277183681821	79743643029,046	22313083205,0631	1,83747963741859	0,0574998476199406	0,0213475922163138	0,279810181194452	-1.83747963741859	3,57385137213808	1.83747963741859	104
Q9QUG9	sp|Q9QUG9|GRP2_MOUSE RAS guanyl-releasing protein 2 OS=Mus musculus GN=Rasgrp2 PE=1 SV=2;sp|Q9QUG9-2|GRP2_MOUSE Isoform 2 of RAS guanyl-releasing protein 2 OS=Mus musculus GN=Rasgrp2	−3,54	0,00020963737792878	0,0066079448501301	23,2219874868566	23,7251359806086	23,8751848769123	22,4148399246451	22,78470180127	21,829564652429	17888718769,9034	5051926332,34085	1,82414455516215	0,0144926974191877	0,0215548195617125	0,282408505456544	-1.82414455516215	3,54096983865054	1.82414455516215	105
Q9Z277	sp|Q9Z277|BAZ1B_MOUSE Tyrosine-protein kinase BAZ1B OS=Mus musculus GN=Baz1b PE=1 SV=2;sp|Q9Z277-2|BAZ1B_MOUSE Isoform 2 of Tyrosine-protein kinase BAZ1B OS=Mus musculus GN=Baz1b	−3,54	0,0665477689263774	0,383812354710072	25,7816672426511	28,0716553884336	26,5688885730431	25,8313075112595	25,4860362621515	25,3139278902866	438841323461,614	124026020246,126	1,82305655842949	0,0434013278388378	0,0103146625428834	0,282621561861584	-1.82305655842949	3,53830045171768	1.82305655842949	106
Q61458	sp|Q61458|CCNH_MOUSE Cyclin-H OS=Mus musculus GN=Ccnh PE=2 SV=2	−3,54	0,376970257001627	0,74727992066734	20,744494383141	20,3022376764705	21,9306752260833	21,5219605614353	17,0984389848419	20,5686348760599	1308921163,08103	370245975,281029	1,82182224766816	0,0401126431651667	0,117996876574301	0,282863464755599	-1.82182224766817	3,53527452145163	1.82182224766817	107
Q80XE1	sp|Q80XE1|RIC8B_MOUSE Synembryn-B OS=Mus musculus GN=Ric8b PE=1 SV=2;sp|Q80XE1-1|RIC8B_MOUSE Isoform 1 of Synembryn-B OS=Mus musculus GN=Ric8b	−3,48	0,000749894786567238	0,0188053725758491	24,3731216083975	25,5517501914311	25,3969460609751	23,8670266020545	23,8733808899669	23,8407893507363	80158568834,9289	23037725483,9427	1,79885846419328	0,0255100426827641	0,0007240889030539	0,28740190623144	-1.79885846419328	3,47944804233384	1.79885846419328	108
Q8CGK7	sp|Q8CGK7|GNAL_MOUSE Guanine nucleotide-binding protein G(olf) subunit alpha OS=Mus musculus GN=Gnal PE=1 SV=1	−3,44	5,65×10^−05	0,00236164967161283	27,4208032405863	26,584740539218	27,2983095342163	25,9864544591337	26,0654090296045	25,5454020810795	588759982770,561	171141269521,725	1,78249191906044	0,0166602225079569	0,0108339598346941	0,290680879356603	-1.78249191906045	3,44019875752892	1.78249191906045	109
O35551-2	sp|O35551-2|RABE1_MOUSE Isoform 2 of Rab GTPase-binding effector protein 1 OS=Mus musculus GN=Rabep1;sp|O35551-3|RABE1_MOUSE Isoform 3 of Rab GTPase-binding effector protein 1 OS=Mus musculus GN=Rabep1;sp|O35551|RABE1_MOUSE Rab GTPase-binding effector p	−3,39	0,907006521509613	0,980398950801751	23,3241799096996	0	22,8515326580154	20,9058908208185	21,602992944783	0	4837478,58403902	1424922,41112215	1,76337190953263	0,866161496484333	0,866374678970083	0,294558908399843	-1.76337190953263	3,39490666037699	1.76337190953263	110
Q8BKY8	sp|Q8BKY8|MTEF2_MOUSE Transcription termination factor 2, mitochondrial OS=Mus musculus GN=Mterf2 PE=1 SV=1	−3,36	0,000187562325129649	0,00603791059406721	25,5223313428076	24,9114903418552	25,2054719742707	23,835941088441	23,6309486811925	24,5323306407483	89106479132,0795	26482239475,9529	1,75050520805272	0,012116396658635	0,0196872151629233	0,297197686788849	-1.75050520805272	3,3647637395996	1.75050520805272	111
P24529	sp|P24529|TY3H_MOUSE Tyrosine 3-monooxygenase OS=Mus musculus GN=Th PE=1 SV=3	−3,35	3,80×10^−13	6,39×10^−11	27,7796180261822	28,2936711973808	28,1757537010239	26,9273907888982	26,9378482697719	26,7527581374521	1571441250588,91	468436191560,992	1,74616388856568	0,00958880640677404	0,0038691576196259	0,298093353083001	-1.74616388856568	3,3546538010915	1.74616388856568	112
Q9R0N8-2	sp|Q9R0N8-2|SYT6_MOUSE Isoform 2 of Synaptotagmin-6 OS=Mus musculus GN=Syt6;sp|Q9R0N8|SYT6_MOUSE Synaptotagmin-6 OS=Mus musculus GN=Syt6 PE=1 SV=2	−3,35	1,13×10^−10	1,39×10^−08	26,8505074822295	26,4619673766869	26,4927145297487	25,6721471841847	25,2629129193492	25,2415366291153	357260054037,205	106583964537,243	1,74498421008009	0,00811960735802747	0,0095571792747076	0,298337200962701	-1.74498421008009	3,35191185267245	1.74498421008009	113
P26339	sp|P26339|CMGA_MOUSE Chromogranin-A OS=Mus musculus GN=Chga PE=1 SV=1	−3,35	0,000102369995741401	0,00384014388983652	26,5000720757695	25,9481071296484	25,8555074920393	24,6164760537761	25,3648968031627	24,6958846864592	216580664469,711	64660683581,9545	1,74394378556548	0,0133517701207022	0,0165150462062532	0,298552429600646	-1.74394378556548	3,34949543481403	1.74394378556548	114
Q69ZX8	sp|Q69ZX8|ABLM3_MOUSE Actin-binding LIM protein 3 OS=Mus musculus GN=Ablim3 PE=1 SV=2	−3,34	0,00892187139614528	0,112489928519732	22,8020772128933	22,1923188032946	21,2911396120514	20,966955718834	20,6190916890494	21,0856256932374	3942885066,79182	1182099226,49669	1,73790051544279	0,0344029050841235	0,0116066086752016	0,29980565156532	-1.73790051544279	3,33549416022976	1.73790051544279	115
O35643	sp|O35643|AP1B1_MOUSE AP-1 complex subunit beta-1 OS=Mus musculus GN=Ap1b1 PE=2 SV=2	−3,04	0,12658129037644	0,509135279119877	29,6015013481049	27,4766700566273	28,9044917431211	27,5612721540117	28,187447373342	26,8991884585141	2800692197502,06	921531375673,678	1,60367824492509	0,037792247372421	0,0233839987992726	0,329037006099989	-1.60367824492509	3,03917183010143	1.60367824492509	116

Q9R0X5	sp|Q9R0X5|RPGR_MOUSE X-linked retinitis pigmentosa GTPase regulator OS=Mus musculus GN=Rpgr PE=1 SV=2;sp|Q9R0X5-4|RPGR_MOUSE Isoform 4 of X-linked retinitis pigmentosa GTPase regulator OS=Mus musculus GN=Rpgr;sp|Q9R0X5-3|RPGR_MOUSE Isoform 3 of X-linked	−3,04	0,194869892163132	0,622304200947129	24,2429818835158	26,5828657246869	23,8445866552698	23,6385289981359	23,8343072841257	23,8645191979705	64513784719,2058	21239287624,2699	1,60287207715772	0,059437634886938	0,00515946751113603	0,329220921028168	-1.60287207715772	3,03747403681688	1.60287207715772	117
Q91WD9	sp|Q91WD9|SEGN_MOUSE Secretagogin OS=Mus musculus GN=Scgn PE=2 SV=1	−3,03	7,10×10^−06	0,0003928141609505	27,5687786150749	27,8216158205759	28,2874146092639	26,9938047890829	26,5491925988097	26,8093381012677	1298983322706,64	428737992721,495	1,59921473583143	0,0130693852337479	0,00833990604828144	0,330056579809008	-1.59921473583143	3,02978356189313	1.59921473583143	118
Q8RWG1	sp|Q8RWG1|Y4139_ARATH Uncharacterized aarF domain-containing protein kinase At4g31390, chloroplastic OS=Arabidopsis thaliana GN=At4g31390 PE=2 SV=1;sp|Q8RWG1-2|Y4139_ARATH Isoform 2 of Uncharacterized aarF domain-containing protein kinase At4g31390, chlo	−3,03	0,397555305486582	0,754338506152154	21,430109728472	22,4030699429728	25,7036960429525	21,8938035010294	22,47968402626	21,8402680937608	11654504230,5235	3849664635,09079	1,59808295965018	0,0966373243418061	0,01607176692347	0,330315606648321	-1.59808295965017	3,0274076667067	1.59808295965017	119
P40240	sp|P40240|CD9_MOUSE CD9 antigen OS=Mus musculus GN=Cd9 PE=1 SV=2	−3,02	0,307670481031933	0,71837258919956	26,7440322826555	26,238618367179	26,1944717091464	23,1523256748072	26,513257270011	26,190885557456	289776020861,479	95796261845,3112	1,59689695052658	0,0115693945431566	0,0733382113113329	0,330587263778822	-1.59689695052658	3,02491992150383	1.59689695052658	120
Q9D7A6	sp|Q9D7A6|SRP19_MOUSE Signal recognition particle 19 kDa protein OS=Mus musculus GN=Srp19 PE=2 SV=1	−2,97	0,0192715060115632	0,19182755654931	24,0352949440679	23,7444339745312	23,6996447819213	21,9143063520067	23,4741435389005	22,8291248292886	22268921756,3035	7507674292,96628	1,56859373776368	0,00764861511610764	0,0344690270278292	0,337136857146716	-1.56859373776368	2,96615448237633	1.56859373776368	121
Q69ZS6	sp|Q69ZS6|SV2C_MOUSE Synaptic vesicle glycoprotein 2C OS=Mus musculus GN=Sv2c PE=1 SV=2	−2,96	0,0129163174521941	0,148423202317744	25,0087957507153	24,5643854343456	23,8772170718654	23,6863570906192	23,6838064405286	22,8254908632616	42957027206,2116	14516495563,0106	1,56520094327232	0,0232856220003743	0,0212101453466321	0,33793063689732	-1.56520094327232	2,95918715503693	1.56520094327232	122
Q9EQK5	sp|Q9EQK5|MVP_MOUSE Major vault protein OS=Mus musculus GN=Mvp PE=1 SV=4	−2,96	0,000839112161710635	0,0206996201195901	24,8340128117331	24,0597088731534	23,7779715892546	23,2912926988169	23,075311364659	23,0507037791687	33136384924,8373	11199137621,6372	1,56502857438715	0,0225740345885402	0,00572077170118831	0,337971014250348	-1.56502857438715	2,95883362133317	1.56502857438715	123
P52194	sp|P52194|CLGN_MOUSE Calmegin OS=Mus musculus GN=Clgn PE=1 SV=2	−2,94	1,11×10^−09	1,23×10^−07	27,1079700259264	26,8042111906981	26,5858634061522	25,7508401370558	25,914114289794	25,5956685115229	450075660906,587	152975096858,072	1,55687073645204	0,00977222814744645	0,00618323004122334	0,339887512579406	-1.55687073645204	2,9421498672046	1.55687073645204	124
P56546	sp|P56546|CTBP2_MOUSE C-terminal-binding protein 2 OS=Mus musculus GN=Ctbp2 PE=1 SV=2;sp|P56546-2|CTBP2_MOUSE Isoform 2 of C-terminal-binding protein 2 OS=Mus musculus GN=Ctbp2	−2,94	0,131097422497065	0,515195844774179	24,9148170158252	24,3517999664098	24,6522337538154	24,0132687336335	24,4753092267965	22,1997126389534	50216872463,1335	17107170381,6285	1,55357102948771	0,0114336394617352	0,0510479374187694	0,340665787065645	-1.55357102948771	2,93542832291316	1.55357102948771	125
Q9CYA6	sp|Q9CYA6|ZCHC8_MOUSE Zinc finger CCHC domain-containing protein 8 OS=Mus musculus GN=Zcchc8 PE=2 SV=3	−2,93	0,0523080750097975	0,335822280720609	23,4522005639565	23,2219874868566	23,3655480943154	22,2942056201138	21,3089099562841	23,2126920867356	13781303822,0784	4705208331,31974	1,55038187771827	0,00498029827098745	0,042748256246746	0,341419679303619	-1.55038187771828	2,92894657402193	1.55038187771828	126
A2A8L5	sp|A2A8L5|PTPRF_MOUSE Receptor-type tyrosine-protein phosphatase F OS=Mus musculus GN=Ptprf PE=1 SV=1	−2,91	0,143161173599502	0,536588412029841	23,3576826764757	22,6869167687749	23,0974215930066	23,0480487104139	20,6574696454777	22,2366110073191	10217219765,2318	3516421843,22592	1,53882261699047	0,0146730913280493	0,055305802035827	0,344166213904095	-1.53882261699047	2,90557282964056	1.53882261699047	127
O35668-2	sp|O35668-2|HAP1_MOUSE Isoform A of Huntingtin-associated protein 1 OS=Mus musculus GN=Hap1;sp|O35668|HAP1_MOUSE Huntingtin-associated protein 1 OS=Mus musculus GN=Hap1 PE=1 SV=1	−2,90	2,57×10^−08	2,48×10^−06	26,308372107962	26,0039735970023	25,9086542006991	25,2712628519499	24,7568685258369	24,9961132911365	210692726227,34	72589168708,7696	1,53731430899358	0,00800706945926818	0,0102929096898687	0,344526220760203	-1.53731430899358	2,90253670038084	1.53731430899358	128
Q8BFW7-4	sp|Q8BFW7-4|LPP_MOUSE Isoform 4 of Lipoma-preferred partner homolog OS=Mus musculus GN=Lpp;sp|Q8BFW7|LPP_MOUSE Lipoma-preferred partner homolog OS=Mus musculus GN=Lpp PE=1 SV=1;sp|Q8BFW7-5|LPP_MOUSE Isoform 5 of Lipoma-preferred partner homolog OS=Mus m	−2,90	0,186499242436945	0,610324485523645	23,5244400838048	23,5108751221745	21,8445984352844	22,4317248569495	22,5214498237658	20,7310314969917	9362434724,54813	3226731016,7289	1,53681043660165	0,0420716978950673	0,0460747827853159	0,344646570220508	-1.53681043660165	2,90152314401443	1.53681043660165	129
P28667	sp|P28667|MRP_MOUSE MARCKS-related protein OS=Mus musculus GN=Marcksl1 PE=1 SV=2	−2,89	7,19×10^−07	5,26×10^−05	26,8845106514255	26,4538794096732	26,501323798867	25,3737724117129	25,3969460609751	25,8813493021154	361395207223,534	124888760029,1	1,53293373305754	0,00887241518512049	0,0112166961458208	0,345573924426321	-1.53293373305754	2,89373685141342	1.53293373305754	130
Q69ZF7	sp|Q69ZF7|CNNM4_MOUSE Metal transporter CNNM4 OS=Mus musculus GN=Cnnm4 PE=1 SV=2	−2,89	0,00225625633970557	0,0423187914294363	22,8392616668271	22,3531370911114	23,4559970658618	22,0749258890454	21,7873155101343	21,602992944783	8667087197,69392	2999599993,78913	1,53077709377009	0,0241542787243101	0,0108993493573535	0,346090898287863	-1.53077709377009	2,88941432712352	1.53077709377009	131
O35598	sp|O35598|ADA10_MOUSE Disintegrin and metalloproteinase domain-containing protein 10 OS=Mus musculus GN=Adam10 PE=1 SV=2	−2,85	0,00442481956836872	0,0704710737572829	23,075311364659	23,4427204772675	23,6104580044563	22,4664435194351	21,6957403679723	22,8225685424939	14195108666,7785	4977784801,10851	1,51181812687163	0,0117089916945909	0,0257962294329657	0,350669016909906	-1.51181812687163	2,8516919139649	1.51181812687163	132
P97366	sp|P97366|EVI5_MOUSE Ecotropic viral integration site 5 protein OS=Mus musculus GN=Evi5 PE=1 SV=2	−2,85	0,0101794659750395	0,124583502622858	22,8773938140701	22,5448185137444	22,5362472274361	20,8934801258364	21,6835797836826	22,242612719966	6886438307,07392	2418848755,75513	1,50943744406974	0,00858762707769032	0,0313727063737382	0,351248155852994	-1.50943744406974	2,84699003635061	1.50943744406974	133
Q8BT51	sp|Q8BT51|COA4_MOUSE Cytochrome c oxidase assembly factor 4 homolog, mitochondrial OS=Mus musculus GN=COA4 PE=3 SV=1	−2,81	0,00770210586228892	0,100459363531406	22,5521406244179	22,0311999668215	22,0223540321112	21,740785022867	20,543586508048	21,2173839147839	4386941866,72611	1558908626,89862	1,49267921960636	0,0136632946150491	0,028353781600673	0,355352013830537	-1.49267921960636	2,81411096906541	1.49267921960636	134

Q9Z1Z0	sp|Q9Z1Z0|USO1_MOUSE General vesicular transport factor p115 OS=Mus musculus GN=Uso1 PE=1 SV=2;sp|Q9Z1Z0-2|USO1_MOUSE Isoform 2 of General vesicular transport factor p115 OS=Mus musculus GN=Uso1;sp|Q9Z1Z0-4|USO1_MOUSE Isoform 4 of General vesicular tran	−2,81	0,137438478808689	0,527876076506694	27,3412496695829	24,9476536404833	26,1438469895555	25,2054719742707	25,1735426829318	24,9546627427534	226101556397,169	80476077233,8112	1,49033903456711	0,0457767218755307	0,0054367804530880	0,355928895475833	-1.4903390345671	2,80954992053433	1.4903390345671	135
P22723-2	sp|P22723-2|GBRG2_MOUSE Isoform 2S of Gamma-aminobutyric acid receptor subunit gamma-2 OS=Mus musculus GN=Gabrg2;sp|P22723|GBRG2_MOUSE Gamma-aminobutyric acid receptor subunit gamma-2 OS=Mus musculus GN=Gabrg2 PE=1 SV=3;sp|Q9R0Y8|GBRG1_MOUSE Gamma-amino	−2,81	0,00236732112845223	0,0436799617969296	24,305821491108	24,1039857732243	24,8315460515407	23,4012908958398	23,8239823554939	22,9194574103533	40065619668,0254	14272163764,2177	1,48916071556613	0,0153848945279741	0,0193564620685393	0,356219718613455	-1.48916071556614	2,80725616170937	1.48916071556614	136
Q03517	sp|Q03517|SCG2_MOUSE Secretogranin-2 OS=Mus musculus GN=Scg2 PE=1 SV=1	−2,80	0,0001259503042659	0,00449513538302206	26,3537858638036	26,385525166963	26,9215383290468	25,5908651221833	25,1577695205742	25,8243737283388	340478035303,784	121640538129,642	1,48493762715856	0,0120143577531956	0,0132518739719121	0,357263980394832	-1.48493762715856	2,79905071564966	1.48493762715856	137
P51880	sp|P51880|FABP7_MOUSE Fatty acid-binding protein, brain OS=Mus musculus GN=Fabp7 PE=1 SV=2	−2,79	7,36×10^−14	1,28×10^−11	31,9425028325732	31,7966976397976	31,6098246071065	30,6034495341565	30,7314349400907	30,9382317000035	63560184465923,7	22798261327534,3	1,47919950793219	0,00524686171505536	0,0054922853692041	0,358687777876999	-1.47919950793219	2,78793999036934	1.47919950793219	138
Q8BGQ7	sp|Q8BGQ7|SYAC_MOUSE Alanine--tRNA ligase, cytoplasmic OS=Mus musculus GN=Aars PE=1 SV=1;sp|P49588|SYAC_HUMAN Alanine--tRNA ligase, cytoplasmic OS=Homo sapiens GN=AARS PE=1 SV=2;sp|P49588-2|SYAC_HUMAN Isoform 2 of Alanine--tRNA ligase, cytoplasmic OS=Ho	−2,77	0,251371111132889	0,673538059877323	24,723259791271	21,7796036535179	22,5764449156736	22,2152717627516	22,0562287492798	21,7460236150581	10005853614,7092	3605914949,7751	1,47240697658642	0,0661216608090732	0,0108447334444393	0,360380542093299	-1.47240697658643	2,77484459674604	1.47240697658643	139
Q9R078	sp|Q9R078|AAKB1_MOUSE 5-AMP-activated protein kinase subunit beta-1 OS=Mus musculus GN=Prkab1 PE=1 SV=2	−2,77	0,0487662972232661	0,323205843530114	22,242612719966	22,47968402626	23,3004795688856	21,7524718215974	20,9058908208185	22,3038750494383	7035670074,79428	2536571569,9984	1,47180796474088	0,0244826601389353	0,0325188956675274	0,360530204377522	-1.47180796474088	2,77369271106303	1.47180796474088	140
Q8K310	sp|Q8K310|MATR3_MOUSE Matrin-3 OS=Mus musculus GN=Matr3 PE=1 SV=1	−2,77	0,0251617289553339	0,219211300821997	25,3979425844979	23,8542629495858	24,391301183712	23,4318412203999	23,6030522836631	23,5506907098139	45813084388,2207	16531410441,3103	1,47054987727545	0,0319235574087182	0,0037286992050405	0,360844738180536	-1.47054987727544	2,7712749950082	1.47054987727545	141
P30999-2	sp|P30999-2|CTND1_MOUSE Isoform 2 of Catenin delta-1 OS=Mus musculus GN=Ctnnd1;sp|P30999-3|CTND1_MOUSE Isoform 3 of Catenin delta-1 OS=Mus musculus GN=Ctnnd1;sp|P30999|CTND1_MOUSE Catenin delta-1 OS=Mus musculus GN=Ctnnd1 PE=1 SV=2	−2,69	0,0901533900087159	0,441176105282424	26,9639504293006	25,1746957465022	26,2649632160102	25,2593441260161	25,5351249444596	24,6402971622954	223915920614,244	83234026838,5464	1,42771176757564	0,0345033741427965	0,0182245912191741	0,371720003697011	-1.42771176757564	2,69019689565886	1.42771176757564	142
Q9CQV6	sp|Q9CQV6|MLP3B_MOUSE Microtubule-associated proteins 1A/1B light chain 3B OS=Mus musculus GN=Map1lc3b PE=1 SV=3	−2,67	0,568691552627556	0,846628370075355	26,9340296968593	22,7884248163617	26,7026272203015	22,6646252533469	26,387321076432	24,4288410238104	115788054968,61	43394380344,9633	1,4159063002823	0,0914441020722251	0,0760277308036269	0,374774240371578	-1.4159063002823	2,66827303554409	1.4159063002823	143
P97467	sp|P97467|AMD_MOUSE Peptidyl-glycine alpha-amidating monooxygenase OS=Mus musculus GN=Pam PE=1 SV=2	−2,66	2,86×10^−12	4,32×10^−10	25,8313075112595	25,8903660723575	25,979047873499	24,6534719708188	25,048560475253	25,0594100709954	177146070404,394	66500868124,2106	1,41349438353253	0,00287115135091946	0,0092815258424675	0,375401316960633	-1.41349438353253	2,66381590798965	1.41349438353253	144
P54869	sp|P54869|HMCS2_MOUSE Hydroxymethylglutaryl-CoA synthase, mitochondrial OS=Mus musculus GN=Hmgcs2 PE=1 SV=2	−2,64	6,51×10^−22	2,27×10^−19	27,1520862359721	26,8602290837401	26,8991884585141	25,9945560886311	26,0769885460403	25,9308965147294	516582715783,347	195888942652,334	1,39896340941402	0,00587527452003621	0,0028170951687790	0,379201503781031	-1.39896340941402	2,63712034374592	1.39896340941402	145
B3EWD4	sp|B3EWD4|HBB_PERCR Hemoglobin subunit beta OS=Peromyscus crinitus PE=1 SV=1;sp|B3EWD6|HBB_PERCA Hemoglobin subunit beta OS=Peromyscus californicus PE=1 SV=1	−2,61	0,144772977697137	0,539068536314443	25,1843097620353	25,4699135689093	26,1622102512306	25,500765153124	23,5108751221745	24,9238571579815	131922156319,588	50496295348,1057	1,38543743170963	0,0196382779548821	0,0415416311494732	0,3827734230312	-1.38543743170963	2,61251157951604	1.38543743170963	146
P11627	sp|P11627|L1CAM_MOUSE Neural cell adhesion molecule L1 OS=Mus musculus GN=L1cam PE=1 SV=1;sp|P32004|L1CAM_HUMAN Neural cell adhesion molecule L1 OS=Homo sapiens GN=L1CAM PE=1 SV=2	−2,61	0,0366092304948057	0,279698526792889	24,5500906114161	24,5094875445525	24,4971412211746	23,985379748144	22,6427415317092	24,0503294468235	44506739275,6072	17051151508,6379	1,3841546361247	0,00112985559976772	0,0337267920009202	0,38311392355771	-1.3841546361247	2,61018965511277	1.3841546361247	147
P62264	sp|P62264|RS14_MOUSE 40S ribosomal protein S14 OS=Mus musculus GN=Rps14 PE=2 SV=3;sp|Q9SIH0|RS141_ARATH 40S ribosomal protein S14-1 OS=Arabidopsis thaliana GN=RPS14A PE=1 SV=1;sp|Q9CAX6|RS142_ARATH 40S ribosomal protein S14-2 OS=Arabidopsis thaliana GN=	−2,61	0,0329151298135633	0,264331930590857	28,6169807290695	28,5376790926525	27,4902079656322	26,9456779435363	27,7823931945621	27,0435496348728	179308002874,56	688104755591,749	1,38173973963311	0,0222896445938604	0,016782704466958	0,383755746809189	-1.38173973963311	2,60582416892696	1.38173973963311	148
Q9DAT5	sp|Q9DAT5|MTU1_MOUSE Mitochondrial tRNA-specific 2-thiouridylase 1 OS=Mus musculus GN=Trmu PE=2 SV=1	−2,58	0,00741965459560141	0,0979006168878919	24,2561303779626	23,7132586719014	24,0288934571652	22,4341047494528	23,2219874868566	23,493812176176	26473961509,6138	10244103794,8997	1,36978031719172	0,0113599679664758	0,0238784708535572	0,386950165776272	-1.36978031719172	2,58431211159678	1.36978031719172	149
P09813	sp|P09813|APOA2_MOUSE Apolipoprotein A-II OS=Mus musculus GN=Apoa2 PE=1 SV=2	−2,58	0,001413377072952	0,0308420185260236	24,8413906379364	24,1143670029103	24,0997473766248	23,168322265652	23,7280173231175	23,3213560979182	37658894531,3438	14623542430,5489	1,36469781617008	0,0174126765006435	0,0123587084869518	0,388315764775772	-1.36469781617008	2,57522380163329	1.36469781617008	150

Q920Q8-4	sp|Q920Q8-4|NS1BP_MOUSE Isoform 4 of Influenza virus NS1A-binding protein homolog OS=Mus musculus GN=Ivns1abp;sp|Q920Q8-3|NS1BP_MOUSE Isoform 3 of Influenza virus NS1A-binding protein homolog OS=Mus musculus GN=Ivns1abp;sp|Q920Q8|NS1BP_MOUSE Influenza v	−2,56	0,000126763015868434	0,00449513538302206	22,4148399246451	22,7369952893588	21,9143063520067	21,5152196899429	21,3395865968786	21,3956711244922	5114679226,1765	2000825742,94566	1,35404823759671	0,0185438573365072	0,0041886330705655	0,391192810822935	-1.35404823759671	2,55628419626717	1.35404823759671	151
P25911-2	sp|P25911-2|LYN_MOUSE Isoform 2 of Tyrosine-protein kinase Lyn OS=Mus musculus GN=Lyn;sp|P25911|LYN_MOUSE Tyrosine-protein kinase Lyn OS=Mus musculus GN=Lyn PE=1 SV=4	−2,55	0,0020988250254816З	0,0396940282944213	22,6095143587135	22,7620598330115	23,160204023241	22,2659402972637	21,4141158051472	22,0488781458796	8336639810,03408	3275199923,22565	1,34788303036941	0,0124464643319779	0,0202035174304411	0,392868109676944	-1.34788303036941	2,54538348969658	1.34788303036941	152
Q3UMY5-4	sp|Q3UMY5-4|EMAL4_MOUSE Isoform 4 of Echinoderm microtubule-associated protein-like 4 OS=Mus musculus GN=Eml4;sp|Q3UMY5-2|EMAL4_MOUSE Isoform 2 of Echinoderm microtubule-associated protein-like 4 OS=Mus musculus GN=Eml4;sp|Q3UMY5-3|EMAL4_MOUSE Isoform 3	−2,54	0,128675004554042	0,512962462222669	25,4007405161479	24,6683213514674	24,9705054088962	23,8072085417944	25,1861283043439	23,2438889303564	72960868919,231	28668799955,966	1,34764139498385	0,0147150562100027	0,0414988221358213	0,392933916229848	-1.34764139498385	2,5449572019511	1.34764139498385	153
Q8C838	sp|Q8C838|TUSC5_MOUSE Tumor suppressor candidate 5 homolog OS=Mus musculus GN=Tusc5 PE=2 SV=1	−2,51	0,00103017586505548	0,0242278147745432	23,3145151636562	23,9931989064267	24,0114271695296	23,1309610408383	22,5716530900109	22,8549362691204	21110725796,7421	8408566125,23179	1,32804446977244	0,0167082017397732	0,0122376816262238	0,39830777047606	-1.32804446977244	2,51062136901018	1.32804446977244	154
P22935	sp|P22935|RABP2_MOUSE Cellular retinoic acid-binding protein 2 OS=Mus musculus GN=Crabp2 PE=2 SV=2	−2,50	0,0659580643025308	0,382601734678419	25,0264181578287	23,9449572067503	23,9631070192037	22,7276309950859	23,5823483342612	23,8813417836964	36169936522,3269	14495542679,2029	1,31918172055143	0,0254697992353328	0,0255906399059702	0,400762181881495	-1.31918172055143	2,49524542287201	1.31918172055143	155
Q64152-2	sp|Q64152-2|BTF3_MOUSE Isoform 2 of Transcription factor BTF3 OS=Mus musculus GN=Btf3;sp|Q64152|BTF3_MOUSE Transcription factor BTF3 OS=Mus musculus GN=Btf3 PE=1 SV=3	−2,48	0,0706634898182645	0,395977259611237	24,5934480971174	25,1239866486859	24,7396961746093	23,075311364659	23,9198035652238	24,7312567640459	60086056112,9824	24179972979,1026	1,31321759797004	0,0110409245575564	0,034632769389974	0,402422367905724	-1.31321759797004	2,48495133410246	1.31321759797004	156
Q5SQY2	sp|Q5SQY2|BOD1_MOUSE Biorientation of chromosomes in cell division protein 1 OS=Mus musculus GN=Bod1 PE=2 SV=1	−2,48	0,605770158950647	0,865467658632982	20,9058908208185	22,376705503092	20,966955718834	17,1235419917144	22,0092765517313	22,3884232655303	2000208671,03545	805586066,159491	1,31203988142149	0,0388535480532391	0,143187435486669	0,40275101184441	-1.31203988142149	2,48292361928644	1.31203988142149	157
O54962	sp|O54962|BAF_MOUSE Barrier-to-autointegration factor OS=Mus musculus GN=Banf1 PE=1 SV=1	−2,48	0,0426793934659114	0,304115803676251	24,8771377433071	24,9676751874106	24,8516604772475	23,1009237110647	24,3939467194112	24,4804791910647	65076183171,5206	26272358272,7555	1,30858393077318	0,00244879163149378	0,0322076597882612	0,403716951307819	-1.30858393077318	2,47698293757682	1.30858393077318	158
P11139	sp|P11139|TBA1_ARATH Tubulin alpha-1 chain OS=Arabidopsis thaliana GN=TUBA1 PE=2 SV=1	−2,47	0,639578353286897	0,885863059323783	28,4918017637118	26,6767161700113	28,3918361374394	23,277183681821	28,8233279788742	28,7501446235731	1249108680195,82	506210668968,554	1,30308918648673	0,036631280141569	0,118038082498251	0,405257506407846	-1.30308918648673	2,46756687831369	1.30308918648673	159
Q9DC04-1	sp|Q9DC04-1|RGS3_MOUSE Isoform 5 of Regulator of G-protein signaling 3 OS=Mus musculus GN=Rgs3;sp|Q9DC04-4|RGS3_MOUSE Isoform 2 of Regulator of G-protein signaling 3 OS=Mus musculus GN=Rgs3;sp|Q9DC04|RGS3_MOUSE Regulator of G-protein signaling 3 OS=Mus	−2,46	0,0267645117585229	0,227925176119954	23,7519013740161	23,2077788046151	23,0297539046255	22,7406104302362	21,7420055571971	22,8039538529234	13552154307,0604	5504636716,84062	1,29980294647598	0,016126245925806	0,0265583331328677	0,406181673564093	-1.29980294647597	2,46195253278015	1.29980294647597	160
Q9QYE9-4	sp|Q9QYE9-4|PKHB1_MOUSE Isoform 4 of Pleckstrin homology domain-containing family B member 1 OS=Mus musculus GN=Plekhb1;sp|Q9QYE9-2|PKHB1_MOUSE Isoform 2 of Pleckstrin homology domain-containing family B member 1 OS=Mus musculus GN=Plekhb1;sp|Q9QYE9-3|P	−2,44	0,00949445258180679	0,118393737002256	24,6569155403246	24,558208524557	24,2472581965185	22,9673450736272	23,7779715892546	24,0352949440679	43128969472,4551	17641804925,256	1,28965907465911	0,00873083493523116	0,0236230797380835	0,409047680504473	-1.2896590746591	2,44470277588841	1.2896590746591	161
P07310	sp|P07310|KCRM_MOUSE Creatine kinase M-type OS=Mus musculus GN=Ckm PE=1 SV=1	−2,43	0,129880091170252	0,513077226998934	24,4661695074578	23,6185544152518	24,0692182003921	23,7280173231175	23,6630592406412	22,0969453884394	27883864943,1507	11466252399,6327	1,28203660332285	0,0176327618719464	0,0398710156994811	0,411214601096726	-1.28203660332285	2,43182026448711	1.28203660332285	162
P09602	sp|P09602|HMGN2_MOUSE Non-histone chromosomal protein HMG-17 OS=Mus musculus GN=Hmgn2 PE=1 SV=2	−2,42	0,00319759675601122	0,0555356062729631	25,369136158521	24,8983311212619	24,6548084016843	23,707498296656	24,1143670029103	24,4487515518704	70163347330,1022	28989676394,1355	1,27517834807149	0,0145406769764602	0,0154094499912672	0,413174078735808	-1.27517834807149	2,42028735940964	1.27517834807149	163

P17426-2	sp|P17426-2|AP2A1_MOUSE Isoform B of AP-2 complex subunit alpha-1 OS=Mus musculus GN=Ap2a1;sp|P17426|AP2A1_MOUSE AP-2 complex subunit alpha-1 OS=Mus musculus GN=Ap2a1 PE=1 SV=1	−2,42	0,0662392418326818	0,383006265832538	32,799089045904	31,2460357843299	32,077801964065	31,0970439805281	31,4825297714827	30,8944000450301	82265718862063,5	34020733573822,9	1,27387711756401	0,0242558557268514	0,0095886875287843	0,413546906833284	-1.27387711756401	2,41810537928443	1.27387711756401	164
Q0PHV7	sp|Q0PHV7|DACT3_MOUSE Dapper homolog 3 OS=Mus musculus GN=Dact3 PE=1 SV=1	−2,40	0,274025376577183	0,695562886495618	26,1449400230198	25,7246189853418	25,1199552746439	23,3453837113846	25,7991721647102	25,2247645740049	139757054574,544	58352817654,5985	1,26004688296705	0,0200773233231718	0,0517723985278705	0,417530391093597	-1.26004688296705	2,39503523894583	1.26004688296705	165
P22005	sp|P22005|PENK_MOUSE Proenkephalin-A OS=Mus musculus GN=Penk PE=2 SV=2	−2,39	2,22×10^−08	2,19×10^−06	24,1861387683333	24,34213713151	24,0050047487481	23,4559970658618	23,0632449784514	23,3944655483203	31642285640,7058	13214350003,7262	1,25974835235893	0,00697840858725368	0,0090645432062035	0,41761679778046	-1.25974835235893	2,39453969599589	1.25974835235893	166
P28063	sp|P28063|PSB8_MOUSE Proteasome subunit beta type-8 OS=Mus musculus GN=Psmb8 PE=1 SV=2	−2,39	0,543107566723731	0,83367103326311	27,596320761955	23,5434255149496	23,1878192907474	23,4741435389005	24,1736455229619	24,0697373811504	57546271865,0393	24108785333,3915	1,2551635005477	0,0988485625835653	0,0157892209038987	0,418946085507203	-1.2551635005477	2,38694198273588	1.2551635005477	167
Q8CD19	sp|Q8CD19|LANC3_MOUSE LanC-like protein 3 OS=Mus musculus GN=Lancl3 PE=2 SV=2	−2,39	0,0521132335695617	0,335456444875023	25,3937401987218	25,5795222243761	24,6092541358523	24,3979637610652	23,7113493878119	24,8641690818116	87435934615,6087	36643213474,182	1,25468029563063	0,0204412577538907	0,0238406152677563	0,419086427511471	-1.25468029563063	2,38614265305127	1.25468029563063	168
O88895	sp|O88895|HDAC3_MOUSE Histone deacetylase 3 OS=Mus musculus GN=Hdac3 PE=1 SV=1;sp|O88895-2|HDAC3_MOUSE Isoform Short of Histone deacetylase 3 OS=Mus musculus GN=Hdac3	−2,38	0,0284419773227845	0,23818844108509	24,132235856752	23,6104580044563	23,9166756183545	22,5971910117934	22,7116660906337	23,7476238449328	23645921703,5011	9930006627,77875	1,25172479246532	0,0109768588639518	0,0275316646891183	0,419945847418943	-1.25172479246532	2,38125940795978	1.25172479246532	169
Q63844	sp|Q63844|MK03_MOUSE Mitogen-activated protein kinase 3 OS=Mus musculus GN=Mapk3 PE=1 SV=5;sp|P21708|MK03_RAT Mitogen-activated protein kinase 3 OS=Rattus norvegicus GN=Mapk3 PE=1 SV=5;sp|P21708-2|MK03_RAT Isoform 2 of Mitogen-activated protein kinase 3	−2,34	0,282169351930876	0,703331514779926	32,9992079572545	32,6569761311727	32,5833119363487	30,3453333642794	32,7587169966684	32,5913113535174	166580313918568	71338259964601,3	1,22346998428025	0,00677722442590299	0,0422479337371185	0,428251443921967	-1.22346998428025	2,33507677368675	1.22346998428025	170
P57722-2	sp|P57722-2|PCBP3_MOUSE Isoform 2 of Poly(rC)-binding protein 3 OS=Mus musculus GN=Pcbp3;sp|P57722|PCBP3_MOUSE Poly(rC)-binding protein 3 OS=Mus musculus GN=Pcbp3 PE=2 SV=3	−2,32	7,84×10^−19	1,98×10^−16	29,6188712147222	29,3328195161808	29,3473309859604	28,6120839248889	28,5498933163116	28,6073269178441	6061674748419,82	2608425902973,66	1,21653699183831	0,00547434293592369	0,0012107251193686	0,430314395151874	-1.2165369918383	2,32388228529297	1.2165369918383	171
Q99880	sp|Q99880|H2B1L_HUMAN Histone H2B type 1-L OS=Homo sapiens GN=HIST1H2BL PE=1 SV=3;sp|Q99879|H2B1M_HUMAN Histone H2B type 1-M OS=Homo sapiens GN=HIST1H2BM PE=1 SV=3;sp|Q99877|H2B1N_HUMAN Histone H2B type 1-N OS=Homo sapiens GN=HIST1H2BN PE=1 SV=3;sp|Q93	−2,30	0,0366026001417582	0,279698526792889	27,9313864300688	28,0469322356155	27,7146979344159	26,4776551762052	27,7938677088192	26,9215383290468	1305584827550,16	567413265230,89	1,20222441262259	0,00604579312383141	0,0247420479221363	0,434604671605752	-1.20222441262259	2,30094167259008	1.20222441262259	172
Q9Z0L0	sp|Q9Z0L0|TPBG_MOUSE Trophoblast glycoprotein OS=Mus musculus GN=Tpbg PE=1 SV=3	−2,30	3,53×10^−05	0,00156991426989324	24,5525942656835	25,1379645009733	25,0441535538573	24,2411722072097	23,9449572067503	24,0531878454148	65910972937,2288	28688793692,6824	1,20003135992151	0,012620696455812	0,0062245706474000	0,435265820154173	-1.20003135992151	2,29744664914372	1.20003135992151	173
P41778-2	sp|P41778-2|PBX1_MOUSE Isoform PBX1b of Pre-B-cell leukemia transcription factor 1 OS=Mus musculus GN=Pbx1;sp|P41778|PBX1_MOUSE Pre-B-cell leukemia transcription factor 1 OS=Mus musculus GN=Pbx1 PE=1 SV=2	−2,29	2,54×10^−05	0,0011411325802856	26,8802456280836	26,5247335525472	26,8149454374718	25,839608189605	26,223070070365	25,668783905484	410226331630,763	178970600894,385	1,19669747995817	0,0070771308128524	0,0109553880415484	0,43627282574214	-1.19669747995817	2,29214367935686	1.19669747995817	174
Q91WE3	sp|Q91WE3|RPP25_MOUSE Ribonuclease P protein subunit p25 OS=Mus musculus GN=Rpp25 PE=2 SV=1	−2,29	0,006836657747831	0,0927193592980021	22,8462665924133	23,0175812679144	22,954648045036	22,7116660906337	21,7524718215974	21,8690502708892	9172710244,61715	4006014636,01467	1,19518037532122	0,00377760057962012	0,0236711222815138	0,436731841427732	-1.19518037532122	2,28973458113535	1.19518037532122	175
Q9CTN8	sp|Q9CTN8|LHPL3_MOUSE Lipoma HMGIC fusion partner-like 3 protein OS=Mus musculus GN=Lhfpl3 PE=2 SV=2	−2,29	7,76×10^−06	0,00041263171826067	24,2627398725708	24,0297670368369	23,7476238449328	22,9695985789723	23,252854447264	23,3326444849489	26845845558,1354	11725508318,4906	1,19504837878146	0,010741881389971	0,0082289729420916	0,436771801174923	-1.19504837878146	2,28952509596541	1.19504837878146	176
Q9WV32	sp|Q9WV32|ARC1B_MOUSE Actin-related protein 2/3 complex subunit 1B OS=Mus musculus GN=Arpc1b PE=2 SV=4	−2,28	0,204311730616057	0,635648869851434	23,6204012521585	24,8802711146083	25,3711772595212	23,3258529818771	24,567454870373	23,5046582728801	49436253842,4218	21672760700,1354	1,18968648628581	0,0366754504098859	0,0282026225393031	0,438398119105412	-1.18968648628581	2,28103168426129	1.18968648628581	177
P20918	sp|P20918|PLMN_MOUSE Plasminogen OS=Mus musculus GN=Plg PE=1 SV=3	−2,27	0,207976850287038	0,638546806637671	23,7163979450698	23,1560227153882	22,7826561503887	23,1651192385014	21,2326685501382	22,7962312002305	12122862104,5452	5337409872,3882	1,18351863836891	0,020241813405705	0,0458044434605048	0,440276382454854	-1.18351863836891	2,27130057357219	1.18351863836891	178
Q9JJR8	sp|Q9JJR8|TMM9B_MOUSE Transmembrane protein 9B OS=Mus musculus GN=Tmem9b PE=1 SV=1	−2,27	1,38×10^−06	9,78×10^−05	23,9115536096075	23,5141970803436	23,3576826764757	22,6960719862823	22,8163576649882	22,8146495088482	17658406374,9948	7786778383,82276	1,18125667736907	0,0121015319715396	0,0030277522538494	0,440967220850078	-1.18125667736907	2,26774225547251	1.18125667736907	179

Q9DAW9	sp|Q9DAW9|CNN3_MOUSE Calponin-3 OS=Mus musculus GN=Cnn3 PE=1 SV=1	−2,26	0,208065813398792	0,638546806637671	30,5343188057023	30,0920533297203	30,2268071384523	29,9877542613183	30,2119986671099	28,2047593538113	14201846272748,4	6278616444470,81	1,17755990854024	0,00748525996424291	0,0373242105260013	0,442098606328299	-1.17755990854024	2,26193882017671	1.17755990854024	180
Q9Z1S8	sp|Q9Z1S8|GAB2_MOUSE GRB2-associated-binding protein 2 OS=Mus musculus GN=Gab2 PE=1 SV=2	−2,21	0,338792444120811	0,729620681454069	23,8091764130591	23,6693854597697	23,4615901922734	21,2017394076376	23,6580266549454	23,6951786116361	18605393616,9219	8401265823,69563	1,14704229143961	0,0073963050339616	0,0625330689140015	0,451550018057911	-1.14704229143961	2,21459408705361	1.14704229143961	181
E9Q5R7	sp|E9Q5R7|NAL12_MOUSE NACHT, LRR and PYD domains-containing protein 12 OS=Mus musculus GN=Nlrp12 PE=2 SV=1	−2,21	0,126638847791659	0,509135279119877	26,6691328676683	25,3969460609751	25,9719342937484	25,5170328077361	25,6510636985623	24,49311460887	198225493189,517	89759858747,9701	1,14300020442192	0,0244907943807811	0,0251148233999953	0,452816927347249	-1.14300020442192	2,20839800724397	1.14300020442192	182
Q9CQ49	sp|Q9CQ49|NCBP2_MOUSE Nuclear cap-binding protein subunit 2 OS=Mus musculus GN=Ncbp2 PE=1 SV=1	−2,21	0,173322769373735	0,587537005367725	22,4569741763383	22,3038750494383	23,6185544152518	22,7607929420049	21,3656840009079	21,8769368165872	7923782736,61927	3588994511,42338	1,14260960642782	0,0315411478340355	0,032080095605728	0,452939540459264	-1.14260960642782	2,20780018230697	1.14260960642782	183
Q9DC63-3	sp|Q9DC63-3|FBX3_MOUSE Isoform 3 of F-box only protein 3 OS=Mus musculus GN=Fbxo3;sp|Q9DC63-2|FBX3_MOUSE Isoform 2 of F-box only protein 3 OS=Mus musculus GN=Fbxo3;sp|Q9DC63|FBX3_MOUSE F-box only protein 3 OS=Mus musculus GN=Fbxo3 PE=2 SV=1	−2,17	0,00232897339765283	0,0431477969467192	25,6757304025179	25,2978380035958	25,4643078754517	24,7005673453217	24,3086666205643	25,10451441028	116282928108,902	53587560188,7001	1,11766926783715	0,00743314575046841	0,0161079056811946	0,460837726226794	-1.11766926783715	2,16996123166328	1.11766926783715	184
P50153	sp|P50153|GBG4_MOUSE Guanine nucleotide-binding protein G(I)/G(S)/G(O) subunit gamma-4 OS=Mus musculus GN=Gng4 PE=2 SV=1	−2,16	0,0132785556121675	0,14987902972529	27,7842060823852	27,2578083209851	26,9742992198464	26,2503112648405	26,5121165550575	26,9415112903228	746581935717,889	345403696058,125	1,11201708085904	0,0150328167677691	0,0131349750784277	0,462646736457662	-1.11201708085904	2,16147639483352	1.11201708085904	185
Q6NZK8-2	sp|Q6NZK8-2|PTPC1_MOUSE Isoform 2 of Protein tyrosine phosphatase domain-containing protein 1 OS=Mus musculus GN=Ptpdc1;sp|Q6NZK8|PTPC1_MOUSE Protein tyrosine phosphatase domain-containing protein 1 OS=Mus musculus GN=Ptpdc1 PE=2 SV=1;sp|Q6NZK8-3|PTPC1_	−2,15	2,45×10^−05	0,00111028049832639	24,6631481372109	24,3302859701029	24,2934651158925	23,9166756183545	23,4411557402849	23,6328120342077	40678535530,441	18921180588,9344	1,10426563302384	0,00833605642937376	0,0101102878565443	0,465139178247336	-1.10426563302383	2,14989415376284	1.10426563302383	186
Q8BHK2	sp|Q8BHK2|SCN3B_MOUSE Sodium channel subunit beta-3 OS=Mus musculus GN=Scn3b PE=2 SV=1	−2,15	0,0043774648364393	0,0699623693401338	24,5323306407483	24,5917632179099	24,3651120990611	23,8813417836964	24,0874845308129	23,2262234311461	43516324735,0606	20255318713,9346	1,10325593005637	0,00479741894834138	0,0189498589596226	0,465464830434431	-1.10325593005636	2,14839002780656	1.10325593005636	187
O35136-2	sp|O35136-2|NCAM2_MOUSE Isoform Short of Neural cell adhesion molecule 2 OS=Mus musculus GN=Ncam2;sp|O35136|NCAM2_MOUSE Neural cell adhesion molecule 2 OS=Mus musculus GN=Ncam2 PE=2 SV=1	−2,14	0,000690544655235141	0,0176234557416597	25,9308965147294	25,7769511586397	25,484150064461	25,0087957507153	24,6324302907486	25,2615134752874	149515501387,116	69707924239,4559	1,10090049428821	0,0088198755626366	0,012678857389526	0,466225398655973	-1.10090049428822	2,14488529128354	1.10090049428822	188
P62960	sp|P62960|YBOX1_MOUSE Nuclease-sensitive element-binding protein 1 OS=Mus musculus GN=Ybx1 PE=1 SV=3	−2,14	0,0979332838769655	0,46120994380421	26,9395921810098	26,2722099307776	27,02680358158	25,2026579862367	26,3230679595192	26,4316752166706	412788791259,772	192969964130,897	1,09702748801397	0,0154339175373069	0,0261832121744168	0,467478691807451	-1.09702748801398	2,13913493283217	1.09702748801398	189
Q8BMQ2	sp|Q8BMQ2|TF3C4_MOUSE General transcription factor 3C polypeptide 4 OS=Mus musculus GN=Gtf3c4 PE=2 SV=2;sp|Q8BMQ2-3|TF3C4_MOUSE Isoform 3 of General transcription factor 3C polypeptide 4 OS=Mus musculus GN=Gtf3c4;sp|Q8BMQ2-2|TF3C4_MOUSE Isoform 2 of Gen	−2,12	0,0332488120926771	0,265230857800811	25,0866146564925	25,5077187593753	25,2754018283628	23,8848298274034	24,7672234633243	24,9622562481828	96220834698,5685	45369376122,0795	1,08463049338628	0,00834034910054436	0,0233960247830298	0,471513017572633	-1.08463049338628	2,12083222038713	1.08463049338628	190
Q9DB27	sp|Q9DB27|MCTS1_MOUSE Malignant T-cell-amplified sequence 1 OS=Mus musculus GN=Mcts1 PE=2 SV=1;sp|Q9DB27-2|MCTS1_MOUSE Isoform 2 of Malignant T-cell-amplified sequence 1 OS=Mus musculus GN=Mcts1	−2,12	0,0152270268145988	0,165744543672575	28,2288491072506	27,4410098735373	27,2481132134366	26,7447857389232	26,9078396230827	27,0100387989245	1008337513890,28	475462913101,766	1,08457390510349	0,0187983602851205	0,0049757190603737	0,471531512566042	-1.0845739051035	2,12074903447719	1.0845739051035	191
Q7TNG8	sp|Q7TNG8|LDHD_MOUSE Probable D-lactate dehydrogenase, mitochondrial OS=Mus musculus GN=Ldhd PE=1 SV=1	−2,12	0,172995087169709	0,586864499748362	24,9892097138287	24,5032761075892	24,5199292078442	24,5565050782037	22,8730078302888	24,3302859701029	51807719994,7016	24450902384,5991	1,08327938319775	0,0111821340863315	0,0381979301708054	0,471954804942192	-1.08327938319775	2,11884695214086	1.08327938319775	192
Q9Z2M6	sp|Q9Z2M6|UBL3_MOUSE Ubiquitin-like protein 3 OS=Mus musculus GN=Ubl3 PE=1 SV=1	−2,12	0,063503895417872	0,376789779479374	22,9337468186366	23,6519620919524	22,6809000175204	21,8141497464129	22,4285357176485	22,7738720080143	10650467690,1229	5030838418,12584	1,08204602578827	0,0218188882674971	0,0217605614224068	0,472358450774079	-1.08204602578827	2,11703632773214	1.08204602578827	193
Q9D387	sp|Q9D387|LAMP5_MOUSE Lysosome-associated membrane glycoprotein 5 OS=Mus musculus GN=Lamp5 PE=1 SV=2	−2,11	6,25×10^−12	9,15×10^−10	26,140845402906	26,1771723436785	26,0949756141312	25,5256101773492	25,4664846831164	25,1824661258207	224617423868,352	106511663098,531	1,07645842865162	0,00157590922369446	0,0072246165974038	0,474191455249515	-1.07645842865162	2,1088528461016	1.07645842865162	194
Q9CZ83	sp|Q9CZ83|RM55_MOUSE 39S ribosomal protein L55, mitochondrial OS=Mus musculus GN=Mrpl55 PE=2 SV=1;sp|Q9CZ83-2|RM55_MOUSE Isoform 2 of 39S ribosomal protein L55, mitochondrial OS=Mus musculus GN=Mrpl55	−2,10	0,137260566163534	0,527876076506694	23,9303837516722	23,9753136992594	23,5823483342612	23,4079437247829	23,7163979450698	22,1323356543618	22333387545,2207	10615267246,2842	1,07306140434981	0,00902605106893838	0,0363776727309399	0,475309319949353	-1.07306140434981	2,10389310293044	1.07306140434981	195

Q60604	sp|Q60604|ADSV_MOUSE Adseverin OS=Mus musculus GN=Scin PE=1 SV=3;sp|Q60604-2|ADSV_MOUSE Isoform 2 of Adseverin OS=Mus musculus GN=Scin	−2,09	0,187674286023697	0,612844305224145	23,6723843197498	21,968529841547	22,8163576649882	22,4253348908122	21,5496008959617	22,2659402972637	8132145369,33121	3884623978,35114	1,06586104866258	0,0373341138517002	0,0211252154841636	0,477687473837007	-1.06586104866259	2,09341892925836	1.06586104866259	196
Q3U1J4	sp|Q3U1J4|DDB1_MOUSE DNA damage-binding protein 1 OS=Mus musculus GN=Ddb1 PE=1 SV=2	−2,09	0,269835466996079	0,690018695602932	22,484003771526	21,882883155524	20,744494383141	21,829564652429	20,4890801292557	20,5785356206181	2665863267,99411	1274381501,20829	1,06480555644735	0,0407060146966895	0,0357460263721682	0,478037083337429	-1.06480555644735	2,0918879201138	1.06480555644735	197
Q8BTU1	sp|Q8BTU1|CFA20_MOUSE Cilia- and flagella-associated protein 20 OS=Mus musculus GN=Cfap20 PE=2 SV=1	−2,08	0,357945434130797	0,732843629012038	26,4776551762052	26,2550580787104	24,7119475537275	24,2686184132209	26,190885557456	24,7814467480862	162653325632,602	78026699492,8232	1,059760539409	0,0372512193748517	0,0396874745174123	0,479711676286708	-1.059760539409	2,08458549047769	1.059760539409	198
Q99JB8	sp|Q99JB8|PACN3_MOUSE Protein kinase C and casein kinase II substrate protein 3 OS=Mus musculus GN=Pacsin3 PE=1 SV=1	−2,08	0,337578101163338	0,729620681454069	23,9469638537138	23,8225765445107	23,9878581132619	21,665177458907	24,021910159197	23,8733808899669	24431346495,238	11746519677,7566	1,05650000703586	0,00359895220497267	0,0569234742025903	0,480797064543543	-1.05650000703586	2,07987958693004	1.05650000703586	199
P29391	sp|P29391|FRIL1_MOUSE Ferritin light chain 1 OS=Mus musculus GN=Ftl1 PE=1 SV=2;sp|P49945|FRIL2_MOUSE Ferritin light chain 2 OS=Mus musculus GN=Ftl2 PE=2 SV=2	−2,07	6,93×10^−06	0,00039209630007597	26,4815901695906	26,2857612704694	26,072361667895	25,35089230623	25,5815878578865	25,7291565919797	258950916555,677	125287748746,213	1,04743331716815	0,00778835061719533	0,0074606436442383	0,483828172584494	-1.04743331716815	2,06684946570647	1.04743331716815	200
Q61137-2	sp|Q61137-2|ASTN1_MOUSE Isoform 2 of Astrotactin-1 OS=Mus musculus GN=Astn1;sp|Q61137|ASTN1_MOUSE Astrotactin-1 OS=Mus musculus GN=Astn1 PE=2 SV=4	−2,06	0,057756889587593	0,355202783224204	25,3969460609751	25,3323809620451	25,4222737780244	24,5687279499805	24,0597088731534	25,3615584974045	105699598748,653	51421991778,3236	1,03951250236156	0,00182602870012402	0,026600648109228	0,486491835230159	-1.03951250236156	2,05553295571117	1.03951250236156	201
Q64338-2	sp|Q64338-2|PDE1C_MOUSE Isoform 2 of Calcium/calmodulin-dependent 3,5-cyclic nucleotide phosphodiesterase 1C OS=Mus musculus GN=Pde1c;sp|Q64338-1|PDE1C_MOUSE Isoform 1 of Calcium/calmodulin-dependent 3,5-cyclic nucleotide phosphodiesterase 1C OS=Mus	−2,05	1,67×10^−05	0,00080617261727272	23,0900203571021	23,0829978435626	22,7144546071874	22,4077897022244	22,0311999668215	22,2942056201138	9386055205,16093	4577406598,01278	1,03598849763871	0,00935588644524883	0,0086840858673808	0,48768161895062	-1.03598849763871	2,05051812728102	1.03598849763871	202
P07759	sp|P07759|SPA3K_MOUSE Serine protease inhibitor A3K OS=Mus musculus GN=Serpina3k PE=1 SV=2	−2,04	7,40×10^−06	0,0003996089980128	29,8451084763157	30,3198830461946	30,0523063560686	29,5177234927931	29,2565888136709	29,2978797219439	11489246492167,7	5620250449463,36	1,03157785740786	0,00791509987120743	0,0047815219809198	0,489174851744605	-1.03157785740786	2,0442588093676	1.03157785740786	203
Q6ZWY3	sp|Q6ZWY3|RS27L_MOUSE 40S ribosomal protein S27-like OS=Mus musculus GN=Rps27l PE=2 SV=3;sp|Q6ZWU9|RS27_MOUSE 40S ribosomal protein S27 OS=Mus musculus GN=Rps27 PE=1 SV=3;sp|O64650|RS271_ARATH 40S ribosomal protein S27-1 OS=Arabidopsis thaliana GN=RPS27	−2,03	1,47×10^−06	0,00010142846166440	26,8016686908167	26,6015089080294	26,6541371203836	25,7191474648085	26,0346229337524	26,1745346539103	388582590793,976	191107452274,103	1,02383722941241	0,00388828676309115	0,0089800150066886	0,491806521449199	-1.02383722941242	2,03331992640788	1.02383722941242	204
Q8R0N6-2	sp|Q8R0N6-2|HOT_MOUSE Isoform 2 of Hydroxyacid-oxoacid transhydrogenase, mitochondrial OS=Mus musculus GN=Adhfe1;sp|Q8R0N6|HOT_MOUSE Hydroxyacid-oxoacid transhydrogenase, mitochondrial OS=Mus musculus GN=Adhfe1 PE=1 SV=2	−2,03	1,24×10^−05	0,00061295066481661	24,4430889369582	24,5231160339711	24,2965262702175	23,5823483342612	23,5506907098139	24,0084076421851	40352145952,9106	19896617156,4175	1,02012223588309	0,00470545388530322	0,0107791506873103	0,493074573521718	-1.02012223588309	2,02809078727714	1.02012223588309	205
P62281	sp|P62281|RS11_MOUSE 40S ribosomal protein S11 OS=Mus musculus GN=Rps11 PE=1 SV=3;sp|P62280|RS11_HUMAN 40S ribosomal protein S11 OS=Homo sapiens GN=RPS11 PE=1 SV=3	−2,03	8,34×10^−05	0,00323598342950993	29,1231989742319	28,9384359919015	28,6363686210369	27,9843470437695	28,3588682131998	28,2346990589835	3554851936882,05	1753505609089	1,0195474259424	0,0085039783855746	0,0067666765355812	0,493271067325238	-1.01954742594241	2,0272829002976	1.01954742594241	206
Q9WVI9-5	sp|Q9WVI9-5|JIP1_MOUSE Isoform JIP-1e of C-Jun-amino-terminal kinase-interacting protein 1 OS=Mus musculus GN=Mapk8ip1;sp|Q9WVI9-4|JIP1_MOUSE Isoform JIP-1d of C-Jun-amino-terminal kinase-interacting protein 1 OS=Mus musculus GN=Mapk8ip1;sp|Q9WVI9-3|JIP	−2,03	0,481417984109515	0,802303567138125	24,6044469627772	24,7017312592705	24,1529970070456	21,8106908372461	24,6249971441151	24,9059090438174	43082888774,4046	21269337993,6259	1,01833985782747	0,0119575405409029	0,0719792614660165	0,493684119117424	-1.01833985782747	2,02558672899532	1.01833985782747	207
P52912	sp|P52912|TIA1_MOUSE Nucleolysin TIA-1 OS=Mus musculus GN=Tia1 PE=1 SV=1	−2,02	0,0100881916230778	0,124430167872691	25,6777150180066	24,9575896134171	25,2267225649949	24,9114903418552	24,3120591899788	24,5262771526765	95973927098,8747	47465812950,6154	1,01575373463104	0,0143894040928401	0,0123560936805331	0,494569873146016	-1.01575373463105	2,02195898759236	1.01575373463105	208
P02089	sp|P02089|HBB2_MOUSE Hemoglobin subunit beta-2 OS=Mus musculus GN=Hbb-b2 PE=1 SV=2	−2,01	9,49×10^−15	1,79×10^−12	26,5982162339486	26,5520528301124	26,4538794096732	25,6863781120229	25,956784137851	25,8662283570416	334103331528,923	166200966667,01	1,00736559544078	0,00277800726160206	0,0053272903102778	0,497453784451779	-1.00736559544078	2,01023699337629	1.00736559544078	209
Q91Z67	sp|Q91Z67|SRGP2_MOUSE SLIT-ROBO Rho GTPase-activating protein 2 OS=Mus musculus GN=Srgap2 PE=1 SV=2	−2,00	0,222918197283345	0,652238880554131	26,9261638707406	25,1396857445748	25,3890942487746	25,3115808292264	25,114611226522	24,9510957123413	163211806622,999	81654485402,675	0,999141382124152	0,0374732460669432	0,0071839050565690	0,500297662847932	-0.999141382124155	1,99881005701191	0.999141382124155	210

Q9CQE7	sp|Q9CQE7|ERGI3_MOUSE Endoplasmic reticulum-Golgi intermediate compartment protein 3 OS=Mus musculus GN=Ergic3 PE=2 SV=1;sp|Q9CQE7-2|ERGI3_MOUSE Isoform 2 of Endoplasmic reticulum-Golgi intermediate compartment protein 3 OS=Mus musculus GN=Ergic3	−1,99	0,617162652559441	0,870166479711399	23,0507037791687	22,4605995625438	24,473229654249	24,547663319805	23,0651904909605	20,3022376764705	13530032285,2081	6787603896,19033	0,995191000682667	0,0443501439142084	0,0951772270794833	0,501669453044172	-0.995191000682668	1,99334441021258	0.995191000682668	211
Q8CAM5	sp|Q8CAM5|RAB36_MOUSE Ras-related protein Rab-36 OS=Mus musculus GN=Rab36 PE=2 SV=1	−1,99	0,00634945051200206	0,0889510983764734	22,8730078302888	23,2631031706819	22,5552010672857	22,4605995625438	21,9501523629703	22,2152717627516	8791964556,92059	4416770291,91307	0,993193767939395	0,0154851754705324	0,0114949471416707	0,502364433263827	-0.993193767939397	1,99058678080187	0.993193767939397	212
O88533	sp|O88533|DDC_MOUSE Aromatic-L-amino-acid decarboxylase OS=Mus musculus GN=Ddc PE=2 SV=1	−1,98	0,0731925194248714	0,404161612736608	27,4336352949716	26,6226931767961	27,067317939297	26,8127695771732	26,4706027605894	25,7861812046239	554435031166,107	279569804874,203	0,987809865055856	0,0150178421924232	0,0198325292769598	0,50424267796752	-0.987809865055856	1,98317207902901	0.987809865055856	213
P97792-2	sp|P97792-2|CXAR_MOUSE Isoform 2 of Coxsackievirus and adenovirus receptor homolog OS=Mus musculus GN=Cxadr;sp|P97792|CXAR_MOUSE Coxsackievirus and adenovirus receptor homolog OS=Mus musculus GN=Cxadr PE=1 SV=1;sp|P97792-3|CXAR_MOUSE Isoform 3 of Coxsac	−1,98	0,00501632932935968	0,0767764173652645	23,6075161552656	23,2674949779094	23,732793378787	22,6095143587135	23,2491539067247	22,6991159284757	16654309310,2975	8409221441,37984	0,98585138218992	0,0102297452606089	0,0151553969087917	0,504927660745459	-0.98585138218992	1,98048171598211	0.98585138218992	214
P47753	sp|P47753|CAZA1_MOUSE F-actin-capping protein subunit alpha-1 OS=Mus musculus GN=Capza1 PE=1 SV=4	−1,97	2,45×10^−06	0,00015861440451278	28,9032203325384	28,5146694615578	28,4918017637118	27,8519734871773	28,0364216970831	27,9817500837541	2733390381525,57	1384991459798,25	0,980814439316192	0,00807408910804767	0,0033887499616538	0,506693617259769	-0.980814439316185	1,97357923197861	0.980814439316185	215
P97929	sp|P97929|BRCA2_MOUSE Breast cancer type 2 susceptibility protein homolog OS=Mus musculus GN=Brca2 PE=1 SV=2	−1,97	0,00179318289851816	0,035733820916081	24,0856454874652	23,677772468097	23,8225765445107	23,5193424313764	23,1361785433251	22,8966186104492	23074597329,895	11713952953,9712	0,978077466548642	0,00866542643150518	0,0135485171121933	0,507655790759773	-0.97807746654864	1,96983865485582	0.97807746654864	216
Q8CFI0-3	sp|Q8CFI0-3|NED4L_MOUSE Isoform 3 of E3 ubiquitin-protein ligase NEDD4-like OS=Mus musculus GN=Nedd4l;sp|Q8CFI0|NED4L_MOUSE E3 ubiquitin-protein ligase NEDD4-like OS=Mus musculus GN=Nedd4l PE=1 SV=2;sp|Q8CFI0-2|NED4L_MOUSE Isoform 2 of E3 ubiquitin-prot	−1,95	0,286128170851443	0,704836107321858	24,5231160339711	23,7695776688911	25,914114289794	24,0804450555623	24,0742215615509	24,0418006571554	55275889963,8929	28281934798,4948	0,966769528304905	0,0439829206762327	0,0008622074323535	0,511650464912804	-0.966769528304908	1,95445928143625	0.966769528304908	217
Q9D1I5	sp|Q9D1I5|MCEE_MOUSE Methylmalonyl-CoA epimerase, mitochondrial OS=Mus musculus GN=Mcee PE=1 SV=1	−1,95	0,0236626538800755	0,21524005202738	25,7127945872499	25,6404580141254	25,4301759017466	25,347826740016	25,0419212187464	24,3880879517679	130478743803,081	66865254437,7856	0,964486162458521	0,00573612332049449	0,0196689723103726	0,512460899674961	-0.964486162458523	1,95136838856247	0.964486162458523	218
P63276	sp|P63276|RS17_MOUSE 40S ribosomal protein S17 OS=Mus musculus GN=Rps17 PE=1 SV=2	−1,95	3,64×10^−05	0,0016038564901426	27,8832358468707	27,8785137992401	27,6401569056551	26,9494249747504	27,0475341194994	27,4089401757686	1184847887450,28	609131346240,087	0,959876602318419	0,00499983562929276	0,0089183990043685	0,514100883912534	-0.959876602318414	1,94514351422538	0.959876602318414	219
Q6P5F6	sp|Q6P5F6|S39AA_MOUSE Zinc transporter ZIP10 OS=Mus musculus GN=Slc39a10 PE=1 SV=1	−1,94	0,64969549300201	0,89012008534142	23,5843529479607	24,524511933321	26,7143626330946	26,3667324253046	23,9801195100244	22,484003771526	67884654067,5293	34941878446,9984	0,958128308772694	0,0643936200571838	0,0806653932115023	0,514724261719584	-0.958128308772695	1,94278776885164	0.958128308772695	220
Q00898	sp|Q00898|A1AT5_MOUSE Alpha-1-antitrypsin 1-5 OS=Mus musculus GN=Serpina1e PE=1 SV=1	−1,94	0,0133401912335354	0,14987902972529	28,3357637084218	29,0597621446406	29,0311658380649	28,3845625088349	28,0944458379701	27,9562316171464	3247469619404,85	1672064134790,68	0,957685843655479	0,014231542381652	0,0077672032474181	0,514882148488617	-0.957685843655477	1,94219201993193	0.957685843655477	221
Q8K009	sp|Q8K009|AL1L2_MOUSE Mitochondrial 10-formyltetrahydrofolate dehydrogenase OS=Mus musculus GN=Aldh1l2 PE=1 SV=2	−1,93	0,00301139852158301	0,0531857505426664	24,7655337624833	24,9705054088962	24,3741949927678	23,7858998078014	24,0899649888494	24,2549465990353	53524824257,7457	27669700275,114	0,951901137293886	0,012264304675803	0,0098959947024075	0,516950791690079	-0.951901137293887	1,934420095829	0.951901137293887	222
Q62318	sp|Q62318|TIF1B_MOUSE Transcription intermediary factor 1-beta OS=Mus musculus GN=Trim28 PE=1 SV=3;sp|Q62318-2|TIF1B_MOUSE Isoform 2 of Transcription intermediary factor 1-beta OS=Mus musculus GN=Trim28	−1,93	0,243664093640821	0,667588863741216	29,9047847149208	28,2102303198389	29,3677682146643	28,759343091525	28,7707322599102	27,9775310134962	4617745661174,09	2390528184076,42	0,949859298711998	0,029697467565959	0,0159530665020895	0,517682947368871	-0.949859298711998	1,93168425786963	0.949859298711998	223
Q8BU31	sp|Q8BU31|RAP2C_MOUSE Ras-related protein Rap-2c OS=Mus musculus GN=Rap2c PE=1 SV=1	−1,93	0,00231234360513864	0,0431477969467192	25,2820160355893	24,9321216980073	24,6838243385297	24,2549465990353	24,1179472560925	24,5525942656835	69597003999,4546	36061658277,0193	0,94855946261495	0,0120375910449169	0,0091415148353511	0,518149578354004	-0.94855946261495	1,92994463717732	0.94855946261495	224
Q9WUD8	sp|Q9WUD8|FAIM1_MOUSE Fas apoptotic inhibitory molecule 1 OS=Mus musculus GN=Faim PE=1 SV=1	−1,93	0,0898798284297165	0,441176105282424	24,8371424988238	24,391301183712	24,275977743973	24,3163006006741	24,0516859730864	23,1698545542957	43737592469,7477	22707180719,9159	0,94572521469583	0,0120956489342045	0,0251730737016754	0,519168510146551	-0.945725214695832	1,92615688443377	0.945725214695832	225
Q920I9-2	sp|Q920I9-2|WDR7_MOUSE Isoform 2 of WD repeat-containing protein 7 OS=Mus musculus GN=Wdr7;sp|Q920I9|WDR7_MOUSE WD repeat-containing protein 7 OS=Mus musculus GN=Wdr7 PE=1 SV=3	−1,92	0,160810610937168	0,567767985643373	22,490566066182	21,7524718215974	21,6835797836826	20,9532739285652	22,0969453884394	20,9183799039599	3498286883,71543	1821391136,88912	0,941607836167278	0,0203569276383715	0,0314497613982593	0,520652307095716	-0.941607836167277	1,92066756714892	0.941607836167277	226

O88522	sp|O88522|NEMO_MOUSE NF-kappa-B essential modulator OS=Mus musculus GN=Ikbkg PE=1 SV=2	−1,92	0,00903413520570614	0,113589860661219	25,3393502864799	25,1249989581468	25,1379645009733	25,0006538060705	24,1834776243377	24,4630513056042	88014837796,339	45869255373,0433	0,940219270606788	0,00476921636006717	0,0169180453061565	0,521153665921444	-0.94021927060679	1,91881985178386	0.94021927060679	227
P62082	sp|P62082|RS7_MOUSE 40S ribosomal protein S7 OS=Mus musculus GN=Rps7 PE=2 SV=1	−1,90	0,00175656154229506	0,0352789063737934	29,4384942443743	29,2784329903639	28,8067679360488	28,6488796693929	28,3824394113629	28,5655362258062	4681150630836,53	2462713981326	0,926614105568176	0,0112571308949108	0,0047769341714288	0,526091590623716	-0.926614105568179	1,9008097027638	0.926614105568178	228
Q6PGF3	sp|Q6PGF3|MED16_MOUSE Mediator of RNA polymerase II transcription subunit 16 OS=Mus musculus GN=Med16 PE=2 SV=2	−1,89	0,563420646754282	0,845110794140252	22,9259100978125	22,5552010672857	24,7499941271413	22,5318902919992	24,3850652312053	21,4030055681932	14689054047,5523	7768324677,05509	0,919066086992695	0,0501856013741386	0,0661132512550721	0,528851255629328	-0.919066086992699	1,89089084946959	0.919066086992699	229
Q9JJI8	sp|Q9JJI8|RL38_MOUSE 60S ribosomal protein L38 OS=Mus musculus GN=Rpl38 PE=2 SV=3	−1,88	0,00179495729651167	0,035733820916081	27,3001174003902	27,3878476077223	27,0927002045124	26,2691378786882	26,7598691530864	26,8583139812733	690182393175,167	367175952366,159	0,910506095808495	0,00555990809105616	0,0118518604125877	0,531998434032742	-0.910506095808497	1,87970478112057	0.910506095808496	230
Q7TNG5	sp|Q7TNG5|EMAL2_MOUSE Echinoderm microtubule-associated protein-like 2 OS=Mus musculus GN=Eml2 PE=2 SV=1;sp|Q7TNG5-2|EMAL2_MOUSE Isoform 2 of Echinoderm microtubule-associated protein-like 2 OS=Mus musculus GN=Eml2	−1,88	0,00195525395055923	0,0377655220493122	27,9786139581145	27,9221362330221	27,918409250953	27,6095679757196	27,3974954083987	26,9257179606655	1361652040344,8	726080781545,709	0,907156108741539	0,00120741458517497	0,012817123111796	0,533235187869178	-0.907156108741538	1,8753451061548	0.907156108741538	231
P27546-4	sp|P27546-4|MAP4_MOUSE Isoform 4 of Microtubule-associated protein 4 OS=Mus musculus GN=Map4	−1,87	3,75×10^−09	4,06×10^−07	27,615484436771	27,5162976633754	27,5915719767911	27,1149582581361	26,9656720986252	26,7629318109403	945000561839,519	505015044011,848	0,903988821785932	0,00187718505511384	0,0065566754296312	0,534407136254814	-0.903988821785932	1,87123249702112	0.903988821785932	232
O23657	sp|O23657|RABC1_ARATH Ras-related protein RABC1 OS=Arabidopsis thaliana GN=RABC1 PE=1 SV=1	−1,87	0,020720990261413	0,199524718486067	29,8158570876731	30,1644509291729	29,9092926391212	28,8465312886039	29,4999946244056	29,6647193245074	10300362643547,4	5507223696846,06	0,903298015741731	0,00602208542656909	0,0147505938941207	0,534663087837594	-0.90329801574173	1,87033670875696	0.90329801574173	233
Q8R409	sp|Q8R409|HEXI1_MOUSE Protein HEXIM1 OS=Mus musculus GN=Hexim1 PE=1 SV=1;sp|Q5M9G1|HEXI1_RAT Protein HEXIM1 OS=Rattus norvegicus GN=Hexim1 PE=2 SV=1	−1,86	0,119902495383724	0,494761296860658	23,675109750947	24,3651120990611	24,155861296033	22,7554239515794	23,8383590411367	23,732793378787	28278313733,8295	15164031681,1756	0,899042717511399	0,0147012815920567	0,0254710069872051	0,536242430291548	-0.899042717511401	1,86482818872858	0.899042717511401	234
P62267	sp|P62267|RS23_MOUSE 40S ribosomal protein S23 OS=Mus musculus GN=Rps23 PE=1 SV=3	−1,86	5,86×10^−07	4,58×10^−05	26,9791446367106	26,7982568071736	26,8660517354712	26,1166289768567	26,1771723436785	26,4815901695906	472427937860,841	253457965596,679	0,898347778375426	0,00339956870329634	0,0074486410009833	0,536500797866315	-0.898347778375426	1,86393012643605	0.898347778375426	235
P60867	sp|P60867|RS20_MOUSE 40S ribosomal protein S20 OS=Mus musculus GN=Rps20 PE=1 SV=1	−1,86	3,92×10^−05	0,00171062013013349	28,0535845608097	27,7421650657753	27,6959168630177	27,0158477199576	27,2551673122194	27,3567816774972	1220834181728,11	655894355772,144	0,896331896121905	0,00698973251185368	0,0064331254581728	0,537250976085643	-0.896331896121904	1,86132746986502	0.896331896121903	236
Q9CQ25	sp|Q9CQ25|MZT2_MOUSE Mitotic-spindle organizing protein 2 OS=Mus musculus GN=Mzt2 PE=1 SV=1	−1,86	0,152072791395182	0,553091666781035	23,7476238449328	24,4818124226021	24,3111380538731	22,8505765759992	23,7144489157279	24,1126737547988	31719308725,8416	17046880842,9112	0,895853544109332	0,0158895144950727	0,0273865617722689	0,537429141039987	-0.895853544109336	1,86071041489281	0.895853544109336	237
Q570Y9-3	sp|Q570Y9-3|DPTOR_MOUSE Isoform 3 of DEP domain-containing mTOR-interacting protein OS=Mus musculus GN=Deptor;sp|Q570Y9|DPTOR_MOUSE DEP domain-containing mTOR-interacting protein OS=Mus musculus GN=Deptor PE=1 SV=2;sp|Q570Y9-2|DPTOR_MOUSE Isoform 2 of D	−1,86	0,1088984789452	0,473794072647748	22,993646883691	24,0114271695296	24,1861387683333	23,1854445652742	22,8991140368772	23,245900105076	20229429233,2731	10879573773,8492	0,894833577684134	0,027138299319973	0,0080158648090816	0,537809230719897	-0.894833577684137	1,85939538200456	0.894833577684137	238
Q52KR3-3	sp|Q52KR3-3|PRUN2_MOUSE Isoform 3 of Protein prune homolog 2 OS=Mus musculus GN=Prune2;sp|Q52KR3-2|PRUN2_MOUSE Isoform 2 of Protein prune homolog 2 OS=Mus musculus GN=Prune2;sp|Q52KR3|PRUN2_MOUSE Protein prune homolog 2 OS=Mus musculus GN=Prune2 PE=2 SV	−1,86	0,112219773008506	0,480080631183422	26,3883602649067	25,8273556009734	27,1190534987677	25,7970224427502	26,0271456176258	25,6555739842145	305410656147,225	164566516579,807	0,892079571786725	0,0244925624520603	0,0072615507754412	0,538836852177406	-0.892079571786726	1,85584930941353	0.892079571786726	239
P14115	sp|P14115|RL27A_MOUSE 60S ribosomal protein L27a OS=Mus musculus GN=Rpl27a PE=2 SV=5;sp|P46776|RL27A_HUMAN 60S ribosomal protein L27a OS=Homo sapiens GN=RPL27A PE=1 SV=2	−1,86	0,159929298453155	0,566681565713405	25,8459890590547	25,0243761876188	25,3917468837871	24,0965663332202	24,961398664519	25,3493149492024	109665870976,825	59095856503,7209	0,891985730948061	0,0161903916618717	0,0258573833235564	0,538871902236652	-0.891985730948064	1,85572859866952	0.891985730948064	240
O35085	sp|O35085|ARX_MOUSE Homeobox protein ARX OS=Mus musculus GN=Arx PE=2 SV=3	−1,86	0,083682680261373	0,430545618027756	23,4971371284695	24,0050047487481	23,02646262069	22,4569741763383	23,1523256748072	23,0651904909605	16220388425,0264	8742803840,378	0,891640432605673	0,0208166115783791	0,0165485583841128	0,539000892659805	-0.891640432605671	1,85528449696123	0.891640432605671	241
Q9JL35	sp|Q9JL35|HMGN5_MOUSE High mobility group nucleosome-binding domain-containing protein 5 OS=Mus musculus GN=Hmgn5 PE=1 SV=2	−1,85	0,00128158659377593	0,0287976314314303	26,7527581374521	26,6710608514719	26,4430714013401	26,3063436289981	25,7260541994384	25,9818743454278	364683903224,328	196662990236,01	0,890921037820134	0,00602874230636799	0,0111833653308625	0,539269730572771	-0.890921037820134	1,85435959651931	0.890921037820134	242
Q3UQA7	sp|Q3UQA7|SELH_MOUSE Selenoprotein H OS=Mus musculus GN=Selh PE=2 SV=2	−1,85	0,0348926364204042	0,273411790496096	23,7695776688911	22,9959305482374	23,1651192385014	22,5636759051768	22,4762920876387	23,0384874615479	13289088898,1369	7167469388,14581	0,890706453700175	0,0174481288860396	0,0133315204528285	0,53934994664312	-0.890706453700172	1,85408380259225	0.890706453700172	243

P62867	sp|P62867|RS30_MUSSI 40S ribosomal protein S30 OS=Mus spicilegus GN=Fau PE=3 SV=1;sp|P62862|RS30_MOUSE 40S ribosomal protein S30 OS=Mus musculus GN=Fau PE=1 SV=1	−1,85	0,098862565245129 5	0,462035382641852	25,9318139841019	25,8693686586924	25,2553298211587	24,5231160339711	25,102146468860 3	25,5795222243761	142913323583,744	77091708974,5969	0,890492807626411	0,0145548240624672	0,0211030838742253	0,539429823905977	-0.890492807626413	1,85380925503722	0.890492807626413	244
Q80WB5	sp|Q80WB5|NTAQ1_MOUSE Protein N-terminal glutamine amidohydrolase OS=Mus musculus GN=Wdyhv1 PE=1 SV=1	−1,85	0,0356712271193817	0,275959769531894	23,5542435875806	23,8261006818307	24,2505125478808	23,6248549139189	22,8906559375229	23,2674949779094	23422250238,8076	12650998010,4805	0,888628485534673	0,0146965516390883	0,0157836086942485	0,54012735247442	-0.888628485534675	1,85141521794596	0.888628485534675	245
Q922F4	sp|Q922F4|TBB6_MOUSE Tubulin beta-6 chain OS=Mus musculus GN=Tubb6 PE=1 SV=1	−1,85	0,409575510756809	0,763162250954497	28,5402325045527	28,197075208073	30,5244583269409	28,3342637623689	28,7942855809832	28,2953913503404	4289774770847,12	2324780862639,14	0,883807170837579	0,0431950312385556	0,0097453964966053	0,541935413121947	-0.883807170837579	1,84523833613172	0.883807170837579	246
P51807	sp|P51807|DYLT1_MOUSE Dynein light chain Tctex-type 1 OS=Mus musculus GN=Dynlt1 PE=1 SV=1	−1,84	0,300394089318058	0,713946294657213	22,5636759051768	23,7779715892546	24,2549465990353	22,7738720080143	22,4725237041215	23,5141970803436	16592191517,9485	8997365034,69448	0,882929990666673	0,0370560657432921	0,0233868405199639	0,542265018153969	-0.882929990666676	1,8441167446211	0.882929990666676	247
Q9DCV5	sp|Q9DCV5|TM254_MOUSE Transmembrane protein 254 OS=Mus musculus GN=Tmem254 PE=2 SV=1	−1,84	0,000506302793678805	0,0139279295788369	23,9823398794781	23,7144489157279	23,6328120342077	23,0651904909605	23,4427204772675	22,9874166300028	21184457433,2449	11494209220,0406	0,882098964754319	0,00769026229042029	0,0105133557630104	0,542577465401718	-0.882098964754319	1,84305479634989	0.882098964754319	248
Q3TY86-3	sp|Q3TY86-3|AIFM3_MOUSE Isoform 3 of Apoptosis-inducing factor 3 OS=Mus musculus GN=Aifm3;sp|Q3TY86-2|AIFM3_MOUSE Isoform 2 of Apoptosis-inducing factor 3 OS=Mus musculus GN=Aifm3;sp|Q3TY86|AIFM3_MOUSE Apoptosis-inducing factor 3 OS=Mus musculus GN=Aifm	−1,84	0,266318653490452	0,685404159524725	24,4132109090808	25,0419212187464	24,9376594065974	25,1686290907268	23,4404474775681	23,9566725543437	58811142849,5576	31986604440,8176	0,878621675659098	0,013587826456467 7	0,0366753373475535	0,543886802585035	-0.878621675659098	1,83861788013077	0.878621675659098	249
P63325	sp|P63325|RS10_MOUSE 40S ribosomal protein S10 OS=Mus musculus GN=Rps10 PE=1 SV=1	−1,83	2,79×10^−53	3,17×10^−50	28,3695269666755	28,4487032309194	28,4457488486355	27,7887330476403	27,7863646768548	27,8762541482515	2204069077801,89	1204538101243,26	0,871689411388512	0,00157923296785055	0,0018415894402804	0,546506510787104	-0.87168941138851	1,82980436694113	0.87168941138851	250
Q8JZQ9	sp|Q8JZQ9|EIF3B_MOUSE Eukaryotic translation initiation factor 3 subunit B OS=Mus musculus GN=Eif3b PE=1 SV=1	−1,83	0,257970754423133	0,67968190098123	30,1787354002317	28,5498933163116	29,5120517470896	29,004558008889 2	29,1075726323578	28,3160857868948	5944931683264,55	3249120551286,22	0,871610958597524	0,02784006738054 6	0,0149368264371135	0,546536230253537	-0.871610958597522	1,82970486610943	0.871610958597522	251
Q6NT99	sp|Q6NT99|DUS23_MOUSE Dual specificity protein phosphatase 23 OS=Mus musculus GN=Dusp23 PE=2 SV=1	−1,83	0,191978340647199	0,618802084535046	23,9449572067503	23,779469321038	23,4522005639565	22,2553767996608	23,4260786804776	23,6838064405286	20131314276,5899	11006523435,4833	0,871082516517602	0,0105694341382322	0,0329252842750947	0,546736456659586	-0.871082516517609	1,82903478964936	0.871082516517609	252
Q8BUM6	sp|Q8BUM6|F163B_MOUSE Protein FAM163B OS=Mus musculus GN=Fam163b PE=2 SV=1	−1,82	0,393528356732543	0,753881747531964	23,1763774222025	23,1467880451938	22,7026757793855	21,0988721291394	22,6960719862823	23,4277205768759	9829105292,7079	5388618588,34888	0,867144624333874	0,01153319358115 4	0,0531486127584899	0,548230833618868	-0.867144624333874	1,82404917541578	0.867144624333874	253
Q80TZ3-2	sp|Q80TZ3-2|AUXI_MOUSE Isoform 2 of Putative tyrosine-protein phosphatase auxilin OS=Mus musculus GN=Dnajc6;sp|Q80TZ3|AUXI_MOUSE Putative tyrosine-protein phosphatase auxilin OS=Mus musculus GN=Dnajc6 PE=2 SV=2;sp|Q80TZ3-3|AUXI_MOUSE Isoform 3 of Putati	−1,82	0,294710676524909	0,711969538636193	30,5191558658654	28,8632336136022	30,1229682803372	29,4161611861033	29,6053749310012	28,6821011242862	9062075134212,53	4970520839022,13	0,866444418994669	0,0289817762280038	0,0166821593928951	0,548496979489462	-0.866444418994671	1,82316409641999	0.866444418994671	254
Q62188	sp|Q62188|DPYL3_MOUSE Dihydropyrimidinase-related protein 3 OS=Mus musculus GN=Dpysl3 PE=1 SV=1	−1,82	0,0180092543993775	0,181507055244037	32,390014054121	33,1187251555991	33,0839850192138	32,4429895051478	32,1886810722787	32,1601216416315	187395300799982	102812775825514	0,866065226542048	0,0125078226304287	0,0048266137650687	0,548641163287505	-0.866065226542051	1,82268496590361	0.866065226542051	255
O35633-2	sp|O35633-2|VIAAT_MOUSE Isoform 2 of Vesicular inhibitory amino acid transporter OS=Mus musculus GN=Slc32a1;sp|O35633|VIAAT_MOUSE Vesicular inhibitory amino acid transporter OS=Mus musculus GN=Slc32a1 PE=1 SV=3;sp|Q9H598|VIAAT_HUMAN Vesicular inhibitory	−1,82	0,000396498888007967	0,0117628003442364	29,3328195161808	29,0435033047516	29,5745822065934	28,801510817816	28,7755965557594	28,5775854342843	5397561225124,8	2965986879361,41	0,863795487232134	0,00906962974203946	0,0042652284739128	0,549504999694159	-0.863795487232136	1,81981965688497	0.863795487232136	256
Q8BHC4	sp|Q8BHC4|DCAKD_MOUSE Dephospho-CoA kinase domain-containing protein OS=Mus musculus GN=Dcakd PE=2 SV=1	−1,82	0,0354023358842	0,275156168798602	27,2571389591788	27,7219629002009	27,7031011328236	26,50132379886 7	27,0796340180657	27,3081622510207	932126461502,334	512742713234,5	0,862290614239247	0,00954584111416491	0,0154234756022666	0,550078486569406	-0.862290614239247	1,8179223954686	0.862290614239247	257
Q9JMG1	sp|Q9JMG1|EDF1_MOUSE Endothelial differentiation-related factor 1 OS=Mus musculus GN=Edf1 PE=1 SV=1	−1,82	0,116167139203718	0,487484510268456	26,147377349823	25,8617916340518	25,3674906455573	24,5876185868543	25,4960085354994	25,502807308767	159007924329,87	87550196815,1301	0,860916338559426	0,0152980488652922	0,0208939394155321	0,550602727405603	-0.860916338559425	1,81619151200344	0.860916338559424	258
P07309	sp|P07309|TTHY_MOUSE Transthyretin OS=Mus musculus GN=Ttr PE=1 SV=1	−1,81	1,22×10^−07	1,06×10^−05	28,0023313775896	27,8539827882304	27,6999254581679	27,1537780055346	27,3929021595667	27,2232737570987	1247396723863,18	687722539137,054	0,859021841193437	0,0054291076573868	0,0045129039685549	0,551326234854282	-0.859021841193441	1,81380811719272	0.859021841193441	259
Q7TNC4-2	sp|Q7TNC4-2|LC7L2_MOUSE Isoform 2 of Putative RNA-binding protein Luc7-like 2 OS=Mus musculus GN=Luc7l2;sp|Q7TNC4-3|LC7L2_MOUSE Isoform 3 of Putative RNA-binding protein Luc7-like 2 OS=Mus musculus GN=Luc7l2;sp|Q7TNC4-4|LC7L2_MOUSE Isoform 4 of Putative	−1,81	1,60×10^−06	0,000106806438256 97	26,4067100219599	26,2339091105729	26,0446839509163	25,6128898625493	25,7580916626282	25,530459038085	245959902382,927	135713722311,418	0,857856537166938	0,0069037715488766 5	0,0044960044605070	0,551771735947958	-0.85785653716694	1,81234364656605	0.85785653716694	260

Q8K1C0-3	sp|Q8K1C0-3|ANGE2_MOUSE Isoform 3 of Protein angel homolog 2 OS=Mus musculus GN=Angel2;sp|Q8K1C0-2|ANGE2_MOUSE Isoform 2 of Protein angel homolog 2 OS=Mus musculus GN=Angel2;sp|Q8K1C0|ANGE2_MOUSE Protein angel homolog 2 OS=Mus musculus GN=Angel2 PE=2 SV	−1,81	0,00330330323583234	0,0559466171173246	25,2582028351988	24,8903374302592	25,1895497765879	24,8413906379364	24,4368206009609	24,2783394430416	80594535039,6517	44504236654,813	0,856739333324961	0,0077890595062904	0,0118419222166562	0,552199186122451	-0.856739333324962	1,8109407350308	0.856739333324962	261
B1AZP2-3	sp|B1AZP2-3|DLGP4_MOUSE Isoform 3 of Disks large-associated protein 4 OS=Mus musculus GN=Dlgap4;sp|B1AZP2-2|DLGP4_MOUSE Isoform 2 of Disks large-associated protein 4 OS=Mus musculus GN=Dlgap4;sp|B1AZP2|DLGP4_MOUSE Disks large-associated protein 4 OS=Mus	−1,81	0,107986505876472	0,472983636494304	25,9147147066523	25,0758097375073	25,0023493395826	24,4430889369582	25,1775723938195	24,595883751326	100252508765,683	55455590193,9649	0,854233536280851	0,0200103267647111	0,0156656779464002	0,553159126656665	-0.854233536280854	1,80779806715669	0.854233536280854	262
Q08331	sp|Q08331|CALB2_MOUSE Calretinin OS=Mus musculus GN=Calb2 PE=1 SV=3	−1,81	0,00325211718111269	0,0556565269932807	32,839500065196	33,3243876702912	33,5034225927072	32,6101813602848	32,5868757685581	32,6953467176655	268114910767801	148380536465822	0,853549593056719	0,010339464674082	0,0017498703715314	0,553421426808768	-0.85354959305672	1,80694124144482	0.85354959305672	263
Q8BLE7	sp|Q8BLE7|VGLU2_MOUSE Vesicular glutamate transporter 2 OS=Mus musculus GN=Slc17a6 PE=1 SV=1	−1,80	0,0105425728896745	0,12788285364156	27,5261939943834	27,9817500837541	28,2148956435959	27,4336352949716	27,4236219219812	27,0965384478712	1318628425303,56	731184667765,639	0,850730362672466	0,0125515986630387	0,0070209451727104	0,554503947992259	-0.850730362672467	1,80341367021964	0.850730362672467	264
Q8K4M5	sp|Q8K4M5|COMD1_MOUSE COMM domain-containing protein 1 OS=Mus musculus GN=Commd1 PE=2 SV=2	−1,80	0,130589332206956	0,514825425964414	26,3577801409492	26,0494943656724	25,7318503388321	25,0736508555744	25,1555905514942	26,1456344965372	205020323149,272	113866434114,268	0,848424399765802	0,0120161274277498	0,0234369354834626	0,555390960101861	-0.848424399765809	1,80053344731538	0.848424399765809	265
Q8K2Y9-2	sp|Q8K2Y9-2|CCM2_MOUSE Isoform 2 of Malcavernin OS=Mus musculus GN=Ccm2;sp|Q8K2Y9|CCM2_MOUSE Malcavernin OS=Mus musculus GN=Ccm2 PE=1 SV=1	−1,79	6,18×10^−07	4,68×10^−05	24,3413012075385	24,0503294468235	24,3792155998351	23,7113493878119	23,7427329093668	23,5687032910947	34249874978,9737	19124993231,6412	0,840639484633687	0,00741803754082471	0,003917899757739	0,55839600125204	-0.840639484633687	1,79084376993709	0.840639484633688	266
Q8VEE0	sp|Q8VEE0|RPE_MOUSE Ribulose-phosphate 3-epimerase OS=Mus musculus GN=Rpe PE=2 SV=1	−1,79	0,00660513004493847	0,0910375021402342	25,2712628519499	24,8053173699664	25,2798116120726	24,3404652835671	24,4478937746508	24,8222977128199	81087712653,1168	45314168472,5203	0,839521106008524	0,010809244094451	0,0103086935642295	0,558829038209139	-0.839521106008525	1,78945604402496	0.839521106008525	267
Q8K124	sp|Q8K124|PKHO2_MOUSE Pleckstrin homology domain-containing family O member 2 OS=Mus musculus GN=Plekho2 PE=1 SV=1	−1,78	0,653694029529806	0,891901943526937	24,882110644788	24,7637322819112	22,7429179730565	21,5346685350988	25,266100588418	23,8514133380406	30154106904,6252	16902501863,6141	0,835117700301073	0,0498287990745856	0,0799890684582793	0,56053730647952	-0.83511770030108	1,78400257831284	0.83511770030108	268
P62852	sp|P62852|RS25_MOUSE 40S ribosomal protein S25 OS=Mus musculus GN=Rps25 PE=1 SV=1	−1,78	0,0515518100871682	0,334319560904408	28,9293487073306	28,8657160830252	28,4613395369887	27,7031011328236	28,2400659404567	28,586024909645	3068269309654,82	1725257322312,33	0,830613561285276	0,00882848890277368	0,0157895265444291	0,562290056118451	-0.830613561285275	1,77844155186223	0.830613561285275	269
Q8K4I3	sp|Q8K4I3|ARHG6_MOUSE Rho guanine nucleotide exchange factor 6 OS=Mus musculus GN=Arhgef6 PE=1 SV=1	−1,77	0,0970689207442946	0,457999824592883	23,5596170779061	23,9261106778015	24,4408004316673	23,6996447819213	23,5630146461116	22,9428230979447	25848267019,5695	14564130223,1265	0,827648013613769	0,0184631637451219	0,0172352778649577	0,563447066377801	-0.827648013613775	1,77478961143349	0.827648013613775	270
P08228	sp|P08228|SODC_MOUSE Superoxide dismutase [Cu-Zn] OS=Mus musculus GN=Sod1 PE=1 SV=2;sp|P07632|SODC_RAT Superoxide dismutase [Cu-Zn] OS=Rattus norvegicus GN=Sod1 PE=1 SV=2	−1,77	0,194576355597616	0,621976904521672	32,504668068945	32,3276858928127	32,0149961289679	31,9453672776117	30,9004869372115	32,2862250679884	104734535381447	59126072036422,7	0,824870896119741	0,00768065393868575	0,0227702562486563	0,564532719041369	-0.824870896119747	1,77137651418698	0.824870896119747	271
Q91VK1	sp|Q91VK1|BZW2_MOUSE Basic leucine zipper and W2 domain-containing protein 2 OS=Mus musculus GN=Bzw2 PE=1 SV=1;sp|Q2L4X1|BZW2_MUSMM Basic leucine zipper and W2 domain-containing protein 2 OS=Mus musculus molossinus GN=Bzw2 PE=2 SV=1	−1,76	0,166570016305257	0,575385119523278	26,3947233703869	26,0654090296045	27,3974954083987	26,1820526790869	26,0926202587236	25,879283072998	363559668166,721	206036396108,762	0,819292951224438	0,0260652526118906	0,0059710207232124	0,566719617573966	-0.81929295122444	1,76454099874085	0.81929295122444	272
Q8CFV9	sp|Q8CFV9|RIFK_MOUSE Riboflavin kinase OS=Mus musculus GN=Rfk PE=1 SV=2	−1,76	0,34524626932575	0,730303241451822	26,0981905549866	26,7237904447108	26,8528957989924	26,5887417463319	26,5000720757695	24,8846009566493	342073774695,587	194002767153,062	0,818230272809003	0,0151988254501098	0,0369092889490623	0,567137212800677	-0.818230272809007	1,76324172956616	0.818230272809007	273
P24452	sp|P24452|CAPG_MOUSE Macrophage-capping protein OS=Mus musculus GN=Capg PE=1 SV=2	−1,76	0,676744090491436	0,902391723484321	25,7926120679867	22,1514329050445	25,5244369585765	22,5716530900109	24,6548084016843	24,5493168001552	43216749510,4856	24581486877,6787	0,814018382257523	0,0828622755147368	0,0490462196007176	0,568795366521366	-0.814018382257522	1,75810152272476	0.814018382257522	274
Q9QXV0	sp|Q9QXV0|PCSK1_MOUSE ProSAAS OS=Mus musculus GN=Pcsk1n PE=1 SV=2	−1,75	0,0255072869860689	0,221372037533015	24,4611241663019	24,3832569119608	24,4199170205648	23,6663250302842	23,5542435875806	24,3563980610013	40373226814,1571	23005263924,1106	0,811434890638214	0,00159514588198049	0,0182069315996415	0,569814843634041	-0.811434890638217	1,75495603733735	0.811434890638217	275
P83882	sp|P83882|RL36A_MOUSE 60S ribosomal protein L36a OS=Mus musculus GN=Rpl36a PE=1 SV=2;sp|P61486|RL36A_TAKRU 60S ribosomal protein L36a OS=Takifugu rubripes GN=rpl36a PE=3 SV=2	−1,75	0,0236368595483037	0,21524005202738	24,524511933321	24,4150238637808	23,9989811215525	23,3777626719801	23,9668942617015	23,9154891156968	36218612202,3456	20699637323,4807	0,807125776625645	0,0114041379531376	0,0137374009228086	0,57151933949971	-0.807125776625645	1,74972206693017	0.807125776625645	276
Q91V64	sp|Q91V64|ISOC1_MOUSE Isochorismatase domain-containing protein 1 OS=Mus musculus GN=Isoc1 PE=1 SV=1	−1,75	0,00157653977781739	0,0327329861064157	28,7457012936542	29,2687031531706	29,1996198193394	28,4931602800957	28,6351446174494	28,4137586698619	4222047948259,89	2418143442467,64	0,804043136219411	0,0097732032058853	0,0039334177475059	0,572741823897162	-0.804043136219413	1,74598738607145	0.804043136219413	277
Q8K0X8	sp|Q8K0X8|FEZ1_MOUSE Fasciculation and elongation protein zeta-1 OS=Mus musculus GN=Fez1 PE=1 SV=2	−1,74	0,54661088583092	0,836220697939517	21,3878092046391	23,6385289981359	23,4411557402849	23,4209979293226	21,6767034556501	21,7012179112435	8159901877,38607	4678794985,87877	0,802414789451559	0,0546123379628157	0,0449139281715729	0,573388633366455	-0.802414789451558	1,74401782980741	0.802414789451558	278

Q9UBL3-3	sp|Q9UBL3-3|ASH2L_HUMAN Isoform 3 of Set1/Ash2 histone methyltransferase complex subunit ASH2 OS=Homo sapiens GN=ASH2L;sp|Q91X20|ASH2L_MOUSE Set1/Ash2 histone methyltransferase complex subunit ASH2 OS=Mus musculus GN=Ash2l PE=1 SV=1;sp|Q9UBL3|ASH2L_HUMA	−1,74	0,00609407768179416	0,0872587337465732	23,9175054815775	24,1461515325522	24,1659420657239	23,8903181590625	23,3766304858985	23,29481600134	28596009773,1669	16400673129,7901	0,802058822103434	0,00573491713900491	0,0137240633179383	0,573530127450848	-0.802058822103429	1,7435875678313	0.802058822103429	279
Q8CI75	sp|Q8CI75|DI3L2_MOUSE DIS3-like exonuclease 2 OS=Mus musculus GN=Dis3l2 PE=1 SV=1;sp|Q8CI75-2|DI3L2_MOUSE Isoform 2 of DIS3-like exonuclease 2 OS=Mus musculus GN=Dis3l2	−1,74	0,354719921356122	0,732843629012038	27,1802077444987	25,6042732888588	26,5902926789079	26,0926202587236	26,4553495397667	25,1612997878741	309510515446,513	177651546042,808	0,800938181005802	0,0300934626716404	0,0257698861432141	0,573975801069377	-0.8009381810058	1,74223372855945	0.8009381810058	280
Q7TPB0	sp|Q7TPB0|LPPR3_MOUSE Lipid phosphate phosphatase-related protein type 3 OS=Mus musculus GN=Lppr3 PE=2 SV=1	−1,74	0,000818113492878139	0,0204033909020542	25,0023493395826	24,7814467480862	24,5010625962898	24,1996408868827	24,3497113967141	24,0742215615509	56732867946,1456	32609123508,2944	0,79890913281881	0,0101459779284429	0,0056976793937605	0,574783625239059	-0.79890913281881	1,73978512276526	0.79890913281881	281
Q8R2W9	sp|Q8R2W9|PANK3_MOUSE Pantothenate kinase 3 OS=Mus musculus GN=Pank3 PE=2 SV=1	−1,74	0,294880734437919	0,711969538636193	24,6363202435582	24,3017734131296	24,4879210503984	24,6338720357762	22,914286016571	24,2210527725757	42609294010,8641	24527733036,2091	0,796754248174207	0,00684885258374668	0,0375238401997014	0,575642793564129	-0.796754248174206	1,73718842862331	0.796754248174206	282
Q9ET26	sp|Q9ET26|RN114_MOUSE E3 ubiquitin-protein ligase RNF114 OS=Mus musculus GN=Rnf114 PE=2 SV=2	−1,73	0,163263744658512	0,569625464688209	23,1493509179357	23,7216479337666	23,7026990686974	22,9565167887036	22,3810757628557	23,5843529479607	16466040961,5954	9494531451,94674	0,794325008793113	0,0138189101489047	0,0261960982658319	0,576612889163298	-0.794325008793115	1,73426577656123	0.794325008793115	283
Q6IR34	sp|Q6IR34|GPSM1_MOUSE G-protein-signaling modulator 1 OS=Mus musculus GN=Gpsm1 PE=1 SV=3;sp|Q6IR34-2|GPSM1_MOUSE Isoform 2 of G-protein-signaling modulator 1 OS=Mus musculus GN=Gpsm1;sp|Q6IR34-3|GPSM1_MOUSE Isoform 3 of G-protein-signaling modulator 1 O	−1,73	1,29×10^−22	4,90×10^−20	28,2756209716639	28,2826760859385	28,3794989601019	27,802797894039	27,6721550228499	27,8119809890143	1976994093624,62	1140298632049,04	0,793896860629026	0,00205013459846218	0,0028172193333988	0,576784035787592	-0.793896860629021	1,73375117540227	0.79389686062902	284
Q9JI44	sp|Q9JI44|DMAP1_MOUSE DNA methyltransferase 1-associated protein 1 OS=Mus musculus GN=Dmap1 PE=1 SV=1;sp|Q9JI44-2|DMAP1_MOUSE Isoform 2 of DNA methyltransferase 1-associated protein 1 OS=Mus musculus GN=Dmap1	−1,73	0,538894192995984	0,831485169227261	23,9621918362882	23,7897040107311	24,3563980610013	24,0418006571554	24,6483181932764	21,7796036535179	27462793704,303	15905129454,0109	0,787986279598433	0,0120854064521924	0,0643635126635484	0,579151910955032	-0.787986279598428	1,7266626960636	0.787986279598428	285
Q76N33	sp|Q76N33|STALP_MOUSE AMSH-like protease OS=Mus musculus GN=Stambpl1 PE=2 SV=1;sp|Q76N33-2|STALP_MOUSE Isoform 2 of AMSH-like protease OS=Mus musculus GN=Stambpl1	−1,72	0,332413610584384	0,72953198881961	22,9576116421202	22,7554239515794	23,0737103227927	22,9159360119671	21,2812138014139	22,9604916551048	9076144324,37254	5273080108,02734	0,783433636076365	0,00702483818303787	0,0427469792882188	0,580982399527002	-0.783433636076364	1,72122253757452	0.783433636076364	286
P48024	sp|P48024|EIF1_MOUSE Eukaryotic translation initiation factor 1 OS=Mus musculus GN=Eif1 PE=2 SV=2	−1,72	7,02×10^−05	0,00278869643469304	24,8802711146083	24,5806585090775	24,7462638463834	24,3120591899788	24,2840779865289	23,9823398794781	55282992955,9129	32122600518,0986	0,783247031204204	0,00606748785301514	0,0075568750840960	0,581057551347043	-0.783247031204204	1,72099992106073	0.783247031204205	287
Q9D0M5	sp|Q9D0M5|DYL2_MOUSE Dynein light chain 2, cytoplasmic OS=Mus musculus GN=Dynll2 PE=1 SV=1	−1,72	0,000828189862368259	0,0205418239633307	28,4931602800957	28,4428094286615	28,1195541428091	28,0318362877081	27,7259995969464	27,6705231091959	2056118882043,54	1195341456256,69	0,782500890311847	0,00715070881806883	0,0069967885958798	0,581358143585872	-0.782500890311848	1,72011007505959	0.782500890311848	288
P50114	sp|P50114|S100B_MOUSE Protein S100-B OS=Mus musculus GN=S100b PE=2 SV=2	−1,72	0,0806881769918665	0,423283113906046	28,6215713507648	28,7194223068834	28,6821011242862	28,586024909645	28,2685915515089	27,541410950126	2838693216896,35	1650351047204,57	0,782454008945206	0,00172216791466334	0,019036430984078	0,581377035525156	-0.782454008945202	1,72005417980898	0.782454008945202	289
Q9JLY7	sp|Q9JLY7|DUS14_MOUSE Dual specificity protein phosphatase 14 OS=Mus musculus GN=Dusp14 PE=2 SV=2	−1,72	0,188781178766802	0,613397297160539	25,3227647390092	24,6343822529779	24,8569465581742	23,6787354581344	24,723259791271	24,7860991569549	67678283057,5169	39360612432,8365	0,781940300417119	0,0140861736844512	0,0254955668987338	0,581584086573024	-0.78194030041712	1,7194418194839	0.78194030041712	290
P25785	sp|P25785|TIMP2_MOUSE Metalloproteinase inhibitor 2 OS=Mus musculus GN=Timp2 PE=1 SV=2	−1,72	0,233407184704815	0,657279264635888	22,4664435194351	23,800646758244	22,8392616668271	22,1728119761975	22,3959226638979	22,914286016571	10096459433,2084	5877164289,78722	0,780657332895426	0,0298842972869439	0,0169112880459808	0,582101510798584	-0.780657332895425	1,71791342480473	0.780657332895425	291
Q8VCE2	sp|Q8VCE2|GPN1_MOUSE GPN-loop GTPase 1 OS=Mus musculus GN=Gpn1 PE=1 SV=1	−1,71	0,21973458553236	0,648792713611515	22,0092765517313	21,2326685501382	22,7383944477728	21,5621146118181	21,3591905727963	21,4438630713332	3561495993,90152	2078798844,63924	0,776733205199866	0,0342369149122245	0,0047505823742189	0,583686981032365	-0.776733205199866	1,71324705278042	0.776733205199866	292
P62900	sp|P62900|RL31_MOUSE 60S ribosomal protein L31 OS=Mus musculus GN=Rpl31 PE=1 SV=1	−1,71	0,141689251398801	0,532831410189858	26,0846949698555	27,0856899088401	26,6246811115036	26,4316752166706	25,6586432911233	26,0926202587236	356056523854,207	208036543025,524	0,775269316962566	0,0188363702423809	0,0148684376221972	0,584279542960173	-0.775269316962565	1,71150951979875	0.775269316962565	293
P14206	sp|P14206|RSSA_MOUSE 40S ribosomal protein SA OS=Mus musculus GN=Rpsa PE=1 SV=4	−1,71	0,000449813665282539	0,0129221786501104	30,8437717553966	30,3275359257424	30,6358451815377	30,0107694072549	30,0981104206294	30,0920533297203	19518508546379	11426744347828,1	0,772428401028458	0,00848755596498836	0,0016221100665418	0,585431223941952	-0.772428401028452	1,70814257781911	0.772428401028452	294
Q62468	sp|Q62468|VILI_MOUSE Villin-1 OS=Mus musculus GN=Vil1 PE=1 SV=3	−1,71	0,000116044738273461	0,00418607638176181	25,5153070978226	25,2118538008189	25,0513945111618	24,7401928293227	24,7625063508653	24,6713112340392	93340363323,5152	54674876137,5611	0,771623036000318	0,00932720242924503	0,0019226913330656	0,585758124253914	-0.771623036000316	1,7071892963904	0.771623036000316	295

Q80SZ7	sp|Q80SZ7|GBG5_MOUSE Guanine nucleotide-binding protein G(I)/G(S)/G(O) subunit gamma-5 OS=Mus musculus GN=Gng5 PE=3 SV=2	−1,70	0,23877140765664 6	0,663676313137488	26,2691378786882	25,2754018283628	25,1761940416215	24,473229654249	25,2573533322883	25,3937401987218	127780261195,821	75051551053,9027	0,76771120101972	0,0236342183943601	0,019838641875382	0,587348549388919		-0.767711201019724	1,70256656126997	0.767711201019724	296
P97461	sp|P97461|RS5_MOUSE 40S ribosomal protein S5 OS=Mus musculus GN=Rps5 PE=1 SV=3	−1,70	0,0170870997714102	0,178300688102698	29,0458241396507	28,7823143927038	28,62011627812	27,9281151032895	28,3714205600626	28,5524438127231	3270895566291,48	1921242366682,47	0,767646169610389	0,00745603535622 77	0,0113567863065045	0,587375025507391		-0.767646169610389	1,70248981753382	0.767646169610389	297
Q80TB8	sp|Q80TB8|VAT1L_MOUSE Synaptic vesicle membrane protein VAT-1 homolog-like OS=Mus musculus GN=Vat1l PE=2 SV=2	−1,70	2,70×10^−11	3,61×10^−09	31,0545441670067	30,9004869372115	30,8605365941129	30,413494702835	30,4957910271004	30,31031921555	27316787779749,9	16046870817078,5	0,767495849657966	0,0033112107027144 1	0,0030563025022237	0,587436229561887		-0.767495849657968	1,7023124378042	0.767495849657968	298
Q9CZX8	sp|Q9CZX8|RS19_MOUSE 40S ribosomal protein S19 OS=Mus musculus GN=Rps19 PE=1 SV=3	−1,70	0,0295669994455738	0,242684648252187	29,6230833170184	29,469468974537	29,0002145179698	28,5486023400049	28,8657160830252	29,0865735685853	5658928069395,54	3328792241430,7	0,765529968504778	0,0110505107935073	0,0093785283607117	0,588237242214366		-0.76552996850478	1,69999437001913	0.76552996850478	299
P27641	sp|P27641|XRCC5_MOUSE X-ray repair cross-complementing protein 5 OS=Mus musculus GN=Xrcc5 PE=2 SV=4	−1,70	0,688221812057586	0,907856236295823	22,6703028947368	24,7729139695752	22,4077897022244	24,839512426261	22,2553767996608	21,1647787321785	12941031740,7685	7613757557,29879	0,765272106255302	0,0556779073679408	0,0829453684795243	0,588342391071722		-0.765272106255299	1,69969054614338	0.765272106255299	300
Q9DBG7	sp|Q9DBG7|SRPR_MOUSE Signal recognition particle receptor subunit alpha OS=Mus musculus GN=Srpr PE=1 SV=1	−1,70	0,0915579922449589	0,445606408925815	25,4142056413629	25,807698406866	26,1062400879502	25,1667818406085	24,8730804771037	25,698996365124	156457155925,654	92113396680,1767	0,764284747364988	0,013466035503508	0,0165829753138455	0,588745181613473		-0.764284747364987	1,69852770133841	0.764284747364987	301
P06837	sp|P06837|NEUM_MOUSE Neuromodulin OS=Mus musculus GN=Gap43 PE=1 SV=1	−1,70	0,00498041019533608	0,0767764173652645	32,209626068356	31,687944408101	32,0651775689469	31,2665446190202	31,4753765257957	31,6337004171238	77988514501987,2	45948417321611,1	0,7632465023191 1	0,00842070109843115	0,0058539270935680	0,589169028478422		-0.7632465023191 1	1,69730578435629	0.7632465023191 1	302
Q8BG05-2	sp|Q8BG05-2|ROA3_MOUSE Isoform 2 of Heterogeneous nuclear ribonucleoprotein A3 OS=Mus musculus GN=Hnrnpa3	−1,70	0,961681756203232	0,991306002794532	0	23,6663250302842	23,775612781693	0	22,3326153441959	23,5235637626522	7377749,98978884	4348724,0387695	0,762588739978741	0,866032297261002	0,866901178753869	0,589437707266896		-0.762588739978742	1,69653211471115	0.762588739978742	303
Q99LJ0	sp|Q99LJ0|CT2NL_MOUSE CTTNBP2 N-terminal-like protein OS=Mus musculus GN=Cttnbp2nl PE=1 SV=1	−1,70	0,140281795239892	0,532098608288336	25,003648975935	25,3937401987218	24,2429818835158	24,2429818835158	24,2234160190066	24,5893142582617	63870516169,8949	37661494883,8388	0,762059844254334	0,0235223632987255	0,0084525579643045	0,589653836265541		-0.762059844254335	1,6959102756514	0.762059844254335	304
Q9EQF6	sp|Q9EQF6|DPYL5_MOUSE Dihydropyrimidinase-related protein 5 OS=Mus musculus GN=Dpysl5 PE=1 SV=1	−1,69	0,0010869072851809	0,02517077636447	32,2862250679884	32,7396907713201	32,7587169966684	32,1601216416315	32,033953090713	32,0093513947963	143144770041522	84503047019753,2	0,760399692486066	0,00820589594559404	0,0025223472276954	0,590332758893262		-0.760399692486065	1,69395986405154	0.760399692486065	305
Q8R059	sp|Q8R059|GALE_MOUSE UDP-glucose 4-epimerase OS=Mus musculus GN=Gale PE=2 SV=1	−1,69	0,102272814726507	0,46388602391647	26,2722099307776	25,2629129193492	26,1207629911894	25,4761717842147	25,3211500311766	25,2931187432148	174518271429,308	103566544615,982	0,752820048716325	0,0210270773383938	0,0038872842895699	0,593442415901613		-0.752820048716321	1,68508346084549	0.752820048716321	306
P20152	sp|P20152|VIME_MOUSE Vimentin OS=Mus musculus GN=Vim PE=1 SV=3	−1,68	0,00432869145976155	0,0698611208659772	25,7318503388321	26,1582354818588	26,262865549841	25,6844554864885	25,5077187593753	25,4105254331557	205967410173,082	122850404229,528	0,745513475824862	0,0107974966918738	0,0054388355881008	0,596455546662903		-0.745513475824858	1,6765708787434	0.745513475824858	307
Q8BFQ8	sp|Q8BFQ8|PDDC1_MOUSE Parkinson disease 7 domain-containing protein 1 OS=Mus musculus GN=Pddc1 PE=1 SV=1	−1,67	0,0129141930194393	0,148423202317744	28,3886961162417	28,1859618455125	28,0890345798713	27,333361039548	27,869672887315	27,9171142157975	1804366807876,17	1078633467000,68	0,742287949926222	0,00541834437071291	0,0117012733391518	0,597790572455878		-0.742287949926225	1,67282664879063	0.742287949926225	308
Q8BTY1	sp|Q8BTY1|KAT1_MOUSE Kynurenine--oxoglutarate transaminase 1 OS=Mus musculus GN=Ccbl1 PE=1 SV=1	−1,67	0,0160130863059214	0,170071828647511	25,6176425530405	25,9468628589073	25,2357243145929	25,003648975935	25,0588842328911	25,1963348706422	131211528008,33	78494104721,2836	0,741238268149262	0,0139020892342703	0,0039552762357450	0,598225673557436		-0.741238268149263	1,67160996961791	0.741238268149263	309
Q9Z0R4	sp|Q9Z0R4|ITSN1_MOUSE Intersectin-1 OS=Mus musculus GN=Itsn1 PE=1 SV=2;sp|Q9Z0R4-2|ITSN1_MOUSE Isoform 2 of Intersectin-1 OS=Mus musculus GN=Itsn1;sp|Q15811-4|ITSN1_HUMAN Isoform 4 of Intersectin-1 OS=Homo sapiens GN=ITSN1;sp|Q15811-9|ITSN1_HUMAN Isofo	−1,67	0,745590516792846	0,927700293161169	24,4611241663019	21,4780831635369	20,8156783455322	20,543586508048	20,7079743413186	23,9648611641733	4610621767,28054	2760857999,76776	0,73984463183718	0,0872714350086694	0,088761565239638	0,598803835821081		-0.73984463183718	1,66999598228825	0.73984463183718	310
Q8C5Q4	sp|Q8C5Q4|GRSF1_MOUSE G-rich sequence factor 1 OS=Mus musculus GN=Grsf1 PE=1 SV=2	−1,67	0,0282226104394787	0,237741997546121	25,2573533322883	24,839512426261	25,0087957507153	24,9148170158252	24,391301183712	24,2617206040686	74586137544,4794	44672008507,7358	0,739536397073482	0,00839497560074317	0,014100573178206	0,598931785160422		-0.739536397073481	1,66963922232338	0.739536397073481	311
Q9JMG3	sp|Q9JMG3|TMUB1_MOUSE Transmembrane and ubiquitin-like domain-containing protein 1 OS=Mus musculus GN=Tmub1 PE=1 SV=1	−1,67	0,206904545043284	0,637641589296298	23,9242273809443	23,5262499539463	24,8284747330514	23,3845394580678	23,5452376736717	23,8148854521229	29070333467,6667	17431664612,1055	0,737837276656625	0,0276973933459096	0,0092215885065898	0,599637586940437		-0.737837276656622	1,6676739780479	0.737837276656622	312
Q8BIJ7	sp|Q8BIJ7|RUFY1_MOUSE RUN and FYVE domain-containing protein 1 OS=Mus musculus GN=Rufy1 PE=1 SV=1	−1,67	1,54×10^−06	0,000104048993954 47	25,5121220870885	25,3401904290326	25,2573533322883	25,0023493395826	24,7472989515349	24,8266336914146	104232374744,883	62520520065,0507	0,737401766360156	0,00512240572603211	0,0052506649540501	0,599818628502657		-0.737401766360157	1,6671706287221	0.737401766360157	313
P62270	sp|P62270|RS18_MOUSE 40S ribosomal protein S18 OS=Mus musculus GN=Rps18 PE=1 SV=3	−1,66	0,0159566809475985	0,170071828647511	29,8841798123692	29,6067416513827	29,4300026872135	28,8167867144637	29,1996198193394	29,3758840404762	7457997409364,54	4480534775486,9	0,735117360140457	0,00772399429525429	0,0098122376034589	0,600769151496483		-0.735117360140458	1,66453287008671	0.735117360140458	314
Q9D0M0	sp|Q9D0M0|EXOS7_MOUSE Exosome complex exonuclease RRP42 OS=Mus musculus GN=Exosc7 PE=2 SV=2	−1,66	0,581330974382909	0,853379506269256	24,295877056704	23,088004659985	23,8383590411367	23,9703394650702	21,5318623747088	24,1940816395417	20439740760,9871	12290525194,6612	0,73383033276914 1	0,025686797840845 1	0,0635624082977475	0,601305336421872		-0.733830332769134	1,66304860347756	0.733830332769134	315
Q5XJE5	sp|Q5XJE5|LEO1_MOUSE RNA polymerase-associated protein LEO1 OS=Mus musculus GN=Leo1 PE=1 SV=2	−1,66	0,141860504219873	0,533033798554639	25,2687133583084	24,7119475537275	24,5607808475858	24,7055174223122	24,5010625962898	23,8100825694553	61798643615,9526	37174449816,2939	0,733263783088984	0,015004894370999 1	0,0192790094766805	0,601541516789824		-0.733263783088983	1,66239564866043	0.733263783088983	316
Q60790	sp|Q60790|RASA3_MOUSE Ras GTPase-activating protein 3 OS=Mus musculus GN=Rasa3 PE=1 SV=2	−1,66	0,0630872893274378	0,375566342186827	27,6754662525446	27,2152024674969	27,0475341194994	26,5143677630458	27,183957488667	26,7178490875963	727394115610,7	437959136118,06	0,73194098979205 4	0,01190394380037 9	0,0128060632281661	0,602093317390067		-0.731940989792056	1,66087211254024	0.731940989792056	317

Q9JLI6-2	sp|Q9JLI6-2|SCLY_MOUSE Isoform 2 of Selenocysteine lyase OS=Mus musculus GN=Scly;sp|Q9JLI6|SCLY_MOUSE Selenocysteine lyase OS=Mus musculus GN=Scly PE=1 SV=1	−1,66	0,125125816717161	0,505290108611383	25,2227384535695	24,5589890746974	24,6644462690852	24,0050047487481	24,8222977128199	24,0997473766248	59866999624,2992	36080435870,3238	0,730544200791578	0,0143738168358112	0,0183895342918723	0,602676534597522	-0.730544200791576	1,65926486696187	0.730544200791576	318
Q3UDP0	sp|Q3UDP0|WDR41_MOUSE WD repeat-containing protein 41 OS=Mus musculus GN=Wdr41 PE=2 SV=1;sp|Q3UDP0-2|WDR41_MOUSE Isoform 2 of WD repeat-containing protein 41 OS=Mus musculus GN=Wdr41	−1,66	0,0131117589131966	0,149533351022612	24,9566019926909	25,1761940416215	25,5297811363375	24,5401576917216	24,9376594065974	24,6663146621165	89800740370,713	54133041599,0817	0,73021788772565	0,0114662304984505	0,0082186660254948	0,602812865190322	-0.730217887725652	1,65888961192671	0.730217887725652	319
Q9JK38	sp|Q9JK38|GNA1_MOUSE Glucosamine 6-phosphate N-acetyltransferase OS=Mus musculus GN=Gnpnat1 PE=2 SV=1	−1,66	0,0261149764512545	0,223652600211782	24,2310959464558	24,1698893423587	23,8482598894016	23,5004085167368	23,2867314959679	23,9449572067503	28783890964,0389	17358805562,6784	0,729593945466522	0,00853925122425195	0,014242185373455	0,603073628383515	-0.729593945466518	1,65817232413298	0.729593945466518	320
P29699	sp|P29699|FETUA_MOUSE Alpha-2-HS-glycoprotein OS=Mus musculus GN=Ahsg PE=1 SV=1	−1,66	0,0236209398048628	0,21524005202738	27,6529615702981	26,9675427036421	27,12666654682	26,8480719120113	26,8042111906981	26,5794042509612	682519503567,84	411837950360,902	0,728793494173107	0,0131647108913162	0,0053893427178038	0,603408324902128	-0.728793494173107	1,6572525746346	0.728793494173107	321
Q91WU5	sp|Q91WU5|AS3MT_MOUSE Arsenite methyltransferase OS=Mus musculus GN=As3mt PE=2 SV=2	−1,66	0,00447672909569093	0,0706579765279923	28,3325463587444	27,869672887315	27,9628273575294	27,7361956183784	27,5509487648623	27,3642301077782	1528053065086,54	922596822204,653	0,727922416778886	0,00872643451015135	0,0067506405701389	0,603772763711189	-0.727922416778883	1,65625225267423	0.727922416778883	322
Q9CZG3	sp|Q9CZG3|COMD8_MOUSE COMM domain-containing protein 8 OS=Mus musculus GN=Commd8 PE=2 SV=1	−1,66	0,00316684905720265	0,0555356062729631	25,6812465519977	25,3839467459252	25,2084612346714	24,7462638463834	24,9190543270454	25,0954650688965	110088619572,097	66486255615,6539	0,727537302658173	0,00940011967354929	0,0070064971769817	0,603933956789349	-0.727537302658177	1,65581019043246	0.727537302658176	323
Q9Z2X1-2	sp|Q9Z2X1-2|HNRPF_MOUSE Isoform 2 of Heterogeneous nuclear ribonucleoprotein F OS=Mus musculus GN=Hnrnpf;sp|Q9Z2X1|HNRPF_MOUSE Heterogeneous nuclear ribonucleoprotein F OS=Mus musculus GN=Hnrnpf PE=1 SV=3	−1,65	8,19×10^−06	0,00042706851220924	28,6463208267188	28,5178789364239	28,3974533755861	27,9728013953815	28,1859618455125	27,8961549127051	2433985239657,91	1472976134338,99	0,724586363660146	0,00436370396552838	0,0053595902622081	0,605170528702965	-0.724586363660145	1,65242679967125	0.724586363660145	324
Q9Z266	sp|Q9Z266|SNAPN_MOUSE SNARE-associated protein Snapin OS=Mus musculus GN=Snapin PE=1 SV=1;sp|Q9Z266-2|SNAPN_MOUSE Isoform 2 of SNARE-associated protein Snapin OS=Mus musculus GN=Snapin	−1,65	0,0175616437873703	0,179292377689348	25,1895497765879	25,2582028351988	24,8684973230597	24,6220110958197	24,2952278431904	24,8955478776018	80009934483,0667	48472490714,3447	0,723012928278763	0,00828625037656853	0,0122154983636266	0,605830901218941	-0.723012928278762	1,6506256085452	0.723012928278762	325
Q9WUU8-2	sp|Q9WUU8-2|TNIP1_MOUSE Isoform 2 of TNFAIP3-interacting protein 1 OS=Mus musculus GN=Tnip1;sp|Q9WUU8-3|TNIP1_MOUSE Isoform 3 of TNFAIP3-interacting protein 1 OS=Mus musculus GN=Tnip1;sp|Q9WUU8|TNIP1_MOUSE TNFAIP3-interacting protein 1 OS=Mus musculus G	−1,65	0,444694241617639	0,783837990028056	23,6540441959298	24,024166766318	22,8146495088482	21,9012572974974	23,6204012521585	23,4679001027352	16028274518,9024	9710946106,41476	0,72293535960161	0,0263748155932164	0,0413795155088087	0,605863475507765	-0.722935359601608	1,65053686255293	0.722935359601608	326
Q8BV13	sp|Q8BV13|CSN7B_MOUSE COP9 signalosome complex subunit 7b OS=Mus musculus GN=Cops7b PE=1 SV=1	−1,65	0,0804300343045862	0,422536951051524	26,924184307304	26,7734005079762	26,8268300909926	25,807698406866	26,7598691530864	26,4634183601821	454049307275,53	275998694720,33	0,718187530740481	0,002848233034223	0,0184959455726529	0,607860622839462	-0.718187530740481	1,64511396597589	0.718187530740481	327
O09174	sp|O09174|AMACR_MOUSE Alpha-methylacyl-CoA racemase OS=Mus musculus GN=Amacr PE=1 SV=4	−1,64	0,344638391018686	0,730213633407042	25,8984378098441	25,4561566173449	25,1651628272617	25,3711772595212	24,0578211291045	25,6003102586315	119500423409,036	72711802174,3931	0,716754271432607	0,0144758469537678	0,0332803532203553	0,608464807907074	-0.716754271432602	1,64348042319766	0.716754271432602	328
Q9CQT7	sp|Q9CQT7|DESI1_MOUSE Desumoylating isopeptidase 1 OS=Mus musculus GN=Desi1 PE=1 SV=1	−1,64	0,510377843322823	0,816856498886564	24,3040535920527	22,2607396283641	23,897846537075	22,5224468316406	23,835941088441	22,61434833139	15867623079,6879	9656644685,84197	0,716492133179543	0,0460535559207442	0,0318933753697702	0,608575376245445	-0.716492133179547	1,64318182929026	0.716492133179547	329
Q9QUH0	sp|Q9QUH0|GLRX1_MOUSE Glutaredoxin-1 OS=Mus musculus GN=Glrx PE=1 SV=3	−1,64	0,0370376069640573	0,280189496683094	29,2155065783681	29,3627419253404	29,2928257170814	29,1461064414382	28,8887694965435	28,3553953189567	5255824854527,61	3208283213821,11	0,712115697494132	0,00251443443555151	0,0140052576928749	0,610424301155573	-0.712115697494134	1,63820476692513	0.712115697494134	330
P62301	sp|P62301|RS13_MOUSE 40S ribosomal protein S13 OS=Mus musculus GN=Rps13 PE=1 SV=2	−1,64	0,00767589441328302	0,100406007901705	28,801510817816	28,6442862543706	28,3357637084218	27,8772178137753	28,1238312869463	28,304589714348	2619104531828,86	1601368360553,2	0,709768433473981	0,00828618357450406	0,0076340065884181	0,611418269523974	-0.709768433473983	1,6355415757834	0.709768433473983	331
Q8K4Z0-2	sp|Q8K4Z0-2|LGI2_MOUSE Isoform 2 of Leucine-rich repeat LGI family member 2 OS=Mus musculus GN=Lgi2;sp|Q8K4Z0|LGI2_MOUSE Leucine-rich repeat LGI family member 2 OS=Mus musculus GN=Lgi2 PE=2 SV=1	−1,63	0,000249778446965854	0,00771254673998647	22,1461242459219	22,4341047494528	22,3326153441959	21,6089602302478	21,8876980909906	21,9456502500876	4859889055,55676	2976764642,1129	0,707178220098751	0,0065487911375814	0,0082518273955908	0,612516995364182	-0.707178220098754	1,6326077603862	0.707178220098754	332
P62242	sp|P62242|RS8_MOUSE 40S ribosomal protein S8 OS=Mus musculus GN=Rps8 PE=1 SV=2	−1,63	0,0132768510405591	0,14987902972529	27,9358995293517	28,3939473383592	28,5701797962947	27,7704056984658	27,924640903582	27,7361956183784	1952260187867,94	1196490821899,76	0,706336010962259	0,0115691939059484	0,0036098448192144	0,612874671795897	-0.706336010962261	1,63165496310969	0.706336010962261	333
Q91ZR2	sp|Q91ZR2|SNX18_MOUSE Sorting nexin-18 OS=Mus musculus GN=Snx18 PE=1 SV=1	−1,63	0,0103788296067808	0,126298947949539	23,0480487104139	23,3986660676142	23,5891400062898	22,8515326580154	22,6774724465421	23,0429429798347	13763485280,4044	8449017743,03365	0,703990312102346	0,0117568473371629	0,0079976097159756	0,613871964179221	-0.70399031210235	1,62900418711425	0.70399031210235	334

P43135	sp|P43135|COT2_MOUSE COUP transcription factor 2 OS=Mus musculus GN=Nr2f2 PE=1 SV=2;sp|Q60632|COT1_MOUSE COUP transcription factor 1 OS=Mus musculus GN=Nr2f1 PE=2 SV=2	−1,63	0,154674702356734	0,558167424885968	23,1077379050397	22,847371833861	22,8884318811075	22,6026322326214	21,8248730561356	22,9522447247002	9249609826,5667	5678293992,62023	0,70393496388639	0,00610005215602554	0,0256936260742756	0,613895515496346	-0.70393496388864	1,62894169244987	0.70393496388864	335
O89084	sp|O89084|PDE4A_MOUSE cAMP-specific 3,5-cyclic phosphodiesterase 4A OS=Mus musculus GN=Pde4a PE=2 SV=2	−1,63	0,0121089545395876	0,141656042925742	28,7679720830482	28,1697161700063	28,2400659404567	27,9728013953815	27,9254032200445	27,8165098051471	2141622277077,29	1315061839722,79	0,703573408346987	0,0115168234633416	0,0028717812739858	0,614049383870567	-0.703573408346995	1,62853351255994	0.703573408346995	336
Q8CFI5	sp|Q8CFI5|SYPM_MOUSE Probable proline--tRNA ligase, mitochondrial OS=Mus musculus GN=Pars2 PE=2 SV=2	−1,63	0,0127905553885283	0,14790206178145	24,7292894662142	24,3480812542672	24,6180184843432	23,8277112897959	24,3751804819673	24,0297670368369	46612274045,9979	28625189995,6108	0,70342462959692	0,00798001899678263	0,0114980609063404	0,614112711329271	-0.703424629596921	1,62836557777067	0.703424629596921	337
Q8R3K3	sp|Q8R3K3|PTCD2_MOUSE Pentatricopeptide repeat-containing protein 2, mitochondrial OS=Mus musculus GN=Ptcd2 PE=2 SV=1	−1,63	0,0667190397399146	0,38382474192582	23,5957802327646	23,8956506099416	23,7897040107311	23,5680656793277	22,7738720080143	23,4769036420011	20844965318,1159	12803022461,2265	0,703214538511516	0,00640018861906668	0,0186744100005492	0,614202147417327	-0.703214538511521	1,62812846585594	0.703214538511522	338
Q61550	sp|Q61550|RAD21_MOUSE Double-strand-break repair protein rad21 homolog OS=Mus musculus GN=Rad21 PE=1 SV=3	−1,63	0,522452800997369	0,823948365557954	24,2663462043832	22,1014692905163	23,6149347963874	23,3877341976412	23,0974215930066	22,0365622706126	13521994656,2971	8308730671,93173	0,702607984279429	0,0476121316668804	0,0311403269446641	0,61446043155049	-0.702607984279431	1,62744409347346	0.702607984279431	339
Q8VCC9	sp|Q8VCC9|SPON1_MOUSE Spondin-1 OS=Mus musculus GN=Spon1 PE=2 SV=1	−1,62	0,223802905517592	0,652238880554131	24,6548084016843	23,7871003649615	23,9621918362882	24,1039857732243	23,088004659985	23,7565991016167	30308686785,9231	18657794752,994	0,699952866639471	0,0190122857786369	0,0218348461018849	0,61559231796409	-0.699952866639469	1,62445172042958	0.699952866639469	340
Q8R3Y8-2	sp|Q8R3Y8-2|I2BP1_MOUSE Isoform 2 of Interferon regulatory factor 2-binding protein 1 OS=Mus musculus GN=Irf2bp1;sp|Q8R3Y8|I2BP1_MOUSE Interferon regulatory factor 2-binding protein 1 OS=Mus musculus GN=Irf2bp1 PE=1 SV=2	−1,62	0,00090254157596570[illegible]	0,0220249258780019	24,4006943482069	24,0804450555623	23,9261106778015	23,6723843197498	23,5680656793277	23,7113493878119	30340522347,7506	18677768356,8036	0,699923833158383	0,0100298208976363	0,0031324479255925[illegible]	0,615604706561301	-0.699923833158387	1,62441902951959	0.699923833158387	341
Q9QZS8-2	sp|Q9QZS8-2|SH2D3_MOUSE Isoform 2 of SH2 domain-containing protein 3C OS=Mus musculus GN=Sh2d3c;sp|Q9QZS8|SH2D3_MOUSE SH2 domain-containing protein 3C OS=Mus musculus GN=Sh2d3c PE=1 SV=1	−1,62	0,065920925554002	0,382601734678419	22,7448418742479	23,4022584280165	22,6191277512212	22,3912477426894	22,6338453533699	22,2860368944435	9014281751,51886	5549886589,47749	0,699754253157489	0,0183479828875629	0,0079497685762472[illegible]	0,615677071392001	-0.699754253157488	1,62422810019394	0.699754253157488	342
Q923B0	sp|Q923B0|GGACT_MOUSE Gamma-glutamylaminecyclotransferase OS=Mus musculus GN=Ggact PE=1 SV=1;sp|Q923B0-2|GGACT_MOUSE Isoform 2 of Gamma-glutamylaminecyclotransferase OS=Mus musculus GN=Ggact	−1,62	0,000153365224311819	0,00531392941336906	24,0187780694206	24,0336069781538	23,6768094780596	23,5278189761452	23,3964502955791	23,351891275967	24202735684,758	14911325311,993	0,698761638073743	0,00844227863190435	0,0039044746472590[illegible]	0,616100820428478	-0.698761638073742	1,62311096957237	0.698761638073742	343
Q9D820	sp|Q9D820|PRXD1_MOUSE Prolyl-tRNA synthetase associated domain-containing protein 1 OS=Mus musculus GN=Prorsd1 PE=2 SV=1;sp|Q9D820-2|PRXD1_MOUSE Isoform 2 of Prolyl-tRNA synthetase associated domain-containing protein 1 OS=Mus musculus GN=Prorsd1	−1,62	0,500597235269865	0,812083935271593	24,167659258453	23,8670266020545	23,1854445652742	22,2295117621056	24,4713782819228	23,0737103227927	20425367603,6366	12615601790,654	0,695153015839469	0,0211991723653107	0,0486822994439875	0,617643806244539	-0.695153015839475	1,61905614512724	0.695153015839475	344
P07758	sp|P07758|A1AT1_MOUSE Alpha-1-antitrypsin 1-1 OS=Mus musculus GN=Serpina1a PE=1 SV=4	−1,62	0,00876281731285417	0,110950504365343	30,2552727654907	30,8064361728851	30,727262227369	30,2413049199915	30,0650549662063	30,0419786001201	19400573082856,5	12002220962175,8	0,692797874117265	0,00973972918139029	0,0036203795458540[illegible]	0,618652908391746	-0.69279787411727	1,6164152571425	0.69279787411727	345
P53996-2	sp|P53996-2|CNBP_MOUSE Isoform 2 of Cellular nucleic acid-binding protein OS=Mus musculus GN=Cnbp;sp|P53996-3|CNBP_MOUSE Isoform 3 of Cellular nucleic acid-binding protein OS=Mus musculus GN=Cnbp	−1,61	0,00809935702520532	0,104440288458542	28,0805683689805	27,6943403199442	27,6388890090614	27,1000546991755	27,4766700566273	27,4032091695388	1189553607182,32	737583133192,506	0,689542718034847	0,0086532129100532	0,0073057128804991[illegible]	0,62005035228266	-0.68954271803485	1,61277224715475	0.68954271803485	346
Q8VE37	sp|Q8VE37|RCC1_MOUSE Regulator of chromosome condensation OS=Mus musculus GN=Rcc1 PE=1 SV=1	−1,61	0,0440716110895518	0,30775545036227	24,5072217421758	24,1600126179529	24,3957976508923	23,7647352552483	23,5758934844069	24,2905635733946	37753499248,6393	23424869740,4736	0,688569343870369	0,00727909569096609	0,0155102790576301	0,620468836178619	-0.68856934387037	1,61168449032648	0.68856934387037	347
Q3V384	sp|Q3V384|LACE1_MOUSE Lactation elevated protein 1 OS=Mus musculus GN=Lace1 PE=2 SV=1	−1,61	0,0146921152243707	0,161862890784997	25,0138713240005	24,6644462690852	25,0338656906186	24,275977743973	24,7358047995479	24,2686184132209	65417856582,2735	40590517571,127	0,688541754247101	0,00834214143744038	0,0109564504444758	0,620480701933086	-0.688541754247101	1,61165366929952	0.688541754247101	348
P43276	sp|P43276|H15_MOUSE Histone H1.5 OS=Mus musculus GN=Hist1h1b PE=1 SV=2;sp|P16401|H15_HUMAN Histone H1.5 OS=Homo sapiens GN=HIST1H1B PE=1 SV=3	−1,61	0,215262074634428	0,646799042967852	23,1374797714001	22,2687401280658	21,9541395813542	21,6835797836826	22,1779631888277	22,0698626041024	5641710633,4562	3503874496,6192	0,687181570419671	0,0272970606380514	0,0118263217295387	0,621065971700266	-0.687181570419669	1,61013490605892	0.687181570419669	349
Q62465	sp|Q62465|VAT1_MOUSE Synaptic vesicle membrane protein VAT-1 homolog OS=Mus musculus GN=Vat1 PE=1 SV=3	−1,61	5,67×10^−05	0,00236164967161283	30,9037738640846	30,5191558658654	30,6477397566522	30,161244964118	30,2028177448941	30,279068162713	21310545546428,6	13241500055218,7	0,686500960120241	0,00637977716202001	0,0019777363447905[illegible]	0,621359036837882	-0.686500960120243	1,60937548295593	0.686500960120243	350

Q8C050-2	sp|Q8C050-2|KS6A5_MOUSE Isoform 1 of Ribosomal protein S6 kinase alpha-5 OS=Mus musculus GN=Rps6ka5;sp|Q8C050|KS6A5_MOUSE Ribosomal protein S6 kinase alpha-5 OS=Mus musculus GN=Rps6ka5 PE=1 SV=2	−1,61	0,16462196237754	0,57258167603958	26,7044826370826	25,8205849296101	25,8577700523384	26,0003863356302	25,5777956432148	25,3797969498621	222370898718,914	138295652923,875	0,68521218883265	0,0191341635077016	0,0123572246714719	0,621914349946871	-0.685212188832655	1,60793845661453	0.685212188832655	351
P58802	sp|P58802|TB10A_MOUSE TBC1 domain family member 10A OS=Mus musculus GN=Tbc1d10a PE=2 SV=1	−1,61	0,241027596280863	0,664601444032504	25,4695074078681	26,0003863356302	26,7968413620251	25,8217373765899	25,3979425844979	25,6236903849764	213929359534,416	133112008780,305	0,684493759949312	0,0256078291367656	0,00827845891300714	0,62222412608537	-0.684493759949306	1,60713793965424	0.684493759949306	352
Q8K2P6	sp|Q8K2P6|RFESD_MOUSE Rieske domain-containing protein OS=Mus musculus GN=Rfesd PE=1 SV=1	−1,61	0,148433827192997	0,546867809763809	23,5787835721751	23,3145151636562	22,6290908514132	22,5476991420516	23,0310308799512	22,5224468316406	11598363672,7541	7221960457,82467	0,68345885427154	0,021151315428618	0,0126261986107024	0,622670633685154	-0.683458854271545	1,60598548558761	0.683458854271545	353
P63082	sp|P63082|VATL_MOUSE V-type proton ATPase 16 kDa proteolipid subunit OS=Mus musculus GN=Atp6v0c PE=2 SV=1	−1,61	0,0927088823939676	0,446241375595142	32,0149961289679	31,8925920404403	31,2898972692622	31,0218212612287	31,5898004571495	31,1656015127658	60429296594148,5	37639472280947,2	0,683001747853495	0,0122321407922688	0,00944690494815529	0,622867953167469	-0.683001747853497	1,60547672249744	0.683001747853497	354
Q920Q6-3	sp|Q920Q6-3|MSI2H_MOUSE Isoform 3 of RNA-binding protein Musashi homolog 2 OS=Mus musculus GN=Msi2;sp|Q920Q6-2|MSI2H_MOUSE Isoform 2 of RNA-binding protein Musashi homolog 2 OS=Mus musculus GN=Msi2;sp|Q920Q6|MSI2H_MOUSE RNA-binding protein Musashi homol	−1,61	0,000133490975591565	0,0046970196760474	26,5284979350479	26,2029568269526	26,5828657246869	25,9760107033085	26,0204577171094	25,8984378098441	303335966613,773	188991663661,213	0,682593969570722	0,00777106325533595	0,0023783900185343	0,623044031906211	-0.682593969570724	1,60502299803834	0.682593969570724	355
Q3TZX8-3	sp|Q3TZX8-3|NOL9_MOUSE Isoform 3 of Polynucleotide 5-hydroxyl-kinase NOL9 OS=Mus musculus GN=Nol9;sp|Q3TZX8-2|NOL9_MOUSE Isoform 2 of Polynucleotide 5-hydroxyl-kinase NOL9 OS=Mus musculus GN=Nol9;sp|Q3TZX8|NOL9_MOUSE Polynucleotide 5-hydroxyl-kinase	−1,60	0,424899567032437	0,7711031271846	24,2532325976359	23,80523416359	23,7163979450698	22,3236330895835	24,1307029648063	23,9032676843376	24574002253,6025	15321678831,7961	0,681558456518722	0,01202701586829	0,0419676927500384	0,6234913903595	-0.681558456518725	1,60387138533446	0.681558456518725	356
Q5SSL4	sp|Q5SSL4|ABR_MOUSE Active breakpoint cluster region-related protein OS=Mus musculus GN=Abr PE=1 SV=1;sp|Q5SSL4-2|ABR_MOUSE Isoform 2 of Active breakpoint cluster region-related protein OS=Mus musculus GN=Abr	−1,60	0,207136560122328	0,637641589296298	30,392909761289	29,3179435122904	29,8993775648934	29,3492574870933	29,7814723531849	29,0627516643017	9384466573549,09	5852132075903,77	0,681312412730093	0,0180140741748495	0,0123074963434028	0,623597732491104	-0.681312412730092	1,60359787712067	0.681312412730092	357
Q9CR23	sp|Q9CR23|TMEM9_MOUSE Transmembrane protein 9 OS=Mus musculus GN=Tmem9 PE=2 SV=1	−1,60	0,264084390587514	0,68394025335819	22,3263702104077	22,9780575036453	23,1077379050397	23,0239680619191	21,9456502500876	22,026190417643	8010789891,72803	4996163941,46801	0,681123686842719	0,0183624334829701	0,0268972893576142	0,623679313650089	-0.68112368684272	1,60338811647847	0.68112368684272	358
Q9EPJ9-2	sp|Q9EPJ9-2|ARFG1_MOUSE Isoform 2 of ADP-ribosylation factor GTPase-activating protein 1 OS=Mus musculus GN=Arfgap1;sp|Q9EPJ9|ARFG1_MOUSE ADP-ribosylation factor GTPase-activating protein 1 OS=Mus musculus GN=Arfgap1 PE=1 SV=2	−1,60	1,03×10^−07	9,19×10^−06	29,7243921412505	29,5382706477389	29,6067416513827	29,0196626152411	29,258547537147	29,1759515219542	7331012941333,01	4573899433718,63	0,680587906735	0,00317783460769959	0,00416179551394248	0,623910975239249	-0.680587906735	1,60279276961995	0.680587906735	359
P20108	sp|P20108|PRDX3_MOUSE Thioredoxin-dependent peroxide reductase, mitochondrial OS=Mus musculus GN=Prdx3 PE=1 SV=1	−1,60	0,0394327040771164	0,290089212003293	31,7073332672964	31,4485046525447	31,2428831501822	30,6260640465842	31,2146660379869	31,143072998167	46303578186224,2	28892438337211,4	0,680431721173633	0,00739625863720935	0,0103619188889096	0,623978523236617	-0.680431721173635	1,60261926133761	0.680431721173635	360
Q8C863-2	sp|Q8C863-2|ITCH_MOUSE Isoform 2 of E3 ubiquitin-protein ligase Itchy OS=Mus musculus GN=Itch;sp|Q8C863|ITCH_MOUSE E3 ubiquitin-protein ligase Itchy OS=Mus musculus GN=Itch PE=1 SV=2	−1,60	0,450774689651697	0,786343703431612	26,3998169118976	24,6467589088478	26,0516722330455	25,2652188489382	25,7650431834166	24,6548084016843	144915408276,313	90476574726,57	0,679594781303691	0,0361135942350772	0,022040062218225	0,624340612242258	-0.679594781303689	1,60168981544961	0.679594781303689	361
Q8QZZ7	sp|Q8QZZ7|TPRKB_MOUSE EKC/KEOPS complex subunit Tprkb OS=Mus musculus GN=Tprkb PE=2 SV=1	−1,60	0,0647782636125536	0,38006509013234	25,6027289502231	25,0100063220039	24,9190543270454	24,5212483266218	24,6220110958197	24,9762304365785	85969910988,8323	53690215742,5569	0,679172610503587	0,0147458392237351	0,00967225922556784	0,624523337584139	-0.679172610503588	1,60122118713502	0.679172610503588	362
Q3TC46	sp|Q3TC46|PATL1_MOUSE Protein PAT1 homolog 1 OS=Mus musculus GN=Patl1 PE=1 SV=2	−1,60	0,15341306272402	0,556628210794851	22,8052947352916	22,9673450736272	22,2806686370197	22,47968402626	21,7012179112435	22,4605995625438	7107640354,30234	4439616489,17997	0,67893562650998	0,0158230797247318	0,0199893883758693	0,624625933203361	-0.67893562650998	1,60095818447939	0.67893562650998	363
P30713	sp|P30713|GSTT2_RAT Glutathione S-transferase theta-2 OS=Rattus norvegicus GN=Gstt2 PE=1 SV=3	−1,60	0,514040472437379	0,819539762695211	22,0749258890454	22,5318902919992	22,5476991420516	22,6869167687749	22,5476991420516	20,5085414095425	5267588164,93687	3290764591,8317	0,678719730484538	0,011995106204572	0,0556477018784883	0,624719414045372	-0.678719730484536	1,60071862266053	0.678719730484536	364
Q9CPT4	sp|Q9CPT4|CS010_MOUSE UPF0556 protein C19orf10 homolog OS=Mus musculus GN=D17Wsu104e PE=2 SV=1	−1,60	0,12938070532851	0,512962462222669	27,9843470437695	27,7726589193272	27,6030325495586	26,8522659694571	27,8490417699696	27,2481132134366	1168426986564,22	730119204033,439	0,678363652700918	0,00687536266711146	0,018373227406937	0,624873622767279	-0.678363652700919	1,60032359114705	0.678363652700919	365
P16015	sp|P16015|CAH3_MOUSE Carbonic anhydrase 3 OS=Mus musculus GN=Ca3 PE=1 SV=3	−1,60	0,517064111646507	0,82147497471596	21,4244040100507	21,569596405202	23,6506356046121	21,9172465363468	21,6429764340917	21,6767034556501	4444257601,99033	2779792556,85806	0,676965216650919	0,0560669027760501	0,0068780435386011	0,625479620176191	-0.67696521665092	1,59877311385191	0.676965216650919	366
P62245	sp|P62245|RS15A_MOUSE 40S ribosomal protein S15a OS=Mus musculus GN=Rps15a PE=1 SV=2	−1,60	0,00018590062008987	0,00603791059406721	28,4095407476388	28,2627967575825	28,0716553884336	27,6226377001941	27,8646384205141	27,8507513155764	1853316089435,6	1159885580592,44	0,676126464336416	0,0059978808897395	0,0048916865070000	0,625843366495386	-0.676126464336424	1,59784389119569	0.676126464336424	367
P10107	sp|P10107|ANXA1_MOUSE Annexin A1 OS=Mus musculus GN=Anxa1 PE=1 SV=2	−1,60	0,709318727699304	0,916805961322165	25,1715068365119	24,0874845308129	22,0969453884394	23,393552816274	21,7674627603317	24,7917929036	21371246882,7853	13387708230,9946	0,674762068293092	0,0655612626427093	0,0649121140673394	0,626435523599631	-0.674762068293094	1,59633348098426	0.674762068293094	368
Q5BKP2	sp|Q5BKP2|UBP13_MOUSE Ubiquitin carboxyl-terminal hydrolase 13 OS=Mus musculus GN=Usp13 PE=2 SV=1	−1,59	0,599233598340288	0,86182550787914	23,6978719068565	21,711461611698	23,3535376645311	21,8534467936578	23,7382122559989	21,7715837835218	9004200800,78188	5647135586,60061	0,673078959832857	0,0463117139912882	0,0495472671874995	0,627166775990851	-0.673078959832857	1,59447221740998	0.673078959832856	369

P62500-2	sp|P62500-2|T22D1_MOUSE Isoform 2 of TSC22 domain family protein 1 OS=Mus musculus GN=Tsc22d1;sp|P62500-3|T22D1_MOUSE Isoform 3 of TSC22 domain family protein 1 OS=Mus musculus GN=Tsc22d1;sp|P62500|T22D1_MOUSE TSC22 domain family protein 1 OS=Mus muscul	−1,59	0,00697147675624774	0,0936199201083092	24,6103867125833	24,6083461525581	24,3040535920527	24,3206206984587	23,8988976203711	23,9042039329199	44006161085,446	27604392638,7336	0,672807663693293	0,00719268454842839	0,010064567012965	0,62728472463514	-0.67280766369329	1,5941724080427	0.67280766369329	370
Q7TQF7	sp|Q7TQF7|AMPH_MOUSE Amphiphysin OS=Mus musculus GN=Amph PE=1 SV=1	−1,59	0,314259747635035	0,722907157640623	32,4800692515926	31,209001882346	31,8925920404403	31,5462290964587	31,7896349241163	30,8467694077559	68685167643387,1	43085651370705,2	0,67279109222651	0,0199663517369548	0,0155910928066968	0,627291929960856	-0.672791092226509	1,59415409674153	0.672791092226509	371
P61965	sp|P61965|WDR5_MOUSE WD repeat-containing protein 5 OS=Mus musculus GN=Wdr5 PE=1 SV=1	−1,59	0,0228870222363567	0,211576769716543	26,3021104634812	25,7918978973668	25,7958217310218	25,4532890416025	25,7149021750404	25,3243138240262	188672151438,916	118419788759,391	0,671971307292424	0,0113023179304701	0,0078051673878050	0,627648478359194	-0.671971307292419	1,59324850530063	0.671971307292419	372
P39688	sp|P39688|FYN_MOUSE Tyrosine-protein kinase Fyn OS=Mus musculus GN=Fyn PE=1 SV=4;sp|P39688-2|FYN_MOUSE Isoform 2 of Tyrosine-protein kinase Fyn OS=Mus musculus GN=Fyn	−1,59	0,258949811536917	0,680980993375472	25,7852122009926	26,3465902358295	26,2458051503349	24,9476536404833	25,8175097401103	26,2158504682045	221983567480,075	139361616522,494	0,671619620155662	0,0114557142999779	0,0252741411541182	0,627801499473617	-0.671619620155662	1,59286016493821	0.671619620155662	373
P59999	sp|P59999|ARPC4_MOUSE Actin-related protein 2/3 complex subunit 4 OS=Mus musculus GN=Arpc4 PE=1 SV=3	−1,59	0,121285485584897	0,496854529846432	32,3276858928127	32,1651705876441	31,9282291432254	31,2308454760787	31,6172183495446	32,1814288440177	90861898839465,3	57138402605965	0,66921475124378	0,00625010482801444	0,0150918254354458	0,628848872142955	-0.669214751243779	1,5902071933313	0.669214751243779	374
Q64323	sp|Q64323|PIGA_MOUSE N-acetylglucosaminyl-phosphatidylinositol biosynthetic protein OS=Mus musculus GN=Piga PE=2 SV=1	−1,59	0,44591032478706	0,784849692944763	24,9200121770116	26,9663839376261	25,5872669040152	25,4581618858169	25,2054719742707	25,4204801455624	164233386170,941	103348368337,804	0,668231823377162	0,0404118458635832	0,0053751461899611	0,629277461467151	-0.668231823377164	1,58912413241135	0.668231823377164	375
Q9QX66-3	sp|Q9QX66-3|DPF1_MOUSE Isoform 3 of Zinc finger protein neuro-d4 OS=Mus musculus GN=Dpf1;sp|Q9QX66|DPF1_MOUSE Zinc finger protein neuro-d4 OS=Mus musculus GN=Dpf1 PE=1 SV=2;sp|Q9QX66-2|DPF1_MOUSE Isoform 2 of Zinc finger protein neuro-d4 OS=Mus musculus	−1,59	0,162951323781495	0,569625464688209	23,2107589666823	23,2262234311461	23,5324035752081	22,2997639006898	23,3895979642284	22,8906559375229	13461891215,955	8471775400,9509	0,668144856562339	0,0077777842287621	0,0238653979568643	0,629315395960872	-0.668144856562339	1,58902834162058	0.668144856562339	376
Q07417	sp|Q07417|ACADS_MOUSE Short-chain specific acyl-CoA dehydrogenase, mitochondrial OS=Mus musculus GN=Acads PE=1 SV=2	−1,59	0,00087312321608006	0,021422196096148 3	28,2400659404567	27,7989733088988	27,8721796264246	27,4627343947558	27,5472752363342	27,5162976633754	1404084060550,29	884925323488,5	0,66600169014414	0,00845119075902 11	0,0015547875888737	0,63025095744031 1	-0.666001690144143	1,58666954519416	0.666001690144143	377
Q80Y14	sp|Q80Y14|GLRX5_MOUSE Glutaredoxin-related protein 5, mitochondrial OS=Mus musculus GN=Glrx5 PE=1 SV=2	−1,59	0,07393914199187 1	0,404916706615332	28,3553953189567	28,1677723504312	27,7647974432772	27,3454914633062	27,5668665706527	27,9932539266564	1591962059172,04	1004191631358,2	0,664771345739822	0,0107407402863125	0,0119139309733818	0,63078867085593	-0.664771345739825	1,58531699474418	0.664771345739825	378
Q6W8Q3	sp|Q6W8Q3|PC4L1_MOUSE Purkinje cell protein 4-like protein 1 OS=Mus musculus GN=Pcp4l1 PE=1 SV=1	−1,58	0,00046371325778937	0,0131409831217642	26,9415112903228	26,8149454374718	26,7646133507678	26,2250524440376	26,2970734250463	26,6173376487642	453543350482,707	286161213526,679	0,664412311200772	0,00339584135757681	0,0079160369949638	0,630945670842968	-0.664412311200773	1,58492251585459	0.664412311200773	379
O08738	sp|O08738|CASP6_MOUSE Caspase-6 OS=Mus musculus GN=Casp6 PE=2 SV=1	−1,58	0,0739459813290995	0,404916706615332	23,9284441294148	24,5413781637526	24,7472989515349	23,7937470144796	24,0144583554802	24,0288934571652	39743298854,8569	25089107105,8652	0,663650499384811	0,0174529997188356	0,0055038102991722	0,631278928241236	-0.663650499384808	1,5840858220724	0.663650499384808	380
Q8JZV7	sp|Q8JZV7|NAGA_MOUSE Putative N-acetylglucosamine-6-phosphate deacetylase OS=Mus musculus GN=Amdhd2 PE=2 SV=1	−1,58	0,0250682150758841	0,219211300821997	23,7584773744894	23,1309610408383	23,4291787490228	23,0974215930066	23,0529989350732	22,7884248163617	15123853778,9768	9548167754,01734	0,663529987380326	0,0133913225213058	0,0072698691257222	0,631331662786237	-0.663529987380328	1,58395350486103	0.663529987380328	381
Q91WK1	sp|Q91WK1|SPRY4_MOUSE SPRY domain-containing protein 4 OS=Mus musculus GN=Spryd4 PE=1 SV=1	−1,58	0,0162201482381756	0,171615974016501	27,322661625401	26,9791446367106	26,8602290837401	26,3382310811099	26,677510608511	26,76973984521	561575341972,855	354920803077,232	0,661982452225573	0,00887612988316918	0,0085442536215278	0,632009236428312	-0.661982452225571	1,58225535698074	0.661982452225571	382
Q8VEH3	sp|Q8VEH3|ARL8A_MOUSE ADP-ribosylation factor-like protein 8A OS=Mus musculus GN=Arl8a PE=2 SV=1	−1,58	9,17×10^−05	0,00349938418374435	30,3249842800509	30,3537800086882	30,1999089850511	29,6482708054861	30,0196926870257	29,8358818720028	14323047817947	9057507805659,55	0,661152468929163	0,00270036657135659	0,0062247888975937	0,63237293631809	-0.661152468929165	1,58134534634321	0.661152468929165	383
Q8VDU5	sp|Q8VDU5|SNRK_MOUSE SNF-related serine/threonine-protein kinase OS=Mus musculus GN=Snrk PE=2 SV=1;sp|Q9NRH2|SNRK_HUMAN SNF-related serine/threonine-protein kinase OS=Homo sapiens GN=SNRK PE=1 SV=2;sp|Q9NRH2-2|SNRK_HUMAN Isoform 2 of SNF-related serine/	−1,58	0,0032056243321252 1	0,0555356062729631	26,8583139812733	26,696871523607	26,4776551762052	26,2212886157278	26,4086406469355	26,030206424081	385425424688,101	243905143111,696	0,660131562646501	0,00716176192213 48	0,0072166227675217	0,632820586003303	-0.6601315626465	1,58022672163004	0.6601315626465	384
Q8VHC3	sp|Q8VHC3|SELM_MOUSE Selenoprotein M OS=Mus musculus GN=Selm PE=1 SV=3	−1,58	0,0157426497381727	0,169728948127235	25,1379645009733	25,3445401043115	24,8846009566493	24,4879210503984	24,5790747392146	24,9294153710934	81377814254,0857	51532081215,8594	0,659164671741812	0,00916975096594378	0,0094497499590799	0,633244843059571	-0.659164671741813	1,57916801212059	0.659164671741813	385
Q8BMD5-3	sp|Q8BMD5-3|CDAC1_MOUSE Isoform 3 of Cytidine and dCMP deaminase domain-containing protein 1 OS=Mus musculus GN=Cdadc1;sp|Q8BMD5-2|CDAC1_MOUSE Isoform 2 of Cytidine and dCMP deaminase domain-containing protein 1 OS=Mus musculus GN=Cdadc1;sp|Q8BMD5-4|CDA	−1,58	0,162226553123515	0,569625464688209	22,0605658022871	22,7898514546523	21,7012179112435	21,7145801016844	21,6228591796111	21,8445984352844	4308597962,40196	2729395261,10615	0,658637150480611	0,0250043666365302	0,0051280755709971	0,633476431294733	-0.658637150480612	1,57859069508892	0.658637150480612	386

Q3TPE9-2	sp|Q3TPE9-2|ANKY2_MOUSE Isoform 2 of Ankyrin repeat and MYND domain-containing protein 2 OS=Mus musculus GN=Ankmy2;sp|Q3TPE9|ANKY2_MOUSE Ankyrin repeat and MYND domain-containing protein 2 OS=Mus musculus GN=Ankmy2 PE=1 SV=1	−1,58	0,264468903901181	0,68394025335819	26,596578656695	26,4201728012214	27,683736916332	26,4634183601821	26,613068974371	26,2569962681467	481495533256,716	305279789059,821	0,657390334928241	0,025438631987223	0,0067609178697051	0,634024135166913	-0.657390334928242	1,57722702423109	0.657390334928242	387
Q8BP67	sp|Q8BP67|RL24_MOUSE 60S ribosomal protein L24 OS=Mus musculus GN=Rpl24 PE=1 SV=2	−1,58	0,00708700749411835	0,0948906401646111	26,9928732615539	27,1039852603133	27,0220262352562	26,2637346192989	26,6691328676683	26,8191926913355	553555719929,572	350988767836,421	0,657303680543327	0,00213055423147469	0,0108088985219158	0,634062218490086	-0.657303680543325	1,57713229212952	0.657303680543325	388
Q9QZ88	sp|Q9QZ88|VPS29_MOUSE Vacuolar protein sorting-associated protein 29 OS=Mus musculus GN=Vps29 PE=1 SV=1;sp|Q9QZ88-2|VPS29_MOUSE Isoform 2 of Vacuolar protein sorting-associated protein 29 OS=Mus musculus GN=Vps29	−1,58	0,00500931881415341	0,0767764173652645	30,2457298105003	29,9610005225932	29,7939275700076	29,3586082207491	29,6166517537297	29,6607221554744	10688818393437,5	6782232063793,51	0,656270323447272	0,00761461588925859	0,0055236478985094	0,634516540009467	-0.656270323447266	1,57600304632733	0.656270323447266	389
P62254	sp|P62254|UB2G1_MOUSE Ubiquitin-conjugating enzyme E2 G1 OS=Mus musculus GN=Ube2g1 PE=2 SV=3	−1,58	0,258856307525879	0,680980993375472	25,7538649352021	25,8555074920393	25,4898256936456	24,4753092267965	25,5484527128798	25,7112604959504	144961308664,378	91995682039,6398	0,656029832008726	0,0073446359254381	0,0266005248200746	0,634622320171191	-0.656029832008723	1,57574035487792	0.656029832008723	390
P62858	sp|P62858|RS28_MOUSE 40S ribosomal protein S28 OS=Mus musculus GN=Rps28 PE=2 SV=1	−1,58	0,00688421001129315	0,0927223419621947	27,4236219219812	27,1940541511622	27,1403798463766	26,513257270011	26,9015107380824	26,9791446367106	685000432439,275	434721260245,077	0,656014245831514	0,00552049899440634	0,0093143605019756	0,634629176359848	-0.656014245831513	1,57572333143565	0.656014245831513	391
O88834	sp|O88834|SHD_MOUSE SH2 domain-containing adapter protein D OS=Mus musculus GN=Shd PE=1 SV=1;sp|O88834-2|SHD_MOUSE Isoform 2 of SH2 domain-containing adapter protein D OS=Mus musculus GN=Shd	−1,57	0,335167799893345	0,72953198881961	24,4804791910647	23,8848298274034	23,3681777993606	24,1103167979477	22,9454843610105	23,3177018894452	24237388873,6041	15403095758,8855	0,654013946613111	0,0232785857800075	0,025361489088201	0,63550970111555	-0.654013946613113	1,5735401021332	0.654013946613113	392
Q9JME5	sp|Q9JME5|AP3B2_MOUSE AP-3 complex subunit beta-2 OS=Mus musculus GN=Ap3b2 PE=1 SV=2	−1,57	0,0749743816198171	0,406581503192772	24,7568685258369	24,53802616775	24,361859950797	24,5429385120525	23,9631070192037	23,7908431155132	46015841202,8764	29244661532,468	0,653957310343963	0,00805987737544725	0,0163502245650135	0,635534649981362	-0.65395731034396	1,57347833045661	0.65395731034396	393
P61600	sp|P61600|NAA20_MOUSE N-alpha-acetyltransferase 20 OS=Mus musculus GN=Naa20 PE=2 SV=1	−1,57	0,0901039017563411	0,441176105282424	25,0898230655906	25,0688342117586	24,602257940586	24,4288410238104	24,8569465581742	24,1153516205821	66489180534,7022	42257445532,9471	0,653914038030628	0,0110607292665776	0,0152151890362472	0,635553712545637	-0.653914038030627	1,57343113612635	0.653914038030627	394
A2AQ25-9	sp|A2AQ25-9|SKT_MOUSE Isoform 9 of Sickle tail protein OS=Mus musculus GN=Skt;sp|A2AQ25-6|SKT_MOUSE Isoform 6 of Sickle tail protein OS=Mus musculus GN=Skt;sp|A2AQ25-5|SKT_MOUSE Isoform 5 of Sickle tail protein OS=Mus musculus GN=Skt;sp|A2AQ25-8|SKT_MO	−1,57	0,0458594971112512	0,313885648890293	25,4561566173449	25,1359541100947	25,8849767619169	25,1063810759423	25,10451441028	24,9079666938932	117812768832,342	74915056355,8548	0,653168306017254	0,01474251788124	0,0045535656565088	0,635882316478493	-0.653168306017249	1,57261803652284	0.653168306017249	395
Q60597-2	sp|Q60597-2|ODO1_MOUSE Isoform 2 of 2-oxoglutarate dehydrogenase, mitochondrial OS=Mus musculus GN=Ogdh;sp|Q60597|ODO1_MOUSE 2-oxoglutarate dehydrogenase, mitochondrial OS=Mus musculus GN=Ogdh PE=1 SV=3;sp|Q60597-4|ODO1_MOUSE Isoform 4 of 2-oxoglutarate	−1,57	0,405114150172394	0,759526281549977	26,6541371203836	24,846359962555	25,7084262444795	25,2191352268015	25,5677487880404	25,066593920213 5	150361431458,622	95700814373,4432	0,65183144858584	0,0351336735652927	0,010160003356316	0,636471822894152	-0.651831448585844	1,57116146234537	0.651831448585844	396
Q99KR7	sp|Q99KR7|PPIF_MOUSE Peptidyl-prolyl cis-trans isomerase F, mitochondrial OS=Mus musculus GN=Ppif PE=1 SV=1	−1,57	0,187918641430958	0,613201088033873	27,4953994502371	27,2041523196231	26,894872517499	26,1671183698062	26,9639504293006	27,1149582581361	648638526881,641	413811207299,839	0,648441999012367	0,011041511334097 1	0,019039131842962	0,637968899703283	-0.648441999012367	1,56747452809235	0.648441999012367	397
Q9DC53	sp|Q9DC53|CPNE8_MOUSE Copine-8 OS=Mus musculus GN=Cpne8 PE=2 SV=3	−1,56	0,255563745902081	0,67796834754503	23,3393648146177	23,8645191979705	23,936307566344	23,489949026874	23,7203509510732	22,5875559426552	19888294767,4559	12713776083,9387	0,64552699916026	0,0137434637718718	0,0257327189115877	0,639259234267928	-0.645526999160252	1,5643106057673	0.645526999160252	398
Q9DAI2	sp|Q9DAI2|IFT22_MOUSE Intraflagellar transport protein 22 homolog OS=Mus musculus GN=Ift22 PE=1 SV=1	−1,56	0,217833490821129	0,647933299369006	27,4078116367401	26,6573625763755	26,2550580787104	26,1107739160253	26,2985172198808	26,5688885730431	424174499926,23	271183053233,486	0,645390868642909	0,0218527681801884	0,0087478307667006	0,639319556646259	-0.645390868642906	1,5641630067533	0.645390868642906	399
Q8VDN4	sp|Q8VDN4|CCD92_MOUSE Coiled-coil domain-containing protein 92 OS=Mus musculus GN=Ccdc92 PE=2 SV=1	−1,56	0,133517489780264	0,518639007654437	26,7207981765655	26,387321076432	26,794197060117	26,6843310444171	25,7564189171455	26,1226195471115	369015851369,361	236163414394,362	0,643897304926743	0,00814180071262906	0,0178471074081365	0,639981760994808	-0.643897304926745	1,56254453009031	0.643897304926745	400
Q9D8B6	sp|Q9D8B6|F210B_MOUSE Protein FAM210B OS=Mus musculus GN=Fam210b PE=2 SV=3	−1,56	0,0363194042377497	0,279309982475748	22,8085879999257	22,5362472274361	22,4725237041215	21,8085797576661	22,4425016638024	22,2295117621056	6570043631,85326	4207766106,82442	0,642848438446266	0,00789612362798931	0,0145574088846417	0,640447208968916	-0.642848438446266	1,56140894361917	0.642848438446266	401
Q9D2U5-2	sp|Q9D2U5-2|LSMD1_MOUSE Isoform 2 of N-alpha-acetyltransferase 38, NatC auxiliary subunit OS=Mus musculus GN=Naa38;sp|Q9D2U5|LSMD1_MOUSE N-alpha-acetyltransferase 38, NatC auxiliary subunit OS=Mus musculus GN=Naa38 PE=2 SV=1	−1,56	0,0539275247581531	0,3415618119154 7	23,1262887967585	22,5083970764616	22,9159360119671	22,6901014055072	22,252583800289	22,2752567028884	8389168907,939	5380142168,95991	0,640883597730784	0,0137480614085418	0,0109933512783058	0,641320043499001	-0.640883597730784	1,55928387103585	0.640883597730784	402
P67984	sp|P67984|RL22_MOUSE 60S ribosomal protein L22 OS=Mus musculus GN=Rpl22 PE=1 SV=2	−1,56	0,00633926530804677	0,0889510983764734	27,9495817820908	27,683736916332	27,5648173464815	27,0782792312648	27,3089720088055	27,4793096785935	1107041231329,35	710229248129,095	0,64035227706606	0,00710360121430509	0,0073755664816695	0,641556274535721	-0.640352277066055	1,55870971836988	0.640352277066055	403
P08074	sp|P08074|CBR2_MOUSE Carbonyl reductase [NADPH] 2 OS=Mus musculus GN=Cbr2 PE=1 SV=1	−1,56	0,377508891405642	0,74727992066734	23,2058538104204	24,2257383334506	24,5500906114161	23,8261006818307	22,9565167887036	23,8690116229415	26327878975,0676	16899384382,7981	0,639620607337153	0,0292352575369426	0,0218631492138333	0,641881725406052	-0.639620607337149	1,55791941166015	0.639620607337149	404

Q9JLV2-2	sp|Q9JLV2-2|TP4AP_MOUSE Isoform 2 of Short transient receptor potential channel 4-associated protein OS=Mus musculus GN=Trpc4ap;sp|Q9JLV2|TP4AP_MOUSE Short transient receptor potential channel 4-associated protein OS=Mus musculus GN=Trpc4ap PE=1 SV=2	−1,56	0,0599540253848322	0,362841761629005	22,5716530900109	22,3884232655303	22,1461242459219	22,3263702104077	21,7460236150581	21,7051251708901	5183433615,82567	3328673340,9671	0,638960787536154	0,00954218658356162	0,0158475975050614	0,642175358589381	-0.638960787536155	1,55720705664669	0.638960787536155	405
P11031	sp|P11031|TCP4_MOUSE Activated RNA polymerase II transcriptional coactivator p15 OS=Mus musculus GN=Sub1 PE=1 SV=3	−1,56	0,106723835274032	0,47075990957772	29,2013315647317	28,9271249389734	28,6238583935333	28,0588742128856	28,5681116849645	28,796752241005	3619794235322,03	2324623902734,9	0,638910366919926	0,00998907375538358	0,0132653891411641	0,642197802309084	-0.638910366919928	1,55715263491155	0.638910366919928	406
Q9Z160	sp|Q9Z160|COG1_MOUSE Conserved oligomeric Golgi complex subunit 1 OS=Mus musculus GN=Cog1 PE=1 SV=3	−1,56	0,437445470751719	0,778453531535867	24,5212483266218	23,0956367782024	24,2181219065247	23,0429429798347	24,2341635524642	23,2303027245149	25071619273,4452	16106193948,8382	0,63843956558779	0,0313645614698298	0,0272546980088692	0,642407407881197	-0.638439565587791	1,55664456500933	0.638439565587791	407
Q99P72-1	sp|Q99P72-1|RTN4_MOUSE Isoform 3 of Reticulon-4 OS=Mus musculus GN=Rtn4;sp|Q9NQC3-3|RTN4_HUMAN Isoform 3 of Reticulon-4 OS=Homo sapiens GN=RTN4	−1,56	0,495814714946086	0,808951470575228	24,1179472560925	23,0297539046255	23,405784578485	23,735889620616	23,5406755707304	21,9501523629703	16355475639,7157	10509784508,0102	0,638040626914887	0,0235005229383236	0,0424482534824647	0,642585073006956	-0.638040626914894	1,55621417615652	0.638040626914894	408
Q61578	sp|Q61578|ADRO_MOUSE NADPH:adrenodoxin oxidoreductase, mitochondrial OS=Mus musculus GN=Fdxr PE=2 SV=1	−1,56	0,00448325561578801	0,0706579765279923	27,3917352883225	27,0927002045124	26,9928732615539	26,647166109089	26,9128048037016	26,5925627340672	623804539415,035	401113880665,387	0,637082159309424	0,00764224597724027	0,0064122224447060	0,643012122100821	-0.637082159309425	1,55518063443788	0.637082159309425	409
Q9ERA0-3	sp|Q9ERA0-3|TFCP2_MOUSE Isoform 3 of Alpha-globin transcription factor CP2 OS=Mus musculus GN=Tfcp2;sp|Q9ERA0|TFCP2_MOUSE Alpha-globin transcription factor CP2 OS=Mus musculus GN=Tfcp2 PE=1 SV=1;sp|Q9ERA0-2|TFCP2_MOUSE Isoform 2 of Alpha-globin transcri	−1,56	0,439265769813627	0,780009157984922	22,6399360793508	23,5630146461116	23,5262499539463	21,8876980909906	22,9793957310975	23,5376585795527	12426124317,1393	7991019086,8582	0,636924987416557	0,0224862741076665	0,0368057273178719	0,643082177750002	-0.636924987416553	1,55501121722697	0.636924987416553	410
P49962	sp|P49962|SRP09_MOUSE Signal recognition particle 9 kDa protein OS=Mus musculus GN=Srp9 PE=1 SV=2	−1,55	0,130889742578307	0,514825425964414	25,2978380035958	25,346821793299	24,7545101478558	24,2783394430416	24,7764875068518	25,0272681171071	82252255462,3182	53025124693,896	0,633379131536478	0,0130801636687224	0,015437423529885	0,644664689081847	-0.633379131536477	1,55119400354351	0.633379131536477	411
Q8BGC4	sp|Q8BGC4|ZADH2_MOUSE Zinc-binding alcohol dehydrogenase domain-containing protein 2 OS=Mus musculus GN=Zadh2 PE=1 SV=1	−1,55	0,101028609775879	0,46324407107743	28,952674136364	28,7339849515457	28,1288847943823	27,9743749343137	28,3325463587444	28,1925735549002	2648941637553,99	1708263174152,5	0,632885805133283	0,0149179887809825	0,0064084582655698	0,644885168451614	-0.632885805133279	1,55066366683704	0.632885805133279	412
Q8K2F0	sp|Q8K2F0|BRD3_MOUSE Bromodomain-containing protein 3 OS=Mus musculus GN=Brd3 PE=1 SV=2;sp|Q8K2F0-2|BRD3_MOUSE Isoform 2 of Bromodomain-containing protein 3 OS=Mus musculus GN=Brd3	−1,55	0,00386802901730708	0,0645477342263119	22,4474378958212	22,8844377169443	22,4148399246451	22,1514329050445	22,1209126219968	22,1585650899638	6417140898,04619	4138655743,15003	0,632768411148722	0,0116117466906202	0,0009032060598798	0,644937645737234	-0.632768411148722	1,55053749243757	0.632768411148722	413
Q91W67	sp|Q91W67|UBL7_MOUSE Ubiquitin-like protein 7 OS=Mus musculus GN=Ubl7 PE=1 SV=2	−1,55	0,0128057964926437	0,14790206178145	26,1971184130789	25,6704655448343	25,8263359153978	25,5815878578865	25,4918299528731	25,3047067573104	176744900726,579	113989805513,646	0,632763787232087	0,0104462241783769	0,0055486417190183	0,644939712801028	-0.632763787232093	1,55053252288794	0.632763787232093	414
P62630	sp|P62630|EF1A1_RAT Elongation factor 1-alpha 1 OS=Rattus norvegicus GN=Eef1a1 PE=1 SV=1;sp|P10126|EF1A1_MOUSE Elongation factor 1-alpha 1 OS=Mus musculus GN=Eef1a1 PE=1 SV=3;sp|P68104|EF1A1_HUMAN Elongation factor 1-alpha 1 OS=Homo sapiens GN=EEF1A1 PE	−1,55	0,0227889600012917	0,211531880257389	33,0157551936444	32,9720957537316	33,5323458377698	32,7587169966684	32,6101813602848	32,839500065196	255284296521960	164862483233899	0,630841664313834	0,00939375215600076	0,0035531235618349	0,645799547719996	-0.630841664313833	1,54846810210771	0.630841664313834	415
Q9QZB9	sp|Q9QZB9|DCTN5_MOUSE Dynactin subunit 5 OS=Mus musculus GN=Dctn5 PE=1 SV=1	−1,55	5,59×10^−05	0,00236164967161283	27,3661184166555	27,0983319088859	27,1451359480015	26,6292631829283	26,8570170371907	26,8149454374718	651925041027,35	421495618449,612	0,629188457442814	0,00525758113404859	0,0045275166103402	0,646540003717895	-0.629188457442816	1,54669470450328	0.629188457442816	416
Q9Z1B5	sp|Q9Z1B5|MD2L1_MOUSE Mitotic spindle assembly checkpoint protein MAD2A OS=Mus musculus GN=Mad2l1 PE=1 SV=2	−1,54	0,102984441318021	0,464658428571071	24,6164760537761	23,8733808899669	24,1600126179529	24,0912088731561	23,5922800981017	23,6613231107722	32896206602,4266	21292144255,6282	0,62759998466307	0,0154756572024291	0,0113675625084735	0,647252265677878	-0.627599984663067	1,54499266055513	0.627599984663067	417
Q9D6H2	sp|Q9D6H2|IFT25_MOUSE Intraflagellar transport protein 25 homolog OS=Mus musculus GN=Hspb11 PE=1 SV=2	−1,54	0,134384871544999	0,51956808513011	23,9597534682194	23,5787835721751	23,6248549139189	22,78470180127	23,393552816274	23,6813632656051	20042696328,4013	12978231545,6478	0,626982795636118	0,00876574369555847	0,0196598260408328	0,647529221268354	-0.626982795636118	1,54433184967505	0.626982795636118	418
Q9Z0F7	sp|Q9Z0F7|SYUG_MOUSE Gamma-synuclein OS=Mus musculus GN=Sncg PE=1 SV=1	−1,54	0,157028277927179	0,561270396169617	25,6369206672453	25,4119626398757	25,1944575563232	24,4250582171058	25,2995911794629	25,2169254586012	108981824163,921	70616167152,4347	0,626017121511654	0,00870536420455601	0,0193278864057736	0,647962792825159	-0.626017121511659	1,54329849039624	0.626017121511658	419
P61963	sp|P61963|DCAF7_MOUSE DDB1- and CUL4-associated factor 7 OS=Mus musculus GN=Dcaf7 PE=2 SV=1	−1,54	0,140894182574536	0,532098608288336	27,8869880507204	27,2082466545883	26,9056299926328	26,9791446367106	26,8127695771732	26,9096572194275	742747188467,336	481669394005,414	0,624827983180782	0,0183857292546562	0,0031063606174002	0,648497094952782	-0.624827983180784	1,54202695398784	0.624827983180784	420

Q9FZB1	sp|Q9FZB1|Y5188_ARATH Probable LRR receptor-like serine/threonine-protein kinase At1g51880 OS=Arabidopsis thaliana GN=At1g51880 PE=2 SV=1;sp|C0LGG6-2|Y5189_ARATH Isoform 2 of Probable LRR receptor-like protein kinase At1g51890 OS=Arabidopsis thaliana GN=	−1,54	0,663359429832816	0,89612751547951	22,7297871435156	23,2844462621002	21,0220202458726	21,1739565399968	23,3421578262587	21,232668550138 2	5063976513,43093	3296941218,62497	0,619142548270242	0,0527702124700506	0,0563605163603017	0,65105776258650 9	-0.619142548270245	1,53596202589955	0.619142548270245	421
P48320	sp|P48320|DCE2_MOUSE Glutamate decarboxylase 2 OS=Mus musculus GN=Gad2 PE=2 SV=1;sp|Q05329|DCE2_HUMAN Glutamate decarboxylase 2 OS=Homo sapiens GN=GAD2 PE=1 SV=1	−1,53	0,00434034723159409	0,0698611208659772	30,2998683979432	30,7552693729942	30,6814880843035	30,2476801384455	30,1305251963861	30,0740303655199	19064999539432,4	12425168304465,6	0,617661102341828	0,00799332822601783	0,0029376761181374	0,651726651173867	-0.617661102341829	1,53438561734254	0.617661102341829	422
Q9CQR2	sp|Q9CQR2|RS21_MOUSE 40S ribosomal protein S21 OS=Mus musculus GN=Rps21 PE=2 SV=1	−1,53	0,19047121049418	0,617094093099987	27,4793096785935	27,2015892625047	26,8888307780833	26,2113399560879	26,9857690304365	27,0927002045124	643321159147,859	419894669701,054	0,615511666228772	0,0108647898803808	0,0179711276955307	0,652698366485015	-0.6155116662287 7	1,53210127579346	0.6155116662287 7	423
Q64676	sp|Q64676|CGT_MOUSE 2-hydroxyacylsphingosine 1-beta-galactosyltransferase OS=Mus musculus GN=Ugt8 PE=2 SV=2	−1,53	0,241899603989616	0,665847363559046	23,8166383799036	24,0268686400196	24,6012207533644	24,2796892913244	23,4559970658618	23,4291787490228	30721930685,794	20052540723,9479	0,615483840937791	0,01681817689348 6	0,0203816879759345	0,652710955214163	-0.615483840937789	1,53207172640742	0.615483840937789	424
Q9D486	sp|Q9D486|CMIP_MOUSE C-Maf-inducing protein OS=Mus musculus GN=Cmip PE=2 SV=3;sp|Q9D486-2|CMIP_MOUSE Isoform 2 of C-Maf-inducing protein OS=Mus musculus GN=Cmip;sp|Q9D486-3|CMIP_MOUSE Isoform 3 of C-Maf-inducing protein OS=Mus musculus GN=Cmip	−1,53	0,0244219783864136	0,218641735494934	25,2026579862367	24,9294153710934	25,0651396225333	24,7860991569549	24,8358716084403	24,295877056704	76897381330,1648	50200087534,9352	0,615244589530863	0,00545055928936324	0,0121122581899953	0,652819207449956	-0.615244589530862	1,5318176741554 6	0.615244589530861	425
Q3TY60	sp|Q3TY60|F131B_MOUSE Protein FAM131B OS=Mus musculus GN=Fam131b PE=1 SV=1;sp|Q3TY60-2|F131B_MOUSE Isoform 2 of Protein FAM131B OS=Mus musculus GN=Fam131b	−1,53	0,245763251598242	0,669981621023675	25,769120591376	25,4412526778006	25,343368132376	25,6510636985623	24,4689312575944	25,1585755949886	120861826527,191	79011310526,0938	0,613227554056179	0,00873851823525228	0,0236641067759425	0,653732553912034	-0.613227554056173	1,52967753252588	0.613227554056173	426
Q62084	sp|Q62084|PP14B_MOUSE Protein phosphatase 1 regulatory subunit 14B OS=Mus musculus GN=Ppp1r14b PE=1 SV=2	−1,53	0,102506293080339	0,46388602391647	23,6104580044563	24,1228372462825	24,0144583554802	23,6434784339963	23,0785352794043	23,750582547653	24352926614,0047	15920372579,9376	0,613221059407763	0,0112907748423112	0,0153711498952498	0,653735496857377	-0.613221059407758	1,52967064631977	0.613221059407758	427
Q3TNH5-2	sp|Q3TNH5-2|F172A_MOUSE Isoform 2 of Protein FAM172A OS=Mus musculus GN=Fam172a;sp|Q3TNH5-3|F172A_MOUSE Isoform 3 of Protein FAM172A OS=Mus musculus GN=Fam172a;sp|Q3TNH5|F172A_MOUSE Protein FAM172A OS=Mus musculus GN=Fam172a PE=2 SV=2	−1,53	0,354408847219336	0,732843629012038	22,7337848548684	22,9194574103533	22,7318219676303	22,8329525082737	21,4618051524005	22,8163576649882	7938747950,76447	5191932290,90815	0,612639923582759	0,00472775189517475	0,035175344073958	0,653998882834942	-0.612639923582759	1,52905459970393	0.612639923582759	428
Q8BGR6	sp|Q8BGR6|ARL15_MOUSE ADP-ribosylation factor-like protein 15 OS=Mus musculus GN=Arl15 PE=2 SV=1	−1,53	6,17×10^−07	4,68×10^−05	25,0796635391775	25,0723990419734	24,9114903418552	24,6548084016843	24,4665538370577	24,6683213514674	73546544972,4361	48100634091,0088	0,612601656388712	0,00379946745109128	0,004585690664426	0,654016230253046	-0.612601656388715	1,52901404237795	0.612601656388715	429
Q9CWW6	sp|Q9CWW6|PIN4_MOUSE Peptidyl-prolyl cis-trans isomerase NIMA-interacting 4 OS=Mus musculus GN=Pin4 PE=2 SV=1	−1,53	0,0204725896050579	0,199410051968579	25,0371457729701	24,752533055932	24,5934480971174	24,391301183712	24,1365945678446	24,5823748158982	58621985479,9723	38352677858,9194	0,612114518755102	0,00906612094927229	0,009177060206607	0,654237101403231	-0.612114518755101	1,52849784559017	0.612114518755101	430
Q8CBC4	sp|Q8CBC4|CNST_MOUSE Consortin OS=Mus musculus GN=Cnst PE=1 SV=1;sp|Q8CBC4-3|CNST_MOUSE Isoform 3 of Consortin OS=Mus musculus GN=Cnst	−1,53	0,16327004396549	0,569625464688209	25,1009475178655	24,2514976759772	24,6690327146511	24,2840779865289	24,5429385120525	23,921929541055	51964465673,2119	34000758831,2202	0,61195847219118	0,0172144038962969	0,0128633943845634	0,654307869632303	-0.61195847219118	1,5283325272581	0.61195847219118	431
P62855	sp|P62855|RS26_MOUSE 40S ribosomal protein S26 OS=Mus musculus GN=Rps26 PE=2 SV=3	−1,53	0,0207321835657252	0,199524718486067	28,3414907470162	28,1842803916153	27,9786139581145	27,4718930142898	27,7704056984658	27,9902351106764	1711050055598,18	1119807533236,66	0,61163117491946	0,00646037965104342	0,0093772637561196	0,654456326144811	-0.611631174919459	1,52798584114951	0.611631174919459	432
Q03173-6	sp|Q03173-6|ENAH_MOUSE Isoform 6 of Protein enabled homolog OS=Mus musculus GN=Enah;sp|Q03173-3|ENAH_MOUSE Isoform 2 of Protein enabled homolog OS=Mus musculus GN=Enah;sp|Q03173-4|ENAH_MOUSE Isoform 3 of Protein enabled homolog OS=Mus musculus GN=Enah;	−1,53	0,378244740312292	0,748410146590014	24,5749811329927	23,6951786116361	24,1795012645539	23,1051187112	24,49311460887	23,5823483342612	30772491756,4235	20157868621,9657	0,610298163912991	0,0182463893593223	0,0297212588589263	0,65506130545174	-0.61029816391299	1,52657467580135	0.61029816391299	433
Q9D1K7	sp|Q9D1K7|CT027_MOUSE UPF0687 protein C20orf27 homolog OS=Mus musculus PE=2 SV=3	−1,53	0,0197850439555202	0,1954017511157	27,6464734713961	27,4732502744015	27,2176411250769	26,7629318109403	27,1164940541076	27,1900125023832	830910803992,208	544506720890,799	0,609743759576507	0,00786026317777204	0,0084494449673125	0,655313083275188	-0.609743759576507	1,52598815058308	0.609743759576507	434

Q8K4K6	sp|Q8K4K6|PANK1_MOUSE Pantothenate kinase 1 OS=Mus musculus GN=Pank1 PE=1 SV=1;sp|Q8K4K6-2|PANK1_MOUSE Isoform 2 of Pantothenate kinase 1 OS=Mus musculus GN=Pank1	−1,53	0,593292078092682	0,860080030359509	22,2687401280658	23,7280173231175	24,7637322819112	23,3421578262587	23,6043336988036	22,5476991420516	17523872147,7004	11489854003,9805	0,608961127590815	0,0531429585003902	0,0237519991991828	0,655668673403799	-0.608961127590816	1,52516055831776	0.608961127590816	435
P83940	sp|P83940|ELOC_MOUSE Transcription elongation factor B polypeptide 1 OS=Mus musculus GN=Tceb1 PE=1 SV=1	−1,52	0,117867272207617	0,491275985813013	29,0597621446406	28,6169807290695	28,4869189419964	28,0865111619099	28,0992599920198	28,7125758401729	2974867467622,52	1951167442911,49	0,608487806097099	0,0104560726017652	0,0126445967619186	0,655883821429813	-0.608487806097098	1,52466026348999	0.608487806097098	436
Q64523	sp|Q64523|H2A2C_MOUSE Histone H2A type 2-C OS=Mus musculus GN=Hist2h2ac PE=1 SV=3;sp|Q16777|H2A2C_HUMAN Histone H2A type 2-C OS=Homo sapiens GN=HIST2H2AC PE=1 SV=4;sp|Q6GSS7|H2A2A_MOUSE Histone H2A type 2-A OS=Mus musculus GN=Hist2h2aa1 PE=1 SV=3;sp|Q6	−1,52	0,394110267673207	0,753881747531964	28,1896196889447	27,9843470437695	27,7105926216416	27,6784770577248	26,6579118643942	28,2841998398392	139166193925,36	91317762905,07	0,6078413684939	0,0085949547004962	0,0298441968524591	0,656177773344232	-0.607841368493901	1,52397725223679	0.6078413684939	437
Q9CZH3	sp|Q9CZH3|PSMG3_MOUSE Proteasome assembly chaperone 3 OS=Mus musculus GN=Psmg3 PE=1 SV=1	−1,52	0,0771585379626787	0,415489698739721	24,8971877540398	24,7462638463834	24,1881015331326	24,1659420657239	24,0278810485924	24,3751804819673	48776657779,6871	32021323122,9674	0,607157985433059	0,015177462187535	0,0072287521781799	0,656488668567665	-0.607157985433058	1,52325553795439	0.607157985433058	438
P62849-2	sp|P62849-2|RS24_MOUSE Isoform 2 of 40S ribosomal protein S24 OS=Mus musculus GN=Rps24;sp|P62849-3|RS24_MOUSE Isoform 3 of 40S ribosomal protein S24 OS=Mus musculus GN=Rps24;sp|P62849|RS24_MOUSE 40S ribosomal protein S24 OS=Mus musculus GN=Rps24 PE=2 SV	−1,52	0,0465687895863557	0,314182682726068	27,1760569863579	27,1876699138534	26,6089362359476	26,3957362099935	26,6292631829283	26,6885628808377	527222085881,903	346513433722,416	0,605499523387511	0,0122571233625652	0,005826275050066	0,657243774495506	-0.605499523387513	1,52150547301508	0.605499523387513	439
P83877	sp|P83877|TXN4A_MOUSE Thioredoxin-like protein 4A OS=Mus musculus GN=Txnl4a PE=2 SV=1	−1,52	0,183213393739666	0,603487368783993	23,493812176176	23,1903792338134	23,4721841914717	22,3849401581905	23,1374797714001	23,3777626719801	14327670919,9307	9425904500,10632	0,604101137113804	0,00723908725127873	0,0225529959678255	0,657881141518562	-0.604101137113807	1,52003141128462	0.604101137113807	440
P63323	sp|P63323|RS12_MOUSE 40S ribosomal protein S12 OS=Mus musculus GN=Rps12 PE=1 SV=2	−1,52	0,0797337230432392	0,420338407541536	28,9577992893341	29,356105721571	28,6907897587117	28,3160857868948	28,6424177181693	28,7908543389162	3937491349815,34	2591140601367,88	0,60368945242015	0,011544558166939	0,0084971667147078	0,658068900008986	-0.603689452420149	1,51959771991405	0.603689452420149	441
A6H6A9-3	sp|A6H6A9-3|RBG1L_MOUSE Isoform 3 of Rab GTPase-activating protein 1-like OS=Mus musculus GN=Rabgap1l	−1,52	0,442544852807358	0,782208367170015	24,8479407160407	24,2991722917027	24,7729139695752	24,7267151368333	23,1903792338134	24,7499941271413	50236565376,0687	33085562058,8931	0,602536043652654	0,0120757856824653	0,0368997776891025	0,658595224637991	-0.602536043652652	1,51838331434861	0.602536043652652	442
Q9CQL5	sp|Q9CQL5|RM18_MOUSE 39S ribosomal protein L18, mitochondrial OS=Mus musculus GN=Mrpl18 PE=2 SV=1	−1,52	0,252753023920209	0,674733937962647	24,8003069736163	24,4861190808891	23,8690116229415	23,732793378787	24,4506102071283	23,7203509510732	38934473077,0121	25652790113,3444	0,601932353167821	0,0194293421420432	0,0174428937184747	0,658870869077986	-0.601932353167823	1,51774808529536	0.601932353167822	443
P31650	sp|P31650|S6A11_MOUSE Sodium- and chloride-dependent GABA transporter 3 OS=Mus musculus GN=Slc6a11 PE=1 SV=2	−1,52	0,0123304551563555	0,143876442042924	28,6255772652186	29,139589158536	28,9933056026423	28,4660063045051	28,6310783417307	28,410000627506	3627231044488,74	2390112992411,17	0,601789820763235	0,00915772754050733	0,0040324841388318	0,658935966056736	-0.601789820763237	1,51759814536197	0.601789820763237	444
P62257	sp|P62257|UBE2H_MOUSE Ubiquitin-conjugating enzyme E2 H OS=Mus musculus GN=Ube2h PE=2 SV=1	−1,52	0,0288037039184956	0,238644139389349	26,2285373005935	25,8351165872011	25,8629458516474	25,31335724764	25,806618885234	25,5562998340598	190998850467,592	125900614097,367	0,601278635367689	0,00845215848386317	0,0096499213651228	0,659169486042165	-0.601278635367688	1,51706051504944	0.601278635367689	445
O88792	sp|O88792|JAM1_MOUSE Junctional adhesion molecule A OS=Mus musculus GN=F11r PE=1 SV=2	−1,52	0,966667818916808	0,991571803404156	0	21,4919382859881	21,3591905727963	0	20,9840740472232	20,6190916890494	1597115,29542071	1053598,40135253	0,600143402686641	0,866037870393103	0,866125377955488	0,659688379651384	-0.60014340268664	1,51586723496396	0.60014340268664	446
Q8BGT5	sp|Q8BGT5|ALAT2_MOUSE Alanine aminotransferase 2 OS=Mus musculus GN=Gpt2 PE=1 SV=1	−1,51	0,116313224194919	0,487484510268456	25,7001815061086	26,4086406469355	26,4988608819045	25,7650431834166	25,9318139841019	25,6663122517335	239677716908,904	158294512770,813	0,598484540561454	0,0166932445742314	0,0052038732400070	0,660447349099946	-0.598484540561453	1,51412523854141	0.598484540561454	447
Q9D8X2	sp|Q9D8X2|CC124_MOUSE Coiled-coil domain-containing protein 124 OS=Mus musculus GN=Ccdc124 PE=2 SV=1	−1,51	0,00192599759597239	0,0373594149064901	24,6395384667734	24,6548084016843	24,6912518557112	24,5047987626687	24,1764319247229	24,0624940540361	51346686392,2434	33941634263,5838	0,597215145436601	0,00107734403692304	0,0094709999307869	0,661028717691725	-0.597215145436598	1,5127935795164	0.597215145436598	448
Q6ZWN5	sp|Q6ZWN5|RS9_MOUSE 40S ribosomal protein S9 OS=Mus musculus GN=Rps9 PE=1 SV=3	−1,51	1,47×10^−11	2,03×10^−09	29,2163908422627	29,2041525384792	29,0951539669868	28,663140619517	28,796752241005	28,8140965858009	4668687579612,74	3086307418677,82	0,597135277196964	0,00228796704516867	0,0028723861632038	0,661065313548743	-0.597135277196967	1,51270983290861	0.597135277196967	449
Q9CPS6	sp|Q9CPS6|HINT3_MOUSE Histidine triad nucleotide-binding protein 3 OS=Mus musculus GN=Hint3 PE=2 SV=1	−1,51	0,0177486961593594	0,180065108755229	27,2268519579805	27,0003491513743	26,7237904447108	26,4067100219599	26,5688885730431	26,7376285098563	523427230233,601	346474722376,099	0,595238877552864	0,00933696304495335	0,0062274514941895	0,661934844737578	-0.595238877552867	1,51072270624528	0.595238877552867	450
	sp|P61922|GABT_MOUSE 4-aminobutyrate aminotransferase, mitochondrial OS=Mus musculus GN=Abat PE=1 SV=1;sp|P61922-2|GABT_MOUSE																			

P61922	[illegible] Isoform 2 of 4-aminobutyrate aminotransferase, mitochondrial OS=Mus musculus GN=Abat	−1,51	0,000563192425812362	0,0151262155074693	34,1536747954851	33,8225092961663	33,8575362770501	33,4570130838302	33,6444146179947	33,4947052049967	552002370308646	365411160363908	0,595153764640152	0,00535967826379887	0,00295608607266647	0,661973897249014	-0.595153764640151	1,51063358261667	0.595153764640151	451
Q9CYI0	sp|Q9CYI0|NJMU_MOUSE Protein Njmu-R1 OS=Mus musculus PE=2 SV=2	−1,51	0,0540298261633195	0,34156181191547	25,520279157572	25,2553298211587	25,6510636985623	24,8846009566493	24,8846009566493	25,4222737780244	115849475348,152	76750461040,7279	0,594004188726677	0,00791432249393024	0,0123854018811077	0,662501585010002	-0.594004188726677	1,50943035100045	0.594004188726676	452
P11758	sp|P11758|HBB_MYOVE Hemoglobin subunit beta OS=Myotis velifer GN=HBB PE=1 SV=1	−1,51	0,00428688473144325	0,0698611208659772	32,6101813602848	32,4938038454955	32,3027531536818	31,8397012538833	32,1137938104902	32,2182618919671	126202993556507	83615691751308,2	0,593900515291148	0,00478051827003411	0,0060988225791156	0,662549194713591	-0.593900515291144	1,50932188579941	0.593900515291144	453
Q62179	sp|Q62179|SEM4B_MOUSE Semaphorin-4B OS=Mus musculus GN=Sema4b PE=1 SV=2	−1,51	0,139433240908703	0,531391671271705	22,840723799719	22,8598253519744	22,9959305482374	23,0354461367347	22,2152717627516	22,2120966659598	8807122152,82619	5837712460,88873	0,593267523879549	0,00369604647429229	0,0210981616854334	0,662839956070715	-0.593267523879548	1,50865980670199	0.593267523879549	454
Q99JH8	sp|Q99JH8|ERD21_MOUSE ER lumen protein-retaining receptor 1 OS=Mus musculus GN=Kdelr1 PE=2 SV=1	−1,51	0,000994584104656033	0,023512589849134	24,9961132911365	24,7695581872001	25,0006538060705	24,4317373548227	24,7074766283013	24,3934704398956	66609192342,9398	44151591357,0095	0,593255850987277	0,00530179416102466	0,0069894086842487	0,662845319151949	-0.593255850987278	1,50864760013605	0.593255850987278	455
Q9CRB0	sp|Q9CRB0|SNX24_MOUSE Sorting nexin-24 OS=Mus musculus GN=Snx24 PE=1 SV=1	−1,51	0,352680745621708	0,732843629012038	23,0698661387242	23,2244008906573	22,9811816841146	21,8690502708892	22,8146495088482	23,3607684831536	10681896587,7169	7086726901,97026	0,591976463839746	0,005330302024092	0,0332747760245124	0,663433393478016	-0.591976463839748	1,5073103190624	0.591976463839748	456
Q6DFW0	sp|Q6DFW0|CI072_MOUSE Protein C9orf72 homolog OS=Mus musculus PE=1 SV=1	−1,51	0,343648164488529	0,730103496880899	25,0286278335686	24,4377243018323	24,524511933321	24,5863610385453	24,7017312592705	23,4721841914717	51436884855,257	34129416264,7269	0,591787532743156	0,012936845498398	0,0279972614420666	0,663520280451797	-0.591787532743156	1,50711293906358	0.591787532743156	457
Q9D328	sp|Q9D328|TMM35_MOUSE Transmembrane protein 35 OS=Mus musculus GN=Tmem35 PE=2 SV=1	−1,51	0,0156376690878848	0,168998523785498	26,982191551991	26,8060735616088	26,7075038532864	26,1356881565921	26,4927145297487	26,6381041414573	449734384584,559	298539754733,923	0,591150130671689	0,0051862218936281	0,0097846015591159	0,663813497404025	-0.591150130671691	1,50644722337027	0.591150130671691	458
P15209-2	sp|P15209-2|NTRK2_MOUSE Isoform GP95-TRKB of BDNF/NT-3 growth factors receptor OS=Mus musculus GN=Ntrk2;sp|P15209|NTRK2_MOUSE BDNF/NT-3 growth factors receptor OS=Mus musculus GN=Ntrk2 PE=1 SV=1;sp|P15209-4|NTRK2_MOUSE Isoform L10 of BDNF/NT-3 growth fa	−1,51	0,0368709916620839	0,279698526792889	25,8942049257386	25,5531568912004	25,2415366291153	25,2415366291153	25,1651628272617	25,0535866622164	126431456028,678	83945087870,5684	0,590837637334324	0,0127702199592359	0,0037578505702807	0,663957297553604	-0.590837637334322	1,50612095639971	0.590837637334322	459
Q9ERE8	sp|Q9ERE8|MESD1_MOUSE Mesoderm development candidate 1 OS=Mus musculus GN=Mesdc1 PE=1 SV=1	−1,51	0,327083812993703	0,726927682919497	23,5928619631574	23,2979771565564	23,4577010632101	23,3339271274258	22,2332585619834	23,5542435875806	15275459055,8395	10147329716,0886	0,590115606203926	0,00629491982720064	0,030715389729334	0,664289673979355	-0.590115606203932	1,50536737084834	0.590115606203932	460
Q9D1G1	sp|Q9D1G1|RAB1B_MOUSE Ras-related protein Rab-1B OS=Mus musculus GN=Rab1b PE=1 SV=1	−1,50	0,234316619127309	0,657331974177289	30,5147582056784	30,3961101691837	30,2374046939875	29,3206469921288	30,1905445773157	30,413494702835	15669820067438,4	10421534685571,9	0,588420867836278	0,00458016477639882	0,0192647125929421	0,665070475648132	-0.588420867836282	1,50360004934134	0.588420867836282	461
Q9R0H0-2	sp|Q9R0H0-2|ACOX1_MOUSE Isoform 2 of Peroxisomal acyl-coenzyme A oxidase 1 OS=Mus musculus GN=Acox1;sp|Q9R0H0|ACOX1_MOUSE Peroxisomal acyl-coenzyme A oxidase 1 OS=Mus musculus GN=Acox1 PE=1 SV=5	−1,50	0,0847546909555743	0,432248923873429	27,3712277656425	26,9782391770371	27,3835409950841	27,2212697752775	26,5887417463319	26,7044826370826	679304949473,174	452550284357,364	0,585981262107183	0,00846153182811648	0,012548843067685	0,666196065122643	-0.585981262107183	1,50105960144917	0.585981262107183	462
Q9CYR0	sp|Q9CYR0|SSBP_MOUSE Single-stranded DNA-binding protein, mitochondrial OS=Mus musculus GN=Ssbp1 PE=1 SV=1	−1,50	0,218327941228368	0,648158974427884	27,1281225775127	27,1365607688038	26,9675427036421	26,0255942843937	27,0327780523882	26,9594848677855	574869378044,906	383505219637,508	0,583987970351915	0,00351733167527911	0,0210529372823029	0,667117147449712	-0.583987970351917	1,49898710267432	0.583987970351917	463
Q6P1B3	sp|Q6P1B3|PIANP_MOUSE PILR alpha-associated neural protein OS=Mus musculus GN=Pianp PE=1 SV=1	−1,50	0,350179568159988	0,732843629012038	25,1266580132717	24,389762517735	25,0866146564925	24,8569465581742	24,7900819061277	23,7427329093668	63080554904,0695	42097483067,3017	0,583461371484573	0,0166630464327018	0,0255437962459878	0,667360696673039	-0.583461371484571	1,4984400564571	0.583461371484571	464
Q80ZM7	sp|Q80ZM7|T2AG_MOUSE Transcription initiation factor IIA subunit 2 OS=Mus musculus GN=Gtf2a2 PE=2 SV=2	−1,50	0,00284539567862884	0,0508474448240013	24,0516859730864	24,3957976508923	23,9524385927456	23,7308311655272	23,7584773744894	23,6978719068565	30266502370,2396	20202256619,6416	0,583205512414428	0,00964130211867206	0,0012786652047125	0,667479062248899	-0.58320551241443	1,49817433468363	0.58320551241443	465
Q9Z0G0	sp|Q9Z0G0|GIPC1_MOUSE PDZ domain-containing protein GIPC1 OS=Mus musculus GN=Gipc1 PE=1 SV=1	−1,50	0,00137188119545498	0,030375457298391	27,8204849529863	27,6060239625494	27,4252531095558	27,1124388235916	27,2405304600518	27,2900373208184	986327225665,873	659228161150,194	0,581288483663345	0,00716418414676387	0,0033677533969409	0,668366586662096	-0.581288483663345	1,49618490803695	0.581288483663345	466
Q3TXU5	sp|Q3TXU5|DHYS_MOUSE Deoxyhypusine synthase OS=Mus musculus GN=Dhps PE=2 SV=2	−1,50	0,0041769533606039	0,0684447339486682	24,8141487793	24,9493555858095	24,511216945845	24,2573141568899	24,4468590871809	24,361859950797	56541484068,5611	37791286543,96	0,58125611701986	0,00906187710210322	0,0038981298180603	0,668381581532862	-0.58125611701986	1,49615134173298	0.581256117019859	467
Q61097-2	sp|Q61097-2|KSR1_MOUSE Isoform 2 of Kinase suppressor of Ras 1 OS=Mus musculus GN=Ksr1;sp|Q61097|KSR1_MOUSE Kinase suppressor of Ras 1 OS=Mus musculus GN=Ksr1 PE=1 SV=1	−1,50	0,368131890224614	0,739685989256097	25,0214896836834	23,6905689153765	24,4430889369582	24,143043453479	24,2701951976947	23,5344531294744	38930707757,8797	26031259125,7549	0,580663476788421	0,0273674233490629	0,01639726544341	0,668656200335482	-0.580663476788419	1,49553686857054	0.580663476788419	468
Q9CRB6	sp|Q9CRB6|TPPP3_MOUSE Tubulin polymerization-promoting protein family member 3 OS=Mus musculus GN=Tppp3 PE=1 SV=1	−1,49	0,0877867083522557	0,440959089722961	29,97721683577	29,6772864366557	29,5469002992119	28,9750635875556	29,3608168934757	29,6593636472282	8188904060934,61	5477927997552,02	0,580040082494387	0,00742100709079787	0,0116963675677522	0,668945191785141	-0.580040082494386	1,49489078071016	0.580040082494386	469
P70414	sp|P70414|NAC1_MOUSE Sodium/calcium exchanger 1 OS=Mus musculus GN=Slc8a1 PE=1 SV=1	−1,49	0,167269177205227	0,576050679312994	26,9293606494445	26,3883602649067	26,3762555589271	26,262865549841	26,4958547362392	25,7334731810382	344258554674,36	230626300105,57	0,577935461070566	0,0118917028678585	0,0149317734600387	0,669921769478534	-0.577935461070562	1,49271160538401	0.577935461070562	470

P28352	sp|P28352|APEX1_MOUSE DNA-(apurinic or apyrimidinic site) lyase OS=Mus musculus GN=Apex1 PE=1 SV=2	−1,49	0,0018924228454442	0,0369928785341906	29,4746082875131	29,1799288907371	29,1644059935391	28,7307284512667	29,002551104323	28,8917477037687	5165283249977,06	3469421864849,01	0,574152188700811	0,00597092290445371	0,0047335813038723	0,671680854068249	-0.57415218870081	1,48880229940035	0.57415218870081	471
O88851	sp|O88851|RBBP9_MOUSE Putative hydrolase RBBP9 OS=Mus musculus GN=Rbbp9 PE=1 SV=2	−1,49	0,0200764826891484	0,196394730444062	28,9711760736878	28,7636310588687	28,5437339219033	28,130530652555	28,5192469795579	28,4367014913091	3090993526653,33	2077444623170,85	0,573260612113539	0,00743235166944292	0,0072208382943656	0,67209607695948	-0.573260612113542	1,48788251305369	0.573260612113542	472
P52795	sp|P52795|EFNB1_MOUSE Ephrin-B1 OS=Mus musculus GN=Efnb1 PE=1 SV=1	−1,49	0,348853965099963	0,732843629012038	24,5607808475858	24,3684748551525	24,6395384667734	24,6686770330592	23,3098271341832	24,4006943482069	44686236811,1149	30058144870,6354	0,572074583592956	0,00568607623121881	0,0298326518700458	0,672648829161622	-0.57207458359296	1,48665983890343	0.57207458359296	473
Q61324-2	sp|Q61324-2|ARNT2_MOUSE Isoform 2 of Aryl hydrocarbon receptor nuclear translocator 2 OS=Mus musculus GN=Arnt2;sp|Q61324| ARNT2_MOUSE Aryl hydrocarbon receptor nuclear translocator 2 OS=Mus musculus GN=Arnt2 PE=1 SV=2	−1,49	0,161631458811559	0,569600303995083	23,47931822413	23,245900105076	22,6991159284757	23,0529989350732	22,4950198036394	22,6869167687749	11225398292,3573	7551246625,18554	0,571979892848271	0,0173045533041436	0,0124636151330139	0,672692979662622	-0.571979892848272	1,48656226574794	0.571979892848272	474
Q9D0J4	sp|Q9D0J4|ARL2_MOUSE ADP-ribosylation factor-like protein 2 OS=Mus musculus GN=Arl2 PE=1 SV=1	−1,49	0,023270226891185	0,21338092900826	27,2435367500068	26,7080610722193	26,7489665802186	26,4796440426437	26,4722972144417	26,5613075057639	481507735823,634	324131862926,173	0,5709781284047	0,0110798928524599	0,0018640674758378	0,673160239828204	-0.570978128404702	1,48553039950073	0.570978128404702	475
Q8BH48-2	sp|Q8BH48-2|UBAP1_MOUSE Isoform 2 of Ubiquitin-associated protein 1 OS=Mus musculus GN=Ubap1;sp|Q8BH48|UBAP1_MOUSE Ubiquitin-<br>associated protein 1 OS=Mus musculus GN=Ubap1 PE=1 SV=1	−1,49	0,509664424821046	0,816791532413287	23,4485091486631	23,8330236397602	23,9753136992594	22,1997126389534	23,9037358086288	23,9668942617015	20676883262,0375	13922622931,5739	0,570587705697453	0,0114736553858231	0,0429232090998223	0,673342435372533	-0.57058770569746	1,48512843906346	0.57058770569746	476
Q8BH69	sp|Q8BH69|SPS1_MOUSE Selenide, water dikinase 1 OS=Mus musculus GN=Sephs1 PE=2 SV=1	−1,48	0,0158298152657121	0,170071828647511	26,9435946391298	26,5448095234348	26,7434074548887	26,4927145297487	26,4356893840667	26,1186864653252	411855009561,392	277484281803,807	0,569730485116474	0,00745563692091862	0,0076477516846410	0,67374264088548	-0.569730485116477	1,48424626751504	0.569730485116477	477
Q9Z218	sp|Q9Z218|DPP6_MOUSE Dipeptidyl aminopeptidase-like protein 6 OS=Mus musculus GN=Dpp6 PE=1 SV=1	−1,48	0,334429308732002	0,72953198881961	29,9112106332108	28,736881630549	29,3820077371923	28,9577992893341	29,339015370216	28,5486023400049	5541945160046	3733892358310,83	0,569712097047011	0,0200425991547474	0,0136549192036306	0,673751228220352	-0.569712097047011	1,48422734996922	0.569712097047011	478
Q69ZP3-4	sp|Q69ZP3-4|PNKD_MOUSE Isoform 4 of Probable hydrolase PNKD OS=Mus musculus GN=Pnkd;sp|Q69ZP3|PNKD_MOUSE Probable hydrolase<br>PNKD OS=Mus musculus GN=Pnkd PE=2 SV=2;sp|Q69ZP3-3|PNKD_MOUSE Isoform 3 of Probable hydrolase PNKD OS=Mus musculus GN=Pnkd	−1,48	0,0248053658329444	0,219211300821997	22,5052388583284	22,4379044725108	22,6504567909222	22,3810757628557	22,2047387177875	21,8248730561356	6097837521,40994	4110850776,41269	0,56886070671983	0,00482123933018068	0,0128401001142087	0,674148952309602	-0.568860706719829	1,4833517082153	0.568860706719829	479
O54941	sp|O54941|SMCE1_MOUSE SWI/SNF-related matrix-associated actin-dependent regulator of chromatin subfamily E member 1 OS=Mus musculus<br>GN=Smarce1 PE=1 SV=1	−1,48	0,197349448741648	0,626240510020104	25,3346416265303	26,0516722330455	25,3812269421496	25,5419778283143	25,1906725328726	24,8569465581742	129789567877,335	87642537633,6591	0,566471265844125	0,0156787557631769	0,0135952528039004	0,675266426008065	-0.566471265844127	1,48089696375347	0.566471265844127	480
Q9CZV8-4	sp|Q9CZV8-4|FXL20_MOUSE Isoform 2 of F-box/LRR-repeat protein 20 OS=Mus musculus GN=Fbxl20;sp|Q9CZV8|FXL20_MOUSE F-box/LRR-<br>repeat protein 20 OS=Mus musculus GN=Fbxl20 PE=1 SV=3;sp|Q9CZV8-3|FXL20_MOUSE Isoform 3 of F-box/LRR-repeat protein 20 OS=Mus musc	−1,48	0,288784320799424	0,708193823372149	23,3339271274258	23,7203509510732	22,5875559426552	22,9293534018626	22,502151721827	23,0327998585375	12069466537,8525	8151232616,69558	0,566271768320993	0,0248062013117402	0,012326283639055	0,675359809079508	-0.566271768320993	1,48069219778264	0.566271768320993	481
Q9CZP5	sp|Q9CZP5|BCS1_MOUSE Mitochondrial chaperone BCS1 OS=Mus musculus GN=Bcs1l PE=1 SV=1	−1,48	0,294126199719873	0,711969538636193	25,7246189853418	25,5847097988631	25,5041690285268	24,4760775927859	25,5562998340598	25,6042732888588	131793121787,342	89028012579,037	0,565943823588571	0,00435663180672062	0,0253038613152204	0,675513345246426	-0.565943823588576	1,48035565401776	0.565943823588575	482
Q6Y685-2	sp|Q6Y685-2|TACC1_MOUSE Isoform 2 of Transforming acidic coiled-coil-containing protein 1 OS=Mus musculus GN=Tacc1;sp|Q6Y685| TACC1_MOUSE Transforming acidic coiled-coil-containing protein 1 OS=Mus musculus GN=Tacc1 PE=1 SV=1	−1,48	0,393152483241297	0,753881747531964	25,7487841328276	25,7291565919797	25,5599556845734	24,4060266307704	25,5297811363375	25,9259085166726	142029242028,738	95963981111,9854	0,565623078131811	0,0040429082719914	0,0311791802106796	0,675663544642224	-0.565623078131817	1,48002657229275	0.565623078131817	483
Q8VE80	sp|Q8VE80|THOC3_MOUSE THO complex subunit 3 OS=Mus musculus GN=Thoc3 PE=2 SV=1	−1,48	0,252434357565029	0,67459732861346	22,9406662221807	23,0480487104139	23,9952333945482	22,8052947352916	23,0456642223253	22,9576116421202	13527395679,4758	9142413072,83349	0,565237206166541	0,0248776322148226	0,0053020185126772	0,675844285881624	-0.565237206166546	1,4796307683441	0.565237206166546	484
Q07813	sp|Q07813|BAX_MOUSE Apoptosis regulator BAX OS=Mus musculus GN=Bax PE=1 SV=1	−1,48	0,129477249695592	0,512962462222669	27,6263820326853	27,2314097361691	27,0846207130066	26,6579118643942	27,3177386114909	26,7919179286233	728415521753,027	492383047081,026	0,56498057473814	0,0102582411575655	0,0129543437245217	0,67596451802131	-0.564980574738141	1,47936759007294	0.564980574738141	485
Q9R0Q7	sp|Q9R0Q7|TEBP_MOUSE Prostaglandin E synthase 3 OS=Mus musculus GN=Ptges3 PE=1 SV=1	−1,48	0,000428638071920676	0,0125521819899868	30,1444136462248	30,0218464917338	29,9252115172164	29,4808389247203	29,7971159967527	29,6389646388719	11017329429073,4	7448048642140,48	0,564840160825727	0,00365816271021714	0,0053354930750431	0,676030311164698	-0.564840160825726	1,47922361391925	0.564840160825726	486

P13595	sp|P13595|NCAM1_MOUSE Neural cell adhesion molecule 1 OS=Mus musculus GN=Ncam1 PE=1 SV=3;sp|P13595-2|NCAM1_MOUSE Isoform 2 of Neural cell adhesion molecule 1 OS=Mus musculus GN=Ncam1;sp|P13595-3|NCAM1_MOUSE Isoform 3 of Neural cell adhesion molecule 1 O	−1,48	0,0944335123347863	0,450242157645468	29,0254710300426	28,2756209716639	28,4598031167191	28,2810559679654	28,2304457403943	28,0758284429146	2601124687333,98	1759017828272,07	0,564365452756732	0,0136693020196772	0,0037919169088693	0,676252790509237	-0.564365452756736	1,47873696646334	0.564365452756736	487
Q9D1G2	sp|Q9D1G2|PMVK_MOUSE Phosphomevalonate kinase OS=Mus musculus GN=Pmvk PE=2 SV=3;sp|Q9D1G2-2|PMVK_MOUSE Isoform 2 of Phosphomevalonate kinase OS=Mus musculus GN=Pmvk	−1,48	0,0206877588036837	0,199524718486067	24,6522337538154	24,602257940586	24,3850652312053	23,8690116229415	24,361859950797	24,2355146437319	45752806690,0339	30944529650,4792	0,564175854248177	0,00578649467750429	0,0105976272949264	0,676341669268996	-0.564175854248178	1,47854264410593	0.564175854248178	488
Q80XQ2	sp|Q80XQ2|TBCD5_MOUSE TBC1 domain family member 5 OS=Mus musculus GN=Tbc1d5 PE=1 SV=2	−1,48	0,530405304084881	0,829603609662741	24,7637322819112	24,0194215071423	24,6164760537761	24,803332574449	22,9293534018626	24,4941949562678	42236190318,063	28570112118,5379	0,563973012342009	0,0161100283937666	0,041729486476431	0,676436769116447	-0.563973012342008	1,47833477666536	0.563973012342008	489
Q8BFT9	sp|Q8BFT9|SVOP_MOUSE Synaptic vesicle 2-related protein OS=Mus musculus GN=Svop PE=2 SV=1	−1,48	0,34980079551133	0,732843629012038	24,7940826638887	24,6838243385297	24,4081168167823	24,5268886798457	24,7428706679691	23,4448047131735	49670379496,8663	33613312147,9116	0,563353061257984	0,00807210521357256	0,0286954156609337	0,676727508192931	-0.563353061257983	1,47769964704155	0.563353061257983	490
Q8VHI3	sp|Q8VHI3|OFUT2_MOUSE GDP-fucose protein O-fucosyltransferase 2 OS=Mus musculus GN=Pofut2 PE=1 SV=1	−1,48	0,29997037178782	0,713946294657213	23,8072085417944	23,635106284079	24,7178927598584	23,9878581132619	23,5871603405659	23,4148105713821	27942160901,7468	18916045020,2957	0,562833115075891	0,024190490219056	0,0124246819635529	0,676971444220449	-0.562833115075893	1,47716718118225	0.562833115075893	491
Q64378	sp|Q64378|FKBP5_MOUSE Peptidyl-prolyl cis-trans isomerase FKBP5 OS=Mus musculus GN=Fkbp5 PE=1 SV=1	−1,48	0,318866492185589	0,722907157640623	25,9818743454278	25,7355004334847	24,7747333955639	24,9023550022812	25,0651396225333	25,3545598909119	118404124191,977	80164892390,9781	0,562676870350958	0,0250155219341765	0,0091223734911013	0,677044764597904	-0.562676870350955	1,47700721176671	0.562676870350955	492
Q9WUC3	sp|Q9WUC3|LY6H_MOUSE Lymphocyte antigen 6H OS=Mus musculus GN=Ly6h PE=2 SV=2	−1,48	0,0861613245725005	0,437456657980514	28,0364216970831	27,7105926216416	27,3252327797371	27,1253677426794	27,4568631562061	27,3216488345134	1061547678057,53	719119399718,246	0,561865934504226	0,0128566212795301	0,0061052978546450	0,677425437012989	-0.561865934504227	1,47617722241042	0.561865934504227	493
Q9CQE5	sp|Q9CQE5|RGS10_MOUSE Regulator of G-protein signaling 10 OS=Mus musculus GN=Rgs10 PE=2 SV=1	−1,47	0,152787329500803	0,554801350883317	25,979047873499	25,6594416194182	25,4252755505049	24,8701728545749	25,5908651221833	25,4374912672445	143259238289,747	97148808485,0042	0,560359969762843	0,0108215263282713	0,01500592751407	0,678132940289109	-0.56035996976284	1,47463711108573	0.56035996976284	494
P62830	sp|P62830|RL23_MOUSE 60S ribosomal protein L23 OS=Mus musculus GN=Rpl23 PE=1 SV=1	−1,47	0,00646871468760027	0,0900659385491338	27,4606749995793	27,3454914633062	27,1769933127962	26,7995725862334	26,8480719120113	27,1705289713067	738376670688,621	500759186355,441	0,560239988852558	0,00522083027735143	0,0074847544268756	0,678189339173495	-0.560239988852559	1,47451447883078	0.560239988852559	495
Q9D8T7	sp|Q9D8T7|SLIRP_MOUSE SRA stem-loop-interacting RNA-binding protein, mitochondrial OS=Mus musculus GN=Slirp PE=1 SV=2;sp|Q9D8T7-2|SLIRP_MOUSE Isoform 2 of SRA stem-loop-interacting RNA-binding protein, mitochondrial OS=Mus musculus GN=Slirp	−1,47	0,053546888316046	0,340882645254604	26,1961885123238	25,6579078890505	25,5847097988631	25,3144985329333	25,4600043280656	25,500765153124	162336211320,775	110147844148,357	0,559543590330799	0,0129360996417416	0,0038512696059751	0,678516784716047	-0.559543590330793	1,47380289261155	0.559543590330793	496
Q99N28	sp|Q99N28|CADM3_MOUSE Cell adhesion molecule 3 OS=Mus musculus GN=Cadm3 PE=1 SV=1	−1,47	0,0147897036747779	0,162395634253656	30,9161912299983	30,4506158641339	30,7148429894456	30,3583558022131	30,4196223089356	30,1444136462248	21388689307369,8	14533303416629	0,557485412774789	0,00760719294447407	0,0047674815778113	0,679485461113381	-0.557485412774794	1,47170183503476	0.557485412774793	497
Q2WF71	sp|Q2WF71|LRFN1_MOUSE Leucine-rich repeat and fibronectin type III domain-containing protein 1 OS=Mus musculus GN=Lrfn1 PE=1 SV=1;sp|Q2WF71-3|LRFN1_MOUSE Isoform 3 of Leucine-rich repeat and fibronectin type III domain-containing protein 1 OS=Mus musculu	−1,47	1,24×10^−37	7,06×10^−35	25,1577695205742	25,1454481767251	25,10451441028	24,801127570362	24,7267151368333	24,723259791271	82487343180,7275	56098084218,4814	0,556221267070868	0,00110913786510158	0,0017774866495695	0,680081113723981	-0.55622126707087	1,47041283726321	0.55622126707087	498
Q9R020-2	sp|Q9R020-2|ZRAB2_MOUSE Isoform 2 of Zinc finger Ran-binding domain-containing protein 2 OS=Mus musculus GN=Zranb2;sp|Q9R020|ZRAB2_MOUSE Zinc finger Ran-binding domain-containing protein 2 OS=Mus musculus GN=Zranb2 PE=1 SV=2	−1,47	0,0744814205314705	0,405847740446992	25,3412575352839	24,9755754828187	24,7672234633243	24,903996713159	24,5775770341929	24,4478937746508	74050921889,888	50394901221,5778	0,555239922008865	0,0116105158389369	0,0095367989974250	0,680543873532241	-0.555239922008866	1,46941297819592	0.555239922008866	499
O08997	sp|O08997|ATOX1_MOUSE Copper transport protein ATOX1 OS=Mus musculus GN=Atox1 PE=1 SV=1	−1,47	0,449922913368166	0,786343703431612	28,2478477131411	27,1665322140943	27,2429325656057	26,4269514236666	27,5836630656943	27,4925739899774	924424757859,226	629209114016,804	0,555016330283872	0,0219019900601904	0,0236732066316571	0,680649353738557	-0.555016330283872	1,46918526331858	0.555016330283871	500
O08967	sp|O08967|CYH3_MOUSE Cytohesin-3 OS=Mus musculus GN=Cyth3 PE=1 SV=1	−1,47	0,33203992177938	0,72953198881961	24,2081894088514	24,4958624117327	24,7608480287448	24,8266336914146	23,7983315076674	23,6863570906192	43165178321,131	29385702884,5613	0,554753541518553	0,0112873050373899	0,0260753097323997	0,680773346189928	-0.554753541518552	1,46891767369666	0.554753541518552	501
Q9CQ22	sp|Q9CQ22|LTOR1_MOUSE Ragulator complex protein LAMTOR1 OS=Mus musculus GN=Lamtor1 PE=1 SV=1	−1,47	0,0643900106994407	0,38006509013234	24,8549736207267	24,8783295174502	24,6425366875427	24,0531878454148	24,4487515518704	24,7207953569625	58479763518,1932	39817734936,912	0,554526322745464	0,00524035886110854	0,0137540401744639	0,680880573747953	-0.554526322745465	1,46868634317973	0.554526322745465	502
Q9D7M8	sp|Q9D7M8|RPB4_MOUSE DNA-directed RNA polymerase II subunit RPB4 OS=Mus musculus GN=Polr2d PE=2 SV=2	−1,47	0,520861761282871	0,823916573667872	22,7318219676303	22,0223540321112	22,5052388583284	21,0533396385392	22,0605658022871	22,993646883691	5455035438,29645	3715766333,90006	0,553928788314056	0,0161628422989842	0,0440368612489752	0,681162638800465	-0.553928788314053	1,46807816964391	0.553928788314053	503
Q9D187	sp|Q9D187|MIP18_MOUSE Mitotic spindle-associated MMXD complex subunit MIP18 OS=Mus musculus GN=Fam96b PE=2 SV=1	−1,47	0,00458464447820044	0,0720058868046776	24,8266336914146	24,547663319805	24,4818124226021	24,0899649888494	24,2310959464558	24,3832569119608	49177554382,3318	33498704914,2944	0,55389467002046	0,00743574966098546	0,0060524820056412	0,681178747805515	-0.553894670020461	1,46804345147525	0.553894670020461	504
Q9JIY5	sp|Q9JIY5|HTRA2_MOUSE Serine protease HTRA2, mitochondrial OS=Mus musculus GN=Htra2 PE=1 SV=2	−1,47	0,0631995567880722	0,375566342186827	26,6160829578104	25,9585083386998	26,3932386786055	25,9874283403252	25,7943002713021	26,0346229337524	270249107700,703	184107165914,293	0,553744073423553	0,0127049890950349	0,0049089459271624	0,681249856773585	-0.553744073423553	1,46789021686701	0.553744073423553	505

P19324	sp|P19324|SERPH_MOUSE Serpin H1 OS=Mus musculus GN=Serpinh1 PE=1 SV=3;sp|P50454|SERPH_HUMAN Serpin H1 OS=Homo sapiens GN=SERPINH1 PE=1 SV=2	−1,47	0,785793219364323	0,941085863507826	29,6347178699823	25,9168429770321	26,6900416088642	28,6927585562291	26,4330141857912	25,9650637893661	804806316900,77	548404419661,642	0,553401430829624	0,0715616904153648	0,0539630964927917	0,681411674020519	-0.553401430829623	1,46754163177117	0.553401430829623	506
Q62087	sp|Q62087|PON3_MOUSE Serum paraoxonase/lactonase 3 OS=Mus musculus GN=Pon3 PE=1 SV=2	−1,47	0,972324788094184	0,993288144763853	23,8542629495858	0	23,6204012521585	0	23,2567715611815	23,0672620680928	7458672,94725668	5082656,12971709	0,553336340265916	0,866056925393544	0,866047144089961	0,681442418196726	-0.553336340265915	1,46747542168898	0.553336340265915	507
O88845	sp|O88845|AKA10_MOUSE A-kinase anchor protein 10, mitochondrial OS=Mus musculus GN=Akap10 PE=1 SV=3;sp|O88845-2|AKA10_MOUSE Isoform 2 of A-kinase anchor protein 10, mitochondrial OS=Mus musculus GN=Akap10;sp|O88845-3|AKA10_MOUSE Isoform 3 of A-kinase an	−1,47	0,0372811639427975	0,28062886092265	25,426892640985	25,8054283782832	25,5454020810795	25,4301759017466	24,9340519903573	25,2652188489382	130230835018,755	88814483875,251	0,552204203046757	0,00756573202169704	0,0100222710154253	0,681977381642838	-0.552204203046753	1,46632429009752	0.552204203046753	508
O70589	sp|O70589|CSKP_MOUSE Peripheral plasma membrane protein CASK OS=Mus musculus GN=Cask PE=1 SV=2;sp|O70589-3|CSKP_MOUSE Isoform 3 of Peripheral plasma membrane protein CASK OS=Mus musculus GN=Cask;sp|O70589-4|CSKP_MOUSE Isoform 4 of Peripheral plasma memb	−1,47	0,349064185155151	0,732843629012038	29,3922159985882	28,2538132214311	28,8699406290185	28,4428094286615	28,8811067240288	28,046173766494	3345564706532,21	2283422750859,31	0,551051764084626	0,0197597901107568	0,0146763232836732	0,682522369512367	-0.551051764084626	1,46515344356326	0.551051764084626	509
B0F2B4	sp|B0F2B4|NLGN4_MOUSE Neuroligin 4-like OS=Mus musculus GN=Nlgn4l PE=1 SV=1	−1,46	0,481663106243803	0,802303567138125	25,0688342117586	23,5004085167368	23,4260786804776	23,7427329093668	23,4971371284695	23,6149347963874	26447843735,9606	18083545585,4564	0,548472535480821	0,038658048837233	0,0052007123650480	0,683743664171328	-0.548472535480824	1,46253640421219	0.548472535480823	510
P26231	sp|P26231|CTNA1_MOUSE Catenin alpha-1 OS=Mus musculus GN=Ctnna1 PE=1 SV=1	−1,46	0,047566197960737	0,318190913217903	25,6245416244004	25,2670557453431	25,2026579862367	25,1651628272617	25,0796635391775	24,7090851139904	103698323510,672	70907153437,65	0,548389484721657	0,00896034818630468	0,0097033216114908	0,683783025965243	-0.548389484721658	1,46245221368047	0.548389484721658	511
Q5RJG7	sp|Q5RJG7|ISPD_MOUSE Isoprenoid synthase domain-containing protein OS=Mus musculus GN=Ispd PE=2 SV=1;sp|Q5RJG7-5|ISPD_MOUSE Isoform 5 of Isoprenoid synthase domain-containing protein OS=Mus musculus GN=Ispd	−1,46	0,0237569625663636	0,215665706177449	26,0066860711259	25,520279157572	25,6475927807525	25,4918299528731	25,3412575352839	25,2054719742707	148648852935,742	101790894032,136	0,546299823415583	0,0098052825197802	0,0056514460323882	0,684774164225392	-0.546299823415581	1,46033546860108	0.546299823415581	512
Q8K2Q0	sp|Q8K2Q0|COMD9_MOUSE COMM domain-containing protein 9 OS=Mus musculus GN=Commd9 PE=2 SV=3	−1,46	0,0464811545595665	0,314182682726068	26,3762555589271	26,410633198047	26,2339091105729	25,6908631453629	25,8861812107947	26,308372107962	275062988052,437	188394779310,744	0,54600304008076	0,00355721018799011	0,0121572495712812	0,684915046712243	-0.546003040080762	1,46003508727139	0.546003040080762	513
Q8R516-2	sp|Q8R516-2|MIB2_MOUSE Isoform 2 of E3 ubiquitin-protein ligase MIB2 OS=Mus musculus GN=Mib2;sp|Q8R516|MIB2_MOUSE E3 ubiquitin-protein ligase MIB2 OS=Mus musculus GN=Mib2 PE=1 SV=2	−1,46	0,269512836721898	0,689582167914709	23,8680352044495	23,3326444849489	23,5572160930767	23,4666785516388	23,5572160930767	22,6026322326214	17508726676,741	12008040360,9741	0,544073436286265	0,011398756993789	0,0227043380018957	0,685831733093753	-0.544073436286261	1,45808359652454	0.544073436286261	514
Q6PAM1	sp|Q6PAM1|TXLNA_MOUSE Alpha-taxilin OS=Mus musculus GN=Txlna PE=2 SV=1;sp|Q6PAM1-2|TXLNA_MOUSE Isoform 2 of Alpha-taxilin OS=Mus musculus GN=Txlna	−1,46	0,771003523228436	0,936218563920244	28,1113694390224	25,2687133583084	25,784338153794	27,8990401696981	25,2820160355893	24,8549736207267	288551759853,994	198094483260,006	0,542641426793443	0,0573919050769512	0,0633594279386948	0,686512823072855	-0.54264142679344	1,45663703049852	0.54264142679344	515
Q9D3D0	sp|Q9D3D0|TTPAL_MOUSE Alpha-tocopherol transfer protein-like OS=Mus musculus GN=Ttpal PE=2 SV=3	−1,45	0,000167381575687668	0,00558636008857592	27,3974954083987	27,081221549861	27,2537191581988	26,8042111906981	26,8740206459646	26,9359933509139	679175481464,727	467847579907,358	0,537745787255275	0,00581240963225773	0,0024535336469158	0,68884639194923	-0.537745787255275	1,45170245745252	0.537745787255275	516
Q8K2G4-2	sp|Q8K2G4-2|BBS7_MOUSE Isoform 2 of Bardet-Biedl syndrome 7 protein homolog OS=Mus musculus GN=Bbs7;sp|Q8K2G4|BBS7_MOUSE Bardet-Biedl syndrome 7 protein homolog OS=Mus musculus GN=Bbs7 PE=1 SV=1	−1,45	0,0587452621967679	0,359463031694725	25,6571152896275	25,4374912672445	25,2227384535695	25,3393502864799	24,8141487793	25,0467789403677	111703625892,114	76975985642,3637	0,537195675811844	0,00853775652950151	0,0104987734000777	0,68910910480837	-0.537195675811845	1,45114901692974	0.537195675811845	517
Q62422	sp|Q62422|OSTF1_MOUSE Osteoclast-stimulating factor 1 OS=Mus musculus GN=Ostf1 PE=1 SV=2	−1,45	0,00031660549341145	0,00958927199046521	24,903996713159	24,6713112340392	24,8266336914146	24,5589890746974	24,2920359350875	24,4368206009609	58990792707,6084	40691370107,8008	0,535766938159184	0,00477807658597379	0,0054703263441851	0,689791885141977	-0.535766938159182	1,44971261845763	0.535766938159182	518
Q9JJF3	sp|Q9JJF3|NO66_MOUSE Bifunctional lysine-specific demethylase and histidyl-hydroxylase NO66 OS=Mus musculus GN=No66 PE=1 SV=2	−1,45	0,0924040887046832	0,446058049245187	23,6693854597697	24,2796892913244	24,2840779865289	23,6075161552656	23,871854383327	23,6408917707753	28629903320,9824	19756612909,7309	0,535187167298759	0,0146871394954317	0,0060721762175641	0,690069145125321	-0.535187167298761	1,44913014451384	0.535187167298761	519

Q64735-2	sp|Q64735-2|CR1L_MOUSE Isoform 2 of Complement component receptor 1-like protein OS=Mus musculus GN=Cr1l;sp|Q64735|CR1L_MOUSE Complement component receptor 1-like protein OS=Mus musculus GN=Cr1l PE=1 SV=1	−1,45	0,0659780835862332	0,382601734678419	24,2860801827562	23,8870579935421	23,7444339745312	23,5787835721751	23,405784578485	23,8201140153781	25771216080,6104	17783923678,4278	0,53518695403782	0,0117115415568404	0,0088172857345120	0,69006924713219	-0.535186954037823	1,44912993030168	0.535186954037823	520
Q8R3G1	sp|Q8R3G1|PP1R8_MOUSE Nuclear inhibitor of protein phosphatase 1 OS=Mus musculus GN=Ppp1r8 PE=1 SV=1	−1,45	0,0643586971810619	0,38006509013234	24,9274050418198	24,3850652312053	24,7655337624833	24,1086932997909	24,5231160339711	24,333802902532	52952864822,9975	36547212155,9945	0,534947377417399	0,0112752288297931	0,0085301362863730	0,690183850829578	-0.534947377417399	1,44888930507144	0.534947377417399	521
O88986	sp|O88986|KBL_MOUSE 2-amino-3-ketobutyrate coenzyme A ligase, mitochondrial OS=Mus musculus GN=Gcat PE=1 SV=2	−1,45	0,07478267292915	0,406513236437429	27,122691076725	26,7516578076357	26,8289507902745	26,2070979591574	26,5785183382483	26,8060735616088	481946954263,317	332718720347,072	0,534571322799828	0,00727688950417782	0,0113964265085242	0,690363778427963	-0.53457132279983	1,44851168506713	0.53457132279983	522
Q9DBG9	sp|Q9DBG9|TX1B3_MOUSE Tax1-binding protein 3 OS=Mus musculus GN=Tax1bp3 PE=1 SV=1	−1,45	0,049548407301662	0,325942348901802	24,361859950797	24,2965262702175	24,1908952126927	23,775612781693	23,6905689153765	24,2731664169751	35157148691,3884	24284789335,1139	0,533765098664908	0,00355268837390511	0,0131600908154962	0,690749683607373	-0.53376509866491	1,44770243654343	0.53376509866491	523
Q9P2E7-2	sp|Q9P2E7-2|PCD10_HUMAN Isoform 2 of Protocadherin-10 OS=Homo sapiens GN=PCDH10;sp|Q9P2E7|PCD10_HUMAN Protocadherin-10 OS=Homo sapiens GN=PCDH10 PE=2 SV=2	−1,45	0,409945444802644	0,763224927792945	22,6991159284757	22,1710415493333	22,026190417643	22,2687401280658	21,1351427286112	22,3849401581905	4833238765,80245	3341239462,39938	0,532606884293533	0,0158835081699139	0,0314859976660509	0,69130444910777	-0.532606884293534	1,44654066857323	0.532606884293534	524
Q71KT5	sp|Q71KT5|ERG24_MOUSE Delta(14)-sterol reductase OS=Mus musculus GN=Tm7sf2 PE=2 SV=2	−1,45	0,243104341282211	0,667544225698703	22,0882083383777	22,3016141097192	23,0175812679144	21,9746816952802	22,3884232655303	21,9376699279833	5730877928,06447	3962964908,22587	0,532175973710493	0,0216655775880433	0,0113230980332359	0,691510961840416	-0.532175973710495	1,44610867387924	0.532175973710495	525
P63089	sp|P63089|PTN_MOUSE Pleiotrophin OS=Mus musculus GN=Ptn PE=2 SV=1;sp|P21246|PTN_HUMAN Pleiotrophin OS=Homo sapiens GN=PTN PE=1 SV=1	−1,44	0,301200353831691	0,714288613397098	25,4023621789469	24,7251231135384	24,4760775927859	24,0997473766248	24,8641690818116	24,5401576917216	63091651702,7593	43732529752,2776	0,52874231522039	0,0192760360028704	0,0156597450539018	0,693158739262567	-0.52874231522039	1,44267098336504	0.52874231522039	526
Q61183	sp|Q61183|PAPOA_MOUSE Poly(A) polymerase alpha OS=Mus musculus GN=Papola PE=1 SV=4;sp|Q61183-4|PAPOA_MOUSE Isoform 4 of Poly(A) polymerase alpha OS=Mus musculus GN=Papola;sp|Q61183-3|PAPOA_MOUSE Isoform 3 of Poly(A) polymerase alpha OS=Mus musculus GN=P	−1,44	0,224695183901501	0,652238880554131	25,2995911794629	25,270357393125	24,9200121770116	24,9183070413901	25,2026579862367	24,2701951976947	84779554651,4584	58779171825,2187	0,528411355767401	0,00839385836310666	0,0192728787712798	0,693317770621334	-0.528411355767402	1,44234006738905	0.528411355767402	527
P51910	sp|P51910|APOD_MOUSE Apolipoprotein D OS=Mus musculus GN=Apod PE=2 SV=1	−1,44	0,089321342863194	0,441176105282424	25,6555739842145	25,9200419149704	26,2569962681467	25,668783905484	25,32730793297	25,7383458751693	185107774224,679	128365295132,888	0,528110279465339	0,011618714440547	0,0086010465955918	0,693462474337148	-0.528110279465343	1,4420390965724	0.528110279465343	528
Q8WUR0	sp|Q8WUR0|CS012_MOUSE Protein C19orf12 homolog OS=Mus musculus PE=2 SV=1	−1,44	0,133610205619731	0,518639007654437	25,1789507460175	25,1577695205742	24,6467589088478	24,2920359350875	24,8499747977413	24,7436143644013	71609462390,9859	49663778378,3489	0,52795621141743	0,0120559665873662	0,0120281216820294	0,693536534420352	-0.52795621141743	1,44188510679655	0.52795621141743	529
Q9WVJ3-2	sp|Q9WVJ3-2|CBPQ_MOUSE Isoform 2 of Carboxypeptidase Q OS=Mus musculus GN=Cpq;sp|Q9WVJ3|CBPQ_MOUSE Carboxypeptidase Q OS=Mus musculus GN=Cpq PE=2 SV=1	−1,44	0,106421913094633	0,47075990957772	22,6586817635727	22,9386828286442	23,29481600134	22,5362472274361	22,4077897022244	22,8515326580154	9400795947,01785	6522497527,43036	0,527358419989578	0,013883672945125	0,0101038628108975	0,693823965990821	-0.527358419989578	1,44128777473396	0.527358419989578	530
Q3UNZ8	sp|Q3UNZ8|QORL2_MOUSE Quinone oxidoreductase-like protein 2 OS=Mus musculus PE=1 SV=1;sp|Q3UNZ8-2|QORL2_MOUSE Isoform 2 of Quinone oxidoreductase-like protein 2 OS=Mus musculus	−1,44	0,00155371984754789	0,0327329861064157	27,9148680147052	27,7229066479733	28,0545669515707	27,4101106762398	27,5612721540117	27,6254440730023	1305291110165,3	905973265628,71	0,526831213591328	0,00596882651139938	0,0040155197054275	0,694077557544982	-0.526831213591332	1,44076117881854	0.526831213591332	531
Q9CY34	sp|Q9CY34|UB2FA_MOUSE NEDD8-conjugating enzyme UBE2F OS=Mus musculus GN=Ube2f PE=1 SV=1	−1,44	0,0491100606448981	0,323997914632547	24,9261116536089	24,5934480971174	24,595883751326	24,0558013103755	24,5589890746974	24,4060266307704	53617847964,98	37225964999,8298	0,526404067759773	0,00774592232713861	0,0105979813318919	0,694283086932985	-0.526404067759773	1,44033466869765	0.526404067759773	532
Q9D6U8	sp|Q9D6U8|F162A_MOUSE Protein FAM162A OS=Mus musculus GN=Fam162a PE=2 SV=1	−1,44	0,0866514319521725	0,439453463274761	25,4434411168015	25,4779842302653	24,9238571579815	24,690050667465	24,9975459496661	25,0631252435222	95439734703,895	66263647916,5856	0,526372404540287	0,0122789910551949	0,0079938125687180	0,694298324719477	-0.526372404540286	1,44030305762878	0.526372404540286	533
P61458	sp|P61458|PHS_MOUSE Pterin-4-alpha-carbinolamine dehydratase OS=Mus musculus GN=Pcbd1 PE=1 SV=2	−1,44	0,0213181310318184	0,202889111080377	26,9456779435363	27,0553730818285	27,0177302526787	26,35933783101	26,677510608511	26,8914057622921	535389537305,701	372220181026,307	0,524432668277592	0,00206395788395588	0,0100489480424888	0,695232452429816	-0.524432668277593	1,43836783870636	0.524432668277593	534
P57759	sp|P57759|ERP29_MOUSE Endoplasmic reticulum resident protein 29 OS=Mus musculus GN=Erp29 PE=1 SV=2	−1,44	0,101184178471115	0,463446000081122	29,2251086014483	29,6976512816979	29,9490951305956	29,3120180582318	29,1339128359005	29,3362258860121	7337003782861,87	5102332615663,63	0,524034079225494	0,0124081013950707	0,0037757896919744	0,695424558398335	-0.524034079225494	1,43797050006854	0.524034079225494	535
O54774	sp|O54774|AP3D1_MOUSE AP-3 complex subunit delta-1 OS=Mus musculus GN=Ap3d1 PE=1 SV=1	−1,44	0,0520434778954848	0,335456444875023	24,5917632179099	24,143043453479	24,1143670029103	24,018150513668	24,0319190122397	23,7132586719014	35155885876,2242	24479684324,5814	0,522181294701308	0,0110254182570304	0,0075304035011610	0,696318232763889	-0.522181294701306	1,43612496836498	0.522181294701306	536
Q62426	sp|Q62426|CYTB_MOUSE Cystatin-B OS=Mus musculus GN=Cstb PE=1 SV=1	−1,43	0,13427801740698	0,51956808513011	29,4026825687162	29,1249791637666	28,9940526281682	28,5178789364239	28,7045051167192	29,2163908422627	4678060827461,83	3260575942349,34	0,520783798697426	0,0071523560372976	0,0125519056014385	0,696993062426344	-0.520783798697423	1,43473451015257	0.520783798697423	537

Q9CZT8	sp|Q9CZT8|RAB3B_MOUSE Ras-related protein Rab-3B OS=Mus musculus GN=Rab3b PE=1 SV=1	−1,43	0,162236736886942	0,569625464688209	28,6907897587117	29,21883644974	29,3120180582318	28,9032203325384	28,3747558938646	28,8639896558021	4232785581802,1	2953430899859,95	0,519215550347374	0,0115231455197328	0,0102541753325417	0,697751124592173	-0.519215550347373	1,43317576246758	0.519215550347373	538
Q9R0Y5	sp|Q9R0Y5|KAD1_MOUSE Adenylate kinase isoenzyme 1 OS=Mus musculus GN=Ak1 PE=1 SV=1;sp|Q9R0Y5-2|KAD1_MOUSE Isoform 2 of Adenylate kinase isoenzyme 1 OS=Mus musculus GN=Ak1	−1,43	0,0587421471354021	0,359463031694725	32,9002084952502	32,5441233278785	32,5562887960631	32,0248903492098	32,3661802272999	32,5334327252068	153830665659908	107463005269414	0,517503039112148	0,0061887014642075 8	0,0080223379076095	0,698579862528811	-0.517503039112144	1,43147556011716	0.517503039112144	539
P62331	sp|P62331|ARF6_MOUSE ADP-ribosylation factor 6 OS=Mus musculus GN=Arf6 PE=1 SV=2	−1,43	0,210586833836682	0,641644645910089	29,7856451223423	29,7453450924431	29,3922159985882	28,7849374021746	29,41407607155	29,6482708054861	7463672772855,74	5214311085020,62	0,517409079600145	0,0073024606216377 3	0,0152469925152529	0,698625360959592	-0.517409079600147	1,43138233434076	0.517409079600147	540
Q8QGX4	sp|Q8QGX4|PRKDC_CHICK DNA-dependent protein kinase catalytic subunit OS=Gallus gallus GN=PRKDC PE=2 SV=1	−1,43	0,249674335563975	0,673464050610029	22,7780591977932	23,0407523644372	22,3849401581905	21,9944238728401	22,3016141097192	22,8346140135788	7473161006,86477	5225853646,18382	0,516051883533977	0,0145179953111813	0,0189982830882478	0,699282892658596	-0.516051883533976	1,43003641372966	0.516051883533976	541
Q3USH1	sp|Q3USH1|F196A_MOUSE Protein FAM196A OS=Mus musculus GN=Fam196a PE=2 SV=1	−1,43	0,0230889527816667	0,212147280720617	22,0311999668215	22,547699142051 6	22,3388119487222	22,0428427182337	21,9088833413978	21,8938035010294	4867779357,02684	3404996857,20594	0,5156103095201	0,0116479193972485	0,0037379240934975	0,699496958975901	-0.515610309520099	1,42959878119277	0.515610309520099	542
Q8BK64	sp|Q8BK64|AHSA1_MOUSE Activator of 90 kDa heat shock protein ATPase homolog 1 OS=Mus musculus GN=Ahsa1 PE=2 SV=2	−1,43	0,0135263266339139	0,151015968905346	30,6668670710889	30,3786052252511	30,4337492602583	30,153305359808	30,3249842800509	29,9295036034965	17497407660499,9	12242456668918,4	0,515248104727795	0,0050179913521651 6	0,0065805824050709	0,699672597590301	-0.515248104727797	1,42923990941483	0.515248104727797	543
P17047-3	sp|P17047-3|LAMP2_MOUSE Isoform LAMP-2C of Lysosome-associated membrane glycoprotein 2 OS=Mus musculus GN=Lamp2;sp|P17047|LAMP2_MOUSE Lysosome-associated membrane glycoprotein 2 OS=Mus musculus GN=Lamp2 PE=2 SV=2;sp|P17047-2|LAMP2_MOUSE Isoform LAMP-2B	−1,43	0,0924010507573582	0,446058049245187	26,5771116775476	26,4513611202461	26,2409001964166	25,7112604959504	26,3465902358295	26,1438469895555	298825112565,307	209341467982,081	0,513443273818797	0,0064290290550596 2	0,0124493499776909	0,700548445158973	-0.513443273818797	1,42745302899512	0.513443273818797	544
Q5HZI2	sp|Q5HZI2|C2C4C_MOUSE C2 calcium-dependent domain-containing protein 4C OS=Mus musculus GN=C2cd4cC2CD4 family PE=2 SV=1	−1,43	0,0510519753413053	0,332938097807737	28,0224070708488	27,5950102812602	28,0297486856985	27,3642301077782	27,4732502744015	27,7421650657753	1285782604034,66	900799448998,687	0,513368887008995	0,0089269275249256	0,0070667446621694	0,700584567073832	-0.513368887008992	1,42737942997624	0.513368887008992	545
Q05186	sp|Q05186|RCN1_MOUSE Reticulocalbin-1 OS=Mus musculus GN=Rcn1 PE=1 SV=1	−1,43	0,0811420667371178	0,423824903244853	25,5517501914311	25,0126127548663	25,5940945146071	24,9101751598063	25,1824661258207	25,0006538060705	105941457098,755	74279315919,1302	0,51223482259884	0,0127702296423609	0,0055401070623933	0,701135494576873	-0.512234822598844	1,42625784564435	0.512234822598844	546
Q80U57	sp|Q80U57|RIMS3_MOUSE Regulating synaptic membrane exocytosis protein 3 OS=Mus musculus GN=Rims3 PE=2 SV=2	−1,42	0,132157094574273	0,516676186281332	26,6482374292033	26,0967000396117	26,0003863356302	25,7383458751693	26,1321115412	25,8123716078073	250930355173,565	176092690054,696	0,510951983487975	0,0133176137887813	0,0080817277765938	0,701759218939037	-0.510951983487976	1,42499018610951	0.510951983487975	547
Q9D8L5	sp|Q9D8L5|CCD91_MOUSE Coiled-coil domain-containing protein 91 OS=Mus musculus GN=Ccdc91 PE=2 SV=2	−1,42	0,169853614862592	0,581421989337334	25,4982180151912	24,6663146621165	24,9825849920557	24,5199292078442	24,801127570362	24,7637322819112	75623976787,3416	53072762520,3409	0,510872072117895	0,0167636841861057	0,0061836130153585	0,70179809069793	-0.510872072117895	1,42491125760333	0.510872072117895	548
Q8BYI9-2	sp|Q8BYI9-2|TENR_MOUSE Isoform 2 of Tenascin-R OS=Mus musculus GN=Tnr;sp|Q8BYI9|TENR_MOUSE Tenascin-R OS=Mus musculus GN=Tnr PE=1 SV=2	−1,42	0,693866213199682	0,910309912045398	23,7216479337666	21,9088833413978	21,3020994376969	23,0095077948973	21,3795262247439	21,4835907437214	4892048184,0684	3435889179,51405	0,509755108884853	0,0564240337498439	0,0415581190272604	0,702341647145561	-0.509755108884853	1,42380848987693	0.509755108884853	549
Q8BHW2	sp|Q8BHW2|OSCP1_MOUSE Protein OSCP1 OS=Mus musculus GN=Oscp1 PE=2 SV=1	−1,42	0,0203607584777384	0,19874727468915	28,5376790926525	28,0588742128856	28,2458362215109	27,9313864300688	27,8216158205759	28,0318362877081	1915110633617,36	1346163098823,06	0,508574522293105	0,0085326215461185 6	0,0037648100195646	0,702916622775133	-0.508574522293105	1,42264383512795	0.508574522293105	550
Q8R3Q6	sp|Q8R3Q6|CCD58_MOUSE Coiled-coil domain-containing protein 58 OS=Mus musculus GN=Ccdc58 PE=2 SV=1	−1,42	0,0925953018788866	0,446167808097947	25,6315175429121	25,426892640985	25,3572525299008	24,7917929036	25,109689615665	25,4581618858169	115425088407,104	81175674845,103	0,507837459024249	0,0055968104952862 2	0,0132684296827551	0,703275829937393	-0.507837459024252	1,42191720151825	0.507837459024252	551
Q9D898	sp|Q9D898|ARP5L_MOUSE Actin-related protein 2/3 complex subunit 5-like protein OS=Mus musculus GN=Arpc5l PE=1 SV=1	−1,42	0,331510816280293	0,72953198881961	30,2152266398378	29,5962766892579	29,4120521281494	28,8732918283341	29,5194704291796	29,7759040277786	8249594408124,49	5803924417787,73	0,507294459021247	0,014146673623124	0,0158259943345167	0,703540577955181	-0.507294459021247	1,42138212255854	0.507294459021247	552
P35762	sp|P35762|CD81_MOUSE CD81 antigen OS=Mus musculus GN=Cd81 PE=1 SV=2	−1,42	0,0121056690659353	0,141656042925742	28,7707322599102	28,8212517944698	28,8313933089507	28,2826760859385	28,3564695698073	28,7307284512667	3243883874215,92	2283258493293,26	0,506627974483488	0,0011279298820707 8	0,0084420123471150	0,70386566900307	-0.506627974483492	1,42072563564062	0.506627974483492	553
Q9CQ92	sp|Q9CQ92|FIS1_MOUSE Mitochondrial fission 1 protein OS=Mus musculus GN=Fis1 PE=1 SV=1	−1,42	5,44×10^−07	4,41×10^−05	29,0635435904717	29,0088476558689	29,002551104323	28,5454840142889	28,7725746006029	28,7045051167192	4030779040708,29	2838190765052,01	0,506087138281565	0,0011557091605260 9	0,0040643291395541	0,704129582988325	-0.506087138281567	1,4201931351272	0.506087138281567	554
Q921J2	sp|Q921J2|RHEB_MOUSE GTP-binding protein Rheb OS=Mus musculus GN=Rheb PE=1 SV=1	−1,42	0,181819547540769	0,601053022412213	28,6623100861355	28,5094418456896	28,3325463587444	27,6705231091959	28,3097572274689	28,4722196117525	2387893621898,98	1681711794920,12	0,505808084079399	0,0057901541310768 7	0,0150552655872875	0,704265792871767	-0.505808084079399	1,41991845993588	0.505808084079399	555

Q9D404	sp|Q9D404|OXSM_MOUSE 3-oxoacyl-[acyl-carrier-protein] synthase, mitochondrial OS=Mus musculus GN=Oxsm PE=1 SV=1	−1,42	0,41663369216954	0,767877733621866	26,2087814532622	25,048560475253	25,4719455493022	25,3954169770848	25,5795222243761	24,7031955909906	128145113671,633	90267645298,1043	0,505497588798199	0,0229557075356153	0,018316904156853	0,704417380512937	-0.505497588798199	1,41961289948841	0.505497588798199	556
A2AQ07	sp|A2AQ07|TBB1_MOUSE Tubulin beta-1 chain OS=Mus musculus GN=Tubb1 PE=1 SV=1	−1,42	0,558968268428938	0,842909292491345	26,4316752166706	25,3115808292264	25,3211500311766	25,2133112519035	26,2070979591574	24,5934480971174	143289852891,339	100956183816,446	0,505207166365716	0,0250676887168561	0,032126255613555	0,704559197872889	-0.505207166365714	1,41932715237991	0.505207166365714	557
Q9DBT5	sp|Q9DBT5|AMPD2_MOUSE AMP deaminase 2 OS=Mus musculus GN=Ampd2 PE=1 SV=1	−1,42	0,0246177395846749	0,219211300821997	30,312297883722	30,1478585778955	29,9047847149208	29,7389386091483	29,9610005225932	29,6149568203859	12068828154133,7	8504631250467,39	0,504965012655601	0,00680630582811441	0,0058889328072952	0,7046774667642	-0.504965012655598	1,41908894091915	0.504965012655598	558
P70349	sp|P70349|HINT1_MOUSE Histidine triad nucleotide-binding protein 1 OS=Mus musculus GN=Hint1 PE=1 SV=3	−1,42	0,00133770372701354	0,0297639079260512	31,1804486408323	31,0315490815031	30,9004869372115	30,5433566106407	30,7635181652725	30,7592274156069	30158710007399,3	21278126380715,8	0,503203599165086	0,00451310673481317	0,0041021606912160	0,705538346152581	-0.503203599165088	1,41735740580674	0.503203599165088	559
Q60870	sp|Q60870|REEP5_MOUSE Receptor expression-enhancing protein 5 OS=Mus musculus GN=Reep5 PE=1 SV=1	−1,42	0,311070181089192	0,719371512809526	29,0045580088892	28,100354168705	28,0252316570059	27,7640796168976	28,0831095688452	28,2371702788332	2107902749506,71	1487502747651,72	0,502915974824339	0,0192067396863151	0,0086089085523634	0,705679020533478	-0.502915974824341	1,41707486109481	0.502915974824341	560
Q92562	sp|Q92562|FIG4_HUMAN Polyphosphoinositide phosphatase OS=Homo sapiens GN=FIG4 PE=1 SV=1;sp|Q91WF7|FIG4_MOUSE Polyphosphoinositide phosphatase OS=Mus musculus GN=Fig4 PE=1 SV=1	−1,41	0,227155590441094	0,652569129754509	24,018150513668	23,1166209487376	23,5787835721751	23,3535376645311	23,3379615440826	22,9811816841146	17251846385,3369	12194208516,8751	0,500554654213266	0,0191255897043532	0,0090692973443048	0,706834981282902	-0.500554654213267	1,41475737121132	0.500554654213267	561
Q99KV1	sp|Q99KV1|DJB11_MOUSE DnaJ homolog subfamily B member 11 OS=Mus musculus GN=Dnajb11 PE=1 SV=1	−1,41	0,17298126707304	0,586864499748362	28,3270681210585	27,8915107942788	28,6513548480099	27,6980271242862	28,0023313775896	28,1288847943823	1932775021483,05	1366236641303,96	0,50046632532324	0,0134774749734754	0,0079252348453708	0,706878258523656	-0.500466325323248	1,41467075545447	0.500466325323248	562
Q9EPB4	sp|Q9EPB4|ASC_MOUSE Apoptosis-associated speck-like protein containing a CARD OS=Mus musculus GN=Pycard PE=1 SV=1	−1,41	0,000209119705019239	0,0066079448501301	24,9476536404833	24,9418514401016	24,7822925530271	24,6867200139736	24,4263753482029	24,5185951445093	64543110104,8191	45632993593,9412	0,500185798003228	0,00377014514859836	0,0053782684641065	0,707015722047364	-0.500185798003226	1,41439570410714	0.500185798003226	563
Q9WUL7	sp|Q9WUL7|ARL3_MOUSE ADP-ribosylation factor-like protein 3 OS=Mus musculus GN=Arl3 PE=1 SV=1	−1,41	0,00660153646288249	0,0910375021402342	29,8880330606952	29,6792282244892	29,656852932955	29,1893852573842	29,4939123683025	29,5056687085734	8251131068140,5	5843330909673,65	0,497800906219787	0,00428710851793089	0,0060997068930505	0,708185442870503	-0.497800906219789	1,41205952489884	0.497800906219789	564
Q9WVJ5	sp|Q9WVJ5|CRBB1_MOUSE Beta-crystallin B1 OS=Mus musculus GN=Crybb1 PE=1 SV=3	−1,41	0,225875237698154	0,652238880554131	24,3684748551525	24,9238571579815	24,9689418598456	24,7436143644013	24,024166766318	24,4591970269996	56288605220,7402	39874095692,6346	0,497391099739757	0,0135100927653137	0,0148446019379419	0,708386635914413	-0.497391099739754	1,41165847758994	0.497391099739754	565
P63037	sp|P63037|DNJA1_MOUSE DnaJ homolog subfamily A member 1 OS=Mus musculus GN=Dnaja1 PE=1 SV=1	−1,41	1,09×10^−06	7,88×10^−05	29,8297582429479	29,6607221554744	29,8660763732758	29,4774744514319	29,4671293506286	29,3780373232367	8623558409073,12	6109587503305,51	0,497208325253302	0,00367937495483124	0,0018569054848890	0,708476386833238	-0.497208325253303	1,41147964644216	0.497208325253303	566
Q8BH82	sp|Q8BH82|NAPEP_MOUSE N-acyl-phosphatidylethanolamine-hydrolyzing phospholipase D OS=Mus musculus GN=Napepld PE=1 SV=1	−1,41	0,520343089577634	0,823916573667872	25,8525777820242	25,4860362621515	25,4779842302653	25,7291565919797	24,2210527725757	25,8337781042129	131929398704,657	93509526547,1947	0,496580829504136	0,00835698853045402	0,0357235042519435	0,708784603472114	-0.49658082950414	1,41086586122401	0.49658082950414	567
Q9QUI0	sp|Q9QUI0|RHOA_MOUSE Transforming protein RhoA OS=Mus musculus GN=Rhoa PE=1 SV=1	−1,41	0,00016346371306482	0,00553702831045685	31,4594116268809	31,3626131085233	31,5630720591258	30,9766739283493	31,1804486408323	31,1956272436472	46093770724089,4	32673428223401,2	0,496453956992357	0,00318638379318986	0,0039291880962047	0,708846937669291	-0.496453956992357	1,41074179326785	0.496453956992357	568
Q9Z1X4-2	sp|Q9Z1X4-2|ILF3_MOUSE Isoform 2 of Interleukin enhancer-binding factor 3 OS=Mus musculus GN=Ilf3;sp|Q9Z1X4|ILF3_MOUSE Interleukin enhancer-binding factor 3 OS=Mus musculus GN=Ilf3 PE=1 SV=2;sp|Q9Z1X4-3|ILF3_MOUSE Isoform 3 of Interleukin enhancer-bindi	−1,41	0,189820777842935	0,615865983294555	26,2659948811303	26,0128955673314	26,2266208709123	25,9923002404933	25,3323809620451	26,1491158481579	231652391640,064	164240723846,97	0,496149686345275	0,00520438970680518	0,0167833947069328	0,708996452331748	-0.496149686345278	1,41044429307255	0.496149686345278	569
O88910	sp|O88910|MPP3_MOUSE MAGUK p55 subfamily member 3 OS=Mus musculus GN=Mpp3 PE=1 SV=2	−1,41	0,212029632559143	0,64374749310097	26,8191926913355	26,984703803127	26,3465902358295	26,6737413947028	25,9984600276786	26,446728601505	400840291795,91	284209271329,128	0,496073914909066	0,0123945644111997	0,0130295790365486	0,709033690340278	-0.496073914909065	1,41037021741531	0.496073914909065	570
Q8CFP6	sp|Q8CFP6|DJC27_MOUSE DnaJ homolog subfamily C member 27 OS=Mus musculus GN=Dnajc27 PE=1 SV=1	−1,41	0,243406402358932	0,667588863741216	24,7917929036	25,2432823524486	25,5441223434088	25,2075881976355	24,5268886798457	24,8141487793	87339255995,8038	61946805975,7895	0,495600343659367	0,0150307336562145	0,0137518501107273	0,70926647209779	-0.495600343659366	1,40990733291299	0.495600343659366	571
Q9DC51	sp|Q9DC51|GNAI3_MOUSE Guanine nucleotide-binding protein G(k) subunit alpha OS=Mus musculus GN=Gnai3 PE=1 SV=3	−1,41	0,0131107364159751	0,149533351022612	26,7968413620251	26,3652422189801	26,4583956916079	26,1456344965372	26,2847000887215	26,1606183781872	335927015498,88	238344828469,224	0,49509750023345	0,0085575958701499	0,0029137947159490	0,709513726114769	-0.495097500233449	1,40941600309258	0.495097500233449	572
Q9D2M8	sp|Q9D2M8|UB2V2_MOUSE Ubiquitin-conjugating enzyme E2 variant 2 OS=Mus musculus GN=Ube2v2 PE=2 SV=4;sp|Q9D2M8-2|UB2V2_MOUSE Isoform 2 of Ubiquitin-conjugating enzyme E2 variant 2 OS=Mus musculus GN=Ube2v2	−1,41	0,160655988745951	0,567767985643373	27,8721796264246	27,333361039548	28,0173481149224	27,1500360627144	27,4718930142898	27,5736087892473	1116213117284,72	792543017580,892	0,494051356602935	0,0129908573792865	0,0080703394865031	0,710028403454725	-0.494051356602932	1,40839436159791	0.494051356602932	573
P01942	sp|P01942|HBA_MOUSE Hemoglobin subunit alpha OS=Mus musculus GN=Hba PE=1 SV=2	−1,41	0,00156307652085247	0,0327329861064157	35,5823748158424	35,5541042014945	35,505272310377	35,0044436776321	35,2475710661237	35,3634810211889	2741414553707570	1947193022843950	0,493524603697245	0,00109728619693306	0,0052048208159042	0,710287694435163	-0.493524603697244	1,40788022632889	0.493524603697244	574

Q9CQ48	sp|Q9CQ48|NUDC2_MOUSE NudC domain-containing protein 2 OS=Mus musculus GN=Nudcd2 PE=1 SV=1	−1,41	0,120438540378471	0,495438733051429	26,1186864653252	25,9353037091857	25,5351249444596	25,32730793297	25,7852122009926	25,4520364849735	170676941053,337	121298328507,499	0,492708489033414	0,0115383591621319	0,0092756057288065	0,710689609029218	-0.492708489033409	1,40708403119327	0.492708489033409	575
Q9CY50	sp|Q9CY50|SSRA_MOUSE Translocon-associated protein subunit alpha OS=Mus musculus GN=Ssr1 PE=1 SV=1	−1,41	0,535205766683608	0,8300431463043	22,080652069264	22,5116389434821	23,8999487036671	22,5521406244179	22,4108797662297	22,5052388583284	8227487933,19514	5848316913,2551	0,492430514112965	0,0416412559894887	0,0031991394754314	0,71082655614226	-0.492430514112967	1,40681294383136	0.492430514112966	576
Q9CQX0	sp|Q9CQX0|OTUB2_MOUSE Ubiquitin thioesterase OTUB2 OS=Mus musculus GN=Otub2 PE=2 SV=1	−1,41	0,276300294096601	0,698678013874357	23,5891400062898	23,732793378787	23,8157619160132	23,1581133693146	22,9811816841146	23,9753136992594	19871752747,4748	14129579176,0566	0,492000629926347	0,00483529050005636	0,022690740414585	0,711038394831685	-0.492000629926352	1,40639381398907	0.492000629926352	577
Q8BTJ4-2	sp|Q8BTJ4-2|ENPP4_MOUSE Isoform 2 of Bis(5-adenosyl)-triphosphatase enpp4 OS=Mus musculus GN=Enpp4;sp|Q8BTJ4|ENPP4_MOUSE Bis(5-adenosyl)-triphosphatase enpp4 OS=Mus musculus GN=Enpp4 PE=1 SV=1	−1,41	0,259656550806229	0,682211215860657	22,2860368944435	21,8445984352844	22,0488781458796	22,1623409962791	21,2173839147839	21,7767731508675	3805974193,05588	2706257949,87843	0,491966421354908	0,0100147249793418	0,0218766616332049	0,71105525487169 2	-0.491966421354907	1,40636046657225	0.491966421354907	578
Q9CYK1	sp|Q9CYK1|SYWM_MOUSE Tryptophan--tRNA ligase, mitochondrial OS=Mus musculus GN=Wars2 PE=2 SV=2	−1,41	0,165549635456501	0,574048735933582	25,0631252435222	24,4317373548227	24,875004184539	24,615571684966	24,6012207533644	24,1307029648063	58363445308,0555	41508544492,6254	0,491656706502719	0,013076755870075	0,0112842158282274	0,71120791916128 1	-0.491656706502721	1,40605858435785	0.491656706502721	579
Q8BSZ2	sp|Q8BSZ2|AP3S2_MOUSE AP-3 complex subunit sigma-2 OS=Mus musculus GN=Ap3s2 PE=1 SV=1	−1,41	0,16215560906623	0,569625464688209	26,7026272203015	26,6258838312392	26,377343207	25,8132134394717	26,2939048107703	26,5771116775476	345624266224,13	245871933697,463	0,491297451875423	0,00639984859463916	0,014723923404267	0,711385043601135	-0.491297451875422	1,40570849639719	0.491297451875422	580
Q8BXQ2	sp|Q8BXQ2|PIGT_MOUSE GPI transamidase component PIG-T OS=Mus musculus GN=Pigt PE=1 SV=2	−1,41	0,478639109631866	0,799905345588749	27,275090612165	27,6254440730023	27,5162976633754	26,2550580787104	27,860378365634	27,2799384547728	853215038610,659	606998182057,865	0,49121719892169	0,00652574001439956	0,0299592916367579	0,711424616994886	-0.49121719892169	1,40563030307284	0.49121719892169	581
Q80YQ8	sp|Q80YQ8|RMD5A_MOUSE Protein RMD5 homolog A OS=Mus musculus GN=Rmnd5a PE=2 SV=2	−1,40	0,0243445429324928	0,218641735494934	24,4468590871809	24,4263753482029	24,0624940540361	23,8119599107002	24,0997473766248	24,0050047487481	36184963496,9098	25763828405,2887	0,490043327942604	0,0088945209092453	0,0061181276716523	0,712003714125314	-0.490043327942604	1,4044870555605	0.490043327942604	582
Q91ZS8-5	sp|Q91ZS8-5|RED1_MOUSE Isoform 5 of Double-stranded RNA-specific editase 1 OS=Mus musculus GN=Adarb1;sp|Q91ZS8-4|RED1_MOUSE Isoform 4 of Double-stranded RNA-specific editase 1 OS=Mus musculus GN=Adarb1;sp|Q91ZS8-3|RED1_MOUSE Isoform 3 of Double-stranded	−1,40	0,1133196607395	0,481608558142876	23,4721841914717	22,8884318811075	22,9259100978125	22,7080917274642	22,6114053377269	22,9507064919501	10721412247,9947	7640543062,26579	0,488747864693284	0,0141476993360444	0,0076813519131657	0,712643342643116	-0.488747864693284	1,40322646710079	0.488747864693283	583
O35609	sp|O35609|SCAM3_MOUSE Secretory carrier-associated membrane protein 3 OS=Mus musculus GN=Scamp3 PE=1 SV=3	−1,40	0,388607427177129	0,753319093070654	26,6940235846991	26,1555786458727	26,7568583343213	25,8362365598065	26,882581127589	25,8727859475999	334360923231,758	238396528808,642	0,48804301999168	0,0124552750755201	0,0226679816645022	0,712991597536057	-0.488043019991678	1,40254107265188	0.488043019991678	584
O08848	sp|O08848|RO60_MOUSE 60 kDa SS-A/Ro ribonucleoprotein OS=Mus musculus GN=Trove2 PE=1 SV=1	−1,40	0,064838903074746	0,38006509013234	27,1149582581361	27,5736087892473	27,5713979478984	26,886722967848	27,1320858547966	27,2268519579805	809747519547,441	577449556153,356	0,487777220145989	0,00963407067662557	0,0064815696280819	0,713122970078483	-0.487777220145988	1,40228269451192	0.487777220145988	585
Q810A3	sp|Q810A3|TTC9C_MOUSE Tetratricopeptide repeat protein 9C OS=Mus musculus GN=Ttc9c PE=2 SV=1	−1,40	0,0307286204380981	0,249959154423884	26,251725356772	26,0401650290287	25,8205849296101	25,5637107751137	25,8813493021154	25,6547165375052	203207156657,89	144989264434,722	0,487005131126892	0,00827970092609669	0,0063647649628865	0,713504715184904	-0.487005131126898	1,40153243379877	0.487005131126898	586
O54879	sp|O54879|HMGB3_MOUSE High mobility group protein B3 OS=Mus musculus GN=Hmgb3 PE=2 SV=3;sp|O15347|HMGB3_HUMAN High mobility group protein B3 OS=Homo sapiens GN=HMGB3 PE=1 SV=4	−1,40	0,110762964079337	0,476995345309403	27,0534472647175	27,6170252356209	27,738528557757	27,1340590772315	27,1551566172011	27,1079700259264	850990684873,416	607364681058,038	0,486580323349256	0,0133070632382728	0,000871182055565	0,713714840660545	-0.486580323349257	1,40111980728115	0.486580323349257	587
P70677	sp|P70677|CASP3_MOUSE Caspase-3 OS=Mus musculus GN=Casp3 PE=1 SV=1	−1,40	0,0990439363979999	0,462035382641852	26,5664270729435	26,9359933509139	27,2571389591788	26,6691328676683	26,5421786038889	26,5380610554905	491070298034,362	350674463513,396	0,485797189168564	0,0128395460949151	0,0028030625140383	0,714102369695465	-0.485797189168561	1,40035944766079	0.485797189168562	588
P61148	sp|P61148|FGF1_MOUSE Fibroblast growth factor 1 OS=Mus musculus GN=Fgf1 PE=2 SV=1	−1,40	0,204156021368298	0,635648869851434	26,3073973295377	26,0949756141312	25,7852122009926	25,3191904935594	25,7958217310218	26,062710168417	208358998151,367	148805729652,42	0,485641327934453	0,0100757818681072	0,014641200695699	0,714179521751764	-0.485641327934456	1,40020816831483	0.485641327934456	589
P09528	sp|P09528|FRIH_MOUSE Ferritin heavy chain OS=Mus musculus GN=Fth1 PE=1 SV=2	−1,40	0,0018870910712419 5	0,0369928785341906	28,7408088025449	28,5192469795579	28,4258373734941	28,1252116374367	28,2371702788332	28,3139767888204	2536901030143,87	1812001047603,4	0,485483448450863	0,00566391268662038	0,0033631529505700	0,714257681349374	-0.485483448450866	1,40005494671167	0.485483448450866	590
Q68ED7	sp|Q68ED7|CRTC1_MOUSE CREB-regulated transcription coactivator 1 OS=Mus musculus GN=Crtc1 PE=1 SV=1	−1,40	0,650243905084537	0,890602620753987	27,6244221741423	26,5089478029815	26,5398052352229	26,8845106514255	25,3098334236589	27,4696095384182	477131706565,652	340830527709,043	0,485332998603504	0,0236249173292975	0,0420654524761063	0,714332170801032	-0.485332998603501	1,39990895115172	0.485332998603501	591
Q9JKF6	sp|Q9JKF6|PVRL1_MOUSE Nectin-1 OS=Mus musculus GN=Pvrl1 PE=1 SV=3	−1,40	0,0727233566267059	0,402678673043606	28,0940222760243	27,8064691473181	27,4646552402382	27,5376339052084	27,4305707381541	27,3890281684233	1170418181768,27	836431894849,45	0,484704105209246	0,0113383037044499	0,0027929216535185	0,714643627276677	-0.484704105209244	1,39929884187276	0.484704105209244	592
Q8BTZ7	sp|Q8BTZ7|GMPPB_MOUSE Mannose-1-phosphate guanyltransferase beta OS=Mus musculus GN=Gmppb PE=2 SV=1	−1,40	0,034275906230123 6	0,269633168766951	29,002551104323	28,5904583519206	28,7339849515457	28,4357489471046	28,62011627812	28,2648305123125	3141321862545,61	2246134629875,81	0,483927367075348	0,00726948171692374	0,0062476741624806	0,715028490603515	-0.48392736707535	1,39854567075496	0.48392736707535	593
Q9JHI5	sp|Q9JHI5|IVD_MOUSE Isovaleryl-CoA dehydrogenase, mitochondrial OS=Mus musculus GN=Ivd PE=1 SV=1	−1,40	0,0148120274467975	0,162395634253656	30,7195270753918	30,295446665439	30,4606427564896	30,192477358805	30,239302803755	30,038498203068	17476393891734,1	12500121187744,2	0,483465445669732	0,00701032893475951	0,0034837715733631	0,715257464736844	-0.483465445669726	1,39809795675172	0.483465445669726	594

Q8BHF7	sp|Q8BHF7|PGPS1_MOUSE CDP-diacylglycerol--glycerol-3-phosphate 3-phosphatidyltransferase, mitochondrial OS=Mus musculus GN=Pgs1 PE=2 SV=1	−1,40	0,0410296163667918	0,298930062100912	26,1062400879502	26,0840270683502	26,1869977779283	25,4719455493022	25,9896298932137	25,9104073439626	221958214488,172	158760232419,24	0,483438523084479	0,0020741309259519	0,0108117995400622	0,715270812505568	-0.483438523084479	1,39807186665011	0.483438523084479	595
B9EHT4	sp|B9EHT4|CLIP3_MOUSE CAP-Gly domain-containing linker protein 3 OS=Mus musculus GN=Clip3 PE=1 SV=1	−1,40	0,461590430053346	0,793320318823225	24,7872986927083	25,6369206672453	24,8485211097813	24,4331596823421	25,4815166293187	24,3532110414183	78857912170,9492	56412829953,5495	0,483232202740389	0,01885158840953	0,0254330088119114	0,71537311096009	-0.483232202740388	1,39787194217842	0.483232202740388	596
Q9CY45	sp|Q9CY45|N6MT2_MOUSE N(6)-adenine-specific DNA methyltransferase 2 OS=Mus musculus GN=N6amt2 PE=2 SV=1	−1,40	0,228243996415952	0,652923723647267	24,0390384667258	23,6863570906192	23,1009237110647	23,3607684831536	23,2704101887496	23,1903792338134	17912651529,0919	12814621174,6745	0,483188078411435	0,020070725687824	0,0036627700602705	0,715394990733912	-0.483188078411429	1,39782918940223	0.483188078411429	597
Q9JJU8	sp|Q9JJU8|SH3L1_MOUSE SH3 domain-binding glutamic acid-rich-like protein OS=Mus musculus GN=Sh3bgrl PE=3 SV=1	−1,40	0,0912161851038526	0,44519383245848	29,3473309859604	29,1565083052916	28,9750635875556	28,5094418456896	28,8941100043926	29,0726137546512	4611776667465,04	3301468663400,56	0,482214697540438	0,00638394101061555	0,0099844823972936	0,715877827886076	-0.482214697540436	1,39688639743587	0.482214697540436	598
Q8BUV3	sp|Q8BUV3|GEPH_MOUSE Gephyrin OS=Mus musculus GN=Gphn PE=1 SV=2	−1,40	0,42679474213357	0,773341850117474	31,3009254761233	32,20118575714	30,766243128978	31,1636297730172	31,0706333494719	31,0315490815031	44334522898258	31740166221882,4	0,482120866662641	0,0230785164085498	0,0021824515527887	0,715924389097904	-0.482120866662639	1,39679554884287	0.482120866662639	599
Q8C0D4	sp|Q8C0D4|RHG12_MOUSE Rho GTPase-activating protein 12 OS=Mus musculus GN=Arhgap12 PE=1 SV=2	−1,40	0,172931210270486	0,586864499748362	25,4159343606792	24,9183070413901	24,7090851139904	24,7940826638887	24,8203758830295	24,4263753482029	73052352019,7141	52300824098,8008	0,482097044251966	0,0145155499408415	0,0089252954317169	0,715936210851729	-0.482097044251967	1,39677248453508	0.482097044251967	600
Q60749	sp|Q60749|KHDR1_MOUSE KH domain-containing, RNA-binding, signal transduction-associated protein 1 OS=Mus musculus GN=Khdrbs1 PE=1 SV=2	−1,40	0,324265875488172	0,726844368394785	28,1529200921064	29,0218456153508	28,7010859141301	28,5376790926525	28,4931602800957	27,8436592712793	2702731039581,01	1935718222593,78	0,481548991634967	0,0153498670827212	0,0137313081973734	0,716208233170647	-0.481548991634969	1,39624197780164	0.481548991634969	601
P50580	sp|P50580|PA2G4_MOUSE Proliferation-associated protein 2G4 OS=Mus musculus GN=Pa2g4 PE=1 SV=3;sp|Q9UQ80|PA2G4_HUMAN Proliferation-associated protein 2G4 OS=Homo sapiens GN=PA2G4 PE=1 SV=3;sp|P50580-2|PA2G4_MOUSE Isoform 2 of Proliferation-associated pro	−1,40	7,06×10^−07	5,25×10^−05	30,1229682803372	29,9085130255149	30,0650549662063	29,6593636472282	29,7293035998322	29,7066894223823	11035944655362,4	7904491223361,14	0,481465615910931	0,00369414487739884	0,0012017516629168	0,71624962522083	-0.481465615910932	1,39616128900826	0.481465615910932	602
P62717	sp|P62717|RL18A_MOUSE 60S ribosomal protein L18a OS=Mus musculus GN=Rpl18a PE=1 SV=1	−1,40	0,0461977021505435	0,313909236618738	26,223070070365	26,0218395960174	25,9556095100875	25,7136461877525	25,9967084867646	25,4898256936456	209259244696,367	149923868078,506	0,481061279317882	0,00534368667532378	0,0098711322703730	0,716450392889662	-0.481061279317885	1,39577004901441	0.481061279317885	603
Q9R0K7	sp|Q9R0K7|AT2B2_MOUSE Plasma membrane calcium-transporting ATPase 2 OS=Mus musculus GN=Atp2b2 PE=1 SV=2	−1,40	0,187257695352527	0,61192417509368	26,6015089080294	26,0926202587236	26,0401650290287	25,8825371944796	26,2339091105729	25,6181538792891	250009494459,93	179157903212,317	0,480751197570357	0,0118141958077568	0,0119213323584842	0,716604397762307	-0.480751197570358	1,39547008519991	0.480751197570359	604
P70236	sp|P70236|MP2K6_MOUSE Dual specificity mitogen-activated protein kinase kinase 6 OS=Mus musculus GN=Map2k6 PE=1 SV=1	−1,39	0,0492581334013988	0,324503145876559	28,2774605455253	27,8373225183337	28,0535845608097	27,5185848493879	27,7477528357763	27,9035914445976	1529745567199,62	1096681272042,12	0,48014742174496	0,00784427673925352	0,0069855900322026	0,71690436341628	-0.480147421744971	1,3948861954678	0.480147421744971	605
Q8CAK1	sp|Q8CAK1|CAF17_MOUSE Putative transferase CAF17 homolog, mitochondrial OS=Mus musculus GN=Iba57 PE=1 SV=1	−1,39	0,129512003019867	0,512962462222669	26,3327598269784	26,4120453633107	27,0009567310953	26,2191523428398	26,1649342395901	26,3652422189801	350252668319,016	251265570650,222	0,479183040254854	0,0137331975395173	0,0039469264829061	0,717383744301314	-0.479183040254855	1,39395408377135	0.479183040254855	606
Q9Z2N8	sp|Q9Z2N8|ACL6A_MOUSE Actin-like protein 6A OS=Mus musculus GN=Actl6a PE=1 SV=2	−1,39	0,0329614172096112	0,264331930590857	26,1107739160253	25,7149021750404	25,6338439500692	25,462854440618	25,5847097988631	25,4159343606792	163460958642,953	117280327403,452	0,478985064504362	0,00988368030500894	0,0034179515539949	0,717482194996953	-0.478985064504365	1,39376280968791	0.478985064504365	607
P62748	sp|P62748|HPCL1_MOUSE Hippocalcin-like protein 1 OS=Mus musculus GN=Hpcal1 PE=2 SV=2	−1,39	0,082259824103159	0,427205196343523	28,9271249389734	28,6623100861355	28,5243734839213	28,1083623316671	28,3886961162417	28,624551630105	2925840296890,84	2101915159992,12	0,47714658533508	0,00713054575888721	0,0091074580246665	0,718397091675076	-0.477146585335082	1,39198781786312	0.477146585335082	608
Q8BG95	sp|Q8BG95|MYPT2_MOUSE Protein phosphatase 1 regulatory subunit 12B OS=Mus musculus GN=Ppp1r12b PE=1 SV=2	−1,39	0,423333938509207	0,7711031271846	23,6905689153765	24,4583038673233	24,4288410238104	24,2127444651752	24,1659420657239	23,2077788046151	32114429038,2896	23078219060,1668	0,476689751131534	0,0179805735399399	0,0237694145392922	0,718624610534129	-0.47668975113153	1,39154711005059	0.47668975113153	609
P28658	sp|P28658|ATX10_MOUSE Ataxin-10 OS=Mus musculus GN=Atxn10 PE=1 SV=2	−1,39	0,00685818262102469	0,0927193592980021	30,4732906815785	30,1096724551239	30,3721900067996	29,9739816452622	30,0920533297203	29,9023714560052	14692883563505,1	10574523812849,5	0,474524862919262	0,00619048406439205	0,0031939417079720	0,719703778168842	-0.474524862919262	1,38946053964636	0.474524862919262	610
Q9JMK2	sp|Q9JMK2|KC1E_MOUSE Casein kinase I isoform epsilon OS=Mus musculus GN=Csnk1e PE=1 SV=2	−1,39	0,103832663384374	0,465707963539203	27,6483028203886	27,2232737570987	27,4758239742999	26,8719382927359	27,4194342735147	27,0697573276934	833695043584,41	600108693335,284	0,474295929014929	0,00778778286722335	0,0102224176218833	0,719817993345818	-0.474295929014929	1,38924007074602	0.474295929014929	611
P26645	sp|P26645|MARCS_MOUSE Myristoylated alanine-rich C-kinase substrate OS=Mus musculus GN=Marcks PE=1 SV=2	−1,39	0,160221081906886	0,567272613709327	31,0862260035438	31,8752640211253	31,6276420584925	31,2250526281598	31,2180758996145	31,1607157709667	49337753470561,3	35525839927573,2	0,473823266810692	0,0127984716421357	0,0011314810800532	0,720053861973481	-0.473823266810694	1,38878499624911	0.473823266810694	612
Q9D1M4	sp|Q9D1M4|MCA3_MOUSE Eukaryotic translation elongation factor 1 epsilon-1 OS=Mus musculus GN=Eef1e1 PE=2 SV=1	−1,39	0,172992967152364	0,586864499748362	28,2856963956054	28,0355976856729	27,7704056984658	27,3252327797371	27,8568791263886	27,924640903582	1491146783556,19	1073827985347,65	0,473659369736703	0,00919289969614947	0,0118495953719712	0,720135668191371	-0.473659369736708	1,38862723257621	0.473659369736708	613

Q61411	sp|Q61411|RASH_MOUSE GTPase HRas OS=Mus musculus GN=Hras PE=1 SV=2;sp|Q61411-2|RASH_MOUSE Isoform 2 of GTPase HRas OS=Mus musculus GN=Hras;sp|P23175|RASH_MSVNS GTPase HRas OS=Murine sarcoma virus NS.C58 GN=H-RAS PE=3 SV=1;sp|P01113|RASH_MSVMO GTPase HR	−1,39	0,0363675258081443	0,279309982475748	29,9490951305956	29,6461657797128	29,6117382906239	29,2041525384792	29,4203370103753	29,5991882691668	8204192338242,28	5911333793169,15	0,472877627575244	0,00624282406935477	0,0067264821366954	0,720525988355318	-0.472877627575242	1,38787499154973	0.472877627575242	614
P63028	sp|P63028|TCTP_MOUSE Translationally-controlled tumor protein OS=Mus musculus GN=Tpt1 PE=1 SV=1	−1,39	0,00946835906686627	0,118393613786518	31,1488485026459	31,0751300491871	30,8553810226389	30,6103162203833	30,8796473041202	30,6061730946971	29827538441537	21492221747111,8	0,472830293886123	0,0049206122266605	0,0051047119780342	0,720549628633864	-0.472830293886122	1,38782945721027	0.472830293886122	615
A2RSX7	sp|A2RSX7|TYW5_MOUSE tRNA wybutosine-synthesizing protein 5 OS=Mus musculus GN=Tyw5 PE=2 SV=2	−1,39	0,582822748714537	0,853640676481215	22,3388119487222	23,8445866552698	23,4148105713821	22,4950198036394	22,4633145269286	23,6580266549454	11895226932,0805	8575029999,7418	0,472169171472612	0,0334342372458625	0,029765471057511	0,720879899871066	-0.472169171472609	1,38719362293061	0.472169171472609	616
Q50H33-2	sp|Q50H33-2|KCTD8_MOUSE Isoform 2 of BTB/POZ domain-containing protein KCTD8 OS=Mus musculus GN=Kctd8;sp|Q50H33|KCTD8_MOUSE BTB/POZ domain-containing protein KCTD8 OS=Mus musculus GN=Kctd8 PE=1 SV=1	−1,39	0,0116779978187341	0,137322362951383	23,19435933754	23,2969409758378	23,493812176176	22,8730078302888	22,9428230979447	23,1878192907474	13532646059,9819	9756667551,38376	0,471983583525047	0,00652340499556728	0,0071878698437727	0,720972639655131	-0.471983583525048	1,38701518614956	0.471983583525048	617
Q68EF0	sp|Q68EF0|RAB3I_MOUSE Rab-3A-interacting protein OS=Mus musculus GN=Rab3ip PE=1 SV=1	−1,39	0,387651611145704	0,752909997000577	25,6510636985623	26,0234593655428	25,8662283570416	24,8260555298346	26,0481993746539	25,6863781120229	167947478648,983	121139790065,117	0,47133732061714	0,00723286982363561	0,024599632120995	0,721295675526657	-0.471337320617145	1,38639400446959	0.471337320617145	618
P61028	sp|P61028|RAB8B_MOUSE Ras-related protein Rab-8B OS=Mus musculus GN=Rab8b PE=1 SV=1	−1,39	0,143816840195841	0,538157162117825	27,0584803792591	26,8777286255423	26,8427056892879	26,1971184130789	26,6843310444171	26,9195750813523	494248813323,307	356764420000,166	0,470265759166774	0,0043005919329078	0,013852406800412	0,721831616754521	-0.470265759166774	1,38536464292901	0.470265759166774	619
Q8BVG8	sp|Q8BVG8|NAT14_MOUSE N-acetyltransferase 14 OS=Mus musculus GN=Nat14 PE=2 SV=1	−1,39	0,225800142167966	0,652238880554131	25,2191352268015	25,500765153124	25,347826740016	25,4007405161479	24,53802616775	25,1516291000811	102785388019,911	74207564398,3481	0,469997023899539	0,00556039291729709	0,0177388082810476	0,721966087085968	-0.46999702389954	1,38510661080529	0.469997023899539	620
O70274	sp|O70274|TP4A2_MOUSE Protein tyrosine phosphatase type IVA 2 OS=Mus musculus GN=Ptp4a2 PE=1 SV=1	−1,38	0,065374591269751	0,381898674097039	26,9868342577459	26,5664270729435	26,4706027605894	26,1961885123238	26,4513611202461	26,3998169118976	384273879725,565	277509826825,358	0,469596056074932	0,0102942983093093	0,0051208012253233	0,722166770803016	-0.469596056074932	1,38472170200804	0.469596056074932	621
Q60571	sp|Q60571|CRHBP_MOUSE Corticotropin-releasing factor-binding protein OS=Mus musculus GN=Crhbp PE=2 SV=1	−1,38	0,675315543318675	0,901266728168293	21,3956711244922	22,7962312002305	23,1722651329508	22,0882083383777	21,1880773735381	23,1133185411363	5648876348,14197	4082061977,54427	0,46866583411344	0,0416945077913064	0,0435292447478475	0,722632560170474	-0.468665834113438	1,38382914792006	0.468665834113438	622
Q8BYM5	sp|Q8BYM5|NLGN3_MOUSE Neuroligin-3 OS=Mus musculus GN=Nlgn3 PE=1 SV=2	−1,38	0,352091006442378	0,732843629012038	28,0758284429146	27,4149626112445	27,4525390811037	27,4732502744015	27,7067011958313	26,7900481326491	1016896758138,91	735144709872,714	0,468073044070531	0,013425923969287	0,0174342104678816	0,72292954421268	-0.468073044070531	1,38326066212866	0.468073044070531	623
P28474	sp|P28474|ADHX_MOUSE Alcohol dehydrogenase class-3 OS=Mus musculus GN=Adh5 PE=1 SV=3	−1,38	0,00283448394398944	0,0508474448240013	30,3808159827936	30,0876771272862	30,31031921555	29,9407135560488	30,0419786001201	29,8229827561781	13854124589780,7	10016200727619,8	0,467980173415555	0,00505652819356676	0,0036612733596914	0,722976082877738	-0.467980173415552	1,38317162031086	0.467980173415552	624
Q9JKW0	sp|Q9JKW0|AR6P1_MOUSE ADP-ribosylation factor-like protein 6-interacting protein 1 OS=Mus musculus GN=Arl6ip1 PE=2 SV=1	−1,38	0,420321164560374	0,769672248783603	27,333361039548	27,2162114865181	27,2818929651463	26,1622102512306	27,4591263032037	27,2381962259367	701969058463,104	507710679357,037	0,467400834002837	0,00215265507431469	0,0257427022427261	0,723266464861888	-0.467400834002837	1,38261629507592	0.467400834002837	625
Q9Z2Q5	sp|Q9Z2Q5|RM40_MOUSE 39S ribosomal protein L40, mitochondrial OS=Mus musculus GN=Mrpl40 PE=2 SV=2	−1,38	0,422881147595672	0,7711031271846	24,5308608808026	24,8771377433071	24,4804791910647	23,5647953275257	24,5032761075892	24,8499747977413	49711026422,9316	35972313531,641	0,466678943777502	0,00876759211398611	0,0273557825601928	0,723628460728122	-0.466678943777502	1,38192463988189	0.466678943777502	626
Q9JJR9	sp|Q9JJR9|NRIP3_MOUSE Nuclear receptor-interacting protein 3 OS=Mus musculus GN=Nrip3 PE=2 SV=1	−1,38	0,234669710394645	0,657510075193589	24,9190543270454	25,5223313428076	25,7127945872499	25,2931187432148	24,846359962555	25,0452755773747	105790520321,2	76578696470,363	0,46619534837958	0,016323817071952	0,0089310184920813	0,723871063662941	-0.466195348379579	1,38146149252021	0.466195348379579	627
Q61191	sp|Q61191|HCFC1_MOUSE Host cell factor 1 OS=Mus musculus GN=Hcfc1 PE=1 SV=2	−1,38	0,5742475233635	0,849998193752035	25,0588842328911	23,5344531294744	24,6240536772299	24,5712773674685	24,2779559835035	23,4012908958398	39746873195,2668	28796165770,9043	0,464964642382995	0,0321768712224952	0,0252765654415554	0,724488832855749	-0.464964642382998	1,38028352494856	0.464964642382998	628
Q8VDP6	sp|Q8VDP6|CDIPT_MOUSE CDP-diacylglycerol--inositol 3-phosphatidyltransferase OS=Mus musculus GN=Cdipt PE=1 SV=1	−1,38	0,205323859032183	0,636399775345126	28,8639896558021	28,6410826370046	28,5963004492755	28,01920864903	28,2826760859385	28,8343162844237	2913737043741,31	2112163034440,84	0,464149485977007	0,00499581101977997	0,0146573566453828	0,724898301642474	-0.464149485977007	1,37950385279452	0.464149485977007	629
Q8K3C3	sp|Q8K3C3|LZIC_MOUSE Protein LZIC OS=Mus musculus GN=Lzic PE=1 SV=1	−1,38	0,26280290708412	0,682806179310143	27,7259995969464	27,3125156226977	27,3269456084881	26,6322995250514	27,333361039548	27,436658796006	838729102087,041	608404901413,995	0,463173139625158	0,00854740966149715	0,0161286306439456	0,725389043852274	-0.463173139625157	1,3785705870182	0.463173139625157	630
Q8VE95	sp|Q8VE95|CH082_MOUSE UPF0598 protein C8orf82 homolog OS=Mus musculus PE=2 SV=1	−1,38	0,0566615883344242	0,352794169341497	25,3783577930722	25,6281918452435	25,5740351178429	24,9067459177116	25,3797969498621	25,3314691252537	121948124884,123	88476604458,3495	0,462899650717507	0,00514860836136396	0,0103264743414145	0,725526567484505	-0.462899650717507	1,37830927882783	0.462899650717507	631
Q9Z1P7	sp|Q9Z1P7|KANK3_MOUSE KN motif and ankyrin repeat domain-containing protein 3 OS=Mus musculus GN=Kank3 PE=2 SV=1	−1,38	0,137229577946759	0,527876076506694	24,0965663332202	24,3120591899788	24,0297670368369	23,5244400838048	24,1996408868827	23,7519013740161	30657122478,0469	22243762034,1332	0,462821481653605	0,00610982476704022	0,0144189773743323	0,72556587951337	-0.46282148165361	1,37823460037935	0.46282148165361	632
Q8K2Q7	sp|Q8K2Q7|BROX_MOUSE BRO1 domain-containing protein BROX OS=Mus musculus GN=Brox PE=2 SV=1	−1,38	0,151400261976259	0,552437900049208	28,1750053595332	27,5509487648623	27,6170252356209	27,3125156226977	27,6606396741905	27,4078116367401	1161801716561,53	843075465585,831	0,462630185607902	0,0123401024348669	0,0065514331658219	0,725662093253742	-0.462630185607904	1,37805186366588	0.462630185607904	633

Q9QXA5	sp|Q9QXA5|LSM4_MOUSE U6 snRNA-associated Sm-like protein LSm4 OS=Mus musculus GN=Lsm4 PE=2 SV=1;sp|F4K4E3|LSM4_ARATH Sm-like protein LSM4 OS=Arabidopsis thaliana GN=LSM4 PE=1 SV=1	−1,38	0,150565485689346	0,551142531890274	26,6864288116258	26,2003292010332	26,0820246055589	25,7876482760269	26,0003863356302	26,2191523428398	270334938310,409	196199071470,255	0,462429765130199	0,0121685018419446	0,0082976604743878	0,725762909879491	-0.4624297651302	1,37786043677273	0.4624297651302	634
P68037	sp|P68037|UB2L3_MOUSE Ubiquitin-conjugating enzyme E2 L3 OS=Mus musculus GN=Ube2l3 PE=2 SV=1	−1,38	0,126585797746903	0,509135279119877	30,2310954123744	30,108137787763	29,6815604610594	29,4746082875131	29,6715275253764	29,9143750348548	10760802185068,5	7813209549852,62	0,461798417606294	0,00961138331910225	0,0074202156336959	0,726080585394841	-0.461798417606291	1,37725759387466	0.461798417606291	635
Q99JY0	sp|Q99JY0|ECHB_MOUSE Trifunctional enzyme subunit beta, mitochondrial OS=Mus musculus GN=Hadhb PE=1 SV=1	−1,38	0,0107096991916484	0,128601387912413	30,4899709322161	30,1381873875547	30,2654579925308	29,9373485722038	30,1173421092281	29,8788419232647	14394569079282,9	10452310825974,1	0,461702667933391	0,00587888540858149	0,0041463977942037	0,726128775957412	-0.461702667933394	1,37716619022774	0.461702667933394	636
Q9CQN6	sp|Q9CQN6|TM14C_MOUSE Transmembrane protein 14C OS=Mus musculus GN=Tmem14c PE=1 SV=1	−1,38	0,131505221392816	0,515905099310279	23,9912209416627	24,0770833167058	24,1736455229619	23,8645191979705	24,0531878454148	23,3644875152294	28713979367,7993	20852329791,2916	0,461544700093102	0,00378995477460889	0,0149779710757464	0,726208287753944	-0.461544700093104	1,37701540572176	0.461544700093104	637
P01027	sp|P01027|CO3_MOUSE Complement C3 OS=Mus musculus GN=C3 PE=1 SV=3	−1,38	0,14874807279596	0,547137360146565	26,9547500236637	27,4198860408206	27,4149626112445	27,1925405628277	26,6482374292033	26,9898009594195	692240695930,932	502834193316,225	0,461191000110297	0,00979842719039201	0,0102097370069218	0,726386351267616	-0.461191000110295	1,37667784954233	0.461191000110296	638
Q9DCT5	sp|Q9DCT5|SDF2_MOUSE Stromal cell-derived factor 2 OS=Mus musculus GN=Sdf2 PE=2 SV=1	−1,37	0,656030941713079	0,892226633215307	25,9276015798895	25,4336098486706	26,5613075057639	26,4042232161596	24,4006943482069	26,1671183698062	190739217397,451	138945181055,234	0,457085703589175	0,0217635210092121	0,0426673071876399	0,728456281571651	-0.457085703589181	1,37276597827187	0.457085703589181	639
Q8BNE1-2	sp|Q8BNE1-2|F115A_MOUSE Isoform 2 of Protein FAM115A OS=Mus musculus GN=Fam115a;sp|Q8BNE1|F115A_MOUSE Protein FAM115A OS=Mus musculus GN=Fam115a PE=2 SV=1;sp|Q8BNE1-3|F115A_MOUSE Isoform 3 of Protein FAM115A OS=Mus musculus GN=Fam115a	−1,37	0,0514690973497919	0,334319560904408	24,4971412211746	24,1881015331326	24,0659982136174	24,1023003631839	23,9424348342287	23,7584773744894	34026778549,9362	24807309649,8386	0,455905288521255	0,00916368912944874	0,0071884854014450	0,729052549404067	-0.455905288521258	1,37164323863541	0.455905288521257	640
Q90694	sp|Q90694|CDC42_CHICK Cell division control protein 42 homolog OS=Gallus gallus GN=CDC42 PE=2 SV=1;sp|P60766|CDC42_MOUSE Cell division control protein 42 homolog OS=Mus musculus GN=Cdc42 PE=1 SV=2	−1,37	0,0601671311259902	0,36316304279371	27,5285613999933	27,1900125023832	27,1009588259087	26,7912227971815	26,9252720505903	27,1551566172011	699182460653,296	509765755897,247	0,455834532644106	0,00827210334539909	0,0068277795297969	0,72908830610673	-0.455834532644106	1,37157596908928	0.455834532644106	641
Q9C5W6	sp|Q9C5W6|14312_ARATH 14-3-3-like protein GF14 iota OS=Arabidopsis thaliana GN=GRF12 PE=2 SV=1	−1,37	0,285470299228382	0,704836107321858	30,9382317000035	31,5630720591258	31,5283219649286	31,2118339601665	31,271198233726	30,6034495341565	40943270357398,2	29898434667073,9	0,453556388628782	0,0112033962890569	0,0119109160827262	0,730240510982324	-0.453556388628785	1,36941183755307	0.453556388628785	642
Q9DAM7	sp|Q9DAM7|TM263_MOUSE Transmembrane protein 263 OS=Mus musculus GN=Tmem263 PE=2 SV=1	−1,37	0,352279866543076	0,732843629012038	25,2526747161242	25,2357243145929	24,6675378862814	24,1908952126927	24,9238571579815	25,0980650300174	75846619409,8808	55386726062,495	0,453544616380485	0,0132941035071399	0,0194606905155952	0,730246469696705	-0.453544616380486	1,36940066333403	0.453544616380486	643
Q3TC93	sp|Q3TC93|H1BP3_MOUSE HCLS1-binding protein 3 OS=Mus musculus GN=Hs1bp3 PE=1 SV=2	−1,37	0,168430909045247	0,577859331939817	25,4876194191801	24,9190543270454	25,1215085600284	24,8971877540398	25,1125314478412	24,5790747392146	85866600226,7404	62781815959,0032	0,451750311960875	0,0114466441000271	0,0107941069999012	0,731155254699974	-0.451750311960876	1,36769857505893	0.451750311960876	644
Q8BTS4	sp|Q8BTS4|NUP54_MOUSE Nuclear pore complex protein Nup54 OS=Mus musculus GN=Nup54 PE=1 SV=1	−1,37	0,11298260713029	0,481076973512557	25,6889101757867	26,133625476344	26,0701199334524	25,3937401987218	25,8761040310522	25,6844554864885	188849932471,561	138126150521,918	0,451253786419258	0,00926386885260775	0,0094681456399755	0,731406936259924	-0.451253786419259	1,36722794168939	0.451253786419259	645
P62838	sp|P62838|UB2D2_MOUSE Ubiquitin-conjugating enzyme E2 D2 OS=Mus musculus GN=Ube2d2 PE=1 SV=1;sp|P61079|UB2D3_MOUSE Ubiquitin-conjugating enzyme E2 D3 OS=Mus musculus GN=Ube2d3 PE=1 SV=1	−1,37	0,154403972697975	0,557993337640214	29,5724244467374	29,709391036643	29,7759040277786	28,9936791154053	29,4054016596301	29,7220781608845	7805943164976,11	5712738634147,95	0,450390432463181	0,0034950161469523	0,012433957342757	0,731844764099744	-0.450390432463181	1,36640999437923	0.450390432463181	646
P42232	sp|P42232|STA5B_MOUSE Signal transducer and activator of transcription 5B OS=Mus musculus GN=Stat5b PE=1 SV=1;sp|P42230|STA5A_MOUSE Signal transducer and activator of transcription 5A OS=Mus musculus GN=Stat5a PE=1 SV=1;sp|Q9TUM3|STA5B_BOVIN Signal tran	−1,37	0,00341917601899081	0,0574801479637011	27,064290140851	26,8410458836077	26,7099050263889	26,6065658100871	26,5548176288872	26,517535165276	468006029790,697	342534950517,889	0,45027591679296	0,00666782965959552	0,0016834098744388	0,731902857471897	-0.450275916792961	1,36630153823166	0.450275916792961	647
P61089	sp|P61089|UBE2N_MOUSE Ubiquitin-conjugating enzyme E2 N OS=Mus musculus GN=Ube2n PE=1 SV=1	−1,37	0,266821276495411	0,685404159524725	32,0014621620972	31,9095496846577	31,6995393111186	31,2799527489482	31,3069394690914	32,0900263529684	69349754265769,1	50802813711596,8	0,448982367694383	0,00485604610575545	0,0145791728176511	0,732559390432807	-0.448982367694382	1,36507703410803	0.448982367694382	648
Q9JIQ3	sp|Q9JIQ3|DBLOH_MOUSE Diablo homolog, mitochondrial OS=Mus musculus GN=Diablo PE=1 SV=2	−1,36	0,225959977987549	0,652238880554131	25,2783415939017	24,9148170158252	24,4925993676552	24,3532110414183	24,7396961746093	24,6631481372109	64844158018,641	47564464935,1933	0,447092455117757	0,0157956338696079	0,0083240892764174	0,733519662966705	-0.447092455117757	1,36328997092664	0.447092455117757	649
Q8C726	sp|Q8C726|BTBD9_MOUSE BTB/POZ domain-containing protein 9 OS=Mus musculus GN=Btbd9 PE=2 SV=1	−1,36	0,457067002267576	0,790936760691012	21,7927037897341	22,2911985568517	22,3211019379397	22,3165440216705	21,0792559200743	22,080652069264	4103070480,99042	3010837161,16366	0,446539253402933	0,0134093181276877	0,0300990956544748	0,733800985167793	-0.44653925340293	1,3627673173147	0.44653925340293	650

Q61187	sp|Q61187|TS101_MOUSE Tumor susceptibility gene 101 protein OS=Mus musculus GN=Tsg101 PE=1 SV=2;sp|Q99816|TS101_HUMAN Tumor susceptibility gene 101 protein OS=Homo sapiens GN=TSG101 PE=1 SV=2;sp|Q99816-2|TS101_HUMAN Isoform 2 of Tumor susceptibility gen	−1,36	0,0774194528911564	0,415866149908827	29,4671293506286	29,0568893313668	29,1365569873341	29,119788155716	28,9160873320475	28,6988938908549	4899684296954,56	3598685633333,23	0,445218714810916	0,00744458948525946	0,0072802352026463	0,734472960955877	-0.445218714810916	1,3615205094801	0.445218714810916	651
Q8R123	sp|Q8R123|FAD1_MOUSE FAD synthase OS=Mus musculus GN=Flad1 PE=1 SV=1;sp|Q8R123-2|FAD1_MOUSE Isoform 2 of FAD synthase OS=Mus musculus GN=Flad1	−1,36	0,0230180781655121	0,211925064489371	28,1697161700063	27,854757020185	28,2234557658422	27,8099832106879	27,6361586212546	27,8772178137753	1570857846865,57	1154228435239,92	0,444623852983474	0,00709248739509719	0,0044787637040052	0,73477586628416	-0.444623852983481	1,36095923380977	0.444623852983481	652
A2AQ19	sp|A2AQ19|RTF1_MOUSE RNA polymerase-associated protein RTF1 homolog OS=Mus musculus GN=Rtf1 PE=1 SV=1	−1,36	0,0469359169547671	0,315151075529124	24,9662926902838	25,1735426829318	25,3227647390092	24,7017312592705	25,0736508555744	24,7672234633243	84009862314,8699	61822622402,6944	0,442423850642252	0,00711696105089559	0,0079903698322798	0,735897199438115	-0.442423850642252	1,35888545406007	0.442423850642252	653
O70325-2	sp|O70325-2|GPX41_MOUSE Isoform Cytoplasmic of Phospholipid hydroperoxide glutathione peroxidase, mitochondrial OS=Mus musculus GN=Gpx4;sp|O70325|GPX41_MOUSE Phospholipid hydroperoxide glutathione peroxidase, mitochondrial OS=Mus musculus GN=Gpx4 PE=1 SV	−1,36	0,204430231948933	0,635648869851434	29,41407607155	29,1293211094485	28,8709870434371	28,470179165499	28,9668367465018	29,0597621446406	4513653701406,95	3324230558282,31	0,441275289255335	0,00932289148418566	0,0109944283326564	0,736483296723919	-0.441275289255338	1,35780404585993	0.441275289255338	654
Q08460-5	sp|Q08460-5|KCMA1_MOUSE Isoform 5 of Calcium-activated potassium channel subunit alpha-1 OS=Mus musculus GN=Kcnma1;sp|Q08460|KCMA1_MOUSE Calcium-activated potassium channel subunit alpha-1 OS=Mus musculus GN=Kcnma1 PE=1 SV=2;sp|Q08460-3|KCMA1_MOUSE Isof	−1,36	0,466049256338081	0,795722853632858	24,1399594220105	24,2686184132209	25,4494598461444	24,3517999664098	24,2181219065247	24,3708201043853	49209173375,7711	36245485012,355	0,441125987756783	0,029317279253473	0,0034225691667597	0,736559517787003	-0.441125987756783	1,35766353682389	0.441125987756783	655
Q8VDQ8-2	sp|Q8VDQ8-2|SIR2_MOUSE Isoform 2 of NAD-dependent protein deacetylase sirtuin-2 OS=Mus musculus GN=Sirt2;sp|Q8VDQ8|SIR2_MOUSE NAD-dependent protein deacetylase sirtuin-2 OS=Mus musculus GN=Sirt2 PE=1 SV=2;sp|Q8VDQ8-4|SIR2_MOUSE Isoform 4 of NAD-dependen	−1,35	0,00044009872613183	0,0127236185854292	29,9997657346318	29,8078729257526	29,7170699033562	29,5120517470896	29,5830171708225	29,5194704291796	9120716871409,55	6733928254302,18	0,437698871743043	0,00483650467888349	0,0013205649894273	0,738311291671692	-0.437698871743043	1,35444224039401	0.437698871743043	656
Q9D6L8	sp|Q9D6L8|PPIL3_MOUSE Peptidyl-prolyl cis-trans isomerase-like 3 OS=Mus musculus GN=Ppil3 PE=2 SV=1;sp|Q9D6L8-2|PPIL3_MOUSE Isoform 2 of Peptidyl-prolyl cis-trans isomerase-like 3 OS=Mus musculus GN=Ppil3	−1,35	0,598371679840241	0,861677999617657	23,1878192907474	22,8225685424939	23,3482940068699	22,6937862021931	21,9237820258192	23,8315975050895	10982408621,1059	8110201719,51253	0,437384792397016	0,011653665953983	0,042066195729076	0,738472041909496	-0.437384792397016	1,35414740606057	0.437384792397016	657
Q9Z2L7	sp|Q9Z2L7|CRLF3_MOUSE Cytokine receptor-like factor 3 OS=Mus musculus GN=Crlf3 PE=2 SV=1;sp|Q9Z2L7-2|CRLF3_MOUSE Isoform 2 of Cytokine receptor-like factor 3 OS=Mus musculus GN=Crlf3	−1,35	0,563124371131454	0,845110794140252	21,968529841547	22,2228918150151	22,5429895762164	22,2228918150151	22,6264019676859	20,9771283438076	4579262263,49932	3383378856,62679	0,436650460265586	0,0129403719209442	0,0391818442479725	0,73884802003922	-0.436650460265588	1,35345832008444	0.436650460265588	658
O88448	sp|O88448|KLC2_MOUSE Kinesin light chain 2 OS=Mus musculus GN=Klc2 PE=1 SV=1	−1,35	0,0218956017621037	0,20661982619166	29,142784813033	29,4203370103753	29,1101735891658	29,0578855856183	28,7572558211707	28,9505536434768	4920502641846,21	3635974758411,33	0,436463513937073	0,0058321283014104	0,0052676218906359	0,738943767144709	-0.436463513937073	1,35328294853073	0.436463513937073	659
Q9CQW2	sp|Q9CQW2|ARL8B_MOUSE ADP-ribosylation factor-like protein 8B OS=Mus musculus GN=Arl8b PE=2 SV=1	−1,35	0,166695622862549	0,575385119523278	28,8948809578225	28,7217355155172	28,4070567271281	28,0563316653839	28,4457488486355	28,6150528659003	2839240587251,96	2098780952526,92	0,435953501157721	0,00862473238334357	0,0100977005371302	0,7392050402316	-0.43595350115772	1,35280462872199	0.43595350115772	660
Q9WUM4	sp|Q9WUM4|COR1C_MOUSE Coronin-1C OS=Mus musculus GN=Coro1c PE=1 SV=2	−1,35	0,00054026059020754	0,0145966834461432	31,2748215534313	30,9950183754577	31,2180758996145	30,8553810226389	30,8944000450301	30,8315955900058	34179207576141,2	25265447983224,3	0,435953188708718	0,00474639494905256	0,0010274846852606	0,739205200323627	-0.435953188708719	1,35280433574087	0.435953188708719	661
P52503	sp|P52503|NDUS6_MOUSE NADH dehydrogenase [ubiquinone] iron-sulfur protein 6, mitochondrial OS=Mus musculus GN=Ndufs6 PE=1 SV=2	−1,35	0,21970023256822	0,648792713611515	25,3797969498621	25,1555905514942	24,9274050418198	24,6363202435582	25,2687133583084	24,6534719708188	84015251186,9451	62151152022,5804	0,434870109289179	0,00899246620648612	0,0144958569670706	0,739760354751375	-0.434870109289178	1,35178912140552	0.434870109289178	662
Q8BXC6	sp|Q8BXC6|COMD2_MOUSE COMM domain-containing protein 2 OS=Mus musculus GN=Commd2 PE=1 SV=1	−1,35	0,226439699326217	0,652238880554131	26,5203006373062	26,3021104634812	25,9504924356993	25,6171312267919	26,0031584198129	26,251725356772	253247855510,127	187555067854,133	0,433235806370522	0,0109490427976753	0,0123190105658132	0,740598839332059	-0.433235806370521	1,35025866486895	0.433235806370521	663
Q8C5H8-2	sp|Q8C5H8-2|NAKD2_MOUSE Isoform 2 of NAD kinase 2, mitochondrial OS=Mus musculus GN=Nadk2;sp|Q8C5H8-3|NAKD2_MOUSE Isoform 3 of NAD kinase 2, mitochondrial OS=Mus musculus GN=Nadk2;sp|Q8C5H8|NAKD2_MOUSE NAD kinase 2, mitochondrial OS=Mus musculus GN=Nadk	−1,35	0,0228867690015131	0,211576769716543	29,2130685094284	28,808297486709	28,9271249389734	28,7707322599102	28,5833286268197	28,6943108318933	3864410685520,64	2862712084943,83	0,43286584327851	0,00717858771720057	0,0032852960343052	0,74078878201791	-0.432865843278509	1,34991245045045	0.432865843278509	664

P60762-2	sp|P60762-2|MO4L1_MOUSE Isoform 2 of Mortality factor 4-like protein 1 OS=Mus musculus GN=Morf4l1;sp|P60762|MO4L1_MOUSE Mortality factor 4-like protein 1 OS=Mus musculus GN=Morf4l1 PE=1 SV=2	−1,35	0,481699682437064	0,802303567138125	22,9902222541556	22,61434833139	22,7520392588109	22,4181695734126	23,2303027245149	21,8085797576661	7863806508,61478	5826509931,58991	0,432595853613755	0,00834576392680462	0,0317207167088362	0,740927428110926	-0.432595853613755	1,34965984799565	0.432595853613754	665
Q8BXK8	sp|Q8BXK8|AGAP1_MOUSE Arf-GAP with GTPase, ANK repeat and PH domain-containing protein 1 OS=Mus musculus GN=Agap1 PE=2 SV=1	−1,35	0,0455916196991502	0,312598733858675	26,4430714013401	26,3107417857097	25,9455567246878	25,9650637893661	25,9039797918375	25,9318139841019	247115902378,057	183158766183,421	0,432093102125046	0,0098226741728089	0,0011792408928953	0,741185672070633	-0.432093102125042	1,34918959942429	0.432093102125042	666
Q9QX11-2	sp|Q9QX11-2|CYH1_MOUSE Isoform 2 of Cytohesin-1 OS=Mus musculus GN=Cyth1;sp|Q9QX11|CYH1_MOUSE Cytohesin-1 OS=Mus musculus GN=Cyth1 PE=1 SV=2	−1,35	0,042519801767977	0,303932882243854	25,8025240721104	25,5098805984377	25,8132134394717	25,6064342261004	25,3917468837871	25,2310892455359	146243572007,442	108471877755,893	0,431052153489349	0,00669530613974689	0,0074113063522843	0,741720653201586	-0.431052153489344	1,34821646894093	0.431052153489344	667
Q8BPM2-2	sp|Q8BPM2-2|M4K5_MOUSE Isoform 2 of Mitogen-activated protein kinase kinase kinase kinase 5 OS=Mus musculus GN=Map4k5;sp|Q8BPM2|M4K5_MOUSE Mitogen-activated protein kinase kinase kinase kinase 5 OS=Mus musculus GN=Map4k5 PE=1 SV=2	−1,35	0,113761257170135	0,482581632051629	24,4263753482029	24,4925993676552	24,2127444651752	24,2886600925541	23,7458020638621	24,2030232322995	38627864337,1577	28671280986,925	0,430035552523981	0,00599963961344606	0,0121206895838151	0,74224349388493	-0.43003555252398	1,34726677732931	0.43003555252398	668
P62046	sp|P62046|LRCH1_MOUSE Leucine-rich repeat and calponin homology domain-containing protein 1 OS=Mus musculus GN=Lrch1 PE=1 SV=2	−1,35	0,142697503516395	0,535292535918115	23,8182850170703	23,4643347891491	23,2969409758378	23,19435933754	23,0175812679144	23,4741435389005	16498251799,3596	12248811083,0847	0,42967143812103	0,0113139113898708	0,0099105702987474	0,742430848555733	-0.429671438121025	1,346926790482	0.429671438121025	669
Q8C3W1	sp|Q8C3W1|CA198_MOUSE Uncharacterized protein C1orf198 homolog OS=Mus musculus PE=2 SV=1	−1,35	0,451654393449075	0,786543907075754	26,647166109089	26,5056129060047	25,5484527128798	26,2170897415584	25,5653482547121	26,0255942843937	247269311442,782	183597327737,712	0,429538137719326	0,022783061022134	0,0129155782833798	0,74249944995781	-0.429538137719325	1,34680234450924	0.429538137719325	670
P70404	sp|P70404|IDHG1_MOUSE Isocitrate dehydrogenase [NAD] subunit gamma 1, mitochondrial OS=Mus musculus GN=Idh3g PE=1 SV=1;sp|P51553-2|IDH3G_HUMAN Isoform 2 of Isocitrate dehydrogenase [NAD] subunit gamma, mitochondrial OS=Homo sapiens GN=IDH3G;sp|P51553|ID	−1,35	0,0412171756695738	0,299022406336846	32,1359937740807	31,6726545295256	31,8186731138519	31,6726545295256	31,5035229610434	31,559954806254	69738510972284,1	51815516444904,5	0,428571374944855	0,0074316169393573	0,0027268884480006	0,742997172186503	-0.428571374944853	1,34590014260375	0.428571374944853	671
Q9CXY9	sp|Q9CXY9|GPI8_MOUSE GPI-anchor transamidase OS=Mus musculus GN=Pigk PE=2 SV=2	−1,35	0,249224327798676	0,673464050610029	24,504203243946	24,2210527725757	24,0381319453118	24,2820927735618	24,0503294468235	23,5406755707304	34164832208,0798	25391981285,4632	0,428139071415515	0,00968172331224224	0,0158316111967256	0,74321984462895	-0.428139071415518	1,34549690408125	0.428139071415517	672
O09106	sp|O09106|HDAC1_MOUSE Histone deacetylase 1 OS=Mus musculus GN=Hdac1 PE=1 SV=1	−1,35	0,574440899830172	0,849998193752035	23,1401154868964	23,5596170779061	23,4176157562769	23,8591535596167	22,1096485360386	23,2593273389152	14142488052,1132	10514731857,8359	0,427623892610407	0,00912898774675813	0,0385264054828523	0,743485291915433	-0.42762389261041	1,34501651999559	0.42762389261041	673
Q8VHQ9	sp|Q8VHQ9|ACO11_MOUSE Acyl-coenzyme A thioesterase 11 OS=Mus musculus GN=Acot11 PE=2 SV=1	−1,34	0,0921340478276484	0,446058049245187	28,0674871197023	28,6090306171416	28,3637482883521	28,0940222760243	27,9018726638348	28,1612225425277	2045688128000,44	1524019691633,57	0,424704674359748	0,0095662224251934	0,0047980963882123	0,744991218736369	-0.424704674359752	1,34229770076508	0.424704674359752	674
Q8BX57-2	sp|Q8BX57-2|PXK_MOUSE Isoform 2 of PX domain-containing protein kinase-like protein OS=Mus musculus GN=Pxk;sp|Q8BX57|PXK_MOUSE PX domain-containing protein kinase-like protein OS=Mus musculus GN=Pxk PE=1 SV=2;sp|Q8BX57-3|PXK_MOUSE Isoform 3 of PX domain	−1,34	0,564162612823245	0,845110794140252	22,2659402972637	22,4317248569495	22,5528552230193	21,1169734730192	22,6225346670564	22,6290908514132	5438886153,98488	4053580211,87418	0,424114536555438	0,00642532339394168	0,0393771278468273	0,745296021484889	-0.424114536555439	1,34174874301308	0.424114536555439	675
Q9CSU0-2	sp|Q9CSU0-2|RPR1B_MOUSE Isoform 2 of Regulation of nuclear pre-mRNA domain-containing protein 1B OS=Mus musculus GN=Rprd1b;sp|Q9CSU0|RPR1B_MOUSE Regulation of nuclear pre-mRNA domain-containing protein 1B OS=Mus musculus GN=Rprd1b PE=1 SV=2;sp|Q9CSU0-3|	−1,34	0,0178931547467516	0,180884252551237	26,6277756439543	26,2361110611353	26,3439130720978	26,1745346539103	26,0312420351845	26,1207629911894	292754416549,073	218236802478,582	0,423796523845219	0,00766288302260851	0,0027724600897944	0,745460324906832	-0.423796523845217	1,34145301445115	0.423796523845217	676
Q9CY58-2	sp|Q9CY58-2|PAIRB_MOUSE Isoform 2 of Plasminogen activator inhibitor 1 RNA-binding protein OS=Mus musculus GN=Serbp1;sp|Q9CY58-3|PAIRB_MOUSE Isoform 3 of Plasminogen activator inhibitor 1 RNA-binding protein OS=Mus musculus GN=Serbp1;sp|Q9CY58|PAIRB_MOU	−1,34	0,0020242600027388	0,0387684225840989	29,1461064414382	29,0627516643017	28,9766402388757	28,7281279757244	28,9252452807151	28,6513548480099	4182092275190,03	3118092964303,27	0,423560952550133	0,00291574308554774	0,0049110750380350	0,745582057765951	-0.423560952550135	1,34123399240103	0.423560952550135	677
Q60692	sp|Q60692|PSB6_MOUSE Proteasome subunit beta type-6 OS=Mus musculus GN=Psmb6 PE=1 SV=3	−1,34	0,108985970374818	0,473794072647748	30,6814880843035	30,4559240288616	30,4016042683483	29,9008251465763	30,3309155382186	30,4270212494486	17849657932130	13310673011595,2	0,423312908598918	0,004864040411235	0,0092723695993640	0,745710257429389	-0.423312908598916	1,3410034125683	0.423312908598916	678
Q9DB60	sp|Q9DB60|PGFS_MOUSE Prostamide/prostaglandin F synthase OS=Mus musculus GN=Fam213b PE=1 SV=1	−1,34	0,000162235287754072	0,00553673662493031	29,6742040431969	29,7066894223823	29,7991526231768	29,3922159985882	29,3411392654344	29,5664435234524	8130812960132,25	6063245068220,56	0,423309472104663	0,00218083951531738	0,0040134353668306	0,74571203371058	-0.423309472104665	1,34100021830694	0.423309472104665	679
Q9R1P3	sp|Q9R1P3|PSB2_MOUSE Proteasome subunit beta type-2 OS=Mus musculus GN=Psmb2 PE=1 SV=1	−1,34	0,0694423291992155	0,392038224173183	30,7034268677074	30,5414934586332	30,4288703183794	30,0196926870257	30,2703137998586	30,5053526695314	18669834611127,7	13930749664305,4	0,422436250645736	0,00451648097773415	0,0080248038843988	0,746163528197637	-0.42243625064574	1,34018879536434	0.42243625064574	680
P51150	sp|P51150|RAB7A_MOUSE Ras-related protein Rab-7a OS=Mus musculus GN=Rab7a PE=1 SV=2	−1,34	0,213650443633174	0,645012843958033	31,736695594508	31,849738476019	31,5082584125245	31,0083247513143	31,471637043656	31,736695594508	58393799529375,9	43577081009755,2	0,422245625074868	0,0054879790137826	0,0117385246685689	0,746262126475142	-0.422245625074867	1,34001172580384	0.422245625074867	681

Q9WU28	sp|Q9WU28|PFD5_MOUSE Prefoldin subunit 5 OS=Mus musculus GN=Pfdn5 PE=1 SV=1	−1,34	0,10287925316088	0,464645701589286	26,3932386786055	26,4451428857265	26,3382310811099	25,7538649352021	26,2087814532622	26,336120588715	289726790790,09	216225783439,537	0,42215453075612	0,0020257273070753	0,0117281819902187	0,746309248274506	-0.42215453075612	1,33992711776256	0.42215453075612	682
Q6PDJ6	sp|Q6PDJ6|FBX42_MOUSE F-box only protein 42 OS=Mus musculus GN=Fbxo42 PE=2 SV=1	−1,34	0,0871368460598655	0,440251927134614	25,1410841300026	24,939766410663	24,8340128117331	24,9676751874106	24,5643854343456	24,5072217421758	69990203378,5238	52273782305,4989	0,421065449985847	0,00624686556682965	0,01016922330659	0,746872844800718	-0.421065449985847	1,3389159974973	0.421065449985847	683
P02088	sp|P02088|HBB1_MOUSE Hemoglobin subunit beta-1 OS=Mus musculus GN=Hbb-b1 PE=1 SV=2	−1,34	0,0261068036516065	0,223652600211782	36,3824391571779	36,2026315719732	35,9416998882443	35,8547745671773	35,9416998882443	35,8547745671773	5138770306621820	3838083982896620	0,421036887668051	0,00612596707448587	0,0013985817528295	0,746887631453553	-0.421036887668051	1,33888949004799	0.421036887668051	684
Q9CQX8	sp|Q9CQX8|RT36_MOUSE 28S ribosomal protein S36, mitochondrial OS=Mus musculus GN=Mrps36 PE=1 SV=1	−1,34	0,130742345889017	0,514825425964414	27,024059925362	26,9179630935536	26,5089478029815	26,3744753513748	26,4583956916079	26,7440322826555	443068522768,526	331082836346,641	0,420337614084962	0,0101426698045579	0,0073037497420165	0,747249735273137	-0.420337614084963	1,33824068821447	0.420337614084963	685
Q61247	sp|Q61247|A2AP_MOUSE Alpha-2-antiplasmin OS=Mus musculus GN=Serpinf2 PE=1 SV=1	−1,34	0,12911535063918	0,512962462222669	23,8201140153781	24,3182133554805	24,2494398701144	23,8568543067188	23,635106284079	24,021910159197	30144120927,4391	22526470394,9389	0,420255377928152	0,0111865816178808	0,0081421881202345	0,747292331037389	-0.420255377928148	1,33816440831368	0.420255377928148	686
P24549	sp|P24549|AL1A1_MOUSE Retinal dehydrogenase 1 OS=Mus musculus GN=Aldh1a1 PE=1 SV=5	−1,34	0,294339677446323	0,711969538636193	28,100354168705	28,9293487073306	28,9160873320475	28,4381222529815	28,3063564870261	28,3294382730544	2766479629624,85	2068763781263,09	0,41928237824697	0,0165745740760193	0,0024813042087382	0,747796498882454	-0.419282378246972	1,33726221170392	0.419282378246972	687
Q14CH7	sp|Q14CH7|SYAM_MOUSE Alanine--tRNA ligase, mitochondrial OS=Mus musculus GN=Aars2 PE=2 SV=1	−1,34	0,404916345104877	0,759526281549977	25,1125314478412	24,6333618185744	24,5262771526765	24,8234435167563	24,7031955909906	23,8751848769123	56493427432,1363	42267092347,8905	0,418548161603924	0,0126098554964249	0,0211007425443005	0,748177164479259	-0.418548161603924	1,3365818251029	0.418548161603924	688
Q01768	sp|Q01768|NDKB_MOUSE Nucleoside diphosphate kinase B OS=Mus musculus GN=Nme2 PE=1 SV=1	−1,34	0,00248356701369941	0,0456393144744196	29,9023714560052	29,8297582429479	29,7557574407144	29,3780373232367	29,5685996112172	29,6715275253764	9009455142795,03	6742070255622,25	0,418248199049876	0,00245758742393039	0,0050410565621890	0,748332740300501	-0.418248199049876	1,33630395430573	0.418248199049876	689
Q9QYG0-2	sp|Q9QYG0-2|NDRG2_MOUSE Isoform 2 of Protein NDRG2 OS=Mus musculus GN=Ndrg2	−1,34	0,141041634813345	0,532098608288336	28,5904583519206	28,0423253078252	28,3063564870261	28,2272204810265	28,020951024883	27,8216158205759	1977880070496,9	1480294902293,65	0,418070334203409	0,00968202966863962	0,0072372804362080	0,748425005324897	-0.41807033420341	1,33613921620095	0.41807033420341	690
Q91ZP9	sp|Q91ZP9|NECA2_MOUSE N-terminal EF-hand calcium-binding protein 2 OS=Mus musculus GN=Necab2 PE=1 SV=1;sp|Q91ZP9-2| NECA2_MOUSE Isoform 2 of N-terminal EF-hand calcium-binding protein 2 OS=Mus musculus GN=Necab2	−1,34	0,106100273134678	0,469843063178832	29,0984836692739	28,5325900295205	28,8948809578225	28,496957284198	28,6894779101035	28,470179165499	3356718197091,87	2512262321924,82	0,418064311687328	0,00993798175617248	0,0041899953826867	0,748428129624151	-0.418064311687326	1,33613363851274	0.418064311687326	691
P62751	sp|P62751|RL23A_MOUSE 60S ribosomal protein L23a OS=Mus musculus GN=Rpl23a PE=1 SV=1	−1,34	0,311268043877046	0,719371512809526	27,0796340180657	26,9639504293006	26,4697443055486	26,5284979350479	26,1835363389388	26,9324340242253	452374516927,697	338623971276,534	0,417833556408164	0,0120699651196586	0,0141190843418988	0,748547848310068	-0.417833556408166	1,3359199445401	0.417833556408166	692
P54923	sp|P54923|ADPRH_MOUSE [Protein ADP-ribosylarginine] hydrolase OS=Mus musculus GN=Adprh PE=2 SV=1	−1,34	0,0291010152352731	0,239292587233523	29,6629732680634	29,2449656239036	29,351342606842	29,0951539669868	29,2163908422627	29,0789957161334	5981906821903,35	4477921819381,35	0,417776098059592	0,00738416321261046	0,0025779614674674	0,748577661388003	-0.417776098059594	1,33586673979265	0.417776098059594	693
P70175	sp|P70175|DLG3_MOUSE Disks large homolog 3 OS=Mus musculus GN=Dlg3 PE=1 SV=1	−1,34	0,505035739136904	0,814993646406912	29,9308608554239	28,6297384787753	29,465248371337	28,961551696187	29,450672574706	28,7457012936542	5534095395280,42	4143831068987,45	0,417382322894209	0,0224683831677796	0,0124327829512966	0,748782009164747	-0.417382322894209	1,33550217254215	0.417382322894209	694
Q8BWD8	sp|Q8BWD8|CDK19_MOUSE Cyclin-dependent kinase 19 OS=Mus musculus GN=Cdk19 PE=2 SV=1;sp|Q8R3L8-3|CDK8_MOUSE Isoform 3 of<br>Cyclin-dependent kinase 8 OS=Mus musculus GN=Cdk8;sp|Q8R3L8|CDK8_MOUSE Cyclin-dependent kinase 8 OS=Mus musculus GN=Cdk8 PE=2<br>SV=3	−1,33	0,369181120751094	0,740483034506945	22,7026757793855	23,220546028412	23,3845394580678	23,1493509179357	22,9604916551048	22,3326153441959	10797571542,5568	8092088064,31429	0,416122949977179	0,0154055320696043	0,0187419224753536	0,749435929405116	-0.416122949977176	1,33433688026377	0.416122949977176	695
P63054	sp|P63054|PCP4_MOUSE Purkinje cell protein 4 OS=Mus musculus GN=Pcp4 PE=2 SV=2;sp|P48539|PCP4_HUMAN Purkinje cell protein 4<br>OS=Homo sapiens GN=PCP4 PE=1 SV=3	−1,33	0,260707323977669	0,682439759823898	26,7516578076357	26,325250003904	25,9481071296484	25,9023194601262	25,9945560886311	26,2637346192989	275449913563,129	206493902834,287	0,415690826506067	0,0152620139081682	0,0072077669529647	0,749660437947321	-0.415690826506062	1,33393727263792	0.415690826506062	696
Q9D3A9	sp|Q9D3A9|TTYH1_MOUSE Protein tweety homolog 1 OS=Mus musculus GN=Ttyh1 PE=1 SV=1;sp|Q9D3A9-3|TTYH1_MOUSE Isoform 3 of Protein<br>tweety homolog 1 OS=Mus musculus GN=Ttyh1;sp|Q9D3A9-4|TTYH1_MOUSE Isoform 4 of Protein tweety homolog 1 OS=Mus musculus GN=Tty	−1,33	2,02×10^−05	0,00095457271297424	28,2082847129067	28,4258373734941	28,3251900002394	28,0415499426754	27,9865800133104	28,0692982272581	1991224108693,41	1493996800516,01	0,414478544417241	0,00384457670286245	0,0015017944480357	0,750290634787629	-0.414478544417239	1,3328168494107	0.414478544417239	697
Q8R1U1	sp|Q8R1U1|COG4_MOUSE Conserved oligomeric Golgi complex subunit 4 OS=Mus musculus GN=Cog4 PE=2 SV=1	−1,33	0,339613119957318	0,729620681454069	24,4478937746508	24,0692182003921	23,6978719068565	23,9723375129221	23,3681777993606	24,0132687336335	28457036453,9464	21355917019,0253	0,414149577529098	0,0155791865713828	0,0151866301890026	0,75046173741903	-0.414149577529101	1,33251297186606	0.414149577529101	698
Q80WQ2	sp|Q80WQ2|VAC14_MOUSE Protein VAC14 homolog OS=Mus musculus GN=Vac14 PE=1 SV=1	−1,33	0,11287350456255	0,481076973512557	28,6943108318933	28,6943108318933	28,1645775605123	28,198658713088	28,3076758523563	28,187447373342	2427135993700,37	1822555305390,6	0,413292351954372	0,0107246128585572	0,0023525164079286	0,750907781896458	-0.413292351954378	1,33172144983562	0.413292351954378	699

P36536	sp|P36536|SAR1A_MOUSE GTP-binding protein SAR1a OS=Mus musculus GN=Sar1a PE=2 SV=1	−1,33	0,215113364875118	0,646799042967852	27,4925739899774	27,1451359480015	27,1289472953639	26,6234109353197	26,9761562920847	27,3089720088055	686967218478,704	516072490866,768	0,412667526320417	0,00753704185135229	0,0127117168491338	0,751233067583073	-0.412667526320417	1,33114481131306	0.412667526320417	700
Q9JLM8	sp|Q9JLM8|DCLK1_MOUSE Serine/threonine-protein kinase DCLK1 OS=Mus musculus GN=Dclk1 PE=1 SV=1	−1,33	0,199213246116367	0,629685880307932	31,8823889345278	32,2862250679884	31,5462290964587	31,687944408101	31,6726545295256	31,4973921613793	71803023394759,6	53963573299175,7	0,412058710376996	0,0116130390792705	0,0033485195562471	0,751550154127829	-0.412058710376997	1,33058318797166	0.412058710376997	701
P59114	sp|P59114|PCIF1_MOUSE Phosphorylated CTD-interacting factor 1 OS=Mus musculus GN=Pcif1 PE=1 SV=1	−1,33	0,0895010576441496	0,441176105282424	25,1359541100947	25,035270903413	25,1035425680548	25,1199552746439	24,7401928293227	24,558208524557	78911214779,4678	59314455141,737	0,411846611637423	0,00204829335771054	0,0115543025558327	0,751660651879487	-0.411846611637424	1,33038758580691	0.411846611637424	702
Q8K2I1	sp|Q8K2I1|FNTB_MOUSE Protein farnesyltransferase subunit beta OS=Mus musculus GN=Fntb PE=1 SV=1	−1,33	0,0934206784800819	0,44871583028687	26,886722967848	26,9881320866984	26,9261638707406	26,8636371325686	26,7629318109403	26,3183357187085	497903925708,601	374291803749,073	0,411703933921633	0,001897991485144	0,0108885411241932	0,751734992280675	-0.411703933921634	1,33025602142867	0.411703933921634	703
Q6PGB6-2	sp|Q6PGB6-2|NAA50_MOUSE Isoform 2 of N-alpha-acetyltransferase 50 OS=Mus musculus GN=Naa50;sp|Q6PGB6|NAA50_MOUSE N-alpha-acetyltransferase 50 OS=Mus musculus GN=Naa50 PE=1 SV=1;sp|Q6PGB6-4|NAA50_MOUSE Isoform 4 of N-alpha-acetyltransferase 50 OS=Mus mus	−1,33	0,0924310442384414	0,446058049245187	26,5247335525472	26,6267436086175	26,5664270729435	25,9546021761348	26,4776551762052	26,4297525807184	347015312687,74	260882680797,179	0,41159815454986	0,00193008954633225	0,010996002045637	0,75179011201714	-0.41159815454986	1,33015848973709	0.41159815454986	704
P62835	sp|P62835|RAP1A_MOUSE Ras-related protein Rap-1A OS=Mus musculus GN=Rap1a PE=2 SV=1	−1,33	0,100981791933521	0,46324407107743	26,4937458641267	26,0846949698555	25,9864544591337	25,879283072998	25,7861812046239	26,0475252294948	236283556431,38	177871965896,231	0,409680084255217	0,0102735658434311	0,0051134350252378	0,752790285463168	-0.409680084255215	1,32839121241414	0.409680084255215	705
Q99KE1	sp|Q99KE1|MAOM_MOUSE NAD-dependent malic enzyme, mitochondrial OS=Mus musculus GN=Me2 PE=2 SV=1	−1,33	0,129841077372747	0,513077226998934	28,159207652022	28,6907897587117	28,6062432168209	28,3695269666755	28,0738882662073	28,1645775605123	2349946181935,24	1771174965264,31	0,407920981262285	0,0100278668122547	0,0053702245601868	0,753708735493552	-0.407920981262285	1,3267724691358	0.407920981262285	706
Q9JI78	sp|Q9JI78|NGLY1_MOUSE Peptide-N(4)-(N-acetyl-beta-glucosaminyl)asparagine amidase OS=Mus musculus GN=Ngly1 PE=1 SV=2	−1,33	0,226143726163815	0,652238880554131	24,2586916472883	23,6996447819213	23,675109750947	23,8514133380406	23,3986660676142	23,5376585795527	23442475200,8368	17683762131,029	0,406699672964061	0,0138235659383473	0,0098291215066675	0,754347055058323	-0.406699672964061	1,3256497699493	0.40669967296406	707
Q3TYS2	sp|Q3TYS2|CQ062_MOUSE Uncharacterized protein C17orf62 homolog OS=Mus musculus PE=2 SV=2	−1,33	0,293394667760212	0,711969538636193	23,2474511581564	22,6264019676859	22,8462665924133	22,2373996301638	22,7607929420049	22,8773938140701	8876796485,73887	6698808167,55036	0,406134683322022	0,0137472914970945	0,0150656742051648	0,754642531042862	-0.406134683322024	1,32513071933286	0.406134683322024	708
Q99KK2	sp|Q99KK2|NEUA_MOUSE N-acylneuraminate cytidylyltransferase OS=Mus musculus GN=Cmas PE=1 SV=2;sp|Q99KK2-2|NEUA_MOUSE Isoform 2 of N-acylneuraminate cytidylyltransferase OS=Mus musculus GN=Cmas	−1,32	0,00205599116941504	0,0390466272718614	29,5939809288885	29,4384942443743	29,6094844022266	29,3648762637523	29,1231989742319	29,3099584234601	6795759009673,85	5129407238997,91	0,405842577023523	0,0032004256452326	0,0043289312445794	0,754795340990776	-0.405842577023524	1,3248624437551	0.405842577023524	709
P68040	sp|P68040|GBLP_MOUSE Guanine nucleotide-binding protein subunit beta-2-like 1 OS=Mus musculus GN=Gnb2l1 PE=1 SV=3	−1,32	0,109080151116302	0,473794072647748	29,9652839885513	30,3642246304452	30,5354699767686	30,0952385083161	29,9903371484726	29,9373485722038	14257813648649,6	10768447105614,6	0,40494255303386	0,00965956705989071	0,0026778096387292	0,755266366287128	-0.404942553033849	1,32403618728049	0.404942553033849	710
Q9QZB7	sp|Q9QZB7|ARP10_MOUSE Actin-related protein 10 OS=Mus musculus GN=Actr10 PE=1 SV=2	−1,32	0,283317411680493	0,704458182186602	29,5194704291796	29,1625520471545	28,6927585562291	28,9217714421477	28,9750635875556	28,6363686210369	4454460238184,81	3364838947847,84	0,404713171760134	0,0142365414684873	0,0063139235417658	0,755386459397157	-0.404713171760133	1,32382568890374	0.404713171760133	711
Q8BNU0	sp|Q8BNU0|ARMC6_MOUSE Armadillo repeat-containing protein 6 OS=Mus musculus GN=Armc6 PE=2 SV=1	−1,32	0,0533850527484451	0,340808374718977	28,7942855809832	28,624551630105	28,7823143927038	28,6513548480099	28,5119947056563	28,197075208073	3012276440183,1	2276078041707,04	0,404304148590532	0,00329680457922781	0,0081791412937399	0,755600651834162	-0.40430414859053	1,32345041997063	0.40430414859053	712
Q9JKC8	sp|Q9JKC8|AP3M1_MOUSE AP-3 complex subunit mu-1 OS=Mus musculus GN=Ap3m1 PE=1 SV=1	−1,32	0,395625979868063	0,753881747531964	26,9469309955916	26,2906517440577	27,0158477199576	26,1649342395901	26,3210361882821	26,9302714663707	414833672024,013	313818500363,554	0,402602597179835	0,0149632286837468	0,0152767861726337	0,756492352302077	-0.402602597179834	1,32189042884162	0.402602597179834	713
Q3TBW2	sp|Q3TBW2|RM10_MOUSE 39S ribosomal protein L10, mitochondrial OS=Mus musculus GN=Mrpl10 PE=2 SV=2	−1,32	0,250120311130778	0,673538059877323	24,2783394430416	24,3497113967141	23,9376551234288	23,7584773744894	23,6519620919524	24,3182133554805	31986144269,3314	24198365842,045	0,402537471953151	0,00910381629219587	0,0149687787264762	0,756526502171961	-0.402537471953151	1,32183075824711	0.402537471953151	714
Q9Z255	sp|Q9Z255|UBE2A_MOUSE Ubiquitin-conjugating enzyme E2 A OS=Mus musculus GN=Ube2a PE=2 SV=1	−1,32	0,405717532386171	0,760245992583427	25,0126127548663	24,961398664519	24,7031955909906	24,0381319453118	25,1499790121567	24,6522337538154	64659594424,541	48919808690,361	0,402445696268942	0,00666254143055878	0,0226273598485984	0,756574629422573	-0.402445696268944	1,32174667390474	0.402445696268944	715
Q921D4	sp|Q921D4|MED6_MOUSE Mediator of RNA polymerase II transcription subunit 6 OS=Mus musculus GN=Med6 PE=1 SV=2	−1,32	0,654533019142593	0,891901943526937	23,2373012347919	22,3570874543215	21,3395865968786	22,0311999668215	22,5448185137444	21,5219605614353	4894241247,66838	3703927872,47585	0,402029211802503	0,0425651369918946	0,0232123831587138	0,756793072724072	-0.402029211802506	1,32136516049295	0.402029211802506	716
Q99LE6	sp|Q99LE6|ABCF2_MOUSE ATP-binding cassette sub-family F member 2 OS=Mus musculus GN=Abcf2 PE=2 SV=1	−1,32	0,262285034420618	0,682806179310143	26,7284847671728	26,7455391951909	26,242967719549	26,3346641672483	26,593052849711	25,956784137851	346909772211,936	262845887496,372	0,400343318345951	0,0107391415987893	0,012169650362422	0,757677957067732	-0.400343318345951	1,3198219516245	0.400343318345951	717
Q8K0T0	sp|Q8K0T0|RTN1_MOUSE Reticulon-1 OS=Mus musculus GN=Rtn1 PE=1 SV=1	−1,32	0,199662927678584	0,630229505377673	33,0034445863308	33,0286081008433	32,6186624488613	32,26957919978	32,8262003127847	32,72254043549	191052946432177	144761206540174	0,400297469919844	0,00698716365521265	0,0090784885177343	0,757702036234043	-0.400297469919839	1,31978000873567	0.400297469919839	718

Q8BP27-2	sp|Q8BP27-2|SFR1_MOUSE Isoform 2 of Swi5-dependent recombination DNA repair protein 1 homolog OS=Mus musculus GN=Sfr1;sp|Q8BP27|SFR1_MOUSE Swi5-dependent recombination DNA repair protein 1 homolog OS=Mus musculus GN=Sfr1 PE=1 SV=2;sp|Q8BP27-3|SFR1_MOUSE	−1,32	0,381339444269736	0,749797813889279	25,4960085354994	26,0601159193524	25,9168429770321	25,4695074078681	26,0446839509163	25,1266580132717	164195311061,719	124422614670,485	0,400164199449235	0,0113541710258433	0,0181582041305582	0,757772032988908	-0.400164199449237	1,31965809830123	0.400164199449237	719
Q8BH43	sp|Q8BH43|WASF2_MOUSE Wiskott-Aldrich syndrome protein family member 2 OS=Mus musculus GN=Wasf2 PE=1 SV=1	−1,32	0,277294903202071	0,699203439845458	25,4287308522879	24,567454870373	24,8541011789319	24,7178927598584	24,6103867125833	24,690050667465	68499729538,5364	51909970230,4893	0,400086631439528	0,0175786486787592	0,0022614347655526	0,757812776491124	-0.400086631439528	1,31958714740898	0.400086631439528	720
P60843	sp|P60843|IF4A1_MOUSE Eukaryotic initiation factor 4A-I OS=Mus musculus GN=Eif4a1 PE=1 SV=1	−1,32	0,107145133988687	0,471708790664063	30,9746901743204	30,5213672527491	30,5414934586332	30,3063347984404	30,577500263056	30,3224336631228	21076580868717,7	15975694267513,3	0,399762217124867	0,0083481344323493	0,0050037095105920	0,757983202637234	-0.399762217124867	1,31929044934073	0.399762217124867	721
Q9D385-2	sp|Q9D385-2|AR2BP_MOUSE Isoform 2 of ADP-ribosylation factor-like protein 2-binding protein OS=Mus musculus GN=Arl2bp;sp|Q9D385|AR2BP_MOUSE ADP-ribosylation factor-like protein 2-binding protein OS=Mus musculus GN=Arl2bp PE=1 SV=1	−1,32	0,0137903844957049	0,153418027514717	24,7487693428392	24,8859998394022	24,8641690818116	24,3497113967141	24,6838243385297	24,6343822529779	60929263978,8309	46187390401,1517	0,399636276940024	0,00296945491691687	0,0073436313051397	0,758049373732775	-0.399636276940024	1,31917528679671	0.399636276940024	722
Q9CUN6	sp|Q9CUN6|SMUF1_MOUSE E3 ubiquitin-protein ligase SMURF1 OS=Mus musculus GN=Smurf1 PE=2 SV=2	−1,32	0,68551267626037	0,90636237621492	25,1612997878741	23,393552816274	25,0309060428071	23,5057520442414	24,6878806630467	24,5643854343456	44939645512,3479	34103733903,5071	0,398059040723406	0,0401624259487556	0,0267926248454098	0,758878569572529	-0.398059040723408	1,31773387745459	0.398059040723408	723
Q8BG94	sp|Q8BG94|COMD7_MOUSE COMM domain-containing protein 7 OS=Mus musculus GN=Commd7 PE=2 SV=1	−1,32	0,292604593082925	0,711969538636193	27,8119809890143	27,1953399119946	27,0652717104084	26,8468256315125	27,1340590772315	27,2661868502051	760719756793,904	577721171118,768	0,396991709515066	0,014581081609291	0,0079168868371042	0,759440209037828	-0.396991709515066	1,31675935524529	0.396991709515066	724
Q80XU3	sp|Q80XU3|NUCKS_MOUSE Nuclear ubiquitous casein and cyclin-dependent kinase substrate 1 OS=Mus musculus GN=Nucks1 PE=1 SV=1	−1,32	0,154698087796111	0,558167424885968	25,9481071296484	25,3260413000039	25,7162865377133	25,4434411168015	25,4651569064471	25,2573533322883	139799951868,138	106206425941,308	0,396492806026491	0,0122499828630975	0,0044990133383969	0,759702879164679	-0.396492806026491	1,31630408074738	0.396492806026491	725
Q8BR07-3	sp|Q8BR07-3|BICD1_MOUSE Isoform 3 of Protein bicaudal D homolog 1 OS=Mus musculus GN=Bicd1;sp|Q8BR07|BICD1_MOUSE Protein bicaudal D homolog 1 OS=Mus musculus GN=Bicd1 PE=1 SV=2;sp|Q8BR07-4|BICD1_MOUSE Isoform 4 of Protein bicaudal D homolog 1 OS=Mus mus	−1,32	0,438068873086543	0,779151494882374	23,732793378787	22,5286124376778	23,2077788046151	22,8005593253074	23,0041717325947	22,840723799719	11394480104,152	8658594824,23105	0,396130281591615	0,026071964027876	0,0047132374016104	0,759893803410651	-0.396130281591611	1,31597335773982	0.396130281591611	726
Q9WU63	sp|Q9WU63|HEBP2_MOUSE Heme-binding protein 2 OS=Mus musculus GN=Hebp2 PE=1 SV=1	−1,32	0,631467853732383	0,879752175595853	22,9075582228068	22,2553767996608	23,1763774222025	23,0737103227927	21,504041661994	22,9386828286442	7818595836,96296	5942990359,34865	0,395720492615778	0,0207908761786809	0,0386525188523962	0,760109677399201	-0.395720492615778	1,31559961638906	0.395720492615778	727
Q80U04-2	sp|Q80U04-2|PJA2_MOUSE Isoform 2 of E3 ubiquitin-protein ligase Praja-2 OS=Mus musculus GN=Pja2;sp|Q80U04|PJA2_MOUSE E3 ubiquitin-protein ligase Praja-2 OS=Mus musculus GN=Pja2 PE=1 SV=2	−1,32	0,514997912676074	0,819626762144705	23,2126920867356	24,18201000329	22,8329525082737	22,9752239618567	23,3825283651281	23,0480487104139	14672167950,9431	11156264464,5331	0,395228018882721	0,0297177545786101	0,0093886277569143	0,760369190281522	-0.395228018882712	1,31515060418184	0.395228018882712	728
Q811I0	sp|Q811I0|ATPF1_MOUSE ATP synthase mitochondrial F1 complex assembly factor 1 OS=Mus musculus GN=Atpaf1 PE=2 SV=1	−1,31	0,0421119026824314	0,301968287955065	27,3782962363329	27,4793096785935	27,6721550228499	27,3835409950841	27,0364602305821	27,2883154174475	885944648915,004	673736921652,375	0,395031203425349	0,00542719580068275	0,0065844198588732	0,760472928503474	-0.395031203425349	1,31497120083931	0.395031203425349	729
Q9CZB0	sp|Q9CZB0|C560_MOUSE Succinate dehydrogenase cytochrome b560 subunit, mitochondrial OS=Mus musculus GN=Sdhc PE=2 SV=1	−1,31	0,0520299480118945	0,335456444875023	25,2894343129533	25,0513945111618	24,9865539749528	24,6453225574227	24,8473603223002	25,0138713240005	80307399326,6402	61084138347,5027	0,394735114641156	0,00635148595359303	0,0074312744239065	0,760629018741482	-0.394735114641157	1,31470135290733	0.394735114641156	730
Q9JKL4	sp|Q9JKL4|NDUF3_MOUSE NADH dehydrogenase [ubiquinone] 1 alpha subcomplex assembly factor 3 OS=Mus musculus GN=Ndufaf3 PE=2 SV=1	−1,31	0,107498597480166	0,472047814185685	25,7149021750404	25,6487332316027	25,3839467459252	25,0817452967474	25,2931187432148	25,5531568912004	128928959000,21	98108545556,1115	0,394125636608953	0,00684566205680714	0,0093295229702300	0,760950420424567	-0.394125636608949	1,3141460641313	0.394125636608949	731
P83870	sp|P83870|PHF5A_MOUSE PHD finger-like domain-containing protein 5A OS=Mus musculus GN=Phf5a PE=1 SV=1	−1,31	0,210794178278602	0,641644645910089	25,2415366291153	24,8846009566493	24,7280852384171	24,3708201043853	24,784693819856	24,8802711146083	68589658395,4141	52212878342,5526	0,393585378058225	0,0105470708035245	0,010972709924805	0,761235433504412	-0.393585378058226	1,31365403656582	0.393585378058226	732
Q99J95-2	sp|Q99J95-2|CDK9_MOUSE Isoform 2 of Cyclin-dependent kinase 9 OS=Mus musculus GN=Cdk9;sp|Q99J95|CDK9_MOUSE Cyclin-dependent kinase 9 OS=Mus musculus GN=Cdk9 PE=1 SV=1;sp|Q99J95-3|CDK9_MOUSE Isoform 3 of Cyclin-dependent kinase 9 OS=Mus musculus GN=Cdk9	−1,31	0,229669009530601	0,654468548759245	24,1424393315207	24,6197060572759	24,8936242018883	24,333802902532	24,163506496647	24,3404652835671	46000734324,9976	35022558786,9787	0,393372399051953	0,0154835947097675	0,0041310682741046	0,761347819787886	-0.393372399051954	1,31346012165452	0.393372399051954	733
Q6P9N1	sp|Q6P9N1|HYCCI_MOUSE Hyccin OS=Mus musculus GN=Fam126a PE=1 SV=2	−1,31	0,488388083678041	0,806046930006372	22,4317248569495	23,2404230130127	22,4605995625438	22,9454843610105	21,9376699279833	22,4317248569495	7298362251,60377	5556826721,01341	0,393311507041464	0,0202014707984065	0,0224588973578686	0,761379954768939	-0.393311507041464	1,31340468544838	0.393311507041464	734
Q9CWE0	sp|Q9CWE0|MFR1L_MOUSE Mitochondrial fission regulator 1-like OS=Mus musculus GN=Mtfr1l PE=1 SV=1	−1,31	0,704115565906652	0,915491421841963	23,6309486811925	22,8392616668271	24,534409034057	22,4525488290568	24,2663462043832	23,4679001027352	19009533638,0566	14473690336,9275	0,393290327960218	0,0358365823557802	0,0388559281754138	0,761391132076567	-0.393290327960215	1,31338540451958	0.393290327960215	735

Q60605-2	sp|Q60605-2|MYL6_MOUSE Isoform Smooth muscle of Myosin light polypeptide 6 OS=Mus musculus GN=Myl6;sp|Q60605|MYL6_MOUSE Myosin light polypeptide 6 OS=Mus musculus GN=Myl6 PE=1 SV=3	−1,31	0,0892170148235766	0,441176105282424	30,2298776072897	30,0876771272862	30,0330859729261	29,5620727820686	29,9023714560052	30,0689715847678	12011435086334,1	9147227473574,9	0,393002096130223	0,00337332615704777	0,008656942802418	0,761543263384246	-0.393002096130222	1,31312303329435	0.393002096130222	736
Q07235	sp|Q07235|GDN_MOUSE Glia-derived nexin OS=Mus musculus GN=Serpine2 PE=2 SV=2	−1,31	0,0177724540036005	0,180065108755229	27,6281717314036	27,2661868502051	27,5100018761904	27,2584776827915	27,1138793678718	27,2152024674969	849675333264,386	647155972500,618	0,392798221670191	0,00672018905173145	0,002728933052114	0,761650888480304	-0.392798221670191	1,31293748241437	0.392798221670191	737
Q9CR70	sp|Q9CR70|LAGE3_MOUSE EKC/KEOPS complex subunit Lage3 OS=Mus musculus GN=Lage3 PE=2 SV=1	−1,31	0,427828363450761	0,773941056137542	21,9601516514935	23,0041717325947	22,8619222612621	22,1271865824891	22,4569741763383	22,4253348908122	6589534518,53962	5019010049,35638	0,392773722819285	0,0250428068590427	0,008146239774885	0,761663822419538	-0.392773722819284	1,31291518720602	0.392773722819284	738
Q8K0C9	sp|Q8K0C9|GMDS_MOUSE GDP-mannose 4,6 dehydratase OS=Mus musculus GN=Gmds PE=2 SV=1	−1,31	0,0762356103914135	0,411454739080411	28,01920864903	27,5000243142379	27,7741719005353	27,5424060245662	27,5020965064079	27,4336352949716	1142760894774,63	870829992907,727	0,392060570839091	0,00935469573121329	0,002000136950754	0,762040420607382	-0.392060570839089	1,31226634829023	0.392060570839089	739
O89106	sp|O89106|FHIT_MOUSE Bis(5-adenosyl)-triphosphatase OS=Mus musculus GN=Fhit PE=2 SV=3	−1,31	0,303679163607886	0,716532404521184	25,8617916340518	25,6777150180066	25,3545598909119	24,9274050418198	25,6315175429121	25,520279157572	135380546705,694	103179282007,796	0,391867135638775	0,0100179660936704	0,0149258831951135	0,762142601123477	-0.391867135638769	1,3120904126418	0.391867135638769	740
Q60875-2	sp|Q60875-2|ARHG2_MOUSE Isoform 2 of Rho guanine nucleotide exchange factor 2 OS=Mus musculus GN=Arhgef2;sp|Q60875-3|ARHG2_MOUSE Isoform 3 of Rho guanine nucleotide exchange factor 2 OS=Mus musculus GN=Arhgef2;sp|Q60875-5|ARHG2_MOUSE Isoform 5 of Rho gu	−1,31	0,978977801106555	0,994969692078273	22,9522447247002	21,0792559200743	0	21,1423031271158	22,0749258890454	0	2367078,09594513	1804407,79659594	0,391581880179009	0,868372749757868	0,866630143472121	0,762293309919491	-0.391581880179013	1,31183100650013	0.391581880179013	741
Q9DCJ1	sp|Q9DCJ1|LST8_MOUSE Target of rapamycin complex subunit LST8 OS=Mus musculus GN=Mlst8 PE=1 SV=1	−1,31	0,445941255365619	0,784849692944763	26,3165025351457	26,9761562920847	26,7743864526719	27,0068439722442	26,1926661523628	26,0548622267635	389845012524,417	297334652385,777	0,390813067952477	0,0126648282369945	0,0194745500243807	0,762699644303271	-0.390813067952477	1,3111321795648	0.390813067952477	742
P99026	sp|P99026|PSB4_MOUSE Proteasome subunit beta type-4 OS=Mus musculus GN=Psmb4 PE=1 SV=1	−1,31	0,108233964788213	0,472983636494304	30,4225096278016	30,1444136462248	30,0424084078288	29,6831401315376	30,0602349868501	30,0535494719522	13093153299558	9987028299107,71	0,390685227370729	0,0065136127325647	0,007209994057615	0,76276723189706	-0.390685227370724	1,31101594062048	0.390685227370724	743
P14131	sp|P14131|RS16_MOUSE 40S ribosomal protein S16 OS=Mus musculus GN=Rps16 PE=2 SV=4	−1,31	0,409043760156646	0,76291790520755	28,399855058049	29,1319348639858	28,5498933163116	28,1954506384403	28,1709716265096	28,9032203325384	2894676316070,51	2208233960635,61	0,390509001855438	0,0134767597299562	0,0146316193954888	0,762860409772262	-0.390509001855441	1,31085580951636	0.390509001855441	744
P70460	sp|P70460|VASP_MOUSE Vasodilator-stimulated phosphoprotein OS=Mus musculus GN=Vasp PE=1 SV=4	−1,31	0,151406201291301	0,552437900049208	26,5816409484069	26,0204577171094	26,2312364204502	25,8693686586924	25,9677083343354	26,1853542778432	258400969811,643	197198582368,883	0,389962304225422	0,0107875106309738	0,006217562045066	0,763149544340439	-0.38996230422542	1,3103591654035	0.38996230422542	745
P16220-3	sp|P16220-3|CREB1_HUMAN Isoform 3 of Cyclic AMP-responsive element-binding protein 1 OS=Homo sapiens GN=CREB1;sp|Q01147-2|CREB1_MOUSE Isoform 2 of Cyclic AMP-responsive element-binding protein 1 OS=Mus musculus GN=Creb1;sp|P16220-2|CREB1_HUMAN Isoform C	−1,31	0,178210258962166	0,597412382148649	26,1383002868415	25,4876194191801	25,7191474648085	25,5599556845734	25,587817854652	25,3877731654233	157342226549,178	120131030103,538	0,389297054019314	0,0127927270268296	0,004247126448206	0,76350152618433	-0.389297054019311	1,30975507671555	0.389297054019311	746
O35435	sp|O35435|PYRD_MOUSE Dihydroorotate dehydrogenase (quinone), mitochondrial OS=Mus musculus GN=Dhodh PE=2 SV=2	−1,31	0,138498518687842	0,529162269633094	26,5056129060047	26,140845402906	26,190885557456	25,7471346094699	26,2312364204502	26,0516722330455	258746491528,066	197699575709,675	0,388229525677822	0,00752470295241422	0,009408863823401	0,764066691463643	-0.388229525677818	1,30878627634507	0.388229525677819	747
O35681	sp|O35681|SYT3_MOUSE Synaptotagmin-3 OS=Mus musculus GN=Syt3 PE=1 SV=2	−1,31	0,119042161817671	0,493003989498548	24,9428329797975	25,2084612346714	24,9676751874106	24,5185951445093	25,0371457729701	24,758319535241	74917733911,8664	57287797750,1769	0,387079383106342	0,00585934369499128	0,0104766543210282	0,764676062113285	-0.387079383106341	1,30774330405527	0.387079383106341	748
Q8BHN3	sp|Q8BHN3|GANAB_MOUSE Neutral alpha-glucosidase AB OS=Mus musculus GN=Ganab PE=1 SV=1;sp|Q8BHN3-2|GANAB_MOUSE Isoform 2 of Neutral alpha-glucosidase AB OS=Mus musculus GN=Ganab;sp|Q8BHN3-3|GANAB_MOUSE Isoform 3 of Neutral alpha-glucosidase AB OS=Mus mus	−1,31	0,627451097397197	0,876847454152056	31,246940384094	29,7621840572139	30,7404493890883	30,5191558658654	30,7747464720785	29,6508210992447	19147461744646,7	14641891433533,5	0,387051223646075	0,0246791380779649	0,0194341166854301	0,764690987703742	-0.387051223646076	1,3077177789199	0.387051223646076	749
Q9CPX6	sp|Q9CPX6|ATG3_MOUSE Ubiquitin-like-conjugating enzyme ATG3 OS=Mus musculus GN=Atg3 PE=1 SV=1;sp|Q9NT62|ATG3_HUMAN Ubiquitin-like-conjugating enzyme ATG3 OS=Homo sapiens GN=ATG3 PE=1 SV=1;sp|Q9NT62-2|ATG3_HUMAN Isoform 2 of Ubiquitin-like-conjugating en	−1,31	0,267190026051519	0,685404159524725	28,0971533682127	27,7477528357763	28,4815245373331	27,9689280949635	27,9434810304077	27,6110235759658	1612505420413,38	1233829957098,08	0,386160430043322	0,0130572837953992	0,007172696738189	0,765163292773166	-0.386160430043328	1,30691057640222	0.386160430043327	750
Q9CXI5	sp|Q9CXI5|MANF_MOUSE Mesencephalic astrocyte-derived neurotrophic factor OS=Mus musculus GN=Manf PE=1 SV=1	−1,31	0,0350723813351076	0,273707222546134	27,7421650657753	27,7048655608525	27,6110235759658	27,1789632879833	27,5118748975731	27,5648173464815	1056537393924,83	808585162212,563	0,385872194275604	0,00244064589064261	0,007628832496503	0,765316179873982	-0.385872194275598	1,30664949506838	0.385872194275598	751
Q8CIG8	sp|Q8CIG8|ANM5_MOUSE Protein arginine N-methyltransferase 5 OS=Mus musculus GN=Prmt5 PE=1 SV=3	−1,31	0,109556325669918	0,474953354551822	28,3855524287391	28,3824394113629	28,0407745775257	28,2288491072506	27,8119809890143	27,966093558593	1893766475075,2	1449598713795,86	0,385604859140272	0,00700983126553995	0,007527249986775	0,765458008088509	-0.385604859140277	1,30640739195764	0.385604859140277	752
Q99KD5	sp|Q99KD5|UN45A_MOUSE Protein unc-45 homolog A OS=Mus musculus GN=Unc45a PE=1 SV=2	−1,31	0,567447827526238	0,845527592693687	24,7957915615014	24,7822925530271	24,1103167979477	23,6978719068565	25,0758097375073	24,1143670029103	46503820346,7119	35614411694,2531	0,384888081323831	0,0159558490619033	0,0290870906916013	0,765838407011033	-0.384888081323831	1,30575848748938	0.384888081323831	753

Q9NZI2-3	sp|Q9NZI2-3|KCIP1_HUMAN Isoform 3 of Kv channel-interacting protein 1 OS=Homo sapiens GN=KCNIP1;sp|Q9JJ57-3|KCIP1_MOUSE Isoform 3 of Kv channel-interacting protein 1 OS=Mus musculus GN=Kcnip1;sp|Q9JJ57-2|KCIP1_MOUSE Isoform 2 of Kv channel-interacting p	−1,31	0,0465840373374408	0,314182682726068	25,3115808292264	25,6338439500692	25,4023621789469	24,9975459496661	25,2978380035958	25,2526747161242	112842888327,29	86437394266,0243	0,384588012130606	0,00652953662365927	0,0064297574248199	0,765997711927764	-0.3845880121306	1,30548692826161	0.384588012130601	754
P84084	sp|P84084|ARF5_MOUSE ADP-ribosylation factor 5 OS=Mus musculus GN=Arf5 PE=1 SV=2	−1,31	0,226702195595698	0,652505558534478	30,3147859837767	29,8475138120678	29,8297582429479	29,4384942443743	29,9548618864297	29,7991526231768	10658221473349	8164660416350,59	0,384501929381329	0,00916909636917435	0,0089091297391713	0,766043418854301	-0.384501929381327	1,30540903477195	0.384501929381327	755
P28740-1	sp|P28740-1|KIF2A_MOUSE Isoform 1 of Kinesin-like protein KIF2A OS=Mus musculus GN=Kif2a	−1,30	0,157634822635172	0,562098346210542	25,4374912672445	24,882110644788	24,8971877540398	24,8955478776018	24,7487693428392	24,7747333955639	77400820791,2601	59328177892,0235	0,383631390485201	0,0126189880024142	0,0031576881926217	0,766505797813486	-0.383631390485201	1,30462157344742	0.383631390485201	756
Q8C2Q3	sp|Q8C2Q3|RBM14_MOUSE RNA-binding protein 14 OS=Mus musculus GN=Rbm14 PE=1 SV=1;sp|Q8C2Q3-2|RBM14_MOUSE Isoform 2 of RNA-binding protein 14 OS=Mus musculus GN=Rbm14	−1,30	0,266197850628539	0,685404159524725	28,6351446174494	28,8266770279669	28,0831095688452	28,3918361374394	28,2613796269497	28,0948693999158	2420456008040,64	1855845904837,59	0,383201948208111	0,0135393495765504	0,0052690636516689	0,766733995029266	-0.383201948208111	1,3042332888368	0.383201948208111	757
P32261	sp|P32261|ANT3_MOUSE Antithrombin-III OS=Mus musculus GN=Serpinc1 PE=1 SV=1	−1,30	0,386943170375765	0,752468171504595	24,6240536772299	25,5890196137344	25,2737175242021	24,9892097138287	24,6655484483854	25,035270903413	84690020086,3985	64936542140,1836	0,383161408276762	0,0195547980229643	0,008092776036884	0,766755540663907	-0.383161408276762	1,30419664021487	0.383161408276762	758
Q9D883	sp|Q9D883|U2AF1_MOUSE Splicing factor U2AF 35 kDa subunit OS=Mus musculus GN=U2af1 PE=1 SV=4	−1,30	0,0207129079558373	0,199524718486067	27,139097165185	27,1320858547966	27,4793096785935	26,9868342577459	26,9837240922818	26,984703803127	683275670938,899	524172876784,816	0,38242505474714	0,00728350617385436	5,89×10^−05	0,767146993635149	-0.382425054747141	1,30353114630805	0.382425054747141	759
Q8R146-2	sp|Q8R146-2|APEH_MOUSE Isoform 2 of Acylamino-acid-releasing enzyme OS=Mus musculus GN=Apeh;sp|Q8R146|APEH_MOUSE Acylamino-acid-releasing enzyme OS=Mus musculus GN=Apeh PE=2 SV=3	−1,30	0,0669991530346851	0,384081971353649	28,5614572625613	28,3022437834415	28,0865111619099	28,1372043839272	27,9843470437695	28,0347736742627	1985193293961,81	1523617210330,8	0,381778996928143	0,00839802039333479	0,0027765859414583	0,767490609083284	-0.381778996928145	1,30294753859521	0.381778996928145	760
P60766-1	sp|P60766-1|CDC42_MOUSE Isoform 1 of Cell division control protein 42 homolog OS=Mus musculus GN=Cdc42	−1,30	0,024974455690271	0,219211300821997	31,2428831501822	31,1930833520968	31,0176093622394	30,7592274156069	31,0751300491871	30,82620630466	33790200651857,6	25941226861342,4	0,381358205638108	0,00379873979898905	0,0053891870576693	0,767714495945625	-0.381358205638105	1,30256756291707	0.381358205638106	761
Q9QWR8	sp|Q9QWR8|NAGAB_MOUSE Alpha-N-acetylgalactosaminidase OS=Mus musculus GN=Naga PE=2 SV=2	−1,30	0,125882632287745	0,507443399603972	25,1986693862256	25,30131699854	25,3346416265303	24,8524446071923	25,3493149492024	24,8404515320987	95101381870,1835	73025195532,7988	0,381071988281229	0,00280360848189312	0,0116091561378959	0,767866818512486	-0.38107198828123	1,30230917118831	0.38107198828123	762
Q922B1	sp|Q922B1|MACD1_MOUSE O-acetyl-ADP-ribose deacetylase MACROD1 OS=Mus musculus GN=Macrod1 PE=1 SV=2	−1,30	0,130805275628012	0,514825425964414	26,5121165550575	26,6033375672616	26,5728087866134	26,417345126627	25,9585083386998	26,5203006373062	343603530824,602	263868849434,675	0,380923815564244	0,00174809546539498	0,0113729317562914	0,767945686708822	-0.380923815564246	1,30217542374083	0.380923815564246	763
A3KMP2	sp|A3KMP2|TTC38_MOUSE Tetratricopeptide repeat protein 38 OS=Mus musculus GN=Ttc38 PE=2 SV=2;sp|A3KMP2-2|TTC38_MOUSE Isoform 2 of Tetratricopeptide repeat protein 38 OS=Mus musculus GN=Ttc38	−1,30	0,000915185578230087	0,0222140499443121	26,3210361882821	26,1926661523628	26,2677834736084	25,8889079988918	26,1288765661872	25,9719342937484	253973373928,684	195059982922,927	0,380759420044241	0,00245595565182537	0,0046878039367067	0,768033199329391	-0.380759420044236	1,30202704892595	0.380759420044236	764
Q9QXS6	sp|Q9QXS6|DREB_MOUSE Drebrin OS=Mus musculus GN=Dbn1 PE=1 SV=4;sp|Q9QXS6-3|DREB_MOUSE Isoform E2 of Drebrin OS=Mus musculus GN=Dbn1;sp|Q9QXS6-2|DREB_MOUSE Isoform A2 of Drebrin OS=Mus musculus GN=Dbn1	−1,30	0,336237259442746	0,72953198881961	29,7220781608845	28,8067679360488	29,4570920219591	29,1173946133026	29,0776180365338	29,0002145179698	5460961776040,39	4195676568587,06	0,380251589302901	0,0160586959089134	0,0020501714147556	0,768303595713728	-0.380251589302899	1,30156881417564	0.380251589302899	765
O88848	sp|O88848|ARL6_MOUSE ADP-ribosylation factor-like protein 6 OS=Mus musculus GN=Arl6 PE=2 SV=1;sp|O88848-2|ARL6_MOUSE Isoform 2 of ADP-ribosylation factor-like protein 6 OS=Mus musculus GN=Arl6	−1,30	0,210413916518315	0,641644645910089	27,7477528357763	27,7259995969464	27,5424060245662	27,0177302526787	27,5376339052084	27,6721550228499	1041885136081,09	801037068118,614	0,37925532444386	0,00407642367537099	0,0126091933018642	0,768834337277914	-0.379255324443854	1,30067031545513	0.379255324443854	766
Q8C6E0	sp|Q8C6E0|CFA36_MOUSE Cilia- and flagella-associated protein 36 OS=Mus musculus GN=Cfap36 PE=2 SV=1	−1,30	0,149020526728242	0,547695684874083	26,7056831906351	26,190885557456	26,1853542778432	26,0346229337524	26,2142780507656	26,0446839509163	280724895918,352	215852608821,356	0,379110484569447	0,0113361466851051	0,0038679409661386	0,768911528545498	-0.379110484569447	1,30053974075748	0.379110484569447	767
	sp|Q9Z2D6-2|MECP2_MOUSE Isoform B of Methyl-CpG-binding protein 2 OS=Mus musculus GN=Mecp2;sp|Q9Z2D6|MECP2_MOUSE Methyl-CpG-																			

Q9Z2D6-2	binding protein 2 OS=Mus musculus GN=Mecp2 PE=1 SV=1;sp|P51608|MECP2_HUMAN Methyl-CpG-binding protein 2 OS=Homo sapiens GN=MECP2	−1,30	0,368027587687872	0,739685989256097	24,0659982136174	23,7476238449328	24,1749746788762	24,0670079530992	23,2165788422459	23,917090549966	26388625776,8972	20293350649,0734	0,378909133208358	0,00925401227011226	0,0191269088327362	0,769018850039546	-0.378909133208358	1,30035824212706	0.378909133208358	768
Q63829	sp|Q63829|COMD3_MOUSE COMM domain-containing protein 3 OS=Mus musculus GN=Commd3 PE=2 SV=1	−1,30	0,216587919433569	0,64735591278721	27,1813739338955	27,1516611790053	26,9898009594195	26,4743118091212	26,8845106514255	27,1769933127962	592497223641,022	455778108766,045	0,37847676080459	0,00380348266787522	0,013148694742478	0,769249357769461	-0.378476760804591	1,29996858612873	0.378476760804591	769
P70459	sp|P70459|ERF_MOUSE ETS domain-containing transcription factor ERF OS=Mus musculus GN=Erf PE=2 SV=1	−1,30	0,188638021381279	0,613397297160539	23,6274438234	23,6408917707753	23,1560227153882	23,4522005639565	23,0956367782024	23,0900203571021	15666430935,2323	12053479773,6016	0,378226843379876	0,0117632197704122	0,0089392118951649	0,769382626038614	-0.378226843379868	1,29974341264864	0.378226843379868	770
Q9R0P5	sp|Q9R0P5|DEST_MOUSE Destrin OS=Mus musculus GN=Dstn PE=1 SV=3	−1,30	0,0408175969068099	0,29833822489943	32,3661802272999	32,099937215965	31,9827023770707	31,7691737448374	31,9593241419042	31,9339369527224	91705787515207,5	70559559156204,2	0,378171237377657	0,00611219645799312	0,0032375740561754	0,769412281035189	-0.378171237377656	1,29969331741699	0.378171237377656	771
Q91VH2	sp|Q91VH2|SNX9_MOUSE Sorting nexin-9 OS=Mus musculus GN=Snx9 PE=1 SV=1	−1,30	0,028917003010386	0,238644139389349	24,7832884827572	24,845323645973	24,4941949562678	24,534409034057	24,3570462707292	24,4468590871809	53749616103,3799	41381708535,1351	0,377261239283028	0,0075847501020016	0,0036277267177845	0,769897750628454	-0.377261239283031	1,2988737795781	0.37726123928303	772
P35821	sp|P35821|PTN1_MOUSE Tyrosine-protein phosphatase non-receptor type 1 OS=Mus musculus GN=Ptpn1 PE=1 SV=2	−1,30	0,479629884389161	0,800971319073732	24,0132687336335	25,0796635391775	24,4958624117327	23,8790570595803	24,5687279499805	24,3570462707292	44985148017,3474	34640075265,5057	0,377006705185088	0,0217695060556521	0,0145581330088966	0,770033595357908	-0.377006705185086	1,29864463840075	0.377006705185086	773
Q9D967	sp|Q9D967|MGDP1_MOUSE Magnesium-dependent phosphatase 1 OS=Mus musculus GN=Mdp1 PE=1 SV=1	−1,30	0,252144051288691	0,67459732861346	28,5524438127231	28,1207709623458	28,304589714348	27,6889296233201	28,1954506384403	28,3097572274689	2003536189112,65	1542942612422,84	0,376864164959656	0,00764764079589459	0,0117734336492629	0,770109679479359	-0.376864164959653	1,29851633688861	0.376864164959653	774
Q8WTY4	sp|Q8WTY4|CPIN1_MOUSE Anamorsin OS=Mus musculus GN=Ciapin1 PE=1 SV=1	−1,30	0,315998666824239	0,722907157640623	28,8685229978762	28,2731322206399	28,3382640165987	27,9358995293517	28,5539886874427	28,2082847129067	2368567390455,62	1825224866512,98	0,375940506017876	0,0114614596089423	0,0109719662351654	0,770602885891238	-0.375940506017875	1,29768525177978	0.375940506017875	775
Q80U28-3	sp|Q80U28-3|MADD_MOUSE Isoform 3 of MAP kinase-activating death domain protein OS=Mus musculus GN=Madd;sp|Q80U28-15|MADD_MOUSE Isoform 15 of MAP kinase-activating death domain protein OS=Mus musculus GN=Madd;sp|Q80U28|MADD_MOUSE MAP kinase-activating de	−1,30	0,315557673973208	0,722907157640623	23,1467880451938	23,5193424313764	23,252854447264	22,9902222541556	23,4769036420011	22,672615926723	13237615961,9453	10209460298,3865	0,374736719131335	0,0082358465821333	0,0175772236956021	0,771246146416095	-0.374736719131341	1,29660291289221	0.374736719131341	776
O08992	sp|O08992|SDCB1_MOUSE Syntenin-1 OS=Mus musculus GN=Sdcbp PE=1 SV=1	−1,30	0,0728976835114748	0,403023855613379	27,2152024674969	26,9164428189902	27,0553730818285	26,9998623123902	26,8109304958059	26,5982162339486	566271485269,827	436914348531,623	0,374143398877348	0,00552434147416131	0,0074969344381838	0,771563392995913	-0.374143398877348	1,29606978386713	0.374143398877348	777
Q60865	sp|Q60865|CAPR1_MOUSE Caprin-1 OS=Mus musculus GN=Caprin1 PE=1 SV=2	−1,30	0,222843319815045	0,652238880554131	28,4120046915943	28,0563316653839	27,7067011958313	27,8187164630971	27,8935880061131	27,6856496210637	1533150448518,34	1183287893428,97	0,373698152585667	0,0125686690073877	0,0037884993199394	0,771801550573538	-0.373698152585668	1,295669850957	0.373698152585668	778
P56395	sp|P56395|CYB5_MOUSE Cytochrome b5 OS=Mus musculus GN=Cyb5a PE=1 SV=2	−1,30	0,0391578438864606	0,289869612893965	27,2883154174475	26,9028762084041	26,9612195741421	26,8359983838767	26,7178490875963	26,8215162251178	559776667632,347	432041678637,192	0,37368085989816	0,00767997356711657	0,0024052524124968	0,771810801733792	-0.37368085989816	1,29565432066201	0.37368085989816	779
Q8R2H9	sp|Q8R2H9|PHOP1_MOUSE Phosphoethanolamine/phosphocholine phosphatase OS=Mus musculus GN=Phospho1 PE=2 SV=1	−1,30	0,785125350760303	0,941030886480332	23,8956506099416	25,3070616187359	27,1464541510055	25,4412526778006	25,1183229982551	25,0126127548663	112894786224,407	87135435668,6787	0,373647411185874	0,0640514053167455	0,0088645658461345	0,771828696282528	-0.373647411185874	1,2956242814195	0.373647411185874	780
Q8R5C5	sp|Q8R5C5|ACTY_MOUSE Beta-centractin OS=Mus musculus GN=Actr1b PE=1 SV=1	−1,30	0,0843058942506444	0,431901189620401	30,5297954510822	30,0707031025332	30,2028177448941	30,0350880404903	30,1011524211797	29,8904491527376	13967748361446,4	10781971375745,5	0,373478488591921	0,00780824582905001	0,0035911276498994	0,771919073621469	-0.373478488591923	1,29547258795988	0.373478488591923	781
Q9BCZ4	sp|Q9BCZ4|SELS_MOUSE Selenoprotein S OS=Mus musculus GN=Vimp PE=2 SV=3	−1,29	0,595898971236679	0,860936823333794	24,0597088731534	23,9166756183545	23,0804133432428	22,8107698771548	23,3535376645311	24,1179472560925	19343055018,9951	14941647214,9699	0,372476466131643	0,0223326951865545	0,028031811625264	0,772455395504848	-0.372476466131641	1,29457313110803	0.372476466131641	782
Q9CR86	sp|Q9CR86|CHSP1_MOUSE Calcium-regulated heat stable protein 1 OS=Mus musculus GN=Carhsp1 PE=1 SV=1	−1,29	0,0349369549433214	0,273411790496096	26,1207629911894	25,9864544591337	25,8289851792492	25,5484527128798	25,7725191212397	25,8417503358081	191611210340,845	148063546969,998	0,371965474451229	0,00562159915011091	0,0059604884455549	0,772729041827027	-0.371965474451226	1,29411468428263	0.371965474451226	783
P55258	sp|P55258|RAB8A_MOUSE Ras-related protein Rab-8A OS=Mus musculus GN=Rab8a PE=1 SV=2	−1,29	0,220941669090114	0,650359426718565	27,9723912373162	27,6294369835375	27,8772178137753	27,5035939037474	27,2883154174475	27,9148680147052	1215709141735,5	939793285170,158	0,371382740629947	0,00636251765306107	0,0115472222859385	0,773041225821947	-0.371382740629951	1,29359207063858	0.371382740629951	784
P20444	sp|P20444|KPCA_MOUSE Protein kinase C alpha type OS=Mus musculus GN=Prkca PE=1 SV=3	−1,29	0,134969845187415	0,521116154813056	29,9092926391212	29,6015013481049	29,3179435122904	29,3143252751957	29,3868050777055	29,356105721571	7232306842095,84	5592301439090,9	0,371013759983447	0,0099885684735369	0,0012394485743263	0,773238962503742	-0.371013759983447	1,29326126655854	0.371013759983447	785
O54833	sp|O54833|CSK22_MOUSE Casein kinase II subunit alpha OS=Mus musculus GN=Csnk2a2 PE=2 SV=1	−1,29	0,028549079275132	0,238644139389349	29,9571855137959	29,7220781608845	29,7016909671295	29,465248371337	29,7040018754618	29,441616430095	8693976515580,17	6725689465851,73	0,370334029344306	0,00476581205245185	0,0049141185225449	0,773603362488829	-0.370334029344305	1,29265208566676	0.370334029344305	786

Q505B7-2	sp|Q505B7-2|ARCH_MOUSE Isoform 2 of Protein archease OS=Mus musculus GN=Zbtb8os;sp|Q505B7|ARCH_MOUSE Protein archease OS=Mus musculus GN=Zbtb8os PE=2 SV=2	−1,29	0,0302112395406876	0,247078948243569	25,4600043280656	25,35089230623	25,2783415939017	25,1330874985912	25,2754018283628	24,9114903418552	103525045863,766	80109468266,002	0,369935169596892	0,00360533914454198	0,0073044162179366	0,773817269025142	-0.369935169596885	1,29229475746878	0.369935169596885	787
Q8BU88	sp|Q8BU88|RM22_MOUSE 39S ribosomal protein L22, mitochondrial OS=Mus musculus GN=Mrpl22 PE=2 SV=1	−1,29	0,154144824154428	0,557500682738605	24,3767709733963	24,3163006006741	24,5863610385453	23,9597534682194	24,0659982136174	24,4846098063473	40577417822,3302	31401468493,042	0,369845032436089	0,00580214036348988	0,0114806243524031	0,773865617337568	-0.369845032436089	1,29221401958706	0.369845032436089	788
Q9D1P4	sp|Q9D1P4|CHRD1_MOUSE Cysteine and histidine-rich domain-containing protein 1 OS=Mus musculus GN=Chordc1 PE=1 SV=1	−1,29	0,114314349547811	0,484023164736488	29,0951539669868	28,5486023400049	28,8233279788742	28,5775854342843	28,624551630105	28,496957284198	3291489877888,25	2548089001217	0,369325091321525	0,00948142629249557	0,0022590549582904	0,7741445654549	-0.369325091321529	1,29174839509773	0.369325091321529	789
P70697	sp|P70697|DCUP_MOUSE Uroporphyrinogen decarboxylase OS=Mus musculus GN=Urod PE=1 SV=2	−1,29	0,541131644911529	0,832408322819698	27,3283963991727	26,7618369074011	26,1720458506377	25,9967084867646	26,7131195606615	26,7858549456868	416059057553,255	322239533147,953	0,368654828103132	0,0216121759816817	0,0164588540922462	0,774504309659709	-0.368654828103129	1,29114839972855	0.368654828103129	790
Q9D0S9	sp|Q9D0S9|HINT2_MOUSE Histidine triad nucleotide-binding protein 2, mitochondrial OS=Mus musculus GN=Hint2 PE=1 SV=1	−1,29	0,0266625624254955	0,227483779791963	28,9124490208349	28,787551864115	28,6410826370046	28,4026187469632	28,6868977896118	28,485463756269	3156109373304,36	2444821991146,76	0,368417776482193	0,00471939947414502	0,0051254677044233	0,774631580206328	-0.368417776482194	1,29093626641667	0.368417776482194	791
Q91WM2	sp|Q91WM2|CECR5_MOUSE Cat eye syndrome critical region protein 5 homolog OS=Mus musculus GN=Cecr5 PE=2 SV=1	−1,29	0,120142252061997	0,494850891206355	25,8593137304936	25,3625137240185	25,4703197299504	25,2227384535695	25,4354333008474	25,2687133583084	126568444430,94	98071408088,4928	0,368013265291898	0,01022415562456	0,0044219080816176	0,77484880634686	-0.3680132652919	1,29057435696991	0.3680132652919	792
Q91V76	sp|Q91V76|CK054_MOUSE Ester hydrolase C11orf54 homolog OS=Mus musculus PE=2 SV=1	−1,29	0,100494348139509	0,46324407107743	27,4505481052565	27,2773560240938	27,7082701854407	27,343085730949	27,0435496348728	27,2849017576652	858733762147,676	665527441158,809	0,367712761324461	0,00789099545195733	0,0058341518183088	0,775010219109516	-0.367712761324461	1,29030556674336	0.367712761324461	793
Q9WVR4	sp|Q9WVR4|FXR2_MOUSE Fragile X mental retardation syndrome-related protein 2 OS=Mus musculus GN=Fxr2 PE=1 SV=1	−1,29	0,298016708471759	0,71307213481988	26,9044098251012	26,4796440426437	26,3678330475435	26,5822533365469	26,4297525807184	25,9760107033085	350968497297,83	272073713903,441	0,367343962022424	0,0106489306397101	0,0119770787203562	0,775208361998828	-0.367343962022428	1,28997576525305	0.367343962022428	794
Q9CR21	sp|Q9CR21|ACPM_MOUSE Acyl carrier protein, mitochondrial OS=Mus musculus GN=Ndufab1 PE=1 SV=1	−1,29	0,268307191701669	0,687660272802866	26,1042219466073	25,6458690980175	25,549542709295	25,2509320978723	25,5041690285268	25,7816672426511	154977314430,292	120179959999,838	0,366860702539152	0,0115023873657931	0,0104051940977072	0,77546807699971	-0.36686070253915	1,28954373450034	0.36686070253915	795
P61164	sp|P61164|ACTZ_MOUSE Alpha-centractin OS=Mus musculus GN=Actr1a PE=2 SV=1	−1,29	0,0629808998066677	0,375566342186827	32,2512806916565	31,8619322988203	31,9425028325732	31,7107286381385	31,8989697166028	31,6843541136303	80443158151170,9	62406100822982,1	0,366282648207168	0,00641873325129874	0,0036845952607815	0,775778851269202	-0.366282648207168	1,2890271478321	0.366282648207168	796
Q8BFY9-2	sp|Q8BFY9-2|TNPO1_MOUSE Isoform 2 of Transportin-1 OS=Mus musculus GN=Tnpo1;sp|Q8BFY9|TNPO1_MOUSE Transportin-1 OS=Mus musculus GN=Tnpo1 PE=1 SV=2	−1,29	0,221227105063966	0,650415221831493	28,1810020312617	27,5899208736384	27,5359993615751	27,4766700566273	27,5424060245662	27,5261939943834	1147921565578,6	890536587403,731	0,366277279563809	0,0128864592403884	0,0012446205329664	0,775781738149386	-0.366277279563812	1,28902235103585	0.366277279563812	797
Q61205	sp|Q61205|PA1B3_MOUSE Platelet-activating factor acetylhydrolase IB subunit gamma OS=Mus musculus GN=Pafah1b3 PE=1 SV=1	−1,29	0,164764419554859	0,572638361684154	28,6120839248889	28,9475574493059	29,1855954237581	28,7995948141363	28,5402325045527	28,6442862543706	3611263881397,56	2802046589735,91	0,366022900686291	0,00996476321373711	0,004553951724264	0,775918537598398	-0.36602290068629	1,28879508807094	0.36602290068629	798
Q9WVA3	sp|Q9WVA3|BUB3_MOUSE Mitotic checkpoint protein BUB3 OS=Mus musculus GN=Bub3 PE=2 SV=2	−1,29	0,0225828634483929	0,210372034803286	29,0238572486725	28,6599523335218	28,8212517944698	28,6029701816381	28,6297384787753	28,5119947056563	3333421796619,05	2587123582796,96	0,365654910394881	0,00632365603621492	0,0021594584612420	0,776116477494979	-0.365654910394883	1,28846639518289	0.365654910394883	799
Q60972	sp|Q60972|RBBP4_MOUSE Histone-binding protein RBBP4 OS=Mus musculus GN=Rbbp4 PE=1 SV=5	−1,29	0,00118964215471712	0,0271346017098544	29,2745375866608	29,1687376261914	29,4005511866025	29,0635435904717	29,0726137546512	28,9475574493059	5208304393840,91	4042582884545	0,365536414364336	0,00396340553926895	0,0024021916718693	0,776180226586903	-0.365536414364337	1,28836057109738	0.365536414364337	800
Q8QZT1	sp|Q8QZT1|THIL_MOUSE Acetyl-CoA acetyltransferase, mitochondrial OS=Mus musculus GN=Acat1 PE=1 SV=1	−1,29	0,0110729054158262	0,132262941269566	33,4859878172862	33,1690884811052	33,2226868067924	33,0034445863308	33,0667878409893	33,0476252564011	287598838084688	223243873855930	0,36543726102959	0,00509482370871574	0,0009832120020427	0,776233573621714	-0.365437261029591	1,28827202788234	0.365437261029591	801
Q9CQY6	sp|Q9CQY6|UQCC2_MOUSE Ubiquinol-cytochrome-c reductase complex assembly factor 2 OS=Mus musculus GN=Uqcc2 PE=1 SV=1	−1,29	0,413689779644891	0,766530246241387	25,4189974157507	24,8569465581742	25,0419212187464	24,504203243946	24,690050667465	25,3648968031627	80053024973,3147	62164518896,9754	0,364864538334007	0,0114094308098803	0,0182234078416516	0,776541784869437	-0.364864538334007	1,28776070970622	0.364864538334007	802
P47957	sp|P47957|APT_MUSSI Adenine phosphoribosyltransferase OS=Mus spicilegus GN=Aprt PE=3 SV=1;sp|P08030|APT_MOUSE Adenine phosphoribosyltransferase OS=Mus musculus GN=Aprt PE=1 SV=2;sp|P47956|APT_MUSPA Adenine phosphoribosyltransferase OS=Mus pahari GN=Aprt	−1,29	0,00060170354393759	0,0160406138566499	28,1925735549002	28,0390196313671	28,2810559679654	27,8785137992401	27,9281151032895	27,9479032340272	1715769958879,53	1332632438247,42	0,364577220604851	0,00434730302479868	0,0012803922963596	0,776696451264181	-0.364577220604845	1,28750427322329	0.364577220604845	803

P12815-2	sp|P12815-2|PDCD6_MOUSE Isoform 2 of Programmed cell death protein 6 OS=Mus musculus GN=Pdcd6	−1,29	0,145809571614274	0,541152612884047	29,1101735891658	29,1101735891658	28,9855437086712	28,4788737237828	28,9940526281682	28,9750635875556	4210616945108,47	3270606790614,81	0,364473312812615	0,00247535210021113	0,0101371248835129	0,776752393592658	-0.364473312812616	1,28741154613605	0.364473312812616	804
Q8VE09	sp|Q8VE09|TT39C_MOUSE Tetratricopeptide repeat protein 39C OS=Mus musculus GN=Ttc39c PE=2 SV=2	−1,29	0,167601473423493	0,57675745858168	27,0421427414941	27,5796655021705	27,5736087892473	27,1665322140943	27,0652717104084	27,2058942963641	792511096350,537	615622265450,817	0,36438572417526	0,0112635968649712	0,0026724580764609	0,776799553073412	-0.364385724175259	1,28733338741442	0.364385724175259	805
Q9CQF4	sp|Q9CQF4|CF203_MOUSE Uncharacterized protein C6orf203 homolog OS=Mus musculus PE=1 SV=1	−1,29	0,447546670375005	0,78574459315198	26,4988608819045	26,485190850542	26,056977654842	25,5153070978226	26,2537200098112	26,5143677630458	276924237741,017	215120668591,026	0,364345186639346	0,00953687664762392	0,01985939190749	0,776821380265781	-0.364345186639348	1,28729721581281	0.364345186639348	806
P55302	sp|P55302|AMRP_MOUSE Alpha-2-macroglobulin receptor-associated protein OS=Mus musculus GN=Lrpap1 PE=1 SV=1	−1,29	0,0568914744176914	0,353736424574922	27,9171142157975	27,5713979478984	27,6192585353685	27,3015176496588	27,4953994502371	27,5544638957705	1074192393191,02	834801936520,646	0,363746558023921	0,00676175329091897	0,0048205279858932	0,77714377965456	-0.363746558023924	1,28676317842305	0.363746558023924	807
P17095	sp|P17095|HMGA1_MOUSE High mobility group protein HMG-I/HMG-Y OS=Mus musculus GN=Hmga1 PE=1 SV=4;sp|P17095-1|HMGA1_MOUSE Isoform HMG-Y of High mobility group protein HMG-I/HMG-Y OS=Mus musculus GN=Hmga1	−1,29	0,384904030023524	0,751739350763028	25,6205393435449	25,4993244819944	24,9261116536089	24,7568685258369	25,0998804841175	25,4336098486706	102042832958,436	79322391185,853	0,363374783747383	0,0146327795530575	0,0134830518881802	0,77734407097618	-0.363374783747377	1,28643162961829	0.363374783747377	808
Q91XD6	sp|Q91XD6|VPS36_MOUSE Vacuolar protein-sorting-associated protein 36 OS=Mus musculus GN=Vps36 PE=1 SV=1	−1,29	0,0883847728194617	0,441176105282424	27,2638746068392	26,8016686908167	26,8756565229089	26,7982568071736	26,6810725947884	26,7069466343535	521721595286,435	405650861189,528	0,363041599928492	0,00920178833567345	0,0023032057113409	0,777523615764492	-0.36304159992849	1,28613456842306	0.36304159992849	809
Q9DAK9	sp|Q9DAK9|PHP14_MOUSE 14 kDa phosphohistidine phosphatase OS=Mus musculus GN=Phpt1 PE=2 SV=1	−1,29	0,316184494873475	0,722907157640623	29,0789957161334	28,8685229978762	28,410000627506	28,2371702788332	28,7546109971162	28,6120839248889	3173447906829,28	2468475164466,03	0,362431030433539	0,011883481853878	0,0093667303464450	0,777852744692565	-0.36243103043354	1,28559037275781	0.36243103043354	810
P61924	sp|P61924|COPZ1_MOUSE Coatomer subunit zeta-1 OS=Mus musculus GN=Copz1 PE=2 SV=1	−1,29	0,361693192056392	0,735816953537952	28,6662319760378	28,0327491345595	28,100354168705	28,0287075649402	28,3342637623689	27,683736916332	1887822365396,52	1468950644524,99	0,361937097329145	0,0123068810717988	0,0116171962309487	0,778119102438142	-0.361937097329148	1,28515030265498	0.361937097329148	811
Q8BFR5	sp|Q8BFR5|EFTU_MOUSE Elongation factor Tu, mitochondrial OS=Mus musculus GN=Tufm PE=1 SV=1;sp|Q8BFR5-2|EFTU_MOUSE Isoform 2 of Elongation factor Tu, mitochondrial OS=Mus musculus GN=Tufm	−1,28	0,000116203045627228	0,00418607638176181	33,0839850192138	32,8687527944652	32,9459102626137	32,72254043549	32,7396907713201	32,6843873490794	207513191159336	161502141510427	0,361649753229304	0,0033077328192207	0,00086529517872811	0,77827409721833	-0.361649753229302	1,28489436250564	0.361649753229302	812
Q8BUL6	sp|Q8BUL6|PKHA1_MOUSE Pleckstrin homology domain-containing family A member 1 OS=Mus musculus GN=Plekha1 PE=2 SV=1;sp|Q8BUL6-2|PKHA1_MOUSE Isoform 2 of Pleckstrin homology domain-containing family A member 1 OS=Mus musculus GN=Plekha1	−1,28	0,53348633580083	0,8300431463043	25,1478806807997	24,0912088731561	24,1399594220105	24,6110501334689	23,9242273809443	24,0935577863108	41947429472,0753	32666364173,3985	0,360776516293019	0,0243868625520755	0,0147799424379165	0,778745314898133	-0.360776516293018	1,28411687475874	0.360776516293018	813
P63040	sp|P63040|CPLX1_MOUSE Complexin-1 OS=Mus musculus GN=Cplx1 PE=1 SV=1	−1,28	0,359218273935841	0,733314212552756	28,4598031167191	28,2385123208996	27,9943201847396	27,860378365634	27,6454609497024	28,4381222529815	1821859030061,86	1419492490741,66	0,360036115002128	0,00824754144633557	0,0146503762106444	0,779145075068438	-0.360036115002122	1,2834580259808	0.360036115002122	814
Q6ZPF3-3	sp|Q6ZPF3-3|TIAM2_MOUSE Isoform 3 of T-lymphoma invasion and metastasis-inducing protein 2 OS=Mus musculus GN=Tiam2;sp|Q6ZPF3-2|TIAM2_MOUSE Isoform 2 of T-lymphoma invasion and metastasis-inducing protein 2 OS=Mus musculus GN=Tiam2;sp|Q6ZPF3|TIAM2_MOUSE	−1,28	0,203541901711259	0,635648869851434	27,8080490843163	27,6959168630177	27,2268519579805	27,4591263032037	27,3454914633062	27,1802077444987	947354596313,42	738786764564,369	0,358746509268676	0,0111805352586155	0,0051321945053054	0,779841853767658	-0.358746509268676	1,28231127268778	0.358746509268676	815
Q3THG9	sp|Q3THG9|AASD1_MOUSE Alanyl-tRNA editing protein Aarsd1 OS=Mus musculus GN=Aarsd1 PE=1 SV=2	−1,28	0,0393389913070461	0,289869612893965	27,1995228618436	26,8968700970283	26,9524248585844	26,7786940840882	26,894872517499	26,6292631829283	540776878127,168	421721056206,089	0,358744411895438	0,00596351680230055	0,0049743376713795	0,779842987493481	-0.358744411895438	1,28230940847995	0.358744411895438	816
P84096	sp|P84096|RHOG_MOUSE Rho-related GTP-binding protein RhoG OS=Mus musculus GN=Rhog PE=2 SV=1	−1,28	0,116786886693893	0,488567445809752	26,2102331083515	25,9276015798895	25,7970224427502	25,5297811363375	25,8577700523384	25,8014926834204	191525292072,231	149368357600,766	0,35866036334562	0,008130503168255	0,0068166617309860	0,779888420921629	-0.358660363345613	1,28223470585479	0.358660363345613	817
P13020-2	sp|P13020-2|GELS_MOUSE Isoform 2 of Gelsolin OS=Mus musculus GN=Gsn;sp|P13020|GELS_MOUSE Gelsolin OS=Mus musculus GN=Gsn PE=1 SV=3	−1,28	0,0709163906383061	0,396415636831615	30,2626911460235	30,0239777147737	29,8158570876731	29,8229827561781	29,8416907060905	29,6954546184483	11058000578471,3	8633830319194,93	0,357017907391416	0,00744456956640706	0,0026716806184957	0,780776801188095	-0.357017907391411	1,28077575880625	0.357017907391411	818
P58069	sp|P58069|RASA2_MOUSE Ras GTPase-activating protein 2 OS=Mus musculus GN=Rasa2 PE=1 SV=2	−1,28	0,122648959677407	0,49968194743413	25,8727859475999	25,6027289502231	25,3532998395674	25,4094933692325	25,426892640985	25,2509320978723	132467730917,079	103458806889,761	0,356584503309289	0,0101450726808533	0,0038229447573951	0,781011391782073	-0.356584503309296	1,28039105513973	0.356584503309296	819

A2BE28-2	sp|A2BE28-2|LAS1L_MOUSE Isoform 2 of Ribosomal biogenesis protein LAS1L OS=Mus musculus GN=Las1l;sp|A2BE28|LAS1L_MOUSE Ribosomal biogenesis protein LAS1L OS=Mus musculus GN=Las1l PE=1 SV=1	−1,28	0,610770485159355	0,866610575848175	25,5244369585765	24,8701728545749	24,0516859730864	24,817993141014	24,1039857732243	24,7832884827572	59869434043,318	46766008677,3173	0,356359327439265	0,029735525976905	0,0163864044608477	0,78113330156888	-0.356359327439265	1,28019122727393	0.356359327439265	820
P31786	sp|P31786|ACBP_MOUSE Acyl-CoA-binding protein OS=Mus musculus GN=Dbi PE=1 SV=2	−1,28	0,60653996080769	0,865805356251592	31,0218212612287	30,5898070211484	30,4559240288616	29,6508210992447	30,4732906815785	31,2024247921078	21288414143810	16629159349974,1	0,356353243625763	0,00963687462720555	0,025499803602946	0,781136595597908	-0.356353243625768	1,28018582874685	0.356353243625768	821
P98192	sp|P98192|GNPAT_MOUSE Dihydroxyacetone phosphate acyltransferase OS=Mus musculus GN=Gnpat PE=2 SV=1	−1,28	0,50035372890196	0,812083935271593	24,1795012645539	25,3545598909119	24,9961132911365	24,6044469627772	24,7917929036	24,3934704398956	61566977377,011	48101062446,1111	0,356087981069851	0,0242114392459	0,0081018622098750	0,781280233258161	-0.35608798106985	1,27995046774666	0.35608798106985	822
O70493	sp|O70493|SNX12_MOUSE Sorting nexin-12 OS=Mus musculus GN=Snx12 PE=1 SV=1	−1,28	0,140820443618781	0,532098608288336	29,5156147134624	29,4260099986843	29,2370422808287	28,9044917431211	29,4085535576116	29,126082642873	5823304486079,53	4551035844904,25	0,355643106356495	0,0048382111468806	0,0086680730372741	0,781521188834173	-0.355643106356495	1,27955583839223	0.355643106356495	823
Q8BWQ6	sp|Q8BWQ6|CP062_MOUSE UPF0505 protein C16orf62 homolog OS=Mus musculus PE=2 SV=2;sp|Q8BWQ6-3|CP062_MOUSE Isoform 3 of UPF0505 protein C16orf62 homolog OS=Mus musculus	−1,28	0,497932711462517	0,809992604135071	27,3623338424334	26,1684627487745	26,6267436086175	26,6107194319358	26,5444468376233	26,262865549841	401783838836,617	314005793440,957	0,355628357711796	0,0225410867113451	0,0069769684916134	0,78152917835166	-0.355628357711795	1,27954275758344	0.355628357711796	824
Q99MR0	sp|Q99MR0|ACL6B_MOUSE Actin-like protein 6B OS=Mus musculus GN=Actl6b PE=1 SV=1	−1,28	0,0544074857882871	0,341924788314513	27,097740617644	26,9015107380824	26,6820304486377	26,6160829578104	26,7355071353139	26,5902926789079	478422754140,626	373914970662,912	0,355575772394489	0,00773277580721842	0,0029074350274220	0,781557665112652	-0.355575772394491	1,27949611991313	0.355575772394491	825
P97930	sp|P97930|KTHY_MOUSE Thymidylate kinase OS=Mus musculus GN=Dtymk PE=2 SV=2	−1,28	0,25347933677332	0,674868697133729	27,0652717104084	27,311293764426	26,886722967848	26,6267436086175	26,7056831906351	27,1925405628277	580852572777,129	454132929033,735	0,355057387189478	0,00786986748931504	0,011416126503995	0,781838542717421	-0.355057387189478	1,27903645748177	0.355057387189478	826
Q8VEB1	sp|Q8VEB1|GRK5_MOUSE G protein-coupled receptor kinase 5 OS=Mus musculus GN=Grk5 PE=1 SV=2	−1,28	0,322299695896595	0,725294159481728	20,8724090496779	21,430109728472	20,9840740472232	20,9996327546211	21,0597463648342	20,4890801292557	1451017602,10449	1134532395,03543	0,354967216854738	0,0139879803185318	0,0150394531499156	0,781887410180246	-0.354967216854745	1,2789565185216	0.354967216854745	827
Q9JJF0	sp|Q9JJF0|NP1L5_MOUSE Nucleosome assembly protein 1-like 5 OS=Mus musculus GN=Nap1l5 PE=2 SV=1	−1,28	0,196868745948068	0,626219869672844	25,4105254331557	25,7441964423723	26,0548622267635	25,5700819737257	25,4294533770172	25,4719455493022	150394553458,134	117592798506,753	0,354952609848398	0,0125206024991322	0,0028295157073439	0,781895326678089	-0.3549526098484	1,27894356940147	0.3549526098484	828
Q8BGG7	sp|Q8BGG7|UBS3B_MOUSE Ubiquitin-associated and SH3 domain-containing protein B OS=Mus musculus GN=Ubash3b PE=1 SV=1;sp|Q8BGG7-2|UBS3B_MOUSE Isoform 2 of Ubiquitin-associated and SH3 domain-containing protein B OS=Mus musculus GN=Ubash3b	−1,28	0,19553231139367	0,623259242567324	25,3648968031627	25,9967084867646	25,7624652702272	25,4471000951404	25,3937401987218	25,5463841594127	146168151721,839	114336095162,377	0,354348074764499	0,0124243236374873	0,0030423207286260	0,782223034330768	-0.354348074764502	1,27840776365727	0.354348074764502	829
Q9D0P8	sp|Q9D0P8|IFT27_MOUSE Intraflagellar transport protein 27 homolog OS=Mus musculus GN=Ift27 PE=1 SV=1	−1,28	0,498837344085131	0,810677660151239	23,29481600134	23,7427329093668	23,5647953275257	22,6368476224383	23,4589281980348	23,7711154655879	16624024516,6645	13009735022,1674	0,353678108968332	0,00958307016190925	0,02515886522259	0,782586371256009	-0.353678108968328	1,27781422821746	0.353678108968328	830
P62908	sp|P62908|RS3_MOUSE 40S ribosomal protein S3 OS=Mus musculus GN=Rps3 PE=1 SV=1	−1,28	0,341341877172193	0,729721949431128	30,0580803905391	30,5960927494958	30,7933119851167	30,4157949618138	29,9739816452622	30,3224336631228	17313281535763,5	13549942550091	0,35359246231025	0,0124842746848458	0,0077009571249272	0,782632831453778	-0.353592462310246	1,27773837208241	0.353592462310246	831
Q9CR30	sp|Q9CR30|JOS2_MOUSE Josephin-2 OS=Mus musculus GN=Josd2 PE=2 SV=1	−1,28	0,304342337316736	0,716532404521184	24,6534719708188	24,3532110414183	24,0531878454148	23,7818895341833	24,2472581965185	24,295877056704	37713738666,7524	29520229050,2067	0,353386260453633	0,0123244977177421	0,0117701576752533	0,782744699777831	-0.353386260453634	1,27755576024192	0.353386260453634	832
Q9DCA2	sp|Q9DCA2|RT11_MOUSE 28S ribosomal protein S11, mitochondrial OS=Mus musculus GN=Mrps11 PE=2 SV=2	−1,28	0,251214606085698	0,673538059877323	25,5098805984377	25,1535971658607	24,9566019926909	24,7957915615014	24,8516604772475	25,2380141227444	88537608396,8471	69307714260,0683	0,353274463730545	0,0111254117135533	0,0096473337707877	0,782805358254249	-0.353274463730546	1,2774567642589	0.353274463730546	833
P21836	sp|P21836|ACES_MOUSE Acetylcholinesterase OS=Mus musculus GN=Ache PE=1 SV=1	−1,28	0,365064925992356	0,739315231400715	24,2341635524642	25,0338656906186	24,9224630227272	24,4164001122609	24,3532110414183	24,6867200139736	54976083854,6894	43042064667,6042	0,353056858508301	0,0175148662105511	0,0072349339520673	0,782923439606416	-0.353056858508301	1,27726409686075	0.353056858508301	834
Q8BK67	sp|Q8BK67|RCC2_MOUSE Protein RCC2 OS=Mus musculus GN=Rcc2 PE=1 SV=1	−1,28	0,400650829491335	0,755487293133664	26,984703803127	27,7522601319392	27,8187164630971	27,4902079656322	27,1009588259087	27,2306522722493	893632213090,988	699715518980,6	0,352912702600028	0,0168441105736387	0,0072670873673779	0,783001674212651	-0.352912702600029	1,27713647739713	0.352912702600029	835
Q9D0M1	sp|Q9D0M1|KPRA_MOUSE Phosphoribosyl pyrophosphate synthase-associated protein 1 OS=Mus musculus GN=Prpsap1 PE=1 SV=1	−1,28	0,115911270713939	0,487149312750528	28,3747558938646	27,8851022330226	27,9581743773589	27,7863646768548	27,8851022330226	27,8140234660062	1555281231403,64	1218323430149,91	0,352278298610249	0,00940938220207472	0,0018303215419882	0,783346063432125	-0.352278298610242	1,27657499881806	0.352278298610242	836
O55013	sp|O55013|TPPC3_MOUSE Trafficking protein particle complex subunit 3 OS=Mus musculus GN=Trappc3 PE=1 SV=1	−1,28	0,279109860429543	0,699221280539289	29,1644059935391	28,9989754780626	28,7093526612442	28,3553953189567	28,8489065316386	28,9384359919015	3768047370234,65	2954190974773,3	0,351054009318009	0,00795418303582494	0,0109346847605226	0,784011102967989	-0.351054009318009	1,27549214062703	0.351054009318009	837

Q99JF8	sp|Q99JF8|PSIP1_MOUSE PC4 and SFRS1-interacting protein OS=Mus musculus GN=Psip1 PE=1 SV=1;sp|Q99JF8-2|PSIP1_MOUSE Isoform 2 of PC4 and SFRS1-interacting protein OS=Mus musculus GN=Psip1	−1,28	0,0467674720505184	0,31495186296358	29,9980024503029	29,6815604610594	29,6926508385983	29,643927476549	29,5745822065934	29,4242425054228	8668682346252,72	6797554716030,58	0,350796859048069	0,00602811469125535	0,0038007631262420	0,78415085990191	-0.350796859048068	1,27526481336141	0.350796859048068	838
P21614	sp|P21614|VTDB_MOUSE Vitamin D-binding protein OS=Mus musculus GN=Gc PE=1 SV=2	−1,28	0,457412729377297	0,790957322506963	25,6908631453629	26,5758572324515	26,6843310444171	26,223070070365	26,0846949698555	25,914114289794	268741863543,724	210754498052,189	0,350657653798706	0,0207079274350716	0,0059353239067981	0,78422652605406	-0.350657653798704	1,27514176934518	0.350657653798704	839
P15116	sp|P15116|CADH2_MOUSE Cadherin-2 OS=Mus musculus GN=Cdh2 PE=1 SV=2	−1,28	0,0160233466901596	0,17007182864751 1	24,6992019327018	24,4792576365056	24,5323306407483	24,1749746788762	24,4023850233445	24,4042625278283	46852181278,4465	36742773727,9136	0,350655676195424	0,00467125163034355	0,0054194775584844	0,784227601049099	-0.350655676195427	1,27514002142012	0.350655676195427	840
P26369	sp|P26369|U2AF2_MOUSE Splicing factor U2AF 65 kDa subunit OS=Mus musculus GN=U2af2 PE=1 SV=3	−1,27	0,066062405742859	0,382601734678419	27,7219629002009	28,1288847943823	28,0160964439985	27,8165098051471	27,6483028203886	27,6736691686645	1383514402338,33	1085245413317,02	0,350316337237812	0,00751503781161799	0,0032722408629480	0,784412082362718	-0.350316337237811	1,27484012865778	0.350316337237811	841
P23591	sp|P23591|FCL_MOUSE GDP-L-fucose synthase OS=Mus musculus GN=Tsta3 PE=2 SV=3	−1,27	0,0167528752896426	0,17602152995298 1	27,3902087291243	27,0796340180657	27,3749832819008	27,0207057287637	27,0441476199735	27,0515185236898	705102258221,902	553092240169,451	0,350312399777975	0,00641753829842756	0,0005950618406008	0,784414223213831	-0.350312399777975	1,27483664931889	0.350312399777975	842
Q8CBY0	sp|Q8CBY0|GATC_MOUSE Glutamyl-tRNA(Gln) amidotransferase subunit C, mitochondrial OS=Mus musculus GN=Gatc PE=2 SV=1	−1,27	0,356413478568808	0,732843629012038	24,2796892913244	23,8940402301816	23,804353255327	23,4227004245546	23,6613231107722	24,1659420657239	26296304657,6493	20629512511,7221	0,350150346229046	0,0105264548475018	0,015977319418256	0,784502339028127	-0.350150346229046	1,27469345883511	0.350150346229046	843
Q9JIS5	sp|Q9JIS5|SV2A_MOUSE Synaptic vesicle glycoprotein 2A OS=Mus musculus GN=Sv2a PE=1 SV=1	−1,27	0,262490517824561	0,682806179310143	31,8850101738384	31,7123495077502	31,1864065676514	31,3898117118546	31,4180321721597	31,2478449838581	52644808715236,3	41300523414541,3	0,350131209160861	0,0115173270277112	0,0029092059598807	0,784512745367583	-0.350131209160859	1,27467655038727	0.350131209160859	844
Q9CQJ2	sp|Q9CQJ2|PIHD1_MOUSE PIH1 domain-containing protein 1 OS=Mus musculus GN=Pih1d1 PE=1 SV=1	−1,27	0,39531811930364	0,753881747531964	24,8152314567564	24,4443019138267	24,040363892071	24,4989816470079	24,1881015331326	23,8848298274034	40855164963,3557	32052392585,5264	0,350086424897576	0,015861588736648	0,0126943228458731	0,784537098657543	-0.350086424897579	1,27463698238253	0.350086424897578	845
Q9CRA7	sp|Q9CRA7|ATP5S_MOUSE ATP synthase subunit s, mitochondrial OS=Mus musculus GN=Atp5s PE=2 SV=1	−1,27	0,356298148425929	0,732843629012038	27,158095079911	27,0475341194994	26,8162660140065	26,3230679595192	26,8162660140065	27,1570050725175	535945548625,93	420809615520,791	0,348918761518825	0,00645863909833499	0,0156651530973167	0,785172330658726	-0.348918761518825	1,27360575628161	0.348918761518825	846
O88712	sp|O88712|CTBP1_MOUSE C-terminal-binding protein 1 OS=Mus musculus GN=Ctbp1 PE=1 SV=2;sp|O88712-2|CTBP1_MOUSE Isoform 2 of C-terminal-binding protein 1 OS=Mus musculus GN=Ctbp1;sp|O88712-3|CTBP1_MOUSE Isoform 3 of C-terminal-binding protein 1 OS=Mus mus	−1,27	0,0306356039276343	0,249959154423884	30,8170158313743	30,4337492602583	30,5864926907295	30,3862745237618	30,3537800086882	30,3721900067996	19715362316318	15482758714601,2	0,348657670139054	0,0063028072977374	0,0005365415665444	0,785314439886628	-0.348657670139053	1,2733752866487	0.348657670139053	847
Q8BL95	sp|Q8BL95|CU059_MOUSE UPF0769 protein C21orf59 homolog OS=Mus musculus PE=2 SV=1	−1,27	0,302120348551078	0,715641870477102	26,4261270803001	25,956784137851	25,6830722098595	25,7355004334847	25,9481071296484	25,6594416194182	200082271418,646	157236423881,615	0,347657883604289	0,014441439499147	0,0058034401546719	0,785858850795523	-0.34765788360429	1,27249314426847	0.34765788360429	848
Q61239	sp|Q61239|FNTA_MOUSE Protein farnesyltransferase/geranylgeranyltransferase type-1 subunit alpha OS=Mus musculus GN=Fnta PE=1 SV=1	−1,27	0,419073820938685	0,769072485508496	27,355199209797	27,1340590772315	26,6810725947884	26,4619673766869	27,2268519579805	26,7585971508416	563130347064,304	442543895422,602	0,347648338180224	0,0127008405120986	0,0143809979888479	0,785864050356476	-0.347648338180225	1,27248472499332	0.347648338180225	849
P61202	sp|P61202|CSN2_MOUSE COP9 signalosome complex subunit 2 OS=Mus musculus GN=Cops2 PE=1 SV=1;sp|P61202-2|CSN2_MOUSE Isoform 2 of COP9 signalosome complex subunit 2 OS=Mus musculus GN=Cops2	−1,27	0,059616275578866	0,361762399535392	30,6302391026061	30,4288703183794	30,282502562327 1	30,102498361754	30,3606214007766	30,1559838428577	16712933462373,8	13135871265520,3	0,347453084610777	0,00573424470736944	0,0045102405167771	0,78597041597 1163	-0.34745308461078	1,27231251924969	0.34745308461078	850
P52293	sp|P52293|IMA1_MOUSE Importin subunit alpha-1 OS=Mus musculus GN=Kpna2 PE=1 SV=2	−1,27	0,361829354548735	0,735816953537952	22,7260651701659	23,3339271274258	23,5758934844069	22,8225685424939	23,0567184762801	23,0354461367347	12045559555,2768	9471732925,71063	0,346801105987039	0,0188629247174983	0,0056366749009328	0,78632568974858	-0.346801105987038	1,27173766931072	0.346801105987038	851
Q8CG76	sp|Q8CG76|ARK72_MOUSE Aflatoxin B1 aldehyde reductase member 2 OS=Mus musculus GN=Akr7a2 PE=1 SV=3	−1,27	0,151396876859979	0,552437900049208	28,4457488486355	28,9032203325384	28,9001884639333	28,485463756269	28,4026187469632	28,6410826370046	3060866663123,63	2407769011728,96	0,346243205417906	0,00915662591835045	0,0042465706342123	0,786629826361603	-0.346243205417905	1,27124597426632	0.346243205417905	852
O54901	sp|O54901|OX2G_MOUSE OX-2 membrane glycoprotein OS=Mus musculus GN=Cd200 PE=1 SV=1	−1,27	0,153802770841827	0,557151457981685	28,3063564870261	27,913295506351	27,9689280949635	28,0241091157644	27,8187164630971	27,6263820326853	1540087007980,71	1211728785081,25	0,345945034940802	0,0075794420890357	0,0071487176449617	0,786792420689278	-0.345945034940797	1,27098326534963	0.345945034940797	853
Q9QZI8	sp|Q9QZI8|SERC1_MOUSE Serine incorporator 1 OS=Mus musculus GN=Serinc1 PE=1 SV=1	−1,27	0,673835253105275	0,900894908348996	22,6461403806329	22,7144546071874	22,9293534018626	22,840723799719	23,3004795688856	21,430109728472	7690995992,01307	6052704490,69183	0,345590524283613	0,0064926279225883 1	0,0432714350297907	0,786985781422514	-0.345590524283613	1,27067098746365	0.345590524283613	854
Q00493	sp|Q00493|CBPE_MOUSE Carboxypeptidase E OS=Mus musculus GN=Cpe PE=1 SV=2	−1,27	0,14546640262562 1	0,540763310006302	29,3914357617658	29,497763870862	29,8788419232647	29,4883704069559	29,3143252751957	29,2467701061378	7087453224887,75	5577835586945,95	0,345561898808043	0,00866138219378923	0,0042470531422630	0,787001396687777	-0.345561898808042	1,27064577548231	0.345561898808042	855
Q9D924	sp|Q9D924|ISCA1_MOUSE Iron-sulfur cluster assembly 1 homolog, mitochondrial OS=Mus musculus GN=Isca1 PE=2 SV=1	−1,27	0,146090402431767	0,541684557075751	27,8140234660062	27,6776121109565	27,5472752363342	27,1640538722618	27,4982193752751	27,6583684625279	1049816956507,65	826291871480,144	0,345414424418944	0,00481890864923964	0,0091912780382986	0,787081849229135	-0.345414424418937	1,2705158948582	0.345414424418937	856
Q9JLQ0	sp|Q9JLQ0|CD2AP_MOUSE CD2-associated protein OS=Mus musculus GN=Cd2ap PE=1 SV=3	−1,27	0,060445784748814	0,364360447509783	24,6867200139736	24,9004151407677	24,6402971622954	24,6878806630467	24,3480812542672	24,473229654249	55657208496,5866	43807192675,7301	0,345400787219789	0,00560710173907892	0,0070135329465467	0,787089289223277	-0.345400787219791	1,27050388525402	0.345400787219791	857
Q9CQ60	sp|Q9CQ60|6PGL_MOUSE 6-phosphogluconolactonase OS=Mus musculus GN=Pgls PE=2 SV=1	−1,27	0,094019253550711	0,450242157645468	30,9049840027489	30,4798415039778	30,6159575258508	30,5591116316026	30,3275359257424	30,3961101691837	20819843817491,7	16388252921720,3	0,345297182756483	0,00707948232958102	0,0039093873652812	0,787145814607494	-0.34529718275648	1,27041264965456	0.34529718275648	858

Q9JMD0-4	sp|Q9JMD0-4|ZN207_MOUSE Isoform 4 of BUB3-interacting and GLEBS motif-containing protein ZNF207 OS=Mus musculus GN=Znf207;sp|Q9JMD0-3|ZN207_MOUSE Isoform 3 of BUB3-interacting and GLEBS motif-containing protein ZNF207 OS=Mus musculus GN=Znf207;sp|Q9JMD0	−1,27	0,484658760105146	0,803590359311294	25,7991721647102	26,6350593510396	26,9102965508651	26,1521497135295	26,1217410182589	26,3537858638036	306405744254,789	241280388484,754	0,344732687432912	0,0218788824416389	0,0048117714876429	0,787453867980097	-0.344732687432915	1,26991566193599	0.344732687432915	859
P11574	sp|P11574|VATB1_ARATH V-type proton ATPase subunit B1 OS=Arabidopsis thaliana GN=VHA-B1 PE=1 SV=2;sp|Q9SZN1|VATB2_ARATH V-type proton ATPase subunit B2 OS=Arabidopsis thaliana GN=VHA-B2 PE=1 SV=1;sp|Q8W4E2|VATB3_ARATH V-type proton ATPase subunit B3 OS=	−1,27	0,49011802827803	0,806615565755611	29,1687376261914	30,1038443023282	30,161244964118	29,8255701629118	29,4440052105859	29,4486387307347	8848558062468,65	6970708843522,85	0,344137007009641	0,0186907092169872	0,0074044987604388	0,787779070252052	-0.344137007009642	1,26939142935093	0.344137007009642	860
Q6P8X1	sp|Q6P8X1|SNX6_MOUSE Sorting nexin-6 OS=Mus musculus GN=Snx6 PE=1 SV=2	−1,27	0,0987365926556446	0,462026179447393	29,5874435150517	29,2389697441818	29,3411392654344	29,1855954237581	29,3052903367405	28,961551696187	5801770090218,59	4571271389622,9	0,343897654081002	0,00609530556658706	0,0059857377781723	0,787909779005164	-0.343897654081001	1,26918084614031	0.343897654081	861
P29758	sp|P29758|OAT_MOUSE Ornithine aminotransferase, mitochondrial OS=Mus musculus GN=Oat PE=1 SV=1	−1,27	0,0126565303170163	0,146925808462754	30,8636183629237	30,5614058829116	30,8215053344581	30,4856864089267	30,5297954510822	30,5173788922969	22597111254307,2	17813070189824,6	0,343202159658328	0,00532330524828173	0,0007454918220157	0,788289706120258	-0.34320215965833	1,26856914689616	0.34320215965833	862
Q91XI1	sp|Q91XI1|DUS3L_MOUSE tRNA-dihydrouridine(47) synthase [NAD(P)(+)]-like OS=Mus musculus GN=Dus3l PE=2 SV=1;sp|Q91XI1-2|DUS3L_MOUSE Isoform 2 of tRNA-dihydrouridine(47) synthase [NAD(P)(+)]-like OS=Mus musculus GN=Dus3l	−1,27	0,0624479001863112	0,373453252892841	25,1160031518505	24,8661841819719	24,8042210143347	24,8090721097822	24,5262771526765	24,7376841280185	67056593756,0094	52865200836,0045	0,343060837913081	0,00662101825827273	0,0059560659880612	0,788366928215272	-0.34306083791308	1,26844488804703	0.34306083791308	863
Q5EBG8	sp|Q5EBG8|CA050_MOUSE Uncharacterized protein C1orf50 homolog OS=Mus musculus PE=2 SV=1	−1,27	0,310282806829706	0,719371512809526	23,8383590411367	23,4291787490228	24,1495233367792	23,6185544152518	23,7216479337666	23,3644875152294	21811147784,787	17200941450,2797	0,342578162652006	0,0151762891286189	0,0077995417414256	0,788630732321072	-0.342578162652008	1,26802058177068	0.342578162652008	864
O55057	sp|O55057|PDE6D_MOUSE Retinal rod rhodopsin-sensitive cGMP 3,5-cyclic phosphodiesterase subunit delta OS=Mus musculus GN=Pde6d PE=1 SV=1;sp|O43924|PDE6D_HUMAN Retinal rod rhodopsin-sensitive cGMP 3,5-cyclic phosphodiesterase subunit delta OS=Homo sap	−1,27	0,335066285183198	0,72953198881961	26,5828657246869	26,4055239012705	26,3399692238663	25,7400196200249	26,377343207	26,4988608819045	304758739735,693	240361025513,6	0,342464612070262	0,00475223786755824	0,0155537402746512	0,788692805732354	-0.342464612070262	1,26792078326546	0.342464612070262	865
Q8CGE9	sp|Q8CGE9|RGS12_MOUSE Regulator of G-protein signaling 12 OS=Mus musculus GN=Rgs12 PE=1 SV=2	−1,27	0,162483647590506	0,569625464688209	25,4494598461444	24,9442410806871	24,9622562481828	24,939766410663	24,817993141014	24,8891462278101	81075965003,4977	64009793145,3234	0,340981644020839	0,0114110114498007	0,0024585534362588	0,789503931807188	-0.34098164402084	1,26661813793756	0.34098164402084	866
Q9D8B4	sp|Q9D8B4|NDUAB_MOUSE NADH dehydrogenase [ubiquinone] 1 alpha subcomplex subunit 11 OS=Mus musculus GN=Ndufa11 PE=2 SV=2	−1,27	0,610626056124348	0,866610575848175	23,635106284079	23,3727250633184	22,5636759051768	22,4725237041215	22,7962312002305	23,594321097961	11789821571,8324	9310034812,03835	0,340683417187503	0,0240820669163725	0,025153125156846	0,789667151051834	-0.340683417187506	1,26635633591698	0.340683417187506	867
Q9CXU9	sp|Q9CXU9|EIF1B_MOUSE Eukaryotic translation initiation factor 1b OS=Mus musculus GN=Eif1b PE=2 SV=2	−1,27	0,0856440048442836	0,435317063816577	28,0692982272581	27,8477202619897	27,6454609497024	27,5000243142379	27,6440499937839	27,7105926216416	1249993932190,51	987282750364,949	0,340385865675745	0,00761078441260617	0,0038972056918773	0,789830034322501	-0.340385865675741	1,26609518066476	0.340385865675741	868
Q9CQX2	sp|Q9CQX2|CYB5B_MOUSE Cytochrome b5 type B OS=Mus musculus GN=Cyb5b PE=1 SV=1	−1,27	0,0196414507309682	0,194656211501888	28,7294282134955	28,5057267905654	28,4095407476388	28,2437154901872	28,3607504246018	28,3325463587444	2502301232060,63	1976477675218,56	0,34032381482316	0,00574904216759165	0,0021573137471785	0,78986400593782	-0.340323814823216	1,26604072660923	0.340323814823216	869
O89114	sp|O89114|DNJB5_MOUSE DnaJ homolog subfamily B member 5 OS=Mus musculus GN=Dnajb5 PE=2 SV=1	−1,27	0,138215157550944	0,52900134279042	26,9215383290468	26,4187355803847	26,6350593510396	26,5451722092463	26,3911397781081	26,3327598269784	378107513796,481	298795114034,218	0,339639961971601	0,00946100871174428	0,0041530818068705	0,790238498658999	-0.339639961971601	1,26544075199697	0.339639961971602	870
Q9WVF8	sp|Q9WVF8|TUSC2_MOUSE Tumor suppressor candidate 2 OS=Mus musculus GN=Tusc2 PE=1 SV=3	−1,27	0,307505651404229	0,71837258919956	25,5454020810795	25,286634641881	25,0214896836834	24,7157174558305	25,1293167074687	25,3025335055744	95702360642,7664	75635321222,3224	0,339494390017821	0,0103605994508869	0,0120364057269415	0,790318239950743	-0.339494390017821	1,2653130719371	0.339494390017821	871
Q8BGF6	sp|Q8BGF6|ELMD2_MOUSE ELMO domain-containing protein 2 OS=Mus musculus GN=Elmod2 PE=2 SV=1	−1,26	0,680354985173224	0,904284415139462	23,160204023241	23,0900203571021	23,3553531113101	21,8402680937608	23,4824267429013	23,5787835721751	11924478771,5924	9429976764,70284	0,338600085024425	0,00592554088536277	0,0425435743036171	0,790808298235039	-0.338600085024431	1,26452896641556	0.338600085024431	872
P85094	sp|P85094|ISC2A_MOUSE Isochorismatase domain-containing protein 2A, mitochondrial OS=Mus musculus GN=Isoc2a PE=1 SV=1	−1,26	2,02×10^−05	0,00095457271297424	29,469468974537	29,339015370216	29,3492574870933	29,2163908422627	29,1446312403025	29,0935725463537	5782827951624,93	4574559578250,36	0,33814232755943	0,00246858969931487	0,0021165002132653	0,791059256218222	-0.338142327559431	1,26412780349812	0.338142327559431	873
P14231	sp|P14231|AT1B2_MOUSE Sodium/potassium-transporting ATPase subunit beta-2 OS=Mus musculus GN=Atp1b2 PE=1 SV=2	−1,26	0,110390400244998	0,475842380543255	30,0134696913558	29,8229827561781	29,9308608554239	29,8175332931728	29,8306347317633	29,4161611861033	9888936942361,78	7823160438715,1	0,338063887744796	0,00319233357507895	0,0079360201996206	0,79110226754532	-0.338063887744795	1,26405907431268	0.338063887744795	874
Q80VD1	sp|Q80VD1|FA98B_MOUSE Protein FAM98B OS=Mus musculus GN=Fam98b PE=2 SV=1	−1,26	0,162650331222575	0,569625464688209	29,6135756219551	29,126082642873	29,126082642873	29,0665390787366	29,119788155716	28,9766402388757	5246489501883,15	4150801197223,35	0,337962582879442	0,00960969086905299	0,0024902207871335	0,79115782004967	-0.33796258287944	1,26397031623503	0.33796258287944	875

Q04207	sp|Q04207|TF65_MOUSE Transcription factor p65 OS=Mus musculus GN=Rela PE=1 SV=1;sp|Q04207-2|TF65_MOUSE Isoform p65 delta of Transcription factor p65 OS=Mus musculus GN=Rela	−1,26	0,648914833558383	0,889714023663571	23,4666785516388	24,155861296033	23,4032259601932	22,5407241994077	23,9894755268835	23,7937470144796	19144001564,7516	15150745570,3755	0,337503629232736	0,0176312545126837	0,0335327132604582	0,791409545132477	-0.337503629232743	1,26356828288267	0.337503629232743	876
Q6NS60	sp|Q6NS60|FBX41_MOUSE F-box only protein 41 OS=Mus musculus GN=Fbxo41 PE=1 SV=3	−1,26	0,564595628417967	0,845110794140252	26,234878662775	25,0138713240005	26,0039735970023	25,8243737283388	25,404963452821	25,3227647390092	152572825193,491	120795794333,738	0,336927799875913	0,0251893914252382	0,0105431410606957	0,791725487029202	-0.336927799875922	1,26306404982907	0.336927799875922	877
P48318	sp|P48318|DCE1_MOUSE Glutamate decarboxylase 1 OS=Mus musculus GN=Gad1 PE=2 SV=2	−1,26	0,19757136161022	0,626240510020104	30,4288703183794	30,8467694077559	30,7768742173066	30,6939719257556	30,3920639022539	30,2664339291469	21181966831780,3	16773531473408,7	0,336650091985769	0,00729503470790294	0,0072163009540166	0,79187790286984	-0.336650091985774	1,26282094294576	0.336650091985774	878
Q9JME7	sp|Q9JME7|TPC2L_MOUSE Trafficking protein particle complex subunit 2-like protein OS=Mus musculus GN=Trappc2l PE=2 SV=1	−1,26	0,226993824120017	0,652517395617958	27,2429325656057	26,7226120902923	26,8468256315125	26,4815901695906	26,8427056892879	26,7885005791647	499791457053,048	395835864759,189	0,336423907739259	0,010087895559324	0,0072923815154966	0,792002062406551	-0.336423907739259	1,26262297469458	0.336423907739259	879
P35979	sp|P35979|RL12_MOUSE 60S ribosomal protein L12 OS=Mus musculus GN=Rpl12 PE=1 SV=2	−1,26	0,327228449891093	0,726927682919497	28,0603919717577	27,6226377001941	27,3216488345134	27,322661625401	27,343085730949	27,6401569056551	1037905824611,56	822242680899,825	0,336039379061151	0,013426070450591	0,0064771923114362	0,79221318678653	-0.336039379061146	1,26228648636401	0.336039379061146	880
Q9ERD6-3	sp|Q9ERD6-3|RGPS2_MOUSE Isoform 3 of Ras-specific guanine nucleotide-releasing factor RalGPS2 OS=Mus musculus GN=Ralgps2;sp|Q9ERD6-4|RGPS2_MOUSE Isoform 4 of Ras-specific guanine nucleotide-releasing factor RalGPS2 OS=Mus musculus GN=Ralgps2;sp|Q9ERD6|R	−1,26	0,740342825731883	0,925497610201997	21,4141158051472	21,8402680937608	23,4485091486631	22,3211019379397	22,343926520924	21,3395865968786	4531404092,11018	3590431947,74227	0,335800731991073	0,0482546801402199	0,0260609331217694	0,792344243585277	-0.335800731991075	1,2620776993029	0.335800731991074	881
Q6WVG3	sp|Q6WVG3|KCD12_MOUSE BTB/POZ domain-containing protein KCTD12 OS=Mus musculus GN=Kctd12 PE=1 SV=1	−1,26	0,258156333397585	0,67968190098123	30,1150222657986	29,5350096320515	29,6772864366557	29,3328195161808	29,6067416513827	29,6912193119391	8539920189287,15	6770482652635,17	0,33496390306824	0,0101521628323369	0,0063417788298953	0,792803972703206	-0.33496390306824	1,26134584894967	0.334963903068241	882
P56387	sp|P56387|DYLT3_MOUSE Dynein light chain Tctex-type 3 OS=Mus musculus GN=Dynlt3 PE=1 SV=1	−1,26	0,0171317242265395	0,178300688102698	26,4656131249857	26,2158504682045	26,3957362099935	26,1262269484152	26,2361110611353	26,0184741068914	280283268255,521	222220811781,148	0,334891687399501	0,00488848469731415	0,0041650582620595	0,792843658361225	-0.334891687399502	1,26128271249209	0.334891687399502	883
Q62417-2	sp|Q62417-2|SRBS1_MOUSE Isoform 2 of Sorbin and SH3 domain-containing protein 1 OS=Mus musculus GN=Sorbs1;sp|Q62417|SRBS1_MOUSE Sorbin and SH3 domain-containing protein 1 OS=Mus musculus GN=Sorbs1 PE=1 SV=2;sp|Q62417-5|SRBS1_MOUSE Isoform 5 of Sorbin an	−1,26	0,0541685240856691	0,341924788314513	25,8047943938068	25,4664846831164	25,807698406866	25,5351249444596	25,3944926619741	25,4540678038503	143987524103,13	114201294570,125	0,334364808805138	0,007635034837274	0,0027724139439502	0,793133261242336	-0.334364808805135	1,26082217058157	0.334364808805135	884
P61255	sp|P61255|RL26_MOUSE 60S ribosomal protein L26 OS=Mus musculus GN=Rpl26 PE=1 SV=1	−1,26	0,575365466051444	0,849998193752035	26,8427056892879	26,4261270803001	25,9896298932137	25,5244369585765	26,5591097026922	26,4796440426437	297740325987,348	236149339099,475	0,33435513571961	0,0161462879319749	0,0219874471004634	0,793138579117126	-0.334355135719613	1,26081371695869	0.334355135719612	885
Q62288	sp|Q62288|TICN1_MOUSE Testican-1 OS=Mus musculus GN=Spock1 PE=2 SV=2	−1,26	0,683020407818847	0,904912326646161	23,5823483342612	21,9979846941957	21,8345749480041	22,4365116962145	22,1958548520544	22,0882083383777	5745231205,89526	4558190806,94337	0,333903635133476	0,0429594401622195	0,0080185591338347	0,79338683572319	-0.333903635133475	1,260419199026	0.333903635133475	886
O09012-2	sp|O09012-2|PEX5_MOUSE Isoform 2 of Peroxisomal targeting signal 1 receptor OS=Mus musculus GN=Pex5;sp|O09012|PEX5_MOUSE Peroxisomal targeting signal 1 receptor OS=Mus musculus GN=Pex5 PE=2 SV=2	−1,26	0,213159794744243	0,645012843958033	25,9864544591337	25,6908631453629	25,4571592515809	25,526381389398	25,6347612167554	25,2798116120726	146676055125,198	116402293160,585	0,333513890124201	0,0103163957515298	0,0071389585197766	0,793601198649825	-0.333513890124201	1,2600787419441	0.333513890124201	887
Q8K1E6	sp|Q8K1E6|ALKB3_MOUSE Alpha-ketoglutarate-dependent dioxygenase alkB homolog 3 OS=Mus musculus GN=Alkbh3 PE=1 SV=1	−1,26	0,711015811409161	0,916805961322165	22,1209126219968	22,4525488290568	22,5905470800734	22,8687955606518	22,6546864138481	20,9472431782709	5284283336,21789	4193948056,92203	0,333398830628417	0,0107816178647948	0,0475272334869659	0,793664493381189	-0.333398830628416	1,25997825068345	0.333398830628416	888
P0C192	sp|P0C192|LRC4B_MOUSE Leucine-rich repeat-containing protein 4B OS=Mus musculus GN=Lrrc4b PE=1 SV=1	−1,26	0,495358229440849	0,808788130730939	26,8999307211861	25,9419377124712	26,5143677630458	26,0883849331964	26,6015089080294	25,9735589231531	307603893519,769	244174976358,901	0,333158407408038	0,0182225555525157	0,0127517541658419	0,793796767540618	-0.333158407408039	1,25976829446944	0.333158407408039	889
Q8R0X7	sp|Q8R0X7|SGPL1_MOUSE Sphingosine-1-phosphate lyase 1 OS=Mus musculus GN=Sgpl1 PE=1 SV=1	−1,26	0,230793228806244	0,654468548759245	25,0736508555744	25,6387500127636	25,5883688052889	25,3047067573104	25,2267225649949	25,0773874416878	111088152662,973	88205832097,8127	0,332759010419116	0,0122960444233203	0,0045832306846543	0,794016553371067	-0.332759010419117	1,25941958735547	0.332759010419117	890
P28472-4	sp|P28472-4|GBRB3_HUMAN Isoform 4 of Gamma-aminobutyric acid receptor subunit beta-3 OS=Homo sapiens GN=GABRB3;sp|P28472-3|GBRB3_HUMAN Isoform 3 of Gamma-aminobutyric acid receptor subunit beta-3 OS=Homo sapiens GN=GABRB3;sp|P63080|GBRB3_MOUSE Gamma-ami	−1,26	0,0899607547183496	0,441176105282424	25,4127860971458	25,1070055387849	25,1160031518505	24,8042210143347	25,1110816865738	25,0286278335686	89002615681,5547	70671638609,6481	0,332716375736741	0,00690162383162449	0,0063570860584954	0,794040018582222	-0.33271637573674	1,25938236939937	0.33271637573674	891
Q9D289	sp|Q9D289|TPC6B_MOUSE Trafficking protein particle complex subunit 6B OS=Mus musculus GN=Trappc6b PE=2 SV=1	−1,26	0,433482914962406	0,7767413243707	28,198658713088	27,8663186708468	27,8204849529863	27,2091856375552	27,8869880507204	28,0992599920198	1392080879126,62	1106044470650,51	0,331833640328443	0,00738091981256133	0,0167635968809588	0,794526012988867	-0.331833640328447	1,25861203239674	0.331833640328447	892
A3KFX0	sp|A3KFX0|5NT1A_MOUSE Cytosolic 5-nucleotidase 1A OS=Mus musculus GN=Nt5c1a PE=2 SV=1	−1,26	0,0101829652947963	0,124583502622858	24,0114271695296	23,9460675522639	23,7728608943411	23,5733620117965	23,750582547653	23,7163979450698	24212104686,5148	19237246683,454	0,331826164758638	0,00515593492477049	0,0039702860911122	0,794530129971245	-0.331826164758642	1,25860551070126	0.331826164758642	893

Q6PGN3-5	sp|Q6PGN3-5|DCLK2_MOUSE Isoform 5 of Serine/threonine-protein kinase DCLK2 OS=Mus musculus GN=Dclk2;sp|Q6PGN3-4|DCLK2_MOUSE Isoform 4 of Serine/threonine-protein kinase DCLK2 OS=Mus musculus GN=Dclk2;sp|Q6PGN3-3|DCLK2_MOUSE Isoform 3 of Serine/threonine	−1,26	0,127488775830818	0,511193951851664	29,2283500040983	29,0513152204935	28,7572558211707	28,6599523335218	28,8240709718089	28,8632336136022	3980016238549,74	3162610698910,51	0,331658338552055	0,00820194236508402	0,0037471377039021	0,794622561656412	-0.331658338552056	1,25845910782532	0.331658338552056	894
Q91VI7	sp|Q91VI7|RINI_MOUSE Ribonuclease inhibitor OS=Mus musculus GN=Rnh1 PE=1 SV=1	−1,26	0,181189525177098	0,600744525039334	30,2434645802855	29,8416907060905	29,6912193119391	29,7525593270964	29,7341360948367	29,6015013481049	9918850955705,92	7885648596855,57	0,330943580034988	0,00954014971799476	0,0027751646760802	0,795016341315147	-0.330943580034987	1,25783578026303	0.330943580034987	895
Q9WTZ1	sp|Q9WTZ1|RBX2_MOUSE RING-box protein 2 OS=Mus musculus GN=Rnf7 PE=2 SV=1	−1,26	0,10978268637678	0,475080634873879	24,6343822529779	24,4164001122609	24,3404652835671	24,0084076421851	24,343352667187	24,3517999664098	42118344381,9572	33490246761,0833	0,330707720914516	0,00623666106914839	0,0080820527820820	0,795146325253705	-0.330707720914517	1,25763015968279	0.330707720914517	896
Q921E2	sp|Q921E2|RAB31_MOUSE Ras-related protein Rab-31 OS=Mus musculus GN=Rab31 PE=1 SV=1	−1,26	0,343755440573562	0,730103496880899	26,6322995250514	26,0481993746539	26,1288765661872	25,784338153794	26,0066860711259	26,3309093895965	256345459258,333	203848832342,483	0,330589649959954	0,0120488904980771	0,0105551704825554	0,795211403128673	-0.330589649959959	1,25752723875137	0.330589649959959	897
P55264	sp|P55264|ADK_MOUSE Adenosine kinase OS=Mus musculus GN=Adk PE=1 SV=2;sp|Q64640|ADK_RAT Adenosine kinase OS=Rattus norvegicus GN=Adk PE=1 SV=3	−1,26	0,200596987693761	0,6308537814738	30,727262227369	30,7354901350717	30,3043185118729	30,2058798248574	30,5694328473646	30,3043185118729	19259462701118,7	15315355391021,4	0,330588610662945	0,00806159040024225	0,0061935891043416	0,795211975987876	-0.330588610662948	1,25752633284694	0.330588610662948	898
Q61151	sp|Q61151|2A5E_MOUSE Serine/threonine-protein phosphatase 2A 56 kDa regulatory subunit epsilon isoform OS=Mus musculus GN=Ppp2r5e PE=2 SV=3	−1,26	0,0598100377998397	0,362453620258307	30,3275359257424	30,31031921555	30,1714331301119	29,8880330606952	29,97721683577	30,2570757598888	13995581059814,2	11131223672401,7	0,330359186003072	0,00282757271473377	0,0064098270071271	0,795338444672584	-0.330359186003069	1,25732637055118	0.330359186003069	899
O89029	sp|O89029|MATN4_MOUSE Matrilin-4 OS=Mus musculus GN=Matn4 PE=2 SV=1;sp|O89029-2|MATN4_MOUSE Isoform Short of Matrilin-4 OS=Mus musculus GN=Matn4	−1,26	0,108781961148162	0,473794072647748	25,8188439996991	26,3063436289981	26,0089907009257	25,8205849296101	25,8344529325275	25,7943002713021	204682556262,644	162907117570,156	0,32933851827829	0,00943402537539675	0,0007899535759507	0,795901324200374	-0.329338518278296	1,25643716073055	0.329338518278296	900
P35285	sp|P35285|RB22A_MOUSE Ras-related protein Rab-22A OS=Mus musculus GN=Rab22a PE=1 SV=2	−1,26	0,100646065185696	0,46324407107743	27,0871885833272	26,9044098251012	26,8042111906981	26,485190850542	26,7761234567643	26,8505074822295	497040094606,763	395707285368,669	0,32892860697503	0,00532761147733206	0,0072296126686234	0,796127494868871	-0.328928606975031	1,25608022137799	0.328928606975031	901
P32883-2	sp|P32883-2|RASK_MOUSE Isoform 2B of GTPase KRas OS=Mus musculus GN=Kras;sp|P32883|RASK_MOUSE GTPase KRas OS=Mus musculus GN=Kras PE=1 SV=1;sp|P01117|RASK_MSVKI GTPase KRas OS=Kirsten murine sarcoma virus GN=K-RAS PE=1 SV=1	−1,25	0,306531773395381	0,71837258919956	27,2818929651463	26,8246682302946	26,8316021926563	26,4553495397667	26,7885005791647	27,0138855393607	521193804967,791	415429413178,903	0,327216570491107	0,00971112106238397	0,0105034881152278	0,797072814794059	-0.327216570491109	1,25459052352497	0.327216570491108	902
Q62433	sp|Q62433|NDRG1_MOUSE Protein NDRG1 OS=Mus musculus GN=Ndrg1 PE=1 SV=1	−1,25	0,295799713958346	0,711969538636193	27,2661868502051	27,2136596408025	27,275090612165	27,1437352154627	27,3252327797371	26,6060338018682	684288672296,029	545517432714,648	0,326979764747484	0,00121815612941737	0,0138394939298951	0,797203658047188	-0.326979764747484	1,25438461038874	0.326979764747484	903
O08807	sp|O08807|PRDX4_MOUSE Peroxiredoxin-4 OS=Mus musculus GN=Prdx4 PE=1 SV=1	−1,25	0,439753482171222	0,780009157984922	30,8286913985065	30,8120815400983	30,5297954510822	29,9670200277696	30,6232978272985	30,9004869372115	22032126434244,2	17565097119147,8	0,326897190318814	0,00546741430930364	0,0157193199028536	0,797249288286883	-0.326897190318818	1,25431281619427	0.326897190318818	904
Q3TZM9	sp|Q3TZM9|ALG11_MOUSE GDP-Man:Man(3)GlcNAc(2)-PP-Dol alpha-1,2-mannosyltransferase OS=Mus musculus GN=Alg11 PE=2 SV=1;sp|Q3TZM9-2|ALG11_MOUSE Isoform 2 of GDP-Man:Man(3)GlcNAc(2)-PP-Dol alpha-1,2-mannosyltransferase OS=Mus musculus GN=Alg11;sp|Q3TZM9-3|	−1,25	0,247914485923141	0,673304679179655	24,9622562481828	24,4117497112943	24,5749811329927	24,3570462707292	24,6343822529779	24,2796892913244	50723865877,6813	40465109192,9161	0,325986214977117	0,0114707284222482	0,0076362606547368	0,797752862340899	-0.32598621497712	1,25352104292755	0.325986214977119	905
Q9CZQ9	sp|Q9CZQ9|BBS5_MOUSE Bardet-Biedl syndrome 5 protein homolog OS=Mus musculus GN=Bbs5 PE=1 SV=1	−1,25	0,345812287355725	0,730405757239477	22,0932341720107	21,7962886665004	21,4244040100507	21,3089099562841	21,7962886665004	21,5318623747088	2852064484,51214	2276003746,19426	0,325503668894534	0,0153924628538791	0,0113239964068186	0,798019735722624	-0.32550366889453	1,25310184101459	0.32550366889453	906
P29387	sp|P29387|GBB4_MOUSE Guanine nucleotide-binding protein subunit beta-4 OS=Mus musculus GN=Gnb4 PE=2 SV=4	−1,25	0,341782123764752	0,729721949431128	26,0312420351845	26,438944362597	26,6691328676683	26,2970734250463	26,3040142907628	25,8629458516474	286147454043,705	228471624013,033	0,32474377589272	0,0122455855970043	0,0096606489434156	0,798440177553134	-0.324743775892723	1,25244198389986	0.324743775892723	907
P62313	sp|P62313|LSM6_MOUSE U6 snRNA-associated Sm-like protein LSm6 OS=Mus musculus GN=Lsm6 PE=3 SV=1	−1,25	0,446674732404498	0,785227192247876	27,1340590772315	26,7376285098563	26,5203006373062	26,1085492621562	26,7663350011133	26,8446549836125	434442487511,99	347204489057,01	0,323379601702473	0,0116132512350444	0,015213943256276	0,799195518480286	-0.32337960170247	1,25125826769093	0.32337960170247	908
Q9JHU2	sp|Q9JHU2|PALMD_MOUSE Palmdelphin OS=Mus musculus GN=Palmd PE=1 SV=1	−1,25	0,604110310492497	0,86472932807488	26,1085492621562	25,9147147066523	25,6392323739276	26,1541626625147	24,8485211097813	25,9874283403252	174903246513,625	139784907651,761	0,323348464786229	0,00911015678426147	0,0276887934469822	0,799212767276288	-0.323348464786232	1,2512312627437	0.323348464786231	909
Q80V91	sp|Q80V91|DTX3_MOUSE Probable E3 ubiquitin-protein ligase DTX3 OS=Mus musculus GN=Dtx3 PE=2 SV=2	−1,25	0,0159513692422026	0,170071828647511	25,5091258895633	25,2573533322883	25,3552094720957	25,2310892455359	25,0392995070299	25,1789507460175	104650936148,391	83639341032,6685	0,323331616632394	0,00500207869314084	0,0039430440957233	0,799222100737552	-0.323331616632391	1,25121665063712	0.323331616632391	910

Q8CCJ4	sp|Q8CCJ4|AMER2_MOUSE APC membrane recruitment protein 2 OS=Mus musculus GN=Amer2 PE=1 SV=2;sp|Q8CCJ4-2|AMER2_MOUSE Isoform 2 of APC membrane recruitment protein 2 OS=Mus musculus GN=Amer2	−1,25	0,250836293904226	0,673538059877323	26,2142780507656	26,475991979372	26,6477017691461	26,4091064430994	26,3290904224167	25,9276015798895	305736849439,5	244365969887,234	0,323247054752526	0,00825319104692337	0,0098398157505061	0,79926894757771	-0.323247054752525	1,25114331418809	0.323247054752525	911
Q64487-12	sp|Q64487-12|PTPRD_MOUSE Isoform L of Receptor-type tyrosine-protein phosphatase delta OS=Mus musculus GN=Ptprd;sp|Q64487-5|PTPRD_MOUSE Isoform E of Receptor-type tyrosine-protein phosphatase delta OS=Mus musculus GN=Ptprd;sp|Q64487|PTPRD_MOUSE Receptor	−1,25	0,0199655837198995	0,196155377715636	29,0156519772701	28,8395124734223	28,7408088025449	28,7125758401729	28,5376790926525	28,6735464306224	3435983842918,76	2746276530287,35	0,323246350674772	0,00482335881563089	0,0032054399388011	0,799269337644636	-0.323246350674774	1,25114270359338	0.323246350674774	912
P59279	sp|P59279|RAB2B_MOUSE Ras-related protein Rab-2B OS=Mus musculus GN=Rab2b PE=2 SV=1	−1,25	0,226123084447595	0,652238880554131	25,3944926619741	25,8525777820242	25,9104073439626	25,369136158521	25,4540678038503	25,6624905516586	147804939143,666	118151259969,816	0,323059465950763	0,010989539594672	0,0059206026584170	0,799372880597537	-0.323059465950759	1,2509806427915	0.323059465950759	913
P00375	sp|P00375|DYR_MOUSE Dihydrofolate reductase OS=Mus musculus GN=Dhfr PE=1 SV=3	−1,25	0,303994984737475	0,716532404521184	22,7554239515794	23,0429429798347	22,7607929420049	22,2373996301638	22,8107698771548	22,8392616668271	8413078456,84541	6725309103,44273	0,32303322108815	0,00719690283067289	0,0150053979082468	0,799387422563568	-0.323033221088117	1,2509578857184	0.323033221088116	914
Q810N6	sp|Q810N6|ANR45_MOUSE Ankyrin repeat domain-containing protein 45 OS=Mus musculus GN=Ankrd45 PE=2 SV=1	−1,25	0,567532543328741	0,845527592693687	23,2810705328774	23,4227004245546	24,0804450555623	23,8182850170703	23,5046582728801	22,7898514546523	17663013739,6714	14121038034,9747	0,32288537808533	0,0180783255844175	0,0225535204513195	0,79946934555447	-0.322885378085329	1,25082969792476	0.322885378085329	915
P40201	sp|P40201|CHD1_MOUSE Chromodomain-helicase-DNA-binding protein 1 OS=Mus musculus GN=Chd1 PE=1 SV=3;sp|Q5NCC9|TRI58_MOUSE E3 ubiquitin-protein ligase TRIM58 OS=Mus musculus GN=Trim58 PE=2 SV=1	−1,25	0,566056338523286	0,845163895632417	22,1997126389534	20,9058908208185	21,3956711244922	21,430109728472	21,3020994376969	21,0988721291394	2175283551,33855	1739784511,32265	0,322294844804514	0,0303826994140633	0,0078505368020727	0,799796656510404	-0.322294844804514	1,25031780498196	0.322294844804514	916
Q91VR7	sp|Q91VR7|MLP3A_MOUSE Microtubule-associated proteins 1A/1B light chain 3A OS=Mus musculus GN=Map1lc3a PE=1 SV=1	−1,25	0,168026772067522	0,577689763615299	30,6703686802763	30,6742077219904	30,413494702835	30,1584841687995	30,3063347984404	30,6232978272985	19201772371174,6	15358736761550,5	0,322179920490637	0,004885460982174	0,0078213981269608	0,799860370421159	-0.322179920490635	1,25021820930253	0.322179920490635	917
Q80VJ3	sp|Q80VJ3|DNPH1_MOUSE 2-deoxynucleoside 5-phosphate N-hydrolase 1 OS=Mus musculus GN=Dnph1 PE=2 SV=2	−1,25	0,353852300519182	0,732843629012038	22,6368476224383	22,2659402972637	21,9854071197575	21,8174060215298	22,0655043166933	22,3355549008786	4820121697,59154	3855712864,67649	0,322071953903901	0,01465565242339	0,0117407703921496	0,799920231599768	-0.322071953903903	1,25012465055433	0.322071953903902	918
Q4VAE3	sp|Q4VAE3|TMM65_MOUSE Transmembrane protein 65 OS=Mus musculus GN=Tmem65 PE=2 SV=1	−1,25	0,0782654440224607	0,417369905631425	26,7655997645261	26,6005971337788	26,385525166963	26,2599380141522	26,475991979372	26,3465902358295	350949212106,354	280780784770,253	0,321818056675688	0,00716923072703138	0,0041246630873598	0,800061020468008	-0.321818056675689	1,24990466279064	0.321818056675689	919
Q921F2	sp|Q921F2|TADBP_MOUSE TAR DNA-binding protein 43 OS=Mus musculus GN=Tardbp PE=1 SV=1	−1,25	0,0148703911720495	0,162642663927549	30,4410482207959	30,1871752749852	30,2998683979432	30,0920533297203	30,1781158654775	29,9890457048955	14560943604154,1	11650904899466,7	0,321661840558701	0,00419680746033967	0,0031463231320182	0,800147656374605	-0.321661840558702	1,24976932948964	0.321661840558701	920
Q9Z0S1	sp|Q9Z0S1|BPNT1_MOUSE 3(2),5-bisphosphate nucleotidase 1 OS=Mus musculus GN=Bpnt1 PE=1 SV=2	−1,25	0,0896130087650211	0,441176105282424	31,3949762767942	31,0040688696121	31,0680797664268	30,9037738640846	31,0315490815031	30,8636183629237	33943154141305,1	27165813338753	0,321328390786089	0,00672972383605184	0,0028349145674394	0,800332615692163	-0.321328390786089	1,24948050397166	0.32132839078609	921
O88844	sp|O88844|IDHC_MOUSE Isocitrate dehydrogenase [NADP] cytoplasmic OS=Mus musculus GN=Idh1 PE=1 SV=2	−1,25	0,00138596135947769	0,0305382456828604	31,5209983110802	31,3009254761233	31,4546926223212	31,2478449838581	31,1972620093183	31,1636297730172	44456789157407,6	35583823533731,2	0,32118221644816	0,003592487203853	0,0013585581476687	0,800413709765228	-0.321182216448162	1,24935391260766	0.321182216448162	922
Q9CZS1	sp|Q9CZS1|AL1B1_MOUSE Aldehyde dehydrogenase X, mitochondrial OS=Mus musculus GN=Aldh1b1 PE=1 SV=1	−1,25	0,0330245626858403	0,264371234622626	29,0211872608148	29,3362258860121	29,3290713947903	29,0254710300426	28,9889107169626	29,0045580088892	4942182645086,94	3956232312144	0,32102118397782	0,00615344167035809	0,0006323881557967	0,800503056291722	-0.321021183977819	1,24921446850239	0.321021183977819	923
Q9JL56	sp|Q9JL56|GDE1_MOUSE Glycerophosphodiester phosphodiesterase 1 OS=Mus musculus GN=Gde1 PE=2 SV=1	−1,25	0,0196116711868439	0,194656211501888	27,738528557757	27,5237275215483	27,4718930142898	27,4312593139902	27,3724149190948	27,2638746068392	948407119407,001	759441865785,469	0,32056696007548	0,00512634829104535	0,0031040160779457	0,800755129569584	-0.32056696007548	1,24882122270951	0.32056696007548	924
Q9CQP2	sp|Q9CQP2|TPPC2_MOUSE Trafficking protein particle complex subunit 2 OS=Mus musculus GN=Trappc2 PE=1 SV=1;sp|F1SRI0|TPPC2_PIG Trafficking protein particle complex subunit 2 OS=Sus scrofa GN=TRAPPC2 PE=2 SV=2	−1,25	0,361163113527244	0,735120794753435	25,5972870851289	25,5584985256053	25,6245416244004	24,9376594065974	25,4007405161479	25,7758152556667	130343930313,49	104390557141,779	0,320332186970062	0,00129677698267866	0,0165480497886485	0,800885448909738	-0.320332186970062	1,24861801567418	0.320332186970062	925
Q91X96	sp|Q91X96|MSS4_MOUSE Guanine nucleotide exchange factor MSS4 OS=Mus musculus GN=Rabif PE=2 SV=1	−1,25	0,389515919441711	0,753319093070654	26,7099050263889	26,6443531342949	26,4531431572982	25,9168429770321	26,4697443055486	26,7551771799298	357523562498,047	286380778089,185	0,32010366347391	0,00501484496519786	0,0161565406149531	0,801012319546769	-0.32010366347391	1,24842024972328	0.32010366347391	926
Q62165	sp|Q62165|DAG1_MOUSE Dystroglycan OS=Mus musculus GN=Dag1 PE=1 SV=4	−1,25	0,282350338868413	0,703396371088763	26,7753723973676	26,4349457329772	26,4493758498685	26,387321076432	26,6005971337788	26,0066860711259	340346933425,392	272671813452,494	0,31984053677189	0,00725015566341946	0,0114258394360159	0,80115842592795	-0.31984053677189	1,24819257669509	0.31984053677189	927
Q9DCB8	sp|Q9DCB8|ISCA2_MOUSE Iron-sulfur cluster assembly 2 homolog, mitochondrial OS=Mus musculus GN=Isca2 PE=2 SV=2	−1,25	0,33595787975021	0,72953198881961	27,4118429955384	27,3351344193571	27,0036061767615	26,6595208620373	27,1210844296353	27,3070559481143	683296372929,663	547824278752,93	0,318798263179132	0,00796209701887081	0,0123347495875491	0,801737431159058	-0.318798263179132	1,24729114687126	0.318798263179132	928
Q8JZM7	sp|Q8JZM7|CDC73_MOUSE Parafibromin OS=Mus musculus GN=Cdc73 PE=2 SV=1	−1,25	0,335493754529319	0,72953198881961	24,8479407160407	25,4159343606792	25,5400198303093	25,2169254586012	24,952119175502	24,9719414710983	94132101617,3041	75469570211,4468	0,318791746986197	0,0146036632705556	0,0058881588109021	0,801741052359266	-0.318791746986198	1,24728551326806	0.318791746986198	929

Q8C0E2	sp|Q8C0E2|VP26B_MOUSE Vacuolar protein sorting-associated protein 26B OS=Mus musculus GN=Vps26b PE=1 SV=1;sp|Q8C0E2-2|VP26B_MOUSE Isoform 2 of Vacuolar protein sorting-associated protein 26B OS=Mus musculus GN=Vps26b	−1,25	0,0265386766722907	0,226853207938847	30,9481182033933	30,6159575258508	30,7195270753918	30,507269860691	30,5614058829116	30,5524835884731	22878093087262,3	18345151719087,5	0,318567970910616	0,00552502811040568	0,0009503709053724	0,801865419863224	-0.318567970910615	1,24709206212007	0.318567970910615	930
Q9QYR9	sp|Q9QYR9|ACOT2_MOUSE Acyl-coenzyme A thioesterase 2, mitochondrial OS=Mus musculus GN=Acot2 PE=1 SV=2	−1,25	0,20702980771158	0,637641589296298	29,7678736961036	29,2410664809566	29,469468974537	29,2802995314596	29,4161611861033	29,119788155716	6435193654945,18	5160646394145,7	0,318431783267811	0,00895735915039675	0,0050682167972839	0,801941117992612	-0.318431783267808	1,24697434457926	0.318431783267809	931
P97346-2	sp|P97346-2|NXN_MOUSE Isoform 2 of Nucleoredoxin OS=Mus musculus GN=Nxn;sp|P97346|NXN_MOUSE Nucleoredoxin OS=Mus musculus GN=Nxn PE=1 SV=1	−1,25	0,450681386669821	0,786343703431612	23,835941088441	22,9811816841146	23,5687032910947	23,0632449784514	23,5344531294744	23,1262887967585	15466496746,2566	12404547401,1373	0,318277357502146	0,0186372627980558	0,0110063664085851	0,802026962191009	-0.318277357502153	1,24684087585804	0.318277357502153	932
Q9DBP5	sp|Q9DBP5|KCY_MOUSE UMP-CMP kinase OS=Mus musculus GN=Cmpk1 PE=1 SV=1	−1,25	0,388222878983757	0,753319093070654	31,3817725927108	31,1656015127658	30,8665321302372	30,5244583269409	31,0040688696121	31,2250526281598	33346326931492,9	26758142663091,4	0,317549879506377	0,00830912645315558	0,0115844006140933	0,802431485724459	-0.317549879506377	1,24621231568097	0.317549879506377	933
P62340	sp|P62340|TBPL1_MOUSE TATA box-binding protein-like protein 1 OS=Mus musculus GN=Tbpl1 PE=2 SV=1	−1,25	0,23004523952822	0,654468548759245	25,5297811363375	25,3047067573104	24,961398664519	25,0609738563947	24,9023550022812	25,1735426829318	93881155806,9536	75366133045,4484	0,316919232087111	0,0113291157977074	0,0054398294917630	0,802782330464941	-0.316919232087112	1,24566767609451	0.316919232087112	934
Q9CR26	sp|Q9CR26|VTA1_MOUSE Vacuolar protein sorting-associated protein VTA1 homolog OS=Mus musculus GN=Vta1 PE=1 SV=1	−1,25	0,03334700630837	0,265547476550336	28,7045051167192	28,4643306249264	28,5904583519206	28,5192469795579	28,299685761744	28,2826760859385	2599736902584,35	2087948127342,3	0,31627975739801	0,00420261885209606	0,0046514412175587	0,803138242668598	-0.316279757398007	1,24511565615066	0.316279757398007	935
Q9D6N5	sp|Q9D6N5|NC2A_MOUSE Dr1-associated corepressor OS=Mus musculus GN=Drap1 PE=2 SV=3	−1,24	0,25095110622251	0,673538059877323	26,0271456176258	26,336120588715	26,5351193087617	26,1944717091464	25,8417503358081	26,2070979591574	264065189191,76	212266132865,25	0,315019921387807	0,0097326443737252	0,0079515328854877	0,803839890880527	-0.315019921387807	1,2440288312945	0.315019921387807	936
Q9JJK2	sp|Q9JJK2|LANC2_MOUSE LanC-like protein 2 OS=Mus musculus GN=Lancl2 PE=1 SV=1	−1,24	0,000268054880110117	0,00822095338391772	31,1238583488395	30,9306955015522	31,0218212612287	30,7896398570902	30,7820965217206	30,8504306917222	29797880136190	23959571942495,8	0,314607565528793	0,00311462837512254	0,0012161208845776	0,804069679889628	-0.31460756552879	1,24367330967792	0.31460756552879	937
Q9CQF0	sp|Q9CQF0|RM11_MOUSE 39S ribosomal protein L11, mitochondrial OS=Mus musculus GN=Mrpl11 PE=2 SV=1	−1,24	0,135014892720897	0,521116154813056	24,6613896974429	24,2294265771732	24,2586916472883	24,1126737547988	24,1341293512829	24,2494398701144	38857591780,7516	31254035018,913	0,31415403252	0,00989985458064541	0,0030438035538318	0,804322491091559	-0.31415403252	1,24328240360764	0.31415403252	938
Q3TRR0-2	sp|Q3TRR0-2|MAP9_MOUSE Isoform 2 of Microtubule-associated protein 9 OS=Mus musculus GN=Map9;sp|Q3TRR0|MAP9_MOUSE Microtubule-associated protein 9 OS=Mus musculus GN=Map9 PE=2 SV=2	−1,24	0,61953074165802	0,870879639674993	24,457410707647	23,804353255327	22,9874166300028	23,4643347891491	23,3919312518076	23,7401078459667	20624115106,626	16590973659,5989	0,313933665827444	0,031011638420051	0,0078072338069391	0,804445357961983	-0.31393366582744	1,24309251101087	0.31393366582744	939
Q8BRJ3	sp|Q8BRJ3|RELL2_MOUSE RELT-like protein 2 OS=Mus musculus GN=Rell2 PE=2 SV=1	−1,24	0,40237243915777	0,757239901094806	25,3025335055744	24,6808051491555	24,9662926902838	24,9294153710934	24,9762304365785	24,391301183712	70806061496,442	56961930582,4313	0,313874826749159	0,0124567355860051	0,0131245764695133	0,804478167244109	-0.31387482674916	1,24304181358419	0.31387482674916	940
P70698	sp|P70698|PYRG1_MOUSE CTP synthase 1 OS=Mus musculus GN=Ctps1 PE=1 SV=2	−1,24	0,201776461036793	0,632779967349437	27,4879183321299	27,3412496695829	27,2620275033788	27,3623338424334	26,8410458836077	27,235518194489	765451608905,67	615868501957,359	0,313688830143622	0,00418843858726954	0,0100144507201608	0,804581889687105	-0.313688830143622	1,24288156720616	0.313688830143622	941
Q00915	sp|Q00915|RET1_MOUSE Retinol-binding protein 1 OS=Mus musculus GN=Rbp1 PE=2 SV=2	−1,24	0,225451748651372	0,652238880554131	28,6410826370046	28,485463756269	28,3607504246018	28,0224070708488	28,2415791179015	28,5719407915324	2374399325763	1910989576620,72	0,313242678159852	0,00492879384169406	0,0097824816256302	0,804830744300618	-0.313242678159854	1,24249726676257	0.313242678159854	942
B2RXS4	sp|B2RXS4|PLXB2_MOUSE Plexin-B2 OS=Mus musculus GN=Plxnb2 PE=1 SV=1	−1,24	0,267102294593476	0,685404159524725	25,6624905516586	25,6555739842145	25,3363157103993	25,2300086282913	25,6547165375052	25,1183229982551	124985064372,626	100593088353,997	0,313224521663985	0,00729322639506262	0,0111710057959477	0,804840873259003	-0.313224521663992	1,2424816298789	0.313224521663992	943
Q99020	sp|Q99020|ROAA_MOUSE Heterogeneous nuclear ribonucleoprotein A/B OS=Mus musculus GN=Hnrnpab PE=1 SV=1	−1,24	0,0434640746345745	0,30674678910092	29,2687031531706	28,9766402388757	29,2041525384792	28,8859884835841	29,0533026951177	28,8605637488314	4566791414849,91	3677612851049,9	0,312411284458354	0,00526321167125032	0,0036191159186951	0,805294684379792	-0.312411284458351	1,24178144894892	0.312411284458351	944
Q8VEA4	sp|Q8VEA4|MIA40_MOUSE Mitochondrial intermembrane space import and assembly protein 40 OS=Mus musculus GN=Chchd4 PE=1 SV=1	−1,24	0,181944669044807	0,601053022412213	25,3172002676571	25,149119368486	25,0758097375073	24,6730051571631	25,1160031518505	25,1035425680548	86266725354,9147	69471582825,17	0,312381225228998	0,00491475713063619	0,01010427212291	0,805311463247888	-0.312381225228998	1,24175557611824	0.312381225228998	945
P54071	sp|P54071|IDHP_MOUSE Isocitrate dehydrogenase [NADP], mitochondrial OS=Mus musculus GN=Idh2 PE=1 SV=3	−1,24	0,00865507272260807	0,110352177213253	31,42290936091	31,1804486408323	31,2146660379869	31,1056636057856	31,0680797664268	30,9950183754577	38154876120056,8	30729797914625,7	0,312229162996568	0,00419622250344121	0,0018116350092317	0,805396348763717	-0.312229162996566	1,24162470010573	0.312229162996566	946

P04406	sp|P04406|G3P_HUMAN Glyceraldehyde-3-phosphate dehydrogenase OS=Homo sapiens GN=GAPDH PE=1 SV=3;sp|P04406-2|G3P_HUMAN Isoform 2 of Glyceraldehyde-3-phosphate dehydrogenase OS=Homo sapiens GN=GAPDH	−1,24	0,17129213987268	0,585022590580958	28,9384359919015	28,4283424016431	28,5598383543679	28,4598031167191	28,4722196117525	28,3456414045074	2748855014762,1	2214146337198,48	0,312080239778864	0,0092462911018084	0,0024545364402437	0,805479490663535	-0.312080239778864	1,24149653913127	0.312080239778864	947
Q8CHQ0	sp|Q8CHQ0|FBX4_MOUSE F-box only protein 4 OS=Mus musculus GN=Fbxo4 PE=1 SV=2	−1,24	0,343517772525237	0,730103496880899	24,0144583554802	23,4666785516388	23,6905689153765	23,6951786116361	23,6458420059548	23,1840498105516	20098317475,7143	16201291680,6684	0,310965892232187	0,0116088392872131	0,0119931542917953	0,806101888889214	-0.310965892232185	1,24053796893836	0.310965892232185	948
Q8C4Q6	sp|Q8C4Q6|AIDA_MOUSE Axin interactor, dorsalization-associated protein OS=Mus musculus GN=Aida PE=1 SV=1	−1,24	0,11730333231426 6	0,490276082296919	26,1835363389388	25,8263359153978	26,2285373005935	25,7605581739283	25,9073997517755	25,9245627621526	211918976372,314	170870801760,182	0,31060688849749	0,00845002235541054	0,0034852442883896	0,806302506199276	-0.310606888497492	1,24022930886544	0.310606888497492	949
P70353	sp|P70353|NFYC_MOUSE Nuclear transcription factor Y subunit gamma OS=Mus musculus GN=Nfyc PE=2 SV=2	−1,24	0,289539306160047	0,708855938867559	23,8296632028795	23,1629885154419	23,3825283651281	23,2704101887496	23,141829678946	23,3177018894452	15411708676,4914	12429194270,9787	0,310294044519041	0,0144831107200469	0,0039156750937275	0,806477369374225	-0.31029404451904	1,239960398115	0.31029404451904	950
P48428	sp|P48428|TBCA_MOUSE Tubulin-specific chaperone A OS=Mus musculus GN=Tbca PE=2 SV=3	−1,24	0,0214729553784077	0,20390323109329	28,7093526612442	28,6310783417307	28,5094418456896	28,2648305123125	28,4258373734941	28,5146694615578	2679427539279,66	2161403976920,53	0,30995605713421	0,00352019708541153	0,0044590542814689	0,806666328995635	-0.30995605713421	1,239669940414	0.30995605713421	951
Q04735	sp|Q04735|CDK16_MOUSE Cyclin-dependent kinase 16 OS=Mus musculus GN=Cdk16 PE=1 SV=1;sp|Q04735-2|CDK16_MOUSE Isoform Short of Cyclin-dependent kinase 16 OS=Mus musculus GN=Cdk16	−1,24	0,239888422917596	0,664340177927377	24,8284747330514	25,2509320978723	25,4094933692325	24,9798946960453	25,0286278335686	24,8358716084403	84749588978,6197	68365310695,2898	0,309941899872483	0,0119354621969398	0,0040174091659388	0,806674244904441	-0.309941899872484	1,23965777551068	0.309941899872484	952
Q91VL8	sp|Q91VL8|TE2IP_MOUSE Telomeric repeat-binding factor 2-interacting protein 1 OS=Mus musculus GN=Terf2ip PE=1 SV=1	−1,24	0,319168526968894	0,722907157640623	23,4291787490228	24,021910159197	23,9338055309889	23,7983315076674	23,508629348876	23,43383578992	21578532961,4747	17409215838,0878	0,309745563813725	0,013441010657293 4	0,0081643596170439	0,806784032499772	-0.309745563813725	1,23948908222881	0.309745563813725	953
Q9JKS5	sp|Q9JKS5|HABP4_MOUSE Intracellular hyaluronan-binding protein 4 OS=Mus musculus GN=Habp4 PE=1 SV=2	−1,24	0,266989788184301	0,685404159524725	27,1925405628277	27,7823931945621	27,6817373259402	27,3878476077223	27,4149626112445	27,210872020632	924227134042,255	745927375001,569	0,309212272065927	0,0114522247101402	0,0040543221605026	0,80708231507891	-0.309212272065927	1,23903099016886	0.309212272065927	954
P62889	sp|P62889|RL30_MOUSE 60S ribosomal protein L30 OS=Mus musculus GN=Rpl30 PE=2 SV=2	−1,24	0,439608954297327	0,780009157984922	26,3643567050085	26,1720458506377	26,0782885894433	25,482818253974	26,1234980759642	26,3667324253046	240238267333,306	193979098739,16	0,308564764618643	0,00556521982744676	0,017567864772939	0,807444629418817	-0.308564764618642	1,23847501558067	0.308564764618642	955
O70475	sp|O70475|UGDH_MOUSE UDP-glucose 6-dehydrogenase OS=Mus musculus GN=Ugdh PE=1 SV=1;sp|O60701-3|UGDH_HUMAN Isoform 3 of UDP-glucose 6-dehydrogenase OS=Homo sapiens GN=UGDH;sp|O60701|UGDH_HUMAN UDP-glucose 6-dehydrogenase OS=Homo sapiens GN=UGDH PE=1 SV=1	−1,24	0,140851306778459	0,532098608288336	24,1086932997909	24,5308608808026	24,4846098063473	24,2627398725708	24,1529970070456	24,0670079530992	38530706392,2155	31113727088,7037	0,308457410977297	0,00949938070414288	0,0040605206339535	0,807504715122215	-0.308457410977299	1,2383828617628	0.308457410977299	956
Q68FH4	sp|Q68FH4|GALK2_MOUSE N-acetylgalactosamine kinase OS=Mus musculus GN=Galk2 PE=1 SV=1	−1,24	0,548290459539198	0,837660853533632	27,232167200089	26,056977654842	26,8528957989924	26,3643567050085	26,6511319794876	26,485190850542	399713369646,65	322776674778,002	0,308429501878297	0,0224518171350681	0,0054330729710640	0,807520336543507	-0.308429501878296	1,23835890533776	0.308429501878296	957
P56399	sp|P56399|UBP5_MOUSE Ubiquitin carboxyl-terminal hydrolase 5 OS=Mus musculus GN=Usp5 PE=1 SV=1	−1,24	0,476803133135458	0,799905345588749	32,5913113535174	31,6843541136303	32,0248903492098	31,9425028325732	32,099937215965	31,6172183495446	87283736944029,6	70494287871219,7	0,308206509021132	0,014272279934067	0,0077203422764977	0,8076451620812 7	-0.308206509021132	1,23816751086955	0.308206509021132	958
P60879-2	sp|P60879-2|SNP25_MOUSE Isoform 2 of Synaptosomal-associated protein 25 OS=Mus musculus GN=Snap25	−1,24	0,397816681805087	0,754338506152154	26,7885005791647	27,1124388235916	27,5544638957705	27,1190534987677	26,9857690304365	26,7099050263889	619266220966,504	500184325548,776	0,30809990571612	0,0141609671534558	0,0077463723487395	0,807704842625077	-0.30809990571612	1,23807602384796	0.30809990571612	959
Q6ZWM4	sp|Q6ZWM4|LSM8_MOUSE U6 snRNA-associated Sm-like protein LSm8 OS=Mus musculus GN=Lsm8 PE=3 SV=3	−1,24	0,0716771030201851	0,399193092771313	26,9195750813523	26,9340296968593	26,8127695771732	26,4531431572982	26,8091718647215	26,7646133507678	476051296817,564	384666588034,209	0,307508516003082	0,00246319657887409	0,0072715790770446	0,808036004955205	-0.307508516003083	1,23756861559088	0.307508516003083	960
Q61081	sp|Q61081|CDC37_MOUSE Hsp90 co-chaperone Cdc37 OS=Mus musculus GN=Cdc37 PE=2 SV=1	−1,24	0,193888075856752	0,621726099655746	29,9631043655379	29,5767627429087	29,6792282244892	29,656852932955	29,6285973016842	29,2944931553283	8237338781797,74	6656718735101,65	0,307367112831709	0,00672984525179646	0,0068259554753329	0,808115207038853	-0.30736711283171	1,23744732346303	0.30736711283171	961
Q80XN0	sp|Q80XN0|BDH_MOUSE D-beta-hydroxybutyrate dehydrogenase, mitochondrial OS=Mus musculus GN=Bdh1 PE=1 SV=2	−1,24	0,2352309881895 4	0,658269701228311	29,7799328650923	30,1011524211797	30,3309155382186	30,0171565154699	29,748150804332	29,8078729257526	11468978004611,7	9269279313435,81	0,307207760416339	0,00920343738108847	0,0047311846518529	0,808204472073154	-0.307207760416335	1,23731064916637	0.307207760416335	962
Q9JJZ2	sp|Q9JJZ2|TBA8_MOUSE Tubulin alpha-8 chain OS=Mus musculus GN=Tuba8 PE=1 SV=1;sp|Q9NY65|TBA8_HUMAN Tubulin alpha-8 chain OS=Homo sapiens GN=TUBA8 PE=1 SV=1;sp|Q9NY65-2|TBA8_HUMAN Isoform 2 of Tubulin alpha-8 chain OS=Homo sapiens GN=TUBA8	−1,24	0,0131515268484711	0,149610978358923	33,5634667763458	33,3807460089899	33,3701779207136	33,1717080697328	33,3423047078514	33,161783050067 7	33265606534036 0	268874347271117	0,307099221400305	0,00324997742144998	0,0030543181284655	0,80826527842207 2	-0.307099221400306	1,23721756544119	0.307099221400306	963

Q3USJ8-3	sp|Q3USJ8-3|FCSD2_MOUSE Isoform 3 of FCH and double SH3 domains protein 2 OS=Mus musculus GN=Fchsd2;sp|Q3USJ8-2|FCSD2_MOUSE Isoform 2 of FCH and double SH3 domains protein 2 OS=Mus musculus GN=Fchsd2;sp|Q3USJ8|FCSD2_MOUSE FCH and double SH3 domains prot	−1,24	0,360654391155819	0,734744291497425	23,7280173231175	24,0390384667258	23,6434784339963	23,2810705328774	23,5278189761452	23,9631070192037	21763746284,9595	17591215752,222	0,307071722293963	0,00875106816547532	0,0146385949263471	0,808280684855206	-0.307071722293957	1,23719398315096	0.307071722293957	964
Q99KJ8	sp|Q99KJ8|DCTN2_MOUSE Dynactin subunit 2 OS=Mus musculus GN=Dctn2 PE=1 SV=3	−1,24	0,00626290246675097	0,0888740760486895	31,4485046525447	31,2250526281598	31,3439939544913	31,143072998167	31,1921742262174	31,0449398168704	40778811850837,2	32973621648076,3	0,306507387279629	0,00356753537297015	0,0024083986903357	0,808596919613276	-0.30650738727963	1,23671012805523	0.30650738727963	965
Q9DC61	sp|Q9DC61|MPPA_MOUSE Mitochondrial-processing peptidase subunit alpha OS=Mus musculus GN=Pmpca PE=1 SV=1	−1,24	0,114835232050014	0,484871737930245	28,5192469795579	28,3623185321326	28,7125758401729	28,3478835582042	28,456269909736	28,1529200921064	2460487090697,54	1989738859744,02	0,306364848306599	0,0061491456110755	0,0054281614054517	0,808676813329647	-0.3063648483066	1,23658794652786	0.306364848306601	966
Q80ZJ1	sp|Q80ZJ1|RAP2A_MOUSE Ras-related protein Rap-2a OS=Mus musculus GN=Rap2a PE=1 SV=2	−1,24	0,0791699884172227	0,418825847815587	29,6661513123182	29,5856163544791	29,4883704069559	29,1733008248421	29,3820077371923	29,5501594072729	7021837308531,94	5682937173980,55	0,30521180409481	0,00300950363182077	0,0064284080575894	0,809323389916119	-0.305211804094811	1,23560002399491	0.305211804094811	967
Q6P3D0	sp|Q6P3D0|NUD16_MOUSE U8 snoRNA-decapping enzyme OS=Mus musculus GN=Nudt16 PE=1 SV=1	−1,23	0,160934538488365	0,567767985643373	27,2015892625047	26,9742992198464	27,0138855393607	26,5866027765041	27,0036061767615	26,9663839376261	566791873548,703	458945649948,275	0,304495759139541	0,0044866176469987	0,0085938444530920	0,809725176676936	-0.30449575913954	1,23498691754151	0.30449575913954	968
Q8K0V4	sp|Q8K0V4|CNOT3_MOUSE CCR4-NOT transcription complex subunit 3 OS=Mus musculus GN=Cnot3 PE=1 SV=1;sp|O75175|CNOT3_HUMAN CCR4-NOT transcription complex subunit 3 OS=Homo sapiens GN=CNOT3 PE=1 SV=1;sp|O75175-2|CNOT3_HUMAN Isoform 2 of CCR4-NOT transcripti	−1,23	0,389232615205719	0,753319093070654	26,341020672108	25,8362365598065	25,6433260264358	25,9481071296484	25,7400196200249	25,4993244819944	184367045387,75	149289081950,541	0,304472145040961	0,0138887743562889	0,0087289578975343	0,809738430404224	-0.30447214504096	1,23496670338445	0.30447214504096	969
Q6P9S0	sp|Q6P9S0|MTSSL_MOUSE MTSS1-like protein OS=Mus musculus GN=Mtss1l PE=1 SV=1	−1,23	0,246294635549644	0,67102722134444	27,5067207470531	27,3519448291009	26,9717991804572	26,8911690458988	27,139097165185	27,1684490497881	701734935939,091	568483667316,971	0,303807288195653	0,010091340336597	0,0056277923131028	0,810111679214321	-0.303807288195654	1,23439770794298	0.303807288195654	970
Q8BSY0	sp|Q8BSY0|ASPH_MOUSE Aspartyl/asparaginyl beta-hydroxylase OS=Mus musculus GN=Asph PE=1 SV=1	−1,23	0,124438127930531	0,504307734532748	25,7084262444795	25,347826740016	25,5721737946229	25,3445401043115	25,1746957465022	25,4779842302653	123908462757,049	100397856431,575	0,303546257679161	0,00712859665532417	0,0060005299230303	0,810258268060577	-0.303546257679165	1,23417438540132	0.303546257679165	971
Q9Z2W0	sp|Q9Z2W0|DNPEP_MOUSE Aspartyl aminopeptidase OS=Mus musculus GN=Dnpep PE=2 SV=2	−1,23	0,100282993846077	0,46324407107743	30,1213977470374	29,8286939050459	29,7621840572139	29,5962766892579	29,8158570876731	29,6699505699435	9709169877747,57	7869598146551,05	0,303057984459429	0,00639069685322044	0,0037634766248457	0,810532542497518	-0.30305798445943	1,23375675567408	0.30305798445943	972
P46414	sp|P46414|CDN1B_MOUSE Cyclin-dependent kinase inhibitor 1B OS=Mus musculus GN=Cdkn1b PE=1 SV=2	−1,23	0,395471848801448	0,753881747531964	24,8115612153573	25,5185024275634	25,5419778283143	25,0513945111618	25,1906725328726	25,0023493395826	96294832166,5396	78116889707,6701	0,30182386714799	0,0164129807071047	0,0038952267936728	0,811226188883831	-0.301823867147989	1,23270182065486	0.301823867147989	973
P07724	sp|P07724|ALBU_MOUSE Serum albumin OS=Mus musculus GN=Alb PE=1 SV=3	−1,23	0,24244546509287	0,666355294143363	34,3323405522909	34,8802329166851	34,7531101143992	34,5639686668958	34,3323405522909	34,4432127689793	1123502981291090	911859766510429	0,301120076067776	0,00827469945239647	0,0033631623658247	0,811622026549989	-0.301120076067768	1,23210061739054	0.301120076067768	974
Q99L45	sp|Q99L45|IF2B_MOUSE Eukaryotic translation initiation factor 2 subunit 2 OS=Mus musculus GN=Eif2s2 PE=1 SV=1	−1,23	0,0601606629924296	0,36316304279371	28,6255772652186	28,2856963956054	28,4722196117525	28,161225425277	28,2591635841223	28,3382640165987	2293647415341,18	1862393084175,46	0,300486028004981	0,00598042686554771	0,0031390659130422	0,811978803594113	-0.300486028004982	1,23155924215464	0.300486028004983	975
Q8CHC4	sp|Q8CHC4|SYNJ1_MOUSE Synaptojanin-1 OS=Mus musculus GN=Synj1 PE=1 SV=3	−1,23	0,449707549982378	0,786343703431612	23,779469321038	23,3877341976412	23,3186569620764	23,7565991016167	23,0737103227927	23,0310308799512	15990918861,7887	12985685012,2114	0,300330720473409	0,0105774827983807	0,0174837529166707	0,812066218611209	-0.300330720473404	1,23142667073406	0.300330720473404	976
Q9QZE7	sp|Q9QZE7|TSNAX_MOUSE Translin-associated protein X OS=Mus musculus GN=Tsnax PE=1 SV=1	−1,23	0,261637060156809	0,682806179310143	28,5750404298675	28,6662319760378	28,8941100043926	28,8140965858009	28,41000627506	28,2874146092639	2946956729757,98	2393642246129,08	0,300018334356845	0,00572384865502629	0,0096686892376571	0,812242073987173	-0.300018334356845	1,23116005932954	0.300018334356845	977
Q9R1K9	sp|Q9R1K9|CETN2_MOUSE Centrin-2 OS=Mus musculus GN=Cetn2 PE=2 SV=1	−1,23	0,520580470853625	0,823916573667872	25,8337781042129	25,5419778283143	25,1266580132717	24,8524446071923	25,3070616187359	25,7191474648085	118811572244,876	96507307091,3846	0,299965275560606	0,0139349926953695	0,0171402086726326	0,812271946814059	-0.299965275560605	1,23111478110534	0.299965275560605	978
Q99J56	sp|Q99J56|DERL1_MOUSE Derlin-1 OS=Mus musculus GN=Derl1 PE=1 SV=1	−1,23	0,239654415933965	0,664270737934997	24,1365945678446	24,0697373811504	23,9032676843376	23,5141970803436	23,9166756183545	24,0558013103755	27474749260,8541	22323177743,7604	0,299563902987083	0,00499885286002871	0,0118037781540477	0,812497960648047	-0.299563902987082	1,23077231997284	0.299563902987082	979
P83887	sp|P83887|TBG1_MOUSE Tubulin gamma-1 chain OS=Mus musculus GN=Tubg1 PE=1 SV=1;sp|Q8VCK3|TBG2_MOUSE Tubulin gamma-2 chain OS=Mus musculus GN=Tubg2 PE=1 SV=1	−1,23	0,131980535126311	0,516676186281332	27,6030325495586	27,4832478791861	27,6568502118367	27,6110235759658	27,3368848768073	27,173042131448	951250761702,446	773081408303,103	0,299205360616945	0,00322197796035267	0,0080841703357539	0,812699909873949	-0.299205360616945	1,23046648320055	0.299205360616945	980
P24288	sp|P24288|BCAT1_MOUSE Branched-chain-amino-acid aminotransferase, cytosolic OS=Mus musculus GN=Bcat1 PE=2 SV=2	−1,23	0,157645118908575	0,562098346210542	30,0816756695027	29,6647193245074	29,8531115187151	29,4939123683025	29,763614849229	29,7200994190062	9350979105461,98	7600300324684,03	0,299061004468145	0,00699112521381158	0,0048819585760676	0,812781232742209	-0.299061004468144	1,23034336881296	0.299061004468144	981
P11835	sp|P11835|ITB2_MOUSE Integrin beta-2 OS=Mus musculus GN=Itgb2 PE=1 SV=2	−1,23	0,526843144988758	0,82805770353786	23,9556768269568	23,6328120342077	23,0041717325947	23,3186569620764	23,0327998585375	23,6206335365283	16570450647,1875	13474032062,2636	0,298431200963576	0,0205632677311436	0,0126030394673037	0,813136127022022	-0.298431200963579	1,22980638391057	0.298431200963579	982

Q78T54	sp|Q78T54|VMA21_MOUSE Vacuolar ATPase assembly integral membrane protein Vma21 OS=Mus musculus GN=Vma21 PE=1 SV=1;sp|Q78T54-2|VMA21_MOUSE Isoform 2 of Vacuolar ATPase assembly integral membrane protein Vma21 OS=Mus musculus GN=Vma21	−1,23	0,709283988675802	0,916805961322165	26,6416157587629	26,6511319794876	26,0422096112122	25,2084612346714	26,6710608514719	26,8348651408146	305429794268,719	248356009435,618	0,298431146087198	0,013191417478076	0,034128647699806	0,813136157951614	-0.298431146087197	1,22980633713192	0.298431146087197	983
Q9CZY3	sp|Q9CZY3|UB2V1_MOUSE Ubiquitin-conjugating enzyme E2 variant 1 OS=Mus musculus GN=Ube2v1 PE=1 SV=1;sp|Q9CZY3-2|UB2V1_MOUSE Isoform 2 of Ubiquitin-conjugating enzyme E2 variant 1 OS=Mus musculus GN=Ube2v1	−1,23	0,393897630949495	0,753881747531964	31,1310784705769	31,1032923983988	30,8286913985065	30,4270212494486	30,8215053344581	31,1956272436472	29665941587628	24135814750459	0,297632045682953	0,0053879760228645	0,0124728692526431	0,81358667410458	-0.297632045682957	1,22912534316099	0.297632045682957	984
P59108	sp|P59108|CPNE2_MOUSE Copine-2 OS=Mus musculus GN=Cpne2 PE=2 SV=1	−1,23	0,33610249564742	0,72953198881961	26,4619673766869	27,0584803792591	27,1340590772315	26,6219754182725	26,7080610722193	26,7056831906351	474171833584,861	385795482163,491	0,297573718543115	0,013693935124113	0,0018377878564871	0,813619567503152	-0.297573718543114	1,22907565149744	0.297573718543114	985
Q8R0F6	sp|Q8R0F6|ILKAP_MOUSE Integrin-linked kinase-associated serine/threonine phosphatase 2C OS=Mus musculus GN=Ilkap PE=2 SV=1;sp|Q8R0F6-2|ILKAP_MOUSE Isoform 2 of Integrin-linked kinase-associated serine/threonine phosphatase 2C OS=Mus musculus GN=Ilkap	−1,23	0,137464093948207	0,527876076506694	25,3750798339753	25,0264181578287	25,1125314478412	25,0467789403677	24,784693819856	25,0651396225333	85462468067,9742	69565699596,3763	0,296914842044248	0,0072152964290132	0,0062840233124678	0,813991231110315	-0.296914842044248	1,228514462786	0.296914842044248	986
Q9CZL5	sp|Q9CZL5|PHS2_MOUSE Pterin-4-alpha-carbinolamine dehydratase 2 OS=Mus musculus GN=Pcbd2 PE=1 SV=2	−1,23	0,415764355742946	0,767871528551302	25,2204546504586	25,4069322364685	25,266100588418	24,6754463944538	25,0723990419734	25,528533155215	96985678328,2368	78953601746,888	0,296766642068968	0,0038423609377866	0,0170127224178683	0,814074852161973	-0.29676664206897	1,22838827086263	0.29676664206897	987
Q9CQI7	sp|Q9CQI7|RU2B_MOUSE U2 small nuclear ribonucleoprotein B OS=Mus musculus GN=Snrpb2 PE=1 SV=1	−1,23	0,588968912322136	0,858211843669398	25,4554077650705	25,806618885234	26,1835363389388	25,9896298932137	24,9798946960453	25,8593137304936	162702247213,225	132468772221,843	0,296581874089318	0,0141056463848013	0,021446266058608	0,814179118548004	-0.296581874089317	1,2282309595257	0.296581874089317	988
Q99M87-2	sp|Q99M87-2|DNJA3_MOUSE Isoform 2 of DnaJ homolog subfamily A member 3, mitochondrial OS=Mus musculus GN=Dnaja3;sp|Q99M87|DNJA3_MOUSE DnaJ homolog subfamily A member 3, mitochondrial OS=Mus musculus GN=Dnaja3 PE=1 SV=1;sp|Q99M87-3|DNJA3_MOUSE Isoform 3	−1,23	0,475765488163115	0,79947899324683	26,9970599843299	26,7839028148677	26,5603842110093	26,5866027765041	26,1207629911894	27,0177302526787	427170472448,091	347848197263,233	0,296354082325819	0,0081536487183056	0,0168803395162241	0,814307682058953	-0.29635408232582	1,22803704549554	0.29635408232582	989
Q922H4	sp|Q922H4|GMPPA_MOUSE Mannose-1-phosphate guanyltransferase alpha OS=Mus musculus GN=Gmppa PE=2 SV=1	−1,23	0,229963406247159	0,654468548759245	27,5128434547509	27,1537780055346	27,3399629165513	27,219419790962	27,3081622510207	26,8636371325686	744164631063,548	606157925086,094	0,295928108557665	0,0065692456870222	0,0086711314953944	0,814548152093419	-0.295928108557666	1,22767450571211	0.295928108557666	990
Q9CWZ3-2	sp|Q9CWZ3-2|RBM8A_MOUSE Isoform 2 of RNA-binding protein 8A OS=Mus musculus GN=Rbm8a;sp|Q9CWZ3|RBM8A_MOUSE RNA-binding protein 8A OS=Mus musculus GN=Rbm8a PE=1 SV=3	−1,23	0,340095858888981	0,729620681454069	26,6246811115036	26,2872690468727	26,0995196682378	25,8716928723719	26,3924755423625	26,1321115412	274209050064,624	223369529215,373	0,295843791878266	0,0101040245702032	0,0099644269710198	0,814595758829734	-0.295843791878269	1,22760275776125	0.295843791878269	991
P63005	sp|P63005|LIS1_MOUSE Platelet-activating factor acetylhydrolase IB subunit alpha OS=Mus musculus GN=Pafah1b1 PE=1 SV=2;sp|P63005-2|LIS1_MOUSE Isoform 2 of Platelet-activating factor acetylhydrolase IB subunit alpha OS=Mus musculus GN=Pafah1b1	−1,23	0,0847348047503324	0,432248923873429	31,9095496846577	31,5898004571495	31,5739501993876	31,5739501993876	31,4680190706971	31,4180321721597	57978891103070,9	47259137972582,3	0,294934426699475	0,0059748257919927	0,0025285051559498	0,815109379870137	-0.294934426699474	1,22682921420843	0.294934426699474	992
Q9D6Y7-2	sp|Q9D6Y7-2|MSRA_MOUSE Isoform 2 of Mitochondrial peptide methionine sulfoxide reductase OS=Mus musculus GN=Msra;sp|Q9D6Y7|MSRA_MOUSE Mitochondrial peptide methionine sulfoxide reductase OS=Mus musculus GN=Msra PE=1 SV=1;sp|Q9D6Y7-4|MSRA_MOUSE Isoform 4	−1,23	0,541184613492164	0,832408322819698	26,7397710425091	27,1516611790053	27,3089720088055	27,4384765453677	26,5520528301124	26,596578656695	568803807941,729	463637736557,431	0,294933127886529	0,010859647116185	0,018591804951466	0,815110113687791	-0.29493312788653	1,22682810973319	0.29493312788653	993
P18654	sp|P18654|KS6A3_MOUSE Ribosomal protein S6 kinase alpha-3 OS=Mus musculus GN=Rps6ka3 PE=1 SV=2	−1,23	0,244458167043155	0,668311722366731	26,7663350011133	26,6691328676683	26,4958547362392	26,696871523607	26,1555786458727	26,4656131249857	372601082985,711	303714645523,431	0,294915388705764	0,0051423510932247	0,0102725706535417	0,815120136231805	-0.294915388705763	1,22681302491541	0.294915388705763	994
Q8BLR2	sp|Q8BLR2|CPNE4_MOUSE Copine-4 OS=Mus musculus GN=Cpne4 PE=1 SV=1	−1,23	0,366069636527385	0,739315231400715	28,8541446687965	29,5576191780415	29,3880565211407	29,21883644974	28,9967840041252	28,9711760736878	5132463064028,54	4183902373746,36	0,294802151509622	0,012544200019837	0,0046864068255168	0,815184117557459	-0.294802151509622	1,22671673608694	0.294802151509622	995
Q9D706	sp|Q9D706|RPAP3_MOUSE RNA polymerase II-associated protein 3 OS=Mus musculus GN=Rpap3 PE=1 SV=1	−1,23	0,120035870077231	0,494850891206355	26,5591097026922	26,6147061508255	26,2872690468727	26,1288765661872	26,417345126627	26,3021104634812	318544589353,544	259695956454,88	0,294671781732287	0,0066151533781232	0,0055246368838202	0,815257785360311	-0.294671781732288	1,22660588829341	0.294671781732288	996

Q8VHR5	sp|Q8VHR5|P66B_MOUSE Transcriptional repressor p66-beta OS=Mus musculus GN=Gatad2b PE=1 SV=1;sp|Q8VHR5-2|P66B_MOUSE Isoform 2 of Transcriptional repressor p66-beta OS=Mus musculus GN=Gatad2b	−1,23	0,34741899343986	0,732776399267436	26,1808314726337	26,2191523428398	25,5815878578865	25,6830722098595	25,7705950123853	25,9154627291353	194530971620,866	158609331925,222	0,294522211711239	0,013755153662051	0,0045509834501695	0,815342310808718	-0.294522211711238	1,2264787276992	0.294522211711238	997
Q9R1Q9	sp|Q9R1Q9|VAS1_MOUSE V-type proton ATPase subunit S1 OS=Mus musculus GN=Atp6ap1 PE=1 SV=1	−1,23	0,0695992349242664	0,39214291659106	29,1893852573842	28,8247153553848	28,9871137767746	28,8443921491069	28,8247153553848	28,7217355155172	3932926009703,04	3208894731380,28	0,29352658264275	0,00629983965894661	0,0022875503676755	0,815905186993989	-0.293526582642751	1,22563260528379	0.293526582642751	998
P36993-2	sp|P36993-2|PPM1B_MOUSE Isoform Beta-2 of Protein phosphatase 1B OS=Mus musculus GN=Ppm1b;sp|P36993-4|PPM1B_MOUSE Isoform Beta-4 of Protein phosphatase 1B OS=Mus musculus GN=Ppm1b;sp|P36993|PPM1B_MOUSE Protein phosphatase 1B OS=Mus musculus GN=Ppm1b PE=	−1,23	0,231216276771866	0,654704104970368	25,9023194601262	25,7112604959504	25,4105254331557	25,4392340632725	25,3314691252537	25,6433260264358	141377831066,694	115362256598,755	0,293384623728093	0,00965646638020905	0,0062183026142904	0,81598547472647	-0.293384623728093	1,2255120109034	0.293384623728094	999
Q923Z3	sp|Q923Z3|MTO1_MOUSE Protein MTO1 homolog, mitochondrial OS=Mus musculus GN=Mto1 PE=2 SV=1	−1,23	0,500314063485843	0,812083935271593	23,6434784339963	23,7937470144796	22,914286016571	23,1722651329508	23,0672620680928	23,5022385635319	15290595783,3212	12478278564,7704	0,293225699424427	0,0200600026387019	0,0097636249130185	0,816075366950813	-0.293225699424424	1,22537701846878	0.293225699424424	1000
Q61171	sp|Q61171|PRDX2_MOUSE Peroxiredoxin-2 OS=Mus musculus GN=Prdx2 PE=1 SV=3	−1,22	0,323193537820313	0,726585174921446	33,9275094387217	33,7277908946237	33,4446452460359	33,2226868067924	33,5323458377698	33,7374582476704	432231249099930	353002750309857	0,292123954904717	0,00719971613217674	0,0077363712688527	0,816698818155659	-0.292123954904717	1,22444159066899	0.292123954904717	1001
Q9D6R2	sp|Q9D6R2|IDH3A_MOUSE Isocitrate dehydrogenase [NAD] subunit alpha, mitochondrial OS=Mus musculus GN=Idh3a PE=1 SV=1;sp|Q9D6R2-2|IDH3A_MOUSE Isoform 2 of Isocitrate dehydrogenase [NAD] subunit alpha, mitochondrial OS=Mus musculus GN=Idh3a	−1,22	0,192027426913901	0,618802084535046	34,4014332388837	33,8956357977178	34,0061355969496	33,9115726182197	33,8575362770501	33,9275094387217	645514126901707	527343248563630	0,291706348748574	0,007798187373937	0,0010818821834783	0,816935256079886	-0.291706348748575	1,22408721200082	0.291706348748575	1002
P52483	sp|P52483|UB2E3_MOUSE Ubiquitin-conjugating enzyme E2 E3 OS=Mus musculus GN=Ube2e3 PE=1 SV=2	−1,22	0,569923588248222	0,847324981021513	23,3877341976412	23,4615901922734	23,5469090051832	23,7251359806086	23,493812176176	22,5716530900109	15520244907,4975	12683068461,7521	0,291247498986186	0,00339462044477699	0,0262319276988028	0,817195124003822	-0.291247498986184	1,22369795245538	0.291247498986184	1003
Q8R035	sp|Q8R035|ICT1_MOUSE Peptidyl-tRNA hydrolase ICT1, mitochondrial OS=Mus musculus GN=Ict1 PE=1 SV=1;sp|Q8R035-2|ICT1_MOUSE Isoform 2 of Peptidyl-tRNA hydrolase ICT1, mitochondrial OS=Mus musculus GN=Ict1	−1,22	0,307118545256347	0,71837258919956	25,0243761876188	24,9825849920557	24,7957915615014	24,3684748551525	24,8599080669657	24,9689418598456	67422923864,7175	55101434871,3167	0,291149304790286	0,00488149219749959	0,0129337917148483	0,817250746672992	-0.291149304790286	1,22361466669927	0.291149304790286	1004
Q9DBE0	sp|Q9DBE0|CSAD_MOUSE Cysteine sulfinic acid decarboxylase OS=Mus musculus GN=Csad PE=2 SV=1	−1,22	0,0175310697779956	0,179292377689348	26,0415130488842	25,9556095100875	25,9217837339592	25,6268016812449	25,8889079988918	25,7991721647102	190509664843,011	155767022380,656	0,290474358608789	0,00237644977516989	0,0051687266647413	0,817633176295887	-0.290474358608785	1,22304234831846	0.290474358608785	1005
Q924H7-2	sp|Q924H7-2|WAC_MOUSE Isoform 2 of WW domain-containing adapter protein with coiled-coil OS=Mus musculus GN=Wac;sp|Q924H7|WAC_MOUSE WW domain-containing adapter protein with coiled-coil OS=Mus musculus GN=Wac PE=1 SV=2;sp|Q924H7-3|WAC_MOUSE Isoform 3 of	−1,22	0,725804302057242	0,921775525192452	21,736986923389	22,8052947352916	22,3631795486139	21,1351427286112	22,61434833139	22,5521406244179	4847943359,78005	3964096886,8627	0,290380619398232	0,0240693606605204	0,0378560959437781	0,817686303794306	-0.290380619398234	1,22296288363851	0.290380619398235	1006
Q8BGE6	sp|Q8BGE6|ATG4B_MOUSE Cysteine protease ATG4B OS=Mus musculus GN=Atg4b PE=2 SV=2	−1,22	0,00116446734978298	0,0266945318215402	27,3454914633062	27,3252327797371	27,1520862359721	27,0645280083663	27,0856899088401	27,0716328405969	699946793399,591	572884666101,413	0,289000536522196	0,00389737667241509	0,0003977846212546	0,8184688772113	-0.289000536522196	1,22179355604481	0.289000536522196	1007
Q9QYJ3	sp|Q9QYJ3|DNJB1_MOUSE DnaJ homolog subfamily B member 1 OS=Mus musculus GN=Dnajb1 PE=2 SV=3	−1,22	0,204600792873528	0,635648869851434	28,3588682131998	27,8490417699696	27,9400277759159	27,8373225183337	27,8373225183337	27,8740286712693	1519363459395,49	1244254787834,07	0,288185091591706	0,00969420061095876	0,0007609566856448	0,818931625701412	-0.28818509159171	1,22110316492357	0.28818509159171	1008
Q8BH50-2	sp|Q8BH50-2|CR025_MOUSE Isoform 2 of Uncharacterized protein C18orf25 homolog OS=Mus musculus;sp|Q8BH50|CR025_MOUSE Uncharacterized protein C18orf25 homolog OS=Mus musculus PE=2 SV=1	−1,22	0,319833610694155	0,723342215945077	24,0050047487481	24,3767709733963	24,0899649888494	24,0578211291045	23,6328120342077	24,18201000329	30999809729,8075	25388133637,9627	0,28810501876746	0,00806390011160059	0,012021384726281	0,818977079512557	-0.28810501876746	1,22103539282831	0.28810501876746	1009
P0C7M9	sp|P0C7M9|CLC2L_MOUSE C-type lectin domain family 2 member L OS=Mus musculus GN=Clec2l PE=3 SV=1	−1,22	0,513837432233138	0,819539762695211	24,0031450361206	24,7625063508653	24,7764875068518	23,9801195100244	24,4971412211746	24,4665538370577	44290954152,5982	36282626545,4815	0,287733179119617	0,0180512301735931	0,0119301240497821	0,819188189544864	-0.287733179119617	1,2207207242033	0.287733179119617	1010
Q8VD66	sp|Q8VD66|ABHD4_MOUSE Abhydrolase domain-containing protein 4 OS=Mus musculus GN=Abhd4 PE=2 SV=1	−1,22	0,00573232697182151	0,0831278981632518	25,7400196200249	25,6146994185905	25,789671987075	25,5972870851289	25,4252755505049	25,5244369585765	147161580710,045	120590522438,198	0,287284551888642	0,00350661818185171	0,0033838231115398	0,819442967766156	-0.287284551888643	1,22034118216433	0.287284551888643	1011
P62141	sp|P62141|PP1B_MOUSE Serine/threonine-protein phosphatase PP1-beta catalytic subunit OS=Mus musculus GN=Ppp1cb PE=1 SV=3	−1,22	0,098257706638634	0,461211717096959	32,077801964065	31,7533387554372	31,9879882223648	31,8823889345278	31,687944408101	31,6520009366872	74342926562572,3	60931907925865,5	0,286997566697165	0,00524527969191233	0,0039050208711440	0,819605990014139	-0.286997566697165	1,2200984524073	0.286997566697165	1012
Q99L27	sp|Q99L27|GMPR2_MOUSE GMP reductase 2 OS=Mus musculus GN=Gmpr2 PE=2 SV=2	−1,22	0,157931437191806	0,562677231878812	26,2537200098112	25,7725191212397	25,9468628589073	25,7918978973668	25,7790605133414	25,8054283782832	193982540910,409	158993463723,399	0,286959353752655	0,00937332601146105	0,0005112199395746	0,819627699365121	-0.286959353752657	1,22006613584996	0.286959353752657	1013

Q9QYS9-8	sp|Q9QYS9-8|QKI_MOUSE Isoform 8 of Protein quaking OS=Mus musculus GN=Qki;sp|Q9QYS9-4|QKI_MOUSE Isoform 4 of Protein quaking OS=Mus musculus GN=Qki;sp|Q9QYS9-3|QKI_MOUSE Isoform 3 of Protein quaking OS=Mus musculus GN=Qki;sp|Q9QYS9-2|QKI_MOUSE Isoform	−1,22	0,0738538544568519	0,404916706615332	28,0738882662073	27,7288300481742	27,8064691473181	27,596320761955	27,6943403199442	27,7219629002009	1269607805196,29	1040658353174,3	0,286886391197911	0,0064951621548156	0,0023861277444812	0,819669152091742	-0.286886391197905	1,22000443404277	0.286886391197905	1014
P62962	sp|P62962|PROF1_MOUSE Profilin-1 OS=Mus musculus GN=Pfn1 PE=1 SV=2	−1,22	0,231963481279235	0,656410375016488	32,773886273776	32,5334327252068	32,420921979523	32,1406176218661	32,4047952999906	32,5868757685581	140479168410490	115169902089676	0,286592470211813	0,0055349037029324	0,0069302644267770	0,819836160712038	-0.286592470211812	1,21975590724308	0.286592470211812	1015
P54822	sp|P54822|PUR8_MOUSE Adenylosuccinate lyase OS=Mus musculus GN=Adsl PE=2 SV=2	−1,22	0,330610093984747	0,72953198881961	29,5601460600447	29,1940485808107	29,4746082875131	28,8755859989111	29,441616430095	29,315804571717	5921441170277,84	4854864623711,77	0,28651727699419	0,0065118389282718 4	0,0101746750307002	0,819878891659068	-0.28651727699419	1,2196923352624	0.28651727699419	1016
Q8VDI7	sp|Q8VDI7|UBAC1_MOUSE Ubiquitin-associated domain-containing protein 1 OS=Mus musculus GN=Ubac1 PE=2 SV=2	−1,22	0,149216141150422	0,547936585006497	26,4091064430994	26,0532668033667	26,5038887636353	26,1042219466073	26,1438469895555	26,1226195471115	270107897557,33	221472181640,831	0,286410324546043	0,00902588193837446	0,0007590601506399	0,819939674639924	-0.286410324546043	1,21960191820106	0.286410324546043	1017
Q9QYB1	sp|Q9QYB1|CLIC4_MOUSE Chloride intracellular channel protein 4 OS=Mus musculus GN=Clic4 PE=1 SV=3	−1,22	0,299150412395876	0,71351128730767	29,3492574870933	29,5072210768158	29,8358818720028	29,5576191780415	29,1279023668535	29,4120521281494	6910894153774,03	5668002569065,9	0,286031971058451	0,0083970633418668 1	0,0074423522437759	0,820154735834091	-0.286031971058452	1,2192821138599	0.286031971058452	1018
Q80TS3-4	sp|Q80TS3-4|LPHN3_MOUSE Isoform 4 of Latrophilin-3 OS=Mus musculus GN=Lphn3;sp|Q80TS3-2|LPHN3_MOUSE Isoform 2 of Latrophilin-3 OS=Mus musculus GN=Lphn3;sp|Q80TS3-5|LPHN3_MOUSE Isoform 5 of Latrophilin-3 OS=Mus musculus GN=Lphn3;sp|Q80TS3|LPHN3_MOUSE La	−1,22	0,804220657768524	0,947160759110361	22,9717251193296	21,0533396385392	22,5716530900109	21,5846616401495	23,0829978435626	21,334360086982	4373834622,59007	3587326986,93679	0,285989418440307	0,0455903257014704	0,0429822658161516	0,820178926841196	-0.285989418440309	1,21924615138719	0.285989418440309	1019
P46467	sp|P46467|VPS4B_MOUSE Vacuolar protein sorting-associated protein 4B OS=Mus musculus GN=Vps4b PE=1 SV=2	−1,22	0,263196300561673	0,683437075657571	26,8329937693396	27,2928682782776	27,3163381242936	27,1079700259264	26,8888307780833	26,8535256285277	616546792740,319	506155013873,161	0,284631102875483	0,0100391415454456	0,0051148878400539	0,820951499274681	-0.284631102875483	1,21809875599656	0.284631102875483	1020
P17742	sp|P17742|PPIA_MOUSE Peptidyl-prolyl cis-trans isomerase A OS=Mus musculus GN=Ppia PE=1 SV=2	−1,22	0,309313320620249	0,719081702439134	35,211446325403	35,1702667677213	35,0731894837483	34,6005663418216	35,016095172406	35,247571066123 7	1845698501382730	1515837055917970	0,284052224927665	0,0020194437157023 4	0,0093788827095341	0,821280970203075	-0.284052224927662	1,21761009481656	0.284052224927662	1021
P0CW02	sp|P0CW02|LY6C1_MOUSE Lymphocyte antigen 6C1 OS=Mus musculus GN=Ly6c1 PE=2 SV=1;sp|P0CW03|LY6C2_MOUSE Lymphocyte antigen 6C2 OS=Mus musculus GN=Ly6c2 PE=1 SV=1	−1,22	0,306953852573262	0,71837258919956	24,4023850233445	24,3880879517679	24,2384213358069	23,8383590411367	24,1206114949404	24,4792576365056	37326328417,9539	30655442641,9759	0,284050369533069	0,00373081279610013	0,0133026679113955	0,82128202642161 5	-0.284050369533071	1,21760852889606	0.284050369533071	1022
Q9WTT4	sp|Q9WTT4|VATG2_MOUSE V-type proton ATPase subunit G 2 OS=Mus musculus GN=Atp6v1g2 PE=2 SV=1	−1,22	0,549215624298717	0,838092765530185	31,0680797664268	30,6232978272985	30,1011524211797	30,1327617042981	30,3514194051073	30,7195270753918	19423601310228,1	15962064617765,9	0,283163444754563	0,015817601366437	0,0097541367248144	0,821787080718166	-0.283163444754561	1,21686021046485	0.283163444754561	1023
Q9R1V4	sp|Q9R1V4|ADA11_MOUSE Disintegrin and metalloproteinase domain-containing protein 11 OS=Mus musculus GN=Adam11 PE=1 SV=2	−1,22	0,609143185831877	0,866608542040832	25,0849820401459	25,0392995070299	25,5677487880404	24,882110644788	25,6922263661063	24,5294754056694	90686721071,5935	74540179481,8789	0,282873024734034	0,0116051262016708	0,0238144902786136	0,821952526247282	-0.282873024734034	1,21661527651191	0.282873024734033	1024
Q9QXB9	sp|Q9QXB9|DRG2_MOUSE Developmentally-regulated GTP-binding protein 2 OS=Mus musculus GN=Drg2 PE=1 SV=1	−1,22	0,168254211830673	0,577689763615299	29,3820077371923	28,9070949594495	29,0726137546512	28,8842531253939	28,9822197969154	28,9070949594495	4435103863332,14	3645529095070,57	0,282839674857615	0,0082780407702939	0,0017720544081501	0,821971527027925	-0.282839674857616	1,21658715310467	0.282839674857616	1025
Q9D8C2	sp|Q9D8C2|TSN13_MOUSE Tetraspanin-13 OS=Mus musculus GN=Tspan13 PE=2 SV=1;sp|Q9CQ88|TSN31_MOUSE Tetraspanin-31 OS=Mus musculus GN=Tspan31 PE=1 SV=1	−1,22	0,747714113313827	0,928122477394312	21,711461611698	22,4968102137115	23,0076169888695	22,5286124376778	22,8291248292886	21,2714023663618	5376461448,6518	4421360146,31728	0,282166711201093	0,0291406837495221	0,0372078662014465	0,8223550356575 1	-0.282166711201092	1,21601979271697	0.282166711201091	1026
Q8BGZ1	sp|Q8BGZ1|HPCL4_MOUSE Hippocalcin-like protein 4 OS=Mus musculus GN=Hpcal4 PE=2 SV=3	−1,22	0,200694615652824	0,6308537814738	32,2317495018469	31,8989697166028	31,8280562746036	31,6602286177643	31,7533387554372	31,9593241419042	77885311694628,8	64067852844559,2	0,281750636507064	0,00673845066273885	0,0048144630874624	0,8225922378760 6	-0.281750636507065	1,21566914195788	0.281750636507065	1027
Q9Z2I9	sp|Q9Z2I9|SUCB1_MOUSE Succinyl-CoA ligase [ADP-forming] subunit beta, mitochondrial OS=Mus musculus GN=Sucla2 PE=1 SV=2	−1,22	0,0387253865288074	0,288186689063277	33,5323458377698	33,2547258597017	33,2755034398491	33,1006254059203	33,1743276583604	33,2027166374192	30587318761917 4	25169099328560 5	0,28128005711967	0,00463615987914877	0,0015891504980992	0,822860595414371	-0.281280057119671	1,21527267871714	0.281280057119671	1028
P08556	sp|P08556|RASN_MOUSE GTPase NRas OS=Mus musculus GN=Nras PE=2 SV=1	−1,22	0,449788583173451	0,786343703431612	26,9109358823026	26,7159035528554	26,6511319794876	26,0840270683502	26,7143626330946	26,894872517499	418241061285,196	344176601709,003	0,281185685667096	0,00505375258060054	0,0160255931511018	0,82291442320703	-0.281185685667097	1,21519318631315	0.281185685667097	1029

P32020	sp|P32020|NLTP_MOUSE Non-specific lipid-transfer protein OS=Mus musculus GN=Scp2 PE=1 SV=3;sp|P32020-2|NLTP_MOUSE Isoform SCP2 of Non-specific lipid-transfer protein OS=Mus musculus GN=Scp2	−1,21	0,450455195532909	0,786343703431612	29,3099584234601	29,1075726323578	28,7849374021746	28,4869189419964	28,9317927292942	29,1996198193394	4205816171485,52	3461687017708,53	0,280910502004546	0,00910968589302455	0,0124683846951431	0,823071403162597	-0.280910502004547	1,21496141909143	0.280910502004547	1030
Q8BSK8-2	sp|Q8BSK8-2|KS6B1_MOUSE Isoform Alpha II of Ribosomal protein S6 kinase beta-1 OS=Mus musculus GN=Rps6kb1;sp|Q8BSK8|KS6B1_MOUSE Ribosomal protein S6 kinase beta-1 OS=Mus musculus GN=Rps6kb1 PE=1 SV=2	−1,21	0,664023503661284	0,896648481102019	23,4577010632101	23,5718044898966	23,8903181590625	23,8999487036671	22,5875559426552	23,8482598894016	18479747458,2917	15210546079,5832	0,280873092481058	0,00948448779729923	0,031701020915091	0,823092745932432	-0.280873092481057	1,21492991517883	0.280873092481057	1031
Q80X19-2	sp|Q80X19-2|COEA1_MOUSE Isoform 2 of Collagen alpha-1(XIV) chain OS=Mus musculus GN=Col14a1;sp|Q80X19|COEA1_MOUSE Collagen alpha-1(XIV) chain OS=Mus musculus GN=Col14a1 PE=2 SV=2	−1,21	0,775290065723924	0,938619589104496	21,2911396120514	22,7276309950859	20,6348824710516	21,4618051524005	21,7927037897341	20,8156783455322	2288626499,58218	1884124504,087	0,28058773417153	0,0496649828902833	0,0232674160447127	0,823255565917363	-0.280587734171529	1,2146896315067	0.280587734171529	1032
Q9JM96	sp|Q9JM96|BORG4_MOUSE Cdc42 effector protein 4 OS=Mus musculus GN=Cdc42ep4 PE=1 SV=1	−1,21	0,262595783733596	0,682806179310143	25,6404580141254	25,4142056413629	25,1249989581468	25,2573533322883	25,0264181578287	25,3139278902866	106692944797,939	87879564321,7796	0,279865156854268	0,0101747417356379	0,0060443089588807	0,823667998743598	-0.279865156854262	1,21408140358175	0.279865156854262	1033
Q60668-3	sp|Q60668-3|HNRPD_MOUSE Isoform 3 of Heterogeneous nuclear ribonucleoprotein D0 OS=Mus musculus GN=Hnrnpd;sp|Q60668|HNRPD_MOUSE Heterogeneous nuclear ribonucleoprotein D0 OS=Mus musculus GN=Hnrnpd PE=1 SV=2;sp|Q60668-4|HNRPD_MOUSE Isoform 4 of Heterogen	−1,21	0,0544152198522838	0,341924788314513	30,9542765997972	30,7933119851167	30,9837659274804	30,7768742173066	30,8180134544735	30,5561361051944	26560639136763	21889056521818,2	0,279080091355142	0,00331641006525145	0,0045847364786827	0,824116332785125	-0.279080091355145	1,21342092155906	0.279080091355145	1034
P22599	sp|P22599|A1AT2_MOUSE Alpha-1-antitrypsin 1-2 OS=Mus musculus GN=Serpina1b PE=1 SV=2	−1,21	0,379105983624319	0,748785300113128	27,6606396741905	28,1245214621915	27,7510871215627	27,8140234660062	27,8257406617401	27,3163381242936	123911970275,23	1021235295017,55	0,278991255236778	0,00883113306299645	0,0105156912962392	0,824167080551013	-0.278991255236777	1,21334620564004	0.278991255236777	1035
Q8BSL7	sp|Q8BSL7|ARF2_MOUSE ADP-ribosylation factor 2 OS=Mus musculus GN=Arf2 PE=1 SV=2	−1,21	0,146191580175764	0,541684557075751	26,0654090296045	25,9455567246878	26,1633273553353	25,8417503358081	26,0782885894433	25,6741775613297	207437868571,666	170967416560,061	0,27895788913488	0,00418561866677166	0,0078498355975879	0,824186141794042	-0.278957889134876	1,21331814415522	0.278957889134876	1036
P59326	sp|P59326|YTHD1_MOUSE YTH domain-containing family protein 1 OS=Mus musculus GN=Ythdf1 PE=2 SV=1	−1,21	0,582051563283875	0,853379506269256	24,6669262741989	25,4982180151912	25,520279157572	25,2432823524486	24,6164760537761	25,2470629747378	90487221819,6889	74614979903,0244	0,278248777088617	0,0192813915622191	0,0144986729183848	0,824591344529368	-0.278248777088616	1,21272192175476	0.278248777088616	1037
P50096	sp|P50096|IMDH1_MOUSE Inosine-5-monophosphate dehydrogenase 1 OS=Mus musculus GN=Impdh1 PE=2 SV=2	−1,21	0,0577735026113987	0,355202783224204	26,1521497135295	26,4634183601821	26,2771103257199	26,1606183781872	26,0128955673314	26,140845402906	263563316524,218	217352533249,372	0,278112675646446	0,00595639510965531	0,0030719118830342	0,824669138769925	-0.278112675646446	1,21260752098908	0.278112675646446	1038
Q60803	sp|Q60803|TRAF3_MOUSE TNF receptor-associated factor 3 OS=Mus musculus GN=Traf3 PE=1 SV=2	−1,21	0,319481560401833	0,723255263174026	24,1475198498776	24,132235856752	24,7137513679622	24,1861387683333	24,1399594220105	24,0899649888494	36888623154,7328	30429784725,0169	0,277691814761161	0,013620970985393	0,0019926310930902	0,824909745136768	-0.277691814761164	1,21225383248952	0.277691814761164	1039
Q9Z131-3	sp|Q9Z131-3|3BP5_MOUSE Isoform 3 of SH3 domain-binding protein 5 OS=Mus musculus GN=Sh3bp5;sp|Q9Z131-2|3BP5_MOUSE Isoform 2 of SH3 domain-binding protein 5 OS=Mus musculus GN=Sh3bp5;sp|Q9Z131|3BP5_MOUSE SH3 domain-binding protein 5 OS=Mus musculus GN=Sh	−1,21	0,756796932421707	0,931806144200878	22,5083970764616	20,5963942637238	22,0562287492798	21,9376699279833	21,1423031271158	21,504041661994	2710340283,49816	2236112950,94586	0,27748092976252	0,046006510198872	0,0184979758817393	0,82503033458949	-0.277480929762523	1,21207664503338	0.277480929762523	1040
P70429-2	sp|P70429-2|EVL_MOUSE Isoform 1 of Ena/VASP-like protein OS=Mus musculus GN=Evl;sp|P70429|EVL_MOUSE Ena/VASP-like protein OS=Mus musculus GN=Evl PE=1 SV=2	−1,21	0,0993801399601177	0,463127777493814	28,092191112154	27,7199698313342	27,791225622099	27,6281717314036	27,6904097335274	27,7082701854407	1267155222829,23	1045605455467,97	0,277254687693531	0,00708958872872869	0,0015193243859494	0,825159725209831	-0.277254687693522	1,2118865832257	0.277254687693522	1041
P84078	sp|P84078|ARF1_MOUSE ADP-ribosylation factor 1 OS=Mus musculus GN=Arf1 PE=1 SV=2;sp|P61205|ARF3_MOUSE ADP-ribosylation factor 3 OS=Mus musculus GN=Arf3 PE=2 SV=2	−1,21	0,168224859480097	0,577689763615299	34,7308080145928	34,5639686668958	34,4014332388837	34,1841736380791	34,4014332388837	34,5341448386175	1026984575534680	847447979230324	0,277217797777713	0,00476465073801311	0,0051400298182435	0,825180824930225	-0.277217797777711	1,21185559551091	0.27721779777771	1042
P21278	sp|P21278|GNA11_MOUSE Guanine nucleotide-binding protein subunit alpha-11 OS=Mus musculus GN=Gna11 PE=1 SV=1	−1,21	0,37567741259191	0,746362141983349	29,4521853631682	29,0156519772701	28,8042521530299	28,696381711632	29,0311658380649	28,9686502897513	4304561962769,19	3552712858789,97	0,276945344448006	0,0113583092500722	0,0061596791619599	0,82533667525707	-0.276945344448009	1,21162675787857	0.276945344448009	1043
Q8CJG1	sp|Q8CJG1|AGO1_MOUSE Protein argonaute-1 OS=Mus musculus GN=Ago1 PE=1 SV=2	−1,21	0,357834758990432	0,732843629012038	26,2070979591574	25,7281474875069	25,5531568912004	25,7523846556078	25,502807308767	25,6579078890505	165042267705,697	136242190462,659	0,276662013770594	0,0131067183678986	0,0049150218362235	0,825498778928594	-0.276662013770591	1,21138883003302	0.276662013770591	1044

Q9JJG6	sp|Q9JJG6|TMM47_MOUSE Transmembrane protein 47 OS=Mus musculus GN=Tmem47 PE=1 SV=1;sp|Q9JJG6-2|TMM47_MOUSE Isoform 2 of Transmembrane protein 47 OS=Mus musculus GN=Tmem47	−1,21	0,303656292507043	0,716532404521184	23,2704101887496	23,2474511581564	22,7116660906337	22,84403303344	22,8549362691204	22,9565167887036	10519632875,1391	8687595411,72251	0,276055534512399	0,0137010246709288	0,0027107101402481	0,825845874550792	-0.276055534512396	1,21087969416077	0.276055534512396	1045
Q80ZW2	sp|Q80ZW2|THEM6_MOUSE Protein THEM6 OS=Mus musculus GN=Them6 PE=2 SV=1	−1,21	0,394962024907329	0,753881747531964	27,5915719767911	27,4433741140667	27,1705289713067	26,8505074822295	27,2983095342163	27,4832478791861	795172588682,18	656828179596,576	0,275752001217364	0,00779416905172441	0,0119564496165895	0,826019645225851	-0.275752001217363	1,2106249600475	0.275752001217363	1046
O35098	sp|O35098|DPYL4_MOUSE Dihydropyrimidinase-related protein 4 OS=Mus musculus GN=Dpysl4 PE=1 SV=1;sp|O35098-2|DPYL4_MOUSE Isoform 2 of Dihydropyrimidinase-related protein 4 OS=Mus musculus GN=Dpysl4	−1,21	0,464267271042813	0,79405478152268	31,3267661620555	32,043087513796	32,099937215965	31,7786943855435	31,5082584125245	31,6098246071065	66171014172399,6	54665785445655,2	0,27556123851364	0,013540972695077	0,0043185875219384	0,826128874241385	-0.275561238513642	1,2104648937713	0.275561238513642	1047
Q80YN3	sp|Q80YN3|BCAS1_MOUSE Breast carcinoma-amplified sequence 1 homolog OS=Mus musculus GN=Bcas1 PE=2 SV=3	−1,21	0,308668815855923	0,71853230316343	28,1512065915307	28,0488815319204	27,913295506351	27,8568791263886	27,539642809425	28,1438826384783	1501963763180,39	1240829873804,87	0,275544680639216	0,00425648369606879	0,0108538836955289	0,826138355813211	-0.27554468063922	1,21045100129221	0.27554468063922	1048
O08915	sp|O08915|AIP_MOUSE AH receptor-interacting protein OS=Mus musculus GN=Aip PE=1 SV=1	−1,21	0,0847189526017279	0,432248923873429	28,1636447074317	27,8519734871773	27,8762541482515	27,8588173640719	27,7647974432772	27,6959168630177	1395058474629	1152756588472	0,275237683301838	0,006199403434434	0,0029443124052403	0,826314172084122	-0.275237683301837	1,21019345157522	0.275237683301837	1049
Q9CWF6	sp|Q9CWF6|BBS2_MOUSE Bardet-Biedl syndrome 2 protein homolog OS=Mus musculus GN=Bbs2 PE=1 SV=1	−1,21	0,393366067940488	0,753881747531964	25,4664846831164	24,7672234633243	24,9050721699232	24,9566019926909	24,7376841280185	24,8730804771037	75414100627,7944	62334997705,7217	0,274791912683041	0,0147884155938355	0,0044447810640785	0,826569529926181	-0.274791912683041	1,20981957814161	0.274791912683041	1050
P70318	sp|P70318|TIAR_MOUSE Nucleolysin TIAR OS=Mus musculus GN=Tial1 PE=1 SV=1	−1,21	5,83×10^−05	0,00240735438485748	27,7704056984658	27,9101208805373	27,889164479335	27,6294369835375	27,6913415808811	27,6776121109565	1253002373522,84	1035732710598,56	0,274737409786244	0,00270482543698054	0,0011750941265183	0,826600757097194	-0.274737409786249	1,20977387380062	0.274737409786249	1051
Q9D125	sp|Q9D125|RT25_MOUSE 28S ribosomal protein S25, mitochondrial OS=Mus musculus GN=Mrps25 PE=1 SV=1	−1,21	0,154089094831231	0,557500682738605	26,1853542778432	25,9719342937484	25,9004504146625	25,6586432911233	25,8152971897798	26,0128955673314	199533170724,605	164956122198,31	0,274546279169923	0,005696838381243	0,0068729118142876	0,826710273781906	-0.274546279169917	1,20961361158046	0.274546279169917	1052
Q00897	sp|Q00897|A1AT4_MOUSE Alpha-1-antitrypsin 1-4 OS=Mus musculus GN=Serpina1d PE=2 SV=1	−1,21	0,303191085451007	0,716532404521184	26,7629318109403	27,2974019823772	27,322661625401	26,9915324948691	26,8968700970283	26,924184307304	604498568891,194	499827545263,414	0,274308514129321	0,0116530753373752	0,0018086916013106	0,826846531961567	-0.274308514129322	1,20941427622325	0.274308514129322	1053
Q62384	sp|Q62384|ZPR1_MOUSE Zinc finger protein ZPR1 OS=Mus musculus GN=Zpr1 PE=1 SV=1	−1,21	0,112081975793827	0,479943479366209	24,6781682335195	24,9975459496661	25,0609738563947	24,7472989515349	24,6912518557112	24,7280852384171	65954394422,4005	54540643622,5486	0,274137061557679	0,00823566519037263	0,0011520572146718	0,826944801786014	-0.274137061557678	1,2092705557133	0.274137061557678	1054
P32037	sp|P32037|GTR3_MOUSE Solute carrier family 2, facilitated glucose transporter member 3 OS=Mus musculus GN=Slc2a3 PE=1 SV=1	−1,21	0,227565583321517	0,652923723647267	27,8317214951593	27,9495817820908	27,9434810304077	27,5493288208279	28,0224070708488	27,5855513418751	1319483431137,42	1092069618601,6	0,272908404843869	0,00237763585926765	0,009498829063567	0,827649360977739	-0.272908404843875	1,20824113102517	0.272908404843875	1055
P56380	sp|P56380|AP4A_MOUSE Bis(5-nucleosyl)-tetraphosphatase [asymmetrical] OS=Mus musculus GN=Nudt2 PE=1 SV=3	−1,21	0,151404286387065	0,552437900049208	28,4660063045051	28,3294382730544	28,1167813912022	27,9581743773589	28,1438826384783	28,2437154901872	1960215089950,27	1622935239611,06	0,272406533861802	0,00621778126246203	0,0051540271227141	0,827937325822868	-0.272406533861799	1,20782089273016	0.272406533861799	1056
P30416	sp|P30416|FKBP4_MOUSE Peptidyl-prolyl cis-trans isomerase FKBP4 OS=Mus musculus GN=Fkbp4 PE=1 SV=5	−1,21	0,454639173385378	0,788460969617685	29,8358818720028	30,6442031737676	30,3862745237618	30,2570757598888	30,0689715847678	29,9739816452622	14264378381696,4	11810495052134,7	0,272347439570695	0,0136316021200624	0,0047865992953715	0,827971239692404	-0.272347439570698	1,20777142014197	0.272347439570698	1057
Q80ZK0	sp|Q80ZK0|RT10_MOUSE 28S ribosomal protein S10, mitochondrial OS=Mus musculus GN=Mrps10 PE=2 SV=1	−1,21	0,109238221534643	0,474027043542775	25,8577700523384	25,7816672426511	25,6003102586315	25,3783577930722	25,6487332316027	25,6464523299023	151914320489,344	125785987119,212	0,272286663363689	0,00513726880704442	0,0060821661309356	0,828006120252733	-0.272286663363686	1,20772054160031	0.272286663363686	1058
Q8BTV2-2	sp|Q8BTV2-2|CPSF7_MOUSE Isoform 2 of Cleavage and polyadenylation specificity factor subunit 7 OS=Mus musculus GN=Cpsf7;sp|Q8BTV2|CPSF7_MOUSE Cleavage and polyadenylation specificity factor subunit 7 OS=Mus musculus GN=Cpsf7 PE=1 SV=2	−1,21	0,419694326196198	0,769072485508496	27,6683860242425	28,43987847634	28,1810020312617	28,020951024883	27,8507513155764	27,8519734871773	1592652812306,51	1318995980857,27	0,271991634711044	0,0139747011153679	0,0035084679473952	0,828175463393729	-0.271991634711046	1,20747359007977	0.271991634711047	1059
P45952	sp|P45952|ACADM_MOUSE Medium-chain specific acyl-CoA dehydrogenase, mitochondrial OS=Mus musculus GN=Acadm PE=1 SV=1	−1,21	0,218238229009437	0,648158974427884	29,6976512816979	29,4054016596301	29,5484224910215	29,323260678621	29,6000841427096	29,1625520471545	6817349056201,45	5645984061751,49	0,271985796440101	0,00494529162716932	0,0075376114004283	0,828178814845278	-0.271985796440101	1,20746870370842	0.271985796440101	1060
O35188	sp|O35188|X3CL1_MOUSE Fractalkine OS=Mus musculus GN=Cx3cl1 PE=2 SV=3	−1,21	0,447661493256448	0,78574459315198	23,9042039329199	23,8201140153781	23,8091764130591	23,7163979450698	24,0493524445907	23,2024655836816	22674303604,8643	18780256919,4348	0,271841442370309	0,00218059325915905	0,0180357135864197	0,828261685417579	-0.271841442370304	1,2073478921047	0.271841442370304	1061
Q3UGC7	sp|Q3UGC7|EI3JA_MOUSE Eukaryotic translation initiation factor 3 subunit J-A OS=Mus musculus GN=Eif3j1 PE=2 SV=1;sp|Q66JS6|EI3JB_MOUSE Eukaryotic translation initiation factor 3 subunit J-B OS=Mus musculus GN=Eif3j2 PE=1 SV=1	−1,21	0,258155711894773	0,67968190098123	28,7253092782756	28,3342637623689	28,6090306171416	28,1288847943823	28,4754028990078	28,4991310077434	2522322498142,08	2089208148319,22	0,271796511382555	0,00703189741161678	0,0073058836313443	0,828287481025171	-0.271796511382554	1,20731029130405	0.271796511382554	1062
Q921I1	sp|Q921I1|TRFE_MOUSE Serotransferrin OS=Mus musculus GN=Tf PE=1 SV=1	−1,21	0,264736997741279	0,68394025335819	31,1607157709667	31,1184365708611	30,7354901350717	30,9306955015522	30,9037738640846	30,6159575258508	29190978606442,5	24186336055620,7	0,271330342355321	0,00755542235363866	0,0056612777160317	0,828555163624516	-0.271330342355322	1,20692024369928	0.271330342355322	1063
Q8VHL1	sp|Q8VHL1|SETD7_MOUSE Histone-lysine N-methyltransferase SETD7 OS=Mus musculus GN=Setd7 PE=2 SV=2	−1,21	0,000104952395081457	0,00387625266185097	29,1591429707384	29,183067170165	29,2130685094284	28,995304264796	28,9188433011032	29,0776180365338	4730693303310,33	3920558369597,61	0,27099249322658	0,00092580760766302	0,0027383745166893	0,828749216706603	-0.270992493226581	1,2066376412082	0.270992493226581	1064

Q8CHS8-2	sp|Q8CHS8-2|VP37A_MOUSE Isoform 2 of Vacuolar protein sorting-associated protein 37A OS=Mus musculus GN=Vps37a;sp|Q8CHS8-3|VP37A_MOUSE Isoform 3 of Vacuolar protein sorting-associated protein 37A OS=Mus musculus GN=Vps37a;sp|Q8CHS8|VP37A_MOUSE Vacuolar	−1,21	0,359306192711463	0,733314212552756	24,0639628296117	23,4260786804776	23,8680352044495	23,4721841914717	23,5787835721751	23,7444339745312	21386496850,2867	17728994542,9557	0,270589466009384	0,0137372291848319	0,0058134321220414	0,828980766091106	-0.270589466009384	1,20630060539921	0.270589466009384	1065
Q9CXW4	sp|Q9CXW4|RL11_MOUSE 60S ribosomal protein L11 OS=Mus musculus GN=Rpl11 PE=1 SV=4	−1,21	0,281271805011913	0,701479518103887	27,0177302526787	26,7414467689503	26,6277756439543	26,3678330475435	26,6443531342949	26,8127695771732	433713878943,014	359621737323,841	0,270263383368652	0,00748436878766401	0,0084427122925963	0,829168156205331	-0.270263383368652	1,20602798421068	0.270263383368652	1066
P63240	sp|P63240|NEC1_MUSCO Neuroendocrine convertase 1 OS=Mus cookii GN=Pcsk1 PE=2 SV=1;sp|P63239|NEC1_MOUSE Neuroendocrine convertase 1 OS=Mus musculus GN=Pcsk1 PE=1 SV=1	−1,21	0,597435886981904	0,861464850775537	22,8549362691204	23,3681777993606	23,245900105076	22,343926520924	23,3845394580678	23,1798328591074	11393832735,5725	9451439983,34106	0,269647077938445	0,0115772299188431	0,0240009982311543	0,829522444526755	-0.269647077938441	1,20551289069762	0.26964707793844	1067
Q921Z5	sp|Q921Z5|TFIP8_MOUSE Tumor necrosis factor alpha-induced protein 8 OS=Mus musculus GN=Tnfaip8 PE=2 SV=1;sp|Q921Z5-2|TFIP8_MOUSE Isoform 2 of Tumor necrosis factor alpha-induced protein 8 OS=Mus musculus GN=Tnfaip8	−1,21	0,371985680863217	0,744143071638935	25,7958217310218	25,6181538792891	26,0846949698555	25,6205393435449	25,3853556236696	25,9331697089281	165608133559,479	137426476138,557	0,269113554195721	0,00911508954268709	0,0107156724894508	0,82982926734815	-0.269113554195722	1,20506716182192	0.269113554195722	1068
Q69ZF3	sp|Q69ZF3|GBA2_MOUSE Non-lysosomal glucosylceramidase OS=Mus musculus GN=Gba2 PE=1 SV=2	−1,20	0,451492923644953	0,786543907075754	27,6568502118367	26,8403763238565	27,1591850873045	27,1273945621664	27,072984791289	26,8968700970283	662180407297,032	549577964581,503	0,268900164446201	0,0151178210529875	0,0044575302080030	0,829952016890437	-0.2689001644462	1,20488893291287	0.2689001644462	1069
Q78ZA7	sp|Q78ZA7|NP1L4_MOUSE Nucleosome assembly protein 1-like 4 OS=Mus musculus GN=Nap1l4 PE=1 SV=1	−1,20	0,423270495004674	0,7711031271846	29,0665390787366	29,5975087341519	29,339015370216	29,4203370103753	29,2013315647317	28,8240709718089	5492224026181,24	4561076786512,77	0,26801601536654	0,00905135012405888	0,0103474311180465	0,830460805089209	-0.26801601536654	1,2041507484421	0.26801601536654	1070
Q9CPW4	sp|Q9CPW4|ARPC5_MOUSE Actin-related protein 2/3 complex subunit 5 OS=Mus musculus GN=Arpc5 PE=2 SV=3	−1,20	0,0258642399587334	0,223189705651504	29,4570920219591	29,4671293506286	29,3261521639233	29,1006990223782	29,2543000086044	29,339015370216	5964170966510,13	4954602872775,82	0,267552188489551	0,00267384572635984	0,0041327587516182	0,830727841404411	-0.267552188489549	1,20376367585011	0.267552188489549	1071
Q9ES28-3	sp|Q9ES28-3|ARHG7_MOUSE Isoform C of Rho guanine nucleotide exchange factor 7 OS=Mus musculus GN=Arhgef7;sp|Q9ES28|ARHG7_MOUSE Rho guanine nucleotide exchange factor 7 OS=Mus musculus GN=Arhgef7 PE=1 SV=2;sp|Q9ES28-2|ARHG7_MOUSE Isoform A of Rho guanine	−1,20	0,248946490717634	0,673464050610029	29,8014346095597	29,4883704069559	29,2543000086044	29,3099584234601	29,2944931553283	29,383338090787	6577669867309,27	5464333232172,3	0,267531134871555	0,00930099404318356	0,0016183265235021	0,830739964516887	-0.267531134871553	1,20374610914686	0.267531134871553	1072
Q9DCS3	sp|Q9DCS3|MECR_MOUSE Trans-2-enoyl-CoA reductase, mitochondrial OS=Mus musculus GN=Mecr PE=1 SV=2	−1,20	0,420776531360021	0,769812444918636	29,183067170165	28,801510817816	28,496957284198	28,6410826370046	28,8443921491069	28,43987847634	3307383217954,94	2747697545738,12	0,267467009088569	0,0119253559624896	0,0070616322832486	0,830776890570639	-0.267467009088569	1,20369260549981	0.267467009088569	1073
P47964	sp|P47964|RL36_MOUSE 60S ribosomal protein L36 OS=Mus musculus GN=Rpl36 PE=2 SV=2	−1,20	0,71159008630491	0,916805961322165	24,49311460887	23,2567715611815	23,9424348342287	23,9989811215525	24,1228372462825	23,0151791532857	23907076376,531	19867131247,9528	0,267054145081136	0,0259190280855247	0,0255951305046436	0,831014672603626	-0.267054145081137	1,20334818742361	0.267054145081137	1074
Q9DAR7	sp|Q9DAR7|DCPS_MOUSE m7GpppX diphosphatase OS=Mus musculus GN=Dcps PE=1 SV=1	−1,20	0,164276940081901	0,572258657737337	28,8605637488314	28,429387167353	28,6000440221418	28,3908102287772	28,5325900295205	28,4120046915943	2715502982582,15	2257174629792,33	0,266701408680193	0,00758447124844451	0,0026885467651732	0,831217879070787	-0.266701408680188	1,20305400687229	0.266701408680188	1075
P97797-2	sp|P97797-2|SHPS1_MOUSE Isoform 2 of Tyrosine-protein phosphatase non-receptor type substrate 1 OS=Mus musculus GN=Sirpa	−1,20	0,257088479414579	0,67968190098123	30,5474125413229	30,3063347984404	29,9903371484726	30,049400664825	30,1253373252879	30,1150222657986	14158857219201,2	11770137883648,1	0,266573607006294	0,00922600810161899	0,0013685449803895	0,831291516075627	-0.266573607006299	1,20294743860832	0.266573607006298	1076
Q9D358	sp|Q9D358|PPAC_MOUSE Low molecular weight phosphotyrosine protein phosphatase OS=Mus musculus GN=Acp1 PE=1 SV=3	−1,20	0,321535210183333	0,724291969738039	29,472404298724	29,2944931553283	29,1687376261914	28,8266770279669	29,1625520471545	29,3922159985882	5370157534654,07	4464366138436,72	0,266509058045659	0,00520532999909077	0,0097650093446028	0,83132871049458	-0.26650905804566	1,20289361762217	0.26650905804566	1077
Q8CJG0	sp|Q8CJG0|AGO2_MOUSE Protein argonaute-2 OS=Mus musculus GN=Ago2 PE=1 SV=3	−1,20	0,335801784949526	0,72953198881961	27,7483704429287	27,1802077444987	27,1854010346971	27,1210844296353	27,235518194489	27,2041523196231	771287017193,093	641399480708,008	0,266044640971309	0,0119300041471907	0,0021749968716834	0,831596366087195	-0.266044640971307	1,2025064571953	0.266044640971307	1078
Q9CXV1	sp|Q9CXV1|DHSD_MOUSE Succinate dehydrogenase [ubiquinone] cytochrome b small subunit, mitochondrial OS=Mus musculus GN=Sdhd PE=2 SV=2	−1,20	0,317629965392229	0,722907157640623	24,1446543239673	23,9952333945482	23,6630592406412	23,5235637626522	23,8091764130591	23,917090549966	24805113367,8611	20628585061,503	0,265992682358638	0,0102993959937454	0,0085617122429958	0,831626316541272	-0.265992682358638	1,20246314974614	0.265992682358638	1079
P28740	sp|P28740|KIF2A_MOUSE Kinesin-like protein KIF2A OS=Mus musculus GN=Kif2a PE=1 SV=2	−1,20	0,334907788304766	0,72953198881961	30,1253373252879	29,8120687934487	29,6053749310012	29,7871965151757	29,4300026872135	29,7730411693604	9175827378034,89	7632323658425,11	0,265715898674282	0,00877106613241811	0,0068186193992087	0,831785880878206	-0.265715898674282	1,20223247711802	0.265715898674282	1080
Q9CQ02	sp|Q9CQ02|COMD4_MOUSE COMM domain-containing protein 4 OS=Mus musculus GN=Commd4 PE=2 SV=1	−1,20	0,383852350594732	0,751327160407723	26,6308893490547	26,2285373005935	26,1042219466073	25,8742929382005	26,1869977779283	26,3498310529515	269872698777,709	224477336873,272	0,265709237841227	0,010459007279775	0,0092463856739704	0,831789721190625	-0.265709237841224	1,20222692649844	0.265709237841224	1081
P53994	sp|P53994|RAB2A_MOUSE Ras-related protein Rab-2A OS=Mus musculus GN=Rab2a PE=1 SV=1	−1,20	0,211593332417257	0,642852835235561	31,7211883042871	31,5739501993876	31,3817725927108	31,1656015127658	31,42290936091	31,5364546439614	50802687134468,7	42265341061735,7	0,26542971643361	0,0053931726407583	0,0060562250992604	0,831950895626138	-0.265429716433608	1,20199401822554	0.265429716433608	1082

Q3TX08	sp|Q3TX08|TRM1_MOUSE tRNA (guanine(26)-N(2))-dimethyltransferase OS=Mus musculus GN=Trmt1 PE=1 SV=2	−1,20	0,663140578639988	0,896098566968415	23,2077788046151	23,326766206189	22,1514329050445	22,8640191705498	22,9492007514796	22,3211019379397	8776345886,79325	7302193504,17544	0,265290485364604	0,0282577998377672	0,0150019414875409	0,832031189103869	-0.265290485364606	1,20187802223741	0.265290485364606	1083
P14094	sp|P14094|AT1B1_MOUSE Sodium/potassium-transporting ATPase subunit beta-1 OS=Mus musculus GN=Atp1b1 PE=1 SV=1	−1,20	0,0489187958812929	0,323205843530114	34,4336965995204	34,1909777177394	34,1841736380791	34,0600793369985	34,0257535658343	34,172245247307	764020689413794	635876863736527	0,264864288877372	0,00414765953472098	0,0022476989022641	0,832277021482773	-0.264864288877372	1,2015230196052	0.264864288877372	1084
P52825	sp|P52825|CPT2_MOUSE Carnitine O-palmitoyltransferase 2, mitochondrial OS=Mus musculus GN=Cpt2 PE=1 SV=2	−1,20	0,230516483666156	0,654468548759245	27,6856496210637	28,1173618978386	28,1435044081122	27,8721796264246	27,7726589193272	27,7510871215627	1420701636430,05	1182488094404,4	0,26477794574361	0,00918900609655978	0,0023235829731332	0,83232683350444	-0.264777945743608	1,2014511240687	0.264777945743608	1085
Q91V57	sp|Q91V57|CHIN_MOUSE N-chimaerin OS=Mus musculus GN=Chn1 PE=1 SV=2	−1,20	0,279259991082464	0,699221280539289	25,5795222243761	25,5484527128798	25,8054283782832	25,7564189171455	25,2798116120726	25,346821793299	137167380408,546	114177197298,644	0,264662882793748	0,00546936388015271	0,010133527212114	0,832393218843818	-0.264662882793747	1,20135529382253	0.264662882793746	1086
O35972	sp|O35972|RM23_MOUSE 39S ribosomal protein L23, mitochondrial OS=Mus musculus GN=Mrpl23 PE=2 SV=1	−1,20	0,304514432324601	0,716532404521184	25,6870742225767	25,4898256936456	25,3086497347007	25,035270903413	25,4982180151912	25,4023621789469	118145554854,694	98364948769,1356	0,264349125644489	0,0074237208026289	0,0096538506693790	0,832574267310468	-0.264349125644494	1,20109405162182	0.264349125644494	1087
Q8BT60	sp|Q8BT60|CPNE3_MOUSE Copine-3 OS=Mus musculus GN=Cpne3 PE=2 SV=2	−1,20	0,503750303246315	0,814993646406912	25,5677487880404	26,2439255198213	26,1808314726337	26,0981905549866	25,8301463452543	25,5153070978226	195241272747,63	162598205766,766	0,263946723882634	0,0143672173248957	0,0113019459857599	0,832806524350727	-0.263946723882635	1,20075908480619	0.263946723882635	1088
P58854	sp|P58854|GCP3_MOUSE Gamma-tubulin complex component 3 OS=Mus musculus GN=Tubgcp3 PE=2 SV=2	−1,20	0,497255361195992	0,809698318764119	27,2773560240938	26,9324340242253	26,7117428695641	26,4907634434962	27,2015892625047	26,6820304486377	518312565723,705	431900490048,331	0,263123416685507	0,0105684357212191	0,0137294109484902	0,833281920235294	-0.26312341668551	1,20007403942909	0.26312341668551	1089
Q8BYK5-4	sp|Q8BYK5-4|PHAR3_MOUSE Isoform 4 of Phosphatase and actin regulator 3 OS=Mus musculus GN=Phactr3;sp|Q8BYK5-3|PHAR3_MOUSE Isoform 3 of Phosphatase and actin regulator 3 OS=Mus musculus GN=Phactr3;sp|Q8BYK5|PHAR3_MOUSE Phosphatase and actin regulator 3 O	−1,20	0,607909143896055	0,866342104911834	24,946752196482	25,0187798247048	24,5917632179099	24,0268686400196	25,1070055387849	24,8783295174502	62126082537,703	51804070562,8201	0,262133621896194	0,00919830351131755	0,0230721213795437	0,833853809008178	-0.262133621896196	1,19925098284248	0.262133621896196	1090
Q9CQF3	sp|Q9CQF3|CPSF5_MOUSE Cleavage and polyadenylation specificity factor subunit 5 OS=Mus musculus GN=Nudt21 PE=1 SV=1	−1,20	0,278440755117422	0,699221280539289	29,356105721571	29,5501594072729	29,7243921412505	29,2283500040983	29,2370422808287	29,6207138314607	6770204595958,05	5646377878926,6	0,261873749654273	0,00623594893800128	0,0076310878653082	0,834004024383193	-0.261873749654274	1,19903498156328	0.261873749654274	1091
Q9JIG8	sp|Q9JIG8|PRAF2_MOUSE PRA1 family protein 2 OS=Mus musculus GN=Praf2 PE=2 SV=1	−1,20	0,519974518674634	0,823916573667872	27,9281151032895	27,615484436771	27,55952828088	27,1551566172011	27,386252220826	28,0173481149224	1072531230830,6	894549119316,785	0,261787050551704	0,00717051866214453	0,0162172641577333	0,834054145559957	-0.261787050551705	1,19896292743516	0.261787050551705	1092
Q3UDK1-2	sp|Q3UDK1-2|TRAD1_MOUSE Isoform 2 of TRAF-type zinc finger domain-containing protein 1 OS=Mus musculus GN=Trafd1;sp|Q3UDK1|TRAD1_MOUSE TRAF-type zinc finger domain-containing protein 1 OS=Mus musculus GN=Trafd1 PE=1 SV=1	−1,20	0,47528327077448	0,79947899324683	23,0310308799512	23,4971371284695	23,43283850516	22,7318219676303	23,2726265887226	23,4130659507513	13424342556,0648	11199909136,1197	0,261364407502116	0,0108317220161619	0,0155448847854379	0,834298520716744	-0.261364407502114	1,19861173808735	0.261364407502114	1093
E9Q414	sp|E9Q414|APOB_MOUSE Apolipoprotein B-100 OS=Mus musculus GN=Apob PE=1 SV=1	−1,20	0,790924834349603	0,942055274591853	24,9689418598456	23,9703394650702	22,9717251193296	24,163506496647	24,1475198498776	23,0567184762801	25714875674,4952	21455530350,0895	0,261253615719299	0,0416601749707747	0,0266691810862083	0,834362593141749	-0.2612536157193	1,19851969421897	0.261253615719301	1094
Q99JI6	sp|Q99JI6|RAP1B_MOUSE Ras-related protein Rap-1b OS=Mus musculus GN=Rap1b PE=2 SV=2	−1,20	0,232420237236968	0,657279264635888	30,9678402142619	30,6330421420719	30,9123179140515	30,4606427564896	30,8437717553966	30,6656706138036	24697753980436,1	20607888223461,2	0,261183175294639	0,00581847105018837	0,0062538423508797	0,8344033323753	-0.261183175294642	1,19846117722624	0.261183175294642	1095
Q2TBE6	sp|Q2TBE6|P4K2A_MOUSE Phosphatidylinositol 4-kinase type 2-alpha OS=Mus musculus GN=Pi4k2a PE=1 SV=1	−1,20	0,425049575725021	0,7711031271846	25,2931187432148	25,6586432911233	25,6974368156947	25,5721737946229	24,9961132911365	25,5382714274755	124769384965,465	104124468996,295	0,260954840961509	0,00873117920259413	0,0127418723971252	0,834535403257103	-0.260954840961508	1,198271512625	0.260954840961508	1096
Q8R3F5	sp|Q8R3F5|FABD_MOUSE Malonyl-CoA-acyl carrier protein transacylase, mitochondrial OS=Mus musculus GN=Mcat PE=1 SV=3	−1,20	0,306107875978443	0,71837258919956	26,6089362359476	26,0592651454691	26,3883602649067	26,030206424081	26,2857612704694	26,1988563938474	278361708723,136	232372617350,024	0,260520696094173	0,0104968575430478	0,0049648519049255	0,834786574690653	-0.26052069609417	1,19791097547367	0.260520696094169	1097
P06728	sp|P06728|APOA4_MOUSE Apolipoprotein A-IV OS=Mus musculus GN=Apoa4 PE=1 SV=3	−1,20	0,304854291200744	0,7166363473128	24,4296234518414	24,7499941271413	24,1764319247229	24,3767709733963	24,1749746788762	24,2627398725708	41627069479,4504	34751729411,2049	0,260437222209543	0,0117551064291294	0,0041687849915469	0,834834876578579	-0.260437222209544	1,19784166672375	0.260437222209544	1098
Q9CRC8	sp|Q9CRC8|LRC40_MOUSE Leucine-rich repeat-containing protein 40 OS=Mus musculus GN=Lrrc40 PE=2 SV=2	−1,20	0,356073504184213	0,732843629012038	27,1451359480015	27,612789504722	27,6980271242862	27,4280292367288	27,3642301077782	27,122691076725	864413885911,604	721778017619,021	0,260167042416925	0,0108302414825381	0,0058985736755469	0,834991234387494	-0.260167042416924	1,19761736269429	0.260167042416924	1099
P41105	sp|P41105|RL28_MOUSE 60S ribosomal protein L28 OS=Mus musculus GN=Rpl28 PE=1 SV=2	−1,20	0,299300614900596	0,71351128730767	26,5491925988097	26,4253543694505	26,2819309123858	25,9276015798895	26,3998169118976	26,3883602649067	297543407905,749	248471234414,125	0,260021314912908	0,00506269249717094	0,0102668352664757	0,835075581620117	-0.260021314912906	1,19749639674521	0.260021314912906	1100
P21279	sp|P21279|GNAQ_MOUSE Guanine nucleotide-binding protein G(q) subunit alpha OS=Mus musculus GN=Gnaq PE=1 SV=4	−1,20	0,13940970198323	0,531391671271705	31,2024247921078	30,9542765997972	30,9997610762329	30,7005833571881	31,0273688047328	30,8893105450994	30604071024248,2	25569488531178,8	0,25930027380427	0,00425446808105678	0,0053136783009966	0,835493046363655	-0.259300273804271	1,19689805241628	0.259300273804272	1101
P63011	sp|P63011|RAB3A_MOUSE Ras-related protein Rab-3A OS=Mus musculus GN=Rab3a PE=1 SV=1	−1,20	0,41751942342571	0,768188351410335	32,7396907713201	33,1743276583604	33,2547258597017	33,1006254059203	32,5833119363487	32,9459102626137	227062879319752	189728623227289	0,25915452475963	0,00838202443087722	0,0080764701488331	0,835577456763029	-0.25915452475963	1,19677714125263	0.25915452475963	1102
Q3U7U3	sp|Q3U7U3|FBX7_MOUSE F-box only protein 7 OS=Mus musculus GN=Fbxo7 PE=2 SV=2	−1,20	0,642709869175976	0,887784569747338	26,0218395960174	26,501323798867	26,675228782357	26,6553553897964	26,4412629368967	25,5630102279109	291837815658,184	243863915883,978	0,259090535530167	0,0128182378758965	0,0220766392933575	0,835614518749018	-0.259090535530166	1,19672406063155	0.259090535530165	1103

Q99J39-2	sp|Q99J39-2|DCMC_MOUSE Isoform Cytoplasmic+peroxisomal of Malonyl-CoA decarboxylase, mitochondrial OS=Mus musculus GN=Mlycd;sp|Q99J39|DCMC_MOUSE Malonyl-CoA decarboxylase, mitochondrial OS=Mus musculus GN=Mlycd PE=1 SV=1	−1,20	0,082641237237972	0,4277178561828	27,183957488667	27,0327780523882	27,0296671524623	26,794197060117	27,081221549861	26,8329937693396	577592379569,864	482769005365,003	0,258718652780876	0,00325658400104549	0,0057885020548286	0,835829942431934	-0.258718652780877	1,19641562144854	0.258718652780877	1104
Q8R2K1	sp|Q8R2K1|FUCM_MOUSE Fucose mutarotase OS=Mus musculus GN=Fuom PE=1 SV=1;sp|Q8R2K1-5|FUCM_MOUSE Isoform 5 of Fucose mutarotase OS=Mus musculus GN=Fuom;sp|Q8R2K1-4|FUCM_MOUSE Isoform 4 of Fucose mutarotase OS=Mus musculus GN=Fuom;sp|Q8R2K1-2|FUCM_MOUSE	−1,20	0,384945189554991	0,751739350763028	25,9455567246878	25,8448215763115	26,4519728460025	26,072361667895	25,8132134394717	25,8188439996991	212197592082,857	177364546404,148	0,25869062878365	0,0124758978096636	0,0057147588303375	0,835846178381196	-0.258690628783651	1,19639238159433	0.258690628783651	1105
Q8K183	sp|Q8K183|PDXK_MOUSE Pyridoxal kinase OS=Mus musculus GN=Pdxk PE=1 SV=1	−1,20	0,243488660205603	0,667588863741216	32,5416809718635	32,0651775689469	32,1651705876441	32,1651705876441	32,077801964065	31,9922601162753	102137702454988	85403729721915,5	0,25814453049565	0,00778948755573979	0,0026951722849071	0,836162628188674	-0.258144530495657	1,19593960108722	0.258144530495658	1106
Q91WJ8	sp|Q91WJ8|FUBP1_MOUSE Far upstream element-binding protein 1 OS=Mus musculus GN=Fubp1 PE=1 SV=1;sp|Q91WJ8-2|FUBP1_MOUSE Isoform 2 of Far upstream element-binding protein 1 OS=Mus musculus GN=Fubp1	−1,20	0,367671552813171	0,739685989256097	29,8788419232647	29,6629732680634	29,21883644974	29,3820077371923	29,4883704069559	29,3536338319982	7070016151567,81	5911992269913,36	0,258069127775631	0,0113738730886301	0,0024153477938365	0,836206331523351	-0.258069127775632	1,19587709671877	0.258069127775632	1107
Q8BP92	sp|Q8BP92|RCN2_MOUSE Reticulocalbin-2 OS=Mus musculus GN=Rcn2 PE=2 SV=1	−1,20	0,156786611071727	0,561270396169617	29,0002145179698	28,9338137105631	29,1775307111958	28,8395124734223	29,0458241396507	28,6907897587117	4080278301796,22	3413327679913,01	0,257488636834359	0,00433908291913203	0,0061781674006827	0,83654285993443	-0.257488636834359	1,19539601363447	0.257488636834359	1108
Q9CPW7	sp|Q9CPW7|ZMAT2_MOUSE Zinc finger matrin-type protein 2 OS=Mus musculus GN=Zmat2 PE=2 SV=1	−1,20	0,987156476236992	0,998141007543842	0	23,709903168677	23,591698233046	0	23,3177018894452	23,4485091486631	7040575,4668305	5889825,99474204	0,257468340842238	0,866033515998278	0,866035566708123	0,836554628593946	-0.257468340842238	1,19537919679049	0.257468340842238	1109
P62806	sp|P62806|H4_MOUSE Histone H4 OS=Mus musculus GN=Hist1h4a PE=1 SV=2;sp|P62805|H4_HUMAN Histone H4 OS=Homo sapiens GN=HIST1H4A PE=1 SV=2;sp|P59259|H4_ARATH Histone H4 OS=Arabidopsis thaliana GN=At1g07660 PE=1 SV=2	−1,19	0,42797797930652	0,773941056137542	27,2868283286901	27,072984791289	26,8329937693396	26,5597469568507	26,9179630935536	27,183957488667	567365164857,045	475304987091,169	0,255424035540977	0,00838902336353209	0,0116500922365424	0,837740870486697	-0.255424035540978	1,19368653867757	0.255424035540978	1110
Q7TNR6	sp|Q7TNR6|IGS21_MOUSE Immunoglobulin superfamily member 21 OS=Mus musculus GN=Igsf21 PE=2 SV=1	−1,19	0,82457490975475	0,95563531252039	23,0785352794043	23,3607684831536	24,1698893423587	21,9237820258192	24,2779559835035	23,8772170718654	16662019780,1339	13962649293,2609	0,254990589107896	0,0240681809172025	0,0539194374929523	0,837992600987579	-0.254990589107905	1,19332795876896	0.254990589107905	1111
Q64213-2	sp|Q64213-2|SF01_MOUSE Isoform CW17E of Splicing factor 1 OS=Mus musculus GN=Sf1;sp|Q64213-3|SF01_MOUSE Isoform 3 of Splicing factor 1 OS=Mus musculus GN=Sf1;sp|Q64213|SF01_MOUSE Splicing factor 1 OS=Mus musculus GN=Sf1 PE=1 SV=6	−1,19	0,0660849878801236	0,382601734678419	28,8632336136022	28,7995948141363	28,6442862543706	28,7253092782756	28,4835717147051	28,5681116849645	3120574474895,3	2615119453844,02	0,254934795491167	0,00391485674145608	0,0042904910503585	0,83802500945976	-0.254934795491164	1,19328180986467	0.254934795491164	1112
Q08117	sp|Q08117|AES_HUMAN Amino-terminal enhancer of split OS=Homo sapiens GN=AES PE=1 SV=4;sp|P63002|AES_MOUSE Amino-terminal enhancer of split OS=Mus musculus GN=Aes PE=1 SV=1;sp|P63002-2|AES_MOUSE Isoform 2 of Amino-terminal enhancer of split OS=Mus muscul	−1,19	0,519336545871734	0,82335612354586	23,0551430075932	23,6204012521585	23,7711154655879	22,9811816841146	23,5262499539463	23,4099585585598	15783326081,4642	13230581036,4363	0,254524841458856	0,0160742133838134	0,0123177557115081	0,838263175210843	-0.254524841458854	1,19294277688922	0.254524841458854	1113
Q9CXX9	sp|Q9CXX9|CUED2_MOUSE CUE domain-containing protein 2 OS=Mus musculus GN=Cuedc2 PE=2 SV=1	−1,19	0,817788162694464	0,952267950351507	24,3206206984587	23,1854445652742	25,1410841300026	25,0588842328911	23,6385289981359	23,4209979293226	32866392385,342	27555566054,4461	0,254269342411371	0,0405534629582966	0,0370021880416047	0,83841164346153	-0.254269342411371	1,19273152728572	0.254269342411371	1114
Q61165	sp|Q61165|SL9A1_MOUSE Sodium/hydrogen exchanger 1 OS=Mus musculus GN=Slc9a1 PE=1 SV=1;sp|P19634|SL9A1_HUMAN Sodium/hydrogen exchanger 1 OS=Homo sapiens GN=SLC9A1 PE=1 SV=2	−1,19	0,333920788365226	0,72953198881961	24,9050721699232	24,6308082037023	24,5094875445525	24,6675378862814	24,2277572078905	24,6220110958197	52379928241,196	43925845958,7383	0,253944012179252	0,00821091479465279	0,0098686453932958	0,838600728058869	-0.253944012179253	1,19246259458268	0.253944012179253	1115
Q9D8N0	sp|Q9D8N0|EF1G_MOUSE Elongation factor 1-gamma OS=Mus musculus GN=Eef1g PE=1 SV=3	−1,19	0,054463895561003	0,341924788314513	32,5868757685581	32,3120570949621	32,3737547971211	32,2862250679884	32,2862250679884	32,1724065672547	120687927728902	101216769719438	0,253833034894242	0,00444712431589073	0,0020377251342348	0,838665238720466	-0.253833034894243	1,19237086960428	0.253833034894243	1116

Q9JHS4	sp|Q9JHS4|CLPX_MOUSE ATP-dependent Clp protease ATP-binding subunit clpX-like, mitochondrial OS=Mus musculus GN=Clpx PE=1 SV=2;sp|O76031|CLPX_HUMAN ATP-dependent Clp protease ATP-binding subunit clpX-like, mitochondrial OS=Homo sapiens GN=CLPX PE=1 SV=2	−1,19	0,535307681689301	0,8300431463043	25,6347612167554	26,35933783101	26,2041255291928	26,2439255198213	25,6734309040907	25,7538649352021	209099250504,364	175412664831,875	0,253434976305427	0,0146360862035967	0,0119266604825061	0,838896669446522	-0.253434976305427	1,19204192413801	0.253434976305427	1117
P62488	sp|P62488|RPB7_MOUSE DNA-directed RNA polymerase II subunit RPB7 OS=Mus musculus GN=Polr2g PE=2 SV=1	−1,19	0,385829958919233	0,75210648037178	25,0272681171071	25,0866146564925	24,5493168001552	24,4941949562678	24,7957915615014	24,846359962555	64358393091,2298	53992743423,8753	0,253362781723107	0,0118361736034326	0,0077051639292869	0,838938650120414	-0.253362781723106	1,19198227409891	0.253362781723107	1118
P61211	sp|P61211|ARL1_MOUSE ADP-ribosylation factor-like protein 1 OS=Mus musculus GN=Arl1 PE=2 SV=1	−1,19	0,23604887949346	0,658933495707757	27,2041523196231	27,210872020632	27,2596824498093	26,761141553313	27,1437352154627	27,2435367500068	666230979452,509	559031154030,553	0,253093755565991	0,0011322111749399	0,0094138006766318	0,839095105559261	-0.253093755565992	1,19176002025836	0.253093755565993	1119
P70296	sp|P70296|PEBP1_MOUSE Phosphatidylethanolamine-binding protein 1 OS=Mus musculus GN=Pebp1 PE=1 SV=3	−1,19	0,397139687567918	0,754338506152154	34,1841736380791	34,0600793369985	33,9705548759834	33,5541074777048	33,8956357977178	34,2388631383902	626771161977721	525936783475807	0,253049401342268	0,00314850379057346	0,0101007829800839	0,83912090310004	-0.253049401342269	1,19172338134542	0.253049401342269	1120
Q3TIU4	sp|Q3TIU4|PDE12_MOUSE 2,5-phosphodiesterase 12 OS=Mus musculus GN=Pde12 PE=2 SV=2	−1,19	0,308275226826109	0,71853230316343	26,5858634061522	26,9868342577459	26,7056831906351	26,3399692238663	26,7455391951909	26,6674061630996	418298146704,968	351088744124,436	0,252695861770594	0,00769162304760074	0,0080941349474400	0,839326559034612	-0.252695861770595	1,19143137940278	0.252695861770595	1121
P42208	sp|P42208|SEPT2_MOUSE Septin-2 OS=Mus musculus GN=Sept2 PE=1 SV=2;sp|Q15019|SEPT2_HUMAN Septin-2 OS=Homo sapiens GN=SEPT2 PE=1 SV=1;sp|Q15019-3|SEPT2_HUMAN Isoform 3 of Septin-2 OS=Homo sapiens GN=SEPT2;sp|Q15019-2|SEPT2_HUMAN Isoform 2 of Septin-2 OS=	−1,19	0,382330285378704	0,749797813889279	29,9407135560488	29,5056687085734	29,6000841427096	29,4671293506286	29,7856451223423	29,2687031531706	7776717649236,11	6528244642190,09	0,252466237048551	0,00770968534661944	0,0088380393070584	0,839460160011254	-0.25246623704855	1,1912417618325	0.25246623704855	1122
Q9CQZ0-2	sp|Q9CQZ0-2|ORML2_MOUSE Isoform 2 of ORM1-like protein 2 OS=Mus musculus GN=Ormdl2;sp|Q9CQZ0|ORML2_MOUSE ORM1-like protein 2 OS=Mus musculus GN=Ormdl2 PE=2 SV=1;sp|Q9CPZ6|ORML3_MOUSE ORM1-like protein 3 OS=Mus musculus GN=Ormdl3 PE=2 SV=1	−1,19	0,413329722326792	0,766530246241387	23,1651192385014	23,1051187112	22,7260651701659	22,5936782818681	22,7383944477728	23,1401154868964	9732802385,42877	8172680142,54429	0,252045990630021	0,0103512137690948	0,0124041245173267	0,839704724178909	-0.252045990630022	1,19089481243283	0.252045990630022	1123
Q8C7V8-2	sp|Q8C7V8-2|CC134_MOUSE Isoform 2 of Coiled-coil domain-containing protein 134 OS=Mus musculus GN=Ccdc134;sp|Q8C7V8|CC134_MOUSE Coiled-coil domain-containing protein 134 OS=Mus musculus GN=Ccdc134 PE=2 SV=1	−1,19	0,358615836725316	0,732893868931206	23,3777626719801	23,3845394580678	23,7458020638621	23,5057520442414	23,0384874615479	23,4404474775681	16109925311,55	13530726787,8448	0,251710471326683	0,00895891132784509	0,0108469059007467	0,839900032195925	-0.25171047132669	1,19061788506603	0.25171047132669	1124
Q8K1J6	sp|Q8K1J6|TRNT1_MOUSE CCA tRNA nucleotidyltransferase 1, mitochondrial OS=Mus musculus GN=Trnt1 PE=1 SV=1	−1,19	0,132359629333609	0,517022682913298	28,9871137767746	28,7010859141301	28,8140965858009	28,7466735660147	28,4991310077434	28,7339849515457	3330350797282,58	2798010771190,62	0,251272633026111	0,00499613979813142	0,0048639448875764	0,840154969102256	-0.251272633026109	1,19025660357463	0.251272633026109	1125
Q63932	sp|Q63932|MP2K2_MOUSE Dual specificity mitogen-activated protein kinase kinase 2 OS=Mus musculus GN=Map2k2 PE=1 SV=2	−1,19	0,307562473937691	0,71837258919956	29,2631523510523	28,8167867144637	28,759343091525	28,6735464306224	28,8842531253939	28,759343091525	3726264546345,64	3131022932282,37	0,251096027002468	0,00952765363120895	0,0036825826087153	0,840257822100413	-0.251096027002466	1,19011090845935	0.251096027002466	1126
Q8R464	sp|Q8R464|CADM4_MOUSE Cell adhesion molecule 4 OS=Mus musculus GN=Cadm4 PE=1 SV=1	−1,19	0,531665861845084	0,829883431939119	27,7318880781206	28,4869189419964	28,4187987708268	28,2731322206399	28,0488815319204	27,7938677088192	1788744805175,01	1503214834774,43	0,250896367657867	0,0148034849106925	0,0085523713660185	0,840374116210141	-0.250896367657867	1,18994621646574	0.250896367657867	1127
P99029-2	sp|P99029-2|PRDX5_MOUSE Isoform Cytoplasmic+peroxisomal of Peroxiredoxin-5, mitochondrial OS=Mus musculus GN=Prdx5;sp|P99029|PRDX5_MOUSE Peroxiredoxin-5, mitochondrial OS=Mus musculus GN=Prdx5 PE=1 SV=2	−1,19	0,21952772103702	0,648792713611515	33,6211455554601	33,450829164933	33,5634667763458	33,1282548359372	33,404240792523	33,5825229115311	370230598113675	311267207295767	0,250270539621591	0,00258229491350292	0,0068584669297048	0,840738741966962	-0.250270539621592	1,18943014052194	0.250270539621592	1128
P49817	sp|P49817|CAV1_MOUSE Caveolin-1 OS=Mus musculus GN=Cav1 PE=1 SV=1;sp|P49817-2|CAV1_MOUSE Isoform 2 of Caveolin-1 OS=Mus musculus GN=Cav1;sp|Q2QL90|CAV1_MICMU Caveolin-1 OS=Microcebus murinus GN=CAV1 PE=3 SV=1	−1,19	0,298976469281183	0,71351128730767	24,7545101478558	24,49311460887	24,4227555609276	24,1179472560925	24,5094875445525	24,5231160339711	46225315486,6185	38871102487,8337	0,249985139095314	0,00711836576958579	0,0094363345127246	0,84090507719923	-0.249985139095314	1,18919486528808	0.249985139095314	1129
Q80X95	sp|Q80X95|RRAGA_MOUSE Ras-related GTP-binding protein A OS=Mus musculus GN=Rraga PE=2 SV=1;sp|Q6NTA4|RRAGB_MOUSE Ras-related GTP-binding protein B OS=Mus musculus GN=Rragb PE=2 SV=1	−1,19	0,294208303901407	0,711969538636193	28,3294382730544	28,304589714348	28,7755965557594	28,3886961162417	28,3225587196079	28,1788730675729	2313713320149,36	1945829151518,93	0,249825075303488	0,00930998531399359	0,0037910186609060	0,840998378914687	-0.24982507530349	1,18906293409329	0.24982507530349	1130
Q9DBR0	sp|Q9DBR0|AKAP8_MOUSE A-kinase anchor protein 8 OS=Mus musculus GN=Akap8 PE=1 SV=1	−1,19	0,0839799331532849	0,430717419867526	25,3625137240185	25,1944575563232	25,0272681171071	25,0703734773173	25,0120600285972	24,9825849920557	87486161701,1898	73582483075,0562	0,249692472267842	0,00665309295397534	0,0017855207863233	0,841075681504673	-0.249692472267841	1,18895364827457	0.249692472267841	1131
P61620	sp|P61620|S61A1_MOUSE Protein transport protein Sec61 subunit alpha isoform 1 OS=Mus musculus GN=Sec61a1 PE=2 SV=2	−1,19	0,614871295840802	0,868726908818952	25,0836030464032	23,9566725543437	24,7157174558305	24,3017734131296	24,5917632179099	24,343352667187	47563477242,9953	40006046124,1722	0,24963613929318	0,0233744344862942	0,0064232817943476	0,841108523663793	-0.249636139293182	1,18890722405724	0.249636139293182	1132
P97494	sp|P97494|GSH1_MOUSE Glutamate--cysteine ligase catalytic subunit OS=Mus musculus GN=Gclc PE=2 SV=4	−1,19	0,256272285292954	0,679450877888271	28,7178629849572	28,5161702425418	28,2272204810265	28,2458362215109	28,2792329488929	28,4187987708268	2353876288173,25	1980999531358,67	0,248809960234295	0,00865694160545648	0,0032404274023962	0,841590333915146	-0.248809960234294	1,18822657497493	0.248809960234294	1133

Q9CQG1	sp|Q9CQG1|CHAC2_MOUSE Putative glutathione-specific gamma-glutamylcyclotransferase 2 OS=Mus musculus GN=Chac2 PE=2 SV=1;sp|Q9CQG1-2|CHAC2_MOUSE Isoform 2 of Putative glutathione-specific gamma-glutamylcyclotransferase 2 OS=Mus musculus GN=Chac2	−1,19	0,301910812616154	0,715599570999855	23,7401078459667	23,4404474775681	23,4209979293226	23,1374797714001	23,3553531113101	23,591698233046	16619641974,3553	13988625498,192	0,248635091075259	0,00760131101881785	0,00972419240796450	0,841692349316366	-0.248635091075263	1,18808255868336	0.248635091075263	1134
P49442	sp|P49442|INPP_MOUSE Inositol polyphosphate 1-phosphatase OS=Mus musculus GN=Inpp1 PE=1 SV=2	−1,19	0,103735523626165	0,465707963539203	29,9349818756405	29,5745822065934	29,7503945006217	29,5442070726591	29,6034944632424	29,5962766892579	8350307893783,83	7030831369868,47	0,248134101081457	0,00605705989403301	0,00109352574050311	0,841984685990129	-0.248134101081456	1,18767005699641	0.248134101081456	1135
Q8CC86	sp|Q8CC86|PNCB_MOUSE Nicotinate phosphoribosyltransferase OS=Mus musculus GN=Naprt PE=2 SV=1	−1,19	0,413454962711986	0,766530246241387	25,5630102279109	25,8313075112595	25,8804365348968	25,9468628589073	25,482818253974	25,3293144456964	153697377469,722	129420399427,456	0,248027513710612	0,00663317098658366	0,0125662482326517	0,842046894736063	-0.248027513710616	1,18758231430026	0.248027513710616	1136
Q11136	sp|Q11136|PEPD_MOUSE Xaa-Pro dipeptidase OS=Mus musculus GN=Pepd PE=2 SV=3	−1,19	0,0288671722705673	0,238644139389349	28,6297384787753	28,4367014913091	28,5057267905654	28,4613395369887	28,304589714348	28,2910319082209	2442530204190,47	2057104210094,73	0,247761521911334	0,00342921153302734	0,0033385769898347	0,842202158468914	-0.247761521911337	1,18736337819171	0.247761521911337	1137
Q9D7B6	sp|Q9D7B6|ACAD8_MOUSE Isobutyryl-CoA dehydrogenase, mitochondrial OS=Mus musculus GN=Acad8 PE=1 SV=2	−1,19	0,453518372725496	0,788322280554728	28,1431261777461	28,0224070708488	28,2518043492866	27,5436012651194	28,2731322206399	28,0865111619099	1662115879120,55	1400356854148,59	0,247226449942382	0,00407799981246987	0,0135509144765803	0,842514575391422	-0.247226449942382	1,18692308620941	0.247226449942382	1138
P63213	sp|P63213|GBG2_MOUSE Guanine nucleotide-binding protein G(I)/G(S)/G(O) subunit gamma-2 OS=Mus musculus GN=Gng2 PE=2 SV=2	−1,19	0,609654700243582	0,866608542040832	29,0836947794638	28,6029701816381	28,4381222529815	28,0072704850482	28,6821011242862	28,9217714421477	2936567225434,15	2474470831975,97	0,247010628722207	0,011683704648661З	0,0166167273338495	0,842640621520298	-0.247010628722205	1,18674554069776	0.247010628722205	1139
Q9JHU9	sp|Q9JHU9|INO1_MOUSE Inositol-3-phosphate synthase 1 OS=Mus musculus GN=Isyna1 PE=2 SV=1	−1,19	0,525848169059072	0,827322301337652	27,8740286712693	28,663140619517	28,6943108318933	28,2742677874623	28,2148956435959	28,2304457403943	2180321315340,8	1838313560053,56	0,246157894303445	0,0163620317569017	0,0010902147052175	0,843138828721774	-0.246157894303445	1,18604429772975	0.246157894303445	1140
Q99K28	sp|Q99K28|ARFG2_MOUSE ADP-ribosylation factor GTPase-activating protein 2 OS=Mus musculus GN=Arfgap2 PE=1 SV=1;sp|Q99K28-2|ARFG2_MOUSE Isoform 2 of ADP-ribosylation factor GTPase-activating protein 2 OS=Mus musculus GN=Arfgap2	−1,19	0,355895952869717	0,732843629012038	26,9807462280019	27,4982193752751	27,3712277656425	27,0900402227433	27,2953748650026	26,9530356848158	706364895196,665	595589152097,078	0,246096168659023	0,00988459580565475	0,0063550160180981	0,843174903151515	-0.246096168659024	1,18599355396173	0.246096168659024	1141
Q6PHQ8	sp|Q6PHQ8|NAA35_MOUSE N-alpha-acetyltransferase 35, NatC auxiliary subunit OS=Mus musculus GN=Naa35 PE=1 SV=1	−1,19	0,341806713725065	0,729721949431128	26,9898009594195	26,8991884585141	26,6033375672616	26,9247281789472	26,4743118091212	26,5828657246869	449218687971,661	378936786388,118	0,245460746171145	0,00753279252425823	0,0088168714789421	0,843546354002136	-0.245460746171146	1,18547130842968	0.245460746171146	1142
Q9D164-2	sp|Q9D164-2|FXYD6_MOUSE Isoform 2 of FXYD domain-containing ion transport regulator 6 OS=Mus musculus GN=Fxyd6;sp|Q9D164|FXYD6_MOUSE FXYD domain-containing ion transport regulator 6 OS=Mus musculus GN=Fxyd6 PE=1 SV=2	−1,19	0,528766780061415	0,829327026502683	28,9070949594495	28,9104405271909	28,8917477037687	28,2400659404567	28,7908543389162	29,1687376261914	3568242868312,71	3010777002838,77	0,245077957052371	0,000344872687439234	0,0162533419384251	0,843770201175363	-0.245077957052371	1,18515681000231	0.245077957052371	1143
Q6ZWX6	sp|Q6ZWX6|IF2A_MOUSE Eukaryotic translation initiation factor 2 subunit 1 OS=Mus musculus GN=Eif2s1 PE=1 SV=3	−1,18	0,385061465195867	0,751739350763028	29,9281463515691	29,5601460600447	29,4054016596301	29,2978797219439	29,6926508385983	29,3941367297357	7390609745917,19	6237220481556,63	0,244790137526421	0,00906220794838362	0,0069867278701564	0,843938551213893	-0.244790137526422	1,18492039326991	0.244790137526422	1144
Q9D0T1	sp|Q9D0T1|NH2L1_MOUSE NHP2-like protein 1 OS=Mus musculus GN=Nhp2l1 PE=1 SV=4	−1,18	0,0808015863231979	0,423283113906046	26,0637890078749	25,9154627291353	25,8337781042129	25,6663122517335	25,9023194601262	25,7355004334847	183903428881,37	155209871616,339	0,244728060721341	0,00449552109569556	0,0047082568167039	0,843974865288998	-0.24472806072134	1,18486940918267	0.24472806072134	1145
P61327	sp|P61327|MGN_MOUSE Protein mago nashi homolog OS=Mus musculus GN=Magoh PE=2 SV=1;sp|Q9CQL1|MGN2_MOUSE Protein mago nashi homolog 2 OS=Mus musculus GN=Magohb PE=2 SV=1	−1,18	0,0590510657749384	0,359775553761672	26,3309093895965	26,2799304014618	26,234878662775	25,9437232703096	26,2170897415584	26,1771723436785	259469799000,297	219071015345,443	0,244168007664458	0,00182809574311778	0,0056547332508407	0,844302559255431	-0.244168007664459	1,18440953309661	0.244168007664459	1146
Q8BLJ3	sp|Q8BLJ3|PLCX3_MOUSE PI-PLC X domain-containing protein 3 OS=Mus musculus GN=Plcxd3 PE=2 SV=1	−1,18	0,0881594986468958	0,441176105282424	28,8732918283341	29,0045580088892	29,2069428457707	28,8185754978522	28,8875389662165	28,8709870434371	4044035630134,08	3414437346165,28	0,244147847059948	0,00579035736378988	0,0012475148941178	0,844314357846564	-0.244147847059949	1,18439298195818	0.244147847059949	1147
P22907-2	sp|P22907-2|HEM3_MOUSE Isoform 2 of Porphobilinogen deaminase OS=Mus musculus GN=Hmbs;sp|P22907|HEM3_MOUSE Porphobilinogen deaminase OS=Mus musculus GN=Hmbs PE=1 SV=2	−1,18	0,240517926894779	0,664601444032504	26,1149474128652	25,7852122009926	25,8889079988918	25,8966554254401	25,8337781042129	25,5517501914311	182440367700,888	154078478010,178	0,243759625604132	0,0065025075404829	0,0071306360175891	0,844541588859273	-0.243759625604129	1,18407431107177	0.243759625604129	1148
P24369	sp|P24369|PPIB_MOUSE Peptidyl-prolyl cis-trans isomerase B OS=Mus musculus GN=Ppib PE=1 SV=2	−1,18	0,326989341876229	0,726927682919497	30,4196223089356	30,3862745237618	30,1253373252879	29,9229385752534	30,0859791046874	30,4157949618138	14576203038519,8	12311696736025,4	0,243585359856536	0,00531645048010464	0,0083302564091192	0,844643608729235	-0.243585359856531	1,18393129322851	0.243585359856531	1149
P62309	sp|P62309|RUXG_MOUSE Small nuclear ribonucleoprotein G OS=Mus musculus GN=Snrpg PE=1 SV=1	−1,18	0,549948129694353	0,838418252843802	25,7062486810014	25,5244369585765	25,3815883916897	24,8622015383108	25,484150064461	25,7605581739283	123243101547,93	104136661417,336	0,243028834635714	0,00637179946299316	0,0181371434478335	0,844969496137166	-0.243028834635713	1,18347467520611	0.243028834635714	1150
Q99L47	sp|Q99L47|F10A1_MOUSE Hsc70-interacting protein OS=Mus musculus GN=St13 PE=1 SV=1	−1,18	0,108941318698442	0,473794072647748	30,9355404205447	30,5824842469731	30,6837245297255	30,5719607650948	30,5591116316026	30,5656049997843	22262313764653,4	18812809914465,6	0,242888194083813	0,00591531979299661	0,0002101937516533	0,845051871667325	-0.242888194083814	1,18335931027164	0.242888194083814	1151

Q8K0C1	sp|Q8K0C1|IPO13_MOUSE Importin-13 OS=Mus musculus GN=Ipo13 PE=2 SV=1;sp|Q8K0C1-2|IPO13_MOUSE Isoform 2 of Importin-13 OS=Mus musculus GN=Ipo13	−1,18	0,266036885399069	0,685404159524725	25,8539662924407	25,6220992401053	25,3372129119629	25,3917468837871	25,4737767577379	25,4427882169683	131783485065,376	111367786485,299	0,24283759648644	0,010108783919459	0,0016284501291341	0,845081509493021	-0.242837596486446	1,18331780871636	0.242837596486446	1152
Q9QXW9	sp|Q9QXW9|LAT2_MOUSE Large neutral amino acids transporter small subunit 2 OS=Mus musculus GN=Slc7a8 PE=1 SV=1	−1,18	0,285780562172616	0,704836107321858	26,239003526529	25,8217373765899	25,8716928723719	25,9627882430883	25,6624905516586	25,8025240721104	191370643252,148	161741888757,648	0,242676169788268	0,00877148130721627	0,0058220318747947	0,845176073032991	-0.242676169788271	1,1831854117822	0.242676169788271	1153
Q9CR51	sp|Q9CR51|VATG1_MOUSE V-type proton ATPase subunit G 1 OS=Mus musculus GN=Atp6v1g1 PE=2 SV=3	−1,18	0,426628688406052	0,773341850117474	28,2028134144342	27,9328192440591	27,7178014437035	27,576491819139	27,6784770577248	28,0940222760243	1377297966917,42	1164132221292,89	0,242585780459676	0,00869462532774923	0,0098671876965562	0,845229027599871	-0.242585780459671	1,18311128386068	0.242585780459671	1154
Q9Z1K5	sp|Q9Z1K5|ARI1_MOUSE E3 ubiquitin-protein ligase ARIH1 OS=Mus musculus GN=Arih1 PE=1 SV=3;sp|Q9Y4X5|ARI1_HUMAN E3 ubiquitin-protein ligase ARIH1 OS=Homo sapiens GN=ARIH1 PE=1 SV=2	−1,18	0,514345900566196	0,819610722309211	26,8403763238565	27,6784770577248	27,4696095384182	27,2405304600518	27,1802077444987	27,0647658758816	739683064362,629	625510962999,382	0,241872074538348	0,0159632306055469	0,0032881102850242	0,845647268588437	-0.241872074538349	1,18252613961517	0.241872074538349	1155
P62821	sp|P62821|RAB1A_MOUSE Ras-related protein Rab-1A OS=Mus musculus GN=Rab1A PE=1 SV=3	−1,18	0,385818747609636	0,75210648037178	32,1724065672547	32,0248903492098	31,849738476019	31,5115733756014	32,0093513947963	32,0248903492098	80210734153761	67869308627098,4	0,2410360006904	0,00504537702284834	0,0091677915567063	0,846137482010767	-0.2410360006904	1,18184103796418	0.2410360006904	1156
Q8BUH1	sp|Q8BUH1|TXN4B_MOUSE Thioredoxin-like protein 4B OS=Mus musculus GN=Txnl4b PE=2 SV=2	−1,18	0,0517490847833333	0,33507716951719	23,0112645071695	22,840723799719	22,7276309950859	22,6513182672976	22,7337848548684	22,6937862021931	8470650140,2048	7168506046,41076	0,240800218509995	0,00624601461802332	0,0018172794168888	0,846275779043973	-0.240800218509994	1,18164790339349	0.240800218509994	1157
Q91WV0	sp|Q91WV0|NC2B_MOUSE Protein Dr1 OS=Mus musculus GN=Dr1 PE=2 SV=1	−1,18	0,455477466774719	0,788787570274876	25,8813493021154	25,7758152556667	25,4993244819944	25,1735426829318	25,7605581739283	25,7260541994384	147756233215,356	125225884414,212	0,238686192196168	0,00767082774308211	0,012890863292427	0,847516762502291	-0.238686192196169	1,17991766563708	0.238686192196169	1158
Q8R5M8-4	sp|Q8R5M8-4|CADM1_MOUSE Isoform 4 of Cell adhesion molecule 1 OS=Mus musculus GN=Cadm1;sp|Q8R5M8-3|CADM1_MOUSE Isoform 3 of Cell adhesion molecule 1 OS=Mus musculus GN=Cadm1;sp|Q8R5M8-2|CADM1_MOUSE Isoform 2 of Cell adhesion molecule 1 OS=Mus musculus G	−1,18	0,247092446009444	0,671718952804191	28,7995948141363	29,2155065783681	29,0627516643017	29,002551104323	28,8326097047498	28,7466735660147	4034691742959,98	3419828290760	0,238534564101221	0,00724812945198738	0,0045119193486328	0,84760584169216	-0.238534564101222	1,17979366211493	0.238534564101222	1159
Q91V41	sp|Q91V41|RAB14_MOUSE Ras-related protein Rab-14 OS=Mus musculus GN=Rab14 PE=1 SV=3	−1,18	0,043749904965599	0,307468517592271	31,7107286381385	31,7896349241163	31,687944408101	31,4485046525447	31,5364546439614	31,7073332672964	60244716416295,3	51063829313001,6	0,238532989079682	0,00168184448247039	0,0041696068098105	0,847606767042389	-0.238532989079681	1,17979237410924	0.238532989079681	1160
Q3UHH2	sp|Q3UHH2|S22AN_MOUSE Solute carrier family 22 member 23 OS=Mus musculus GN=Slc22a23 PE=2 SV=1	−1,18	0,474618469521378	0,799366691338604	23,5221721571896	23,3655480943154	23,0785352794043	22,7898514546523	23,4318412203999	23,2491539067247	13447851318,6185	11400794245,6648	0,238241344708437	0,00964704940863768	0,0142841761091998	0,847778130167192	-0.238241344708431	1,17955390026726	0.238241344708431	1161
P15105	sp|P15105|GLNA_MOUSE Glutamine synthetase OS=Mus musculus GN=Glul PE=1 SV=6;sp|Q9QY94|GLNA_ACOCA Glutamine synthetase OS=Acomys cahirinus GN=GLUL PE=2 SV=3	−1,18	0,0553592749204461	0,346587239812283	35,1702667677213	34,9927921828581	35,016095172406	35,0044436776321	34,9023917375126	34,7771307544105	168361227040800	142743481097955	0,23813506824057	0,00275086079994836	0,0032627851660142	0,8478405842419	-0.238135068240569	1,1794670113535	0.238135068240569	1162
P84104-2	sp|P84104-2|SRSF3_MOUSE Isoform Short of Serine/arginine-rich splicing factor 3 OS=Mus musculus GN=Srsf3;sp|P84104|SRSF3_MOUSE Serine/arginine-rich splicing factor 3 OS=Mus musculus GN=Srsf3 PE=1 SV=1	−1,18	0,454473323922848	0,788460969617685	28,8780890237702	28,4598031167191	28,6392790424527	28,4095407476388	28,2272204810265	28,8465312886039	2795569786524,16	2371232170387,1	0,237505433922373	0,00732205394186148	0,0111692986456326	0,848210687430323	-0.237505433922374	1,17895236975795	0.237505433922374	1163
Q8BHG2	sp|Q8BHG2|CA123_MOUSE UPF0587 protein C1orf123 homolog OS=Mus musculus PE=2 SV=1;sp|Q8BHG2-3|CA123_MOUSE Isoform 3 of UPF0587 protein C1orf123 homolog OS=Mus musculus;sp|Q8BHG2-2|CA123_MOUSE Isoform 2 of UPF0587 protein C1orf123 homolog OS=Mus musculus	−1,18	0,354174557766265	0,732843629012038	27,9951791545785	27,8568791263886	27,7219629002009	27,4129341317333	27,7031011328236	27,964154597457	1254812230753,82	1064361942655,37	0,237482665059736	0,00490385472417333	0,0099568264037573	0,848224074143716	-0.237482665059741	1,17893376347459	0.237482665059742	1164
Q6PE15-2	sp|Q6PE15-2|ABHDA_MOUSE Isoform 2 of Mycophenolic acid acyl-glucuronide esterase, mitochondrial OS=Mus musculus GN=Abhd10;sp|Q6PE15|ABHDA_MOUSE Mycophenolic acid acyl-glucuronide esterase, mitochondrial OS=Mus musculus GN=Abhd10 PE=2 SV=1	−1,18	0,230675772612006	0,654468548759245	27,8187164630971	27,5668665706527	27,9171142157975	27,7433277429496	27,4384765453677	27,6281717314036	1146306040766,68	972684235661,703	0,236948824937017	0,00650541568527549	0,0055767521677722	0,84853800038526	-0.23694882493702	1,1784976035793	0.23694882493702	1165
Q61016	sp|Q61016|GBG7_MOUSE Guanine nucleotide-binding protein G(I)/G(S)/G(O) subunit gamma-7 OS=Mus musculus GN=Gng7 PE=2 SV=2	−1,18	0,607764264156377	0,866342104911834	26,4159750442456	25,7471346094699	25,5614805926799	25,3797969498621	25,8539662924407	25,9984600276786	178561112907,082	151533782422,793	0,236778465056656	0,0173473901523879	0,0125714557374611	0,848638205462164	-0.236778465056659	1,17835844952963	0.236778465056659	1166

Q6PDI6	sp|Q6PDI6|FA63B_MOUSE Protein FAM63B OS=Mus musculus GN=Fam63b PE=2 SV=1;sp|Q6PDI6-3|FA63B_MOUSE Isoform 3 of Protein FAM63B OS=Mus musculus GN=Fam63b;sp|Q6PDI6-2|FA63B_MOUSE Isoform 2 of Protein FAM63B OS=Mus musculus GN=Fam63b	−1,18	0,185341173302524	0,609238393171755	25,5400198303093	25,5637107751137	25,2582028351988	25,4287308522879	25,272388648783	25,1686290907268	113376254859,46	96221262804,2035	0,236690880199744	0,00667712523452187	0,0051773099363692	0,848689727169753	-0.236690880199743	1,17828691450625	0.236690880199743	1167
Q9CQE3	sp|Q9CQE3|RT17_MOUSE 28S ribosomal protein S17, mitochondrial OS=Mus musculus GN=Mrps17 PE=2 SV=1	−1,18	0,417181425668984	0,767877733621866	24,803332574449	25,4699135689093	25,0849820401459	24,8499747977413	24,9689418598456	25,048560475253	81137362974,7323	68893364972,5588	0,23600136903481	0,0133212291772671	0,0040046940410921	0,849095440703606	-0.23600136903481	1,17772390718686	0.23600136903481	1168
A2BDX3	sp|A2BDX3|MOCS3_MOUSE Adenylyltransferase and sulfurtransferase MOCS3 OS=Mus musculus GN=Mocs3 PE=2 SV=1	−1,18	0,39011311349487	0,753319093070654	26,5089478029815	26,5771116775476	26,870933519598	26,6381041414573	26,1808314726337	26,6482374292033	375803035016,881	319191694069,533	0,235553607550432	0,00721733169931709	0,0100788714582122	0,849359010778598	-0.235553607550434	1,17735844008214	0.235553607550434	1169
G5E829	sp|G5E829|AT2B1_MOUSE Plasma membrane calcium-transporting ATPase 1 OS=Mus musculus GN=Atp2b1 PE=1 SV=1	−1,18	0,239686559985521	0,664270737934997	28,0865111619099	27,8355439715928	27,615484436771	27,6333084505179	27,6856496210637	27,7288300481742	1239645445268,86	1052928072909,31	0,235520662976792	0,00846382274721527	0,0017279432216881	0,849378406485369	-0.235520662976789	1,17733155489305	0.235520662976789	1170
Q8BGH4	sp|Q8BGH4|REEP1_MOUSE Receptor expression-enhancing protein 1 OS=Mus musculus GN=Reep1 PE=1 SV=1	−1,18	0,398047428753606	0,754338506152154	26,262865549841	26,5398052352229	26,3529925413592	25,8727859475999	26,3744753513748	26,4187355803847	287710645472,045	244382133923,673	0,235479784956138	0,00535355611767432	0,0115641810795473	0,849402473525847	-0.235479784956136	1,17729819628265	0.235479784956137	1171
Q9R0L7	sp|Q9R0L7|AKP8L_MOUSE A-kinase anchor protein 8-like OS=Mus musculus GN=Akap8l PE=1 SV=1	−1,18	0,836039900703922	0,958279068003814	24,4516913640593	24,0578211291045	22,2942056201138	24,1143670029103	23,5485803366946	22,6513182672976	17778209716,3322	15101932242,1069	0,235376901232719	0,0486808482747499	0,0314768079959232	0,849463049602419	-0.235376901232718	1,17721424194736	0.235376901232718	1172
Q8BFR4	sp|Q8BFR4|GNS_MOUSE N-acetylglucosamine-6-sulfatase OS=Mus musculus GN=Gns PE=2 SV=1	−1,18	0,442793946969798	0,782343995833365	24,5032761075892	24,7280852384171	24,801127570362	24,361859950797	24,8971877540398	24,2840779865289	52155543164,9082	44305525283,6176	0,235333965968201	0,00629060100425586	0,0136164478246177	0,849488330387626	-0.235333965968201	1,1771792080342	0.235333965968201	1173
O88543	sp|O88543|CSN3_MOUSE COP9 signalosome complex subunit 3 OS=Mus musculus GN=Cops3 PE=1 SV=3	−1,18	0,317530352374465	0,722907157640623	30,4157949618138	29,9997657346318	30,076831063814	29,8687792032057	30,1999089850511	29,9349818756405	12592601649424,5	10699555929640,8	0,235025455905515	0,00733744921026781	0,0058404888870411	0,849670006843247	-0.23502545590551	1,17692750355549	0.23502545590551	1174
Q9Z2I8	sp|Q9Z2I8|SUCB2_MOUSE Succinyl-CoA ligase [GDP-forming] subunit beta, mitochondrial OS=Mus musculus GN=Suclg2 PE=1 SV=3;sp|Q9Z2I8-2|SUCB2_MOUSE Isoform 2 of Succinyl-CoA ligase [GDP-forming] subunit beta, mitochondrial OS=Mus musculus GN=Suclg2	−1,18	0,283876770417428	0,704836107321858	29,6067416513827	29,1775307111958	29,3897530370284	29,0533026951177	29,3206469921288	29,3120180582318	5814301648631,87	4941331351443,88	0,234706119093014	0,00730180616525455	0,0051976943414989	0,849858099915851	-0.234706119093015	1,17666702252884	0.234706119093016	1175
Q6A068	sp|Q6A068|CDC5L_MOUSE Cell division cycle 5-like protein OS=Mus musculus GN=Cdc5l PE=1 SV=2;sp|O08837|CDC5L_RAT Cell division cycle 5-like protein OS=Rattus norvegicus GN=Cdc5l PE=1 SV=2	−1,18	0,389838091040519	0,753319093070654	27,6503089516748	27,3623338424334	27,0764572082296	27,2082466545883	27,3540715475072	27,039238096365	764917126944,014	650182393691,881	0,234458961266811	0,0104859175405726	0,0057924971582744	0,850003707315955	-0.234458961266812	1,17646545702452	0.234458961266812	1176
P26043	sp|P26043|RADI_MOUSE Radixin OS=Mus musculus GN=Rdx PE=1 SV=3	−1,18	0,288800785869541	0,708193823372149	28,7807657090108	28,5757099628488	28,3225587196079	28,5437339219033	28,2841998398392	28,3637482883521	2531107653306,39	2151588128980,01	0,234366935354773	0,00803663828717982	0,0046823801855572	0,850057928657988	-0.234366935354772	1,17639041562583	0.234366935354772	1177
P03930	sp|P03930|ATP8_MOUSE ATP synthase protein 8 OS=Mus musculus GN=Mtatp8 PE=1 SV=1	−1,18	0,292465602367368	0,711969538636193	26,2939048107703	26,1288765661872	25,8188439996991	25,7958217310218	25,9353037091857	26,0234593655428	212146262718,775	180355354843,073	0,234217005452751	0,00924791178677072	0,0044285167684074	0,850146274234184	-0.23421700545275	1,17626816738191	0.23421700545275	1178
Q9D1X9	sp|Q9D1X9|TM50B_MOUSE Transmembrane protein 50B OS=Mus musculus GN=Tmem50b PE=2 SV=1	−1,18	0,420763496839715	0,769812444918636	24,8730804771037	24,7147344118964	24,3700164161251	24,2617206040686	24,4760775927859	24,7331922094988	50873624070,2762	43252900664,0003	0,234120983453378	0,0104338446356998	0,0096388541932273	0,850202859624297	-0.234120983453377	1,17618988066201	0.234120983453377	1179
Q9D7G0	sp|Q9D7G0|PRPS1_MOUSE Ribose-phosphate pyrophosphokinase 1 OS=Mus musculus GN=Prps1 PE=1 SV=4	−1,18	0,195095588884724	0,622304200947129	31,2604931538048	30,9222771011418	30,9261836153758	30,7747464720785	30,9678402142619	30,8796473041202	30123236203808,7	25611894715305,6	0,234062785659563	0,00625561451620492	0,0031310172440119	0,850237157190545	-0.234062785659563	1,17614243454652	0.234062785659563	1180
P45377	sp|P45377|ALD2_MOUSE Aldose reductase-related protein 2 OS=Mus musculus GN=Akr1b8 PE=1 SV=2	−1,18	0,386655382164028	0,752468171504595	26,4451428857265	26,6724274176718	26,8101342985368	26,7885005791647	26,3143352941035	26,3382310811099	372151996550,174	316426128954,03	0,234023240280105	0,00691820107232985	0,0100878050922774	0,850260463163655	-0.234023240280105	1,17611019602063	0.234023240280105	1181
Q3THE2	sp|Q3THE2|ML12B_MOUSE Myosin regulatory light chain 12B OS=Mus musculus GN=Myl12b PE=1 SV=2	−1,18	0,489544011399523	0,806156092714947	30,0707031025332	29,8638697170678	29,6629732680634	29,3290713947903	29,7550162373898	30,0268702489686	9344870470338,99	7945704841209,64	0,233999464165046	0,00682626243572678	0,011841088460565	0,850274475866695	-0.233999464165047	1,17609081347607	0.233999464165047	1182
Q6NSR8	sp|Q6NSR8|PEPL1_MOUSE Probable aminopeptidase NPEPL1 OS=Mus musculus GN=Npepl1 PE=2 SV=1	−1,18	0,482596719920009	0,802890545076804	23,508629348876	23,3379615440826	22,9406662221807	22,8859967988134	23,4079437247829	23,0076169888695	12668938388,4393	10775266123,0047	0,23357213604629	0,012527495653172	0,0118219796065603	0,850526365558568	-0.23357213604629	1,1757425054581	0.23357213604629	1183
Q9JJV2	sp|Q9JJV2|PROF2_MOUSE Profilin-2 OS=Mus musculus GN=Pfn2 PE=1 SV=3;sp|Q9JJV2-3|PROF2_MOUSE Isoform 3 of Profilin-2 OS=Mus musculus GN=Pfn2;sp|Q9JJV2-2|PROF2_MOUSE Isoform 2 of Profilin-2 OS=Mus musculus GN=Pfn2	−1,18	0,356365335584613	0,73284362901 2038	32,5208763535181	32,3661802272999	32,1886810722787	31,9282291432254	32,2317495018469	32,4315691732345	113019243623941	96174234597707,6	0,23284609116677	0,00513706205070935	0,0078716258115834	0,850954505745205	-0.232846091166774	1,17515095489655	0.232846091166774	1184
Q8BMJ3	sp|Q8BMJ3|IF1AX_MOUSE Eukaryotic translation initiation factor 1A, X-chromosomal OS=Mus musculus GN=Eif1ax PE=2 SV=3	−1,17	0,64024688947833	0,88600019248236	26,3183357187085	26,5816409484069	26,4634183601821	25,6236903849764	26,5695977401837	26,6864288116258	308338798068,535	262427050875,229	0,232599994189271	0,00498519824198198	0,0221646768902957	0,851099675159591	-0.232599994189272	1,1749505130671	0.232599994189272	1185
Q8VCD5	sp|Q8VCD5|MED17_MOUSE Mediator of RNA polymerase II transcription subunit 17 OS=Mus musculus GN=Med17 PE=1 SV=1	−1,17	0,494519393301745	0,808741313308728	22,4077897022244	22,0428427182337	22,1382342375959	21,736986923389	21,9306752260833	22,4379044725108	4362402985,81014	3713307678,80752	0,232418188433594	0,00852724383192204	0,0164263678729672	0,85120693592179 1	-0.232418188433594	1,17480245730972	0.232418188433594	1186

Q9JII5-2	sp|Q9JII5-2|DAZP1_MOUSE Isoform 2 of DAZ-associated protein 1 OS=Mus musculus GN=Dazap1;sp|Q9JII5|DAZP1_MOUSE DAZ-associated protein 1 OS=Mus musculus GN=Dazap1 PE=2 SV=2	−1,17	0,353093973719277	0,732843629012038	28,4187987708268	28,0649683586343	28,198658713088	28,2538132214311	28,1512065915307	27,7954933381178	1815669308804,47	1546225192741,42	0,231751016708186	0,00632953135646488	0,0085697653344250	0,851600666070373	-0.231751016708184	1,17425929762878	0.231751016708184	1187
P53986	sp|P53986|MOT1_MOUSE Monocarboxylate transporter 1 OS=Mus musculus GN=Slc16a1 PE=1 SV=1	−1,17	0,694020630755622	0,910309912045398	23,3681777993606	22,8966186104492	24,1331519049364	22,9337468186366	23,4148105713821	23,5680656793277	15529119434,7532	13227198289,0985	0,231468514864343	0,0265934598430393	0,0142015926266824	0,851767438886255	-0.231468514864341	1,17402938213695	0.231468514864341	1188
Q99P72	sp|Q99P72|RTN4_MOUSE Reticulon-4 OS=Mus musculus GN=Rtn4 PE=1 SV=2;sp|Q99P72-3|RTN4_MOUSE Isoform 2 of Reticulon-4 OS=Mus musculus GN=Rtn4	−1,17	0,268667491862779	0,687806963093714	30,2654579925308	30,3135389013481	30,148766825788	30,0134696913558	29,8880330606952	30,3453333642794	13620374417662,4	11602929270062,4	0,23127728945341	0,00280178884811089	0,0078547159056039	0,851880345889475	-0.231277289453407	1,173873777961	0.231277289453407	1189
Q5KU39	sp|Q5KU39|VPS41_MOUSE Vacuolar protein sorting-associated protein 41 homolog OS=Mus musculus GN=Vps41 PE=2 SV=1	−1,17	0,325855945774228	0,726927682919497	27,0832508388608	26,5858634061522	26,8202978882426	26,8109304958059	26,5451722092463	26,6526229894318	448782431288,598	382339667153,054	0,231161313812791	0,00927438534886636	0,0050127419300834	0,851948829759744	-0.231161313812792	1,17377941616753	0.231161313812792	1190
O35381	sp|O35381|AN32A_MOUSE Acidic leucine-rich nuclear phosphoprotein 32 family member A OS=Mus musculus GN=Anp32a PE=1 SV=1	−1,17	0,260299928172617	0,682439759823898	31,1184365708611	31,4372046772841	31,3310729947621	31,2898972692622	31,1864065676514	30,9306955015522	39038576503757,6	33269642023258,8	0,23069410455942	0,0051867517094788	0,0059384739304837	0,85222477361736	-0.23069410455942	1,17339935537827	0.23069410455942	1191
P47740	sp|P47740|AL3A2_MOUSE Fatty aldehyde dehydrogenase OS=Mus musculus GN=Aldh3a2 PE=2 SV=2	−1,17	0,121018009524418	0,496600715486755	28,4428094286615	28,198658713088	28,3294382730544	28,0890345798713	28,3139767888204	28,0890345798713	1998934634077,61	1704026875447,7	0,230283206641242	0,00431366770635383	0,0046112190833177	0,852467532653466	-0.230283206641243	1,17306520388795	0.230283206641243	1192
Q9WUT3	sp|Q9WUT3|KS6A2_MOUSE Ribosomal protein S6 kinase alpha-2 OS=Mus musculus GN=Rps6ka2 PE=1 SV=1	−1,17	0,272894800017551	0,6939324914732	27,7129019603996	27,738528557757	27,3902087291243	27,5687786150749	27,5237275215483	27,270293282656	983004710547,377	837985365014,723	0,230273281746085	0,00703009194648638	0,0058610167952376	0,852473397149947	-0.230273281746084	1,17305713391559	0.230273281746084	1193
Q8BI72	sp|Q8BI72|CARF_MOUSE CDKN2A-interacting protein OS=Mus musculus GN=Cdkn2aip PE=2 SV=1	−1,17	0,435431406725495	0,778119352412213	25,789671987075	26,3643567050085	26,1456344965372	26,0995196682378	25,7112604959504	26,0103345112904	216290387762,598	184399706882,42	0,230133188911572	0,0111134843616104	0,0078397183411565	0,852556180558604	-0.230133188911576	1,17294322978785	0.230133188911576	1194
Q9Y333	sp|Q9Y333|LSM2_HUMAN U6 snRNA-associated Sm-like protein LSm2 OS=Homo sapiens GN=LSM2 PE=1 SV=1;sp|O35900|LSM2_MOUSE U6 snRNA-associated Sm-like protein LSm2 OS=Mus musculus GN=Lsm2 PE=3 SV=1	−1,17	0,494558298664565	0,808741313308728	26,8359983838767	26,8583139812733	26,4743118091212	26,1876191399344	26,6455238891739	26,8570170371907	403271038426,044	343820889534,52	0,230092598604936	0,00806615239512754	0,0128814273444897	0,852580167612442	-0.230092598604934	1,17291022942792	0.230092598604934	1195
Q80W21	sp|Q80W21|GSTM7_MOUSE Glutathione S-transferase Mu 7 OS=Mus musculus GN=Gstm7 PE=1 SV=1	−1,17	0,336731938162801	0,7295967392874	28,2885021532674	27,8772178137753	28,4137586698619	27,9865800133104	28,102857773251	28,0120626766497	1754420196040,62	1496026168644,15	0,229858913613627	0,00995594885774276	0,0021802084329046	0,852718278107141	-0.229858913613626	1,17272025905179	0.229858913613626	1196
Q62095	sp|Q62095|DDX3Y_MOUSE ATP-dependent RNA helicase DDX3Y OS=Mus musculus GN=Ddx3y PE=1 SV=2	−1,17	0,394773833396587	0,753881747531964	30,2825025623271	29,9739816452622	29,6715275253764	29,9252115172164	29,709391036643	29,8158570876731	10433093225262,2	8897752925532,03	0,229654011358972	0,0101912362789543	0,0036192143224651	0,852839396085087	-0.229654011358975	1,17255371244627	0.229654011358975	1197
Q8VCN9	sp|Q8VCN9|TBCC_MOUSE Tubulin-specific chaperone C OS=Mus musculus GN=Tbcc PE=2 SV=1	−1,17	0,360888317865026	0,734891016056238	27,8477202619897	27,2944083728934	27,6244221741423	27,4433741140667	27,3070559481143	27,5386383573167	958705975256,136	817641221790,319	0,229620491827829	0,0100897942912035	0,0042434353136325	0,852859211158949	-0.229620491827828	1,17252646968672	0.229620491827828	1198
Q501J6	sp|Q501J6|DDX17_MOUSE Probable ATP-dependent RNA helicase DDX17 OS=Mus musculus GN=Ddx17 PE=1 SV=1;sp|Q501J6-2|DDX17_MOUSE Isoform 2 of Probable ATP-dependent RNA helicase DDX17 OS=Mus musculus GN=Ddx17	−1,17	0,0408826560668529	0,2983382248943	29,1279023668535	29,1550985480598	28,9121400873384	28,8941100043926	28,8948809578225	28,9293487073306	4195556062718,37	3579031049639,88	0,229292972728101	0,00457998989821576	0,0006962614183752	0,85305284833233	-0.229292972728102	1,17226031418211	0.229292972728102	1199
Q05BC3-2	sp|Q05BC3-2|EMAL1_MOUSE Isoform 2 of Echinoderm microtubule-associated protein-like 1 OS=Mus musculus GN=Eml1;sp|Q05BC3|EMAL1_MOUSE Echinoderm microtubule-associated protein-like 1 OS=Mus musculus GN=Eml1 PE=1 SV=1;sp|Q05BC3-3|EMAL1_MOUSE Isoform 3 of E	−1,17	0,0944182442900113	0,450242157645468	27,8961549127051	27,6999254581679	27,6464734713961	27,6606396741905	27,6294369835375	27,4766700566273	1123553950474,67	958768582503,722	0,228814861286969	0,00473803051589244	0,0035685157152514	0,853335598258249	-0.228814861286965	1,17187188960722	0.228814861286965	1200
Q91WK5	sp|Q91WK5|GCSH_MOUSE Glycine cleavage system H protein, mitochondrial OS=Mus musculus GN=Gcsh PE=1 SV=2	−1,17	0,354541400129411	0,732843629012038	26,0003863356302	25,8662283570416	25,6475927807525	25,4159343606792	25,7487841328276	25,8742929382005	166468036973,329	142082040431,997	0,22852099094508	0,00689194310481345	0,0092238860209987	0,853509436497776	-0.228520990945086	1,17163320900507	0.228520990945086	1201
P97351	sp|P97351|RS3A_MOUSE 40S ribosomal protein S3a OS=Mus musculus GN=Rps3a PE=1 SV=3	−1,17	0,467764589737383	0,796164546189264	29,0513152204935	29,5874435150517	29,709391036643	29,4242425054228	29,142784813033	29,3062942508444	6161758092596,23	5259763553000,36	0,228344097860251	0,0118877780984947	0,0048254275048562	0,85361409421774	-0.228344097860251	1,17148956041595	0.228344097860251	1202

Q9Z0H3-2	sp|Q9Z0H3-2|SNF5_MOUSE Isoform B of SWI/SNF-related matrix-associated actin-dependent regulator of chromatin subfamily B member 1 OS=Mus musculus GN=Smarcb1;sp|Q12824-2|SNF5_HUMAN Isoform B of SWI/SNF-related matrix-associated actin-dependent regulator o	−1,17	0,283385168623692	0,704458182186602	26,882581127589	26,8999307211861	26,9993754734062	26,9378482697719	26,485190850542	26,8845106514255	494738795341,246	422385084435,688	0,228108143910021	0,00234047747330743	0,0092414593425201	0,853753714916066	-0.228108143910022	1,17129797801034	0.228108143910022	1203
P56375	sp|P56375|ACYP2_MOUSE Acylphosphatase-2 OS=Mus musculus GN=Acyp2 PE=2 SV=2	−1,17	0,578478563878327	0,852070158615807	28,1788730675729	27,9562316171464	27,8588173640719	27,311293764426	28,0336619814108	28,1757537010239	1443329937157,99	1232710454305,59	0,227567158030414	0,00585971521602376	0,0166505245799595	0,854073917937902	-0.227567158030409	1,17085884371042	0.227567158030409	1204
Q9R1P1	sp|Q9R1P1|PSB3_MOUSE Proteasome subunit beta type-3 OS=Mus musculus GN=Psmb3 PE=1 SV=1	−1,17	0,522254357112912	0,823948365557954	30,5898070211484	30,5524835884731	30,5053526695314	29,9143750348548	30,5331676346361	30,727262227369	18507819351900,7	15809017678329,8	0,227387196425387	0,00138537046592883	0,0139687149600959	0,854180461660183	-0.227387196425384	1,1707128000287	0.227387196425384	1205
Q8BTZ5	sp|Q8BTZ5|ANR46_MOUSE Ankyrin repeat domain-containing protein 46 OS=Mus musculus GN=Ankrd46 PE=2 SV=1;sp|Q76K24|ANR46_RAT Ankyrin repeat domain-containing protein 46 OS=Rattus norvegicus GN=Ankrd46 PE=2 SV=1	−1,17	0,583838997643177	0,85430213098078	22,3570874543215	21,9746816952802	22,1323356543618	21,4673136439321	22,2860368944435	22,2381882530085	4184703128,44367	3574816192,65278	0,227256214280385	0,00867452311176709	0,0208890697802957	0,854258016143259	-0.227256214280386	1,17060651595021	0.227256214280386	1206
P62631	sp|P62631|EF1A2_MOUSE Elongation factor 1-alpha 2 OS=Mus musculus GN=Eef1a2 PE=1 SV=1;sp|Q05639|EF1A2_HUMAN Elongation factor 1-alpha 2 OS=Homo sapiens GN=EEF1A2 PE=1 SV=1	−1,17	0,200475162983424	0,6308537814738	34,7873562853203	34,4663738267566	34,5341448386175	34,4014332388837	34,5822675043587	34,3323405522909	1058848420684030	904750591229001	0,226904024808491	0,00489027269057459	0,0037475192969268	0,854466582331511	-0.226904024808483	1,17032078337269	0.226904024808483	1207
P60335	sp|P60335|PCBP1_MOUSE Poly(rC)-binding protein 1 OS=Mus musculus GN=Pcbp1 PE=1 SV=1	−1,17	0,0782135385275774	0,417369905631425	32,099937215965	31,8110328188897	31,8850101738384	31,736695594508	31,8094472578625	31,7786943855435	73771485895686,6	63049783589559,5	0,226571875830935	0,00469977968241085	0,0011493461042043	0,854663327219847	-0.226571875830936	1,17005137362442	0.226571875830936	1208
Q8K2Q5	sp|Q8K2Q5|CHCH7_MOUSE Coiled-coil-helix-coiled-coil-helix domain-containing protein 7 OS=Mus musculus GN=Chchd7 PE=3 SV=1	−1,17	0,454582341049445	0,788460969617685	23,5718044898966	23,4691216538316	23,1903792338134	23,1333909033916	23,02646262069	23,6005714162421	14690033768,162	12556131891,1989	0,226445623875299	0,00843079843931643	0,0131297329461761	0,85473812309486	-0.226445623875306	1,16994898551988	0.226445623875306	1209
Q8K012-2	sp|Q8K012-2|FBP1L_MOUSE Isoform 2 of Formin-binding protein 1-like OS=Mus musculus GN=Fnbp1l;sp|Q8K012|FBP1L_MOUSE Formin-binding protein 1-like OS=Mus musculus GN=Fnbp1l PE=1 SV=2	−1,17	0,336581880904201	0,7295967392874	25,5883688052889	25,2191352268015	25,4918299528731	25,0688342117586	25,2783415939017	25,482818253974	111035002714,643	94954596862,9623	0,225704794254516	0,00752924829413734	0,008189257484893	0,855177147219	-0.225704794254516	1,16934836630277	0.225704794254516	1210
P17427	sp|P17427|AP2A2_MOUSE AP-2 complex subunit alpha-2 OS=Mus musculus GN=Ap2a2 PE=1 SV=2	−1,17	0,369389263306089	0,740573262432129	33,9705548759834	33,5825229115311	33,404240792523	33,5109505079196	33,5728260749869	33,404240792523	412162797452625	352476754670686	0,225686173523734	0,00860438544110077	0,002545989091401	0,855188184982175	-0.225686173523734	1,16933327372951	0.225686173523734	1211
Q9DAZ9	sp|Q9DAZ9|ANCHR_MOUSE Abscission/NoCut checkpoint regulator OS=Mus musculus GN=Zfyve19 PE=1 SV=2	−1,17	0,582769752773102	0,853640676481215	24,0899649888494	23,1210148714882	23,7401078459667	23,5370595761934	23,5022385635319	23,4427204772675	18673338013,951	15970453658,1038	0,225574549661291	0,0207468146602609	0,00203053045589081	0,855254354961718	-0.225574549661298	1,16924280384958	0.225574549661298	1212
Q8HW98	sp|Q8HW98|IGLO5_MOUSE IgLON family member 5 OS=Mus musculus GN=Iglon5 PE=2 SV=2	−1,17	0,489661235429634	0,806156092714947	24,3378985272248	24,0336069781538	23,5718044898966	23,9042039329199	23,7805294570105	23,7897040107311	25993265748,8219	22232309860,6716	0,225480055685977	0,0160850105479698	0,00289227401917811	0,855310374444936	-0.22548005568598	1,16916622302046	0.22548005568598	1213
Q9JKV1	sp|Q9JKV1|ADRM1_MOUSE Proteasomal ubiquitin receptor ADRM1 OS=Mus musculus GN=Adrm1 PE=1 SV=2	−1,17	0,171870035338832	0,586114267770819	28,0072704850482	27,6683860242425	27,7259995969464	27,7105926216416	27,6659486438165	27,5571437909233	1184748978293,66	1013632658218,53	0,225046504302144	0,00652239845902787	0,0028551137727547	0,855567446598197	-0.225046504302135	1,1688149239 1521	0.225046504302135	1214
D3Z4S3	sp|D3Z4S3|PTRD1_MOUSE Putative peptidyl-tRNA hydrolase PTRHD1 OS=Mus musculus GN=Ptrhd1 PE=3 SV=1	−1,17	0,55200571084002	0,838797478215494	25,9719342937484	25,5170328077361	25,2432823524486	25,1160031518505	25,5599556845734	25,5890196137344	128271697072,863	109770700196,575	0,224709853299021	0,014390032379851 7	0,0104283018730952	0,855767115439513	-0.224709853299014	1,16854221429905	0.224709853299014	1215
Q3TXX4	sp|Q3TXX4|VGLU1_MOUSE Vesicular glutamate transporter 1 OS=Mus musculus GN=Slc17a7 PE=1 SV=2	−1,17	0,264547810667671	0,68394025335819	30,3198830461946	29,8531115187151	30,0816756695027	29,9997657346318	29,8955901225831	29,8931980979023	11633268438926,2	9959201215272,27	0,224154548205595	0,00775812590613576	0,0020330461421690	0,856096570586103	-0.224154548205589	1,1680925194168	0.224154548205589	1216
Q91X97	sp|Q91X97|NCALD_MOUSE Neurocalcin-delta OS=Mus musculus GN=Ncald PE=1 SV=4	−1,17	0,605389114411623	0,865294279541889	30,9837659274804	30,8405947262776	30,7106727634419	30,111798004507	30,9355404205447	31,0218212612287	24878152859616,3	21299823355207,3	0,224037907092668	0,00442859744698015	0,0163687011504409	0,856165788328379	-0.22403790709267	1,16799808358665	0.22403790709267	1217
Q61214	sp|Q61214|DYR1A_MOUSE Dual specificity tyrosine-phosphorylation-regulated kinase 1A OS=Mus musculus GN=Dyrk1a PE=1 SV=1	−1,17	0,450106470140225	0,786343703431612	24,7729139695752	25,0023493395826	25,0817452967474	24,8549736207267	25,0817452967474	24,4554828608451	68653379915,472	58799582575,7019	0,223524835043194	0,00642725913926672	0,0127866602711463	0,856470324521497	-0.223524835043194	1,16758277709002	0.223524835043194	1218
Q8BXA0	sp|Q8BXA0|LRFN5_MOUSE Leucine-rich repeat and fibronectin type-III domain-containing protein 5 OS=Mus musculus GN=Lrfn5 PE=1 SV=1;sp|Q8BXA0-2|LRFN5_MOUSE Isoform 2 of Leucine-rich repeat and fibronectin type-III domain-containing protein 5 OS=Mus musculu	−1,17	0,608913979374412	0,866608542040832	23,088004659985	23,4808724835156	23,114783803093	22,8598253519744	23,6185544152518	22,7406104302362	12238921313,9016	10482748390,794	0,22345939513925	0,00944987525733355	0,0206393681042327	0,856509174455363	-0.223459395139249	1,16752981733778	0.223459395139249	1219
Q8K2C9	sp|Q8K2C9|HACD3_MOUSE Very-long-chain (3R)-3-hydroxyacyl-CoA dehydratase 3 OS=Mus musculus GN=ptplad1 PE=1 SV=2	−1,17	0,34389981853522	0,730103496880899	26,7117428695641	26,3327598269784	26,5000720757695	26,6015089080294	26,2614070104605	26,2170897415584	327534181457,267	280545535794,69	0,22341051813769	0,00716291572852582	0,0079788212893541	0,856538192583397	-0.223410518137688	1,1674902633167	0.223410518137688	1220

Q6P069-2	sp|Q6P069-2|SORCN_MOUSE Isoform 2 of Sorcin OS=Mus musculus GN=Sri;sp|Q6P069|SORCN_MOUSE Sorcin OS=Mus musculus GN=Sri PE=1 SV=1	−1,17	0,431481914007237	0,775108167118334	29,5484224910215	29,3648762637523	29,1488133739494	28,8830163528639	29,323260678621	29,3914357617658	5601398284942,47	4798083089624,83	0,223328872759425	0,00681422210142844	0,0094512385740189	0,856586667390339	-0.223328872759426	1,16742419426931	0.223328872759426	1221
Q8VCT3	sp|Q8VCT3|AMPB_MOUSE Aminopeptidase B OS=Mus musculus GN=Rnpep PE=2 SV=2	−1,17	0,444149982574756	0,783519926508673	30,0689715847678	30,6509513772108	30,5824842469731	30,447826850692	30,1964189422983	30,1944393020597	16495945449700	14133397058580,7	0,223003198025463	0,0104517162188042	0,0048126607512832	0,856780055540117	-0.223003198025463	1,16716068906342	0.223003198025463	1222
Q9D8E6	sp|Q9D8E6|RL4_MOUSE 60S ribosomal protein L4 OS=Mus musculus GN=Rpl4 PE=1 SV=3	−1,17	0,252407091969586	0,67459732861346	27,9628273575294	27,5376339052084	27,9035914445976	27,6931390764562	27,6281717314036	27,6192585353685	1185695813341,84	1015961375114,69	0,222888383643933	0,00828370173900811	0,0014587166391879	0,856848243607473	-0.222888383643927	1,16706780630119	0.222888383643927	1223
P50285	sp|P50285|FMO1_MOUSE Dimethylaniline monooxygenase [N-oxide-forming] 1 OS=Mus musculus GN=Fmo1 PE=1 SV=1	−1,17	0,451755885822095	0,786543907075754	24,9376594065974	25,1199552746439	25,4471000951404	25,2961103046975	24,875004184539	24,8701728545749	85197528214,4965	73002713448,9188	0,222861486260358	0,0102561646973156	0,0097759123318453	0,856864218702735	-0.222861486260358	1,16704604787205	0.222861486260358	1224
O55142	sp|O55142|RL35A_MOUSE 60S ribosomal protein L35a OS=Mus musculus GN=Rpl35a PE=1 SV=2	−1,17	0,53684827957061	0,831016807971437	26,7334809843027	26,3210361882821	26,1785692818595	25,9245627621526	26,275921090209	26,5695977401837	295232440860,341	253009750430,374	0,222658272723031	0,0109118790796751	0,0122996082070247	0,856984922432896	-0.22265827272303	1,16688167297168	0.22265827272303	1225
Q9R1Z7	sp|Q9R1Z7|PTPS_MOUSE 6-pyruvoyl tetrahydrobiopterin synthase OS=Mus musculus GN=Pts PE=2 SV=2	−1,17	0,296462269909442	0,711969538636193	26,7585971508416	26,6553553897964	26,4201728012214	26,2799304014618	26,4376778659567	26,6541371203836	360722612666,151	309198240329,579	0,22235775557496	0,006517732996388	0,0071008680973230	0,857163453225878	-0.222357755574958	1,16663863378282	0.222357755574958	1226
Q8CI32	sp|Q8CI32|BAG5_MOUSE BAG family molecular chaperone regulator 5 OS=Mus musculus GN=Bag5 PE=1 SV=1	−1,17	0,354770450204873	0,732843629012038	27,8617602051029	27,570534038433	27,9420598071018	27,4203446407034	27,8436592712793	27,6483028203886	1174015864226,1	1006435078245,06	0,22219779157298	0,00703408636176115	0,0076659228216880	0,857258499576147	-0.222197791572977	1,16650928569904	0.222197791572977	1227
Q9DCD0	sp|Q9DCD0|6PGD_MOUSE 6-phosphogluconate dehydrogenase, decarboxylating OS=Mus musculus GN=Pgd PE=1 SV=3	−1,17	0,106797129417634	0,47075990957772	30,8504306917222	30,5561361051944	30,7077418394737	30,6232978272985	30,5561361051944	30,4732906815785	21622802508164,6	18539194058211,1	0,221974993317696	0,00479306010862596	0,0024595045198858	0,857390897928742	-0.221974993317697	1,16632915326693	0.221974993317697	1228
Q9D273	sp|Q9D273|MMAB_MOUSE Cob(I)yrinic acid a,c-diamide adenosyltransferase, mitochondrial OS=Mus musculus GN=Mmab PE=1 SV=1;sp|Q9D273-2|MMAB_MOUSE Isoform 2 of Cob(I)yrinic acid a,c-diamide adenosyltransferase, mitochondrial OS=Mus musculus GN=Mmab	−1,17	0,636422703759481	0,883588334123886	26,0967000396117	27,1281225775127	26,4967677565846	26,6443531342949	26,2102331083515	26,4055239012705	347441957041,222	297903878079,491	0,221925079663096	0,0195686754941705	0,0082294959647256	0,857420562031161	-0.221925079663099	1,16628880188163	0.221925079663099	1229
P47856-2	sp|P47856-2|GFPT1_MOUSE Isoform 2 of Glutamine--fructose-6-phosphate aminotransferase [isomerizing] 1 OS=Mus musculus GN=Gfpt1;sp|P47856|GFPT1_MOUSE Glutamine--fructose-6-phosphate aminotransferase [isomerizing] 1 OS=Mus musculus GN=Gfpt1 PE=1 SV=3	−1,17	0,318362902919301	0,722907157640623	27,7120449619398	27,3835409950841	27,1995228618436	27,2620275033788	27,2250623653521	27,3465665285104	819289173454,886	702481895117,251	0,221911706762081	0,00946471365159514	0,0022833515978427	0,857428509832411	-0.22191170676208	1,16627799114757	0.22191170676208	1230
Q80TL0	sp|Q80TL0|PPM1E_MOUSE Protein phosphatase 1E OS=Mus musculus GN=Ppm1e PE=1 SV=2	−1,17	0,323775560671392	0,726844368394785	30,6837245297255	30,5018948562202	30,2570757598888	30,4385207524286	30,4045709486719	30,1381873875547	17285660172435,7	14821397499339,3	0,221894219159705	0,00702400392578668	0,0054233812818055	0,857438903199891	-0.221894219159707	1,16626385421525	0.221894219159707	1231
P32233	sp|P32233|DRG1_MOUSE Developmentally-regulated GTP-binding protein 1 OS=Mus musculus GN=Drg1 PE=1 SV=1	−1,17	0,365434666081211	0,739315231400715	27,8373225183337	27,4305707381541	27,4953994502371	27,2462324621921	27,6388890090614	27,4170667080726	957665353052,833	821224804187,525	0,221744405003191	0,00792166604821165	0,0071764526038866	0,857527947074242	-0.221744405003191	1,16614275186232	0.221744405003191	1232
P16254	sp|P16254|SRP14_MOUSE Signal recognition particle 14 kDa protein OS=Mus musculus GN=Srp14 PE=1 SV=1	−1,17	0,507871034423004	0,816124555116335	27,02680358158	26,8109304958059	26,593052849711	26,2659948811303	26,8446549836125	26,8591980973836	440097578694,785	377416801276,444	0,221664786305138	0,00808929563733495	0,012693507086817	0,857575273183197	-0.221664786305138	1,1660783971629	0.221664786305138	1233
Q8R016	sp|Q8R016|BLMH_MOUSE Bleomycin hydrolase OS=Mus musculus GN=Blmh PE=1 SV=1	−1,17	0,121978563608859	0,498344464645016	30,8944000450301	30,7235744445341	30,9123179140515	30,5960927494958	30,8467694077559	30,6271401858965	24838868022564,5	21305807009673,9	0,221352729202311	0,00337785244931839	0,0044525980644751	0,857760788064857	-0.22135272920231	1,16582619993162	0.22135272920231	1234
Q80TG9	sp|Q80TG9|LRFN2_MOUSE Leucine-rich repeat and fibronectin type-III domain-containing protein 2 OS=Mus musculus GN=Lrfn2 PE=1 SV=2	−1,17	0,463020697340416	0,793595716606975	24,4331596823421	24,163506496647	23,921929541055	24,1881015331326	24,1454988690372	23,7251359806086	31487775875,4623	27012857525,7709	0,221145595126573	0,0105797773225928	0,0106531063299492	0,857883949397055	-0.22114559512657	1,16565882914913	0.221145595126569	1235
Q99PU5	sp|Q99PU5|ACBG1_MOUSE Long-chain-fatty-acid--CoA ligase ACSBG1 OS=Mus musculus GN=Acsbg1 PE=1 SV=1	−1,17	0,380125055902431	0,74951678051309	31,138693565225	31,0083247513143	30,6402261423119	30,6923605230542	30,9568503354048	30,6783315069509	27060101410585,2	23215643476163,3	0,221069976831357	0,00835813089944544	0,0050984772884603	0,857928916226528	-0.221069976831358	1,16559773319956	0.221069976831358	1236
P62305	sp|P62305|RUXE_MOUSE Small nuclear ribonucleoprotein E OS=Mus musculus GN=Snrpe PE=2 SV=1	−1,17	0,535440919228683	0,8300431463043	27,8099832106879	28,0865111619099	27,8935880061131	27,3309114068393	27,8832358468707	28,1167813912022	1348518177962	1157144230000,09	0,220806271585663	0,00507767534274609	0,0145288275287145	0,858085748424147	-0.220806271585653	1,16538469708473	0.220806271585654	1237
Q80Z24	sp|Q80Z24|NEGR1_MOUSE Neuronal growth regulator 1 OS=Mus musculus GN=Negr1 PE=1 SV=1	−1,17	0,0885275176044105	0,441176105282424	29,5915968002301	29,4348108568803	29,5856163544791	29,2370422808287	29,465248371337	29,450672574706	6728284590981,42	5773624000439,95	0,220761572526281	0,0030078650154148	0,0043477342484206	0,858112334929902	-0.220761572526282	1,16534859049857	0.220761572526282	1238

Q8CAK3	sp|Q8CAK3|CS066_MOUSE UPF0515 protein C19orf66 homolog OS=Mus musculus PE=2 SV=1;sp|Q8CAK3-2|CS066_MOUSE Isoform 2 of UPF0515 protein C19orf66 homolog OS=Mus musculus	−1,17	0,486100730741064	0,804557984450102	24,3413012075385	23,6613231107722	24,1153516205821	23,9566725543437	23,9964472236921	23,7058662910714	27551568753,2045	23642899865,4006	0,220727469652714	0,0144057943563115	0,0065956254015146	0,858132619495604	-0.220727469652715	1,16532104395214	0.220727469652715	1239
Q9DBL1	sp|Q9DBL1|ACDSB_MOUSE Short/branched chain specific acyl-CoA dehydrogenase, mitochondrial OS=Mus musculus GN=Acadsb PE=1 SV=1	−1,16	0,436528882255715	0,778450481049573	29,3963002344319	29,0458241396507	29,2687031531706	28,7501446235731	29,2657804305218	29,2370422808287	4982448282172,77	4277211715889,8	0,220184209631442	0,00606710360782606	0,0099628580671579	0,858455818035019	-0.22018420963144	1,16488231425698	0.22018420963144	1240
P97855	sp|P97855|G3BP1_MOUSE Ras GTPase-activating protein-binding protein 1 OS=Mus musculus GN=G3bp1 PE=1 SV=1	−1,16	0,428855380858556	0,774601899608828	27,2983095342163	27,8140234660062	27,8881308498074	27,6075461660519	27,5100018761904	27,4252531095558	1036448709570,17	889803999910,051	0,2200892350463	0,0116126238276083	0,0033154196951327	0,858512333214313	-0.220089235046299	1,16480563098721	0.220089235046299	1241
P63087	sp|P63087|PP1G_MOUSE Serine/threonine-protein phosphatase PP1-gamma catalytic subunit OS=Mus musculus GN=Ppp1cc PE=1 SV=1;sp|P63087-2|PP1G_MOUSE Isoform Gamma-2 of Serine/threonine-protein phosphatase PP1-gamma catalytic subunit OS=Mus musculus GN=Ppp1cc	−1,16	0,301054939186263	0,714288613397098	27,8355439715928	27,6705231091959	27,9743749343137	27,6623996166401	27,4758239742999	27,8851022330226	1216274975448,5	1044377302875,31	0,219826420689176	0,00546645087394738	0,0074041132009417	0,858668741819832	-0.219826420689167	1,1645934588007	0.219826420689167	1242
Q56WH1	sp|Q56WH1|TBA3_ARATH Tubulin alpha-3 chain OS=Arabidopsis thaliana GN=TUBA3 PE=1 SV=2;sp|B9DHQ0|TBA5_ARATH Tubulin alpha-5 chain OS=Arabidopsis thaliana GN=TUBA5 PE=1 SV=2	−1,16	0,32419185508352	0,726844368394785	35,3451936065751	35,211446325403	35,4378163435131	34,9023917375126	35,3451936065751	35,2909566904371	2209373586332390	1897880271122430	0,219248404837586	0,00322109004420743	0,0068654219512616	0,859012836427068	-0.219248404837586	1,16412695782213	0.219248404837586	1243
Q9D365	sp|Q9D365|SPCS3_MOUSE Signal peptidase complex subunit 3 OS=Mus musculus GN=Spcs3 PE=2 SV=1	−1,16	0,213617535664991	0,645012843958033	25,10451441028	25,3243138240262	25,0126127548663	25,11461226522	25,0272681171071	24,8439697910874	83419421957,7621	71666055245,0738	0,219093369697696	0,00636907678495049	0,0055261099359167	0,859105152770785	-0.219093369697696	1,16400186493446	0.219093369697696	1244
Q9D7X3	sp|Q9D7X3|DUS3_MOUSE Dual specificity protein phosphatase 3 OS=Mus musculus GN=Dusp3 PE=1 SV=1	−1,16	0,430619799736565	0,775108167118334	30,2703137998586	30,333769820244	30,2664339291469	29,7669313833442	30,2434645802855	30,4045709486719	14284162420798,6	12271766141437,7	0,219073548266238	0,0012481334351461	0,0110015358213763	0,859116956243039	-0.219073548266232	1,1639858726256	0.219073548266232	1245
P15864	sp|P15864|H12_MOUSE Histone H1.2 OS=Mus musculus GN=Hist1h1c PE=1 SV=2	−1,16	0,0778418783293223	0,416490629392219	26,8222501839952	26,8719382927359	26,6568132883569	26,5203006373062	26,6793021818755	26,696871523607	428547429023,994	368297957364,315	0,218581486033085	0,00420528356165434	0,0036523106569609	0,859410026570698	-0.218581486033085	1,16358893785577	0.218581486033085	1246
Q80W22	sp|Q80W22|THNS2_MOUSE Threonine synthase-like 2 OS=Mus musculus GN=Thnsl2 PE=2 SV=1	−1,16	0,409158716167355	0,76291790520755	25,4336098486706	24,8473603223002	24,974920529059	24,8868677141354	25,0214896836834	24,8936242018883	78416241121,4402	67405595221,1044	0,218284136016639	0,0122907430277298	0,0030419863800178	0,859587175528038	-0.218284136016638	1,16334913836483	0.218284136016638	1247
Q9ERN0	sp|Q9ERN0|SCAM2_MOUSE Secretory carrier-associated membrane protein 2 OS=Mus musculus GN=Scamp2 PE=2 SV=1	−1,16	0,620385467579909	0,871535016200931	23,7711154655879	24,305821491108	23,9469638537138	23,3681777993606	24,2627398725708	23,93947115125	26701002188,6393	22954877414,8011	0,218093164985986	0,011351347682859 8	0,0189872669916358	0,85970096750031	-0.218093164985988	1,16319515483114	0.218093164985988	1248
Q91YI0	sp|Q91YI0|ARLY_MOUSE Argininosuccinate lyase OS=Mus musculus GN=Asl PE=1 SV=1	−1,16	0,371349638014883	0,74351830919698	28,2599624963169	28,159207652022	28,3022437834415	27,9434810304077	27,9101208805373	28,415569281999	1839419797897,05	1582020163837,48	0,217482785532485	0,00260204874171314	0,0100635759627186	0,860064769144136	-0.217482785532483	1,162703131062	0.217482785532484	1249
Q8R550	sp|Q8R550|SH3K1_MOUSE SH3 domain-containing kinase-binding protein 1 OS=Mus musculus GN=Sh3kbp1 PE=1 SV=1;sp|Q8R550-2|SH3K1_MOUSE Isoform 2 of SH3 domain-containing kinase-binding protein 1 OS=Mus musculus GN=Sh3kbp1;sp|Q8R550-3|SH3K1_MOUSE Isoform 3 of	−1,16	0,17493736759677	0,590462219359101	28,2185163002376	27,9512595440036	27,8869880507204	27,9495817820908	27,7954933381178	27,860378365634	1473882657503,91	1268028559913,32	0,217034429712436	0,00627490901563154	0,0027760171251429	0,860332098662987	-0.217034429712439	1,16234184631036	0.217034429712439	1250
O55074	sp|O55074|AKA7A_MOUSE A-kinase anchor protein 7 isoform alpha OS=Mus musculus GN=Akap7 PE=1 SV=4;sp|Q7TN79|AKA7G_MOUSE A-kinase anchor protein 7 isoform gamma OS=Mus musculus GN=Akap7 PE=1 SV=2	−1,16	0,611058952939175	0,866640865424611	24,8100392117994	24,5823748158982	24,0715250354999	23,9835441013259	24,3563980610013	24,6730051571631	43151356366,3421	37128440623,2937	0,216881184042751	0,0154442249937896	0,014180198908141	0,860423489544207	-0.216881184042751	1,16221838681988	0.216881184042751	1251
Q91W53	sp|Q91W53|GOGA7_MOUSE Golgin subfamily A member 7 OS=Mus musculus GN=Golga7 PE=2 SV=1;sp|Q91W53-2|GOGA7_MOUSE Isoform 2 of Golgin subfamily A member 7 OS=Mus musculus GN=Golga7	−1,16	0,732126631161511	0,924701393764781	24,7695581872001	23,7584773744894	23,8514133380406	23,5506907098139	23,8277112897959	24,5500906114161	30060653670,9889	25865198211,3269	0,216864133790361	0,0231635378724926	0,0215202724557534	0,860433658376798	-0.21686413379036	1,16220465141554	0.21686413379036	1252
P46656	sp|P46656|ADX_MOUSE Adrenodoxin, mitochondrial OS=Mus musculus GN=Fdx1 PE=1 SV=1	−1,16	0,564554353603371	0,845110794140252	24,473229654249	24,7487693428392	23,9578712584091	24,0319190122397	24,2532325976359	24,4443019138267	39252857660,6682	33780554882,991	0,216604661764696	0,0164579354588983	0,0085127634636215	0,860588423268849	-0.216604661764697	1,1619956450281	0.216604661764697	1253
P36552	sp|P36552|HEM6_MOUSE Oxygen-dependent coproporphyrinogen-III oxidase, mitochondrial OS=Mus musculus GN=Cpox PE=1 SV=2	−1,16	0,192319400115044	0,618802084535046	27,5713979478984	27,2481132134366	27,4078116367401	27,2537191581988	27,3782962363329	27,1451359480015	800984633237,639	689374462671,63	0,216486708820016	0,00589753836294475	0,0042801026718213	0,860658786779876	-0.216486708820016	1,16190064559901	0.216486708820016	1254
Q9CXY6	sp|Q9CXY6|ILF2_MOUSE Interleukin enhancer-binding factor 2 OS=Mus musculus GN=Ilf2 PE=1 SV=1	−1,16	0,185801994532179	0,609360732067603	29,151901871807	28,7693521714792	28,9889107169626	28,8326097047498	28,8525957718364	28,7755965557594	3815355343427,55	3284604431799,89	0,216097793035436	0,00662651948684944	0,0013863803862185	0,860890830904663	-0.216097793035438	1,16158746742506	0.216097793035438	1255

P42227-2	sp|P42227-2|STAT3_MOUSE Isoform Stat3B of Signal transducer and activator of transcription 3 OS=Mus musculus GN=Stat3;sp|P42227-3|STAT3_MOUSE Isoform Del-701 of Signal transducer and activator of transcription 3 OS=Mus musculus GN=Stat3;sp|P42227|STAT3_	−1,16	0,331489686303791	0,72953198881961	27,5237275215483	27,0441476199735	27,1138793678718	27,02680358158	27,1479973377852	27,0584803792591	667797955650,446	575071623660,79	0,215670025716111	0,00951665050827265	0,0023212838040738	0,861146127799479	-0.215670025716111	1,16124310116256	0.215670025716111	1256
Q9D7X8	sp|Q9D7X8|GGCT_MOUSE Gamma-glutamylcyclotransferase OS=Mus musculus GN=Ggct PE=1 SV=1	−1,16	0,379424144141924	0,748785300113128	27,55952828088	27,6736691686645	27,7048655608525	27,2849017576652	27,3878476077223	27,8187164630971	1015112950453,21	874707407681,39	0,214767846540023	0,00276754995937462	0,0102991457515168	0,861684807873711	-0.214767846540019	1,16051715298033	0.214767846540019	1257
Q9CQM9	sp|Q9CQM9|GLRX3_MOUSE Glutaredoxin-3 OS=Mus musculus GN=Glrx3 PE=1 SV=1	−1,16	0,659098322712772	0,89341619202324	30,3721900067996	29,9945463914196	29,5382706477389	29,402682568762	30,215226639837 8	29,8416907060905	10353395836647,8	8924903562509,85	0,214195553174242	0,01393394326287 4	0,0136389418267846	0,862026691852973	-0.214195553174236	1,16005688623219	0.214195553174236	1258
Q9Z110-2	sp|Q9Z110-2|P5CS_MOUSE Isoform Short of Delta-1-pyrroline-5-carboxylate synthase OS=Mus musculus GN=Aldh18a1;sp|Q9Z110|P5CS_MOUSE Delta-1-pyrroline-5-carboxylate synthase OS=Mus musculus GN=Aldh18a1 PE=1 SV=2	−1,16	0,3521792148167	0,732843629012038	29,483312762562	29,3492574870933	28,961551696187	29,1339128359005	29,1591429707384	29,0558930771154	5122723336757,08	4416268808694,13	0,214082989670596	0,00925898968703034	0,0018486942429697	0,862093952450267	-0.214082989670594	1,15996637855743	0.214082989670594	1259
Q9JLT4-2	sp|Q9JLT4-2|TRXR2_MOUSE Isoform 2 of Thioredoxin reductase 2, mitochondrial OS=Mus musculus GN=Txnrd2;sp|Q9JLT4-4|TRXR2_MOUSE Isoform 4 of Thioredoxin reductase 2, mitochondrial OS=Mus musculus GN=Txnrd2;sp|Q9JLT4|TRXR2_MOUSE Thioredoxin reductase 2, mi	−1,16	0,617731883396433	0,870166479711399	26,9675427036421	27,7887330476403	27,7823931945621	27,5780928315527	27,3412496695829	27,1751206599197	888579225811,5	766285807307,011	0,213617827616527	0,0171663155798904	0,0074006524898748	0,86237195856925	-0.213617827616528	1,1595924357966	0.213617827616528	1260
Q91V09	sp|Q91V09|WDR13_MOUSE WD repeat-containing protein 13 OS=Mus musculus GN=Wdr13 PE=1 SV=1	−1,16	0,652840932422504	0,891901943526937	28,9559879547382	28,2648305123125	27,8646384205141	28,1113694390224	28,159207652022	28,3714205600626	2076736864298,71	1791365361910,8	0,213258813757957	0,0194663628508546	0,0049058365713678	0,86258658605539	-0.213258813757957	1,15930390776537	0.213258813757957	1261
O55091	sp|O55091|IMPCT_MOUSE Protein IMPACT OS=Mus musculus GN=Impact PE=1 SV=2	−1,16	0,375594257708249	0,746362141983349	29,7939275700076	29,41407607155	29,5039457329552	29,4260099986843	29,6309744420806	29,2120692784127	6956167496122,58	6001424096393,14	0,212987788527749	0,00671330052339381	0,0071192088617323	0,862748647116157	-0.21298778852775	1,15908614095498	0.21298778852775	1262
O35685	sp|O35685|NUDC_MOUSE Nuclear migration protein nudC OS=Mus musculus GN=Nudc PE=1 SV=1	−1,16	0,194581891258157	0,621976904521672	30,9907657784686	30,6703686802763	30,7513155117469	30,5474125413229	30,7552693729942	30,6680635283742	23882091638375,2	20612424635674,5	0,212414977265286	0,00540852067265787	0,0034046448790795	0,863091263017901	-0.212414977265284	1,15862602583113	0.212414977265284	1263
P29515	sp|P29515|TBB7_ARATH Tubulin beta-7 chain OS=Arabidopsis thaliana GN=TUBB7 PE=2 SV=1	−1,16	0,614977329824968	0,868726908818952	26,0592651454691	26,0460311816041	25,8727859475999	25,6392323739276	26,4113392806788	25,4860362621515	194304837785,907	167715675060,739	0,212304288947963	0,00400320583406768	0,0191890354062066	0,863157484763889	-0.212304288947964	1,15853713563469	0.212304288947964	1264
O35345	sp|O35345|IMA7_MOUSE Importin subunit alpha-7 OS=Mus musculus GN=Kpna6 PE=1 SV=2	−1,16	0,590154341335927	0,858561075424286	27,1684490497881	28,0091322344659	27,2837384201724	27,4854015248497	27,275090612165	27,2596824498093	865961743245,488	747543396173,692	0,212145957825833	0,0165804190318397	0,004612519940452	0,863252218708897	-0.212145957825833	1,15840999690175	0.212145957825833	1265
Q02248	sp|Q02248|CTNB1_MOUSE Catenin beta-1 OS=Mus musculus GN=Ctnnb1 PE=1 SV=1	−1,16	0,397133222554232	0,754338506152154	30,5053526695314	30,2598956411556	29,9112106332108	30,108137787763	30,0580803905391	30,0707031025332	13389424764579,8	11564645522760,5	0,211372935594768	0,00987781862114944	0,0008655020327155	0,863714888884058	-0.211372935594771	1,15778946602626	0.211372935594771	1266
P27659	sp|P27659|RL3_MOUSE 60S ribosomal protein L3 OS=Mus musculus GN=Rpl3 PE=1 SV=3	−1,16	0,478235279832406	0,799905345588749	26,0840270683502	25,5872669040152	25,663480608275	25,3969460609751	25,6268016812449	25,8716928723719	156803331829,065	135442568430,776	0,211274978037394	0,0103781824545628	0,0092624380362815	0,863773536256392	-0.211274978037396	1,15771085594265	0.211274978037396	1267
Q8R361	sp|Q8R361|RFIP5_MOUSE Rab11 family-interacting protein 5 OS=Mus musculus GN=Rab11fip5 PE=1 SV=2	−1,16	0,357728358731978	0,732843629012038	26,3976388083925	26,262865549841	25,9776537154619	26,2537200098112	26,0333327348677	25,9119492395323	242124854537,37	209153442616,936	0,211189437491625	0,00818061331549105	0,0066468435721357	0,863824752797755	-0.211189437491628	1,15764221476775	0.211189437491628	1268
O54950	sp|O54950|AAKG1_MOUSE 5-AMP-activated protein kinase subunit gamma-1 OS=Mus musculus GN=Prkag1 PE=1 SV=2	−1,16	0,538257918932829	0,831485169227261	26,8468256315125	26,3810756525348	26,2170897415584	26,1684627487745	26,6234109353197	26,2142780507656	316840292652,376	273723391025,648	0,211037089502081	0,0123366876761602	0,0095114768140298	0,863915977144883	-0.211037089502081	1,15751997469112	0.211037089502082	1269
P08752	sp|P08752|GNAI2_MOUSE Guanine nucleotide-binding protein G(i) subunit alpha-2 OS=Mus musculus GN=Gnai2 PE=1 SV=5	−1,16	0,0438273035651463	0,307468517592271	29,6878677659393	29,4546675630231	29,6230833170184	29,4054016596301	29,4774744514319	29,4440052105859	7081731448867,59	6118228892276,51	0,210988054022696	0,00406835385501614	0,0012250017971731	0,863945341114968	-0.210988054022695	1,15748063263984	0.210988054022695	1270
Q61548-3	sp|Q61548-3|AP180_MOUSE Isoform 3 of Clathrin coat assembly protein AP180 OS=Mus musculus GN=Snap91;sp|Q61548-2|AP180_MOUSE Isoform Short of Clathrin coat assembly protein AP180 OS=Mus musculus GN=Snap91;sp|Q61548|AP180_MOUSE Clathrin coat assembly prot	−1,16	0,320697637576652	0,723842156618807	28,7339849515457	28,399855058049	28,5614572625613	28,5948343218379	28,4487032309194	28,2133967375042	2544865945115,26	2198899440386,03	0,210808033362793	0,00584961088272865	0,0067718231143059	0,864053151643097	-0.210808033362791	1,1573362102764	0.210808033362791	1271
Q8VBZ3	sp|Q8VBZ3|CLPT1_MOUSE Cleft lip and palate transmembrane protein 1 homolog OS=Mus musculus GN=Clptm1 PE=2 SV=1	−1,16	0,437400510563263	0,778453531535867	27,0605273992974	26,7207981765655	26,9639504293006	26,9015107380824	26,9524248585844	26,4531431572982	488737807538,083	422318772680,701	0,210728333745085	0,00650398165307146	0,0102635304321947	0,864100886338312	-0.210728333745085	1,15727227666386	0.210728333745085	1272
Q8R4E6	sp|Q8R4E6|PURG_MOUSE Purine-rich element-binding protein gamma OS=Mus musculus GN=Purg PE=2 SV=1	−1,16	0,764124617244938	0,933610131271809	24,6467589088478	24,7090851139904	23,5957802327646	24,0493524445907	24,7872986927083	23,6768094780596	36377201582,1209	31433924302,377	0,210712170292693	0,0257246454185632	0,0233822259863109	0,864110567477695	-0.210712170292694	1,15725931106127	0.210712170292694	1273
Q9WTM5	sp|Q9WTM5|RUVB2_MOUSE RuvB-like 2 OS=Mus musculus GN=Ruvbl2 PE=2 SV=3	−1,16	0,0365476740333285	0,279698526792889	29,3897530370284	29,2762930224565	29,1870270397349	29,1319348639858	29,2069428457707	29,0763948194066	5224382368150,94	4514990399270,12	0,210537570439722	0,00346954273322867	0,0022484227464083	0,864215151401352	-0.210537570439723	1,15711926408426	0.210537570439723	1274

Q8CI08	sp|Q8CI08|SLAI2_MOUSE SLAIN motif-containing protein 2 OS=Mus musculus GN=Slain2 PE=1 SV=2;sp|Q8CI08-2|SLAI2_MOUSE Isoform 2 of SLAIN motif-containing protein 2 OS=Mus musculus GN=Slain2	−1,16	0,666103373351444	0,89771450844655	22,61434833139	21,6345534049339	21,5718826381945	21,7927037897341	21,7767731508675	21,8141497464129	3377026610,26698	2919103043,06297	0,210228409282777	0,0266458423020847	0,0008605822191852	0,864400367526921	-0.210228409282777	1,15687132672217	0.210228409282777	1275
P56565	sp|P56565|S10A1_MOUSE Protein S100-A1 OS=Mus musculus GN=S100a1 PE=1 SV=2	−1,16	0,285317060054589	0,704836107321858	29,0218456153508	28,9940526281682	28,8780890237702	28,5655362258062	28,9044917431211	28,9871137767746	3794836422530,87	3280599344347,57	0,210078289209519	0,00263257817079508	0,00775181139834467	0,864490317651072	-0.210078289209519	1,15675095438561	0.210078289209519	1276
Q9JLV6	sp|Q9JLV6|PNKP_MOUSE Bifunctional polynucleotide phosphatase/kinase OS=Mus musculus GN=Pnkp PE=1 SV=2;sp|Q9JLV6-2|PNKP_MOUSE Isoform 2 of Bifunctional polynucleotide phosphatase/kinase OS=Mus musculus GN=Pnkp	−1,16	0,601031385136324	0,862877020598131	23,7451180191967	24,656098949068	24,4478937746508	24,1529970070456	24,0692182003921	24,1908952126927	35155148414,4446	30399848852,1303	0,209671834502625	0,0196574051154562	0,0025795120183483	0,864733907356783	-0.209671834502624	1,15642510544854	0.209671834502624	1277
Q8C080	sp|Q8C080|SNX16_MOUSE Sorting nexin-16 OS=Mus musculus GN=Snx16 PE=1 SV=2	−1,16	0,279325213060748	0,699221280539289	27,0553730818285	26,6595208620373	26,9970599843299	26,8246682302946	26,7968413620251	26,6553553897964	483339258571,818	418086837888,093	0,209233554951542	0,0079434369590967	0,0033929689061251	0,864996646710358	-0.209233554951543	1,15607384583867	0.209233554951543	1278
Q8BX94	sp|Q8BX94|OSBL2_MOUSE Oxysterol-binding protein-related protein 2 OS=Mus musculus GN=Osbpl2 PE=2 SV=1	−1,16	0,594033895487289	0,860080030359509	26,6511319794876	27,1953399119946	27,4089401757686	27,210872020632	26,6864288116258	26,9234814664797	579329568632,833	501194840812,844	0,209012737218913	0,0144263158619301	0,0097483811368795	0,865129052528114	-0.209012737218913	1,1558969116547	0.209012737218913	1279
P05977-2	sp|P05977-2|MYL1_MOUSE Isoform MLC3 of Myosin light chain 1/3, skeletal muscle isoform OS=Mus musculus GN=Myl1;sp|P05977|MYL1_MOUSE Myosin light chain 1/3, skeletal muscle isoform OS=Mus musculus GN=Myl1 PE=1 SV=2;sp|P09542|MYL3_MOUSE Myosin light chain	−1,16	0,400323410036058	0,755487293133664	26,2170897415584	26,2985172198808	25,9834974475046	25,7487841328276	26,0820246055589	26,234878662775	231158194166,316	200062529151,24	0,208429522588161	0,00624925317757701	0,0095520721456238	0,865478854741775	-0.208429522588155	1,15542973062971	0.208429522588155	1280
Q91V81	sp|Q91V81|RBM42_MOUSE RNA-binding protein 42 OS=Mus musculus GN=Rbm42 PE=1 SV=1;sp|Q91V81-2|RBM42_MOUSE Isoform 2 of RNA-binding protein 42 OS=Mus musculus GN=Rbm42	−1,16	0,534563816411361	0,8300431463043	23,1051187112	23,6978719068565	23,6519620919524	23,2491539067247	23,1698545542957	23,6030522836631	15827016735,0365	13700345862,4277	0,20817703053851	0,0140418697593558	0,0098818266543834	0,865630339045453	-0.208177030538514	1,15522753176918	0.208177030538514	1281
Q9CWQ0	sp|Q9CWQ0|DPH5_MOUSE Diphthine synthase OS=Mus musculus GN=Dph5 PE=2 SV=2	−1,15	0,563666745276353	0,845110794140252	23,2262234311461	23,5733620117965	23,7382122559989	23,0151791532857	23,7026990686974	23,3877341976412	16270170425,9399	14087271177,1275	0,207837186405433	0,0111151873261627	0,0147275680899635	0,865834272680257	-0.207837186405436	1,15495543610722	0.207837186405436	1282
Q00612	sp|Q00612|G6PD1_MOUSE Glucose-6-phosphate 1-dehydrogenase X OS=Mus musculus GN=G6pdx PE=1 SV=3;sp|P11413|G6PD_HUMAN Glucose-6-phosphate 1-dehydrogenase OS=Homo sapiens GN=G6PD PE=1 SV=4;sp|P11413-2|G6PD_HUMAN Isoform Long of Glucose-6-phosphate 1-dehydr	−1,15	0,423875515806466	0,7711031271846	29,6094844022266	30,1853711148321	30,0689715847678	29,8841798123692	29,7503945006217	29,7971159967527	10212249366138,8	8842258413424,09	0,207813868974557	0,010165392674413	0,0022776772186532	0,865848266763124	-0.207813868974563	1,15493676939308	0.207813868974563	1283
Q80TR1-2	sp|Q80TR1-2|LPHN1_MOUSE Isoform 2 of Latrophilin-1 OS=Mus musculus GN=Lphn1;sp|Q80TR1|LPHN1_MOUSE Latrophilin-1 OS=Mus musculus GN=Lphn1 PE=1 SV=2	−1,15	0,799255395729478	0,944947668931349	22,7369952893588	22,3912477426894	20,9996327546211	22,0223540321112	22,1585650899638	21,5152196899429	3741024204,01416	3239591042,51898	0,20762159743267	0,041721541703492	0,0154815516448715	0,865963668196229	-0.207621597432673	1,15478285836514	0.207621597432673	1284
Q8CFA2	sp|Q8CFA2|GCST_MOUSE Aminomethyltransferase, mitochondrial OS=Mus musculus GN=Amt PE=1 SV=1	−1,15	0,200218188930648	0,6308537814738	24,7499941271413	24,5294754056694	24,7251231135384	24,5413781637526	24,3651120990611	24,6675378862814	51672809350,3746	44764267143,2235	0,20705775499044	0,00489613364160455	0,006193889190608	0,866302175283198	-0.20705775499044	1,15433162761377	0.20705775499044	1285
P51174	sp|P51174|ACADL_MOUSE Long-chain specific acyl-CoA dehydrogenase, mitochondrial OS=Mus musculus GN=Acadl PE=1 SV=2	−1,15	0,179641207856744	0,598356502628386	31,3898117118546	31,1488485026459	31,2730098935786	31,1607157709667	31,2478449838581	30,9727064202915	38074150479440,4	32985495760646,7	0,20698005898985	0,00385347583742338	0,0045175387210231	0,866348831038489	-0.206980058989852	1,15426946303062	0.206980058989852	1286
P70303	sp|P70303|PYRG2_MOUSE CTP synthase 2 OS=Mus musculus GN=Ctps2 PE=2 SV=1;sp|P70303-3|PYRG2_MOUSE Isoform 3 of CTP synthase 2 OS=Mus musculus GN=Ctps2;sp|P70303-2|PYRG2_MOUSE Isoform 2 of CTP synthase 2 OS=Mus musculus GN=Ctps2	−1,15	0,468242986561314	0,796311321094718	26,1771723436785	26,6200366760221	26,2503112648405	26,4605063694145	25,9701667726243	26,1876191399344	277524048650,171	240526817445,188	0,20641503690521	0,0090100692125716	0,0093753564475425	0,866688197347469	-0.206415036905212	1,15381748945069	0.206415036905212	1287
P50247	sp|P50247|SAHH_MOUSE Adenosylhomocysteinase OS=Mus musculus GN=Ahcy PE=1 SV=3	−1,15	0,0513063016475169	0,334116647314317	31,0970439805281	30,8553810226389	30,9306955015522	30,7847908092274	30,8286913985065	30,8405947262776	27938873998288,4	24215781016247,7	0,206326343928495	0,00399392776493369	0,0009536990382452	0,866741480624137	-0.206326343928493	1,15374655806239	0.206326343928493	1288
Q8BXZ1	sp|Q8BXZ1|TMX3_MOUSE Protein disulfide-isomerase TMX3 OS=Mus musculus GN=Tmx3 PE=1 SV=2	−1,15	0,226268898557777	0,652238880554131	28,9363589553108	28,9450977968638	28,9363589553108	28,5681116849645	28,8574544305787	28,963339565092	3699696490039,81	3206823160971,68	0,206262122908131	0,000174343450658882	0,0071046405708980	0,86678006414985	-0.206262122908129	1,1536952006168	0.206262122908129	1289
Q8VE22	sp|Q8VE22|RT23_MOUSE 28S ribosomal protein S23, mitochondrial OS=Mus musculus GN=Mrps23 PE=1 SV=1	−1,15	0,737355266028102	0,924801202680728	27,3642301077782	27,2429325656057	27,840803271956	26,7284847671728	28,046173766494	27,2462324621921	862115694631,117	747721933104,766	0,205379637234704	0,0114995856025248	0,0242814320941752	0,867310429170069	-0.205379637234703	1,15298970975929	0.205379637234703	1290

P48722	sp|P48722|HS74L_MOUSE Heat shock 70 kDa protein 4L OS=Mus musculus GN=Hspa4l PE=1 SV=2;sp|P48722-2|HS74L_MOUSE Isoform 2 of Heat shock 70 kDa protein 4L OS=Mus musculus GN=Hspa4l	−1,15	0,430501988296779	0,775108167118334	32,7150311669666	32,3737547971211	32,20118575714	32,2317495018469	32,4800692515926	32,1512238864603	121385260408364	105283821360769	0,20530948948102	0,0080634573407883	0,0053088060818065	0,86735260118546	-0.205309489481027	1,15293364962905	0.205309489481027	1291
Q8K0G5	sp|Q8K0G5|TSSC1_MOUSE Protein TSSC1 OS=Mus musculus GN=Tssc1 PE=2 SV=2	−1,15	0,219692828450479	0,648792713611515	28,5161702425418	28,1438826384783	28,3588682131998	28,2774605455253	28,1810020312617	28,1337438378691	2031184760782,37	1761879297930	0,205206384027116	0,00659454823795962	0,0025977045471821	0,867414590709789	-0.205206384027118	1,15285125557056	0.205206384027118	1292
P70444	sp|P70444|BID_MOUSE BH3-interacting domain death agonist OS=Mus musculus GN=Bid PE=1 SV=2	−1,15	0,617464969805304	0,870166479711399	25,484150064461	24,9733867043026	24,6878806630467	24,5842883547047	25,0187798247048	25,1160031518505	75581129355,2079	65568229581,3186	0,205029134988769	0,0161051745238539	0,0113675471581375	0,867521167528051	-0.205029134988767	1,15270962534487	0.205029134988767	1293
Q91YT2	sp|Q91YT2|RN185_MOUSE E3 ubiquitin-protein ligase RNF185 OS=Mus musculus GN=Rnf185 PE=2 SV=1;sp|Q91YT2-2|RN185_MOUSE Isoform 2 of E3 ubiquitin-protein ligase RNF185 OS=Mus musculus GN=Rnf185	−1,15	0,761064381271294	0,933389703428013	23,9964472236921	22,5429895762164	23,7584773744894	23,3205769736667	23,489949026874	23,0613359467876	15019843415,0665	13031308551,6744	0,20488781171763	0,0332694809788999	0,0092685848547494	0,867606152178831	-0.20488781171763	1,15259671394525	0.20488781171763	1294
Q09167	sp|Q09167|SRSF5_RAT Serine/arginine-rich splicing factor 5 OS=Rattus norvegicus GN=Srsf5 PE=2 SV=1;sp|O35326|SRSF5_MOUSE Serine/arginine-rich splicing factor 5 OS=Mus musculus GN=Srsf5 PE=1 SV=2;sp|Q13243|SRSF5_HUMAN Serine/arginine-rich splicing factor	−1,15	0,460869345314242	0,792808510675944	26,593052849711	26,0422096112122	26,030206424081	25,9818743454278	26,1107739160253	26,147377349823	244339126912,609	212033164794,253	0,204594967062288	0,0122627068124851	0,0033333957724594	0,867782280609071	-0.204594967062292	1,15236277848187	0.204594967062292	1295
Q60963	sp|Q60963|PAFA_MOUSE Platelet-activating factor acetylhydrolase OS=Mus musculus GN=Pla2g7 PE=2 SV=2	−1,15	0,513564208983497	0,819539762695211	24,7376841280185	25,3674906455573	25,3115808292264	24,8358716084403	25,0631252435222	25,0924879023731	82735824737,7962	71800809318,2195	0,204512048039087	0,0138670193422318	0,0056186637387191	0,867832157904613	-0.204512048039087	1,15229654823406	0.204512048039087	1296
Q68FL4	sp|Q68FL4|SAHH3_MOUSE Putative adenosylhomocysteinase 3 OS=Mus musculus GN=Ahcyl2 PE=1 SV=1;sp|Q68FL4-2|SAHH3_MOUSE Isoform 2 of Putative adenosylhomocysteinase 3 OS=Mus musculus GN=Ahcyl2	−1,15	0,554684110708904	0,839796924118652	28,8917477037687	28,4120046915943	28,1113694390224	28,4643306249264	28,198658713088	28,3270681210585	2317956925660,73	2011736012454,82	0,204412755440611	0,0138242454789963	0,0046897440324384	0,867891887974311	-0.204412755440607	1,1522172448621 8	0.204412755440607	1297
B1AVZ0	sp|B1AVZ0|UPP_MOUSE Uracil phosphoribosyltransferase homolog OS=Mus musculus GN=Uprt PE=2 SV=1	−1,15	0,781818064629329	0,940251150142017	25,404963452821	23,9894755268835	24,2532325976359	24,2965262702175	24,0084076421851	24,9183070413901	45876730244,6022	39824454076,8694	0,204107985264567	0,0306622635613765	0,0190527275858572	0,868075250013161	-0.204107985264569	1,15197386399951	0.20410798526457	1298
P97379	sp|P97379|G3BP2_MOUSE Ras GTPase-activating protein-binding protein 2 OS=Mus musculus GN=G3bp2 PE=1 SV=2;sp|P97379-2|G3BP2_MOUSE Isoform B of Ras GTPase-activating protein-binding protein 2 OS=Mus musculus GN=G3bp2	−1,15	0,423354544110785	0,7711031271846	28,7785417083211	29,1591429707384	29,1006990223782	28,6392790424527	29,0935725463537	28,8811067240288	3981957176111,16	3456644502970,72	0,204105467788048	0,00706456389337469	0,0078730326196551	0,868076764789955	-0.204105467788047	1,15197185382789	0.204105467788047	1299
Q8VHN8	sp|Q8VHN8|SDOS_MOUSE Protein syndesmos OS=Mus musculus GN=Nudt16l1 PE=1 SV=2;sp|Q8VHN8-2|SDOS_MOUSE Isoform 2 of Protein syndesmos OS=Mus musculus GN=Nudt16l1	−1,15	0,546438229182843	0,836220697939517	26,4907634434962	25,8761040310522	25,8919891587445	25,848124338265	25,809182802241	26,1785692818595	213368284419,875	185309296836,272	0,203410484233056	0,0134315086760745	0,0078226154594071	0,868495040582567	-0.203410484233059	1,15141705280116	0.203410484233059	1300
Q5NCF2	sp|Q5NCF2|TPPC1_MOUSE Trafficking protein particle complex subunit 1 OS=Mus musculus GN=Trappc1 PE=1 SV=1	−1,15	0,536984423392867	0,831016807971437	27,0207057287637	27,039238096365	26,8032741998557	26,3883602649067	26,9663839376261	27,0856899088401	508334741931,185	441515090787,018	0,203316085178287	0,00486790857475985	0,0139095805548506	0,868551870190268	-0.203316085178287	1,15134171524026	0.203316085178287	1301
Q05A62-2	sp|Q05A62-2|DNAL1_MOUSE Isoform 2 of Dynein light chain 1, axonemal OS=Mus musculus GN=Dnal1;sp|Q05A62|DNAL1_MOUSE Dynein light chain 1, axonemal OS=Mus musculus GN=Dnal1 PE=2 SV=2;sp|Q05A62-3|DNAL1_MOUSE Isoform 3 of Dynein light chain 1, axonemal OS=M	−1,15	0,513623060438792	0,819539762695211	27,210872020632	27,102742443713	26,7858549456868	26,5520528301124	26,9993754734062	27,1253677426794	549984795370,893	477707925772,91	0,203262921972988	0,00817100505995171	0,0112036713576769	0,868583876852011	-0.203262921972988	1,15129928916512	0.203262921972987	1302
Q60737	sp|Q60737|CSK21_MOUSE Casein kinase II subunit alpha OS=Mus musculus GN=Csnk2a1 PE=1 SV=2	−1,15	0,144570117755551	0,539068536314443	30,7404493890883	30,4410482207959	30,5719607650948	30,381645732265	30,5244583269409	30,4253950011429	19172270859463,6	16656689444668,9	0,202919536891324	0,00490749019278433	0,0024034381216012	0,868790638665895	-0.202919536891326	1,15102529366061	0.202919536891326	1303
O08663	sp|O08663|MAP2_MOUSE Methionine aminopeptidase 2 OS=Mus musculus GN=Metap2 PE=1 SV=1	−1,15	0,396929876552692	0,754338506152154	27,2974019823772	27,6376773679146	27,7767708216757	27,5899208736384	27,401725558271	27,2983095342163	941383768592,379	817883155812,943	0,202888226182544	0,00894503347053612	0,0053899180116697	0,868809494172496	-0.202888226182545	1,15100031331087	0.202888226182545	1304
Q99JT9	sp|Q99JT9|MTND_MOUSE 1,2-dihydroxy-3-keto-5-methylthiopentene dioxygenase OS=Mus musculus GN=Adi1 PE=1 SV=1	−1,15	0,534594595486874	0,8300431463043	25,806618885234	25,3493149492024	25,3243138240262	25,0279479753378	25,5223313428076	25,5091258895633	117936936977,04	102500659457,536	0,202382438899416	0,0106509718149483	0,0111109602417467	0,869114139173301	-0.20238243889942	1,15059686055873	0.20238243889942	1305
Q9D1H6	sp|Q9D1H6|NDUF4_MOUSE NADH dehydrogenase [ubiquinone] 1 alpha subcomplex assembly factor 4 OS=Mus musculus GN=Ndufaf4 PE=2 SV=1	−1,15	0,591731725983721	0,85975361851476	25,8132134394717	25,9945560886311	25,8503583907544	25,2615134752874	25,8471838387831	26,1288765661872	174648748011,956	151804289652,027	0,202243741971053	0,00370056855866111	0,0171860734497248	0,869197697550256	-0.202243741971051	1,1504862505025	0.202243741971051	1306
Q99KK9	sp|Q99KK9|SYHM_MOUSE Probable histidine--tRNA ligase, mitochondrial OS=Mus musculus GN=Hars2 PE=2 SV=1	−1,15	0,360597914490424	0,734744291497425	26,4634183601821	25,9834974475046	26,0564348417916	26,0184741068914	25,979047873499	26,0872422456991	231485610257,558	201338768564,464	0,20129751906884	0,00988282122216288	0,0021037249454385	0,869767966745096	-0.201297519068837	1,1497319264841	0.201297519068837	1307
Q8BWY3	sp|Q8BWY3|ERF1_MOUSE Eukaryotic peptide chain release factor subunit 1 OS=Mus musculus GN=Etf1 PE=1 SV=4	−1,15	0,284678825377377	0,704836107321858	28,9686502897513	28,7849374021746	29,0269449893171	28,9293487073306	28,5757099628488	28,8574544305787	3654002386781,18	3178734784112,34	0,20102492525331	0,00436632869658781	0,0064927012204886	0,869932322871988	-0.201024925253308	1,1495147078782	0.201024925253308	1308

Q8BW00	sp|Q8BW00|PTH_MOUSE Probable peptidyl-tRNA hydrolase OS=Mus musculus GN=Ptrh1 PE=2 SV=1	−1,15	0,175096309155618	0,590462219359101	23,4209979293226	23,3432131075201	23,3432131075201	23,0384874615479	23,3706667882325	23,2810705328774	14095781165,7249	12265728720,8137	0,200630483397912	0,00192172605203989	0,0073982095213024	0,870170200331917	-0.20063048339791	1,14920046632091	0.200630483397911	1309
Q78JE5	sp|Q78JE5|FBX22_MOUSE F-box only protein 22 OS=Mus musculus GN=Fbxo22 PE=2 SV=2	−1,15	0,581705318095637	0,853379506269256	28,0044923642566	27,7433277429496	27,9562316171464	27,2868283286901	27,9581743773589	28,0423253078252	1310396101223,76	1140448442149,16	0,20040174350932	0,00498050786551812	0,0149135852306092	0,870308177110808	-0.200401743509314	1,14901827456078	0.200401743509314	1310
Q8R574	sp|Q8R574|KPRB_MOUSE Phosphoribosyl pyrophosphate synthase-associated protein 2 OS=Mus musculus GN=Prpsap2 PE=1 SV=1	−1,15	0,246775130219231	0,67153016550665	29,9945463914196	29,6285973016842	29,6461657797128	29,5991882691668	29,643927476549	29,6094844022266	8376376102922,43	7290063574168,31	0,200394825496012	0,0069361879547289	0,0007911026318736	0,870312350423817	-0.200394825496012	1,14901276479994	0.200394825496012	1311
O88545	sp|O88545|CSN6_MOUSE COP9 signalosome complex subunit 6 OS=Mus musculus GN=Cops6 PE=1 SV=1	−1,15	0,332160603694361	0,72953198881961	30,3309155382186	29,9023714560052	30,038498203068	30,0268702489686	30,0107694072549	29,8175332931728	11699825765268,2	10182832362188,2	0,200348141337487	0,00727704044460104	0,0038893032968271	0,870340513310608	-0.200348141337486	1,14897558450565	0.200348141337486	1312
Q9DBG5	sp|Q9DBG5|PLIN3_MOUSE Perilipin-3 OS=Mus musculus GN=Plin3 PE=1 SV=1	−1,15	0,726410659545445	0,92223768495148	26,3399692238663	25,1686290907268	25,6663122517335	25,5872669040152	25,9391068700574	25,2329334459052	148666323020,672	129433845240,158	0,199862962251472	0,022852078038032	0,0137997902236788	0,870633258496346	-0.199862962251473	1,1485892483904	0.199862962251473	1313
Q71M36-2	sp|Q71M36-2|CSPG5_MOUSE Isoform 2 of Chondroitin sulfate proteoglycan 5 OS=Mus musculus GN=Cspg5;sp|Q71M36|CSPG5_MOUSE Chondroitin sulfate proteoglycan 5 OS=Mus musculus GN=Cspg5 PE=1 SV=2;sp|Q71M36-4|CSPG5_MOUSE Isoform 4 of Chondroitin sulfate proteog	−1,15	0,479566909826083	0,800971319073732	24,9780625663119	25,5614805926799	25,2687133583084	25,3227647390092	25,0836030464032	24,9865539749528	94269056997,9265	82081124647,5783	0,199733798046382	0,0115439794975842	0,0068854784573573	0,87071120961127	-0.199733798046381	1,14848642002261	0.199733798046381	1314
Q8CHX7	sp|Q8CHX7|RFTN2_MOUSE Raftlin-2 OS=Mus musculus GN=Rftn2 PE=2 SV=3;sp|Q8CHX7-2|RFTN2_MOUSE Isoform 2 of Raftlin-2 OS=Mus musculus GN=Rftn2	−1,15	0,659972626666082	0,89341619202324	23,3482940068699	22,6546864138481	22,4253348908122	22,9029360555361	22,7144546071874	22,3959226638979	8054030104,306	7013512218,1109	0,199573768528959	0,0210678605655873	0,0113038816674829	0,870807797746026	-0.199573768528959	1,14835903237014	0.199573768528959	1315
Q9ERR7	sp|Q9ERR7|SEP15_MOUSE 15 kDa selenoprotein OS=Mus musculus GN=Sep15 PE=1 SV=3	−1,15	0,413528701267009	0,766530246241387	26,3382310811099	26,2691378786882	26,2361110611353	25,8459890590547	26,1633273553353	26,4201728012214	259276269298,28	225855911209,536	0,199087493937177	0,00198281518460328	0,0110018240537135	0,871101361573912	-0.199087493937176	1,14797203185769	0.199087493937176	1316
Q923D5	sp|Q923D5|WBP11_MOUSE WW domain-binding protein 11 OS=Mus musculus GN=Wbp11 PE=1 SV=2	−1,15	0,241443642117179	0,664995565272982	25,1330874985912	25,0736508555744	24,7695581872001	24,9224630227272	24,8284747330514	24,8115612153573	71438219280,696	62233938548,3487	0,198994567500801	0,00780261343269021	0,0024039506396149	0,871157472498275	-0.198994567500801	1,14789809141192	0.198994567500801	1317
Q9CQE1	sp|Q9CQE1|NPS3B_MOUSE Protein NipSnap homolog 3B OS=Mus musculus GN=Nipsnap3b PE=1 SV=1	−1,15	0,545720672112342	0,835985869293933	27,9689280949635	28,468006362801	28,706063719518	28,3063564870261	28,3200413611814	28,102857773251	2116954028460,13	1844234140333,39	0,198968111164004	0,0132550405209396	0,0043066517468127	0,871173448048319	-0.198968111164003	1,14787704129446	0.198968111164003	1318
Q3TCJ1	sp|Q3TCJ1|F175B_MOUSE BRISC complex subunit Abro1 OS=Mus musculus GN=Fam175b PE=2 SV=1	−1,15	0,402326149129063	0,757239901094806	27,7534331423157	27,3032177671707	27,3070559481143	27,3782962363329	27,1789632879833	27,3929021595667	838238876698,161	730299500271,809	0,198873190435422	0,00942761460502819	0,0043755110466384	0,871230767950626	-0.198873190435422	1,14780152031622	0.198873190435422	1319
B2RUR8	sp|B2RUR8|OTU7B_MOUSE OTU domain-containing protein 7B OS=Mus musculus GN=Otud7b PE=1 SV=1	−1,15	0,296277378933323	0,711969538636193	24,4753092267965	24,1307029648063	24,0770833167058	24,1736455229619	24,0390384667258	24,0578211291045	33262567506,9552	28988596716,5053	0,198414036291315	0,00891981615107306	0,0030261629615145	0,871508091203236	-0.198414036291313	1,14743627752138	0.198414036291313	1320
Q9CQ80	sp|Q9CQ80|VPS25_MOUSE Vacuolar protein-sorting-associated protein 25 OS=Mus musculus GN=Vps25 PE=2 SV=1;sp|Q9CQ80-2|VPS25_MOUSE Isoform 2 of Vacuolar protein-sorting-associated protein 25 OS=Mus musculus GN=Vps25	−1,15	0,212615495647786	0,644663817465132	27,5924349407401	27,4494037111634	27,3661184166555	27,1570050725175	27,4410098735373	27,3974954083987	850694594605,361	741422729562,983	0,198344943023634	0,00416701595861091	0,0055965488396297	0,871549830297124	-0.198344943023634	1,1473813260443	0.198344943023634	1321
P80560	sp|P80560|PTPR2_MOUSE Receptor-type tyrosine-protein phosphatase N2 OS=Mus musculus GN=Ptprn2 PE=1 SV=2	−1,15	0,251313474539647	0,673538059877323	24,8358716084403	25,1035425680548	25,1963348706422	25,0100063220039	24,8684973230597	24,845323645973	75337938964,5288	65672258640,0385	0,198092491586906	0,0074732070556701	0,0035790244353476	0,871702352661371	-0.198092491586904	1,14718056793919	0.198092491586904	1322
O88544	sp|O88544|CSN4_MOUSE COP9 signalosome complex subunit 4 OS=Mus musculus GN=Cops4 PE=1 SV=1	−1,15	0,240941466352537	0,664601444032504	31,0083247513143	30,6173890438023	30,8944000450301	30,7106727634419	30,7235744445341	30,6742077219904	24754736161963,3	21580652464553,1	0,197966089418249	0,00651936551013929	0,0008339126815911	0,87177873047634	-0.197966089418248	1,14708006176476	0.197966089418248	1323
Q9D1P0	sp|Q9D1P0|RM13_MOUSE 39S ribosomal protein L13, mitochondrial OS=Mus musculus GN=Mrpl13 PE=2 SV=1	−1,15	0,33565435754778	0,72953198881961	24,8877355888685	24,4526054481224	24,5360198544844	24,5823748158982	24,4132109090808	24,4697558524359	49510649782,0222	43171534546,2727	0,197658508701559	0,0093781080753314	0,0035166363678404	0,871964612388277	-0.197658508701561	1,14683553184692	0.197658508701561	1324
Q9CR09	sp|Q9CR09|UFC1_MOUSE Ubiquitin-fold modifier-conjugating enzyme 1 OS=Mus musculus GN=Ufc1 PE=2 SV=1	−1,15	0,383457989121031	0,751193704195234	28,520313058957	28,4205387528905	28,1742570180425	28,0252316570059	28,2437154901872	28,4357489471046	2097365067988,94	1829197931850,81	0,197366806118903	0,00627815198053626	0,0072747070438511	0,872140935199582	-0.197366806118899	1,14660367337437	0.197366806118898	1325
P45878	sp|P45878|FKBP2_MOUSE Peptidyl-prolyl cis-trans isomerase FKBP2 OS=Mus musculus GN=Fkbp2 PE=1 SV=1	−1,15	0,352236373951586	0,732843629012038	29,4486387307347	29,383338090787	29,2163908422627	28,991263159107	29,209851847461	29,4384942443743	5575794241263,02	4865560733685,95	0,196571245042957	0,00408095612574129	0,0076552500956845	0,872622002024201	-0.196571245042956	1,14597156349522	0.196571245042956	1326
Q9Z1L5	sp|Q9Z1L5|CA2D3_MOUSE Voltage-dependent calcium channel subunit alpha-2/delta-3 OS=Mus musculus GN=Cacna2d3 PE=1 SV=1	−1,15	0,723529526132732	0,921631098094813	21,9376699279833	21,0988721291394	21,0533396385392	20,7310314969917	21,5846616401495	21,3656840009079	1896534098,35387	1655097427,59969	0,196449166482867	0,02330843494595	0,0208865794191398	0,872695845034508	-0.196449166482862	1,14587459730653	0.196449166482862	1327

P10711	sp|P10711|TCEA1_MOUSE Transcription elongation factor A protein 1 OS=Mus musculus GN=Tcea1 PE=1 SV=2;sp|P10711-2|TCEA1_MOUSE Isoform 1 of Transcription elongation factor A protein 1 OS=Mus musculus GN=Tcea1	−1,15	0,394499251904378	0,753881747531964	28,4258373734941	27,9723912373162	28,0108060548788	27,8935880061131	28,1229950257761	27,9843470437695	1657522093887,35	1446705509966,55	0,196256822733624	0,00893657426115077	0,0041261535443481	0,872812202806675	-0.196256822733626	1,14572183659249	0.196256822733626	1328
P18872	sp|P18872|GNAO_MOUSE Guanine nucleotide-binding protein G(o) subunit alpha OS=Mus musculus GN=Gnao1 PE=1 SV=3	−1,15	0,405833011683112	0,760245992583427	34,6457890411816	34,1391253660482	34,2337174910887	34,1068660507989	34,2285718437873	34,2755682575707	819359428466590	715261064664687	0,196026547508634	0,00784518778357285	0,0025454735222047	0,872951527516172	-0.196026547508634	1,14553897722743	0.196026547508634	1329
Q91Z38	sp|Q91Z38|TTC1_MOUSE Tetratricopeptide repeat protein 1 OS=Mus musculus GN=Ttc1 PE=2 SV=1	−1,15	0,273835450510039	0,695545109045926	27,4149626112445	27,1570050725175	27,1786552140376	27,067317939297	27,2928682782776	26,9837240922818	683305325569,89	596674019339,27	0,195587411230907	0,00525104434500377	0,0058976251021522	0,873217282247335	-0.195587411230905	1,14519034417914	0.195587411230905	1330
Q923G2	sp|Q923G2|RPAB3_MOUSE DNA-directed RNA polymerases I, II, and III subunit RPABC3 OS=Mus musculus GN=Polr2h PE=2 SV=3	−1,15	0,614208573709632	0,868502403759507	27,1900125023832	27,0009567310953	26,7786940840882	26,4722972144417	26,7912227971815	27,2998217180003	526695159168,1	459979262185,472	0,195399379977294	0,00762813452934716	0,0155419091557469	0,873331098983321	-0.195399379977292	1,14504109743045	0.195399379977292	1331
O88685	sp|O88685|PRS6A_MOUSE 26S protease regulatory subunit 6A OS=Mus musculus GN=Psmc3 PE=1 SV=2	−1,14	0,217068891663832	0,64735591278721	30,3642246304452	30,0981104206294	30,3808159827936	30,0338596200803	30,1644509291729	30,239302803755	14154452355488,8	12364753948438,2	0,195022400356737	0,00523917296104697	0,0034490358891617	0,873559332279176	-0.195022400356732	1,14474193457579	0.195022400356732	1332
Q5FWK3	sp|Q5FWK3|RHG01_MOUSE Rho GTPase-activating protein 1 OS=Mus musculus GN=Arhgap1 PE=1 SV=1	−1,14	0,325834901630421	0,726927682919497	31,246940384094	30,8944000450301	31,1804486408323	30,8138966768348	31,1032923983988	30,9997610762329	32337964365892,4	28255711134890	0,194686366688082	0,00602170116404892	0,0047344488485550	0,873762826107012	-0.194686366688081	1,14447533142996	0.194686366688081	1333
Q80YV4-2	sp|Q80YV4-2|PANK4_MOUSE Isoform 2 of Pantothenate kinase 4 OS=Mus musculus GN=Pank4;sp|Q80YV4|PANK4_MOUSE Pantothenate kinase 4 OS=Mus musculus GN=Pank4 PE=1 SV=2;sp|Q80YV4-3|PANK4_MOUSE Isoform 3 of Pantothenate kinase 4 OS=Mus musculus GN=Pank4	−1,14	0,348090399686307	0,732843629012038	27,8990401696981	27,6889296233201	27,4606749995793	27,4793096785935	27,683736916332	27,4813946984528	1053228787471,06	920467102077,336	0,194380794611198	0,00791988210457884	0,0042626770730287	0,873947914286978	-0.194380794611203	1,14423294987306	0.194380794611203	1334
P63158	sp|P63158|HMGB1_MOUSE High mobility group protein B1 OS=Mus musculus GN=Hmgb1 PE=1 SV=2	−1,14	0,549812335197146	0,838418252843802	30,192477358805	30,8286913985065	30,8709415115779	30,5898070211484	30,4225096278016	30,4762511723909	20079156902898,5	17552004463806,1	0,194062895955453	0,0124091957504478	0,0028007511712054	0,874140510415176	-0.194062895955456	1,14398084528201	0.194062895955457	1335
P97493	sp|P97493|THIOM_MOUSE Thioredoxin, mitochondrial OS=Mus musculus GN=Txn2 PE=1 SV=1	−1,14	0,277432340859588	0,699203439845458	28,2518043492866	28,1148719832427	27,9977571851328	27,7938677088192	28,0423253078252	28,1252116374367	1633061907066,56	1427769974651,05	0,193815914274495	0,00452153844881946	0,0061610017784914	0,874290171409195	-0.193815914274503	1,14378501863767	0.193815914274503	1336
Q9D1R9	sp|Q9D1R9|RL34_MOUSE 60S ribosomal protein L34 OS=Mus musculus GN=Rpl34 PE=1 SV=2	−1,14	0,518941641158859	0,82335612354586	26,2458051503349	25,8539662924407	25,8175097401103	25,5562998340598	25,8617916340518	26,0967000396117	190406493066,452	166500447809,153	0,193556619455393	0,00914250553769617	0,0104870542224877	0,874447321242581	-0.193556619455395	1,14357946523183	0.193556619455395	1337
Q9EQU5-2	sp|Q9EQU5-2|SET_MOUSE Isoform 2 of Protein SET OS=Mus musculus GN=Set;sp|Q9EQU5|SET_MOUSE Protein SET OS=Mus musculus GN=Set PE=1 SV=1	−1,14	0,0789993826504785	0,418825847815587	30,049400664825	29,8175332931728	29,9548618864297	29,8306347317633	29,8531115187151	29,7364644042824	10070169098372	8808487708686,4	0,193121653875564	0,00389403972304933	0,0020762666028986	0,87471100262958 1	-0.193121653875562	1,14323473352201	0.193121653875562	1338
Q6DFY8	sp|Q6DFY8|BRNP2_MOUSE BMP/retinoic acid-inducible neural-specific protein 2 OS=Mus musculus GN=Brinp2 PE=2 SV=1	−1,14	0,737782876022495	0,924869192631503	24,9939508693433	24,5893142582617	24,34213713151	24,2198565302694	25,2075881976355	24,0965663332202	50326657962,5983	44024128124,8991	0,193028363598854	0,013353789072788 7	0,0248484550999523	0,8747675666 7643	-0.193028363598857	1,14316080990448	0.193028363598857	1339
P23198	sp|P23198|CBX3_MOUSE Chromobox protein homolog 3 OS=Mus musculus GN=Cbx3 PE=1 SV=2	−1,14	0,326329590354749	0,726927682919497	28,8859884835841	28,7527330443723	28,6073269178441	28,4120046915943	28,6463208267188	28,787551864115	3057696024929,47	2675861393787,07	0,19244160287818	0,0048480456673371	0,0066288525864782	0,87512341709925	-0.192441602878176	1,14269596774667	0.192441602878176	1340
Q9DA03	sp|Q9DA03|LYRM7_MOUSE Complex III assembly factor LYRM7 OS=Mus musculus GN=Lyrm7 PE=2 SV=2	−1,14	0,566235604013699	0,845163895632417	24,1219424658587	23,9127580426146	23,6274438234	23,4856512423187	23,6723843197498	24,1039857732243	23667803135,055	20712580614,5571	0,19241848763741	0,010391432158 5634	0,0133512314886896	0,875137438669969	-0.192418487637409	1,14267765931691	0.192418487637409	1341
P62317	sp|P62317|SMD2_MOUSE Small nuclear ribonucleoprotein Sm D2 OS=Mus musculus GN=Snrpd2 PE=2 SV=1	−1,14	0,490803079706278	0,807151440380692	28,6537286050758	28,6868977896118	28,0992599920198	28,2856963956054	28,3939473383592	28,3607504246018	2337170507703,81	2045775503504,56	0,192115152137556	0,0115911172679031	0,0019564045687359	0,875321461040711	-0.192115152137553	1,14243742957136	0.192115152137553	1342
Q7TN22-2	sp|Q7TN22-2|TXD16_MOUSE Isoform 2 of Thioredoxin domain-containing protein 16 OS=Mus musculus GN=Txndc16;sp|Q7TN22|TXD16_MOUSE Thioredoxin domain-containing protein 16 OS=Mus musculus GN=Txndc16 PE=2 SV=1	−1,14	0,552667011018536	0,838891344884342	22,6960719862823	21,9810470764094	22,1831864358735	22,1382342375959	22,2806686370197	22,0428427182337	4775519016,47358	4181413556,03558	0,19166679982449	0,0165387255744244	0,0054024710696563	0,875593530590376	-0.191666799824491	1,14208244472266	0.191666799824491	1343
Q9D1L0	sp|Q9D1L0|CHCH2_MOUSE Coiled-coil-helix-coiled-coil-helix domain-containing protein 2, mitochondrial OS=Mus musculus GN=Chchd2 PE=2 SV=1	−1,14	0,188603496813735	0,613397297160539	27,102742443713	26,8740206459646	26,8215162251178	26,7655997645261	26,7315571743892	26,9028762084041	497449445650,34	435609069766,125	0,191515923623584	0,00555204162003 08	0,0033841552497445	0,875685104436355	-0.191515923623585	1,14196301265586	0.191515923623585	1344

P17710-3	sp|P17710-3|HXK1_MOUSE Isoform HK1 of Hexokinase-1 OS=Mus musculus GN=Hk1;sp|P17710-4|HXK1_MOUSE Isoform HK1-SC of Hexokinase-1 OS=Mus musculus GN=Hk1;sp|P17710|HXK1_MOUSE Hexokinase-1 OS=Mus musculus GN=Hk1 PE=1 SV=3;sp|P17710-2|HXK1_MOUSE Isoform HK1	−1,14	0,704775151899303	0,915824338525891	33,1377845162753	32,1359937740807	32,704254865978	32,7488011970875	32,6953467176655	32,1359937740807	152676800110986	133712608001915	0,191345348991559	0,0153831352325925	0,01043534458108	0,875788645719027	-0.191345348991566	1,14182800255305	0.191345348991566	1345
Q91YJ3	sp|Q91YJ3|THYN1_MOUSE Thymocyte nuclear protein 1 OS=Mus musculus GN=Thyn1 PE=1 SV=1	−1,14	0,0016370684628848	0,0333213172781798	24,5643854343456	24,6110501334689	24,53802616775	24,5094875445525	24,389762517735	24,4164001122609	46893922128,3508	41070258465,3269	0,191306922095384	0,00150499685257186	0,0025722001881282	0,875811973093565	-0.191306922095385	1,14179758980432	0.191306922095385	1346
Q6TEK5	sp|Q6TEK5|VKORL_MOUSE Vitamin K epoxide reductase complex subunit 1-like protein 1 OS=Mus musculus GN=Vkorc1l1 PE=1 SV=1	−1,14	0,42223168581083	0,7711031271846	24,5452995178382	24,5500906114161	24,3708201043853	24,1307029648063	24,6502909577635	24,2886600925541	43184036763,6117	37836925899,0107	0,190703229962047	0,00417117881547433	0,0109360352999634	0,876178531111602	-0.190703229962047	1,14131990740666	0.190703229962047	1347
P63085	sp|P63085|MK01_MOUSE Mitogen-activated protein kinase 1 OS=Mus musculus GN=Mapk1 PE=1 SV=3	−1,14	0,338044526784291	0,729620681454069	33,0476252564011	32,5913113535174	32,9187789512132	32,6728334389707	32,7488011970875	32,7396907713201	185221190975821	162295814663241	0,190623369692463	0,00716081546807907	0,0012677373253540	0,876227033247115	-0.190623369692462	1,14125673148226	0.190623369692462	1348
Q9D009	sp|Q9D009|LIPT2_MOUSE Putative lipoyltransferase 2, mitochondrial OS=Mus musculus GN=Lipt2 PE=1 SV=2	−1,14	0,465170203873529	0,795257083006383	24,8203758830295	24,4368206009609	24,4296234518414	24,2081894088514	24,4377243018323	24,6453225574227	46479319651,8354	40737386271,5978	0,190235531894715	0,00910145188634061	0,0089502498511650	0,876462619865158	-0.190235531894716	1,1409499701811	0.190235531894716	1349
Q9R0X0	sp|Q9R0X0|MED20_MOUSE Mediator of RNA polymerase II transcription subunit 20 OS=Mus musculus GN=Med20 PE=1 SV=1;sp|Q9R0X0-2|MED20_MOUSE Isoform 2 of Mediator of RNA polymerase II transcription subunit 20 OS=Mus musculus GN=Med20	−1,14	0,563081125372628	0,845110794140252	23,5452376736717	23,252854447264	23,1051187112	22,7962312002305	23,3326444849489	23,3797067172341	13168194841,7199	11545106072,6948	0,189776159518048	0,00961269357919533	0,0139898972099505	0,876741741101614	-0.189776159518045	1,14058673508976	0.189776159518045	1350
P63216	sp|P63216|GBG3_MOUSE Guanine nucleotide-binding protein G(I)/G(S)/G(O) subunit gamma-3 OS=Mus musculus GN=Gng3 PE=1 SV=1	−1,14	0,608514616369921	0,866608542040832	28,5775854342843	28,1132288221562	27,9018726638348	27,7534331423157	28,1567892511131	28,2885021532674	1762161518165,15	1545303605775,28	0,189455854219835	0,012258516931043	0,0099332869358048	0,876936415785726	-0.189455854219829	1,1403335315982	0.189455854219829	1351
Q2YDW2	sp|Q2YDW2|MSTO1_MOUSE Protein misato homolog 1 OS=Mus musculus GN=Msto1 PE=2 SV=1;sp|Q2YDW2-2|MSTO1_MOUSE Isoform 2 of Protein misato homolog 1 OS=Mus musculus GN=Msto1	−1,14	0,521189900429947	0,823916573667872	24,0297670368369	24,5615634697321	24,558208524557	24,3880879517679	24,3182133554805	24,0493524445907	38857994725,9864	34076866319,0048	0,189418779702157	0,0125524613847899	0,0073744244183927	0,876958951672712	-0.189418779702156	1,14030422757257	0.189418779702156	1352
Q8BU11	sp|Q8BU11|TOX4_MOUSE TOX high mobility group box family member 4 OS=Mus musculus GN=Tox4 PE=1 SV=3	−1,14	0,43588944471668	0,778450481049573	24,9641191392659	24,7005673453217	24,4468590871809	24,6992019327018	24,5863610385453	24,4331596823421	53548226993,2794	46976229302,5333	0,188907892001595	0,0104698519200466	0,0054340495505435	0,877269555692835	-0.188907892001593	1,13990049410781	0.188907892001593	1353
Q61686	sp|Q61686|CBX5_MOUSE Chromobox protein homolog 5 OS=Mus musculus GN=Cbx5 PE=1 SV=1	−1,14	0,735763753860275	0,924701393764781	25,4898256936456	25,4600043280656	25,5091258895633	26,102075650852	24,8100392117994	25,1555905514942	117102872444,529	102784644343,052	0,188151717317681	0,000971025260953641	0,0263806164184755	0,877729488589108	-0.188151717317684	1,13930318281483	0.188151717317684	1354
Q60780	sp|Q60780|GAS7_MOUSE Growth arrest-specific protein 7 OS=Mus musculus GN=Gas7 PE=1 SV=1;sp|Q60780-2|GAS7_MOUSE Isoform 2 of Growth arrest-specific protein 7 OS=Mus musculus GN=Gas7	−1,14	0,527412380274447	0,828347679607515	29,5039457329552	29,2130685094284	29,4939123683025	29,3536338319982	29,541802057065	28,9252452807151	5886261566832,99	5168276534247,46	0,187668387442346	0,00561555899638648	0,0107940958286464	0,87802359367937	-0.187668387442345	1,13892155882678	0.187668387442345	1355
P28652	sp|P28652|KCC2B_MOUSE Calcium/calmodulin-dependent protein kinase type II subunit beta OS=Mus musculus GN=Camk2b PE=1 SV=2	−1,14	0,00628521225195623	0,0888740760486895	34,698424438377	34,698424438377	34,7975818162301	34,5341448386175	34,6457890411816	34,6244837935552	1212519592850250	1064703293692480	0,187556616434075	0,00164831858329371	0,0017130089001178	0,878091620102979	-0.187556616434078	1,13883332571005	0.187556616434078	1356
P27601	sp|P27601|GNA13_MOUSE Guanine nucleotide-binding protein subunit alpha-13 OS=Mus musculus GN=Gna13 PE=1 SV=1;sp|Q14344|GNA13_HUMAN Guanine nucleotide-binding protein subunit alpha-13 OS=Homo sapiens GN=GNA13 PE=1 SV=2;sp|Q14344-2|GNA13_HUMAN Isoform 2 o	−1,14	0,397193565519477	0,754338506152154	27,081221549861	26,642274809204	26,8740206459646	26,5794042509612	26,8777286255423	26,7506588434913	465249194749,617	408570603922,546	0,187418245342986	0,00817349182359049	0,0055994514366628	0,878175843254122	-0.187418245342988	1,13872410369938	0.187418245342988	1357
Q9CWK3	sp|Q9CWK3|CD2B2_MOUSE CD2 antigen cytoplasmic tail-binding protein 2 OS=Mus musculus GN=Cd2bp2 PE=1 SV=1	−1,14	0,506568114920108	0,815694818794863	24,7625063508653	24,2127444651752	24,8203758830295	24,4925993676552	24,4958624117327	24,4180530212713	48196017640,5047	42333233094,4483	0,187123268729353	0,0136333784872869	0,0017986781090782	0,87835541538334	-0.187123268729353	1,1384913014552	0.187123268729353	1358
Q0KL01	sp|Q0KL01|UBX2B_MOUSE UBX domain-containing protein 2B OS=Mus musculus GN=Ubxn2b PE=1 SV=2	−1,14	0,565874444909538	0,845163895632417	25,1293167074687	24,690050667465	24,5893142582617	24,7695581872001	24,8771377433071	24,3731216083975	59123474238,3577	51935713557,6305	0,187004100137671	0,0115769784186248	0,0107584191498416	0,878427971743515	-0.18700410013767	1,13839726439401	0.18700410013767	1359
Q810J8	sp|Q810J8|ZFYV1_MOUSE Zinc finger FYVE domain-containing protein 1 OS=Mus musculus GN=Zfyve1 PE=2 SV=2	−1,14	0,284915915235235	0,704836107321858	27,0100387989245	26,6482374292033	26,7995725862334	26,7376285098563	26,7618369074011	26,5703771561402	444085495431,67	390208685687,411	0,186591559890704	0,00677513420459876	0,0039061902788498	0,878679195113345	-0.186591559890705	1,13807178497154	0.186591559890705	1360
B1AR13	sp|B1AR13|CISD3_MOUSE CDGSH iron-sulfur domain-containing protein 3, mitochondrial OS=Mus musculus GN=Cisd3 PE=1 SV=1	−1,14	0,546848098499355	0,8363017247603	25,9437232703096	25,6392323739276	25,3600660664926	25,2553298211587	25,6245416244004	25,6757304025179	137607862844,264	120936802094,497	0,186309571530379	0,0113819349490758	0,0089885215581792	0,878850958039845	-0.186309571530376	1,13784935983954	0.186309571530376	1361
Q9Z140	sp|Q9Z140|CPNE6_MOUSE Copine-6 OS=Mus musculus GN=Cpne6 PE=2 SV=1	−1,14	0,344788758077602	0,730213633407042	31,3310729947621	31,7073332672964	31,7608767264179	31,5082584125245	31,4444699733367	31,4594116268809	52917806034325,7	46511157109529,3	0,186176417068899	0,00741226030808774	0,0010601037200169	0,878932075894442	-0.1861764170689	1,13774434615138	0.1861764170689	1362

Q9CWN7	sp|Q9CWN7|CNO11_MOUSE CCR4-NOT transcription complex subunit 11 OS=Mus musculus GN=Cnot11 PE=2 SV=1	−1,14	0,179940935997906	0,598356502628386	25,2267225649949	25,1160031518505	25,3191904935594	25,0061922723973	25,2432823524486	25,0264181578287	89780957643,603	78940817326,1326	0,18563802828442	0,004033609200099	0,0052381290445072	0,879260139321506	-0.185638028284421	1,13731983889508	0.185638028284421	1363
Q9CQW1	sp|Q9CQW1|YKT6_MOUSE Synaptobrevin homolog YKT6 OS=Mus musculus GN=Ykt6 PE=2 SV=1	−1,14	0,571378105467169	0,848098502523048	30,4045709486719	30,0350880404903	29,9877542613183	29,7200994190062	29,9652839885513	30,3560902036496	12322784751220,3	10835236055529,9	0,185597734554111	0,00757123178736714	0,0106871391154016	0,879284696947815	-0.1855977345541	1,13728807458064	0.1855977345541	1364
Q3UYV9	sp|Q3UYV9|NCBP1_MOUSE Nuclear cap-binding protein subunit 1 OS=Mus musculus GN=Ncbp1 PE=1 SV=2	−1,14	0,478620386103988	0,799905345588749	26,8888307780833	26,4605063694145	26,308372107962	26,4697443055486	26,4815901695906	26,3210361882821	340121842896,21	299123854741,953	0,185308691932341	0,0113353029614086	0,0033860172157639	0,879460878474754	-0.185308691932345	1,1370602427868	0.185308691932345	1365
P97807-2	sp|P97807-2|FUMH_MOUSE Isoform Cytoplasmic of Fumarate hydratase, mitochondrial OS=Mus musculus GN=Fh;sp|P97807|FUMH_MOUSE Fumarate hydratase, mitochondrial OS=Mus musculus GN=Fh PE=1 SV=3	−1,14	0,0814769570981594	0,425084952032811	32,8687527944652	32,6477383197789	32,839500065196	32,6477383197789	32,6186624488613	32,704254865978	173176148727182	152301802589259	0,185307226530971	0,00366177639838603	0,0013327404281813	0,879461771777775	-0.185307226530965	1,13705908783115	0.185307226530965	1366
Q8VHI6	sp|Q8VHI6|WASF3_MOUSE Wiskott-Aldrich syndrome protein family member 3 OS=Mus musculus GN=Wasf3 PE=2 SV=1;sp|Q9UPY6|WASF3_HUMAN Wiskott-Aldrich syndrome protein family member 3 OS=Homo sapiens GN=WASF3 PE=1 SV=2;sp|Q9UPY6-2|WASF3_HUMAN Isoform 2 of Wisk	−1,14	0,51480726356104	0,819610722309211	28,468006362801	29,089228896999	28,8685229978762	28,5934970858939	28,6150528659003	28,8320015068502	3246459344325,64	2855259418545,97	0,18524531289325	0,0109314124287422	0,0045996564454971	0,879499514921253	-0.185245312893248	1,13701029168792	0.185245312893248	1367
Q99J09	sp|Q99J09|MEP50_MOUSE Methylosome protein 50 OS=Mus musculus GN=Wdr77 PE=1 SV=1	−1,14	0,264305694479942	0,68394025335819	27,541410950126	27,2212697752775	27,3878476077223	27,275090612165	27,3623338424334	27,1281225775127	780741108296,314	686713041562,151	0,185136871269674	0,00584712017316264	0,0043429538964073	0,879565625871366	-0.185136871269673	1,13692483037786	0.185136871269673	1368
P70362	sp|P70362|UFD1_MOUSE Ubiquitin fusion degradation protein 1 homolog OS=Mus musculus GN=Ufd1l PE=1 SV=2	−1,14	0,316892361715374	0,722907157640623	28,3456414045074	27,9902351106764	28,3498300207127	28,1238312869463	28,0091322344659	28,1677723504312	1817655946272,02	1598753358617,6	0,185131755972366	0,00731222665831456	0,0029148446894210	0,879568744512191	-0.185131755972366	1,13692079924304	0.185131755972366	1369
Q3UHK1	sp|Q3UHK1|MYCT_MOUSE Proton myo-inositol cotransporter OS=Mus musculus GN=Slc2a13 PE=2 SV=2	−1,14	0,719498464735036	0,920149210230605	24,0804450555623	24,0144583554802	23,2474511581564	23,7695776688911	23,1968846809395	23,9912209416627	21274709491,7262	18714437039,9625	0,184987781572775	0,0194719154964832	0,0173297466431901	0,879656525850123	-0.184987781572777	1,13680734538242	0.184987781572777	1370
P51863	sp|P51863|VA0D1_MOUSE V-type proton ATPase subunit d 1 OS=Mus musculus GN=Atp6v0d1 PE=1 SV=2	−1,14	0,306943394602843	0,71837258919956	32,8606940502048	32,4366499180038	32,6101813602848	32,5208763535181	32,5416809718635	32,4603983928778	149130335760793	131187918826874	0,184938889853736	0,00653218901710795	0,0012988988260326	0,879686337173552	-0.184938889853736	1,13676882059237	0.184938889853736	1371
Q9ER88-2	sp|Q9ER88-2|RT29_MOUSE Isoform 2 of 28S ribosomal protein S29, mitochondrial OS=Mus musculus GN=Dap3;sp|Q9ER88|RT29_MOUSE 28S ribosomal protein S29, mitochondrial OS=Mus musculus GN=Dap3 PE=2 SV=1	−1,14	0,65735413634887	0,892285512053452	26,8522659694571	25,9086542006991	26,6381041414573	26,3698715068758	26,385525166963	26,2599380141522	312022593501,976	274562537701,443	0,184515705746873	0,01869164721997	0,0025984062659909	0,879944412421866	-0.184515705746873	1,13643542237822	0.184515705746873	1372
Q8VEK3	sp|Q8VEK3|HNRPU_MOUSE Heterogeneous nuclear ribonucleoprotein U OS=Mus musculus GN=Hnrnpu PE=1 SV=1	−1,14	0,534361324374547	0,8300431463043	27,6192585353685	27,2596824498093	26,9594848677855	27,1273945621664	27,2944083728934	27,0337631233805	703599602122,257	619299819116,752	0,18411664230469	0,0121091722071163	0,0048627645644896	0,880187847248303	-0.184116642304691	1,13612111678917	0.184116642304691	1373
Q9CWM4	sp|Q9CWM4|PFD1_MOUSE Prefoldin subunit 1 OS=Mus musculus GN=Pfdn1 PE=2 SV=1	−1,14	0,473949746816361	0,799218469891531	28,187447373342	27,9786139581145	27,7199698313342	27,6583684625279	27,791225622099	28,0535845608097	1392344854690,71	1225527993065,22	0,184113142726239	0,00837495717946353	0,0072253472125796	0,880189982342739	-0.184113142726244	1,13611836087747	0.184113142726244	1374
Q9JK81	sp|Q9JK81|MYG1_MOUSE UPF0160 protein MYG1, mitochondrial OS=Mus musculus GN=Myg1 PE=1 SV=1	−1,14	0,342799406578064	0,729891123744941	27,7229066479733	27,4627343947558	27,5836630656943	27,2435367500068	27,5334727094064	27,6095679757196	959586247331,281	844653632900,202	0,184052624524121	0,00471890602548897	0,0070331593578058	0,880226905345174	-0.184052624524121	1,13607070395997	0.184052624524121	1375
Q8CB27	sp|Q8CB27|OTU1_MOUSE Ubiquitin thioesterase OTU1 OS=Mus musculus GN=Yod1 PE=1 SV=1	−1,14	0,864216827582478	0,967731774645151	24,4941949562678	22,383010120132	24,2257383334506	22,9239850676747	24,0187780694206	23,779469321038	19642887228,2551	17301819149,0194	0,183083267351453	0,0484900563507222	0,0244154312082883	0,880818534870565	-0.183083267351449	1,13530762627168	0.183083267351449	1376
Q9Z0Y1	sp|Q9Z0Y1|DCTN3_MOUSE Dynactin subunit 3 OS=Mus musculus GN=Dctn3 PE=2 SV=2	−1,13	0,642083262870932	0,887729494417046	30,0650549662063	29,5295487853568	29,6357657449474	29,183067170165	29,8014346095597	29,8660763732758	8268353390873,78	7285151739080,83	0,18264102928475	0,00953254235901088	0,0127313588130042	0,881088578908811	-0.182641029284751	1,13495966686852	0.182641029284751	1377
Q6PIC6	sp|Q6PIC6|AT1A3_MOUSE Sodium/potassium-transporting ATPase subunit alpha-3 OS=Mus musculus GN=Atp1a3 PE=1 SV=1	−1,13	0,595960233292527	0,860936823333794	36,0203651980554	35,3817684358028	35,2909566904371	35,3817684358028	35,5682395086685	35,3634810211889	2788732095603380	2457275609211500	0,182549665872558	0,0111772244841933	0,0031973681505446	0,881144378510061	-0.182549665872559	1,13488779408763	0.182549665872559	1378
P56382	sp|P56382|ATP5E_MOUSE ATP synthase subunit epsilon, mitochondrial OS=Mus musculus GN=Atp5e PE=1 SV=2	−1,13	0,749112713775668	0,928854093158237	27,9533352167808	27,4118429955384	27,1601478692373	26,7498127118549	27,6999254581679	27,6980271242862	884635937432,341	780022366669,604	0,181568358465306	0,0147344203891389	0,0200127417257363	0,881743928393438	-0.181568358465306	1,13411611670752	0.181568358465306	1379
Q9CWL8	sp|Q9CWL8|CTBL1_MOUSE Beta-catenin-like protein 1 OS=Mus musculus GN=Ctnnbl1 PE=2 SV=1	−1,13	0,605458169602344	0,865294279541889	25,8273556009734	26,4519728460025	26,417345126627	26,275921090209	25,8448215763115	26,2003292010332	246893899481,724	217839405415,099	0,180626239555522	0,013382547460836	0,0088174721548778	0,882319919092307	-0.180626239555526	1,13337574995332	0.180626239555526	1380

P16330	sp|P16330|CN37_MOUSE 2,3-cyclic-nucleotide 3-phosphodiesterase OS=Mus musculus GN=Cnp PE=1 SV=3;sp|P16330-2|CN37_MOUSE Isoform CNPI of 2,3-cyclic-nucleotide 3-phosphodiesterase OS=Mus musculus GN=Cnp	−1,13	0,332615805104087	0,72953198881961	32,2785243609912	31,9593241419042	31,9593241419042	32,0369458698543	31,9879882223648	31,7966976397976	84327084375913,7	74404974022368,2	0,180597004174276	0,00574726867791783	0,0039747846661388	0,882337798976724	-0.180597004174277	1,13335278298145	0.180597004174277	1381
Q9QXA1	sp|Q9QXA1|CYHR1_MOUSE Cysteine and histidine-rich protein 1 OS=Mus musculus GN=Cyhr1 PE=1 SV=1	−1,13	0,366970085116944	0,739315231400715	24,6180184843432	24,1736455229619	24,5032761075892	24,2586916472883	24,3832569119608	24,2775725239655	40787712344,312	35990002472,5444	0,180538391753173	0,00944199854552325	0,0027619937521364	0,88237364647305	-0.180538391753173	1,13330673915423	0.180538391753173	1382
Q61584-5	sp|Q61584-5|FXR1_MOUSE Isoform D of Fragile X mental retardation syndrome-related protein 1 OS=Mus musculus GN=Fxr1;sp|Q61584-6|FXR1_MOUSE Isoform F of Fragile X mental retardation syndrome-related protein 1 OS=Mus musculus GN=Fxr1;sp|Q61584-4|FXR1_MOUS	−1,13	0,521716348282165	0,823948365557954	27,6623996166401	28,1925735549002	27,5987969361743	27,791225622099	27,6192585353685	27,6683860242425	1205509444732,35	1063893839789,95	0,180288754692136	0,0117194709227037	0,0031985540857758	0,882526341405942	-0.180288754692132	1,1331106541329	0.180288754692132	1383
Q9CQI6	sp|Q9CQI6|COTL1_MOUSE Coactosin-like protein OS=Mus musculus GN=Cotl1 PE=1 SV=3	−1,13	0,554147438002065	0,839722577101535	30,7092073014578	31,0631349310244	30,9766739283493	30,4762511723909	31,1056636057856	30,7933119851167	26717468551695,9	23587599723628,1	0,179754703388459	0,00596826127523508	0,0102205698599215	0,88285309208798	-0.17975470338846	1,13269128121301	0.17975470338846	1384
P62754	sp|P62754|RS6_MOUSE 40S ribosomal protein S6 OS=Mus musculus GN=Rps6 PE=1 SV=1	−1,13	0,577184009535177	0,850873081935748	28,1463259904393	28,6581410656887	28,8185754978522	28,5833286268197	28,4070567271281	28,2591635841223	2484305313938,05	2193492882405,32	0,17961246249242	0,0123014788196406	0,0057110949272323	0,88294014028745	-0.179612462492422	1,13257961029435	0.179612462492422	1385
Q9D8S9	sp|Q9D8S9|BOLA1_MOUSE BolA-like protein 1 OS=Mus musculus GN=Bola1 PE=1 SV=1	−1,13	0,467213355287527	0,796164546189264	26,4269514236666	26,2087814532622	25,9984600276786	25,9086542006991	26,0415130488842	26,3107417857097	241805043938,227	213514306861,529	0,179511595701258	0,00817421797157609	0,0078530296742490	0,883001873675037	-0.179511595701259	1,13250042815653	0.179511595701259	1386
O88696	sp|O88696|CLPP_MOUSE ATP-dependent Clp protease proteolytic subunit, mitochondrial OS=Mus musculus GN=Clpp PE=1 SV=1	−1,13	0,415805882203822	0,767871528551302	28,2102303198389	28,3918361374394	28,6150528659003	28,4530620211488	28,1173618978386	28,2756209716639	2169909203845,17	1917445531046,4	0,17844908120264	0,00713826235848543	0,0059380979588508	0,883652425478728	-0.17844908120264	1,1316666725135	0.17844908120264	1387
Q8QZS1	sp|Q8QZS1|HIBCH_MOUSE 3-hydroxyisobutyryl-CoA hydrolase, mitochondrial OS=Mus musculus GN=Hibch PE=1 SV=1	−1,13	0,505443294698272	0,814993646406912	30,3147859837767	29,6954546184483	29,9407135560488	29,9440608870272	29,8451084763157	29,7910678192371	10513185909005	9291109218876,65	0,178277180802285	0,0104020129502637	0,0025983663579593	0,883757720951021	-0.17827718080228	1,13153183988468	0.17827718080228	1388
Q6PHZ2-5	sp|Q6PHZ2-5|KCC2D_MOUSE Isoform 4 of Calcium/calmodulin-dependent protein kinase type II subunit delta OS=Mus musculus GN=Camk2d;sp|Q6PHZ2-2|KCC2D_MOUSE Isoform 2 of Calcium/calmodulin-dependent protein kinase type II subunit delta OS=Mus musculus GN=Cam	−1,13	0,482901781903431	0,802890545076804	30,1905445773157	30,577500263056	30,6125340776466	30,5213672527491	30,3560902036496	30,1327617042981	16931433364617,8	14965066788929,6	0,178105395077541	0,00768801515863733	0,0064286439562482	0,883862958714566	-0.178105395077543	1,13139711325196	0.178105395077543	1389
P41241	sp|P41241|CSK_MOUSE Tyrosine-protein kinase CSK OS=Mus musculus GN=Csk PE=1 SV=2	−1,13	0,683744111575988	0,905376951547671	24,6889656652559	24,9376594065974	25,5077187593753	25,1917952891573	24,9952475903761	24,5775770341929	75302658537,8199	66571338648,4929	0,177799620759454	0,01676023627138 1	0,0125864132496451	0,884050310322818	-0.177799620759453	1,1311573428834	0.177799620759453	1390
P51906	sp|P51906|EAA3_MOUSE Excitatory amino acid transporter 3 OS=Mus musculus GN=Slc1a1 PE=1 SV=2	−1,13	0,782773647770665	0,940251150142017	23,8315975050895	24,2034797259004	23,9042039329199	24,1475198498776	23,0076169888695	24,4150238637808	25958381682,9553	22953131987,2851	0,177509419708759	0,00822020063048092	0,031329121567124	0,884228156732764	-0.177509419708755	1,13092983115921	0.177509419708755	1391
P97427	sp|P97427|DPYL1_MOUSE Dihydropyrimidinase-related protein 1 OS=Mus musculus GN=Crmp1 PE=1 SV=1	−1,13	0,304488548655014	0,716532404521184	33,8014606732477	34,172245247307	34,0837055790221	33,9705548759834	33,8225092961663	33,8956357977178	594735072803291	525953185255070	0,17731276514337	0,00569214040599648	0,0021838591306464	0,884348694580905	-0.17731276514337	1,13077568398956	0.17731276514337	1392
Q8BHS6	sp|Q8BHS6|ARMX3_MOUSE Armadillo repeat-containing X-linked protein 3 OS=Mus musculus GN=Armcx3 PE=1 SV=1	−1,13	0,60147714228068	0,862899589595385	25,9896298932137	25,5141697482735	25,668783905484	25,2978380035958	25,9468628589073	25,5599556845734	148551051262,666	131405287558,541	0,176935486052419	0,00942789188779593	0,0127533583373947	0,884579990795164	-0.176935486052418	1,13048001357241	0.176935486052418	1393
Q9Z2W1	sp|Q9Z2W1|STK25_MOUSE Serine/threonine-protein kinase 25 OS=Mus musculus GN=Stk25 PE=1 SV=2	−1,13	0,497264361783689	0,809698318764119	24,9493555858095	24,6958846864592	25,1183229982551	24,6240536772299	24,7157174558305	25,0570831721089	66547895393,1031	58890842200,9546	0,176349735255434	0,00853182227927333	0,0092019416557249	0,884939213375303	-0.176349735255433	1,13002111883576	0.176349735255433	1394
Q8VE47	sp|Q8VE47|UBA5_MOUSE Ubiquitin-like modifier-activating enzyme 5 OS=Mus musculus GN=Uba5 PE=2 SV=2	−1,13	0,311070159244335	0,719371512809526	29,656852932955	29,2631523510523	29,5576191780415	29,4120521281494	29,3699426896834	29,3290713947903	6433510665324,5	5693551896549,6	0,176277256214442	0,00694274818776298	0,0014127137923068	0,88498367263722	-0.176277256214443	1,12996434953431	0.176277256214443	1395
Q99JW4	sp|Q99JW4|LIMS1_MOUSE LIM and senescent cell antigen-like-containing domain protein 1 OS=Mus musculus GN=Lims1 PE=1 SV=3;sp|Q91XD2|LIMS2_MOUSE LIM and senescent cell antigen-like-containing domain protein 2 OS=Mus musculus GN=Lims2 PE=1 SV=1	−1,13	0,474915358274922	0,79947899324683	25,346821793299	24,8141487793	25,1239866486859	25,1070055387849	24,8549736207267	24,9566019926909	79179696092,2155	70076993667,0331	0,176189645988479	0,0106602205427953	0,0050775173425855	0,885037416478827	-0.176189645988479	1,12989573252005	0.176189645988479	1396
Q61271	sp|Q61271|ACV1B_MOUSE Activin receptor type-1B OS=Mus musculus GN=Acvr1b PE=1 SV=1	−1,13	0,762065293086693	0,933389703428013	22,914286016571	23,6236389235436	23,2937395266692	23,2979771565564	23,6723843197498	22,4950198036394	12857864557,9102	11380043558,9748	0,176144979070791	0,015249449617681	0,0259787328171466	0,885064818323487	-0.176144979070796	1,12986075064448	0.176144979070796	1397

Q8BJU0	sp|Q8BJU0|SGTA_MOUSE Small glutamine-rich tetratricopeptide repeat-containing protein alpha OS=Mus musculus GN=Sgta PE=1 SV=2;sp|Q8BJU0-2|SGTA_MOUSE Isoform 2 of Small glutamine-rich tetratricopeptide repeat-containing protein alpha OS=Mus musculus GN=Sg	−1,13	0,514623587615322	0,819610722309211	29,7525593270964	29,2467701061378	29,1591429707384	29,2762930224565	29,3037700876513	29,2130685094284	5784236380748,7	5121032559949,6	0,175691779313591	0,0109005014640214	0,0015893057421729	0,885342891067454	-0.175691779313591	1,12950587855775	0.175691779313591	1398
Q9R190	sp|Q9R190|MTA2_MOUSE Metastasis-associated protein MTA2 OS=Mus musculus GN=Mta2 PE=1 SV=1	−1,13	0,234669821946159	0,657510075193589	27,343085730949	27,3177386114909	27,0428461881834	27,1802077444987	27,097740617644	27,0605273992974	672694310033,595	595594113152,7	0,175621560819767	0,00611389830779627	0,0022590614491946	0,885385983304891	-0.175621560819767	1,1294509048667	0.175621560819767	1399
Q8BUN5	sp|Q8BUN5|SMAD3_MOUSE Mothers against decapentaplegic homolog 3 OS=Mus musculus GN=Smad3 PE=1 SV=2	−1,13	0,560920684794825	0,844170752083458	23,8514133380406	23,2107589666823	23,5344531294744	23,2631031706819	23,5957802327646	23,3727250633184	16592364855,9883	14691517973,4437	0,175536056251656	0,0136125380810655	0,0072416546330466	0,885438459252626	-0.175536056251651	1,12938396740082	0.175536056251651	1400
P40336	sp|P40336|VP26A_MOUSE Vacuolar protein sorting-associated protein 26A OS=Mus musculus GN=Vps26a PE=1 SV=1;sp|P40336-2|VP26A_MOUSE Isoform 2 of Vacuolar protein sorting-associated protein 26A OS=Mus musculus GN=Vps26a	−1,13	0,198680809124226	0,628440552344853	29,8563790555124	29,6094844022266	29,7293035998322	29,5469002992119	29,5501594072729	29,7341360948367	8171898338009,13	7238235668559,71	0,175033175472535	0,00415263857722983	0,0036194110183976	0,88574715068263	-0.175033175472535	1,12899036618895	0.175033175472535	1401
Q9D6K8	sp|Q9D6K8|FUND2_MOUSE FUN14 domain-containing protein 2 OS=Mus musculus GN=Fundc2 PE=2 SV=1	−1,13	0,714994879437704	0,918558942968132	26,6885628808377	26,5801731397537	26,126952961226	25,7861812046239	26,5328938470056	26,7131195606615	311675886850,138	276109916276,76	0,174803841435548	0,011257294475401	0,018655938739806	0,885887962226353	-0.174803841435548	1,12881091361358	0.174803841435548	1402
Q8R3U1	sp|Q8R3U1|HRSL3_MOUSE HRAS-like suppressor 3 OS=Mus musculus GN=Pla2g16 PE=1 SV=2;sp|Q8R3U1-2|HRSL3_MOUSE Isoform 2 of HRAS-like suppressor 3 OS=Mus musculus GN=Pla2g16	−1,13	0,458124432886276	0,790957322506963	23,7144489157279	23,4589281980348	23,6951786116361	23,3766304858985	23,3453837113846	23,7832496113561	18166624129,8074	16094679731,7192	0,174706482234744	0,00602334965837105	0,0103942004349416	0,88594774773324	-0.174706482234747	1,12873473921975	0.174706482234747	1403
Q3V1L4	sp|Q3V1L4|5NTC_MOUSE Cytosolic purine 5-nucleotidase OS=Mus musculus GN=Nt5c2 PE=1 SV=2	−1,13	0,444122540110757	0,783519926508673	27,6913415808811	28,1709716265096	28,1238312869463	27,9366295152886	27,8881308498074	27,7989733088988	1439592924327,32	1275778748637,19	0,174282764424033	0,00944295553781974	0,0025048522833858	0,88620798774301	-0.174282764424039	1,12840327985171	0.174282764424039	1404
Q61301-2	sp|Q61301-2|CTNA2_MOUSE Isoform 2 of Catenin alpha-2 OS=Mus musculus GN=Ctnna2;sp|Q61301-3|CTNA2_MOUSE Isoform 3 of Catenin alpha-2 OS=Mus musculus GN=Ctnna2;sp|Q61301|CTNA2_MOUSE Catenin alpha-2 OS=Mus musculus GN=Ctnna2 PE=1 SV=3	−1,13	0,574012410459667	0,849998193752035	31,039393479464	30,5354699767686	30,381645732265	30,5414934586332	30,6668670710889	30,3862745237618	20514841782293,4	18183669333316	0,174024673336739	0,0112244105753518	0,0046037816615233	0,886366540199717	-0.174024673336735	1,12820143207874	0.174024673336735	1405
Q9D1N9	sp|Q9D1N9|RM21_MOUSE 39S ribosomal protein L21, mitochondrial OS=Mus musculus GN=Mrpl21 PE=2 SV=1;sp|Q9D1N9-2|RM21_MOUSE Isoform 2 of 39S ribosomal protein L21, mitochondrial OS=Mus musculus GN=Mrpl21	−1,13	0,312717387521494	0,720856062638073	25,6830722098595	25,4494598461444	25,4982180151912	25,286634641881	25,3750798339753	25,6077300356249	124004458439,837	109934236119,46	0,173751246414976	0,0048245516481278	0,0065238508151235	0,886534544826842	-0.173751246414982	1,1279876298507	0.173751246414982	1406
Q9CR41	sp|Q9CR41|HYPK_MOUSE Huntingtin-interacting protein K OS=Mus musculus GN=Hypk PE=2 SV=2;sp|Q9CR41-2|HYPK_MOUSE Isoform 2 of Huntingtin-interacting protein K OS=Mus musculus GN=Hypk	−1,13	0,0200563634226009	0,196394730444062	24,1749746788762	24,2472581965185	24,1086932997909	24,0297670368369	24,018150513668	24,1219424658587	31617461743,1694	28032208944,4147	0,173636118921826	0,0028665335425842	0,0023639356664776	0,886605293369913	-0.173636118921828	1,12789761969397	0.173636118921828	1407
Q3TDX8	sp|Q3TDX8|NB5R4_MOUSE Cytochrome b5 reductase 4 OS=Mus musculus GN=Cyb5r4 PE=2 SV=3;sp|Q3TDX8-5|NB5R4_MOUSE Isoform 2 of Cytochrome b5 reductase 4 OS=Mus musculus GN=Cyb5r4	−1,13	0,485378850592112	0,804063723663356	23,277183681821	23,6043336988036	23,7858998078014	23,4577010632101	23,5733620117965	23,2759764901913	16988555178,2348	15065600284,9783	0,173305003292855	0,0109445039084939	0,0063966893836449	0,886808803157073	-0.173305003292858	1,12763878351225	0.173305003292858	1408
O70209	sp|O70209|PDLI3_MOUSE PDZ and LIM domain protein 3 OS=Mus musculus GN=Pdlim3 PE=1 SV=1	−1,13	0,463595967254052	0,793595716606975	24,1341293512829	23,675109750947	23,8940402301816	23,8870579935421	23,5891400062898	23,8670266020545	23994563260,1144	21280880284,04	0,173149724725666	0,00960588123994967	0,0070022883055869	0,886904256324378	-0.173149724725669	1,12751742126521	0.173149724725669	1409
P52480	sp|P52480|KPYM_MOUSE Pyruvate kinase PKM OS=Mus musculus GN=Pkm PE=1 SV=4;sp|P14618|KPYM_HUMAN Pyruvate kinase PKM OS=Homo sapiens GN=PKM PE=1 SV=4;sp|P14618-3|KPYM_HUMAN Isoform 3 of Pyruvate kinase PKM OS=Homo sapiens GN=PKM	−1,13	0,478088786881937	0,799905345588749	29,6259603347948	30,1667006838448	30,0350880404903	29,9085130255149	29,8046256258292	29,7550162373898	10090172231876,6	8950390658387,88	0,172928242121036	0,00941766484722459	0,0026265715724998	0,887040424355894	-0.172928242121029	1,12734433802848	0.172928242121029	1410
Q99KP3	sp|Q99KP3|CRYL1_MOUSE Lambda-crystallin homolog OS=Mus musculus GN=Cryl1 PE=2 SV=3	−1,13	0,698629064480792	0,912827852392638	27,854757020185	28,3308483775731	28,6351446174494	27,7510871215627	28,2518043492866	28,4585732117269	1901346315738,81	1686744677308,2	0,172779722550411	0,0139116701372681	0,0129210965176343	0,887131746250434	-0.172779722550407	1,12722828850012	0.172779722550407	1411
P60521	sp|P60521|GBRL2_MOUSE Gamma-aminobutyric acid receptor-associated protein-like 2 OS=Mus musculus GN=Gabarapl2 PE=1 SV=1;sp|P60520|GBRL2_HUMAN Gamma-aminobutyric acid receptor-associated protein-like 2 OS=Homo sapiens GN=GABARAPL2 PE=1 SV=1	−1,13	0,595948586902111	0,860936823333794	28,787551864115	28,4137586698619	28,2028134144342	28,092191112154	28,3498300207127	28,6029701816381	2309474940490,41	2048912823016,7	0,172706289985964	0,010401997594686	0,0090090962222920	0,887176902028484	-0.172706289985969	1,12717091451947	0.172706289985969	1412

O88569	sp|O88569|ROA2_MOUSE Heterogeneous nuclear ribonucleoproteins A2/B1 OS=Mus musculus GN=Hnrnpa2b1 PE=1 SV=2;sp|O88569-3| ROA2_MOUSE Isoform 3 of Heterogeneous nuclear ribonucleoproteins A2/B1 OS=Mus musculus GN=Hnrnpa2b1;sp|O88569-2|ROA2_MOUSE Isoform 2 o	−1,13	0,47828831634007	0,799905345588749	32,5652501037116	31,9922601162753	32,3661802272999	32,1814288440177	32,2182618919671	32,1651705876441	107433891953786	95322510734911	0,172560332409237	0,00900421877057733	0,0008450898323504	0,887266662329567	-0.172560332409237	1,12705688431305	0.172560332409237	1413
Q9D023	sp|Q9D023|MPC2_MOUSE Mitochondrial pyruvate carrier 2 OS=Mus musculus GN=Mpc2 PE=1 SV=1	−1,13	0,484739018240653	0,803590359311294	25,4540678038503	25,2907819321046	25,1612997878741	24,9261116536089	25,1800505226162	25,4412526778006	97395889191,3245	86418846348,9536	0,172514909706755	0,00579830107073788	0,0102285053661631	0,887294598021405	-0.172514909706754	1,12702139991601	0.172514909706755	1414
Q8CGK3	sp|Q8CGK3|LONM_MOUSE Lon protease homolog, mitochondrial OS=Mus musculus GN=Lonp1 PE=1 SV=2	−1,13	0,731411857119119	0,924701393764781	31,1056636057856	30,076831063814	30,6923605230542	30,6159575258508	30,7404493890883	30,161244964118	19963999268292,7	17722993135602,3	0,171778483038329	0,0169051083356453	0,0099943939444158	0,887747635001689	-0.171778483038332	1,12644625631483	0.171778483038332	1415
P61226	sp|P61226|RAP2B_MOUSE Ras-related protein Rap-2b OS=Mus musculus GN=Rap2b PE=1 SV=1	−1,13	0,554410942413954	0,839722577101535	28,496957284198	28,1337438378691	28,0603919717577	27,8286564820045	28,1757537010239	28,3308483775731	1820922514336,86	1617257377366,89	0,171120238819386	0,0082084160752227	0,0091470830537147	0,888152771264877	-0.171120238819386	1,12593242103589	0.171120238819386	1416
Q91YS8	sp|Q91YS8|KCC1A_MOUSE Calcium/calmodulin-dependent protein kinase type 1 OS=Mus musculus GN=Camk1 PE=1 SV=1	−1,13	0,419321365836776	0,769072485508496	29,2904693642646	28,8042521530299	29,1030743452272	28,9363589553108	29,0196626152411	28,8859884835841	4199270577762,74	3729654376040,28	0,171096807125567	0,00843693463622977	0,0023321600976519	0,888167196415179	-0.171096807125566	1,1259141342263	0.171096807125566	1417
Q99JX4	sp|Q99JX4|EIF3M_MOUSE Eukaryotic translation initiation factor 3 subunit M OS=Mus musculus GN=Eif3m PE=2 SV=1	−1,13	0,664952959420569	0,896678990733797	28,9933056026423	28,3200413611814	28,6120839248889	28,6062432168209	28,8067679360488	28,1567892511131	2747768644487,07	2440604331296,24	0,171022112236242	0,0117874902033446	0,0116690396274092	0,888213182064253	-0.171022112236239	1,12585584203552	0.171022112236238	1418
O08586	sp|O08586|PTEN_MOUSE Phosphatidylinositol 3,4,5-trisphosphate 3-phosphatase and dual-specificity protein phosphatase PTEN OS=Mus musculus GN=Pten PE=1 SV=1	−1,13	0,572986305744858	0,849652022795136	28,8326097047498	28,2792329488929	28,3886961162417	28,3607504246018	28,6073269178441	28,1771173987933	2384903050987,39	2118504604352,99	0,170884355978643	0,010282366183045	0,0076059780592345	0,888297997470335	-0.17088435597864	1,12574834441569	0.17088435597864	1419
P61082	sp|P61082|UBC12_MOUSE NEDD8-conjugating enzyme Ubc12 OS=Mus musculus GN=Ube2m PE=2 SV=1	−1,13	0,571148092573119	0,84806207361736	29,8851245258515	29,8904491527376	29,8306347317633	29,3411392654344	29,8904491527376	30,0196926870257	9371892224588,45	8326189588213,92	0,170683954340962	0,00110831651432369	0,012110621860555	0,8884213975881	-0.170683954340962	1,12559198001626	0.170683954340962	1420
P31230	sp|P31230|AIMP1_MOUSE Aminoacyl tRNA synthase complex-interacting multifunctional protein 1 OS=Mus musculus GN=Aimp1 PE=1 SV=2	−1,13	0,327522626470806	0,726927682919497	29,2839283038169	28,963339565092	29,1143759145037	28,8597896366355	29,126082642873	29,0211872608148	4434996434767,4	3940596328148,65	0,170518976360718	0,00550757023055504	0,0046252053814927	0,888522997957116	-0.170518976360719	1,12546327140568	0.170518976360719	1421
Q8VBV7	sp|Q8VBV7|CSN8_MOUSE COP9 signalosome complex subunit 8 OS=Mus musculus GN=Cops8 PE=1 SV=1	−1,13	0,431362170718011	0,775108167118334	29,6357657449474	29,3868050777055	29,4599545956542	29,1996198193394	29,2978797219439	29,6309744420806	6444029373058,9	5726685282017,79	0,170262749804029	0,00433846734634187	0,0076960484979023	0,888680816068255	-0.170262749804029	1,12526340382169	0.170262749804029	1422
Q7TSZ8	sp|Q7TSZ8|NACC1_MOUSE Nucleus accumbens-associated protein 1 OS=Mus musculus GN=Nacc1 PE=1 SV=1	−1,13	0,47459695900085	0,799366691338604	23,620865820898	23,9042039329199	24,0031450361206	23,8296632028795	23,4877467946751	23,8568543067188	22634435161,4537	20115465179,6991	0,170214203463074	0,00832175322907308	0,0086704668636784	0,888710720466999	-0.170214203463072	1,125225539616	0.170214203463072	1423
Q99P31	sp|Q99P31|HPBP1_MOUSE Hsp70-binding protein 1 OS=Mus musculus GN=Hspbp1 PE=2 SV=1	−1,13	0,437504947994712	0,778453531535867	27,6095679757196	27,1620716602265	27,1921188951442	27,1164940541076	27,2761384034076	27,2176411250769	733616935944,313	652074416131,207	0,169990327409079	0,0091555026240883	0,0029689682024681	0,888848640458192	-0.169990327409078	1,12505094172672	0.169990327409078	1424
Q8BUY8	sp|Q8BUY8|GASP2_MOUSE G-protein coupled receptor-associated sorting protein 2 OS=Mus musculus GN=Gprasp2 PE=2 SV=2	−1,12	0,384721983080508	0,751739350763028	23,9952333945482	23,6149347963874	23,9294139405435	23,7818895341833	23,8100825694553	23,594321097961	22720362304,1957	20196282717,5044	0,169896062379417	0,00852313291194814	0,0049425900342275	0,888906719316479	-0.169896062379415	1,12497743381774	0.169896062379415	1425
Q9QXJ1	sp|Q9QXJ1|APBB1_MOUSE Amyloid beta A4 precursor protein-binding family B member 1 OS=Mus musculus GN=Apbb1 PE=1 SV=3;sp|Q9QXJ1-2| APBB1_MOUSE Isoform 2 of Amyloid beta A4 precursor protein-binding family B member 1 OS=Mus musculus GN=Apbb1;sp|O00213-2|AP	−1,12	0,0838375788891039	0,430545618027756	27,3081622510207	27,2725327823275	27,1320858547966	27,1854010346971	27,0421427414941	27,1320858547966	674740243547,942	599808661415,22	0,169829855794744	0,00341780820577104	0,0026698260442818	0,888947512988533	-0.169829855794744	1,12492580876696	0.169829855794744	1426
Q921F4	sp|Q921F4|HNRLL_MOUSE Heterogeneous nuclear ribonucleoprotein L-like OS=Mus musculus GN=Hnrnpll PE=1 SV=3;sp|Q921F4-4| HNRLL_MOUSE Isoform 2 of Heterogeneous nuclear ribonucleoprotein L-like OS=Mus musculus GN=Hnrnpll	−1,12	0,486968532258898	0,804641488140932	28,796752241005	29,351342606842	29,2283500040983	29,034879459619	28,9871137767746	29,002551104323	4456931395808,51	3963630233561,85	0,169228374145324	0,00999998109587383	0,0008402979015132	0,889318206084441	-0.169228374145326	1,12445690772809	0.169228374145326	1427
P30275	sp|P30275|KCRU_MOUSE Creatine kinase U-type, mitochondrial OS=Mus musculus GN=Ckmt1 PE=1 SV=1	−1,12	0,422600447148108	0,7711031271846	34,4595513825974	34,0159445813919	34,1068660507989	34,1536747954851	34,1068660507989	33,9705548759834	708464219199867	630183339100252	0,168923379417093	0,00685269698594912	0,0027917808429552	0,889506233373332	-0.168923379417093	1,1242192156514	0.168923379417093	1428
Q8BMS1	sp|Q8BMS1|ECHA_MOUSE Trifunctional enzyme subunit alpha, mitochondrial OS=Mus musculus GN=Hadha PE=1 SV=1	−1,12	0,332936457951298	0,72953198881961	30,9568503354048	30,9766739283493	30,6271401858965	30,688685623596	30,7005833571881	30,8202582060154	25091614348637,9	22320107333228,7	0,168861329262178	0,00636332447511467	0,0023676222281732	0,889544491761262	-0.168861329262177	1,12417086414648	0.168861329262177	1429
Q3UDE2	sp|Q3UDE2|TTL12_MOUSE Tubulin--tyrosine ligase-like protein 12 OS=Mus musculus GN=Ttll12 PE=2 SV=1	−1,12	0,472721507035977	0,798839508725353	28,4137586698619	28,3637482883521	27,9358995293517	28,197075208073	28,1612225425277	28,0044923642566	1834516660601,41	1632167559211,72	0,168610834053682	0,0093013031118497	0,0036420916287249	0,889698956823126	-0.168610834053689	1,12397569125036	0.168610834053689	1430

P46737	sp|P46737|BRCC3_MOUSE Lys-63-specific deubiquitinase BRCC36 OS=Mus musculus GN=Brcc3 PE=2 SV=1;sp|P46737-2|BRCC3_MOUSE Isoform 2 of Lys-63-specific deubiquitinase BRCC36 OS=Mus musculus GN=Brcc3	−1,12	0,70020144251244	0,913439194888196	26,6900416088642	27,067317939297	27,2232737570987	27,3216488345134	26,8832357761076	26,4253543694505	528624631203,624	470351595112,943	0,168504051768363	0,0101566593774325	0,0166754734712135	0,889764811075868	-0.168504051768364	1,12389250232411	0.168504051768364	1431
Q9JL62	sp|Q9JL62|GLTP_MOUSE Glycolipid transfer protein OS=Mus musculus GN=Gltp PE=2 SV=3	−1,12	0,402820135057738	0,75772921385291	28,5833286268197	28,4918017637118	28,5178789364239	28,1512065915307	28,6062432168209	28,4869189419964	2459558821621,68	2189708582277,13	0,167660676974729	0,00165271909525159	0,0083033390271781	0,890285104396638	-0.167660676974731	1,12323568603084	0.167660676974731	1432
Q91WE1	sp|Q91WE1|SNX15_MOUSE Sorting nexin-15 OS=Mus musculus GN=Snx15 PE=2 SV=1	−1,12	0,102535910204056	0,46388602391647	26,2113399560879	26,0089907009257	26,2212886157278	26,0128955673314	26,0168115282551	26,0637890078749	226770974960,834	201925644050,047	0,167411856646279	0,00458181423778114	0,0010879522792290	0,890438664317256	-0.167411856646282	1,12304197927743	0.167411856646282	1433
Q9WVJ2	sp|Q9WVJ2|PSD13_MOUSE 26S proteasome non-ATPase regulatory subunit 13 OS=Mus musculus GN=Psmd13 PE=1 SV=1	−1,12	0,215570132864842	0,646799042967852	30,6232978272985	30,348015515816	30,4300715036396	30,3323426792313	30,4385207524286	30,2825025623271	17049266228005,8	15181853744490,8	0,167361691007654	0,00463886548356628	0,0026254831805648	0,890469627341057	-0.167361691007655	1,12300292934864	0.167361691007655	1434
Q8R0A0	sp|Q8R0A0|T2FB_MOUSE General transcription factor IIF subunit 2 OS=Mus musculus GN=Gtf2f2 PE=1 SV=1	−1,12	0,869569385178723	0,970488182770155	23,4176157562769	23,7805294570105	23,9894755268835	24,0011855134086	24,558208524557	22,2806686370197	20205221809,9589	17994900065,3641	0,167140099021364	0,0121944146408938	0,0502795420562495	0,890606410294127	-0.167140099021361	1,12283045399341	0.167140099021361	1435
O55234	sp|O55234|PSB5_MOUSE Proteasome subunit beta type-5 OS=Mus musculus GN=Psmb5 PE=1 SV=3	−1,12	0,366036254679362	0,739315231400715	30,766243128978	30,6034495341565	30,5213672527491	30,3043185118729	30,6402261423119	30,59946784483	20072128065827	17879409028045,3	0,166894529089939	0,00406900630937652	0,0060071695944697	0,890758018751643	-0.166894529089935	1,12263934643154	0.166894529089936	1436
A2APY7	sp|A2APY7|NDUF5_MOUSE NADH dehydrogenase [ubiquinone] 1 alpha subcomplex assembly factor 5 OS=Mus musculus GN=Ndufaf5 PE=2 SV=1;sp|A2APY7-2|NDUF5_MOUSE Isoform 2 of NADH dehydrogenase [ubiquinone] 1 alpha subcomplex assembly factor 5 OS=Mus musculus GN=N	−1,12	0,593920504776812	0,860080030359509	26,0475252294948	25,3783577930722	25,5777956432148	25,530459038085	25,4127860971458	25,7136461877525	140418472246,914	125089723862,635	0,166769459899458	0,0133848675971843	0,0059334872535539	0,890835243120117	-0.166769459899467	1,12254202752188	0.166769459899467	1437
A2ADY9	sp|A2ADY9|DDI2_MOUSE Protein DDI1 homolog 2 OS=Mus musculus GN=Ddi2 PE=1 SV=1;sp|Q5TDH0-2|DDI2_HUMAN Isoform 2 of Protein DDI1 homolog 2 OS=Homo sapiens GN=DDI2;sp|Q5TDH0|DDI2_HUMAN Protein DDI1 homolog 2 OS=Homo sapiens GN=DDI2 PE=1 SV=1;sp|Q5TDH0-3|D	−1,12	0,522813285168829	0,823975521312956	27,7082701854407	27,1591850873045	27,4140252679283	27,4568631562061	27,1601478692373	27,3187839702351	815575780773,241	726810532685,85	0,166239607157073	0,010018467126486	0,0054363156206844	0,891162476645354	-0.166239607157072	1,12212983177248	0.166239607157072	1438
Q9QYJ0	sp|Q9QYJ0|DNJA2_MOUSE DnaJ homolog subfamily A member 2 OS=Mus musculus GN=Dnaja2 PE=1 SV=1	−1,12	0,0793148611380556	0,419103788947188	30,413494702835	30,239302803755	30,2626911460235	30,2028177448941	30,2457298105003	30,1213977470374	14499900223659,9	12922379478357,7	0,166171225906405	0,00311973644425437	0,0020918229285275	0,89120471720708	-0.166171225906399	1,12207664601896	0.166171225906399	1439
Q9D2P4	sp|Q9D2P4|URM1_MOUSE Ubiquitin-related modifier 1 OS=Mus musculus GN=Urm1 PE=1 SV=1	−1,12	0,248549055499018	0,673464050610029	24,6953323909291	24,7860991569549	24,803332574449	24,5607808475858	24,5452995178382	24,8340128117331	56731079723,5899	50573710643,1657	0,165751687781221	0,00234324475734048	0,0065892883986266	0,8914639187122	-0.165751687781221	1,12175039169795	0.165751687781221	1440
O55137	sp|O55137|ACOT1_MOUSE Acyl-coenzyme A thioesterase 1 OS=Mus musculus GN=Acot1 PE=1 SV=1	−1,12	0,734654407802643	0,924701393764781	26,6005971337788	25,8025240721104	25,7136461877525	26,2771103257199	25,6433260264358	25,8525777820242	203498091732,092	181466707618,443	0,165310374238265	0,0187584662547537	0,0124565746979323	0,89173665499206	-0.165310374238265	1,12140730607166	0.165310374238265	1441
P10630-2	sp|P10630-2|IF4A2_MOUSE Isoform 2 of Eukaryotic initiation factor 4A-II OS=Mus musculus GN=Eif4a2;sp|P10630|IF4A2_MOUSE Eukaryotic initiation factor 4A-II OS=Mus musculus GN=Eif4a2 PE=2 SV=2	−1,12	0,138223307457466	0,52900134279042	33,1690884811052	32,9255457365668	33,0960166649535	32,9255457365668	32,9187789512132	33,0034445863308	228727005861713	204023525602288	0,164891198738768	0,00377985003260023	0,0014279573456663	0,891995787002255	-0.164891198738765	1,12108152815465	0.164891198738764	1442
Q5EG47	sp|Q5EG47|AAPK1_MOUSE 5-AMP-activated protein kinase catalytic subunit alpha-1 OS=Mus musculus GN=Prkaa1 PE=1 SV=2;sp|P54645|AAPK1_RAT 5-AMP-activated protein kinase catalytic subunit alpha-1 OS=Rattus norvegicus GN=Prkaa1 PE=1 SV=2	−1,12	0,678819990049317	0,903732687282123	23,7832496113561	24,5790747392146	24,6308082037023	24,155861296033	24,2411722072097	24,2532325976359	36884018262,5401	32900555101,4384	0,164883910666404	0,0195268512710376	0,0021918485777734	0,892000293114827	-0.164883910666404	1,12107586479377	0.164883910666404	1443
O89050	sp|O89050|MKLN1_MOUSE Muskelin OS=Mus musculus GN=Mkln1 PE=1 SV=1;sp|Q9UL63|MKLN1_HUMAN Muskelin OS=Homo sapiens GN=MKLN1 PE=1 SV=2	−1,12	0,775182622548416	0,938619589104496	24,18201000329	23,3706667882325	23,1309610408383	23,0956367782024	23,9115536096075	23,3339271274258	17080659052,7149	15237710888,329	0,164717454306678	0,0233784110560614	0,0178937011158045	0,89210321693688	-0.164717454306677	1,12094652391636	0.164717454306676	1444
P07901	sp|P07901|HS90A_MOUSE Heat shock protein HSP 90-alpha OS=Mus musculus GN=Hsp90aa1 PE=1 SV=4	−1,12	0,542152600461255	0,833050322780513	34,5639686668958	34,2388631383902	33,9275094387217	34,0837055790221	34,1977817973996	34,1068660507989	744286443474118	664098197589654	0,164461375774361	0,00929387667717457	0,0017667589427436	0,892261579412668	-0.164461375774361	1,12074757343945	0.164461375774361	1445
Q91W90	sp|Q91W90|TXND5_MOUSE Thioredoxin domain-containing protein 5 OS=Mus musculus GN=Txndc5 PE=1 SV=2	−1,12	0,350873214275071	0,732843629012038	28,2810559679654	27,9203165194517	27,9254032200445	27,8832358468707	27,9400277759159	27,9628273575294	1508683555804,95	1346725213202,27	0,16383472211989	0,00737530652536409	0,0014675839297880	0,892649229204153	-0.163834722119881	1,12026086763281	0.16383472211988	1446
Q8R2Y8	sp|Q8R2Y8|PTH2_MOUSE Peptidyl-tRNA hydrolase 2, mitochondrial OS=Mus musculus GN=Ptrh2 PE=2 SV=1	−1,12	0,312307501872103	0,720394556114739	26,4042232161596	26,0782885894433	26,2029568269526	26,0532668033667	26,234878662775	26,056977654842	245973475571,45	219592861742,664	0,163671593911192	0,00627030342656931	0,0039746830022292	0,892750168417558	-0.163671593911191	1,12013420481628	0.163671593911191	1447

Q9Z1J3-2	sp|Q9Z1J3-2|NFS1_MOUSE Isoform Cytoplasmic of Cysteine desulfurase, mitochondrial OS=Mus musculus GN=Nfs1;sp|Q9Z1J3|NFS1_MOUSE Cysteine desulfurase, mitochondrial OS=Mus musculus GN=Nfs1 PE=2 SV=3	−1,12	0,342874227120961	0,729891123744941	29,2120692784127	28,8541446687965	28,9439646135663	28,8067679360488	28,9855437086712	28,8780890237702	3944695392137,3	3522293853284,48	0,163398626734561	0,00642048046325913	0,0031150575693722	0,892919098469614	-0.163398626734563	1,11992228827215	0.163398626734563	1448
Q9CQH7	sp|Q9CQH7|BT3L4_MOUSE Transcription factor BTF3 homolog 4 OS=Mus musculus GN=Btf3l4 PE=2 SV=1	−1,12	0,784355410536835	0,940443821602512	27,8216158205759	27,3497540213979	26,4412629368967	26,9717991804572	27,2481132134366	27,0534472647175	652587408324,891	582805813421,886	0,163155882701561	0,0257898120756682	0,0052399815685991	0,893069351304026	-0.163155882701561	1,11973386897651	0.163155882701561	1449
Q8BG32	sp|Q8BG32|PSD11_MOUSE 26S proteasome non-ATPase regulatory subunit 11 OS=Mus musculus GN=Psmd11 PE=1 SV=3	−1,12	0,20433879701111	0,635648869851434	30,9631543867588	30,6814880843035	30,8437717553966	30,6477397566522	30,766243128978	30,7354901350717	24494541404017,2	21877744581846,2	0,162996265566172	0,00458576236362998	0,0020019799857686	0,893168164326529	-0.162996265566171	1,11960999052628	0.162996265566172	1450
Q64737-2	sp|Q64737-2|PUR2_MOUSE Isoform Short of Trifunctional purine biosynthetic protein adenosine-3 OS=Mus musculus GN=Gart;sp|Q64737|PUR2_MOUSE Trifunctional purine biosynthetic protein adenosine-3 OS=Mus musculus GN=Gart PE=2 SV=3	−1,12	0,274148932350884	0,695562886495618	26,3498310529515	26,030206424081	26,1321115412	26,0637890078749	26,126952961226	25,9834974475046	232165505351,631	207434155949,536	0,16250016884536	0,00623872612333909	0,0027591428598781	0,893475349128039	-0.162500168845361	1,11922505861625	0.162500168845361	1451
Q3TMH2	sp|Q3TMH2|SCRN3_MOUSE Secernin-3 OS=Mus musculus GN=Scrn3 PE=1 SV=1	−1,12	0,327836798786605	0,726927682919497	28,7010859141301	28,8367004893606	28,468006362801	28,6266029003322	28,5524438127231	28,4901679344148	2822368632034,87	2522836254798,98	0,161859860965235	0,0065047405951589	0,0023918864812605	0,893871986162228	-0.161859860965238	1,11872842586042	0.161859860965238	1452
P25444	sp|P25444|RS2_MOUSE 40S ribosomal protein S2 OS=Mus musculus GN=Rps2 PE=2 SV=3;sp|P15880|RS2_HUMAN 40S ribosomal protein S2 OS=Homo sapiens GN=RPS2 PE=1 SV=2	−1,12	0,634014457916103	0,882745957862852	28,9989754780626	29,6389646388719	29,7525593270964	29,3677682146643	29,3723329959481	29,3143252751957	6249357751751,75	5587066535218,18	0,161616930068371	0,0137892499159814	0,0010988848039778	0,894022515137988	-0.161616930068372	1,11854006254603	0.161616930068372	1453
Q8BH61	sp|Q8BH61|F13A_MOUSE Coagulation factor XIII A chain OS=Mus musculus GN=F13a1 PE=2 SV=3	−1,12	0,383018291230885	0,750656314290582	25,1735426829318	24,7358047995479	25,0138713240005	24,8599080669657	24,845323645973	24,882110644788	70185408400,2657	62751445627,1339	0,161522428989379	0,00886993667353738	0,0007450795898303	0,894081078352696	-0.161522428989379	1,11846679704088	0.161522428989379	1454
Q9CRB2	sp|Q9CRB2|NHP2_MOUSE H/ACA ribonucleoprotein complex subunit 2 OS=Mus musculus GN=Nhp2 PE=2 SV=1	−1,12	0,982020156692502	0,996986564004246	21,0597463648342	21,1169734730192	13,2874558662933	9,94482308139412	22,311282263451	22,8730078302888	106969956,010371	95665764,8520732	0,161131016331488	0,243613732073514	0,397648387547677	0,894323681340938	-0.161131016331487	1,11816339079897	0.161131016331487	1455
O88952	sp|O88952|LIN7C_MOUSE Protein lin-7 homolog C OS=Mus musculus GN=Lin7c PE=1 SV=2	−1,12	0,467742702950621	0,796164546189264	27,3878476077223	27,7557362941429	27,6659486438165	27,4879183321299	27,3151166099119	27,6754662525446	972540459553,144	870935476732,745	0,159192429534689	0,00694880049414913	0,0065553483119398	0,895526215056303	-0.15919242953469	1,11666189463491	0.15919242953469	1456
Q60759	sp|Q60759|GCDH_MOUSE Glutaryl-CoA dehydrogenase, mitochondrial OS=Mus musculus GN=Gcdh PE=1 SV=2	−1,12	0,449237572660841	0,786343703431612	27,2082466545883	27,0782792312648	27,0647658758816	27,1451359480015	27,1451359480015	26,7306224117162	598143933801,772	535766768133,576	0,158887589476824	0,0029216378588037	0,0088613994131503	0,895715458866681	-0.158887589476822	1,1164259699897	0.158887589476822	1457
Q9WTN0	sp|Q9WTN0|GGPPS_MOUSE Geranylgeranyl pyrophosphate synthase OS=Mus musculus GN=Ggps1 PE=2 SV=1;sp|O95749|GGPPS_HUMAN Geranylgeranyl pyrophosphate synthase OS=Homo sapiens GN=GGPS1 PE=1 SV=1	−1,12	0,155433540519148	0,559486788593506	27,7433277429496	27,8832358468707	27,9512595440036	27,8080490843163	27,7842060823852	27,6568502118367	1256403484417,11	1126010299794,31	0,158079825134261	0,00380543343202929	0,0029294274824547	0,896217109996878	-0.158079825134264	1,11580105852195	0.158079825134264	1458
Q9CWX2	sp|Q9CWX2|CIA30_MOUSE Complex I intermediate-associated protein 30, mitochondrial OS=Mus musculus GN=Ndufaf1 PE=2 SV=2	−1,12	0,825923063299649	0,955744170441451	22,7593573011881	23,1878192907474	23,5108751221745	23,5572160930767	23,4209979293226	22,1514329050445	11352273903,216	10175163556,3627	0,157929319042507	0,0162827299430747	0,0336456483694478	0,896310610817817	-0.15792931904251	1,11568466102122	0.15792931904251	1459
P97370	sp|P97370|AT1B3_MOUSE Sodium/potassium-transporting ATPase subunit beta-3 OS=Mus musculus GN=Atp1b3 PE=1 SV=1	−1,12	0,473927982832317	0,799218469891531	27,4549509257709	26,9302714663707	27,232167200089	27,081221549861	27,0965384478712	27,1124388235916	653622974608,062	586086332314,684	0,157345500870636	0,00967925736364773	0,0005760674425791	0,896673395952956	-0.157345500870636	1,11523326610714	0.157345500870636	1460
Q6P9Z1	sp|Q6P9Z1|SMRD3_MOUSE SWI/SNF-related matrix-associated actin-dependent regulator of chromatin subfamily D member 3 OS=Mus musculus GN=Smarcd3 PE=1 SV=2	−1,12	0,713312719912009	0,917934955801873	22,8598253519744	23,5758934844069	23,4277205768759	23,4099585585598	23,3482940068699	22,7780591977932	12994774314,0593	11652313579,3171	0,157315146147409	0,0162308115693463	0,0150307035807616	0,896692262420459	-0.157315146147405	1,1152098015174	0.157315146147405	1461
Q9CZU6	sp|Q9CZU6|CISY_MOUSE Citrate synthase, mitochondrial OS=Mus musculus GN=Cs PE=1 SV=1	−1,11	0,31489343477453	0,722907157640623	34,7531101143992	34,4014332388837	34,6457890411816	34,4336965995204	34,5341448386175	34,5060432263395	1063254410468520	953628108651103	0,156988173720583	0,00520903744515085	0,0015024877718674	0,896895511800313	-0.156988173720576	1,11495707899433	0.156988173720576	1462
O55102	sp|O55102|BL1S1_MOUSE Biogenesis of lysosome-related organelles complex 1 subunit 1 OS=Mus musculus GN=Bloc1s1 PE=1 SV=2	−1,11	0,690828260936712	0,908901267924358	24,3237320068627	24,3741949927678	23,5843529479607	23,80523416359	24,132235856752	24,0194215071423	29102598883,9752	26111208646,9232	0,15647875335029	0,018351742156542	0,0069249406739389	0,897212264479268	-0.156478753350288	1,11456345347708	0.156478753350288	1463

Q9Z199	sp|Q9Z199|SPT4B_MOUSE Transcription elongation factor SPT4-B OS=Mus musculus GN=Supt4h1b PE=2 SV=1;sp|P63272|SPT4H_HUMAN Transcription elongation factor SPT4 OS=Homo sapiens GN=SUPT4H1 PE=1 SV=1;sp|P63271|SPT4A_MOUSE Transcription elongation factor SPT4	−1,11	0,682843160808261	0,904912326646161	25,3572525299008	25,0452755773747	24,6713112340392	24,5749811329927	25,0087957507153	25,1647282924477	73799154641,984	66214704133,9377	0,156452662235497	0,013723973967735	0,0122647301938217	0,897228490694234	-0.156452662235496	1,11454329679862	0.156452662235496	1464
Q8CAY6	sp|Q8CAY6|THIC_MOUSE Acetyl-CoA acetyltransferase, cytosolic OS=Mus musculus GN=Acat2 PE=1 SV=2	−1,11	0,508562149809775	0,816124555116335	30,1714331301119	29,8788419232647	29,9571855137959	29,6259603347948	30,0893751498851	29,9670200277696	10713083374410,1	9612817730957,94	0,156342483405635	0,00504840560628009	0,0080335111780898	0,89729701478098 1	-0.15634248340563	1,11445818221527	0.15634248340563	1465
P61750	sp|P61750|ARF4_MOUSE ADP-ribosylation factor 4 OS=Mus musculus GN=Arf4 PE=1 SV=2	−1,11	0,311761498343315	0,720145262585398	28,995304264796	28,6809841596718	28,963339565092	28,8240709718089	28,696381711632	28,7942855809832	3486348385565,87	3128514544711,45	0,156238931762998	0,0059898207810533 9	0,0023218849292547	0,89736142195773 7	-0.156238931762998	1,11437819314579	0.156238931762998	1466
Q8CIP4	sp|Q8CIP4|MARK4_MOUSE MAP/microtubule affinity-regulating kinase 4 OS=Mus musculus GN=Mark4 PE=1 SV=1	−1,11	0,693337325958372	0,910309912045398	26,4187355803847	26,6160829578104	26,275921090209	26,8410458836077	26,0601159193524	26,0846949698555	302974114910,451	271877905131,071	0,156235628209175	0,0064609628685458 5	0,016861685792282 6	0,897363476782262	-0.156235628209172	1,1143756413909	0.156235628209172	1467
Q8BGB5	sp|Q8BGB5|LIMD2_MOUSE LIM domain-containing protein 2 OS=Mus musculus GN=Limd2 PE=2 SV=1	−1,11	0,476535359111157	0,799905345588749	24,389762517735	23,9878581132619	23,9062094718821	24,0288934571652	23,9338055309889	23,9964472236921	29117640618,9938	26130840201,093	0,156139946483214	0,0107430300325344	0,0020151606744742	0,897422993264349	-0.156139946483216	1,11430173675686	0.156139946483216	1468
Q9JJY4	sp|Q9JJY4|DDX20_MOUSE Probable ATP-dependent RNA helicase DDX20 OS=Mus musculus GN=Ddx20 PE=1 SV=2	−1,11	0,689107003958654	0,907856236295823	25,6464523299023	25,1876434359737	25,35089230623	25,2754018283628	25,698996365124	24,8859998394022	106882509283,341	95921822174,1215	0,156094813300079	0,00915780278243859	0,0160802510920491	0,89745106862935 7	-0.15609481330008	1,11426687755497	0.15609481330008	1469
Q921G7	sp|Q921G7|ETFD_MOUSE Electron transfer flavoprotein-ubiquinone oxidoreductase, mitochondrial OS=Mus musculus GN=Etfdh PE=1 SV=1	−1,11	0,529400361551305	0,829460904757119	29,1293211094485	29,6742040431969	29,5975087341519	29,3914357617658	29,3179435122904	29,3677682146643	6271340826257,3	5629541763823,71	0,155756433438906	0,0100095579449732	0,0012777634005625	0,89766158781445	-0.155756433438907	1,11400556019637	0.155756433438907	1470
Q8R3T5	sp|Q8R3T5|STXB6_MOUSE Syntaxin-binding protein 6 OS=Mus musculus GN=Stxbp6 PE=2 SV=1	−1,11	0,609814426807318	0,866608542040832	27,840803271956	28,470179165499	28,4367014913091	28,2133967375042	28,1742570180425	28,0364216970831	1855597710990,18	1665853106154,54	0,155622781236145	0,0125349274571712	0,0033033641666266	0,897744751617316	-0.15562278123614	1,11390236278014	0.15562278123614	1471
P46061	sp|P46061|RAGP1_MOUSE Ran GTPase-activating protein 1 OS=Mus musculus GN=Rangap1 PE=1 SV=2	−1,11	0,30033662577104	0,713946294657213	28,4754028990078	28,8326097047498	28,6662319760378	28,5757099628488	28,527812319833	28,5471743747981	2792843941873,36	2507311599489,61	0,155593661004827	0,00623708275609717	0,0008439437162279	0,897762872424507	-0.155593661004824	1,11387987932648	0.155593661004824	1472
Q8BW22	sp|Q8BW22|CREST_MOUSE Calcium-responsive transactivator OS=Mus musculus GN=Ss18l1 PE=1 SV=1	−1,11	0,697362162023655	0,911796014563862	23,1840498105516	22,7738720080143	22,7080917274642	23,0567184762801	22,9695985789723	22,3165440216705	8718135053,74111	7827849749,45875	0,155403488210674	0,0112680700977285	0,0177579314062474	0,89788122129396	-0.155403488210673	1,11373305987943	0.155403488210673	1473
Q9JLQ2	sp|Q9JLQ2|GIT2_MOUSE ARF GTPase-activating protein GIT2 OS=Mus musculus GN=Git2 PE=1 SV=2	−1,11	0,735812760019773	0,924701393764781	24,4554828608451	24,1454988690372	23,5278189761452	24,0841927204206	24,1126737547988	23,6104580044563	27651161428,3864	24841334752,3243	0,154597385840486	0,0196423747409869	0,0117853842949415	0,898383050442953	-0.154597385840489	1,11311093804246	0.154597385840489	1474
Q9CZD3	sp|Q9CZD3|SYG_MOUSE Glycine--tRNA ligase OS=Mus musculus GN=Gars PE=1 SV=1	−1,11	0,408557677098344	0,76291790520755	30,836402611215	30,9382317000035	30,5561361051944	30,6302391026061	30,5898070211484	30,7896398570902	23240637347737,6	20881721585319,6	0,154408974300296	0,00642917172925407	0,0034448741250142	0,898500384170934	-0.154408974300299	1,11296557866552	0.154408974300299	1475
Q9D7A8	sp|Q9D7A8|ARMC1_MOUSE Armadillo repeat-containing protein 1 OS=Mus musculus GN=Armc1 PE=1 SV=1	−1,11	0,578570013254597	0,852070158615807	26,9881320866984	26,9509270425352	26,8410458836077	26,6246811115036	27,1940541511622	26,6409567083219	494444956776,497	444358418971,098	0,154086102172831	0,00284019377699173	0,0120854858433139	0,898701489177006	-0.154086102172832	1,11271652717051	0.154086102172833	1476
Q9CQL4	sp|Q9CQL4|RM20_MOUSE 39S ribosomal protein L20, mitochondrial OS=Mus musculus GN=Mrpl20 PE=1 SV=1	−1,11	0,680951995868481	0,904517472334246	23,871854383327	23,8772170718654	23,7983315076674	23,2262234311461	24,0132687336335	23,9878581132619	22779670284,3895	20474582846,4968	0,1539128070702	0,0018482078677378	0,0188373947580118	0,898809446795534	-0.153912807070201	1,11258287678799	0.153912807070201	1477
Q0VE82	sp|Q0VE82|CPNE7_MOUSE Copine-7 OS=Mus musculus GN=Cpne7 PE=2 SV=1	−1,11	0,556784632113062	0,841293423821966	25,3674906455573	25,4392340632725	25,8903660723575	25,6104311364534	25,4105254331557	25,3572525299008	126777184153,655	113993017466,364	0,153349673595434	0,0110871507684382	0,0052428133944226	0,899160351504601	-0.153349673595432	1,11214868218629	0.153349673595433	1478
Q9DCD2	sp|Q9DCD2|SYF1_MOUSE Pre-mRNA-splicing factor SYF1 OS=Mus musculus GN=Xab2 PE=2 SV=1	−1,11	0,775427317875892	0,938619589104496	24,3957976508923	24,1023003631839	23,1629885154419	23,7897040107311	23,735889620616	23,8166383799036	23659459320,193	21273840381,2306	0,15333661075649	0,0269594248907886	0,0017288475245314	0,89916849296187	-0.153336610756494	1,11213861231502	0.153336610756494	1479
Q6PD24	sp|Q6PD24|AN13D_MOUSE Ankyrin repeat domain-containing protein 13D OS=Mus musculus GN=Ankrd13d PE=2 SV=2	−1,11	0,655432525846701	0,892226633215307	25,3954169770848	24,7545101478558	24,6663146621165	24,7487693428392	24,9546627427534	24,7940826638887	67726763408,2282	60900359605,3013	0,153275305502577	0,0159566346180683	0,0043568173735288	0,899206702647516	-0.153275305502576	1,1120913545859	0.153275305502576	1480
Q91XU0-2	sp|Q91XU0-2|WRIP1_MOUSE Isoform 2 of ATPase WRNIP1 OS=Mus musculus GN=Wrnip1;sp|Q91XU0|WRIP1_MOUSE ATPase WRNIP1 OS=Mus musculus GN=Wrnip1 PE=1 SV=2;sp|Q96S55-3|WRIP1_HUMAN Isoform 3 of ATPase WRNIP1 OS=Homo sapiens GN=WRNIP1;sp|Q96S55-2|WRIP1_HUMAN Is	−1,11	0,647573982793044	0,889714023663571	26,4967677565846	26,7568583343213	25,9701667726243	26,2893784167721	26,3183357187085	26,2985172198808	294319266192,616	264756697472,313	0,152714804337433	0,01517722310483	0,0005628164862953	0,899556121137663	-0.152714804337435	1,1116593801 1217	0.152714804337436	1481

P31938	sp|P31938|MP2K1_MOUSE Dual specificity mitogen-activated protein kinase kinase 1 OS=Mus musculus GN=Map2k1 PE=1 SV=2	−1,11	0,481495119957113	0,802303567138125	33,2755034398491	32,7587169966684	33,0667878409893	32,9187789512132	32,9459102626137	32,9187789512132	221993546117535	199697077477732	0,152704515179668	0,00787015659304587	0,0004757153266092	0,899562536705468	-0.152704515179668	1,11165145189613	0.152704515179668	1482
Q9JMC3	sp|Q9JMC3|DNJA4_MOUSE DnaJ homolog subfamily A member 4 OS=Mus musculus GN=Dnaja4 PE=2 SV=1	−1,11	0,145684175148393	0,541129681668214	27,4758239742999	27,3089720088055	27,5436012651194	27,3712277656425	27,2998217180003	27,3399629165513	828430747691,8	745263834870,019	0,15262984876903	0,00440008280287714	0,0013093920503027	0,899609094600239	-0.15262984876903	1,111593920073	0.15262984876903	1483
Q64008	sp|Q64008|RAB34_MOUSE Ras-related protein Rab-34 OS=Mus musculus GN=Rab34 PE=1 SV=2	−1,11	0,749574077925117	0,928854093158237	22,0428427182337	22,9695985789723	22,5136465441664	22,7467657754392	22,080652069264	22,383010120132	5962143774,89759	5366683668,34564	0,15180031282926	0,0205874993855086	0,0148873379845491	0,900126510021611	-0.15180031282926	1,1109549478506	0.15180031282926	1484
P63044	sp|P63044|VAMP2_MOUSE Vesicle-associated membrane protein 2 OS=Mus musculus GN=Vamp2 PE=1 SV=2	−1,11	0,575053087353321	0,849998193752035	33,5634667763458	33,404240792523	33,2027166374192	32,9992079572545	33,3243876702912	33,5323458377698	317069275964538	285514468577942	0,151234230282069	0,00541440916323135	0,0080728744025012	0,900479769632031	-0.151234230282068	1,11051911850128	0.151234230282068	1485
O35127	sp|O35127|C10_MOUSE Protein C10 OS=Mus musculus GN=Grcc10 PE=2 SV=1	−1,11	0,521392056541083	0,823916573667872	27,311293764426	27,0605273992974	26,8570170371907	26,8427056892879	26,9096572194275	27,1620716602265	574220554242,353	517087627650,782	0,151196186894594	0,00840394800300583	0,0062443447957510	0,900503515296567	-0.151196186894594	1,11048983486829	0.151196186894594	1486
Q8CAB8	sp|Q8CAB8|GATL2_MOUSE GATS-like protein 2 OS=Mus musculus GN=Gatsl2 PE=2 SV=1	−1,11	0,276510289898764	0,698819713725218	25,7219669464862	25,4184137485655	25,5223313428076	25,4944981642208	25,4761717842147	25,3783577930722	12533266338,834	112889569169,235	0,150850259583765	0,00603701403526019	0,0024534429934406	0,90071946260362	-0.150850259583768	1,11022359515736	0.150850259583768	1487
Q3UMG5-2	sp|Q3UMG5-2|LRCH2_MOUSE Isoform 2 of Leucine-rich repeat and calponin homology domain-containing protein 2 OS=Mus musculus GN=Lrch2;sp| Q3UMG5-3|LRCH2_MOUSE Isoform 3 of Leucine-rich repeat and calponin homology domain-containing protein 2 OS=Mus musculus	−1,11	0,709343658426461	0,916805961322165	23,7308311655272	22,8515326580154	23,1840498105516	22,9837162468125	23,1009237110647	23,3681777993606	12581221868,512	11332485399,5939	0,150807738794677	0,0190913723746433	0,0085115484716607	0,900746010048244	-0.150807738794675	1,11019087383628	0.150807738794675	1488
Q9DB29	sp|Q9DB29|IAH1_MOUSE Isoamyl acetate-hydrolyzing esterase 1 homolog OS=Mus musculus GN=Iah1 PE=1 SV=1	−1,11	0,495558124256685	0,80882356202841	26,2029568269526	26,3515957296419	26,6160829578104	26,4656131249857	26,1456344965372	26,2458051503349	289150120455,379	260451957588,187	0,150801422527001	0,00792917943129312	0,0062267539420326	0,900749953615805	-0.150801422526998	1,11018601331677	0.150801422526998	1489
Q8VE97	sp|Q8VE97|SRSF4_MOUSE Serine/arginine-rich splicing factor 4 OS=Mus musculus GN=Srsf4 PE=2 SV=1	−1,11	0,419441648388522	0,769072485508496	27,1789632879833	26,7811512810363	26,8090056281754	26,8060735616088	26,8505074822295	26,7995725862334	492637805101,092	443834630730,304	0,15050510478428	0,00824844291043552	0,0010336747459157	0,900934979277984	-0.15050510478428	1,10995801361981	0.15050510478428	1490
P62702	sp|P62702|RS4X_MOUSE 40S ribosomal protein S4, X isoform OS=Mus musculus GN=Rps4x PE=2 SV=2;sp|P47836|RS4_CHICK 40S ribosomal<br>protein S4 OS=Gallus gallus GN=RPS4 PE=2 SV=2	−1,11	0,649111778735222	0,889714023663571	28,9598110128047	29,5252640648693	29,6976512816979	29,3952184820838	29,2338980175545	29,2410664809566	583189461726,37	5254263685645,78	0,150301594208087	0,0131309918546732	0,0031116207659533	0,901062076636799	-0.150301594208085	1,10980145089724	0.150301594208085	1491
Q8K341-4	sp|Q8K341-4|ATAT_MOUSE Isoform 4 of Alpha-tubulin N-acetyltransferase 1 OS=Mus musculus GN=Atat1;sp|Q8K341-3|ATAT_MOUSE Isoform 3 of<br>Alpha-tubulin N-acetyltransferase 1 OS=Mus musculus GN=Atat1;sp|Q8K341|ATAT_MOUSE Alpha-tubulin N-acetyltransferase 1 OS	−1,11	0,427710813474162	0,773941056137542	27,6110235759658	27,270293282656	27,6736691686645	27,4925739899774	27,3749832819008	27,3749832819008	893425399631,64	805058018624,229	0,150254511725768	0,00788843980945729	0,0024764930596157	0,901091483358493	-0.150254511725768	1,10976523301814	0.150254511725767	1492
P58137	sp|P58137|ACOT8_MOUSE Acyl-coenzyme A thioesterase 8 OS=Mus musculus GN=Acot8 PE=1 SV=1	−1,11	0,539335865790252	0,831485169227261	23,8119599107002	24,0011855134086	24,3480812542672	23,871854383327	24,0770833167058	23,8999487036671	27951654397,6615	25187880953,2398	0,150203921781699	0,0113037450643773	0,0046459634909595	0,901123081836153	-0.150203921781701	1,10972631836527	0.150203921781701	1493
Q9EST5	sp|Q9EST5|AN32B_MOUSE Acidic leucine-rich nuclear phosphoprotein 32 family member B OS=Mus musculus GN=Anp32b PE=1 SV=1;sp| Q9EST5-2|AN32B_MOUSE Isoform 2 of Acidic leucine-rich nuclear phosphoprotein 32 family member B OS=Mus musculus GN=Anp32b	−1,11	0,511042129347761	0,817055380454204	27,0846207130066	27,4140252679283	27,6254440730023	27,2928682782776	27,2789110284764	27,2405304600518	773890853657,238	697501096210,481	0,149934624697098	0,00995620951156411	0,0009938124130937	0,901291303436711	-0.149934624697099	1,10951919339164	0.149934624697099	1494
A2A8Z1	sp|A2A8Z1|OSBL9_MOUSE Oxysterol-binding protein-related protein 9 OS=Mus musculus GN=Osbpl9 PE=1 SV=1;sp|A2A8Z1-2|OSBL9_MOUSE<br>Isoform 2 of Oxysterol-binding protein-related protein 9 OS=Mus musculus GN=Osbpl9	−1,11	0,557593718043491	0,841889439813753	25,3877731654233	25,7487841328276	25,4204801455624	25,6522026352665	25,4069322364685	25,1861283043439	120994688062,684	109051678921,161	0,149931729979076	0,00782392924431025	0,0091734552935624	0,901293111848549	-0.149931729979083	1,10951696718175	0.149931729979083	1495
Q8BGS2	sp|Q8BGS2|BOLA2_MOUSE BolA-like protein 2 OS=Mus musculus GN=Bola2 PE=1 SV=1;sp|Q8BGS2-2|BOLA2_MOUSE Isoform 2 of BolA-like<br>protein 2 OS=Mus musculus GN=Bola2	−1,11	0,556184451226153	0,840946443742674	26,5887417463319	26,517535165276	26,3833279274518	26,1062400879502	26,3932386786055	26,6793021818755	321587320237,982	289936271639,483	0,149474695199863	0,00393654036658289	0,0108563621723615	0,901578679858781	-0.149474695199861	1,10916553634191	0.149474695199861	1496
Q9R0D8	sp|Q9R0D8|WDR54_MOUSE WD repeat-containing protein 54 OS=Mus musculus GN=Wdr54 PE=2 SV=1	−1,11	0,627360431736576	0,876847454152056	27,3163381242936	27,7954933381178	28,0390196313671	27,6060239625494	27,6060239625494	27,6294369835375	1089729225779,35	982954139976,644	0,148773687041156	0,0132657890450551	0,0004895197370577	0,902016865036961	-0.148773687041155	1,10862672169552	0.148773687041155	1497
P47199	sp|P47199|QOR_MOUSE Quinone oxidoreductase OS=Mus musculus GN=Cryz PE=1 SV=1	−1,11	0,398381843182979	0,754338506152154	29,0477196217778	29,2004756920356	29,441616430095	29,0789957161334	29,0919148705394	29,209851847461	4947666899535,41	4463350403002,29	0,148621302200628	0,00679420592384496	0,0024757264557342	0,90211214571082	-0.148621302200628	1,10850962904623	0.148621302200628	1498

O54984	sp|O54984|ASNA_MOUSE ATPase Asna1 OS=Mus musculus GN=Asna1 PE=1 SV=2;sp|O43681|ASNA_HUMAN ATPase ASNA1 OS=Homo sapiens GN=ASNA1 PE=1 SV=2	−1,11	0,230988899562086	0,654468548759245	30,0740303655199	29,7799328650923	29,9008251465763	29,7991526231768	29,8175332931728	29,8297582429479	9847736939655,35	8885851302833,31	0,148282224679415	0,00494086681470724	0,0005166993413867	0,902324194612808	-0.148282224679415	1,10824912594647	0.148282224679415	1499
Q9JMF7	sp|Q9JMF7|DOPP1_MOUSE Dolichyldiphosphatase 1 OS=Mus musculus GN=Dolpp1 PE=2 SV=1	−1,11	0,862940043818199	0,967731774645151	21,8769368165872	22,3326153441959	23,2912926988169	23,1184157525276	22,4202327163321	21,6547649475765	5912186817,86139	5336332585,02549	0,14784327282185	0,0320854285266184	0,0326854856379345	0,902598775956102	-0.14784327282185	1,10791198330701	0.14784327282185	1500
Q8R1X6-2	sp|Q8R1X6-2|SPG20_MOUSE Isoform 2 of Spartin OS=Mus musculus GN=Spg20;sp|Q8R1X6|SPG20_MOUSE Spartin OS=Mus musculus GN=Spg20 PE=1 SV=1	−1,11	0,440845604948713	0,780702789085957	25,1239866486859	25,2961103046975	24,8903374302592	25,0120600285972	24,8891462278101	25,1021464688603	79854984094,9763	72085415202,0694	0,147675061895158	0,00811291394534709	0,0042766447802873	0,902704020532193	-0.147675061895157	1,10778281391773	0.147675061895157	1501
Q91YP0	sp|Q91YP0|L2HDH_MOUSE L-2-hydroxyglutarate dehydrogenase, mitochondrial OS=Mus musculus GN=L2hgdh PE=1 SV=1	−1,11	0,481870489836864	0,802303567138125	28,1954506384403	27,7129019603996	27,9221362330221	27,8851022330226	27,8785137992401	27,7598510714024	1366803991198,22	1233844106053,53	0,147646241571618	0,00865970475711793	0,0025318235137075	0,902722053783199	-0.147646241571613	1,10776068426502	0.147646241571613	1502
Q60710	sp|Q60710|SAMH1_MOUSE Deoxynucleoside triphosphate triphosphohydrolase SAMHD1 OS=Mus musculus GN=Samhd1 PE=1 SV=2	−1,11	0,65556031563 6085	0,892226633215307	26,9731145224765	26,8480719120113	26,3810756525348	26,8602290837401	26,3722038170175	26,6630776319574	407818215713,042	368179653275,306	0,147516315394836	0,0116721639496058	0,0092185888334569	0,902803354753464	-0.147516315394838	1,10766092608626	0.147516315394839	1503
Q8VCH8	sp|Q8VCH8|UBXN4_MOUSE UBX domain-containing protein 4 OS=Mus musculus GN=Ubxn4 PE=1 SV=1	−1,11	0,539485310282944	0,831485169227261	26,5758572324515	26,794197060117	26,9947363166119	26,9150426236507	26,5801731397537	26,5628276441229	430521687233,124	388676987453,31	0,147514222210532	0,00782068496761208	0,0074396167632805	0,902804664618079	-0.147514222210532	1,10765931899902	0.147514222210532	1504
P56812	sp|P56812|PDCD5_MOUSE Programmed cell death protein 5 OS=Mus musculus GN=Pdcd5 PE=1 SV=3	−1,11	0,36256452481021	0,73698180838045	28,1351226080958	27,9689280949635	27,8663186708468	27,7318880781206	27,9366295152886	27,9960381244174	1432042874962,95	1293257363069,37	0,147065281687331	0,00484633772058064	0,0049690687125698	0,903085644766627	-0.147065281687331	1,10731468913828	0.147065281687331	1505
Q3TLS3	sp|Q3TLS3|GDPP1_MOUSE GDP-D-glucose phosphorylase 1 OS=Mus musculus GN=Gdpgp1 PE=2 SV=2	−1,11	0,504987526500809	0,814993646406912	26,446728601505	26,1107739160253	26,1217410182589	26,234878662775	25,9147147066523	26,2250524440376	245463601860,797	221764520595,668	0,146480541154399	0,00727802045017217	0,0069694653873204	0,903451749727975	-0.146480541154396	1,10686597297653	0.146480541154396	1506
Q80X73	sp|Q80X73|PELO_MOUSE Protein pelota homolog OS=Mus musculus GN=Pelo PE=2 SV=3	−1,11	0,744538189556943	0,927403634028255	25,9923002404933	25,3372129119629	25,1070055387849	25,1454481767251	25,6908631453629	25,2961103046975	116230318487,489	105026010512,699	0,146239775612973	0,0180277391723649	0,0110989620830499	0,903602535718802	-0.146239775612974	1,10668126800299	0.146239775612975	1507
Q9CQC9	sp|Q9CQC9|SAR1B_MOUSE GTP-binding protein SAR1b OS=Mus musculus GN=Sar1b PE=1 SV=1	−1,11	0,497001041293266	0,809698318764119	28,7725746006029	28,7461874298344	28,5775854342843	28,3730882269636	28,5775854342843	28,8422515451511	2908860350404,2	2629044887377,22	0,1459152624591 2	0,0036860693920048	0,0082252873738722	0,903805810757433	-0.145915262459123	1,10643236422872	0.145915262459122	1508
Q8R2T8-2	sp|Q8R2T8-2|TF3C5_MOUSE Isoform 2 of General transcription factor 3C polypeptide 5 OS=Mus musculus GN=Gtf3c5;sp|Q8R2T8|TF3C5_MOUSE General transcription factor 3C polypeptide 5 OS=Mus musculus GN=Gtf3c5 PE=2 SV=2	−1,11	0,740765690442586	0,925497610201997	22,6338453533699	22,7708085059644	23,4856512423187	23,1698545542957	22,702675779385 5	22,7144546071874	9394920744,79558	8491472941,99559	0,145866163921744	0,0199189699191093	0,0116519473950281	0,90383657006362	-0.145866163921744	1,10639471019591	0.145866163921744	1509
Q62186	sp|Q62186|SSRD_MOUSE Translocon-associated protein subunit delta OS=Mus musculus GN=Ssr4 PE=2 SV=1	−1,11	0,531695092397333	0,829883431939119	25,4392340632725	25,3853556236696	25,0924879023731	24,9925963663285	25,2118538008189	25,4094933692325	97751319043,4766	88356698045,1085	0,145776664964797	0,00737365720886375	0,0082739460037213	0,903892642162816	-0.145776664964796	1,10632607607826	0.145776664964796	1510
Q99JY9	sp|Q99JY9|ARP3_MOUSE Actin-related protein 3 OS=Mus musculus GN=Actr3 PE=1 SV=3	−1,11	0,393308098408119	0,753881747531964	32,5465656838934	32,2785243609912	32,3520069298941	32,3276858928127	32,42092197952 3	32,1255304366215	116903001311944	105673940501177	0,145692321591644	0,0042758000167079 4	0,0046763408438242	0,9039454874147 9	-0.145692321591648	1,10626139952302	0.145692321591648	1511
Q8BGQ1-2	sp|Q8BGQ1-2|SPE39_MOUSE Isoform 2 of Spermatogenesis-defective protein 39 homolog OS=Mus musculus GN=Vipas39;sp|Q8BGQ1|SPE39_MOUSE Spermatogenesis-defective protein 39 homolog OS=Mus musculus GN=Vipas39 PE=1 SV=1	−1,11	0,636884287082795	0,883588334123886	26,2212886157278	26,6630776319574	26,9015107380824	26,5038887636353	26,3577801409492	26,6219754182725	354967590748,903	320949012411,022	0,1453431879920 1	0,0129774874422267	0,004995181890988	0,904164269571458	-0.145343187992008	1,1059937155822	0.145343187992008	1512
Q69ZS7-2	sp|Q69ZS7-2|HBS1L_MOUSE Isoform 2 of HBS1-like protein OS=Mus musculus GN=Hbs1l;sp|Q69ZS7|HBS1L_MOUSE HBS1-like protein OS=Mus musculus GN=Hbs1l PE=1 SV=2	−1,11	0,790853700276039	0,942055274591853	25,2357243145929	24,5700026587245	24,1206114949404	24,6569155403246	24,8115612153573	24,15586129603 3	50342365839,2174	45521292352,714	0,145231501097605	0,0227676430958527	0,0139666417665691	0,904234268570117	-0.145231501097605	1,10590809788853	0.145231501097605	1513
	sp|Q80ZJ6-2|ZFER1_MOUSE Isoform 2 of Protein zer-1 homolog OS=Mus musculus GN=Zer1;sp|Q80ZJ6|ZFER1_MOUSE Protein zer-1 homolog																			

Q80ZJ6-2	[illegible] OS=Mus musculus GN=Zer1 PE=1 SV=1;sp|Q80ZJ6-3|ZER1_MOUSE Isoform 3 of Protein zer-1 homolog OS=Mus musculus GN=Zer1	−1,11	0,389347871401062	0,753319093070654	28,0563316653839	27,7842060823852	27,7120449619398	27,840803271956	27,6623996166401	27,7477528357763	1245877109270,96	1126705016839,22	0,145051918647782	0,00651915179391241	0,0032154404929471	0,904346832006991		-0.145051918647782	1,10577044625758	0.145051918647782	1514
P97328	sp|P97328|KHK_MOUSE Ketohexokinase OS=Mus musculus GN=Khk PE=1 SV=1	−1,11	0,788109330146619	0,942041082015698	23,2759764901913	22,9259100978125	23,5221721571896	23,5630146461116	23,3727250633184	22,4867335334843	12404844454,3613	11218436907,79	0,145031969513967	0,0128923269045707	0,0248216824530214	0,904359337117348		-0.145031969513968	1,10575515611693	0.145031969513968	1515
P21300	sp|P21300|ALD1_MOUSE Aldose reductase-related protein 1 OS=Mus musculus GN=Akr1b7 PE=2 SV=4	−1,11	0,688334778899295	0,907856236295823	26,9530356848158	27,4854015248497	27,741133248696	27,3476415937145	27,1097993894113	27,4208032405863	788330702123,351	713000582670,97	0,144897706804258	0,0146770321500223	0,0059578589257759	0,90444350416712		-0.144897706804259	1,10565225510782	0.144897706804259	1516
Q3UHD2	sp|Q3UHD2|GFOD1_MOUSE Glucose-fructose oxidoreductase domain-containing protein 1 OS=Mus musculus GN=Gfod1 PE=2 SV=1	−1,11	0,666706450046886	0,897903408907067	26,4376778659567	26,5121165550575	26,0637890078749	26,0234593655428	26,6107194319358	26,0782885894433	274402307758,958	248197125676,244	0,144806206826099	0,00912199693778843	0,0123635089152583	0,904500868463055		-0.144806206826097	1,10558213360173	0.144806206826096	1517
Q9CQI3	sp|Q9CQI3|GMFB_MOUSE Glia maturation factor beta OS=Mus musculus GN=Gmfb PE=1 SV=3	−1,11	0,583545822853372	0,854148497236845	30,4517424809733	30,5053526695314	30,3606214007766	30,0602349868501	30,2860766360239	30,6703686802763	16580341299354,2	14997331997592,7	0,144767834102147	0,00240384821899329	0,0101677040585445	0,904524926647731		-0.144767834102147	1,10555272777955	0.144767834102147	1518
P11499	sp|P11499|HS90B_MOUSE Heat shock protein HSP 90-beta OS=Mus musculus GN=Hsp90ab1 PE=1 SV=3;sp|P08238|HS90B_HUMAN Heat shock protein HSP 90-beta OS=Homo sapiens GN=HSP90AB1 PE=1 SV=4	−1,11	0,276675943725833	0,698849253517838	35,6500093773241	35,6500093773241	35,3817684358028	35,4378163435131	35,505272310377	35,4378163435131	2778244727320040	2513120657879010	0,144693749267363	0,00435507270379417	0,0010982907306982	0,904571376727933		-0.144693749267363	1,10549595723143	0.144693749267363	1519
Q9CQ86	sp|Q9CQ86|MIEN1_MOUSE Migration and invasion enhancer 1 OS=Mus musculus GN=Mien1 PE=1 SV=1	−1,11	0,531944264291357	0,829883431939119	27,1854010346971	26,9261638707406	26,7811512810363	26,6482374292033	26,9742992198464	26,9699393539991	513357709616,606	464468077165,195	0,144385007900984	0,00759512704044382	0,0069611703179802	0,904764978619054		-0.144385007900986	1,10525940286317	0.144385007900986	1520
Q9CW79	sp|Q9CW79|GOGA1_MOUSE Golgin subfamily A member 1 OS=Mus musculus GN=Golga1 PE=1 SV=2;sp|Q9CW79-2|GOGA1_MOUSE Isoform 2 of Golgin subfamily A member 1 OS=Mus musculus GN=Golga1	−1,11	0,581949756716407	0,853379506269256	25,1396857445748	24,7957915615014	24,5700026587245	24,6395384667734	24,882110644788	24,6838243385297	61062269276,8491	55251306089,6767	0,144272637002034	0,0115511527911553	0,0052239865922254	0,904835453120385		-0.144272637002033	1,10517331803416	0.144272637002033	1521
P20917-2	sp|P20917-2|MAG_MOUSE Isoform S-MAG of Myelin-associated glycoprotein OS=Mus musculus GN=Mag;sp|P20917|MAG_MOUSE Myelin-associated glycoprotein OS=Mus musculus GN=Mag PE=1 SV=2	−1,10	0,806334654851864	0,948420056587875	24,9361022925742	24,8730804771037	23,8670266020545	24,8868677141354	24,2514976759772	24,2384213358069	46315219422,0825	41915809537,5113	0,1439918554148	0,0244257888146665	0,0151545289003385	0,905011572017435		-0.143991855414801	1,10495824685514	0.143991855414801	1522
Q8C008	sp|Q8C008|DZAN1_MOUSE Double zinc ribbon and ankyrin repeat-containing protein 1 OS=Mus musculus GN=Dzank1 PE=1 SV=2	−1,10	0,633737945359782	0,882643919603575	28,7923191655361	28,8780890237702	28,5793494389656	28,415569281999	28,5094418456896	29,0254710300426	3061478880497,43	2770809742562,41	0,143921079069616	0,00535016958919	0,0114624046687488	0,905055971548041		-0.143921079069615	1,10490404067448	0.143921079069616	1523
P97355	sp|P97355|SPSY_MOUSE Spermine synthase OS=Mus musculus GN=Sms PE=1 SV=1	−1,10	0,539718753237541	0,831562600456619	29,1075726323578	28,5524438127231	28,7501446235731	28,6868977896118	28,7093526612442	28,7146432611685	3229624598017,6	2922998933934,96	0,143917177103074	0,00976858048365181	0,0005132120491482	0,905058419399316		-0.143917177103074	1,10490105231406	0.143917177103074	1524
P47941	sp|P47941|CRKL_MOUSE Crk-like protein OS=Mus musculus GN=Crkl PE=1 SV=2	−1,10	0,478513284004496	0,799905345588749	28,8167867144637	28,5701797962947	28,527812319833	28,3225587196079	28,6442862543706	28,6488796693929	2738029481306,3	2478252724253,47	0,14381466428645	0,0054491266479702	0,0065556735743206	0,905122731940457		-0.143814664286451	1,10482254473505	0.143814664286451	1525
P70699	sp|P70699|LYAG_MOUSE Lysosomal alpha-glucosidase OS=Mus musculus GN=Gaa PE=1 SV=2	−1,10	0,424446509565096	0,7711031271846	27,9221362330221	28,0674871197023	28,0738882662073	27,8449372025876	27,7741719005353	28,1463259904393	1477201506329,81	1337484461199,17	0,143344508318663	0,00306289571423571	0,0070779171156607	0,905417747997174		-0.143344508318671	1,10446255577831	0.143344508318671	1526
Q8BFV2	sp|Q8BFV2|PCID2_MOUSE PCI domain-containing protein 2 OS=Mus musculus GN=Pcid2 PE=2 SV=1	−1,10	0,342024313190581	0,729721949431128	25,2329334459052	25,0061922723973	25,0264181578287	25,1396857445748	24,9079666938932	24,9200121770116	78668969436,7919	71232964907,6383	0,143249644041873	0,00500143429828544	0,0052200480144518	0,905477285613507		-0.143249644041872	1,10438993433441	0.143249644041872	1527
Q9D2C2	sp|Q9D2C2|SAAL1_MOUSE Protein SAAL1 OS=Mus musculus GN=Saal1 PE=1 SV=1;sp|Q9D2C2-2|SAAL1_MOUSE Isoform 2 of Protein SAAL1 OS=Mus musculus GN=Saal1	−1,10	0,924177746660049	0,983296707494544	21,5621146118181	24,3237320068627	23,8542629495858	23,8343072841257	23,6540441959298	21,9541395813542	12471391639,6617	11293543418,4789	0,143124247973081	0,0635642854750804	0,0448169701759013	0,905555991246639		-0.143124247973084	1,10429394721727	0.143124247973084	1528
Q91ZZ3	sp|Q91ZZ3|SYUB_MOUSE Beta-synuclein OS=Mus musculus GN=Sncb PE=1 SV=1	−1,10	0,513923833398858	0,819539762695211	31,2665446190202	31,1238583488395	30,9631543867588	30,9004869372115	30,8893105450994	31,2665446190202	32682224460136,9	29599562609511	0,1429303239981	0,00487772282802224	0,0069197627740478	0,905677722323158		-0.1429303239981	1,10414552036777	0.1429303239981	1529
Q05816	sp|Q05816|FABP5_MOUSE Fatty acid-binding protein, epidermal OS=Mus musculus GN=Fabp5 PE=1 SV=3	−1,10	0,581646019169815	0,853379506269256	30,295446665439	30,4662615796495	30,392909761289	29,9807891911943	30,2998683979432	30,577500263056	15702996481918,2	14225434174350,2	0,142567198087465	0,00282017326300865	0,0098591702636482	0,905905709826185		-0.142567198087472	1,10386764224267	0.142567198087472	1530
P09411	sp|P09411|PGK1_MOUSE Phosphoglycerate kinase 1 OS=Mus musculus GN=Pgk1 PE=1 SV=4	−1,10	0,239717001264051	0,664270737934997	35,3817684358028	35,1168009278487	35,211446325403	35,1702667677213	35,1702667677213	35,0731894837483	2009519224041370	1820536573337730	0,142486655154675	0,00381059901610191	0,0015950749417262	0,905956286238668		-0.142486655154675	1,10380601712228	0.142486655154675	1531

Q9JIF0-3	sp|Q9JIF0-3|ANM1_MOUSE Isoform 3 of Protein arginine N-methyltransferase 1 OS=Mus musculus GN=Prmt1;sp|Q9JIF0-2|ANM1_MOUSE Isoform 2 of Protein arginine N-methyltransferase 1 OS=Mus musculus GN=Prmt1;sp|Q9JIF0|ANM1_MOUSE Protein arginine N-methyltransfe	−1,10	0,470102330172154	0,796715861328205	30,1781158654775	29,714294747174	29,9023714560052	29,7871965151757	29,8638697170678	29,8475138120678	9979898370521,35	9041624854926,56	0,142443063874784	0,00779401609420273	0,0013536244116250	0,905983660277918	-0.142443063874783	1,10377266593665	0.142443063874783	1532
P61957	sp|P61957|SUMO2_MOUSE Small ubiquitin-related modifier 2 OS=Mus musculus GN=Sumo2 PE=1 SV=1	−1,10	0,701853313760952	0,914121145239874	27,9902351106764	27,6583684625279	27,3594544203214	27,2058942963641	27,7577253480226	27,7483704429287	1039075679841,1	941427674295,4	0,142378566685376	0,011403727899985	0,0114590606943397	0,906024164129573	-0.142378566685376	1,10372332172918	0.142378566685376	1533
B2RSH2	sp|B2RSH2|GNAI1_MOUSE Guanine nucleotide-binding protein G(i) subunit alpha-1 OS=Mus musculus GN=Gnai1 PE=2 SV=1	−1,10	0,517583938881929	0,822013120568606	31,5568375533821	31,0970439805281	31,3178401286448	31,1310784705769	31,3724626453784	31,1726430469682	40160587526610,6	36392896519892,5	0,142123495038404	0,00734125597986067	0,0041328208015816	0,906184365350193	-0.142123495038405	1,10352819827514	0.142123495038405	1534
Q8R3P0	sp|Q8R3P0|ACY2_MOUSE Aspartoacylase OS=Mus musculus GN=Aspa PE=1 SV=2	−1,10	0,759357236576411	0,933136198610489	25,9735589231531	26,4947769969841	27,0716328405969	26,3183357187085	26,4261270803001	26,5000720757695	327031691890,785	296361241681,215	0,142073667393069	0,0207168235272699	0,0034598711965192	0,906215663588309	-0.14207366739307	1,10349008539572	0.14207366739307	1535
Q9CR39	sp|Q9CR39|WIPI3_MOUSE WD repeat domain phosphoinositide-interacting protein 3 OS=Mus musculus GN=Wdr45b PE=2 SV=2	−1,10	0,736374337853838	0,924701393764781	25,7471346094699	25,528533155215	26,2857612704694	25,7580916626282	25,4312369546124	26,0769885460403	169109061556,552	153265730795,603	0,1419188113531	0,0150740669442267	0,0125365351832986	0,906312940210772	-0.141918811353103	1,10337164530327	0.141918811353103	1536
Q9ERY9	sp|Q9ERY9|ERG28_MOUSE Probable ergosterol biosynthetic protein 28 OS=Mus musculus GN=ORF11 PE=2 SV=1	−1,10	0,672804213371575	0,900894908348996	25,3532998395674	25,0570831721089	24,7358047995479	24,6686770330592	25,0023493395826	25,1800505226162	75600540562,812	68517770031,2133	0,14191835165889	0,0123292590247262	0,0104054936515902	0,90631322899452	-0.141918351658888	1,10337129372967	0.141918351658888	1537
Q99L13	sp|Q99L13|3HIDH_MOUSE 3-hydroxyisobutyrate dehydrogenase, mitochondrial OS=Mus musculus GN=Hibadh PE=1 SV=1	−1,10	0,614458861441551	0,868585727836563	29,323260678621	29,7592429065261	29,8931980979023	29,6954546184483	29,5991882691668	29,3868050777055	7595425147392,61	6885801508829,03	0,141506126443507	0,0100479619083325	0,0053423746010822	0,906572229362673	-0.141506126443508	1,10305606945737	0.141506126443508	1538
Q8BMK4	sp|Q8BMK4|CKAP4_MOUSE Cytoskeleton-associated protein 4 OS=Mus musculus GN=Ckap4 PE=1 SV=2	−1,10	0,868021874584979	0,969953542279966	26,0446839509163	28,020951024883	26,6579118643942	26,8916424786853	26,8676217628967	26,6710608514719	485210641869,943	440029833228,076	0,141009853493013	0,0375935958583581	0,0045138252029575	0,906884134965083	-0.141009853493013	1,10267669423779	0.141009853493013	1539
P53395	sp|P53395|ODB2_MOUSE Lipoamide acyltransferase component of branched-chain alpha-keto acid dehydrogenase complex, mitochondrial OS=Mus musculus GN=Dbt PE=1 SV=2	−1,10	0,417142372572074	0,767877733621866	29,3536338319982	28,9668367465018	29,0776180365338	28,9822197969154	29,1030743452272	29,0196626152411	4489202589922,29	4071308594900,96	0,140966625786059	0,00683746789354466	0,0021307811775245	0,906911308489519	-0.140966625786062	1,10264365505104	0.140966625786062	1540
P61027	sp|P61027|RAB10_MOUSE Ras-related protein Rab-10 OS=Mus musculus GN=Rab10 PE=1 SV=1	−1,10	0,336031543424902	0,72953198881961	31,5462290964587	31,8078616968354	31,7308789356197	31,4680190706971	31,6520009366872	31,6726545295256	58204856350771,4	52801366076653,9	0,14056427465989	0,00424224392164001	0,0035654165849542	0,907164270940669	-0.140564274659891	1,10233618323952	0.140564274659891	1541
Q3UUQ7	sp|Q3UUQ7|PGAP1_MOUSE GPI inositol-deacylase OS=Mus musculus GN=Pgap1 PE=1 SV=3	−1,10	0,78342970624053	0,940251150142017	26,0564348417916	25,8593137304936	24,9925963663285	25,7246189853418	25,6181538792891	25,2737175242021	136026421498,54	123416435653,686	0,140352370543259	0,0220763446325086	0,0092284801796548	0,907297525687027	-0.140352370543255	1,10217428317439	0.140352370543255	1542
Q61282	sp|Q61282|PGCA_MOUSE Aggrecan core protein OS=Mus musculus GN=Acan PE=1 SV=2;sp|P16112-3|PGCA_HUMAN Isoform 3 of Aggrecan core protein OS=Homo sapiens GN=ACAN;sp|P16112-2|PGCA_HUMAN Isoform 2 of Aggrecan core protein OS=Homo sapiens GN=ACAN;sp|P16112|P	−1,10	0,483444530576546	0,803263869194775	25,668783905484	26,0981905549866	25,9318139841019	25,8525777820242	25,8716928723719	25,6830722098595	177031965310,735	160642561902,132	0,140155697804052	0,00835953891869083	0,0040237651835525	0,90742121977895	-0.140155697804048	1,10202404154005	0.140155697804048	1543
Q91WG5-2	sp|Q91WG5-2|AAKG2_MOUSE Isoform B of 5-AMP-activated protein kinase subunit gamma-2 OS=Mus musculus GN=Prkag2;sp|Q91WG5|AAKG2_MOUSE 5-AMP-activated protein kinase subunit gamma-2 OS=Mus musculus GN=Prkag2 PE=1 SV=2	−1,10	0,851510328334978	0,962641439679318	23,5605386807846	24,8439697910874	24,6220110958197	24,2494398701144	24,8152314567564	23,6723843197498	37296793294,0116	33866264743,4318	0,139202721148499	0,0281795904961018	0,0235684335117155	0,908020817673604	-0.139202721148498	1,1012963365334	0.139202721148498	1544
P63242	sp|P63242|IF5A1_MOUSE Eukaryotic translation initiation factor 5A-1 OS=Mus musculus GN=Eif5a PE=1 SV=2	−1,10	0,215518964447598	0,646799042967852	30,8138966768348	30,8665321302372	30,7592274156069	30,6173890438023	30,6775354171231	30,8553810226389	24099657408109,5	21883816227503,8	0,139148292132687	0,00174131660821881	0,0040287978731873	0,908055075510738	-0.139148292132688	1,10125478835912	0.139148292132688	1545
Q62419	sp|Q62419|SH3G1_MOUSE Endophilin-A2 OS=Mus musculus GN=Sh3gl1 PE=1 SV=1	−1,10	0,345441960651479	0,730303241451822	29,6461657797128	29,4440052105859	29,4486387307347	29,4546675630231	29,2687031531706	29,5272518601179	6566069853877,88	5964666092376,86	0,138588721512697	0,00391025589439875	0,0045332259524968	0,908407346421111	-0.138588721512697	1,10082773321874	0.138588721512697	1546
Q99M31	sp|Q99M31|HSP7E_MOUSE Heat shock 70 kDa protein 14 OS=Mus musculus GN=Hspa14 PE=2 SV=2;sp|Q99M31-2|HSP7E_MOUSE Isoform 2 of Heat shock 70 kDa protein 14 OS=Mus musculus GN=Hspa14	−1,10	0,651890641782127	0,891901943526937	24,53802616775	25,109689615665	25,0452755773747	24,7637322819112	24,6380315993696	25,0061922723973	65000694209,4175	59109178343,807	0,137072959926217	0,0125761245392027	0,0075451880294205	0,909362262399393	-0.137072959926218	1,0996717604725	0.137072959926218	1547

Q8BL48-2	sp|Q8BL48-2|UNK_MOUSE Isoform 2 of RING finger protein unkempt homolog OS=Mus musculus GN=Unk;sp|Q8BL48|UNK_MOUSE RING finger protein unkempt homolog OS=Mus musculus GN=Unk PE=2 SV=1	−1,10	0,642292304019853	0,887748102297842	23,7871003649615	23,9648611641733	23,3156309902351	23,6204012521585	23,4721841914717	23,6905689153765	19412781115,1353	17656764175,8927	0,136785841131804	0,0141627893714965	0,0047253897780098	0,909543257669891	-0.136785841131808	1,09945293043219	0.136785841131807	1548
Q923D4	sp|Q923D4|SF3B5_MOUSE Splicing factor 3B subunit 5 OS=Mus musculus GN=Sf3b5 PE=2 SV=1	−1,10	0,659507210322252	0,89341619202324	24,1698893423587	23,9835441013259	24,0912088731561	23,6005714162421	24,0459906620689	24,3139660285988	28739762072,5725	26142879666,7875	0,136630053904478	0,0038846155285453	0,0150230914088612	0,909641478616717	-0.136630053904475	1,09933421409135	0.136630053904476	1549
Q8CFE4	sp|Q8CFE4|SCYL2_MOUSE SCY1-like protein 2 OS=Mus musculus GN=Scyl2 PE=1 SV=1	−1,10	0,71132730459358	0,916805961322165	28,2826760859385	27,4525390811037	27,8950448565735	27,7989733088988	27,9268847299193	27,6204662728737	1278557162408,87	1163097829199,02	0,13654421450796	0,0149002528505031	0,0055396748951831	0,909695603290573	-0.13654421450796	1,0992688063818	0.13654421450796	1550
O08739	sp|O08739|AMPD3_MOUSE AMP deaminase 3 OS=Mus musculus GN=Ampd3 PE=2 SV=2	−1,10	0,580773089387418	0,853379506269256	26,385525166963	26,69891974877	26,2250524440376	26,3533892025814	26,5089478029815	26,1633273553353	302848682469,139	275509547551,828	0,13649481997126	0,00911660135420529	0,0065711699537486	0,909726749694224	-0.13649481997126	1,09923117060823	0.13649481997126	1551
Q9Z0X1	sp|Q9Z0X1|AIFM1_MOUSE Apoptosis-inducing factor 1, mitochondrial OS=Mus musculus GN=Aifm1 PE=1 SV=1	−1,10	0,441005056447762	0,780702789085957	27,8449372025876	28,2458362215109	27,9817500837541	27,9101208805373	27,8808173603113	27,9977571851328	1481645636618,92	1347894878398,37	0,136492455393539	0,00727189343586978	0,002178567653147	0,909728240737936	-0.136492455393537	1,0992293689694	0.136492455393537	1552
Q8BYB9	sp|Q8BYB9|PGLT1_MOUSE Protein O-glucosyltransferase 1 OS=Mus musculus GN=Poglut1 PE=1 SV=2	−1,10	0,290625249245871	0,709970241239482	26,0346229337524	25,8007350073515	25,7670259217681	25,7281474875069	25,7624652702272	25,8289851792492	171433502463,511	156012085496,673	0,135991284304291	0,00563426309273333	0,0019892234649212	0,910044321878564	-0.135991284304297	1,09884757913301	0.135991284304297	1553
Q9D892	sp|Q9D892|ITPA_MOUSE Inosine triphosphate pyrophosphatase OS=Mus musculus GN=Itpa PE=1 SV=2	−1,10	0,402393179431741	0,757239901094806	29,6926508385983	29,763614849229	29,6034944632424	29,3880565211407	29,7016909671295	29,6878677659393	7811254677639,78	7110107065504,95	0,135683014504102	0,0027026432973201	0,0059887172140778	0,910238797597791	-0.135683014504103	1,09861280648452	0.135683014504103	1554
Q8BG73	sp|Q8BG73|SH3L2_MOUSE SH3 domain-binding glutamic acid-rich-like protein 2 OS=Mus musculus GN=Sh3bgrl2 PE=1 SV=1	−1,10	0,531361811986184	0,829883431939119	28,9822197969154	28,995304264796	28,8167867144637	28,5750404298675	28,8917477037687	29,0458241396507	3670822713146,9	3341822472084,97	0,13546834438087	0,00343934856982656	0,0083234533189152	0,91037424937913	-0.135468344380869	1,09844934726789	0.135468344380868	1555
Q9WV98	sp|Q9WV98|TIM9_MOUSE Mitochondrial import inner membrane translocase subunit Tim9 OS=Mus musculus GN=Timm9 PE=1 SV=1	−1,10	0,706718977415587	0,916805961322165	26,7080610722193	26,0446839509163	26,4376778659567	26,2847000887215	26,584740539218	26,0401650290287	291063597892,277	265054654657,028	0,135044542727241	0,0126368199993124	0,0103697942854248	0,910641717399251	-0.135044542727243	1,09812671755908	0.135044542727243	1556
Q8R3V5-1	sp|Q8R3V5-1|SHLB2_MOUSE Isoform 1 of Endophilin-B2 OS=Mus musculus GN=Sh3glb2;sp|Q8R3V5|SHLB2_MOUSE Endophilin-B2 OS=Mus musculus GN=Sh3glb2 PE=2 SV=2	−1,10	0,512671219948588	0,819082952251546	31,1864065676514	31,4825297714827	31,1310784705769	31,2428831501822	31,2859805099124	30,9907657784686	37926515591934,4	34542439446262,6	0,134836861497568	0,00604395956034372	0,0051154127715202	0,910772817042242	-0.134836861497566	1,09796864957776	0.134836861497566	1557
Q3U5Q7	sp|Q3U5Q7|CMPK2_MOUSE UMP-CMP kinase 2, mitochondrial OS=Mus musculus GN=Cmpk2 PE=2 SV=2	−1,10	0,375266763718619	0,746362141983349	29,4883704069559	29,2251086014483	29,2802995314596	29,2631523510523	29,1101735891658	29,3411392654344	5475252521894,33	4988493461332,14	0,134321320706931	0,00473325110890402	0,0040185298834135	0,911098335900354	-0.134321320706931	1,09757636535665	0.134321320706931	1558
P60123	sp|P60123|RUVB1_RAT RuvB-like 1 OS=Rattus norvegicus GN=Ruvbl1 PE=1 SV=1;sp|P60122|RUVB1_MOUSE RuvB-like 1 OS=Mus musculus GN=Ruvbl1 PE=1 SV=1	−1,10	0,296184501471033	0,711969538636193	29,1143759145037	28,8443921491069	29,0935725463537	28,9450977968638	28,9439646135663	28,8842531253939	4000525606288,31	3645222463151,08	0,13418269691528	0,00517724095475885	0,0012033484254994	0,911185884530088	-0.13418269691528	1,09747090794291	0.13418269691528	1559
P70451-2	sp|P70451-2|FER_MOUSE Isoform 2 of Tyrosine-protein kinase Fer OS=Mus musculus GN=Fer;sp|P70451-5|FER_MOUSE Isoform 5 of Tyrosine-protein kinase Fer OS=Mus musculus GN=Fer;sp|P70451|FER_MOUSE Tyrosine-protein kinase Fer OS=Mus musculus GN=Fer PE=1 SV=2;	−1,10	0,610055883685051	0,866610575848175	24,5199292078442	24,4418972929147	23,9648611641733	24,2523651368066	24,2860801827562	24,1103167979477	36076080343,5377	32884065272,6294	0,133653936333935	0,0123611761718047	0,0038519675635921	0,911519903478647	-0.133653936333936	1,09706874878287	0.133653936333936	1560
Q9DCR2	sp|Q9DCR2|AP3S1_MOUSE AP-3 complex subunit sigma-1 OS=Mus musculus GN=Ap3s1 PE=1 SV=2	−1,10	0,711440453024569	0,916805961322165	27,7411133248696	27,3151166099119	27,2584776827915	26,9594848677855	27,6204662728737	27,4568631562061	824659056095,219	751701218124,785	0,133638438560331	0,0096151921356755	0,0125888531458007	0,911529695295058	-0.133638438560329	1,09705696387248	0.13363843856033	1561
P04627	sp|P04627|ARAF_MOUSE Serine/threonine-protein kinase A-Raf OS=Mus musculus GN=Araf PE=2 SV=2	−1,10	0,790951391720058	0,942055274591853	26,8162660140065	27,1500360627144	27,5020965064079	27,4953994502371	27,173042131448	26,5234452786041	621954552964,667	567191194060,753	0,132974030429481	0,0126290401650485	0,0182927118859659	0,911949581134387	-0.132974030429482	1,09655184967143	0.132974030429481	1562
Q8BM85	sp|Q8BM85|TBCK_MOUSE TBC domain-containing protein kinase-like protein OS=Mus musculus GN=Tbck PE=2 SV=1;sp|Q8BM85-2|TBCK_MOUSE Isoform 2 of TBC domain-containing protein kinase-like protein OS=Mus musculus GN=Tbck	−1,10	0,436536269969472	0,778450481049573	27,268187615531	27,0177302526787	26,8651963070805	26,9469309955916	26,9881320866984	26,9395921810098	559534705219,194	510276422686,136	0,1329486337794	0,0075218513079257	0,0009705603181110	0,911965634886291	-0.1329486337794	1,09653254656321	0.1329486337794	1563
Q8BM72	sp|Q8BM72|HSP13_MOUSE Heat shock 70 kDa protein 13 OS=Mus musculus GN=Hspa13 PE=2 SV=1	−1,10	0,537465408430876	0,831425511350952	26,0204577171094	26,4722972144417	26,4067100219599	26,3021104634812	26,1377623120407	26,1835363389388	264160220323,295	240937412648,005	0,132754796668774	0,00928328254954419	0,0032363958873001	0,912088172674642	-0.132754796668774	1,09638522892755	0.132754796668774	1564
Q8C525	sp|Q8C525|M21D2_MOUSE Protein MB21D2 OS=Mus musculus GN=Mb21d2 PE=1 SV=1	−1,10	0,768018962774078	0,935597979611256	25,270357393125	26,0255942843937	26,0926202587236	26,0333327348677	25,5630102279109	25,5170328077361	159640563256,757	145648000212,971	0,132341381188967	0,0177009253261848	0,0111163448013855	0,912349576083109	-0.132341381188959	1,09607109622738	0.132341381188959	1565
O89023	sp|O89023|TPP1_MOUSE Tripeptidyl-peptidase 1 OS=Mus musculus GN=Tpp1 PE=1 SV=2	−1,10	0,708770629996194	0,916805961322165	28,3139767888204	27,8080490843163	27,5924349407401	27,9268847299193	27,572598641113	27,9400277759159	1314950674925,31	1199793269321,66	0,132222840323982	0,0132728698103056	0,0074944657709262	0,912424543521231	-0.132222840323978	1,09598103985761	0.132222840323978	1566

Q9WV18	sp|Q9WV18|GABR1_MOUSE Gamma-aminobutyric acid type B receptor subunit 1 OS=Mus musculus GN=Gabbr1 PE=1 SV=1;sp|Q9WV18-2|GABR1_MOUSE Isoform 1B of Gamma-aminobutyric acid type B receptor subunit 1 OS=Mus musculus GN=Gabbr1	−1,10	0,557294963467809	0,841784305883656	25,3600660664926	24,9274050418198	25,1293167074687	24,974920529059	25,2329334459052	24,9340519903573	82736713131,6918	75492714242,1113	0,132190227496192	0,00861176801685406	0,0064699601521719	0,912445169557917	-0.132190227496192	1,0959562649496	0.132190227496192	1567
Q8VEJ9	sp|Q8VEJ9|VPS4A_MOUSE Vacuolar protein sorting-associated protein 4A OS=Mus musculus GN=Vps4a PE=1 SV=1	−1,10	0,558626228627431	0,842673463522735	26,5038887636353	26,3678330475435	26,2158504682045	26,269865312127	26,501323798867	26,0415130488842	281254022652,655	256629877890,674	0,132184586099487	0,00546580885762252	0,0087513985947478	0,912448737515867	-0.132184586099484	1,09595197942023	0.132184586099484	1568
Q63850	sp|Q63850|NUP62_MOUSE Nuclear pore glycoprotein p62 OS=Mus musculus GN=Nup62 PE=1 SV=2	−1,10	0,804831228073771	0,947633967374021	24,2934651158925	25,3711772595212	25,149119368486	25,1035425680548	24,9148170158252	24,520588767233	67670798112,0862	61747301866,9593	0,132157306314403	0,0228187212401453	0,0119716992354971	0,912465991086502	-0.132157306314403	1,09593125636307	0.132157306314403	1569
Q8VCY8-2	sp|Q8VCY8-2|LPPR2_MOUSE Isoform 2 of Lipid phosphate phosphatase-related protein type 2 OS=Mus musculus GN=Lppr2;sp|Q8VCY8|LPPR2_MOUSE Lipid phosphate phosphatase-related protein type 2 OS=Mus musculus GN=Lppr2 PE=2 SV=1	−1,10	0,581043991380972	0,853379506269256	23,4769036420011	22,9837162468125	23,3927420340408	23,316746816814	23,1651192385014	23,0974215930066	12951195977,2701	11820437748,2083	0,131801865567714	0,011304845165634	0,0048422687462712	0,912690825538713	-0.131801865567711	1,09566128202259	0.131801865567711	1570
Q8CE90-2	sp|Q8CE90-2|MP2K7_MOUSE Isoform 2 of Dual specificity mitogen-activated protein kinase kinase 7 OS=Mus musculus GN=Map2k7;sp|Q8CE90-8|MP2K7_MOUSE Isoform 8 of Dual specificity mitogen-activated protein kinase kinase 7 OS=Mus musculus GN=Map2k7;sp|Q8CE90	−1,10	0,454417781808701	0,788460969617685	27,2058942963641	26,8832357761076	26,8403763238565	26,9293606494445	26,9195750813523	26,8071250785447	519691982727,155	474417514452,076	0,131499530766173	0,00740688796176743	0,0025264439652105	0,912882111366246	-0.131499530766174	1,09543169654554	0.131499530766174	1571
Q8VEB6	sp|Q8VEB6|RNZ1_MOUSE Zinc phosphodiesterase ELAC protein 1 OS=Mus musculus GN=Elac1 PE=2 SV=1	−1,10	0,343156567101058	0,729891123744941	24,7509761402325	24,473229654249	24,4591970269996	24,4958624117327	24,4630513056042	24,4516913640593	46426408074,9771	42390990728,9452	0,131187982262064	0,0066999155692881	0,0009373534270523	0,913079268602584	-0.131187982262064	1,09519516474232	0.131187982262064	1572
Q8BHE3	sp|Q8BHE3|ATCAY_MOUSE Caytaxin OS=Mus musculus GN=Atcay PE=1 SV=1	−1,10	0,461559258516092	0,793320318823225	29,3002269215392	29,3880565211407	29,5620727820686	29,5072210768158	29,2928257170814	29,1775307111958	5964136549838,89	5445763965586,47	0,131178835368986	0,00453027049035315	0,0057051736169708	0,913085057674204	-0.131178835368987	1,09518822107021	0.131178835368987	1573
Q99LD9	sp|Q99LD9|EI2BB_MOUSE Translation initiation factor eIF-2B subunit beta OS=Mus musculus GN=Eif2b2 PE=2 SV=1	−1,10	0,59276541033383	0,860080030359509	26,4055239012705	25,8385679589886	26,140845402906	26,0769885460403	26,0883849331964	25,9468628589073	222526586445,043	203190970005,639	0,13114142405790 8	0,0108574055078558	0,0030196821825216	0,913108735687278	-0.131141424057913	1,09515982151602	0.131141424057913	1574
P58389	sp|P58389|PTPA_MOUSE Serine/threonine-protein phosphatase 2A activator OS=Mus musculus GN=Ppp2r4 PE=1 SV=1	−1,09	0,61826104804346	0,870166479711399	30,8709415115779	30,279068162713	30,3961101691837	30,4045709486719	30,4090370110476	30,4606427564896	17891972798080,6	16341847069751,2	0,130741413892294	0,0102716302278237	0,0010242956168983	0,91336194472106	-0.130741413892293	1,09485621311429	0.130741413892293	1575
P97352	sp|P97352|S10AD_MOUSE Protein S100-A13 OS=Mus musculus GN=S100a13 PE=1 SV=1	−1,09	0,681521040731982	0,904517472334246	26,2771103257199	26,3290904224167	26,147377349823	25,7334731810382	26,3789076381336	26,3698715068758	251621723464,637	229863335399,039	0,130480115200951	0,00356509291229028	0,014145650323088	0,913527386403679	-0.130480115200953	1,09465793240938	0.130480115200953	1576
Q8R050-2	sp|Q8R050-2|ERF3A_MOUSE Isoform 2 of Eukaryotic peptide chain release factor GTP-binding subunit ERF3A OS=Mus musculus GN=Gspt1;sp|Q8R050|ERF3A_MOUSE Eukaryotic peptide chain release factor GTP-binding subunit ERF3A OS=Mus musculus GN=Gspt1 PE=1 SV=2	−1,09	0,56538385671546	0,845110794140252	29,5120517470896	29,4911659736167	29,0919148705394	29,3037700876513	29,3914357617658	29,1293211094485	5663392425641,02	5174906149855,5	0,130133801290401	0,00806289762024655	0,0045578473006642	0,913746701787095	-0.130133801290401	1,09439519512816	0.130133801290401	1577
Q91VC3	sp|Q91VC3|IF4A3_MOUSE Eukaryotic initiation factor 4A-III OS=Mus musculus GN=Eif4a3 PE=2 SV=3	−1,09	0,460891139195245	0,792808510675944	28,525661745392	28,1113694390224	28,2559271319953	28,2346990589835	28,1925735549002	28,1954506384403	1947665851186,25	1779893091442,36	0,129955595610194	0,00743080522047253	0,0008343374187501	0,913859577277229	-0.129955595610189	1,0942600207566	0.129955595610189	1578
Q9JJT9	sp|Q9JJT9|PHAX_MOUSE Phosphorylated adapter RNA export protein OS=Mus musculus GN=Phax PE=1 SV=1	−1,09	0,910340728199373	0,980429909122681	21,2017394076376	22,8505765759992	22,8687955606518	21,0636421850033	22,8254908632616	22,7620598330115	4873300091,60972	4453981685,08806	0,129803438857087	0,0429128838591705	0,0449832705986584	0,913955964410319	-0.129803438857089	1,09414461849395	0.129803438857089	1579
Q925E7	sp|Q925E7|2ABD_MOUSE Serine/threonine-protein phosphatase 2A 55 kDa regulatory subunit B delta isoform OS=Mus musculus GN=Ppp2r2d PE=2 SV=1	−1,09	0,5514956088921	0,838423390503939	26,9324340242253	26,4776551762052	26,8446549836125	26,5491925988097	26,7414467689503	26,6946283395729	415015370848,769	379361685568,811	0,129590792195321	0,00901826749043784	0,0037602174222965	0,91409068727493	-0.12959079219532	1,09398335845777	0.12959079219532	1580
Q8JZS9	sp|Q8JZS9|RM48_MOUSE 39S ribosomal protein L48, mitochondrial OS=Mus musculus GN=Mrpl48 PE=1 SV=1	−1,09	0,779954260724938	0,940251150142017	23,4260786804776	23,0327998585375	23,2404230130127	22,5528552230193	23,5376585795527	23,3393648146177	12302895982,4894	11246163355,8609	0,129565043997582	0,00846816110193952	0,0225057658789027	0,914107001462701	-0.129565043997584	1,09396383399302	0.129565043997584	1581
Q3UFK8	sp|Q3UFK8|FRMD8_MOUSE FERM domain-containing protein 8 OS=Mus musculus GN=Frmd8 PE=1 SV=2	−1,09	0,935684904315447	0,98517137107117	21,5346685350988	20,8156783455322	23,709903168677	21,0792559200743	23,2979771565564	21,4141158051472	3657637740,0598	3344053822,41561	0,129314126961642	0,0684346014757137	0,0545401523983428	0,914265999005395	-0.129314126961643	1,0937735856828	0.129314126961643	1582
Q8BJI1	sp|Q8BJI1|S6A17_MOUSE Sodium-dependent neutral amino acid transporter SLC6A17 OS=Mus musculus GN=Slc6a17 PE=1 SV=1	−1,09	0,49150208060165	0,807429585179475	28,8525957718364	29,0984836692739	29,0701689048778	29,126082642873	28,8266770279669	28,7995948141363	3959277923412,94	3619842002125,75	0,129310613269043	0,00463806186468746	0,0062656484349485	0,914268225708542	-0.129310613269042	1,09377092179378	0.129310613269042	1583

P68181	sp|P68181|KAPCB_MOUSE cAMP-dependent protein kinase catalytic subunit beta OS=Mus musculus GN=Prkacb PE=1 SV=2;sp|P68181-2|KAPCB_MOUSE Isoform 2 of cAMP-dependent protein kinase catalytic subunit beta OS=Mus musculus GN=Prkacb;sp|P68181-3|KAPCB_MOUSE Is	−1,09	0,394025237347122	0,753881747531964	31,7123495077502	31,3898117118546	31,4680190706971	31,3817725927108	31,4086456757793	31,5115733756014	49027056254068	44834417792817,9	0,12897147663681	0,00533724337953684	0,0021795955878315	0,914483169466202	-0.128971476636811	1,09351383753045	0.128971476636811	1584
Q61029-4	sp|Q61029-4|LAP2B_MOUSE Isoform Gamma of Lamina-associated polypeptide 2, isoforms beta/delta/epsilon/gamma OS=Mus musculus GN=Tmpo;sp|Q61029-2|LAP2B_MOUSE Isoform Delta of Lamina-associated polypeptide 2, isoforms beta/delta/epsilon/gamma OS=Mus musculu	−1,09	0,577805845677489	0,851513225172117	27,1403798463766	26,6573625763755	26,924184307304	26,6724274176718	26,9435946391298	26,8380058322934	484948681050,664	443520249638,453	0,12883210977151	0,0089919961671835 3	0,0050967646510965	0,914571514407557	-0.128832109771509	1,09340820728249	0.128832109771509	1585
Q9Z1S5	sp|Q9Z1S5|SEPT3_MOUSE Neuronal-specific septin-3 OS=Mus musculus GN=Sept3 PE=1 SV=2	−1,09	0,53761486578873	0,831425511350952	32,6843873490794	32,2253332409013	32,3364067192113	32,262142011148	32,312057094962 1	32,4047952999906	119624139185067	109432766133213	0,128463771531982	0,0073884369423913	0,0022393501506215	0,914805045860458	-0.12846377153198	1,09312908201048	0.12846377153198	1586
Q59J78	sp|Q59J78|MIMIT_MOUSE Mimitin, mitochondrial OS=Mus musculus GN=Ndufaf2 PE=2 SV=1	−1,09	0,609676238997794	0,866608542040832	27,2837384201724	26,9215383290468	26,8812927262519	26,7143626330946	26,9915324948691	27,1138793678718	547624950025,503	501026500551,717	0,128301264684747	0,0082004784391105 8	0,0075982817228616	0,914908096368 48	-0.128301264684747	1,09300595761396	0.128301264684747	1587
Q921H8	sp|Q921H8|THIKA_MOUSE 3-ketoacyl-CoA thiolase A, peroxisomal OS=Mus musculus GN=Acaa1a PE=1 SV=1;sp|Q8VCH0|THIKB_MOUSE 3-ketoacyl-CoA thiolase B, peroxisomal OS=Mus musculus GN=Acaa1b PE=1 SV=1	−1,09	0,542625892649101	0,833495406678264	29,7066894223823	29,3143252751957	29,7040018754618	29,3952184820838	29,6117382906239	29,4521853631682	6986532786880,74	6393997093239,86	0,127858577331818	0,007633463233085 4	0,0038064095475370	0,915188876698104	-0.127858577331817	1,09267062292965	0.127858577331817	1588
Q08943	sp|Q08943|SSRP1_MOUSE FACT complex subunit SSRP1 OS=Mus musculus GN=Ssrp1 PE=1 SV=2;sp|Q08943-2|SSRP1_MOUSE Isoform 2 of FACT complex subunit SSRP1 OS=Mus musculus GN=Ssrp1	−1,09	0,880642455566815	0,973716798448825	22,5971910117934	22,1183968246739	20,9472431782709	21,1542211809417	22,2120966659598	22,031199966821 5	3203822093,52712	2932650928,17051	0,127588679795667	0,038781742041729 8	0,025956046638718	0,91536010507435	-0.127588679795668	1,09246622663195	0.127588679795668	1589
Q8VE33	sp|Q8VE33|GD1L1_MOUSE Ganglioside-induced differentiation-associated protein 1-like 1 OS=Mus musculus GN=Gdap1l1 PE=2 SV=1	−1,09	0,682002484946529	0,904517472334246	29,3868050777055	28,7546109971162	28,8639896558021	28,8811067240288	29,0865735685853	28,7725746006029	3938424594666,16	3605271867170,86	0,12751059940478 9	0,01165023954433 42	0,0055155608051752	0,915409646804844	-0.12751059940479	1,0924071026457	0.12751059940479	1590
O88874	sp|O88874|CCNK_MOUSE Cyclin-K OS=Mus musculus GN=Ccnk PE=1 SV=3	−1,09	0,603871090138464	0,86472932807488	24,1424393315207	23,8407893507363	23,6613231107722	23,9823398794781	23,7308311655272	23,6663250302842	23529417043,4102	21539738285,3169	0,127464856514108	0,010180683302948 9	0,0070177538878341	0,91543867175194	-0.127464856514112	1,09237246672814	0.127464856514112	1591
Q8BYM8	sp|Q8BYM8|SYCM_MOUSE Probable cysteine--tRNA ligase, mitochondrial OS=Mus musculus GN=Cars2 PE=2 SV=2	−1,09	0,596768077397419	0,861464850775537	25,4204801455624	25,9735589231531	25,839608189605	25,6387500127636	25,6579078890505	25,6721471841847	151605726942,513	138795624673,855	0,12736216287555	0,011207531895431 4	0,0006532065477630	0,915503836649156	-0.127362162875549	1,09229471245048	0.127362162875549	1592
P35700	sp|P35700|PRDX1_MOUSE Peroxiredoxin-1 OS=Mus musculus GN=Prdx1 PE=1 SV=1	−1,09	0,721345612932837	0,920750207284069	33,1816073307961	32,799089045904	32,4603983928778	32,4800692515926	32,9377590356243	32,7587169966684	178159130162348	163121280894012	0,127221410359499	0,010996211006671 3	0,0070478409909758	0,915593159583611	-0.127221410359497	1,09218815096301	0.127221410359497	1593
Q9D0D3	sp|Q9D0D3|PAPD1_MOUSE Poly(A) RNA polymerase, mitochondrial OS=Mus musculus GN=Mtpap PE=2 SV=1	−1,09	0,50085918261925	0,812218588749116	25,3086497347007	25,5740351178429	25,4737767577379	25,1593816694031	25,4204801455624	25,512122087088 5	113169651456,126	103619837905,801	0,127186892695152	0,0052650753739048 6	0,0072160731757794	0,915615066164383	-0.127186892695149	1,09216201977662	0.127186892695148	1594
Q9CQG2-2	sp|Q9CQG2-2|MET16_MOUSE Isoform 2 of Methyltransferase-like protein 16 OS=Mus musculus GN=Mettl16;sp|Q9CQG2|MET16_MOUSE Methyltransferase-like protein 16 OS=Mus musculus GN=Mettl16 PE=2 SV=1	−1,09	0,684347064084082	0,905612630868118	23,0456642223253	23,754344369546	23,4589281980348	23,3066932565955	23,2262234311461	23,4615901922734	14825960910,6269	13575089467,3088	0,127163906554183	0,015199607575557 3	0,0051273409884281	0,915629654572912	-0.127163906554191	1,09214461873938	0.127163906554191	1595
Q60996-3	sp|Q60996-3|2A5G_MOUSE Isoform 3 of Serine/threonine-protein phosphatase 2A 56 kDa regulatory subunit gamma isoform OS=Mus musculus GN=Ppp2r5c;sp|Q60996|2A5G_MOUSE Serine/threonine-protein phosphatase 2A 56 kDa regulatory subunit gamma isoform OS=Mus mus	−1,09	0,643863502610976	0,888837116286867	28,0390196313671	28,5307522510992	28,5750404298675	28,4562699090736	28,234699058983 5	28,1896196889447	2118234562433,08	1939651730125,08	0,127064719077675	0,010482568282158 3	0,0050445546629193	0,915692607667173	-0.1270647190776 8	1,09206953471822	0.1270647190776 8	1596
P23953	sp|P23953|EST1C_MOUSE Carboxylesterase 1C OS=Mus musculus GN=Ces1c PE=1 SV=4	−1,09	0,576857059034614	0,850667378478919	27,1320858547966	27,2176411250769	27,5000243142379	27,0441476199735	27,1273945621664	27,4140252679283	706260814156,947	646726388888,568	0,12704557389474	0,0070572161572542 1	0,0071348890495888	0,915704759382064	-0.127045573894742	1,09205504258252	0.127045573894742	1597
Q99KC8	sp|Q99KC8|VMA5A_MOUSE von Willebrand factor A domain-containing protein 5A OS=Mus musculus GN=Vwa5a PE=1 SV=2	−1,09	0,5388749832136	0,831485169227261	28,696381711632	28,4381222529815	28,2047593538113	28,3770459552226	28,3637482883521	28,3357637084218	2260079501257,57	2070585742699,04	0,126334576453658	0,0086448914757676 7	0,0007430502732218	0,916156153598539	-0.126334576453659	1,09151698220018	0.126334576453659	1598
Q7TSQ8	sp|Q7TSQ8|PDPR_MOUSE Pyruvate dehydrogenase phosphatase regulatory subunit, mitochondrial OS=Mus musculus GN=Pdpr PE=2 SV=1	−1,09	0,514730215657857	0,819610722309211	27,9328192440591	27,5780928315527	27,5118748975731	27,5713979478984	27,6464734713961	27,5424060245662	1044189731880,67	956703299016,319	0,126240398714089	0,0081791310240557 1	0,0019468537384572	0,916215961339918	-0.126240398714082	1,09144573135088	0.126240398714082	1599
Q9CQT1	sp|Q9CQT1|MTNA_MOUSE Methylthioribose-1-phosphate isomerase OS=Mus musculus GN=Mri1 PE=2 SV=1	−1,09	0,242460042062128	0,666355294143363	27,5376339052084	27,3216488345134	27,4032091695388	27,270293282656	27,4078116367401	27,322661625401	810429860985,597	742722887874,198	0,125863295128768	0,0039774674352325 2	0,0025392688550925	0,91645548076294	-0.125863295128769	1,09116047750351	0.1258632951287 7	1600
P17182	sp|P17182|ENOA_MOUSE Alpha-enolase OS=Mus musculus GN=Eno1 PE=1 SV=3	−1,09	0,213916585680608	0,645161051431415	36,5127737376142	36,314388636062	36,5127737376142	36,314388636062	36,3824391571779	36,3824391571779	673871312387988 0	617790523473027 0	0,125355368567867	0,0031426127321253 9	0,0010805624510461	0,916778192090967	-0.125355368567866	1,09077638258304	0.125355368567866	1601

Q8VD37-3	sp|Q8VD37-3|SGIP1_MOUSE Isoform 3 of SH3-containing GRB2-like protein 3-interacting protein 1 OS=Mus musculus GN=Sgip1;sp|Q8VD37-8|SGIP1_MOUSE Isoform 7 of SH3-containing GRB2-like protein 3-interacting protein 1 OS=Mus musculus GN=Sgip1;sp|Q8VD37-6|SGI	−1,09	0,563417020262159	0,845110794140252	27,8950448565735	27,9281151032895	27,5887500513035	27,9203165194517	27,6583684625279	27,572598641113	1188805341067,26	1089886350384,1	0,125334799199688	0,00672990082883141	0,0065354958242480	0,916791263240494	-0.12533479919969	1,09076083084103	0.12533479919969	1602
P35922-3	sp|P35922-3|FMR1_MOUSE Isoform ISO3 of Fragile X mental retardation protein 1 homolog OS=Mus musculus GN=Fmr1;sp|P35922-2|FMR1_MOUSE Isoform ISO2 of Fragile X mental retardation protein 1 homolog OS=Mus musculus GN=Fmr1;sp|P35922|FMR1_MOUSE Fragile X me	−1,09	0,433633513098778	0,7767413243707	27,0220262352562	26,8359983838767	26,7527581374521	26,9068501601645	26,642274809204	26,8016686908167	467311043461,703	428517702448,029	0,125028326687075	0,0051307665465397	0,0049735719751769	0,916986038407515	-0.125028326687075	1,09052914451855	0.125028326687075	1603
Q9D1T0	sp|Q9D1T0|LIGO1_MOUSE Leucine-rich repeat and immunoglobulin-like domain-containing nogo receptor-interacting protein 1 OS=Mus musculus GN=Lingo1 PE=1 SV=1	−1,09	0,528493281018188	0,829184584355878	28,6558298268788	28,4095407476388	28,2792329488929	28,2102303198389	28,4901679344148	28,3845625088349	2264106180016,18	2076393452699	0,124861774239741	0,00672281027010525	0,0049844728555104	0,917091906301038	-0.124861774239743	1,09040325525646	0.124861774239743	1604
Q8K4R4	sp|Q8K4R4|PITC1_MOUSE Cytoplasmic phosphatidylinositol transfer protein 1 OS=Mus musculus GN=Pitpnc1 PE=1 SV=1;sp|Q8K4R4-3|PITC1_MOUSE Isoform 3 of Cytoplasmic phosphatidylinositol transfer protein 1 OS=Mus musculus GN=Pitpnc1;sp|Q8K4R4-2|PITC1_MOUSE Is	−1,09	0,720092039135427	0,920444315864743	28,3794989601019	27,5571437909233	28,0023313775896	27,964154597457	27,8617602051029	27,854757020185	1417134573544,2	1300223481214,91	0,124217151909594	0,0147123372834527	0,0021954627463511	0,917501771171315	-0.124217151909597	1,08991615212183	0.124217151909597	1605
Q80ZK9	sp|Q80ZK9|WDTC1_MOUSE WD and tetratricopeptide repeats protein 1 OS=Mus musculus GN=Wdtc1 PE=1 SV=1	−1,09	0,689438898640046	0,907856236295823	24,1454988690372	23,4176157562769	23,8157619160132	23,6560354254376	23,6663250302842	23,7983315076674	21535290544,7858	19759442615,0865	0,124160536741786	0,0153186892215144	0,0033471814771525	0,917537777073118	-0.124160536741788	1,08987338176956	0.124160536741788	1606
P99024	sp|P99024|TBB5_MOUSE Tubulin beta-5 chain OS=Mus musculus GN=Tubb5 PE=1 SV=1	−1,09	0,30876512403304	0,71853230316343	34,2388631383902	34,0159445813919	34,1977817973996	34,0061355969496	34,162960021396	34,0257535658343	678471216734242	622615163062186	0,123946900086255	0,00347384151605312	0,0025082762891717	0,917673657637366	-0.123946900086257	1,08971200347473	0.123946900086257	1607
A2A6Q5	sp|A2A6Q5|CDC27_MOUSE Cell division cycle protein 27 homolog OS=Mus musculus GN=Cdc27 PE=1 SV=1;sp|A2A6Q5-2|CDC27_MOUSE Isoform 2 of Cell division cycle protein 27 homolog OS=Mus musculus GN=Cdc27;sp|A2A6Q5-3|CDC27_MOUSE Isoform 3 of Cell division cycle	−1,09	0,681421992679101	0,904517472334246	23,5506907098139	23,2937395266692	23,0135041204645	23,3825283651281	23,2937395266692	22,9239850676747	12970950524,5183	11903333451,6449	0,123918558089098	0,0115381619025865	0,010482518671095	0,917691685674435	-0.123918558089093	1,08969059610154	0.123918558089093	1608
Q3UKC1	sp|Q3UKC1|TAXB1_MOUSE Tax1-binding protein 1 homolog OS=Mus musculus GN=Tax1bp1 PE=1 SV=2	−1,09	0,605294395501328	0,865294279541889	26,3911397781081	26,1633273553353	25,8217373765899	26,0393274349537	26,062710168417	26,0168115282551	221879771732,409	203639350639,516	0,123761776058189	0,0109697650854699	0,0008813756671852	0,917791419422897	-0.123761776058186	1,08957218256496	0.123761776058186	1609
Q8C5W3	sp|Q8C5W3|TBCEL_MOUSE Tubulin-specific chaperone cofactor E-like protein OS=Mus musculus GN=Tbcel PE=1 SV=1;sp|Q8C5W3-2|TBCEL_MOUSE Isoform 2 of Tubulin-specific chaperone cofactor E-like protein OS=Mus musculus GN=Tbcel	−1,09	0,575402690145579	0,849998193752035	25,7705950123853	25,31335724764	25,769120591376	25,5098805984377	25,5637107751137	25,5223313428076	133543212355,482	122573199913,864	0,123663074863106	0,0102882710786124	0,0011037479179570	0,917854211770669	-0.123663074863105	1,08949764262765	0.123663074863105	1610
Q8C0M9	sp|Q8C0M9|ASGL1_MOUSE Isoaspartyl peptidase/L-asparaginase OS=Mus musculus GN=Asrgl1 PE=1 SV=1	−1,09	0,332529260599071	0,72953198881961	30,9746901743204	30,8893105450994	31,1656015127658	30,8665321302372	30,9837659274804	30,9222771011418	29336905441184,5	26928106803197,2	0,123603894687324	0,00456192441083986	0,0018962612212427	0,917891863447679	-0.123603894687323	1,08945295172779	0.123603894687323	1611
P62700	sp|P62700|YPEL5_MOUSE Protein yippee-like 5 OS=Mus musculus GN=Ypel5 PE=2 SV=1	−1,09	0,679779484709554	0,904048954320734	24,0288934571652	23,3535376645311	23,5068458156028	23,5928619631574	23,5605386807846	23,47931822413	18292536628,5222	16793195687,1371	0,123378351391354	0,014983565457735	0,0024846985815898	0,918035373013971	-0.123378351391359	1,08928264574047	0.123378351391359	1612
Q8CIB5	sp|Q8CIB5|FERM2_MOUSE Fermitin family homolog 2 OS=Mus musculus GN=Fermt2 PE=1 SV=1	−1,09	0,470236129653726	0,796715861328205	28,7294282134955	28,6735464306224	28,4026187469632	28,6363686210369	28,4815245373331	28,4314922090982	2640170102282,17	2424052371135,34	0,123210015034108	0,0061114692099656	0,0037456655974771	0,918142497348933	-0.123210015034104	1,08915555361768	0.123210015034104	1613
O35887	sp|O35887|CALU_MOUSE Calumenin OS=Mus musculus GN=Calu PE=1 SV=1	−1,09	0,484706249987767	0,803590359311294	28,3478835582042	28,0120626766497	28,102857773251	28,1559691605545	27,9313864300688	28,1195541428091	1687497878828,19	1549525562265,75	0,123059133687248	0,00617003821162149	0,0042942050579500	0,918238524448843	-0.123059133687245	1,08904165244018	0.123059133687245	1614
O35215	sp|O35215|DOPD_MOUSE D-dopachrome decarboxylase OS=Mus musculus GN=Ddt PE=1 SV=3	−1,09	0,668371263042376	0,898352728146089	30,5864926907295	30,4385207524286	30,2457298105003	30,0239777147737	30,3742922372541	30,6173890438023	16322752095542,1	14992227238246,1	0,12266959792588	0,00561643188186522	0,0098328895349520	0,918486487480296	-0.122669597925876	1,088747644773	0.122669597925876	1615
Q6ZWV7	sp|Q6ZWV7|RL35_MOUSE 60S ribosomal protein L35 OS=Mus musculus GN=Rpl35 PE=2 SV=1	−1,09	0,815648645556326	0,950996455735978	26,7734005079762	26,6674061630996	25,9245627621526	25,9308965147294	26,4159750442456	26,7663350011133	308541793510,98	283667567738,412	0,121264708858504	0,0174833837190531	0,0159085578102279	0,919381340564863	-0.121264708858504	1,08768794392282	0.121264708858504	1616

Q9CZ44	sp|Q9CZ44|NSF1C_MOUSE NSFL1 cofactor p47 OS=Mus musculus GN=Nsfl1c PE=1 SV=1;sp|Q9CZ44-3|NSF1C_MOUSE Isoform 3 of NSFL1 cofactor p47 OS=Mus musculus GN=Nsfl1c;sp|Q9CZ44-2|NSF1C_MOUSE Isoform 2 of NSFL1 cofactor p47 OS=Mus musculus GN=Nsfl1c	−1,09	0,513252854705502	0,819539762695211	31,5115733756014	31,0862260035438	31,209001882346	31,1939924779762	31,143072998167	31,2180758996145	38012408196060,2	34953758747619,5	0,12102282304617	0,00700105219199061	0,0012279363864493	0,919535499233176	-0.121022823046168	1,0875055947638	0.121022823046168	1617
Q61249	sp|Q61249|IGBP1_MOUSE Immunoglobulin-binding protein 1 OS=Mus musculus GN=Igbp1 PE=1 SV=1	−1,09	0,418140091996018	0,768519642229897	27,0965384478712	27,0296671524623	27,0605273992974	26,7926130600652	27,1403798463766	27,0036061767615	566217622448,682	520922107137,043	0,120288986929425	0,00123673014436682	0,0064939124777743	0,920003345858872	-0.120288986929424	1,08695256870664	0.120288986929424	1618
P11404	sp|P11404|FABPH_MOUSE Fatty acid-binding protein, heart OS=Mus musculus GN=Fabp3 PE=1 SV=5	−1,09	0,494299961920825	0,808741313308728	31,9879882223648	31,8823889345278	31,7073332672964	31,6098246071065	31,7966976397976	31,9210716119724	68594728800348,3	63107745000129,8	0,120280642806843	0,00444934262060968	0,0049303407571174	0,92000866690225	-0.120280642806842	1,08694628211176	0.120280642806842	1619
P68404-2	sp|P68404-2|KPCB_MOUSE Isoform Beta-II of Protein kinase C beta type OS=Mus musculus GN=Prkcb	−1,09	0,57650986467985	0,850431678837126	31,5739501993876	31,4444699733367	31,0862260035438	31,3069394690914	31,2250526281598	31,3230324235924	41980040375054	38628372977595,5	0,120042641459816	0,00805419071204977	0,0016794882974686	0,920160453217425	-0.120042641459813	1,08676698341404	0.120042641459813	1620
P51175	sp|P51175|PPOX_MOUSE Protoporphyrinogen oxidase OS=Mus musculus GN=Ppox PE=1 SV=1;sp|P56602|PPOX_BOVIN Protoporphyrinogen oxidase OS=Bos taurus GN=PPOX PE=1 SV=2	−1,09	0,486801940601197	0,804641488140932	23,5871603405659	23,2912926988169	23,4971371284695	23,2844462621002	23,5376585795527	23,3041913196319	15413815520,9347	14184732594,7643	0,119885075666082	0,00646524204095262	0,0060250754325412	0,920260955212479	-0.119885075666084	1,08664829724207	0.119885075666084	1621
Q9JI91	sp|Q9JI91|ACTN2_MOUSE Alpha-actinin-2 OS=Mus musculus GN=Actn2 PE=1 SV=2	−1,09	0,62667866220161	0,876307593263434	29,2338980175545	28,7501446235731	28,8875389662165	28,8699406290185	29,034879459619	28,7178629849572	3766600058034,52	3466711933969,24	0,119694893441605	0,00860875404732842	0,0054911152606988	0,920382275939918	-0.119694893441604	1,08650506006189	0.119694893441604	1622
Q99JI4	sp|Q99JI4|PSMD6_MOUSE 26S proteasome non-ATPase regulatory subunit 6 OS=Mus musculus GN=Psmd6 PE=1 SV=1	−1,09	0,682124544569316	0,904517472334246	30,6923605230542	30,2626911460235	29,9997657346318	30,2374046939875	30,2626911460235	30,2058798248574	14691239326953,1	13521812275725,7	0,119667580864059	0,0115318815302237	0,0009413720384946	0,920399700447196	-0.119667580864055	1,08648449093815	0.119667580864055	1623
Q8JZN5	sp|Q8JZN5|ACAD9_MOUSE Acyl-CoA dehydrogenase family member 9, mitochondrial OS=Mus musculus GN=Acad9 PE=1 SV=2	−1,09	0,642948713242924	0,887844298573054	29,3941367297357	29,9047847149208	29,9327417629286	29,7170699033562	29,5915968002301	29,6742040431969	8271919779915,24	7613597547582,39	0,11964388313656	0,0101942167708624	0,0021501961137782	0,920414819068809	-0.119643883136559	1,08646664447635	0.119643883136559	1624
Q62277	sp|Q62277|SYPH_MOUSE Synaptophysin OS=Mus musculus GN=Syp PE=1 SV=2	−1,09	0,261142516654457	0,682581316525843	33,4446452460359	33,2027166374192	33,3161706276563	33,268184717111	33,2547258597017	33,1921566422038	295939898404078	272417175216598	0,119486548246116	0,00363258455853314	0,0012206251994511	0,920515201517837	-0.11948654824612	1,08634816497446	0.11948654824612	1625
O35231-2	sp|O35231-2|KIFC3_MOUSE Isoform 2 of Kinesin-like protein KIFC3 OS=Mus musculus GN=Kifc3;sp|O35231|KIFC3_MOUSE Kinesin-like protein KIFC3 OS=Mus musculus GN=Kifc3 PE=1 SV=4	−1,09	0,814557165788271	0,950652201935115	21,7145801016844	21,1880773735381	20,8466617883996	21,4030055681932	20,6734853937543	21,4244040100507	1693008715,20827	1558461557,72513	0,119466831187409	0,0205760315238197	0,0201965551197382	0,92052778212735	-0.119466831187409	1,08633331814712	0.119466831187409	1626
Q5F2E7	sp|Q5F2E7|NUFP2_MOUSE Nuclear fragile X mental retardation-interacting protein 2 OS=Mus musculus GN=Nufip2 PE=1 SV=1;sp|Q5F2E7-2|NUFP2_MOUSE Isoform 2 of Nuclear fragile X mental retardation-interacting protein 2 OS=Mus musculus GN=Nufip2	−1,09	0,201377088279174	0,632123515697905	25,6146994185905	25,4944981642208	25,4761717842147	25,4312369546124	25,3711772595212	25,5351249444596	122142771502,114	112458082167,064	0,119181137562831	0,0029476357784309	0,0032596472914178	0,920710090200611	-0.119181137562834	1,08611821532456	0.119181137562834	1627
P05063	sp|P05063|ALDOC_MOUSE Fructose-bisphosphate aldolase C OS=Mus musculus GN=Aldoc PE=1 SV=4	−1,09	0,498470905658733	0,810372292544766	34,8145794845827	34,5341448386175	34,6244837935552	34,5060432263395	34,7531101143992	34,4663738267566	1126324463429870	1037069894434310	0,119109359073379	0,00413021551042416	0,0044935993951163	0,920755899482319	-0.11910935907338	1,08606417896669	0.119109359073379	1628
Q08642	sp|Q08642|PADI2_MOUSE Protein-arginine deiminase type-2 OS=Mus musculus GN=Padi2 PE=1 SV=2	−1,09	0,492603893682709	0,807777844445743	27,2136596408025	27,2058942963641	26,9293606494445	27,1620716602265	27,039238096365	26,8999307211861	597670157241,606	550309578007,379	0,119106069523579	0,00597223658900472	0,0048515921388890	0,920757998939068	-0.119106069523577	1,08606170258878	0.119106069523577	1629
Q05909	sp|Q05909|PTPRG_MOUSE Receptor-type tyrosine-protein phosphatase gamma OS=Mus musculus GN=Ptprg PE=1 SV=1	−1,09	0,764120912692889	0,933610131271809	22,4181695734126	22,5528552230193	22,484003771526	22,6191277512212	22,7260651701659	21,8627446203422	5822581299,72665	5362230412,6799	0,118825666062694	0,00299526034252583	0,0210071370840931	0,920936975655735	-0.118825666062694	1,08585063520549	0.118825666062695	1630
Q99L88	sp|Q99L88|SNTB1_MOUSE Beta-1-syntrophin OS=Mus musculus GN=Sntb1 PE=1 SV=4;sp|Q99L88-2|SNTB1_MOUSE Isoform 2 of Beta-1-syntrophin OS=Mus musculus GN=Sntb1	−1,09	0,67550328469454	0,901266728168293	25,048560475253	25,663480608275	25,6027289502231	25,3532998395674	25,3227647390092	25,3917468837871	111607788950,012	102788277783,397	0,118761968748551	0,0133205298302267	0,0013632551424442	0,920977637406963	-0.118761968748558	1,08580269420605	0.118761968748558	1631
P61329	sp|P61329|FGF12_MOUSE Fibroblast growth factor 12 OS=Mus musculus GN=Fgf12 PE=2 SV=1;sp|P61329-2|FGF12_MOUSE Isoform 2 of Fibroblast growth factor 12 OS=Mus musculus GN=Fgf12	−1,09	0,70569998861086	0,916763665799856	28,0252316570059	27,4758239742999	28,1788730675729	27,8005133029123	27,741133248696	27,8915107942788	1299901445401,91	1197246924489,5	0,118681517066708	0,013251358647764	0,0027237349364946	0,921028997024716	-0.118681517066715	1,08574214626292	0.118681517066715	1632
Q8BYL4	sp|Q8BYL4|SYYM_MOUSE Tyrosine--tRNA ligase, mitochondrial OS=Mus musculus GN=Yars2 PE=1 SV=2	−1,09	0,69876316802761	0,912827852392638	25,6128898625493	25,0286278335686	25,6064342261004	25,4651569064471	25,3625137240185	25,1746957465022	109149460159,669	100570229928,739	0,118101682748988	0,0131994018842314	0,0058141845863670	0,92139924266799	-0.118101682748998	1,08530586274894	0.118101682748998	1633
P70261	sp|P70261|PALD_MOUSE Paladin OS=Mus musculus GN=Pald1 PE=1 SV=1	−1,09	0,80589050261033	0,948143336274835	23,2404230130127	22,5224468316406	22,11588102735	22,6461403806329	22,490566066182	22,4968102137115	6705877989,98419	6179514549,74111	0,117932726918434	0,0251659909799758	0,0039066590164298	0,921507155210809	-0.117932726918434	1,0851787686567	0.117932726918434	1634

Q8BGN8-2	sp|Q8BGN8-2|SYNPR_MOUSE Isoform 2 of Synaptoporin OS=Mus musculus GN=Synpr;sp|Q8BGN8|SYNPR_MOUSE Synaptoporin OS=Mus musculus GN=Synpr PE=1 SV=1	−1,09	0,569130716162401	0,846700858951536	29,0359313240441	29,1759515219542	29,4546675630231	29,2873491997899	29,0763948194066	29,0578855856183	4909452133187,93	4524567921703,48	0,117782009879841	0,00729452806463276	0,0043744589549487	0,92160342925382	-0.117782009879841	1,08506540693935	0.117782009879841	1635
Q99K85	sp|Q99K85|SERC_MOUSE Phosphoserine aminotransferase OS=Mus musculus GN=Psat1 PE=1 SV=1	−1,09	0,480142756825462	0,801532906668177	31,5898004571495	31,1930833520968	31,3724626453784	31,2748215534313	31,3178401286448	31,3178401286448	42695535893360,1	39349351233307,4	0,117745384516043	0,00632978339623311	0,000793418803035	0,921626826082933	-0.117745384516041	1,08503786098563	0.117745384516041	1636
P19157	sp|P19157|GSTP1_MOUSE Glutathione S-transferase P 1 OS=Mus musculus GN=Gstp1 PE=1 SV=2;sp|P46425|GSTP2_MOUSE Glutathione S-transferase P 2 OS=Mus musculus GN=Gstp2 PE=2 SV=2	−1,08	0,458870961348641	0,790967157530661	32,4938038454955	32,5652501037116	32,4938038454955	32,2466587951576	32,4429895051478	32,6186624488613	132502054235776	122129562280028	0,117602269952912	0,00126852818091135	0,005737353671422	0,92171825549745	-0.117602269952911	1,08493023115865	0.117602269952911	1637
Q9DCL9	sp|Q9DCL9|PUR6_MOUSE Multifunctional protein ADE2 OS=Mus musculus GN=Paics PE=1 SV=4	−1,08	0,463337623927581	0,793595716606975	30,381645732265	30,0171565154699	30,2511880637938	30,0740303655199	30,153305359808	30,1781158654775	13271811119011,7	12232904537406,4	0,11759826656424	0,00611189146198537	0,001804067813015	0,921720813211614	-0.117598266564242	1,08492722054917	0.117598266564242	1638
Q9Z0P5	sp|Q9Z0P5|TWF2_MOUSE Twinfilin-2 OS=Mus musculus GN=Twf2 PE=1 SV=1;sp|Q9Z0P5-2|TWF2_MOUSE Isoform 2 of Twinfilin-2 OS=Mus musculus GN=Twf2	−1,08	0,776387748155582	0,938645209528714	30,0171565154699	29,4785745078136	30,1695834981336	29,6742040431969	29,5542026275958	30,192477358805	9558368541803,94	8810465068586,81	0,117546219463278	0,0121465776642297	0,0113804059589002	0,921754066089193	-0.117546219463278	1,0848880810939	0.117546219463278	1639
Q91WG4	sp|Q91WG4|ELP2_MOUSE Elongator complex protein 2 OS=Mus musculus GN=Elp2 PE=1 SV=1;sp|Q91WG4-2|ELP2_MOUSE Isoform 2 of Elongator complex protein 2 OS=Mus musculus GN=Elp2	−1,08	0,530018169194547	0,829480190327658	26,5785183382483	26,3073973295377	26,2003292010332	26,3165025351457	26,3698715068758	26,1555786458727	281129603660,248	259144302489,886	0,117479705982806	0,00739451066721693	0,004244731372122	0,92179656327858	-0.117479705982807	1,0848380649666	0.117479705982807	1640
Q91X72	sp|Q91X72|HEMO_MOUSE Hemopexin OS=Mus musculus GN=Hpx PE=1 SV=2	−1,08	0,735938359877252	0,924701393764781	27,5950102812602	28,2810559679654	28,2826760859385	28,100354168705	27,9786139581145	27,8355439715928	1524845351085,45	1405626227313,73	0,117449917132328	0,0141360421757541	0,004738686650440	0,92181559678373	-0.117449917132325	1,08481566539887	0.117449917132325	1641
Q9CR16	sp|Q9CR16|PPID_MOUSE Peptidyl-prolyl cis-trans isomerase D OS=Mus musculus GN=Ppid PE=1 SV=3	−1,08	0,484344770045746	0,803590359311294	31,6172183495446	31,2604931538048	31,2799527489482	31,3009254761233	31,3230324235924	31,2898972692622	42728535177243,3	39393359982197,5	0,11724738514302	0,00639057974314902	0,000539007859856	0,921945014468409	-0.117247385143023	1,08466338480782	0.117247385143023	1642
P35564	sp|P35564|CALX_MOUSE Calnexin OS=Mus musculus GN=Canx PE=1 SV=1;sp|P35565|CALX_RAT Calnexin OS=Rattus norvegicus GN=Canx PE=1 SV=1	−1,08	0,656844437671002	0,892226633215307	32,6339988306786	32,2512806916565	32,0369458698543	32,3120570949621	32,1359937740807	32,2317495018469	107381439238837	99045467036577,6	0,116581792005508	0,00936187558824487	0,002735148660368	0,922370455626707	-0.11658179200551	1,08416308642556	0.11658179200551	1643
Q9D0G0	sp|Q9D0G0|RT30_MOUSE 28S ribosomal protein S30, mitochondrial OS=Mus musculus GN=Mrps30 PE=2 SV=1	−1,08	0,634394649603133	0,882745957862852	25,8629458516474	25,3812269421496	25,7991721647102	25,5829712431895	25,7421229489083	25,4761717842147	142287427514,683	131256858475,48	0,116415382371889	0,0101888430204476	0,005227711404508	0,922476853845258	-0.116415382371882	1,08403803936282	0.116415382371883	1644
Q9CQ89	sp|Q9CQ89|CUTA_MOUSE Protein CutA OS=Mus musculus GN=Cuta PE=2 SV=3	−1,08	0,470225062669071	0,796715861328205	27,0435496348728	27,0832508388608	27,0327780523882	26,7761234567643	26,982191551991	27,1591850873045	561115633775,241	517616279752,646	0,116415116852203	0,000982594131794172	0,007107781057978	0,922477023621803	-0.116415116852205	1,08403783985191	0.116415116852205	1645
P31750	sp|P31750|AKT1_MOUSE RAC-alpha serine/threonine-protein kinase OS=Mus musculus GN=Akt1 PE=1 SV=2;sp|P31748|AKT_MLVAT AKT kinase-transforming protein OS=AKT8 murine leukemia virus GN=V-AKT PE=1 SV=1	−1,08	0,652163302036228	0,891901943526937	26,6173376487642	27,1975304598764	26,9791446367106	26,9656720986252	26,7753723973676	26,8109304958059	496742480953,539	458239738492,1	0,11639555558603	0,0108808534348518	0,003768218470054	0,922489531421734	-0.11639555558603	1,08402314165973	0.11639555558603	1646
Q99JB2	sp|Q99JB2|STML2_MOUSE Stomatin-like protein 2, mitochondrial OS=Mus musculus GN=Stoml2 PE=1 SV=1	−1,08	0,713491101668058	0,917934955801873	24,3866384851643	23,9621918362882	23,8343072841257	23,800646758244	24,2617206040686	23,8790570595803	28156550619,4394	25976933478,6371	0,116239470473401	0,012016545103798	0,0102875827831008	0,922589340922411	-0.1162394704734	1,08390586758806	0.1162394704734	1647
Q8BWF0	sp|Q8BWF0|SSDH_MOUSE Succinate-semialdehyde dehydrogenase, mitochondrial OS=Mus musculus GN=Aldh5a1 PE=1 SV=1	−1,08	0,266745663939949	0,685404159524725	32,2253332409013	32,2317495018469	32,2512806916565	32,20118575714	32,2512806916565	32,0149961289679	99992984534588,6	92277444804088,5	0,115848823740272	0,000419245340062284	0,003871875336810	0,922839189505028	-0.115848823740272	1,08361241197002	0.115848823740272	1648
Q06138	sp|Q06138|CAB39_MOUSE Calcium-binding protein 39 OS=Mus musculus GN=Cab39 PE=1 SV=2	−1,08	0,539116789377691	0,831485169227261	30,6358451815377	30,2268071384523	30,3135389013481	30,31031921555	30,381645732265	30,2434645802855	15816324842611,1	14596601487242,4	0,115781900289971	0,00709161292375863	0,002279726462552	0,92288199897851	-0.115781900289978	1,08356214673907	0.115781900289978	1649
Q99J08	sp|Q99J08|S14L2_MOUSE SEC14-like protein 2 OS=Mus musculus GN=Sec14l2 PE=1 SV=1	−1,08	0,587665093625586	0,857137487135776	29,2978797219439	28,9889107169626	28,8811067240288	29,0597621446406	29,0477196217778	28,8212517944698	4157627524807,5	3839044104024,61	0,115013380878161	0,0074451710650984	0,004636992299790	0,923373746473924	-0.11501338087816	1,08298509007722	0.11501338087816	1650
Q9JHQ5	sp|Q9JHQ5|LZTL1_MOUSE Leucine zipper transcription factor-like protein 1 OS=Mus musculus GN=Lztfl1 PE=1 SV=1	−1,08	0,671390482512218	0,900583196162456	28,2910319082209	27,9366295152886	27,7577253480226	27,7741719005353	27,854757020185	28,1173618978386	1439229366112,48	1328976579496,26	0,114980848550392	0,00969537593529465	0,006428691117300	0,923394568501597	-0.114980848550394	1,08296066937313	0.114980848550394	1651
O35855	sp|O35855|BCAT2_MOUSE Branched-chain-amino-acid aminotransferase, mitochondrial OS=Mus musculus GN=Bcat2 PE=2 SV=2	−1,08	0,430167028022827	0,775108167118334	27,4433741140667	27,219419790962	27,2773560240938	27,3368848768073	27,2773560240938	27,0871885833272	727866369348,911	672191984626,55	0,114800267335889	0,00425588694888159	0,004788843533618	0,923510156442366	-0.114800267335889	1,08282512436278	0.114800267335889	1652
Q9CQV4	sp|Q9CQV4|F134C_MOUSE Protein FAM134C OS=Mus musculus GN=Fam134c PE=1 SV=1	−1,08	0,458047209042478	0,790957322506963	24,1495233367792	24,4804791910647	24,4391865491359	24,3040535920527	24,2991722917027	24,2277572078905	37831062410,0123	34943362166,4588	0,114552864583753	0,00740433932259538	0,001759296011182	0,923668539565274	-0.114552864583753	1,08263945037107	0.114552864583753	1653

Q9WTP6-2	sp|Q9WTP6-2|KAD2_MOUSE Isoform 2 of Adenylate kinase 2, mitochondrial OS=Mus musculus GN=Ak2;sp|Q9WTP6|KAD2_MOUSE Adenylate kinase 2, mitochondrial OS=Mus musculus GN=Ak2 PE=1 SV=5	−1,08	0,72116313267825	0,920750207284069	27,869672887315	27,9943201847396	28,2627967575825	28,3200413611814	27,6754662525446	27,8935880061131	1508690657786,28	1393767841681,96	0,114306752574558	0,00716415878961765	0,0117244345456754	0,923826123326734	-0.114306752574554	1,08245477666183	0.114306752574554	1654
Q8BH04	sp|Q8BH04|PCKGM_MOUSE Phosphoenolpyruvate carboxykinase [GTP], mitochondrial OS=Mus musculus GN=Pck2 PE=1 SV=1	−1,08	0,678425488079869	0,903732687282123	28,5454840142889	29,1488133739494	29,0665390787366	28,8887694965435	28,8422515451511	28,7923191655361	3630090962114,14	3353794148130,01	0,114211558720879	0,0113131094152358	0,0016724471277307	0,923887082481477	-0.114211558720877	1,08238335502439	0.114211558720877	1655
Q99PG2	sp|Q99PG2|OGFR_MOUSE Opioid growth factor receptor OS=Mus musculus GN=Ogfr PE=2 SV=1;sp|Q99PG2-2|OGFR_MOUSE Isoform 2 of Opioid growth factor receptor OS=Mus musculus GN=Ogfr	−1,08	0,497740637230119	0,809992604135071	27,2799384547728	26,982191551991	26,9128048037016	27,0327780523882	26,9324340242253	26,9731145224765	563995214505,114	521222160591,89	0,113784497728257	0,00720831396532099	0,0018706981833906	0,924160608435737	-0.113784497728256	1,08206299951762	0.113784497728256	1656
Q8CE50	sp|Q8CE50|SNX30_MOUSE Sorting nexin-30 OS=Mus musculus GN=Snx30 PE=2 SV=1	−1,08	0,617943267116268	0,870166479711399	26,584740539218	26,9656720986252	27,0871885833272	26,7226120902923	26,7982568071736	26,8802456280836	471507293235,781	435766117457,376	0,113726061002796	0,00975281635965114	0,0029416772456134	0,924198042551736	-0.113726061002797	1,08201917117134	0.113726061002797	1657
Q8BG40	sp|Q8BG40|KTNB1_MOUSE Katanin p80 WD40 repeat-containing subunit B1 OS=Mus musculus GN=Katnb1 PE=1 SV=1	−1,08	0,670242748913887	0,900104854523341	28,0287075649402	27,8119809890143	27,4312593139902	27,7129019603996	27,763130170804	27,55952828088	1134616649404,44	1048645174950,9	0,113678336766318	0,0108963038651704	0,0038320721629398	0,924228615454686	-0.113678336766312	1,08198337865576	0.113678336766312	1658
Q9CQU0	sp|Q9CQU0|TXD12_MOUSE Thioredoxin domain-containing protein 12 OS=Mus musculus GN=Txndc12 PE=2 SV=1	−1,08	0,724122692704994	0,92164812633208	26,3924755423625	26,5695977401837	26,251725356772	26,0218395960174	26,2439255198213	26,7117428695641	293340400020,519	271122340016,739	0,113631784870745	0,00603238201130391	0,0133773484964307	0,924258438311853	-0.113631784870743	1,08194846652035	0.113631784870743	1659
Q91Z61	sp|Q91Z61|DIRA1_MOUSE GTP-binding protein Di-Ras1 OS=Mus musculus GN=Diras1 PE=2 SV=1	−1,08	0,571166723738328	0,84806207361736	27,9117081934441	28,0160964439985	27,9380967665236	27,6419884256892	27,8762541482515	28,1113694390224	1383033606447,52	1278281019346,78	0,113631177538353	0,00194163285327834	0,0084189306571820	0,924258827397688	-0.113631177538353	1,0819480110518	0.113631177538352	1660
Q60739-2	sp|Q60739-2|BAG1_MOUSE Isoform 2 of BAG family molecular chaperone regulator 1 OS=Mus musculus GN=Bag1;sp|Q60739|BAG1_MOUSE BAG family molecular chaperone regulator 1 OS=Mus musculus GN=Bag1 PE=1 SV=3	−1,08	0,889594303721348	0,976830327013275	23,3421578262587	24,1996408868827	24,8701728545749	24,7320198340803	23,9175054815775	23,5262499539463	30388310728,5537	28087536010,357	0,113586409320918	0,0317314070655131	0,0255684812969381	0,924287508484806	-0.113586409320913	1,08191443768326	0.113586409320913	1661
Q99J36	sp|Q99J36|THUM1_MOUSE THUMP domain-containing protein 1 OS=Mus musculus GN=Thumpd1 PE=1 SV=1	−1,08	0,306332574510124	0,71837258919956	28,759343091525	28,5750404298675	28,7807657090108	28,5471743747981	28,6768520225877	28,6558298268788	2927148167990,94	2706341236598,15	0,113152017704028	0,00394006380317198	0,0024312731221781	0,924565850882656	-0.113152017704027	1,08158872517877	0.113152017704027	1662
P70265	sp|P70265|F262_MOUSE 6-phosphofructo-2-kinase/fructose-2,6-bisphosphatase 2 OS=Mus musculus GN=Pfkfb2 PE=1 SV=2	−1,08	0,85132209748941	0,962641439679318	25,0120600285972	26,2409001964166	26,1684627487745	25,6555739842145	25,5740351178429	25,9585083386998	161398288923,827	149322238213,018	0,112196245172014	0,0267178847238684	0,0078735800711065	0,925178570409081	-0.112196245172011	1,08087241964309	0.112196245172011	1663
Q9CR50	sp|Q9CR50|ZN363_MOUSE RING finger and CHY zinc finger domain-containing protein 1 OS=Mus musculus GN=Rchy1 PE=1 SV=1	−1,08	0,87117110930304	0,971009960968743	20,7897257103873	21,4835907437214	21,6228591796111	20,4890801292557	21,2911396120514	21,882883155524	1777947082,53228	1645046188,59644	0,112084293891906	0,0209530224208422	0,0329645160026567	0,925250365862097	-0.112084293891908	1,08078854858734	0.112084293891909	1664
P47809	sp|P47809|MP2K4_MOUSE Dual specificity mitogen-activated protein kinase kinase 4 OS=Mus musculus GN=Map2k4 PE=1 SV=2	−1,08	0,3022937465656	0,715641870477102	30,038498203068	29,8451084763157	29,9281463515691	29,9112106332108	29,7592429065261	29,9085130255149	10036514517609	9287174903979,73	0,11194662655095	0,00324063861521181	0,0029126369311903	0,925338660915146	-0.111946626550947	1,08068542063401	0.111946626550947	1665
Q9D8U8	sp|Q9D8U8|SNX5_MOUSE Sorting nexin-5 OS=Mus musculus GN=Snx5 PE=1 SV=1	−1,08	0,591547068007092	0,85975361851476	29,4054016596301	29,0120311248533	29,0435033047516	28,9889107169626	29,0238572486725	29,2155065783681	4584239601569,64	4242150774374,7	0,111886552503794	0,00749801552949622	0,0041957088847813	0,925377192963952	-0.111886552503793	1,08064042166096	0.111886552503793	1666
Q9D868-2	sp|Q9D868-2|PPIH_MOUSE Isoform 2 of Peptidyl-prolyl cis-trans isomerase H OS=Mus musculus GN=Ppih;sp|Q9D868|PPIH_MOUSE Peptidyl-prolyl cis-trans isomerase H OS=Mus musculus GN=Ppih PE=2 SV=1	−1,08	0,743962331617341	0,92694071457895	26,4873971485829	26,126952961226	26,0255942843937	25,769120591376	26,2266208709123	26,4120453633107	242269068332,775	224227935778,275	0,111644190976428	0,00925912166630588	0,012661427512727	0,925532662181541	-0.111644190976428	1,08045889773672	0.111644190976428	1667
Q922V4	sp|Q922V4|PLRG1_MOUSE Pleiotropic regulator 1 OS=Mus musculus GN=Plrg1 PE=2 SV=1	−1,08	0,652423545037603	0,891901943526937	25,9245627621526	26,3722038170175	26,1876191399344	26,0481993746539	25,9104073439626	26,2939048107703	230026856482,527	212917480009,752	0,111507914538653	0,00859905885940798	0,0074475343864148	0,925620091782306	-0.111507914538653	1,08035684281061	0.111507914538653	1668
Q7TMM9	sp|Q7TMM9|TBB2A_MOUSE Tubulin beta-2A chain OS=Mus musculus GN=Tubb2a PE=1 SV=1	−1,08	0,562778382337247	0,845110794140252	36,314388636062	36,0203651980554	36,314388636062	36,0203651980554	36,314388636062	36,084202935126	5352717781601970	4957372495195630	0,110695923074659	0,004687230121335	0,0042793936478061	0,926141204797833	-0.110695923074659	1,07974895709158	0.110695923074659	1669
	sp|Q9D0L4|ADCK1_MOUSE Uncharacterized aarF domain-containing protein kinase 1 OS=Mus musculus GN=Adck1 PE=2 SV=1;sp|Q9D0L4-2|																				

Q9D0L4	[illegible] ADCK1_MOUSE Isoform 2 of Uncharacterized aarF domain-containing protein kinase 1 OS=Mus musculus GN=Adck1	−1,08	0,934559186803381	0,98467134375593	21,7051251708901	24,0381319453118	24,2627398725708	24,1418352095624	22,6646252533469	22,9695985789723	13627182247,038	12621737303,3883	0,110576778563969	0,0606916777008471	0,0335307895617418	0,926217693032744	-0.110576778563965	1,07965979004965	0.110576778563965	1670
Q505D7	sp|Q505D7|OPA3_MOUSE Optic atrophy 3 protein homolog OS=Mus musculus GN=Opa3 PE=1 SV=1	−1,08	0,877721093823071	0,973716798448825	26,6649401997547	27,4925739899774	26,5652571906599	27,6317151295644	26,4967677565846	26,3643567050085	485085237628,958	449295451922,343	0,110573817357128	0,0189186933931221	0,0259640123411913	0,926219594144832	-0.110573817357125	1,07965757399387	0.110573817357126	1671
Q8BHJ5	sp|Q8BHJ5|TBL1R_MOUSE F-box-like/WD repeat-containing protein TBL1XR1 OS=Mus musculus GN=Tbl1xr1 PE=1 SV=1	−1,08	0,735513813138771	0,924701393764781	25,3133572476	25,7564189171455	25,6757304025179	25,810667197616	25,5153070978226	25,1917952891573	128839788834,641	119421273836,056	0,109518338526361	0,00922464128194578	0,0121361085019607	0,926897466351228	-0.109518338526357	1,07886798303219	0.109518338526358	1672
Q9QYG0	sp|Q9QYG0|NDRG2_MOUSE Protein NDRG2 OS=Mus musculus GN=Ndrg2 PE=1 SV=1	−1,08	0,701320797962553	0,913830966176594	32,6477383197789	32,0567659011701	32,2317495018469	32,2785243609912	32,0651775689469	32,3661802272999	107884742516078	100043617892771	0,108861711676271	0,00939481713680945	0,0048024047070156	0,927319429602027	-0.108861711676282	1,07837705981116	0.108861711676282	1673
Q9M2J6	sp|Q9M2J6|MCC15_ARATH MATH domain and coiled-coil domain-containing protein At3g58200 OS=Arabidopsis thaliana GN=At3g58200 PE=2 SV=1	−1,08	0,664733326247884	0,896678990733797	26,7506588434913	26,7900481326491	26,3327598269784	26,4229344891842	26,7334809843027	26,4907634434962	365484217406,275	338930069706,034	0,108821470380395	0,00951797771575477	0,0061499227155651	0,927345295814174	-0.108821470380394	1,07834698090751	0.108821470380394	1674
Q78JW9	sp|Q78JW9|UBFD1_MOUSE Ubiquitin domain-containing protein UBFD1 OS=Mus musculus GN=Ubfd1 PE=1 SV=2	−1,08	0,406427751941499	0,761046025603326	27,4902079656322	27,1854010346971	27,3454914633062	27,3089720088055	27,2432346578062	27,2429325656057	747774142811,066	693520343453,627	0,108664382666658	0,00557666516538612	0,0013952290087067	0,927446275216891	-0.108664382666657	1,07822957159016	0.108664382666657	1675
P60840-2	sp|P60840-2|ENSA_MOUSE Isoform 2 of Alpha-endosulfine OS=Mus musculus GN=Ensa;sp|P60840|ENSA_MOUSE Alpha-endosulfine OS=Mus musculus GN=Ensa PE=1 SV=1;sp|P60840-3|ENSA_MOUSE Isoform 3 of Alpha-endosulfine OS=Mus musculus GN=Ensa	−1,08	0,808163142774747	0,94879038498679	26,417345126627	25,8471838387831	25,7564189171455	25,4993244819944	26,0103345112904	26,2857612704694	197101099114,163	182827103684,554	0,108455859076099	0,0137759269771069	0,0153886679499904	0,927580335707101	-0.108455859076103	1,0780737382037	0.108455859076103	1676
Q9QXT0	sp|Q9QXT0|CNPY2_MOUSE Protein canopy homolog 2 OS=Mus musculus GN=Cnpy2 PE=1 SV=1	−1,08	0,644893507052438	0,8894474714406	27,9035914445976	27,6642808277829	27,4591263032037	27,4203446407034	27,6254440730023	27,7577253480226	1045656665323,2	970591342278,781	0,10747333328513	0,00803779017181131	0,0061589787068737	0,928212265522912	-0.107473333285135	1,0773397822282	0.107473333285136	1677
Q9CQN7	sp|Q9CQN7|RM41_MOUSE 39S ribosomal protein L41, mitochondrial OS=Mus musculus GN=Mrpl41 PE=2 SV=1	−1,08	0,673355945530195	0,900894908348996	25,4069322364685	25,4127860971458	25,0796635391775	24,9575896134171	25,3047067573104	25,4142056413629	97176423032,6263	90218529585,6842	0,107182556582696	0,00753606936524904	0,0094503542175385	0,928399366535584	-0.107182556582695	1,07712266514313	0.107182556582695	1678
P14733	sp|P14733|LMNB1_MOUSE Lamin-B1 OS=Mus musculus GN=Lmnb1 PE=1 SV=3	−1,08	0,756316461535346	0,931775125431143	24,3979637610652	25,1478806807997	24,6333618185744	24,5615634697321	24,8340128117331	24,5607808475858	54769652270,2665	50848631318,2619	0,107167778906757	0,0155104736169589	0,0063899217043243	0,928408876275936	-0.107167778906757	1,0771163211971	0.107167778906757	1679
Q9D4H8	sp|Q9D4H8|CUL2_MOUSE Cullin-2 OS=Mus musculus GN=Cul2 PE=1 SV=2;sp|Q9D4H8-2|CUL2_MOUSE Isoform 2 of Cullin-2 OS=Mus musculus GN=Cul2	−1,08	0,650780271143671	0,891068371258258	29,9739816452622	29,7856451223423	29,4242425054228	29,6878677659393	29,5939809288885	29,6792282244892	8141181430898,87	7558488153601,25	0,107140474615249	0,00939765216920313	0,0017499231979613	0,928426447408962	-0.107140474615249	1,07709124701346	0.107140474615249	1680
Q8K2F8	sp|Q8K2F8|LS14A_MOUSE Protein LSM14 homolog A OS=Mus musculus GN=Lsm14a PE=1 SV=1	−1,08	0,80125763440085	0,945787729921946	26,014799565769	26,7585971508416	26,8071250785447	26,7270880304719	26,2649632160102	26,3678330475435	331482414992,368	307978177357,743	0,106104209571318	0,0167416800401334	0,0091718817880939	0,929093561010872	-0.106104209571317	1,07631786718227	0.106104209571317	1681
Q9LR02	sp|Q9LR02|H3L3_ARATH Histone H3-like 3 OS=Arabidopsis thaliana GN=At1g75600 PE=2 SV=3;sp|Q71DI3|H32_HUMAN Histone H3.2 OS=Homo sapiens GN=HIST2H3A PE=1 SV=3;sp|Q6LBF0|H31_MUSPA Histone H3.1 OS=Mus pahari GN=H3110 PE=2 SV=3;sp|Q6LBE8|H32_MUSPA Histone H	−1,08	0,818554463396146	0,952427251821356	24,8446467185302	23,871854383327	24,4665538370577	24,024166766318	24,4288410238104	24,5094875445525	39294549094,0508	36509260519,8189	0,106066749083	0,0201029699604691	0,0106930773271909	0,929117685825447	-0.106066749083	1,0762899202716	0.106066749083	1682
Q9R0E1	sp|Q9R0E1|PLOD3_MOUSE Procollagen-lysine,2-oxoglutarate 5-dioxygenase 3 OS=Mus musculus GN=Plod3 PE=1 SV=1	−1,08	0,892994990563938	0,977069018898103	23,0200748010908	21,504041661994	22,5116389434821	21,9979846941957	21,9143063520067	22,9029360555361	5063135549,74047	4704297865,31525	0,106051697250522	0,034530421956072	0,0246154347273308	0,92912737948649	-0.10605169725052	1,07627869125187	0.10605169725052	1683
Q8CCS6-2	sp|Q8CCS6-2|PABP2_MOUSE Isoform 2 of Polyadenylate-binding protein 2 OS=Mus musculus GN=Pabpn1;sp|Q8CCS6|PABP2_MOUSE Polyadenylate-binding protein 2 OS=Mus musculus GN=Pabpn1 PE=2 SV=3	−1,08	0,601691386003391	0,862899589595385	27,7681093764479	27,4591263032037	27,2900373208184	27,436658796006	27,410110676239	27,4505481052565	882264440187,619	819892599576,808	0,105776199310767	0,00881354963191503	0,0007489780288231	0,92930482316895	-0.105776199310768	1,07607318402801	0.105776199310768	1684
	sp|Q80Y81-2|RNZ2_MOUSE Isoform 2 of Zinc phosphodiesterase ELAC protein 2 OS=Mus musculus GN=Elac2;sp|Q80Y81|RNZ2_MOUSE Zinc																			

Q80Y81-2	[illegible] phosphodiesterase ELAC protein 2 OS=Mus musculus GN=Elac2 PE=1 SV=1	−1,08	0,756900033855174	0,931806144200878	26,3652422189801	25,6547165375052	25,7918978973668	26,0066860711259	25,6958788171413	25,8903660723575	183831525214,949	170894171237,073	0,105281003980811	0,0145317800472643	0,0060714288992496	0,929623855523427	-0.105281003980811	1,07570389255657	0.105281003980811	1685
Q8BP48	sp|Q8BP48|MAP11_MOUSE Methionine aminopeptidase 1 OS=Mus musculus GN=Metap1 PE=2 SV=1	−1,08	0,533461260541832	0,8300431463043	26,4356893840667	26,072361667895	26,2537200098112	26,2029568269526	26,2580903346101	26,0820246055589	252309838545,615	234570946403,414	0,1051721292798 1	0,00691949690584635	0,0034398474302813	0,929694013343067	-0.105172129279807	1,07562271634311	0.105172129279807	1686
Q60596	sp|Q60596|XRCC1_MOUSE DNA repair protein XRCC1 OS=Mus musculus GN=Xrcc1 PE=1 SV=2	−1,08	0,427622472459653	0,773941056137542	22,8419647179408	22,6823866658236	22,5286124376778	22,5905470800734	22,6368476224383	22,6079015202486	7106823912,20523	6609444233,25999	0,104675988393545	0,0069071979739734	0,0010344552212703	0,930013788847217	-0.104675988393546	1,07525287473375	0.104675988393546	1687
Q8K273	sp|Q8K273|MMGT1_MOUSE Membrane magnesium transporter 1 OS=Mus musculus GN=Mmgt1 PE=2 SV=1	−1,07	0,54931173770705	0,838092765530185	25,3372129119629	25,3979425844979	25,1642937576338	25,0651396225333	25,2133112519035	25,4043835260839	97178605681,7975	90409163634,0293	0,104169725011297	0,0047917513274 3982	0,0067415597758009	0,930340201937717	-0.104169725011297	1,07487561852879	0.104169725011297	1688
Q8BXV2	sp|Q8BXV2|BRI3B_MOUSE BRI3-binding protein OS=Mus musculus GN=Bri3bp PE=2 SV=1	−1,07	0,752683511159215	0,929556973032509	28,2272204810265	27,9056879546371	27,7361956183784	27,5359993615751	27,9960381244174	28,1207709623458	1384510852385,64	1288203184074	0,104016199238185	0,0089212125904 8461	0,0110453415185883	0,930439210248375	-0.1040161992381 8	1,07476124069734	0.1040161992381 8	1689
Q62148	sp|Q62148|AL1A2_MOUSE Retinal dehydrogenase 2 OS=Mus musculus GN=Aldh1a2 PE=2 SV=2	−1,07	0,772089735217505	0,936785701190125	25,4944981642208	25,7926120679867	25,6522026352665	26,0401650290287	25,3572525299008	25,3260413000039	137437845962,222	127896385696,822	0,103803835892456	0,00581525374990456	0,015781018614438	0,930576180100911	-0.103803835892464	1,07460304850223	0.103803835892464	1690
Q6PAK3	sp|Q6PAK3|ANM8_MOUSE Protein arginine N-methyltransferase 8 OS=Mus musculus GN=Prmt8 PE=2 SV=2	−1,07	0,766862108483158	0,935443996346427	27,7823931945621	27,0435496348728	27,6095679757196	27,5950102812602	27,322661625401	27,3023677084148	858543857106,978	799042419220,441	0,103619739126926	0,014064297555 8352	0,0059625702181438	0,930694934925004	-0.103619739126926	1,07446593128884	0.103619739126926	1691
P62334	sp|P62334|PRS10_MOUSE 26S protease regulatory subunit 10B OS=Mus musculus GN=Psmc6 PE=1 SV=1	−1,07	0,696741831394733	0,911421490959632	30,5694328473646	29,9903371484726	30,148766825788	30,1559838428577	30,279068162713	30,0580803905391	13533361031677,7	12595710877286,3	0,103587632158847	0,00989808145950476	0,0036709769607860	0,930715647635748	-0.103587632158851	1,07444201947206	0.103587632158851	1692
Q9D4V0	sp|Q9D4V0|EKI1_MOUSE Ethanolamine kinase 1 OS=Mus musculus GN=Etnk1 PE=2 SV=2	−1,07	0,782547262567943	0,940251150142017	26,5398052352229	26,2212886157278	25,7400196200249	25,9776537154619	25,9834974475046	26,325250003904	231313049730,023	215336348788,257	0,1032547921168 1	0,015387423391 6333	0,0076265858634312	0,930930395148855	-0.103254792116806	1,07419416662199	0.103254792116806	1693
Q62376	sp|Q62376|RU17_MOUSE U1 small nuclear ribonucleoprotein 70 kDa OS=Mus musculus GN=Snrnp70 PE=1 SV=2;sp|Q62376-2|RU17_MOUSE Isoform 2 of U1 small nuclear ribonucleoprotein 70 kDa OS=Mus musculus GN=Snrnp70	−1,07	0,783853349226127	0,940251150142017	27,1164940541076	27,8869880507204	27,541410950126	27,6721550228499	27,4505481052565	27,2082466545883	890424677549,648	829135913655,777	0,102884965973377	0,0140260845633914	0,0084548193246256	0,931169064111598	-0.102884965973377	1,07391883873856	0.102884965973377	1694
Q9WVE8	sp|Q9WVE8|PACN2_MOUSE Protein kinase C and casein kinase substrate in neurons protein 2 OS=Mus musculus GN=Pacsin2 PE=1 SV=1	−1,07	0,7521156491242	0,929192414636566	29,1565083052916	29,7341360948367	29,8120687934487	29,6647193245074	29,4637941408889	29,3608168934757	6934784289003,19	6458663042620,58	0,102615452479879	0,0121118183053244	0,0052399385755503	0,931343034398688	-0.102615452479878	1,07371823599415	0.102615452479878	1695
P51855	sp|P51855|GSHB_MOUSE Glutathione synthetase OS=Mus musculus GN=Gss PE=2 SV=1	−1,07	0,741376803413629	0,925497610201997	27,4646552402382	27,1149582581361	27,7421650657753	27,1601478692373	27,55952828088	27,3890281684233	826605055246,892	769932053875,313	0,10246705249373 5	0,0114536517614813	0,0073220043543791	0,931438840094376	-0.102467052493736	1,0736077957611	0.102467052493736	1696
Q9D6T0-2	sp|Q9D6T0-2|NOSIP_MOUSE Isoform 2 of Nitric oxide synthase-interacting protein OS=Mus musculus GN=Nosip;sp|Q9D6T0|NOSIP_MOUSE Nitric oxide synthase-interacting protein OS=Mus musculus GN=Nosip PE=2 SV=1	−1,07	0,800778619965305	0,945787729921946	23,316746816814	23,5406755707304	23,8733808899669	23,9424348342287	23,168322265652	23,4079437247829	17351320081,5546	16166929571,1289	0,101999718961296	0,0118795277669027	0,0168596020558362	0,931740610808928	-0.1019997189613	1,0732600773211	0.101999718961301	1697
P26516	sp|P26516|PSMD7_MOUSE 26S proteasome non-ATPase regulatory subunit 7 OS=Mus musculus GN=Psmd7 PE=1 SV=2	−1,07	0,613321584370994	0,868059454773913	30,0218464917338	29,57918938533	29,8078729257526	29,7910678192371	29,6207138314607	29,7856451223423	8775366062536,86	8178056045190,35	0,101701358531774	0,00742775931777313	0,0032565903186980	0,931933321859187	-0.101701358531774	1,07303814183296	0.101701358531774	1698
P11798	sp|P11798|KCC2A_MOUSE Calcium/calmodulin-dependent protein kinase type II subunit alpha OS=Mus musculus GN=Camk2a PE=1 SV=2	−1,07	0,359085955857818	0,733314212552756	34,9023917375126	34,7531101143992	34,698424438377	34,741959064496	34,7771307544105	34,6244837935552	1278727831230440	1192137772089870	0,101158255046292	0,0030351424275682	0,0023026671999617	0,932284214806485	-0.101158255046294	1,07263427195061	0.101158255046294	1699
Q8BVQ5	sp|Q8BVQ5|PPME1_MOUSE Protein phosphatase methylesterase 1 OS=Mus musculus GN=Ppme1 PE=1 SV=5	−1,07	0,694693809806053	0,910462689446474	31,4372046772841	31,090420390194	31,1939924779762	31,2024247921078	31,4047907964564	30,9049840027489	36948241905230	34456996944480,5	0,100708747970871	0,00569824484396187	0,0080654502433528	0,932574736109518	-0.100708747970873	1,07230011845674	0.100708747970873	1700
Q3B7Z2	sp|Q3B7Z2|OSBP1_MOUSE Oxysterol-binding protein 1 OS=Mus musculus GN=Osbp PE=1 SV=3;sp|P22059|OSBP1_HUMAN Oxysterol-binding protein 1 OS=Homo sapiens GN=OSBP PE=1 SV=1;sp|Q3B7Z2-2|OSBP1_MOUSE Isoform 2 of Oxysterol-binding protein 1 OS=Mus musculus GN=O	−1,07	0,594487440573796	0,860080030359509	29,4999946244056	29,2505827592898	29,0533026951177	29,1702498422972	29,2389697441818	29,1855954237581	5139413204197,35	4793450726023,98	0,100539045885938	0,00764837726247191	0,0012353835260199	0,932684439949132	-0.100539045885938	1,07217399279711	0.100539045885938	1701
Q9ER35	sp|Q9ER35|FN3K_MOUSE Fructosamine-3-kinase OS=Mus musculus GN=Fn3k PE=2 SV=1	−1,07	0,806887983348177	0,948497813008292	27,1289472953639	26,6924412252218	26,4091064430994	26,8740206459646	26,2872690468727	26,8602290837401	411674292404,524	383973582729,542	0,100496302934691	0,0135594190267712	0,0125535329626159	0,932712073146014	-0.100496302934691	1,07214222780137	0.100496302934691	1702

Q8R001-2	sp|Q8R001-2|MARE2_MOUSE Isoform 2 of Microtubule-associated protein RP/EB family member 2 OS=Mus musculus GN=Mapre2;sp|Q8R001|MARE2_MOUSE Microtubule-associated protein RP/EB family member 2 OS=Mus musculus GN=Mapre2 PE=1 SV=1	−1,07	0,208289776010463	0,638802225210469	30,9004869372115	30,8796473041202	30,9222771011418	30,9084445981046	30,727262227369	30,8577552537318	26305621586948,1	24535791044003,8	0,10048335530469	0,000689841752364198	0,0030318196409183	0,932720443913691	-0.100483355304685	1,07213260578272	0.100483355304685	1703
Q99LH9	sp|Q99LH9|3BP5L_MOUSE SH3 domain-binding protein 5-like OS=Mus musculus GN=Sh3bp5l PE=2 SV=1	−1,07	0,477318131632237	0,799905345588749	22,7662261675712	23,0567184762801	22,8107698771548	22,7593573011881	22,8828786350751	22,7826561503887	8624776101,97326	8044845093,62122	0,100422363489689	0,00683849965302319	0,0028775240322644	0,932759876720816	-0.10042236348969	1,07208728093619	0.10042236348969	1704
Q9DBB8	sp|Q9DBB8|DHDH_MOUSE Trans-1,2-dihydrobenzene-1,2-diol dehydrogenase OS=Mus musculus GN=Dhdh PE=2 SV=1	−1,07	0,64428707686366	0,889151426538204	26,7912227971815	26,4958547362392	26,7516578076357	26,7839028148677	26,6630776319574	26,3833279274518	386183486471,162	360263949005,805	0,100232183786704	0,00600963354156201	0,0077216762206414	0,932882843587636	-0.100232183786706	1,0719459649984	0.100232183786706	1705
Q9EQC5	sp|Q9EQC5|NTKL_MOUSE N-terminal kinase-like protein OS=Mus musculus GN=Scyl1 PE=1 SV=1	−1,07	0,751120537169235	0,928854093158237	26,8090056281754	26,1892833237637	26,1234980759642	26,3276898796021	26,2819309123858	26,3040142907628	284480062593,895	265410899867,167	0,100099926340979	0,0143406923725092	0,0008699675263044	0,932968368493542	-0.100099926340977	1,07184770006157	0.100099926340977	1706
Q9WVA4	sp|Q9WVA4|TAGL2_MOUSE Transgelin-2 OS=Mus musculus GN=Tagln2 PE=1 SV=4	−1,07	0,710775182690889	0,916805961322165	28,4835717147051	28,0940222760243	28,0364216970831	27,8915107942788	28,2910319082209	28,2234557658422	1774734403692,59	1655845421087,2	0,100035136983271	0,00862429197372265	0,0076004886644696	0,933010267700888	-0.100035136983272	1,07179956600498	0.100035136983272	1707
Q9ERD7	sp|Q9ERD7|TBB3_MOUSE Tubulin beta-3 chain OS=Mus musculus GN=Tubb3 PE=1 SV=1	−1,07	0,604990648201667	0,865294279541889	34,9429327686499	34,7771307544105	34,8580740958575	34,8145794845827	34,9927921828581	34,5639686668958	1377958162906830	1286172023502970	0,0994484723116257	0,00237837602193274	0,0061921575883680	0,933389748778558	-0.0994484723116298	1,07136381271447	0.0994484723116299	1708
Q3UA37	sp|Q3UA37|QRIC1_MOUSE Glutamine-rich protein 1 OS=Mus musculus GN=Qrich1 PE=2 SV=1	−1,07	0,612796752131919	0,868059454773913	25,1800505226162	24,875004184539	24,8260555298346	24,7970047172291	25,0467789403677	24,8315460515407	69207154226,1124	64619145236,1857	0,0989595156813512	0,00768484679201485	0,0054372352065126	0,933706145827976	-0.0989595156813529	1,07100076878388	0.098959515681353	1709
O88428	sp|O88428|PAPS2_MOUSE Bifunctional 3-phosphoadenosine 5-phosphosulfate synthase 2 OS=Mus musculus GN=Papss2 PE=1 SV=2;sp|O95340|PAPS2_HUMAN Bifunctional 3-phosphoadenosine 5-phosphosulfate synthase 2 OS=Homo sapiens GN=PAPSS2 PE=1 SV=2;sp|O95340-2|P	−1,07	0,902607531702063	0,977644369603516	22,4525488290568	23,405784578485	23,5680656793277	23,43383578992	22,1997126389534	23,5871603405659	11233127128,5192	10488755230,369	0,0989161336384754	0,0260427362156529	0,0329665230503805	0,93373422925302	-0.0989161336384716	1,07096856412427	0.0989161336384715	1710
Q9CZC8	sp|Q9CZC8|SCRN1_MOUSE Secernin-1 OS=Mus musculus GN=Scrn1 PE=1 SV=1	−1,07	0,489193936372585	0,806156092714947	32,20118575714	31,8850101738384	32,0900263529684	32,0501774277855	31,9922601162753	31,9282291432254	83740241573171,6	78194640794785,2	0,0988513466431135	0,00500309018351385	0,0019068225898266	0,933776154997825	-0.0988513466431121	1,0709204713011	0.0988513466431121	1711
Q9QXG4	sp|Q9QXG4|ACSA_MOUSE Acetyl-coenzyme A synthetase, cytoplasmic OS=Mus musculus GN=Acss2 PE=1 SV=2	−1,07	0,729589775434226	0,924255649091809	27,8915107942788	27,7120449619398	27,2661868502051	27,683736916332	27,4627343947558	27,5185848493879	992256555683,428	926813986123,604	0,098433373502103	0,0116561311113076	0,0041704849787306	0,934046724927154	-0.0984333735021018	1,07061025247745	0.0984333735021018	1712
Q99K95	sp|Q99K95|RTF2_MOUSE Protein RTF2 homolog OS=Mus musculus GN=Rtfdc1 PE=2 SV=1	−1,07	0,915519354083108	0,980665300581806	23,3727250633184	21,5496008959617	22,6264019676859	22,1585650899638	23,1722651329508	22,0134108257316	6007308464,4118	5611480074,31802	0,0983374017596486	0,0407057541537399	0,0281248047110139	0,934108862157033	-0.0983374017596475	1,07053903513003	0.0983374017596474	1713
Q9CYZ2	sp|Q9CYZ2|TPD54_MOUSE Tumor protein D54 OS=Mus musculus GN=Tpd52l2 PE=1 SV=1	−1,07	0,738432645266067	0,925385360812446	28,9967840041252	29,441616430095	29,5601460600447	29,4440052105859	29,0627516643017	29,2873491997899	5483961358737,08	5122696235633,38	0,0983150598320084	0,0101256260513081	0,0065482669300156	0,934123328106948	-0.0983150598320082	1,07052245662953	0.0983150598320081	1714
Q9QZD9	sp|Q9QZD9|EIF3I_MOUSE Eukaryotic translation initiation factor 3 subunit I OS=Mus musculus GN=Eif3i PE=1 SV=1	−1,07	0,481810669899533	0,802303567138125	29,4348108568803	29,2069428457707	29,1249791637666	29,1365569873341	29,1918467273542	29,2338980175545	5076167289232,44	4741780958686,97	0,0983105944924288	0,00548748293832738	0,0016726486221965	0,934126219351602	-0.0983105944924282	1,07051914322041	0.0983105944924282	1715
Q7TT45	sp|Q7TT45|RRAGD_MOUSE Ras-related GTP-binding protein D OS=Mus musculus GN=Rragd PE=2 SV=2	−1,07	0,688029730555527	0,907856236295823	24,939766410663	24,4971412211746	24,7017312592705	24,8100392117994	24,4331596823421	24,6912518557112	54034017040,3068	50478680542,6438	0,0981937395991928	0,00896387199361547	0,00781839421112064	0,934201884434931	-0.0981937395991939	1,07043243720801	0.098193739599194	1716
Q8CGV2	sp|Q8CGV2|TPH2_MOUSE Tryptophan 5-hydroxylase 2 OS=Mus musculus GN=Tph2 PE=2 SV=2	−1,07	0,746013077358034	0,927700293161169	25,4043835260839	26,0168115282551	25,9073997517755	25,7725191212397	25,5256101773492	25,8263359153978	156480661203,246	146187381593,74	0,0981655834205369	0,0126711875669587	0,0062377956076257	0,934220116847944	-0.0981655834205368	1,0704115464501	0.0981655834205366	1717
Q6PGF2	sp|Q6PGF2|MORN4_MOUSE MORN repeat-containing protein 4 OS=Mus musculus GN=Morn4 PE=2 SV=1	−1,07	0,770225172725557	0,936218563920244	25,5256101773492	24,9925963663285	24,9428329797975	24,8115612153573	25,1876434359737	25,2582028351988	83966172064,2833	78455898044,4147	0,0979263099557937	0,0128434775238666	0,0095713880229077	0,934375071717572	-0.0979263099557921	1,070234031567	0.0979263099557919	1718
Q2TA57	sp|Q2TA57|ASPH1_MOUSE Aspartate beta-hydroxylase domain-containing protein 1 OS=Mus musculus GN=Asphd1 PE=2 SV=2	−1,07	0,889860837639099	0,976830327013275	23,5485803366946	24,0670079530992	24,8358716084403	23,709903168677	23,8261006818307	24,7119475537275	30790949389,2436	28771480357,2518	0,0978668983569519	0,0268190454996509	0,0227580989935487	0,934413550993096	-0.0978668983569541	1,07018995918584	0.0978668983569543	1719
P05202	sp|P05202|AATM_MOUSE Aspartate aminotransferase, mitochondrial OS=Mus musculus GN=Got2 PE=1 SV=1	−1,07	0,652078151513759	0,891901943526937	35,8547745671773	35,3451936065751	35,5541042014945	35,4772270626791	35,5682395086685	35,505272310377	2845999707347620	2659495825822030	0,0977827409171524	0,007198705363302	0,0013123182622429	0,934468060188451	-0.0977827409171528	1,07012753308907	0.0977827409171527	1720
Q9CWR2	sp|Q9CWR2|SMYD3_MOUSE Histone-lysine N-methyltransferase SMYD3 OS=Mus musculus GN=Smyd3 PE=2 SV=1	−1,07	0,654303583889413	0,891901943526937	25,5170328077361	25,0503231987898	25,270357393125	25,346821793299	25,1428879762118	25,1454481767251	95199240415,5475	88983724165,6726	0,097408582715601	0,00923609731283139	0,0046410631090603	0,934710442827653	-0.0974085827156006	1,06985003502778	0.0974085827156007	1721
Q91V12-2	sp|Q91V12-2|BACH_MOUSE Isoform A of Cytosolic acyl coenzyme A thioester hydrolase OS=Mus musculus GN=Acot7;sp|Q91V12-4|BACH_MOUSE Isoform D of Cytosolic acyl coenzyme A thioester hydrolase OS=Mus musculus GN=Acot7;sp|Q91V12|BACH_MOUSE Cytosolic acyl coe	−1,07	0,750190988577539	0,928854093158237	31,9282291432254	31,2665446190202	31,7896349241163	31,471637043656	31,6858278589972	31,625103247153	56286146896532,2	52623788341091,7	0,0970647803797722	0,0110217472516219	0,0034940290890784	0,934933216122027	-0.0970647803797721	1,0695951141279	0.0970647803797722	1722
P35585	sp|P35585|AP1M1_MOUSE AP-1 complex subunit mu-1 OS=Mus musculus GN=Ap1m1 PE=1 SV=3	−1,07	0,564373759034023	0,845110794140252	30,2598956411556	29,8851245258515	30,1964189422983	30,0707031025332	30,0268702489686	30,0424084078288	11974650062198,5	11196928174128,1	0,0968805065864816	0,00666063244024668	0,0007396378685544	0,935052641703033	-0.0968805065864852	1,06945850468769	0.0968805065864852	1723

Q9JMH6-2	sp|Q9JMH6-2|TRXR1_MOUSE Isoform 2 of Thioredoxin reductase 1, cytoplasmic OS=Mus musculus GN=Txnrd1;sp|Q9JMH6|TRXR1_MOUSE Thioredoxin reductase 1, cytoplasmic OS=Mus musculus GN=Txnrd1 PE=1 SV=3	−1,07	0,454186132041736	0,788460969617685	29,3057922937924	29,5830171708225	29,5350096320515	29,4570920219591	29,4005511866025	29,3648762637523	6319153438782,1	5909051777716,55	0,0968046565953967	0,00502652107733445	0,0015812174872637	0,935101803582002	-0,0968046565953961	1,06940227916297	0,0968046565953961	1724
Q6PB93-2	sp|Q6PB93-2|GALT2_MOUSE Isoform 2 of Polypeptide N-acetylgalactosaminyltransferase 2 OS=Mus musculus GN=Galnt2;sp|Q6PB93|GALT2_MOUSE Polypeptide N-acetylgalactosaminyltransferase 2 OS=Mus musculus GN=Galnt2 PE=2 SV=1	−1,07	0,902498627459049	0,977644369603516	23,0632449784514	24,1908952126927	24,3880879517679	24,2991722917027	23,1184157525276	24,024166766318	23511199388,6727	21991421230,3718	0,0964072941438616	0,029932334373254	0,0259435929037358	0,935359394764305	-0,096407294143864	1,06910777354408	0,096407294143864	1725
Q9SCN8	sp|Q9SCN8|CD48D_ARATH Cell division control protein 48 homolog D OS=Arabidopsis thaliana GN=CDC48D PE=1 SV=1;sp|P54609|CD48A_ARATH Cell division control protein 48 homolog A OS=Arabidopsis thaliana GN=CDC48A PE=1 SV=1;sp|Q9LZF6|CD48E_ARATH Cell division	−1,07	0,595453773510819	0,860936823333794	28,9450977968638	28,7253092782756	28,525661745392	28,6000440221418	28,7146432611685	28,6809841596718	3007177178387,9	2812862641444,37	0,0963707675991699	0,00730190863582741	0,0020548516440077	0,935383076747178	-0,0963707675991679	1,06908070592589	0,0963707675991681	1726
Q9D8S4	sp|Q9D8S4|ORN_MOUSE Oligoribonuclease, mitochondrial OS=Mus musculus GN=Rexo2 PE=1 SV=2	−1,07	0,892596555826075	0,977069018898103	26,4519728460025	26,6226931767961	25,3909018070988	26,7044826370826	25,784338153794	25,7769511586397	228588499040,49	213860688109,314	0,0960815086052479	0,025515925200011	0,0204453702454388	0,935570638973541	-0,0960815086052483	1,06886637774048	0,0960815086052486	1727
Q80WC7-2	sp|Q80WC7-2|AGFG2_MOUSE Isoform 2 of Arf-GAP domain and FG repeat-containing protein 2 OS=Mus musculus GN=Agfg2;sp|Q80WC7|AGFG2_MOUSE Arf-GAP domain and FG repeat-containing protein 2 OS=Mus musculus GN=Agfg2 PE=1 SV=1	−1,07	0,71888450748011	0,920149210230605	26,3529925413592	26,3789076381336	26,4055239012705	26,5247335525472	26,4674873848154	25,9455567246878	285966743705,303	267555418561,231	0,0960096327361359	0,000995728385973822	0,0121291159381756	0,935617250784079	-0,0960096327361369	1,06881312754972	0,0960096327361368	1728
Q3UJP5	sp|Q3UJP5|CH037_MOUSE Protein C8orf37 homolog OS=Mus musculus PE=1 SV=1	−1,07	0,75976018145175	0,933136198610489	25,8471838387831	26,2458051503349	26,5887417463319	26,2041255291928	26,0967000396117	26,1820526790869	245667191906,419	229911299230,029	0,0956278325564498	0,0141504479215921	0,0021686535444099	0,935864888778507	-0,0956278325564511	1,06853031029426	0,095627832556451	1729
Q9R0N0	sp|Q9R0N0|GALK1_MOUSE Galactokinase OS=Mus musculus GN=Galk1 PE=2 SV=2	−1,07	0,575841472964862	0,849998193752035	27,3519448291009	27,1789632879833	27,2928682782776	27,0764572082296	27,4198860408206	27,1289472953639	700172193033,631	655346658024,586	0,0954515176163035	0,00322377148491957	0,0067992626795938	0,935979269878129	-0,0954515176163023	1,06839973082973	0,0954515176163023	1730
Q9R0P6	sp|Q9R0P6|SC11A_MOUSE Signal peptidase complex catalytic subunit SEC11A OS=Mus musculus GN=Sec11a PE=2 SV=1	−1,07	0,77351881611517	0,93826881516482	25,0723990419734	24,8971877540398	24,8070307785967	24,4377243018323	24,9908094544841	25,1499790121567	66838105170,544	62567027319,9255	0,0952682737966209	0,00541359481251079	0,0150381728971867	0,936098160776395	-0,0952682737966207	1,06826403672303	0,0952682737966206	1731
O35286	sp|O35286|DHX15_MOUSE Putative pre-mRNA-splicing factor ATP-dependent RNA helicase DHX15 OS=Mus musculus GN=Dhx15 PE=1 SV=2	−1,07	0,672767763955808	0,900894908348996	29,7910678192371	29,4999946244056	29,3143252751957	29,339015370216	29,4859248540224	29,5830171708225	6713417418775,8	6285830766707,87	0,0949439163449844	0,00813625756169914	0,0041685918085348	0,93630864500216	-0,0949439163449826	1,06802388863738	0,0949439163449826	1732
Q8N5M9	sp|Q8N5M9|JAGN1_HUMAN Protein jagunal homolog 1 OS=Homo sapiens GN=JAGN1 PE=1 SV=1;sp|Q5XKN4|JAGN1_MOUSE Protein jagunal homolog 1 OS=Mus musculus GN=Jagn1 PE=2 SV=2	−1,07	0,617753690788259	0,870166479711399	23,8911102657415	23,9303837516722	23,6075161552656	23,6309486811925	23,6951786116361	23,9062094718821	21898195159,3965	20508643223,7306	0,0945799167837609	0,00739902099559724	0,0060649540820583	0,936544910411503	-0,0945799167837624	1,06775445457348	0,0945799167837624	1733
O08583	sp|O08583|THOC4_MOUSE THO complex subunit 4 OS=Mus musculus GN=Alyref PE=1 SV=3;sp|O08583-2|THOC4_MOUSE Isoform 2 of THO complex subunit 4 OS=Mus musculus GN=Alyref	−1,07	0,81934730942128	0,952523073888973	28,0160964439985	27,8617602051029	28,4734319680636	28,3097572274689	27,6170252356209	28,2288491072506	1625922081780,31	1523265025470,68	0,0940911503895793	0,011313328340866	0,0135021060000118	0,936862253449916	-0,0940911503895796	1,06739277446347	0,0940911503895796	1734
Q9Z1Q5	sp|Q9Z1Q5|CLIC1_MOUSE Chloride intracellular channel protein 1 OS=Mus musculus GN=Clic1 PE=1 SV=3;sp|O00299|CLIC1_HUMAN Chloride intracellular channel protein 1 OS=Homo sapiens GN=CLIC1 PE=1 SV=4	−1,07	0,725036773295863	0,921649709826669	27,7199698313342	28,1372043839272	28,344311301084	28,0390196313671	27,9562316171464	28,0108060548788	1546726439193,01	1449180153578,75	0,0939811045591127	0,0113303045294983	0,0015030225921894	0,936933718114269	-0,0939811045591104	1,06731135902832	0,0939811045591106	1735
Q8BVG4	sp|Q8BVG4|DPP9_MOUSE Dipeptidyl peptidase 9 OS=Mus musculus GN=Dpp9 PE=2 SV=2;sp|Q8BVG4-2|DPP9_MOUSE Isoform 2 of Dipeptidyl peptidase 9 OS=Mus musculus GN=Dpp9	−1,07	0,845854697357881	0,961034911466188	27,5020965064079	27,3642301077782	26,7159035528554	27,541410950126	27,1039852603133	26,7414467689503	646007352567,185	605274347900,82	0,0939613755595541	0,0154374888071278	0,0147652563621826	0,936946530864556	-0,0939613755595549	1,06729676353811	0,093961375559555	1736
P46471	sp|P46471|PRS7_MOUSE 26S protease regulatory subunit 7 OS=Mus musculus GN=Psmc2 PE=1 SV=5	−1,07	0,459001430477145	0,790967157530661	30,9084445981046	30,620839005307	30,688685623596	30,6509513772108	30,6837245297255	30,688685623596	22383004870164,8	20977128550922,9	0,0935865195412663	0,00489083007854894	0,0006684497220575	0,93719000967936	-0,0935865195412702	1,06701948342592	0,0935865195412703	1737
P63024	sp|P63024|VAMP3_MOUSE Vesicle-associated membrane protein 3 OS=Mus musculus GN=Vamp3 PE=1 SV=1	−1,07	0,674610336419043	0,901104029547498	26,6952330944468	26,8788895373272	26,3998169118976	26,5597469568507	26,4988608819045	26,7207981765655	377931896328,48	354202754321,314	0,0935508522127788	0,00906667314134874	0,0043121658710023	0,937213179840894	-0,0935508522127766	1,06699310414069	0,0935508522127767	1738

Q9D824-4	sp|Q9D824-4|FIP1_MOUSE Isoform 4 of Pre-mRNA 3-end-processing factor FIP1 OS=Mus musculus GN=Fip1l1;sp|Q9D824-3|FIP1_MOUSE Isoform 3 of Pre-mRNA 3-end-processing factor FIP1 OS=Mus musculus GN=Fip1l1;sp|Q9D824-2|FIP1_MOUSE Isoform 2 of Pre-mRNA 3-end	−1,07	0,913293408867841	0,980665300581806	23,4427204772675	23,4148105713821	21,736986923389	22,6546864138481	22,5905470800734	23,1560227153882	8512821911,30331	7981711798,04054	0,0929392622274361	0,0427227368604599	0,0135798982382675	0,937610569233504	-0.0929392622274375	1,06654087828543	0.0929392622274376	1739
O88271	sp|O88271|CFDP1_MOUSE Craniofacial development protein 1 OS=Mus musculus GN=Cfdp1 PE=1 SV=1	−1,07	0,791921983909429	0,942359320384531	27,2944083728934	26,5794042509612	27,2405304600518	26,9530356848158	27,1253677426794	26,8427056892879	552718330884,948	518239350416,762	0,0929258953667048	0,0147261365517699	0,0052814352910620	0,937619256424909	-0.092925895366705	1,06653099661471	0.0929258953667051	1740
P45376	sp|P45376|ALDR_MOUSE Aldose reductase OS=Mus musculus GN=Akr1b1 PE=1 SV=3	−1,07	0,573695281674191	0,849998193752035	31,8989697166028	31,6520009366872	31,5115733756014	31,5898004571495	31,6424407003006	31,63710827341	57771383641310,4	54168272619642,8	0,0929069628354214	0,0061893369859876	0,0009162742651112	0,937631560911913	-0.0929069628354233	1,06651700058755	0.0929069628354232	1741
Q9CT10	sp|Q9CT10|RANB3_MOUSE Ran-binding protein 3 OS=Mus musculus GN=Ranbp3 PE=1 SV=2	−1,07	0,636534289780391	0,883588334123886	27,6419884256892	27,9732115534468	27,6913415808811	27,539642809425	27,7288300481742	27,8449372025876	1147775901103,52	1076213545203,56	0,0928766190149001	0,0064351519928678 9	0,0055622107651316	0,937651282074178	-0.09287661901490 8	1,06649456905546	0.0928766190149079	1742
Q69ZK9	sp|Q69ZK9|NLGN2_MOUSE Neuroligin-2 OS=Mus musculus GN=Nlgn2 PE=1 SV=2	−1,07	0,762569125506523	0,933454063694469	28,4991310077434	27,9465752622869	27,9732115534468	28,0044923642566	28,2953913503404	27,9268847299193	1662991615762,25	1559816779892,4	0,0924043187122525	0,0110738344749547	0,0069194928432581	0,937958294622815	-0.0924043187122556	1,06614548400804	0.0924043187122555	1743
Q9CPR1	sp|Q9CPR1|RWDD4_MOUSE RWD domain-containing protein 4 OS=Mus musculus GN=Rwdd4 PE=2 SV=1	−1,07	0,835685865085701	0,958131770704506	24,5010625962898	24,9261116536089	25,286634641881	24,4296234518414	24,9825849920557	25,109689615665	65453314120,0618	61397360742,1857	0,0922896019777784	0,0157893482846231	0,0145557251571342	0,938032879886934	-0.0922896019777776	1,06606071220077	0.0922896019777775	1744
Q9CQA1	sp|Q9CQA1|TPPC5_MOUSE Trafficking protein particle complex subunit 5 OS=Mus musculus GN=Trappc5 PE=1 SV=1	−1,07	0,657370071008316	0,892285512053452	28,4245313453588	28,2400659404567	28,092191112154	27,9786139581145	28,3270681210585	28,2599624963169	1861237675547,83	1746348568662,33	0,0919207482625801	0,0058935277898595 1	0,0065586403488383	0,938272737332332	-0.0919207482625773	1,06578818739119	0.0919207482625774	1745
P59325	sp|P59325|IF5_MOUSE Eukaryotic translation initiation factor 5 OS=Mus musculus GN=Eif5 PE=1 SV=1	−1,07	0,7504635483199 4	0,928854093158237	28,7146432611685	29,258547537147	29,2155065783681	29,1918467273542	28,8709870434371	28,934830454227	4186554202842,12	3928274584333,37	0,0918675268510057	0,01040382253783 8	0,0058568871083045	0,93830735110669	-0.0918675268510047	1,06574887090093	0.0918675268510048	1746
Q8C561-3	sp|Q8C561-3|LMBD2_MOUSE Isoform 3 of LMBR1 domain-containing protein 2 OS=Mus musculus GN=Lmbrd2;sp|Q8C561-2|LMBD2_MOUSE Isoform 2 of LMBR1 domain-containing protein 2 OS=Mus musculus GN=Lmbrd2;sp|Q8C561|LMBD2_MOUSE LMBR1 domain-containing protein 2 OS=	−1,07	0,919774254524823	0,98176599643834	23,9198035652238	22,6225346670564	21,9456502500876	23,220546028412	22,7197042232794	22,3570874543215	8215837197,8753	7709963075,4797	0,0916836431962836	0,0439431043615132	0,0190447828707789	0,938426953916951	-0.0916836431962836	1,06561304087233	0.0916836431962836	1747
Q9D1Q6	sp|Q9D1Q6|ERP44_MOUSE Endoplasmic reticulum resident protein 44 OS=Mus musculus GN=Erp44 PE=1 SV=1	−1,07	0,673470511307769	0,900894908348996	28,8140965858009	29,0568893313668	29,0865735685853	28,6809841596718	29,089228896999	28,9967840041252	3876109893757,17	3637553126702,94	0,0916411551551025	0,00515714375978683	0,0074013258609639	0,938454591434973	-0.0916411551551036	1,06558165853387	0.0916411551551034	1748
P50136	sp|P50136|ODBA_MOUSE 2-oxoisovalerate dehydrogenase subunit alpha, mitochondrial OS=Mus musculus GN=Bckdha PE=1 SV=1	−1,07	0,694832067909801	0,910462689446474	26,8660517354712	26,6533800549077	27,0337631233805	26,5628276441229	26,9600309433248	26,8407111037321	458426115290,481	430346512322,035	0,0911904560810868	0,00709967627855715	0,0076082341447549	0,938747811191661	-0.0911904560810845	1,06524882197124	0.0911904560810844	1749
Q91W18-2	sp|Q91W18-2|TDRD3_MOUSE Isoform 2 of Tudor domain-containing protein 3 OS=Mus musculus GN=Tdrd3;sp|Q91W18-3|TDRD3_MOUSE Isoform 3 of Tudor domain-containing protein 3 OS=Mus musculus GN=Tdrd3;sp|Q91W18|TDRD3_MOUSE Tudor domain-containing protein 3 OS=Mu	−1,06	0,599648311810359	0,861904596278927	23,9322127236138	24,0997473766248	23,735889620616	23,93947115125	23,7476238449328	23,8919023724206	24516607294,5414	23020841380,1307	0,090818784017667	0,00761282071867555	0,0041871365867073	0,938989685789692	-0.0908187840176704	1,0649744242494	0.0908187840176704	1750
Q99MN1	sp|Q99MN1|SYK_MOUSE Lysine--tRNA ligase OS=Mus musculus GN=Kars PE=1 SV=1	−1,06	0,613911847179861	0,868502403759507	29,6607221554744	29,6259603347948	29,2904693642646	29,5295487853568	29,3941367297357	29,465248371337	6650527460668,46	6246097359370,17	0,0905137097301043	0,00692517440204519	0,0022989723673237	0,939188266841975	-0.0905137097301047	1,06474924709452	0.0905137097301046	1751
P62746	sp|P62746|RHOB_MOUSE Rho-related GTP-binding protein RhoB OS=Mus musculus GN=Rhob PE=1 SV=1	−1,06	0,861789997705825	0,967230529051203	27,9932539266564	28,8294358832546	29,142784813033	28,5402325045527	28,4357489471046	28,801510817816	2784691490166,42	2615554987282,58	0,0904004030422467	0,0207380289507448	0,0065891033070787	0,93926203190511	-0.0904004030422441	1,06466562687698	0.090400403042244	1752
P84091	sp|P84091|AP2M1_MOUSE AP-2 complex subunit mu OS=Mus musculus GN=Ap2m1 PE=1 SV=1	−1,06	0,505813221099733	0,815013020393559	33,1052341468871	33,0157551936444	33,1690884811052	33,1282548359372	33,1052341468871	32,8687527944652	236434565566493	222084841215290	0,090330043226659	0,00232720161357395	0,0043482365483946	0,939307840556979	-0.0903300432266597	1,06461370471158	0.0903300432266596	1753
Q9D7N9	sp|Q9D7N9|APMAP_MOUSE Adipocyte plasma membrane-associated protein OS=Mus musculus GN=Apmap PE=1 SV=1	−1,06	0,515949718130521	0,820430016377981	29,7730411693604	29,4486387307347	29,5542026275958	29,541802057065	29,531702480219	29,5156147134624	7106001627531,9	6677110278985,22	0,0898141511559957	0,00559153519653198	0,0004472542767914	0,939643786896282	-0.0898141511559958	1,06423307847656	0.0898141511559957	1754
Q3U1V6	sp|Q3U1V6|UEVLD_MOUSE Ubiquitin-conjugating enzyme E2 variant 3 OS=Mus musculus GN=Uevld PE=2 SV=1	−1,06	0,777177022502835	0,938755143139587	26,2158504682045	25,5041690285268	26,062710168417	25,7926120679867	25,8849767619169	25,9187584767025	182055342059,195	171088893294,066	0,089630968125985	0,0144475912297158	0,0025246436986399	0,939763103674468	-0.0896309681259895	1,06409795840037	0.0896309681259895	1755
Q99J31	sp|Q99J31|OPHN1_MOUSE Oligophrenin-1 OS=Mus musculus GN=Ophn1 PE=1 SV=1	−1,06	0,712658346860779	0,917434617372953	27,0003491513743	26,9395921810098	26,6065658100871	26,8725182103567	26,9302714663707	26,5575313626245	457405303868,243	429880749223,285	0,0895365892175657	0,00789604898956891	0,0074894710832276	0,939824583553831	-0.0895365892175663	1,06402834901235	0.0895365892175664	1756

Q9DBX2	sp|Q9DBX2|PHLP_MOUSE Phosducin-like protein OS=Mus musculus GN=Pdcl PE=1 SV=1	−1,06	0,855149728426948	0,964114410663169	25,3293144456964	24,7472989515349	24,7436143644013	25,1630860770961	25,1478806807997	24,3237320068627	67816808933,8539	63749866163,4679	0,089220587910473	0,0135162172831583	0,0193049104068076	0,940030460967977	-0.0892205879104735	1,0637953146436	0.089220587910473	1757
O88342	sp|O88342|WDR1_MOUSE WD repeat-containing protein 1 OS=Mus musculus GN=Wdr1 PE=1 SV=3	−1,06	0,747615475437658	0,928122477394312	32,1886810722787	32,7488011970875	32,6477383197789	32,6186624488613	32,3276858928127	32,4536789640953	133939170731530	125920990741467	0,0890591438440617	0,00917718081422115	0,0044945475020958	0,940135660492219	-0.0890591438440654	1,0636762778219	0.089059143844065	1758
Q91YL3	sp|Q91YL3|UCKL1_MOUSE Uridine-cytidine kinase-like 1 OS=Mus musculus GN=Uckl1 PE=2 SV=1	−1,06	0,826037537043801	0,955744170441451	26,7551771799298	27,4559070409885	27,4482593170704	26,8591980973836	27,23216720089	27,3835409950841	662827875853,607	623305183856,613	0,088695566446687	0,0147825228045099	0,0099366579656590	0,940372616426104	-0.088695566446688	1,06340825172102	0.088695566446687	1759
Q99LL5	sp|Q99LL5|PWP1_MOUSE Periodic tryptophan protein 1 homolog OS=Mus musculus GN=Pwp1 PE=1 SV=1	−1,06	0,824538307866196	0,95563531252039	23,4391885073479	23,9198035652238	23,7832496113561	23,1990977632119	23,766323408059	23,9931989064267	19901890443,1722	18720281761,2715	0,088303326902725	0,0104436269742641	0,0172928710575083	0,94062831944158	-0.0883033269027243	1,06311917186766	0.0883033269027244	1760
Q9D7P6	sp|Q9D7P6|ISCU_MOUSE Iron-sulfur cluster assembly enzyme ISCU, mitochondrial OS=Mus musculus GN=Iscu PE=1 SV=1	−1,06	0,787816487625203	0,942041082015698	27,680487545215	27,5128434547509	27,2381962259367	27,0626686517635	27,4854015248497	27,6999254581679	857404587837,284	806523235934,717	0,088260038785806	0,00812647214931601	0,0118269559007382	0,940656543451779	-0.0882600387858048	1,06308727341699	0.0882600387858048	1761
Q9CQK7	sp|Q9CQK7|RWDD1_MOUSE RWD domain-containing protein 1 OS=Mus musculus GN=Rwdd1 PE=1 SV=1	−1,06	0,711512342553586	0,916805961322165	27,3594544203214	27,2268519579805	27,612789504722	27,4482593170704	27,4696095384182	27,097740617644	793483535650,266	746406721037,328	0,088238311206877	0,00715668124129547	0,0076378777269302	0,94067071023174	-0.0882383112068784	1,06307126300727	0.0882383112068784	1762
O88735	sp|O88735|MAP7_MOUSE Ensconsin OS=Mus musculus GN=Map7 PE=1 SV=1;sp|O88735-2|MAP7_MOUSE Isoform 2 of Ensconsin OS=Mus musculus GN=Map7	−1,06	0,929331550384261	0,98433518830764	22,8146495088482	20,5085414095425	22,0428427182337	21,4780831635369	21,8938035010294	21,8106908372461	2902034497,63241	2729886401,04163	0,088223751971295	0,0538754765734674	0,0101246898834313	0,940680203239753	-0.0882237519712936	1,06306053487247	0.0882237519712936	1763
Q68FF6	sp|Q68FF6|GIT1_MOUSE ARF GTPase-activating protein GIT1 OS=Mus musculus GN=Git1 PE=1 SV=1	−1,06	0,751305740117272	0,928854093158237	30,7148429894456	30,6923605230542	30,1871752749852	30,5864926907295	30,5191558658654	30,3063347984404	18182116114261,3	17109605011867,1	0,087713661958673	0,00977256921174898	0,0047993459568079	0,941012855948436	-0.0877136619586735	1,06268473770437	0.0877136619586736	1764
P62073	sp|P62073|TIM10_MOUSE Mitochondrial import inner membrane translocase subunit Tim10 OS=Mus musculus GN=Timm10 PE=1 SV=1	−1,06	0,840404914688203	0,959771286845933	24,163506496647	24,4227555609276	24,1461515325522	24,0624940540361	23,7565991016167	24,7312567640459	33813902227,4476	31822835713,9987	0,087554118150916	0,00639043809758978	0,0206116070602951	0,941116925811872	-0.0875541181509152	1,06256722472325	0.0875541181509151	1765
O70492	sp|O70492|SNX3_MOUSE Sorting nexin-3 OS=Mus musculus GN=Snx3 PE=1 SV=3	−1,06	0,726574567390723	0,92223768495148	29,4440052105859	29,3758840404762	29,1173946133026	28,9936791154053	29,2784329903639	29,483312762562	5373109755999,28	5057069541395,7	0,087455690572874	0,00587818515520545	0,008406326471697	0,941181135514548	-0.087455690572874	1,06249473376163	0.087455690572874	1766
Q9CR00	sp|Q9CR00|PSMD9_MOUSE 26S proteasome non-ATPase regulatory subunit 9 OS=Mus musculus GN=Psmd9 PE=1 SV=1	−1,06	0,75129267607724	0,928854093158237	27,889164479335	28,1636447074317	28,5161702425418	28,2478477131411	28,0563316653839	28,0831095688452	1748290920436,39	1645550863948,44	0,087374652423037	0,0111498821142532	0,003686916087094	0,941234004428562	-0.087374652423035	1,06243505365822	0.087374652423034	1767
P70288	sp|P70288|HDAC2_MOUSE Histone deacetylase 2 OS=Mus musculus GN=Hdac2 PE=1 SV=1	−1,06	0,782293085771054	0,940251150142017	27,6870999533187	28,3076758523563	28,2731322206399	28,187447373342	27,9977571851328	27,9018726638348	1581354185695,06	1488850915590,62	0,086961234726116	0,0124156067283048	0,0051850695743178	0,941503762445362	-0.086961234726118	1,06213064661867	0.086961234726118	1768
O89086	sp|O89086|RBM3_MOUSE RNA-binding protein 3 OS=Mus musculus GN=Rbm3 PE=2 SV=1	−1,06	0,82924501777787	0,956900945145962	27,0148666296592	26,7995725862334	26,0967000396117	26,5613075057639	26,5816409484069	26,5887417463319	370102717344,459	348613629411,583	0,086296753914265	0,018026605538527	0,000535762242388	0,941937502953063	-0.0862967539142639	1,06164155993886	0.086296753914264	1769
Q8BVE3	sp|Q8BVE3|VATH_MOUSE V-type proton ATPase subunit H OS=Mus musculus GN=Atp6v1h PE=1 SV=1	−1,06	0,642600936110486	0,887784569747338	32,72254043549	32,3520069298941	32,4429895051478	32,3737547971211	32,574532753597	32,390014054121	130951170256711	123356608781872	0,086193942989103	0,0059404164790465	0,0034371548277403	0,942004630734105	-0.0861939429891035	1,06156590676279	0.086193942989103	1770
Q9JII1	sp|Q9JII1|INP5E_MOUSE 72 kDa inositol polyphosphate 5-phosphatase OS=Mus musculus GN=Inpp5e PE=1 SV=1	−1,06	0,612904107750884	0,868059454773913	21,9944238728401	21,740785022867	21,6429764340917	21,6695873010279	21,8402680937608	21,6895799900045	2913813214,35223	2745269617,13877	0,085960553072943	0,00832342899496179	0,004293360256073	0,942157034506095	-0.0859605530729427	1,06139418735458	0.085960553072942	1771
Q8R1B5	sp|Q8R1B5|CPLX3_MOUSE Complexin-3 OS=Mus musculus GN=Cplx3 PE=1 SV=1	−1,06	0,818083945090897	0,952368049953214	25,2075881976355	25,0588842328911	24,8234435167563	24,5294754056694	25,0609738563947	25,3211500311766	74195696176,6689	69914094068,8094	0,085752184164917	0,00773862244201489	0,0161595214956803	0,942293120376356	-0.0857521841649162	1,06124090092114	0.0857521841649162	1772
Q64521	sp|Q64521|GPDM_MOUSE Glycerol-3-phosphate dehydrogenase, mitochondrial OS=Mus musculus GN=Gpd2 PE=1 SV=2	−1,06	0,29661093779587	0,711969538636193	32,574532753597	32,5441233278785	32,4047952999906	32,4047952999906	32,4603983928778	32,4800692515926	131209595625733	123643183560122	0,085690524803700	0,00278412522814198	0,001203145788086	0,942333393914316	-0.0856905248036977	1,06119554550237	0.085690524803697	1773
Q9CX00	sp|Q9CX00|IST1_MOUSE IST1 homolog OS=Mus musculus GN=Ist1 PE=2 SV=1	−1,06	0,809993994673675	0,94879038498679	27,7577253480226	27,4194342735147	27,1365607688038	27,0435496348728	27,5920034587656	27,5000243142379	824387725449,464	776859853737,432	0,085668665790412	0,0113344661566382	0,0107281394191347	0,942347671799554	-0.0856686657904127	1,06117946690562	0.0856686657904127	1774
Q64471	sp|Q64471|GSTT1_MOUSE Glutathione S-transferase theta-1 OS=Mus musculus GN=Gstt1 PE=1 SV=4	−1,06	0,733945026023324	0,924701393764781	26,794197060117	27,0697573276934	26,9378482697719	27,173042131448	26,7926130600652	26,6595208620373	498034017162,83	469558479360,62	0,084939441908578	0,00511703276099724	0,009916378487314	0,942824110761695	-0.0849394419085782	1,06064321922369	0.0849394419085781	1775

O55128	sp|O55128|SAP18_MOUSE Histone deacetylase complex subunit SAP18 OS=Mus musculus GN=Sap18 PE=1 SV=1	−1,06	0,647819819735202	0,889714023663571	24,6838243385297	24,6622689173269	24,3086666205643	24,517555127317	24,5493168001552	24,4117497112943	45985254397,6581	43363065032,084	0,0847045873249401	0,00857992576782174	0,0029407783160956	0,942977604453404	-0.08470458732494	1,06047057244763	0.0847045873249402	1776
Q921Q7	sp|Q921Q7|RIN1_MOUSE Ras and Rab interactor 1 OS=Mus musculus GN=Rin1 PE=1 SV=1	−1,06	0,769496462113732	0,936218563920244	28,2133967375042	28,2559271319953	27,6931390764562	27,8257406617401	28,100354168705	28,0603919717577	1526737642946,09	1439757195026,15	0,0846266366357375	0,0111702074649194	0,0052995574791321	0,943028556136143	-0.084626636635736	1,06041327539145	0.084626636635736	1777
Q9Z1P6	sp|Q9Z1P6|NDUA7_MOUSE NADH dehydrogenase [ubiquinone] 1 alpha subcomplex subunit 7 OS=Mus musculus GN=Ndufa7 PE=1 SV=3	−1,06	0,776468177977491	0,938645209528714	25,5956685115229	25,3944926619741	24,903996713159	25,2820160355893	25,2204546504586	25,2157838114411	97007354182,0157	91482923722,6985	0,0845916490755997	0,0140634536815176	0,0014645617617219	0,943051426297519	-0.0845916490756006	1,06038755906034	0.0845916490756006	1778
P16675	sp|P16675|PPGB_MOUSE Lysosomal protective protein OS=Mus musculus GN=Ctsa PE=1 SV=1	−1,06	0,648753693426108	0,889714023663571	26,587342146856	26,6277756439543	26,3000797338373	26,2985172198808	26,5575313626245	26,4832460049278	324342483257,608	305871687524,78	0,0845914317324295	0,0067409994121175	0,0050431667207706	0,94305156836899	-0.0845914317324258	1,06038739931211	0.0845914317324259	1779
Q8BVU5	sp|Q8BVU5|NUDT9_MOUSE ADP-ribose pyrophosphatase, mitochondrial OS=Mus musculus GN=Nudt9 PE=2 SV=1	−1,06	0,550398564046479	0,838423390503939	26,871435906167	26,8446549836125	26,9234814664797	26,6308893490547	26,9044098251012	26,9283757191714	471817196558,308	444948759122,118	0,0845887991589633	0,00149116706158319	0,0061619282134659	0,943053289214164	-0.0845887991589625	1,06038546436044	0.0845887991589624	1780
Q8BRT1	sp|Q8BRT1|CLAP2_MOUSE CLIP-associating protein 2 OS=Mus musculus GN=Clasp2 PE=1 SV=1	−1,06	0,7591005552031	0,933136198610489	26,1820526790869	25,810667197616	25,5382714274755	25,8152971897798	25,8362365598065	25,7036960429525	167401970821,456	157876133530,135	0,0845234204071346	0,0125042854605293	0,0027632664542953	0,943096026620374	-0.0845234204071388	1,06033741185778	0.0845234204071389	1781
Q8BYP3	sp|Q8BYP3|RHOF_MOUSE Rho-related GTP-binding protein RhoF OS=Mus musculus GN=Rhof PE=2 SV=1	−1,06	0,580404015669663	0,853379506269256	24,7358047995479	24,7832884827572	24,567454870373	24,4792576365056	24,6690327146511	24,7625063508653	53103891613,1819	50082237137,0406	0,0845185820968765	0,00459261664228392	0,0058581571893235	0,943099189450152	-0.0845185820968757	1,06033385585139	0.0845185820968757	1782
Q8K2C7-2	sp|Q8K2C7-2|OS9_MOUSE Isoform 2 of Protein OS-9 OS=Mus musculus GN=Os9;sp|Q8K2C7|OS9_MOUSE Protein OS-9 OS=Mus musculus GN=Os9 PE=1 SV=2	−1,06	0,713929868934088	0,917934955801873	26,0103345112904	25,7624652702272	25,6236903849764	25,6077300356249	25,9627882430883	25,6510636985623	160062476725,609	150997229476,991	0,0841130590654436	0,00759222071735423	0,0075250680876972	0,943364319770221	-0.0841130590654455	1,06003585151872	0.0841130590654457	1783
Q9DBS1	sp|Q9DBS1|TMM43_MOUSE Transmembrane protein 43 OS=Mus musculus GN=Tmem43 PE=1 SV=1	−1,06	0,770960958587566	0,936218563920244	25,6586432911233	25,0467789403677	25,0723990419734	25,1963348706422	25,2191352268015	25,1876434359737	93317524600,553	88038305322,7103	0,0840166633902645	0,013701950082609	0,0006453751240491	0,943427354074806	-0.0840166633902639	1,05996502611552	0.0840166633902639	1784
Q9ERB0	sp|Q9ERB0|SNP29_MOUSE Synaptosomal-associated protein 29 OS=Mus musculus GN=Snap29 PE=2 SV=1	−1,06	0,813818262376764	0,950652201935115	26,5628276441229	27,1253677426794	27,3125156226977	27,1138793678718	26,9469309959916	26,7655997645261	532174352663,522	502135936252,933	0,0838210057926275	0,0144514996979805	0,0064653109654277	0,943555309908778	-0.0838210057926276	1,05982128392312	0.0838210057926275	1785
Q61576	sp|Q61576|FKB10_MOUSE Peptidyl-prolyl cis-trans isomerase FKBP10 OS=Mus musculus GN=Fkbp10 PE=1 SV=2	−1,06	0,854427295611373	0,963607051252994	22,6869167687749	21,8141497464129	21,9088833413978	22,3703619013105	21,8565573461608	22,0092765517313	4109840069,61578	3878568321,04762	0,0835580389736003	0,0216335036569764	0,0119504141697444	0,943727311853821	-0.0835580389736025	1,05962812291152	0.0835580389736025	1786
P62881-2	sp|P62881-2|GBB5_MOUSE Isoform 2 of Guanine nucleotide-binding protein subunit beta-5 OS=Mus musculus GN=Gnb5;sp|P62881|GBB5_MOUSE Guanine nucleotide-binding protein subunit beta-5 OS=Mus musculus GN=Gnb5 PE=1 SV=1	−1,06	0,673038635892484	0,900894908348996	30,3763944677086	29,9201519045597	30,1853711148321	30,1282886884742	30,1478585778955	30,0323123257719	12548712195876,2	11843731592884,1	0,0834156149532952	0,00759680769742966	0,0020543044845249	0,943820481975531	-0.0834156149532952	1,05952352073021	0.0834156149532952	1787
Q8BWG8	sp|Q8BWG8|ARRB1_MOUSE Beta-arrestin-1 OS=Mus musculus GN=Arrb1 PE=1 SV=1;sp|Q8BWG8-2|ARRB1_MOUSE Isoform 1B of Beta-arrestin-1 OS=Mus musculus GN=Arrb1	−1,06	0,637889298569056	0,884355383691187	29,3586082207491	29,0169853290817	29,2784329903639	29,1625520471545	29,2745375866608	29,0435033047516	4888999741892,71	4614375458803,31	0,0834039323306647	0,00611428729326616	0,0039620833747359	0,943828124854209	-0.0834039323306638	1,05951494097982	0.0834039323306638	1788
Q8CD92-2	sp|Q8CD92-2|TTC27_MOUSE Isoform 2 of Tetratricopeptide repeat protein 27 OS=Mus musculus GN=Ttc27;sp|Q8CD92|TTC27_MOUSE Tetratricopeptide repeat protein 27 OS=Mus musculus GN=Ttc27 PE=2 SV=2	−1,06	0,833617813239369	0,957953242267243	25,6721471841847	24,8684973230597	25,1800505226162	25,4178300813803	24,9183070413901	25,2111392176563	91557376348,1984	86414856379,2572	0,0833967610812323	0,016053371795518	0,0099671244821895	0,943832816381895	-0.0833967610812317	1,0595096744288	0.0833967610812317	1789
P17225	sp|P17225|PTBP1_MOUSE Polypyrimidine tract-binding protein 1 OS=Mus musculus GN=Ptbp1 PE=1 SV=2	−1,06	0,666166820230551	0,89771450844655	26,1671183698062	26,5902926789079	26,4330141857912	26,4261270803001	26,251725356772	26,3399692238663	291063825447,141	274790241938,664	0,0830047732081749	0,00810316889886869	0,0033107572113134	0,944089295591862	-0.0830047732081728	1,05922183915144	0.0830047732081728	1790
P62196	sp|P62196|PRS8_MOUSE 26S protease regulatory subunit 8 OS=Mus musculus GN=Psmc5 PE=1 SV=1	−1,06	0,428597723331003	0,774444691958369	30,8405947262776	30,5924988627797	30,7235744445341	30,6837245297255	30,6563201848299	30,6442031737676	21930277770913,5	20705406439997,3	0,08291656284267	0,00404032413111105	0,0006603563039142	0,944147021587626	-0.0829165628426723	1,05915707737811	0.0829165628426721	1791
Q80XR2	sp|Q80XR2|AT2C1_MOUSE Calcium-transporting ATPase type 2C member 1 OS=Mus musculus GN=Atp2c1 PE=1 SV=2	−1,06	0,799426977020573	0,944947668931349	26,5728087866134	26,1785692818595	25,8115297761429	26,1853542778432	26,1745346539103	26,0312420351845	236127074470,469	222986482277,458	0,0826072165217277	0,014538176866478	0,0032920941662995	0,944349489686942	-0.0826072165217263	1,05892999458443	0.0826072165217262	1792
Q920Q4	sp|Q920Q4|VPS16_MOUSE Vacuolar protein sorting-associated protein 16 homolog OS=Mus musculus GN=Vps16 PE=1 SV=3;sp|Q920Q4-2|VPS16_MOUSE Isoform 2 of Vacuolar protein sorting-associated protein 16 homolog OS=Mus musculus GN=Vps16	−1,06	0,765110913301463	0,934060902494713	27,8851022330226	27,5035939037474	27,4581127618677	27,4054318063668	27,8286564820045	27,4410098735373	984700101436,311	929921540024,388	0,0825754094487713	0,00849152030997411	0,0085184060499046	0,944370309973543	-0.0825754094487714	1,05890664863026	0.0825754094487713	1793

Q9QUR7	sp|Q9QUR7|PIN1_MOUSE Peptidyl-prolyl cis-trans isomerase NIMA-interacting 1 OS=Mus musculus GN=Pin1 PE=1 SV=1;sp|Q13526|PIN1_HUMAN Peptidyl-prolyl cis-trans isomerase NIMA-interacting 1 OS=Homo sapiens GN=PIN1 PE=1 SV=1	−1,06	0,70865828320912	0,916805961322165	29,748150804332	29,7040018754618	29,5194704291796	29,3868050777055	29,5601460600447	29,8531115187151	7585105995518,47	7163507745241,93	0,0825031380153571	0,00409038215077879	0,0079627200964463	0,944417619143934	-0.0825031380153577	1,05885360430532	0.0825031380153577	1794
Q8VE88-2	sp|Q8VE88-2|F1142_MOUSE Isoform 2 of Protein FAM114A2 OS=Mus musculus GN=Fam114a2;sp|Q8VE88|F1142_MOUSE Protein FAM114A2 OS=Mus musculus GN=Fam114a2 PE=2 SV=2	−1,06	0,831597361791033	0,957953242267243	26,5603842110093	27,3661184166555	27,2998217180003	27,1289472953639	27,0501953007888	26,8756565229089	573739586666,159	541856136868,073	0,0824861979360115	0,016519865977453	0,0047978236959061	0,944428708530726	-0.0824861979360115	1,05884117135292	0.0824861979360116	1795
Q8K4X7	sp|Q8K4X7|PLCD_MOUSE 1-acyl-sn-glycerol-3-phosphate acyltransferase delta OS=Mus musculus GN=Agpat4 PE=2 SV=1	−1,06	0,856179059322685	0,964447573623737	22,9604916551048	23,5572160930767	24,0352949440679	23,5452376736717	23,3777626719801	23,4589281980348	16352842364,1601	15446416133,234	0,0822692752519794	0,0228973370581447	0,0035698367945880	0,9445707228908	-0.0822692752519795	1,0586819766545	0.0822692752519794	1796
Q99N87	sp|Q99N87|RT05_MOUSE 28S ribosomal protein S5, mitochondrial OS=Mus musculus GN=Mrps5 PE=2 SV=1	−1,06	0,826432666405113	0,9559576638157	25,3401904290326	25,7260541994384	25,7487841328276	25,7705950123853	25,1009475178655	25,7725191212397	131860395114,858	124555912758,292	0,082217800491782	0,00896783657640342	0,0151549040009781	0,944604425383351	-0.0822178004917716	1,05864420399488	0.0822178004917717	1797
P16045	sp|P16045|LEG1_MOUSE Galectin-1 OS=Mus musculus GN=Lgals1 PE=1 SV=3	−1,06	0,796424824840115	0,944339896000096	28,2792329488929	27,7640796168976	27,7477528357763	27,612789504722	27,9902351106764	28,01920864903	1348960125095,57	1275140844623,67	0,0811910956640539	0,0108214633045178	0,0081346373964307	0,945276899517938	-0.0811910956640581	1,05789107986239	0.0811910956640581	1798
O88447	sp|O88447|KLC1_MOUSE Kinesin light chain 1 OS=Mus musculus GN=Klc1 PE=1 SV=3	−1,06	0,782433695516231	0,940251150142017	29,0088476558689	29,5039457329552	29,5501594072729	29,4599545956542	29,3362258860121	29,0984836692739	5602968142482,02	5297315949812,67	0,0809297312685216	0,0102225751789134	0,0062702722050472	0,945448165169479	-0.0809297312685213	1,05769944544844	0.0809297312685213	1799
Q811D0	sp|Q811D0|DLG1_MOUSE Disks large homolog 1 OS=Mus musculus GN=Dlg1 PE=1 SV=1;sp|Q811D0-2|DLG1_MOUSE Isoform 2 of Disks large homolog 1 OS=Mus musculus GN=Dlg1;sp|Q811D0-3|DLG1_MOUSE Isoform 3 of Disks large homolog 1 OS=Mus musculus GN=Dlg1	−1,06	0,719658448186527	0,920149210230605	30,0077005409481	29,6715275253764	29,6149568203859	29,714294747174	29,8734429990539	29,5382706477389	8446120129409,21	7985667310857	0,0808757973584718	0,00713303100135122	0,0056433684723292	0,945483510594536	-0.0808757973584728	1,05765990500583	0.0808757973584728	1800
Q922E4	sp|Q922E4|PCY2_MOUSE Ethanolamine-phosphate cytidylyltransferase OS=Mus musculus GN=Pcyt2 PE=1 SV=1	−1,06	0,531003096318557	0,829883431939119	29,0935725463537	28,7942855809832	28,8979126911188	28,8574544305787	28,9014392327936	28,8597896366355	3660388015251,19	3462092992830,86	0,0803521114197599	0,00525374982787803	0,0008571388768907	0,945826775305207	-0.08035211141976	1,05727605319411	0.0803521114197599	1801
Q8CEC0-3	sp|Q8CEC0-3|NUP88_MOUSE Isoform 3 of Nuclear pore complex protein Nup88 OS=Mus musculus GN=Nup88;sp|Q8CEC0-2|NUP88_MOUSE Isoform 2 of Nuclear pore complex protein Nup88 OS=Mus musculus GN=Nup88;sp|Q8CEC0|NUP88_MOUSE Nuclear pore complex protein Nup88 OS	−1,06	0,463673901837783	0,793595716606975	24,4958624117327	24,4430889369582	24,5413781637526	24,4846098063473	24,5294754056694	24,2991722917027	43387758272,6801	41037515654,4004	0,0803446528192131	0,00200826205824341	0,0049960622172780	0,945831665155202	-0.0803446528192142	1,05727058718838	0.0803446528192144	1802
Q99K48	sp|Q99K48|NONO_MOUSE Non-POU domain-containing octamer-binding protein OS=Mus musculus GN=Nono PE=1 SV=3	−1,06	0,78177718507702	0,940251150142017	30,7235744445341	31,3310729947621	31,3009254761233	31,1184365708611	30,9950183754577	31,0751300491871	32704189508129,7	30933525062318,5	0,0803042146491501	0,0110020836093404	0,00201581787346	0,94585817681337	-0.0803042146491518	1,05724095272828	0.0803042146491516	1803
Q91ZT5	sp|Q91ZT5|FGD4_MOUSE FYVE, RhoGEF and PH domain-containing protein 4 OS=Mus musculus GN=Fgd4 PE=2 SV=1;sp|Q91ZT5-2|FGD4_MOUSE Isoform 2 of FYVE, RhoGEF and PH domain-containing protein 4 OS=Mus musculus GN=Fgd4;sp|Q91ZT5-3|FGD4_MOUSE Isoform 3 of FYVE,	−1,06	0,805658644522635	0,948116304767499	24,8053173699664	24,3206206984587	24,723259791271	24,4199170205648	24,8983311212619	24,3639618883844	49064386689,3959	46407952739,3636	0,0803041711622869	0,0105383742970538	0,0119581492529874	0,9585820532418	-0.0803041711622896	1,05724092086008	0.0803041711622896	1804
Q8BJY1	sp|Q8BJY1|PSMD5_MOUSE 26S proteasome non-ATPase regulatory subunit 5 OS=Mus musculus GN=Psmd5 PE=1 SV=4	−1,06	0,74290981262941	0,926639317607059	29,4005511866025	29,2720468450267	29,5252640648693	29,5895854758228	29,3780373232367	29,0635435904717	5860683585322,81	5543915465885,59	0,0801636899263908	0,00430668060451431	0,0090204805952486	0,945950311968638	-0.0801636899263897	1,05713797791226	0.0801636899263899	1805
Q99J45	sp|Q99J45|NRBP_MOUSE Nuclear receptor-binding protein OS=Mus musculus GN=Nrbp1 PE=1 SV=1	−1,06	0,779076367521174	0,940251150142017	26,8257491606436	27,1479973377852	27,3476415937145	27,3001174003902	26,8911690458988	26,9639504293006	592211317899,294	560304165105,758	0,0799018453865892	0,00971462044332788	0,0080642816933126	0,946122014508744	-0.0799018453865891	1,05694612815793	0.0799018453865889	1806
Q9QZE5	sp|Q9QZE5|COPG1_MOUSE Coatomer subunit gamma-1 OS=Mus musculus GN=Copg1 PE=2 SV=1	−1,06	0,71768494588322	0,919958195245393	29,6690585737809	29,4808389247203	29,1733008248421	29,483312762562	29,356105721571	29,3179435122904	6110722358765,34	5782095823673,88	0,0797504154822081	0,00850039029075774	0,0029465972727367	0,94622132772567	-0.079750415482207	1,05683519352031	0.0797504154822072	1807
Q8C031	sp|Q8C031|LRC4C_MOUSE Leucine-rich repeat-containing protein 4C OS=Mus musculus GN=Lrrc4c PE=1 SV=2	−1,06	0,719581073343814	0,920149210230605	24,7900819061277	24,4132109090808	24,2783394430416	24,4506102071283	24,4227555609276	24,4430889369582	43406603437,5097	41081274033,7117	0,0794336123065452	0,0108286857413519	0,0005896377002762	0,946429132444199	-0.0794336123065454	1,05660314726096	0.0794336123065452	1808
Q3U186	sp|Q3U186|SYRM_MOUSE Probable arginine--tRNA ligase, mitochondrial OS=Mus musculus GN=Rars2 PE=1 SV=1	−1,06	0,786889455856697	0,941831485187835	26,2250524440376	26,8316021926563	26,7885005791647	26,6173376487642	26,446728601505	26,6160829578104	362051295358,686	342675487614,072	0,0793511163799849	0,0127159679738809	0,0036950603392601	0,946483252530782	-0.0793511163799861	1,05654273049853	0.0793511163799861	1809

Q923T9-2	sp|Q923T9-2|KCC2G_MOUSE Isoform 2 of Calcium/calmodulin-dependent protein kinase type II subunit gamma OS=Mus musculus GN=Camk2g;sp|Q923T9|KCC2G_MOUSE Calcium/calmodulin-dependent protein kinase type II subunit gamma OS=Mus musculus GN=Camk2g PE=1 SV=1;	−1,06	0,752820320085372	0,929556973032509	29,7503945006217	30,2744604699913	30,2703137998586	30,0650549662063	30,0424084078288	30,0229121032537	11791376676988,5	11161131899551,9	0,0792488222822057	0,0100131509974256	0,0007020179209415	0,946550365177753	-0.0792488222821951	1,05646781913418	0.0792488222821951	1810
Q9D0W5	sp|Q9D0W5|PPIL1_MOUSE Peptidyl-prolyl cis-trans isomerase-like 1 OS=Mus musculus GN=Ppil1 PE=2 SV=1	−1,06	0,660436675506591	0,893775214706147	25,7650431834166	26,0820246055589	25,8593137304936	25,6889101757867	25,8903660723575	25,9627882430883	177480605154,456	168021003675,128	0,0790197872568461	0,00628403699901544	0,00549049114536	0,946700646692659	-0.0790197872568457	1,05630011291694	0.0790197872568458	1811
O35343	sp|O35343|IMA3_MOUSE Importin subunit alpha-3 OS=Mus musculus GN=Kpna4 PE=1 SV=1	−1,06	0,822194607476811	0,954216651326321	27,9434810304077	28,5719407915324	28,2742677874623	28,4991310077434	28,1896196889447	27,9366295152886	1881762342325,34	1781470157749,49	0,0790161176125056	0,0111231181387235	0,0099872033674965	0,946703054726923	-0.0790161176125024	1,05629742611156	0.0790161176125023	1812
P59913	sp|P59913|PCMD1_MOUSE Protein-L-isoaspartate O-methyltransferase domain-containing protein 1 OS=Mus musculus GN=Pcmtd1 PE=2 SV=1	−1,06	0,670725474733774	0,900184189774275	25,2470629747378	24,8340128117331	24,9952475903761	25,0570831721089	24,9566019926909	24,8983311212619	73860286521,5801	69923812635,9964	0,0790150083529279	0,00831848821722769	0,0032160066771443	0,946703782628388	-0.0790150083529263	1,05629661394575	0.0790150083529264	1813
Q61735-2	sp|Q61735-2|CD47_MOUSE Isoform 2 of Leukocyte surface antigen CD47 OS=Mus musculus GN=Cd47;sp|Q61735|CD47_MOUSE Leukocyte surface antigen CD47 OS=Mus musculus GN=Cd47 PE=1 SV=2	−1,06	0,891605886802812	0,977064007773531	28,5757099628488	27,322661625401	28,2591635841223	27,9420598071018	27,8005133029123	28,2518043492866	1524231895660,02	1443549056329,73	0,0784622745103332	0,0232272844297031	0,0082437811208687	0,947066558861535	-0.0784622745103373	1,05589199686461	0.0784622745103371	1814
Q9CR67	sp|Q9CR67|TMM33_MOUSE Transmembrane protein 33 OS=Mus musculus GN=Tmem33 PE=2 SV=1	−1,06	0,750457526139066	0,928854093158237	27,6999254581679	27,7082701854407	27,6006069372341	27,2974019823772	27,6870999533187	27,8617602051029	1039333606038,73	984520653622,733	0,0781654289317949	0,00216468574413587	0,0104625035376008	0,947261445124527	-0.0781654289317939	1,05567476133111	0.0781654289317937	1815
O88935-1	sp|O88935-1|SYN1_MOUSE Isoform Ib of Synapsin-1 OS=Mus musculus GN=Syn1	−1,06	0,862859165722246	0,967731774645151	28,6809841596718	28,0327491345595	28,0971533682127	28,6463208267188	27,8317214951593	28,1703438982579	1895105364332,25	1795184180355,41	0,0781461940866278	0,0126323964797437	0,0145032344156243	0,947274074646479	-0.0781461940866313	1,05566068655811	0.0781461940866311	1816
P99028	sp|P99028|QCR6_MOUSE Cytochrome b-c1 complex subunit 6, mitochondrial OS=Mus musculus GN=Uqcrh PE=1 SV=2	−1,06	0,654274313778033	0,891901943526937	24,9925963663285	24,9662926902838	24,7568685258369	24,6613896974429	24,9428329797975	24,9493555858095	65495844019,0063	62049156482,7029	0,0779917666107177	0,00518668008996776	0,0066156429886801	0,947375477208856	-0.0779917666107195	1,05554769366227	0.0779917666107195	1817
Q61189	sp|Q61189|ICLN_MOUSE Methylosome subunit pICln OS=Mus musculus GN=Clns1a PE=2 SV=1	−1,06	0,890013166776299	0,976830327013275	24,3700164161251	25,2133112519035	25,6387500127636	24,9839800051415	25,2357243145929	24,8404515320987	77537373331,104	73463311157,2641	0,0778679398836762	0,0257530777141981	0,0079971880284109	0,947456794074741	-0.0778679398836764	1,05545709973674	0.0778679398836765	1818
Q8BJ71	sp|Q8BJ71|NUP93_MOUSE Nuclear pore complex protein Nup93 OS=Mus musculus GN=Nup93 PE=2 SV=1;sp|Q8BJ71-2|NUP93_MOUSE Isoform 2 of Nuclear pore complex protein Nup93 OS=Mus musculus GN=Nup93	−1,06	0,770224746338166	0,936218563920244	27,2584776827915	27,1060915962256	26,7839028148677	27,1464541510055	26,7568583343213	27,0832508388608	559042143402,334	529670582637,906	0,0778616597062383	0,00895756191771124	0,0077453568076979	0,947460918445839	-0.0778616597062402	1,05545250524987	0.0778616597062401	1819
Q9CPW2	sp|Q9CPW2|ADXL_MOUSE Adrenodoxin-like protein, mitochondrial OS=Mus musculus GN=Fdx1l PE=2 SV=1	−1,06	0,842055601312975	0,959771286845933	26,0680641034151	25,9217837339592	25,5795222243761	25,3839467459252	25,8555074920393	26,1684627487745	169557287709,289	160673307679,538	0,077642516885033	0,00969726576343201	0,015305452506254	0,947604847012044	-0.0776425168850355	1,05529219605953	0.0776425168850354	1820
Q8VDQ1	sp|Q8VDQ1|PTGR2_MOUSE Prostaglandin reductase 2 OS=Mus musculus GN=Ptgr2 PE=1 SV=2;sp|Q8VDQ1-2|PTGR2_MOUSE Isoform 2 of Prostaglandin reductase 2 OS=Mus musculus GN=Ptgr2	−1,06	0,693541633442681	0,910309912045398	28,7164765416841	28,2685915515089	28,5192469795579	28,3637482883521	28,4734319680636	28,5057267905654	2387906979957,6	2262826207701,41	0,0776208527792384	0,0078758460702017	0,0026160874448722	0,947619076745439	-0.077620852779233	1,05527634947416	0.0776208527792332	1821
P07091	sp|P07091|S10A4_MOUSE Protein S100-A4 OS=Mus musculus GN=S100a4 PE=1 SV=1	−1,06	0,89647051837634	0,977357990376388	21,4552101793533	21,3089099562841	20,3022376764705	21,2714023663618	20,5963942637238	21,0373734260528	1348311991,96612	1277779980,3279	0,0775149253964352	0,0298596236834483	0,0163460648850938	0,947688656588028	-0.0775149253964357	1,05519887048169	0.0775149253964355	1822
P62983	sp|P62983|RS27A_MOUSE Ubiquitin-40S ribosomal protein S27a OS=Mus musculus GN=Rps27a PE=1 SV=2	−1,05	0,882339743976512	0,973716798448825	33,5071865503134	33,1921566422038	32,5913113535174	32,5652501037116	33,2226868067924	33,3423047078514	236480022463196	224167324383554	0,0771423117524053	0,0140590590137616	0,01266209352157	0,947933453526468	-0.0771423117524074	1,05492637302739	0.0771423117524075	1823
Q9CQC6	sp|Q9CQC6|BZW1_MOUSE Basic leucine zipper and W2 domain-containing protein 1 OS=Mus musculus GN=Bzw1 PE=1 SV=1	−1,05	0,598348586741604	0,861677999617657	29,3445457208605	29,0635435904717	29,1565083052916	29,1249791637666	29,2410664809566	29,0383137035681	4745411207895,65	4498595934355,32	0,0770583183613379	0,00490460207661254	0,0034917556501844	0,94798864361224	-0.0770583183613359	1,05486495723153	0.0770583183613358	1824
Q921G8	sp|Q921G8|GCP2_MOUSE Gamma-tubulin complex component 2 OS=Mus musculus GN=Tubgcp2 PE=2 SV=2	−1,05	0,767653140454394	0,935597979611256	27,9743749343137	27,5544638957705	27,386252220826	27,572598641113	27,6694545667192	27,5128434547509	1007369590456,72	954988899877,233	0,0770372165391583	0,010959586534911	0,0028651326049966	0,948002509629322	-0.07703721653916	1,05484952818428	0.0770372165391602	1825
Q5EBJ4	sp|Q5EBJ4|ERMIN_MOUSE Ermin OS=Mus musculus GN=Ermn PE=1 SV=1	−1,05	0,738879017510544	0,925497610201997	27,0900402227433	26,6065658100871	26,8505074822295	26,6409567083219	26,8788895373272	26,8676217628967	457497765891,741	433788389140,974	0,0767732601827248	0,00900369533830425	0,0050095918827316	0,94817597260928	-0.0767732601827259	1,05465654993145	0.0767732601827257	1826
P14869	sp|P14869|RLA0_MOUSE 60S acidic ribosomal protein P0 OS=Mus musculus GN=Rplp0 PE=1 SV=3	−1,05	0,77587589740473	0,938619589104496	27,6006069372341	27,918409250953	28,2185163002376	27,7823931945621	27,964154597457	27,8317214951593	1325102365647,36	1256590347854,84	0,0765894102113609	0,0110701939084173	0,0033738605231492	0,948296811198394	-0.0765894102113599	1,05452215824312	0.07658941021136	1827
Q61206	sp|Q61206|PA1B2_MOUSE Platelet-activating factor acetylhydrolase IB subunit beta OS=Mus musculus GN=Pafah1b2 PE=1 SV=2	−1,05	0,739601505742932	0,925497610201997	30,507269860691	30,6767393272954	30,9037738640846	30,8190110775727	30,447826850692	30,6639825913764	21432459418122	20339925420097,3	0,0754830223807132	0,006481225575755	0,0060837846368113	0,949024329093052	-0.0754830223807155	1,05371376617464	0.0754830223807153	1828

Q8C167-2	sp|Q8C167-2|PPCEL_MOUSE Isoform 2 of Prolyl endopeptidase-like OS=Mus musculus GN=Prepl;sp|Q8C167|PPCEL_MOUSE Prolyl endopeptidase-like OS=Mus musculus GN=Prepl PE=2 SV=1;sp|Q8C167-3|PPCEL_MOUSE Isoform 3 of Prolyl endopeptidase-like OS=Mus musculus GN=	−1,05	0,827487677698233	0,955959931044103	28,9317927292942	29,6135756219551	29,4780244796227	29,5228299333287	29,1644059935391	29,1799288907371	5529568744068,21	5248980269061,33	0,0751297933295233	0,0123007554728382	0,0069173895651292	0,949256716392598	-0.0751297933295251	1,05345580677084	0.0751297933295251	1829
P62878	sp|P62878|RBX1_MOUSE E3 ubiquitin-protein ligase RBX1 OS=Mus musculus GN=Rbx1 PE=1 SV=1	−1,05	0,790214830727402	0,942055274591853	27,9254032200445	27,5261939943834	27,3724149190948	27,3519448291009	27,6110235759658	27,7048655608525	977245800656,417	927672553196,242	0,0751058226322144	0,010338566506959 6	0,0066335476360844	0,949272488633999	-0.0751058226322153	1,05343830351493	0.0751058226322153	1830
Q9CWG9	sp|Q9CWG9|BL1S2_MOUSE Biogenesis of lysosome-related organelles complex 1 subunit 2 OS=Mus musculus GN=Bloc1s2 PE=1 SV=1	−1,05	0,891375075529007	0,977046961561497	24,155861296033	23,8119599107002	23,4227004245546	23,1166209487376	23,9922099240447	24,1256842871187	21619046462,3914	20523540608,3943	0,0750232542054696	0,0154144078968759	0,0230841831190979	0,949326819020309	-0.0750232542054758	1,05337801478313	0.0750232542054757	1831
Q8BHL5-2	sp|Q8BHL5-2|ELMO2_MOUSE Isoform 2 of Engulfment and cell motility protein 2 OS=Mus musculus GN=Elmo2;sp|Q8BHL5|ELMO2_MOUSE Engulfment and cell motility protein 2 OS=Mus musculus GN=Elmo2 PE=1 SV=1;sp|Q8BHL5-3|ELMO2_MOUSE Isoform 3 of Engulfment and cell	−1,05	0,735747143876981	0,924701393764781	28,8887694965435	28,8830163528639	28,4314922090982	28,7146432611685	28,6266029003322	28,706063719518	3014412353559,32	2861699019125,52	0,0750048387322822	0,00913065551404072	0,0016924413067732	0,949338936906399	-0.0750048387322806	1,05336456888484	0.0750048387322807	1832
Q93092	sp|Q93092|TALDO_MOUSE Transaldolase OS=Mus musculus GN=Taldo1 PE=1 SV=2;sp|P37837|TALDO_HUMAN Transaldolase OS=Homo sapiens GN=TALDO1 PE=1 SV=2	−1,05	0,712307039408055	0,917434617372953	32,0567659011701	31,6424407003006	31,7691737448374	31,7608767264179	31,8925920404403	31,6602286177643	66139909076557	62816095952659,2	0,0743867805779551	0,00667141925987871	0,0036676997099272	0,949745725836265	-0.0743867805779539	1,05291339860412	0.0743867805779539	1833
Q8CEE7	sp|Q8CEE7|RDH13_MOUSE Retinol dehydrogenase 13 OS=Mus musculus GN=Rdh13 PE=2 SV=1	−1,05	0,817343872579259	0,952267950351507	22,6504567909222	23,0076169888695	23,4079437247829	23,0407523644372	22,9780575036453	22,8927270958949	9961622878,3014	9461647141,82607	0,0742894366111618	0,0164602687853514	0,0032346044170662	0,949809810852769	-0.0742894366111624	1,05284235704216	0.0742894366111624	1834
Q8BG05	sp|Q8BG05|ROA3_MOUSE Heterogeneous nuclear ribonucleoprotein A3 OS=Mus musculus GN=Hnrnpa3 PE=1 SV=1	−1,05	0,808073235306835	0,94879038498679	31,6049790076691	30,9037738640846	31,2730098935786	31,2551943052669	31,0862260035438	31,2859805099124	37696470618138,8	35805893054205,3	0,074232404958355	0,0112207610902448	0,0034460550465505	0,949847358839377	-0.0742324049583516	1,05280073760684	0.0742324049583516	1835
Q641P0	sp|Q641P0|ARP3B_MOUSE Actin-related protein 3B OS=Mus musculus GN=Actr3b PE=2 SV=1;sp|Q641P0-2|ARP3B_MOUSE Isoform 2 of Actin-related protein 3B OS=Mus musculus GN=Actr3b	−1,05	0,615132911421924	0,868726908818952	30,5244583269409	30,1999089850511	30,4196223089356	30,3537800086882	30,3742922372541	30,2626911460235	15647462413142,3	14868328521382,3	0,0736862402191013	0,00545165422408122	0,0019585596902299	0,950207013048607	-0.0736862402191041	1,05240225158057	0.073686240219104	1836
Q8R010	sp|Q8R010|AIMP2_MOUSE Aminoacyl tRNA synthase complex-interacting multifunctional protein 2 OS=Mus musculus GN=Aimp2 PE=1 SV=2	−1,05	0,725096081469715	0,921649709826669	27,6440499937839	27,2537191581988	27,6060239625494	27,5924349407401	27,4208032405863	27,3373962883802	878309058517,81	834594228592,64	0,0736537391197131	0,00782589135025145	0,0047372215894620	0,950228419596468	-0.0736537391197127	1,05237854328191	0.0736537391197125	1837
P20357	sp|P20357|MTAP2_MOUSE Microtubule-associated protein 2 OS=Mus musculus GN=Map2 PE=1 SV=2	−1,05	0,279904178616411	0,699221280539289	30,0036031524467	30,1173421092281	29,9945463914196	30,038498203068	29,9691108569161	29,9548618864297	11105895592431,3	10553622854897	0,0735874048938138	0,00227813238708442	0,0014921209743324	0,950272111516096	-0.0735874048938026	1,05233015667961	0.0735874048938026	1838
P26443	sp|P26443|DHE3_MOUSE Glutamate dehydrogenase 1, mitochondrial OS=Mus musculus GN=Glud1 PE=1 SV=1	−1,05	0,789571143128607	0,942055274591853	34,0061355969496	34,5060432263395	34,4527289384383	34,4527289384383	34,2755682575707	34,0837055790221	804816905244420	764824561190197	0,0735317553446821	0,00799870337273751	0,0053853775607817	0,950308767380977	-0.0735317553446822	1,05228956558611	0.0735317553446821	1839
Q9CQB5	sp|Q9CQB5|CISD2_MOUSE CDGSH iron-sulfur domain-containing protein 2 OS=Mus musculus GN=Cisd2 PE=1 SV=1;sp|Q8N5K1|CISD2_HUMAN CDGSH iron-sulfur domain-containing protein 2 OS=Homo sapiens GN=CISD2 PE=1 SV=1	−1,05	0,808817501673469	0,94879038498679	27,04657919394	26,8819369269204	26,6595208620373	26,5089478029815	26,8060735616088	27,1210844296353	463781324324,081	440878544313,844	0,073063457484568	0,00723109056793723	0,0114169571142409	0,950617287050927	-0.0730634574845685	1,05194804851727	0.0730634574845686	1840
Q8K1M6-3	sp|Q8K1M6-3|DNM1L_MOUSE Isoform 3 of Dynamin-1-like protein OS=Mus musculus GN=Dnm1l	−1,05	0,812019851459092	0,949948723398244	25,0006538060705	24,3040535920527	24,5615634697321	24,6958846864592	24,5047987626687	24,5138570468075	49344408293,575	46910787595,4118	0,0729668850403524	0,0143051361430493	0,0043873484310855	0,950680922472829	-0.072966885040354	1,05187763461045	0.072966885040354	1841
O88456	sp|O88456|CPNS1_MOUSE Calpain small subunit 1 OS=Mus musculus GN=Capns1 PE=2 SV=1	−1,05	0,812921401079202	0,950502380087197	27,9380967665236	28,2774605455253	28,520313058957	28,4508773233213	28,1083623316671	28,0252316570059	1848305008961,87	1757342376952,67	0,0728075572104143	0,0103535241117214	0,0080018525826649	0,950785919224289	-0.0728075572104143	1,05176147414537	0.0728075572104143	1842
Q99MR8	sp|Q99MR8|MCCA_MOUSE Methylcrotonoyl-CoA carboxylase subunit alpha, mitochondrial OS=Mus musculus GN=Mccc1 PE=1 SV=2	−1,05	0,473958009610033	0,799218469891531	28,9505536434768	29,0254710300426	28,7942855809832	28,9070949594495	28,8597896366355	28,8525957718364	3641572651914,28	3463012956117,19	0,0725338431295021	0,00407814991568047	0,0010254413341976	0,950966323381403	-0.0725338431295007	1,05156194852857	0.0725338431295006	1843

Q9CZ30	sp|Q9CZ30|OLA1_MOUSE Obg-like ATPase 1 OS=Mus musculus GN=Ola1 PE=1 SV=1;sp|Q9CZ30-2|OLA1_MOUSE Isoform 2 of Obg-like ATPase 1 OS=Mus musculus GN=Ola1	−1,05	0,646878103067534	0,889714023663571	31,5035229610434	31,1636297730172	31,1972620093183	31,2118339601665	31,2308454760787	31,271198233726	38749472076117,9	36853039836175,1	0,0723930297584043	0,00598581832068581	0,0009704455089304	0,951059146400304	-0.0723930297584054	1,05145931647357	0.0723930297584055	1844
P35486	sp|P35486|ODPA_MOUSE Pyruvate dehydrogenase E1 component subunit alpha, somatic form, mitochondrial OS=Mus musculus GN=Pdha1 PE=1 SV=1	−1,05	0,65967741893063	0,89341619202324	33,2226868067924	32,839500065196	33,0476252564011	32,9459102626137	33,0157551936444	32,9992079572545	222645968980563	211862332465877	0,0716243817830787	0,00580661884198067	0,0011064473654631	0,951565992575292	-0.0716243817830728	1,05089926269184	0.071624381783073	1845
P18760	sp|P18760|COF1_MOUSE Cofilin-1 OS=Mus musculus GN=Cfl1 PE=1 SV=3	−1,05	0,530595382941416	0,829615033817116	34,9927921828581	34,8802329166851	34,7771307544105	34,741959064496	34,8580740958575	34,9023917375126	1411437783903850	1343617008680070	0,0710435725777747	0,00309216742931259	0,0023784328079923	0,951949157095542	-0.071043572577775	1,05047627023597	0.071043572577775	1846
Q8BVL3	sp|Q8BVL3|SNX17_MOUSE Sorting nexin-17 OS=Mus musculus GN=Snx17 PE=1 SV=2	−1,05	0,814696528883253	0,950652201935115	26,3143352941035	26,868788726629	26,7270880304719	26,3327598269784	26,7397710425091	26,6900416088642	369988348075,684	352220824948,539	0,0709996265311767	0,0108142912509405	0,0083508737972203	0,951978154935002	-0.0709996265311753	1,0504442720834	0.0709996265311752	1847
Q91WG8	sp|Q91WG8|GLCNE_MOUSE Bifunctional UDP-N-acetylglucosamine 2-epimerase/N-acetylmannosamine kinase OS=Mus musculus GN=Gne PE=1 SV=1	−1,05	0,696286010576478	0,911315513842744	23,5022385635319	23,1698545542957	23,2759764901913	23,16404962761 4	23,4176157562769	23,220546028412	13366577378,7298	12732247738,2884	0,0701429652108753	0,00728135153745415	0,0057212682473868	0,952543600170167	-0.0701429652108731	1,04982071143132	0.0701429652108729	1848
Q91VC7	sp|Q91VC7|PP14A_MOUSE Protein phosphatase 1 regulatory subunit 14A OS=Mus musculus GN=Ppp1r14a PE=2 SV=1	−1,05	0,882541418402035	0,973716798448825	24,2472581965185	23,7113493878119	23,4404474775681	23,5382575829121	24,2064354818055	23,508629348876	21680628883,3746	20652608566,2832	0,0700825896684829	0,0172521200016532	0,0166141219606077	0,952583464131904	-0.0700825896684807	1,04977677825985	0.0700825896684808	1849
Q7TNM2	sp|Q7TNM2|TRI46_MOUSE Tripartite motif-containing protein 46 OS=Mus musculus GN=Trim46 PE=2 SV=1	−1,05	0,715038203604516	0,918558942968132	27,2953748650026	26,9293606494445	26,8777286255423	26,9675427036421	27,0220262352562	26,9675427036421	550534088136,836	524496127139,874	0,0698997757502105	0,00842236618784526	0,0011656572599593	0,952704180253248	-0.06989977575021	1,04964376217409	0.0698997757502099	1850
Q8BJH1	sp|Q8BJH1|ZC21A_MOUSE Zinc finger C2HC domain-containing protein 1A OS=Mus musculus GN=Zc2hc1a PE=2 SV=1;sp|Q8BJH1-2|ZC21A_MOUSE Isoform 2 of Zinc finger C2HC domain-containing protein 1A OS=Mus musculus GN=Zc2hc1a	−1,05	0,624671659713928	0,87511872328442	29,7557574407144	29,472404298724	29,6482708054861	29,472404298724	29,6135756219551	29,6461657797128	7348207449411,5	7003155156886,47	0,0693873050242078	0,00482844883729287	0,0031227263960121	0,953042657695699	-0.0693873050242069	1,04927097640922	0.0693873050242069	1851
Q9D0K2	sp|Q9D0K2|SCOT1_MOUSE Succinyl-CoA:3-ketoacid coenzyme A transferase 1, mitochondrial OS=Mus musculus GN=Oxct1 PE=1 SV=1	−1,05	0,841119114421123	0,959771286845933	32,8500916619242	33,6516402612364	33,3423047078514	33,2547258597017	33,3079535850214	33,1377845162753	284383712825216	271094338018049	0,0690438596786082	0,0121460348810668	0,0026193391301209	0,953269564296972	-0.0690438596786108	1,04902121860724	0.069043859678611	1852
Q62283	sp|Q62283|TSN7_MOUSE Tetraspanin-7 OS=Mus musculus GN=Tspan7 PE=2 SV=2	−1,05	0,815143945787942	0,950652201935115	26,7982568071736	26,6308893490547	26,9699393539991	26,4988608819045	27,0989232001278	26,6579118643942	435471491725,573	415147057076,171	0,0689558042036079	0,00632580250801416	0,0116207046423325	0,953327749265823	-0.0689558042036094	1,04895719312704	0.0689558042036094	1853
Q8BZM1	sp|Q8BZM1|GLMN_MOUSE Glomulin OS=Mus musculus GN=Glmn PE=2 SV=1	−1,05	0,843373622494478	0,959771286845933	25,109689615665	25,3393502864799	25,8742929382005	25,5614805926799	25,3115808292264	25,3070616187359	111926802645,925	106709320825,649	0,0688693559461713	0,0154210108927691	0,0057338505688007	0,953384875678248	-0.0688693559461682	1,04889434006239	0.0688693559461683	1854
Q9D819	sp|Q9D819|IPYR_MOUSE Inorganic pyrophosphatase OS=Mus musculus GN=Ppa1 PE=1 SV=1	−1,05	0,808569286060276	0,94879038498679	31,0706333494719	31,5082584125245	31,7107286381385	31,4825297714827	31,2915687270389	31,3724626453784	44649912640601,2	42570687604624,3	0,0687969598411535	0,0104094472235583	0,0030543187736882	0,953432718833895	-0.0687969598411548	1,04884170665242	0.0687969598411547	1855
Q9CQ71	sp|Q9CQ71|RFA3_MOUSE Replication protein A 14 kDa subunit OS=Mus musculus GN=Rpa3 PE=2 SV=1	−1,05	0,859568865361082	0,966502999979251	24,3120591899788	23,7519013740161	23,4328385 0516	23,8612171319506	23,8690116229415	23,6236389235436	22398646197,8488	21356512377,0386	0,0687354695259986	0,0186757544574821	0,0058638887082573	0,953473356755361	-0.0687354695259992	1,04879700404316	0.0687354695259993	1856
Q61233	sp|Q61233|PLSL_MOUSE Plastin-2 OS=Mus musculus GN=Lcp1 PE=1 SV=4	−1,05	0,770864986877754	0,936218563920244	27,570534038433	28,0287075649402	28,0469322356155	27,9171142157975	27,745330412391	27,840803271956	1285357423688,81	1225556284586,17	0,0687328477283465	0,00968154731292362	0,0030922092346416	0,953475089496104	-0.0687328477283496	1,04879509807486	0.0687328477283496	1857
Q8R366	sp|Q8R366|IGSF8_MOUSE Immunoglobulin superfamily member 8 OS=Mus musculus GN=Igsf8 PE=1 SV=2	−1,05	0,656933555453967	0,892226633215307	30,0239777147737	30,0107694072549	29,7220781608845	29,7939275700076	29,9327417629286	29,8880330606952	9854425513979,73	9398464723179,05	0,0683466625212235	0,005702632910320 1	0,0023720472862831	0,953730352910594	-0.0683466625212233	1,04851439083196	0.0683466625212232	1858
P62869	sp|P62869|ELOB_MOUSE Transcription elongation factor B polypeptide 2 OS=Mus musculus GN=Tceb2 PE=1 SV=1	−1,05	0,801572754513705	0,945787729921946	30,1381873875547	29,9008251465763	29,8014346095597	29,6015013481049	29,9490951305956	30,1478585778955	10132971365893,5	9664543728792,77	0,06828375996566 9	0,00577767596562875	0,0092488711999671	0,95377193715582 7	-0.0682837599656683	1,04846867583673	0.0682837599656681	1859
	sp|Q8BG67|EFR3A_MOUSE Protein EFR3 homolog A OS=Mus musculus GN=Efr3a PE=1 SV=1;sp|Q8BG67-2|EFR3A_MOUSE Isoform 2 of Protein																			

Q8BG67	[illegible] EFR3 homolog A OS=Mus musculus GN=Efr3a	−1,05	0,90196325988637	0,977644369603516	25,2084612346714	25,5447622122442	24,2886600925541	25,0836030464032	24,9575896134171	24,8599080669657	73017222857,5473	69669855755,8597	0,067702221900447	0,0259953530107996	0,004491767908496	0,95415647198445	-0.0677022219004475	1,04804613222421	0.067702221900447	1860
Q8R0A7	sp|Q8R0A7|K0513_MOUSE Uncharacterized protein KIAA0513 OS=Mus musculus GN=Kiaa0513 PE=2 SV=1	−1,05	0,845014178060987	0,960697384679244	30,0547925878358	30,6639825913764	30,5115424211656	30,6071786088968	30,4016042683483	30,0816756695027	16104274777272	15370730227144	0,067257987698501	0,0104244850474143	0,008721563135419	0,954450320782948	-0.0672579876984944	1,04772346786964	0.067257987698494	1861
P17918	sp|P17918|PCNA_MOUSE Proliferating cell nuclear antigen OS=Mus musculus GN=Pcna PE=1 SV=2	−1,05	0,885796781319417	0,975149870380105	25,7112604959504	26,4356893840667	26,7355071353139	26,4412629368967	26,0637890078749	26,238233207829	262666850614,176	250759893221,742	0,066927518730857	0,0200268075321778	0,007197462218632	0,95466897568310 3	-0.0669275187308582	1,04748350001053	0.066927518730858	1862
P61971	sp|P61971|NTF2_MOUSE Nuclear transport factor 2 OS=Mus musculus GN=Nutf2 PE=2 SV=1	−1,05	0,710923732942732	0,916805961322165	28,3308483775731	28,0545669515707	27,9366295152886	28,0469322356155	27,9932539266564	28,1431261777461	1610149715310,26	1537385117237,37	0,066716232042911	0,0071988339492341 2	0,002706042520079	0,954808799839542	-0.0667162320429082	1,04733010438116	0.066716232042908	1863
Q9DBZ5	sp|Q9DBZ5|EIF3K_MOUSE Eukaryotic translation initiation factor 3 subunit K OS=Mus musculus GN=Eif3k PE=1 SV=1;sp|Q9DBZ5-2|EIF3K_MOUSE Isoform 2 of Eukaryotic translation initiation factor 3 subunit K OS=Mus musculus GN=Eif3k	−1,05	0,800846960511086	0,945787729921946	28,5057267905654	28,1351226080958	28,2841998398392	28,046173766494	28,197075208073	28,5437339219033	196861181 1685,19	1880065311180,78	0,0663958756535	0,0065874072182833 6	0,009026768715273	0,955020842616701	-0.0663958756535012	1,04709756622699	0.066395875653501	1864
Q9DCV4	sp|Q9DCV4|RMD1_MOUSE Regulator of microtubule dynamics protein 1 OS=Mus musculus GN=Rmdn1 PE=1 SV=2	−1,05	0,606578769085715	0,865805356251592	27,1948465255442	27,04657919394	27,2944083728934	27,232167200089	27,0553730818285	27,1109039589626	636093475870,457	607619528147,63	0,066070552474690	0,00458852384277195	0,003332154866864	0,955236221085491	-0.0660705524746915	1,04686147565012	0.066070552474691	1865
P62898	sp|P62898|CYC_RAT Cytochrome c, somatic OS=Rattus norvegicus GN=Cycs PE=1 SV=2;sp|P62897|CYC_MOUSE Cytochrome c, somatic OS=Mus musculus GN=Cycs PE=1 SV=2	−1,05	0,775522492006574	0,938619589104496	32,9187789512132	33,0839850192138	32,799089045904	32,6339988306786	32,9255457365668	33,1052341468871	20092463540511 1	191950678352906	0,065918805338421	0,0043436237183709 3	0,007231153285256	0,955336701076444	-0.0659188053384171	1,04675136930595	0.065918805338417	1866
Q99LB7	sp|Q99LB7|SARDH_MOUSE Sarcosine dehydrogenase, mitochondrial OS=Mus musculus GN=Sardh PE=1 SV=1	−1,05	0,807851059723302	0,94879038498679	27,173042131448	26,7001686947059	26,6864288116258	26,6767161700113	27,0003491513743	26,7455391951909	459411983785,297	438898861151,772	0,065899963262430	0,0103177653628267	0,006359815366086	0,955349178172262	-0.0658999632624306	1,04673769847498	0.065899963262430	1867
Q99KL7	sp|Q99KL7|RAB28_MOUSE Ras-related protein Rab-28 OS=Mus musculus GN=Rab28 PE=2 SV=1	−1,05	0,86897967238077	0,970240308853386	24,7251231135384	25,3600660664926	25,5890196137344	25,0849820401459	25,0773874416878	25,3750798339753	90149592341,9677	86132252517,4801	0,065767406880755	0,0177431204554938	0,006740612666730	0,95543696072137	-0.0657674068807548	1,04664152750066	0.065767406880754	1868
P54227	sp|P54227|STMN1_MOUSE Stathmin OS=Mus musculus GN=Stmn1 PE=1 SV=2	−1,05	0,832144342037963	0,957953242267243	29,9691108569161	30,3453333642794	30,2310954123744	29,8358818720028	30,1038443023282	30,4692900893302	12817679496489,1	12247448610756,5	0,065653862910894	0,0063907885863231 9	0,010550448768922 1	0,95551215913233	-0.065653862910898	1,04655915724617	0.065653862910898	1869
Q9JHS3	sp|Q9JHS3|LTOR2_MOUSE Ragulator complex protein LAMTOR2 OS=Mus musculus GN=Lamtor2 PE=1 SV=1	−1,05	0,720672724259409	0,920750207284069	25,4779842302653	25,3877731654233	25,2204546504586	25,1125314478412	25,4294533770172	25,4079277565894	103420669970,851	98827083353,7728	0,065546187265897	0,0051523606898421 2	0,006994942159751	0,955583476510325	-0.0655461872659002	1,04648105014528	0.065546187265900	1870
Q9JM76	sp|Q9JM76|ARPC3_MOUSE Actin-related protein 2/3 complex subunit 3 OS=Mus musculus GN=Arpc3 PE=1 SV=3	−1,05	0,790194803534203	0,942055274591853	31,4546926223212	30,9631543867588	31,1056636057856	30,9950183754577	31,2898972692622	31,1032923983988	3458715679848 1,6	33061900102664,3	0,065066783092886	0,0081122877461892 6	0,004791718523962	0,955901067419214	-0.0650667830928902	1,04613336472136	0.065066783092890	1871
Q3THS6	sp|Q3THS6|METK2_MOUSE S-adenosylmethionine synthase isoform type-2 OS=Mus musculus GN=Mat2a PE=2 SV=2	−1,05	0,61953521844251	0,870879639674993	29,9670200277696	29,6593636472282	29,7799328650923	29,7550162373898	29,7621840572139	29,7557574407144	8767786652861,17	8386568705492,95	0,064132028767864	0,0052017142431717 1	0,000132464612939	0,956520617749769	-0.0641320287678648	1,04545577109725	0.064132028767864	1872
Q9Z1Q2	sp|Q9Z1Q2|ABHGA_MOUSE Abhydrolase domain-containing protein 16A OS=Mus musculus GN=Abhd16a PE=1 SV=3;sp|O95870|ABHGA_HUMAN Abhydrolase domain-containing protein 16A OS=Homo sapiens GN=ABHD16A PE=1 SV=3;sp|O95870-2|ABHGA_HUMAN Isoform 2 of Abhydrolase do	−1,05	0,874708493607963	0,972636416581711	27,3567816774972	28,1061634244686	27,8005133029123	27,5987969361743	28,0596330923216	27,4718930142898	1131410426016,58	1082298007142,37	0,064024575552490	0,013576369400959 9	0,011161156048545 4	0,95659186291298 1	-0.0640245755525006	1,04537790751725	0.064024575552500	1873
Q9WTX5	sp|Q9WTX5|SKP1_MOUSE S-phase kinase-associated protein 1 OS=Mus musculus GN=Skp1 PE=1 SV=3;sp|P63208|SKP1_HUMAN S-phase kinase-associated protein 1 OS=Homo sapiens GN=SKP1 PE=1 SV=2;sp|P63208-2|SKP1_HUMAN Isoform 2 of S-phase kinase-associated protein 1	−1,05	0,723072983039966	0,921631098094813	31,0040688696121	30,6260640465842	30,9568503354048	30,7513155117469	30,8856809268552	30,8170158313743	2531270983487 3,2	24215259058502,2	0,06394552525737 2	0,0066737662302108 1	0,002180159319333	0,956644279354909	-0.0639455252573706	1,0453206291834 3	0.063945525257370	1874
Q8VD04	sp|Q8VD04|GRAP1_MOUSE GRIP1-associated protein 1 OS=Mus musculus GN=Gripap1 PE=1 SV=1	−1,05	0,834799213092677	0,957953242267243	29,7991526231768	29,1173946133026	29,3328195161808	29,3362258860121	29,5039457329552	29,2762930224565	5962169758655,22	5703806531780,94	0,063912405601476	0,0118467442130943	0,004017498184921	0,956666241094659	-0.0639124056014767	1,04529663224633	0.063912405601476	1875
Q5SV85-2	sp|Q5SV85-2|SYNRG_MOUSE Isoform 2 of Synergin gamma OS=Mus musculus GN=Synrg;sp|Q5SV85|SYNRG_MOUSE Synergin gamma OS=Mus musculus GN=Synrg PE=1 SV=1	−1,05	0,710268559292413	0,916805961322165	27,6376773679146	27,2900373208184	27,3151166099119	27,4505481052565	27,3163381242936	27,343085730949	805136044567,003	770257455344,779	0,063891836092868	0,0070720907660603	0,002595364540939	0,956679881049194	-0.0638918360928677	1,04528172883002	0.063891836092867	1876
Q9CX86	sp|Q9CX86|ROA0_MOUSE Heterogeneous nuclear ribonucleoprotein A0 OS=Mus musculus GN=Hnrnpa0 PE=1 SV=1	−1,05	0,562904175925008	0,845110794140252	28,5948343218379	28,5963004492755	28,7923191655361	28,6943108318933	28,5819748597617	28,5750404298675	2801430546814,68	2680725833489,53	0,063540047882403	0,0039634506382028 7	0,002339469162692	0,956913187277695	-0.0635400478824014	1,04502687735434	0.063540047882401	1877

Q7TMK9-2	sp|Q7TMK9-2|HNRPQ_MOUSE Isoform 2 of Heterogeneous nuclear ribonucleoprotein Q OS=Mus musculus GN=Syncrip;sp|O60506-3|HNRPQ_HUMAN Isoform 3 of Heterogeneous nuclear ribonucleoprotein Q OS=Homo sapiens GN=SYNCRIP;sp|Q7TMK9|HNRPQ_MOUSE Heterogeneous nucle	−1,04	0,784428356154904	0,940443821602512	29,3758840404762	29,7557574407144	29,7910678192371	29,7678736961036	29,469468974537	29,5542026275958	7462436610027,89	7143198535139,65	0,0630765518348586	0,00776600139386928	0,0051955958730481	0,95722066510297	-0.0630765518348576	1,04469119447231	0.0630765518348574	1878
Q9Z1G3	sp|Q9Z1G3|VATC1_MOUSE V-type proton ATPase subunit C 1 OS=Mus musculus GN=Atp6v1c1 PE=1 SV=4	−1,04	0,516225628282775	0,820430016377981	33,268184717111	33,0476252564011	33,1052341468871	33,0753864301016	33,1187251555991	33,0960166649535	246984831597175	236438539987512	0,0629572255351593	0,00345185333091123	0,00065497949973200	0,95729984087904	-0.0629572255351924	1,04460479078504	0.0629572255351925	1879
Q6P8M1-2	sp|Q6P8M1-2|TATD1_MOUSE Isoform 2 of Putative deoxyribonuclease TATDN1 OS=Mus musculus GN=Tatdn1;sp|Q6P8M1|TATD1_MOUSE Putative deoxyribonuclease TATDN1 OS=Mus musculus GN=Tatdn1 PE=1 SV=1	−1,04	0,828670383592118	0,956839193875508	23,9753136992594	24,2294265771732	24,5876185868543	24,3086666205643	24,3139660285988	24,040363892071	34496359065,9961	33040464594,5271	0,0622101268345034	0,0126781103291106	0,0064595560123054	0,957795706245877	-0.0622101268345017	1,04406398303825	0.0622101268345016	1880
O08547	sp|O08547|SC22B_MOUSE Vesicle-trafficking protein SEC22b OS=Mus musculus GN=Sec22b PE=1 SV=3	−1,04	0,925351483065359	0,983416151166861	30,1058851287532	30,6783315069509	30,8138966768348	30,9542765997972	30,8315955900058	29,6831401315376	18204764063946,5	17437963813502,1	0,0620844532585934	0,0123081085681989	0,0229967825314878	0,957879143736721	-0.0620844532585901	1,04397303828849	0.06208445325859	1881
Q3TDQ1	sp|Q3TDQ1|STT3B_MOUSE Dolichyl-diphosphooligosaccharide--protein glycosyltransferase subunit STT3B OS=Mus musculus GN=Stt3b PE=1 SV=2;sp|Q8TCJ2|STT3B_HUMAN Dolichyl-diphosphooligosaccharide--protein glycosyltransferase subunit STT3B OS=Homo sapiens GN=ST	−1,04	0,916958442729557	0,980665300581806	23,3919312518076	23,2126920867356	23,2631031706819	22,4425016638024	23,5235637626522	23,7728608943411	13013357537,0952	12466473695,3777	0,0619397977041797	0,0039689710603547	0,0304215727103833	0,957975192784907	-0.0619397977041719	1,04386836687589	0.061939797704172	1882
Q9CYN9	sp|Q9CYN9|RENR_MOUSE Renin receptor OS=Mus musculus GN=Atp6ap2 PE=2 SV=2	−1,04	0,508883430482899	0,816124555116335	28,470179165499	28,3845625088349	28,299685761744	28,2685915515089	28,415569281999	28,3414907470162	2125034482770,28	2035746830097,55	0,0619280960645538	0,00300326026606928	0,0025929699727026	0,957982962913462	-0.0619280960645509	1,04385990013721	0.0619280960645507	1883
Q505F5	sp|Q505F5|LRC47_MOUSE Leucine-rich repeat-containing protein 47 OS=Mus musculus GN=Lrrc47 PE=2 SV=1	−1,04	0,822047763899251	0,954216651326321	28,6073269178441	29,0211872608148	29,0789957161334	29,0435033047516	28,6378238317448	28,8979126911188	3566134308411,29	3416871933729,86	0,0616848632702619	0,00890093008868934	0,0071211669944585	0,958144488745314	-0.0616848632702625	1,04368392423725	0.0616848632702625	1884
A2AG50-2	sp|A2AG50-2|MA7D2_MOUSE Isoform 2 of MAP7 domain-containing protein 2 OS=Mus musculus GN=Map7d2;sp|A2AG50|MA7D2_MOUSE MAP7 domain-containing protein 2 OS=Mus musculus GN=Map7d2 PE=1 SV=1;sp|A2AG50-3|MA7D2_MOUSE Isoform 3 of MAP7 domain-containing protei	−1,04	0,69208575431965	0,909753037606976	27,5736087892473	27,2789110284764	27,3623338424334	27,2725327823275	27,4832478791861	27,3309114068393	797662346408,582	764303416624,087	0,0616326976428008	0,00554282564347285	0,0039760230061256	0,958179134398544	-0.0616326976428022	1,04364618691859	0.061632697642802	1885
Q80YA9-2	sp|Q80YA9-2|CNKR2_MOUSE Isoform 2 of Connector enhancer of kinase suppressor of ras 2 OS=Mus musculus GN=Cnksr2;sp|Q80YA9|CNKR2_MOUSE Connector enhancer of kinase suppressor of ras 2 OS=Mus musculus GN=Cnksr2 PE=1 SV=1	−1,04	0,845326855887251	0,960697384679244	26,0184741068914	25,8417503358081	25,5121220870885	25,8448215763115	25,9613397477305	25,4392340632725	158779482910,377	152200585473,562	0,0610505945606058	0,00996460492384264	0,0106439627626008	0,958565821501456	-0.0610505945605969	1,04322517824978	0.0610505945605597	1886
Q80ZU0	sp|Q80ZU0|ARL5A_MOUSE ADP-ribosylation factor-like protein 5A OS=Mus musculus GN=Arl5a PE=2 SV=1;sp|Q9D4P0|ARL5B_MOUSE ADP-ribosylation factor-like protein 5B OS=Mus musculus GN=Arl5b PE=2 SV=3;sp|Q6P068|ARL5C_MOUSE ADP-ribosylation factor-like protein	−1,04	0,821126815263253	0,9536454688891	24,2514976759772	24,1418352095624	23,8813417836964	24,0503294468235	23,7897040107311	24,3086666205643	29028914533,3126	27835184661,4653	0,0605809726275735	0,00789200449464138	0,0107894728014814	0,958877901876854	-0.0605809726275712	1,04288564586029	0.0605809726275711	1887
Q8BZW8	sp|Q8BZW8|NHLC2_MOUSE NHL repeat-containing protein 2 OS=Mus musculus GN=Nhlrc2 PE=2 SV=1	−1,04	0,7741084003387	0,938569590855925	25,6281918452435	25,6663122517335	25,2300086282913	25,4860362621515	25,3867743256002	25,526381389398	119690000549,503	114793112767,365	0,0602665406103571	0,00947337496324149	0,0028211945171818	0,95908690985332	-0.060266540610359	1,042658376135	0.0602665406103589	1888
Q9Z2H5	sp|Q9Z2H5|E41L1_MOUSE Band 4.1-like protein 1 OS=Mus musculus GN=Epb41l1 PE=1 SV=2	−1,04	0,907433370606504	0,980398950801751	30,8665321302372	29,748150804332	30,3642246304452	30,3323426792313	30,5147582056784	30,0077005409481	14809685697565,1	14209527533621	0,0596824371683223	0,0184709042719685	0,0084805135797045	0,959475293655775	-0.0596824371683209	1,04223632084347	0.0596824371683208	1889
P28656	sp|P28656|NP1L1_MOUSE Nucleosome assembly protein 1-like 1 OS=Mus musculus GN=Nap1l1 PE=1 SV=2	−1,04	0,56481876406331	0,845110794140252	30,4253950011429	30,5474125413229	30,6563201848299	30,5018948562202	30,5474125413229	30,4559240288616	18393943629028,1	17649768954076,4	0,0595815262933117	0,00378235393348299	0,0014997320661837	0,959542407546727	-0.0595815262933144	1,04216342303902	0.0595815262933144	1890
Q9CW07	sp|Q9CW07|PP13G_MOUSE Protein phosphatase 1 regulatory subunit 3G OS=Mus musculus GN=Ppp1r3g PE=2 SV=2	−1,04	0,839349352150245	0,959771286845933	23,0974215930066	22,4148399246451	22,6937862021931	22,7520392588109	22,6026322326214	22,7276309950859	7478882653,85747	7176666445,51634	0,0595088781513479	0,015094718393838	0,0035316988545891	0,959590727341436	-0.0595088781513467	1,04211094533033	0.0595088781513467	1891
P62627	sp|P62627|DLRB1_MOUSE Dynein light chain roadblock-type 1 OS=Mus musculus GN=Dynlrb1 PE=1 SV=3	−1,04	0,81495363270665	0,950652201935115	29,4161611861033	29,4599545956542	29,2631523510523	29,0458241396507	29,3536338319982	29,6166517537297	5747327294163,89	5516160881633,94	0,0592266745483175	0,00351699697624365	0,0097382133216907	0,959778449930164	-0.0592266745483186	1,04190711936985	0.0592266745483187	1892
Q8BZ36-2	sp|Q8BZ36-2|RINT1_MOUSE Isoform 2 of RAD50-interacting protein 1 OS=Mus musculus GN=Rint1;sp|Q8BZ36|RINT1_MOUSE RAD50-interacting protein 1 OS=Mus musculus GN=Rint1 PE=2 SV=2	−1,04	0,936824198846901	0,98548775757167	23,5370595761934	22,2942056201138	23,5928619631574	22,7383944477728	22,8884318811075	23,675109750947	11224623399,1131	10776625930,1549	0,0587614882111936	0,0317267728684488	0,0217796964763518	0,96008797328616	-0.0587614882111824	1,04157121828871	0.0587614882111825	1893
Q9Z2B9	sp|Q9Z2B9|KS6A4_MOUSE Ribosomal protein S6 kinase alpha-4 OS=Mus musculus GN=Rps6ka4 PE=1 SV=2	−1,04	0,920668891221426	0,981888180745783	24,7055174223122	24,1341293512829	24,4060266307704	23,6519620919524	24,9004151407677	24,5712773674685	40123356903,8673	38524164603,8179	0,0586786412270323	0,0117060181020985	0,0265464731438058	0,960143108068426	-0.0586786412270346	1,04151140761897	0.0586786412270345	1894

Q8BH15-2	sp|Q8BH15-2|CNO10_MOUSE Isoform 2 of CCR4-NOT transcription complex subunit 10 OS=Mus musculus GN=Cnot10;sp|Q8BH15|CNO10_MOUSE CCR4-NOT transcription complex subunit 10 OS=Mus musculus GN=Cnot10 PE=2 SV=1;sp|Q8BH15-3|CNO10_MOUSE Isoform 3 of CCR4-NOT tr	−1,04	0,832977524826938	0,957953242267243	26,1582354818588	25,6757304025179	25,6347612167554	25,956784137851	25,663480608275	25,7281474875069	163963394192,101	157517764271,554	0,057859220895413	0,0112738191683408	0,005976906868241	0,960688603988063	-0.0578592208954127	1,04092001908709	0.057859220895412	1895
P62743	sp|P62743|AP2S1_MOUSE AP-2 complex subunit sigma OS=Mus musculus GN=Ap2s1 PE=1 SV=1	−1,04	0,736670041725376	0,924701393764781	30,5924988627797	30,5115424211656	30,348015515816	30,2860766360239	30,4337492602583	30,6125340776466	17339685212026,3	16661470120641,1	0,057562005679606	0,00408554732478753	0,0053696944807895	0,960886539571387	-0.0575620056796103	1,04070559719367	0.057562005679610	1896
Q8BHA3-2	sp|Q8BHA3-2|DTD2_MOUSE Isoform 2 of Probable D-tyrosyl-tRNA(Tyr) deacylase 2 OS=Mus musculus GN=Dtd2;sp|Q8BHA3|DTD2_MOUSE Probable D-tyrosyl-tRNA(Tyr) deacylase 2 OS=Mus musculus GN=Dtd2 PE=2 SV=1	−1,04	0,915941644551252	0,980665300581806	24,6602962520587	23,620865820898	24,4774642448869	23,8225765445107	24,3302859701029	24,4861190808891	34110648297,5543	32777032066,1688	0,057536949196614	0,0228805040855046	0,0143300323633147	0,960903228230901	-0.0575369491966132	1,04068752255217	0.057536949196613	1897
P29595	sp|P29595|NEDD8_MOUSE NEDD8 OS=Mus musculus GN=Nedd8 PE=1 SV=2	−1,04	0,735216610863325	0,924701393764781	28,8498970986966	28,5437339219033	28,7546109971162	28,6073269178441	28,5948343218379	28,8266770279669	2959616078290,33	2844133087184,12	0,057421066028446	0,00545604184393198	0,004547236954296	0,960980415009463	-0.0574210660284462	1,04060393362975	0.05742106602844	1898
O35684	sp|O35684|NEUS_MOUSE Neuroserpin OS=Mus musculus GN=Serpini1 PE=1 SV=1	−1,04	0,816187280980218	0,951380089463074	25,5763604830779	25,114611226522	25,1735426829318	25,3600660664926	25,0688342117586	25,3163007018966	96053528751,2802	92308339521,1891	0,057377622785922	0,00993794192971621	0,006219835599393	0,961009353026594	-0.0573776227859217	1,04057259885204	0.057377622785921	1899
Q91VH6	sp|Q91VH6|MEMO1_MOUSE Protein MEMO1 OS=Mus musculus GN=Memo1 PE=1 SV=1	−1,04	0,860332099659828	0,966595891177217	26,0883849331964	26,4907634434962	26,5801731397537	26,6649401997547	26,0995196682378	26,275921090209	288061692865,844	276864388965,654	0,057198317846758	0,00992806688753885	0,010980318589122	0,961128799220781	-0.0571983178467558	1,04044327962156	0.057198317846755	1900
Q9H0R8	sp|Q9H0R8|GBRL1_HUMAN Gamma-aminobutyric acid receptor-associated protein-like 1 OS=Homo sapiens GN=GABARAPL1 PE=1 SV=1;sp|Q8R3R8|GBRL1_MOUSE Gamma-aminobutyric acid receptor-associated protein-like 1 OS=Mus musculus GN=Gabarapl1 PE=1 SV=2;sp|Q9H0R8-2|G	−1,04	0,793081342448932	0,942848667725432	27,1916972274608	27,3399629165513	27,6361586212546	27,4758239742999	27,3594544203214	27,2136596408025	785253884162,681	754745174351,181	0,057169546467192	0,0082622297319305 9	0,004802881228497	0,961147967011928	-0.0571695464671919	1,04042253047557	0.057169546467192	1901
P45591	sp|P45591|COF2_MOUSE Cofilin-2 OS=Mus musculus GN=Cfl2 PE=1 SV=1	−1,04	0,824663560531566	0,95563531252039	30,60874741464	30,0523063560686	30,343830029212	30,1496750736804	30,3862745237618	30,3514194051073	14938475764603,9	14364624491694,3	0,056512671799439	0,00917510729129431	0,004216210174024	0,961585687726634	-0.0565126717994457	1,03994892266355	0.056512671799445	1902
P63330	sp|P63330|PP2AA_MOUSE Serine/threonine-protein phosphatase 2A catalytic subunit alpha isoform OS=Mus musculus GN=Ppp2ca PE=1 SV=1	−1,04	0,813510178405144	0,950652201935115	28,4530620211488	28,3325463587444	28,1946741447067	28,5819748597617	28,1677723504312	28,1132288221562	2005191817191,63	1928297639333,7	0,056412498211407	0,00456427260810359	0,009061906802083	0,961652457785498	-0.0564124982114103	1,03987671627524	0.056412498211410	1903
Q8BLQ9-3	sp|Q8BLQ9-3|CADM2_MOUSE Isoform 3 of Cell adhesion molecule 2 OS=Mus musculus GN=Cadm2;sp|Q8BLQ9-2|CADM2_MOUSE Isoform 2 of Cell adhesion molecule 2 OS=Mus musculus GN=Cadm2;sp|Q8BLQ9|CADM2_MOUSE Cell adhesion molecule 2 OS=Mus musculus GN=Cadm2 PE=1 SV	−1,04	0,841852133629715	0,959771286845933	30,2552727654907	30,82620630466	30,8190110775727	30,6742077219904	30,5173788922969	30,5924988627797	20135322276861,3	19369003742743,6	0,055978813697382	0,010693239128024	0,002563760790304	0,961941580890497	-0.0559788136973779	1,0395641688285	0.055978813697377	1904
P62761	sp|P62761|VISL1_MOUSE Visinin-like protein 1 OS=Mus musculus GN=Vsnl1 PE=1 SV=2	−1,04	0,825892747560344	0,955744170441451	33,4570130838302	33,2755034398491	33,1779674945783	32,9567183360255	33,3807460089899	33,4570130838302	290752839311244	279724361106833	0,055787377085052	0,00425219642618131	0,008102854114431	0,962069232993439	-0.0557873770850532	1,03942623431428	0.055787377085053	1905
Q91VR5	sp|Q91VR5|DDX1_MOUSE ATP-dependent RNA helicase DDX1 OS=Mus musculus GN=Ddx1 PE=1 SV=1	−1,04	0,824029028277636	0,95563531252039	31,2250526281598	31,0273688047328	30,6639825913764	30,8215053344581	30,9912486313499	30,9878465239167	28250567865605,2	27180841829913,7	0,05568972833478	0,0091883854861702 8	0,003136859409236	0,962134352810873	-0.0556897283347806	1,0393558831763	0.055689728334780	1906
Q8VDM4	sp|Q8VDM4|PSMD2_MOUSE 26S proteasome non-ATPase regulatory subunit 2 OS=Mus musculus GN=Psmd2 PE=1 SV=1	−1,04	0,850379867652423	0,96244931500623	31,6726545295256	31,5283219649286	31,0631349310244	31,2604931538048	31,5568375533821	31,3310729947621	44271864359052,5	42596841956653,9	0,055643652976207	0,0101371753165029	0,00493251649788	0,962165080991081	-0.0556436529762092	1,03932268979243	0.055643652976209	1907
Q9D4H2	sp|Q9D4H2|GCC1_MOUSE GRIP and coiled-coil domain-containing protein 1 OS=Mus musculus GN=Gcc1 PE=1 SV=2	−1,04	0,820266129431243	0,95319712275689	22,5567734350707	22,252583800289	22,4425016638024	22,6586817635727	22,2997639006898	22,1779631888277	5441313384,89145	5235891438,08994	0,055519736312787	0,00685428478280049	0,0111675283828841	0,962247727291008	-0.0555197363127856	1,03923342361668	0.055519736312785	1908

P47857	sp|P47857|PFKAM_MOUSE ATP-dependent 6-phosphofructokinase, muscle type OS=Mus musculus GN=Pfkm PE=1 SV=3;sp|P47857-2|PFKAM_MOUSE Isoform 2 of ATP-dependent 6-phosphofructokinase, muscle type OS=Mus musculus GN=Pfkm;sp|P47857-3|PFKAM_MOUSE Isoform 3 of A	−1,04	0,750197647605726	0,928854093158237	33,404240792523	33,2226868067924	33,0286081008433	33,2755034398491	33,0960166649535	33,1690884811052	267064655395380	257027156349062	0,0552682592644007	0,00565501381153382	0,002720239224943	0,962415471895905	-0.0552682592643999	1,03905228999494	0.0552682592643998	1909
Q8C996	sp|Q8C996|TM163_MOUSE Transmembrane protein 163 OS=Mus musculus GN=Tmem163 PE=1 SV=1	−1,04	0,84806345331141	0,961859074108069	26,8109304958059	27,3198293289793	27,3782962363329	27,3001174003902	27,0782792312648	27,0158477199576	630440963598,202	606769478393,945	0,0552127613132	0,0114857091613292	0,0055064693252486	0,962452495045446	-0.0552127613132005	1,03901232024213	0.0552127613132003	1910
Q9DBC7	sp|Q9DBC7|KAP0_MOUSE cAMP-dependent protein kinase type I-alpha regulatory subunit OS=Mus musculus GN=Prkar1a PE=1 SV=3	−1,04	0,782703730957616	0,940251150142017	30,076831063814	29,6912193119391	29,7678736961036	29,853115187151	29,9008251465763	29,6681665776184	9154878513014,73	8814046408372,81	0,0547362484904816	0,0068397738228853	0,0041227952302590	0,962770439372037	-0.0547362484904818	1,03866919787467	0.0547362484904819	1911
P63094	sp|P63094|GNAS2_MOUSE Guanine nucleotide-binding protein G(s) subunit alpha isoforms short OS=Mus musculus GN=Gnas PE=1 SV=1;sp|Q6R0H7|GNAS1_MOUSE Guanine nucleotide-binding protein G(s) subunit alpha isoforms XLas OS=Mus musculus GN=Gnas PE=2 SV=1;sp|P	−1,04	0,649203094596552	0,889714023663571	29,4282378281537	29,2163908422627	29,1607446221874	29,2199061542331	29,21883644974	29,2543000086044	5141971921820,52	4952998190460,57	0,0540196383378573	0,00482236234127348	0,00069012965300474	0,96324878193947	-0.0540196383378575	1,03815340205937	0.0540196383378576	1912
Q8BGR9	sp|Q8BGR9|UBCP1_MOUSE Ubiquitin-like domain-containing CTD phosphatase 1 OS=Mus musculus GN=Ublcp1 PE=1 SV=1	−1,04	0,767382456665795	0,935597979611256	26,868788726629	26,446728601505	26,6783050470106	26,6924412252218	26,5858634061522	26,6033375672616	380445000468,09	366481568423,04	0,0539472614451413	0,00792673236566444	0,0021466784713722	0,963297107261576	-0.0539472614451429	1,0381013214529	0.0539472614451428	1913
P62342	sp|P62342|SELT_MOUSE Selenoprotein T OS=Mus musculus GN=Selt PE=2 SV=2	−1,04	0,860263800410665	0,966595891177217	25,5419778283143	25,2882777697561	25,1499790121567	24,9361022925742	25,4532890416025	25,4794674273275	99831027633,4426	96192726838,2665	0,0535604615424393	0,0078498185879822	0,0121170124102412	0,963555410763324	-0.0535604615424393	1,03782303418109	0.0535604615424393	1914
Q8K0D7	sp|Q8K0D7|WRB_MOUSE Tail-anchored protein insertion receptor WRB OS=Mus musculus GN=Wrb PE=2 SV=1	−1,04	0,809883196903305	0,94879038498679	25,0061922723973	24,8090721097822	25,1843097620353	25,0392995070299	24,7499941271413	25,0998804841175	71994678828,4979	69393430557,8988	0,0530911899725207	0,00750800274976942	0,0074906073830103	0,963868881521152	-0.0530911899725208	1,03748551195244	0.0530911899725207	1915
Q99LT0	sp|Q99LT0|DPY30_MOUSE Protein dpy-30 homolog OS=Mus musculus GN=Dpy30 PE=1 SV=1	−1,04	0,899517399352833	0,977644369603516	26,4201728012214	27,1138793678718	27,1958332984449	27,0871885833272	26,9530356848158	26,5801731397537	486236915496,655	468811123547,862	0,0526526268937908	0,0158359359033661	0,0097756825406766	0,964161931368387	-0.0526526268937893	1,0371701759483	0.0526526268937892	1916
P61967	sp|P61967|AP1S1_MOUSE AP-1 complex subunit sigma-1A OS=Mus musculus GN=Ap1s1 PE=1 SV=1	−1,04	0,865588501876862	0,968277164979127	27,6776121109565	27,6503089516748	27,2306522722493	27,1975304598764	27,5067207470531	27,745330412391	894494369198,398	862580118978,311	0,0524139358455522	0,00910418494526288	0,0099936257389915	0,964321463254501	-0.0524139358455516	1,03699859238338	0.0524139358455515	1917
Q80UW2	sp|Q80UW2|FBX2_MOUSE F-box only protein 2 OS=Mus musculus GN=Fbxo2 PE=1 SV=1	−1,04	0,488105683623334	0,805933684236564	29,2565888136709	29,1006990223782	29,1918467273542	29,1030743452272	29,1461064414382	29,1918467273542	4721014620841,92	4553918428434,06	0,0519885013436767	0,00268363834511096	0,0015230748098704	0,964605872714272	-0.0519885013436747	1,03669283827407	0.0519885013436748	1918
Q62159	sp|Q62159|RHOC_MOUSE Rho-related GTP-binding protein RhoC OS=Mus musculus GN=Rhoc PE=1 SV=2	−1,04	0,97368491656078	0,994052144909892	23,4643347891491	25,914114289794	24,1582085472173	25,8471838387831	24,5360198544844	23,0456642223253	44210104509,7146	42649837687,7806	0,0518358936315306	0,051512054216637	0,0572682644505521	0,964707913739694	-0.0518358936315298	1,03658318311446	0.05183589363153	1919
Q99PL6	sp|Q99PL6|UBXN6_MOUSE UBX domain-containing protein 6 OS=Mus musculus GN=Ubxn6 PE=1 SV=1;sp|Q99PL6-2|UBXN6_MOUSE Isoform 2 of UBX domain-containing protein 6 OS=Mus musculus GN=Ubxn6	−1,04	0,861244117925481	0,967141774186977	29,0776180365338	29,6357657449474	29,643927476549	29,4085535576116	29,5664435234524	29,2745375866608	6180603806916,72	5962503480701,34	0,0518295841191785	0,0110221259032995	0,0049671284757890	0,964712132822475	-0.0518295841191797	1,03657864971003	0.0518295841191798	1920
P52480-2	sp|P52480-2|KPYM_MOUSE Isoform M1 of Pyruvate kinase PKM OS=Mus musculus GN=Pkm;sp|P14618-2|KPYM_HUMAN Isoform M1 of Pyruvate kinase PKM OS=Homo sapiens GN=PKM	−1,04	0,829167937590242	0,956900945145962	35,9416998882443	36,5127737376142	36,2026315719732	36,2026315719732	36,1442586580311	36,2026315719732	5366944064550870	5177889491021540	0,0517366772269595	0,00789336561729018	0,0009314168414125	0,964774260499927	-0.0517366772269592	1,03651189811161	0.0517366772269592	1921
Q6NVF0	sp|Q6NVF0|OCRL_MOUSE Inositol polyphosphate 5-phosphatase OCRL-1 OS=Mus musculus GN=Ocrl PE=1 SV=1;sp|Q6NVF0-2|OCRL_MOUSE Isoform 2 of Inositol polyphosphate 5-phosphatase OCRL-1 OS=Mus musculus GN=Ocrl	−1,04	0,879160668313707	0,973716798448825	28,6090306171416	27,8740286712693	28,2008611250849	28,2304457403943	28,3456414045074	28,0003802943019	1816574083026,53	1752660110828,8	0,0516739577133863	0,0130455800643366	0,0062352986477556	0,964816203866982	-0.0516739577133893	1,03646683792416	0.0516739577133894	1922
Q08509	sp|Q08509|EPS8_MOUSE Epidermal growth factor receptor kinase substrate 8 OS=Mus musculus GN=Eps8 PE=1 SV=2	−1,04	0,825447293928251	0,955744170441451	26,9915324948691	26,8215162251178	26,6147061508255	26,8570170371907	26,5380610554905	26,9257179606655	439653003185,857	424254218503,391	0,0514363185123877	0,00703907442193693	0,0077260311156396	0,964975140461041	-0.0514363185123867	1,03629612626313	0.0514363185123865	1923
Q61207	sp|Q61207|SAP_MOUSE Prosaposin OS=Mus musculus GN=Psap PE=1 SV=2	−1,04	0,854443270135879	0,963607051252994	29,9229385752534	29,7991526231768	29,5272518601179	29,4348108568803	29,7453450924431	29,9631043655379	8320812477304,32	8031722307041,83	0,0510150160802419	0,00680377682605013	0,0089350889502140	0,96525697808345	-0.0510150160802413	1,03599354649115	0.0510150160802413	1924
Q7TN29	sp|Q7TN29|SMAP2_MOUSE Stromal membrane-associated protein 2 OS=Mus musculus GN=Smap2 PE=1 SV=1;sp|Q7TN29-2|SMAP2_MOUSE Isoform 2 of Stromal membrane-associated protein 2 OS=Mus musculus GN=Smap2	−1,04	0,88150478637723	0,973716798448825	28,9750635875556	28,4053017035069	28,3564695698073	28,4245313453588	28,7995948141363	28,4070567271281	2580346875320,16	2491055491792,59	0,050807859768618	0,0120339149773337	0,0077691210034600	0,965395589104085	-0.050807859768614	1,03584479905075	0.050807859768614	1925

Q5DTN8	sp|Q5DTN8|JKIP3_MOUSE Janus kinase and microtubule-interacting protein 3 OS=Mus musculus GN=Jakmip3 PE=2 SV=2;sp|Q5DTN8-2|JKIP3_MOUSE Isoform 2 of Janus kinase and microtubule-interacting protein 3 OS=Mus musculus GN=Jakmip3;sp|Q5DTN8-3|JKIP3_MOUSE Isof	−1,04	0,850492223505175	0,96244931500623	24,534409034057	24,2886600925541	24,6197060572759	24,6425366875427	24,1331519049364	24,5615634697321	42848010919,4086	41367057455,8919	0,050745894780292	0,00702103887818333	0,0111974135966482	0,965437054562412	-0.0507458947802942	1,0358003095844	0.0507458947802941	1926
Q8BPA8	sp|Q8BPA8|DPCD_MOUSE Protein DPCD OS=Mus musculus GN=Dpcd PE=2 SV=1;sp|Q8BPA8-2|DPCD_MOUSE Isoform 2 of Protein DPCD OS=Mus musculus GN=Dpcd	−1,04	0,868360562187634	0,970093180351876	25,2133112519035	25,5721737946229	25,5653482547121	25,6315175429121	25,5454020810795	25,0688342117586	112957532572,555	109069516133,434	0,050532541253063	0,00806464270848105	0,0119251276348679	0,965579839161026	-0.050532541253061	1,03564714117155	0.0505325412530608	1927
Q99PW4	sp|Q99PW4|PRPK_MOUSE TP53-regulating kinase OS=Mus musculus GN=Tp53rk PE=2 SV=1	−1,04	0,928094132631847	0,984256838321484	24,2636612879042	24,903996713159	25,1021464688603	24,9865539749528	24,1418352095624	25,0371457729701	56448891470,1956	54520629276,3999	0,050143036170167	0,0177021324630579	0,0203439405963661	0,965840565800769	-0.0501430361701667	1,03536757039285	0.0501430361701666	1928
P70441	sp|P70441|NHRF1_MOUSE Na(+)/H(+) exchange regulatory cofactor NHE-RF1 OS=Mus musculus GN=Slc9a3r1 PE=1 SV=3	−1,04	0,668841575898808	0,898718742748576	29,9548618864297	29,6831401315376	29,8175332931728	29,763614849229	29,8255701629118	29,7621840572139	8912819685620,78	8608659143556,14	0,050093373483957	0,0045563481205321	0,0012150912650601	0,965873814034929	-0.0500933734839568	1,03533192997801	0.050093373483957	1929
Q9R0A0	sp|Q9R0A0|PEX14_MOUSE Peroxisomal membrane protein PEX14 OS=Mus musculus GN=Pex14 PE=1 SV=1	−1,04	0,847537561866322	0,961525279907636	25,8825371944796	26,3947233703869	25,9735589231531	26,1217410182589	26,1491158481579	25,8761040310522	212797425474,484	205556539786,778	0,049945424513689	0,010475995862405	0,0057716394070754	0,965972869871143	-0.0499454245136863	1,03522576170633	0.0499454245136861	1930
Q8BJZ4	sp|Q8BJZ4|RT35_MOUSE 28S ribosomal protein S35, mitochondrial OS=Mus musculus GN=Mrps35 PE=2 SV=2	−1,04	0,950228070760114	0,988334326693467	24,8859998394022	24,7747333955639	25,3393502864799	24,3182133554805	24,573149001652	26,0052225567599	72006904016,3969	69565062635,6968	0,049772309285527	0,0119632535991694	0,0364254074568245	0,966088788095318	-0.0497723092855283	1,03510154793488	0.0497723092855283	1931
P56480	sp|P56480|ATPB_MOUSE ATP synthase subunit beta, mitochondrial OS=Mus musculus GN=Atp5b PE=1 SV=2	−1,04	0,797729121488173	0,944662792182316	34,5341448386175	34,1536747954851	34,2755682575707	34,2285718437873	34,4336965995204	34,1977817973996	804409269474259	777172472170209	0,049694905528562	0,00566076547142795	0,0037403602893008	0,966140622270736	-0.0496949055285617	1,03504601395362	0.0496949055285618	1932
P51163	sp|P51163|HEM4_MOUSE Uroporphyrinogen-III synthase OS=Mus musculus GN=Uros PE=2 SV=1	−1,04	0,842459901525556	0,959771286845933	26,8914057622921	27,1464541510055	27,1039852603133	27,3070559481143	26,7858549456868	26,9456779435363	557808597101,384	538936152722,705	0,049655801427358	0,00505235379250433	0,0098848031959433	0,966166809768174	-0.0496558014273583	1,03501795951772	0.0496558014273582	1933
Q9CWD8	sp|Q9CWD8|NUBPL_MOUSE Iron-sulfur protein NUBPL OS=Mus musculus GN=Nubpl PE=2 SV=2	−1,03	0,803556782039972	0,946624509130401	26,126952961226	26,3439130720978	26,5771116775476	26,2266208709123	26,3327598269784	26,385525166963	277566367478,754	268191904189,768	0,049567080368324	0,00854398202508728	0,0030755237751224	0,966226227715778	-0.0495670803683267	1,03495431123213	0.0495670803683268	1934
P47911	sp|P47911|RL6_MOUSE 60S ribosomal protein L6 OS=Mus musculus GN=Rpl6 PE=1 SV=3	−1,03	0,701429188402567	0,913830966176594	27,4581127618677	27,3601226677854	27,436658796006	27,3177386114909	27,2837384201724	27,5509487648623	808379994565,652	781235073719,294	0,049276898186196	0,0018786977485988	0,0053116804214845	0,966420593002251	-0.0492768981861975	1,03474616253099	0.0492768981861975	1935
Q99KN9	sp|Q99KN9|EPN4_MOUSE Clathrin interactor 1 OS=Mus musculus GN=Clint1 PE=1 SV=2;sp|Q99KN9-2|EPN4_MOUSE Isoform 2 of Clathrin interactor 1 OS=Mus musculus GN=Clint1	−1,03	0,91736172422788	0,980665300581806	27,1705289713067	27,6317151295644	26,6952330944468	27,4236219219812	27,1876699138534	26,7839028148677	628012389531,048	606961713000,045	0,049187506628822	0,0172370513399053	0,0119235765744056	0,966480475732776	-0.0491875066288239	1,03468205008674	0.0491875066288239	1936
Q8BVF2	sp|Q8BVF2|PDCL3_MOUSE Phosducin-like protein 3 OS=Mus musculus GN=Pdcl3 PE=2 SV=1	−1,03	0,79137624190763	0,942055274591853	25,4993244819944	25,6571152896275	25,8984378098441	25,6870742225767	25,5441223434088	25,7219669464862	142835462637,96	138073847275,123	0,048914127642226	0,00782591351941429	0,0036737292632620	0,966663633281981	-0.0489141276422328	1,03448600482138	0.0489141276422329	1937
Q9CR11	sp|Q9CR11|YETS4_MOUSE YEATS domain-containing protein 4 OS=Mus musculus GN=Yeats4 PE=2 SV=1	−1,03	0,843473818732855	0,959771286845933	23,5434255149496	23,8514133380406	24,0187780694206	23,9556768269568	23,6104580044563	23,7458020638621	21786121468,8407	21060090307,7096	0,048897756968621	0,0101290790355407	0,0073176446530642	0,966674602353177	-0.0488977569686206	1,03447426627916	0.0488977569686205	1938
Q9JKF7	sp|Q9JKF7|RM39_MOUSE 39S ribosomal protein L39, mitochondrial OS=Mus musculus GN=Mrpl39 PE=2 SV=4	−1,03	0,735776926789017	0,924701393764781	24,9114903418552	24,6622689173269	24,8413906379364	24,6402971622954	24,8070307785967	24,8661841819719	59251087173,0856	57277327837,0626	0,048877437627946	0,00518134772054436	0,0047286973837110	0,966688217378068	-0.0488774376279458	1,03445969654237	0.0488774376279458	1939
Q60864	sp|Q60864|STIP1_MOUSE Stress-induced-phosphoprotein 1 OS=Mus musculus GN=Stip1 PE=1 SV=1	−1,03	0,859593731328417	0,966502999979251	32,4429895051478	33,0034445863308	32,9720957537316	32,8872840950895	32,7150311669666	32,7150311669666	176824108135756	170959672224656	0,048658937584617	0,00959939917924904	0,0030345695250903	0,966834635995463	-0.0486589375846229	1,03430303670326	0.0486589375846231	1940
P51859	sp|P51859|HDGF_MOUSE Hepatoma-derived growth factor OS=Mus musculus GN=Hdgf PE=1 SV=2	−1,03	0,881754027561286	0,973716798448825	29,541802057065	29,2657804305218	28,808297486709	29,3062942508444	29,1607446221874	29,0477196217778	4827227126111,94	4667226737889,65	0,048629152327452	0,0126851787542339	0,0044436318767130	0,9668545970508	-0.0486291523274529	1,03428168315102	0.048629152327453	1941
Q9DBG3	sp|Q9DBG3|AP2B1_MOUSE AP-2 complex subunit beta OS=Mus musculus GN=Ap2b1 PE=1 SV=1;sp|Q9DBG3-2|AP2B1_MOUSE Isoform 2 of AP-2 complex subunit beta OS=Mus musculus GN=Ap2b1	−1,03	0,906134056568047	0,979971999705115	34,1068660507989	33,3079535850214	33,7374582476704	33,9115726182197	33,7804120503292	33,3596098324373	439837282157024	425320804259891	0,048418472213035	0,0118583423218738	0,0085610462971083	0,966995799387578	-0.0484184722130359	1,03413065561745	0.0484184722130361	1942
Q8BTG3	sp|Q8BTG3|T11L1_MOUSE T-complex protein 11-like protein 1 OS=Mus musculus GN=Tcp11l1 PE=2 SV=1	−1,03	0,849904059546861	0,962394740430361	26,6710608514719	27,2405304600518	27,097740617644	27,0584803792591	26,9164428189902	26,9340296968593	533705829513,966	516143655099,02	0,048272111548762	0,0109725813620246	0,0028709853335369	0,967093905586642	-0.0482721115487625	1,0340257489198	0.0482721115487626	1943

P70333	sp|P70333|HNRH2_MOUSE Heterogeneous nuclear ribonucleoprotein H2 OS=Mus musculus GN=Hnrnph2 PE=2 SV=1	−1,03	0,601101712863004	0,862877020598131	29,1702498422972	29,0412682162963	29,2598728637706	29,1293211094485	29,1143759145037	29,1279023668535	4600243291376,8	4449738576832,85	0,0479895825673916	0,00376893272939227	0,00028325950740766	0,96728331416165		-0.0479895825673935	1,03382327117542	0.0479895825673935	1944
Q9EPR4	sp|Q9EPR4|S23A2_MOUSE Solute carrier family 23 member 2 OS=Mus musculus GN=Slc23a2 PE=1 SV=2	−1,03	0,881371073088703	0,973716798448825	23,6506356046121	23,0151791532857	23,0239680619191	23,3276794305009	23,1166209487376	23,1467880451938	12263921855,1411	11867026097,7137	0,0474619715949799	0,0156853831159985	0,00492076578582400	0,967637126025797		-0.0474619715949731	1,03344525866543	0.0474619715949731	1945
O35114	sp|O35114|SCRB2_MOUSE Lysosome membrane protein 2 OS=Mus musculus GN=Scarb2 PE=1 SV=3	−1,03	0,880594512016333	0,973716798448825	28,4357489471046	28,2885021532674	27,7534331423157	28,1337438378691	28,2385123208996	28,0072704850482	1695888792851,72	1641298612425,89	0,0472038270388231	0,0127509841018306	0,00411677703082663	0,967810282928969		-0.0472038270388243	1,03326035860418	0.0472038270388241	1946
P14873	sp|P14873|MAP1B_MOUSE Microtubule-associated protein 1B OS=Mus musculus GN=Map1b PE=1 SV=2	−1,03	0,898197714446743	0,977644369603516	30,3961101691837	31,2308454760787	30,8467694077559	30,9049840027489	30,8405947262776	30,6302391026061	24374899693676,7	23592247475936,5	0,0470834209201258	0,0135544449859368	0,00466646841089120	0,967891058934563		-0.0470834209201306	1,03317412715929	0.0470834209201305	1947
Q8VE99	sp|Q8VE99|CC115_MOUSE Coiled-coil domain-containing protein 115 OS=Mus musculus GN=Ccdc115 PE=2 SV=1	−1,03	0,93120407547764	0,98433518830764	23,8645191979705	23,9338055309889	23,3258529818771	23,0551430075932	23,8182850170703	24,1529970070456	19782414767,2403	19148208754,4153	0,0470091018035596	0,014037707605837	0,0237666695335996	0,967940920242192		-0.0470091018035568	1,03312090550918	0.0470091018035569	1948
Q8C6I2	sp|Q8C6I2|SDHF2_MOUSE Succinate dehydrogenase assembly factor 2, mitochondrial OS=Mus musculus GN=Sdhaf2 PE=2 SV=1;sp|Q8C6I2-2|SDHF2_MOUSE Isoform 2 of Succinate dehydrogenase assembly factor 2, mitochondrial OS=Mus musculus GN=Sdhaf2	−1,03	0,969102252333167	0,992417383342423	23,2024655836816	24,723259791271	22,4202327163321	24,1582085472173	23,6236389235436	22,4664435194351	15262317167,2071	14773442745,2617	0,0469679474662568	0,0499425177156547	0,0369304624129138	0,967968532131163		-0.046967947466259	1,03309143510927	0.046967947466259	1949
P62137	sp|P62137|PP1A_MOUSE Serine/threonine-protein phosphatase PP1-alpha catalytic subunit OS=Mus musculus GN=Ppp1ca PE=1 SV=1	−1,03	0,846829187155429	0,961355015563972	30,1478585778955	29,6508210992447	29,6647193245074	29,8416907060905	29,7939275700076	29,7312890475709	8936212883556,86	8653362657741,5	0,046402688435572	0,00949118766788451	0,00185866121903294	0,968347863966421		-0.0464026884355725	1,03268674121295	0.0464026884355723	1950
Q9ESP1	sp|Q9ESP1|SDF2L_MOUSE Stromal cell-derived factor 2-like protein 1 OS=Mus musculus GN=Sdf2l1 PE=2 SV=2	−1,03	0,898761021533772	0,977644369603516	24,7005673453217	25,0773874416878	25,2169254586012	25,3625137240185	24,7178927598584	24,817993141014	71882121665,9606	69607155526,9103	0,0463973710181346	0,0106851722315546	0,013895027667504	0,968351433063952		-0.0463973710181339	1,0326829349918	0.0463973710181339	1951
A6H5Z3	sp|A6H5Z3|EXC6B_MOUSE Exocyst complex component 6B OS=Mus musculus GN=Exoc6b PE=2 SV=1;sp|A6H5Z3-2|EXC6B_MOUSE Isoform 2 of Exocyst complex component 6B OS=Mus musculus GN=Exoc6b	−1,03	0,767717585062071	0,935597979611256	28,2008611250849	28,046173766494	27,8740286712693	28,0173481149224	28,0971533682127	27,9101208805373	1505813682644,81	1458175998573,66	0,0463784132627433	0,00583066512173814	0,00335082807536700	0,968364157783798		-0.0463784132627412	1,03266936509567	0.0463784132627414	1952
Q640R3	sp|Q640R3|HECAM_MOUSE Hepatocyte cell adhesion molecule OS=Mus musculus GN=Hepacam PE=1 SV=2	−1,03	0,881789985034471	0,973716798448825	25,7441964423723	26,3924755423625	26,35933783101	26,1785692818595	26,0680641034151	26,1541626625147	230919870248,201	223706034870,737	0,0457881436179565	0,0139533553944261	0,00222134572329004	0,968760438979503		-0.0457881436179573	1,03224694131132	0.0457881436179572	1953
P57776-3	sp|P57776-3|EF1D_MOUSE Isoform 3 of Elongation factor 1-delta OS=Mus musculus GN=Eef1d	−1,03	0,763470083913209	0,933454063694469	29,5177234927931	29,4637941408889	29,4026825687162	29,2543000086044	29,4348108568803	29,6000841427096	6236249447121,83	6041851794609,32	0,0456878408456163	0,00195366874618666	0,0058766425630132	0,968827793987261		-0.0456878408456158	1,032175177267	0.0456878408456157	1954
Q99M04	sp|Q99M04|LIAS_MOUSE Lipoyl synthase, mitochondrial OS=Mus musculus GN=Lias PE=2 SV=1	−1,03	0,957930322467854	0,990217657408697	23,1629885154419	22,6960719862823	24,0493524445907	22,7369952893588	22,9159360119671	24,1607946176825	13191048801,8452	12781210158,5197	0,045534834910555	0,0294981143351945	0,03332668157493	0,968930549080512		-0.0455348349105587	1,03206571508038	0.0455348349105587	1955
Q9JIW9	sp|Q9JIW9|RALB_MOUSE Ras-related protein Ral-B OS=Mus musculus GN=Ralb PE=2 SV=1	−1,03	0,874519653125172	0,972636416581711	25,1667818406085	25,5256101773492	25,7112604959504	25,6571152896275	25,2470629747378	25,404963452821	114963919350,442	111398597255,113	0,0454500859136646	0,0108682520435276	0,00813101534109749	0,968987469151422		-0.0454500859136659	1,03200508967958	0.0454500859136659	1956
Q9JII6	sp|Q9JII6|AK1A1_MOUSE Alcohol dehydrogenase [NADP(+)] OS=Mus musculus GN=Akr1a1 PE=1 SV=3	−1,03	0,759548553002677	0,933136198610489	31,8397012538833	31,5035229610434	31,63710827341	31,6276420584925	31,6843541136303	31,5739501993876	56209721002529,9	54468778969464,4	0,0453901275580676	0,005346332200348	0,00174553273101597	0,969027741073699		-0.0453901275580684	1,03196220047528	0.0453901275580685	1957
Q80YD6	sp|Q80YD6|SRGEF_MOUSE Secretion-regulating guanine nucleotide exchange factor OS=Mus musculus GN=Sergef PE=2 SV=2	−1,03	0,854259779181171	0,963607051252994	22,5136465441664	23,0112645071695	22,9565167887036	22,7337848548684	22,9274759558395	22,7260651701659	8197889773,03933	7944735356,63903	0,0452534285189122	0,011953842658734	0,00500625144924543	0,969119563276777		-0.045253428518913	1,03186442405394	0.0452534285189131	1958
P63261	sp|P63261|ACTG_HUMAN Actin, cytoplasmic 2 OS=Homo sapiens GN=ACTG1 PE=1 SV=1;sp|P63260|ACTG_MOUSE Actin, cytoplasmic 2 OS=Mus musculus GN=Actg1 PE=1 SV=1;sp|P63258|ACTG_BOVIN Actin, cytoplasmic 2 OS=Bos taurus GN=ACTG1 PE=1 SV=1	−1,03	0,317310507862914	0,722907157640623	37,8289878833996	37,7354459507886	37,7354459507886	37,7354459507886	37,7354459507886	37,7354459507886	25225369141363100	24450961880868700	0,0449841607644028	0,00143000487050901	0	0,969300458750292		-0.0449841607644008	1,0316718525949	0.0449841607644006	1959
Q52KF3	sp|Q52KF3|SPIR1_MOUSE Protein spire homolog 1 OS=Mus musculus GN=Spire1 PE=2 SV=1;sp|Q52KF3-2|SPIR1_MOUSE Isoform 2 of Protein spire homolog 1 OS=Mus musculus GN=Spire1;sp|Q52KF3-3|SPIR1_MOUSE Isoform 3 of Protein spire homolog 1 OS=Mus musculus GN=Spir	−1,03	0,834780191671759	0,957953242267243	26,9837240922818	26,6089362359476	26,4937458641267	26,69891974877	26,660624355741	26,6335512707477	392369066815,684	380352817147,464	0,0448730176958592	0,00959692457292862	0,00123177124574089	0,969375135084579		-0.04487301769586	1,03159237719951	0.0448730176958599	1960
Q9ES56	sp|Q9ES56|TPPC4_MOUSE Trafficking protein particle complex subunit 4 OS=Mus musculus GN=Trappc4 PE=1 SV=1	−1,03	0,826979432367455	0,955959931044103	28,1750053595332	28,3478835582042	28,3160857868948	28,0488815319204	28,2047593538113	28,4931602800957	1912931952913,04	1855051773688,11	0,0443261024449695	0,00325378727503145	0,00797939773163894	0,969742687847945		-0.0443261024449636	1,03120138211014	0.0443261024449636	1961

Q9JKP5	sp|Q9JKP5|MBNL1_MOUSE Muscleblind-like protein 1 OS=Mus musculus GN=Mbnl1 PE=1 SV=1;sp|Q8C181-3|MBNL2_MOUSE Isoform 3 of Muscleblind-like protein 2 OS=Mus musculus GN=Mbnl2;sp|Q8C181-2|MBNL2_MOUSE Isoform 2 of Muscleblind-like protein 2 OS=Mus musculus	−1,03	0,921859934258995	0,982113422958876	27,0626686517635	26,1449400230198	26,8016686908167	26,3515957296419	26,8329937693396	26,7334809843027	382409982201,624	370958794653,423	0,043861238937349	0,0177305895688093	0,009539532386068	0,970055207549045	-0.04386123893735	1,03086916313414	0.04386123893735	1962
P49443	sp|P49443|PPM1A_MOUSE Protein phosphatase 1A OS=Mus musculus GN=Ppm1a PE=1 SV=1	−1,03	0,781512624424647	0,940251150142017	29,6149568203859	29,3914357617658	29,5895854758228	29,3473309859604	29,5228299333287	29,6347178699823	6692393413755,55	6492223961695,11	0,043809487728933	0,00414415317267455	0,0049103059239227	0,970090005221598	-0.0438094877289333	1,03083218527911	0.0438094877289334	1963
Q9R0Q9	sp|Q9R0Q9|MPU1_MOUSE Mannose-P-dolichol utilization defect 1 protein OS=Mus musculus GN=Mpdu1 PE=2 SV=1	−1,03	0,914655773497892	0,980665300581806	24,3990475359719	23,5506907098139	23,9703394650702	23,8201140153781	23,7832496113561	24,2257383334506	25792748847,5817	25022318078,8491	0,043750088111562	0,017694077437809	0,0102544234348715	0,970129947246595	-0.0437500881115649	1,0307897440319	0.0437500881115647	1964
Q7TSS2	sp|Q7TSS2|UB2Q1_MOUSE Ubiquitin-conjugating enzyme E2 Q1 OS=Mus musculus GN=Ube2q1 PE=1 SV=2;sp|Q7TSS2-2|UB2Q1_MOUSE Isoform 2 of Ubiquitin-conjugating enzyme E2 Q1 OS=Mus musculus GN=Ube2q1	−1,03	0,85769997665978	0,965434916957169	26,6292631829283	26,5652571906599	26,2799304014618	26,4330141857912	26,2503112648405	26,7001686947059	319966964567,146	310411512905,134	0,043740892874037	0,00702054144324279	0,0085501160774683	0,970136130537918	-0.0437408928740353	1,03078317415673	0.0437408928740352	1965
Q9D8W5	sp|Q9D8W5|PSD12_MOUSE 26S proteasome non-ATPase regulatory subunit 12 OS=Mus musculus GN=Psmd12 PE=1 SV=4	−1,03	0,736617743812988	0,924701393764781	30,6767393272954	30,4856864089267	30,5924988627797	30,6402261423119	30,413494702835	30,6103162203833	19181643431027,4	18609233939968,7	0,043707664606074	0,00313057090161723	0,0040314740436149	0,970158475048452	-0.0437076646060723	1,03075943334934	0.0437076646060724	1966
Q8R404	sp|Q8R404|QIL1_MOUSE Protein QIL1 OS=Mus musculus GN=Qil1 PE=2 SV=1	−1,03	0,783782128357331	0,940251150142017	24,5823748158982	24,5700026587245	24,3302859701029	24,4023850233445	24,3792155998351	24,6103867125833	43421526102,8419	42128730458,9755	0,043605990909191	0,00580164339910372	0,0052038293735252	0,970226849217495	-0.0436059909091938	1,03068679330666	0.0436059909091937	1967
Q8R081	sp|Q8R081|HNRPL_MOUSE Heterogeneous nuclear ribonucleoprotein L OS=Mus musculus GN=Hnrnpl PE=1 SV=2	−1,03	0,885675357195588	0,975149870380105	30,0459928945559	30,6837245297255	30,5433566106407	30,5561361051944	30,295446665439	30,3309155382186	16335438611405,1	15849611468938,7	0,043557716942066	0,0110143924254303	0,0046517736052137	0,97025931448653	-0.043557716942063	1,03065230610974	0.0435577169420629	1968
P48057	sp|P48057|SC6A1_MUSCO Sodium- and chloride-dependent GABA transporter 1 OS=Mus cookii GN=Slc6a1 PE=2 SV=1;sp|P31648|SC6A1_MOUSE Sodium- and chloride-dependent GABA transporter 1 OS=Mus musculus GN=Slc6a1 PE=1 SV=2	−1,03	0,852667257483313	0,962750418337502	28,8256961916759	29,3099584234601	29,0412682162963	29,1231989742319	28,8685229978762	29,0951539669868	4170154815811,98	4046844859378,05	0,043303401674188	0,00834909612549491	0,0048106154672543	0,970430364847277	-0.0433034016741872	1,03047064088661	0.0433034016741873	1969
Q3V0K9	sp|Q3V0K9|PLSI_MOUSE Plastin-1 OS=Mus musculus GN=Pls1 PE=2 SV=1	−1,03	0,965529716506904	0,991306002794532	26,8676217628967	26,0184741068914	25,5763604830779	27,2551673122194	25,7441964423723	25,3737724117129	228351539322,722	221652953276,312	0,042953930067800	0,0250910720862944	0,0381502739404618	0,970665465771426	-0.0429539300678027	1,03022105479488	0.0429539300678026	1970
P70704-3	sp|P70704-3|AT8A1_MOUSE Isoform 3 of Phospholipid-transporting ATPase IA OS=Mus musculus GN=Atp8a1;sp|P70704-2|AT8A1_MOUSE Isoform 2 of Phospholipid-transporting ATPase IA OS=Mus musculus GN=Atp8a1;sp|P70704|AT8A1_MOUSE Phospholipid-transporting ATPase	−1,03	0,901123484556624	0,977644369603516	27,1640538722618	26,6409567083219	26,4796440426437	26,6526229894318	26,9699393539991	26,5728087866134	419173830631,446	406882538150,265	0,042936284282827	0,013369441667412	0,0078583778192750	0,970677338175751	-0.0429362842828249	1,0302084541083	0.0429362842828248	1971
Q99KQ4	sp|Q99KQ4|NAMPT_MOUSE Nicotinamide phosphoribosyltransferase OS=Mus musculus GN=Nampt PE=1 SV=1	−1,03	0,890131642141404	0,976830327013275	28,8699406290185	29,450672574706	29,5137526899067	29,3158045711717	29,3120180582318	29,1173946133026	5191905865809,39	5039891890926,29	0,042871438863838	0,0121216679301282	0,0038796906210862	0,970720968597646	-0.0428714388638392	1,03016214993754	0.0428714388638391	1972
Q60631	sp|Q60631|GRB2_MOUSE Growth factor receptor-bound protein 2 OS=Mus musculus GN=Grb2 PE=1 SV=1;sp|Q60631-2|GRB2_MOUSE Isoform 2 of Growth factor receptor-bound protein 2 OS=Mus musculus GN=Grb2	−1,03	0,889936663633324	0,976830327013275	29,8416907060905	30,343830029212	30,5018948562202	30,343830029212	30,1496750736804	30,1058851287532	13438415241704,8	13049836800130	0,042331250055694	0,0114036198725371	0,0041935221489486	0,971084504044131	-0.0423312500556929	1,02977649816823	0.0423312500556929	1973
Q99M51	sp|Q99M51|NCK1_MOUSE Cytoplasmic protein NCK1 OS=Mus musculus GN=Nck1 PE=1 SV=1	−1,03	0,827324304443855	0,955959931044103	27,7557362941429	27,3594544203214	27,5780928315527	27,4002419470032	27,5736087892473	27,6317151295644	935575653212,888	908616201996,71	0,042183287408660	0,00720109375986925	0,0043734735168177	0,971184103472984	-0.0421832874086613	1,02967088981787	0.0421832874086614	1974
Q91W86	sp|Q91W86|VPS11_MOUSE Vacuolar protein sorting-associated protein 11 homolog OS=Mus musculus GN=Vps11 PE=1 SV=3	−1,03	0,879300603691376	0,973716798448825	28,2841998398392	28,0241091157644	27,7483704429287	27,8808173603113	28,2185163002376	27,869672887315	1473841145481,3	1431392480928,59	0,042161728959944	0,00956328914797557	0,0070835430772654	0,971198616158292	-0.0421617289599455	1,0296555033775	0.0421617289599455	1975
P57780	sp|P57780|ACTN4_MOUSE Alpha-actinin-4 OS=Mus musculus GN=Actn4 PE=1 SV=1	−1,03	0,866646609462902	0,968655247562697	33,2027166374192	32,8095502245621	32,8500916619242	32,839500065196	33,1617830500677	32,773886273776	205018125446068	199145427062074	0,041929110830083	0,00656196007004588	0,0063058237949128	0,97135522348955	-0.0419291108300885	1,02948949654849	0.0419291108300886	1976
Q61598	sp|Q61598|GDIB_MOUSE Rab GDP dissociation inhibitor beta OS=Mus musculus GN=Gdi2 PE=1 SV=1;sp|Q61598-2|GDIB_MOUSE Isoform 2 of Rab GDP dissociation inhibitor beta OS=Mus musculus GN=Gdi2	−1,03	0,750728408203431	0,928854093158237	34,1536747954851	33,9705548759834	34,172245247307	34,1977817973996	34,0061355969496	34,0061355969496	644067707853644	625778545817885	0,041560162064879	0,00326910229737082	0,0032476351057679	0,971603665557605	-0.0415601620648783	1,02922625289407	0.0415601620648783	1977
Q99K70	sp|Q99K70|RRAGC_MOUSE Ras-related GTP-binding protein C OS=Mus musculus GN=Rragc PE=1 SV=1	−1,03	0,84899857315517	0,96228660095196	28,8811067240288	28,4070567271281	28,6424177181693	28,5119947056563	28,7164765416841	28,6169807290695	2752489955867,3	2675481978175,04	0,040938488151730	0,0082750614116582	0,0035733937786628	0,972022430989037	-0.0409384881517269	1,02878284298696	0.0409384881517268	1978
Q8BH58	sp|Q8BH58|TIPRL_MOUSE TIP41-like protein OS=Mus musculus GN=Tiprl PE=2 SV=1	−1,03	0,885200978937907	0,975149870380105	27,8881308498074	28,187447373342	28,2756209716639	28,2792329488929	28,2102303198389	27,7767708216757	1625873617910,59	1580472069967,53	0,040859578258325	0,00722360293871215	0,0096968121592258	0,972075598347302	-0.0408595782583284	1,02872657404442	0.0408595782583284	1979

Q8BIF2-4	sp|Q8BIF2-4|RFOX3_MOUSE Isoform 4 of RNA binding protein fox-1 homolog 3 OS=Mus musculus GN=Rbfox3;sp|Q8BIF2-5|RFOX3_MOUSE Isoform 5 of RNA binding protein fox-1 homolog 3 OS=Mus musculus GN=Rbfox3;sp|Q8BIF2-2|RFOX3_MOUSE Isoform 2 of RNA binding protei	−1,03	0,879258087397543	0,973716798448825	25,6958788171413	25,1293167074687	25,2118538008189	25,3172002676571	25,2227384535695	25,4127860971458	101739667527,984	98919768513,4114	0,040551516051866	0,0120759541106738	0,0037532969094526	0,97283190194258	-0.0405515160518731	1,02850693099014	0.04055151605187	1980
Q8C985	sp|Q8C985|NRX3B_MOUSE Neurexin-3-beta OS=Mus musculus GN=Nrxn3 PE=1 SV=2;sp|Q6P9K9-2|NRX3A_MOUSE Isoform 2a of Neurexin-3 OS=Mus musculus GN=Nrxn3;sp|Q6P9K9|NRX3A_MOUSE Neurexin-3 OS=Mus musculus GN=Nrxn3 PE=1 SV=2	−1,03	0,917080344535988	0,980665300581806	23,7908431155132	24,3731216083975	24,3357757397456	24,5072217421758	23,9578712584091	23,9524385927456	31290493131,9354	30444682879,5262	0,039534109845324	0,0134869373786733	0,0132045718428778	0,97296909803105	-0.0395341098453266	1,02778187100047	0.03953410984532	1981
Q8BG18	sp|Q8BG18|NECA1_MOUSE N-terminal EF-hand calcium-binding protein 1 OS=Mus musculus GN=Necab1 PE=1 SV=1	−1,03	0,75458188172478	0,930504185076609	27,9465752622869	27,7598510714024	27,802797894039	27,7796180261822	27,9358995293517	27,7120449619398	1228000159954,3	1195024144636,42	0,039270981471589	0,00351317754348851	0,0041288278170464	0,973146570828535	-0.0392709814715923	1,02759443436008	0.03927098147159	1982
Q8R1T1	sp|Q8R1T1|CHMP7_MOUSE Charged multivesicular body protein 7 OS=Mus musculus GN=Chmp7 PE=1 SV=1;sp|Q8R1T1-2|CHMP7_MOUSE Isoform 2 of Charged multivesicular body protein 7 OS=Mus musculus GN=Chmp7	−1,03	0,913847109419892	0,980665300581806	27,3216488345134	28,1463259904393	27,8477202619897	27,8663186708468	27,7229066479733	27,6454609497024	1151283315122,22	1120611262941,89	0,038957006867563	0,0150344140176121	0,0040388960162415	0,973358380359161	-0.0389570068675548	1,02737082268815	0.03895700686755	1983
Q8BG39	sp|Q8BG39|SV2B_MOUSE Synaptic vesicle glycoprotein 2B OS=Mus musculus GN=Sv2b PE=1 SV=1	−1,03	0,796624248024756	0,944339896000096	30,4662615796495	30,2081872493644	30,3198830461946	30,4511791725536	30,2310954123744	30,2310954123744	14886024837856,4	14489663741808,9	0,038934433252050	0,00426702407081834	0,0041929614374923	0,97337361045915	-0.0389344332520461	1,02735474770914	0.03893443325204	1984
Q3UM45	sp|Q3UM45|PP1R7_MOUSE Protein phosphatase 1 regulatory subunit 7 OS=Mus musculus GN=Ppp1r7 PE=1 SV=2	−1,03	0,921222644252866	0,982113422958876	31,5806609515781	30,9261836153758	31,3069394690914	31,0449398168704	31,6049790076691	31,0862260035438	38100988604468,6	37127594991186	0,03733656676842	0,0105110613668944	0,0099888015200752	0,974452274102714	-0.037336566768429	1,02621752401451	0.037336566768429	1985
Q9R1T2	sp|Q9R1T2|SAE1_MOUSE SUMO-activating enzyme subunit 1 OS=Mus musculus GN=Sae1 PE=1 SV=1;sp|Q9R1T2-2|SAE1_MOUSE Isoform 2 of SUMO-activating enzyme subunit 1 OS=Mus musculus GN=Sae1	−1,03	0,868829865930658	0,970240308853386	29,6309744420806	29,2338980175545	29,2762930224565	29,351342606842	29,5156147134624	29,1969573151545	5750963339622,02	5604764815586,2	0,037149804447177	0,00742150177670511	0,0054285887873287	0,974578428794952	-0.0371498044471772	1,02608468487906	0.03714980444717	1986
O35737	sp|O35737|HNRH1_MOUSE Heterogeneous nuclear ribonucleoprotein H OS=Mus musculus GN=Hnrnph1 PE=1 SV=3	−1,03	0,900985925463971	0,977644369603516	31,7608767264179	31,2206366567217	31,4180321721597	31,5035229610434	31,6276420584925	31,1921742262174	46316305474534,4	45154591362034,1	0,036647488288789	0,00868745176532353	0,0071353050582056	0,97491781564617	-0.0366474882887892	1,02572748589808	0.03664748828878	1987
Q9D832	sp|Q9D832|DNJB4_MOUSE DnaJ homolog subfamily B member 4 OS=Mus musculus GN=Dnajb4 PE=2 SV=1	−1,03	0,852262345419343	0,962750418337502	27,5472752363342	27,2849017576652	27,4280292367288	27,6204662728737	27,2661868502051	27,2974019823772	809812635433,593	789515288428,587	0,036620950262827	0,00479093464562299	0,0071603014735394	0,974935749188282	-0.0366209502628276	1,02570861806287	0.03662095026282	1988
Q99LD8	sp|Q99LD8|DDAH2_MOUSE N(G),N(G)-dimethylarginine dimethylaminohydrolase 2 OS=Mus musculus GN=Ddah2 PE=1 SV=1;sp|O95865|DDAH2_HUMAN N(G),N(G)-dimethylarginine dimethylaminohydrolase 2 OS=Homo sapiens GN=DDAH2 PE=1 SV=1	−1,03	0,898737939730319	0,977644369603516	27,4832478791861	28,01920864903	28,0044923642566	27,9960381244174	27,7105926216416	27,7242460573172	1227069142375,83	1196345091393,19	0,036582941896487	0,0109671433321018	0,0057894186196509	0,974961434591084	-0.0365829418964833	1,02568159572324	0.03658294189648	1989
Q0VGB7	sp|Q0VGB7|PP4R2_MOUSE Serine/threonine-protein phosphatase 4 regulatory subunit 2 OS=Mus musculus GN=Ppp4r2 PE=1 SV=1	−1,03	0,902460004938792	0,977644369603516	25,1642937576338	25,7943002713021	25,4794674273275	25,6487332316027	25,3293144456964	25,3839467459252	116290105882,458	113378559542,579	0,036580510446895	0,0123630793166939	0,0067118517697950	0,974963077746082	-0.0365805104469042	1,02567986708973	0.03658051044690	1990
Q9WUM5	sp|Q9WUM5|SUCA_MOUSE Succinyl-CoA ligase [ADP/GDP-forming] subunit alpha, mitochondrial OS=Mus musculus GN=Suclg1 PE=1 SV=4	−1,03	0,883251411116667	0,973900246537713	31,9696296459334	32,209626068356	32,2182618919671	32,390014054121	31,8823889345278	32,0501774277855	90150883760493,2	87926891391679,8	0,036037170711537	0,00439184608696431	0,0080550032846973	0,975330332038431	-0.0360371707115382	1,02529365400747	0.03603717071153	1991
Q3UE37	sp|Q3UE37|UBE2Z_MOUSE Ubiquitin-conjugating enzyme E2 Z OS=Mus musculus GN=Ube2z PE=2 SV=2	−1,03	0,592339289431224	0,860080030359509	28,2385123208996	28,1272472497798	28,1173618978386	28,1061634244686	28,1167813912022	28,1859618455125	1698965223334,04	1657451371963,94	0,035689778167432	0,00238891705561779	0,0015401080129978	0,975565214166872	-0.0356897781674401	1,02504679900256	0.03568977816744	1992
Q5E924	sp|Q5E924|G3PP2_ARATH Glyceraldehyde-3-phosphate dehydrogenase GAPCP2, chloroplastic OS=Arabidopsis thaliana GN=GAPCP2 PE=2 SV=1;sp|Q9SAJ6|G3PP1_ARATH Glyceraldehyde-3-phosphate dehydrogenase GAPCP1, chloroplastic OS=Arabidopsis thaliana GN=GAPCP1 PE=1 S	−1,02	0,787038295509465	0,941831485187835	33,6444146179947	33,3509572701444	33,4715004505582	33,404240792523	33,4947052049967	33,4947052049967	350001111538912	341562024046442	0,035211923352142	0,00440460024950518	0,001560745692626	0,975888398024325	-0.0352119233521441	1,02470733541303	0.03521192335214	1993

P58252	sp|P58252|EF2_MOUSE Elongation factor 2 OS=Mus musculus GN=Eef2 PE=1 SV=2;sp|P13639|EF2_HUMAN Elongation factor 2 OS=Homo sapiens GN=EEF2 PE=1 SV=4	−1,02	0,861962434459598	0,967230529051203	33,5825229115311	33,5071865503134	33,1617830500677	33,3596098324373	33,4947052049967	33,3243876702912	325754182234637	317945437111023	0,0350044965092587	0,00671365623359232	0,0026923933682545	0,976028718741086	-0.0350044965092576	1,02456001631779	0.0350044965092575	1994
P38647	sp|P38647|GRP75_MOUSE Stress-70 protein, mitochondrial OS=Mus musculus GN=Hspa9 PE=1 SV=3	−1,02	0,835074273228855	0,957953242267243	32,5833119363487	32,5833119363487	32,2966368508442	32,574532753597	32,4715364843887	32,3444630888764	128603298218434	125523081712911	0,0349749657404337	0,00509459471878766	0,0035499739200852	0,976048697442493	-0.0349749657404412	1,02453904464016	0.0349749657404412	1995
Q9CPR4	sp|Q9CPR4|RL17_MOUSE 60S ribosomal protein L17 OS=Mus musculus GN=Rpl17 PE=2 SV=3	−1,02	0,893332546469956	0,977069018898103	25,784338153794	26,0346229337524	26,269865312127	26,0415130488842	26,1820526790869	25,7926120679867	201611577564,729	196787951699,74	0,034936593436064	0,00932793167897529	0,0075836931455958	0,97607465839386	-0.0349365934360646	1,02451179466693	0.0349365934360647	1996
P61979-2	sp|P61979-2|HNRPK_MOUSE Isoform 2 of Heterogeneous nuclear ribonucleoprotein K OS=Mus musculus GN=Hnrnpk;sp|P61979|HNRPK_MOUSE Heterogeneous nuclear ribonucleoprotein K OS=Mus musculus GN=Hnrnpk PE=1 SV=1	−1,02	0,884774032718594	0,974991341225953	32,3444630888764	32,8500916619242	32,7488011970875	32,7396907713201	32,5208763535181	32,6101813602848	150922158916688	147313314898093	0,0349168088208754	0,00819419662477141	0,0033724399689787	0,976088044032108	-0.0349168088208819	1,02449774496685	0.0349168088208819	1997
Q3UE31	sp|Q3UE31|K0930_MOUSE Uncharacterized protein KIAA0930 homolog OS=Mus musculus PE=1 SV=2	−1,02	0,89475330663579	0,977357990376388	24,2886600925541	24,7147344118964	24,7860991569549	24,7428706679691	24,4180530212713	24,5565050782037	48097588955,4331	46955973040,315	0,0346558883800319	0,010935406322266	0,0066333051153107	0,976264591637308	-0.0346558883800315	1,02431247488233	0.0346558883800315	1998
Q3UBX0	sp|Q3UBX0|TM109_MOUSE Transmembrane protein 109 OS=Mus musculus GN=Tmem109 PE=1 SV=2	−1,02	0,890747019940063	0,976830327013275	27,1464541510055	27,0846207130066	26,9501760086428	26,7306224117162	27,1124388235916	27,268187615531	565183874958,078	552148544998,36	0,0336638565754098	0,00370846453199667	0,0102300865542151	0,976936125503077	-0.03366385657541	1,02360837509724	0.0336638565754101	1999
P58064	sp|P58064|RT06_MOUSE 28S ribosomal protein S6, mitochondrial OS=Mus musculus GN=Mrps6 PE=2 SV=3	−1,02	0,884477341009055	0,974901080825668	25,6734309040907	25,6867261672998	25,5463841594127	25,3098334236589	25,7162865377133	25,810667197616	135944662106,375	132820228619,333	0,0335446178297684	0,00302214381041193	0,0103906679077687	0,977016872611025	-0.0335446178297679	1,02352377736073	0.0335446178297678	2000
Q6PIE5	sp|Q6PIE5|AT1A2_MOUSE Sodium/potassium-transporting ATPase subunit alpha-2 OS=Mus musculus GN=Atp1a2 PE=1 SV=1	−1,02	0,902689480724463	0,977644369603516	31,1032923983988	30,7195270753918	30,4899709322161	30,7404493890883	30,8553810226389	30,6477397566522	23101764950833,6	22574830858836,1	0,0332878978513343	0,0100703810841769	0,0033829554502338	0,977190743083096	-0.0332878978513367	1,02334166290292	0.0332878978513368	2001
Q9D853	sp|Q9D853|MET10_MOUSE Methyltransferase-like protein 10 OS=Mus musculus GN=Mettl10 PE=2 SV=1	−1,02	0,90491986284421	0,979225550630981	24,1331519049364	24,5156871626391	24,573149001652	24,6092541358523	24,3990475359719	24,1446543239673	39807825734,7342	38902278738,9995	0,0331974100028574	0,00979937403918507	0,0095409615328348	0,977252035773846	-0.0331974100028572	1,02327747949703	0.0331974100028574	2002
Q9D1A2	sp|Q9D1A2|CNDP2_MOUSE Cytosolic non-specific dipeptidase OS=Mus musculus GN=Cndp2 PE=1 SV=1	−1,02	0,717248288278506	0,919756927921409	30,7820965217206	30,5694328473646	30,6680635283742	30,6442031737676	30,6302391026061	30,6767393272954	20950793395498,5	20478442591875,9	0,0328988780973382	0,00346963359039S3	0,0007784645155002	0,977454276088461	-0.0328988780973394	1,02306575812606	0.0328988780973394	2003
Q9Z1D1	sp|Q9Z1D1|EIF3G_MOUSE Eukaryotic translation initiation factor 3 subunit G OS=Mus musculus GN=Eif3g PE=1 SV=2;sp|O75821|EIF3G_HUMAN Eukaryotic translation initiation factor 3 subunit G OS=Homo sapiens GN=EIF3G PE=1 SV=2	−1,02	0,908631180662976	0,980429909122681	28,7651471605025	28,0948693999158	28,3456414045074	28,3855524287391	28,429387167353	28,3225587196079	2161634983955,19	2113076599055,91	0,0327778626422259	0,0119238593425839	0,0018922260234326	0,977536269879186	-0.0327778626422259	1,02297994541276	0.0327778626422259	2004
Q9QYY8	sp|Q9QYY8|SPAST_MOUSE Spastin OS=Mus musculus GN=Spast PE=1 SV=3	−1,02	0,935496583120778	0,98517137107117	25,286634641881	25,5382714274755	25,4695074078681	25,914114289794	25,3172002676571	24,9952475903761	110853035787,001	108374000486,761	0,032629592146454	0,00511409533064308	0,018349491301506	0,977636739646866	-0.0326295921464562	1,02287481581473	0.0326295921464562	2005
Q5M8N0	sp|Q5M8N0|CNRP1_MOUSE CB1 cannabinoid receptor-interacting protein 1 OS=Mus musculus GN=Cnrip1 PE=1 SV=1	−1,02	0,908340893509272	0,980429909122681	31,4680190706971	31,3267661620555	30,9950183754577	31,0040688696121	31,2428831501822	31,4753765257957	37797643275575,6	36956999677988,4	0,0324486460752904	0,0077666055963281	0,0075433750740800	0,977759364745092	-0.0324486460752905	1,02274653258955	0.0324486460752904	2006
Q8R0P4	sp|Q8R0P4|AAMDC_MOUSE Mth938 domain-containing protein OS=Mus musculus GN=Aamdc PE=1 SV=1;sp|Q8R0P4-2|AAMDC_MOUSE Isoform 2 of Mth938 domain-containing protein OS=Mus musculus GN=Aamdc	−1,02	0,898496860485477	0,977644369603516	26,3744753513748	26,2127010125378	26,0524841852733	25,9259085166726	26,2113399560879	26,4356893840667	242246147127,892	236917848125,571	0,0320868324602026	0,00614179447425421	0,0097552522767798	0,978004607852409	-0.0320868324602016	1,02249007005794	0.0320868324602014	2007
P35614	sp|P35614|ERF1Z_ARATH Eukaryotic peptide chain release factor subunit 1-3 OS=Arabidopsis thaliana GN=ERF1-3 PE=1 SV=1	−1,02	0,817713203870663	0,952267950351507	26,8410458836077	26,6055017936493	26,8916424786853	26,7207981765655	26,8348651408146	26,7159035528554	426721203892,884	417349132539,773	0,0320390279656003	0,00570242580695779	0,0025157370415426	0,978037015110541	-0.0320390279656022	1,02245618984776	0.0320390279656022	2008
Q9CWJ9	sp|Q9CWJ9|PUR9_MOUSE Bifunctional purine biosynthesis protein PURH OS=Mus musculus GN=Atic PE=1 SV=2	−1,02	0,904133428105994	0,978741147191297	30,2664339291469	30,7758103446925	30,7710464061561	30,7235744445341	30,5433566106407	30,4798415039778	19558483105123,2	19129591011415,5	0,0319884543564436	0,00956472868151755	0,0041343358906860	0,97807130075464	-0.0319884543564428	1,02242034832066	0.0319884543564427	2009
P15532	sp|P15532|NDKA_MOUSE Nucleoside diphosphate kinase A OS=Mus musculus GN=Nme1 PE=1 SV=1	−1,02	0,875243996627946	0,972992598310849	33,1187251555991	33,0667878409893	33,0157551936444	33,3079535850214	32,8500916619242	32,9774514497747	229537928310585	224560340790547	0,0316294025074753	0,00155700559861593	0,0071514975368650	0,978314749302334	-0.031629402507477	1,02216592432357	0.0316294025074769	2010

P63034-2	sp|P63034-2|CYH2_MOUSE Isoform 2 of Cytohesin-2 OS=Mus musculus GN=Cyth2;sp|P63034|CYH2_MOUSE Cytohesin-2 OS=Mus musculus GN=Cyth2 PE=1 SV=2;sp|P63034-3|CYH2_MOUSE Isoform 3 of Cytohesin-2 OS=Mus musculus GN=Cyth2	−1,02	0,900762318579654	0,977644369603516	28,8941100043926	28,3414907470162	28,7651471605025	28,5539886874427	28,7194223068834	28,6623100861355	2817626474149,64	2757209849276,38	0,0312712957523945	0,01008511129629	0,0029333805175693	0,978557617403317	-0.0312712957523928	1,02191223308197	0.0312712957523929	2011
Q9JK23	sp|Q9JK23|PSMG1_MOUSE Proteasome assembly chaperone 1 OS=Mus musculus GN=Psmg1 PE=2 SV=1	−1,02	0,897280738353719	0,977644369603516	26,5473065949047	26,587342146856	27,0207057287637	26,8329937693396	26,6649401997547	26,5925627340672	401491215212,502	392904428352,269	0,0311899931125502	0,00982557507878752	0,0046203034041102	0,9861276527386	-0.0311899931125476	1,02185464515186	0.0311899931125477	2012
P56959	sp|P56959|FUS_MOUSE RNA-binding protein FUS OS=Mus musculus GN=Fus PE=2 SV=1	−1,02	0,914642766851021	0,980665300581806	30,279068162713	30,8437717553966	30,8001322897362	30,7820965217206	30,507269860691	30,5694328473646	20286783603800	19857437603961,6	0,0308606790733625	0,0102539998871539	0,0047067197086449	0,978836171951971	-0.0308606790733589	1,02162142006443	0.0308606790733587	2013
Q91WC3	sp|Q91WC3|ACSL6_MOUSE Long-chain-fatty-acid--CoA ligase 6 OS=Mus musculus GN=Acsl6 PE=2 SV=1;sp|Q91WC3-2|ACSL6_MOUSE Isoform 2 of Long-chain-fatty-acid--CoA ligase 6 OS=Mus musculus GN=Acsl6	−1,02	0,87617740292163	0,973077864414309	31,3988833526896	31,2898972692622	30,9727064202915	31,271198233726	31,2206366567217	31,1056636057856	36215042269156	35450772694502,3	0,0307719860007801	0,00709157996906609	0,0027189896970977	0,978896350058809	-0.0307719860007796	1,02155861541411	0.0307719860007795	2014
Q9CWZ7	sp|Q9CWZ7|SNAG_MOUSE Gamma-soluble NSF attachment protein OS=Mus musculus GN=Napg PE=1 SV=1	−1,02	0,84907641260467	0,96228660095196	31,687944408101	31,3069394690914	31,471637043656	31,4444699733367	31,4546926223212	31,5035229610434	47361951293279,3	46364808608135,3	0,030698321096052	0,00606831357461946	0,0010029340694212	0,97894633439046	-0.0306983210960514	1,02150645532847	0.0306983210960514	2015
A6H611	sp|A6H611|MIPEP_MOUSE Mitochondrial intermediate peptidase OS=Mus musculus GN=Mipep PE=2 SV=1	−1,02	0,911017331766501	0,980429909122681	24,9998552831891	24,9546627427534	24,6738015764935	25,0631252435222	24,5401576917216	24,961398664519	63614453269,8645	62279232881,7477	0,0306034102895737	0,00710128732234786	0,011155735726389	0,979010738606012	-0.0306034102895745	1,02143925553245	0.0306034102895746	2016
Q8BI08	sp|Q8BI08|MAL2_MOUSE Protein MAL2 OS=Mus musculus GN=Mal2 PE=1 SV=1	−1,02	0,939745502539831	0,986144884061661	29,643927476549	29,5228299333287	29,323260678621	28,9686502897513	29,6015013481049	29,8563790555124	6460143816983,75	6324867686057,17	0,0305309833710374	0,00548962776485173	0,0155070656113734	0,979059888640414	-0.0305309833710365	1,02138797800068	0.0305309833710365	2017
Q9JIG7	sp|Q9JIG7|CCD22_MOUSE Coiled-coil domain-containing protein 22 OS=Mus musculus GN=Ccdc22 PE=1 SV=1	−1,02	0,910771911983563	0,980429909122681	28,0423253078252	27,8881308498074	27,4410098735373	27,8216158205759	27,8242150678396	27,6623996166401	1172886214497,96	1148422259834,98	0,0304098599777654	0,01123847970788	0,0033375922630083	0,979142090374511	-0.0304098599777654	1,02130222960542	0.0304098599777654	2018
Q920M7	sp|Q920M7|SYT17_MOUSE Synaptotagmin-17 OS=Mus musculus GN=Syt17 PE=2 SV=1	−1,02	0,912237555074406	0,980665300581806	26,0494943656724	26,5989713295845	26,6015089080294	26,3911397781081	26,308372107962	26,4873971485829	296899104069,768	290722879098,747	0,0303281277060135	0,0120369403950714	0,0033943938385548	0,979197562786954	-0.0303281277060123	1,02124437192617	0.0303281277060122	2019
Q9CR95	sp|Q9CR95|NECP1_MOUSE Adaptin ear-binding coat-associated protein 1 OS=Mus musculus GN=Necap1 PE=1 SV=2	−1,02	0,92651947815563	0,983810446072053	30,688685623596	30,4517424809733	30,2298776072897	30,3912180432188	30,1415815593921	30,7747464720785	16873552489697,6	16524226481149,4	0,0301810057708858	0,00753347480918744	0,0104788659699028	0,979297423659808	-0.0301810057708862	1,02114023364099	0.0301810057708861	2020
Q8BGT8	sp|Q8BGT8|PHIPL_MOUSE Phytanoyl-CoA hydroxylase-interacting protein-like OS=Mus musculus GN=Phyhipl PE=2 SV=1;sp|Q8BGT8-2|PHIPL_MOUSE Isoform 2 of Phytanoyl-CoA hydroxylase-interacting protein-like OS=Mus musculus GN=Phyhipl	−1,02	0,807677096424717	0,94879038498679	31,2206366567217	30,9837659274804	31,1607157709667	31,0545441670067	31,1939924779762	31,0545441670067	32808413854902,8	32136929526173,2	0,0298337519644562	0,00395734894484764	0,0025886772175875	0,979533166958352	-0.0298337519644545	1,02089447681001	0.0298337519644547	2021
Q62425	sp|Q62425|NDUA4_MOUSE Cytochrome c oxidase subunit NDUFA4 OS=Mus musculus GN=Ndufa4 PE=1 SV=2	−1,02	0,918564346472237	0,981488599020124	28,8343162844237	28,5119947056563	28,3076758523563	28,3588682131998	28,787551864115	28,4457488486355	2510062947589,67	2458869912884,17	0,029728133850801	0,0092995478560932	0,0079431126907888	0,979604880126748	-0.0297281338508031	1,0208197409864	0.0297281338508032	2022
P08113	sp|P08113|ENPL_MOUSE Endoplasmin OS=Mus musculus GN=Hsp90b1 PE=1 SV=2	−1,02	0,854125194891505	0,963607051252994	33,037004306747	32,9459102626137	32,6953467176655	32,8606940502048	32,9567183360255	32,799089045904	192815282750589	188886447592874	0,0297002121261496	0,00537877824430868	0,0024165878743119	0,979623839450543	-0.0297002121261518	1,02079998437042	0.0297002121261518	2023
Q8C0J6	sp|Q8C0J6|SWAHC_MOUSE Ankyrin repeat domain-containing protein SOWAHC OS=Mus musculus GN=Sowahc PE=1 SV=2	−1,02	0,902162300362741	0,977644369603516	22,6191277512212	23,1523256748072	23,0384874615479	22,993646883691	22,8687955606518	22,8859967988134	9146589939,1639	8960988794,60727	0,0295760391371133	0,012242803891602	0,0029527733246173	0,979708159457119	-0.0295760391371141	1,02071212773621	0.0295760391371142	2024
Q3THK7	sp|Q3THK7|GUAA_MOUSE GMP synthase [glutamine-hydrolyzing] OS=Mus musculus GN=Gmps PE=1 SV=2	−1,02	0,927239719827329	0,983810446072053	30,7592274156069	30,6061730946971	30,1150222657986	30,6680635283742	30,4517424809733	30,2998683979432	17504415139112,4	17153524908253,7	0,0292137901423611	0,0110372717728673	0,0060720589005650	0,979954187096794	-0.0292137901423638	1,02045586739375	0.029213790142364	2025
Q11011	sp|Q11011|PSA_MOUSE Puromycin-sensitive aminopeptidase OS=Mus musculus GN=Npepps PE=1 SV=2	−1,02	0,886789049975739	0,975149870380105	33,1743276583604	32,9567183360255	32,7396907713201	32,9720957537316	33,0286081008433	32,8095502245621	205591489513367	201488084125344	0,0290860239909618	0,00659401908549355	0,0034528475638656	0,980040976415241	-0.0290860239909578	1,02036549906083	0.0290860239909577	2026
Q9JLJ2	sp|Q9JLJ2|AL9A1_MOUSE 4-trimethylaminobutyraldehyde dehydrogenase OS=Mus musculus GN=Aldh9a1 PE=1 SV=1	−1,02	0,842202479659037	0,959771286845933	29,8687792032057	29,5620727820686	29,6389646388719	29,6309744420806	29,7525593270964	29,6259603347948	7837483171556,15	7681464753314,6	0,0290090002364826	0,00537490695713737	0,0024162040051439	0,980093301021969	-0.0290090002364836	1,02031102442724	0.0290090002364835	2027
Q3UMC0	sp|Q3UMC0|SPAT5_MOUSE Spermatogenesis-associated protein 5 OS=Mus musculus GN=Spata5 PE=1 SV=2;sp|Q9D3R6-3|KATL2_MOUSE Isoform 3 of Katanin p60 ATPase-containing subunit A-like 2 OS=Mus musculus GN=Katnal2;sp|Q9D3R6|KATL2_MOUSE Katanin p60 ATPase-contai	−1,02	0,882875482006974	0,973844911987766	29,5469002992119	29,3963002344319	29,1319348639858	29,2251086014483	29,4384942443743	29,351342606842	5625767312778,9	5514020525120,57	0,0289452450374892	0,00715522351481708	0,0036569131297382	0,980136614003836	-0.0289452450374873	1,02026593610764	0.0289452450374873	2028
Q80UM3	sp|Q80UM3|NAA15_MOUSE N-alpha-acetyltransferase 15, NatA auxiliary subunit OS=Mus musculus GN=Naa15 PE=1 SV=1	−1,02	0,900183178570773	0,977644369603516	29,2370422808287	28,9252452807151	28,7164765416841	28,8875389662165	29,0435033047516	28,8875389662165	3775628724919,85	3700640686350,22	0,0289418407957119	0,00904628211948798	0,0031115246835653	0,980138926776699	-0.0289418407957146	1,02026352864957	0.0289418407957145	2029

Q8K114	sp|Q8K114|INT9_MOUSE Integrator complex subunit 9 OS=Mus musculus GN=Ints9 PE=2 SV=1;sp|Q8K114-2|INT9_MOUSE Isoform 2 of Integrator complex subunit 9 OS=Mus musculus GN=Ints9	−1,02	0,938306554920691	0,985646114383563	22,8329525082737	22,4285357176485	22,1958548520544	22,7826561503887	22,1674770243792	22,4474378958212	5827075173,94093	5712125445,72827	0,028744259547307	0,0143372162853311	0,0137098220378819	0,98027316882289	-0.0287442595473071	1,02012381018323	0.0287442595473073	2030
O35639	sp|O35639|ANXA3_MOUSE Annexin A3 OS=Mus musculus GN=Anxa3 PE=1 SV=4	−1,02	0,920508109811711	0,981888180745783	29,1996198193394	28,5655362258062	29,034879459619	28,9668367465018	28,8185754978522	28,9559879547382	3677834222094,57	3606648509617,31	0,028197621571571	0,01371299785602	0,0028583272328492	0,980644665262612	-0.0281976215715684	1,0197373579065	0.028197621571568	2031
Q8VE62	sp|Q8VE62|PAIP1_MOUSE Polyadenylate-binding protein-interacting protein 1 OS=Mus musculus GN=Paip1 PE=2 SV=1	−1,02	0,898204051004871	0,977644369603516	27,6889296233201	27,4606749995793	27,5509487648623	27,7146979344159	27,6503089516748	27,2773560240938	937845564731,34	919829628470,106	0,0279837044758701	0,0041700533545478	0,0085712864828441	0,980790082142794	-0.0279837044758696	1,01958616650694	0.0279837044758695	2032
Q62074	sp|Q62074|KPCI_MOUSE Protein kinase C iota type OS=Mus musculus GN=Prkci PE=1 SV=3	−1,02	0,971876121431942	0,993288144763853	23,0567184762801	23,164049627614	22,8039538529234	24,0516859730864	22,3388119487222	22,5764449156736	9825438770,42994	9638014367,0192	0,0277858829773353	0,00803528806753125	0,040365755521392	0,980924576724777	-0.0277858829773352	1,01944637103386	0.0277858829773353	2033
Q9QZN1-2	sp|Q9QZN1-2|FXL17_MOUSE Isoform 2 of F-box/LRR-repeat protein 17 OS=Mus musculus GN=Fbxl17;sp|Q9QZN1|FXL17_MOUSE F-box/LRR-repeat protein 17 OS=Mus musculus GN=Fbxl17 PE=2 SV=3	−1,02	0,975627834533993	0,994649943013952	20,8934801258364	22,4365116962145	22,2553767996608	21,1029986109079	22,4030699429728	22,0223540321112	3122155734,02356	3063449877,05894	0,0273852544437078	0,0385813363567649	0,0305992809605211	0,981197011947586	-0.0273852544437055	1,01916331564758	0.0273852544437056	2034
P62827	sp|P62827|RAN_MOUSE GTP-binding nuclear protein Ran OS=Mus musculus GN=Ran PE=1 SV=3	−1,02	0,950895180415075	0,988522869262536	31,2898972692622	31,8397012538833	32,1601216416315	32,043087513796	31,4594116268809	31,7308789356197	62316045138420,1	61156627839194,9	0,0270948172146953	0,0138563311504411	0,0092008128545503	0,981394562240755	-0.0270948172146956	1,01895816267492	0.0270948172146956	2035
Q9JI46	sp|Q9JI46|NUDT3_MOUSE Diphosphoinositol polyphosphate phosphohydrolase 1 OS=Mus musculus GN=Nudt3 PE=1 SV=1	−1,02	0,896115285399151	0,977357990376388	30,0893751498851	30,0134696913558	29,8846521691104	29,7220781608845	30,0816756695027	30,1282886884742	10642029634616,7	10447120501650	0,0266679732891492	0,00345030732636669	0,0074154292538718	0,981684966152256	-0.0266679732891447	1,01865673253562	0.0266679732891445	2036
Q8VDN2	sp|Q8VDN2|AT1A1_MOUSE Sodium/potassium-transporting ATPase subunit alpha-1 OS=Mus musculus GN=Atp1a1 PE=1 SV=1	−1,02	0,871320460679817	0,971009960968743	33,3423047078514	33,1052341468871	33,037004306747	33,042314781574	33,268184717111	33,1187251555991	252268317041902	247659239943991	0,0266025786695855	0,00483218507561782	0,0034663908861208	0,981729465071329	-0.0266025786695856	1,01861055981175	0.0266025786695855	2037
Q5SUF2	sp|Q5SUF2|LC7L3_MOUSE Luc7-like protein 3 OS=Mus musculus GN=Luc7l3 PE=1 SV=1;sp|Q5SUF2-3|LC7L3_MOUSE Isoform 3 of Luc7-like protein 3 OS=Mus musculus GN=Luc7l3;sp|Q5SUF2-2|LC7L3_MOUSE Isoform 2 of Luc7-like protein 3 OS=Mus musculus GN=Luc7l3	−1,02	0,943826924594504	0,986987866859505	21,8248730561356	22,7026757793855	22,311282263451	22,1779631888277	22,3849401581905	22,2206266183963	4741457269,96522	4654855266,48907	0,0265942237130559	0,0197381184732136	0,0049091956168574	0,981735150493767	-0.0265942237130557	1,01860466083653	0.0265942237130556	2038
Q9QYI3	sp|Q9QYI3|DNJC7_MOUSE DnaJ homolog subfamily C member 7 OS=Mus musculus GN=Dnajc7 PE=1 SV=2	−1,02	0,91766819323623	0,980761932917176	28,0120626766497	28,5681116849645	28,4357489471046	28,3974533755861	28,3623185321326	28,2008611250849	2029156089129,56	1992101068364,29	0,0265890022907479	0,0102497954011867	0,0037020315164916	0,98173870360995	-0.0265890022907421	1,01860097429479	0.0265890022907419	2039
Q9EQ80	sp|Q9EQ80|GTPC1_MOUSE Putative GTP cyclohydrolase 1 type 2 Nif3l1 OS=Mus musculus GN=Nif3l1 PE=1 SV=4	−1,02	0,942830809136136	0,986987866859505	29,1249791637666	28,3160857868948	28,4788737237828	28,6215713507648	28,7339849515457	28,5094418456896	2742742801673,14	2692970699175,85	0,0264208083482078	0,0149384102526758	0,0039226082328015	0,981853164479393	-0.0264208083482097	1,01848222949939	0.0264208083482097	2040
Q149L6	sp|Q149L6|DJB14_MOUSE DnaJ homolog subfamily B member 14 OS=Mus musculus GN=Dnajb14 PE=2 SV=1;sp|Q149L6-3|DJB14_MOUSE Isoform 3 of DnaJ homolog subfamily B member 14 OS=Mus musculus GN=Dnajb14	−1,02	0,914743885598632	0,980665300581806	24,5268886798457	23,9556768269568	24,2234160190066	24,2775725239655	24,2371377474287	24,1365945678446	33517287091,8093	32911948763,3824	0,0262939281890759	0,0117923783779884	0,0029976030444293	0,981939518948273	-0.0262939281890733	1,01839266136378	0.0262939281890734	2041
O08677-2	sp|O08677-2|KNG1_MOUSE Isoform LMW of Kininogen-1 OS=Mus musculus GN=Kng1;sp|O08677-3|KNG1_MOUSE Isoform 3 of Kininogen-1 OS=Mus musculus GN=Kng1;sp|O08677|KNG1_MOUSE Kininogen-1 OS=Mus musculus GN=Kng1 PE=1 SV=1	−1,02	0,957640483472864	0,990217657408697	24,9340519903573	25,7580916626282	25,8362365598065	25,7127945872499	25,6205393435449	25,1410841300026	119844399912,337	117707984535,038	0,0259503096927843	0,0195945925933996	0,0120405505501487	0,982173423381804	-0.0259503096927853	1,01815013132489	0.0259503096927853	2042
Q9D2N9	sp|Q9D2N9|VP33A_MOUSE Vacuolar protein sorting-associated protein 33A OS=Mus musculus GN=Vps33a PE=1 SV=2;sp|Q96AX1|VP33A_HUMAN Vacuolar protein sorting-associated protein 33A OS=Homo sapiens GN=VPS33A PE=1 SV=1	−1,02	0,940119436616114	0,986144884061661	27,386252220826	27,9018726638348	27,8961549127051	28,0091322344659	27,6736691686645	27,4482593170704	1101939875560,79	1082564168796,68	0,0255929662355682	0,0106771435690541	0,0101850109457455	0,982416729629419	-0.0255929662355681	1,01789797530953	0.0255929662355682	2043
B0V2N1-4	sp|B0V2N1-4|PTPRS_MOUSE Isoform 4 of Receptor-type tyrosine-protein phosphatase S OS=Mus musculus GN=Ptprs;sp|B0V2N1-3|PTPRS_MOUSE Isoform 3 of Receptor-type tyrosine-protein phosphatase S OS=Mus musculus GN=Ptprs;sp|B0V2N1-6|PTPRS_MOUSE Isoform 6 of Re	−1,02	0,910001137553979	0,980429909122681	28,5243734839213	28,2599624963169	27,9865800133104	28,2591635841223	28,2559271319953	28,2028134144342	1870023283616,92	1837269056965,67	0,0254933172846325	0,00951656132670269	0,0011204565548084	0,982484588861429	-0.025493317284633	1,01782767011019	0.0254933172846331	2044
P09671	sp|P09671|SODM_MOUSE Superoxide dismutase [Mn], mitochondrial OS=Mus musculus GN=Sod2 PE=1 SV=3	−1,02	0,908936723161801	0,980429909122681	32,4866102924521	32,1406176218661	32,0567659011701	32,0567659011701	32,2466587951576	32,3276858928127	99184011069728,3	97450940835375,2	0,0254314561327584	0,00707010424148122	0,0043171732307398	0,982526717606382	-0.0254314561327597	1,01778402773228	0.0254314561327597	2045

Q8R332-4	sp|Q8R332-4|NUPL1_MOUSE Isoform 4 of Nucleoporin p58/p45 OS=Mus musculus GN=Nupl1;sp|Q8R332-3|NUPL1_MOUSE Isoform 3 of Nucleoporin p58/p45 OS=Mus musculus GN=Nupl1;sp|Q8R332-2|NUPL1_MOUSE Isoform 2 of Nucleoporin p58/p45 OS=Mus musculus GN=Nupl1;sp|Q8R	−1,02	0,902083017540902	0,977644369603516	23,93947115125	24,4081168167823	24,0697373811504	24,1834776243377	24,0935577863108	24,0874845308129	30442589938,9102	29911433846,952	0,0253940332838667	0,0100211544398073	0,0022284793724843	0,98255204230189		-0.0253940332838662	1,01775762722295	0.0253940332838661	2046
Q8BNY6	sp|Q8BNY6|NCS1_MOUSE Neuronal calcium sensor 1 OS=Mus musculus GN=Ncs1 PE=2 SV=3	−1,02	0,908140691399792	0,980429909122681	28,9044917431211	28,7572558211707	28,5119947056563	28,5307522510992	28,7253092782756	28,8657160830252	2984880285621,29	2933622736749,08	0,0249897179153437	0,0069027344726977	0,0058594875349318	0,982827603130642		-0.0249897179153429	1,01747244055281	0.0249897179153431	2047
P32067	sp|P32067|LA_MOUSE Lupus La protein homolog OS=Mus musculus GN=Ssb PE=1 SV=1	−1,02	0,915035233076589	0,980665300581806	29,3677682146643	28,9439646135663	28,8597896366355	29,0359313240441	28,995304264796	29,089228896999	4162654780113,12	4092408666328,39	0,0245536977133588	0,0093696356928325	0,0016220484918666	0,983124684247099		-0.0245536977133592	1,0171649802139	0.0245536977133591	2048
Q61923	sp|Q61923|KCNA6_MOUSE Potassium voltage-gated channel subfamily A member 6 OS=Mus musculus GN=Kcna6 PE=2 SV=1	−1,02	0,94538659400776	0,987138173908166	23,6663250302842	23,5046582728801	24,2952278431904	23,8330236397602	23,8612171319506	23,7216479337666	22171430488,7698	21802625497,5945	0,0241999786330321	0,017532614160774 9	0,0031001666777849	0,983365755702497		-0.024199978633033	1,01691562290129	0.0241999786330331	2049
Q8R2Q4-3	sp|Q8R2Q4-3|RRF2M_MOUSE Isoform 3 of Ribosome-releasing factor 2, mitochondrial OS=Mus musculus GN=Gfm2;sp|Q8R2Q4-2|RRF2M_MOUSE Isoform 2 of Ribosome-releasing factor 2, mitochondrial OS=Mus musculus GN=Gfm2;sp|Q8R2Q4|RRF2M_MOUSE Ribosome-releasing fact	−1,02	0,943933538232455	0,986987866859505	25,7564189171455	25,030906042807 1	25,2652188489382	25,1761940416215	25,3191904935594	25,5077187593753	102266494708,024	100594936953,554	0,0237758616165987	0,0146053764469223	0,0065635210582303	0,98365488365234		-0.0237758616165877	1,0166167185456	0.0237758616165877	2050
P63001	sp|P63001|RAC1_MOUSE Ras-related C3 botulinum toxin substrate 1 OS=Mus musculus GN=Rac1 PE=1 SV=1	−1,02	0,841793701304151	0,959771286845933	31,849738476019	31,9210716119724	31,9095496846577	31,7308789356197	32,0014621620972	31,8989697166028	70982412963725,9	69831310860137,5	0,0235875629809045	0,0012006831800695 3	0,0042855352936115	0,983783277356651		-0.0235875629809055	1,01648403974392	0.0235875629809055	2051
O35682	sp|O35682|MYADM_MOUSE Myeloid-associated differentiation marker OS=Mus musculus GN=Myadm PE=2 SV=2	−1,02	0,964046759167795	0,991306002794532	23,1309610408383	23,1374797714001	22,3570874543215	22,6504567909222	22,5849545096597	23,3421578262587	8601273312,62163	8464863345,65921	0,0230634710127477	0,01961470179051 6	0,0183533606314162	0,984140724052769		-0.0230634710127466	1,01611484573255	0.0230634710127465	2052
Q7TN98-4	sp|Q7TN98-4|CPEB4_MOUSE Isoform 4 of Cytoplasmic polyadenylation element-binding protein 4 OS=Mus musculus GN=Cpeb4;sp|Q7TN98-5|CPEB4_MOUSE Isoform 5 of Cytoplasmic polyadenylation element-binding protein 4 OS=Mus musculus GN=Cpeb4;sp|Q7TN98-3|CPEB4_MOU	−1,02	0,948176481413629	0,988237209905042	22,9811816841146	22,3165440216705	22,3355549008786	22,5167234850316	22,7297871435156	22,3388119487222	6179028336,11004	6081035671,28348	0,0230629370592881	0,016782869998849 4	0,0086890519982845	0,984141088291521		-0.0230629370592878	1,01611446966005	0.0230629370592877	2053
Q61553	sp|Q61553|FSCN1_MOUSE Fascin OS=Mus musculus GN=Fscn1 PE=1 SV=4	−1,02	0,940520203371432	0,986144884061661	33,192156642203 8	33,7277908946237	33,7804120503292	33,7181235415771	33,5541074777048	33,3807460089899	378329463712988	372400998564299	0,0227861942426683	0,0096972072864700 1	0,0050284753312005	0,984329887790113		-0.0227861942426694	1,01591957371636	0.0227861942426696	2054
Q80WJ7	sp|Q80WJ7|LYRIC_MOUSE Protein LYRIC OS=Mus musculus GN=Mtdh PE=1 SV=1	−1,02	0,95500113254646	0,989938667705093	27,0036061767615	27,1916972274608	26,4253543694505	26,6815515217131	27,1570050725175	26,7355071353139	468851812770,865	461626169326,396	0,0224069987995849	0,0148615172873877	0,0096927920244473	0,984588641341139		-0.0224069987995836	1,01565258627996	0.0224069987995836	2055
Q924M7	sp|Q924M7|MPI_MOUSE Mannose-6-phosphate isomerase OS=Mus musculus GN=Mpi PE=2 SV=1	−1,02	0,927049917397744	0,983810446072053	29,57918938533	29,3445457208605	29,1893852573842	29,2467701061378	29,2130685094284	29,6067416513827	5697451703211,11	5609747107946,31	0,0223810555348368	0,0066816838701826 1	0,0074333562231610	0,984606346866377		-0.0223810555348371	1,01563432247072	0.0223810555348371	2056
Q8VDM6-2	sp|Q8VDM6-2|HNRL1_MOUSE Isoform 2 of Heterogeneous nuclear ribonucleoprotein U-like protein 1 OS=Mus musculus GN=Hnrnpul1;sp|Q8VDM6|HNRL1_MOUSE Heterogeneous nuclear ribonucleoprotein U-like protein 1 OS=Mus musculus GN=Hnrnpul1 PE=1 SV=1;sp|Q8VDM6-3|HN	−1,02	0,893260546608813	0,977069018898103	26,3346641672483	26,3143352941035	26,0031584198129	26,1321115412	26,269865312127	26,2041255291928	243257395490,302	239551467782,698	0,022148013166829	0,0070870804351548 8	0,0026295933754866	0,98476540579523		-0.0221480131668292	1,01547027760634	0.0221480131668293	2057
Q9DB50	sp|Q9DB50|AP1S2_MOUSE AP-1 complex subunit sigma-2 OS=Mus musculus GN=Ap1s2 PE=2 SV=1	−1,02	0,930348779448539	0,98433518830764	24,6333618185744	25,1917952891573	25,0503231987898	24,8783295174502	25,0243761876188	24,9274050418198	69077398861,074	68040588173,5642	0,0218181462298352	0,01163216838757 3	0,0029795791692560	0,984990594541711		-0.0218181462298359	1,01523812058863	0.0218181462298358	2058
Q9CQS8	sp|Q9CQS8|SC61B_MOUSE Protein transport protein Sec61 subunit beta OS=Mus musculus GN=Sec61b PE=1 SV=3	−1,02	0,950400187178809	0,988334326693467	25,9613397477305	25,549542709295	25,4189974157507	25,272388648783	25,773705472882	25,8385679589886	137006374504,963	134956817460,346	0,0217451614850537	0,0110385508952629	0,0120906345523947	0,98504042565886		-0.0217451614850563	1,01518676183379	0.0217451614850562	2059
Q7TPR4	sp|Q7TPR4|ACTN1_MOUSE Alpha-actinin-1 OS=Mus musculus GN=Actn1 PE=1 SV=1	−1,01	0,956128956640857	0,990217657408697	32,7488011970875	32,0501774277855	32,2621420111148	32,3027531536818	32,6569761311727	32,0567659011701	112469914896020	110809289687803	0,0214603051197581	0,0110713590836118	0,0093301836982883	0,985234938518872		-0.0214603051197572	1,01498633564835	0.0214603051197571	2060
Q9R0X4	sp|Q9R0X4|ACOT9_MOUSE Acyl-coenzyme A thioesterase 9, mitochondrial OS=Mus musculus GN=Acot9 PE=1 SV=1;sp|Q32MW3|ACO10_MOUSE Acyl-coenzyme A thioesterase 10, mitochondrial OS=Mus musculus GN=Acot10 PE=2 SV=1	−1,01	0,894626276998444	0,977357990376388	29,7759040277786	29,4746082875131	29,5177234927931	29,6323404854074	29,6347178699823	29,457092021959 1	7087912157521,15	6984515843207,97	0,0212006107660938	0,0055066748047174 5	0,0034445967941142	0,985412303085124		-0.0212006107660941	1,0148036480458	0.0212006107660941	2061
P28271	sp|P28271|ACOC_MOUSE Cytoplasmic aconitate hydratase OS=Mus musculus GN=Aco1 PE=1 SV=3	−1,01	0,93040549881252	0,98433518830764	30,6260640465842	30,2860766360239	30,0859791046874	30,2434645802855	30,4157949618138	30,295446665439	14904832360034,8	14690694800523,3	0,0208775187410515	0,0090016772908514 9	0,0029156203750625	0,985633011204763		-0.0208775187410559	1,01457640788398	0.0208775187410559	2062

Q9D8P4-2	sp|Q9D8P4-2|RM17_MOUSE Isoform 2 of 39S ribosomal protein L17, mitochondrial OS=Mus musculus GN=Mrpl17;sp|Q9D8P4|RM17_MOUSE 39S ribosomal protein L17, mitochondrial OS=Mus musculus GN=Mrpl17 PE=1 SV=1	−1,01	0,961511546168304	0,991306002794532	24,1126737547988	23,7401078459667	23,4769036420011	23,351891275967	23,7711154655879	24,163506496647	21185053525,3248	20882369585,1495	0,0207613456302799	0,0134354522576051	0,0170809952843537	0,98571238256191	-0.0207613456302777	1,01449471234292	0.0207613456302777	2063
Q9D9H8	sp|Q9D9H8|CB069_MOUSE UPF0565 protein C2orf69 homolog OS=Mus musculus PE=2 SV=3	−1,01	0,95835499947145	0,990431088934634	24,2991722917027	24,4006943482069	25,0688342117586	24,3237320068627	24,7331922094988	24,6690327146511	47765380205,5489	47089645686,8637	0,0205554807860328	0,0170051838951955	0,0089614170318929	0,985853048467796	-0.0205554807860336	1,01434995971681	0.0205554807860334	2064
Q9EQH3	sp|Q9EQH3|VPS35_MOUSE Vacuolar protein sorting-associated protein 35 OS=Mus musculus GN=Vps35 PE=1 SV=1;sp|Q96QK1|VPS35_HUMAN Vacuolar protein sorting-associated protein 35 OS=Homo sapiens GN=VPS35 PE=1 SV=2	−1,01	0,933976619783663	0,98467134375593	32,704254865978	32,4429895051478	32,1406176218661	32,4366499180038	32,4366499180038	32,3737547971211	121299927113126	119661121676456	0,0196241919021134	0,00869795059681902	0,0011202143378842	0,986489642032995	-0.0196241919021204	1,01369538755538	0.0196241919021204	2065
O55131	sp|O55131|SEPT7_MOUSE Septin-7 OS=Mus musculus GN=Sept7 PE=1 SV=1	−1,01	0,872512387125923	0,971622601855879	33,8014606732477	33,597564845353	33,5634667763458	33,6211455554601	33,7181235415771	33,5825229115311	412874214991607	407310673683379	0,0195727004402599	0,00382409897230623	0,0020767090114133	0,986524851622567	-0.0195727004402616	1,01365920823512	0.0195727004402615	2066
Q9WUR2-2	sp|Q9WUR2-2|ECI2_MOUSE Isoform 2 of Enoyl-CoA delta isomerase 2, mitochondrial OS=Mus musculus GN=Eci2;sp|Q9WUR2|ECI2_MOUSE Enoyl-CoA delta isomerase 2, mitochondrial OS=Mus musculus GN=Eci2 PE=1 SV=2	−1,01	0,934053103495416	0,98467134375593	28,1195541428091	27,596320761955	27,7557362941429	27,7288300481742	27,8539827882304	27,8490417699696	1212700006095,66	1196735084884,86	0,0191188796299097	0,00963878622000632	0,0025484399527531	0,986835226246762	-0.0191188796299011	1,01334039706234	0.0191188796299012	2067
O35226-2	sp|O35226-2|PSMD4_MOUSE Isoform Rpn10B of 26S proteasome non-ATPase regulatory subunit 4 OS=Mus musculus GN=Psmd4;sp|O35226-3| PSMD4_MOUSE Isoform Rpn10C of 26S proteasome non-ATPase regulatory subunit 4 OS=Mus musculus GN=Psmd4;sp|O35226-5|PSMD4_MOUSE I	−1,01	0,909032619843201	0,980429909122681	29,7040018754618	29,5156147134624	29,5072210768158	29,383338090787	29,7066894223823	29,5975087341519	6990775115649,06	6899789932067,31	0,0188999871509555	0,00376213980882925	0,0055642067846926	0,986984964889219	-0.0188999871509548	1,01318665995306	0.0188999871509548	2068
Q9D662	sp|Q9D662|SC23B_MOUSE Protein transport protein Sec23B OS=Mus musculus GN=Sec23b PE=1 SV=1	−1,01	0,930890705234603	0,98433518830764	25,6220992401053	25,2783415939017	25,1330874985912	25,4127860971458	25,2907819321046	25,2907819321046	101620329359,838	100301851074,048	0,0188408139675467	0,00990869287488376	0,0027806989953052	0,987025447623563	-0.0188408139675527	1,01314510421963	0.0188408139675527	2069
Q64674	sp|Q64674|SPEE_MOUSE Spermidine synthase OS=Mus musculus GN=Srm PE=2 SV=1	−1,01	0,903485652862174	0,978273229566175	29,0435033047516	29,1918467273542	29,3914357617658	29,2657804305218	29,1488133739494	29,1733008248421	4844807350573,54	4782405968255,81	0,0187026967475216	0,00597742143999067	0,0021129386271619	0,987119945582492	-0.01870269674752	1,01304811484678	0.01870269674752	2070
Q8VEE1	sp|Q8VEE1|LMCD1_MOUSE LIM and cysteine-rich domains protein 1 OS=Mus musculus GN=Lmcd1 PE=1 SV=1	−1,01	0,905165274259595	0,979225550630981	26,3667324253046	26,014799565769	26,0981905549866	26,1892833237637	26,140845402906	26,1107739160253	229669582547,289	226716811132,222	0,0186684273575747	0,00703001927667168	0,0015149419564574	0,987143393642652	-0.0186684273575755	1,01302405146015	0.0186684273575757	2071
Q03958	sp|Q03958|PFD6_MOUSE Prefoldin subunit 6 OS=Mus musculus GN=Pfdn6 PE=2 SV=1	−1,01	0,940016670221081	0,986144884061661	24,4506102071283	24,1659420657239	24,343352667187	24,1698893423587	24,1495233367792	24,602257940586	36477749661,7642	36015798987,9605	0,0183868214369141	0,00591150239292048	0,0105199231022714	0,987336097262384	-0.0183868214369142	1,01282633418623	0.0183868214369144	2072
P12368	sp|P12368|KAP2_RAT cAMP-dependent protein kinase type II-alpha regulatory subunit OS=Rattus norvegicus GN=Prkar2a PE=1 SV=3	−1,01	0,943455286900119	0,986987866859505	30,7552693729942	30,2654579925308	30,4856864089267	30,3514194051073	30,7077418394737	30,4090370110476	17656726381621,2	17433232572645,8	0,0183777798304085	0,00804270717536664	0,0062733959587601	0,987342285079071	-0.0183777798304135	1,01281998665733	0.0183777798304135	2073
Q9CZW4	sp|Q9CZW4|ACSL3_MOUSE Long-chain-fatty-acid--CoA ligase 3 OS=Mus musculus GN=Acsl3 PE=1 SV=2	−1,01	0,943416256124693	0,986987866859505	28,5978831612972	28,1529200921064	28,0336619814108	28,3139767888204	28,1946741447067	28,2385123208996	1878488183604,13	1855275684899,94	0,0179384607069039	0,0105219604733952	0,0021362108575383	0,987642989236348	-0.0179384607069029	1,01251161694896	0.017938460706903	2074
P35290	sp|P35290|RAB24_MOUSE Ras-related protein Rab-24 OS=Mus musculus GN=Rab24 PE=1 SV=2	−1,01	0,962150282215437	0,991306002794532	27,7741719005353	27,3951987839827	27,072984791289	27,102742443713	27,4525390811037	27,6503089516748	805008353987,763	795203180707,01	0,0176802273969302	0,0128027541784311	0,0101189594990531	0,987819786922482	-0.0176802273969301	1,01233039997656	0.0176802273969302	2075
Q2PFD7-5	sp|Q2PFD7-5|PSD3_MOUSE Isoform 5 of PH and SEC7 domain-containing protein 3 OS=Mus musculus GN=Psd3;sp|Q2PFD7-2|PSD3_MOUSE Isoform 2 of PH and SEC7 domain-containing protein 3 OS=Mus musculus GN=Psd3;sp|Q2PFD7|PSD3_MOUSE PH and SEC7 domain-containing pr	−1,01	0,889653122136233	0,976830327013275	31,6858278589972	31,3949762767942	31,486087748298	31,5364546439614	31,5115733756014	31,4825297714827	48973345404504,6	48383788749283,7	0,0174730052831639	0,00471946272086836	0,0008565229173533	0,987961683026729	-0.0174730052831645	1,01218500391269	0.0174730052831646	2076
P62320	sp|P62320|SMD3_MOUSE Small nuclear ribonucleoprotein Sm D3 OS=Mus musculus GN=Snrpd3 PE=1 SV=1	−1,01	0,954321142966882	0,989678056389876	28,7546109971162	28,5833286268197	28,2648305123125	28,3294382730544	28,4314922090982	28,8067679360488	2467574282315,52	2438894891701,74	0,0168659312339135	0,0087105944387596	0,0088126641703910	0,988377496548202	-0.0168659312339173	1,01175917449799	0.0168659312339173	2077
O55029	sp|O55029|COPB2_MOUSE Coatomer subunit beta OS=Mus musculus GN=Copb2 PE=2 SV=2	−1,01	0,950447088789818	0,988334326693467	29,5975087341519	29,3608168934757	29,0578855856183	29,2069428457707	29,5295487853568	29,2449656239036	5516347492591,56	5452811255694,14	0,0167131210559095	0,00921949390335011	0,0060117802070590	0,98848219098185	-0.0167131210559097	1,01165201469812	0.0167131210559099	2078
P12367	sp|P12367|KAP2_MOUSE cAMP-dependent protein kinase type II-alpha regulatory subunit OS=Mus musculus GN=Prkar2a PE=1 SV=2	−1,01	0,926703503332922	0,983810446072053	27,0068439722442	27,1813739338955	27,3917352883225	27,2837384201724	27,0832508388608	27,1783471400919	645517224915,946	638111455202,337	0,0166471596547251	0,00708716148326195	0,0036895243464088	0,988527386369011	-0.0166471596547246	1,01160576205494	0.0166471596547248	2079
P60904	sp|P60904|DNJC5_MOUSE DnaJ homolog subfamily C member 5 OS=Mus musculus GN=Dnajc5 PE=1 SV=1	−1,01	0,946228527933733	0,987340526043957	29,9252115172164	30,3400376693966	30,4157949618138	30,3583558022131	30,1695834981336	30,1189132769238	13409904828496,7	13257937236896,7	0,0164426700491691	0,00873720866677982	0,0041761352615430	0,988667511548847	-0.0164426700491681	1,01146238580592	0.0164426700491682	2080

Q3URE9	sp|Q3URE9|LIGO2_MOUSE Leucine-rich repeat and immunoglobulin-like domain-containing nogo receptor-interacting protein 2 OS=Mus musculus GN=Lingo2 PE=2 SV=1	−1,01	0,952375651117238	0,988754135503464	23,5687032910947	23,0551430075932	22,9752239618567	23,2674949779094	23,1210148714882	23,1763774222025	11898641689,9607	11763834123,6156	0,016438542877668	0,0138822277791867	0,0031897030541747	0,988670339870908	-0.0164385428776696	1,0114594922819	0.016438542877669	2081
P63280	sp|P63280|UBC9_MOUSE SUMO-conjugating enzyme UBC9 OS=Mus musculus GN=Ube2i PE=1 SV=1	−1,01	0,92867956132678	0,98433518830764	27,539642809425	27,4494037111634	27,5261939943834	27,2501225437999	27,5780928315527	27,6529615702981	881666912728,01	871712625433,603	0,016381114178151	0,00176999543657873	0,0077931219792712	0,988709696200794	-0.016381114178152	1,01141923037934	0.016381114178151	2082
Q9D5V5	sp|Q9D5V5|CUL5_MOUSE Cullin-5 OS=Mus musculus GN=Cul5 PE=1 SV=3	−1,01	0,932940380278209	0,98467134375593	30,3606214007766	30,192477358805	29,9229385752534	30,1229682803372	30,2081872493644	30,111798004507	12524140172134,6	12386783558500,6	0,015909945032434	0,0073209500508057	0,0017488075601832	0,989032651204303	-0.015909945032439	1,01108896534643	0.015909945032439	2083
Q8BS40	sp|Q8BS40|CPTP_MOUSE Ceramide-1-phosphate transfer protein OS=Mus musculus GN=Cptp PE=1 SV=1	−1,01	0,956407282474704	0,990217657408697	25,1630860770961	25,7219669464862	25,7790605133414	25,4815166293187	25,5721737946229	25,5777956432148	125391228221,01	124034884432,116	0,015690496276699	0,0133185183624948	0,002115461505045	0,989183104686531	-0.0156904962766938	1,01093518001088	0.015690496276693	2084
Q9CQN1	sp|Q9CQN1|TRAP1_MOUSE Heat shock protein 75 kDa, mitochondrial OS=Mus musculus GN=Trap1 PE=1 SV=1	−1,01	0,933409738249834	0,98467134375593	29,3627419253404	29,5895854758228	29,1625520471545	29,3052903367405	29,3473309859604	29,4300026872135	5700793449639,81	5639827917951,07	0,015511587008754	0,00727420743538344	0,002161029398831	0,98930578133951	-0.0155115870087546	1,01080982125265	0.015511587008754	2085
Q3V4B5	sp|Q3V4B5|COMD6_MOUSE COMM domain-containing protein 6 OS=Mus musculus GN=Commd6 PE=2 SV=1	−1,01	0,977069383389938	0,994783659375006	24,6083461525581	24,2341635524642	24,9183070413901	24,0194215071423	24,6663146621165	25,0441535538573	47640015913,5391	47151417429,494	0,014872754379644	0,013933160231736	0,021085865301830	0,989743947925378	-0.0148727543796436	1,01036232865694	0.014872754379643	2086
P58059	sp|P58059|RT21_MOUSE 28S ribosomal protein S21, mitochondrial OS=Mus musculus GN=Mrps21 PE=2 SV=1	−1,01	0,957791466434395	0,990217657408697	25,0773874416878	25,0535866622164	24,7917929036	24,7625063508653	24,8439697910874	25,286634641881	70174839297,5903	69484549904,9314	0,014261628940334	0,00634512882689265	0,011297932843447	0,990163292149034	-0.0142616289403349	1,00993442993591	0.014261628940334	2087
Q8K0F1	sp|Q8K0F1|TBC23_MOUSE TBC1 domain family member 23 OS=Mus musculus GN=Tbc1d23 PE=2 SV=1	−1,01	0,950104244282466	0,988334326693467	25,6579078890505	25,3532998395674	25,3098334236589	25,5740351178429	25,5098805984377	25,2075881976355	111841334854,944	110745576596,788	0,014204409101575	0,00745518599165278	0,007695297562941	0,990202564556502	-0.01420440910157 3	1,00989437494326	0.014204409101572	2088
Q01405	sp|Q01405|SC23A_MOUSE Protein transport protein Sec23A OS=Mus musculus GN=Sec23a PE=1 SV=2	−1,01	0,926538294714751	0,983810446072053	30,1496750736804	30,0077005409481	29,8229827561781	29,9490951305956	29,9252115172164	30,076831063814	10616736535076,6	10513829172188,9	0,014052166697137	0,005461576169513 9	0,002718888513771 4	0,990307062575425	-0.0140521666971354	1,00978781005496	0.014052166697135	2089
O70378	sp|O70378|EMC8_MOUSE ER membrane protein complex subunit 8 OS=Mus musculus GN=Emc8 PE=1 SV=1	−1,01	0,947082434154932	0,987777382497527	25,8861812107947	25,6003102586315	25,6146994185905	25,6757304025179	25,5121220870885	25,8849767619169	145057632029,506	143692736696,023	0,013639064106843	0,00626662132582201	0,007274636495127	0,990590668588845	-0.0136390641068441	1,00949870790178	0.013639064106844	2090
Q8BGU5	sp|Q8BGU5|CCNY_MOUSE Cyclin-Y OS=Mus musculus GN=Ccny PE=1 SV=1;sp|Q8BGU5-2|CCNY_MOUSE Isoform 2 of Cyclin-Y OS=Mus musculus GN=Ccny	−1,01	0,964390273343897	0,991306002794532	27,4766700566273	26,7786940840882	27,1751206599197	27,2306522722493	27,0177302526787	27,1537780055346	614143796264,84	608372691254,218	0,013621094704907	0,012896683841461 5	0,003973704753886	0,990603006908608	-0.0136210947049067	1,00948613422921	0.013621094704906	2091
Q3TDD9	sp|Q3TDD9|PPR21_MOUSE Protein phosphatase 1 regulatory subunit 21 OS=Mus musculus GN=Ppp1r21 PE=2 SV=2	−1,01	0,950435761750825	0,988334326693467	29,2598728637706	29,104176927775	28,787551864115	28,9855437086712	29,139589158536	28,9989754780626	4135105199112,34	4097382406956,63	0,013221487519485	0,00828509533183485	0,002938066513529	0,990877428665223	-0.0132214875194875	1,0092065588244	0.013221487519487	2092
P20029	sp|P20029|GRP78_MOUSE 78 kDa glucose-regulated protein OS=Mus musculus GN=Hspa5 PE=1 SV=3	−1,01	0,922156094085376	0,982113422958876	33,1617830500677	33,0960166649535	32,8687527944652	33,0157551936444	33,0476252564011	33,037004306747	223891834582071	221947411720511	0,012584029014243	0,00465324839459324	0,000491250486826	0,991315347139883	-0.0125840290142438	1,00876073681818	0.012584029014243	2093
Q9QWW1-2	sp|Q9QWW1-2|HOME2_MOUSE Isoform 2 of Homer protein homolog 2 OS=Mus musculus GN=Homer2;sp|Q9QWW1|HOME2_MOUSE Homer protein homolog 2 OS=Mus musculus GN=Homer2 PE=1 SV=1	−1,01	0,966198687085269	0,991314611365289	26,7761234567643	26,4988608819045	26,0883849331964	26,438944362597	26,4451428857265	26,4538794096732	308336150946,891	305736325479,648	0,012216075017989	0,0130795672162762	0,000283724649022	0,991568210671181	-0.0122160750179879	1,00850348895626	0.012216075017987	2094
Q8VCW8	sp|Q8VCW8|ACSF2_MOUSE Acyl-CoA synthetase family member 2, mitochondrial OS=Mus musculus GN=Acsf2 PE=1 SV=1	−1,01	0,946775822480145	0,987684545676253	29,9201519045597	30,2703137998586	30,295446665439	30,2152266398378	30,1559838428577	30,0893751498851	12565433538030	12459799581336,2	0,012179586090377	0,00695571215100074	0,002088036259401	0,991593289927157	-0.0121795860903684	1,0084779820096	0.012179586090368	2095
Q9D1I6	sp|Q9D1I6|RM14_MOUSE 39S ribosomal protein L14, mitochondrial OS=Mus musculus GN=Mrpl14 PE=2 SV=1	−1,01	0,969704838787882	0,992444253271296	24,4391865491359	24,3378985272248	23,9823398794781	23,921929541055	24,2965262702175	24,5156871626391	34119730192,9491	33833400251,5744	0,012158063316704	0,00989261963148066	0,012384164880474	0,991608083072302	-0.0121580633167044	1,00846293719359	0.012158063316704	2096
Q01853	sp|Q01853|TERA_MOUSE Transitional endoplasmic reticulum ATPase OS=Mus musculus GN=Vcp PE=1 SV=4;sp|P55072|TERA_HUMAN Transitional endoplasmic reticulum ATPase OS=Homo sapiens GN=VCP PE=1 SV=4	−1,01	0,960948301904265	0,991306002794532	34,1536747954851	33,9275094387217	33,6516402612364	33,8225092961663	34,0837055790221	33,8014606732477	533746166626415	529290483987622	0,012094087043517	0,00741433289478302	0,004637720152247	0,99165205688135 3	-0.0120940870435168	1,00841821792303	0.012094087043517	2097

Q63570	sp|Q63570|PRS6B_RAT 26S protease regulatory subunit 6B OS=Rattus norvegicus GN=Psmc4 PE=1 SV=1;sp|P54775|PRS6B_MOUSE 26S protease regulatory subunit 6B OS=Mus musculus GN=Psmc4 PE=1 SV=2;sp|P43686|PRS6B_HUMAN 26S protease regulatory subunit 6B OS=Homo s	−1,01	0,962377196407448	0,991306002794532	31,4047907964564	31,0218212612287	30,82620630466	31,0631349310244	31,1607157709667	31,0040688696121	31602987950545,5	31341781343451,2	0,0119737873091181	0,00946795565806984	0,0025456532014999	0,991734749653955	-0.0119737873091172	1,00833413405039	0.0119737873091171	2098
Q9CX80	sp|Q9CX80|CYGB_MOUSE Cytoglobin OS=Mus musculus GN=Cygb PE=2 SV=1	−1,01	0,92319663639519	0,982736757175836	28,1132288221562	28,2874146092639	28,4053017035069	28,2874146092639	28,2478477131411	28,2458362215109	1891986362041,67	1876381269689,67	0,0119486845449365	0,00519791325352773	0,0008296497895757	0,991752005899683	-0.01194868454494	1,00831658927963	0.01194868454494	2099
Q8BVA5-2	sp|Q8BVA5-2|CB043_MOUSE Isoform 2 of UPF0554 protein C2orf43 homolog OS=Mus musculus;sp|Q8BVA5|CB043_MOUSE UPF0554 protein C2orf43 homolog OS=Mus musculus PE=2 SV=1	−1,01	0,994651680758959	0,999841939860314	21,1029986109079	24,3570462707292	24,7872986927083	24,1461515325522	23,2077788046151	22,8687955606518	14768777950,609	14648082834,0067	0,01183859947525	0,086029512720545	0,0282686799156157	0,991827684253502	-0.0118385999475307	1,00823965279075	0.0118385999475307	2100
Q9D7N3	sp|Q9D7N3|RT09_MOUSE 28S ribosomal protein S9, mitochondrial OS=Mus musculus GN=Mrps9 PE=2 SV=3	−1,01	0,965410154608668	0,991306002794532	26,871435906167	26,2733618497883	26,6649401997547	26,5658421318017	26,6350593510396	26,5858634061522	357802138654,142	355072665253,754	0,0110477098086298	0,0114186281440167	0,0013392679266952	0,992371556495848	-0.0110477098086313	1,00768708399008	0.0110477098086314	2101
Q6P2K6	sp|Q6P2K6|P4R3A_MOUSE Serine/threonine-protein phosphatase 4 regulatory subunit 3A OS=Mus musculus GN=Smek1 PE=1 SV=1	−1,01	0,96957477497791	0,992444253271296	26,336120588715	25,8783648256611	25,6734309040907	26,0422096112122	25,9187584767025	25,9039797918375	188551831238,83	187113762900,674	0,0110454842102194	0,0130682963119708	0,0029243454016060	0,992373087396142	-0.0110454842102206	1,00768552946541	0.0110454842102207	2102
Q9CS42	sp|Q9CS42|PRPS2_MOUSE Ribose-phosphate pyrophosphokinase 2 OS=Mus musculus GN=Prps2 PE=1 SV=4	−1,01	0,979158274315233	0,994969692078273	26,6107194319358	26,8468256315125	27,5493288208279	27,139097165185	26,9340296968593	26,9109358823026	533268717649,252	529229272058,471	0,010969839484326	0,0180812683976596	0,0046525559804828	0,992425121787403	-0.010969839484326	1,00763269494726	0.010969839484326	2103
Q15797	sp|Q15797|SMAD1_HUMAN Mothers against decapentaplegic homolog 1 OS=Homo sapiens GN=SMAD1 PE=1 SV=1;sp|P70340|SMAD1_MOUSE Mothers against decapentaplegic homolog 1 OS=Mus musculus GN=Smad1 PE=1 SV=2;sp|P97454|SMAD5_MOUSE Mothers against decapentaplegic h	−1,01	0,969127428594877	0,992417383342423	22,451234435472	22,9428230979447	22,8966186104492	22,9274759558395	22,5318902919992	22,8085879999257	7692861936,49718	7634816560,92833	0,0109269222751041	0,0119254450697587	0,0089194670518575	0,992454644832053	-0.0109269222751038	1,00760272039356	0.0109269222751037	2104
Q9D710	sp|Q9D710|TMX2_MOUSE Thioredoxin-related transmembrane protein 2 OS=Mus musculus GN=Tmx2 PE=2 SV=1	−1,01	0,933218149165192	0,98467134375593	29,0865735685853	29,1279023668535	29,2199061542331	29,2687031531706	28,991263159107	29,151901871807	4543842019422,77	4509869776765,36	0,0108268999806266	0,00234183865158718	0,0047806787114164	0,992523454267953	-0.0108268999806268	1,00753286554579	0.0108268999806269	2105
Q9WU78-3	sp|Q9WU78-3|PDC6I_MOUSE Isoform 3 of Programmed cell death 6-interacting protein OS=Mus musculus GN=Pdcd6ip	−1,01	0,965885248692257	0,991306002794532	31,5082584125245	31,0176093622394	30,9917314842313	31,2180758996145	31,209001882346	31,0680797664268	34519071569054,5	34261812652206,3	0,0107921815343626	0,00933624563690801	0,0026986423260935	0,992547339625472	-0.0107921815343637	1,00750861956604	0.0107921815343636	2106
Q14CH0-2	sp|Q14CH0-2|F171B_MOUSE Isoform 2 of Protein FAM171B OS=Mus musculus GN=Fam171b;sp|Q14CH0|F171B_MOUSE Protein FAM171B OS=Mus musculus GN=Fam171b PE=1 SV=2	−1,01	0,970289899548353	0,992819173591067	24,5712773674685	24,8115612153573	24,8115612153573	25,0158762888834	24,3866384851643	24,7695581872001	55047737451,8195	54639577536,4633	0,010736938975074	0,00560936989737151	0,0128247938595708	0,992585346205856	-0.0107369389750738	1,00747004156619	0.010736938975074	2107
Q8BGD9	sp|Q8BGD9|IF4B_MOUSE Eukaryotic translation initiation factor 4B OS=Mus musculus GN=Eif4b PE=1 SV=1	−1,01	0,963864413447098	0,991306002794532	30,0981104206294	29,9807891911943	29,6323404854074	30,0218464917338	29,9085130255149	29,7592429065261	9705818811689,47	9636066879632,91	0,0104055213973128	0,00810112994684222	0,0044055600088079	0,992813390254869	-0.0104055213973135	1,00723863096094	0.0104055213973135	2108
Q8BTG7-3	sp|Q8BTG7-3|NDRG4_MOUSE Isoform 3 of Protein NDRG4 OS=Mus musculus GN=Ndrg4;sp|Q8BTG7|NDRG4_MOUSE Protein NDRG4 OS=Mus musculus GN=Ndrg4 PE=1 SV=1;sp|Q8BTG7-2|NDRG4_MOUSE Isoform 2 of Protein NDRG4 OS=Mus musculus GN=Ndrg4	−1,01	0,94034417272673	0,986144884061661	30,60874741464	30,381645732265	30,4225096278016	30,3763944677086	30,5864926907295	30,4288703183794	17114849451272,1	16994640727161,1	0,0101687388009622	0,00397288087675764	0,0035893735036210	0,992976349312727	-0.0101687388009664	1,00707333129549	0.0101687388009664	2109
P0C028	sp|P0C028|NUD11_MOUSE Diphosphoinositol polyphosphate phosphohydrolase 3-beta OS=Mus musculus GN=Nudt11 PE=1 SV=1;sp|P0C027|NUD10_MOUSE Diphosphoinositol polyphosphate phosphohydrolase 3-alpha OS=Mus musculus GN=Nudt10 PE=1 SV=1	−1,01	0,975505427940332	0,994649943013952	28,2559271319953	28,2234557658422	27,7887330476403	27,749949888637	28,1771173987933	28,3200413611814	1581463787439,24	1570428376801,13	0,0101023743370335	0,00928702424164726	0,010562529817175	0,993022027614063	-0.0101023743370363	1,00702700664426	0.0101023743370361	2110
P80316	sp|P80316|TCPE_MOUSE T-complex protein 1 subunit epsilon OS=Mus musculus GN=Cct5 PE=1 SV=1	−1,01	0,956228416101472	0,990217657408697	31,63710827341	32,0093513947963	31,9696296459334	31,8850101738384	31,8186731138519	31,8925920404403	69477895766036,5	69020529784262,3	0,0095285131185622	0,00641362068749274	0,0012761637756397	0,993417100838598	-0.00952851311856145	1,0066265208015	0.0095285131185616	2111
Q9Z1N5	sp|Q9Z1N5|DX39B_MOUSE Spliceosome RNA helicase Ddx39b OS=Mus musculus GN=Ddx39b PE=1 SV=1	−1,01	0,975241216761107	0,994649943013952	30,7933119851167	30,0689715847678	30,447826850692	30,4196223089356	30,3808159827936	30,4899709322161	16538357130339,5	16430104495198,5	0,0094742728931639	0,0119033354071742	0,0018183231457573	0,993454450506307	-0.00947427289316514	1,00658867599854	0.0094742728931652	2112
Q9QXE7	sp|Q9QXE7|TBL1X_MOUSE F-box-like/WD repeat-containing protein TBL1X OS=Mus musculus GN=Tbl1x PE=2 SV=2	−1,01	0,984110378569148	0,99751608046569	23,6580266549454	24,4583038673233	24,6083461525581	24,4630513056042	24,1908952126927	24,0516859730864	33726809481,4201	33513387756,2952	0,0091583163372070	0,0210750448503313	0,0086334120509054	0,993672045224365	-0.00915831633720988	1,0063682527913	0.0091583163372099	2113
P10639	sp|P10639|THIO_MOUSE Thioredoxin OS=Mus musculus GN=Txn PE=1 SV=3	−1,01	0,975974772377352	0,994649943013952	31,3364515285498	30,9907657784686	30,9481182033933	30,766243128978	31,1636297730172	31,3267661620555	31841083424289	31643262121022,3	0,0089910901491219	0,00684950552412696	0,0092745402172865	0,993787230772562	-0.00899109014912135	1,00625160903165	0.0089910901491213	2114

Q9D3D9	sp|Q9D3D9|ATPD_MOUSE ATP synthase subunit delta, mitochondrial OS=Mus musculus GN=Atp5d PE=1 SV=1	−1,01	0,982195725013507	0,996986564004246	28,8465312886039	28,1896196889447	28,4585732117269	28,1431261777461	28,5793494389656	28,7546109971162	2380285123973,06	2366332027807,75	0,0084818808771779	0,0115883453058933	0,0110516107304757	0,994138056813118	-0.00848188087718347	1,00589650818285	0.0084818808771833	2115
Q7TNE3	sp|Q7TNE3|SPAG7_MOUSE Sperm-associated antigen 7 OS=Mus musculus GN=Spag7 PE=1 SV=1	−1,00	0,985915876149638	0,998141007543842	24,8446467185302	25,2798116120726	25,2882777697561	25,4119626398757	24,6534719708188	25,3323809620451	82625046683,2512	82215130458,9156	0,0071752570679160	0,0100933290086341	0,0165858271896508	0,995038838212013	-0.00717525706791699	1,0049858976328	0.0071752570679169	2116
Q99LI7	sp|Q99LI7|CSTF3_MOUSE Cleavage stimulation factor subunit 3 OS=Mus musculus GN=Cstf3 PE=1 SV=1	−1,00	0,980526908378643	0,995885351785782	26,7568583343213	26,6455238891739	26,3107417857097	26,8229841428725	26,3165025351457	26,5591097026922	346462815901,274	344789110641,18	0,0069863125283086	0,00873874721758957	0,0095352448807885	0,995169163375469	-0.00698631252831095	1,00485428689143	0.0069863125283109	2117
Q9DBS5	sp|Q9DBS5|KLC4_MOUSE Kinesin light chain 4 OS=Mus musculus GN=Klc4 PE=1 SV=1	−1,00	0,97502120383717	0,994649943013952	25,9200419149704	25,6522026352665	25,8041604093304	25,5463841594127	25,9276015798895	25,8889079988918	158994426727,248	158279967172,891	0,0064975240238877	0,00520794291680856	0,0081364573084268	0,995506386173003	-0.00649752402388462	1,00451389753939	0.0064975240238845	2118
Q9JIA7	sp|Q9JIA7|SPHK2_MOUSE Sphingosine kinase 2 OS=Mus musculus GN=Sphk2 PE=1 SV=2	−1,00	0,98494305419985	0,997872253123923	25,5740351178429	26,269865312127	26,0204577171094	26,1633273553353	25,8054283782832	25,8825371944796	187076984149,225	186264021767,993	0,0062830422107823	0,0135825802409667	0,0072585154190108	0,995654396584758	-0.00628304221078196	1,004364570106	0.0062830422107820	2119
Q1HFZ0	sp|Q1HFZ0|NSUN2_MOUSE tRNA (cytosine(34)-C(5))-methyltransferase OS=Mus musculus GN=Nsun2 PE=1 SV=2;sp|Q1HFZ0-2|NSUN2_MOUSE Isoform 2 of tRNA (cytosine(34)-C(5))-methyltransferase OS=Mus musculus GN=Nsun2	−1,00	0,975405936886119	0,994649943013952	25,4427882169683	25,6812465519977	25,2509320978723	25,4794674273275	25,4944981642208	25,3890942487746	113869886719,58	113418831845,269	0,0057260693685116	0,00846785549258501	0,0022398321058998	0,996038857266787	-0.00572606936850643	1,00397689578505	0.0057260693685065	2120
Q9CR13	sp|Q9CR13|CG055_MOUSE UPF0562 protein C7orf55 homolog OS=Mus musculus PE=3 SV=1	−1,00	0,982547037450035	0,996986564004246	23,7779715892546	23,3797067172341	23,4877467946751	23,3953782803666	23,4291787490228	23,8091764130591	16864472610,372	16798875962,942	0,0056224993494157	0,00874622883522716	0,0097589975585483	0,996110364732683	-0.00562249934941403	1,00390482360693	0.0056224993494141	2121
Q9QX60-2	sp|Q9QX60-2|DGUOK_MOUSE Isoform 2 of Deoxyguanosine kinase, mitochondrial OS=Mus musculus GN=Dguok;sp|Q9QX60|DGUOK_MOUSE Deoxyguanosine kinase, mitochondrial OS=Mus musculus GN=Dguok PE=2 SV=3	−1,00	0,987774371295366	0,998141007543842	26,5421786038889	26,7926130600652	27,2725327823275	27,0687747291807	26,6089362359476	26,9179630935536	466772651643,496	464963472752,231	0,0056026521382273	0,0138127899548321	0,0087254711057770	0,996124068355558	-0.00560265213822633	1,00389101294464	0.0056026521382263	2122
Q8R2R9	sp|Q8R2R9|AP3M2_MOUSE AP-3 complex subunit mu-2 OS=Mus musculus GN=Ap3m2 PE=2 SV=1	−1,00	0,977220545187769	0,994783659375006	29,1918467273542	29,3473309859604	29,5962766892579	29,2904693642646	29,5072210768158	29,3261521639233	5740025684246,96	5717851287270,67	0,0055840942560847	0,00694409224104802	0,0039564283791541	0,996136881924214	-0.00558409425608414	1,00387809963258	0.0055840942560840	2123
P11438	sp|P11438|LAMP1_MOUSE Lysosome-associated membrane glycoprotein 1 OS=Mus musculus GN=Lamp1 PE=1 SV=2	−1,00	0,982709382541307	0,996986564004246	27,3399629165513	27,3015176496588	26,8359983838767	27,1958332984449	27,1925405628277	27,0782792312648	623839930035,273	621592786278,115	0,0052061369999938	0,010328936820642	0,0024650426164276	0,996397884058125	-0.00520613699999231	1,00361513808841	0.0052061369999922	2124
Q3THJ3	sp|Q3THJ3|EIF1A_MOUSE Probable RNA-binding protein EIF1AD OS=Mus musculus GN=Eif1ad PE=2 SV=2	−1,00	0,994416215466597	0,999841939860314	22,9194574103533	22,7406104302362	21,5846616401495	21,9541395813542	22,7898514546523	22,490566066182	5428397604,67298	5410022204,08172	0,0048918800295743	0,0323247095357754	0,0188932481795899	0,996614949395851	-0.00489188002957352	1,00339654809132	0.0048918800295733	2125
Q91WT9-2	sp|Q91WT9-2|CBS_MOUSE Isoform 2 of Cystathionine beta-synthase OS=Mus musculus GN=Cbs;sp|Q91WT9|CBS_MOUSE Cystathionine beta-synthase OS=Mus musculus GN=Cbs PE=2 SV=3	−1,00	0,983173204512601	0,997234229113452	27,0475341194994	27,2883154174475	27,3309114068393	27,4032091695388	26,9717991804572	27,2818929651463	664468724600,947	662288504300,434	0,0047414791165845	0,00561316891293788	0,0081737981202057	0,996718851889045	-0.00474147911658411	1,00329194948479	0.0047414791165839	2126
Q80TY0-2	sp|Q80TY0-2|FNBP1_MOUSE Isoform 2 of Formin-binding protein 1 OS=Mus musculus GN=Fnbp1;sp|Q80TY0-3|FNBP1_MOUSE Isoform 3 of Formin-binding protein 1 OS=Mus musculus GN=Fnbp1;sp|Q80TY0|FNBP1_MOUSE Formin-binding protein 1 OS=Mus musculus GN=Fnbp1 PE=1 SV	−1,00	0,976023539586725	0,994649943013952	27,3497540213979	27,2578083209851	27,0003491513743	27,2250623653521	27,1537780055346	27,219419790962	651561198969,208	649468422657,328	0,0046413095608164	0,00665811494437153	0,0014569329552352	0,996788058719287	-0.00464130956081806	1,00322229109048	0.0046413095608180	2127
P97742	sp|P97742|CPT1A_MOUSE Carnitine O-palmitoyltransferase 1, liver isoform OS=Mus musculus GN=Cpt1a PE=1 SV=4	−1,00	0,983684804729959	0,997307422083825	26,0089907009257	26,0524841852733	25,7538649352021	26,0204577171094	25,6870742225767	26,0981905549866	184045087837,726	183456025295,869	0,0046249565258715	0,00621952731586984	0,0084211543222953	0,996799357435845	-0.00462495652586718	1,00321091957	0.0046249565258671	2128
Q9CR64-2	sp|Q9CR64-2|KISHA_MOUSE Isoform 2 of Protein kish-A OS=Mus musculus GN=Tmem167a;sp|Q9CR64|KISHA_MOUSE Protein kish-A OS=Mus musculus GN=Tmem167a PE=2 SV=1	−1,00	0,977469733600469	0,994783659375006	25,6338439500692	25,506098678661	25,5517501914311	25,3493149492024	25,6594416194182	25,6734309040907	126549276488,242	126148946088,89	0,0045711058759923	0,00253221052262586	0,0071681693617177	0,996836565087836	-0.00457110587599693	1,00317347399058	0.0045711058759968	2129

Q8BGW1	sp|Q8BGW1|FTO_MOUSE Alpha-ketoglutarate-dependent dioxygenase FTO OS=Mus musculus GN=Fto PE=1 SV=1;sp|Q8BGW1-2|FTO_MOUSE Isoform 2 of Alpha-ketoglutarate-dependent dioxygenase FTO OS=Mus musculus GN=Fto;sp|Q8BGW1-3|FTO_MOUSE Isoform 3 of Alpha-ketogluta	−1,00	0,990355264652795	0,998494568249453	25,1110816865738	25,9677083343354	25,7769511586397	25,5795222243761	25,5890196137344	25,6777150180066	133662044232,204	133240146799,538	0,0045609954603497	0,017551061635232	0,0021142954323535	0,996843550949041		-0.0045609954603533	1,00316644376939	0.0045609954603532	2130
Q8BY87-2	sp|Q8BY87-2|UBP47_MOUSE Isoform 2 of Ubiquitin carboxyl-terminal hydrolase 47 OS=Mus musculus GN=Usp47;sp|Q8BY87|UBP47_MOUSE Ubiquitin carboxyl-terminal hydrolase 47 OS=Mus musculus GN=Usp47 PE=1 SV=2	−1,00	0,997909635968322	0,999958878348632	22,9293534018626	26,238618367179	24,7625063508653	25,4336098486706	23,8315975050895	24,6569155403246	50411880408,458	50271674858,9469	0,0040180142863402	0,067272917153523	0,0325124245031299	0,997218799450148		-0.0040180142863962	1,00278895719915	0.0040180142863395	2131
Q6P9R2	sp|Q6P9R2|OXSR1_MOUSE Serine/threonine-protein kinase OSR1 OS=Mus musculus GN=Oxsr1 PE=1 SV=1	−1,00	0,986615218643155	0,998141007543842	27,7178014437035	28,1245214621915	28,1612225425277	27,9817500837541	27,9056879546371	28,1083623316671	1447967284617,89	1444233899222,48	0,0037245953825035	0,0087889045190257	0,0036566993409749	0,99742163691468		-0.0037245953825002	1,00258502822667	0.0037245953825000	2132
Q9D4H1	sp|Q9D4H1|EXOC2_MOUSE Exocyst complex component 2 OS=Mus musculus GN=Exoc2 PE=1 SV=1	−1,00	0,988274654708626	0,998141007543842	29,4808389247203	29,0865735685853	29,0359313240441	29,2155065783681	29,3492574870933	29,0311658380649	4807097587622,83	4795232452424,67	0,0035653389022225	0,0083410556603483	0,0054699427811507	0,997531746551451		-0.0035653389022214	1,00247436079812	0.0035653389022215	2133
Q8VIJ6	sp|Q8VIJ6|SFPQ_MOUSE Splicing factor, proline- and glutamine-rich OS=Mus musculus GN=Sfpq PE=1 SV=1	−1,00	0,990918846904202	0,998548920334855	32,2966368508442	32,077801964065	31,7533387554372	31,9922601162753	32,1601216416315	31,9696296459334	82398843985947,6	82240620939946,7	0,0027729399411818	0,0085309904411986	0,0032478954466273	0,998079790463712		-0.0027729399411812	1,00192390383478	0.0027729399411812	2134
O08529	sp|O08529|CAN2_MOUSE Calpain-2 catalytic subunit OS=Mus musculus GN=Capn2 PE=2 SV=4	−1,00	0,988097539142834	0,998141007543842	29,5745822065934	29,5442070726591	29,1940485808107	29,4300026872135	29,4026825687162	29,4746082875131	6089655451984,66	6078411519193,96	0,0026662526977700	0,0071839625285173	0,0012334446262533	0,998153601155376		-0.0026662526977718	1,00184981433968	0.0026662526977720	2135
Q9D142	sp|Q9D142|NUD14_MOUSE Uridine diphosphate glucose pyrophosphatase OS=Mus musculus GN=Nudt14 PE=2 SV=1	−1,00	0,994349910290186	0,999841939860314	24,4487515518704	24,7940826638887	25,2133112519035	24,7900819061277	24,6947800953991	24,9662926902838	60066322031,2512	59966479253,2602	0,0024000558573155	0,015426849857281	0,0055506529610877	0,998337791051381		-0.0024000558573163	1,0016649764874	0.0024000558573161	2136
Q6PD10	sp|Q6PD10|IP6K1_MOUSE Inositol hexakisphosphate kinase 1 OS=Mus musculus GN=Ip6k1 PE=2 SV=1	−1,00	0,98267392838584	0,996986564004246	25,6974368156947	25,5883688052889	25,7383458751693	25,7852122009926	25,5972870851289	25,6369206672453	141380003915,528	141157197818,608	0,0022753911043618	0,0030195807165422	0,0038584990986525	0,99842406216756		-0.0022753911043606	1,00157842533263	0.0022753911043605	2137
Q80ZJ7	sp|Q80ZJ7|SNX32_MOUSE Sorting nexin-32 OS=Mus musculus GN=Snx32 PE=2 SV=1	−1,00	0,992577756568486	0,999250269845419	25,4178300813803	25,5121220870885	25,426892640985	25,266100588418	25,7471346094699	25,3393502864799	113184107884,983	113023525937,057	0,0020483023930799	0,0020438679563861	0,010183566193091	0,998581232375051		-0.0020483023930789	1,00142078338642	0.0020483023930789	2138
Q8BH55	sp|Q8BH55|THNS1_MOUSE Threonine synthase-like 1 OS=Mus musculus GN=Thnsl1 PE=2 SV=1	−1,00	0,990298013648314	0,998494568249453	29,0311658380649	28,9317927292942	28,7253092782756	28,9475574493059	28,9124490208349	28,8247153553848	3543334262622,01	3539148492064,24	0,0017052752087781	0,0053994430938451	0,0021897239621918	0,998818691591723		-0.0017052752087771	1,00118270554829	0.0017052752087771	2139
Q5DU31	sp|Q5DU31|ICEF1_MOUSE Interactor protein for cytohesin exchange factors 1 OS=Mus musculus GN=Ipcef1 PE=2 SV=2;sp|Q5DU31-2|ICEF1_MOUSE Isoform 2 of Interactor protein for cytohesin exchange factors 1 OS=Mus musculus GN=Ipcef1;sp|Q5DU31-3|ICEF1_MOUSE Isof	−1,00	0,987982996011202	0,998141007543842	26,5902926789079	26,3762555589271	26,446728601505	26,410633198047	26,5234452786041	26,475991979372	313508490560,42	313173593429,123	0,0015419444367929	0,0041206725980521	0,0021398686846428	0,998931776518402		-0.0015419444367946	1,00106936580326	0.0015419444367945	2140
O55229	sp|O55229|CHKB_MOUSE Choline/ethanolamine kinase OS=Mus musculus GN=Chkb PE=1 SV=3	−1,00	0,993506047709336	0,999841939860314	27,270293282656	26,8888307780833	27,1079700259264	27,0036061767615	27,1916972274608	27,0687747291807	581589879699,135	581005490864,823	0,0014503669386033	0,0070668878482064	0,0035259818323781	0,998995187408326		-0.0014503669386039	1,00100582325555	0.0014503669386040	2141
Q9Z2Q6	sp|Q9Z2Q6|SEPT5_MOUSE Septin-5 OS=Mus musculus GN=Sept5 PE=1 SV=2;sp|Q99719|SEPT5_HUMAN Septin-5 OS=Homo sapiens GN=SEPT5 PE=1 SV=1	−1,00	0,992645839773737	0,999250269845419	33,3596098324373	32,9992079572545	33,1921566422038	33,1921566422038	33,1816073307961	33,1743276583604	257916793743780	257669071894599	0,0013863340120550	0,0054349395129653	0,0002701504515907	0,999039528036987		-0.0013863340120525	1,0009613953563	0.0013863340120525	2142
Q8BHH2	sp|Q8BHH2|RAB9B_MOUSE Ras-related protein Rab-9B OS=Mus musculus GN=Rab9b PE=2 SV=1	−1,00	0,995306954666276	0,999929810953105	25,8047943938068	25,9187584767025	26,1085492621562	26,014799565769	25,7084262444795	26,1062400879502	185076306439,09	184913743029,425	0,0012677607972491	0,0059145281457656	0,0080313823315676	0,99912164116092		-0.0012677607972561	1,00087913103159	0.0012677607972562	2143
Q9DCS2	sp|Q9DCS2|CP013_MOUSE UPF0585 protein C16orf13 homolog OS=Mus musculus PE=1 SV=1	−1,00	0,998196448071745	0,999958878348632	26,7069466343535	25,6255477551962	26,2439255198213	26,0701199334524	26,1876191399344	26,3165025351457	237192994572,32	237020831184,167	0,0010475412724544	0,020714464906862	0,0047051727655961	0,999274163267497		-0.0010475412724516	1,00072636395414	0.0010475412724515	2144
Q8K3X4	sp|Q8K3X4|I2BPL_MOUSE Interferon regulatory factor 2-binding protein-like OS=Mus musculus GN=Irf2bpl PE=1 SV=1	−1,00	0,997972701029144	0,999958878348632	26,2580903346101	25,8344529325275	25,8152971897798	26,2312364204502	25,879283072998	25,7958217310218	189808243038,037	189713411176,925	0,0007209784057005	0,0096383215687285	0,0088988645894224	0,999500380702154		-0.0007209784056980	1,00049986904207	0.0007209784056983	2145
P23492	sp|P23492|PNPH_MOUSE Purine nucleoside phosphorylase OS=Mus musculus GN=Pnp PE=1 SV=2	−1,00	0,999044629261762	1	28,9160873320475	29,119788155716	29,2467701061378	29,1870270397349	28,9505536434768	29,1446312403025	4319735104782,4	4319110702849,21	0,0002085513722930	0,0057337182890245	0,0043336695731980	0,999855453652124		-0.0002085513722926	1,00014456724454	0.0002085513722927	2146
Q9DC40	sp|Q9DC40|TELO2_MOUSE Telomere length regulation protein TEL2 homolog OS=Mus musculus GN=Telo2 PE=1 SV=2	1,00	0,999894372494256	1	24,0319190122397	23,4099585585598	23,2593273389152	23,9578712584091	23,0804133432428	23,6630592406412	17180971551,9294	17181767235,9129	−6,68×10^−05	0,0173783674304734	0,018947073663397	1,00004631193184		0.0000668124473081	0,999953690212853	-0.000066812447308	2147

Q9CYI4	sp|Q9CYI4|LUC7L_MOUSE Putative RNA-binding protein Luc7-like 1 OS=Mus musculus GN=Luc7l PE=2 SV=2;sp|Q9CYI4-2|LUC7L_MOUSE Isoform 2 of Putative RNA-binding protein Luc7-like 1 OS=Mus musculus GN=Luc7l	1,00	1,00	0,99933930831658	1	26,1356881565921	25,8289851792492	25,8761040310522	25,7260541994384	26,1449400230198	25,9701667726243	185612274443,066	185636011327,627	−0,000184486161970	0,00636537648197663	0,0081087137836473	1,00012788423951	0.000184486161965112	0,999872132112777	-0.000184486161965	2148
Q8R311	sp|Q8R311|CTGE5_MOUSE cTAGE family member 5 OS=Mus musculus GN=Ctage5 PE=1 SV=1	1,00	1,00	0,997959318801619	0,999958878348632	26,5234452786041	26,2569962681467	26,2691378786882	26,2649632160102	26,3762555589271	26,4091064430994	277714601077,975	277783648824,756	−0,000358650427413	0,00570978205306479	0,0028669775719962	1,0002486284355	0.000358650427417077	0,999751433365235	-0.000358650427417	2149
Q8VCX5	sp|Q8VCX5|MICU1_MOUSE Calcium uptake protein 1, mitochondrial OS=Mus musculus GN=Micu1 PE=1 SV=1;sp|Q8VCX5-3|MICU1_MOUSE Isoform 3 of Calcium uptake protein 1, mitochondrial OS=Mus musculus GN=Micu1;sp|Q8VCX5-2|MICU1_MOUSE Isoform 2 of Calcium uptake pr	1,00	1,00	0,999388716343845	1	24,2034797259004	23,6274438234	23,0567184762801	23,7711154655879	23,4666785516388	23,6506356046121	18282570450,2403	18287370841,7008	−0,000378753738702	0,0242658377885269	0,0064883977480063	1,0002625665067	0.000378753738699462	0,999737502372427	-0.000378753738699	2150
Q8C052	sp|Q8C052|MAP1S_MOUSE Microtubule-associated protein 1S OS=Mus musculus GN=Map1s PE=1 SV=2	1,00	1,00	0,99873754801123	1	28,4613395369887	27,9313864300688	28,0423253078252	28,1636447074317	28,2856963956054	27,9865800133104	1671958971356,41	1672443821390,42	−0,000418305322582	0,00993080688018593	0,0053436612428981	1,0002899891937	0.000418305322587582	0,999710094875649	-0.000418305322587	2151
Q9JLV5	sp|Q9JLV5|CUL3_MOUSE Cullin-3 OS=Mus musculus GN=Cul3 PE=1 SV=1	1,00	1,00	0,998170717242602	0,999958878348632	30,4090370110476	30,4957910271004	30,0254239818711	30,3275359257424	30,2598956411556	30,343830029212	14571431872083,8	14576336353688,2	−0,000485503473251	0,00825830545570307	0,0014684006394772	1,00033658199465	0.000485503473251639	0,999663531254672	-0.000485503473251	2152
P97823-2	sp|P97823-2|LYPA1_MOUSE Isoform 2 of Acyl-protein thioesterase 1 OS=Mus musculus GN=Lypla1;sp|P97823|LYPA1_MOUSE Acyl-protein thioesterase 1 OS=Mus musculus GN=Lypla1 PE=1 SV=1	1,00	1,00	0,997783264516399	0,999958878348632	28,1061634244686	28,4357489471046	28,5402325045527	28,5161702425418	28,4613395369887	28,1061634244686	2074445404005,25	2075502484154	−0,000734970347827	0,0079882919724305	0,0078481834507764	1,00050957241232	0.000734970347824377	0,999490687119471	-0.000734970347824	2153
P70297	sp|P70297|STAM1_MOUSE Signal transducing adapter molecule 1 OS=Mus musculus GN=Stam PE=2 SV=3	1,00	1,00	0,995372619210034	0,999929810953105	28,6894779101035	28,9044917431211	28,7178629849572	28,9160873320475	28,7010859141301	28,696381711632	3125485913639,73	3127280790727,69	−0,000828260661993	0,00406003341739359	0,00436239085021	1,00057427137333	0.000828260661994695	0,999426058224998	-0.000828260661994	2154
Q66GT5	sp|Q66GT5|PTPM1_MOUSE Phosphatidylglycerophosphatase and protein-tyrosine phosphatase 1 OS=Mus musculus GN=Ptpmt1 PE=1 SV=1	1,00	1,00	0,995851120819905	0,999929810953105	24,3929941603799	24,8532287371371	24,9546627427534	24,7251231135384	24,8622015383108	24,6164760537761	55166876502,3863	55220507571,3211	−0,001401850110442	0,0121019848639692	0,0049783125914957	1,00097216069379	0.00140185011044371	0,999028783484731	-0.001401850110443	2155
P54797	sp|P54797|TNG2_MOUSE Transport and Golgi organization 2 homolog OS=Mus musculus GN=Tango2 PE=2 SV=1	1,00	1,00	0,997360755113061	0,999958878348632	26,2637346192989	26,6843310444171	26,9179630935536	26,984703803127	26,8006206385251	26,0840270683502	364579177036,44	364983202893,737	−0,001597906296528	0,0124534878037649	0,0178736446905314	1,00110819784218	0.00159790629653008	0,998893028900799	-0.001597906296530	2156
P55288	sp|P55288|CAD11_MOUSE Cadherin-11 OS=Mus musculus GN=Cdh11 PE=1 SV=1	1,00	1,00	0,994604568987658	0,999841939860314	26,102075650852	25,784338153794	25,7725191212397	25,8385679589886	26,1988563938474	25,6255477551962	174695618790,122	174930986344,796	−0,001942436017299	0,00722200678050678	0,0111941231423824	1,0013473008442	0.00194243601730109	0,998654511933008	-0.001942436017301	2157
Q00PI9	sp|Q00PI9|HNRL2_MOUSE Heterogeneous nuclear ribonucleoprotein U-like protein 2 OS=Mus musculus GN=Hnrnpul2 PE=1 SV=2	1,00	1,00	0,996319845264667	0,999929810953105	29,0919148705394	28,0758284429146	28,4245313453588	28,5978831612972	28,6488796693929	28,3498300207127	2458956575529,42	2462498540086,71	−0,002076611678464	0,0180964402164987	0,0056070005999054	1,00144043395989	0.00207661167846769	0,998561637905716	-0.002076611678467	2158
Q6PFD9	sp|Q6PFD9|NUP98_MOUSE Nuclear pore complex protein Nup98-Nup96 OS=Mus musculus GN=Nup98 PE=1 SV=2	1,00	1,00	0,993888040798493	0,999841939860314	24,1834776243377	24,6363202435582	24,5625402578922	24,6135674323953	24,5984115810658	24,1749746788762	41993445152,9358	42058102725,6498	−0,002219618323871	0,00993293021751752	0,0101773627286328	1,00153970631556	0.00221961832387094	0,998462660735417	-0.002219618323871	2159
Q9DB25	sp|Q9DB25|ALG5_MOUSE Dolichyl-phosphate beta-glucosyltransferase OS=Mus musculus GN=Alg5 PE=2 SV=1	1,00	1,00	0,994775545364437	0,999841939860314	24,4081168167823	24,5749811329927	25,1009475178655	24,946752196482	24,4430889369582	24,6992019327018	53059609314,8653	53148073186,8951	−0,002403368581955	0,0146425615796675	0,0101976115589178	1,00166725449305	0.00240336858195533	0,998335520617677	-0.002403368581955	2160
Q91VM9	sp|Q91VM9|IPYR2_MOUSE Inorganic pyrophosphatase 2, mitochondrial OS=Mus musculus GN=Ppa2 PE=1 SV=1;sp|Q91VM9-2|IPYR2_MOUSE Isoform 2 of Inorganic pyrophosphatase 2, mitochondrial OS=Mus musculus GN=Ppa2	1,00	1,00	0,988653004968496	0,998141007543842	29,8120687934487	29,4161611861033	29,6285973016842	29,6067416513827	29,6976512816979	29,5576191780415	7300342837919,85	7312970758954,8	−0,002493376121412	0,00668931084440443	0,0023985817256030	1,00172977095943	0.00249337612141418	0,998273215981414	-0.002493376121414	2161
Q9D1D4	sp|Q9D1D4|TMEDA_MOUSE Transmembrane emp24 domain-containing protein 10 OS=Mus musculus GN=Tmed10 PE=2 SV=1;sp|Q9D1D4-2|TMEDA_MOUSE Isoform 2 of Transmembrane emp24 domain-containing protein 10 OS=Mus musculus GN=Tmed10	1,00	1,00	0,99019498358568	0,998494568249453	28,2234557658422	28,2478477131411	28,1771173987933	27,9479032340272	28,2774605455253	28,4283424016431	1795204919424,55	1798370441295,84	−0,002541693677378	0,0012733188361746	0,0087070485933643	1,001763320631	0.00254169367738204	0,998239783195608	-0.002541693677382	2162
P40142	sp|P40142|TKT_MOUSE Transketolase OS=Mus musculus GN=Tkt PE=1 SV=1	1,00	1,00	0,991058627057175	0,998548920334855	32,9074754208366	33,5071865503134	33,3701779207136	33,2966158606149	33,1921566422038	33,3022847228181	278827185014746	279405638105115	−0,002989905534015	0,00944782821034172	0,0018642200622214	1,00207459358864	0.00298990553401259	0,997929701439484	-0.002989905534012	2163
Q8R164	sp|Q8R164|BPHL_MOUSE Valacyclovir hydrolase OS=Mus musculus GN=Bphl PE=1 SV=1	1,00	1,00	0,986933421981566	0,998141007543842	29,3880565211407	29,6661513123182	29,763614849229	29,6681665776184	29,6647193245074	29,4911659736167	7206041549734,29	7221019702644,98	−0,002995608642906	0,00658261610963013	0,0034183608750832	1,00207855489138	0.00299560864290852	0,997925756537515	-0.002995608642908	2164
Q91WC0	sp|Q91WC0|SETD3_MOUSE Histone-lysine N-methyltransferase setd3 OS=Mus musculus GN=Setd3 PE=2 SV=1	1,00	1,00	0,991339065590249	0,998599205218406	27,1551566172011	27,6401569056551	27,596320761955	27,6856496210637	27,30041308278	27,4118429955384	846078595683,875	847849148895,659	−0,003015912900270	0,00976765016677083	0,0072176484371866	1,00209265808261	0.00301591290027065	0,997911711990169	-0.003015912900270	2165
Q9JHG2	sp|Q9JHG2|RCAN2_MOUSE Calcipressin-2 OS=Mus musculus GN=Rcan2 PE=2 SV=1	1,00	1,00	0,996405052862061	0,999929810953105	23,2937395266692	22,4108797662297	23,8612171319506	23,591698233046	22,9507064919501	23,0297539046255	11767556606,2464	11792381722,8151	−0,003040337824149	0,031518119116078	0,0150706717528196	1,00210962372219	0.00304033782414083	0,997894817420923	-0.003040337824140	2166

Q8R5L3-2	sp|Q8R5L3-2|VPS39_MOUSE Isoform 2 of Vam6/Vps39-like protein OS=Mus musculus GN=Vps39;sp|Q8R5L3|VPS39_MOUSE Vam6/Vps39-like protein OS=Mus musculus GN=Vps39 PE=2 SV=1	1,00	0,98890506960226	0,998141007543842	27,2306522722493	26,9898009594195	26,7551771799298	26,8316021926563	27,1900125023832	26,9612195741421	527743814907,238	529012601418,087	−0,003464323203413	0,00880799600863148	0,0067228190770268	1,00240417125698	0.00346432320341514	0,997601594919577	-0.00346432320341	2167
Q4VBD2	sp|Q4VBD2|TAPT1_MOUSE Transmembrane anterior posterior transformation protein 1 OS=Mus musculus GN=Tapt1 PE=2 SV=2	1,00	0,987432110586026	0,998141007543842	24,6425366875427	25,1642937576338	24,9418514401016	25,0126127548663	24,8903374302592	24,8532287371371	66218604496,2251	66384292547,2291	−0,00360531269297	0,0105080378434206	0,0033468089660451	1,00250213746219	0.00360531269297921	0,997504107603676	-0.00360531269297	2168
Q9D154	sp|Q9D154|ILEUA_MOUSE Leukocyte elastase inhibitor A OS=Mus musculus GN=Serpinb1a PE=1 SV=1	1,00	0,983488192117909	0,997307422083825	28,0091322344659	27,6419884256892	27,6606396741905	27,7577253480226	27,8204849529863	27,741133248696	1149774299934,17	1152676649692,29	−0,003637174395787	0,00744661270908315	0,0015072868627484	1,00252427781547	0.00363717439578313	0,997482078118877	-0.00363717439578	2169
O09131	sp|O09131|GSTO1_MOUSE Glutathione S-transferase omega-1 OS=Mus musculus GN=Gsto1 PE=2 SV=2	1,00	0,995564645653206	0,999929810953105	28,9475574493059	28,7093526612442	29,6309744420806	29,8451084763157	28,7636310588687	28,6868977896118	4327285333398,29	4338482614589,87	−0,003728295318742	0,016442070461635	0,0222581441633736	1,00258759945991	0.00372829531874274	0,997419078930056	-0.00372829531874	2170
P53811	sp|P53811|PIPNB_MOUSE Phosphatidylinositol transfer protein beta isoform OS=Mus musculus GN=Pitpnb PE=1 SV=2	1,00	0,991069153141336	0,998548920334855	27,8507513155764	28,3770459552226	28,4660063045051	28,4722196117525	27,9817500837541	28,2478477131411	1822568450021,38	1827443545094,32	−0,00385383920782	0,0117786194485117	0,0086962855529765	1,00267484882276	0.003853839207815	0,997332286906467	-0.00385383920781	2171
P11983	sp|P11983|TCPA_MOUSE T-complex protein 1 subunit alpha OS=Mus musculus GN=Tcp1 PE=1 SV=3;sp|P11983-2|TCPA_MOUSE Isoform 2 of T-complex protein 1 subunit alpha OS=Mus musculus GN=Tcp1;sp|P28480|TCPA_RAT T-complex protein 1 subunit alpha OS=Rattus norvegi	1,00	0,987754786307043	0,998141007543842	32,0501774277855	32,4536789640953	32,5334327252068	32,504668068945	32,3520069298941	32,1886810722787	111580011234241	111880451933865	−0,003879384858114	0,00800945733029892	0,0048850402589383	1,0026926032387	0.00387938485812506	0,997314627404244	-0.00387938485812	2172
P56873	sp|P56873|SSA27_MOUSE Sjoegren syndrome/scleroderma autoantigen 1 homolog OS=Mus musculus GN=Sssca1 PE=2 SV=1	1,00	0,98680796087192	0,998141007543842	26,1217410182589	26,2677834736084	26,6443531342949	26,376255589271	26,2550580787104	26,410633198047	276264858674,08	277008938591,044	−0,00388046952061	0,0102353644374185	0,0031018500960203	1,00269335709411	0.00388046952060995	0,997313877592728	-0.00388046952061	2173
P40124	sp|P40124|CAP1_MOUSE Adenylyl cyclase-associated protein 1 OS=Mus musculus GN=Cap1 PE=1 SV=4	1,00	0,988888735351671	0,998141007543842	32,3120570949621	33,1617830500677	32,9255457365668	32,9774514497747	32,8872840950895	32,5465656838934	175699329309386	176398556480833	−0,005730070753232	0,0133719815006797	0,0069278526466976	1,00397968036757	0.00573007075322819	0,996036094708502	-0.00573007075322	2174
Q9QY36	sp|Q9QY36|NAA10_MOUSE N-alpha-acetyltransferase 10 OS=Mus musculus GN=Naa10 PE=1 SV=1	1,00	0,985121296816104	0,997872253123923	25,5599556845734	26,0415130488842	26,2599380141522	26,0654090296045	25,9217837339592	25,8861812107947	186893028347,977	187640047731,764	−0,00575501956111	0,0137991042888345	0,0036553368168510	1,00399704253492	0.00575501956110757	0,996018870210186	-0.00575501956110	2175
Q9R111	sp|Q9R111|GUAD_MOUSE Guanine deaminase OS=Mus musculus GN=Gda PE=1 SV=1	1,00	0,969241299924424	0,992417383342423	33,658865904478	33,4859878172862	33,5109505079196	33,4859878172862	33,7374582476704	33,4446452460359	372752114860730	374281924051638	−0,00590883709035	0,00278500888517393	0,0047226994080539	1,00410409258571	0.00590883709036168	0,995912682145192	-0.00590883709036	2176
Q8CG72	sp|Q8CG72|ARHL2_MOUSE Poly(ADP-ribose) glycohydrolase ARH3 OS=Mus musculus GN=Adprhl2 PE=1 SV=1;sp|Q8CG72-2|ARHL2_MOUSE Isoform 2 of Poly(ADP-ribose) glycohydrolase ARH3 OS=Mus musculus GN=Adprhl2	1,00	0,987752785845015	0,998141007543842	27,5571437909233	27,3712277656425	26,9469309955916	26,9028762084041	27,54141095012 6	27,4433741140667	712301787487,097	715242219544,942	−0,00594328822991	0,0114602494052136	0,0125978363436121	1,00412807058679	0.00594328822991235	0,995888900322864	-0.00594328822991	2177
O08585	sp|O08585|CLCA_MOUSE Clathrin light chain A OS=Mus musculus GN=Clta PE=1 SV=2	1,00	0,962812289616985	0,991306002794532	31,2180758996145	30,9568503354048	31,2604931538048	31,1864065676514	31,1238583488395	31,138693565225	33586314597281,4	33738233223906,3	−0,006510927391021	0,00527969721580584	0,0010492106876219	1,00452323002528	0.00651092739102201	0,995497137457771	-0.00651092739102	2178
Q8JZV9	sp|Q8JZV9|BDH2_MOUSE 3-hydroxybutyrate dehydrogenase type 2 OS=Mus musculus GN=Bdh2 PE=1 SV=1	1,00	0,989759183716449	0,998494568249453	23,8568543067188	23,8315975050895	23,9242273809443	23,6595996010165	24,5749811329927	23,3919312518076	23280759043,766	23388352881,0967	−0,006652167318424	0,00200596246214161	0,0259836651961814	1,00462157772126	0.00665216731841895	0,995399683001292	-0.00665216731841	2179
Q6PF93-2	sp|Q6PF93-2|PK3C3_MOUSE Isoform 2 of Phosphatidylinositol 3-kinase catalytic subunit type 3 OS=Mus musculus GN=Pik3c3;sp|Q6PF93|PK3C3_MOUSE Phosphatidylinositol 3-kinase catalytic subunit type 3 OS=Mus musculus GN=Pik3c3 PE=1 SV=1	1,00	0,979798578460379	0,995368341009772	28,1612225425277	27,8257406617401	27,680487545215	28,0023313775896	28,0407745775257	27,6388890090614	1294505972434,96	1300797067547,83	−0,00699428880422	0,00884073451833536	0,0079503028419880	1,004859842478	0.00699428880422037	0,995163661365927	-0.00699428880422	2180
Q7TSE6	sp|Q7TSE6|ST38L_MOUSE Serine/threonine-protein kinase 38-like OS=Mus musculus GN=Stk38l PE=1 SV=2;sp|Q7TSE6-2|ST38L_MOUSE Isoform 2 of Serine/threonine-protein kinase 38-like OS=Mus musculus GN=Stk38l	1,01	0,976664903998038	0,994783659375006	26,1438469895555	26,6292631829283	26,696871523607	26,58474053921 8	26,4531431572982	26,4478784720452	319490666880,887	321175665900,214	−0,00758880312540	0,0113881483662633	0,0029266471891994	1,00527401640798	0.00758880312540179	0,99475365291263 8	-0.00758880312540	2181
Q60649	sp|Q60649|CLPB_MOUSE Caseinolytic peptidase B protein homolog OS=Mus musculus GN=Clpb PE=1 SV=1	1,01	0,98607501152337	0,998141007543842	26,7455391951909	27,7178014437035	26,9150426236507	27,1769933127962	27,1751206599197	27,0421427414941	603569935638,953	606771978814,676	−0,00763351666619	0,0191463421253601	0,0028498728747854	1,00530517341347	0.00763351666619733	0,994722822926039	-0.00763351666619	2182
Q9ES74	sp|Q9ES74|NEK7_MOUSE Serine/threonine-protein kinase Nek7 OS=Mus musculus GN=Nek7 PE=2 SV=1	1,01	0,979069662033284	0,994969692078273	26,1876191399344	26,5491925988097	26,8719382927359	26,7117428695641	26,4519728460025	26,4619673766869	334616190010,73	336510222370,205	−0,00814308093501	0,0129009605830605	0,0055451197752087	1,00566031296757	0.00814308093501241	0,994371545844479	-0.00814308093501	2183
O55236	sp|O55236|MCE1_MOUSE mRNA-capping enzyme OS=Mus musculus GN=Rngtt PE=1 SV=1	1,01	0,978050071543696	0,99492812073887	22,9565167887036	23,2593273389152	22,6225346670564	23,0041717325947	22,7708085059644	23,0804133432428	9233705475,74522	9286224111,55282	−0,00818238300313	0,0138813612037378	0,0070282264415597	1,00568770965736	0.00818238300313686	0,994344457426753	-0.00818238300313	2184
O09061	sp|O09061|PSB1_MOUSE Proteasome subunit beta type-1 OS=Mus musculus GN=Psmb1 PE=1 SV=1	1,01	0,947701914155766	0,987969450701199	30,5343188057023	30,7635181652725	30,7450551800597	30,6271401858965	30,6509513772108	30,7820965217206	21114139497466,2	21236220663558,9	−0,00831758597042	0,00415024712097084	0,0027192236704556	1,00578196265623	0.00831758597042637	0,994251276249818	-0.00831758597042	2185

Q6PD19	sp|Q6PD19|CJ076_MOUSE UPF0668 protein C10orf76 homolog OS=Mus musculus PE=2 SV=2;sp|Q6PD19-3|CJ076_MOUSE Isoform 3 of UPF0668 protein C10orf76 homolog OS=Mus musculus	1,01	1,01	0,968631919718446	0,992417383342423	25,343368132376	25,7136461877525	25,6268016812449	25,7624652702272	25,4471000951404	25,4918299528731	126217443737,071	126959220414,969	−0,008453864422247	0,00757558489683366	0,0066740292786636	1,00587697433837	0.00845386442224843	0,994157362691158	-0.008453864422248	2186
Q62446	sp|Q62446|FKBP3_MOUSE Peptidyl-prolyl cis-trans isomerase FKBP3 OS=Mus musculus GN=Fkbp3 PE=1 SV=2	1,01	1,01	0,977922379858953	0,99492812073887	28,4869189419964	28,8941100043926	29,1075726323578	29,0238572486725	28,8830163528639	28,6000440221418	3315182727888,72	3335484986270,33	−0,008808155730353	0,0109375934256893	0,0074852582608608	1,00612402393715	0.00880815573035486	0,993913251456631	-0.008808155730354	2187
Q8BZB2	sp|Q8BZB2|COAC_MOUSE Phosphopantothenoylcysteine decarboxylase OS=Mus musculus GN=Ppcdc PE=2 SV=1	1,01	1,01	0,972135417517256	0,993288144763853	24,0624940540361	24,1764319247229	24,5806585090775	24,4526054481224	24,143043453479	24,2429818835158	34810845562,8901	35032554511,6787	−0,009159332878089	0,0112180304697177	0,0065069787700186	1,00636896189115	0.00915933287809305	0,99367134507092	-0.009159332878092	2188
Q8BH95	sp|Q8BH95|ECHM_MOUSE Enoyl-CoA hydratase, mitochondrial OS=Mus musculus GN=Echs1 PE=1 SV=1	1,01	1,01	0,968072078278364	0,992417383342423	30,8893105450994	31,3230324235924	31,3988833526896	31,2460357843299	31,138693565225	31,2460357843299	35613365087083,3	35846069680760,7	−0,009396182634548	0,00881093284467712	0,0019856953750308	1,00653419279836	0.00939618263454609	0,993508225706477	-0.009396182634546	2189
Q9DCX2	sp|Q9DCX2|ATP5H_MOUSE ATP synthase subunit d, mitochondrial OS=Mus musculus GN=Atp5h PE=1 SV=3	1,01	1,01	0,96089720663715	0,991306002794532	30,5018948562202	30,2058798248574	30,1667006838448	30,1150222657986	30,3419338493043	30,4385207524286	14303019550872,2	14403499145070,5	−0,010099587888441	0,0060500288039067	0,0054810264511479	1,00702506165505	0.0100995878884406	0,993023945557515	-0.010099587888440	2190
Q9Z1Z2	sp|Q9Z1Z2|STRAP_MOUSE Serine-threonine kinase receptor-associated protein OS=Mus musculus GN=Strap PE=1 SV=2	1,01	1,01	0,96177991347009	0,991306002794532	30,6939719257556	30,2152266398378	30,3249842800509	30,3961101691837	30,4662615796495	30,392909761289	16125037140288,2	16238842443965,6	−0,010146312870581	0,00824681970728487	0,0013628814083605	1,00705767699555	0.0101463128705811	0,992991784724182	-0.010146312870581	2191
Q9Z2A5	sp|Q9Z2A5|ATE1_MOUSE Arginyl-tRNA--protein transferase 1 OS=Mus musculus GN=Ate1 PE=1 SV=2	1,01	1,01	0,955937205462634	0,990217657408697	25,7355004334847	25,9650637893661	25,9023194601262	25,6547165375052	26,0218395960174	25,9481071296484	171462066813,355	172711386882,086	−0,010473764112859	0,00458675538044577	0,0075055458434080	1,00728627673718	0.0104737641128609	0,992766429062469	-0.010473764112860	2192
P28659-2	sp|P28659-2|CELF1_MOUSE Isoform 2 of CUGBP Elav-like family member 1 OS=Mus musculus GN=Celf1;sp|P28659-3|CELF1_MOUSE Isoform 3 of CUGBP Elav-like family member 1 OS=Mus musculus GN=Celf1;sp|P28659|CELF1_MOUSE CUGBP Elav-like family member 1 OS=Mus musc	1,01	1,01	0,977244751767958	0,994783659375006	26,0949756141312	26,7629318109403	26,9164428189902	26,4519728460025	26,7026272203015	26,642274809204	353606334321,259	356271277063,394	−0,010832058028492	0,016424711228291	0,0049182767968722	1,00753646777072	0.0108320580284953	0,992519905718751	-0.010832058028495	2193
Q80TI0-2	sp|Q80TI0-2|GRM1B_MOUSE Isoform 2 of GRAM domain-containing protein 1B OS=Mus musculus GN=Gramd1b;sp|Q80TI0-3|GRM1B_MOUSE Isoform 3 of GRAM domain-containing protein 1B OS=Mus musculus GN=Gramd1b;sp|Q80TI0|GRM1B_MOUSE GRAM domain-containing protein 1B O	1,01	1,01	0,962645386217671	0,991306002794532	26,8348651408146	26,4697443055486	26,4148721289321	26,5121165550575	26,76973984521	26,4605063694145	347197814509,901	349856039077,347	−0,011003528557068	0,00859127892422717	0,0062323077235346	1,00765622494254	0.0110035285570675	0,992401947456856	-0.011003528557067	2194
Q61545	sp|Q61545|EWS_MOUSE RNA-binding protein EWS OS=Mus musculus GN=Ewsr1 PE=1 SV=2	1,01	1,01	0,969039312947413	0,992417383342423	28,2874146092639	28,0173481149224	27,6226377001941	28,0692982272581	27,9723912373162	27,9088339896986	1411677940416,73	1422600738109,32	−0,011119826852124	0,0119506971896222	0,0028876884471310	1,00773745723431	0.0111198268521288	0,992321951338851	-0.011119826852128	2195
Q61335	sp|Q61335|BAP31_MOUSE B-cell receptor-associated protein 31 OS=Mus musculus GN=Bcap31 PE=1 SV=4	1,01	1,01	0,977334430368778	0,994783659375006	28,4070567271281	28,8639896558021	28,8632336136022	29,0763948194066	28,8042521530299	28,2774605455253	2945874006816,55	2969364796551,19	−0,011458615667579	0,00918074789250717	0,0141428814861083	1,00797413252579	0.0114586156675806	0,992088951225554	-0.011458615667580	2196
Q149F3	sp|Q149F3|ERF3B_MOUSE Eukaryotic peptide chain release factor GTP-binding subunit ERF3B OS=Mus musculus GN=Gspt2 PE=1 SV=1	1,01	1,01	0,964227877144257	0,991306002794532	26,2409001964166	25,9516347704544	25,8014926834204	26,242967719549	25,8205849296101	25,9546021761348	195340341805,855	196917679625,542	−0,011602718575606	0,00859082370824525	0,0082996229741474	1,00807481857104	0.0116027185756045	0,991989861841321	-0.011602718575604	2197
P51660	sp|P51660|DHB4_MOUSE Peroxisomal multifunctional enzyme type 2 OS=Mus musculus GN=Hsd17b4 PE=1 SV=3	1,01	1,01	0,734164081857628	0,924701393764781	29,7016909671295	29,6401923534164	29,6861147613	29,6861147613	29,656852932955	29,709391036643	7728990389473,79	7792006971483,74	−0,011714995860086	0,00107739638905111	0,0008868653559480	1,00815327472729	0.011714995860086	0,991912663548612	-0.011714995860085	2198
P35279	sp|P35279|RAB6A_MOUSE Ras-related protein Rab-6A OS=Mus musculus GN=Rab6a PE=1 SV=4	1,01	1,01	0,947620769590378	0,987969450701199	32,1255304366215	32,390014054121	32,5465656838934	32,3444630888764	32,390014054121	32,3520069298941	112507018790824	113424820352173	−0,011721367380109	0,00657756631618204	0,0007544081036423	1,00815772714639	0.0117213673801026	0,991908282874072	-0.011721367380102	2199
Q9WTX6	sp|Q9WTX6|CUL1_MOUSE Cullin-1 OS=Mus musculus GN=Cul1 PE=1 SV=1	1,01	1,01	0,948935254532381	0,988334326693467	30,4016042683483	30,2298776072897	29,9610005225932	30,2081872493644	30,1853711148321	30,2236511949269	13019822669260,1	13127580566988,7	−0,011891250798188	0,00735424558989917	0,0006375396330624	1,00827644895525	0.011891250798194	0,991791488372231	-0.011891250798194	2200
Q8C0J2-2	sp|Q8C0J2-2|A16L1_MOUSE Isoform 2 of Autophagy-related protein 16-1 OS=Mus musculus GN=Atg16l1;sp|Q8C0J2-5|A16L1_MOUSE Isoform 5 of Autophagy-related protein 16-1 OS=Mus musculus GN=Atg16l1;sp|Q8C0J2|A16L1_MOUSE Autophagy-related protein 16-1 OS=Mus mus	1,01	1,01	0,964578938532731	0,991306002794532	26,5703771561402	26,8512780500818	26,596578656695	26,8812927262519	26,3529925413592	26,8090056281754	383553372808,031	386770358615,93	−0,012049885686702	0,00581749344189613	0,0107355509984072	1,00838732243272	0.0120498856867052	0,991682439627972	-0.012049885686705	2201
Q99LX0	sp|Q99LX0|PARK7_MOUSE Protein DJ-1 OS=Mus musculus GN=Park7 PE=1 SV=1;sp|Q99497|PARK7_HUMAN Protein DJ-1 OS=Homo sapiens GN=PARK7 PE=1 SV=2	1,01	1,01	0,943762532232717	0,986987866859505	31,7285609258969	32,0369458698543	31,7920975405357	31,7533387554372	31,8280562746036	32,0014621620972	68136542023695,1	68712510187593,2	−0,012144056634890	0,00511211114704201	0,0039951944559919	1,00845314638506	0.0121440566348906	0,991617710336507	-0.012144056634890	2202
O35864	sp|O35864|CSN5_MOUSE COP9 signalosome complex subunit 5 OS=Mus musculus GN=Cops5 PE=1 SV=3	1,01	1,01	0,945154158807532	0,987138173908166	30,1695834981336	29,8158570876731	29,8391442128287	30,0459928945559	29,8993775648934	29,9047847149208	10079535198128,3	10165814877166,7	−0,012296751422083	0,00660769696411608	0,0027756701418507	1,00855988667557	0.0122967514220848	0,991512763110393	-0.012296751422084	2203

O55042-2	sp|O55042-2|SYUA_MOUSE Isoform 2 of Alpha-synuclein OS=Mus musculus GN=Snca;sp|O55042|SYUA_MOUSE Alpha-synuclein OS=Mus musculus GN=Snca PE=1 SV=2;sp|P37840-2|SYUA_HUMAN Isoform 2-4 of Alpha-synuclein OS=Homo sapiens GN=SNCA;sp|P37840|SYUA_HUMAN Alpha-	1,01	0,979036734090223	0,994969692078273	32,8872840950895	32,6843873490794	32,209626068356	32,0900263529684	32,8095502245621	32,9074754208366	142985713772409	144218505701810	−0,012385289668429	0,0106707281661731	0,013691629583977	1,00862178393125	0.0123852896684317	0,991451915803718	-0.0123852896684312204
Q8K1R3	sp|Q8K1R3|PNPT1_MOUSE Polyribonucleotide nucleotidyltransferase 1, mitochondrial OS=Mus musculus GN=Pnpt1 PE=1 SV=1;sp|Q8K1R3-2|PNPT1_MOUSE Isoform 2 of Polyribonucleotide nucleotidyltransferase 1, mitochondrial OS=Mus musculus GN=Pnpt1	1,01	0,951063972255892	0,988522869262536	27,924640903582	27,7681093764479	27,4236219219812	27,7229066479733	27,7318880781206	27,6889296233201	1077276702372,32	1087143557688,35	−0,013153602471941	0,00925153752629049	0,0008175778125318	1,00915907240387	0.01315360247194	0,99092405483503	-0.01315360247193	2205
Q9D6Y9	sp|Q9D6Y9|GLGB_MOUSE 1,4-alpha-glucan-branching enzyme OS=Mus musculus GN=Gbe1 PE=2 SV=1	1,01	0,955043275144438	0,989938667705093	27,7875488622476	27,4741157907615	27,2638746068392	27,6254440730023	27,5128434547509	27,4149626112445	884698801391,858	892908586938,264	−0,013326115975874	0,00957980558368084	0,0038275832854405	1,00927975208454	0.0133261159758758	0,990805569946911	-0.01332611597587	2206
Q8C3P7	sp|Q8C3P7|MTA70_MOUSE N6-adenosine-methyltransferase subunit METTL3 OS=Mus musculus GN=Mettl3 PE=1 SV=2;sp|Q8C3P7-2|MTA70_MOUSE Isoform 2 of N6-adenosine-methyltransferase subunit METTL3 OS=Mus musculus GN=Mettl3;sp|Q86U44|MTA70_HUMAN N6-adenosine-methy	1,01	0,895233719160243	0,977357990376388	23,5344531294744	23,6309486811925	23,5596170779061	23,7144489157279	23,5193424313764	23,5193424313764	17317896719,548	17480956514,0526	−0,013520404081706	0,0021232087297866	0,0047762455984067	1,00941568119647	0.0135204040817017	0,990672146894621	-0.0135204040817 01	2207
Q3UHD6-2	sp|Q3UHD6-2|SNX27_MOUSE Isoform 2 of Sorting nexin-27 OS=Mus musculus GN=Snx27;sp|Q3UHD6|SNX27_MOUSE Sorting nexin-27 OS=Mus musculus GN=Snx27 PE=1 SV=2	1,01	0,944671388822302	0,987138173908166	28,5655362258062	28,9686502897513	28,9577992893341	28,8709870434371	28,7807657090108	28,8685229978762	3318924614077,44	3350370044717,07	−0,013604587994207	0,00796616089627425	0,0017819905966922	1,00947458417894	0.0136045879942086	0,990614341036981	-0.0136045879942 08	2208
P50440-3	sp|P50440-3|GATM_HUMAN Isoform 3 of Glycine amidinotransferase, mitochondrial OS=Homo sapiens GN=GATM;sp|P50440-2|GATM_HUMAN Isoform 2 of Glycine amidinotransferase, mitochondrial OS=Homo sapiens GN=GATM;sp|Q9D964|GATM_MOUSE Glycine amidinotransferase,	1,01	0,910151724392356	0,980429909122681	27,4732502744015	27,4312593139902	27,4766700566273	27,3594544203214	27,4170667080726	27,6333084505179	843135244956,672	851225738495,388	−0,013777058497061	0,000920910771588417	0,0052559724349078	1,00959572451408	0.0137777058497061	0,990495478258192	-0.0137777058497 05	2209
O35239	sp|O35239|PTN9_MOUSE Tyrosine-protein phosphatase non-receptor type 9 OS=Mus musculus GN=Ptpn9 PE=1 SV=2	1,01	0,964922402886022	0,991306002794532	27,6904097335274	28,3139767888204	28,2437154901872	28,2518043492866	28,092191112154	27,9328192440591	1570948465200,37	1586056033352,67	−0,013807886583579	0,0121621299177175	0,0056774535816631	1,00961684516517	0.0138078865835741	0,990474757615994	-0.0138078865835 73	2210
Q8CBW3-2	sp|Q8CBW3-2|ABI1_MOUSE Isoform 2 of Abl interactor 1 OS=Mus musculus GN=Abi1;sp|Q8CBW3-4|ABI1_MOUSE Isoform 4 of Abl interactor 1 OS=Mus musculus GN=Abi1;sp|Q8CBW3-3|ABI1_MOUSE Isoform 3 of Abl interactor 1 OS=Mus musculus GN=Abi1;sp|Q8CBW3|ABI1_MOUSE	1,01	0,956451070740535	0,990217657408697	29,6389646388719	30,1559838428577	30,108137787763	29,8788419232647	29,9670200277696	30,0859791046874	10346769105265,4	10446418955307,7	−0,013828129164901	0,00953335212776209	0,0034675950071083	1,00963101128753	0.0138281291648989	0,990460860274834	-0.0138281291648 99	2211
Q8K2B3	sp|Q8K2B3|SDHA_MOUSE Succinate dehydrogenase [ubiquinone] flavoprotein subunit, mitochondrial OS=Mus musculus GN=Sdha PE=1 SV=1	1,01	0,957406612066705	0,990217657408697	30,3537800086882	30,8856809268552	30,5524835884731	30,727262227369	30,6742077219904	30,4196223089356	19419810899585,4	19609411642705,7	−0,014017097232282	0,0087840867953302	0,0053730812394301	1,00976326412758	0.0140170972322801	0,990331135549861	-0.0140170972322 80	2212
Q9ERG2	sp|Q9ERG2|STRN3_MOUSE Striatin-3 OS=Mus musculus GN=Strn3 PE=1 SV=1	1,01	0,954163445007183	0,989678056389876	28,7194223068834	28,4508773233213	28,1529200921064	28,4585732117269	28,5243734839213	28,3695269666755	2248025161578,54	2270053587809,85	−0,014068171394226	0,00996370042158097	0,0027315034981684	1,00979901230992	0.0140681713942264	0,990296076555372	-0.0140681713942 26	2213
Q9CR56	sp|Q9CR56|KBRS2_MOUSE NF-kappa-B inhibitor-interacting Ras-like protein 2 OS=Mus musculus GN=Nkiras2 PE=2 SV=1	1,01	0,901851313902765	0,977644369603516	22,9837162468125	23,0507037791687	23,2244008906573	23,0327998585375	23,160204023241	23,0956367782024	10622854892,2471	10728971683,2663	−0,014340265280299	0,00538078710154259	0,0027582027905238	1,00998947948509	0.0143402652803009	0,99010932322762	-0.0143402652803 01	2214
Q9JLZ3	sp|Q9JLZ3|AUHM_MOUSE Methylglutaconyl-CoA hydratase, mitochondrial OS=Mus musculus GN=Auh PE=1 SV=1	1,01	0,967925636848759	0,992417383342423	30,2744604699913	30,3912180432188	30,8504306917222	30,8504306917222	30,2654579925308	30,4300715036396	17713881877445,6	17891020638973,8	−0,014355288360851	0,00998121026183322	0,0098855261528750	1,00999999676828	0.0143552883608 5	0,99009901306903	-0.0143552883608 49	2215
O08756	sp|O08756|HCD2_MOUSE 3-hydroxyacyl-CoA dehydrogenase type-2 OS=Mus musculus GN=Hsd17b10 PE=1 SV=4	1,01	0,943916926566423	0,986987866859505	30,0602349868501	30,2570757598888	30,5147582056784	30,3808159827936	30,192477358805	30,2888262852398	14102261880055,2	14244232572262,5	−0,014451319654261	0,0075283745559501	0,0031094713656884	1,01006722846412	0.0144513196542569	0,990033110489662	-0.0144513196542 56	2216
Q9D5R2	sp|Q9D5R2|WDR20_MOUSE WD repeat-containing protein 20 OS=Mus musculus GN=Wdr20 PE=2 SV=1	1,01	0,949103944858454	0,988334326693467	24,4418972929147	24,7178927598584	24,9148170158252	24,6502909577635	24,8532287371371	24,6012207533644	52892939047,1791	53426897223,29	−0,014491092470189	0,00962107103653423	0,0054087096354086	1,01009507480071	0.0144910924701895	0,990005817222002	-0.0144910924701 89	2217
Q62241	sp|Q62241|RU1C_MOUSE U1 small nuclear ribonucleoprotein C OS=Mus musculus GN=Snrpc PE=2 SV=1	1,01	0,979186960677164	0,994969692078273	25,5041690285268	25,272388648783	24,2210527725757	24,752533055932	25,1535971658607	25,1239866486859	71947569067,5238	72731393935,6885	−0,015632283928892	0,0273524910200576	0,0089363142267114	1,01089438987757	0.0156322839288925	0,98922301875779	-0.0156322839288 92	2218
P31324	sp|P31324|KAP3_MOUSE cAMP-dependent protein kinase type II-beta regulatory subunit OS=Mus musculus GN=Prkar2b PE=1 SV=3	1,01	0,924805826017671	0,983296707494544	32,0248903492098	31,625103247153	31,8110328188897	31,8078616968354	31,8676070981556	31,8186731138519	65977978185283,5	66710310444446,4	−0,015925186126548	0,00628704947796204	0,0010001244923841	1,01109964687166	0.0159251861265508	0,989022202800679	-0.0159251861265 50	2219
Q6NVF9	sp|Q6NVF9|CPSF6_MOUSE Cleavage and polyadenylation specificity factor subunit 6 OS=Mus musculus GN=Cpsf6 PE=2 SV=1	1,01	0,963265015889211	0,991306002794532	27,8064691473181	28,525661745392	27,869672887315	28,1697161700063	27,9380967665236	28,1272472497798	1546890536846,55	1564133942605,15	−0,015992950808187	0,0141886466752967	0,0043915653032139	1,0114714024546	0.0159929508081897	0,988975748630657	-0.0159929508081 89	2220

O35495-2	sp|O35495-2|CDK14_MOUSE Isoform 2 of Cyclin-dependent kinase 14 OS=Mus musculus GN=Cdk14;sp|O35495|CDK14_MOUSE Cyclin-dependent kinase 14 OS=Mus musculus GN=Cdk14 PE=2 SV=2;sp|O35495-3|CDK14_MOUSE Isoform 3 of Cyclin-dependent kinase 14 OS=Mus musculus	1,01	0,934501575977669	0,98467134375593	26,7489665802186	26,4553495397667	26,410633198047	26,5351193087617	26,7069466343535	26,4067100219599	335308365479,078	339110546947,282	−0,016267178646154	0,00692558476543021	0,0056739339356855	1,01133935761719	0.0162671786461549	0,988787781735391	-0.016267178646154	2221
Q9CRC9	sp|Q9CRC9|GNPI2_MOUSE Glucosamine-6-phosphate isomerase 2 OS=Mus musculus GN=Gnpda2 PE=2 SV=1;sp|Q9CRC9-2|GNPI2_MOUSE Isoform 2 of Glucosamine-6-phosphate isomerase 2 OS=Mus musculus GN=Gnpda2	1,01	0,964028135894051	0,991306002794532	27,0148666296592	27,6870999533187	27,7433277429496	27,6736691686645	27,4433741140667	27,3623338424334	861348288043,048	871189806865,451	−0,016390361812996	0,0147487561517066	0,0058749374011567	1,01142571356909	0.0163903618129967	0,988703358619618	-0.016390361812996	2222
Q8CCN5	sp|Q8CCN5|BCAS3_MOUSE Breast carcinoma-amplified sequence 3 homolog OS=Mus musculus GN=Bcas3 PE=1 SV=2	1,01	0,975885537952122	0,994649943013952	27,219419790962	25,9245627621526	26,6292631829283	26,6089362359476	26,6737413947028	26,5247335525472	353476171273,164	357524738182,314	−0,016430107059913	0,0243793145027667	0,0028085381179075	1,01145357802923	0.0164301070599154	0,988676120903596	-0.016430107059915	2223
A6H630	sp|A6H630|CF211_MOUSE UPF0364 protein C6orf211 homolog OS=Mus musculus PE=2 SV=1;sp|A6H630-2|CF211_MOUSE Isoform 2 of UPF0364 protein C6orf211 homolog OS=Mus musculus;sp|A6H630-4|CF211_MOUSE Isoform 4 of UPF0364 protein C6orf211 homolog OS=Mus musculus	1,01	0,936190934618512	0,985475568699774	28,381044130583	27,9775310134962	28,0241091157644	28,1148719832427	28,2613796269497	28,0407745775257	1643027052158,91	1661943354815,84	−0,016514976346335	0,00784832961655331	0,0039896815567727	1,01151308046455	0.0165149763463341	0,988617961856452	-0.016514976346334	2224
Q91YW3	sp|Q91YW3|DNJC3_MOUSE DnaJ homolog subfamily C member 3 OS=Mus musculus GN=Dnajc3 PE=1 SV=1;sp|Q13217|DNJC3_HUMAN DnaJ homolog subfamily C member 3 OS=Homo sapiens GN=DNAJC3 PE=1 SV=1;sp|Q27968|DNJC3_BOVIN DnaJ homolog subfamily C member 3 OS=Bos taurus	1,01	0,965971899176077	0,991306002794532	26,9078396230827	27,3283963991727	27,5285613999933	27,5796655021705	27,3519448291009	26,868788726629	686541474041,65	694737342046,648	−0,017120767734023	0,0116241256708982	0,0133131318743758	1,01193790661582	0.017120767734023	0,988202925754856	-0.017120767734023	2225
Q64433	sp|Q64433|CH10_MOUSE 10 kDa heat shock protein, mitochondrial OS=Mus musculus GN=Hspe1 PE=1 SV=2	1,01	0,936577563922748	0,98548775757167	31,6520009366872	31,6602286177643	31,3519919643443	31,3519919643443	31,63710827341	31,7107286381385	50588252754417	51192268222820,3	−0,017123519167678	0,0055659964439766	0,0060023674757877	1,0119398365335	0.0171235191676786	0,988201041106945	-0.017123519167678	2226
Q8BG92	sp|Q8BG92|CLVS2_MOUSE Clavesin-2 OS=Mus musculus GN=Clvs2 PE=2 SV=1;sp|Q8BG92-2|CLVS2_MOUSE Isoform 2 of Clavesin-2 OS=Mus musculus GN=Clvs2	1,01	0,944349572177161	0,987138173908166	25,9004504146625	26,0912494688688	26,4513611202461	26,2127010125378	26,0532668033667	26,2127010125378	226879982069,445	229588927750,873	−0,017123744020264	0,0106991496523697	0,003518767008557	1,01193999425035	0.0171237440202655	0,988200887089958	-0.017123744020265	2227
Q9D7H3	sp|Q9D7H3|RTCA_MOUSE RNA 3-terminal phosphate cyclase OS=Mus musculus GN=RtcA PE=2 SV=2	1,01	0,96162877301302	0,991306002794532	27,0364602305821	26,2771103257199	26,9842139477044	26,8802456280836	26,696871523607	26,7568583343213	421012418577,344	426122128466,706	−0,017404183421344	0,0158460381316083	0,0034915203565428	1,01213672011536	0.0174041834213431	0,988008813558338	-0.017404183421343	2228
P17439	sp|P17439|GLCM_MOUSE Glucosylceramidase OS=Mus musculus GN=Gba PE=1 SV=1	1,01	0,943430003614558	0,986987866859505	24,0742215615509	24,4665538370577	24,620547219318	24,4468590871809	24,2934651158925	24,457410707647	39010923722,6375	39487301266,4481	−0,017510611413617	0,0115509544202259	0,0037607653577107	1,01221138846128	0.0175106114136174	0,987935930576867	-0.017510611413617	2229
P61759	sp|P61759|PFD3_MOUSE Prefoldin subunit 3 OS=Mus musculus GN=Vbp1 PE=2 SV=2	1,01	0,941369774539392	0,986704967868576	28,3974533755861	28,8233279788742	28,6581410656887	28,7164765416841	28,4205387528905	28,7785417083211	2705498970161,81	2738739792996,7	−0,017617510284620	0,00750060153918491	0,0066801681200680	1,01228639271406	0.0176175102846178	0,987862730544942	-0.017617510284617	2230
Q924D0	sp|Q924D0|RT4I1_MOUSE Reticulon-4-interacting protein 1, mitochondrial OS=Mus musculus GN=Rtn4ip1 PE=1 SV=2	1,01	0,931081732423722	0,98433518830764	25,502807308767	25,8742929382005	25,4142056413629	25,5890196137344	25,6003102586315	25,6387500127636	130821804451,605	132435286820,326	−0,017684554271905	0,00953654639805773	0,0010180291814568	1,0123343612316	0.0176845542719073	0,987816824296156	-0.017684554271907	2231
Q8C0L8	sp|Q8C0L8|COG5_MOUSE Conserved oligomeric Golgi complex subunit 5 OS=Mus musculus GN=Cog5 PE=2 SV=3	1,01	0,961766922619156	0,991306002794532	26,1622102512306	25,9760107033085	25,3979425844979	25,8783648256611	25,6387500127636	26,0564348417916	167690833864,118	169793680599,986	−0,017978933492461	0,0154196197426433	0,0081057303214497	1,01254002194045	0.0179789334924548	0,987615282686391	-0.017978933492455	2232
P37040	sp|P37040|NCPR_MOUSE NADPH--cytochrome P450 reductase OS=Mus musculus GN=Por PE=1 SV=2	1,01	0,922809479076716	0,982555061114055	29,7887479080092	29,6149568203859	29,3608168934757	29,5484224910215	29,6661513123182	29,5874435150517	7079142312240,54	7168176678056,66	−0,018031618474972	0,00727381945817635	0,0020259312298188	1,01257699900483	0.0180316184749717	0,987579217168478	-0.018031618474971	2233
P62077	sp|P62077|TIM8B_MOUSE Mitochondrial import inner membrane translocase subunit Tim8 B OS=Mus musculus GN=Timm8b PE=3 SV=1	1,01	0,945732120967691	0,987138173908166	26,9663839376261	26,8127695771732	26,5628276441229	26,517535165276	26,8651963070805	26,9970599843299	427260778527,768	432679808403,705	−0,018182909693026	0,0076056834070229	0,0092457224804274	1,01268319056715	0.0181829096930284	0,987475658048547	-0.018182909693028	2234
Q8VCH5	sp|Q8VCH5|RABEK_MOUSE Rab9 effector protein with kelch motifs OS=Mus musculus GN=Rabepk PE=2 SV=2	1,01	0,968977914511084	0,992417383342423	23,735889620616	22,7520392588109	23,2715183887361	23,1581133693146	23,5843529479607	23,0551430075932	12552040631,0216	12712731072,574	−0,018352069986469	0,0211662660955542	0,0120596668578631	1,0128019376511	0.0183520699864721	0,987359880372278	-0.018352069986472	2235
P23818	sp|P23818|GRIA1_MOUSE Glutamate receptor 1 OS=Mus musculus GN=Gria1 PE=1 SV=1	1,01	0,957571640003945	0,990217657408697	27,7146979344159	26,9699393539991	27,064290140851	27,1591850873045	27,2571389591788	27,3712277656425	682919260447,608	691768549590,778	−0,018574401869711	0,0148810514497318	0,0038926560440917	1,0129580312867	0.0185744018697104	0,987207731330942	-0.018574401869710	2236
Q63943	sp|Q63943|MEF2D_MOUSE Myocyte-specific enhancer factor 2D OS=Mus musculus GN=Mef2d PE=1 SV=2;sp|Q63943-2|MEF2D_MOUSE Isoform Muscle of Myocyte-specific enhancer factor 2D OS=Mus musculus GN=Mef2d	1,01	0,960223588298135	0,991306002794532	24,9952475903761	25,6347612167554	25,6369206672453	25,1293167074687	25,52853315215	25,6487332316027	109842111846,556	111303628758,311	−0,019069360265586	0,0145481792415432	0,0106902265314475	1,01330561555296	0.0190693602655923	0,986869099165414	-0.019069360265592	2237

Q05CL8	sp|Q05CL8|LARP7_MOUSE La-related protein 7 OS=Mus musculus GN=Larp7 PE=1 SV=2;sp|Q05CL8-2|LARP7_MOUSE Isoform 2 of La-related protein 7 OS=Mus musculus GN=Larp7	1,01	0,854487416015268	0,963607051252994	24,6912518557112	24,7428706679691	24,6569155403246	24,846359962555	24,6522337538154	24,6324302907486	53183428371,9857	53897036705,0055	−0,019229173945362	0,00175187864171812	0,0047838682155627	1,01341787009345	0.0192291739453631	0,986759785386243	-0.01922917394536	2238
Q9R0N7	sp|Q9R0N7|SYT7_MOUSE Synaptotagmin-7 OS=Mus musculus GN=Syt7 PE=1 SV=1	1,01	0,913298595709061	0,980665300581806	26,0168115282551	26,3276898796021	26,2614070104605	26,0820246055589	26,3537858638036	26,2102331083515	239535980146,941	242762117197,832	−0,019300931808776	0,00624916784346639	0,0051860005165887	1,01346827749598	0.0193009318087765	0,986710706397976	-0.01930093180877	2239
Q9Z223-1	sp|Q9Z223-1|MOC2B_MOUSE Isoform 2 of Molybdopterin synthase catalytic subunit OS=Mus musculus GN=Mocs2;sp|Q9Z223|MOC2B_MOUSE Molybdopterin synthase catalytic subunit OS=Mus musculus GN=Mocs2 PE=2 SV=3;sp|Q9Z223-3|MOC2B_MOUSE Isoform 3 of Molybdopterin s	1,01	0,929906201284153	0,98433518830764	23,0041717325947	23,4559970658618	23,0507037791687	23,0804133432428	23,2006453878741	23,2704101887496	11553922876,1856	11711334532,8004	−0,01952271387658	0,0107258166457433	0,0041455211939494	1,01362408753301	0.0195227138765915	0,986559033372847	-0.01952271387659	2240
Q8C729-2	sp|Q8C729-2|F126B_MOUSE Isoform 2 of Protein FAM126B OS=Mus musculus GN=Fam126b;sp|Q8C729|F126B_MOUSE Protein FAM126B OS=Mus musculus GN=Fam126b PE=2 SV=1	1,01	0,953836202660271	0,989678056389876	27,1783471400919	27,8242150678396	27,9148680147052	27,6483028203886	27,6030325495586	27,7067011958313	1008155366915,99	1021893970496,05	−0,01952752329313	0,0145311007303016	0,0018794807846401	1,01362746658988	0.0195275232931488	0,986555744552059	-0.01952752329314	2241
Q99J83	sp|Q99J83|ATG5_MOUSE Autophagy protein 5 OS=Mus musculus GN=Atg5 PE=1 SV=1;sp|Q9H1Y0|ATG5_HUMAN Autophagy protein 5 OS=Homo sapiens GN=ATG5 PE=1 SV=2	1,01	0,875669903318449	0,972992598310849	27,2232737570987	26,9234814664797	27,0687747291807	27,0645280083663	27,1109039589626	27,081221549861	571678906014,116	579569354462,345	−0,01977625415598	0,00553784604162327	0,0008672216617444	1,01380223822363	0.0197762541559887	0,986385670002257	-0.01977625415598	2242
P48036	sp|P48036|ANXA5_MOUSE Annexin A5 OS=Mus musculus GN=Anxa5 PE=1 SV=1	1,01	0,930664187227224	0,98433518830764	31,9210716119724	32,2621420111148	32,4536789640953	32,2966368508442	32,1724065672547	32,209626068356	97638942330929,5	99008137747201,2	−0,02009044180129	0,00837504817581989	0,0019784036633918	1,01402304637458	0.0200904418012943	0,98617088001627	-0.02009044180129	2243
Q9ERL9	sp|Q9ERL9|GCYA3_MOUSE Guanylate cyclase soluble subunit alpha-3 OS=Mus musculus GN=Gucy1a3 PE=2 SV=2	1,01	0,930876154448719	0,98433518830764	27,9512595440036	27,7242460573172	27,4384765453677	27,8355439715928	27,7146979344159	27,6060239625494	1076418793853,68	1091697881325,41	−0,02033417195057	0,00927468213620409	0,0041420974297972	1,01419437077741	0.0203341719505768	0,986004289526348	-0.02033417195057	2244
Q9DC50	sp|Q9DC50|OCTC_MOUSE Peroxisomal carnitine O-octanoyltransferase OS=Mus musculus GN=Crot PE=1 SV=1	1,01	0,912035468827564	0,980665300581806	26,4538794096732	26,8162660140065	26,6055017936493	26,7099050263889	26,4937458641267	26,7143626330946	365749949622,701	370951751489,625	−0,02037388665761	0,00683546981212995	0,0047338281831781	1,01422229004348	0.0203738866576129	0,98597714703857	-0.02037388665761	2245
Q8BR63	sp|Q8BR63|F177A_MOUSE Protein FAM177A1 OS=Mus musculus GN=Fam177a1 PE=2 SV=1	1,01	0,964198546568406	0,991306002794532	25,4471000951404	26,3382310811099	26,0372835360317	25,9073997517755	25,6777150180066	26,2799304014618	184491932052,966	187119831191,449	−0,02040473757570	0,0174751700963732	0,0117094199664813	1,01424397863495	0.0204047375757026	0,985956062904983	-0.02040473757570	2246
Q91W96	sp|Q91W96|APC4_MOUSE Anaphase-promoting complex subunit 4 OS=Mus musculus GN=Anapc4 PE=1 SV=1	1,01	0,919600287375684	0,98176599643834	25,5637107751137	25,3750798339753	25,1063810759423	25,3853556236696	25,2712628519499	25,4312369546124	102015496144,526	103477338785,221	−0,02052654250912	0,00906684976081386	0,003247895260173	1,01432961359737	0.0205265425091359	0,985872823384749	-0.02052654250913	2247
Q3URE1	sp|Q3URE1|ACSF3_MOUSE Acyl-CoA synthetase family member 3, mitochondrial OS=Mus musculus GN=Acsf3 PE=2 SV=2	1,01	0,939575771703529	0,986144884061661	26,76973984521	27,3951987839827	27,235518194489	27,1705289713067	27,0645280083663	27,2091856375552	608027293113,21	616966690030,248	−0,02105651573705	0,0119768392408527	0,0027587915560660	1,01470229546978	0.021056515737051	0,985510730058053	-0.02105651573705	2248
P80314	sp|P80314|TCPB_MOUSE T-complex protein 1 subunit beta OS=Mus musculus GN=Cct2 PE=1 SV=4	1,01	0,938686403553527	0,985646114383563	32,0093513947963	32,5868757685581	32,5416809718635	32,4715364843887	32,3737547971211	32,3364067192113	115385834565902	117082430116797	−0,02105847393421	0,00991939719201969	0,0021541351235810	1,01470367274525	0.0210584739342118	0,985509392406678	-0.02105847393421	2249
Q9D0B5	sp|Q9D0B5|TSTD3_MOUSE Thiosulfate sulfurtransferase/rhodanese-like domain-containing protein 3 OS=Mus musculus GN=Tstd3 PE=1 SV=1	1,01	0,954355772817468	0,989678056389876	25,6522026352665	25,6128898625493	25,2380141227444	25,0924879023731	25,6338439500692	25,8205849296101	118839008004,582	120587193697,014	−0,02106823424180	0,00896550936236509	0,0148210075507263	1,01471053757336	0.0210682342418045	0,98550272513328	-0.02106823424180	2250
P47791-2	sp|P47791-2|GSHR_MOUSE Isoform Cytoplasmic of Glutathione reductase, mitochondrial OS=Mus musculus GN=Gsr;sp|P47791|GSHR_MOUSE Glutathione reductase, mitochondrial OS=Mus musculus GN=Gsr PE=1 SV=3	1,01	0,94909183662077	0,988334326693467	28,4815245373331	28,9871137767746	29,258547537147	28,952674136364	28,8732918283341	28,9450977968638	3589600211104,57	3642487417023,19	−0,02110081453491	0,0136409781018726	0,0015146196974793	1,01473345297758	0.0211008145349156	0,985480469837328	-0.02110081453491	2251
P70372	sp|P70372|ELAV1_MOUSE ELAV-like protein 1 OS=Mus musculus GN=Elavl1 PE=1 SV=2	1,01	0,957153452585991	0,990217657408697	28,3623185321326	29,151901871807	29,1759515219542	28,9317927292942	28,808297486709	28,9940526281682	3545583907404,17	3597934144970,44	−0,02114554191416	0,0160213621422766	0,0032701593956902	1,01476491289825	0.0211455419141621	0,98544991779812	-0.02114554191416	2252
Q3UTZ3	sp|Q3UTZ3|CG043_MOUSE Uncharacterized protein C7orf43 homolog OS=Mus musculus PE=2 SV=1	1,01	0,945448610019628	0,987138173908166	25,7790605133414	26,4297525807184	26,3643567050085	26,3290904224167	26,1622102512306	26,1262269484152	236936165919,214	240465518186,125	−0,02133160375281	0,0136800209830813	0,0041304351996070	1,0148957937815	0.021331603752816	0,985322834252771	-0.02133160375281	2253
Q9DB15	sp|Q9DB15|RM12_MOUSE 39S ribosomal protein L12, mitochondrial OS=Mus musculus GN=Mrpl12 PE=1 SV=2	1,02	0,945550332897579	0,987138173908166	26,9656720986252	26,647166109089	26,4862939995624	26,3924755423625	26,7655997645261	26,9868342577459	394037033609,083	400095799799,68	−0,02201425548173	0,00913756621838279	0,0112445041828928	1,01537613390067	0.022014255481736	0,984856711333559	-0.02201425548173	2254
O70228	sp|O70228|ATP9A_MOUSE Probable phospholipid-transporting ATPase IIA OS=Mus musculus GN=Atp9a PE=2 SV=3	1,02	0,972498901813299	0,993288144763853	24,4117497112943	24,0742215615509	22,9492007514796	23,897846537075	23,80523416359	23,7779715892546	21943219192,0943	22281385368,1904	−0,02206374388280	0,0321603936261604	0,0026371813643091	1,01541096468735	0.0220637438828066	0,98482292862369	-0.02206374388280	2255

Q6ZQ18-2	sp|Q6ZQ18-2|EFR3B_MOUSE Isoform 2 of Protein EFR3 homolog B OS=Mus musculus GN=Efr3b;sp|Q6ZQ18|EFR3B_MOUSE Protein EFR3 homolog B OS=Mus musculus GN=Efr3b PE=2 SV=2	1,02	0,943697030644096	0,986987866859505	28,527812319833	28,2953913503404	27,7796180261822	28,2756209716639	28,2234557658422	28,1512065915307	1768124622416,31	1796319759332,69	−0,022824220700484	0,0135788104815375	0,00221417270179712	1,01594635160832	0,0228242207004819	0,98430394323177	-0,022824220700482	2256
Q9CYH2	sp|Q9CYH2|F213A_MOUSE Redox-regulatory protein FAM213A OS=Mus musculus GN=Fam213a PE=1 SV=2	1,02	0,787886361518381	0,942041082015698	28,9794072842181	28,9933056026423	28,991263159107	28,9989754780626	28,9032203325384	29,1101735891658	3884409196933,35	3947577175087,61	−0,023272283846133	0,000258879793014229	0,0035709617399277	1,01626192683411	0,0232722838461322	0,983998291774281	-0,023272283846132	2257
Q9R0P9	sp|Q9R0P9|UCHL1_MOUSE Ubiquitin carboxyl-terminal hydrolase isozyme L1 OS=Mus musculus GN=Uchl1 PE=1 SV=1	1,02	0,914663829562981	0,980665300581806	33,7181235415771	34,0837055790221	34,0600793369985	34,1391253660482	33,9705548759834	33,8014606732477	557213444301438	566433218513127	−0,023675807516073	0,00602550790506377	0,00496998893074136	1,01654621636642	0,0236758075160758	0,983723104665558	-0,023675807516075	2258
Q9CYR6	sp|Q9CYR6|AGM1_MOUSE Phosphoacetylglucosamine mutase OS=Mus musculus GN=Pgm3 PE=1 SV=1	1,02	0,924679437166897	0,983296707494544	28,6062432168209	29,0665390787366	29,1702498422972	28,9559879547382	28,9450977968638	28,991263159107	3730925265525,31	3792764556583,66	−0,023716354543161	0,0103705444396993	0,00083317551005910	1,01657478685777	0,023716354543159	0,98369545745965	-0,023716354543159	2259
Q60738	sp|Q60738|ZNT1_MOUSE Zinc transporter 1 OS=Mus musculus GN=Slc30a1 PE=1 SV=1	1,02	0,971723836276467	0,993288144763853	23,9989811215525	23,493812176176	24,5268886798457	24,5253945429987	23,3241799096996	24,2198565302694	26663479556,8511	27109326610,3925	−0,023924214456756	0,0215182826473761	0,0259874605998192	1,0167212629766	0,023924214456754	0,983553739273978	-0,023924214456754	2260
Q9D1F4	sp|Q9D1F4|AKTS1_MOUSE Proline-rich AKT1 substrate 1 OS=Mus musculus GN=Akt1s1 PE=1 SV=1	1,02	0,959486254470245	0,991148864142112	25,3323809620451	24,3639618883844	24,8152314567564	25,1895497765879	24,595883751326	24,7764875068518	61186440149,3774	62221950617,5788	−0,024211658068120	0,0195103729567429	0,0122444687916582	1,01692385544368	0,0242116580681206	0,983357794830867	-0,024211658068120	2261
P60824	sp|P60824|CIRBP_MOUSE Cold-inducible RNA-binding protein OS=Mus musculus GN=Cirbp PE=1 SV=1	1,02	0,965852715597627	0,991306002794532	26,5351193087617	26,3614633838226	26,3290904224167	25,8617916340518	27,1705289713067	26,2439255198213	294503788755,764	299510518791,847	−0,024320476995964	0,00419539466613133	0,0254671549662795	1,01700056239424	0,0243204769959633	0,983283625375566	-0,024320476995963	2262
Q99JW2	sp|Q99JW2|ACY1_MOUSE Aminoacylase-1 OS=Mus musculus GN=Acy1 PE=1 SV=1	1,02	0,852636489054407	0,962750418337502	27,1958332984449	27,0687747291807	26,9435946391298	26,9970599843299	27,0764572082296	27,1854010346971	570284324841,023	580007038496,589	−0,024389029210305	0,00465915355713031	0,00349091544918314	1,01704888812835	0,0243890292103057	0,983236904019703	-0,024389029210305	2263
Q8R570	sp|Q8R570|SNP47_MOUSE Synaptosomal-associated protein 47 OS=Mus musculus GN=Snap47 PE=1 SV=1;sp|Q8R570-2|SNP47_MOUSE Isoform 2 of Synaptosomal-associated protein 47 OS=Mus musculus GN=Snap47	1,02	0,872033007790464	0,971327072972003	29,714294747174	29,7312890475709	29,8734429990539	29,6094844022266	29,9308608554239	29,8306347317633	8516349744879,74	8665118053126,69	−0,02498420589072	0,0029352936076582	0,00551978481284083	1,01746855315993	0,0249842058907203	0,982831358172521	-0,024984205890720	2264
P24527	sp|P24527|LKHA4_MOUSE Leukotriene A-4 hydrolase OS=Mus musculus GN=Lta4h PE=1 SV=4	1,02	0,930531315943682	0,98433518830764	30,3912180432188	30,9481182033933	30,9678402142619	30,9481182033933	30,6814880843035	30,7314349400907	23058574694424,3	23476328759529,8	−0,025903477368249	0,0106395279761463	0,0046038238827544	1,01811708098361	0,0259034773682456	0,982205306912141	-0,025903477368245	2265
P46664	sp|P46664|PURA2_MOUSE Adenylosuccinate synthetase isozyme 2 OS=Mus musculus GN=Adss PE=1 SV=2	1,02	0,881104090217308	0,973716798448825	30,1584841687995	29,7759040277786	30,0952385083161	30,0816756695027	30,0134696913558	29,9890457048955	10792532419877,4	10990624296335,3	−0,026239910940537	0,00683368610963286	0,00159878776499544	1,01835453151784	0,0262399109405413	0,981976285321303	-0,026239910940541	2266
P10852-2	sp|P10852-2|4F2_MOUSE Isoform 2 of 4F2 cell-surface antigen heavy chain OS=Mus musculus GN=Slc3a2;sp|P10852|4F2_MOUSE 4F2 cell-surface antigen heavy chain OS=Mus musculus GN=Slc3a2 PE=1 SV=1	1,02	0,890731000757418	0,976830327013275	30,8796473041202	30,6939719257556	30,4270212494486	30,7314349400907	30,688685623596	30,6358451815377	20818854528840,6	21206352841991,4	−0,026605828916638	0,00741927289557022	0,00156047152723471	1,01861285464164	0,026605828916639	0,98172725333592	-0,026605828916639	2267
Q62086	sp|Q62086|PON2_MOUSE Serum paraoxonase/arylesterase 2 OS=Mus musculus GN=Pon2 PE=1 SV=2	1,02	0,938626836937885	0,985646114383563	26,5920726184234	26,2939048107703	26,1582354818588	26,3554364395044	26,7237904447108	26,0204577171094	277218258681,082	282391864514,717	−0,026676244155148	0,00842311184160694	0,0133425937434412	1,01866257243751	0,0266762441551466	0,981679338239699	-0,026676244155146	2268
Q920P3	sp|Q920P3|BRNP1_MOUSE BMP/retinoic acid-inducible neural-specific protein 1 OS=Mus musculus GN=Brinp1 PE=2 SV=1	1,02	0,932235684720773	0,98467134375593	27,3724149190948	26,8725182103567	26,7900481326491	26,868788726629	27,2638746068392	26,9578668145221	538288457995,748	548348415185,321	−0,026713367399957	0,0116665175831452	0,00766665953930455	1,01868878487016	0,0267133673999582	0,981654078117153	-0,026713367399958	2269
Q8K0V2	sp|Q8K0V2|DCNL3_MOUSE DCN1-like protein 3 OS=Mus musculus GN=Dcun1d3 PE=2 SV=1	1,02	0,961590534772083	0,991306002794532	21,8565573461608	22,6368476224383	23,0008472762563	22,1414207633023	22,6703028947368	22,7383944477728	5899208826,23795	6010092853,87774	−0,026865800185693	0,0259857346257783	0,01451299896742	1,01879642353846	0,026865800185695	0,98155036364068	-0,026865800185695	2270
Q61410	sp|Q61410|KGP2_MOUSE cGMP-dependent protein kinase 2 OS=Mus musculus GN=Prkg2 PE=2 SV=1	1,02	0,913500519012557	0,980665300581806	22,9695985789723	22,4633145269286	22,6646252533469	22,61434833139	22,8966186104492	22,6427415317092	7213206746,40261	7349534571,17248	−0,027012115115835	0,0112296855984177	0,0068413948369033	1,01889975285096	0,0270121151158342	0,981450822028296	-0,027012115115834	2271
P70202	sp|P70202|LXN_MOUSE Latexin OS=Mus musculus GN=Lxn PE=1 SV=2	1,02	0,918897968440822	0,981613998294397	28,2627967575825	28,527812319833	28,8395124734223	28,7178629849572	28,4508773233213	28,5178789364239	2490174365551,34	2537515109853,5	−0,027169647586686	0,0101134718847308	0,00486317300988583	1,01901101583771	0,0271696475866866	0,981343660134937	-0,027169647586686	2272
Q9EST4-3	sp|Q9EST4-3|PSMG2_MOUSE Isoform 3 of Proteasome assembly chaperone 2 OS=Mus musculus GN=Psmg2;sp|Q9EST4|PSMG2_MOUSE Proteasome assembly chaperone 2 OS=Mus musculus GN=Psmg2 PE=2 SV=1	1,02	0,891324347976756	0,977046961561497	25,6268016812449	25,5956685115229	25,9516347704544	25,8861812107947	25,6128898625493	25,7318503388321	148626406363,296	151468038209,62	−0,027322936382196	0,00766366021412788	0,00532273685533137	1,01911929323904	0,0273229363821893	0,981239396245488	-0,027322936382189	2273
Q80W04	sp|Q80W04|TMCC2_MOUSE Transmembrane and coiled-coil domains protein 2 OS=Mus musculus GN=Tmcc2 PE=1 SV=1	1,02	0,927237684977327	0,983810446072053	24,615571684966	24,0288934571652	24,0874845308129	24,4132109090808	24,0841927204206	24,2920359350875	33808673681,6399	34462807001,3587	−0,027646793859154	0,0133284927895876	0,00685816628513879	1,01934809173168	0,0276467938591547	0,981019151466884	-0,027646793859154	2274
Q9DCG9	sp|Q9DCG9|TR112_MOUSE Multifunctional methyltransferase subunit TRM112-like protein OS=Mus musculus GN=Trmt112 PE=2 SV=1	1,02	0,951585888361129	0,988708994871138	25,9546021761348	25,7400196200249	25,8025240721104	25,789671987075	25,347826740016	26,417345126627	165523986714,128	168738264049,295	−0,027746865825014	0,00427228162219372	0,0207895361981411	1,01941880085766	0,0277468658250115	0,980951105824889	-0,027746865825011	2275

Q8BY89-2	sp|Q8BY89-2|CTL2_MOUSE Isoform 2 of Choline transporter-like protein 2 OS=Mus musculus GN=Slc44a2;sp|Q8BY89|CTL2_MOUSE Choline transporter-like protein 2 OS=Mus musculus GN=Slc44a2 PE=1 SV=2	1,02	0,951715898081411	0,988708994871138	24,5401576917216	25,4554077650705	25,3025335055744	25,3909018070988	24,8802711146083	25,0849820401459	79527309933,1758	81081310432,6409	−0,02791903418434	0,0195337183040161	0,0102306801248179	1,01954046353097	0.0279190341843504	0,980834048054069	-0.02791903418435	2276
P17563	sp|P17563|SBP1_MOUSE Selenium-binding protein 1 OS=Mus musculus GN=Selenbp1 PE=1 SV=2;sp|Q63836|SBP2_MOUSE Selenium-binding protein 2 OS=Mus musculus GN=Selenbp2 PE=1 SV=2	1,02	0,930300601018268	0,98433518830764	28,5598383543679	29,1733008248421	29,119788155716	29,1799288907371	28,8313933089507	28,9001884639333	3743251663292,77	3817067427323,11	−0,02817262592900	0,0117367384429549	0,0063720228083643	1,01971969043765	0.0281726259290075	0,980661655725032	-0.02817262592900	2277
P17183	sp|P17183|ENOG_MOUSE Gamma-enolase OS=Mus musculus GN=Eno2 PE=1 SV=2	1,02	0,761308896315674	0,933389703428013	35,2909566904371	35,0731894837483	35,1702667677213	35,211446325403	35,211446325403	35,1702667677213	1895269893477120	1932749134218290	−0,02825108349673	0,00310126581857822	0,0006754707078635	1,0197751469963	0.0282510834967379	0,98060832620353	-0.02825108349673	2278
Q99LM2	sp|Q99LM2|CK5P3_MOUSE CDK5 regulatory subunit-associated protein 3 OS=Mus musculus GN=Cdk5rap3 PE=1 SV=1	1,02	0,941706525844166	0,986704967868576	24,4989816470079	24,9798946960453	24,9493555858095	25,1428879762118	24,4081168167823	24,9361022925742	59510026205,297	60689448839,9111	−0,02831296553692	0,0108536511322142	0,0152605927239158	1,01981888951864	0.0283129655369245	0,980566265517994	-0.02831296553692	2279
Q91YP2	sp|Q91YP2|NEUL_MOUSE Neurolysin, mitochondrial OS=Mus musculus GN=Nln PE=2 SV=1	1,02	0,876059552076801	0,973077864414309	29,1365569873341	28,9160873320475	28,7045051167192	28,9766402388757	28,9271249389734	28,9124490208349	3625632283405,39	3697721984954,1	−0,02840414668994	0,00747054868523564	0,0011622641039339	1,01988334610729	0.028404146689942	0,980504293767341	-0.028404146689942	2280
P80313	sp|P80313|TCPH_MOUSE T-complex protein 1 subunit eta OS=Mus musculus GN=Cct7 PE=1 SV=1	1,02	0,912886129676335	0,980665300581806	31,486087748298	31,9827023770707	32,033953090713	31,9339369527224	31,7608767264179	31,8676070981556	66901850187308,4	68246021762366,4	−0,02869884053871	0,00950556050346968	0,0027410103010092	1,02009169509206	0.0286988405387161	0,98030403032519	-0.028698840538716	2281
P53702	sp|P53702|CCHL_MOUSE Cytochrome c-type heme lyase OS=Mus musculus GN=Hccs PE=1 SV=2	1,02	0,935476443051778	0,98517137107117	24,6402971622954	25,2894343129533	25,2820160355893	25,2798116120726	24,8701728545749	25,1215085600284	77270841066,9512	78825128735,6049	−0,02873151980488	0,0148642934734273	0,0082331004632446	1,02011480200283	0.0287315198048841	0,980281825179543	-0.02873151980488	2282
Q60714	sp|Q60714|S27A1_MOUSE Long-chain fatty acid transport protein 1 OS=Mus musculus GN=Slc27a1 PE=1 SV=1	1,02	0,931987540258074	0,98467134375593	25,4094933692325	26,1262269484152	26,0128955673314	26,0168115282551	25,8041604093304	25,7876482760269	168388328528,363	171790246281,646	−0,02885598245035	0,0149047245334951	0,0049404729367724	1,02020281205363	0.0288559824503477	0,980197259000924	-0.02885598245034	2283
Q7M759	sp|Q7M759|AB17B_MOUSE Alpha/beta hydrolase domain-containing protein 17B OS=Mus musculus GN=Abhd17b PE=2 SV=1	1,02	0,916197166972638	0,980665300581806	24,8701728545749	24,8203758830295	25,3783577930722	25,1789507460175	25,00364897593	24,946752196482	73677908598,2298	75177461294,1359	−0,02906808705431	0,012339916109429	0,0048327150917143	1,02035281299966	0.0290680870543176	0,980053161278764	-0.02906808705431	2284
Q6P1I6-2	sp|Q6P1I6-2|PSD2_MOUSE Isoform 2 of PH and SEC7 domain-containing protein 2 OS=Mus musculus GN=Psd2;sp|Q6P1I6|PSD2_MOUSE PH and SEC7 domain-containing protein 2 OS=Mus musculus GN=Psd2 PE=2 SV=1;sp|Q6P1I6-3|PSD2_MOUSE Isoform 3 of PH and SEC7 domain-con	1,02	0,938741606627042	0,985646114383563	23,7728608943411	23,8903181590625	23,3379615440826	24,0031450361206	23,7625704964307	23,2969409758378	18987503056,2367	19380866810,3109	−0,02958289986510	0,0122949174667992	0,0151568656494453	1,02071698173842	0.0295828998651055	0,979703500471665	-0.02958289986510	2285
Q922R8	sp|Q922R8|PDIA6_MOUSE Protein disulfide-isomerase A6 OS=Mus musculus GN=Pdia6 PE=1 SV=3	1,02	0,844945289654331	0,960697384679244	30,4385207524286	30,1742185244125	30,1559838428577	30,3400376693966	30,3160330662053	30,1742185244125	13807610476704,9	14093898464907,8	−0,02960705510668	0,00522609315804482	0,0029598141214238	1,02073407188636	0.029607055106688	0,979687097298471	-0.029607055106688	2286
P07742	sp|P07742|RIR1_MOUSE Ribonucleoside-diphosphate reductase large subunit OS=Mus musculus GN=Rrm1 PE=1 SV=2	1,02	0,97077310206066	0,993055959121831	21,9854071197575	21,6767034556501	22,6774724465421	23,0900203571021	21,569596405202	21,7420055571971	4014563016,34552	4098447606,9268	−0,02983459563930	0,0231751306919412	0,0376130291855712	1,02089507381992	0.0298345956392993	0,979532594136495	-0.02983459563929	2287
Q9QYI5	sp|Q9QYI5|DNJB2_MOUSE DnaJ homolog subfamily B member 2 OS=Mus musculus GN=Dnajb2 PE=1 SV=2;sp|Q9QYI5-2|DNJB2_MOUSE Isoform 2 of DnaJ homolog subfamily B member 2 OS=Mus musculus GN=Dnajb2	1,02	0,916804303762138	0,980665300581806	25,4222737780244	25,1009475178655	25,4287308522879	25,0214896836834	25,4079277565894	25,5847097988631	98894294341,8068	100965267958,246	−0,02989989846407	0,0074024276779101	0,0113678974246853	1,02094128513907	0.0298998984640699	0,979488257117332	-0.0298998984640	2288
Q8C1Y8	sp|Q8C1Y8|CCZ1_MOUSE Vacuolar fusion protein CCZ1 homolog OS=Mus musculus GN=Ccz1 PE=1 SV=1	1,02	0,850019941481674	0,962394740430361	26,9378482697719	27,024059925362	27,2799384547728	27,0796340180657	27,1769933127962	27,0475341194994	576715866619,993	588820458658,556	−0,02996708452999	0,00656999251741954	0,0024875213185174	1,02098883131048	0.0299670845299971	0,979442643575701	-0.02996708452999	2289
O35344	sp|O35344|IMA4_MOUSE Importin subunit alpha-4 OS=Mus musculus GN=Kpna3 PE=1 SV=1	1,02	0,838800274028711	0,959746519741951	29,0269449893171	29,3062942508444	29,1279023668535	29,209851847461	29,051315220493	29,2631523510523	4584553659908,93	4682124798836	−0,03038210535160	0,00485193872344093	0,0037768609538435	1,02128258194038	0.0303821053516052	0,979160927331256	-0.03038210535160	2290
Q9WVK4	sp|Q9WVK4|EHD1_MOUSE EH domain-containing protein 1 OS=Mus musculus GN=Ehd1 PE=1 SV=1	1,02	0,911733142246136	0,980665300581806	28,3097572274689	28,8525957718364	28,8443921491069	28,7679720830482	28,5614572625613	28,7408088025449	2823264765411,72	2883654077353,67	−0,03053367861977	0,0108503179229622	0,0039112810800811	1,02138988616363	0.0305336786197772	0,979058059558458	-0.03053367861977	2291
P59481	sp|P59481|LMA2L_MOUSE VIP36-like protein OS=Mus musculus GN=Lman2l PE=2 SV=1	1,02	0,908636730965966	0,980429909122681	25,8175097401103	25,9318139841019	26,3515957296419	26,056977654842	25,8966554254401	26,2113399560879	202425917682,208	206794423205,021	−0,03080326194900	0,0108024267020059	0,0060392141917107	1,02158076185517	0.0308032619490037	0,978875128956055	-0.03080326194900	2292

Q60902-2	sp|Q60902-2|EP15R_MOUSE Isoform 2 of Epidermal growth factor receptor substrate 15-like 1 OS=Mus musculus GN=Eps15l1;sp|Q60902|EP15R_MOUSE Epidermal growth factor receptor substrate 15-like 1 OS=Mus musculus GN=Eps15l1 PE=1 SV=3;sp|Q60902-4|EP15R_MOUSE	1,02	0,879286495793301	0,973716798448825	28,6488796693929	28,3588682131998	28,1677723504312	28,381044130583	28,4381222529815	28,4205387528905	2140028104188,99	2186307241596,72	−0,030866413959937	0,00853211638177999	0,0010288020534603	1,02162548114071	0.0308664139599379	0,978832280968007	-0.030866413959937	2293
P62075	sp|P62075|TIM13_MOUSE Mitochondrial import inner membrane translocase subunit Tim13 OS=Mus musculus GN=Timm13 PE=1 SV=1	1,02	0,919905437949385	0,98176599643834	28,1337438378691	27,9977571851328	27,8286564820045	27,6683860242425	28,0120626766497	28,344311301084	1427176550673,43	1458242900398,72	−0,031067234002443	0,00546126535742384	0,0120671123390114	1,02176769910536	0.0310672340024483	0,978696039105148	-0.031067234002448	2294
Q9CPZ8	sp|Q9CPZ8|COXM1_MOUSE COX assembly mitochondrial protein homolog OS=Mus musculus GN=Cmc1 PE=3 SV=1	1,02	0,959163715216235	0,991041225441951	23,3276794305009	23,9668942617015	22,8859967988134	23,0785352794043	23,9723375129221	23,19435933754	14443690275,4814	14758387122,759	−0,031095675235197	0,0232307300294124	0,0207585787673907	1,02178784239176	0.0310956752351953	0,978676745320476	-0.031095675235195	2295
Q8BHE8	sp|Q8BHE8|CB047_MOUSE Uncharacterized protein C2orf47 homolog, mitochondrial OS=Mus musculus PE=2 SV=1	1,02	0,937153540579675	0,985574587741229	25,5777956432148	26,1622102512306	26,1671183698062	26,3498310529515	25,9245627621526	25,6974368156947	189762935355,901	193900351167,034	−0,031117184710493	0,0130477542288269	0,0127420656609199	1,02180307657748	0.031117184710494	0,978662154110444	-0.031117184710494	2296
Q9DBE8	sp|Q9DBE8|ALG2_MOUSE Alpha-1,3/1,6-mannosyltransferase ALG2 OS=Mus musculus GN=Alg2 PE=2 SV=2	1,02	0,858984232596092	0,966502999979251	29,5228299333287	29,1030743452272	29,3158045711717	29,3179435122904	29,3897530370284	29,3002269215392	5381040914445,68	5501129167549,8	−0,031842501846450	0,00715988776740497	0,0016160542869509	1,022316918792	0.0318425018464508	0,978170253879422	-0.031842501846451	2297
O88485	sp|O88485|DC1I1_MOUSE Cytoplasmic dynein 1 intermediate chain 1 OS=Mus musculus GN=Dync1i1 PE=1 SV=2;sp|O88485-2|DC1I1_MOUSE Isoform 1B of Cytoplasmic dynein 1 intermediate chain 1 OS=Mus musculus GN=Dync1i1;sp|O14576-2|DC1I1_HUMAN Isoform 2 of Cytoplas	1,02	0,824890758857653	0,95563531252039	30,0920533297203	29,8955901225831	29,748150804332	29,9327417629286	29,9440608870272	29,9252115172164	9785584202505,77	10003985928851,9	−0,031845045513191	0,00576802103792034	0,0003169618979476	1,02231872127677	0.0318450455131974	0,978168529234307	-0.031845045513197	2298
P38585	sp|P38585|TTL_MOUSE Tubulin--tyrosine ligase OS=Mus musculus GN=Ttl PE=2 SV=2	1,02	0,909781994915799	0,980429909122681	26,3465902358295	26,1207629911894	26,2580903346101	26,5801731397537	26,2771103257199	25,9353037091857	249273000543,768	254914931601,942	−0,032289252515381	0,00433620905867215	0,0122839716049273	1,02263354252513	0.0322892525153818	0,977867396693011	-0.032289252515381	2299
P84086	sp|P84086|CPLX2_MOUSE Complexin-2 OS=Mus musculus GN=Cplx2 PE=1 SV=1	1,02	0,933865261815924	0,98467134375593	29,5382706477389	29,5469002992119	29,3052903367405	28,9967840041252	29,5874435150517	29,8734429990539	6249278259719,16	6390861356088,81	−0,032320809790695	0,00465221039458525	0,0151620146181239	1,02265591168859	0.032320809790695	0,977846007215481	-0.032320809790695	2300
O88935	sp|O88935|SYN1_MOUSE Synapsin-1 OS=Mus musculus GN=Syn1 PE=1 SV=2;sp|O88935-3|SYN1_MOUSE Isoform 3 of Synapsin-1 OS=Mus musculus GN=Syn1	1,02	0,7164981171382	0,919215645474926	35,505272310377	35,5823748158424	35,4772270626791	35,505272310377	35,4772270626791	35,6500093773241	2672056323809150	2732981638683890	−0,032525348814138	0,00153280734281942	0,0026087646774536	1,0228009096709	0.0325253488141375	0,977707382291789	-0.032525348814137	2301
Q8R3S6	sp|Q8R3S6|EXOC1_MOUSE Exocyst complex component 1 OS=Mus musculus GN=Exoc1 PE=1 SV=4	1,02	0,945817122345586	0,987138173908166	28,3200413611814	27,4559070409885	27,9775310134962	27,8762541482515	28,344311301084	27,6006069372341	1332164890282,69	1362565990251,72	−0,032553437808503	0,0155868144100644	0,0134564198308414	1,02282082360133	0.0325534378085083	0,977688346702816	-0.032553437808508	2302
Q9D8V7	sp|Q9D8V7|SC11C_MOUSE Signal peptidase complex catalytic subunit SEC11C OS=Mus musculus GN=Sec11c PE=2 SV=3	1,02	0,82998876868025	0,956900945145962	24,0912088731561	24,1252534040277	23,9964472236921	23,8956506099416	24,1795012645539	24,2064354818055	28437366343,1455	29095880923,9685	−0,033027067146924	0,00277286613598509	0,0071463823494254	1,0231566655251	0.0330270671469251	0,977367429343563	-0.033027067146925	2303
Q56A07	sp|Q56A07|SCN2B_MOUSE Sodium channel subunit beta-2 OS=Mus musculus GN=Scn2b PE=1 SV=1	1,02	0,91610068788903	0,980665300581806	28,1272472497798	27,576491819139	27,9723912373162	27,7598510714024	28,2008611250849	27,7842060823852	1298256641527,56	1328368694198,78	−0,033080022332270	0,0101832963316037	0,0088800330443113	1,02319422193426	0.0330800223322628	0,977331555009746	-0.033080022332262	2304
Q8BZQ7	sp|Q8BZQ7|ANC2_MOUSE Anaphase-promoting complex subunit 2 OS=Mus musculus GN=Anapc2 PE=1 SV=2	1,02	0,900543647303989	0,977644369603516	26,072361667895	25,8825371944796	25,6255477551962	26,0372835360317	25,9874283403252	25,6245416244004	170184481775,826	174132876061,878	−0,033089116384075	0,00867151736028585	0,0087040644599309	1,02320067167612	0.0330891163840769	0,977325394403703	-0.033089116384076	2305
P23927	sp|P23927|CRYAB_MOUSE Alpha-crystallin B chain OS=Mus musculus GN=Cryab PE=1 SV=2	1,02	0,909208364867585	0,980429909122681	27,8588173640719	27,572598641113	27,2974019823772	27,3929021595667	27,5950102812602	27,8099832106879	946723263014,232	968775316568,902	−0,033219334406896	0,0101799734280845	0,0075572062212577	1,02329303019814	0.0332193344068963	0,977237184744992	-0.033219334406896	2306
Q09200	sp|Q09200|B4GN1_MOUSE Beta-1,4 N-acetylgalactosaminyltransferase 1 OS=Mus musculus GN=B4galnt1 PE=2 SV=1	1,02	0,915849286065482	0,980665300581806	22,2626581889544	21,7674627603317	22,343926520924	21,9456502500876	22,1271865824891	22,3703619013105	4060948849,66964	4155642603,99097	−0,033254728392705	0,014102736542647	0,0096217523379267	1,0233181352011	0.0332547283927039	0,977213210243251	-0.033254728392703	2307
Q8R5F3	sp|Q8R5F3|OARD1_MOUSE O-acetyl-ADP-ribose deacetylase 1 OS=Mus musculus GN=Oard1 PE=2 SV=2	1,02	0,972726278284375	0,993296867746407	23,985379748144	22,1623409962791	23,7308311655272	22,6114053377269	23,8119599107002	23,5244400838048	13060400632,8166	13365399943,0061	−0,033303856296726	0,0423858852379935	0,0268849658142169	1,02335298271196	0.0333038562967263	0,977179933897219	-0.033303856296726	2308
Q6P4S6	sp|Q6P4S6|SIK3_MOUSE Serine/threonine-protein kinase SIK3 OS=Mus musculus GN=Sik3 PE=1 SV=3;sp|Q6P4S6-2|SIK3_MOUSE Isoform 2 of Serine/threonine-protein kinase SIK3 OS=Mus musculus GN=Sik3	1,02	0,905228114220664	0,979225550630981	27,3601226677854	27,8016555984756	27,5035939037474	27,2983095342163	27,6568502118367	27,7796180261822	926911618617,22	948605887999,192	−0,033377039380793	0,00817386154473732	0,0090685100714111	1,02340489529556	0.0333770393807934	0,977130366091518	-0.033377039380793	2309
P42932	sp|P42932|TCPQ_MOUSE T-complex protein 1 subunit theta OS=Mus musculus GN=Cct8 PE=1 SV=3	1,02	0,895720235301538	0,977357990376388	32,1814288440177	32,6953467176655	32,7150311669666	32,5913113535174	32,504668068945	32,5652501037116	134233541095206	137376050474787	−0,033385308570939	0,00930057059542374	0,0013655274057937	1,02341076122958	0.0333853085709358	0,977124765425122	-0.033385308570935	2310

Q03141-3	sp|Q03141-3|MARK3_MOUSE Isoform 3 of MAP/microtubule affinity-regulating kinase 3 OS=Mus musculus GN=Mark3;sp|Q03141|MARK3_MOUSE MAP/microtubule affinity-regulating kinase 3 OS=Mus musculus GN=Mark3 PE=1 SV=2;sp|Q03141-2|MARK3_MOUSE Isoform 2 of MAP/mic	1,02	1,02	0,859831846519733	0,966512320790755	26,9578668145221	26,6335512707477	26,6258838312392	26,7811512810363	26,8802456280836	26,6258838312392	409867854598,749	419540924472,494	−0,03365270071186	0,00708684455954066	0,004790690346758	1,02360045991705	0.033652700711859	0,976943679842658	-0,033652700711859	2311
P62880	sp|P62880|GBB2_MOUSE Guanine nucleotide-binding protein G(I)/G(S)/G(T) subunit beta-2 OS=Mus musculus GN=Gnb2 PE=1 SV=3	1,02	1,02	0,907229216617284	0,980398950801751	30,9950183754577	31,3519919643443	31,625103247153	31,5209983110802	31,2665446190202	31,2551943052669	40165834507395,9	41122604680048,8	−0,03396279577793	0,0100871802935423	0,0047943940990185	1,02382049780334	0.0339627957779363	0,976733716648131	-0,033962795777936	2312
O35250	sp|O35250|EXOC7_MOUSE Exocyst complex component 7 OS=Mus musculus GN=Exoc7 PE=1 SV=2;sp|O35250-2|EXOC7_MOUSE Isoform 2 of Exocyst complex component 7 OS=Mus musculus GN=Exoc7	1,02	1,02	0,873718901098209	0,972488987759875	29,3057922937924	28,9855437086712	28,801510817816	29,089228896999	29,0211872608148	29,0533026951177	4054907289961,85	4151840936543,17	−0,03408224334817	0,00879022298665657	0,0011715401606357	1,02390526827118	0.034082243348176	0,976652851575276	-0,034082243348176	2313
P80318	sp|P80318|TCPG_MOUSE T-complex protein 1 subunit gamma OS=Mus musculus GN=Cct3 PE=1 SV=1	1,02	1,02	0,896611295864129	0,977357990376388	31,9339369527224	32,420921979523	32,5127722112315	32,3661802272999	32,26957919978	32,3027531536818	105444976303001	107966005209054	−0,03408676602055	0,00963431520804815	0,001519015770546	1,02390847809391	0.0340867660205473	0,976649789892925	-0,034086766020547	2314
P99027	sp|P99027|RLA2_MOUSE 60S acidic ribosomal protein P2 OS=Mus musculus GN=Rplp2 PE=1 SV=3	1,02	1,02	0,759927516889348	0,933136198610489	27,5648173464815	27,5067207470531	27,4336352949716	27,4606749995793	27,4549509257709	27,6606396741905	878713253872,267	899785165263,74	−0,03418812676847	0,0023901624673693	0,004255594217425	1,02398041829757	0.0341881267684708	0,976581174923798	-0,034188126768470	2315
Q9QUR6	sp|Q9QUR6|PPCE_MOUSE Prolyl endopeptidase OS=Mus musculus GN=Prep PE=2 SV=1	1,02	1,02	0,881385281738258	0,973716798448825	30,6034495341565	30,3309155382186	30,1444136462248	30,5115424211656	30,4270212494486	30,2119986671099	15311004071937,3	15681782108029,3	−0,03452062377589	0,00760411070695722	0,005082924761012	1,02421644160957	0.0345206237758996	0,97635612881637	-0,034520623775899	2316
P32921-2	sp|P32921-2|SYWC_MOUSE Isoform 2 of Tryptophan--tRNA ligase, cytoplasmic OS=Mus musculus GN=Wars;sp|P32921|SYWC_MOUSE Tryptophan--tRNA ligase, cytoplasmic OS=Mus musculus GN=Wars PE=1 SV=2	1,02	1,02	0,907373914910228	0,980398950801751	30,1415815593921	30,7055843535906	30,6767393272954	30,7034268677074	30,5115424211656	30,3808159827936	17759974424436,5	18190642399020,4	−0,03456698827440	0,0104113105456186	0,005314722282653	1,0242493578139	0.0345669882744109	0,976324751752193	-0,034566988274411	2317
O08600	sp|O08600|NUCG_MOUSE Endonuclease G, mitochondrial OS=Mus musculus GN=Endog PE=2 SV=1	1,02	1,02	0,885818364232314	0,975149870380105	25,807698406866	25,8966554254401	26,1491158481579	25,8344529325275	25,8503583907544	26,2409001964166	186399221027,973	190942309188,198	−0,03474098106939	0,0068248849254257	0,008862607608042	1,0243728924143	0.0347409810693992	0,976207011533797	-0,034740981069399	2318
Q8BFZ9	sp|Q8BFZ9|ERLN2_MOUSE Erlin-2 OS=Mus musculus GN=Erlin2 PE=1 SV=1	1,02	1,02	0,871140806361011	0,971009960968743	28,9668367465018	28,520313058957	28,5524438127231	28,7457012936542	28,7466735660147	28,62011627812	2854347936395,4	2924555772989,39	−0,03505629667673	0,00868358132196098	0,002535828512884	1,02459680394908	0.0350562966767341	0,975993674922388	-0,035056296676734	2319
Q9WVH4	sp|Q9WVH4|FOXO3_MOUSE Forkhead box protein O3 OS=Mus musculus GN=Foxo3 PE=1 SV=1	1,02	1,02	0,916695215641811	0,980665300581806	21,8690502708892	21,1029986109079	21,2714023663618	21,4244040100507	21,4368240413319	21,4552101793533	1996145183,44284	2045304967,28346	−0,03509931927113	0,0187989817093534	0,000722944490729	1,02462735889573	0.0350993192711347	0,975964570258726	-0,035099319271134	2320
Q6NXK7	sp|Q6NXK7|DPP10_MOUSE Inactive dipeptidyl peptidase 10 OS=Mus musculus GN=Dpp10 PE=2 SV=1	1,02	1,02	0,901993487944297	0,977644369603516	30,0268702489686	29,6053749310012	29,472404298724	29,6926508385983	29,9229385752534	29,5620727820686	7929014161305,6	8124354530918,13	−0,03511169502146	0,00974609852293504	0,006146640554077	1,02463614840869	0.0351116950214677	0,975956198259303	-0,035111695021467	2321
Q9ER73	sp|Q9ER73|ELP4_MOUSE Elongator complex protein 4 OS=Mus musculus GN=Elp4 PE=2 SV=2	1,03	1,03	0,89905777048267	0,977644369603516	26,2266208709123	25,8813493021154	26,1262269484152	25,8188439996991	26,3947233703869	26,0949756141312	211621620322,394	216931540990,368	−0,03575280251169	0,00681042716960902	0,0110340597180724	1,02509157930028	0.0357528025116975	0,975522597388409	-0,035752802511697	2322
Q9EQG9-2	sp|Q9EQG9-2|C43BP_MOUSE Isoform 2 of Collagen type IV alpha-3-binding protein OS=Mus musculus GN=Col4a3bp;sp|Q9EQG9|C43BP_MOUSE Collagen type IV alpha-3-binding protein OS=Mus musculus GN=Col4a3bp PE=1 SV=1;sp|Q9EQG9-3|C43BP_MOUSE Isoform 3 of Collagen	1,03	1,03	0,888959085023192	0,976830327013275	25,848124338265	25,8041604093304	25,3737724117129	25,8351165872011	25,5185024275634	25,7471346094699	141469840000,188	145036490753,987	−0,03592140650695	0,0102082083595484	0,006359118311227	1,02521138607207	0.0359214065069503	0,975408597276055	-0,035921406506950	2323
Q3ULJ0	sp|Q3ULJ0|GPD1L_MOUSE Glycerol-3-phosphate dehydrogenase 1-like protein OS=Mus musculus GN=Gpd1l PE=1 SV=2;sp|Q3ULJ0-2|GPD1L_MOUSE Isoform 2 of Glycerol-3-phosphate dehydrogenase 1-like protein OS=Mus musculus GN=Gpd1l	1,03	1,03	0,842769253380473	0,959771286845933	31,7920975405357	31,3988833526896	31,4825297714827	31,5283219649286	31,6602286177643	31,559954806254	50745136068165,5	52029663804985,6	−0,03606483891739	0,00656387792826702	0,002180476085359	1,02531331742011	0.0360648389173913	0,975311627197233	-0,036064838917391	2324
Q9QYB8	sp|Q9QYB8|ADDB_MOUSE Beta-adducin OS=Mus musculus GN=Add2 PE=1 SV=4;sp|Q05764|ADDB_RAT Beta-adducin OS=Rattus norvegicus GN=Add2 PE=2 SV=4;sp|Q9QYB8-3|ADDB_MOUSE Isoform 3 of Beta-adducin OS=Mus musculus GN=Add2;sp|Q9QYB8-2|ADDB_MOUSE Isoform 2 of Beta	1,03	1,03	0,920228138586261	0,981879530099445	32,1137938104902	31,4594116268809	31,6858278589972	31,8110328188897	32,0369458698543	31,486087748298	61681866070632,8	63244048590650,2	−0,03608331331743	0,0104659031966632	0,008713844142395	1,02532644713162	0.0360833133174388	0,975299137945316	-0,036083313317438	2325

Q60715-2	sp|Q60715-2|P4HA1_MOUSE Isoform 2 of Prolyl 4-hydroxylase subunit alpha-1 OS=Mus musculus GN=P4ha1;sp|Q60715|P4HA1_MOUSE Prolyl 4-hydroxylase subunit alpha-1 OS=Mus musculus GN=P4ha1 PE=2 SV=2	1,03	0,813181394596766	0,950561511736987	24,7822925530271	25,1330874985912	24,9472029184827	24,9961132911365	25,0225487947601	24,9190543270454	68781064820,0673	70525402732,719	−0,036131548463878	0,00703296385124 46	0,0021526339733204	1,0253607285263	0.0361315484638786	0,975266530284662	-0.036131548463878	2326
Q3UEB3-2	sp|Q3UEB3-2|PUF60_MOUSE Isoform 2 of Poly(U)-binding-splicing factor PUF60 OS=Mus musculus GN=Puf60;sp|Q3UEB3|PUF60_MOUSE Poly(U)-binding-splicing factor PUF60 OS=Mus musculus GN=Puf60 PE=2 SV=2;sp|Q3UEB3-3|PUF60_MOUSE Isoform 3 of Poly(U)-binding-splic	1,03	0,914416326714056	0,980665300581806	28,1238312869463	28,7908543389162	28,8240709718089	28,7093526612442	28,525661745392	28,5793494389656	2582000320695,11	2647899893009,36	−0,036359400548119	0,0138225947796116	0,0033019177220485	1,0255226816922	0.0363594005481208	0,975112513698788	-0.036359400548120	2327
Q9D6K7	sp|Q9D6K7|TTC33_MOUSE Tetratricopeptide repeat protein 33 OS=Mus musculus GN=Ttc33 PE=2 SV=1;sp|Q9D6K7-2|TTC33_MOUSE Isoform 2 of Tetratricopeptide repeat protein 33 OS=Mus musculus GN=Ttc33	1,03	0,899960377908293	0,977644369603516	24,8042210143347	25,0631252435222	25,4374912672445	24,9662926902838	25,2008916720812	25,2133112519035	79706143961,0816	81741843776,0495	−0,036383850014791	0,0126838306886898	0,0055386855074495	1,02554006145325	0.0363838500147921	0,97509598853012	-0.036383850014792	2328
Q8BQM4	sp|Q8BQM4|HEAT3_MOUSE HEAT repeat-containing protein 3 OS=Mus musculus GN=Heatr3 PE=2 SV=1	1,03	0,83981480905824	0,959771286845933	23,8261006818307	23,3953782803666	23,6030522836631	23,6693854597697	23,6005714162421	23,6309486811925	17901978635,0202	18363579880,5843	−0,036728280075646	0,00912425075561358	0,0014591742979533	1,02578492886038	0.0367282800756434	0,974863221192936	-0.036728280075643	2329
O88351	sp|O88351|IKKB_MOUSE Inhibitor of nuclear factor kappa-B kinase subunit beta OS=Mus musculus GN=Ikbkb PE=1 SV=1	1,03	0,941608197331762	0,986704967868576	26,0481993746539	26,0031584198129	25,5098805984377	25,5517501914311	26,4832460049278	25,6027289502231	169098315454,009	173465004969,895	−0,036782353408126	0,0115513865114943	0,0202364402214893	1,02582337679806	0.0367823534081317	0,974826683245743	-0.036782353408131	2330
Q6PDC0	sp|Q6PDC0|RUN3B_MOUSE RUN domain-containing protein 3B OS=Mus musculus GN=Rundc3b PE=2 SV=1	1,03	0,998094641395662	0,999958878348632	23,220546028412	22,0969453884394	0	22,8773938140701	0	22,5167234850316	3633948,9221106	3727962,63908926	−0,036849260108750	0,866823608021048	0,866107405513739	1,02587095168197	0.0368492601087489	0,974781475545681	-0.036849260108749	2331
Q61503	sp|Q61503|5NTD_MOUSE 5-nucleotidase OS=Mus musculus GN=Nt5e PE=1 SV=2	1,03	0,871662845782687	0,971153082230637	21,504041661994	22,0488781458796	21,7715837835218	21,7767731508675	21,7715837835218	21,8534467936578	2862137414,74316	2936843619,2501	−0,037173507935795	0,0125113597428398	0,0021026522616032	1,0261015435954	0.0371735079357961	0,974562416596763	-0.037173507935796	2332
Q8K0E8	sp|Q8K0E8|FIBB_MOUSE Fibrinogen beta chain OS=Mus musculus GN=Fgb PE=2 SV=1	1,03	0,895791875073803	0,977357990376388	27,3089720088055	27,8436592712793	27,8617602051029	27,8490417699696	27,596320761955	27,6464734713961	1041271656109,82	1068501876360,27	−0,037242940751540	0,0113495101310623	0,0048308552181004	1,02615092813741	0.0372429407515454	0,97451551480358	-0.037242940751545	2333
Q99LI8	sp|Q99LI8|HGS_MOUSE Hepatocyte growth factor-regulated tyrosine kinase substrate OS=Mus musculus GN=Hgs PE=1 SV=2	1,03	0,879473550446438	0,973716798448825	29,9440608870272	29,5939809288885	29,3868050777055	29,6699505699435	29,7557574407144	29,5767627429087	7467755722720,82	7663502581093,95	−0,037329185932628	0,00950235791317232	0,0030175356169520	1,02621227389342	0.0373291859326294	0,974457259418684	-0.037329185932629	2334
O54734	sp|O54734|OST48_MOUSE Dolichyl-diphosphooligosaccharide--protein glycosyltransferase 48 kDa subunit OS=Mus musculus GN=Ddost PE=1 SV=2	1,03	0,896457010413956	0,977357990376388	28,0545669515707	27,3902087291243	27,6776121109565	27,6244221741423	27,8080490843163	27,7681093764479	1079439021108,39	1107943686276,9	−0,037602809065663	0,0120250458769851	0,0034822364666956	1,02640692490368	0.0376028090656563	0,974272460305003	-0.037602809065656	2335
P28650	sp|P28650|PURA1_MOUSE Adenylosuccinate synthetase isozyme 1 OS=Mus musculus GN=Adssl1 PE=1 SV=2;sp|P28650-2|PURA1_MOUSE Isoform 2 of Adenylosuccinate synthetase isozyme 1 OS=Mus musculus GN=Adssl1	1,03	0,774180336674286	0,938569590855925	29,1625520471545	28,9001884639333	29,0211872608148	29,0120311248533	29,1550985480598	28,995304264796	4042869887762,4	4150063062766,49	−0,037753485362786	0,00452368668307561	0,0030228993872642	1,02651412931407	0.0377534853627842	0,974170711774044	-0.037753485362784	2336
Q99KH8	sp|Q99KH8|STK24_MOUSE Serine/threonine-protein kinase 24 OS=Mus musculus GN=Stk24 PE=1 SV=1	1,03	0,80675709455994	0,948497813008292	27,918409250953	27,8961549127051	28,0758284429146	27,8740286712693	28,1697161700063	27,9254032200445	1394370538087,59	1431460002953,51	−0,037873368002337	0,00350258469623835	0,0056444642071993	1,02659943239821	0.0378733680023333	0,974089765142307	-0.037873368002333	2337
Q8BTR5	sp|Q8BTR5|DUS28_MOUSE Dual specificity phosphatase 28 OS=Mus musculus GN=Dusp28 PE=1 SV=1	1,03	0,834323868934826	0,957953242267243	24,6889656652559	24,4180530212713	24,656098949068	24,5790747392146	24,473229654249	24,7900819061277	47676568061,2498	48953111390,6184	−0,038120169481697	0,00601275535227788	0,0065534913873609	1,02677506752853	0.0381201694816976	0,973923142102603	-0.038120169481697	2338
Q61990	sp|Q61990|PCBP2_MOUSE Poly(rC)-binding protein 2 OS=Mus musculus GN=Pcbp2 PE=1 SV=1;sp|Q61990-3|PCBP2_MOUSE Isoform 3 of Poly(rC)-binding protein 2 OS=Mus musculus GN=Pcbp2	1,03	0,751356292075075	0,928854093158237	30,6442031737676	30,4045709486719	30,4762511723909	30,4692900893302	30,620839005307	30,5147582056784	17766606375785	18245916790888,4	−0,038405506422966	0,00403158500642609	0,0025468646514589	1,0269781636946	0.0384055064229684	0,973730538147431	-0.038405506422968	2339
Q99KK7	sp|Q99KK7|DPP3_MOUSE Dipeptidyl peptidase 3 OS=Mus musculus GN=Dpp3 PE=2 SV=2	1,03	0,865108138060804	0,968127672252956	30,5591116316026	30,312297883722	30,0981104206294	30,4957910271004	30,2058798248574	30,348015515816	14763415517926	15163243977320,5	−0,038551904544019	0,00760781015982242	0,0047764495251890	1,02708238204832	0.038551904544017	0,973631733421126	-0.038551904544016	2340
P47968	sp|P47968|RPIA_MOUSE Ribose-5-phosphate isomerase OS=Mus musculus GN=Rpia PE=2 SV=2	1,03	0,873017222091046	0,971946325992459	22,9454843610105	23,1840498105516	23,4012908958398	23,3326444849489	23,2829667656251	22,9959305482374	11631022155,1063	11948209437,9714	−0,038816542707098	0,00983677620442707	0,0078334555649815	1,02727080033339	0.0388165427070927	0,973453153419199	-0.038816542707092	2341

O08601	sp|O08601|MTP_MOUSE Microsomal triglyceride transfer protein large subunit OS=Mus musculus GN=Mttp PE=2 SV=2;sp|O08601-2|MTP_MOUSE Isoform 2 of Microsomal triglyceride transfer protein large subunit OS=Mus musculus GN=Mttp	1,03	0,933737019330449	0,98467134375593	23,245900105076	22,1831864358735	22,4108797662297	22,6688521928451	22,5971910117934	22,6546864138481	6619741133,25644	6800372358,0237	−0,03883894290296	0,0247435149472231	0,001676267277719	1,02728675051352	0.0388389429029615	0,97343803908706	-0.03883894290296	2342
Q3ULD5	sp|Q3ULD5|MCCB_MOUSE Methylcrotonoyl-CoA carboxylase beta chain, mitochondrial OS=Mus musculus GN=Mccc2 PE=1 SV=1	1,03	0,886851435322554	0,975149870380105	28,6000440221418	29,1644059935391	29,0763948194066	29,1143759145037	28,8755859989111	28,9317927292942	3728206035889,58	3830123692288,64	−0,03890939273829	0,0104893158639326	0,004308847536982	1,02733691631255	0.0389093927382921	0,973390505219386	-0.0389093927382	2343
Q7TME0	sp|Q7TME0|LPPR4_MOUSE Lipid phosphate phosphatase-related protein type 4 OS=Mus musculus GN=Lppr4 PE=2 SV=2	1,03	0,833915484803845	0,957953242267243	26,7786940840882	26,7026272203015	26,3667324253046	26,6258838312392	26,7044826370826	26,5997264252204	362401267961,13	372448383674,32	−0,03945249828071	0,00823609995403734	0,002046360620989	1,02772373223117	0.0394524982807114	0,97302413930738	-0.03945249828071	2344
Q80U56	sp|Q80U56|AVL9_MOUSE Late secretory pathway protein AVL9 homolog OS=Mus musculus GN=Avl9 PE=2 SV=2	1,03	0,71544482500805	0,918558942968132	27,9865800133104	28,2082847129067	28,1435044081122	28,1788730675729	28,0674871197023	28,1742570180425	1618935113106,39	1663933901871,99	−0,03955296137920	0,00405506073571505	0,002239445165152	1,02779530099836	0.0395529613792068	0,97295638443632	-0.03955296137920	2345
P59016	sp|P59016|VP33B_MOUSE Vacuolar protein sorting-associated protein 33B OS=Mus musculus GN=Vps33b PE=1 SV=1	1,03	0,898266121159916	0,977644369603516	27,860378365634	28,586024909645	28,1272472497798	28,1859618455125	28,1842803916153	28,2856963956054	1751015185532,8	1799709197637,85	−0,03957221495536	0,0130192446124986	0,002057974318270	1,02780901759583	0.0395722149553712	0,972943399873179	-0.03957221495537	2346
Q921W4	sp|Q921W4|QORL1_MOUSE Quinone oxidoreductase-like protein 1 OS=Mus musculus GN=Cryzl1 PE=2 SV=1;sp|Q921W4-2|QORL1_MOUSE Isoform 2 of Quinone oxidoreductase-like protein 1 OS=Mus musculus GN=Cryzl1	1,03	0,921670704869623	0,982113422958876	27,5987969361743	27,268187615531	27,9313864300688	27,386252220826	28,0318362877081	27,4627343947558	968928824803,933	995928164330,048	−0,03965099276088	0,0120147255085555	0,0127675631770412	1,0278651422426	0.0396509927608851	0,972890274125065	-0.03965099276088	2347
Q9DBL2	sp|Q9DBL2|GDAP2_MOUSE Ganglioside-induced differentiation-associated protein 2 OS=Mus musculus GN=Gdap2 PE=2 SV=1;sp|Q9DBL2-2|GDAP2_MOUSE Isoform 2 of Ganglioside-induced differentiation-associated protein 2 OS=Mus musculus GN=Gdap2	1,03	0,679213524206179	0,903825912158267	23,0829978435626	23,1990977632119	23,3066932565955	23,252854447264	23,19435933754	23,2244008906573	11857933266,0329	12189875395,036	−0,03983079612056	0,00482295992458994	0,001259529320086	1,02799325325552	0.0398307961205597	0,972769030179072	-0.03983079612055	2348
Q9CX34	sp|Q9CX34|SUGT1_MOUSE Suppressor of G2 allele of SKP1 homolog OS=Mus musculus GN=Sugt1 PE=2 SV=3	1,03	0,833499883133599	0,957953242267243	30,5561361051944	30,1201555119806	30,2476801384455	30,2888262852398	30,3419338493043	30,3763944677086	14540959630560,6	14949787487046,1	−0,04000249344106	0,00739607495833371	0,001454184903406	1,02811560356899	0.0400024934410745	0,972653266353134	-0.04000249344107	2349
Q8CGC7	sp|Q8CGC7|SYEP_MOUSE Bifunctional glutamate/proline--tRNA ligase OS=Mus musculus GN=Eprs PE=1 SV=4	1,03	0,896532772427691	0,977357990376388	26,7434074548887	27,3802624477312	27,3601226677854	27,1900125023832	27,1149582581361	27,2620275033788	625154208678,811	642735691391,797	−0,04001348045859	0,013328342367642	0,0027047650862184	1,02812343333678	0.0400134804585973	0,972645859023459	-0.040013480458597	2350
Q8BIQ5	sp|Q8BIQ5|CSTF2_MOUSE Cleavage stimulation factor subunit 2 OS=Mus musculus GN=Cstf2 PE=1 SV=2;sp|Q8BIQ5-2|CSTF2_MOUSE Isoform 2 of Cleavage stimulation factor subunit 2 OS=Mus musculus GN=Cstf2	1,03	0,899839064132361	0,977644369603516	26,6843310444171	27,3081622510207	26,7306224117162	26,8651963070805	26,8246682302946	27,1164940541076	485140916767,253	498790928444,45	−0,04003136547016	0,0129173949911743	0,005869230144113	1,02813617900579	0.0400313654701639	0,972633801260647	-0.040031365470164	2351
P97447	sp|P97447|FHL1_MOUSE Four and a half LIM domains protein 1 OS=Mus musculus GN=Fhl1 PE=1 SV=3;sp|Q9WUH4|FHL1_RAT Four and a half LIM domains protein 1 OS=Rattus norvegicus GN=Fhl1 PE=2 SV=1	1,03	0,889709071220537	0,976830327013275	27,8762541482515	28,3270681210585	28,3714205600626	28,3564695698073	28,3294382730544	27,9723912373162	1751652848759,74	1801125794524,11	−0,04018206289070	0,00971850330604038	0,007596572064547	1,02824357908555	0.0401820628907028	0,972532209624236	-0.040182062890702	2352
Q8BMS9	sp|Q8BMS9|RASF2_MOUSE Ras association domain-containing protein 2 OS=Mus musculus GN=Rassf2 PE=2 SV=1;sp|Q8CB96-2|RASF4_MOUSE Isoform 2 of Ras association domain-containing protein 4 OS=Mus musculus GN=Rassf4;sp|Q8CB96|RASF4_MOUSE Ras association domain	1,03	0,930968159765926	0,98433518830764	23,7625704964307	22,9717251193296	22,7554239515794	23,3482940068699	23,2244008906573	23,0008472762563	11472742663,6856	11797821404,0205	−0,04031015287688	0,0228890010949129	0,007592948626522	1,02833487596334	0.0403101528768734	0,972445866978108	-0.040310152876873	2353
P70380	sp|P70380|IL18_MOUSE Interleukin-18 OS=Mus musculus GN=Il18 PE=1 SV=2	1,03	0,956745718190914	0,990217657408697	23,141829678946	23,4808724835156	22,6427415317092	23,4260786804776	22,2206266183963	23,7026990686974	10646331731,2631	10948498084,0093	−0,04037654904819	0,0182603857088901	0,0340905775097642	1,02838220340804	0.0403765490481892	0,97240111379409	-0.040376549048189	2354
Q8VHJ5	sp|Q8VHJ5|MARK1_MOUSE Serine/threonine-protein kinase MARK1 OS=Mus musculus GN=Mark1 PE=1 SV=2	1,03	0,838690042973505	0,959746519741951	29,5501594072729	29,4291202576836	29,089228896999	29,4005511866025	29,3627419253404	29,3897530370284	5613354016131,53	5773783589653,78	−0,04065398585218	0,00814029098684641	0,000662780416552	1,02857998498958	0.0406539858521872	0,972214134625737	-0.040653985852187	2355
P62204	sp|P62204|CALM_MOUSE Calmodulin OS=Mus musculus GN=Calm1 PE=1 SV=2;sp|Q9D6P8|CALL3_MOUSE Calmodulin-like protein 3 OS=Mus musculus GN=Calml3 PE=2 SV=1	1,03	0,886683550409612	0,975149870380105	30,4270212494486	30,59946784483	30,507269860691	30,1742185244125	30,6159575258508	30,8286913985065	17818404237326,4	18331142088852	−0,04092853398102	0,0028282113154391	0,0109316091893554	1,02877574471296	0.040928533981026	0,972029137680548	-0.040928533981026	2356

Q9D0R2	sp|Q9D0R2|SYTC_MOUSE Threonine--tRNA ligase, cytoplasmic OS=Mus musculus GN=Tars PE=1 SV=2;sp|P26639|SYTC_HUMAN Threonine--tRNA ligase, cytoplasmic OS=Homo sapiens GN=TARS PE=1 SV=3;sp|P26639-2|SYTC_HUMAN Isoform 2 of Threonine--tRNA ligase, cytoplasmic	1,03	0,85961062148408	0,966502999979251	30,153305359808	29,7910678192371	29,6188712147222	29,9548618864297	29,8687792032057	29,8255701629118	9238630699654,44	9507199004606,23	−0,041341320280887	0,00913735492436245	0,002202583423363€	1,02907014185141	0.041341320280882	0,971751058874263	-0.0413413202808882	2357
P62996	sp|P62996|TRA2B_MOUSE Transformer-2 protein homolog beta OS=Mus musculus GN=Tra2b PE=2 SV=1	1,03	0,863986957131339	0,967731774645151	27,1190534987677	26,6820304486377	26,6793021818755	26,9324340242253	26,9415112903228	26,6924412252218	447434223124,783	460446349018,722	−0,041357455242332	0,00943484418932327	0,005259757654765€	1,02908165093646	0.041357455242339	0,971740190965419	-0.0413574552423333	2358
O35089	sp|O35089|CNIH2_MOUSE Protein cornichon homolog 2 OS=Mus musculus GN=Cnih2 PE=1 SV=2	1,03	0,926721016555192	0,983810446072053	24,3751804819673	23,9631070192037	23,6540441959298	23,5596170779061	24,2686184132209	24,2505125478808	2642149972O,8398	27193650522,7984	−0,04155747597366€	0,0150762838989004	0,016823965378934€	1,02922433662422	0.041557475973673	0,971605474545933	-0.0415574759736731	2359
Q9CZ04	sp|Q9CZ04|CSN7A_MOUSE COP9 signalosome complex subunit 7a OS=Mus musculus GN=Cops7a PE=1 SV=2;sp|Q9CZ04-2|CSN7A_MOUSE Isoform 2 of COP9 signalosome complex subunit 7a OS=Mus musculus GN=Cops7a	1,03	0,856098514465157	0,964447573623737	29,7312890475709	29,8993775648934	30,2081872493644	30,1381873875547	29,9201519045597	29,8687792032057	10127591234869,2	10429987277133,7	−0,042446316341261	0,00807728371257672	0,004771800995817€	1,02985863422522	0.0424463163412626	0,971007055499726	-0.0424463163412620	2360
P17809	sp|P17809|GTR1_MOUSE Solute carrier family 2, facilitated glucose transporter member 1 OS=Mus musculus GN=Slc2a1 PE=1 SV=4	1,03	0,866455240038136	0,968655247562697	25,698996365124	25,3815883916897	25,109689615665	25,4069322364685	25,3674906455573	25,5041690285268	107071012998,661	110269951790,026	−0,042471758067612	0,0116135334449103	0,002766685915227€	1,02987679579911	0.0424717580676196	0,970989932076361	-0.0424717580676196	2361
O35857	sp|O35857|TIM44_MOUSE Mitochondrial import inner membrane translocase subunit TIM44 OS=Mus musculus GN=Timm44 PE=1 SV=2	1,03	0,863377856329278	0,967731774645151	27,2501225437999	26,7761234567643	26,9501760086428	27,1684490497881	27,0697573276934	26,8268300909926	527883086861,566	543708352234,808	−0,0426145469785€	0,00888294876201436	0,006506297383405€	1,0299787315925	0.0426145469785616	0,970893834335641	-0.0426145469785616	2362
Q5DTY9	sp|Q5DTY9|KCD16_MOUSE BTB/POZ domain-containing protein KCTD16 OS=Mus musculus GN=Kctd16 PE=1 SV=2	1,03	0,927936423218678	0,984256838321484	28,2437154901872	27,5836630656943	27,139097165185	27,6376773679146	27,8099832106879	27,6075461660519	1024772658181,39	1055535054423,17	−0,042670602567767	0,0200973165975659	0,003945194266937€	1,03001875196043	0.0426706025677769	0,970856111208366	-0.0426706025677777	2363
Q9Z130	sp|Q9Z130|HNRDL_MOUSE Heterogeneous nuclear ribonucleoprotein D-like OS=Mus musculus GN=Hnrnpdl PE=1 SV=1	1,03	0,834975338683216	0,957953242267243	27,6454609497024	27,1995228618436	27,539642809425	27,5571437909233	27,5162976633754	27,4002419470032	844104557353,979	869537936845,81	−0,042827258446962	0,00848466868376284	0,002960935722867€	1,03013060321764	0.0428272584469617	0,970750695956879	-0.0428272584469617	2364
Q99JP0	sp|Q99JP0|M4K3_MOUSE Mitogen-activated protein kinase kinase kinase kinase 3 OS=Mus musculus GN=Map4k3 PE=1 SV=4	1,03	0,914535670727587	0,980665300581806	25,8217373765899	25,1410841300026	25,3163007018966	25,2783415939017	25,8351165872011	25,2553298211587	110289445488,847	113635599204,404	−0,043120132004335	0,0138999420750008	0,01289658299262З	1,03033974557335	0.0431201320043371	0,97055364921746	-0.0431201320043371	2365
Q61466	sp|Q61466|SMRD1_MOUSE SWI/SNF-related matrix-associated actin-dependent regulator of chromatin subfamily D member 1 OS=Mus musculus GN=Smarcd1 PE=1 SV=3;sp|Q61466-2|SMRD1_MOUSE Isoform 1 of SWI/SNF-related matrix-associated actin-dependent regulator of c	1,03	0,88254481357534	0,973716798448825	26,7270880304719	27,3567816774972	27,3032177671707	27,1940541511622	27,122691076725	27,1601478692373	605323678912,922	623718115772,848	−0,043187375160414	0,0128688719393154	0,001314345213873€	1,03038777021404	0.0431873751604152	0,970508413344491	-0.0431873751604152	2366
O88746	sp|O88746|TOM1_MOUSE Target of Myb protein 1 OS=Mus musculus GN=Tom1 PE=1 SV=1	1,03	0,894226284253469	0,977357990376388	27,6784770577248	28,2250895728033	28,2478477131411	28,304589714348	28,0782614029079	27,8588173640719	1521125211871,98	1567583159631,3	−0,043403065606616	0,0114920525704178	0,007937700145399€	1,03054183008521	0.0434030656066188	0,97036332811189	-0.0434030656066188	2367
Q2TPA8	sp|Q2TPA8|HSDL2_MOUSE Hydroxysteroid dehydrogenase-like protein 2 OS=Mus musculus GN=Hsdl2 PE=1 SV=1	1,03	0,837597467265996	0,959408706205426	28,102857773251	28,155149069996	28,5539886874427	28,2627967575825	28,3845625088349	28,2559271319953	1895805967857,36	1954382623990,8	−0,043901627380833	0,00872828560758125	0,002557008473572€	1,03089802286024	0.043901627380829	0,970028051101975	-0.0439016273808282	2368
Q8BH57	sp|Q8BH57|WDR48_MOUSE WD repeat-containing protein 48 OS=Mus musculus GN=Wdr48 PE=1 SV=1;sp|Q8BH57-2|WDR48_MOUSE Isoform 2 of WD repeat-containing protein 48 OS=Mus musculus GN=Wdr48;sp|Q8BH57-3|WDR48_MOUSE Isoform 3 of WD repeat-containing protein 48 O	1,03	0,845345969246288	0,960697384679244	29,034879459619	28,8422515451511	28,5025208578432	28,7923191655361	28,8605637488314	28,8185754978522	3196946615442	329629250912 9,47	−0,044149617946110	0,00936141782747994	0,001194261525639€	1,03107524323603	0.0441496179461097	0,969861323468011	-0.0441496179461097	2369
Q8BK08	sp|Q8BK08|TMM11_MOUSE Transmembrane protein 11, mitochondrial OS=Mus musculus GN=Tmem11 PE=2 SV=1	1,03	0,863329876636704	0,967731774645151	24,9510957123413	24,6282909811731	24,5308608808026	24,4861190808891	24,8877355888685	24,8284747330514	53525063517,2739	55193432181,7784	−0,044281999107126	0,00890400759922317	0,008765248672917€	1,0311698586582	0.0442819991071274	0,969772333436164	-0.0442819991071274	2370
Q91VA6	sp|Q91VA6|PDIP2_MOUSE Polymerase delta-interacting protein 2 OS=Mus musculus GN=Poldip2 PE=2 SV=1	1,03	0,7431531306715	0,926639317607059	26,2965204560717	26,102075650852	26,3230679595192	26,1808314726337	26,239003526529	26,3947233703869	248959156245,593	256788713311,35	−0,044672716806244	0,00459817698998483	0,004209544873738€	1,03144916292226	0.0446727168062412	0,969509730529847	-0.0446727168062412	2371
Q922Q9	sp|Q922Q9|CHID1_MOUSE Chitinase domain-containing protein 1 OS=Mus musculus GN=Chid1 PE=2 SV=1;sp|Q922Q9-2|CHID1_MOUSE Isoform 2 of Chitinase domain-containing protein 1 OS=Mus musculus GN=Chid1	1,03	0,794645676336825	0,943719708239887	27,7598510714024	27,3749832819008	27,4549509257709	27,5217488359946	27,5648173464815	27,596320761955	903849251604,17	932338995718,641	−0,044772436969679	0,00737719430581021	0,001358266448083€	1,03152045992615	0.0447724369696794	0,969442719605961	-0.0447724369696794	2372
Q9Z2D1	sp|Q9Z2D1|MTMR2_MOUSE Myotubularin-related protein 2 OS=Mus musculus GN=Mtmr2 PE=1 SV=3	1,03	0,670500094160242	0,900147863766145	26,7900481326491	27,0119621691426	26,9078396230827	26,9359933509139	26,9947363166119	26,8725182103567	483000394945,641	498273997919,631	−0,044914921211281	0,00412689994466594	0,002269376106324€	1,03162234054841	0.0449149212112826	0,9693469796984	-0.0449149212112826	2373

Q8K2V6	sp|Q8K2V6|IPO11_MOUSE Importin-11 OS=Mus musculus GN=Ipo11 PE=1 SV=1;sp|Q8K2V6-2|IPO11_MOUSE Isoform 2 of Importin-11 OS=Mus musculus GN=Ipo11	1,03	0,965970767541406	0,991306002794532	26,8968700970283	24,605749863721	26,1186864653252	26,190885557456	26,1892833237637	25,3346416265303	172518240299,015	177979966992,105	−0,04496595831201	0,0450274826244068	0,0190655083176133	1,03165883609538	0.0449659583120164	0,969312688470539	-0.0449659583120l6	2374
P68368	sp|P68368|TBA4A_MOUSE Tubulin alpha-4A chain OS=Mus musculus GN=Tuba4a PE=1 SV=1	1,03	0,317310507862914	0,722907157640623	37,7354459507886	37,8289878833996	37,8289878833996	37,8289878833996	37,8289878833996	37,8289878833996	26024303314460000	26848541212920500	−0,04498416076440	0,00142882521284277	0	1,0316718525949	0.0449841607643978	0,969300458750293	-0.044984160764397	2375
Q9WVC2-2	sp|Q9WVC2-2|LYNX1_MOUSE Isoform 2 of Ly-6/neurotoxin-like protein 1 OS=Mus musculus GN=Lynx1	1,03	0,792478742127375	0,942626050973835	25,0225487947601	24,8042210143347	24,7304936940114	24,7358047995479	24,9050721699232	25,0100063220039	62125425330,2593	64094716003,0232	−0,04502160146956	0,00611113699385793	0,005559995822963	1,0316986268069	0.0450216014695628	0,969275303869495	-0.045021601469562	2376
Q9R1V6-5	sp|Q9R1V6-5|ADA22_MOUSE Isoform 3 of Disintegrin and metalloproteinase domain-containing protein 22 OS=Mus musculus GN=Adam22;sp|Q9R1V6-4|ADA22_MOUSE Isoform 2 of Disintegrin and metalloproteinase domain-containing protein 22 OS=Mus musculus GN=Adam22;s	1,03	0,907608810590209	0,980398950801751	28,8294358832546	28,1167813912022	28,0805683689805	28,2975385560422	28,5978831612972	28,2250895728033	2036516530563,39	2101145447997,82	−0,04507250856868	0,0148997781179576	0,0069665657073387	1,03173503208273	0.0450725085686819	0,969241102515766	-0.045072508568681	2377
P63017	sp|P63017|HSP7C_MOUSE Heat shock cognate 71 kDa protein OS=Mus musculus GN=Hspa8 PE=1 SV=1;sp|P11142|HSP7C_HUMAN Heat shock cognate 71 kDa protein OS=Homo sapiens GN=HSPA8 PE=1 SV=1;sp|P11142-2|HSP7C_HUMAN Isoform 2 of Heat shock cognate 71 kDa protein	1,03	0,431207681130747	0,775108167118334	36,1442586580311	36,084202935126	36,0203651980554	36,1142307965785	36,084202935126	36,1442586580311	4684062874181770	4832937213961810	−0,04513981116643	0,0017170537177801	0,000831468947011	1,03178316426977	0.0451398111664379	0,969195887885744	-0.04513981116643	2378
Q9CYN2	sp|Q9CYN2|SPCS2_MOUSE Signal peptidase complex subunit 2 OS=Mus musculus GN=Spcs2 PE=1 SV=1	1,03	0,849620004405568	0,962394740430361	26,0422096112122	25,7605581739283	25,9677083343354	25,6741775613297	26,0516722330455	26,1388382616424	181313250816,117	187097558070,194	−0,04530636454710	0,00562960445542068	0,009515933208483	1,03190228639132	0.045306364547099	0,969084004549611	-0.045306364547098	2379
Q9QYF9	sp|Q9QYF9|NDRG3_MOUSE Protein NDRG3 OS=Mus musculus GN=Ndrg3 PE=1 SV=1	1,03	0,790337239276123	0,942055274591853	30,6402261423119	30,2825025623271	30,5474125413229	30,4511791725536	30,6173890438023	30,4957910271004	17444527097232,2	18001083764518,1	−0,04530927924922	0,00608831695142237	0,0028185160744362	1,03190437116373	0.045309279249221	0,969082046694159	-0.045309279249221	2380
P47962	sp|P47962|RL5_MOUSE 60S ribosomal protein L5 OS=Mus musculus GN=Rpl5 PE=1 SV=3	1,03	0,876695112968457	0,973176600088977	25,2907819321046	25,5972870851289	25,8007350073515	25,6181538792891	25,343368132376	25,8217373765899	126427476810,018	130471388711,923	−0,04542342825061	0,01004212768194	0,0093796291032286	1,03198602079184	0.0454234282506207	0,969005373961077	-0.045423428250620	2381
P80315	sp|P80315|TCPD_MOUSE T-complex protein 1 subunit delta OS=Mus musculus GN=Cct4 PE=1 SV=3	1,03	0,834537626702749	0,957953242267243	31,8676070981556	32,3364067192113	32,2862250679884	32,1724065672547	32,1512238864603	32,2621420111148	92980690806227,4	95989265964647,4	−0,04594194044874	0,0080029195620046	0,0018289664997611	1,03235698866434	0.0459419404487404	0,968657170901504	-0.045941940448740	2382
Q99LB6-2	sp|Q99LB6-2|MAT2B_MOUSE Isoform 2 of Methionine adenosyltransferase 2 subunit beta OS=Mus musculus GN=Mat2b;sp|Q99LB6|MAT2B_MOUSE Methionine adenosyltransferase 2 subunit beta OS=Mus musculus GN=Mat2b PE=2 SV=1	1,03	0,746406014063604	0,927700293161169	29,3723329959481	29,0763948194066	29,2657804305218	29,1759515219542	29,3290713947903	29,3052903367405	4988565030470,92	5150445943510,62	−0,04607246978972	0,00512647402582702	0,0028152006440675	1,0324503964669	0.0460724697897256	0,968569534596579	-0.046072469789725	2383
Q9CQU5	sp|Q9CQU5|ZWINT_MOUSE ZW10 interactor OS=Mus musculus GN=Zwint PE=2 SV=1	1,03	0,898127969041856	0,977644369603516	26,1606183781872	26,870933519598	26,868788726629	26,8202978882426	26,6308893490547	26,5451722092463	368772911224,561	380766875261,993	−0,04617529283935	0,0153747615125863	0,0052795593269447	1,0325239833848	0.0461752928393555	0,968500505646193	-0.046175292839355	2384
O35143	sp|O35143|ATIF1_MOUSE ATPase inhibitor, mitochondrial OS=Mus musculus GN=Atpif1 PE=1 SV=2	1,03	0,869875007682384	0,970590624353574	27,1512361220385	27,2868283286901	26,8911690458988	26,8268300909926	27,3198293289793	27,2789110284764	593762057154,636	613138580184,547	−0,04632828088212	0,00741676076227906	0,0100798998396087	1,03263348136923	0.0463282808821246	0,968397808169111	-0.046328280882124	2385
P61290	sp|P61290|PSME3_MOUSE Proteasome activator complex subunit 3 OS=Mus musculus GN=Psme3 PE=1 SV=1	1,03	0,798983072265576	0,94491510292169	27,5493288208279	27,539642809425	27,8915107942788	27,7647974432772	27,5899208736384	27,7229066479733	1029568407156,1	1063452338588,71	−0,04671568707761	0,00724556487022407	0,0032971713437278	1,03291081116815	0.0467156870776217	0,968137799689663	-0.046715687077621	2386
Q8C1B7-3	sp|Q8C1B7-3|SEP11_MOUSE Isoform 3 of Septin-11 OS=Mus musculus GN=Sept11;sp|Q8C1B7-2|SEP11_MOUSE Isoform 2 of Septin-11 OS=Mus musculus GN=Sept11;sp|Q8C1B7|SEP11_MOUSE Septin-11 OS=Mus musculus GN=Sept11 PE=1 SV=4	1,03	0,8205487671957 5	0,953281508651525	32,9720957537316	32,574532753597	33,0034445863308	32,8095502245621	32,9002084952502	32,9377590356243	184749942505120	190849431908928	−0,04686097676904	0,00727844417173442	0,0020044744814770	1,03301483789982	0.0468609767690433	0,968040306209983	-0.046860976769043	2387
Q9D8W7	sp|Q9D8W7|OCAD2_MOUSE OCIA domain-containing protein 2 OS=Mus musculus GN=Ociad2 PE=1 SV=1	1,03	0,718961376103589	0,920149210230605	23,6951786116361	23,7026990686974	23,5141970803436	23,5843529479607	23,6248549139189	23,800646758244	18432076163,0081	19042735744,372	−0,04702217277416	0,00451518163740631	0,0048577517318332	1,03313026573694	0.0470221727741691	0,967932150634166	-0.047022172774169	2388
Q8C7E9	sp|Q8C7E9|CSTFT_MOUSE Cleavage stimulation factor subunit 2 tau variant OS=Mus musculus GN=Cstf2t PE=1 SV=2	1,03	0,931714376200543	0,984645297689002	24,0268686400196	24,9961132911365	24,9294153710934	25,1239866486859	24,35916318947	24,5687279499805	50781558336,5273	52493713825,2004	−0,04784000121804	0,0219614696046449	0,0160111346757315	1,03371608798073	0.0478400012180505	0,967383609123667	-0.047840001218050	2389
Q61418	sp|Q61418|CLCN4_MOUSE H(+)/Cl(-) exchange transporter 4 OS=Mus musculus GN=Clcn4 PE=2 SV=2	1,03	0,896051122129596	0,977357990376388	25,149119368486	24,8599080669657	24,3182133554805	24,9546627427534	24,7320198340803	24,7401928293227	57540042305,779	59483123387,5363	−0,04791412176158	0,0170246316374496	0,0050888777478933	1,03376919800356	0.0479141217615875	0,967333909668832	-0.047914121761587	2390
Q8JZP2	sp|Q8JZP2|SYN3_MOUSE Synapsin-3 OS=Mus musculus GN=Syn3 PE=1 SV=2	1,03	0,902140917700728	0,977644369603516	29,0533026951177	29,9252115172164	29,7887479080092	29,6044346971218	29,6188712147222	29,643927476549	7085612059743,94	7325709523845,51	−0,04807601754890	0,0158502038062654	0,0006745887933422	1,03388521161999	0.0480760175488996	0,967225363861338	-0.048076017548899	2391

Q8BYI8	sp|Q8BYI8|K1467_MOUSE Uncharacterized protein KIAA1467 OS=Mus musculus GN=Kiaa1467 PE=2 SV=1;sp|Q8BYI8-2|K1467_MOUSE Isoform 2 of Uncharacterized protein KIAA1467 OS=Mus musculus GN=Kiaa1467	1,03	0,892240878577998	0,977069018898103	26,8289507902745	26,4229344891842	26,2191523428398	26,7527581374521	26,6258838312392	26,1926661523628	319603139529,626	330465919753,345	−0,04821991710076	0,0117189111446342	0,0110722601615999	1,0339883401637	0.0482199171007644	0,967128894163045	-0.0482199171007644	2392
Q8JZW5	sp|Q8JZW5|SH2D5_MOUSE SH2 domain-containing protein 5 OS=Mus musculus GN=Sh2d5 PE=1 SV=2	1,03	0,720912511588681	0,920750207284069	24,9676751874106	24,8549736207267	24,7331922094988	25,0138713240005	24,8371424988238	24,8053173699664	62095974808,0149	64211215646,6507	−0,04832555911791	0,00471877656270279	0,0045148523487195	1,03406405721427	0.0483255591179113	0,967058078291559	-0.048325559117911	2393
Q9CR20	sp|Q9CR20|IR3IP_MOUSE Immediate early response 3-interacting protein 1 OS=Mus musculus GN=Ier3ip1 PE=2 SV=1	1,03	0,896270795272468	0,977357990376388	25,5815878578865	25,5091258895633	25,0599359090997	25,0279479753378	25,5327595104657	25,6908631453629	105666092196,696	109281199640,891	−0,04853272986722	0,011328062289242	0,0136222307817859	1,03421255928974	0.0485327298672329	0,966919218895157	-0.0485327298672329	2394
Q8BGY7	sp|Q8BGY7|F210A_MOUSE Protein FAM210A OS=Mus musculus GN=Fam210a PE=2 SV=1	1,03	0,618095903427838	0,870166479711399	25,1746957465022	25,0954650688965	25,2712628519499	25,2215409768175	25,2882777697561	25,1330874985912	86246434750,9677	89213845377,2897	−0,04880280391739	0,00349640934846373	0,003087452854548	1,03440618310647	0.0488028039173991	0,966738227527659	-0.0488028039173399	2395
Q8R2U4	sp|Q8R2U4|NTM1A_MOUSE N-terminal Xaa-Pro-Lys N-methyltransferase 1 OS=Mus musculus GN=Ntmt1 PE=2 SV=3	1,03	0,88120387468284	0,973716798448825	25,266100588418	25,5829712431895	25,7943002713021	25,8942049257386	25,5419778283143	25,3098334236589	124527286930,608	128861676726,109	−0,04936136589794	0,0104060435678785	0,0115016348585375	1,03480674719844	0.0493613658979427	0,966364012128186	-0.0493613658979442	2396
Q8R127	sp|Q8R127|SCPDL_MOUSE Saccharopine dehydrogenase-like oxidoreductase OS=Mus musculus GN=Sccpdh PE=2 SV=1	1,04	0,849681213639959	0,962394740430361	29,6034944632424	29,2802995314596	29,3627419253404	29,6135756219551	29,5975087341519	29,139589158536	5956546443974,46	6166943837035,9	−0,05007959710012	0,00570907125187533	0,0091387426335023	1,03532204357682	0.0500795971001294	0,965883037267522	-0.0500795971001290	2397
Q3UNH4	sp|Q3UNH4|GRIN1_MOUSE G protein-regulated inducer of neurite outgrowth 1 OS=Mus musculus GN=Gprin1 PE=1 SV=2	1,04	0,841044362291671	0,959771286845933	25,482818253974	25,1667818406085	25,1686290907268	25,0338656906186	25,4189974157507	25,4695074078681	94582949769,9285	97923934736,5977	−0,05008139293204	0,00719877526650813	0,0094153688934989	1,03532333232148	0.0500813929320462	0,965881834960411	-0.0500813929320460	2398
Q9CQ65	sp|Q9CQ65|MTAP_MOUSE S-methyl-5-thioadenosine phosphorylase OS=Mus musculus GN=Mtap PE=1 SV=1	1,04	0,894145302881312	0,977357990376388	27,1039852603133	27,2638746068392	27,6263820326853	27,5100018761904	26,9509270425352	27,6376773679146	741109308799,819	767344811180,912	−0,05018866109509	0,00979339292858033	0,013347141490397	1,0354003141906	0.0501886610950919	0,965810021780537	-0.0501886610950910	2399
P62874	sp|P62874|GBB1_MOUSE Guanine nucleotide-binding protein G(I)/G(S)/G(T) subunit beta-1 OS=Mus musculus GN=Gnb1 PE=1 SV=3	1,04	0,864651730435422	0,967855537471364	33,0286081008433	33,5634667763458	33,597564845353	33,577674493259	33,4446452460359	33,2718440784801	319106677227133	330420743719205	−0,05026546461530	0,00955487544584025	0,0045868755363295	1,03545543637753	0.0502654646153019	0,965758607148204	-0.0502654646153010	2400
Q9ERI2	sp|Q9ERI2|RB27A_MOUSE Ras-related protein Rab-27A OS=Mus musculus GN=Rab27a PE=1 SV=1	1,04	0,924645956886482	0,983296707494544	22,1623409962791	21,9456502500876	22,4030699429728	22,8884318811075	21,9399864331884	21,7873155101343	4250717927,6935	4401646921,34503	−0,05033689718742	0,0103207687069991	0,0268657971786247	1,03550670644792	0.0503368971874222	0,965710790449905	-0.0503368971874222	2401
Q80UY2	sp|Q80UY2|KCMF1_MOUSE E3 ubiquitin-protein ligase KCMF1 OS=Mus musculus GN=Kcmf1 PE=2 SV=1;sp|Q80UY2-2|KCMF1_MOUSE Isoform 2 of E3 ubiquitin-protein ligase KCMF1 OS=Mus musculus GN=Kcmf1	1,04	0,950038714540541	0,988334326693467	27,0515185236898	26,8348651408146	25,6077300356249	26,0103345112904	27,1512361220385	26,4376778659567	322071014481,512	333558077675,922	−0,05055915112261	0,0293833750853048	0,0217239455276298	1,03566624339946	0.0505591511226153	0,9655620296338	-0.050559151122615	2402
Q14C51	sp|Q14C51|PTCD3_MOUSE Pentatricopeptide repeat domain-containing protein 3, mitochondrial OS=Mus musculus GN=Ptcd3 PE=2 SV=2	1,04	0,798085735699059	0,944726847417452	27,9400277759159	27,6817373259402	27,4879183321299	27,7477528357763	27,802797894039	27,6642808277829	1074877493337,95	1113219279975,99	−0,05056555883137	0,00818747811759384	0,0025143232452039	1,03567084330603	0.050565558831374	0,965557741113802	-0.050565558831373	2403
Q9WTL7	sp|Q9WTL7|LYPA2_MOUSE Acyl-protein thioesterase 2 OS=Mus musculus GN=Lypla2 PE=1 SV=1	1,04	0,856249044445179	0,964447573623737	28,7527330443723	29,2598728637706	29,3062942508444	29,2944931553283	29,0269449893171	29,1030743452272	4372255186890,91	4528917947891,15	−0,05078879534163	0,0105501719398799	0,0047304315125839	1,03583111101794	0.0507887953416334	0,965408346363796	-0.0507887953416330	2404
Q91YJ5	sp|Q91YJ5|IF2M_MOUSE Translation initiation factor IF-2, mitochondrial OS=Mus musculus GN=Mtif2 PE=1 SV=2	1,04	0,810360122181415	0,948974353607184	25,4252755505049	25,0599359090997	25,3625137240185	25,4554077650705	25,1379645009733	25,3600660664926	95517475880,7673	98943298196,7367	−0,05083727856465	0,00772659292031073	0,0064336173053544	1,03586592175285	0.0508372785646524	0,965375903387033	-0.0508372785646520	2405
Q61753	sp|Q61753|SERA_MOUSE D-3-phosphoglycerate dehydrogenase OS=Mus musculus GN=Phgdh PE=1 SV=3	1,04	0,8866616909352	0,975149870380105	30,7005833571881	31,3178401286448	31,4753765257957	31,3364515285498	31,1726430469682	31,090420390194	34246312269495	35474609171571,7	−0,05083814666798	0,013139821191427	0,0040144381212513	1,03586654505778	0.0508381466679887	0,965375322497957	-0.0508381466679880	2406
Q71RI9-2	sp|Q71RI9-2|KAT3_MOUSE Isoform 2 of Kynurenine--oxoglutarate transaminase 3 OS=Mus musculus GN=Ccbl2;sp|Q71RI9|KAT3_MOUSE Kynurenine--oxoglutarate transaminase 3 OS=Mus musculus GN=Ccbl2 PE=1 SV=1	1,04	0,843020312989499	0,959771286845933	25,5531568912004	25,4960085354994	25,9154627291353	25,8503583907544	25,7670259217681	25,4532890416025	138602512597,234	143589511525,209	−0,05099696056088	0,00886679291683322	0,0081501708407225	1,03598058097595	0.050996960560891	0,965269058477858	-0.0509969605608900	2407
Q80T74	sp|Q80T74|KLH29_MOUSE Kelch-like protein 29 OS=Mus musculus GN=Klhl29 PE=2 SV=3;sp|Q96CT2-2|KLH29_HUMAN Isoform 2 of Kelch-like protein 29 OS=Homo sapiens GN=KLHL29;sp|Q96CT2|KLH29_HUMAN Kelch-like protein 29 OS=Homo sapiens GN=KLHL29 PE=2 SV=3	1,04	0,934441263720021	0,98467134375593	22,2120966659598	22,0092765517313	21,4919382859881	21,3020994376969	22,6095143587135	21,9088833413978	3258188385,50451	3376703290,40748	−0,05154539427078	0,0169531660039586	0,0298205854781987	1,0363744789682	0.051545394270792	0,964902185738484	-0.0515453942707920	2408
P70302	sp|P70302|STIM1_MOUSE Stromal interaction molecule 1 OS=Mus musculus GN=Stim1 PE=1 SV=2	1,04	0,911093491227026	0,980429909122681	26,6630776319574	26,1422103225398	25,6116604995013	26,2872690468727	25,9719342937484	26,2649632160102	224913746777,668	233097389890,341	−0,05156100832062	0,0201123356776535	0,0067230226024688	1,03638569553849	0.0515610083206272	0,964891742818215	-0.0515610083206270	2409
Q8R1Q8	sp|Q8R1Q8|DC1L1_MOUSE Cytoplasmic dynein 1 light intermediate chain 1 OS=Mus musculus GN=Dync1li1 PE=1 SV=1	1,04	0,848784157264527	0,96228660095196	30,8120815400983	31,4047907964564	31,2551943052669	31,3178401286448	31,1656015127658	31,0970439805281	33999112840365,2	35250301816236,9	−0,05213850831778	0,00989266984594933	0,0036228733018162	1,03680063599737	0.0521385083177819	0,964505581189226	-0.0521385083177810	2410

Q7TSJ2	sp|Q7TSJ2|MAP6_MOUSE Microtubule-associated protein 6 OS=Mus musculus GN=Map6 PE=1 SV=2;sp|Q7TSJ2-2|MAP6_MOUSE Isoform 2 of Microtubule-associated protein 6 OS=Mus musculus GN=Map6	1,04	0,726854723967071	0,922335362618545	30,8577552537318	30,8138966768348	30,5591116316026	30,8709415115779	30,7450551800597	30,7235744445341	22478667270048,1	23308915560444,6	−0,052325382474850	0,005245925369026	0,0025864089904414	1,03693494282478	0.0523253824748507	0,964380655623231	-0.052325382474850	2411
Q3UCV8	sp|Q3UCV8|OTUL_MOUSE Ubiquitin thioesterase otulin OS=Mus musculus GN=Otulin PE=1 SV=1	1,04	0,93833108761183	0,985646114383563	26,3957362099935	24,8260555298346	25,3867743256002	25,6027289502231	25,7334731810382	25,3815883916897	123090868576,261	127654960620,032	−0,052525861070604	0,0311493482441448	0,0069553765710930	1,03707904653336	0.0525258610706013	0,964246653466479	-0.052525861070601	2412
A2ALU4-2	sp|A2ALU4-2|SHRM2_MOUSE Isoform 2 of Protein Shroom2 OS=Mus musculus GN=Shroom2;sp|A2ALU4|SHRM2_MOUSE Protein Shroom2 OS=Mus musculus GN=Shroom2 PE=1 SV=1	1,04	0,835700304080004	0,958131770704506	28,3498300207127	28,8917477037687	28,8367004893606	28,7643891096856	28,7651471605025	28,6581410656887	2891392698626,49	2998777371291,35	−0,052609856945695	0,0103948952427498	0,0021428469564933	1,03713942859297	0.0526098569456926	0,964190515210331	-0.052609856945692	2413
Q9DCZ4	sp|Q9DCZ4|APOO_MOUSE Apolipoprotein O OS=Mus musculus GN=Apoo PE=2 SV=1	1,04	0,879967063404112	0,973716798448825	23,8612171319506	24,2064354818055	24,4753092267965	24,2636612879042	24,4804791910647	23,9091561309391	31744562259,0021	32933807036,7613	−0,053059808195611	0,0127304962724731	0,0119091689534584	1,03746294461572	0.0530598081956128	0,963889848008408	-0.053059808195612	2414
Q5QNQ6	sp|Q5QNQ6|OSBP2_MOUSE Oxysterol-binding protein 2 OS=Mus musculus GN=Osbp2 PE=2 SV=1	1,04	0,863952730765767	0,967731774645151	23,6408917707753	23,7625704964307	23,0829978435626	23,4877467946751	23,4643347891491	23,6458420059548	15994115392,8629	16599546233,3177	−0,053602602802804	0,0154229447883397	0,0041954997502612	1,0378533495341	0.0536026028027995	0,963527265628526	-0.053602602802799	2415
Q8R1N4	sp|Q8R1N4|NUDC3_MOUSE NudC domain-containing protein 3 OS=Mus musculus GN=Nudcd3 PE=2 SV=3;sp|Q8R1N4-3|NUDC3_MOUSE Isoform 3 of NudC domain-containing protein 3 OS=Mus musculus GN=Nudcd3;sp|Q8R1N4-2|NUDC3_MOUSE Isoform 2 of NudC domain-containing protei	1,04	0,702349673829826	0,914323584944716	29,139589158536	28,8240709718089	28,8859884835841	29,0211872608148	28,961551696187	28,9794072842181	3739162872092,03	3882040682529,9	−0,054099923001448	0,0057748744937631	0,0010558786841824	1,03821117595713	0.0540999230014471	0,963195179514513	-0.054099923001447	2416
Q8K4G5-4	sp|Q8K4G5-4|ABLM1_MOUSE Isoform 4 of Actin-binding LIM protein 1 OS=Mus musculus GN=Ablim1;sp|Q8K4G5-2|ABLM1_MOUSE Isoform 2 of Actin-binding LIM protein 1 OS=Mus musculus GN=Ablim1;sp|Q8K4G5|ABLM1_MOUSE Actin-binding LIM protein 1 OS=Mus musculus GN=Ab	1,04	0,666850053189188	0,897903408907067	26,7646133507678	26,4832460049278	26,5548176288872	26,6630776319574	26,5920726184234	26,660624355741	356960985412,168	370675011314,284	−0,054388459243536	0,0054972921591748	0,0015130392192568	1,03841883696696	0.0543884592435375	0,9630025615876	-0.054388459243537	2417
Q9D7S9	sp|Q9D7S9|CHMP5_MOUSE Charged multivesicular body protein 5 OS=Mus musculus GN=Chmp5 PE=2 SV=1	1,04	0,835122489458357	0,957953242267243	26,3554364395044	26,1541626625147	26,5121165550575	26,3107417857097	26,1541626625147	26,6724274176718	275147150902,547	285957953711,97	−0,055599643703431	0,0068122781818662	0,0100765592174427	1,0392909858378	0.0555996437034338	0,962194432191552	-0.055599643703433	2418
Q9JK48	sp|Q9JK48|SHLB1_MOUSE Endophilin-B1 OS=Mus musculus GN=Sh3glb1 PE=1 SV=1;sp|Q9JK48-2|SHLB1_MOUSE Isoform 2 of Endophilin-B1 OS=Mus musculus GN=Sh3glb1	1,04	0,602778828265104	0,863913199082826	28,6821011242862	28,4722196117525	28,5454840142889	28,525661745392	28,6662319760378	28,6238583935333	2548692461617,76	2649125509332,81	−0,055758895987874	0,00372891470595177	0,0025208246283726	1,03940571458798	0.0557588959878762	0,962088225959387	-0.055758895987876	2419
Q8BK72	sp|Q8BK72|RT27_MOUSE 28S ribosomal protein S27, mitochondrial OS=Mus musculus GN=Mrps27 PE=2 SV=2	1,04	0,723660108748604	0,921631098094813	26,9096572194275	26,593052849711	26,8676217628967	26,9109358823026	26,8268300909926	26,7489665802186	431317627624,941	448381760816,209	−0,055976914544391	0,0064182789787300	0,0030193061910810	1,03956280035488	0.0559769145443935	0,961942847184048	-0.055976914544393	2420
Q6PGE7	sp|Q6PGE7|SC6A7_MOUSE Sodium-dependent proline transporter OS=Mus musculus GN=Slc6a7 PE=2 SV=1;sp|Q99884|SC6A7_HUMAN Sodium-dependent proline transporter OS=Homo sapiens GN=SLC6A7 PE=2 SV=2	1,04	0,831876123850378	0,957953242267243	26,6553553897964	27,1403798463766	27,1620716602265	27,0296671524623	27,1620716602265	26,8832357761076	524617693878,11	545512485208,257	−0,056345749591158	0,0106165027019256	0,0051610277633948	1,03982860580947	0.0563457495911582	0,96169695122162	-0.056345749591158	2421
Q6A028	sp|Q6A028|SWP70_MOUSE Switch-associated protein 70 OS=Mus musculus GN=Swap70 PE=1 SV=2	1,04	0,857809874441125	0,965434916957169	24,7031955909906	25,2820160355893	25,404963452821	25,1577695205742	25,2247645740049	25,1249989581468	82006008818,4966	85277602546,6571	−0,056437255374920	0,014912437691859	0,0020203991959469	1,03989456108517	0.0564372553749203	0,96163595562656	-0.056437255374920	2422
Q8BH74	sp|Q8BH74|NU107_MOUSE Nuclear pore complex protein Nup107 OS=Mus musculus GN=Nup107 PE=2 SV=1	1,04	0,890637385337179	0,976830327013275	24,2198565302694	23,707498296656	23,3706667882325	23,9648611641733	23,9284441294148	23,5221721571896	20962630826,4673	21799636891,7117	−0,056484317190710	0,0179924045676427	0,0103233681915483	1,03992848379449	0.056484317190712	0,961604586837745	-0.056484317190711	2423
Q6P3A8-2	sp|Q6P3A8-2|ODBB_MOUSE Isoform 2 of 2-oxoisovalerate dehydrogenase subunit beta, mitochondrial OS=Mus musculus GN=Bckdhb;sp|Q6P3A8|ODBB_MOUSE 2-oxoisovalerate dehydrogenase subunit beta, mitochondrial OS=Mus musculus GN=Bckdhb PE=1 SV=2	1,04	0,874157498584129	0,972570741527848	26,242967719549	25,6704655448343	25,4427882169683	25,8577700523384	25,9200419149704	25,6958788171413	157928330155,265	164234906201,609	−0,056490793678993	0,0159891562067271	0,0044806966572367	1,03993315220989	0.0564907936789892	0,961600270050983	-0.056490793678989	2424

Q4VC33	sp|Q4VC33|MAEA_MOUSE Macrophage erythroblast attacher OS=Mus musculus GN=Maea PE=1 SV=1;sp|Q4VC33-2|MAEA_MOUSE Isoform 2 of Macrophage erythroblast attacher OS=Mus musculus GN=Maea	1,04	0,891950683352881	0,977069018898103	26,2599380141522	25,3293144456964	26,0066860711259	25,806618885234	26,0564348417916	25,8503583907544	171065582558,774	177897024524,362	−0,056492853706603	0,0186021143632736	0,0051500591923682	1,03993463713392	0.0564928537066036	0,961598896980694	-0.056492853706603	2425
Q3UR70	sp|Q3UR70|TGFA1_MOUSE Transforming growth factor-beta receptor-associated protein 1 OS=Mus musculus GN=Tgfbrap1 PE=2 SV=1	1,04	0,913078498613981	0,980665300581806	25,0651396225333	24,5525942656835	25,3750798339753	24,9428329797975	24,6240536772299	25,5441223434088	71832623353,5532	74719205676,7049	−0,056839913926442	0,016616831666457	0,0186606685891754	1,04018483786878	0.0568399139264436	0,961367598905677	-0.0568399139264443	2426
P53668	sp|P53668|LIMK1_MOUSE LIM domain kinase 1 OS=Mus musculus GN=Limk1 PE=1 SV=1	1,04	0,971506501422904	0,993288144763853	20,6348824710516	23,5278189761452	23,0529989350732	23,0076169888695	21,1542211809417	23,1722651329508	5376123760,11314	5592549491,56288	−0,056939768740206	0,0692445997211796	0,0499278816318101	1,0402568357997	0.0569397687402056	0,961301061031959	-0.0569397687402055	2427
Q9WV60	sp|Q9WV60|GSK3B_MOUSE Glycogen synthase kinase-3 beta OS=Mus musculus GN=Gsk3b PE=1 SV=2	1,04	0,674785767004174	0,901104029547498	30,8215053344581	30,7055843535906	30,8577552537318	30,6767393272954	30,9542765997972	30,8724847418601	23663344327677,6	24618030141499,3	−0,057061343054804	0,00258112293000198	0,0046251551555263	1,04034450078576	0.0570613430548038	0,961220056668451	-0.0570613430548033	2428
Q8BX80	sp|Q8BX80|ENASE_MOUSE Cytosolic endo-beta-N-acetylglucosaminidase OS=Mus musculus GN=Engase PE=2 SV=1	1,04	0,925185154875874	0,983416151166861	24,8891462278101	23,8482598894016	23,9912209416627	23,9175054815775	24,8042210143347	24,1256842871187	33771250017,9151	35135233276,7868	−0,057122896592943	0,0232741704148211	0,019095405247114	1,0403888887189	0.0571228965929436	0,961179046453838	-0.0571228965929433	2429
Q3UI43	sp|Q3UI43|BABA1_MOUSE BRISC and BRCA1-A complex member 1 OS=Mus musculus GN=Babam1 PE=2 SV=1	1,04	0,823092358691002	0,955014370168317	27,5217488359946	26,9578668145221	27,1640538722618	27,183957488667	27,3917352883225	27,1876699138534	659373848720,386	686213218336,28	−0,057560246664889	0,0104838451958008	0,0043627134251854	1,0407043283078	0.0575602466648895	0,96088771113887	-0.0575602466648889	2430
Q9D8N2	sp|Q9D8N2|FA45A_MOUSE Protein FAM45A OS=Mus musculus GN=Fam45a PE=2 SV=2	1,04	0,826830230128082	0,955959931044103	25,8555074920393	25,8727859475999	26,4042232161596	26,1449400230198	26,0592651454691	26,0481993746539	204569215772,694	212909887064,662	−0,057653886844648	0,011977073641113	0,0020299197302370	1,0407718788991	0.0576538868446515	0,960825345375178	-0.0576538868446651	2431
Q00899	sp|Q00899|TYY1_MOUSE Transcriptional repressor protein YY1 OS=Mus musculus GN=Yy1 PE=1 SV=1	1,04	0,89291636765891 6	0,977069018898103	23,3766304858985	24,3357757397456	24,1039857732243	23,8940402301816	23,921929541055	24,1206114949404	24916531429,5081	25935030533,5332	−0,057798819971473	0,020905607997117	0,0051524340877416	1,04087644008182	0.0577988199714759	0,960728825720555	-0.0577988199714750	2432
Q9D0Q7	sp|Q9D0Q7|RM45_MOUSE 39S ribosomal protein L45, mitochondrial OS=Mus musculus GN=Mrpl45 PE=2 SV=1	1,04	0,865668370893434	0,968277164979127	24,9546627427534	25,1715068365119	25,5956685115229	25,4494598461444	25,4023621789469	24,9908094544841	91592268214,8795	95355434233,8889	−0,058089340991816	0,0129173828073812	0,0099801662847093	1,04108606645903	0.0580893409918147	0,960535379559186	-0.0580893409918140	2433
Q8VEB4	sp|Q8VEB4|PAG15_MOUSE Group XV phospholipase A2 OS=Mus musculus GN=Pla2g15 PE=1 SV=1	1,04	0,802923848223504	0,946370123886389	25,6064342261004	25,5815878578865	25,5908651221833	25,9104073439626	25,6464523299023	25,3445401043115	130281378982,312	135711877292,007	−0,058916093359952	0,000490574529678228	0,0110457956080738	1,04168284333583	0.0589160933599625	0,959985091813222	-0.0589160933599620	2434
Q9Z0N1	sp|Q9Z0N1|IF2G_MOUSE Eukaryotic translation initiation factor 2 subunit 3, X-linked OS=Mus musculus GN=Eif2s3x PE=1 SV=2	1,04	0,76664477773783	0,935430281223658	28,9889107169626	28,6424177181693	28,7281279757244	28,6662319760378	28,9794072842181	28,8367004893606	3175497631382,77	3308270241940,02	−0,059094394479055	0,0062691772564004	0,0054390041036845	1,0418115917471	0.059094394479056	0,959866455625647	-0.059094394479056	2435
P62823	sp|P62823|RAB3C_MOUSE Ras-related protein Rab-3C OS=Mus musculus GN=Rab3c PE=1 SV=1	1,04	0,821282251131855	0,9536454688891	29,5620727820686	29,9407135560488	29,8904491527376	30,0893751498851	29,6149568203859	29,8120687934487	8729639264981,37	9095494114527,06	−0,059229976123862	0,0069011869211536 8	0,0079874572354014	1,04190950375387	0.0592299761238622	0,959776253501021	-0.0592299761238620	2436
Q8VCR7	sp|Q8VCR7|ABHEB_MOUSE Alpha/beta hydrolase domain-containing protein 14B OS=Mus musculus GN=Abhd14b PE=2 SV=1	1,04	0,717325297387483	0,919756927921409	26,6234109353197	26,7885005791647	26,9842139477044	26,9172029562719	26,8403763238565	26,7618369074011	435042031452,608	453293382298,915	−0,059290306012178	0,00673980163917284	0,0028943800738399	1,04195307470721	0.0592903060121796	0,959736118904398	-0.059290306012179	2437
Q9D1K2	sp|Q9D1K2|VATF_MOUSE V-type proton ATPase subunit F OS=Mus musculus GN=Atp6v1f PE=1 SV=2	1,04	0,350201727472837	0,732843629012038	29,6772864366557	29,6861147613	29,6135756219551	29,6461657797128	29,7730411693604	29,6815604610594	7598654235793,37	7918760471458,42	−0,059530690207200	0,0013344652929685 1	0,0022043985127280	1,04212670108836	0.0595306902071995	0,959576219432472	-0.0595306902071999	2438
P05201	sp|P05201|AATC_MOUSE Aspartate aminotransferase, cytoplasmic OS=Mus musculus GN=Got1 PE=1 SV=3	1,04	0,59452939538204	0,860080030359509	35,1168009278487	34,9429327686499	34,9927921828581	35,1168009278487	35,1168009278487	34,9429327686499	1614026892197820	1682142838680620	−0,059635600474919	0,0025567354001068	0,0028632668553518	1,0422024854803	0.0596356004749171	0,959506443260058	-0.0596356004749160	2439
P47708	sp|P47708|RP3A_MOUSE Rabphilin-3A OS=Mus musculus GN=Rph3a PE=1 SV=2	1,04	0,771935301635638	0,936785701190125	31,4825297714827	31,2180758996145	31,0083247513143	31,3439939544913	31,2799527489482	31,209001882346	36792316215268,3	38345163789092,9	−0,059640129759945	0,0076074218627927	0,0021589059163179	1,04220575743965	0.0596401297599461	0,959503430931587	-0.0596401297599460	2440
Q3THK3	sp|Q3THK3|T2FA_MOUSE General transcription factor IIF subunit 1 OS=Mus musculus GN=Gtf2f1 PE=1 SV=2	1,04	0,91046911005753	0,980429909122681	22,026190417643	23,0239680619191	22,9648263864877	23,0456642223253	22,4253348908122	22,6688521928451	7017421094,38452	7315664943,54825	−0,060048064554818	0,0246905618464189	0,0137603347027314	1,04250049201157	0.0600480645548198	0,959232161195852	-0.06004806455482	2441
Q62108-3	sp|Q62108-3|DLG4_MOUSE Isoform 3 of Disks large homolog 4 OS=Mus musculus GN=Dlg4;sp|Q62108|DLG4_MOUSE Disks large homolog 4 OS=Mus musculus GN=Dlg4 PE=1 SV=1;sp|Q62108-2|DLG4_MOUSE Isoform 2 of Disks large homolog 4 OS=Mus musculus GN=Dlg4	1,04	0,732446071197803	0,924701393764781	29,8306347317633	29,6034944632424	29,4384942443743	29,7066894223823	29,7170699033562	29,5745822065934	7338883337506,25	7652961737807	−0,060457623083825	0,0066462237138610 1	0,0026777508332109	1,04279648358703	0.0604576230838247	0,958959888855951	-0.0604576230838240	2442
Q9DBC3	sp|Q9DBC3|CMTR1_MOUSE Cap-specific mRNA (nucleoside-2-O-)-methyltransferase 1 OS=Mus musculus GN=Cmtr1 PE=1 SV=1	1,04	0,860738530111333	0,966813211624682	27,4696095384182	27,1760569863579	26,9395921810098	26,9524248585844	27,5796655021705	27,1789632879833	646659834621,755	674351876046,636	−0,060494580122064	0,0097635502342872 2	0,0116615084019614	1,04282319689931	0.0604945801220651	0,958935323814587	-0.0604945801220650	2443
Q8CBY8	sp|Q8CBY8|DCTN4_MOUSE Dynactin subunit 4 OS=Mus musculus GN=Dctn4 PE=1 SV=1;sp|Q8CBY8-2|DCTN4_MOUSE Isoform 2 of Dynactin subunit 4 OS=Mus musculus GN=Dctn4	1,04	0,741248936177711	0,925497610201997	29,258547537147	29,6188712147222	29,6207138314607	29,5745822065934	29,4599545956542	29,5895854758228	6477641071083,1	6755472902688,24	−0,060588235935103	0,0070702144325675 5	0,0024003377629535	1,04289089632419	0.0605882359351038	0,958873074378763	-0.0605882359351030	2444
O08539	sp|O08539|BIN1_MOUSE Myc box-dependent-interacting protein 1 OS=Mus musculus GN=Bin1 PE=1 SV=1	1,04	0,747773496327605	0,928122477394312	31,9922601162753	31,8676070981556	31,6843541136303	32,0014621620972	31,9696296459334	31,6995393111186	67833261693049,7	70752598029998,3	−0,060790259574156	0,0048630758100261	0,0052019500883205	1,04303694476846	0.0607902595741559	0,958738810754188	-0.0607902595741560	2445

Q924K8-2	sp|Q924K8-2|MTA3_MOUSE Isoform 2 of Metastasis-associated protein MTA3 OS=Mus musculus GN=Mta3;sp|Q924K8|MTA3_MOUSE Metastasis-associated protein MTA3 OS=Mus musculus GN=Mta3 PE=1 SV=1	1,04	0,952165399822832	0,988754135503464	21,9501523629703	23,3241799096996	21,5219605614353	21,7012179112435	22,0365622706126	23,1854445652742	4674700552,53426	4876734058,11296	−0,061041347150620	0,0422883058831118	0,0348931198149719	1,04321849138961	0.0610413471506205	0,958571965751834	-0.061041347150620	2446
Q8R1I1	sp|Q8R1I1|QCR9_MOUSE Cytochrome b-c1 complex subunit 9 OS=Mus musculus GN=Uqcr10 PE=1 SV=1	1,04	0,84271828358278	0,959771286845933	26,6033375672616	26,1606183781872	26,0475252294948	26,0524841852733	26,4349457329772	26,4513611202461	256525452070,944	267645811947,208	−0,061223102934766	0,0111815663327613	0,0085776184937658	1,0433499279954	0.0612231029347703	0,958451209098473	-0,061223102934770	2447
Q9CXW2	sp|Q9CXW2|RT22_MOUSE 28S ribosomal protein S22, mitochondrial OS=Mus musculus GN=Mrps22 PE=2 SV=1	1,04	0,763560633010031	0,933454063694469	27,2537191581988	26,8091718647215	27,0804277839634	27,0832508388608	27,1953399119946	26,9928732615539	558082666178,295	582437665205,021	−0,061624817508475	0,00828406302031361	0,0037440137236635	1,04364048644174	0.0616248175084745	0,958184368076277	-0,061624817508474	2448
Q02053	sp|Q02053|UBA1_MOUSE Ubiquitin-like modifier-activating enzyme 1 OS=Mus musculus GN=Uba1 PE=1 SV=1;sp|P22314-2|UBA1_HUMAN Isoform 2 of Ubiquitin-like modifier-activating enzyme 1 OS=Homo sapiens GN=UBA1;sp|P22314|UBA1_HUMAN Ubiquitin-like modifier-activ	1,04	0,84142125800486	0,959771286845933	26,894872517499	26,4033494281023	26,1649342395901	26,5444468376233	26,5794042509612	26,4697443055486	318764597483,505	332930123214,637	−0,062727999959241	0,0140522144842127	0,0021113839221059	1,044438829917	0.0627279999592404	0,957451955400264	-0,062727999959240	2449
Q62261	sp|Q62261|SPTB2_MOUSE Spectrin beta chain, non-erythrocytic 1 OS=Mus musculus GN=Sptbn1 PE=1 SV=2;sp|Q62261-2|SPTB2_MOUSE Isoform 2 of Spectrin beta chain, non-erythrocytic 1 OS=Mus musculus GN=Sptbn1	1,04	0,829446826200317	0,956900945145962	24,2701951976947	24,3792155998351	24,0965663332202	24,4263753482029	23,9261106778015	24,524511933321	33967127090,5464	35483465100,6065	−0,063007699879712	0,00587867615805553	0,0132111986229829	1,04464133825678	0.0630076998797101	0,957266349107653	-0,063007699879710	2450
Q9JMD3	sp|Q9JMD3|PCTL_MOUSE PCTP-like protein OS=Mus musculus GN=Stard10 PE=1 SV=1	1,04	0,880293883267053	0,973716798448825	26,3789076381336	26,7270880304719	27,355199209797	26,9578668145221	26,8591980973836	26,7753723973676	444581084594,986	464462086529,31	−0,063114268057147	0,0184482443104052	0,0034003596305483	1,04471850607957	0.0631142680571476	0,957195640912513	-0,063114268057147	2451
Q64010	sp|Q64010|CRK_MOUSE Adapter molecule crk OS=Mus musculus GN=Crk PE=1 SV=1;sp|Q64010-2|CRK_MOUSE Isoform Crk-I of Adapter molecule crk OS=Mus musculus GN=Crk	1,04	0,355363906580731	0,732843629012038	30,2413049199915	30,153305359808	30,2152266398378	30,1781158654775	30,2476801384455	30,3160330662053	13095358537159	13684382796993,2	−0,063474806983186	0,00149652651288821	0,0022798577353604	1,04497962069254	0.0634748069831892	0,956956461349238	-0,063474806983189	2452
Q3TFQ1	sp|Q3TFQ1|SPRY7_MOUSE SPRY domain-containing protein 7 OS=Mus musculus GN=Spryd7 PE=2 SV=2	1,05	0,809800309169632	0,94879038498679	24,5876185868543	24,504203243946	24,0132687336335	24,4804791910647	24,4150238637808	24,34213713151	38286512668,9479	40016060732,4686	−0,063742894072685	0,0127352223940915	0,0028347836460625	1,04517382083021	0.0637428940726851	0,956778652574431	-0,063742894072685	2453
O55106	sp|O55106|STRN_MOUSE Striatin OS=Mus musculus GN=Strn PE=1 SV=2	1,05	0,782536293206106	0,940251150142017	29,8175332931728	29,531702480219	29,3037700876513	29,6166517537297	29,6912193119391	29,4785745078136	6820827765648,67	7131066067970,79	−0,064170937131206	0,00871120262839689	0,0036455396847926	1,0454839666066	0.0641709371312054	0,956494821480402	-0,064170937131205	2454
Q9R1J0	sp|Q9R1J0|NSDHL_MOUSE Sterol-4-alpha-carboxylate 3-dehydrogenase, decarboxylating OS=Mus musculus GN=Nsdhl PE=2 SV=1	1,05	0,851769049986106	0,962694152860292	26,0516722330455	25,3163007018966	25,7219669464862	25,8984378098441	25,7926120679867	25,5327595104657	144514636083,215	151109382071,181	−0,064377624561665	0,0143341293767823	0,0073099714429129	1,04563375839779	0.0643776245616684	0,956357799247306	-0,064377624561668	2455
Q922U1	sp|Q922U1|PRPF3_MOUSE U4/U6 small nuclear ribonucleoprotein Prp3 OS=Mus musculus GN=Prpf3 PE=2 SV=1	1,05	0,757955565859972	0,93285257956573	24,2905635733946	24,1475198498776	23,8591535596167	24,2523651368066	24,0114271695296	24,167659258453	29248057386,9186	30586274155,4966	−0,064543570574119	0,00911846174134515	0,0050623995352312	1,04575403934952	0.0645435705741179	0,956247800507683	-0,064543570574118	2456
P35282	sp|P35282|RAB21_MOUSE Ras-related protein Rab-21 OS=Mus musculus GN=Rab21 PE=1 SV=4	1,05	0,829975491980972	0,956900945145962	28,5307522510992	28,952674136364	29,2505827592898	28,9439646135663	28,9577992893341	28,9668367465018	3597773803707,68	3762859224767,58	−0,064724831139229	0,0125103626030494	0,0003978287525798	1,0458854364023	0.0647248311392285	0,956127664842392	-0,064724831139228	2457
Q06335-2	sp|Q06335-2|APLP2_MOUSE Isoform 2 of Amyloid-like protein 2 OS=Mus musculus GN=Aplp2;sp|Q06335|APLP2_MOUSE Amyloid-like protein 2 OS=Mus musculus GN=Aplp2 PE=1 SV=4	1,05	0,916927190087222	0,980665300581806	24,2820927735618	23,6248549139189	23,9668942617015	23,675109750947	24,7729139695752	23,5605386807846	25398280615,946	26564833621,7904	−0,064786842728784	0,0137203015984374	0,0278861526059615	1,04593039282793	0.0647868427287884	0,956086568338696	-0,064786842728788	2458
Q9JI90	sp|Q9JI90|RNF14_MOUSE E3 ubiquitin-protein ligase RNF14 OS=Mus musculus GN=Rnf14 PE=2 SV=2	1,05	0,828574700182957	0,956839193875508	28,304589714348	28,8875389662165	28,8422515451511	28,9014392327936	28,6410826370046	28,6266029003322	2849391965203,93	2980289591390,95	−0,064798428671315	0,0113077152017614	0,0053847511440489	1,04593879248117	0.064798428671314	0,95607889026451	-0,064798428671314	2459
Q99K67	sp|Q99K67|AASS_MOUSE Alpha-aminoadipic semialdehyde synthase, mitochondrial OS=Mus musculus GN=Aass PE=1 SV=1	1,05	0,785414919900077	0,941085863507826	25,6594416194182	25,1895497765879	25,3877731654233	25,5883688052889	25,270357393125	25,5141697482735	108743186688,227	113791308696,508	−0,065465358140362	0,00928294705434283	0,0065353166926288	1,04642242113756	0.0654653581403615	0,955637015989114	-0,065465358140361	2460
Q3U0B3	sp|Q3U0B3|DHR11_MOUSE Dehydrogenase/reductase SDR family member 11 OS=Mus musculus GN=Dhrs11 PE=2 SV=1	1,05	0,996390876534111	0,999929810953105	0	20,8620051295316	21,882883155524	21,0856256932374	0	21,7962886665004	1541545,58084063	1613589,00758721	−0,065895612805521	0,86676605810019	0,866382110681019	1,04673454203494	0.0658956128055181	0,955352059038685	-0,065895612805518	2461
Q6P1B1	sp|Q6P1B1|XPP1_MOUSE Xaa-Pro aminopeptidase 1 OS=Mus musculus GN=Xpnpep1 PE=2 SV=1	1,05	0,730934837772131	0,924701393764781	29,1799288907371	29,5915968002301	29,5056687085734	29,5601460600447	29,4260099986843	29,4282378281537	6017731436958,35	6299331477172,28	−0,065979006666984	0,00738006250908194	0,0026061972429905	1,04679504945742	0.0659790066669846	0,955296837254175	-0,065979006666984	2462
P56391	sp|P56391|CX6B1_MOUSE Cytochrome c oxidase subunit 6B1 OS=Mus musculus GN=Cox6b1 PE=1 SV=2	1,05	0,7971970081434	0,944411974778833	27,235518194489	27,4101106762398	27,1537780055346	26,9898009594195	27,4879183321299	27,4591263032037	694507611493,386	727065106083,367	−0,066094052530451	0,0048022056582176	0,0102388899958825	1,04687852811285	0.0660940525304509	0,955220661371902	-0,066094052530450	2463

Q60854	sp|Q60854|SPB6_MOUSE Serpin B6 OS=Mus musculus GN=Serpinb6 PE=1 SV=1	1,05	0,791024660626258	0,942055274591853	30,4300715036396	30,0036031524467	30,0564364891875	30,076831063814	30,4253950011429	30,1253373252879	12583032328531,2	13172965757779,7	−0,066100557392887	0,0077072193549337	0,0062499243926623	1,04688324831773	0,0661005573928885	0,955216354456847	-0,066100557392888	2464
P54823	sp|P54823|DDX6_MOUSE Probable ATP-dependent RNA helicase DDX6 OS=Mus musculus GN=Ddx6 PE=1 SV=1	1,05	0,833404183242438	0,957953242267243	29,3648762637523	29,9571855137959	30,0564364891875	29,8993775648934	29,8120687934487	29,8046256258292	8686860873355,64	9094497364997,46	−0,066158973300340	0,0125509654425207	0,0017657611363259	1,04692563833871	0,0661589733003408	0,955177677744928	-0,066158973300340	2465
Q3U0V1	sp|Q3U0V1|FUBP2_MOUSE Far upstream element-binding protein 2 OS=Mus musculus GN=Khsrp PE=1 SV=2;sp|Q92945|FUBP2_HUMAN Far upstream element-binding protein 2 OS=Homo sapiens GN=KHSRP PE=1 SV=4	1,05	0,74100601240246	0,925497610201997	30,9222771011418	30,8577552537318	30,4798415039778	30,8315955900058	30,8001322897362	30,766243128978	22697849183101,7	23767106708021,7	−0,066410691092117	0,00777158405624098	0,0010611834833615	1,04710831921978	0,0664106910921142	0,955011035291094	-0,066410691092114	2466
Q60625	sp|Q60625|ICAM5_MOUSE Intercellular adhesion molecule 5 OS=Mus musculus GN=Icam5 PE=1 SV=2	1,05	0,883354773076837	0,973900246537713	26,056977654842	25,1516291000811	25,5153070978226	25,9516347704544	25,3615584974045	25,549542709295	127915784522,542	133974054147,52	−0,066759330149858	0,0178137838662455	0,011765493339284	1,0473613920877	0,0667593301498554	0,954780276945959	-0,066759330149855	2467
Q9CQQ8	sp|Q9CQQ8|LSM7_MOUSE U6 snRNA-associated Sm-like protein LSm7 OS=Mus musculus GN=Lsm7 PE=3 SV=1	1,05	0,917236791816036	0,980665300581806	24,3517999664098	23,3964502955791	23,5394665768213	24,3378985272248	23,8919023724206	23,1968846809395	20890749276,916	21881233607,9094	−0,066829838198985	0,0216841897602116	0,024151397032678	1,04741258046153	0,0668298381989857	0,954733615629634	-0,066829838198985	2468
P47754	sp|P47754|CAZA2_MOUSE F-actin-capping protein subunit alpha-2 OS=Mus musculus GN=Capza2 PE=1 SV=3;sp|Q2QL88|CAZA2_MICMU F-actin-capping protein subunit alpha-2 OS=Microcebus murinus GN=CAPZA2 PE=3 SV=3	1,05	0,3574849175826	0,732843629012038	32,26957919978	32,1512238864603	32,2785243609912	32,3364067192113	32,2621420111148	32,2397875300273	99692259211743,6	104420321218013	−0,066849108443624	0,00220442960700774	0,0015669525970650	1,04742657096603	0,0668491084436222	0,954720863227398	-0,066849108443622	2469
Q5RKS2	sp|Q5RKS2|SMIM7_MOUSE Small integral membrane protein 7 OS=Mus musculus GN=Smim7 PE=3 SV=1	1,05	0,909830157636587	0,980429909122681	22,9239850676747	23,141829678946	21,9944238728401	22,3570874543215	22,7593573011881	23,0829978435626	7124078393,73328	7462434459,82912	−0,066942963685806	0,0268607162234902	0,0159970919207918	1,04749471403817	0,0669429636858053	0,954658755407872	-0,066942963685805	2470
Q61771	sp|Q61771|KIF3B_MOUSE Kinesin-like protein KIF3B OS=Mus musculus GN=Kif3b PE=1 SV=1	1,05	0,760904417680079	0,933389703428013	28,5719407915324	28,6363686210369	28,1954506384403	28,3824394113629	28,5701797962947	28,5904583519206	2309194820170,02	2418960906783,49	−0,066997559573692	0,00836570316345343	0,0040223566597492	1,04753435511578	0,0669975595736915	0,954622628953757	-0,066997559573691	2471
Q8K221	sp|Q8K221|ARFP2_MOUSE Arfaptin-2 OS=Mus musculus GN=Arfip2 PE=2 SV=2	1,05	0,763127549296197	0,933454063694469	29,3120180582318	28,8948809578225	28,934830454227	29,2505827592898	29,0218456153508	29,0088476558689	4121520055518,12	4317763504501,19	−0,067107710138097	0,00792399324582921	0,0046734781547507	1,04761433799657	0,0671077101380975	0,954549745770353	-0,067107710138097	2472
P55096	sp|P55096|ABCD3_MOUSE ATP-binding cassette sub-family D member 3 OS=Mus musculus GN=Abcd3 PE=1 SV=2	1,05	0,882377185133727	0,973716798448825	26,3107417857097	27,173042131448	27,2761384034076	27,1109039589626	26,9109358823026	26,8777286255423	491129710908,349	514531606730,824	−0,067155600887659	0,0196925464540427	0,0046774775142345	1,04764911448585	0,0671556008876595	0,95451805969479	-0,067155600887659	2473
Q9WTX8-2	sp|Q9WTX8-2|MD1L1_MOUSE Isoform 2 of Mitotic spindle assembly checkpoint protein MAD1 OS=Mus musculus GN=Mad1l1;sp|Q9WTX8|MD1L1_MOUSE Mitotic spindle assembly checkpoint protein MAD1 OS=Mus musculus GN=Mad1l1 PE=2 SV=1	1,05	0,789593441340594	0,942055274591853	24,9712234399972	24,6071514587832	24,3731216083975	24,7090851139904	24,6644462690852	24,7178927598584	50766312699,6812	53190273146,1031	−0,067290968729497	0,0122268480667838	0,0011602644901122	1,04774741984436	0,0672909687294961	0,954428501621645	-0,067290968729496	2474
P47934	sp|P47934|CACP_MOUSE Carnitine O-acetyltransferase OS=Mus musculus GN=Crat PE=1 SV=3	1,05	0,84698363235081	0,961355015563972	28,2148956435959	28,9121400873384	28,9070949594495	28,8657160830252	28,6735464306224	28,6351446174494	2849154967070,82	2985541869240,82	−0,067458708456804	0,0139865118670536	0,0043006786874310	1,04786924675783	0,0674587084568034	0,954317538274993	-0,067458708456803	2475
Q9Z1G4-3	sp|Q9Z1G4-3|VPP1_MOUSE Isoform A1-III of V-type proton ATPase 116 kDa subunit a isoform 1 OS=Mus musculus GN=Atp6v0a1;sp|Q9Z1G4-2|VPP1_MOUSE Isoform A1-I of V-type proton ATPase 116 kDa subunit a isoform 1 OS=Mus musculus GN=Atp6v0a1;sp|Q9Z1G4|VPP1_MOUS	1,05	0,715581498641841	0,918558942968132	32,7587169966684	32,4715364843887	32,3444630888764	32,5465656838934	32,6477383197789	32,5208763535181	133471024238902	139868916013156	−0,067548803100075	0,00652487769108756	0,0020591284747509	1,04793468702842	0,06754880310007	0,954257944104949	-0,067548803100070	2476
Q9CWK8	sp|Q9CWK8|SNX2_MOUSE Sorting nexin-2 OS=Mus musculus GN=Snx2 PE=1 SV=2	1,05	0,780289111855732	0,940251150142017	28,8597896366355	29,4085535576116	29,2389697441818	29,2928257170814	29,1365569873341	29,21883644974	4655657733500,99	4879544793319,1	−0,067761566219777	0,00963258650978069	0,0026756164775978	1,04808924380482	0,0677615662197767	0,954117224187664	-0,067761566219776	2477
O88597	sp|O88597|BECN1_MOUSE Beclin-1 OS=Mus musculus GN=Becn1 PE=1 SV=3	1,05	0,892642114672373	0,977069018898103	25,8966554254401	26,5628276441229	26,7629318109403	26,870933519598	26,4229344891842	26,0701199334524	294184108251,454	308399743650,606	−0,068082251361049	0,0171751765789265	0,0151711813916773	1,04832224107429	0,0680822513610478	0,953905164670768	-0,068082251361047	2478
Q99JN2	sp|Q99JN2|KLH22_MOUSE Kelch-like protein 22 OS=Mus musculus GN=Klhl22 PE=1 SV=1;sp|Q99JN2-2|KLH22_MOUSE Isoform 2 of Kelch-like protein 22 OS=Mus musculus GN=Klhl22	1,05	0,843437657980066	0,959771286845933	26,1383002868415	26,8071250785447	26,3789076381336	26,7447857389232	26,4478784720452	26,2733618497883	304350043406,483	319069681628,964	−0,068139957001328	0,0128120651555877	0,0089980018119212	1,04836417323201	0,0681399570013294	0,953867010656318	-0,068139957001329	2479
P47955	sp|P47955|RLA1_MOUSE 60S acidic ribosomal protein P1 OS=Mus musculus GN=Rplp1 PE=2 SV=1	1,05	0,843699662882998	0,959787661610508	25,1063810759423	25,6734309040907	25,6205393435449	25,2008916720812	25,6255477551962	25,7162865377133	114837483001,981	120418858345,24	−0,068467729704851	0,0122997968051335	0,0107836156632061	1,04860238310137	0,0684677297048587	0,953650321719068	-0,068467729704858	2480
Q9Z0E0	sp|Q9Z0E0|NCDN_MOUSE Neurochondrin OS=Mus musculus GN=Ncdn PE=1 SV=1	1,05	0,718718805402783	0,920149210230605	33,3243876702912	33,2966158606149	32,9774514497747	33,3807460089899	33,1377845162753	33,2226868067924	262031277820934	274802134583913	−0,068654178785674	0,00580694007611419	0,0037086796836400	1,04873790972277	0,0686541787856768	0,953527083105392	-0,068654178785676	2481

P50516	sp|P50516|VATA_MOUSE V-type proton ATPase catalytic subunit A OS=Mus musculus GN=Atp6v1a PE=1 SV=2;sp|P50516-2|VATA_MOUSE Isoform 2 of V-type proton ATPase catalytic subunit A OS=Mus musculus GN=Atp6v1a	1,05	0,764641162171714	0,933989837216741	34,6005663418216	35,016095172406	34,9023917375126	35,0731894837483	34,7308080145928	34,8580740958575	1351085066174640	1417055040816300	−0,068777284473623	0,00616379057966955	0,0049605055295318	1,04882740272486	0.0687772844736165	0,953445721766984	-0.068777284473616	2482
Q8BWM0	sp|Q8BWM0|PGES2_MOUSE Prostaglandin E synthase 2 OS=Mus musculus GN=Ptges2 PE=1 SV=3	1,05	0,589722561913027	0,858561075424286	28,6150528659003	28,4901679344148	28,7785417083211	28,7339849515457	28,6537286050758	28,6392790424527	2709867444654,01	2842383849637,54	−0,068879113726995	0,00505160321320054	0,0017792567274523	1,04890143436534	0.0688791137269918	0,95337842740683	-0.068879113726991	2483
O35295	sp|O35295|PURB_MOUSE Transcriptional activator protein Pur-beta OS=Mus musculus GN=Purb PE=1 SV=3	1,05	0,814749625053808	0,950652201935115	30,9261836153758	30,3160330662053	30,3912180432188	30,4662615796495	30,7034268677074	30,6071786088968	18420370150750	19322455226415	−0,068976371097225	0,0108922213788201	0,0038990765477911	1,04897214704604	0.0689763710972255	0,95331415883258	-0.068976371097225	2484
O35658	sp|O35658|C1QBP_MOUSE Complement component 1 Q subcomponent-binding protein, mitochondrial OS=Mus musculus GN=C1qbp PE=1 SV=1	1,05	0,701334770826624	0,913830966176594	29,9327417629286	30,0816756695027	30,333769820244	30,1999089850511	30,076831063814	30,2152266398378	12001615930894,2	12590817523562,3	−0,069143293112911	0,00673107165347034	0,0025151998883965	1,04909352174413	0.069143293112900 5	0,953203865311726	-0.069143293112900	2485
Q9WV69	sp|Q9WV69|DEMA_MOUSE Dematin OS=Mus musculus GN=Dmtn PE=1 SV=1;sp|Q9WV69-3|DEMA_MOUSE Isoform 3 of Dematin OS=Mus musculus GN=Dmtn	1,05	0,546469432569285	0,836220697939517	29,8286939050459	29,7170699033562	29,97721683577	29,8391442128287	29,9047847149208	29,9229385752534	9115465095608,93	9563317812826,93	−0,069194952561476	0,00437347633526035	0,0014748842587337	1,04913108793908	0.0691949525614769	0,953169733979006	-0.069194952561477	2486
Q03157	sp|Q03157|APLP1_MOUSE Amyloid-like protein 1 OS=Mus musculus GN=Aplp1 PE=1 SV=1	1,05	0,733474942084217	0,924701393764781	27,1601478692373	27,1601478692373	26,8636371325686	27,1009588259087	27,2900373208184	26,9378482697719	565689374963,365	593685055142,25	−0,069687722665548	0,00632602588637313	0,0065015313681331	1,04948949267555	0.0696877226655479	0,952844222814103	-0.069687722665547	2487
Q921L3	sp|Q921L3|TMCO1_MOUSE Transmembrane and coiled-coil domain-containing protein 1 OS=Mus musculus GN=Tmco1 PE=2 SV=1	1,05	0,833274152901813	0,957953242267243	24,817993141014	24,4042625278283	24,1023003631839	24,2952278431904	24,5607808475858	24,6135674323953	41192361570,6202	43232514852,9388	−0,069739922108236	0,0147003140764272	0,0069665228959628	1,04952746588275	0.0697399221082365	0,952809747726718	-0.069739922108236	2488
Q6PEE3	sp|Q6PEE3|RIR2B_MOUSE Ribonucleoside-diphosphate reductase subunit M2 B OS=Mus musculus GN=Rrm2b PE=1 SV=1	1,05	0,933705833377756	0,98467134375593	23,7897040107311	24,2494398701144	24,6135674323953	23,2126920867356	24,9114903418552	24,6738015764935	32927382314,3441	34561102048,8508	−0,069861397366403	0,0170477578781733	0,0379088538339299	1,04961584006011	0.069861397366401	0,952729524301699	-0.069861397366401	2489
P42128	sp|P42128|FOXK1_MOUSE Forkhead box protein K1 OS=Mus musculus GN=Foxk1 PE=1 SV=2	1,05	0,878175738347544	0,973716798448825	25,9353037091857	25,3737724117129	24,8730804771037	25,2894343129533	25,5400198303093	25,4982180151912	106781678521,721	112088815122,138	−0,069978192478493	0,0209262356498027	0,0052764153844962	1,04970081641241	0.0699781924785057	0,952652398059217	-0.069978192478505	2490
O54865	sp|O54865|GCYB1_MOUSE Guanylate cyclase soluble subunit beta-1 OS=Mus musculus GN=Gucy1b3 PE=2 SV=1	1,05	0,788723036701951	0,942055274591853	29,0120311248533	29,5685996112172	29,483312762562	29,5039457329552	29,3099584234601	29,3963002344319	5604818739612,18	5884844679097,27	−0,07033662129137	0,0102117878778674	0,0033053462733887	1,04996164059794	0.0703366212913697	0,952415746760534	-0.070336621291369	2491
Q99P58	sp|Q99P58|RB27B_MOUSE Ras-related protein Rab-27B OS=Mus musculus GN=Rab27b PE=1 SV=3	1,05	0,812777879593187	0,950502380087197	26,2361110611353	26,6952330944468	26,8845106514255	26,7900481326491	26,5398052352229	26,6322995250514	358532423734,049	376449958991,518	−0,070354507680777	0,0125323566452844	0,0047471771262431	1,04997465799846	0.0703545076807761	0,952403938878174	-0.070354507680776	2492
P49312	sp|P49312|ROA1_MOUSE Heterogeneous nuclear ribonucleoprotein A1 OS=Mus musculus GN=Hnrnpa1 PE=1 SV=2;sp|P09867|ROA1_BOVIN Heterogeneous nuclear ribonucleoprotein A1 OS=Bos taurus GN=HNRNPA1 PE=1 SV=2;sp|P49312-2|ROA1_MOUSE Isoform Short of Heterogeneous	1,05	0,721342148240887	0,920750207284069	30,3063347984404	29,9631043655379	30,3763944677086	30,1695834981336	30,2888262852398	30,333769820244	13253434962670,6	13915992967610,3	−0,070377535986117	0,00732014587412707	0,0028036158799131	1,04999141783287	0.070377535986111	0,952388736723149	-0.070377535986111	2493
Q62178	sp|Q62178|SEM4A_MOUSE Semaphorin-4A OS=Mus musculus GN=Sema4a PE=1 SV=2	1,05	0,535087507260053	0,8300431463043	25,0419212187464	25,2247645740049	25,1667818406085	25,3070616187359	25,1593816694031	25,114611226522	83198005415,9235	87393333261,0009	−0,070974287251163	0,00371603953330219	0,0039972953058286	1,05042582239928	0.0709742872511629	0,951994875483831	-0.070974287251162	2494
Q80T41	sp|Q80T41|GABR2_MOUSE Gamma-aminobutyric acid type B receptor subunit 2 OS=Mus musculus GN=Gabbr2 PE=1 SV=2	1,05	0,666314205542284	0,89771450844655	24,7320198340803	24,4879210503984	24,3929941603799	24,6585297673407	24,620547219318	24,4818124226021	45348594237,3338	47641169813,9314	−0,071151009266958	0,00712770062914087	0,0037834047254404	1,05055450152654	0.0711510092669594	0,951878268616167	-0.071151009266959	2495
Q9D2I5-7	sp|Q9D2I5-7|ARMC9_MOUSE Isoform 7 of LisH domain-containing protein ARMC9 OS=Mus musculus GN=Armc9;sp|Q9D2I5-2|ARMC9_MOUSE Isoform 2 of LisH domain-containing protein ARMC9 OS=Mus musculus GN=Armc9;sp|Q9D2I5-4|ARMC9_MOUSE Isoform 4 of LisH domain-contai	1,05	0,846310557969911	0,961071709438436	22,7708085059644	21,9143063520067	22,1779631888277	22,3884232655303	22,3211019379397	22,3016141097192	4779934508,79139	5021760657,42432	−0,071202418256432	0,0196820626154773	0,0020391272941022	1,05059193764855	0.071202418256431	0,951844349993979	-0.071202418256431	2496
Q99LC5	sp|Q99LC5|ETFA_MOUSE Electron transfer flavoprotein subunit alpha, mitochondrial OS=Mus musculus GN=Etfa PE=1 SV=2	1,05	0,7282845833527	0,923891482347095	31,4973921613793	31,6098246071065	31,9210716119724	31,849738476019	31,6995393111186	31,6276420584925	57115470155763,6	60016458480497,5	−0,071476625907851	0,00692877874388132	0,0035720432990021	1,05079163870703	0.0714766259078495	0,951663453689514	-0.071476625907849	2497
Q63739	sp|Q63739|TP4A1_MOUSE Protein tyrosine phosphatase type IVA 1 OS=Mus musculus GN=Ptp4a1 PE=1 SV=1	1,05	0,831578911608697	0,957953242267243	25,2169254586012	24,8100392117994	24,6683213514674	24,6180184843432	25,1249989581468	25,1009475178655	65050431416,3533	68355531105,9322	−0,071499455746865	0,011437774706614	0,0114644589205857	1,05080826702631	0.0714994557468659	0,951648394268828	-0.071499455746865	2498
Q9JIF7	sp|Q9JIF7|COPB_MOUSE Coatomer subunit beta OS=Mus musculus GN=Copb1 PE=1 SV=1	1,05	0,708251085532129	0,916805961322165	30,7635181652725	30,413494702835	30,3453333642794	30,5173788922969	30,6103162203833	30,5433566106407	17750747508692,3	18652797319766,2	−0,071512224774378	0,00735446993348669	0,0015692296896367	1,05081756757749	0.0715122247743853	0,951639971441817	-0.071512224774385	2499
Q99LC8	sp|Q99LC8|EI2BA_MOUSE Translation initiation factor eIF-2B subunit alpha OS=Mus musculus GN=Eif2b1 PE=2 SV=1	1,05	0,656907799446033	0,892226633215307	27,6401569056551	27,6721550228499	27,8808173603113	27,854757020185	27,8721796264246	27,615484436771	1105195210379,06	1161585534425,75	−0,071794177221124	0,00471281609181204	0,0051632170184404	1,05102295369824	0.0717941772211322	0,95145400629100 7	-0.071794177221132	2500

P05132-2	sp|P05132-2|KAPCA_MOUSE Isoform 2 of cAMP-dependent protein kinase catalytic subunit alpha OS=Mus musculus GN=Prkaca;sp|P05132|KAPCA_MOUSE cAMP-dependent protein kinase catalytic subunit alpha OS=Mus musculus GN=Prkaca PE=1 SV=3	1,05	1,05	0,52032855751727	0,823916573667872	28,9439646135663	28,8699406290185	28,7725746006029	28,7942855809832	28,9559879547382	28,9855437086712	3425127960686,57	3599942832718,77	−0,071816109379157	0,00297827730606583	0,0035610550594883	1,05103893169502	0.0718161093791542	0,951439542194014	-0.071816109379154	2501
P35276	sp|P35276|RAB3D_MOUSE Ras-related protein Rab-3D OS=Mus musculus GN=Rab3d PE=1 SV=1	1,05	1,05	0,819204607959063	0,952523073888973	30,9997610762329	30,9997610762329	31,5035229610434	31,3626131085233	30,9382317000035	31,3519919643443	34352011935195,8	36110769382510,3	−0,072034561376003	0,00933168730867838	0,0077523204545158	1,05119809141404	0.0720345613760072	0,95129548670967	-0.072034561376007	2502
Q8BZ98	sp|Q8BZ98|DYN3_MOUSE Dynamin-3 OS=Mus musculus GN=Dnm3 PE=1 SV=1;sp|Q8BZ98-2|DYN3_MOUSE Isoform 2 of Dynamin-3 OS=Mus musculus GN=Dnm3	1,05	1,05	0,741248019233883	0,925497610201997	32,043087513796	31,8280562746036	31,6172183495446	31,7920975405357	32,0567659011701	31,7896349241163	66581910733998	69998816820730,8	−0,072200263805759	0,00668996780698289	0,004815702438085	1,0513188349367	0.0722002638057607	0,951186230826107	-0.072200263805760	2503
Q91ZH7-2	sp|Q91ZH7-2|ABHD3_MOUSE Isoform 2 of Abhydrolase domain-containing protein 3 OS=Mus musculus GN=Abhd3;sp|Q91ZH7|ABHD3_MOUSE Abhydrolase domain-containing protein 3 OS=Mus musculus GN=Abhd3 PE=2 SV=1	1,05	1,05	0,753104777035662	0,929655312201488	26,9234814664797	26,4531431572982	26,7585971508416	26,642274809204	26,9150426236507	26,7284847671728	398805868985,677	419334206024,426	−0,072413877663708	0,00893406670476027	0,005209876344406	1,0514745108715	0.0724138776637077	0,951045403060791	-0.072413877663707	2504
Q6KAR6	sp|Q6KAR6|EXOC3_MOUSE Exocyst complex component 3 OS=Mus musculus GN=Exoc3 PE=1 SV=2	1,05	1,05	0,797309250353048	0,944411974778833	28,8067679360488	28,6537286050758	28,1512065915307	28,5963004492755	28,586024909645	28,5819748597617	2474932843277,46	2604078726930,41	−0,073383686421698	0,0120178189020661	0,000258317471509	1,05218157090757	0.0733836864217006	0,950406305954819	-0.073383686421700	2505
Q6NS52	sp|Q6NS52|DGKB_MOUSE Diacylglycerol kinase beta OS=Mus musculus GN=Dgkb PE=2 SV=2	1,05	1,05	0,811510754863727	0,949948723398244	28,0407745775257	27,7411133248696	27,3567816774972	27,7421650657753	27,913295506351	27,6361586212546	1085313346630,21	1142080899059,55	−0,073553216295216	0,0123721767637756	0,005036685985449	1,05230521913842	0.0735532162952148	0,950294631075535	-0.073553216295214	2506
Q9DB43	sp|Q9DB43|ZFPL1_MOUSE Zinc finger protein-like 1 OS=Mus musculus GN=Zfpl1 PE=1 SV=1;sp|O95159|ZFPL1_HUMAN Zinc finger protein-like 1 OS=Homo sapiens GN=ZFPL1 PE=1 SV=2	1,05	1,05	0,86913353068911	0,970240308853386	22,0488781458796	22,4202327163321	22,9454843610105	22,5849545096597	22,7780591977932	22,2047387177875	5744632290,5589	6045524854,11864	−0,07365304527604	0,0200475440242611	0,0129516538766792	1,05237803715553	0.0736530452760397	0,950228876595429	-0.073653045276039	2507
Q8BG81	sp|Q8BG81|PDIP3_MOUSE Polymerase delta-interacting protein 3 OS=Mus musculus GN=Poldip3 PE=1 SV=1	1,05	1,05	0,836810245142641	0,958919894648434	25,9504924356993	25,1612997878741	25,7861812046239	25,6777150180066	25,7918978973668	25,5815878578865	135556966975,699	142660510337,151	−0,073686776950131	0,0162446378727142	0,004099252861887	1,05240264310963	0.0736867769501277	0,950206659539741	-0.073686776950127	2508
Q8VCS3	sp|Q8VCS3|XYLK_MOUSE Glycosaminoglycan xylosylkinase OS=Mus musculus GN=Fam20b PE=2 SV=1	1,05	1,05	0,819098736202927	0,952523073888973	23,0354461367347	22,5521406244179	22,7369952893588	22,4762920876387	22,9959305482374	23,0064630885348	7780299600,86462	8190400009,30453	−0,074108202039151	0,0107077527901957	0,0132785685567676	1,05271010494176	0.0741082020391504	0,949929135576526	-0.074108202039150	2509
Q9DBF1-2	sp|Q9DBF1-2|AL7A1_MOUSE Isoform 2 of Alpha-aminoadipic semialdehyde dehydrogenase OS=Mus musculus GN=Aldh7a1;sp|Q9DBF1|AL7A1_MOUSE Alpha-aminoadipic semialdehyde dehydrogenase OS=Mus musculus GN=Aldh7a1 PE=1 SV=4	1,05	1,05	0,39749383480966	0,754338506152154	31,3724626453784	31,4753765257957	31,3267661620555	31,3724626453784	31,5209983110802	31,4372046772841	42970506935595,2	45264999474547,3	−0,075049140543345	0,00242482087369278	0,002368408669912	1,05339691575878	0.0750491405433478	0,949309785362037	-0.075049140543347	2510
Q8K1B8	sp|Q8K1B8|URP2_MOUSE Fermitin family homolog 3 OS=Mus musculus GN=Fermt3 PE=1 SV=1	1,05	1,05	0,737816996368952	0,924869192631503	24,7814467480862	24,2636612879042	24,4331596823421	24,605749863721	24,524511933321	24,504203243946	43357949642,2309	45675216551,1163	−0,075115034265066	0,0107781735608004	0,002189200683797	1,05344502975824	0.075115034265067	0,949266427532049	-0.075115034265066	2511
Q6NS82-3	sp|Q6NS82-3|F134A_MOUSE Isoform 3 of Protein FAM134A OS=Mus musculus GN=Fam134a;sp|Q6NS82|F134A_MOUSE Protein FAM134A OS=Mus musculus GN=Fam134a PE=1 SV=2;sp|Q6NS82-2|F134A_MOUSE Isoform 2 of Protein FAM134A OS=Mus musculus GN=Fam134a	1,05	1,05	0,662113085731538	0,895509921375283	26,6783050470106	27,0119621691426	27,0501953007888	26,9225098977632	26,9807462280019	26,9947363166119	487954258413,592	514261372925,404	−0,075755880738919	0,00760098980404455	0,001420509560270	1,05391307496186	0.0757558807389202	0,948844856143555	-0.075755880738920	2512
P09103	sp|P09103|PDIA1_MOUSE Protein disulfide-isomerase OS=Mus musculus GN=P4hb PE=1 SV=2	1,05	1,05	0,710853807192599	0,916805961322165	31,1939924779762	31,486087748298	31,6049790076691	31,625103247153	31,3949762767942	31,42290936091	44582078996421,7	46991896832351,9	−0,075948108060937	0,00672923121526718	0,003989052773859	1,05405350962039	0.0759481080609397	0,948718438744291	-0.075948108060939	2513
Q9WV80	sp|Q9WV80|SNX1_MOUSE Sorting nexin-1 OS=Mus musculus GN=Snx1 PE=1 SV=1;sp|Q13596|SNX1_HUMAN Sorting nexin-1 OS=Homo sapiens GN=SNX1 PE=1 SV=3;sp|Q13596-2|SNX1_HUMAN Isoform 1A of Sorting nexin-1 OS=Homo sapiens GN=SNX1;sp|Q13596-3|SNX1_HUMAN Isoform 3	1,05	1,05	0,83057992390148	0,957339328234845	29,1940485808107	29,97721683577	29,8416907060905	29,8014346095597	29,6401923534164	29,7293035998322	7690334328307,53	8106144910648,02	−0,075969647124361	0,0141067462218378	0,002717365542900	1,05406924648385	0.0759696471243613	0,948704274729373	-0.075969647124361	2514

Q3TWW8	sp|Q3TWW8|SRSF6_MOUSE Serine/arginine-rich splicing factor 6 OS=Mus musculus GN=Srsf6 PE=2 SV=1	1,05	0,936853860745044	0,98548775757167	23,1968846809395	23,7163979450698	22,2911985568517	22,0932341720107	23,5068458156028	23,7625704964307	10432172673,5232	10996946658,1493	−0,076063355479341	0,0312667666776174	0,0388872906828579	1,05413771438614	0.0760633554793376	0,948642654894795	-0.076063355479337	2515
Q3TBL6	sp|Q3TBL6|TP8L3_MOUSE Tumor necrosis factor alpha-induced protein 8-like protein 3 OS=Mus musculus GN=Tnfaip8l3 PE=2 SV=1	1,05	0,741158276210695	0,925497610201997	25,6104311364534	25,526381389398	25,1396857445748	25,3401904290326	25,530459038085	25,5653482547121	110193023437,929	116210136093,072	−0,076703022521421	0,00987450038813879	0,0047563490278029	1,05460520518826	0.0767030225214121	0,948222135715219	-0.076703022521412	2516
Q6NZB0	sp|Q6NZB0|DNJC8_MOUSE DnaJ homolog subfamily C member 8 OS=Mus musculus GN=Dnajc8 PE=2 SV=2	1,05	0,758719098779048	0,933136198610489	27,4718930142898	27,8808173603113	27,9902351106764	27,966093558593	27,8165098051471	27,7199698313342	1161788598058,98	1225280685899	−0,076764701770765	0,00983450984160536	0,0044553425699353	1,05465029347516	0.076764701770763	0,948181597432563	-0.076764701770763	2517
Q9CR88	sp|Q9CR88|RT14_MOUSE 28S ribosomal protein S14, mitochondrial OS=Mus musculus GN=Mrps14 PE=1 SV=1	1,05	0,86432796087496	0,967731774645151	23,3205769736667	22,78470180127	22,9386828286442	23,6030522836631	22,8346140135788	22,7662261675712	9888653917,02489	10430125671,6044	−0,07691048679499	0,0119878914105737	0,0201430767208135	1,05475687177678	0.0769104867949901	0,9480857880693	-0.07691048679499	2518
Q9WTU6-2	sp|Q9WTU6-2|MK09_MOUSE Isoform Alpha-1 of Mitogen-activated protein kinase 9 OS=Mus musculus GN=Mapk9;sp|Q9WTU6|MK09_MOUSE Mitogen-activated protein kinase 9 OS=Mus musculus GN=Mapk9 PE=1 SV=2;sp|Q9WTU6-3|MK09_MOUSE Isoform Beta-1 of Mitogen-activated p	1,05	0,469818505220082	0,796715861328205	27,5000243142379	27,4032091695388	27,6075461660519	27,4684064394126	27,6075461660519	27,5950102812602	880356887877,132	928640595914,116	−0,077031853834537	0,00371643213937276	0,0027930918685458	1,0548456071644	0.0770318538345364	0,948006033497324	-0.077031853834536	2519
Q91WK2	sp|Q91WK2|EIF3H_MOUSE Eukaryotic translation initiation factor 3 subunit H OS=Mus musculus GN=Eif3h PE=1 SV=1	1,05	0,7159950951473	0,91883000759785	29,0196626152411	28,5471743747981	28,7146432611685	28,7643891096856	28,9121400873384	28,7651471605025	3094023356662,65	3263730946987,28	−0,077038042718614	0,00832940670625681	0,0029529533393484	1,05485013225876	0.0770380427186155	0,948001966742606	-0.077038042718615	2520
Q91ZI0	sp|Q91ZI0|CELR3_MOUSE Cadherin EGF LAG seven-pass G-type receptor 3 OS=Mus musculus GN=Celsr3 PE=2 SV=2	1,05	0,953755948054374	0,989678056389876	24,1996408868827	24,4753092267965	22,47968402626	24,4227555609276	24,4774642448869	22,4148399246451	19984271577,4127	21082024118,6753	−0,077148401291881	0,0455943356825114	0,0494446203541379	1,05493082582521	0.0771484012918763	0,947929452357937	-0.077148401291876	2521
P35505	sp|P35505|FAAA_MOUSE Fumarylacetoacetase OS=Mus musculus GN=Fah PE=1 SV=2	1,06	0,76209346572025	0,933389703428013	28,0297486856985	27,8165098051471	27,6583684625279	27,8317214951593	28,155149069996	27,6784770577248	1226119783209,27	1293598787143,67	−0,077290304288417	0,00669555894360834	0,0087257135349254	1,05503459356783	0.0772903042884142	0,947836218922718	-0.077290304288414	2522
Q9WTR5	sp|Q9WTR5|CAD13_MOUSE Cadherin-13 OS=Mus musculus GN=Cdh13 PE=1 SV=2	1,06	0,722982884316927	0,921631098094813	28,3160857868948	28,0108060548788	27,8099832106879	28,0415499426754	28,0865111619099	28,1703438982579	1513771019584,1	1597511679497,79	−0,077679486123652	0,00908667813060353	0,0023262832859627	1,05531923839888	0.0776794861236492	0,947580564831917	-0.077679486123649	2523
Q3TIX9	sp|Q3TIX9|SNUT2_MOUSE U4/U6.U5 tri-snRNP-associated protein 2 OS=Mus musculus GN=Usp39 PE=2 SV=2	1,06	0,73692451490612	0,924701393764781	25,4860362621515	25,9391068700574	25,8813493021154	25,9556095100875	25,7001815061086	25,8123716078073	155332033049,264	163932545899,07	−0,077746926969916	0,00956985750304842	0,0049579635711295	1,05536857196145	0.0777469269699236	0,947536269856376	-0.077746926969923	2524
Q80UK0	sp|Q80UK0|SESD1_MOUSE SEC14 domain and spectrin repeat-containing protein 1 OS=Mus musculus GN=Sestd1 PE=2 SV=1	1,06	0,570947488465671	0,84806207361736	27,0927002045124	26,9456779435363	26,8060735616088	27,0534472647175	27,04657919394	26,9068501601645	505164810237,981	533269203732,064	−0,078109870322857	0,00531870704967096	0,0030636858974245	1,05563410776939	0.0781098703228583	0,947297925142882	-0.078109870322858	2525
O35449	sp|O35449|PRRT1_MOUSE Proline-rich transmembrane protein 1 OS=Mus musculus GN=Prrt1 PE=1 SV=1	1,06	0,709344634443355	0,916805961322165	27,0296671524623	26,5608458583866	26,9340296968593	26,8832357761076	26,8535256285277	26,9509270425352	454068650405,266	479445540421,717	−0,078456516421504	0,00922917445700563	0,0018560326687774	1,05588778259367	0.0784565164215042	0,947070338804008	-0.078456516421504	2526
Q8K3G9	sp|Q8K3G9|DP13B_MOUSE DCC-interacting protein 13-beta OS=Mus musculus GN=Appl2 PE=2 SV=1	1,06	0,538830814994554	0,831485169227261	27,1124388235916	27,1079700259264	26,9881320866984	26,9663839376261	27,2041523196231	27,2015892625047	570348631723,544	602312252666,472	−0,078667555811745	0,00260491962075963	0,0050339755187637	1,05604225059037	0.0786675558117458	0,946931810200734	-0.078667555811745	2527
Q9R0Q6	sp|Q9R0Q6|ARC1A_MOUSE Actin-related protein 2/3 complex subunit 1A OS=Mus musculus GN=Arpc1a PE=1 SV=1	1,06	0,732584890525311	0,924701393764781	32,1406176218661	31,6172183495446	31,7211883042871	31,8925920404403	31,8752640211253	31,8752640211253	66374988699122,7	70106738167199	−0,078913402326513	0,00870635074173762	0,0003138020782064	1,05622222378059	0.0789134023265146	0,946770459364742	-0.078913402326514	2528
Q8BFQ4	sp|Q8BFQ4|WDR82_MOUSE WD repeat-containing protein 82 OS=Mus musculus GN=Wdr82 PE=1 SV=1	1,06	0,790088338478118	0,942055274591853	27,4032091695388	27,9743749343137	28,0563316653839	27,8869880507204	27,8449372025876	27,8663186708468	1197557589376,04	1264984908568,62	−0,079025138059748	0,0127929744519466	0,0007545527727557	1,0563040306293	0.0790251380597453	0,946697135486876	-0.079025138059745	2529
Q5SSZ5-2	sp|Q5SSZ5-2|TENS3_MOUSE Isoform 2 of Tensin-3 OS=Mus musculus GN=Tns3;sp|Q5SSZ5|TENS3_MOUSE Tensin-3 OS=Mus musculus GN=Tns3 PE=1 SV=1	1,06	0,847557313265136	0,961525279907636	24,8684973230597	25,6104311364534	25,2310892455359	25,5400198303093	25,4119626398757	24,9224630227272	91232092935,6227	96372028047,1485	−0,079073051378204	0,014700765988845	0,0128869752606643	1,05633911210557	0.079073051378205	0,94666569526781	-0.079073051378205	2530
Q9CXW3	sp|Q9CXW3|CYBP_MOUSE Calcyclin-binding protein OS=Mus musculus GN=Cacybp PE=1 SV=1	1,06	0,659974552071805	0,89341619202324	30,2236511949269	30,4300715036396	30,620839005307	30,5614058829116	30,3920639022539	30,4856864089267	16343541195176,2	17265281155797,5	−0,079153218265405	0,00652904708805134	0,0027831152874711	1,05639781180919	0.0791532182654106	0,946613092928881	-0.079153218265410	2531
Q99KI0	sp|Q99KI0|ACON_MOUSE Aconitate hydratase, mitochondrial OS=Mus musculus GN=Aco2 PE=1 SV=1	1,06	0,707359707831689	0,916805961322165	35,4378163435131	35,2475710661237	34,9429327686499	35,2909566904371	35,2909566904371	35,211446325403	1955534709073570	2066129066674560	−0,079367236194194	0,00709001821113806	0,0013017451411869	1,05655453574301	0.0793672361941923	0,946472677150323	-0.079367236194192	2532
Q61655	sp|Q61655|DD19A_MOUSE ATP-dependent RNA helicase DDX19A OS=Mus musculus GN=Ddx19a PE=2 SV=2	1,06	0,783031725036542	0,940251150142017	28,1842803916153	28,7651471605025	28,5833286268197	28,7408088025449	28,5750404298675	28,3824394113629	2410652867959,04	2547404757247,74	−0,079604288709211	0,0104214861644745	0,0062784931827195	1,05672815489377	0.0796042887092101	0,946317172840468	-0.079604288709210	2533
Q8VEH8	sp|Q8VEH8|ERLEC_MOUSE Endoplasmic reticulum lectin 1 OS=Mus musculus GN=Erlec1 PE=2 SV=1	1,06	0,796407870589163	0,944339896000096	25,2215409768175	24,8641690818116	24,8499747977413	24,6947800953991	25,286634641881	25,1199552746439	70477660118,0515	74479518418,3042	−0,079677717422028	0,00842906609630106	0,0121911457325974	1,05678194045531	0.0796777174220285	0,946269009450668	-0.079677717422028	2534

O08582	sp|O08582|GTPB1_MOUSE GTP-binding protein 1 OS=Mus musculus GN=Gtpbp1 PE=1 SV=2;sp|O00178|GTPB1_HUMAN GTP-binding protein 1 OS=Homo sapiens GN=GTPBP1 PE=1 SV=3	1,06	0,565912076569263	0,845163895632417	27,7647974432772	27,9380967665236	27,6317151295644	27,8950448565735	27,840803271956	27,7647974432772	1158564799385,08	1224493645592,45	−0,079846549717082	0,00553059929332115	0,0023506190880189	1,05690561826353	0.0798465497170906	0,946158278203664	-0.079846549717090	2535
Q61656	sp|Q61656|DDX5_MOUSE Probable ATP-dependent RNA helicase DDX5 OS=Mus musculus GN=Ddx5 PE=1 SV=2	1,06	0,748956972464333	0,928854093158237	30,5115424211656	31,039393479464	30,9878465239167	30,9837659274804	30,8605365941129	30,8605365941129	24909261752758,5	26326918231009,2	−0,079856388280875	0,00943447131777532	0,0023023551781252	1,05691282593286	0.0798563882808733	0,946151825830457	-0.079856388280873	2536
O70161	sp|O70161|PI51C_MOUSE Phosphatidylinositol 4-phosphate 5-kinase type-1 gamma OS=Mus musculus GN=Pip5k1c PE=1 SV=2;sp|O70161-3|PI51C_MOUSE Isoform 3 of Phosphatidylinositol 4-phosphate 5-kinase type-1 gamma OS=Mus musculus GN=Pip5k1c	1,06	0,740955749614603	0,925497610201997	31,6424407003006	31,42290936091	31,0706333494719	31,4546926223212	31,4485046525447	31,3988833526896	42420851455534,6	44835747653140,3	−0,07987587702938	0,00919288084653457	0,000973214286120	1,05692710341133	0.079875877029381	0,946139044757592	-0.079875877029381	2537
P63038	sp|P63038|CH60_MOUSE 60 kDa heat shock protein, mitochondrial OS=Mus musculus GN=Hspd1 PE=1 SV=1	1,06	0,531459916384205	0,829883431939119	34,2285718437873	34,4527289384383	34,5060432263395	34,4663738267566	34,486208526548	34,4014332388837	866758351778610	916275803429337	−0,080152089050068	0,00428078186616882	0,001287105333240	1,05712947737869	0.0801520890500688	0,945957918494193	-0.080152089050068	2538
Q6PD03	sp|Q6PD03|2A5A_MOUSE Serine/threonine-protein phosphatase 2A 56 kDa regulatory subunit alpha isoform OS=Mus musculus GN=Ppp2r5a PE=2 SV=1	1,06	0,586700112739061	0,856555745166483	28,3637482883521	28,1645775605123	28,4643306249264	28,4754028990078	28,3908102287772	28,2936711973808	2013479611453,93	2128903359535,68	−0,080419597292438	0,0053847500129353	0,003203550733192	1,05732551123197	0.0804195972924389	0,945782532793351	-0.080419597292438	2539
Q9CQF9	sp|Q9CQF9|PCYOX_MOUSE Prenylcysteine oxidase OS=Mus musculus GN=Pcyox1 PE=1 SV=1	1,06	0,797234978801598	0,944411974778833	29,4859248540224	29,9308608554239	30,0171565154699	30,1213977470374	29,8475138120678	29,6323404854074	8848898243795,34	9356421499044,6	−0,080459015674625	0,00956279683548709	0,0082068698286100	1,05735440065718	0.0804590156746254	0,945756691775688	-0.080459015674625	2540
Q8R1F6-2	sp|Q8R1F6-2|HID1_MOUSE Isoform 2 of Protein HID1 OS=Mus musculus GN=Hid1;sp|Q8R1F6|HID1_MOUSE Protein HID1 OS=Mus musculus GN=Hid1 PE=1 SV=1	1,06	0,762032969514508	0,933389703428013	28,3824394113629	28,1954506384403	27,7863646768548	28,1709716265096	28,2810559679654	28,0805683689805	1632964584373,99	1727216014105,22	−0,08095502250157	0,0108406868447199	0,003563288357119	1,05771798766068	0.0809550225015768	0,945431591091369	-0.080955022501576	2541
Q9D753	sp|Q9D753|EXOS8_MOUSE Exosome complex component RRP43 OS=Mus musculus GN=Exosc8 PE=2 SV=1	1,06	0,829773113265869	0,956900945145962	23,8911102657415	23,9823398794781	23,8407893507363	24,34213713151	23,4691216538316	24,0715250354999	24082385031,7668	25474096270,6702	−0,08105268722744	0,00300165211489883	0,018650879271937	1,05778959339233	0.0810526872274471	0,945367591292895	-0.081052687227447	2542
Q62083	sp|Q62083|PICK1_MOUSE PRKCA-binding protein OS=Mus musculus GN=Pick1 PE=1 SV=2	1,06	0,761607324341457	0,933389703428013	27,158095079911	26,9947363166119	26,9547500236637	26,7618369074011	27,1591850873045	27,355199209797	551473968576,57	583361987159,413	−0,081098593509729	0,00398469859735515	0,0111590000441229	1,05782325259187	0.0810985935097298	0,94533751035422	-0.081098593509729	2543
Q9CZ42-3	sp|Q9CZ42-3|NNRD_MOUSE Isoform 3 of ATP-dependent (S)-NAD(P)H-hydrate dehydratase OS=Mus musculus GN=Carkd;sp|Q9CZ42|NNRD_MOUSE ATP-dependent (S)-NAD(P)H-hydrate dehydratase OS=Mus musculus GN=Carkd PE=1 SV=1;sp|Q9CZ42-2|NNRD_MOUSE Isoform 2 of ATP-depe	1,06	0,79109758609662	0,942055274591853	29,339015370216	29,7503945006217	30,0218464917338	29,9047847149208	29,6508210992447	29,7243921412505	7946495428966,88	8406272913294,31	−0,081147553062827	0,0115741865960192	0,004390874618214	1,05785915167728	0.0811475530628277	0,945305429758258	-0.081147553062827	2544
Q9ET22	sp|Q9ET22|DPP2_MOUSE Dipeptidyl peptidase 2 OS=Mus musculus GN=Dpp7 PE=2 SV=2	1,06	0,763150668993216	0,933454063694469	26,0052225567599	26,5328938470056	26,4907634434962	26,4349457329772	26,5143677630458	26,2484016717649	275805004927,784	291771964102,112	−0,081192626549996	0,0111318828141534	0,005171783157184	1,05789220242217	0.0811926265499971	0,945275896457481	-0.081192626549997	2545
P70168	sp|P70168|IMB1_MOUSE Importin subunit beta-1 OS=Mus musculus GN=Kpnb1 PE=1 SV=2	1,06	0,83452436370013	0,957953242267243	32,3737547971211	32,033953090713	31,5209983110802	32,033953090713	32,2253332409013	31,8397012538833	77108558817133,3	81611872352604,8	−0,081888037326468	0,0134255377039777	0,006019348810897	1,05840225267537	0.0818880373264686	0,944820362458848	-0.081888037326468	2546
Q99NB8	sp|Q99NB8|UBQL4_MOUSE Ubiquilin-4 OS=Mus musculus GN=Ubqln4 PE=1 SV=1	1,06	0,818326023610215	0,952405595170966	25,7861812046239	26,3183357187085	26,5380610554905	26,4376778659567	26,3107417857097	26,0654090296045	242481840408,764	256726262335,21	−0,08235417972350	0,0147480125638333	0,007203544953871	1,05874428329327	0.0823541797235044	0,944515135316203	-0.082354179723504	2547
P60229	sp|P60229|EIF3E_MOUSE Eukaryotic translation initiation factor 3 subunit E OS=Mus musculus GN=Eif3e PE=1 SV=1;sp|P60228|EIF3E_HUMAN Eukaryotic translation initiation factor 3 subunit E OS=Homo sapiens GN=EIF3E PE=1 SV=1	1,06	0,656269468348162	0,892226633215307	29,7200994190062	29,3158045711717	29,4085535576116	29,6149568203859	29,483312762562	29,5177234927931	6362775829630,12	6737192146516,1	−0,082491151837363	0,00718366373040042	0,002311365894544	1,05884480718972	0.0824911518373636	0,944425465573281	-0.082491151837363	2548
Q80WG5	sp|Q80WG5|LRC8A_MOUSE Volume-regulated anion channel subunit LRRC8A OS=Mus musculus GN=Lrrc8a PE=2 SV=1	1,06	0,776517462388262	0,938645209528714	25,5077187593753	25,1630860770961	24,8473603223002	25,1249989581468	25,3532998395674	25,2118538008189	85580365569,0532	90629976904,2714	−0,082708475479734	0,0131207365848752	0,004567296562790	1,05900432069484	0.0827084754797346	0,944283210614167	-0.082708475479734	2549
Q9D5V6	sp|Q9D5V6|SYAP1_MOUSE Synapse-associated protein 1 OS=Mus musculus GN=Syap1 PE=1 SV=1	1,06	0,803486086035226	0,946624509130401	26,238233207829	26,8676217628967	26,7334809843027	26,8991884585141	26,6568132883569	26,4553495397667	361349685746,366	382674436302,935	−0,082721888623126	0,0124566490596598	0,008332573647576	1,05901416660298	0.0827218886231267	0,944274431387186	-0.082721888623126	2550
Q8VDC0	sp|Q8VDC0|SYLM_MOUSE Probable leucine--tRNA ligase, mitochondrial OS=Mus musculus GN=Lars2 PE=1 SV=1	1,06	0,80258215321338	0,946213089204034	27,4198860408206	27,1783471400919	26,6630776319574	27,2176411250769	27,1953399119946	27,0220262352562	580469795168,194	615070310422,967	−0,083530340226581	0,0142712436525525	0,003944838366800	1,05960777897969	0.0835303402265813	0,943745430939466	-0.083530340226581	2551

Q3UV70	sp|Q3UV70|PDP1_MOUSE [Pyruvate dehydrogenase [acetyl-transferring]]-phosphatase 1, mitochondrial OS=Mus musculus GN=Pdp1 PE=2 SV=1	1,06	0,723393147510958	0,921631098094813	27,4252531095558	27,2998217180003	27,333361039548	27,1813739338955	27,7178014437035	27,333361039548	757138441703,051	802377853093,289	−0,08372466672099	0,0023741078489643	0,0100865386846957	1,05975051443496	0.0837246667209906	0,943618319952584	-0.083724666720990	2552
P26041	sp|P26041|MOES_MOUSE Moesin OS=Mus musculus GN=Msn PE=1 SV=3	1,06	0,635042188401656	0,882835066816268	31,0449398168704	30,9049840027489	30,6509513772108	30,9678402142619	30,9727064202915	30,836402611215	25430195902814,5	26967397399468,7	−0,08467371907749	0,00647060097514417	0,002500467296328	1,06044788260888	0.0846737190774888	0,942997780843157	-0.084673719077488	2553
Q3UUI3	sp|Q3UUI3|THEM4_MOUSE Acyl-coenzyme A thioesterase THEM4 OS=Mus musculus GN=Them4 PE=1 SV=1	1,06	0,628460416626379	0,877439099287608	28,4026187469632	28,6392790424527	28,796752241005	28,624551630105	28,6378238317448	28,7527330443723	2669422688627,47	2831146404276,59	−0,08485858932036	0,0069335247881556	0,002458427268106	1,06058377953335	0.084858589320366	0,94287695069219	-0.084858589320366	2554
O08688	sp|O08688|CAN5_MOUSE Calpain-5 OS=Mus musculus GN=Capn5 PE=2 SV=1	1,06	0,767841820442925	0,935597979611256	26,8257491606436	26,3744753513748	26,2965204560717	26,5000720757695	26,3976388083925	26,7761234567643	322353623447,231	341954929524,586	−0,08516198666161	0,0107822460206307	0,007371303991883	1,06080684270814	0.0851619866616126	0,942678685449552	-0.085161986661612	2555
Q922J9-2	sp|Q922J9-2|FACR1_MOUSE Isoform 2 of Fatty acyl-CoA reductase 1 OS=Mus musculus GN=Far1;sp|Q922J9-3|FACR1_MOUSE Isoform 3 of Fatty acyl-CoA reductase 1 OS=Mus musculus GN=Far1;sp|Q922J9|FACR1_MOUSE Fatty acyl-CoA reductase 1 OS=Mus musculus GN=Far1 PE=1	1,06	0,814139950616037	0,950652201935115	23,7382122559989	23,43383578992	23,0480487104139	23,2937395266692	23,3339271274258	23,7695776688911	14635251163,4496	15525475738,2506	−0,08518997197206	0,0147770133652051	0,0112457632248412	1,06082742037419	0.0851899719720685	0,942660399603232	-0.085189971972068	2556
Q91WD5	sp|Q91WD5|NDUS2_MOUSE NADH dehydrogenase [ubiquinone] iron-sulfur protein 2, mitochondrial OS=Mus musculus GN=Ndufs2 PE=1 SV=1	1,06	0,626288887982202	0,876302485373371	27,7048655608525	27,4684064394126	27,5067207470531	27,5887500513035	27,4684064394126	27,8005133029123	931439971837,21	988271470379,15	−0,08544459786383	0,00460499072472702	0,006087696828465	1,06101466574367	0.085444597863831	0,942494041115913	-0.085444597863831	2557
Q8C9H6	sp|Q8C9H6|STRP2_MOUSE Striatin-interacting proteins 2 OS=Mus musculus GN=Strip2 PE=1 SV=1;sp|Q8C9H6-3|STRP2_MOUSE Isoform 3 of Striatin-interacting proteins 2 OS=Mus musculus GN=Strip2;sp|Q8C9H6-4|STRP2_MOUSE Isoform 2 of Striatin-interacting proteins 2	1,06	0,645664072387159	0,889692946522666	26,0820246055589	25,7523846556078	26,0592651454691	26,1186864653252	26,072361667895	25,8804365348968	188914078125,435	200449508230,757	−0,08550866081941	0,00709042172781752	0,004854109777686	1,06106178120649	0.0855086608194108	0,942452190543454	-0.085508660819410	2558
Q3UCQ1-2	sp|Q3UCQ1-2|FOXK2_MOUSE Isoform 2 of Forkhead box protein K2 OS=Mus musculus GN=Foxk2;sp|Q3UCQ1|FOXK2_MOUSE Forkhead box protein K2 OS=Mus musculus GN=Foxk2 PE=1 SV=3	1,06	0,762900396387435	0,933454063694469	22,5286124376778	22,6026322326214	22,0092765517313	22,5602574624914	22,4148399246451	22,343926520924	5243073665,40176	5564509268,47501	−0,08584164664027	0,0144472304074094	0,004914643683212	1,061306711213	0.0858416466402753	0,942234689967307	-0.085841646640275	2559
Q9DCM0	sp|Q9DCM0|ETHE1_MOUSE Persulfide dioxygenase ETHE1, mitochondrial OS=Mus musculus GN=Ethe1 PE=1 SV=2	1,06	0,824862377446998	0,95563531252039	26,8788895373272	27,4236219219812	27,7938677088192	27,5067207470531	27,4089401757686	27,3594544203214	766775366632,27	813847181585,712	−0,08595393110745	0,0168187790221191	0,002732589321156	1,06138931556994	0.0859539311074531	0,942161359013707	-0.085953931107453	2560
P63030	sp|P63030|MPC1_MOUSE Mitochondrial pyruvate carrier 1 OS=Mus musculus GN=Mpc1 PE=1 SV=1	1,06	0,881408655829143	0,973716798448825	25,8385679589886	26,3165025351457	25,2961103046975	25,3412575352839	26,2003292010332	26,0883849331964	163007209408,854	173017264830,195	−0,08598023416292	0,0197751408941648	0,0180485393027382	1,06140866687825	0.0859802341629171	0,942144181789227	-0.085980234162917	2561
Q4KML4	sp|Q4KML4|ABRAL_MOUSE Costars family protein ABRACL OS=Mus musculus GN=Abracl PE=3 SV=1	1,06	0,779911219073998	0,940251150142017	26,1720458506377	26,2041255291928	26,1388382616424	26,4253543694505	26,4619673766869	25,806618885234	232386990279,072	246669086309,312	−0,08604761726270	0,0012473509604055	0,0140386286752258	1,06145824261973	0.086047617262704	0,942100178648528	-0.086047617262704	2562
Q9D071	sp|Q9D071|MMS19_MOUSE MMS19 nucleotide excision repair protein homolog OS=Mus musculus GN=Mms19 PE=2 SV=1;sp|Q9D071-2|MMS19_MOUSE Isoform 2 of MMS19 nucleotide excision repair protein homolog OS=Mus musculus GN=Mms19;sp|Q9D071-3|MMS19_MOUSE Isoform 3 of	1,06	0,929962816644981	0,98433518830764	26,7178490875963	24,4689312575944	25,7926120679867	25,8289851792492	25,9818743454278	25,347826740016	139286314910,715	147864490747,269	−0,08622211681449	0,0440504779222529	0,01286506716471 3	1,06158663786929	0.0862221168144954	0,941986234874904	-0.086222116814495	2563
Q9D4C9	sp|Q9D4C9|CLVS1_MOUSE Clavesin-1 OS=Mus musculus GN=Clvs1 PE=2 SV=1;sp|Q9D4C9-2|CLVS1_MOUSE Isoform 2 of Clavesin-1 OS=Mus musculus GN=Clvs1	1,06	0,754311613818739	0,930504185076609	28,4562699090736	28,7807657090108	29,0836947794638	28,9121400873384	28,7125758401729	28,8755859989111	3134769614711,56	3328137571003,63	−0,08635565139718	0,0109049426886248	0,003684958969877	1,06168490194131	0.0863556513971804	0,94189904949339	-0.086355651397180	2564
P28741	sp|P28741|KIF3A_MOUSE Kinesin-like protein KIF3A OS=Mus musculus GN=Kif3a PE=1 SV=2	1,06	0,735509257908729	0,924701393764781	29,1550985480598	29,0238572486725	28,6310783417307	29,0776180365338	29,0836947794638	28,8294358832546	3690112444032,9	3919229652047,68	−0,08690533355567	0,00942302232371447	0,005003085745822	1,06208949225525	0.0869053335556747	0,941540244293908	-0.086905333555674	2565
Q8BMZ5-2	sp|Q8BMZ5-2|SEN34_MOUSE Isoform 2 of tRNA-splicing endonuclease subunit Sen34 OS=Mus musculus GN=Tsen34;sp|Q8BMZ5|SEN34_MOUSE tRNA-splicing endonuclease subunit Sen34 OS=Mus musculus GN=Tsen34 PE=2 SV=2	1,06	0,741823055019018	0,925800067839242	22,4425016638024	22,4569741763383	22,8225685424939	22,376705503092	22,6937862021931	22,8329525082737	6364584539,81253	6761302625,51302	−0,08723487883079	0,00954085746732613	0,0103308549768412	1,0623321260357	0.0872348788307958	0,9413251990521	-0.087234878830795	2566
D3YZP9	sp|D3YZP9|CCDC6_MOUSE Coiled-coil domain-containing protein 6 OS=Mus musculus GN=Ccdc6 PE=1 SV=1	1,06	0,68192975957514	0,904517472334246	28,0488815319204	28,4245313453588	28,2082847129067	28,1195541428091	28,4643306249264	28,2792329488929	1815228607141,9	1928388711304,65	−0,08724463891207	0,0066793774916304	0,0060996229075125	1,06233931292044	0.0872446389120722	0,941318830845991	-0.087244638912072	2567
Q62420	sp|Q62420|SH3G2_MOUSE Endophilin-A1 OS=Mus musculus GN=Sh3gl2 PE=1 SV=2	1,06	0,792781682430677	0,942739338892545	34,5060432263395	33,7804120503292	33,8956357977178	34,0257535658343	34,1841736380791	34,1536747954851	619972987701707	658641612054619	−0,08728830379396	0,0114489826122703	0,002463409386041	1,06237146637027	0.087288303793969	0,941290341142756	-0.087288303793969	2568
Q8BM55	sp|Q8BM55|TM214_MOUSE Transmembrane protein 214 OS=Mus musculus GN=Tmem214 PE=2 SV=1	1,06	0,877297462609732	0,973607135155397	22,2332585619834	22,8107698771548	22,2366110073191	22,9492007514796	21,8174060215298	22,6960719862823	5493765910,40661	5837448201,11022	−0,08754240462432	0,014824298968361	0,026415050013484	1,06255859756467	0.0875424046243291	0,941124566957486	-0.087542404624328	2569

Q8JZP9	sp|Q8JZP9|GA2L1_MOUSE GAS2-like protein 1 OS=Mus musculus GN=Gas2l1 PE=2 SV=1;sp|Q8JZP9-2|GA2L1_MOUSE Isoform 2 of GAS2-like protein 1 OS=Mus musculus GN=Gas2l1	1,06	0,798469547367517	0,944726847417452	24,1764319247229	23,9242273809443	23,3797067172341	23,8166383799036	23,9154891156968	23,9303837516722	22276288957,8282	23670698005,8932	−0,08759333730737	0,0170900926629507	0,002588023451242	1,06259611063157	0.0875933373073802	0,941091342227515	-0.0875933373073	2570
Q8BYH7	sp|Q8BYH7|TBC17_MOUSE TBC1 domain family member 17 OS=Mus musculus GN=Tbc1d17 PE=2 SV=2	1,06	0,672510254034124	0,900894908348996	27,0782792312648	26,7131195606615	26,6724274176718	26,7753723973676	27,0009567310953	26,870933519598	444971202350,447	473028218720,318	−0,08821428002949	0,00833293192560556	0,004212047830718	1,06305355542486	0.0882142800294948	0,940686379248634	-0.0882142800294	2571
P29341	sp|P29341|PABP1_MOUSE Polyadenylate-binding protein 1 OS=Mus musculus GN=Pabpc1 PE=1 SV=2	1,06	0,260583809509666	0,682439759823898	30,5433566106407	30,4253950011429	30,4253950011429	30,59946784483	30,4762511723909	30,5018948562202	17008180287694,4	18080792287559,9	−0,08822910230336	0,0022355425217564	0,002129779563057	1,06306447731163	0.0882291023033699	0,940676714670104	-0.0882291023036	2572
Q3TIV5	sp|Q3TIV5|ZC3HF_MOUSE Zinc finger CCCH domain-containing protein 15 OS=Mus musculus GN=Zc3h15 PE=1 SV=2;sp|Q8WU90|ZC3HF_HUMAN Zinc finger CCCH domain-containing protein 15 OS=Homo sapiens GN=ZC3H15 PE=1 SV=1;sp|Q3TIV5-2|ZC3HF_MOUSE Isoform 2 of Zinc fin	1,06	0,795873406655653	0,944339896000096	26,5143677630458	27,2091856375552	27,219419790962	27,0716328405969	26,9547500236637	27,1009588259087	522030088438,324	555118382670,636	−0,08866250621182	0,0149786947461926	0,002860337381161	1,06338388335296	0.0886625062118255	0,940394165883813	-0.0886625062118	2573
P36916	sp|P36916|GNL1_MOUSE Guanine nucleotide-binding protein-like 1 OS=Mus musculus GN=Gnl1 PE=2 SV=4;sp|P36916-2|GNL1_MOUSE Isoform 2 of Guanine nucleotide-binding protein-like 1 OS=Mus musculus GN=Gnl1	1,06	0,638722004052299	0,885239443173553	29,2928257170814	29,142784813033	28,8842531253939	29,0865735685853	29,2720468450267	29,1461064414382	4373659629962,12	4651646156434,43	−0,08890040707396	0,00710055066019518	0,003246589570430	1,06355925014556	0.0889004070739663	0,94023910737755	-0.0889004070739	2574
Q80XI4	sp|Q80XI4|PI42B_MOUSE Phosphatidylinositol 5-phosphate 4-kinase type-2 beta OS=Mus musculus GN=Pip4k2b PE=1 SV=1	1,06	0,58732819552687	0,856921465604777	31,0751300491871	30,7148429894456	30,7552693729942	30,922771011418	30,9049840027489	30,9037738640846	24962957380557,8	26557810186095,6	−0,08934733322594	0,0063983665011552	0,000334878547617	1,06388877652693	0.0893473332259445	0,939947879950856	-0.0893473332259	2575
Q62348	sp|Q62348|TSN_MOUSE Translin OS=Mus musculus GN=Tsn PE=1 SV=1	1,06	0,575837153708092	0,849998193752035	29,8734429990539	30,0740303655199	30,2413049199915	30,1253373252879	30,0707031025332	30,1787354002317	11380541134464,5	12108457314835,4	−0,08944591120228	0,00612655545390793	0,001793110756249	1,0639614735161	0.0894459112022783	0,939883656402781	-0.0894459112022	2576
Q61036-2	sp|Q61036-2|PAK3_MOUSE Isoform 2 of Serine/threonine-protein kinase PAK 3 OS=Mus musculus GN=Pak3;sp|Q61036|PAK3_MOUSE Serine/threonine-protein kinase PAK 3 OS=Mus musculus GN=Pak3 PE=1 SV=2	1,06	0,733955806236869	0,924701393764781	28,1083623316671	28,4754028990078	28,6463208267188	28,6150528659003	28,5025208578432	28,299685761744	2179308380622,71	2319609175305,25	−0,09001139216562	0,00967521088235901	0,005613285795949	1,06437858722979	0.090011392165627	0,939515330351253	-0.0900113921656	2577
P62315	sp|P62315|SMD1_MOUSE Small nuclear ribonucleoprotein Sm D1 OS=Mus musculus GN=Snrpd1 PE=2 SV=1	1,06	0,856295157899021	0,964447573623737	27,8539827882304	28,9559879547382	28,7908543389162	28,62011627812	28,5057267905654	28,663140619517	2465974945462,67	2625593012723,82	−0,09048516274498	0,0208293374203591	0,002845065555448	1,06472817883038	0.0904851627449868	0,939206850990375	-0.0904851627449	2578
Q8CGF6	sp|Q8CGF6|WDR47_MOUSE WD repeat-containing protein 47 OS=Mus musculus GN=Wdr47 PE=1 SV=2	1,06	0,80124345392841	0,945787729921946	29,21883644974	28,5539886874427	28,7466735660147	28,8525957718364	29,1893852573842	28,6662319760378	3349502358621,85	3566970201813,66	−0,09075239590938	0,0118611650099971	0,009174098093232	1,06492541873632	0.0907523959093835	0,939032896018802	-0.0907523959093	2579
Q9D0I9	sp|Q9D0I9|SYRC_MOUSE Arginine--tRNA ligase, cytoplasmic OS=Mus musculus GN=Rars PE=2 SV=2	1,06	0,7451344956007	0,927664324783982	29,6534011397899	30,2534697710926	30,1584841687995	30,1871752749852	30,0547925878358	30,0121195493054	10921834062245,8	11631008615744,2	−0,09076106669100	0,0107437140318127	0,003034053496665	1,06493181909345	0.0907610666909985	0,93902725232802	-0.0907610666909	2580
O70443	sp|O70443|GNAZ_MOUSE Guanine nucleotide-binding protein G(z) subunit alpha OS=Mus musculus GN=Gnaz PE=2 SV=4	1,07	0,637114038045103	0,883588334123886	29,4242425054228	29,0359313240441	29,3099584234601	29,41407607155	29,3586082207491	29,1870270397349	5081922500848,8	5413428691938,4	−0,09116826576136	0,00682088662799129	0,0040371725649061	1,06523243733729	0.0911682657613679	0,938762250330687	-0.0911682657613	2581
P47738	sp|P47738|ALDH2_MOUSE Aldehyde dehydrogenase, mitochondrial OS=Mus musculus GN=Aldh2 PE=1 SV=1	1,07	0,53484364857085	0,8300431463043	32,2621420111148	32,0014621620972	31,9922601162753	32,0651775689469	32,2397875300273	32,1406176218661	85993093128347,9	91606887296950,2	−0,09123528002606	0,00477568023060555	0,002724022513449	1,0652819193307	0.0912352800260615	0,938718645134129	-0.0912352800260	2582
Q3TIR1-3	sp|Q3TIR1-3|TPC13_MOUSE Isoform 3 of Trafficking protein particle complex subunit 13 OS=Mus musculus GN=Trappc13;sp|Q3TIR1|TPC13_MOUSE Trafficking protein particle complex subunit 13 OS=Mus musculus GN=Trappc13 PE=2 SV=1;sp|Q3TIR1-2|TPC13_MOUSE Isoform	1,07	0,80518951815028	0,947809964440902	26,1555786458727	26,2599380141522	26,3165025351457	25,8175097401103	26,6595208620373	26,4451428857265	249819975743,981	266167371128,605	−0,09144488502902	0,00311066484986091	0,016633395157588	1,0654367023131	0.0914448850290318	0,938582271315572	-0.0914448850290	2583
A2AAJ9	sp|A2AAJ9|OBSCN_MOUSE Obscurin OS=Mus musculus GN=Obscn PE=2 SV=2;sp|A2AAJ9-3|OBSCN_MOUSE Isoform 3 of Obscurin OS=Mus musculus GN=Obscn;sp|A2AAJ9-2|OBSCN_MOUSE Isoform 2 of Obscurin OS=Mus musculus GN=Obscn	1,07	0,951896520728547	0,988708994871138	29,2449656239036	29,034879459619	27,1975304598764	26,9078396230827	29,2873491997899	29,472404298724	2366559939881,91	2521473008501,49	−0,09147531891855	0,0395314933640858	0,050085244202758	1,0654591781129	0.0914753189185638	0,938562471976789	-0.0914753189185	2584
Q3TJZ6	sp|Q3TJZ6|FA98A_MOUSE Protein FAM98A OS=Mus musculus GN=Fam98a PE=2 SV=1	1,07	0,714084231975416	0,917934955801873	25,0158762888834	25,2215409768175	25,5700819737257	25,426892640985	25,2157838114411	25,3552094720957	94245264044,0191	100420141989,392	−0,09155664214607	0,0110868529287525	0,004237642221827	1,0655192386377	0.0915566421460753	0,93850956767194	-0.0915566421460	2585

P57724	sp|P57724|PCBP4_MOUSE Poly(rC)-binding protein 4 OS=Mus musculus GN=Pcbp4 PE=2 SV=1	1,07	0,832930189042322	0,957953242267243	22,6823866658236	23,0064630885348	23,3777626719801	23,2567715611815	23,3607684831536	22,6399360793508	9963598536,27311	10618094112,5723	−0,09178603654961	0,015113893658339	0,016877747783384	1,06568867401837	0.0917860365496116	0,938360352680973	-0.09178603654961	2586
Q8R3N6	sp|Q8R3N6|THOC1_MOUSE THO complex subunit 1 OS=Mus musculus GN=Thoc1 PE=1 SV=1	1,07	0,779849172947858	0,940251150142017	22,9752239618567	22,7197042232794	22,3016141097192	22,9075582228068	22,7662261675712	22,5136465441664	6974413661,72423	7432615442,57287	−0,09179803128047	0,015003356887804 9	0,0087797787973626	1,06569753431221	0.0917980312804789	0,93835255107857	-0.091798031280478	2587
P24547	sp|P24547|IMDH2_MOUSE Inosine-5-monophosphate dehydrogenase 2 OS=Mus musculus GN=Impdh2 PE=1 SV=2	1,07	0,746839238050752	0,927723946774046	27,5836630656943	28,1512065915307	28,1567892511131	28,1229950257761	28,0160964439985	27,9434810304077	1394959226946,42	1486616973337,28	−0,09181003073069	0,0117757170691322	0,0032218671496534	1,06570639816584	0.0918100307306946	0,938344746471514	-0.091810030730694	2588
Q76MZ3	sp|Q76MZ3|2AAA_MOUSE Serine/threonine-protein phosphatase 2A 65 kDa regulatory subunit A alpha isoform OS=Mus musculus GN=Ppp2r1a PE=1 SV=3	1,07	0,710801441012615	0,916805961322165	33,2966158606149	33,8575362770501	33,5825229115311	33,7374582476704	33,658865904478	33,5323458377698	382937031534049	408245570046251	−0,09233004961857	0,00835280213872776	0,0030759917500086	1,06609060087716	0.0923300496185755	0,938006581408012	-0.092330049618575	2589
Q9DCJ9	sp|Q9DCJ9|NPL_MOUSE N-acetylneuraminate lyase OS=Mus musculus GN=Npl PE=1 SV=1	1,07	0,838487176936834	0,959746519741951	25,2300086282913	25,7334731810382	26,3143352941035	25,7441964423723	25,8727859475999	25,8539662924407	153854373878,672	164084858496,117	−0,09287665707783	0,0210651724308237	0,0026893508692291	1,06649459719301	0.0928766570778326	0,937651257335928	-0.092876657077832	2590
Q80UG5-3	sp|Q80UG5-3|SEPT9_MOUSE Isoform 3 of Septin-9 OS=Mus musculus GN=Sept9;sp|Q80UG5|SEPT9_MOUSE Septin-9 OS=Mus musculus GN=Sept9 PE=1 SV=1;sp|Q80UG5-2|SEPT9_MOUSE Isoform 2 of Septin-9 OS=Mus musculus GN=Sept9	1,07	0,543834521022291	0,834222673511382	30,5173788922969	30,4210659683686	30,2744604699913	30,3129183925351	30,5719607650948	30,5213672527491	16011074850593,4	17076917324056,6	−0,09297740563844	0,00402324077446715	0,0045063729514816	1,06656907693013	0.0929774056384454	0,937585780077431	-0.092977405638445	2591
Q499X9	sp|Q499X9|SYMM_MOUSE Methionine--tRNA ligase, mitochondrial OS=Mus musculus GN=Mars2 PE=2 SV=2	1,07	0,640511687153879	0,886096479119614	25,4918299528731	25,8849767619169	25,9200419149704	25,8014926834204	25,8525777820242	25,8362365598065	154833504174,662	165147085618,244	−0,09303382259759	0,00922753732829524	0,0010100285720860	1,06661078620263	0.09303382259759	0,937549116262197	-0.093033822597596	2592
Q8CI95	sp|Q8CI95|OSB11_MOUSE Oxysterol-binding protein-related protein 11 OS=Mus musculus GN=Osbpl11 PE=1 SV=2	1,07	0,634669453983095	0,882835066816268	25,3815883916897	25,0898230655906	25,1359541100947	25,4664846831164	25,1359541100947	25,1986693862256	88163174414,3447	94044702041,7456	−0,09317050187672	0,00622317296547102	0,0069478229984913	1,06671184047615	0.0931705018767273	0,937460298137899	-0.093170501876727	2593
Q8CBG9-2	sp|Q8CBG9-2|RN170_MOUSE Isoform 2 of E3 ubiquitin-protein ligase RNF170 OS=Mus musculus GN=Rnf170;sp|Q8CBG9|RN170_MOUSE E3 ubiquitin-protein ligase RNF170 OS=Mus musculus GN=Rnf170 PE=1 SV=1;sp|Q8CBG9-5|RN170_MOUSE Isoform 5 of E3 ubiquitin-protein liga	1,07	0,555492555169378	0,840205991686519	24,758319535241	24,9050721699232	24,6631481372109	24,6738015764935	24,9442410806871	24,9023550022812	57526599673,0631	61366661550,9335	−0,09322590378282	0,00491917360728846	0,0058599264073922	1,06675280478412	0.0932259037828267	0,937424298783417	-0.093225903782826	2594
Q61792	sp|Q61792|LASP1_MOUSE LIM and SH3 domain protein 1 OS=Mus musculus GN=Lasp1 PE=1 SV=1	1,07	0,565455272483795	0,845110794140252	31,8280562746036	31,6049790076691	31,8823889345278	31,9696296459334	31,7123495077502	31,8280562746036	62852263979608	67064668821317,8	−0,09358820990447	0,00462706039331602	0,0040474260883791	1,06702073362189	0.0935882099044707	0,937188911602128	-0.093588209904470	2595
Q99KW9	sp|Q99KW9|TIP_MOUSE T-cell immunomodulatory protein OS=Mus musculus GN=Itfg1 PE=2 SV=2	1,07	0,674331813579654	0,901104029547498	26,7414467689503	26,5688885730431	26,3073973295377	26,7397710425091	26,6381041414573	26,4349457329772	335619603741,847	358170024358,924	−0,09381761473078	0,00823451909339565	0,0058337647988386	1,06719041547532	0.0938176147307899	0,937039899814511	-0.093817614730789	2596
Q91Z53	sp|Q91Z53|GRHPR_MOUSE Glyoxylate reductase/hydroxypyruvate reductase OS=Mus musculus GN=Grhpr PE=1 SV=1	1,07	0,68949163804255	0,907856236295823	29,5685996112172	29,8931980979023	30,1213977470374	29,8955901225831	29,9877542613183	29,8955901225831	9300275618554,67	9927318350348,52	−0,09413058573859	0,00930296350003471	0,0017780671134363	1,06742195151108	0.0941305857385945	0,936836645137724	-0.094130585738594	2597
Q9DCC4	sp|Q9DCC4|P5CR3_MOUSE Pyrroline-5-carboxylate reductase 3 OS=Mus musculus GN=Pycrl PE=2 SV=2	1,07	0,654430372345688	0,891901943526937	27,5118748975731	27,6931390764562	27,924640903582	27,9088339896986	27,615484436771	27,802797894039	1082056982705,13	1155674694193,52	−0,09495888147822	0,00746635758963192	0,0053478219128680	1,06803496734927	0.0949588814782322	0,936298932685583	-0.094958881478232	2598
P80317	sp|P80317|TCPZ_MOUSE T-complex protein 1 subunit zeta OS=Mus musculus GN=Cct6a PE=1 SV=3	1,07	0,692985220493784	0,910309912045398	31,7533387554372	32,2466587951576	32,1814288440177	32,2512806916565	32,0501774277855	32,077801964065	83885632136248	89603905921723,6	−0,09513789396295	0,0083585886523558	0,0033931913381087	1,06816749948535	0.0951378939629504	0,936182762049782	-0.095137893962950	2599
P60670	sp|P60670|NPL4_MOUSE Nuclear protein localization protein 4 homolog OS=Mus musculus GN=Nploc4 PE=1 SV=3;sp|P60670-2|NPL4_MOUSE Isoform 2 of Nuclear protein localization protein 4 homolog OS=Mus musculus GN=Nploc4	1,07	0,656458544028662	0,892226633215307	28,2953913503404	28,7045051167192	28,2910319082209	28,5598383543679	28,5486023400049	28,381044130583	2223957669031,43	2376131691603,29	−0,09548546842769	0,00835271983181811	0,0035141531412455	1,06842487367942	0.0954854684276987	0,93595724382213	-0.095485468427698	2600
Q7TQK1	sp|Q7TQK1|INT7_MOUSE Integrator complex subunit 7 OS=Mus musculus GN=Ints7 PE=2 SV=1;sp|Q7TQK1-2|INT7_MOUSE Isoform 2 of Integrator complex subunit 7 OS=Mus musculus GN=Ints7	1,07	0,482018468293824	0,802303567138125	23,9166756183545	23,6506356046121	23,7132586719014	23,8542629495858	23,8940402301816	23,7308311655272	20841040173,7909	22267145099,5815	−0,09548931597579	0,00585394026420216	0,0035717857773297	1,0684277230838	0.0954893159757947	0,935954747705066	-0.095489315975794	2601

Q91WC9	sp|Q91WC9|DGLB_MOUSE Sn1-specific diacylglycerol lipase beta OS=Mus musculus GN=Daglb PE=1 SV=2	1,07	0,802248829538115	0,946065844965837	21,7012179112435	21,8627446203422	21,1739565399968	21,334360086982	21,7145801016844	21,8876980909906	2353822306,56534	2515018743,66287	−0,095563738672836	0,0166925629470094	0,0130768139041557	1,06848284029254	0.0955637386728321	0,935906466898627	-0.095563738672832	2602
Q9ERF3	sp|Q9ERF3|WDR61_MOUSE WD repeat-containing protein 61 OS=Mus musculus GN=Wdr61 PE=2 SV=1	1,07	0,788251163140697	0,942041082015698	27,9366295152886	27,4718930142898	28,2774605455253	27,8477202619897	27,9221362330221	28,1148719832427	1302527458457,74	1391740390567,44	−0,095576335841689	0,0144959336608664	0,0049308251453964	1,06849216999643	0.0955763358416953	0,935898294887219	-0.095576335841695	2603
P08003	sp|P08003|PDIA4_MOUSE Protein disulfide-isomerase A4 OS=Mus musculus GN=Pdia4 PE=1 SV=3	1,07	0,540509297732499	0,832215638537251	30,6166732848265	30,6271401858965	30,3063347984404	30,6125340776466	30,6071786088968	30,5297954510822	17916014430403,8	19147042465965,7	−0,095871831194197	0,00597281471759795	0,0015139266123619	1,0687110428686	0.0958718311941927	0,93570662217154	-0.095871831194192	2604
Q9Z2G0	sp|Q9Z2G0|FEM1B_MOUSE Protein fem-1 homolog B OS=Mus musculus GN=Fem1b PE=1 SV=1	1,07	0,842475448509426	0,959771286845933	22,3884232655303	22,1323356543618	22,1271865824891	21,8627446203422	22,9194574103533	22,0655043166933	4449163038,17946	4755505243,30411	−0,096064660152086	0,00672312624700236	0,0251677179315673	1,06885389510248	0.0960646601520883	0,935581565059572	-0.096064660152088	2605
Q8R0Y6	sp|Q8R0Y6|AL1L1_MOUSE Cytosolic 10-formyltetrahydrofolate dehydrogenase OS=Mus musculus GN=Aldh1l1 PE=1 SV=1	1,07	0,728681552515165	0,924136788730465	32,2466587951576	31,9282291432254	31,6424407003006	31,9095496846577	32,1651705876441	31,9425028325732	74298326677009,9	79417593080480,2	−0,096128918358541	0,00946350608742041	0,0043445231374519	1,06890150333701	0.0961289183585424	0,935539894815466	-0.096128918358542	2606
Q9WTX2	sp|Q9WTX2|PRKRA_MOUSE Interferon-inducible double-stranded RNA-dependent protein kinase activator A OS=Mus musculus GN=Prkra PE=1 SV=1	1,07	0,770984773592015	0,936218563920244	21,7674627603317	22,2077631703379	22,3731430345571	22,4474378958212	22,1323356543618	21,968529841547	4026337490,55486	4303815523,48432	−0,096148135206357	0,014155574744217	0,0109726868376668	1,0689157413109	0.0961481352063579	0,935527433409874	-0.096148135206357	2607
O35874	sp|O35874|SATT_MOUSE Neutral amino acid transporter A OS=Mus musculus GN=Slc1a4 PE=1 SV=1	1,07	0,50701016166672	0,816070611278377	25,9945560886311	26,251725356772	26,3063436289981	26,3073973295377	26,242967719549	26,2029568269526	235319113125,557	251600234048,595	−0,096514760148503	0,00635874018137496	0,0020073009191966	1,0691874140897	0.0965147601485007	0,935289722664195	-0.096514760148500	2608
Q7TPD2-2	sp|Q7TPD2-2|F185A_MOUSE Isoform 2 of Protein FAM185A OS=Mus musculus GN=Fam185a;sp|Q7TPD2|F185A_MOUSE Protein FAM185A OS=Mus musculus GN=Fam185a PE=2 SV=1	1,07	0,664924376892703	0,896678990733797	24,4818124226021	24,5429385120525	24,0597088731534	24,3736583005827	24,4697558524359	24,4418972929147	38024122678,7237	40657011742,0361	−0,096589220759239	0,0108009909887049	0,0020239754629110	1,06924259858823	0.0965892207592397	0,935241451584839	-0.096589220759239	2609
Q91VU6	sp|Q91VU6|DCA11_MOUSE DDB1- and CUL4-associated factor 11 OS=Mus musculus GN=Dcaf11 PE=1 SV=1;sp|Q91VU6-2|DCA11_MOUSE Isoform 2 of DDB1- and CUL4-associated factor 11 OS=Mus musculus GN=Dcaf11	1,07	0,743809912469516	0,92694071457895	24,197055235212	24,7608480287448	24,4042625278283	24,723259791271	24,3017734131296	24,53802616775	41712023767,1211	44600896234,6062	−0,096609390713229	0,011661685440734	0,0086153512948105	1,0692575475027	0.0966093907132273	0,935228376302367	-0.096609390713227	2610
Q9CPR5-2	sp|Q9CPR5-2|RM15_MOUSE Isoform 2 of 39S ribosomal protein L15, mitochondrial OS=Mus musculus GN=Mrpl15;sp|Q9CPR5|RM15_MOUSE 39S ribosomal protein L15, mitochondrial OS=Mus musculus GN=Mrpl15 PE=2 SV=1	1,07	0,813999610512177	0,950652201935115	22,4605995625438	22,7080917274642	22,8085879999257	22,8828786350751	22,2077631703379	23,088004659985	6929774407,89148	7410883415,14403	−0,096837141813457	0,00790384390490003	0,0202654997263825	1,0694263592051	0.0968371418134557	0,935080748096858	-0.096837141813455	2611
Q8K2A7	sp|Q8K2A7|INT10_MOUSE Integrator complex subunit 10 OS=Mus musculus GN=Ints10 PE=1 SV=3	1,07	0,793843945987544	0,943508162041755	24,2617206040686	23,8919023724206	23,3953782803666	23,9631070192037	23,8072085417944	23,9801195100244	22791809721,2488	24374703516,747	−0,096869188255206	0,0182272014623826	0,0039846237331967	1,0694501145305	0.0968691882552072	0,935059977471699	-0.096869188255207	2612
Q811C2	sp|Q811C2|ATG4C_MOUSE Cysteine protease ATG4C OS=Mus musculus GN=Atg4c PE=1 SV=2	1,07	0,652641500721718	0,891901943526937	23,7058662910714	23,4318412203999	23,3886660809348	23,5542435875806	23,779469321038	23,3944655483203	16208330857,5397	17336145353,7088	−0,097047625762889	0,00731776798008789	0,0082043735271780	1,06958239599634	0.0970476257628869	0,934944333174513	-0.097047625762886	2613
P54285	sp|P54285|CACB3_MOUSE Voltage-dependent L-type calcium channel subunit beta-3 OS=Mus musculus GN=Cacnb3 PE=2 SV=2	1,07	0,648946341437592	0,889714023663571	25,5872669040152	26,0333327348677	26,0103345112904	25,9318139841019	25,8984378098441	26,0031584198129	173072782325,879	185157022307,388	−0,097370404277836	0,0097059473847226	0,0020619105608033	1,06982172366511	0.097370404277839	0,934735178655836	-0.097370404277838	2614
Q9D6K5	sp|Q9D6K5|SYJ2B_MOUSE Synaptojanin-2-binding protein OS=Mus musculus GN=Synj2bp PE=1 SV=1;sp|Q9D6K5-2|SYJ2B_MOUSE Isoform 2 of Synaptojanin-2-binding protein OS=Mus musculus GN=Synj2bp;sp|Q9D6K5-4|SYJ2B_MOUSE Isoform 4 of Synaptojanin-2-binding protein	1,07	0,715384296096361	0,918558942968132	26,4531431572982	26,1042219466073	25,9147147066523	26,0564348417916	26,2250524440376	26,3932386786055	229085225919,743	245094246916,377	−0,097452200417830	0,0104413856705889	0,0064214509443570	1,06988238081422	0.0974522004178294	0,934682183698518	-0.097452200417829	2615
O88587-2	sp|O88587-2|COMT_MOUSE Isoform Soluble of Catechol O-methyltransferase OS=Mus musculus GN=Comt;sp|O88587|COMT_MOUSE Catechol O-methyltransferase OS=Mus musculus GN=Comt PE=1 SV=2	1,07	0,729307289549557	0,924155719504589	28,5119947056563	28,410000627506	28,8830163528639	28,6768520225877	28,4367014913091	28,8941100043926	2639658218479,85	2824129164816,79	−0,097454931144432	0,00870341809293658	0,0079806742267658	1,06988440588463	0.0974549311444301	0,934680414538013	-0.097454931144430	2616

Q9D0E3	sp|Q9D0E3|LYSM1_MOUSE LysM and putative peptidoglycan-binding domain-containing protein 1 OS=Mus musculus GN=Lysmd1 PE=2 SV=1	1,07	0,771681222196846	0,936785701190125	25,3615584974045	25,9039797918375	25,8351165872011	26,0052225567599	25,4643078754517	25,8344529325275	145031716821,516	155202174400,987	−0,0977803339120051	0,0114902596121588	0,0107308897624739	1,07012574768033	0.0977803339119989	0,93446961926452	-0,0977803339119981	2617
Q8R3D1	sp|Q8R3D1|TBC13_MOUSE TBC1 domain family member 13 OS=Mus musculus GN=Tbc1d13 PE=2 SV=1	1,07	0,783698965105323	0,940251150142017	26,475991979372	25,9546021761348	26,6477017691461	26,5695977401837	26,1582354818588	26,5548176288872	280385695135,704	300150629414,905	−0,0982739462402466	0,0136934713385179	0,0088298198618208	1,07049194956125	0.0982739462402478	0,934149948918216	-0,0982739462402470	2618
Q61035	sp|Q61035|SYHC_MOUSE Histidine--tRNA ligase, cytoplasmic OS=Mus musculus GN=Hars PE=2 SV=2	1,07	0,656170968638875	0,892226633215307	29,5272518601179	29,9877542613183	29,8880330606952	29,9903371484726	29,8358818720028	29,7814723531849	8758213491137,08	9376526093901,83	−0,0984169006086076	0,00812960483167833	0,0036275559323598	1,07059802816984	0.0984169006086082	0,934057390064015	-0,0984169006086081	2619
Q9Z2X2	sp|Q9Z2X2|PSD10_MOUSE 26S proteasome non-ATPase regulatory subunit 10 OS=Mus musculus GN=Psmd10 PE=1 SV=3	1,07	0,741139746618115	0,925497610201997	23,9631070192037	24,1749746788762	24,5775770341929	24,4818124226021	24,1280009506016	24,3111380538731	33625579561,7694	36007165923,5365	−0,0987249175747611	0,0128774094496677	0,0072794846173240	1,07082662641969	0.0987249175747597	0,933857989078492	-0,0987249175747597	2620
Q8K3A9	sp|Q8K3A9|MEPCE_MOUSE 7SK snRNA methylphosphate capping enzyme OS=Mus musculus GN=Mepce PE=1 SV=2	1,07	0,751432524802169	0,928854093158237	23,4448047131735	23,0008472762563	22,8346140135788	23,2373012347919	23,3145151636562	22,9337468186366	10699062962,7302	11456858757,1075	−0,0987270908853433	0,0136598732376161	0,0086903288006064	1,07082823953995	0.0987270908853	0,933856582293363	-0,0987270908853	2621
Q8C166	sp|Q8C166|CPNE1_MOUSE Copine-1 OS=Mus musculus GN=Cpne1 PE=1 SV=1	1,07	0,695271324367569	0,910775336595785	28,2458362215109	28,8465312886039	28,5471743747981	28,6581410656887	28,5904583519206	28,5963004492755	2498006080153,61	2674989637119,33	−0,0987563140659571	0,0105213574764575	0,0013106460301121	1,07084993041924	0.098756314065955	0,933837666318471	-0,0987563140659551	2622
Q61166	sp|Q61166|MARE1_MOUSE Microtubule-associated protein RP/EB family member 1 OS=Mus musculus GN=Mapre1 PE=1 SV=3	1,07	0,751980280875183	0,929192414636566	30,5524835884731	31,0680797664268	31,2478449838581	31,138693565225	30,9997610762329	30,9355404205447	27802195627679,6	29774249366753,1	−0,0988663153657971	0,0116598456204938	0,0033470468626375	1,070931582724	0.0988663153657935	0,933766466627516	-0,0988663153657931	2623
P51791-2	sp|P51791-2|CLCN3_MOUSE Isoform 2 of H(+)/Cl(-) exchange transporter 3 OS=Mus musculus GN=Clcn3;sp|P51791|CLCN3_MOUSE H(+)/Cl(-) exchange transporter 3 OS=Mus musculus GN=Clcn3 PE=1 SV=2	1,07	0,708528865063494	0,916805961322165	25,7191474648085	25,6510636985623	25,2931187432148	25,7162865377133	25,7816672426511	25,3711772595212	125358479041,546	134259949586,027	−0,0989694248070601	0,0089557677538005	0,0086078294157774	1,07100812495923	0.0989694248070525	0,933699732705639	-0,0989694248070521	2624
Q8R1S0	sp|Q8R1S0|COQ6_MOUSE Ubiquinone biosynthesis monooxygenase COQ6 OS=Mus musculus GN=Coq6 PE=1 SV=3	1,07	0,690837050966961	0,908901267924358	26,0926202587236	25,9259085166726	26,1745346539103	26,1988563938474	26,3652422189801	25,8351165872011	208739829062,295	223588184196,035	−0,0991380457637701	0,00486105482089506	0,0103744495530457	1,07113331078425	0.0991380457637675	0,933590609060444	-0,0991380457637671	2625
O89112	sp|O89112|LANC1_MOUSE LanC-like protein 1 OS=Mus musculus GN=Lancl1 PE=1 SV=1	1,07	0,783273423350754	0,940251150142017	30,59946784483	30,1824637696736	29,7453450924431	30,2310954123744	30,3198830461946	30,1824637696736	12739887076236,5	13646179970444,8	−0,0991446583919641	0,0141537732548987	0,0023039121407336	1,07113822036137	0.0991446583919607	0,933586329934739	-0,0991446583919601	2626
Q60967	sp|Q60967|PAPS1_MOUSE Bifunctional 3-phosphoadenosine 5-phosphosulfate synthase 1 OS=Mus musculus GN=Papss1 PE=1 SV=1	1,07	0,839225812150803	0,959771286845933	26,7306224117162	27,0515185236898	27,6376773679146	27,615484436771	26,9068501601645	27,1039852603133	611964085822,465	655571548844,977	−0,0993062559293841	0,0169472224510798	0,0134425045877158	1,07125820621304	0.0993062559293854	0,933481763967118	-0,0993062559293851	2627
Q91WN1	sp|Q91WN1|DNJC9_MOUSE DnaJ homolog subfamily C member 9 OS=Mus musculus GN=Dnajc9 PE=2 SV=2	1,07	0,851237604131265	0,962641439679318	21,5318623747088	22,7318219676303	22,1096485360386	22,0134108257316	22,1382342375959	22,4285357176485	4059981657,90554	4349790820,54031	−0,09947281443224	0,0271245957204278	0,0095969307703793	1,07138188963747	0.0994728144322431	0,933374000132087	-0,0994728144322431	2628
P97822	sp|P97822|AN32E_MOUSE Acidic leucine-rich nuclear phosphoprotein 32 family member E OS=Mus musculus GN=Anp32e PE=1 SV=2;sp|P97822-2|AN32E_MOUSE Isoform 2 of Acidic leucine-rich nuclear phosphoprotein 32 family member E OS=Mus musculus GN=Anp32e	1,07	0,662954854899819	0,896098566968415	28,0469322356155	28,1195541428091	28,5325900295205	28,3357637084218	28,2082847129067	28,3623185321326	1825774632944,95	1956390515804,22	−0,0996856806796881	0,00927847103856989	0,0029094374846975	1,07153998116875	0.0996856806796871	0,933236293161247	-0,0996856806796871	2629
P59708	sp|P59708|SF3B6_MOUSE Splicing factor 3B subunit 6 OS=Mus musculus GN=Sf3b6 PE=1 SV=1	1,07	0,796211122261254	0,944339896000096	25,5185024275634	25,2615134752874	24,846359962555	24,961398664519	25,5940945146071	25,2783415939017	88723617644,7738	95076239175,8708	−0,0997666457381571	0,0134539798861449	0,0125147875948877	1,07160011843218	0.0997666457381565	0,933183920754943	-0,0997666457381561	2630
Q9Z0E6	sp|Q9Z0E6|GBP2_MOUSE Interferon-induced guanylate-binding protein 2 OS=Mus musculus GN=Gbp2 PE=1 SV=1	1,07	0,739014776668714	0,925497610201997	22,4202327163321	22,6513182672976	23,1210148714882	22,8039538529234	22,840723799719	22,7554239515794	7445348299,81634	7978640480,75424	−0,0998035986798031	0,015709806577386	0,0018765243602248	1,07162756656409	0.0998035986798025	0,933160018649257	-0,0998035986798021	2631
O08599-2	sp|O08599-2|STXB1_MOUSE Isoform 2 of Syntaxin-binding protein 1 OS=Mus musculus GN=Stxbp1	1,07	0,702411619454114	0,914323584944716	27,1437352154627	27,680487545215	27,5571437909233	27,6817373259402	27,4054318063668	27,5020965064079	843187775768,631	903692706312,501	−0,0999783273281981	0,0102378349936997	0,0050931764575114	1,0717573620997	0.0999783273281978	0,933047007991512	-0,0999783273281971	2632
Q6P1F6	sp|Q6P1F6|2ABA_MOUSE Serine/threonine-protein phosphatase 2A 55 kDa regulatory subunit B alpha isoform OS=Mus musculus GN=Ppp2r2a PE=1 SV=1	1,07	0,649035364997653	0,889714023663571	31,9827023770707	31,7211883042871	31,5806609515781	31,7896349241163	32,0149961289679	31,687944408101	62208776747132,2	66675486800294	−0,1000383151340231	0,00642388107864542	0,0052583422547213	1,07180192710296	0.100038315134023	0,933008212350358	-0,100038315134023	2633
P22892	sp|P22892|AP1G1_MOUSE AP-1 complex subunit gamma-1 OS=Mus musculus GN=Ap1g1 PE=1 SV=3	1,07	0,684860915623475	0,905764480190838	30,8467694077559	30,7005833571881	30,2860766360239	30,6799097956272	30,7354901350717	30,6260640465842	19690219053972,4	21104090336375,6	−0,1000434838611131	0,00950144414266398	0,0017833919578125	1,07180576704239	0.100043483861112	0,933004869678452	-0.100043483861111	2634
Q8BHL8	sp|Q8BHL8|PSMF1_MOUSE Proteasome inhibitor PI31 subunit OS=Mus musculus GN=Psmf1 PE=2 SV=1	1,07	0,80702766112316	0,948497813008292	25,0570831721089	25,5400198303093	25,7281474875069	25,502807308767	25,8662283570416	25,1647282924477	111998370979,337	120059663590,195	−0,10027378224005	0,0136058561017712	0,0137518252191973	1,07197687377386	0.100273782240043	0,932855945370845	-0.100273782240043	2635
O08759	sp|O08759|UBE3A_MOUSE Ubiquitin-protein ligase E3A OS=Mus musculus GN=Ube3a PE=1 SV=1	1,07	0,733765206444202	0,924701393764781	29,5962766892579	29,3411392654344	29,0477196217778	29,2130685094284	29,6482708054861	29,3328195161808	5459501086929,17	5853453493145,6	−0,1005189376261031	0,00935957223314462	0,0076472770842225	1,07215904895772	0.100518937626103	0,932697439780166	-0.100518937626103	2636
O08599	sp|O08599|STXB1_MOUSE Syntaxin-binding protein 1 OS=Mus musculus GN=Stxbp1 PE=1 SV=2	1,07	0,701260981907557	0,913830966176594	34,7873562853203	35,2909566904371	35,2475710661237	35,3451936065751	35,0731894837483	35,1168009278487	1768004693753200	1895757088629010	−0,1006520126176761	0,00794886836772258	0,0041527955588680	1,07225795006495	0.100652012617679	0,932611411218196	-0.100652012617679	2637

Q8BGZ2-2	sp|Q8BGZ2-2|F168A_MOUSE Isoform 2 of Protein FAM168A OS=Mus musculus GN=Fam168a;sp|Q8BGZ2|F168A_MOUSE Protein FAM168A OS=Mus musculus GN=Fam168a PE=2 SV=1	1,07	0,704596758596965	0,915824338525891	24,0578211291045	24,6044469627772	24,6103867125833	24,5360198544844	24,4941949562678	24,4526054481224	40485845986,2534	43423795865,3851	−0,101068219614316	0,012992150858839	0,00170273507693446	1,07256733328802	0.101068219614319	0,932342398434273	-0.101068219614319	2638
P62814	sp|P62814|VATB2_MOUSE V-type proton ATPase subunit B, brain isoform OS=Mus musculus GN=Atp6v1b2 PE=1 SV=1	1,07	0,654272594359631	0,891901943526937	33,8575362770501	34,3323405522909	34,2337174910887	34,3323405522909	34,162960021396	34,1391253660482	671945335264227	720866670612073	−0,10138857721146	0,00733889535929939	0,00307933515103567	1,07280552863517	0.101388577211461	0,932135390159864	-0.101388577211461	2639
Q9D1M0	sp|Q9D1M0|SEC13_MOUSE Protein SEC13 homolog OS=Mus musculus GN=Sec13 PE=2 SV=3;sp|P55735-2|SEC13_HUMAN Isoform 2 of Protein SEC13 homolog OS=Homo sapiens GN=SEC13;sp|P55735|SEC13_HUMAN Protein SEC13 homolog OS=Homo sapiens GN=SEC13 PE=1 SV=3;sp|P55735-	1,07	0,566542649031589	0,845222501953069	28,1567892511131	27,9932539266564	28,0347736742627	28,3498300207127	28,0716553884336	27,9743749343137	1538156315393,77	1650259142913,17	−0,101490466164934	0,00302924166448503	0,00692647001277833	1,07288129717213	0.10149046616493	0,93206956131659	-0.10149046616493	2640
Q91ZM2-6	sp|Q91ZM2-6|SH2B1_MOUSE Isoform 6 of SH2B adapter protein 1 OS=Mus musculus GN=Sh2b1;sp|Q91ZM2-2|SH2B1_MOUSE Isoform 2 of SH2B adapter protein 1 OS=Mus musculus GN=Sh2b1;sp|Q91ZM2-3|SH2B1_MOUSE Isoform 3 of SH2B adapter protein 1 OS=Mus musculus GN=Sh2b	1,07	0,730470566423708	0,924701393764781	27,1365607688038	26,7434074548887	26,7414467689503	27,0515185236898	27,1464541510055	26,6350593510396	468970168869,4	503245524673,999	−0,101766281403208	0,008467549893405	0,0100956977405709	1,07308643082188	0.101766281403208	0,931891384773263	-0.101766281403208	2641
Q91ZA3	sp|Q91ZA3|PCCA_MOUSE Propionyl-CoA carboxylase alpha chain, mitochondrial OS=Mus musculus GN=Pcca PE=1 SV=2	1,07	0,596924675994656	0,861464850775537	30,1189132769238	29,9670200277696	29,6831401315376	30,0689715847678	29,9349818756405	29,97721683577	9894740593711,31	10619610256519,6	−0,101997029344742	0,00739206101473029	0,00228391124861092	1,07325807644406	0.101997029344748	0,931742347854689	-0.101997029344748	2642
P59017	sp|P59017|B2L13_MOUSE Bcl-2-like protein 13 OS=Mus musculus GN=Bcl2l13 PE=1 SV=2	1,07	0,610741308210036	0,866610575848175	27,749949888637	28,0992599920198	27,854757020185	28,0649683586343	28,0649683586343	27,7863646768548	1310359050948,27	1406464837702,54	−0,102111306822386	0,00642443355635537	0,005750440903627	1,07334309377626	0.102111306822385	0,931668546430739	-0.102111306822385	2643
Q9JIX0	sp|Q9JIX0|ENY2_MOUSE Transcription and mRNA export factor ENY2 OS=Mus musculus GN=Eny2 PE=2 SV=1	1,07	0,722087083710897	0,921437524026923	24,1659420657239	24,0659982136174	23,8848298274034	23,7476238449328	24,3237320068627	24,2586916472883	27540496789,3973	29569694313,3407	−0,102564745420423	0,00592782160356889	0,013086720576296	1,07368049819365	0.102564745420423	0,931375769311626	-0.102564745420423	2644
Q8C7H1	sp|Q8C7H1|MMAA_MOUSE Methylmalonic aciduria type A homolog, mitochondrial OS=Mus musculus GN=Mmaa PE=2 SV=1	1,07	0,843129617792527	0,959771286845933	25,4737767577379	24,7970047172291	24,4792576365056	25,0866146564925	25,2894343129533	24,5876185868543	66248569148,7109	71138120645,9333	−0,102733565981849	0,0203857625593179	0,0144539458510084	1,07380614494853	0.102733565981849	0,931266788427564	-0.102733565981849	2645
P61804	sp|P61804|DAD1_MOUSE Dolichyl-diphosphooligosaccharide--protein glycosyltransferase subunit DAD1 OS=Mus musculus GN=Dad1 PE=2 SV=3	1,07	0,754612448836087	0,930504185076609	26,6350593510396	26,3911397781081	26,1356881565921	26,1234980759642	26,5284979350479	26,7237904447108	288308164864,59	309615018001,085	−0,10286375726163	0,00946318224842201	0,011572431897656	1,07390305143284	0.102863757261631	0,931182753103984	-0.102863757261631	2646
Q61490	sp|Q61490|CD166_MOUSE CD166 antigen OS=Mus musculus GN=Alcam PE=1 SV=3	1,07	0,71906087532157	0,920149210230605	30,7847908092274	30,5173788922969	30,1107352298154	30,6061730946971	30,5018948562202	30,5191558658654	17114861754759,7	18382273858236	−0,103065597732497	0,0111389945760554	0,00182999955090598	1,07405330651437	0.103065597732501	0,93105248495096	-0.103065597732501	2647
Q8C7K6	sp|Q8C7K6|PCYXL_MOUSE Prenylcysteine oxidase-like OS=Mus musculus GN=Pcyox1l PE=2 SV=1	1,07	0,785729836683515	0,941085863507826	28,3076758523563	28,9750635875556	28,9217714421477	29,0558930771154	28,8395124734223	28,5243734839213	3015651354303,7	3239995353044,91	−0,103522098642672	0,0129073564261662	0,0092785899075302	1,07439321472658	0.103522098642672	0,930757925769747	-0.103522098642672	2648
Q920E5	sp|Q920E5|FPPS_MOUSE Farnesyl pyrophosphate synthase OS=Mus musculus GN=Fdps PE=1 SV=1	1,07	0,582080403483061	0,853379506269256	29,7814723531849	29,3922159985882	29,4203370103753	29,5724244467374	29,6772864366557	29,5601460600447	6688038761565,41	7186933113760,93	−0,103793050664554	0,00735067412204838	0,00217475672432399	1,07459501506818	0.103793050664553	0,93058313688209	-0.103793050664552	2649
Q8VE08	sp|Q8VE08|FBX33_MOUSE F-box only protein 33 OS=Mus musculus GN=Fbxo33 PE=1 SV=3	1,07	0,686192422574181	0,906996914986665	22,6937862021931	22,6774724465421	22,5849545096597	23,0507037791687	22,6688521928451	22,4525488290568	6881283679,41795	7394741183,82583	−0,103821934085874	0,00259111784983489	0,0133282478129091	1,07461652917226	0.103821934085874	0,930564506364206	-0.103821934085874	2650
Q3UQ84	sp|Q3UQ84|SYTM_MOUSE Threonine--tRNA ligase, mitochondrial OS=Mus musculus GN=Tars2 PE=2 SV=1	1,07	0,74613633196661	0,927700293161169	26,2847000887215	26,0883849331964	25,6579078890505	25,8813493021154	26,306343628981	26,0592651454691	197762167284,892	212523702201,148	−0,103857291145472	0,0123259957973338	0,00818305487111283	1,07464286581665	0.103857291145469	0,930541700698002	-0.103857291145469	2651
P84075	sp|P84075|HPCA_MOUSE Neuron-specific calcium-binding protein hippocalcin OS=Mus musculus GN=Hpca PE=1 SV=2;sp|P84074|HPCA_HUMAN Neuron-specific calcium-binding protein hippocalcin OS=Homo sapiens GN=HPCA PE=1 SV=2	1,07	0,689642719265917	0,907856236295823	32,4603983928778	32,2966368508442	32,2253332409013	32,077801964065	32,6339988306786	32,4866102924521	109555913621616	117736519994932	−0,10389453045067	0,00372825541194001	0,00889384286842468	1,07467060519955	0.103894530450665	0,930517681568403	-0.103894530450665	2652
Q8VE52	sp|Q8VE52|OGRL1_MOUSE Opioid growth factor receptor-like protein 1 OS=Mus musculus GN=Ogfrl1 PE=2 SV=2	1,07	0,72477100286961	0,921649709826669	25,1535971658607	25,7036960429525	25,6220992401053	25,4540678038503	25,4520364849735	25,789671987075	117903321503,944	126721646562,278	−0,104058624704376	0,0116447698786574	0,00760212178991397	1,07479284676504	0.104058624704374	0,930411849138969	-0.104058624704374	2653

Q8K0D0-2	sp|Q8K0D0-2|CDK17_MOUSE Isoform 2 of Cyclin-dependent kinase 17 OS=Mus musculus GN=Cdk17;sp|Q8K0D0|CDK17_MOUSE Cyclin-dependent kinase 17 OS=Mus musculus GN=Cdk17 PE=1 SV=2	1,07	0,794233588614506	0,943719708239887	25,5969041943702	26,3309093895965	26,3614633838226	26,3998169118976	26,102075650852	26,0039735970023	215542869247,635	231679793134,616	−0,104157384384863	0,016587250183398	0,0078774287943677	1,07486642422135	0.104157384384863	0,930348159981286	-0.104157384384863	2654
O35454	sp|O35454|CLCN6_MOUSE Chloride transport protein 6 OS=Mus musculus GN=Clcn6 PE=1 SV=1	1,07	0,924616769978391	0,983296707494544	21,3591905727963	22,8379813625276	23,7695776688911	22,3477845269731	22,5116389434821	23,3241799096996	6905494260,99817	7423138576,1076	−0,104284622382199	0,0536512896594235	0,0230061040894383	1,07496122587967	0.104284622382198	0,930266111860616	-0.104284622382198	2655
Q8VEH5	sp|Q8VEH5|EPMIP_MOUSE EPM2A-interacting protein 1 OS=Mus musculus GN=Epm2aip1 PE=2 SV=1	1,08	0,758959389777502	0,933136198610489	28,963339565092	29,6926508385983	29,5442070726591	29,5830171708225	29,5137526899067	29,3206469921288	5865247669022,8	6305682231227,96	−0,104460439091345	0,0131098333963552	0,0046134252024668	1,07509223600758	0.104460439091343	0,930152750161755	-0.104460439091343	2656
P13439	sp|P13439|UMPS_MOUSE Uridine 5-monophosphate synthase OS=Mus musculus GN=Umps PE=2 SV=3	1,08	0,770138191091504	0,936218563920244	26,0039735970023	25,5940945146071	25,500765153124	26,1438469895555	25,6889101757867	25,484150064461	144943679727,904	155872220615,755	−0,104871409317778	0,0104158971709373	0,0131017444187659	1,07539853347429	0.104871409317786	0,929887821930816	-0.104871409317786	2657
P04370-4	sp|P04370-4|MBP_MOUSE Isoform 4 of Myelin basic protein OS=Mus musculus GN=Mbp;sp|P04370-5|MBP_MOUSE Isoform 5 of Myelin basic protein OS=Mus musculus GN=Mbp;sp|P04370-10|MBP_MOUSE Isoform 10 of Myelin basic protein OS=Mus musculus GN=Mbp;sp|P04370-9|M	1,08	0,636069210899857	0,883588334123886	31,3069394690914	31,1726430469682	31,090420390194	30,9878465239167	31,3519919643443	31,4485046525447	35127343799746,6	37779247011804,5	−0,104999457871593	0,00350427114263875	0,0077709396125486	1,07549398631379	0.104999457871595	0,929805292010471	-0.104999457871595	2658
Q61285	sp|Q61285|ABCD2_MOUSE ATP-binding cassette sub-family D member 2 OS=Mus musculus GN=Abcd2 PE=2 SV=1	1,08	0,606921963128729	0,86602288294288	24,5863610385453	24,3979637610652	24,1280009506016	24,53802616775	24,4250582171058	24,3684748551525	38378960781,3371	41288633454,023	−0,105429022841911	0,00945338917763386	0,0035315251348137	1,07581426420751	0.10542902284191	0,929528482071804	-0.10542902284191	2659
Q8VC42	sp|Q8VC42|MIC1_MOUSE Uncharacterized protein C18orf8 homolog OS=Mus musculus GN=Mic1 PE=2 SV=1	1,08	0,730032869452729	0,924558927021746	24,8070307785967	25,502807308767	25,32730793297	25,2157838114411	25,31335724764	25,32730793297	89042712454,7446	95795638845,1884	−0,105462436582902	0,0143518996574306	0,0024030770539113	1,07583918104332	0.105462436582903	0,929506953846229	-0.105462436582903	2660
Q62092	sp|Q62092|NSG1_MOUSE Neuron-specific protein family member 1 OS=Mus musculus GN=Nsg1 PE=2 SV=3	1,08	0,682587655714775	0,904867222339183	23,1184157525276	22,9576116421202	23,0064630885348	22,8549362691204	23,43383578992	23,0135041204645	10016472530,2026	10777847708,4092	−0,105694578047487	0,00358005477168892	0,0129498248268289	1,0760123063195	0.105694578047496	0,929357400586339	-0.105694578047496	2661
Q9WUB3	sp|Q9WUB3|PYGM_MOUSE Glycogen phosphorylase, muscle form OS=Mus musculus GN=Pygm PE=1 SV=3	1,08	0,688962876036521	0,907856236295823	30,5414934586332	30,2664339291469	30,0134696913558	30,2998683979432	30,5414934586332	30,1999089850511	14052185151025,1	15120768022707,2	−0,105736928922758	0,0087233492159568	0,0057870488885298	1,07604389354378	0.105736928922756	0,929330119338027	-0.105736928922756	2662
Q91V93	sp|Q91V93|RHBT2_MOUSE Rho-related BTB domain-containing protein 2 OS=Mus musculus GN=Rhobtb2 PE=2 SV=2	1,08	0,965272062479343	0,991306002794532	25,4703197299504	21,8345749480041	25,2215409768175	25,549542709295	21,829564652429	25,3674906455573	31570170859,6502	33974157615,3861	−0,105875711385203	0,084014656535277	0,0864845734564534	1,07614741036478	0.105875711385206	0,929240725172618	-0.105875711385206	2663
Q3U0K8-3	sp|Q3U0K8-3|OGFD1_MOUSE Isoform 3 of Prolyl 3-hydroxylase OGFOD1 OS=Mus musculus GN=Ogfod1;sp|Q3U0K8|OGFD1_MOUSE Prolyl 3-hydroxylase OGFOD1 OS=Mus musculus GN=Ogfod1 PE=2 SV=1;sp|Q3U0K8-2|OGFD1_MOUSE Isoform 2 of Prolyl 3-hydroxylase OGFOD1 OS=Mus musc	1,08	0,86592358215053	0,968324005760349	25,3260413000039	26,1745346539103	26,0333327348677	26,4583956916079	26,0255942843937	25,270357393125	167564841979,013	180341081085,764	−0,106008596984491	0,0175909514995596	0,0231987306152511	1,07624653809151	0.106008596984498	0,929155137421656	-0.106008596984498	2664
P27773	sp|P27773|PDIA3_MOUSE Protein disulfide-isomerase A3 OS=Mus musculus GN=Pdia3 PE=1 SV=2	1,08	0,699050366802558	0,912827852392638	32,6186624488613	33,1377845162753	33,1187251555991	33,1921566422038	33,0034445863308	32,9002084952502	205895671399419	221609197431448	−0,106104258584225	0,00893149180107934	0,0044818698847022	1,07631790374819	0.106104258584225	0,92909352944663	-0.106104258584225	2665
Q99K74-3	sp|Q99K74-3|MED24_MOUSE Isoform 3 of Mediator of RNA polymerase II transcription subunit 24 OS=Mus musculus GN=Med24;sp|Q99K74-2|MED24_MOUSE Isoform 2 of Mediator of RNA polymerase II transcription subunit 24 OS=Mus musculus GN=Med24;sp|Q99K74|MED24_MOU	1,08	0,733114184158332	0,924701393764781	23,4411557402849	22,954648045036	23,3276794305009	23,5871603405659	23,0370888286754	23,3205769736667	12402464898,5079	13352133845,5257	−0,106443447747727	0,010951773481789	0,011798401431273	1,07657098446068	0.106443447747719	0,928875117789803	-0.106443447747719	2666
Q6ZQ38	sp|Q6ZQ38|CAND1_MOUSE Cullin-associated NEDD8-dissociated protein 1 OS=Mus musculus GN=Cand1 PE=2 SV=2	1,08	0,709609613911443	0,916805961322165	22,9673450736272	22,9874166300028	22,4569741763383	23,02646262069	22,6991159284757	22,9075582228068	8009642457,30171	8623115276,54905	−0,106471322980963	0,013183022898756	0,0072429309949963	1,07659178577791	0.106471322980963	0,928857170573179	-0.106471322980963	2667
Q9DC70	sp|Q9DC70|NDUS7_MOUSE NADH dehydrogenase [ubiquinone] iron-sulfur protein 7, mitochondrial OS=Mus musculus GN=Ndufs7 PE=1 SV=1	1,08	0,687901708352983	0,907856236295823	27,1009588259087	27,2761384034076	26,8591980973836	26,8756565229089	27,2799384547728	27,3023677084148	575649674662,209	619794760480,1	−0,106599466709476	0,007731092124543	0,0088444041102906	1,07668741556016	0.106599466709478	0,928774670854436	-0.106599466709478	2668
P61222	sp|P61222|ABCE1_MOUSE ATP-binding cassette sub-family E member 1 OS=Mus musculus GN=Abce1 PE=2 SV=1	1,08	0,671819506297129	0,900853394116002	29,2543000086044	29,7971159967527	29,714294747174	29,748150804332	29,6357657449474	29,6034944632424	7081948876822,98	7625128856613,75	−0,106615288550813	0,00988332267406695	0,0025599891537159	1,07669922351013	0.106615288550813	0,928764485164125	-0.106615288550813	2669
O54829	sp|O54829|RGS7_MOUSE Regulator of G-protein signaling 7 OS=Mus musculus GN=Rgs7 PE=1 SV=2	1,08	0,809595601650546	0,94879038498679	28,9121400873384	29,6323404854074	29,4348108568803	29,8286939050459	29,1006990223782	29,2720468450267	5448876063972,73	5867676963409,53	−0,106830770844922	0,0126892886215184	0,0129457721467591	1,0768600523337	0.106830770844922	0,928625774382534	-0.106830770844921	2670
O08553	sp|O08553|DPYL2_MOUSE Dihydropyrimidinase-related protein 2 OS=Mus musculus GN=Dpysl2 PE=1 SV=2	1,08	0,585705297109915	0,855898650321049	36,2026315719732	36,6007065050921	36,6007065050921	36,6007065050921	36,5127737376142	36,5127737376142	6884265641194220	7413541535882720	−0,106860132256255	0,00630219853402019	0,0013893024288621	1,07688196857504	0.106860132256254	0,928606875387867	-0.106860132256254	2671

P70345	sp|P70345|B2CL2_MOUSE Bcl-2-like protein 2 OS=Mus musculus GN=Bcl2l2 PE=2 SV=1;sp|P70345-2|B2CL2_MOUSE Isoform 2 of Bcl-2-like protein 2 OS=Mus musculus GN=Bcl2l2	1,08	0,83869635172559 9	0,959746519741951	23,6630592406412	24,1365945678446	23,9322127236138	23,4615901922734	23,8751848769123	24,6180184843432	24224301909,5926	26092954818,4478	−0,107205236098728	0,00993302557576431	0,0244308963503107	1,07713959790582	0.107205236098729	0,928384771987032	-0.107205236098729	2672
Q99LR1	sp|Q99LR1|ABD12_MOUSE Monoacylglycerol lipase ABHD12 OS=Mus musculus GN=Abhd12 PE=1 SV=2	1,08	0,434236160141801	0,777207385995124	29,1319348639858	29,0269449893171	29,0513152204935	29,1446312403025	29,3002269215392	28,9889107169626	4216662374797,29	4542913336570,2	−0,107516273252539	0,00189022381173358	0,0053408920302637	1,07737184834216	0.107516273252541	0,928184638886547	-0.107516273252541	2673
P27612	sp|P27612|PLAP_MOUSE Phospholipase A-2-activating protein OS=Mus musculus GN=Plaa PE=1 SV=4;sp|P54319|PLAP_RAT Phospholipase A-2-activating protein OS=Rattus norvegicus GN=Plaa PE=2 SV=3	1,08	0,648965227848524	0,889714023663571	29,8255701629118	29,5194704291796	29,2873491997899	29,5975087341519	29,714294747174	29,5442070726591	6774115567478,04	7298355645451,57	−0,107538854842283	0,00913739936858499	0,0029372024792010	1,07738871189183	0.107538854842284	0,928170110715248	-0.107538854842284	2674
O70172	sp|O70172|PI42A_MOUSE Phosphatidylinositol 5-phosphate 4-kinase type-2 alpha OS=Mus musculus GN=Pip4k2a PE=1 SV=1	1,08	0,500199310307312	0,812083935271593	29,531702480219	29,1870270397349	29,4521853631682	29,5350096320515	29,4546675630231	29,4054016596301	5808276279131,57	6258904494838,39	−0,107800083382778	0,00614072016563365	0,0022203261558367	1,07758381214163	0.107800083382776	0,928002062329207	-0.107800083382776	2675
Q9QXL8	sp|Q9QXL8|NDK7_MOUSE Nucleoside diphosphate kinase 7 OS=Mus musculus GN=Nme7 PE=2 SV=1	1,08	0,777228867133415	0,938755143139587	24,595883751326	24,7900819061277	24,1179472560925	24,3832569119608	24,9224630227272	24,4227555609276	43730179387,1314	47129195057,4158	−0,10799177450699	0,0141178158121863	0,0122296603020997	1,07772700039014	0.107991774506991	0,927878766735916	-0.107991774506991	2676
Q8BJF9	sp|Q8BJF9|CHM2B_MOUSE Charged multivesicular body protein 2b OS=Mus musculus GN=Chmp2b PE=1 SV=1	1,08	0,74661581205502 3	0,927700293161169	25,7260541994384	26,4376778659567	26,223070070365	26,0271456176258	26,3614633838226	26,223070070365	222664957358,856	239997183292,393	−0,108142946818142	0,0139704074003309	0,0064105695449767	1,07783993556563	0.108142946818139	0,927781544367457	-0.108142946818139	2677
Q9CWU6	sp|Q9CWU6|UQCC1_MOUSE Ubiquinol-cytochrome-c reductase complex assembly factor 1 OS=Mus musculus GN=Uqcc1 PE=2 SV=1	1,08	0,118015657097915	0,491443181254527	25,3552094720957	25,4094933692325	25,502807308767	25,5185024275634	25,4554077650705	25,5185024275634	109863374786,707	118416140678,007	−0,108155226099973	0,00293653378430681	0,0014286797524198	1,07784910947715	0.108155226099971	0,927773647728004	-0.108155226099971	2678
Q8VDH1-2	sp|Q8VDH1-2|FBX21_MOUSE Isoform 2 of F-box only protein 21 OS=Mus musculus GN=Fbxo21;sp|Q8VDH1|FBX21_MOUSE F-box only protein 21 OS=Mus musculus GN=Fbxo21 PE=2 SV=1	1,08	0,746466320582 75	0,927700293161169	25,5463841594127	25,4222737780244	25,0371457729701	25,5327595104657	25,0665939202135	25,6315175429121	100685527106,715	108529767217,088	−0,108234474820757	0,010480815681593	0,0118738476584411	1,07790831846229	0.108234474820758	0,927722685568076	-0.108234474820758	2679
Q9DB30	sp|Q9DB30|PHKG2_MOUSE Phosphorylase b kinase gamma catalytic chain, liver/testis isoform OS=Mus musculus GN=Phkg2 PE=2 SV=2	1,08	0,708901772702531	0,916805961322165	25,6255477551962	25,4532890416025	25,2526747161242	25,1876434359737	25,6171312267919	25,7523846556078	112232356518,018	120999603553,987	−0,108513656636964	0,00733443335195 38	0,0115548514775304	1,0781169290922	0.108513656636965	0,92754317552737	-0.108513656636965	2680
Q8C522	sp|Q8C522|ENDD1_MOUSE Endonuclease domain-containing 1 protein OS=Mus musculus GN=Endod1 PE=1 SV=2	1,08	0,809612797553851	0,94879038498679	25,914114289794	26,5473065949047	26,7386997761827	26,6511319794876	26,0981905549866	26,6767161700113	292006009736,937	314844982258,961	−0,108643613718385	0,0163472437096231	0,0123464599023603	1,07821404957589	0.108643613718387	0,927459626772013	-0.108643613718387	2681
O88741	sp|O88741|GDAP1_MOUSE Ganglioside-induced differentiation-associated protein 1 OS=Mus musculus GN=Gdap1 PE=1 SV=1	1,08	0,628647708941022	0,877439099287608	29,3362258860121	28,8811067240288	28,9475574493059	29,0726137546512	29,0597621446406	29,258547537147	4153462140563,36	4478493404449,01	−0,108699077402041	0,00846107044814391	0,0038188611601743	1,07825550176835	0.108699077402041	0,927423971739523	-0.108699077402041	2682
Q9Z1F9	sp|Q9Z1F9|SAE2_MOUSE SUMO-activating enzyme subunit 2 OS=Mus musculus GN=Uba2 PE=1 SV=1	1,08	0,5076626768761 9	0,816124555116335	29,8078729257526	29,8255701629118	29,4999946244056	29,8734429990539	29,7243921412505	29,763614849229	8005467841716,17	8637635536297,58	−0,109650726838455	0,00616187399837856	0,0025938139558989	1,07896698944779	0.109650726838454	0,926812413892103	-0.109650726838454	2683
P47757	sp|P47757|CAPZB_MOUSE F-actin-capping protein subunit beta OS=Mus musculus GN=Capzb PE=1 SV=3	1,08	0,874220891260987	0,972570741527848	24,2149220663738	24,9908094544841	25,6281918452435	25,5223313428076	24,7137513679622	24,8260555298346	68127114094,8862	73511871480,7856	−0,109748155900891	0,0283734347361906	0,0175066356354543	1,0790398574406	0.10974815590089	0,926749825879391	-0.10974815590089	2684
Q8R0S2	sp|Q8R0S2|IQEC1_MOUSE IQ motif and SEC7 domain-containing protein 1 OS=Mus musculus GN=Iqsec1 PE=1 SV=2;sp|Q8R0S2-2| IQEC1_MOUSE Isoform 2 of IQ motif and SEC7 domain-containing protein 1 OS=Mus musculus GN=Iqsec1	1,08	0,760241829138927	0,933136198610489	24,3182133554805	24,0031450361206	23,4691216538316	24,0352949440679	24,0367134446898	23,9469638537138	24702246185,8544	26657172300,4931	−0,109881519849914	0,0179365795987481	0,0021416172205967	1,07913960940759	0.109881519849912	0,926664160301708	-0.109881519849912	2685
P09405	sp|P09405|NUCL_MOUSE Nucleolin OS=Mus musculus GN=Ncl PE=1 SV=2	1,08	0,447206132631507	0,785601172266968	30,82620630466	30,7710464061561	30,577500263056	30,9261836153758	30,7768742173066	30,7005833571881	22062879640101,2	23812070453552,9	−0,110071964712946	0,00425102158769483	0,0037255896155717	1,07928207205883	0.110071964712943	0,926541842849675	-0.110071964712943	2686
Q9DBG6	sp|Q9DBG6|RPN2_MOUSE Dolichyl-diphosphooligosaccharide--protein glycosyltransferase subunit 2 OS=Mus musculus GN=Rpn2 PE=2 SV=1	1,08	0,780327177816041	0,940251150142017	26,6595208620373	27,0900402227433	26,9530356848158	27,3198293289793	27,10274244371 3	26,5089478029815	481834046510,2	520040844156,096	−0,110088599024806	0,00817631822963828	0,0155602402057302	1,07929451628132	0.110088599024807	0,926531159859382	-0.110088599024807	2687
P50544	sp|P50544|ACADV_MOUSE Very long-chain specific acyl-CoA dehydrogenase, mitochondrial OS=Mus musculus GN=Acadvl PE=1 SV=3	1,08	0,718896539456014	0,920149210230605	29,6690585737809	30,3514194051073	30,1415815593921	30,2640745692772	30,0740303655199	30,0535494719522	11279633226554,2	12176773832140,5	−0,110411792717934	0,0116292559372318	0,0038527987924408	1,07953632778363	0.110411792717939	0,926323620857743	-0.11041179271794	2688

Q3UVL4	sp|Q3UVL4|VPS51_MOUSE Vacuolar protein sorting-associated protein 51 homolog OS=Mus musculus GN=Vps51 PE=2 SV=2;sp|Q3UVL4-2| VPS51_MOUSE Isoform 2 of Vacuolar protein sorting-associated protein 51 homolog OS=Mus musculus GN=Vps51	1,08	0,677776458695285	0,903237623610658	30,2888262852398	29,9980024503029	29,6607221554744	30,0424084078288	30,1229682803372	30,0121195493054	10501266277442,8	11337822909304	−0,110580337001691	0,0104840521919241	0,0019059990667107	1,07966245305656	0.11058033700169	0,9262154085001	-0.11058033700169	2689
Q9D0F9	sp|Q9D0F9|PGM1_MOUSE Phosphoglucomutase-1 OS=Mus musculus GN=Pgm1 PE=1 SV=4	1,08	0,612567076932161	0,868059454773913	31,6995393111186	32,1886810722787	32,043087513796	32,1359937740807	31,9827023770707	32,043087513796	77175458890166,2	83338178740290,2	−0,110835415727979	0,00785431245457693	0,0024090186679375	1,07985336192033	0.11083541572798	0,926051661516037	-0.11083541572798	2690
Q8CIB6	sp|Q8CIB6|TM230_MOUSE Transmembrane protein 230 OS=Mus musculus GN=Tmem230 PE=1 SV=1	1,08	0,780071528077974	0,940251150142017	24,5565050782037	24,275977743973	24,1307029648063	23,9154891156968	24,5625402578922	24,7157174558305	36517664121,155	39434843286,0208	−0,110876424181767	0,00889975966714608	0,0174095029114814	1,07988405707406	0.110876424181769	0,926025338969715	-0.110876424181769	2691
Q5SP85-3	sp|Q5SP85-3|CC85A_MOUSE Isoform 3 of Coiled-coil domain-containing protein 85A OS=Mus musculus GN=Ccdc85a;sp|Q5SP85-2| CC85A_MOUSE Isoform 2 of Coiled-coil domain-containing protein 85A OS=Mus musculus GN=Ccdc85a;sp|Q5SP85|CC85A_MOUSE Coiled-coil domain-	1,08	0,871266881883508	0,971009960968743	22,4108797662297	22,484003771526	21,1880773735381	22,383010120132	22,2659402972637	21,665177458907	3685432181,91152	3980643362,70923	−0,1116781134527	0,0330499592007912	0,0174225373979076	1,08010218781033	0.111167811345269	0,925838324637857	-0.111167811345269	2692
P61161	sp|P61161|ARP2_MOUSE Actin-related protein 2 OS=Mus musculus GN=Actr2 PE=1 SV=1	1,08	0,628398476169991	0,877439099287608	32,5562887960631	32,0900263529684	32,574532753597	32,4938038454955	32,4536789640953	32,504668068945	118620365176629	128127924745836	−0,111233218761384	0,00847395005266705	0,0008267879292438	1,08015115747663	0.111233218761383	0,925796350888638	-0.111233218761383	2693
P28184	sp|P28184|MT3_MOUSE Metallothionein-3 OS=Mus musculus GN=Mt3 PE=1 SV=1	1,08	0,540746354144371	0,832298305005528	24,2257383334506	24,4288410238104	24,5047987626687	24,4516913640593	24,305821491108	24,6333618185744	38985646361,1387	42113108343,3826	−0,111326310055836	0,00591627234321828	0,0067077519360571	1,08022085752467	0.111326310055837	0,925736614909943	-0.111326310055837	2694
Q8VDP2	sp|Q8VDP2|CX056_MOUSE UPF0428 protein CXorf56 homolog OS=Mus musculus PE=2 SV=1;sp|Q8VDP2-2|CX056_MOUSE Isoform 2 of UPF0428 protein CXorf56 homolog OS=Mus musculus	1,08	0,656644306380486	0,892226633215307	22,7898514546523	22,9990398942442	23,199871575543	23,2759764901913	22,8392616668271	23,1051187112	9708370690,90065	10487524967,2684	−0,111373144729851	0,00891554992822108	0,0095379218847141	1,08025592565167	0.111373144729848	0,925706562911698	-0.111373144729848	2695
P13707	sp|P13707|GPDA_MOUSE Glycerol-3-phosphate dehydrogenase [NAD(+)], cytoplasmic OS=Mus musculus GN=Gpd1 PE=1 SV=3	1,08	0,453303173512296	0,788322280554728	31,0631349310244	30,727262227369	30,8315955900058	30,9382317000035	30,9084445981046	31,0083247513143	25609835242685,8	27678222165053	−0,112053306790764	0,00556792918141707	0,0016566990684659	1,08076533498816	0.112053306790762	0,925270239178195	-0.112053306790762	2696
P14211	sp|P14211|CALR_MOUSE Calreticulin OS=Mus musculus GN=Calr PE=1 SV=1	1,08	0,756464273455334	0,931775125431143	32,1651705876441	32,9002084952502	32,8872840950895	32,8500916619242	32,6786103940251	32,6569761311727	151391108397205	163618627838241	−0,112056532712098	0,0128845812631313	0,0032327862911900	1,08076775162353	0.112056532712101	0,925268170240833	-0.112056532712101	2697
Q9DBL7	sp|Q9DBL7|COASY_MOUSE Bifunctional coenzyme A synthase OS=Mus musculus GN=Coasy PE=1 SV=2	1,08	0,667726088138836	0,898352728146089	27,9775310134962	28,5454840142889	28,485463756269	28,3794989601019	28,4095407476388	28,4530620211488	2024126964099,86	2188054501880,49	−0,112348887983935	0,0110116533645554	0,0013017083913530	1,08098678624813	0.112348887983933	0,925080688054276	-0.112348887983933	2698
Q99MP8-2	sp|Q99MP8-2|BRAP_MOUSE Isoform 2 of BRCA1-associated protein OS=Mus musculus GN=Brap;sp|Q99MP8|BRAP_MOUSE BRCA1-associated protein OS=Mus musculus GN=Brap PE=2 SV=1;sp|Q99MP8-3|BRAP_MOUSE Isoform 3 of BRCA1-associated protein OS=Mus musculus GN=Brap	1,08	0,851220429494477	0,962641439679318	24,0874845308129	24,511216945845	23,9835441013259	24,9622562481828	23,6408917707753	24,2127444651752	32162977448,577	34768031323,1059	−0,112360410940285	0,0115532733422754	0,0273018609061083	1,08099542023726	0.112360410940284	0,925073299367467	-0.112360410940284	2699
Q99JP7	sp|Q99JP7|GGT7_MOUSE Gamma-glutamyltransferase 7 OS=Mus musculus GN=Ggt7 PE=2 SV=2	1,08	0,696768577578745	0,911421490959632	26,7858549456868	26,7334809843027	26,2102331083515	26,7237904447108	26,7440322826555	26,4958547362392	348367227851,537	376641315800,481	−0,112582354719774	0,0119765639144768	0,0051703798217027	1,08116173304624	0.112582354719773	0,924930997310126	-0.112582354719773	2700
P17156	sp|P17156|HSP72_MOUSE Heat shock-related 70 kDa protein 2 OS=Mus musculus GN=Hspa2 PE=1 SV=2	1,08	0,67458611089039	0,901104029547498	30,0523063560686	30,5297954510822	30,6232978272985	30,5147582056784	30,3642246304452	30,5614058829116	15971066831540,6	17272375909665	−0,113005862331711	0,0100737337239301	0,0033809175213871	1,08147915802058	0.113005862331717	0,924659520790291	-0.113005862331716	2701
Q7TSV4	sp|Q7TSV4|PGM2_MOUSE Phosphoglucomutase-2 OS=Mus musculus GN=Pgm2 PE=1 SV=1	1,08	0,689127148872306	0,907856236295823	26,4947769969841	27,0337631233805	26,7315571743892	27,0337631233805	26,8380058322934	26,6234109353197	415756573017,271	449646084026,119	−0,113050831931451	0,0100979470535901	0,0076494572036249	1,08151286884751	0.113050831931451	0,92463069909249	-0.11305083193145	2702
Q62193	sp|Q62193|RFA2_MOUSE Replication protein A 32 kDa subunit OS=Mus musculus GN=Rpa2 PE=1 SV=1	1,08	0,688570403046216	0,907856236295823	24,3497113967141	24,6690327146511	24,9762304365785	24,8891462278101	24,6395384667734	24,7017312592705	51507409908,3284	55712589663,7092	−0,113223382908904	0,0127013653804675	0,0052512250365317	1,0816422290087	0.113223382908903	0,924520116893435	-0.113223382908903	2703
Q80SW1	sp|Q80SW1|SAHH2_MOUSE Putative adenosylhomocysteinase 2 OS=Mus musculus GN=Ahcyl1 PE=1 SV=1	1,08	0,654449329997901	0,891901943526937	31,5630720591258	32,0149961289679	32,0014621620972	32,1137938104902	31,9095496846577	31,7920975405357	68636353868342,6	74251592799610,8	−0,113449058684335	0,0080697393962192	0,0050969167532069	1,08181143978072	0.113449058684334	0,924375508732553	-0.113449058684334	2704
Q9QXG2	sp|Q9QXG2|RAE1_MOUSE Rab proteins geranylgeranyltransferase component A 1 OS=Mus musculus GN=Chm PE=2 SV=1	1,08	0,62304139225947	0,873726316984964	28,2304457403943	27,889164479335	27,6943403199442	28,0782614029079	27,9628273575294	28,0091322344659	1359289854136,85	1470671671328,45	−0,113622071356212	0,0097131709902239	0,0020734705032277	1,08194118189923	0.113622071356217	0,924264661268011	-0.113622071356217	2705
Q9WUB0	sp|Q9WUB0|HOIL1_MOUSE RanBP-type and C3HC4-type zinc finger-containing protein 1 OS=Mus musculus GN=Rbck1 PE=1 SV=2	1,08	0,825829313194281	0,955744170441451	22,5448185137444	22,8419647179408	22,78470180127	23,3145151636562	22,9522447247002	22,1414207633023	7393213666,67993	8000156043,60195	−0,113826531767943	0,00693759942885807	0,0263420197324728	1,08209452672218	0.113826531767943	0,924133682691425	-0.113826531767943	2706

Q91WM1-2	sp|Q91WM1-2|STRBP_MOUSE Isoform 2 of Spermatid perinuclear RNA-binding protein OS=Mus musculus GN=Strbp;sp|Q91WM1|STRBP_MOUSE Spermatid perinuclear RNA-binding protein OS=Mus musculus GN=Strbp PE=1 SV=1	1,08	0,684086397947119	0,905531688621164	24,9975459496661	25,0225487947601	24,6585297673407	25,149119368486	24,6613896974429	25,1063810759423	64690153331,8688	70037504490,7531	−0,114581547655286	0,00816833963657058	0,0108159862823963	1,08266097517889	0.114581547655285	0,923650175748476	-0.114581547655285	2707
P24638	sp|P24638|PPAL_MOUSE Lysosomal acid phosphatase OS=Mus musculus GN=Acp2 PE=2 SV=2	1,08	0,733251412337527	0,924701393764781	24,6380315993696	25,1651628272617	25,3260413000039	25,3163007018966	25,0980650300174	24,9531426386628	75174549682,3557	81388741549,972	−0,114584920598002	0,0143706676941827	0,0072766725674531	1,08266350638446	0.114584920598003	0,923648016307012	-0.114584920598003	2708
P57746	sp|P57746|VATD_MOUSE V-type proton ATPase subunit D OS=Mus musculus GN=Atp6v1d PE=1 SV=1	1,08	0,691536149887753	0,909293912033752	31,471637043656	31,6337004171238	32,1137938104902	31,8619322988203	31,7107286381385	31,8850101738384	60866887511745,3	65904227367664,5	−0,114713414513506	0,0105213934954129	0,0029750997944182	1,08275993831535	0.114713414513506	0,923565755079457	-0.114713414513506	2709
Q8BK63	sp|Q8BK63|KC1A_MOUSE Casein kinase I isoform alpha OS=Mus musculus GN=Csnk1a1 PE=1 SV=2;sp|Q8BK63-2|KC1A_MOUSE Isoform 2 of Casein kinase I isoform alpha OS=Mus musculus GN=Csnk1a1	1,08	0,200459049361075	0,6308537814738	27,1060915962256	26,9547500236637	27,0965384478712	27,1876699138534	27,0584803792591	27,1500360627144	560704587952,571	607162388513,784	−0,114841549175497	0,00313295158705175	0,0024489040972332	1,08285610918729	0.114841549175495	0,923483731139979	-0.114841549175495	2710
Q68FD5	sp|Q68FD5|CLH1_MOUSE Clathrin heavy chain 1 OS=Mus musculus GN=Cltc PE=1 SV=3	1,08	0,572171156308144	0,848720548523746	28,1288847943823	27,8935880061131	27,6870999533187	27,9562316171464	27,8950448565735	28,0971533682127	1312809900145,35	1421608292961,96	−0,114865978836619	0,00792199418776583	0,0037037918205579	1,08287444572483	0.114865978836618	0,923468093598465	-0.114865978836618	2711
P46096	sp|P46096|SYT1_MOUSE Synaptotagmin-1 OS=Mus musculus GN=Syt1 PE=1 SV=1	1,08	0,626614053298023	0,876307593263434	33,7374582476704	34,2285718437873	34,1536747954851	34,2388631383902	34,0600793369985	34,0600793369985	607213532799573	657636812026247	−0,115087113846445	0,00777285468755264	0,0030252597386896	1,08304044047602	0.115087113846446	0,923326556079921	-0.115087113846446	2712
Q9DD02	sp|Q9DD02|HIKES_MOUSE Protein Hikeshi OS=Mus musculus GN=L7rn6 PE=2 SV=1;sp|Q9DD02-2|HIKES_MOUSE Isoform 2 of Protein Hikeshi OS=Mus musculus GN=L7rn6	1,08	0,788467600571983	0,942051708080081	25,8919891587445	26,4815901695906	25,9650637893661	26,4086406469355	25,7281474875069	26,4412629368967	219119052819,145	237321996370,222	−0,115130889154229	0,0123079131653378	0,0153718758666073	1,08307330337952	0.115130889154227	0,923298540255491	-0.115130889154227	2713
P56376	sp|P56376|ACYP1_MOUSE Acylphosphatase-1 OS=Mus musculus GN=Acyp1 PE=2 SV=2	1,08	0,608487471362282	0,866608542040832	29,1488133739494	29,2283500040983	29,0254710300426	28,9794072842181	29,1855954237581	29,4774744514319	4496010074843,72	4870214285264,41	−0,115339982639735	0,00350874986752159	0,0085663646231344	1,08323028734176	0.115339982639734	0,923164733931133	-0.115339982639734	2714
Q8K268	sp|Q8K268|ABCF3_MOUSE ATP-binding cassette sub-family F member 3 OS=Mus musculus GN=Abcf3 PE=1 SV=1	1,08	0,493096718941468	0,808293971569275	27,5796655021705	27,4696095384182	27,2152024674969	27,5855513418751	27,4336352949716	27,4854015248497	810966434729,286	878541855072,607	−0,115468816409653	0,00681710462269938	0,0028082614598751	1,08332702495373	0.115468816409652	0,923082298295582	-0.115468816409652	2715
Q6PIP5	sp|Q6PIP5|NUDC1_MOUSE NudC domain-containing protein 1 OS=Mus musculus GN=Nudcd1 PE=2 SV=2	1,08	0,616144968848829	0,869694684228085	26,8512780500818	27,3782962363329	27,0989232001278	27,1320858547966	27,1500360627144	27,2868283286901	593616403401,737	643154030373,793	−0,115633334642572	0,00972604542454448	0,0031128039965998	1,08345056957351	0.115633334642571	0,922977040284945	-0.115633334642571	2716
Q80VL1	sp|Q80VL1|TDRKH_MOUSE Tudor and KH domain-containing protein OS=Mus musculus GN=Tdrkh PE=1 SV=1	1,08	0,780822701638659	0,940251150142017	24,1261151702096	25,0665939202135	24,7428706679691	24,656098949068	24,8955478776018	24,6249971441151	50497681071,5134	54722893670,1596	−0,115927381251569	0,0193866333302483	0,0059874605488591	1,08367141835013	0.11592738125157	0,922788940509734	-0.11592738125157	2717
Q8BLF1	sp|Q8BLF1|NCEH1_MOUSE Neutral cholesterol ester hydrolase 1 OS=Mus musculus GN=Nceh1 PE=1 SV=1	1,08	0,811966344042706	0,949948723398244	30,8315955900058	30,447826850692	30,0419786001201	30,1644509291729	31,0083247513143	30,3912180432188	16600717173919,6	17998890647577,8	−0,116662420186198	0,0129715467544287	0,014308811852827	1,08422367895375	0.116662420186195	0,922318908368591	-0.116662420186195	2718
P63046	sp|P63046|ST4A1_MOUSE Sulfotransferase 4A1 OS=Mus musculus GN=Sult4a1 PE=2 SV=1;sp|P63046-2|ST4A1_MOUSE Isoform 2 of Sulfotransferase 4A1 OS=Mus musculus GN=Sult4a1	1,08	0,43992585248764	0,780009157984922	29,450672574706	29,4291202576836	29,6534011397899	29,7453450924431	29,5056687085734	29,5252640648693	6553790217603,65	7106938113854,26	−0,11689864265672	0,00419290687293524	0,0044972041832647	1,08440122095529	0.11689864265672	0,922167903056268	-0.11689864265672	2719
Q91YR1	sp|Q91YR1|TWF1_MOUSE Twinfilin-1 OS=Mus musculus GN=Twf1 PE=1 SV=2	1,08	0,623100261749356	0,873726316984964	29,5295487853568	29,8046256258292	30,0920533297203	29,9308608554239	29,8297582429479	29,9092926391212	8826172581109,54	9573027065509	−0,117187231139871	0,00943598574878287	0,0017814958898746	1,08461816008424	0.117187231139871	0,921983456299802	-0.117187231139871	2720
Q3TYX3	sp|Q3TYX3|SMYD5_MOUSE SET and MYND domain-containing protein 5 OS=Mus musculus GN=Smyd5 PE=2 SV=2	1,08	0,646883710112849	0,889714023663571	25,6922263661063	25,2075881976355	25,773705472882	25,6338439500692	25,668783905484	25,6146994185905	125785009432,418	136434310145,548	−0,117246497500922	0,011974822059528	0,0010696148391602	1,08466271745086	0.117246497500927	0,921945581710573	-0.117246497500927	2721
	sp|Q9R1T4-2|SEPT6_MOUSE Isoform I of Septin-6 OS=Mus musculus GN=Sept6;sp|Q9R1T4-3|SEPT6_MOUSE Isoform V of Septin-6 OS=Mus musculus GN=Sept6;sp|Q9R1T4|SEPT6_MOUSE Septin-6 OS=Mus musculus GN=Sept6 PE=1 SV=4;sp|Q14141-2|SEPT6_HUMAN Isoform I of																			

Q9R1T4-2	[illegible] Septin-	1,09	0,48274649798925	0,802890545076804	31,2859805099124	30,9917314842313	31,2366860347337	31,1804486408323	31,3988833526896	31,1804486408323	34482256717500,9	37421263869752	−0,118004089347205	0,0050557126572311	0,00403520589013551	1,08523244798997	0.118004089347205	0,921461574294215	-0.118004089347205	2722
P60605	sp|P60605|UB2G2_MOUSE Ubiquitin-conjugating enzyme E2 G2 OS=Mus musculus GN=Ube2g2 PE=1 SV=1	1,09	0,938379088471227	0,985646114383563	23,5141970803436	23,29481600134	20,7897257103873	23,0064630885348	23,4012908958398	21,4368240413319	6108291397,5655	6629925086,44863	−0,118223681074018	0,0671739257271362	0,0459491300487298	1,08539764312669	0.118223681074017	0,9213213298 6701	-0.118223681074017	2723
Q922Q4	sp|Q922Q4|P5CR2_MOUSE Pyrroline-5-carboxylate reductase 2 OS=Mus musculus GN=Pycr2 PE=2 SV=1	1,09	0,621838003658867	0,873035168143395	27,6736691686645	27,966093558593	28,1132288221562	28,1946741447067	27,8355439715928	27,9689280949635	1331948268466,03	1445845523525	−0,118375369980573	0,00801449399066397	0,00648341526440353	1,08551177080634	0.118375369980569	0,921224464712325	-0.118375369980569	2724
Q68FL6	sp|Q68FL6|SYMC_MOUSE Methionine--tRNA ligase, cytoplasmic OS=Mus musculus GN=Mars PE=2 SV=1	1,09	0,73438266203408	0,924701393764781	30,1282886884742	29,4570920219591	29,5295487853568	29,8297582429479	29,9631043655379	29,5685996112172	7956230899808,18	8637670749618,92	−0,118557179402011	0,0124014525811665	0,00673691274853666	1,08564857636638	0.118557179402012	0,921108378686372	-0.118557179402012	2725
Q91ZX7	sp|Q91ZX7|LRP1_MOUSE Prolow-density lipoprotein receptor-related protein 1 OS=Mus musculus GN=Lrp1 PE=1 SV=1	1,09	0,644686625977292	0,889432399790556	26,9028762084041	26,6793021818755	26,3276898796021	26,596578656695	26,7786940840882	26,7811512810363	369945952143,653	401634379214,254	−0,118568253541112	0,0108857111061101	0,00396203499519600	1,08565690984584	0.118568253541112	0,921101308277953	-0.118568253541112	2726
Q8BIW1	sp|Q8BIW1|PRUNE_MOUSE Protein prune homolog OS=Mus musculus GN=Prune PE=2 SV=1	1,09	0,63436121876268	0,882745957862852	29,089228896999	29,6117382906239	29,531702480219	29,6044346971218	29,4746082875131	29,4005511866025	5929078314465,59	6437736100676,76	−0,118745585507626	0,00956877977029328	0,0034992870205507	1,08579036390364	0.118745585507627	0,920988096085874	-0.118745585507627	2727
Q02956	sp|Q02956|KPCZ_MOUSE Protein kinase C zeta type OS=Mus musculus GN=Prkcz PE=1 SV=2	1,09	0,886541326912955	0,975149870380105	23,0613359467876	23,160204023241	24,4250582171058	24,3639618883844	23,1309610408383	23,3986660676142	16871068391,9319	18318848369,3044	−0,118777472100941	0,032290862902398	0,0274449281727565	1,0858143624185	0.118777472100945	0,920967740537749	-0.118777472100945	2728
Q9CPY7	sp|Q9CPY7|AMPL_MOUSE Cytosol aminopeptidase OS=Mus musculus GN=Lap3 PE=1 SV=3;sp|Q9CPY7-2|AMPL_MOUSE Isoform 2 of Cytosol aminopeptidase OS=Mus musculus GN=Lap3	1,09	0,775110732493673	0,938619589104496	29,7364644042824	30,6114251490149	30,5191558658654	30,5297954510822	30,31031921555	30,2744604699913	14267639827373,3	15494791413492,5	−0,119036631951135	0,0158718188631715	0,00455143005714800	1,08600943120002	0.119036631951137	0,920802316509364	-0.119036631951137	2729
Q9D8S3	sp|Q9D8S3|ARFG3_MOUSE ADP-ribosylation factor GTPase-activating protein 3 OS=Mus musculus GN=Arfgap3 PE=1 SV=2	1,09	0,687105851931244	0,907675629195552	24,6012207533644	24,8499747977413	25,0588842328911	25,1215085600284	25,0309060428071	24,6071514587832	61155966222,787	66459285824,6814	−0,119977538498315	0,00922508607440261	0,0110182177327332	1,08671794314515	0.119977538498313	0,920201977254398	-0.119977538498313	2730
O88983	sp|O88983|STX8_MOUSE Syntaxin-8 OS=Mus musculus GN=Stx8 PE=1 SV=1	1,09	0,812029311915662	0,949948723398244	24,1529970070456	24,7655337624833	24,8783295174502	25,1499790121567	24,6730051571631	24,2234160190066	48215839723,8162	52397954754,4894	−0,12000332605919	0,0158667618958298	0,0187726454139572	1,08673736794026	0.12000332605919	0,920185529182036	-0.12000332605919	2731
P97411	sp|P97411|ICA69_MOUSE Islet cell autoantigen 1 OS=Mus musculus GN=Ica1 PE=2 SV=3;sp|P97411-2|ICA69_MOUSE Isoform 2 of Islet cell autoantigen 1 OS=Mus musculus GN=Ica1	1,09	0,655429364876326	0,892226633215307	27,2212697752775	27,7483704429287	27,6529615702981	27,8064691473181	27,5713979478984	27,4953994502371	913790584947,032	993422791041,12	−0,120544267258799	0,0101979546300276	0,00587060338621321	1,08714491854685	0.120544267258798	0,919840568575407	-0.120544267258798	2732
Q9EQ06	sp|Q9EQ06|DHB11_MOUSE Estradiol 17-beta-dehydrogenase 11 OS=Mus musculus GN=Hsd17b11 PE=2 SV=1;sp|Q9EQ06-2|DHB11_MOUSE Isoform 2 of Estradiol 17-beta-dehydrogenase 11 OS=Mus musculus GN=Hsd17b11	1,09	0,55980016591778	0,842973036472956	27,7105926216416	27,964154597457	28,1401525448235	28,0674871197023	28,1148719832427	27,8832358468707	1359720012482,91	1478227695777,25	−0,120558900487773	0,00772931526866078	0,00436701155234216	1,08715594549347	0.120558900487774	0,919831238696939	-0.120558900487774	2733
P41216	sp|P41216|ACSL1_MOUSE Long-chain-fatty-acid--CoA ligase 1 OS=Mus musculus GN=Acsl1 PE=1 SV=2	1,09	0,585872902962657	0,855898650321049	29,4785745078136	29,4026825687162	29,0196626152411	29,4859248540224	29,2839283038169	29,3820077371923	5308373332863,76	5771502906965,68	−0,120677209832954	0,0083954465756504	0,00343766990778763	1,08724510223023	0.120677209832954	0,919755810303246	-0.120677209832953	2734
O70591	sp|O70591|PFD2_MOUSE Prefoldin subunit 2 OS=Mus musculus GN=Pfdn2 PE=2 SV=2	1,09	0,707272128723719	0,916805961322165	26,5695977401837	26,5448095234348	27,0782792312648	26,5771116775476	26,8032741998557	27,064290140851	406518590629,505	442139820991,429	−0,121181345241077	0,0112640571683156	0,00909184834734694	1,0876250955873	0.121181345241074	0,919434466947472	-0.121181345241074	2735
P07356	sp|P07356|ANXA2_MOUSE Annexin A2 OS=Mus musculus GN=Anxa2 PE=1 SV=2;sp|P07355|ANXA2_HUMAN Annexin A2 OS=Homo sapiens GN=ANXA2 PE=1 SV=2;sp|P07355-2|ANXA2_HUMAN Isoform 2 of Annexin A2 OS=Homo sapiens GN=ANXA2	1,09	0,509858802159199	0,816791532413287	29,6715275253764	29,2543000086044	29,5228299333287	29,4999946244056	29,5724244467374	29,6285973016842	6371689783291,61	6930863036092,58	−0,12135898050486	0,00717237815020036	0,00218055115775060	1,08775902026293	0.121358980504858	0,919321266357585	-0.121358980504858	2736
Q8BMP6	sp|Q8BMP6|GCP60_MOUSE Golgi resident protein GCP60 OS=Mus musculus GN=Acbd3 PE=1 SV=3	1,09	0,355563245785119	0,732843629012038	27,8005133029123	27,4953994502371	27,5796655021705	27,7178014437035	27,6931390764562	27,7178014437035	994188587809,032	1081727757901,4	−0,121746008949522	0,00570383537568084	0,00051385925130359	1,08805086999166	0.121746008949525	0,919074675256371	-0.121746008949525	2737
Q9JKF1	sp|Q9JKF1|IQGA1_MOUSE Ras GTPase-activating-like protein IQGAP1 OS=Mus musculus GN=Iqgap1 PE=1 SV=2	1,09	0,575540592370013	0,849998193752035	24,0459906620689	23,7647352552483	23,635106284079	24,040363892071	23,732793378787	23,9261106778015	22021330757,792	23962500900,2316	−0,121876831920315	0,0088201057656318 4	0,00650507545737734	1,08814953845388	0.121876831920313	0,918991337735502	-0.121876831920313	2738
Q8BZJ7	sp|Q8BZJ7|DCNL2_MOUSE DCN1-like protein 2 OS=Mus musculus GN=Dcun1d2 PE=2 SV=3;sp|Q8BZJ7-2|DCNL2_MOUSE Isoform 2 of DCN1-like protein 2 OS=Mus musculus GN=Dcun1d2;sp|Q8BZJ7-4|DCNL2_MOUSE Isoform 3 of DCN1-like protein 2 OS=Mus musculus GN=Dcun1d2	1,09	0,60544809350802	0,865294279541889	25,9701667726243	26,2965204560717	26,3346641672483	26,4055239012705	26,0524841852733	26,3976388083925	239172399417,514	260329931717,199	−0,122290285105369	0,00764648622914194	0,0076693265155611	1,08846142929206	0.122290285105369	0,918728007340052	-0.122290285105369	2739
O55135	sp|O55135|IF6_MOUSE Eukaryotic translation initiation factor 6 OS=Mus musculus GN=Eif6 PE=1 SV=2	1,09	0,634951390501546	0,882835066816268	26,7611411553313	26,9524248585844	27,311293764426	27,0965384478712	26,9578668145221	27,2250623653521	536475425516,897	583993608821,03	−0,122440493902076	0,0103411965801431	0,00493223781362734	1,08857476231712	0.122440493902076	0,918632357295719	-0.122440493902076	2740

Q8C0V0	sp|Q8C0V0|TLK1_MOUSE Serine/threonine-protein kinase tousled-like 1 OS=Mus musculus GN=Tlk1 PE=2 SV=2;sp|O55047-3|TLK2_MOUSE Isoform 3 of Serine/threonine-protein kinase tousled-like 2 OS=Mus musculus GN=Tlk2;sp|O55047-2|TLK2_MOUSE Isoform 2 of Serine/t	1,09	0,84146736128853 8	0,959771286845933	22,5407241994077	21,1647787321785	21,4618051524005	21,6547649475765	21,8534467936578	21,9143063520067	2716027109,48927	2957192073,0382	−0,12273007157877	0,033332037489552	0,0062241249827939	1,08879328291914	0.122730071578773	0,918447987958672	-0.122730071578773	2741
Q9JHK4	sp|Q9JHK4|PGTA_MOUSE Geranylgeranyl transferase type-2 subunit alpha OS=Mus musculus GN=Rabggta PE=2 SV=1	1,09	0,6683211269972 5	0,898352728146089	29,4911659736167	29,9281463515691	30,1038443023282	30,0547925878358	29,7799328650923	29,9440608870272	9115999834629,75	9926828063688,31	−0,12293190614771 6	0,0105722838924176	0,0046210779738284	1,0894561691175	0.122931906147716	0,918319505097049	-0.122931906147716	2742
P06801	sp|P06801|MAOX_MOUSE NADP-dependent malic enzyme OS=Mus musculus GN=Me1 PE=1 SV=2	1,09	0,670269752872636	0,900104854523341	30,1964189422983	30,7847908092274	30,7034268677074	30,7354901350717	30,4899709322161	30,7148429894456	18737454287809,3	20404325301971,7	−0,12295004806614 7	0,0104319051821591	0,0044436047585755	1,0895931051033	0.122950048066143	0,918307957283874	-0.122950048066143	2743
Q8BH86	sp|Q8BH86|CN159_MOUSE UPF0317 protein C14orf159 homolog, mitochondrial OS=Mus musculus PE=1 SV=1;sp|Q8BH86-2|CN159_MOUSE Isoform 2 of UPF0317 protein C14orf159 homolog, mitochondrial OS=Mus musculus	1,09	0,745156230042388	0,927664324783982	25,7383458751693	26,5038887636353	26,4229344891842	26,4513611202461	26,1166289768567	26,3529925413592	244314714009,807	266061737009,914	−0,12302029431744 6	0,0160389640323146	0,0065397637025464	1,08901233430924	0.123020294317445	0,918263245047909	-0.123020294317445	2744
Q80UM7	sp|Q80UM7|MOGS_MOUSE Mannosyl-oligosaccharide glucosidase OS=Mus musculus GN=Mogs PE=2 SV=1	1,09	0,582343643029776	0,853489762903505	27,0716328405969	26,7527581374521	26,584740539218	26,8968700970283	26,9898009594195	26,7786940840882	436932185941,811	475891177739,012	−0,12322232586641 5	0,00922695831310279	0,0039349496347547	1,0916484765988	0.123222325866416	0,918134662671627	-0.123222325866415	2745
P35235	sp|P35235|PTN11_MOUSE Tyrosine-protein phosphatase non-receptor type 11 OS=Mus musculus GN=Ptpn11 PE=1 SV=2;sp|P35235-2|PTN11_MOUSE Isoform 2 of Tyrosine-protein phosphatase non-receptor type 11 OS=Mus musculus GN=Ptpn11	1,09	0,6040896967857 8	0,86472932807488	29,2069428457707	29,7016909671295	29,6699505699435	29,6117382906239	29,6815604610594	29,541802057065	6653703908621,04	7247663591260,59	−0,12335832518637 3	0,0093793123788726	0,0023598513127801	1,0892675253958 9	0.123358325186373	0,918048116450 14	-0.123358325186373	2746
P16546-2	sp|P16546-2|SPTN1_MOUSE Isoform 2 of Spectrin alpha chain, non-erythrocytic 1 OS=Mus musculus GN=Sptan1;sp|P16546|SPTN1_MOUSE Spectrin alpha chain, non-erythrocytic 1 OS=Mus musculus GN=Sptan1 PE=1 SV=4	1,09	0,725486571169063	0,921719793333881	25,8849767619169	25,789671987075	26,4553495397667	25,9147147066523	26,3554364395044	26,1166289768567	204397561040,898	222663323542,413	−0,12348595962328 4	0,0138225352925676	0,0084434420964090	1,0893638965577 5	0.123485959623287	0,917966900830726	-0.123485959623287	2747
O88507	sp|O88507|CNTFR_MOUSE Ciliary neurotrophic factor receptor subunit alpha OS=Mus musculus GN=Cntfr PE=1 SV=2;sp|P26992|CNTFR_HUMAN Ciliary neurotrophic factor receptor subunit alpha OS=Homo sapiens GN=CNTFR PE=1 SV=2	1,09	0,4740022977484	0,799218469891531	25,6003102586315	25,9308965147294	25,9546021761348	25,9353037091857	25,8459890590547	25,9613397477305	164899656456,485	179638231181,835	−0,12350602857903 3	0,0076683310101931	0,0023345872185781	1,0893790505211 8	0.123506028579037	0,91795413132057	-0.123506028579037	2748
Q61245-2	sp|Q61245-2|COBA1_MOUSE Isoform Short of Collagen alpha-1(XI) chain OS=Mus musculus GN=Col11a1;sp|Q61245|COBA1_MOUSE Collagen alpha-1(XI) chain OS=Mus musculus GN=Col11a1 PE=1 SV=2	1,09	0,449309885386207	0,786343703431612	26,2312364204502	26,0637890078749	26,1107739160253	26,0680641034151	26,4148721289321	26,1808314726337	224079435368,085	244200611242,472	−0,12405655819594 6	0,0033046846686182 9	0,0067465366472941	1,08979483477069	0.124056558195945	0,917603908638835	-0.124056558195945	2749
Q91WG7	sp|Q91WG7|DGKG_MOUSE Diacylglycerol kinase gamma OS=Mus musculus GN=Dgkg PE=2 SV=1	1,09	0,597995533969822	0,861677999617657	25,4643078754517	24,9998552831891	25,0100063220039	25,270357393125	25,3260413000039	25,1359541100947	84334468176,0468	91913853569,1214	−0,12415993304159 7	0,0105441387005734	0,0038710984268396	1,08987292570877	0.124159933041596	0,917538161019713	-0.124159933041596	2750
Q6PEB6	sp|Q6PEB6|PHOCN_MOUSE MOB-like protein phocein OS=Mus musculus GN=Mob4 PE=1 SV=1	1,09	0,770507411515978	0,936218563920244	27,8785137992401	28,4187987708268	28,6868977896118	28,7045051167192	28,0890345798713	28,4508773233213	2007818890854,79	2189743814653,29	−0,12513295277485	0,01453534757952 4	0,010885760765466	1,09060823395334	0.125132952774851	0,916919539819636	-0.125132952774851	2751
Q8CFV4	sp|Q8CFV4|NRN1_MOUSE Neuritin OS=Mus musculus GN=Nrn1 PE=1 SV=1	1,09	0,922178323121994	0,982113422958876	25,6387500127636	24,1126737547988	25,2593441260161	23,5687032910947	25,7355004334847	25,9677083343354	72263810745,5684	78836139594,6317	−0,12558379742929 1	0,0317768180858672	0,0527343082953664	1,09094910414016	0.125583797429289	0,916633045670962	-0.125583797429289	2752
Q91YJ2	sp|Q91YJ2|SNX4_MOUSE Sorting nexin-4 OS=Mus musculus GN=Snx4 PE=2 SV=1	1,09	0,565322928594481	0,845110794140252	28,344311301084	28,7755965557594	28,6488796693929	28,8294358832546	28,6513548480099	28,5498933163116	2607976742183,62	2845883506864,64	−0,12594560418761 5	0,00775352643497742	0,0049346524497971	1,09122273248565	0.125945604187617	0,916403196368664	-0.125945604187617	2753
Q99LB2	sp|Q99LB2|DHRS4_MOUSE Dehydrogenase/reductase SDR family member 4 OS=Mus musculus GN=Dhrs4 PE=1 SV=3	1,09	0,578934648418407	0,852070158615807	27,9056879546371	28,2133967375042	28,43987847634	28,3251900002394	28,2250895728033	28,2708618860744	1742463541320,93	1901594422070,2	−0,12608110657632 9	0,00951250579656843	0,0017723570813112	1,09132522831935	0.126081106576335	0,916317128982725	-0.126081106576335	2754
Q8BP47	sp|Q8BP47|SYNC_MOUSE Asparagine--tRNA ligase, cytoplasmic OS=Mus musculus GN=Nars PE=1 SV=2	1,09	0,71091289444594	0,916805961322165	30,447826850692	31,1310784705769	31,1238583488395	31,1032923983988	30,9912486313499	30,8709415115779	26308711167516,2	28716534289106,7	−0,12634107088481	0,0126989043603373	0,0037497766123312	1,09152189578042	0.126341070884807	0,916152029442359	-0.126341070884807	2755
Q9ESN4	sp|Q9ESN4|C1QL3_MOUSE Complement C1q-like protein 3 OS=Mus musculus GN=C1ql3 PE=1 SV=1	1,09	0,507786441422753	0,816124555116335	24,6690327146511	24,9101751598063	24,6483181932764	24,7304936940114	25,035270903413	24,7251231135384	55658947824,4609	60765978408,5435	−0,12665017888234 2	0,00588349161333135	0,0071498892713246	1,09175578741067	0.126650178882342	0,915955758175292	-0.126650178882343	2756
Q923S9	sp|Q923S9|RAB30_MOUSE Ras-related protein Rab-30 OS=Mus musculus GN=Rab30 PE=2 SV=1	1,09	0,679444095039793	0,903867745423687	25,3211500311766	25,6171312267919	25,848124338265	25,7991721647102	25,3890942487746	25,8617916340518	130608290527,685	142606180073,622	−0,12679002800445 1	0,0103203497270223	0,0099968051721743	1,0918616230061 9	0.126790028004447	0,915866973368595	-0.126790028004447	2757

Q9D9V3-2	sp|Q9D9V3-2|ECHD1_MOUSE Isoform 2 of Ethylmalonyl-CoA decarboxylase OS=Mus musculus GN=Echdc1;sp|Q9D9V3|ECHD1_MOUSE Ethylmalonyl-CoA decarboxylase OS=Mus musculus GN=Echdc1 PE=1 SV=2	1,09	0,857045516451095	0,965053237204545	28,2685915515089	29,2745375866608	29,7066894223823	29,356105721571	28,6943108318933	29,4637941408889	4272724746166,38	4666374579381,91	−0,12714574009507	0,0253713110814867	0,014283443691759	1,09213086650825	0.127145740095071	0,915641184281509	-0.127145740095071	2758
Q3U308	sp|Q3U308|CTU2_MOUSE Cytoplasmic tRNA 2-thiolation protein 2 OS=Mus musculus GN=Ctu2 PE=2 SV=1	1,09	0,673652909747269	0,900894908348996	24,6135674323953	25,2737175242021	25,1110816865738	25,0503231987898	24,9939508693433	25,2191352268015	71965706776,2463	78613014244,9969	−0,12745857310771	0,013757905929237 9	0,0046706941266159	1,09236770910092	0.127458573107718	0,915442658793946	-0.127458573107718	2759
Q9R112	sp|Q9R112|SQRD_MOUSE Sulfide:quinone oxidoreductase, mitochondrial OS=Mus musculus GN=Sqrdl PE=1 SV=3	1,09	0,606292352777518	0,865805356251592	24,7428706679691	24,5323306407483	24,3378985272248	24,3751804819673	24,7462638463834	24,7568685258369	45351085320,4114	49542874578,5626	−0,12754050206729	0,00825421515851475	0,008826855632173	1,0924297451436	0.127540502067288	0,915390673354973	-0.127540502067288	2760
Q9Z2I0	sp|Q9Z2I0|LETM1_MOUSE LETM1 and EF-hand domain-containing protein 1, mitochondrial OS=Mus musculus GN=Letm1 PE=2 SV=1	1,09	0,413489158141312	0,766530246241387	30,9878465239167	30,8001322897362	30,6442031737676	30,9355404205447	30,8138966768348	30,9481182033933	24039689971211,4	26263075631930,9	−0,12761758771921	0,0055846294871325 6	0,0023990661206576	1,09248811708396	0.127617587719214	0,915341763779704	-0.127617587719214	2761
Q8VDK1-2	sp|Q8VDK1-2|NIT1_MOUSE Isoform 2 of Nitrilase homolog 1 OS=Mus musculus GN=Nit1;sp|Q8VDK1|NIT1_MOUSE Nitrilase homolog 1 OS=Mus musculus GN=Nit1 PE=2 SV=2	1,09	0,463319775585796	0,793595716606975	28,4053017035069	28,6215713507648	28,7194223068834	28,8247153553848	28,5934970858939	28,5934970858939	2588496843145,07	2827940551619,22	−0,12763723369832	0,0056237098077587 7	0,0046561310384120	1,09250299420231	0.127637233698329	0,915329299147732	-0.127637233698329	2762
Q9ESN6	sp|Q9ESN6|TRIM2_MOUSE Tripartite motif-containing protein 2 OS=Mus musculus GN=Trim2 PE=1 SV=1	1,09	0,509021505956014	0,816124555116335	29,7389386091483	30,1781158654775	30,0196926870257	30,0580803905391	30,0036031524467	30,1415815593921	10463516678030,4	11435629979766,7	−0,12816803713131	0,0074188828447519 3	0,0023113168731188	1,09290502721493	0.128168037131315	0,91499258865001	-0.128168037131315	2763
Q8CF89	sp|Q8CF89|TAB1_MOUSE TGF-beta-activated kinase 1 and MAP3K7-binding protein 1 OS=Mus musculus GN=Tab1 PE=1 SV=2	1,09	0,671419593702082	0,900583196162456	24,5806585090775	24,6754463944538	24,9067459177116	24,8903374302592	24,4554828608451	25,0836030464032	54471870826,3703	59533664485,3541	−0,12819428241748	0,0067852768771843 7	0,012967060946923	1,09292490935585	0.128194282417484	0,91497594339705	-0.128194282417484	2764
Q8C1A5	sp|Q8C1A5|THOP1_MOUSE Thimet oligopeptidase OS=Mus musculus GN=Thop1 PE=1 SV=1	1,09	0,453242549560445	0,788322280554728	30,7314349400907	30,6656706138036	30,3537800086882	30,7195270753918	30,6477397566522	30,6509513772108	19155835689193,5	20941195325206,8	−0,12855982787400	0,0065967417791496 4	0,0013220573326707	1,0932018662605 5	0.128559827873999	0,91474413908626	-0.128559827873999	2765
Q01063-2	sp|Q01063-2|PDE4D_MOUSE Isoform 9 of cAMP-specific 3,5-cyclic phosphodiesterase 4D OS=Mus musculus GN=Pde4d;sp|Q01063|PDE4D_MOUSE cAMP-specific 3,5-cyclic phosphodiesterase 4D OS=Mus musculus GN=Pde4d PE=1 SV=2;sp|Q01063-3|PDE4D_MOUSE Isoform 3 of c	1,09	0,472119415128793	0,798416551888819	27,1097993894113	26,9425529647263	26,7386997761827	26,9547500236637	26,9600309433248	27,1437352154627	496252501681,442	542527924164,365	−0,12862302054171	0,0069011534573311 7	0,0039829958682739	1,09324975154005	0.128623020541711	0,914704072506131	-0.128623020541711	2766
P49070	sp|P49070|CAMLG_MOUSE Calcium signal-modulating cyclophilin ligand OS=Mus musculus GN=Camlg PE=1 SV=2	1,09	0,501316560059717	0,81266995218252	27,6075461660519	27,6454609497024	27,8851022330226	27,9977571851328	27,742165065775 3	27,6659486438165	1085110690117,55	1186414636870,9	−0,12876608482652	0,0054307493769585 1	0,0062512643792650	1,09335816859603	0.128766084826532	0,91461337073476	-0.128766084826532	2767
Q8K4B0	sp|Q8K4B0|MTA1_MOUSE Metastasis-associated protein MTA1 OS=Mus musculus GN=Mta1 PE=1 SV=1	1,09	0,56443457978255	0,845110794140252	27,0687747291807	26,8380058322934	26,5758572324515	26,9782391770371	26,9782391770371	26,794197060117	447770173123,808	489618231371,877	−0,12889884850965	0,0091929826599394 1	0,0039475867613882	1,09345878926263	0.12889884850965	0,91452920751988	-0.12889884850965	2768
Q9WV85	sp|Q9WV85|NDK3_MOUSE Nucleoside diphosphate kinase 3 OS=Mus musculus GN=Nme3 PE=2 SV=3	1,09	0,809906245283734	0,94879038498679	25,6042732888588	26,3998169118976	26,2733618497883	25,6404580141254	26,3346641672483	26,5713233590086	214694949245,58	234834891186,136	−0,12935852460668	0,016384196832646 5	0,0184786693767725	1,09380724610116	0.129358524606692	0,914237863722761	-0.129358524606692	2769
Q9CWS4	sp|Q9CWS4|INT11_MOUSE Integrator complex subunit 11 OS=Mus musculus GN=Cpsf3l PE=2 SV=1	1,09	0,697456656216287	0,911796014563862	22,6290908514132	23,3066932565955	23,2491539067247	23,2969409758378	23,0239680619191	23,1333909033916	10364434315,2648	11338085725,4414	−0,12953570514780	0,016291416785548 7	0,0059338850273856	1,09394158721645	0.12953570514781	0,914125591060594	-0.12953570514781	2770
Q62523	sp|Q62523|ZYX_MOUSE Zyxin OS=Mus musculus GN=Zyx PE=1 SV=2	1,09	0,419676996134793	0,769072485508496	26,517535165276	26,3346641672483	26,1541626625147	26,4187355803847	26,3643567050085	26,4927145297487	273742576074,362	299466662677,203	−0,12957556858524	0,0068989712111176 1	0,0024381147683178	1,09397181458486	0.129575568585246	0,914100332995766	-0.129575568585246	2771
Q8C754	sp|Q8C754|VPS52_MOUSE Vacuolar protein sorting-associated protein 52 homolog OS=Mus musculus GN=Vps52 PE=2 SV=1;sp|Q8C754-2|VPS52_MOUSE Isoform 2 of Vacuolar protein sorting-associated protein 52 homolog OS=Mus musculus GN=Vps52	1,09	0,367964059619041	0,739685989256097	30,1173421092281	30,2511880637938	29,9373485722038	30,1478585778955	30,274460469913	30,153305359808	11833547021596,8	12946865164946,1	−0,12972024318190	0,0052316926253105 4	0,0023706085949286	1,09408152444212	0.129720243181898	0,914008670889411	-0.129720243181898	2772
Q9CQV1	sp|Q9CQV1|TIM16_MOUSE Mitochondrial import inner membrane translocase subunit TIM16 OS=Mus musculus GN=Pam16 PE=2 SV=1	1,09	0,73967488903329	0,925497610201997	24,1023003631839	23,5485803366946	23,3634269361434	23,6506356046121	23,5647953275257	24,0692182003921	19071022601,1537	20869397967,7815	−0,13000677878620	0,016241192029649	0,0113579891846897	1,09429884302686	0.130006778786208	0,913827156422808	-0.130006778786207	2773
Q8C079	sp|Q8C079|STRP1_MOUSE Striatin-interacting protein 1 OS=Mus musculus GN=Strip1 PE=1 SV=2;sp|Q8C079-3|STRP1_MOUSE Isoform 3 of Striatin-interacting protein 1 OS=Mus musculus GN=Strip1;sp|Q8C079-4|STRP1_MOUSE Isoform 4 of Striatin-interacting protein 1 OS	1,09	0,646376403282866	0,889692946522666	28,8842531253939	28,4931602800957	28,2371702788332	28,706063719518	28,663140619517	28,5161702425418	2477310375171,57	2711324003959,02	−0,13022289486209	0,01141920853604 1	0,0034785078555778	1,09446278154438	0.130222894862098	0,913690275140209	-0.130222894862098	2774
Q78IK2	sp|Q78IK2|USMG5_MOUSE Up-regulated during skeletal muscle growth protein 5 OS=Mus musculus GN=Usmg5 PE=1 SV=1	1,09	0,603011188523088	0,863973416889613	28,0108060548788	27,8721796264246	27,7598510714024	27,6659486438165	28,0535845608097	28,1946741447067	1283928435736,43	1405483446799,63	−0,13050167133231	0,0045087039962961 5	0,0097876971771313	1,09467428844154	0.130501671332313	0,913513736970728	-0.130501671332313	2775

P10518	sp|P10518|HEM2_MOUSE Delta-aminolevulinic acid dehydratase OS=Mus musculus GN=Alad PE=1 SV=1;sp|P06214|HEM2_RAT Delta-aminolevulinic acid dehydratase OS=Rattus norvegicus GN=Alad PE=1 SV=1	1,10	0,723955854479575	0,92164812633208	28,0831095688452	28,62011627812	28,9450977968638	28,6821011242862	28,706063719518	28,5325900295205	2505329088796,2	2743489111059,45	−0,13101172792556	0,0152478797434888	0,0032822455707534	1,0950613727068	0.13101172792556	0,913190826490549	-0.13101172792556	2776
Q922H2	sp|Q922H2|PDK3_MOUSE [Pyruvate dehydrogenase (acetyl-transferring)] kinase isozyme 3, mitochondrial OS=Mus musculus GN=Pdk3 PE=1 SV=1	1,10	0,617539777205239	0,870166479711399	28,9766402388757	29,0196626152411	28,9338137105631	28,8422515451511	29,4242425054228	28,9363589553108	3840814316212,46	4206355318620,71	−0,13115850373194	0,00148134471462174	0,010747698745806	1,0951727868919	0.131158503731942	0,913097925705403	-0.131158503731942	2777
Q8CIN4	sp|Q8CIN4|PAK2_MOUSE Serine/threonine-protein kinase PAK 2 OS=Mus musculus GN=Pak2 PE=1 SV=1	1,10	0,561242967556326	0,844368539265838	30,5213672527491	30,9727064202915	30,9355404205447	31,0176093622394	30,8724847418601	30,8129891084665	24019121651451	26311513566835,3	−0,13151084726331	0,0081318743282794	0,0034061626446751	1,09544028914337	0.131510847263316	0,912874950748794	-0.131510847263316	2778
O89079	sp|O89079|COPE_MOUSE Coatomer subunit epsilon OS=Mus musculus GN=Cope PE=2 SV=3	1,10	0,543022038121786	0,83367103326311	28,4205387528905	28,3564695698073	28,8247153553848	28,6558298268788	28,6581410656887	28,5614572625613	2466713694666,62	2702349561191,67	−0,13162403056787	0,00889733916148518	0,0019271634235930	1,09552623275029	0.131624030567872	0,912803336063918	-0.131624030567872	2779
Q9WUA3	sp|Q9WUA3|PFKAP_MOUSE ATP-dependent 6-phosphofructokinase, platelet type OS=Mus musculus GN=Pfkp PE=1 SV=1;sp|Q9WUA3-2|PFKAP_MOUSE Isoform 2 of ATP-dependent 6-phosphofructokinase, platelet type OS=Mus musculus GN=Pfkp	1,10	0,578913263548521	0,852070158615807	33,5071865503134	33,1816073307961	32,9377590356243	33,3243876702912	33,2755034398491	33,3022847228181	264496986044360	289948743765879	−0,13254660479751	0,00860281696803091	0,0007351010292725	1,09622702361247	0.132546604797516	0,912219803435085	-0.132546604797516	2780
P26638	sp|P26638|SYSC_MOUSE Serine--tRNA ligase, cytoplasmic OS=Mus musculus GN=Sars PE=2 SV=3	1,10	0,658427789234358	0,893187009962568	30,5614058829116	31,1488485026459	31,0751300491871	31,1726430469682	31,0449398168704	30,8437717553966	27043329130281,2	29649060061651,5	−0,13271360553252	0,0103467582057937	0,0053447830176414	1,09635392590968	0.132713605532522	0,912114214550073	-0.132713605532522	2781
P52760	sp|P52760|UK114_MOUSE Ribonuclease UK114 OS=Mus musculus GN=Hrsp12 PE=1 SV=3	1,10	0,667791061756344	0,898352728146089	30,2207870528903	29,9548618864297	29,5991882691668	29,8046256258292	30,038498203068	30,2081872493644	9913769211065,24	10870824800065,2	−0,13295582695316	0,0104219861552244	0,0067504742779708	1,09653801380929	0.132955826953165	0,911961087902528	-0.132955826953165	2782
Q8K1M6	sp|Q8K1M6|DNM1L_MOUSE Dynamin-1-like protein OS=Mus musculus GN=Dnm1l PE=1 SV=2;sp|Q8K1M6-2|DNM1L_MOUSE Isoform 2 of Dynamin-1-like protein OS=Mus musculus GN=Dnm1l;sp|Q8K1M6-4|DNM1L_MOUSE Isoform 4 of Dynamin-1-like protein OS=Mus musculus GN=Dnm1l	1,10	0,475222440747109	0,79947899324683	33,0960166649535	33,037004306747	32,773886273776	33,1921566422038	32,9074754208366	33,0839850192138	208085273803781	228191360677721	−0,13306930309477	0,00520197976011836	0,0043465189657469	1,09662426613091	0.133069303094776	0,911889359815264	-0.133069303094776	2783
Q8BJW6	sp|Q8BJW6|EIF2A_MOUSE Eukaryotic translation initiation factor 2A OS=Mus musculus GN=Eif2a PE=2 SV=2;sp|Q8BJW6-2|EIF2A_MOUSE Isoform 2 of Eukaryotic translation initiation factor 2A OS=Mus musculus GN=Eif2a	1,10	0,719439823062411	0,920149210230605	26,4493758498685	27,2799384547728	27,0100387989245	27,0697573276934	27,1000546991755	26,8468256315125	487773844114,234	535007116769,643	−0,13334568404915	0,0157424692792358	0,0051207261188845	1,09683436950413	0.13334568404915	0,911714683459536	-0.13334568404915	2784
Q99KR3	sp|Q99KR3|LACB2_MOUSE Beta-lactamase-like protein 2 OS=Mus musculus GN=Lactb2 PE=1 SV=1	1,10	0,764962507830678	0,934060902494713	25,5940945146071	26,2847000887215	26,587342146856	26,3040142907628	26,3883602649067	26,0524841852733	228631852695,997	250891458671,925	−0,13403694461789	0,0194642739869243	0,0066570143372157	1,09736003847865	0.134036944617894	0,911277944280138	-0.134036944617894	2785
O55060	sp|O55060|TPMT_MOUSE Thiopurine S-methyltransferase OS=Mus musculus GN=Tpmt PE=1 SV=1;sp|Q9QX22|TPMT_MUSSP Thiopurine S-methyltransferase OS=Mus spretus GN=Tpmt PE=2 SV=1	1,10	0,710022100891909	0,916805961322165	25,9556095100875	26,4493758498685	26,7743864526719	26,6147061508255	26,3498310529515	26,4937458641267	289993381584,079	318247225376,192	−0,13412796161148	0,0156206125195406	0,0050065221658538	1,0974292711019	0.134127961611488	0,911220455233459	-0.134127961611488	2786
Q99J23	sp|Q99J23|GHDC_MOUSE GH3 domain-containing protein OS=Mus musculus GN=Ghdc PE=2 SV=2;sp|Q8N2G8-2|GHDC_HUMAN Isoform 2 of GH3 domain-containing protein OS=Homo sapiens GN=GHDC;sp|Q8N2G8-3|GHDC_HUMAN Isoform 3 of GH3 domain-containing protein OS=Homo sapi	1,10	0,84613710945074	0,961071709438436	21,5741688711869	22,7116660906337	21,8085797576661	22,451234435472	22,4605995625438	21,4618051524005	3699529821,16952	4060396165,35013	−0,13427856726572	0,0272615764828927	0,0259426716945559	1,09754383979166	0.134278567265721	0,911125336177759	-0.134278567265721	2787
O70310	sp|O70310|NMT1_MOUSE Glycylpeptide N-tetradecanoyltransferase 1 OS=Mus musculus GN=Nmt1 PE=1 SV=1	1,10	0,601105340191957	0,862877020598131	28,9001884639333	29,483312762562	29,3445457208605	29,3758840404762	29,351342606842	29,2802995314596	5011128803793,7	5500400697436,5	−0,13440110040133	0,0104168111235223	0,0016921864510147	1,09763706198739	0.134401100401336	0,911047954402517	-0.134401100401336	2788
Q91VJ5	sp|Q91VJ5|PQBP1_MOUSE Polyglutamine-binding protein 1 OS=Mus musculus GN=Pqbp1 PE=1 SV=1	1,10	0,755885648928338	0,931775125431143	25,3909018070988	24,5138570468075	25,1746957465022	24,8340128117331	25,2582028351988	25,2670557453431	73937417790,2567	81165582283,7853	−0,13456343266109	0,018257494880972	0,0098528589965963	1,09776057522097	0.134563432661093	0,910945448918777	-0.134563432661093	2789

Q3TLP5	sp|Q3TLP5|ECHD2_MOUSE Enoyl-CoA hydratase domain-containing protein 2, mitochondrial OS=Mus musculus GN=Echdc2 PE=1 SV=2;sp|Q3TLP5-2|ECHD2_MOUSE Isoform 2 of Enoyl-CoA hydratase domain-containing protein 2, mitochondrial OS=Mus musculus GN=Echdc2;sp|Q3T	1,10	0,680010844928018	0,904091747254913	24,343352667187	24,4697558524359	24,7207953569625	24,2355146437319	24,6683213514674	24,9101751598063	44169541943,5881	48492276995,3927	−0,13470312716451	0,00783803512889136	0,0138918527298726	1,09786687526272	0.134703127164517	0,910857247387758	-0.134703127164517	2790
Q61425	sp|Q61425|HCDH_MOUSE Hydroxyacyl-coenzyme A dehydrogenase, mitochondrial OS=Mus musculus GN=Hadh PE=1 SV=2	1,10	0,681127369219925	0,904517472334246	29,3206469921288	29,7364644042824	30,0036031524467	29,9980024503029	29,6389646388719	29,7040018754618	7813740087693,02	8578867161157,3	−0,13477388527706	0,0115931510964171	0,0064237074569116	1,09792072232725	0.134773885277063	0,910812574773443	-0.134773885277063	2791
Q9R1R2	sp|Q9R1R2|TRIM3_MOUSE Tripartite motif-containing protein 3 OS=Mus musculus GN=Trim3 PE=1 SV=1	1,10	0,510157302135199	0,816791532413287	29,1446312403025	29,2565888136709	28,7995948141363	29,1101735891658	29,2283500040983	29,142784813033	4203498578578,98	4615476068951,93	−0,13488887884811	0,00819478979748962	0,0020930256629891	1,09800823829759	0.134888878848112	0,910739979101116	-0.134888878848112	2792
P97390	sp|P97390|VPS45_MOUSE Vacuolar protein sorting-associated protein 45 OS=Mus musculus GN=Vps45 PE=1 SV=1	1,10	0,458699159277418	0,790967157530661	28,5963004492755	28,8979126911188	28,9104405271909	29,0412682162963	28,8118954040246	28,8320015068502	3223701090881,77	3539671500821,91	−0,13489749721907	0,00617545621285956	0,0043960035906094	1,09801479759828	0.134897497219073	0,910734538539305	-0.134897497219073	2793
Q8K2L8	sp|Q8K2L8|TPC12_MOUSE Trafficking protein particle complex subunit 12 OS=Mus musculus GN=Trappc12 PE=1 SV=2	1,10	0,646235689771476	0,889692946522666	27,802797894039	27,1551566172011	27,2789110284764	27,4581127618677	27,5987969361743	27,4606749995793	803536552942,203	882356111387,347	−0,13499737899722	0,0125420077558234	0,0029264521281979	1,09809081883897	0.134997378997222	0,91067148804442	-0.134997378997221	2794
P18242	sp|P18242|CATD_MOUSE Cathepsin D OS=Mus musculus GN=Ctsd PE=1 SV=1	1,10	0,117726639669346	0,491140824870552	30,3560902036496	30,1944393020597	30,312297883722	30,4300715036396	30,312297883722	30,4016042683483	14247593480360,7	15647379141649,6	−0,1352027747082	0,00276039115258881	0,0020226252131212	1,0982471645628	0.135202774708193	0,910541845467079	-0.135202774708193	2795
Q8VDG5	sp|Q8VDG5|PPCS_MOUSE Phosphopantothenate--cysteine ligase OS=Mus musculus GN=Ppcs PE=2 SV=1;sp|Q8VDG5-2|PPCS_MOUSE Isoform 2 of Phosphopantothenate--cysteine ligase OS=Mus musculus GN=Ppcs	1,10	0,733261976513468	0,924701393764781	25,035270903413	25,3867743256002	25,9627882430883	25,6594416194182	25,500765153124	25,506098678661	114245006315,494	125482877305,972	−0,13535940946635	0,0183910370740558	0,0035261271224834	1,09836640876402	0.13535940946636	0,910442992448475	-0.13535940946636	2796
P83917	sp|P83917|CBX1_MOUSE Chromobox protein homolog 1 OS=Mus musculus GN=Cbx1 PE=1 SV=1	1,10	0,589942690221251	0,858561075424286	26,0532668033667	26,5000720757695	26,5591097026922	26,6033375672616	26,4033494281023	26,387321076432	283595905508,731	311501302724,191	−0,13540149329712	0,0104883200374147	0,0045478485082200	1,09839844889651	0.135401493297128	0,910416434950938	-0.135401493297128	2797
Q8VCI5	sp|Q8VCI5|PEX19_MOUSE Peroxisomal biogenesis factor 19 OS=Mus musculus GN=Pex19 PE=1 SV=1;sp|Q8VCI5-2|PEX19_MOUSE Isoform 2 of Peroxisomal biogenesis factor 19 OS=Mus musculus GN=Pex19	1,10	0,729144067516831	0,924155719504589	25,9391068700574	26,3976388083925	26,6173376487642	26,6277756439543	26,0475252294948	26,5603842110093	269013600765,864	295488284945,67	−0,13542181956065	0,0131488959370754	0,0120153235989917	1,09841392444261	0.135421819560659	0,910403608100153	-0.135421819560659	2798
Q8K3W0	sp|Q8K3W0|BRE_MOUSE BRCA1-A complex subunit BRE OS=Mus musculus GN=Bre PE=1 SV=2;sp|Q6SP92|BRE_MESAU BRCA1-A complex subunit BRE OS=Mesocricetus auratus GN=Bre PE=2 SV=1;sp|Q8K3W0-1|BRE_MOUSE Isoform 1 of BRCA1-A complex subunit BRE OS=Mus musculus GN=B	1,10	0,430429229550083	0,775108167118334	26,6455238891739	26,8032741998557	26,9195750813523	26,7056831906351	26,9791446367106	26,9656720986252	431036117766,164	473538935493,876	−0,13567429039679	0,00513437153691903	0,0057336699957496	1,09860616309367	0.135674290396799	0,91024430191071	-0.135674290396799	2799
Q9CZM2	sp|Q9CZM2|RL15_MOUSE 60S ribosomal protein L15 OS=Mus musculus GN=Rpl15 PE=2 SV=4	1,10	0,729230418164805	0,924155719504589	25,8662283570416	26,2142780507656	26,5785183382483	26,6710608514719	26,1671183698062	26,1042219466073	243814838450,685	267968084486,406	−0,13627525282626	0,0135842896060009	0,0118074793692481	1,09906388876576	0.136275252826266	0,909865213680153	-0.136275252826266	2800
Q9QYB5	sp|Q9QYB5|ADDG_MOUSE Gamma-adducin OS=Mus musculus GN=Add3 PE=1 SV=2;sp|Q9QYB5-2|ADDG_MOUSE Isoform 1 of Gamma-adducin OS=Mus musculus GN=Add3	1,10	0,319995473224745	0,723342215945077	30,2511880637938	30,1714331301119	29,9349818756405	30,2119986671099	30,2268071384523	30,2028177448941	12039343445071	13234848661435,1	−0,13658497977331	0,00545986423757478	0,0004006175490341	1,09929986812143	0.136584979773327	0,909669898995697	-0.136584979773327	2801
Q8CB44	sp|Q8CB44|GRAM4_MOUSE GRAM domain-containing protein 4 OS=Mus musculus GN=Gramd4 PE=2 SV=1	1,10	0,736890283767125	0,924701393764781	24,1103167979477	24,3404652835671	24,3639618883844	24,7280852384171	24,5323306407483	23,8383590411367	34754723344,3799	38205996121,3067	−0,13659001453506	0,00577434340066941	0,0191877700311271	1,09930370449877	0.136590014535063	0,909666724407112	-0.136590014535063	2802
P62482	sp|P62482|KCAB2_MOUSE Voltage-gated potassium channel subunit beta-2 OS=Mus musculus GN=Kcnab2 PE=2 SV=1	1,10	0,302559319111161	0,715641870477102	29,8475138120678	29,6230833170184	29,7200994190062	29,8660763732758	29,9112106332108	29,6976512816979	8159729890460,9	8970651581102,71	−0,13669138294815	0,00378597231732398	0,0037738485906333	1,09938094784116	0.136691382948153	0,9096028105305	-0.136691382948153	2803
Q9JIH2	sp|Q9JIH2|NUP50_MOUSE Nuclear pore complex protein Nup50 OS=Mus musculus GN=Nup50 PE=1 SV=3	1,10	0,703940331878931	0,915491421841963	23,8690116229415	24,1206114949404	24,021910159197	24,5525942656835	23,7444339745312	23,9989811215525	26591153592,0656	29236091324,3779	−0,13680407887710	0,00528119941199126	0,0171461694163227	1,0994668291902	0.136804078877108	0,909531759804469	-0.136804078877108	2804
Q99K51	sp|Q99K51|PLST_MOUSE Plastin-3 OS=Mus musculus GN=Pls3 PE=1 SV=3	1,10	0,645628627537393	0,889692946522666	30,1559838428577	30,836402611215	30,6071786088968	30,6799097956272	30,5213672527491	30,6837245297255	18213575787200,9	20031634389709,3	−0,13726594828999	0,0113381934065377	0,0030251542031234	1,09981887267771	0.137265948289995	0,909240625745327	-0.137265948289995	2805
Q6PHN9	sp|Q6PHN9|RAB35_MOUSE Ras-related protein Rab-35 OS=Mus musculus GN=Rab35 PE=1 SV=1	1,10	0,527849756067023	0,828461287271168	29,497763870862	29,9092926391212	29,9440608870272	29,7971159967527	29,8788419232647	29,9610005225932	8607936986705,41	9468448225318,42	−0,13746048632327	0,00833483980846892	0,0027424745172914	1,0999671860914	0.137460486323273	0,909118028832642	-0.137460486323273	2806

Q6NVE9	sp|Q6NVE9|PPTC7_MOUSE Protein phosphatase PTC7 homolog OS=Mus musculus GN=Pptc7 PE=2 SV=1;sp|Q6NVE9-2|PPTC7_MOUSE Isoform 2 of Protein phosphatase PTC7 homolog OS=Mus musculus GN=Pptc7	1,10	0,780646064973437	0,940251150142017	25,6741775613297	26,234878662775	26,8229841428725	26,336120588715	26,4873971485829	26,1944717091464	249821738796,945	274805610975,028	−0,137512439631331	0,0218890973356703	0,00556161026338406	1,10000679804086	0.137512439631329	0,909085290910041	-0.137512439631329	2807
Q91Y86	sp|Q91Y86|MK08_MOUSE Mitogen-activated protein kinase 8 OS=Mus musculus GN=Mapk8 PE=1 SV=1	1,10	0,331662742032019	0,72953198881961	25,8362365598065	25,5327595104657	25,6452858661328	25,7036960429525	25,848124338265	25,7487841328276	140915648699,338	155027471143,932	−0,137692054285294	0,00597621983238477	0,00286774775172355	1,10014375674275	0.137692054285293	0,908972117390135	-0.137692054285293	2808
Q8BRF7	sp|Q8BRF7|SCFD1_MOUSE Sec1 family domain-containing protein 1 OS=Mus musculus GN=Scfd1 PE=1 SV=1;sp|Q8BRF7-3|SCFD1_MOUSE Isoform 3 of Sec1 family domain-containing protein 1 OS=Mus musculus GN=Scfd1;sp|Q8BRF7-2|SCFD1_MOUSE Isoform 2 of Sec1 family domai	1,10	0,707800568115907	0,916805961322165	27,6204662728737	28,4026187469632	28,3270681210585	28,2234557658422	28,1113694390224	28,3022437834415	1625306799580,77	1788424766165,59	−0,137977356737217	0,015344014865523	0,00339992972725729	1,10036133893422	0.13797735673722	0,908792379936369	-0.13797735673722	2809
Q3TDN2	sp|Q3TDN2|FAF2_MOUSE FAS-associated factor 2 OS=Mus musculus GN=Faf2 PE=2 SV=2;sp|Q3TDN2-2|FAF2_MOUSE Isoform 2 of FAS-associated factor 2 OS=Mus musculus GN=Faf2	1,10	0,449112215503698	0,786343703431612	26,9128048037016	26,7611411553313	26,9656720986252	26,7551771799298	27,0645280083663	27,1079700259264	471824384181,674	519374862634,712	−0,138526210422745	0,00394971684049794	0,00713135301339823	1,10078003606259	0.138526210422747	0,908446708006195	-0.138526210422747	2810
Q7TSY6-4	sp|Q7TSY6-4|CELF4_MOUSE Isoform 4 of CUGBP Elav-like family member 4 OS=Mus musculus GN=Celf4;sp|Q7TSY6-2|CELF4_MOUSE Isoform 2 of CUGBP Elav-like family member 4 OS=Mus musculus GN=Celf4;sp|Q7TSY6-3|CELF4_MOUSE Isoform 3 of CUGBP Elav-like family membe	1,10	0,760217540558195	0,933136198610489	25,6392323739276	26,513257270011	26,5575313626245	26,4674873848154	26,3957362099935	26,1356881565921	247994813318,492	273063603213,571	−0,138927081645442	0,0197385041229316	0,00662961896129175	1,10108594433741	0.138927081645443	0,908194319564911	-0.138927081645443	2811
Q6ZPT1	sp|Q6ZPT1|KLHL9_MOUSE Kelch-like protein 9 OS=Mus musculus GN=Klhl9 PE=2 SV=2;sp|Q80TF4-5|KLH13_MOUSE Isoform 5 of Kelch-like protein 13 OS=Mus musculus GN=Klhl13;sp|Q80TF4-3|KLH13_MOUSE Isoform 3 of Kelch-like protein 13 OS=Mus musculus GN=Klhl13;sp|Q	1,10	0,795324598036761	0,944278930287433	23,4277205768759	24,1495233367792	23,8330236397602	24,5138570468075	23,620865820898	23,5647953275257	21761811788,4546	23964499828,1675	−0,139100155507255	0,0151999440574905	0,0222802086252573	1,10121804476232	0.139100155507259	0,908085373969546	-0.139100155507259	2812
Q920A5	sp|Q920A5|RISC_MOUSE Retinoid-inducible serine carboxypeptidase OS=Mus musculus GN=Scpep1 PE=2 SV=2	1,10	0,456337947444186	0,789976332360472	23,9091561309391	24,2371377474287	23,9460675522639	24,1228372462825	23,9952333945482	24,2636612879042	27317330198,6288	30083538825,8761	−0,139157794197709	0,00747604900811155	0,00556500337073368	1,10126204160999	0.139157794197706	0,908049094780433	-0.139157794197706	2813
Q8BWT1	sp|Q8BWT1|THIM_MOUSE 3-ketoacyl-CoA thiolase, mitochondrial OS=Mus musculus GN=Acaa2 PE=1 SV=3	1,10	0,278827499261486	0,699221280539289	30,8605365941129	30,5656049997843	30,7820965217206	30,8437717553966	30,8504306917222	30,8043490382255	22310518657745	24577450572909,5	−0,139611219602714	0,00496991930521443	0,00080779193935124	1,10160821224914	0.139611219602712	0,907763748382299	-0.139611219602712	2814
Q8BPU7	sp|Q8BPU7|ELMO1_MOUSE Engulfment and cell motility protein 1 OS=Mus musculus GN=Elmo1 PE=1 SV=2;sp|Q8BPU7-2|ELMO1_MOUSE Isoform 2 of Engulfment and cell motility protein 1 OS=Mus musculus GN=Elmo1	1,10	0,543332571313999	0,833734462878378	26,6793021818755	26,2970734250463	26,5247335525472	26,8016686908167	26,4055239012705	26,584740539218	322822902336,407	355684892983,31	−0,139856767313241	0,00725558145172546	0,0074583229561359	1,10179572269832	0.139856767313242	0,907609259501373	-0.139856767313242	2815
P60191	sp|P60191|RIMS4_MOUSE Regulating synaptic membrane exocytosis protein 4 OS=Mus musculus GN=Rims4 PE=2 SV=1	1,10	0,694234623667372	0,910309912045398	24,0697373811504	24,343352667187	24,7376841280185	24,7814467480862	24,3741949927678	24,2860801827562	38873996442,1102	42832970190,5809	−0,139916290707167	0,0137710029976122	0,0107947608959342	1,10184118204482	0.139916290707166	0,907571813702023	-0.139916290707166	2816
Q8C460-2	sp|Q8C460-2|ERI3_MOUSE Isoform 2 of ERI1 exoribonuclease 3 OS=Mus musculus GN=Eri3;sp|Q8C460|ERI3_MOUSE ERI1 exoribonuclease 3 OS=Mus musculus GN=Eri3 PE=1 SV=1	1,10	0,446674153156673	0,785227192247876	27,3476415937145	27,183957488667	27,3081622510207	27,1512361220385	27,4312593139902	27,5493288208279	703912886349,739	775888570642,092	−0,140452577098408	0,00313118606370087	0,0074683546388085	1,10225084053454	0.140452577098407	0,907234508902755	-0.140452577098407	2817
P0C0A3	sp|P0C0A3|CHMP6_MOUSE Charged multivesicular body protein 6 OS=Mus musculus GN=Chmp6 PE=2 SV=2	1,10	0,801700598076319	0,945787729921946	26,0234593655428	26,8660517354712	27,2212697752775	26,8071250785447	27,067317939297	26,5284979350479	395570010200,583	436031496615,42	−0,140499297884603	0,023038428958134	0,0100542051237789	1,10228653682396	0.140499297884604	0,907205129150282	-0.140499297884604	2818
Q8K0C4	sp|Q8K0C4|CP51A_MOUSE Lanosterol 14-alpha demethylase OS=Mus musculus GN=Cyp51a1 PE=2 SV=1	1,10	0,486563486960901	0,804557984450102	26,190885557456	26,0103345112904	25,8942049257386	26,238233207829	26,2409001964166	25,9086542006991	202055516651,335	222738112524,876	−0,140596696083834	0,00574303097874666	0,00731199647053675	1,10236095611896	0.140596696083839	0,907143884631638	-0.140596696083839	2819
Q8CG47	sp|Q8CG47|SMC4_MOUSE Structural maintenance of chromosomes protein 4 OS=Mus musculus GN=Smc4 PE=1 SV=1	1,10	0,739863963702428	0,925497610201997	23,9779430843893	24,4331596823421	24,8771377433071	24,6663146621165	24,6631481372109	24,2514976759772	40696726791,441	44867826916,2883	−0,140768547419581	0,0184044137858667	0,00972748330868890	1,10249227526879	0.140768547419581	0,907035833658058	-0.140768547419581	2820
Q3TIR3	sp|Q3TIR3|RIC8A_MOUSE Synembryn-A OS=Mus musculus GN=Ric8a PE=1 SV=2	1,10	0,678844103853081	0,903732687282123	27,4813946984528	28,1757537010239	28,1372043839272	28,1677723504312	27,966093558593	27,9533352167808	1350439117621,22	1488912408137,98	−0,140830283771509	0,0139712193572627	0,00429167286549333	1,10253945454474	0.140830283771516	0,906997020268013	-0.140830283771516	2821

Q8BTI8-3	sp|Q8BTI8-3|SRRM2_MOUSE Isoform 3 of Serine/arginine repetitive matrix protein 2 OS=Mus musculus GN=Srrm2;sp|Q8BTI8-2|SRRM2_MOUSE Isoform 2 of Serine/arginine repetitive matrix protein 2 OS=Mus musculus GN=Srrm2;sp|Q8BTI8|SRRM2_MOUSE Serine/arginine rep	1,10	0,841688175018947	0,959771286845933	21,1542211809417	22,8163576649882	22,080652069264	21,9399864331884	22,1710415493333	22,2332585619834	3646658011,16472	4020862148,9731	−0,14092996770395	0,0378292727238947	0,0069873679892860	1,10261563784229	0.140929967703955	0,906934352896443	-0.140929967703955	2822
Q99JF5	sp|Q99JF5|MVD1_MOUSE Diphosphomevalonate decarboxylase OS=Mus musculus GN=Mvd PE=1 SV=2	1,10	0,557735524459122	0,841889439813753	25,879283072998	25,3144985329333	25,5562998340598	25,6347612167554	25,6663122517335	25,7421229489083	129036413929,342	142280382511,626	−0,14095850810471	0,0110760673419714	0,0021486810195112	1,10263745077057	0.140958508104707	0,906916411465215	-0.140958508104707	2823
O35459	sp|O35459|ECH1_MOUSE Delta(3,5)-Delta(2,4)-dienoyl-CoA isomerase, mitochondrial OS=Mus musculus GN=Ech1 PE=1 SV=1	1,10	0,742331997169079	0,926180575907216	27,4101106762398	28,0297486856985	28,3747558938646	28,2008611250849	27,8772178137753	28,0297486856985	1359591068136,11	1499184697877,16	−0,14100534344382	0,0174955660938736	0,0057751057981379	1,10267324713483	0.141005343443831	0,906886970005287	-0.141005343443831	2824
P50171	sp|P50171|DHB8_MOUSE Estradiol 17-beta-dehydrogenase 8 OS=Mus musculus GN=Hsd17b8 PE=1 SV=2;sp|P50171-2|DHB8_MOUSE Isoform Long of Estradiol 17-beta-dehydrogenase 8 OS=Mus musculus GN=Hsd17b8	1,10	0,439347541201769	0,780009157984922	28,0318362877081	28,2346990589835	28,4562699090736	28,3730882269636	28,3695269666755	28,2742677874623	1840273072039,56	2029805301315,43	−0,14142149199275	0,00751694362884007	0,001977992387459	1,10299136152974	0.141421491992751	0,906625414194631	-0.141421491992751	2825
P04925	sp|P04925|PRIO_MOUSE Major prion protein OS=Mus musculus GN=Prnp PE=1 SV=2;sp|P04156-2|PRIO_HUMAN Isoform 2 of Major prion protein OS=Homo sapiens GN=PRNP;sp|P04156|PRIO_HUMAN Major prion protein OS=Homo sapiens GN=PRNP PE=1 SV=1	1,10	0,287757469375775	0,706812174354121	27,9479032340272	28,0805683689805	28,1709716265096	28,2885021532674	28,1401525448235	28,0649683586343	1545673844505,82	1704922922518,96	−0,1414705926142	0,00399763141535025	0,0040385907635860	1,10302890132947	0.141470592614201	0,906594558669048	-0.141470592614201	2826
P97371	sp|P97371|PSME1_MOUSE Proteasome activator complex subunit 1 OS=Mus musculus GN=Psme1 PE=2 SV=2;sp|Q06323|PSME1_HUMAN Proteasome activator complex subunit 1 OS=Homo sapiens GN=PSME1 PE=1 SV=1;sp|Q06323-3|PSME1_HUMAN Isoform 3 of Proteasome activator com	1,10	0,623054791702409	0,873726316984964	28,7217355155172	28,9822197969154	29,3780373232367	29,139589158536	29,0169853290817	29,2199061542331	4040262893998,85	4456984567176,76	−0,14161879537306	0,0113847278815412	0,0035086829646057	1,10314221725445	0.14161879537307	0,906501432325605	-0.14161879537307	2827
Q8BTX9	sp|Q8BTX9|HSDL1_MOUSE Inactive hydroxysteroid dehydrogenase-like protein 1 OS=Mus musculus GN=Hsdl1 PE=2 SV=1	1,10	0,38729958412637	0,752548292957873	28,1167813912022	28,0535845608097	28,3855524287391	28,2082847129067	28,3794989601019	28,2627967575825	1740695961253,3	1920346607081,05	−0,14170249268082	0,0062540970622851	0,0030925886562495	1,10320621741341	0.141702492680825	0,906448843575785	-0.141702492680825	2828
Q91W50	sp|Q91W50|CSDE1_MOUSE Cold shock domain-containing protein E1 OS=Mus musculus GN=Csde1 PE=2 SV=1	1,10	0,446459626620057	0,785227192247876	30,5656049997843	30,4157949618138	30,1742185244125	30,4225096278016	30,6061730946971	30,4225096278016	15708234197960,5	17334689504638,8	−0,14214098274242	0,00649915281730187	0,0034785162224968	1,10354157483146	0.142140982742426	0,906173381055192	-0.142140982742426	2829
O70439	sp|O70439|STX7_MOUSE Syntaxin-7 OS=Mus musculus GN=Stx7 PE=1 SV=3	1,10	0,673040993628753	0,900894908348996	27,9088339896986	28,5819748597617	28,4508773233213	28,429387167353	28,2133967375042	28,5948343218379	1979559361016,86	2184786895155,57	−0,14231323554040	0,0126038854334754	0,0067321012050657	1,10367334174475	0.142313235540412	0,906065193546441	-0.142313235540412	2830
Q8K2X3	sp|Q8K2X3|STN1_MOUSE CST complex subunit STN1 OS=Mus musculus GN=Obfc1 PE=1 SV=2	1,10	0,919713279192826	0,98176599643834	20,8620051295316	23,1333909033916	21,1387229278635	20,8620051295316	21,4673136439321	23,1009237110647	2686145187,2773	2964816053,48898	−0,14240531306425	0,0570784514400749	0,0531014862285547	1,103743784041	0.142405313064256	0,906007367342827	-0.142405313064256	2831
Q9JMG7	sp|Q9JMG7|HDGR3_MOUSE Hepatoma-derived growth factor-related protein 3 OS=Mus musculus GN=Hdgfrp3 PE=1 SV=2;sp|Q9JMG7-2|HDGR3_MOUSE Isoform 2 of Hepatoma-derived growth factor-related protein 3 OS=Mus musculus GN=Hdgfrp3;sp|Q9Y3E1|HDGR3_HUMAN Hepatoma-d	1,10	0,640241651904525	0,88600019248236	29,3179435122904	29,3897530370284	29,9807891911943	29,6976512816979	29,6681665776184	29,6188712147222	6901974045538,17	7618213617005,96	−0,14244369345724	0,0123039958143697	0,0013419054803332	1,10377314761576	0.142443693457249	0,905983264912795	-0.142443693457249	2832
G3XA57	sp|G3XA57|RFIP2_MOUSE Rab11 family-interacting protein 2 OS=Mus musculus GN=Rab11fip2 PE=1 SV=1;sp|G3XA57-2|RFIP2_MOUSE Isoform 2 of Rab11 family-interacting protein 2 OS=Mus musculus GN=Rab11fip2	1,10	0,619081080905992	0,870879639674993	26,4120453633107	26,924184307304	27,0626686517635	26,9302714663707	26,8636371325686	26,9015107380824	435444320915,946	480682647280,813	−0,14259646578189	0,0127885332907683	0,0012424817359723	1,10389003643384	0.1425964657819	0,905887332066641	-0.1425964657819	2833
P16332	sp|P16332|MUTA_MOUSE Methylmalonyl-CoA mutase, mitochondrial OS=Mus musculus GN=Mut PE=1 SV=2	1,10	0,411438267550095	0,765243184864724	29,2467701061378	29,0935725463537	28,8343162844237	29,1550985480598	29,151901871807	29,1644059935391	4167009072691,95	4600267045624,93	−0,14270537090982	0,00717480957480897	0,0002227981923873	1,10397336923797	0.142705370909827	0,905818951674767	-0.142705370909826	2834
O88520	sp|O88520|SHOC2_MOUSE Leucine-rich repeat protein SHOC-2 OS=Mus musculus GN=Shoc2 PE=2 SV=2	1,10	0,756346071937518	0,931775125431143	26,4330141857912	27,4581127618677	27,1281225775127	27,2232737570987	26,8991884585141	27,1940541511622	535473108698,62	591250401363,761	−0,14295513273401	0,019375926919506	0,0066138617755035	1,10416450753372	0.142955132734019	0,905662148327533	-0.142955132734019	2835
Q62447	sp|Q62447|CCNC_MOUSE Cyclin-C OS=Mus musculus GN=Ccnc PE=2 SV=4	1,10	0,70917776763762	0,916805961322165	24,4792576365056	23,677772468097	24,3767709733963	24,4418972929147	24,1582085472173	24,2310959464558	31647775308,8459	34945909571,4955	−0,14301950914606	0,0180401239606438	0,0060685110620857	1,10421377902439	0.143019509146062	0,905621736475281	-0.143019509146062	2836
Q8VCZ6	sp|Q8VCZ6|SGSM3_MOUSE Small G protein signaling modulator 3 OS=Mus musculus GN=Sgsm3 PE=1 SV=1	1,10	0,766061256787133	0,934969627468889	25,0924879023731	24,9023550022812	24,4689312575944	24,4478937746508	25,4094933692325	24,903996713159	60219258942,2329	66499567822,7277	−0,14312001026635	0,0128756877125605	0,0193018570540224	1,10429070351927	0.143120010266358	0,905558651189484	-0.143120010266358	2837

Q60900-2	sp|Q60900-2|ELAV3_MOUSE Isoform HuC-S of ELAV-like protein 3 OS=Mus musculus GN=Elavl3;sp|Q60900|ELAV3_MOUSE ELAV-like protein 3 OS=Mus musculus GN=Elavl3 PE=1 SV=1	1,10	0,489467690605588	0,806156092714947	26,387321076432	26,0564348417916	26,1422103225398	26,1377623120407	26,4907634434962	26,2550580787104	237948971044,781	262765528470,342	−0,143123808733539	0,00655529230112512	0,0068371644332443	1,10429361100658	0.143123808733543	0,90555626695013	-0.143123808733543	2838
Q3UGR5	sp|Q3UGR5|HDHD2_MOUSE Haloacid dehalogenase-like hydrolase domain-containing protein 2 OS=Mus musculus GN=Hdhd2 PE=1 SV=2;sp|Q3UGR5-2|HDHD2_MOUSE Isoform 2 of Haloacid dehalogenase-like hydrolase domain-containing protein 2 OS=Mus musculus GN=Hdhd2	1,10	0,589318656983146	0,858445887049583	30,6125340776466	30,7758103446925	31,1726430469682	31,0706333494719	30,8043490382255	30,9837659274804	25094316188977,9	27712816317498,2	−0,143192698569419	0,00933619963454367	0,0043871871031933	1,10434634316397	0.143192698569418	0,905513026950388	-0.143192698569418	2839
Q8VCE6	sp|Q8VCE6|NT5M_MOUSE 5(3)-deoxyribonucleotidase, mitochondrial OS=Mus musculus GN=Nt5m PE=2 SV=1	1,10	0,695644046296344	0,911000671130729	26,4697443055486	26,7143626330946	26,9782391770371	26,8446549836125	26,4493758498685	27,1665322140943	402428001104,246	444487461109,564	−0,143412029584694	0,00951724424152466	0,0133930346027794	1,10451424823797	0.143412029584696	0,905375373468746	-0.143412029584696	2840
Q8VCA8	sp|Q8VCA8|SCRN2_MOUSE Secernin-2 OS=Mus musculus GN=Scrn2 PE=2 SV=1	1,10	0,922176170585754	0,982113422958876	21,5941058210057	23,316746816814	20,8620051295316	23,4391885073479	21,4438630713332	21,1880773735381	3323505453,88292	3670925513,51742	−0,143438119749658	0,0574814345708921	0,0559621511663975	1,10453422281242	0.14343811974966	0,905359000514939	-0.14343811974966	2841
Q9R0M6	sp|Q9R0M6|RAB9A_MOUSE Ras-related protein Rab-9A OS=Mus musculus GN=Rab9a PE=1 SV=1	1,10	0,502942182747545	0,814723257491473	26,1633273553353	26,5703771561402	26,6308893490547	26,5822533365469	26,5056129060047	26,5758572324515	308462038290,325	340804382636,475	−0,143850937127666	0,00961203572030439	0,0016013213883192	1,10485032299407	0.143850937127662	0,905099975252817	-0.143850937127662	2842
P62715	sp|P62715|PP2AB_MOUSE Serine/threonine-protein phosphatase 2A catalytic subunit beta isoform OS=Mus musculus GN=Ppp2cb PE=1 SV=1	1,10	0,624206224403089	0,874736663342273	31,8925920404403	32,26957919978	32,5652501037116	32,4603983928778	32,2966368508442	32,26957919978	100630225556041	111183690019454	−0,143881467005982	0,0104566175150457	0,0031923868253483	1,10487370375152	0.143881467005981	0,905080822002162	-0.143881467005981	2843
Q8R5J9	sp|Q8R5J9|PRAF3_MOUSE PRA1 family protein 3 OS=Mus musculus GN=Arl6ip5 PE=1 SV=2	1,11	0,225193662611241	0,652238880554131	28,0173481149224	28,2288491072506	28,020951024883	28,1757537010239	28,1207709623458	28,2708618860744	1580953743034,78	1747362894362,51	−0,144384103313436	0,00431070073334278	0,0026937381332720	1,10525870985213	0.144384103313429	0,904765545918015	-0.144384103313429	2844
Q9D172	sp|Q9D172|ES1_MOUSE ES1 protein homolog, mitochondrial OS=Mus musculus GN=D10Jhu81e PE=1 SV=1	1,11	0,630148627668047	0,878466604204121	30,8064361728851	31,2799527489482	31,4047907964564	31,4594116268809	31,1184365708611	31,2146660379869	34216413533999,1	37831810486349,1	−0,144911271319383	0,0101289426293603	0,0056229084536357	1,10566265072631	0.14491127131938	0,904435000443488	-0.14491127131938	2845
O88958	sp|O88958|GNPI1_MOUSE Glucosamine-6-phosphate isomerase 1 OS=Mus musculus GN=Gnpda1 PE=2 SV=3	1,11	0,550079215018471	0,838418252843802	27,9117081934441	28,2028134144342	28,4487032309194	28,4053017035069	28,1788730675729	28,2810559679654	1744940568667,22	1929747432608,38	−0,145234138202101	0,00953657797743631	0,0040084728223470	1,1059101193815	0.145234138202102	0,904232615720409	-0.145234138202102	2846
Q61599	sp|Q61599|GDIR2_MOUSE Rho GDP-dissociation inhibitor 2 OS=Mus musculus GN=Arhgdib PE=1 SV=3	1,11	0,59372238672983	0,860080030359509	27,2393633429942	27,6095679757196	27,8355439715928	27,7048655608525	27,5185848493879	27,763130170804	932832752015,881	1031663358754,36	−0,145281934924591	0,0109204514126841	0,0046176813967766	1,10594675897143	0.14528193492459	0,904202658842311	-0.14528193492459	2847
Q99LG2	sp|Q99LG2|TNPO2_MOUSE Transportin-2 OS=Mus musculus GN=Tnpo2 PE=2 SV=1	1,11	0,584448353342945	0,85464274349988	30,111798004507	29,7939275700076	29,497763870862	29,97721683577	29,9281463515691	29,8014346095597	8759528090330,14	9691457393225,08	−0,145860484867116	0,0103043295629574	0,0030333448867582	1,10639035496943	0.145860484867115	0,903840127951611	-0.145860484867115	2848
P23780	sp|P23780|BGAL_MOUSE Beta-galactosidase OS=Mus musculus GN=Glb1 PE=2 SV=1	1,11	0,535217290224295	0,8300431463043	26,438944362597	26,9078396230827	26,7178490875963	26,960577018864	26,7506588434913	26,6568132883569	389531676272,455	430989565553,347	−0,14591229008108	0,00883717807878768	0,0058058163175700	1,10643008465349	0.145912290081077	0,903807672866362	-0.145912290081077	2849
Q8VDT9	sp|Q8VDT9|RM50_MOUSE 39S ribosomal protein L50, mitochondrial OS=Mus musculus GN=Mrpl50 PE=2 SV=2	1,11	0,693984455275233	0,910309912045398	24,4317373548227	24,0624940540361	23,6385289981359	23,9753136992594	24,0912088731561	24,3700164161251	27687682301,9247	30638186605,6969	−0,146086617708148	0,016507838305749	0,0084022839222980	1,10656378788221	0.146086617708145	0,903698468132459	-0.146086617708145	2850
Q8CHT0	sp|Q8CHT0|AL4A1_MOUSE Delta-1-pyrroline-5-carboxylate dehydrogenase, mitochondrial OS=Mus musculus GN=Aldh4a1 PE=1 SV=3	1,11	0,450381121091958	0,786343703431612	29,5252640648693	29,9373485722038	29,8955901225831	29,9349818756405	29,8286939050459	29,8993775648934	8628291131721,19	9551166872188,55	−0,14660214284139	0,00761515370571767	0,0018101162711787	1,10695927227982	0.146602142841389	0,903375602916684	-0.14660214284139	2851
Q9CPP6	sp|Q9CPP6|NDUA5_MOUSE NADH dehydrogenase [ubiquinone] 1 alpha subcomplex subunit 5 OS=Mus musculus GN=Ndufa5 PE=1 SV=3	1,11	0,508830418369074	0,816124555116335	26,9509270425352	26,6737413947028	26,6219754182725	26,6335512707477	26,8916424786853	27,02680358158	413896295391,826	458243150743,386	−0,146843981608596	0,00661264054897453	0,0074412608921648	1,10714484726078	0.146843981608593	0,903224182882781	-0.146843981608593	2852
Q91XL9-2	sp|Q91XL9-2|OSBL1_MOUSE Isoform 2 of Oxysterol-binding protein-related protein 1 OS=Mus musculus GN=Osbpl1a;sp|Q91XL9|OSBL1_MOUSE Oxysterol-binding protein-related protein 1 OS=Mus musculus GN=Osbpl1a PE=1 SV=2;sp|Q91XL9-3|OSBL1_MOUSE Isoform 3 of Oxyst	1,11	0,166181434108437	0,575360434338823	29,7293035998322	29,5272518601179	29,6742040431969	29,7364644042824	29,8229827561781	29,6772864366557	7482488210400,86	8285912219588,92	−0,147142436003265	0,00352344690504981	0,0024633730564851	1,10737390913243	0.147142436003265	0,903037349673019	-0.147142436003265	2853
Q6ZQK5	sp|Q6ZQK5|ACAP2_MOUSE Arf-GAP with coiled-coil, ANK repeat and PH domain-containing protein 2 OS=Mus musculus GN=Acap2 PE=1 SV=2;sp|Q6ZQK5-2|ACAP2_MOUSE Isoform 2 of Arf-GAP with coiled-coil, ANK repeat and PH domain-containing protein 2 OS=Mus musculus	1,11	0,616201388957374	0,869694684228085	28,7466735660147	28,4613395369887	28,2133967375042	28,5025208578432	28,8343162844237	28,3908102287772	2322820463319,2	2572456659811,41	−0,147269122212984	0,00937201114651367	0,0080723585071732	1,10747115432912	0.147269122212983	0,902958055468071	-0.147269122212983	2854
Q6P5G6	sp|Q6P5G6|UBXN7_MOUSE UBX domain-containing protein 7 OS=Mus musculus GN=Ubxn7 PE=1 SV=2	1,11	0,69410551019948	0,910309912045398	25,7334731810382	26,3957362099935	26,3744753513748	26,3810756525348	26,4430714013401	25,9864544591337	231511390928,173	256450244539,17	−0,147595767637764	0,0143829075316716	0,0094281231943878	1,10772192897728	0.147595767637767	0,902753636847526	-0.147595767637767	2855
O89053	sp|O89053|COR1A_MOUSE Coronin-1A OS=Mus musculus GN=Coro1a PE=1 SV=5	1,11	0,502470497584308	0,814249763846902	32,9377590356243	32,72254043549	32,6339988306786	32,6569761311727	33,0839850192138	32,8606940502048	169651271266662	187954006057556	−0,147807424559857	0,00476793952116302	0,0064982437339834	1,10788445411721	0.147807424559858	0,902621204119005	-0.147807424559858	2856

Q7SIG6-2	sp|Q7SIG6-2|ASAP2_MOUSE Isoform 2 of Arf-GAP with SH3 domain, ANK repeat and PH domain-containing protein 2 OS=Mus musculus GN=Asap2;sp|Q7SIG6|ASAP2_MOUSE Arf-GAP with SH3 domain, ANK repeat and PH domain-containing protein 2 OS=Mus musculus GN=Asap2 PE=	1,11	0,991783196604169	0,998824923316247	0	0	21,0373734260528	21,3460209259128	0	0	1110,38025607731	1230,70235858872	−0,14842807247693	1,73205080756888	1,73205080756888	1,10836116893548	0.148428072476937	0,902232979670741	-0.148428072476937	2857
Q8BMF3	sp|Q8BMF3|MAON_MOUSE NADP-dependent malic enzyme, mitochondrial OS=Mus musculus GN=Me3 PE=1 SV=2	1,11	0,587920616319886	0,857234718109849	29,6815604610594	30,1787354002317	30,2434645802855	30,0036031524467	30,1824637696736	30,2268071384523	11062551856655	12263208131230,5	−0,14865222839895	0,0102361347143848	0,003920838786861	1,10853339176469	0.148652228398951	0,902092807874816	-0.148652228398951	2858
Q8BKC8-2	sp|Q8BKC8-2|PI4KB_MOUSE Isoform 2 of Phosphatidylinositol 4-kinase beta OS=Mus musculus GN=Pi4kb;sp|Q8BKC8|PI4KB_MOUSE Phosphatidylinositol 4-kinase beta OS=Mus musculus GN=Pi4kb PE=1 SV=2;sp|Q8BKC8-3|PI4KB_MOUSE Isoform 3 of Phosphatidylinositol 4-kina	1,11	0,938463358985743	0,985646114383563	24,0503294468235	26,9128048037016	26,7455391951909	26,9881320866984	26,9456779435363	24,0841927204206	177616247780,763	196907697816,887	−0,14875595141256	0,0620217961238891	0,064003307741346	1,10861309298649	0.148755951412557	0,902027953960114	-0.148755951412557	2859
Q8BVI5	sp|Q8BVI5|STX16_MOUSE Syntaxin-16 OS=Mus musculus GN=Stx16 PE=1 SV=3	1,11	0,817718452049178	0,952267950351507	27,6817373259402	26,1321115412	26,8101342985368	26,9435946391298	27,0100387989245	26,9807462280019	469371806271,114	520536868503,195	−0,14926916393547	0,0289054979395822	0,0012343130452451	1,1090075320854	0.149269163935469	0,90170713098727	-0.14926916393547	2860
Q8BTW3	sp|Q8BTW3|EXOS6_MOUSE Exosome complex component MTR3 OS=Mus musculus GN=Exosc6 PE=1 SV=1	1,11	0,73596218555997	0,924701393764781	23,3895979642284	23,735889620616	24,0448449514442	24,1495233367792	23,9779430843893	23,3535376645311	20089119333,764	22281027352,1327	−0,14940143456025	0,0138175861054491	0,0175813274439703	1,10910921389594	0.149401434560251	0,901624463552445	-0.149401434560251	2861
Q8VCN5	sp|Q8VCN5|CGL_MOUSE Cystathionine gamma-lyase OS=Mus musculus GN=Cth PE=1 SV=1	1,11	0,536412679157853	0,830981962695391	25,3917468837871	24,9531426386628	25,1428879762118	25,484150064461	25,1715068365119	25,1428879762118	84717878749,1086	93964381362,1555	−0,14944751862174	0,00874174819412618	0,0074925366451892	1,10914464277877	0.149447518621749	0,901595663388564	-0.149447518621749	2862
Q60973	sp|Q60973|RBBP7_MOUSE Histone-binding protein RBBP7 OS=Mus musculus GN=Rbbp7 PE=1 SV=1	1,11	0,798295610881899	0,944726847417452	26,4086406469355	26,1149474128652	27,1551566172011	26,9128048037016	26,9395921810098	26,1377623120407	342515092373,056	379980750669,424	−0,14975877585795	0,0201915806420853	0,0170796325533488	1,10938396330741	0.149758775857952	0,901401167742409	-0.149758775857952	2863
Q9D880	sp|Q9D880|TIM50_MOUSE Mitochondrial import inner membrane translocase subunit TIM50 OS=Mus musculus GN=Timm50 PE=1 SV=1	1,11	0,786628619115858	0,941831485187835	26,8316021926563	25,8175097401103	25,9187584767025	25,9504924356993	26,6674061630996	26,2614070104605	236517996350,752	262391072185,212	−0,14976867276520	0,0213277043217356	0,0136730255392447	1,10939157372233	0.149768672765211	0,901394984139563	-0.149768672765211	2864
Q9QZX7	sp|Q9QZX7|SRR_MOUSE Serine racemase OS=Mus musculus GN=Srr PE=1 SV=1;sp|Q9QZX7-2|SRR_MOUSE Isoform 2 of Serine racemase OS=Mus musculus GN=Srr	1,11	0,23798538467414	0,662708994500566	30,8724847418601	30,6563201848299	30,6103162203833	30,8286913985065	30,8709415115779	30,7513155117469	21802382796838,3	24190541057167,9	−0,14995722096912	0,00455785559346731	0,0019686031653651	1,10953657141897	0.149957220969122	0,901277187034146	-0.149957220969122	2865
Q80YD1	sp|Q80YD1|SUV3_MOUSE ATP-dependent RNA helicase SUPV3L1, mitochondrial OS=Mus musculus GN=Supv3l1 PE=2 SV=1	1,11	0,641248727306616	0,886845817563903	27,4280292367288	26,8091718647215	26,7912227971815	26,9639504293006	27,2306522722493	27,1464541510055	537113158584,532	596106761210,892	−0,15034467074798	0,0134245484942697	0,0050283494209184	1,10983458826782	0.150344670747988	0,9010351727826	-0.150344670747988	2866
P97364	sp|P97364|SPS2_MOUSE Selenide, water dikinase 2 OS=Mus musculus GN=Sephs2 PE=1 SV=3	1,11	0,545623714358362	0,835985869293933	25,0100063220039	24,4296234518414	24,7832884827572	24,7747333955639	24,8859998394022	24,875004184539	55573524803,666	61681248434,3534	−0,15043421833824	0,0118223408679561	0,002467771032160	1,10990347746099	0.15043421833824	0,900979247571687	-0.15043421833824	2867
Q7M6Y3-5	sp|Q7M6Y3-5|PICAL_MOUSE Isoform 5 of Phosphatidylinositol-binding clathrin assembly protein OS=Mus musculus GN=Picalm;sp|Q7M6Y3-2|PICAL_MOUSE Isoform 2 of Phosphatidylinositol-binding clathrin assembly protein OS=Mus musculus GN=Picalm;sp|Q7M6Y3-3|PICAL	1,11	0,148342445794516	0,546867809763809	29,8931980979023	30,0419786001201	30,0740303655199	30,1667006838448	30,1381873875547	30,0171565154699	10719321977257,6	11897485552654,1	−0,15044304783598	0,003216042961075	0,0026371873508651	1,10991027024807	0.150443047835986	0,900973733468105	-0.150443047835986	2868
Q9QZM0	sp|Q9QZM0|UBQL2_MOUSE Ubiquilin-2 OS=Mus musculus GN=Ubqln2 PE=1 SV=2	1,11	0,0758950350744257	0,41058886267723	29,6954546184483	29,6166517537297	29,5156147134624	29,7592429065261	29,7200994190062	29,6629732680634	7229856918131,93	8029193458951,06	−0,15128797883931	0,003044614917281	0,0016293249265514	1,1105604923957	0.151287978839315	0,900446222287991	-0.151287978839314	2869
Q9CSN1	sp|Q9CSN1|SNW1_MOUSE SNW domain-containing protein 1 OS=Mus musculus GN=Snw1 PE=1 SV=3	1,11	0,662046718220655	0,895509921375283	24,6502909577635	25,2310892455359	24,817993141014	25,3314691252537	24,7822925530271	24,9004151407677	65139119181,3551	72345971398,3223	−0,15138847061828	0,0120046968059519	0,0115604602887362	1,11063785183988	0.151388470618287	0,900383503356562	-0.151388470618287	2870
Q62421	sp|Q62421|SH3G3_MOUSE Endophilin-A3 OS=Mus musculus GN=Sh3gl3 PE=2 SV=1	1,11	0,529848356720836	0,829480190327658	28,9252452807151	28,3886961162417	28,5376790926525	28,6537286050758	28,8212517944698	28,6927585562291	2680988886439,94	2978913199952,67	−0,15202084782343	0,00967859209328361	0,0030516966008900	1,11112478497005	0.152020847823435	0,899988924310563	-0.152020847823435	2871
Q96PR1-5	sp|Q96PR1-5|KCNC2_HUMAN Isoform 5 of Potassium voltage-gated channel subfamily C member 2 OS=Homo sapiens GN=KCNC2;sp|Q96PR1|KCNC2_HUMAN Potassium voltage-gated channel subfamily C member 2 OS=Homo sapiens GN=KCNC2 PE=1 SV=1;sp|Q14B80|KCNC2_MOUSE Potass	1,11	0,761096843855121	0,933389703428013	24,2034797259004	23,6458420059548	23,3393648146177	23,4691216538316	24,2820927735618	23,754344369546	20212401369,466	22464143789,3049	−0,15238334144783	0,0184621472600404	0,0173051170931874	1,1140400285344	0.152383341447836	0,899762820209914	-0.152383341447836	2872
Q920N2	sp|Q920N2|BPL1_MOUSE Biotin--protein ligase OS=Mus musculus GN=Hlcs PE=2 SV=1	1,11	0,384404991030644	0,751739350763028	25,4719455493022	25,2470629747378	25,0703734773173	25,3890942487746	25,4178300813803	25,2995911794629	93677824546,1346	104122976430,728	−0,15250897988906	0,00796684556515488	0,0024308206858949	1,11150079472063	0.152508979889057	0,899684466938549	-0.152508979889056	2873

Q62393-3	sp|Q62393-3|TPD52_MOUSE Isoform 3 of Tumor protein D52 OS=Mus musculus GN=Tpd52;sp|Q62393-2|TPD52_MOUSE Isoform 2 of Tumor protein D52 OS=Mus musculus GN=Tpd52;sp|Q62393|TPD52_MOUSE Tumor protein D52 OS=Mus musculus GN=Tpd52 PE=1 SV=2	1,11	0,621141588869929	0,872327249963059	28,6363686210369	29,1365569873341	29,2745375866608	29,2784329903639	28,963339565092	29,1231989742319	3994026816159,19	4439918678547,97	−0,152689233283049	0,0115722853542552	0,0054101444075889	1,1163967667537	0.152689233283046	0,899572065465261	-0.152689233283046	2874
Q9CY73	sp|Q9CY73|RM44_MOUSE 39S ribosomal protein L44, mitochondrial OS=Mus musculus GN=Mrpl44 PE=2 SV=3;sp|Q9CY73-2|RM44_MOUSE Isoform 2 of 39S ribosomal protein L44, mitochondrial OS=Mus musculus GN=Mrpl44	1,11	0,725557352188138	0,921719793333881	25,1249989581468	25,9896298932137	26,0393274349537	25,9200419149704	25,7062486810014	25,8471838387831	147631542487,49	164223062571,379	−0,153655749409814	0,0199915416179364	0,0042087201976153	1,11238465577432	0.153655749409816	0,898969609845891	-0.153655749409816	2875
Q9WV92-8	sp|Q9WV92-8|E41L3_MOUSE Isoform 8 of Band 4.1-like protein 3 OS=Mus musculus GN=Epb41l3;sp|Q9WV92-6|E41L3_MOUSE Isoform 6 of Band 4.1-like protein 3 OS=Mus musculus GN=Epb41l3;sp|Q9WV92-4|E41L3_MOUSE Isoform 4 of Band 4.1-like protein 3 OS=Mus musculus	1,11	0,782002703781226	0,940251150142017	30,1011524211797	28,8859884835841	29,2720468450267	29,8120687934487	29,3648762637523	29,4026825687162	5981719892193,09	6656018121150,65	−0,154099006730354	0,021104792076084	0,0083990353477083	1,1127264801947	0.154099006730354	0,898693450545926	-0.154099006730354	2876
Q6P8J2	sp|Q6P8J2|SAT2_MOUSE Diamine acetyltransferase 2 OS=Mus musculus GN=Sat2 PE=1 SV=1	1,11	0,559752255475951	0,842973036472956	24,4713782819228	24,2549465990353	23,9115536096075	24,3939467194112	24,4164001122609	24,1495233367792	32764982113,0673	36477326867,7881	−0,154845265631105	0,0116592712867635	0,0060866057179904	1,1330220605371	0.154845265631104	0,898228706062366	-0.154845265631104	2877
Q9Z2D0	sp|Q9Z2D0|MTMR9_MOUSE Myotubularin-related protein 9 OS=Mus musculus GN=Mtmr9 PE=2 SV=2	1,11	0,599668366391022	0,861904596278927	27,572598641113	28,1567892511131	28,130530652555	28,2028134144342	28,0297486856985	27,9495817820908	1380278189800,42	1536786443957,87	−0,154957632125693	0,0118041032808054	0,0046123065985359	1,1338892066394	0.154957632125693	0,898158748879659	-0.154957632125693	2878
Q5XJY5	sp|Q5XJY5|COPD_MOUSE Coatomer subunit delta OS=Mus musculus GN=Arcn1 PE=1 SV=2	1,11	0,505429440954576	0,814993646406912	29,3608168934757	29,7814723531849	29,7971159967527	29,8904491527376	29,6323404854074	29,7389386091483	7504083196098,16	8355235094530,77	−0,155004599754075	0,00834855256448569	0,0043592238210522	1,1134251681638	0.155004599754075	0,898129509367156	-0.155004599754075	2879
Q60598	sp|Q60598|SRC8_MOUSE Src substrate cortactin OS=Mus musculus GN=Cttn PE=1 SV=2	1,11	0,629697096755755	0,878363590096611	30,2374046939875	30,8043490382255	30,5331676346361	30,8467694077559	30,7106727634419	30,3400376693966	18064572016704,9	20115127909033,7	−0,155117836823069	0,00928947481595793	0,0085613712858889	1,11351256428509	0.155117836823074	0,89805901798875	-0.155117836823074	2880
O88811	sp|O88811|STAM2_MOUSE Signal transducing adapter molecule 2 OS=Mus musculus GN=Stam2 PE=1 SV=1;sp|O88811-2|STAM2_MOUSE Isoform 2 of Signal transducing adapter molecule 2 OS=Mus musculus GN=Stam2	1,11	0,808372226167496	0,94879038498679	23,8239823554939	25,2204546504586	24,6992019327018	24,5893142582617	24,4391865491359	25,0392995070299	47368011717,6873	52773065345,9203	−0,155888669758567	0,0287078294099724	0,0126489281737204	1,11410767377037	0.155888669758566	0,897579312613289	-0.155888669758566	2881
Q9DBQ7-3	sp|Q9DBQ7-3|PACE1_MOUSE Isoform 3 of Protein-associating with the carboxyl-terminal domain of ezrin OS=Mus musculus GN=Scyl3;sp|Q9DBQ7|PACE1_MOUSE Protein-associating with the carboxyl-terminal domain of ezrin OS=Mus musculus GN=Scyl3 PE=2 SV=3	1,11	0,424572342256095	0,7711031271846	23,4032259601932	23,2704101887496	22,9673450736272	23,29481600134	23,2674949779094	23,4032259601932	12066036084,131	13444630533,971	−0,156078307741455	0,00962421074911708	0,0030781915855963	1,11425412954409	0.156078307741452	0,897461336229608	-0.156078307741452	2882
P59823	sp|P59823|IRPL1_MOUSE Interleukin-1 receptor accessory protein-like 1 OS=Mus musculus GN=Il1rapl1 PE=1 SV=1	1,11	0,559939128655202	0,842973036472956	23,2165788422459	23,1262887967585	22,6546864138481	23,2345232311874	23,0613359467876	23,02646262069	9736861592,85028	10850105056,9585	−0,156180272108119	0,0131196199457631	0,0048222346561855	1,11433288370101	0.156180272108122	0,897397909212477	-0.156180272108122	2883
Q62442-2	sp|Q62442-2|VAMP1_MOUSE Isoform 2 of Vesicle-associated membrane protein 1 OS=Mus musculus GN=Vamp1;sp|Q62442|VAMP1_MOUSE Vesicle-associated membrane protein 1 OS=Mus musculus GN=Vamp1 PE=1 SV=1	1,11	0,442259048885109	0,782007722200821	28,1173618978386	27,9035914445976	27,8080490843163	27,889164479335	28,0108060548788	28,2538132214311	1366126950792,71	1522327077193,04	−0,156186804188814	0,00566787675202408	0,0066185979878879	1,11433792906998	0.156186804188814	0,897393846079161	-0.156186804188814	2884
Q91YH5	sp|Q91YH5|ATLA3_MOUSE Atlastin-3 OS=Mus musculus GN=Atl3 PE=2 SV=1;sp|Q91YH5-2|ATLA3_MOUSE Isoform 2 of Atlastin-3 OS=Mus musculus GN=Atl3	1,11	0,677334407488886	0,902913619850824	26,4706027605894	27,2818929651463	27,1512361220385	27,2152024674969	27,0138855393607	27,0003491513743	515246166146,066	574335210221,975	−0,156631145396159	0,0161528186927949	0,0044440231427845	1,1146811911 6321	0.156631145396159	0,897117496848101	-0.156631145396159	2885
Q9QZF2	sp|Q9QZF2|GPC1_MOUSE Glypican-1 OS=Mus musculus GN=Gpc1 PE=1 SV=1	1,11	0,431070303150831	0,775108167118334	26,8093381012677	26,7655997645261	26,513257270011	26,76973984521	26,9970599843299	26,647166109089	392603111948,179	437636858334,321	−0,156662640566899	0,00598663134893376	0,0066235075130904	1,1147055257985	0.1566626405669	0,897097912279272	-0.1566626405669	2886
Q8BMI3	sp|Q8BMI3|GGA3_MOUSE ADP-ribosylation factor-binding protein GGA3 OS=Mus musculus GN=Gga3 PE=2 SV=2	1,11	0,5293739141658	0,829460904757119	28,62011627812	28,2371702788332	28,0488815319204	28,3308483775731	28,4585732117269	28,4428094286615	1956260834504,75	2180867114640,63	−0,156803123604341	0,0102852105178167	0,0024511445189696	1,11481407600369	0.156803123604343	0,897010561245089	-0.156803123604343	2887
Q9QUM9	sp|Q9QUM9|PSA6_MOUSE Proteasome subunit alpha type-6 OS=Mus musculus GN=Psma6 PE=1 SV=1	1,12	0,620017451163898	0,871287681372426	30,1999089850511	30,7106727634419	30,8064361728851	30,8170158313743	30,4559240288616	30,7710464061561	18940797455933,3	21121841336592,7	−0,157238536626406	0,0106654868440868	0,0064063306948225	1,11515058358729	0.157238536626404	0,896739879544458	-0.157238536626404	2888
Q8K0T4	sp|Q8K0T4|KATL1_MOUSE Katanin p60 ATPase-containing subunit A-like 1 OS=Mus musculus GN=Katnal1 PE=1 SV=1	1,12	0,530143651055417	0,829480190327658	26,8740206459646	27,3476415937145	27,1253677426794	27,4149626112445	27,2152024674969	27,0441476199735	597294817908,32	666143466578,732	−0,157389717285319	0,00873882485234044	0,0068170483526506	1,11526744683892	0.157389717285319	0,896645914694602	-0.157389717285319	2889
P42225	sp|P42225|STAT1_MOUSE Signal transducer and activator of transcription 1 OS=Mus musculus GN=Stat1 PE=1 SV=1	1,12	0,611173696898916	0,866640865424611	26,9252720505903	26,7968413620251	26,3210361882821	26,5703771561402	26,9261638707406	26,8740206459646	386752142819,929	431350673688,5	−0,157451924175558	0,0119307636804128	0,0071719609113007	1,11531553656921	0.157451924175558	0,896607253473822	-0.157451924175558	2890
Q9R1Q8	sp|Q9R1Q8|TAGL3_MOUSE Transgelin-3 OS=Mus musculus GN=Tagln3 PE=1 SV=1	1,12	0,390799581453117	0,753542608417884	31,8186731138519	31,8186731138519	31,6520009366872	31,6520009366872	31,9282291432254	32,0369458698543	62308297650433,6	69503066043194,1	−0,157652320974209	0,00302955878750347	0,0062265174242384	1,11547046964957	0.157652320974209	0,896482719362493	-0.157652320974209	2891

Q8R0H9	sp|Q8R0H9|GGA1_MOUSE ADP-ribosylation factor-binding protein GGA1 OS=Mus musculus GN=Gga1 PE=1 SV=1;sp|Q9UJY5-4|GGA1_HUMAN Isoform 4 of ADP-ribosylation factor-binding protein GGA1 OS=Homo sapiens GN=GGA1;sp|Q9UJY5|GGA1_HUMAN ADP-ribosylation factor-bin	1,12	0,587161946363534	0,856921465604777	26,9150426236507	26,6885628808377	26,2893784167721	26,7080610722193	26,8999307211861	26,613068974371	367869701505,151	410381720557,377	−0,15777161316632	0,0118951582151956	0,0054648439587534	1,11556270842172	0.157771613166323	0,896408594918686	-0.157771613166322	2892
Q811Q9-2	sp|Q811Q9-2|PCY1B_MOUSE Isoform 2 of Choline-phosphate cytidylyltransferase B OS=Mus musculus GN=Pcyt1b;sp|Q811Q9|PCY1B_MOUSE Choline-phosphate cytidylyltransferase B OS=Mus musculus GN=Pcyt1b PE=1 SV=2	1,12	0,393383230145853	0,753881747531964	25,7580916626282	25,3363157103993	25,5256101773492	25,6369206672453	25,7246189853418	25,5872669040152	123561624222,788	137873465599,594	−0,158114089593458	0,00827157836025085	0,0027114902630649	1,1582755946135	0.158114089593453	0,896195824812515	-0.158114089593453	2893
Q9QZH3	sp|Q9QZH3|PPIE_MOUSE Peptidyl-prolyl cis-trans isomerase E OS=Mus musculus GN=Ppie PE=2 SV=2	1,12	0,617108082338105	0,870166479711399	24,2505125478808	24,4554828608451	24,8891462278101	24,6602962520587	24,8260555298346	24,4377243018323	45080421622,9216	50304415554,1092	−0,15818403191264	0,0132917726004204	0,0079077223089734	1,1588165645131	0.158184031912641	0,896152378004104	-0.158184031912641	2894
Q9D6F9	sp|Q9D6F9|TBB4A_MOUSE Tubulin beta-4A chain OS=Mus musculus GN=Tubb4a PE=1 SV=3	1,12	0,225473783850346	0,652238880554131	33,8956357977178	33,6211455554601	33,7181235415771	33,7804120503292	33,9275094387217	33,8575362770501	452119796998424	504784432987836	−0,158962329414178	0,00412506625089413	0,0021733075563779	1,1648380880255	0.158962329414178	0,895669056833452	-0.158962329414178	2895
Q9Z1B3-2	sp|Q9Z1B3-2|PLCB1_MOUSE Isoform B of 1-phosphatidylinositol 4,5-bisphosphate phosphodiesterase beta-1 OS=Mus musculus GN=Plcb1;sp|Q9Z1B3-3|PLCB1_MOUSE Isoform C of 1-phosphatidylinositol 4,5-bisphosphate phosphodiesterase beta-1 OS=Mus musculus GN=Plcb1;	1,12	0,698883754536603	0,912827852392638	25,6863781120229	25,5562998340598	26,0218395960174	26,2087814532622	26,0052225567599	25,3812269421496	153173818281,166	171025239856,527	−0,159039532221906	0,00932648070499048	0,0166715205124313	1,11654355669709	0.15903953222191	0,895621128259568	-0.159039532221909	2896
Q9D906	sp|Q9D906|ATG7_MOUSE Ubiquitin-like modifier-activating enzyme ATG7 OS=Mus musculus GN=Atg7 PE=1 SV=1	1,12	0,468567826554457	0,796565305142577	28,991263159107	28,796752241005	28,4901679344148	28,8948809578225	28,9293487073306	28,7849374021746	3090624978627,64	3451128364856,56	−0,159169529975557	0,00878418386843212	0,0026124706183223	1,1166441702639	0.159169529975555	0,895540429646145	-0.159169529975555	2897
Q8K3H0	sp|Q8K3H0|DP13A_MOUSE DCC-interacting protein 13-alpha OS=Mus musculus GN=Appl1 PE=1 SV=1	1,12	0,431164467423429	0,775108167118334	30,348015515816	30,1415815593921	29,8687792032057	30,2640745692772	30,2413049199915	30,1853711148321	12042446820684,2	13453362643081	−0,159838263795802	0,00798098241556557	0,0013397027673696	1,11716188939057	0.159838263795804	0,895125415122707	-0.159838263795803	2898
Q9Z2D3	sp|Q9Z2D3|DFNA5_MOUSE Non-syndromic hearing impairment protein 5 homolog OS=Mus musculus GN=Dfna5 PE=2 SV=1	1,12	0,534711026869279	0,8300431463043	26,6055017936493	27,1097993894113	27,0584803792591	27,1403798463766	26,8812927262519	27,0846207130066	493403854981,285	551237228514,647	−0,15990433808815	0,0103076975595853	0,0050440497754536	1,11721305569361	0.159904338088152	0,895084420025116	-0.159904338088152	2899
Q60989	sp|Q60989|XIAP_MOUSE E3 ubiquitin-protein ligase XIAP OS=Mus musculus GN=Xiap PE=1 SV=2	1,12	0,462677186488002	0,793595716606975	22,7406104302362	23,1493509179357	22,8927270958949	22,9194574103533	23,220546028412	22,9752239618567	9063877220,03976	10126373889,6716	−0,159917434508215	0,00901033192397301	0,0069527683183909	1,1172231975167	0.159917434508209	0,895076294712411	-0.159917434508209	2900
Q8CCF0-3	sp|Q8CCF0-3|PRP31_MOUSE Isoform 3 of U4/U6 small nuclear ribonucleoprotein Prp31 OS=Mus musculus GN=Prpf31;sp|Q8CCF0|PRP31_MOUSE U4/U6 small nuclear ribonucleoprotein Prp31 OS=Mus musculus GN=Prpf31 PE=1 SV=3;sp|Q8CCF0-2|PRP31_MOUSE Isoform 2 of U4/U6 s	1,12	0,730774531370034	0,924701393764781	25,7162865377133	25,7127945872499	26,0840270683502	26,2677834736084	25,35089230623	26,2285373005935	166407049813,622	186010884322,542	−0,160670487927534	0,00825654977239916	0,0199779595922179	1,11780651439273	0.160670487927531	0,894609207518593	-0.160670487927531	2901
Q8BGY2	sp|Q8BGY2|IF5A2_MOUSE Eukaryotic translation initiation factor 5A-2 OS=Mus musculus GN=Eif5a2 PE=2 SV=3	1,12	0,466303779492909	0,795722853632858	25,8115297761429	25,5599556845734	25,8825371944796	25,9677083343354	25,6281918452435	25,9923002404933	152638907165,455	170624909649,267	−0,160705534720786	0,00658235157625761	0,0078681103131497	1,11783366913336	0.160705534720783	0,894587475411511	-0.160705534720783	2902
Q99N94	sp|Q99N94|RM09_MOUSE 39S ribosomal protein L9, mitochondrial OS=Mus musculus GN=Mrpl9 PE=2 SV=2	1,12	0,72428263199527	0,92164812633208	23,6813632656051	24,1219424658587	24,6730051571631	24,0770833167058	24,2039362195013	24,5308608808026	31047070598,1146	34721565175,7754	−0,161374831490497	0,0205658512931809	0,0096464102145375	1,11835237614604	0.161374831490496	0,894172553597197	-0.161374831490496	2903
P61087	sp|P61087|UBE2K_MOUSE Ubiquitin-conjugating enzyme E2 K OS=Mus musculus GN=Ube2k PE=1 SV=3	1,12	0,365291143154488	0,739315231400715	30,0350880404903	30,2476801384455	30,4517424809733	30,4016042683483	30,3043185118729	30,3642246304452	13651040839580,4	15267016051001,1	−0,161407158619173	0,00688850702970367	0,0016166313962610	1,11837743586081	0.161407158619169	0,89415251768765	-0.161407158619169	2904
Q99KB8	sp|Q99KB8|GLO2_MOUSE Hydroxyacylglutathione hydrolase, mitochondrial OS=Mus musculus GN=Hagh PE=1 SV=2	1,12	0,63964321041971	0,885863059323783	28,8240709718089	29,3586082207491	29,541802057065	29,531702480219	29,1702498422972	29,3586082207491	5005176285103,4	5598502017933,66	−0,161619976761072	0,0127532022039894	0,0061587171403084	1,11854242468864	0.161619976761072	0,894020627137462	-0.161619976761072	2905
O54916-3	sp|O54916-3|REPS1_MOUSE Isoform 3 of RalBP1-associated Eps domain-containing protein 1 OS=Mus musculus GN=Reps1;sp|O54916-4|REPS1_MOUSE Isoform 4 of RalBP1-associated Eps domain-containing protein 1 OS=Mus musculus GN=Reps1;sp|O54916|REPS1_MOUSE RalBP1-	1,12	0,76358557799021	0,933454063694469	25,8263359153978	24,6135674323953	25,4007405161479	25,4561566173449	25,549542709295	25,1715068365119	95292278507,1132	106605618576,302	−0,161852253340554	0,0243392765374168	0,0077549534104525	1,11872252659322	0.161852253340554	0,893876699743631	-0.161852253340554	2906
Q3UPH1	sp|Q3UPH1|PRRC1_MOUSE Protein PRRC1 OS=Mus musculus GN=Prrc1 PE=2 SV=1	1,12	0,473801147387177	0,799218469891531	26,1892833237637	26,7178490875963	26,4722972144417	26,4967677565846	26,596578656695	26,6246811115036	309991239784,683	347029574231,509	−0,16283116990538	0,00999644555963353	0,0025297884190769	1,11948187462508	0.16283116990538	0,893270380402458	-0.16283116990538	2907
Q91ZR1	sp|Q91ZR1|RAB4B_MOUSE Ras-related protein Rab-4B OS=Mus musculus GN=Rab4b PE=2 SV=2	1,12	0,214732990689879	0,646613851007495	28,7572558211707	28,6821011242862	28,8657160830252	28,787551864115	28,8294358832546	29,0269449893171	3118451486646,58	3491354592514,81	−0,162957073527943	0,00320871784362242	0,0044267257406597	1,11957957578145	0.162957073527945	0,893192428329192	-0.162957073527945	2908
Q62172	sp|Q62172|RBP1_MOUSE RalA-binding protein 1 OS=Mus musculus GN=Ralbp1 PE=1 SV=4	1,12	0,609808251487981	0,866608542040832	27,7288300481742	27,1684490497881	27,0856899088401	27,5261939943834	27,2596824498093	27,5359993615751	738329719084,801	826631849152,173	−0,162979719396946	0,0128032261898656	0,0057133448007349	1,11959714987069	0.162979719396947	0,893178408068915	-0.162979719396947	2909
Q9EQI8	sp|Q9EQI8|RM46_MOUSE 39S ribosomal protein L46, mitochondrial OS=Mus musculus GN=Mrpl46 PE=1 SV=1	1,12	0,497342787555489	0,809698318764119	25,30131699854	25,4312369546124	25,8243737283388	25,7084262444795	25,5244369585765	25,663480608275	120990261257,693	135483351665,061	−0,163224655772551	0,0106721735508353	0,0037422288430395	1,11978724780584	0.163224655772547	0,89302677982755	-0.163224655772547	2910

Q61831-2	sp|Q61831-2|MK10_MOUSE Isoform Alpha-1 of Mitogen-activated protein kinase 10 OS=Mus musculus GN=Mapk10;sp|Q61831|MK10_MOUSE Mitogen-activated protein kinase 10 OS=Mus musculus GN=Mapk10 PE=1 SV=2	1,12	0,467807135062487	0,796164546189264	29,7592429065261	29,2904693642646	29,450672574706	29,6772864366557	29,6954546184483	29,4671293506286	6482506012346,71	7259195567447,73	−0,163258044735279	0,00807684046322261	0,0042853900616589	1,1981316386313	0.163258044735279	0,893006112332348	-0.163258044735279	2911
Q9DCJ5	sp|Q9DCJ5|NDUA8_MOUSE NADH dehydrogenase [ubiquinone] 1 alpha subcomplex subunit 8 OS=Mus musculus GN=Ndufa8 PE=1 SV=3	1,12	0,568708944780736	0,846628370075355	26,9494249747504	26,7440322826555	26,69891974877	26,5713233590086	26,9612195741421	27,1995228618436	434498792268,438	486590429176,052	−0,16335577702459	0,00498276283106741	0,0117846687107273	1,1988902577992	0.16335577702459	0,892945619592598	-0.16335577702459	2912
P39053-4	sp|P39053-4|DYN1_MOUSE Isoform 4 of Dynamin-1 OS=Mus musculus GN=Dnm1	1,12	0,408615827320836	0,76291790520755	31,4086456757793	31,2146660379869	31,0315490815031	31,2366860347337	31,4973921613793	31,2604931538048	36135140604917,4	40467641011330,7	−0,16336624417636	0,00604050990056892	0,0046004335298155	1,1989715091419	0.163366244176361	0,892939141048518	-0.163366244176361	2913
O54692	sp|O54692|ZW10_MOUSE Centromere/kinetochore protein zw10 homolog OS=Mus musculus GN=Zw10 PE=2 SV=3	1,12	0,470001979481861	0,796715861328205	27,5162976633754	27,5100018761904	27,1097993894113	27,5668665706527	27,3309114068393	27,5780928315527	776994915078,681	870172179380,834	−0,16339573546905	0,00850645679544303	0,0050772096061902	1,1992004386891	0.163395735469059	0,89292088794604	-0.163395735469059	2914
Q9CPQ8	sp|Q9CPQ8|ATP5L_MOUSE ATP synthase subunit g, mitochondrial OS=Mus musculus GN=Atp5l PE=1 SV=1	1,12	0,563563897664215	0,845110794140252	28,5681116849645	28,6558298268788	28,187447373342	28,3623185321326	28,5655362258062	28,8233279788742	2315074447572,92	2592717265497,02	−0,1634063015559	0,00874562310682181	0,0080830068864200	1,1992824602905	0.163406301555897	0,89291434834840	-0.163406301555897	2915
O55125	sp|O55125|NIPS1_MOUSE Protein NipSnap homolog 1 OS=Mus musculus GN=Nipsnap1 PE=1 SV=1	1,12	0,554048067729296	0,839722577101535	30,239302803755	30,6330421420719	30,836402611215	30,836402611215	30,6260640465842	30,5864926907295	18888653598480,1	21156883924127,1	−0,163607288143335	0,00993033246513444	0,0043779129401111	1,12008427778195	0.163607288143341	0,89278996218056	-0.163607288143341	2916
P35803-8	sp|P35803-8|GPM6B_MOUSE Isoform 8 of Neuronal membrane glycoprotein M6-b OS=Mus musculus GN=Gpm6b;sp|P35803-3|GPM6B_MOUSE Isoform 3 of Neuronal membrane glycoprotein M6-b OS=Mus musculus GN=Gpm6b;sp|P35803|GPM6B_MOUSE Neuronal membrane glycoprotein M6-b	1,12	0,141259930158062	0,532098608288336	29,9373485722038	30,1584841687995	29,9980024503029	30,1905445773157	30,1787354002317	30,0650549662063	11026012801259,8	12351249087600,5	−0,16374576809329	0,00380475184086778	0,0022987221149606	1,12019179645695	0.163745768093304	0,89270427007491	-0.163745768093304	2917
Q61701	sp|Q61701|ELAV4_MOUSE ELAV-like protein 4 OS=Mus musculus GN=Elavl4 PE=1 SV=1;sp|Q61701-2|ELAV4_MOUSE Isoform 2 of ELAV-like protein 4 OS=Mus musculus GN=Elavl4	1,12	0,407613542403471	0,762322978561746	27,6583684625279	27,2435367500068	27,2849017576652	27,4696095384182	27,4741157907615	27,5836630656943	790239833781,919	885244278665,062	−0,16378504413206	0,00834075864573037	0,0023478497870729	1,12022229305814	0.16378504413206	0,89267996735724	-0.16378504413206	2918
Q9D882	sp|Q9D882|CJ035_MOUSE Uncharacterized protein C10orf35 homolog OS=Mus musculus PE=2 SV=1	1,12	0,431526401918491	0,775108167118334	27,0647658758816	26,7646133507678	26,675228782357	26,982191551991	26,7900481326491	27,072984791289	451061412289,624	505294951774,425	−0,16380189589148	0,00760394104439372	0,0053606885942455	1,12023537817058	0.163801895891485	0,89266954024703	-0.163801895891485	2919
Q9R0Q3	sp|Q9R0Q3|TMED2_MOUSE Transmembrane emp24 domain-containing protein 2 OS=Mus musculus GN=Tmed2 PE=1 SV=1	1,12	0,469578567118393	0,796715861328205	27,1253677426794	27,0068439722442	26,6710608514719	26,9096572194275	27,1854010346971	27,0501953007888	498278098484,519	558442698707,44	−0,16445809207107	0,00874912405364781	0,0050975440124835	1,12074502252037	0.16445809207107	0,8922636102823	-0.16445809207107	2920
Q8CA95-4	sp|Q8CA95-4|PDE10_MOUSE Isoform 4 of cAMP and cAMP-inhibited cGMP 3,5-cyclic phosphodiesterase 10A OS=Mus musculus GN=Pde10a;sp|Q8CA95-2|PDE10_MOUSE Isoform 2 of cAMP and cAMP-inhibited cGMP 3,5-cyclic phosphodiesterase 10A OS=Mus musculus GN=Pde10a;	1,12	0,597366379427693	0,861464850775537	24,5687279499805	24,620547219318	24,18201000329	24,2472581965185	24,803332574449	24,6644462690852	41839012622,2291	46918552663,2165	−0,16530970482893	0,00979819764483945	0,0117782538908333	1,1214067857397	0.165309704828929	0,89173706875724	-0.165309704828929	2921
P39053-6	sp|P39053-6|DYN1_MOUSE Isoform 6 of Dynamin-1 OS=Mus musculus GN=Dnm1	1,12	0,143442285537794	0,537198460442281	35,5541042014945	35,4378163435131	35,3451936065751	35,5541042014945	35,6500093773241	35,4772270626791	2476703203272650	2777831217337870	−0,16553794998099	0,00295320564512046	0,0024343227994901	1,12158421472033	0.165537949981001	0,89159600043885	-0.165537949981001	2922
O35955	sp|O35955|PSB10_MOUSE Proteasome subunit beta type-10 OS=Mus musculus GN=Psmb10 PE=1 SV=1	1,12	0,586514301593467	0,856555745166483	23,1333909033916	23,277183681821	22,9990398942442	23,245900105076	23,5922800981017	22,915936011967	11170454737,626	12529755239,6234	−0,16567031521826	0,00601207489093045	0,0145456037447196	1,12168712321252	0.165670315218279	0,89151420151458	-0.165670315218279	2923
P62192	sp|P62192|PRS4_MOUSE 26S protease regulatory subunit 4 OS=Mus musculus GN=Psmc1 PE=1 SV=1	1,12	0,584174604035333	0,854517733714592	29,9008251465763	30,5244583269409	30,5173788922969	30,4732906815785	30,4606427564896	30,3537800086882	14631835560473,4	16415347159636	−0,16593449444290	0,0118105831838813	0,0021575847418463	1,12189253985198	0.165934494442905	0,89135096676187	-0.165934494442905	2924
Q9R1P0	sp|Q9R1P0|PSA4_MOUSE Proteasome subunit alpha type-4 OS=Mus musculus GN=Psma4 PE=1 SV=1	1,12	0,55062583795172	0,838423390503939	29,8904491527376	30,1253373252879	30,4957910271004	30,4300715036396	30,161244964118	30,2654579925308	12673392527078,9	14218875903081,9	−0,16600464511431	0,0101155157041962	0,0044753776822337	1,12194709291145	0.166004645114312	0,89130762610650	-0.166004645114312	2925
P68254	sp|P68254|1433T_MOUSE 14-3-3 protein theta OS=Mus musculus GN=Ywhaq PE=1 SV=1;sp|P68254-2|1433T_MOUSE Isoform 2 of 14-3-3 protein theta OS=Mus musculus GN=Ywhaq	1,12	0,607310904516218	0,866033363367613	33,0667878409893	33,5323458377698	33,8225092961663	33,597564845353	33,5109505079196	33,658865904478	344763914059764	386876673837444	−0,16626496869245	0,0113891154661447	0,0022125557815733	1,12214955817673	0.166264968692459	0,89114681079124	-0.166264968692459	2926
Q811S7-2	sp|Q811S7-2|UBIP1_MOUSE Isoform 2 of Upstream-binding protein 1 OS=Mus musculus GN=Ubp1;sp|Q811S7|UBIP1_MOUSE Upstream-binding protein 1 OS=Mus musculus GN=Ubp1 PE=1 SV=1	1,12	0,473489872287619	0,799218469891531	24,4661695074578	24,9428329797975	24,49311460887	24,6631481372109	24,7747333955639	24,8100392117994	49937548517,7439	56038618992,0758	−0,16629640291288	0,0108696164433534	0,0030980851996468	1,12217400844505	0.166296402912889	0,89112739421371	-0.166296402912889	2927
P16125	sp|P16125|LDHB_MOUSE L-lactate dehydrogenase B chain OS=Mus musculus GN=Ldhb PE=1 SV=2	1,12	0,51916199648953	0,82335612354586	35,0731894837483	35,505272310377	35,6500093773241	35,5823748158424	35,4378163435131	35,5541042014945	2388615705861570	2680460814290630	−0,16630628102257	0,00847517229824174	0,0021566674484995	1,1221816919787	0.166306281022576	0,89112129269970	-0.166306281022576	2928

A2ALS5	sp|A2ALS5|RPGP1_MOUSE Rap1 GTPase-activating protein 1 OS=Mus musculus GN=Rap1gap PE=1 SV=2;sp|A2ALS5-3|RPGP1_MOUSE Isoform 3 of Rap1 GTPase-activating protein 1 OS=Mus musculus GN=Rap1gap;sp|A2ALS5-2|RPGP1_MOUSE Isoform 2 of Rap1 GTPase-activating prot	1,12	0,472321825064738	0,798461364606646	29,7453450924431	29,5724244467374	29,2449656239036	29,7341360948367	29,6629732680634	29,5120517470896	6618644595471,04	7428811755722,49	−0,166595665211998	0,0086086750336932	0,0038265149846669	1,12240680830722	0.166595665211999	0,890942564316915	-0.166595665211999	2929
Q05512	sp|Q05512|MARK2_MOUSE Serine/threonine-protein kinase MARK2 OS=Mus musculus GN=Mark2 PE=1 SV=3;sp|Q05512-3|MARK2_MOUSE Isoform 3 of Serine/threonine-protein kinase MARK2 OS=Mus musculus GN=Mark2;sp|Q05512-4|MARK2_MOUSE Isoform 4 of Serine/threonine-prot	1,12	0,337730569239005	0,729620681454069	28,1709716265096	28,102857773251	27,8490417699696	28,2910319082209	28,0545669515707	28,1238312869463	1506721262433,84	1691229196311,83	−0,166659639168216	0,0060503268151212	0,0043175028776685	1,1224565807082	0.166659639168212	0,890903057799405	-0.166659639168212	2930
Q6PAM0	sp|Q6PAM0|AAKB2_MOUSE 5-AMP-activated protein kinase subunit beta-2 OS=Mus musculus GN=Prkab2 PE=1 SV=1	1,12	0,372774274993226	0,744143356508855	25,0441535538573	25,1963348706422	24,845323645973	25,2907819321046	25,0126127548663	25,1293167074687	74094268901,0005	83177035535,817	−0,166823311458367	0,0070328124693797	0,0055552898264175	1,12258392949328	0.166823311458367	0,89080199148351	-0.166823311458367	2931
Q9QXK3-4	sp|Q9QXK3-4|COPG2_MOUSE Isoform 4 of Coatomer subunit gamma-2 OS=Mus musculus GN=Copg2;sp|Q9QXK3|COPG2_MOUSE Coatomer subunit gamma-2 OS=Mus musculus GN=Copg2 PE=2 SV=1	1,12	0,512620071403822	0,819082952251546	28,9217714421477	28,8732918283341	28,4205387528905	29,0002145179698	28,7457012936542	28,8167867144637	3026820994030,27	3398088472105,86	−0,166920057985815	0,0096197215929553 5	0,0045512886674580	1,12265921202735	0.166920057985811	0,890742256676587	-0.166920057985811	2932
Q8BGV0	sp|Q8BGV0|SYNM_MOUSE Probable asparagine--tRNA ligase, mitochondrial OS=Mus musculus GN=Nars2 PE=1 SV=1	1,12	0,77718416568163	0,938755143139587	23,8790570595803	25,0836030464032	25,0225487947601	24,7074766283013	24,6135674323953	25,0120600285972	51340014778,6561	57652610418,6108	−0,167302221090167	0,0275125472599908	0,0084076079889840	1,12295663854345	0.167302221090165	0,890506334507328	-0.167302221090165	2933
P05064	sp|P05064|ALDOA_MOUSE Fructose-bisphosphate aldolase A OS=Mus musculus GN=Aldoa PE=1 SV=2	1,12	0,138491679197055	0,529162269633094	36,6007065050921	36,3824391571779	36,3824391571779	36,5127737376142	36,6007065050921	36,6007065050921	6796573206257750	7634055219713940	−0,167642091091891	0,0034567559515627 6	0,0013881889428941	1,13221215756 95	0.167642091091893	0,890296573792982	-0.167642091091893	2934
P61264	sp|P61264|STX1B_MOUSE Syntaxin-1B OS=Mus musculus GN=Stx1b PE=1 SV=1	1,12	0,536903453787333	0,831016807971437	32,1512238864603	32,6728334389707	32,6728334389707	32,6953467176655	32,6786103940251	32,4715364843887	130053053650256	146078393019457	−0,167642525500359	0,0092664929063681 2	0,0038223541162295	1,12322155396902	0.167642525500358	0,890296305716709	-0.167642525500358	2935
Q922L6	sp|Q922L6|NELFD_MOUSE Negative elongation factor D OS=Mus musculus GN=Nelfcd PE=2 SV=2	1,12	0,337943541049627	0,729620681454069	22,1585650899638	22,5567734350707	22,4762920876387	22,524642240339	22,5083970764616	22,5083970764616	5333162315,84236	5992725363,07422	−0,168221021643396	0,0094008969111952 8	0,0004165953630163	1,12367203699625	0.168221021643396	0,88993938362403	-0.168221021643396	2936
Q99LF4	sp|Q99LF4|RTCB_MOUSE tRNA-splicing ligase RtcB homolog OS=Mus musculus GN=Rtcb PE=2 SV=1	1,12	0,619198595102333	0,870879639674993	29,4939123683025	30,2119986671099	30,1381873875547	30,1415815593921	30,0952385083161	29,9571855137959	10145311643455,6	11400388243176,2	−0,168269774130966	0,0131897700492802	0,0031905810784493	1,12371000949293	0.168269774130975	0,889909310722656	-0.168269774130974	2937
P10637	sp|P10637|TAU_MOUSE Microtubule-associated protein tau OS=Mus musculus GN=Mapt PE=1 SV=3	1,12	0,471074718151209	0,79699412444793	32,5334327252068	32,6339988306786	32,9567183360255	32,8687527944652	32,9720957537316	32,6339988306786	160297080711312	180174893616196	−0,168649841765264	0,0067611964484005 6	0,0052780758416499	1,12400608181183	0.168649841765267	0,889674901392048	-0.168649841765267	2938
P62484	sp|P62484|ABI2_MOUSE Abl interactor 2 OS=Mus musculus GN=Abi2 PE=2 SV=1	1,12	0,495018056666785	0,808788130730939	29,2389697441818	29,7200994190062	29,57918938533	29,7759040277786	29,4486387307347	29,6661513123182	6564863618942,38	7383222829229,74	−0,169485660091647	0,0083811074300955 8	0,0056213965201180	1,12465745791368	0.169485660091646	0,889159621859505	-0.169485660091646	2939
Q9Z2C9	sp|Q9Z2C9|MTMR7_MOUSE Myotubularin-related protein 7 OS=Mus musculus GN=Mtmr7 PE=2 SV=2	1,12	0,524472478376627	0,826303567980393	27,6705231091959	27,2837384201724	27,1060915962256	27,6030325495586	27,4813946984528	27,3283963991727	757622473057,25	852075705226,587	−0,169502491185851	0,0105508129027314	0,0050095178369609	1,12467057872266	0.169502491185851	0,889149248605534	-0.169502491185851	2940
P58400	sp|P58400|NRX1B_HUMAN Neurexin-1-beta OS=Homo sapiens GN=NRXN1 PE=1 SV=2;sp|P0DI97|NRX1B_MOUSE Neurexin-1-beta OS=Mus musculus GN=Nrxn1 PE=1 SV=1;sp|Q9CS84-4|NRX1A_MOUSE Isoform 4a of Neurexin-1 OS=Mus musculus GN=Nrxn1;sp|Q9CS84-5| NRX1A_MOUSE Isoform	1,12	0,11644144206651	0,487571684077389	25,1761940416215	24,9839800051415	25,0898230655906	25,1478806807997	25,30131699854	25,1535971658607	78262341343,8144	88028953562,5853	−0,169659846538307	0,0038380570848782 1	0,0034515892801201	1,12479325370379	0.169659846538306	0,88905227401315	-0.169659846538306	2941
Q3U0D9	sp|Q3U0D9|HACE1_MOUSE E3 ubiquitin-protein ligase HACE1 OS=Mus musculus GN=Hace1 PE=1 SV=1;sp|Q3U0D9-2|HACE1_MOUSE Isoform 2 of E3 ubiquitin-protein ligase HACE1 OS=Mus musculus GN=Hace1;sp|Q3U0D9-3|HACE1_MOUSE Isoform 3 of E3 ubiquitin-protein ligase H	1,13	0,594609194815629	0,860080030359509	28,1148719832427	27,6547108054017	27,4813946984528	27,6568502118367	28,0596330923216	27,889164479335	1126713190259,32	1268110687562,9	−0,170560359253773	0,011797469819675	0,0072548174346219	1,12549555514748	0.170560359253772	0,888497511541896	-0.170560359253772	2942
Q3UHB1	sp|Q3UHB1|NT5D3_MOUSE 5-nucleotidase domain-containing protein 3 OS=Mus musculus GN=Nt5dc3 PE=2 SV=1	1,13	0,653599856316798	0,891901943526937	29,209851847461	29,8660763732758	29,9631043655379	29,9945463914196	29,8078729257526	29,5915968002301	7757471262311,24	8731911089929,06	−0,170710993318346	0,0138061818127941	0,0067674309536260	1,12561307604864	0.170710993318345	0,88840474695835	-0.170710993318345	2943
Q9EP69	sp|Q9EP69|SAC1_MOUSE Phosphatidylinositide phosphatase SAC1 OS=Mus musculus GN=Sacm1l PE=2 SV=1	1,13	0,559397544176897	0,842973036472956	29,1969573151545	29,8014346095597	29,8046256258292	29,7312890475709	29,6792282244892	29,748150804332	7170567023446,17	8073078705050,57	−0,17103174997716	0,0118212176624834	0,0012089043261514	1,12586336319755	0.17103174997716	0,88820724848876	-0.17103174997716	2944
Q8VE19	sp|Q8VE19|MIO_MOUSE WD repeat-containing protein mio OS=Mus musculus GN=Mios PE=2 SV=2	1,13	0,36663935008222	0,739315231400715	26,7226120902923	26,6940235846991	26,3947233703869	26,6952330944468	26,8719382927359	26,6005971337788	357995534018,075	403156034761,121	−0,17139672764987	0,0068267440851262 1	0,0051538263488881	1,12614822379534	0.171396727649872	0,887982575357443	-0.171396727649872	2945
Q3TCH7	sp|Q3TCH7|CUL4A_MOUSE Cullin-4A OS=Mus musculus GN=Cul4a PE=1 SV=1	1,13	0,475419852920813	0,79947899324683	27,791225622099	27,4902079656322	27,2435367500068	27,6361586212546	27,7120449619398	27,5334727094064	884531042396,288	996211509445,369	−0,171539304045436	0,0099712896797805 7	0,0032439256759040	1,12625952249966	0.171539304045435	0,887894823548808	-0.171539304045435	2946

Q8JZS0	sp|Q8JZS0|LIN7A_MOUSE Protein lin-7 homolog A OS=Mus musculus GN=Lin7a PE=1 SV=2	1,13	1,13	0,700323506986323	0,913439194888196	29,3411392654344	29,9652839885513	30,1107352298154	30,2298776072897	29,5469002992119	29,9980024503029	8799530595771,18	9913581341359,34	−0,17197976756514	0,0137174443360591	0,0116058175295263	1,12660342883785	0.171979767565146	0,887623785267151	-0.171979767565146	2947
Q60751	sp|Q60751|IGF1R_MOUSE Insulin-like growth factor 1 receptor OS=Mus musculus GN=Igf1r PE=1 SV=3	1,13	1,13	0,86158390341038	0,967230529051203	22,9274759558395	22,5602574624914	20,9532739285652	22,7026757793855	22,4474378958212	21,6488223621637	4152608172,01421	4678821095,42794	−0,17212731557978	0,0474094727752278	0,0246910565727627	1,12671865526828	0.172127315579778	0,887533010414111	-0.172127315579778	2948
Q9ERK4	sp|Q9ERK4|XPO2_MOUSE Exportin-2 OS=Mus musculus GN=Cse1l PE=2 SV=1	1,13	1,13	0,585495123212165	0,855897701855078	31,5283219649286	31,0449398168704	30,8043490382255	31,3267661620555	31,2604931538048	31,1488485026459	32945318021173,8	37127136968482,2	−0,17240061396106	0,0118457487892315	0,0028777980802399	1,1269321165642	0.172400613961067	0,887364895632582	-0.172400613961067	2949
Q8R317	sp|Q8R317|UBQL1_MOUSE Ubiquilin-1 OS=Mus musculus GN=Ubqln1 PE=1 SV=1	1,13	1,13	0,296895232915023	0,711969538636193	29,5056687085734	29,8687792032057	29,7730411693604	29,8931980979023	29,7678736961036	29,8451084763157	8043051715552,17	9064560025539	−0,17249399994781	0,0063331399167383	0,0021191108400074	1,12700506550413	0.172493999947817	0,88730745815475	-0.172493999947817	2950
Q8BFP9	sp|Q8BFP9|PDK1_MOUSE [Pyruvate dehydrogenase (acetyl-transferring)] kinase isozyme 1, mitochondrial OS=Mus musculus GN=Pdk1 PE=1 SV=2	1,13	1,13	0,16720655691701	0,576050679312994	27,9420598071018	27,7577253480226	27,9101208805373	28,1167813912022	27,9533352167808	27,8990401696981	1269911943989,52	1431464162000,29	−0,17276308798235	0,00353457940680549	0,0040492943382825	1,12721529140299	0.17276308798236	0,887141975119362	-0.17276308798236	2951
Q3URD3-2	sp|Q3URD3-2|SLMAP_MOUSE Isoform 2 of Sarcolemmal membrane-associated protein OS=Mus musculus GN=Slmap;sp|Q3URD3|SLMAP_MOUSE Sarcolemmal membrane-associated protein OS=Mus musculus GN=Slmap PE=1 SV=2;sp|Q3URD3-5|SLMAP_MOUSE Isoform 5 of Sarcolemmal membr	1,13	1,13	0,478462720935781	0,799905345588749	27,4525390811037	26,9915324948691	26,9456779435363	27,2481132134366	27,3379076999531	27,1640538722618	605861050125,663	683180494323,883	−0,17327982485688	0,0103331856396079	0,0031905540897886	1,12761910372384	0.173279824856888	0,8868242802003	-0.173279824856888	2952
Q2M3X8	sp|Q2M3X8|PHAR1_MOUSE Phosphatase and actin regulator 1 OS=Mus musculus GN=Phactr1 PE=1 SV=1;sp|Q2M3X8-4|PHAR1_MOUSE Isoform 4 of Phosphatase and actin regulator 1 OS=Mus musculus GN=Phactr1;sp|Q2M3X8-2|PHAR1_MOUSE Isoform 2 of Phosphatase and actin reg	1,13	1,13	0,399090557939704	0,754780017703466	29,0558930771154	28,961551696187	28,5948343218379	29,0169853290817	29,0120311248533	28,9439646135663	3454710224828,88	3896086443296,31	−0,17346098225466	0,00843655215388647	0,0014074589453272	1,12776070632358	0.173460982254665	0,886712929784484	-0.173460982254665	2953
Q60960	sp|Q60960|IMA5_MOUSE Importin subunit alpha-5 OS=Mus musculus GN=Kpna1 PE=1 SV=2	1,13	1,13	0,53218107164923	0,829883431939119	28,0716553884336	28,6238583935333	28,5655362258062	28,7010859141301	28,4530620211488	28,468006362801	2201918193612,74	2483569856538,04	−0,17365445628979	0,0106748552669509	0,0048731592847481	1,12791195592203	0.173654456289789	0,88659402424947	-0.173654456289789	2954
P29533	sp|P29533|VCAM1_MOUSE Vascular cell adhesion protein 1 OS=Mus musculus GN=Vcam1 PE=1 SV=1	1,13	1,13	0,551168409104267	0,838423390503939	24,9294153710934	24,4941949562678	24,3017734131296	24,8371424988238	24,5615634697321	24,6878806630467	47080649433,4584	53104572744,4309	−0,17370187325396	0,0130844819509536	0,0055859755213931	1,12794902754021	0.173701873253962	0,88656488510014	-0.173701873253962	2955
Q61133	sp|Q61133|GSTT2_MOUSE Glutathione S-transferase theta-2 OS=Mus musculus GN=Gstt2 PE=2 SV=4	1,13	1,13	0,521397412298404	0,823916573667872	24,4760775927859	24,8152314567564	25,0665939202135	25,0264181578287	24,7608480287448	24,9321216980073	58131159003,1692	65575148559,9558	−0,17383749799143	0,0119561126658957	0,0054054880926733	1,12805506864883	0.173837497991436	0,886481544910562	-0.173837497991436	2956
Q9EQP2	sp|Q9EQP2|EHD4_MOUSE EH domain-containing protein 4 OS=Mus musculus GN=Ehd4 PE=1 SV=1	1,13	1,13	0,552176000200021	0,838797478215494	27,6659486438165	28,3097572274689	28,2102303198389	28,1810020312617	28,1252116374367	28,2415791179015	1538730328637,5	1735987610848,61	−0,17401623860191	0,0123499316773352	0,0020651093576472	1,12819483605407	0.174016238601918	0,886371722368062	-0.174016238601918	2957
Q922B2	sp|Q922B2|SYDC_MOUSE Aspartate--tRNA ligase, cytoplasmic OS=Mus musculus GN=Dars PE=2 SV=2	1,13	1,13	0,504932846447618	0,814993646406912	30,7092073014578	31,2478449838581	31,143072998167	31,2859805099124	31,1488485026459	31,0273688047328	30034717980592,5	33887452840281,2	−0,17412008298909	0,00920245923479595	0,0041530622123493	1,12827604581399	0.174120082989095	0,88630792412025	-0.174120082989095	2958
Q8C190-2	sp|Q8C190-2|VP9D1_MOUSE Isoform 2 of VPS9 domain-containing protein 1 OS=Mus musculus GN=Vps9d1;sp|Q8C190|VP9D1_MOUSE VPS9 domain-containing protein 1 OS=Mus musculus GN=Vps9d1 PE=2 SV=1	1,13	1,13	0,792046884174264	0,942359320384531	23,1698545542957	21,736986923389	22,6368476224383	22,8346140135788	22,2607396283641	22,8107698771548	5997227004,18877	6767343854,68645	−0,17429411296748	0,0321666727225907	0,0143431376859155	1,1284121561448	0.174294112967485	0,886201016671502	-0.174294112967485	2959
Q99NB1	sp|Q99NB1|ACS2L_MOUSE Acetyl-coenzyme A synthetase 2-like, mitochondrial OS=Mus musculus GN=Acss1 PE=1 SV=1	1,13	1,13	0,614173312341284	0,868502403759507	27,2091856375552	27,9088339896986	27,2091856375552	27,4525390811037	27,612789504722	27,6244221741423	828101654504,306	934474572115,612	−0,17434752932078	0,0147196359872329	0,0034848918135201	1,12845393682371	0.17434752932078	0,886168205336522	-0.17434752932078	2960
P48455	sp|P48455|PP2BC_MOUSE Serine/threonine-protein phosphatase 2B catalytic subunit gamma isoform OS=Mus musculus GN=Ppp3cc PE=2 SV=1	1,13	1,13	0,467393738777649	0,796164546189264	23,6838064405286	24,0899649888494	24,197055235212	24,2234160190066	24,0715250354999	24,0385852060188	26232778534,6741	29604002838,8303	−0,17442163612945	0,0112861312370119	0,0040889193209293	1,12851190352178	0.174421636129453	0,886122686769429	-0.174421636129453	2961
P21126	sp|P21126|UBL4A_MOUSE Ubiquitin-like protein 4A OS=Mus musculus GN=Ubl4a PE=2 SV=1	1,13	1,13	0,54750695591643	0,836745479092484	25,9874283403252	25,7991721647102	25,6741775613297	25,6003102586315	26,014799565769	26,2087814532622	163529519020,443	184570454096,032	−0,17462054307339	0,00610708213809026	0,0119818032267343	1,12866750420124	0.174620543073404	0,886000523872356	-0.174620543073404	2962
Q9R0P3	sp|Q9R0P3|ESTD_MOUSE S-formylglutathione hydrolase OS=Mus musculus GN=Esd PE=1 SV=1	1,13	1,13	0,375729785069829	0,746362141983349	30,4798415039778	30,8190110775727	30,8856809268552	30,9568503354048	30,8064361728851	30,7847908092274	22134925936913,6	24986557741240,9	−0,17482761685039	0,00708254419471829	0,0030378960984895	1,12882951641468	0.174827616850391	0,885873363035493	-0.174827616850391	2963

Q3UFS0	sp|Q3UFS0|ZY11B_MOUSE Protein zyg-11 homolog B OS=Mus musculus GN=Zyg11b PE=2 SV=2;sp|Q3UFS0-2|ZY11B_MOUSE Isoform 2 of Protein zyg-11 homolog B OS=Mus musculus GN=Zyg11b;sp|Q3UFS0-3|ZY11B_MOUSE Isoform 3 of Protein zyg-11 homolog B OS=Mus musculus GN=Z	1,13	0,206978405719358	0,637641589296298	27,4054318063668	27,355199209797	27,1437352154627	27,3283963991727	27,4280292367288	27,5118748975731	719236025945,514	812000541915,538	−0,17501540416191	0,00508689761267026	0,0033494934682843	1,12897645922015	0.175015404161909	0,885758061501795	-0.175015404161909	2964
Q9WV95	sp|Q9WV95|PHLA3_MOUSE Pleckstrin homology-like domain family A member 3 OS=Mus musculus GN=Phlda3 PE=1 SV=1	1,13	0,629173911353176	0,877903591648345	24,0011855134086	23,4466569309183	23,6838064405286	23,4448047131735	24,0194215071423	24,0319190122397	19831742118,3643	22393765044,894	−0,175285691082948	0,01173443550076	0,0140743709779949	1,1291879912132	0.17528569108295	0,885592131497608	-0.17528569108295	2965
P60202	sp|P60202|MYPR_MOUSE Myelin proteolipid protein OS=Mus musculus GN=Plp1 PE=1 SV=2;sp|P60201|MYPR_HUMAN Myelin proteolipid protein OS=Homo sapiens GN=PLP1 PE=1 SV=2;sp|P60202-2|MYPR_MOUSE Isoform DM-20 of Myelin proteolipid protein OS=Mus musculus GN=Plp	1,13	0,404974337674241	0,759526281549977	28,2538132214311	28,3553953189567	28,5819748597617	28,4070567271281	28,4120046915943	28,736881630549	2151230528932,92	2429356887953,47	−0,175412312301525	0,00591616231273013	0,0066276752052374	1,12928710116368	0.175412312301521	0,885514409019233	-0.175412312301521	2966
Q8BU33	sp|Q8BU33|ILVBL_MOUSE Acetolactate synthase-like protein OS=Mus musculus GN=Ilvbl PE=2 SV=1;sp|Q8BU33-3|ILVBL_MOUSE Isoform 3 of Acetolactate synthase-like protein OS=Mus musculus GN=Ilvbl;sp|Q8BU33-2|ILVBL_MOUSE Isoform 2 of Acetolactate synthase-like	1,13	0,668234208774479	0,898352728146089	25,4520364849735	26,0680641034151	26,1521497135295	26,3577801409492	25,9168429770321	25,7624652702272	175472838350,209	198164394037,059	−0,175450032606173	0,014764200645976	0,0118766014566735	1,12931662757721	0.175450032606179	0,885491256907604	-0.175450032606179	2967
Q9D024	sp|Q9D024|CCD47_MOUSE Coiled-coil domain-containing protein 47 OS=Mus musculus GN=Ccdc47 PE=2 SV=2;sp|Q9D024-2|CCD47_MOUSE Isoform 2 of Coiled-coil domain-containing protein 47 OS=Mus musculus GN=Ccdc47	1,13	0,696164554800688	0,911315513842744	25,6077300356249	26,6107194319358	26,2906517440577	26,4706027605894	26,133625476344	26,269865312127	231929759935,385	261935601934,87	−0,175524211730024	0,0195767292878585	0,0064475261429281	1,12937469520015	0.175524211730018	0,885445728729362	-0.175524211730019	2968
P62071	sp|P62071|RRAS2_MOUSE Ras-related protein R-Ras2 OS=Mus musculus GN=Rras2 PE=1 SV=1	1,13	0,636852023998101	0,883588334123886	25,5721737946229	25,4737767577379	26,0271456176258	26,140845402906	25,8273556009734	25,4703197299504	143705522012,945	162320764877,339	−0,175732068691884	0,0114908324828265	0,0129973462383444	1,12953742210906	0.175732068691881	0,885318166911911	-0.175732068691881	2969
Q9D379	sp|Q9D379|HYEP_MOUSE Epoxide hydrolase 1 OS=Mus musculus GN=Ephx1 PE=1 SV=2	1,13	0,262212731970419	0,682806179310143	27,966093558593	27,6464734713961	27,8317214951593	27,9380967665236	28,0347736742627	27,8373225183337	1201705413493,4	1357588372893,99	−0,175962837443394	0,00576972306940325	0,0035341523365382	1,12971811364936	0.175962837443402	0,885176565656428	-0.175962837443402	2970
O54983	sp|O54983|CRYM_MOUSE Ketimine reductase mu-crystallin OS=Mus musculus GN=Crym PE=1 SV=1	1,13	0,19281532355267	0,619741218143775	32,6569761311727	32,5208763535181	32,8095502245621	32,799089045904	32,704254865978	32,8500916619242	153154383298987	173028688230796	−0,176024599497787	0,00442144371439168	0,0022574116007292	1,12976647813606	0.176024599497785	0,885138671887176	-0.176024599497786	2971
Q6NZL0	sp|Q6NZL0|SOGA3_MOUSE Protein SOGA3 OS=Mus musculus GN=Soga3 PE=2 SV=2	1,13	0,477058788407457	0,799905345588749	30,3453333642794	30,2236511949269	29,7814723531849	30,2664339291469	30,2207870528903	30,2298776072897	12010699227861,3	13572039362364,4	−0,176317376366092	0,00985240814822933	0,0007990137245158	1,12999577334193	0.176317376366085	0,884959062318023	-0.176317376366085	2972
Q9D415-4	sp|Q9D415-4|DLGP1_MOUSE Isoform 4 of Disks large-associated protein 1 OS=Mus musculus GN=Dlgap1;sp|Q9D415-3|DLGP1_MOUSE Isoform 3 of Disks large-associated protein 1 OS=Mus musculus GN=Dlgap1;sp|Q9D415-2|DLGP1_MOUSE Isoform 2 of Disks large-associated p	1,13	0,795536069189944	0,944283006813064	23,9524385927456	24,2627398725708	24,35916318947	24,8730804771037	23,4099585585598	24,6585297673407	32078351076,4021	36255475955,0694	−0,176598928537833	0,00878525905142997	0,0324964798593359	1,13021632155339	0.176598928537834	0,884786373130395	-0.176598928537834	2973
Q7TPM6	sp|Q7TPM6|FSD1_MOUSE Fibronectin type III and SPRY domain-containing protein 1 OS=Mus musculus GN=Fsd1 PE=2 SV=1;sp|Q7TPM6-2|FSD1_MOUSE Isoform 2 of Fibronectin type III and SPRY domain-containing protein 1 OS=Mus musculus GN=Fsd1	1,13	0,491734539943874	0,807519202896252	28,5146694615578	29,1231989742319	28,736881630549	28,9505536434768	28,8979126911188	28,8948809578225	3191727286947,4	3608990096497,15	−0,177257796716741	0,0106951601312404	0,0010826482449720	1,1307326008886	0.177257796716743	0,884382389978088	-0.177257796716743	2974
Q922M7	sp|Q922M7|ASHWN_MOUSE Ashwin OS=Mus musculus PE=1 SV=1	1,13	0,34096965145205	0,72969758766342	23,5262499539463	23,9376551234288	23,8182850170703	23,9668942617015	23,8482598894016	23,835941088441	20852298761,8708	23580794214,3195	−0,177405874464123	0,0089077796044971	0,0030276934418549	1,1308486648694	0.177405874464124	0,884291621916966	-0.177405874464124	2975
P60954-2	sp|P60954-2|NOL4_MOUSE Isoform 2 of Nucleolar protein 4 OS=Mus musculus GN=Nol4	1,13	0,555509623496639	0,840205991686519	24,0381319453118	24,2234160190066	24,0390384667258	23,8239823554939	24,4060266307704	24,4408004316673	29280730573,6678	33126623762,9597	−0,178039622401913	0,00442789280879464	0,0143049828785805	1,13134553386965	0.17803962240191	0,883903255073276	-0.17803962240191	2976
Q7TMS5	sp|Q7TMS5|ABCG2_MOUSE ATP-binding cassette sub-family G member 2 OS=Mus musculus GN=Abcg2 PE=2 SV=1	1,13	0,600134596672456	0,862301656947224	22,3477845269731	21,8085797576661	21,829564652429	21,9854071197575	22,4317248569495	21,9399864331884	3568137706,13934	4038097369,98525	−0,178504403892696	0,0138862549221869	0,0122855303945799	1,13171006910336	0.178504403892698	0,883618540915068	-0.178504403892698	2977
P14685	sp|P14685|PSMD3_MOUSE 26S proteasome non-ATPase regulatory subunit 3 OS=Mus musculus GN=Psmd3 PE=1 SV=3	1,13	0,552709373459011	0,838891344884342	30,1871752749852	30,8504306917222	30,7635181652725	30,7450551800597	30,7195270753918	30,7077418394737	19479323987882,1	22045024181246,3	−0,178509448573107	0,0117799281869438	0,0006208092251931	1,13171402636767	0.178509448573103	0,883615451166216	-0.178509448573103	2978
P39054	sp|P39054|DYN2_MOUSE Dynamin-2 OS=Mus musculus GN=Dnm2 PE=1 SV=2	1,13	0,408266827939278	0,76291790520755	31,5364546439614	31,2748215534313	31,0970439805281	31,3988833526896	31,5739501993876	31,3069394690914	39320746357018,8	44503591381930,9	−0,178631058292559	0,00706119111995697	0,0043162377691976	1,13180942645019	0.178631058292561	0,883540971324476	-0.178631058292561	2979
Q9D855	sp|Q9D855|QCR7_MOUSE Cytochrome b-c1 complex subunit 7 OS=Mus musculus GN=Uqcrb PE=1 SV=3	1,13	0,252509553054477	0,67459732861346	26,5801731397537	26,275921090209	26,2484016717649	26,4538794096732	26,5257288518079	26,4967677565846	282845118571,647	320172368523,442	−0,17883653321539	0,00698262629495713	0,0013645183763476	1,13197063516703	0.178836533215388	0,883415142524824	-0.178836533215388	2980

Q9D0E1-2	sp|Q9D0E1-2|HNRPM_MOUSE Isoform 2 of Heterogeneous nuclear ribonucleoprotein M OS=Mus musculus GN=Hnrnpm;sp|Q9D0E1|HNRPM_MOUSE Heterogeneous nuclear ribonucleoprotein M OS=Mus musculus GN=Hnrnpm PE=1 SV=3	1,13	1,13	0,304474894218787	0,716532404521184	29,2657804305218	29,1893852573842	28,8732918283341	29,2565888136709	29,2155065783681	29,2283500040983	4386206466258,76	4965235273523,02	−0,17888356532849	0,00714838982149973	0,0007189153360517	1,13201129762552	0.178888356532849	0,883383409774776	-0.178888356532848	2981
Q8C570	sp|Q8C570|RAE1L_MOUSE mRNA export factor OS=Mus musculus GN=Rae1 PE=1 SV=1	1,13	1,13	0,691502833016171	0,909293912033752	24,9531426386628	25,0609738563947	25,0631252435222	25,3750798339753	25,5382714274755	24,5360198544844	73882900136,2191	83640222522,1374	−0,178956402426622	0,00251287742291504	0,0213825718184236	1,13206469112513	0.178956402426622	0,883341745254972	-0.178956402426622	2982
P97315	sp|P97315|CSRP1_MOUSE Cysteine and glycine-rich protein 1 OS=Mus musculus GN=Csrp1 PE=1 SV=3	1,13	1,13	0,192926711001796	0,619741218143775	29,7341360948367	30,0602349868501	29,9143750348548	30,0323123257719	30,049400664825	29,9997657346318	9697753456396,28	10980692015074,5	−0,179246495376879	0,00546271821956391	0,0008397552722625	1,13229234630955	0.179246495376877	0,883164143305633	-0.179246495376877	2983
Q8VHH5	sp|Q8VHH5|AGAP3_MOUSE Arf-GAP with GTPase, ANK repeat and PH domain-containing protein 3 OS=Mus musculus GN=Agap3 PE=1 SV=1	1,13	1,13	0,339129449780987	0,729620681454069	30,0338596200803	29,6482708054861	29,6593636472282	29,8563790555124	29,9571855137959	29,9008251465763	8580368903627,31	9716014038591,42	−0,179324898351699	0,00737018124911064	0,0016893757509990	1,13235388218379	0.179324898351699	0,883116149230189	-0.1793248983517	2984
Q8VC31	sp|Q8VC31|CCDC9_MOUSE Coiled-coil domain-containing protein 9 OS=Mus musculus GN=Ccdc9 PE=2 SV=1	1,13	1,13	0,522403894840818	0,823948365557954	24,1881015331326	23,985379748144	23,7216479337666	23,8100825694553	24,2505125478808	24,2081894088514	25579141513,3839	28971975628,2716	−0,179690221463225	0,00975966703141283	0,0100868426314731	1,13264065618123	0.179690221463227	0,882892552499013	-0.179690221463227	2985
Q9QZ06	sp|Q9QZ06|TOLIP_MOUSE Toll-interacting protein OS=Mus musculus GN=Tollip PE=1 SV=1	1,13	1,13	0,522614528407435	0,823948365557954	29,6647193245074	29,9327417629286	30,2598956411556	30,2236511949269	29,9092926391212	30,0981104206294	10190247413153,7	11542050931209,6	−0,179710522558894	0,00995168337104002	0,0052610645412952	1,1326565944131	0.179710522558885	0,882880128833894	-0.179710522558885	2986
Q99719-2	sp|Q99719-2|SEPT5_HUMAN Isoform 2 of Septin-5 OS=Homo sapiens GN=SEPT5	1,13	1,13	0,23938950080117	0,664270737934997	29,4300026872135	29,0726137546512	29,2410664809566	29,3261521639233	29,383338090787	29,4085535576116	5037314947843,08	5706809750572,03	−0,180029532928405	0,0061129963949367	0,0014374484695779	1,13290707642087	0.180029532928407	0,882684926957335	-0.180029532928407	2987
Q9QXK7	sp|Q9QXK7|CPSF3_MOUSE Cleavage and polyadenylation specificity factor subunit 3 OS=Mus musculus GN=Cpsf3 PE=1 SV=2	1,13	1,13	0,431924948683235	0,775517144807438	25,6487332316027	25,7162865377133	25,462854440618	25,7769511586397	25,9516347704544	25,4737767577379	132426207580,382	150032968307,7	−0,180090889496962	0,00512466721361697	0,0093957362417663	1,13295525900061	0.180090889496964	0,882647387931374	-0.180090889496964	2988
Q9CZX0	sp|Q9CZX0|ELP3_MOUSE Elongator complex protein 3 OS=Mus musculus GN=Elp3 PE=1 SV=1;sp|Q9CZX0-2|ELP3_MOUSE Isoform 2 of Elongator complex protein 3 OS=Mus musculus GN=Elp3	1,13	1,13	0,444021073196472	0,783519926508673	27,0697573276934	27,55952828088	27,2868283286901	27,3909720087234	27,5887500513035	27,3125156226977	722058005342,117	818505451624,268	−0,180877287445755	0,00898766195912176	0,0051893444857734	1,13357298938394	0.180877287445756	0,882166397192887	-0.180877287445756	2989
Q8BMF4	sp|Q8BMF4|ODP2_MOUSE Dihydrolipoyllysine-residue acetyltransferase component of pyruvate dehydrogenase complex, mitochondrial OS=Mus musculus GN=Dlat PE=1 SV=2	1,13	1,13	0,298318424417387	0,713417981259494	32,2397875300273	32,6186624488613	32,4866102924521	32,5868757685581	32,4866102924521	32,6477383197789	123634830792334	140150990404826	−0,180896698420545	0,00592673757131573	0,0024978104296649	1,13358824132848	0.18089669842055	0,882154528021635	-0.18089669842055	2990
P14824	sp|P14824|ANXA6_MOUSE Annexin A6 OS=Mus musculus GN=Anxa6 PE=1 SV=3	1,13	1,13	0,527631382192572	0,828404996116251	31,3519919643443	31,9453672776117	31,8619322988203	31,9879882223648	31,7786943855435	31,7691737448374	59664829786455,6	67644383955147,5	−0,181089395600507	0,0101268180860957	0,0038836477842660	1,13373966199604	0.181089395600507	0,882036708709139	-0.181089395600507	2991
Q68FD9	sp|Q68FD9|K1549_MOUSE UPF0606 protein KIAA1549 OS=Mus musculus GN=Kiaa1549 PE=1 SV=3	1,13	1,13	0,229047048450863	0,653672489647647	26,3128537120198	26,1944717091464	25,9600094693325	26,2733618497883	26,2637346192989	26,3073973295377	228723181945,596	259363900269,747	−0,181375095460225	0,00686564570586036	0,0008728668165277	1,13396420102025	0.181375095460223	0,881862054463695	-0.181375095460223	2992
Q8BMA6	sp|Q8BMA6|SRP68_MOUSE Signal recognition particle subunit SRP68 OS=Mus musculus GN=Srp68 PE=2 SV=2	1,13	1,13	0,525002392999537	0,826714516252356	27,5612721540117	28,0003802943019	28,155149069996	28,1351226080958	27,8588173640719	28,100354168705	1315977045569,53	1492436865716,36	−0,18153557818129	0,011039878152363 6	0,0053688142500086	1,13409034811125	0.181535578181296	0,881763963219891	-0.181535578181296	2993
Q9JLB0	sp|Q9JLB0|MPP6_MOUSE MAGUK p55 subfamily member 6 OS=Mus musculus GN=Mpp6 PE=1 SV=1;sp|Q9JLB0-2|MPP6_MOUSE Isoform Alpha of MAGUK p55 subfamily member 6 OS=Mus musculus GN=Mpp6	1,13	1,13	0,625953117723633	0,876102744788026	28,8313933089507	29,5991882691668	29,4260099986843	29,6347178699823	29,2762930224565	29,323260678621	5230513253425,83	5932245096414,12	−0,18162568491968	0,0137512778628681	0,0066232429259337	1,13416118246688	0.181625684919679	0,881708892403575	-0.181625684919679	2994
Q61749	sp|Q61749|EI2BD_MOUSE Translation initiation factor eIF-2B subunit delta OS=Mus musculus GN=Eif2b4 PE=2 SV=2;sp|Q61749-2|EI2BD_MOUSE Isoform 2 of Translation initiation factor eIF-2B subunit delta OS=Mus musculus GN=Eif2b4	1,13	1,13	0,0225212295624073	0,210337162518039	27,2462324621921	27,4002419470032	27,4101106762398	27,4982193752751	27,4482593170704	27,4879183321299	756671486440,291	858225173081,27	−0,181689136947419	0,0033598342389582	0,0009599820999589	1,1342106578065	0.181689136947419	0,881670114293697	-0.181689136947419	2995
P62069	sp|P62069|UBP46_MOUSE Ubiquitin carboxyl-terminal hydrolase 46 OS=Mus musculus GN=Usp46 PE=1 SV=1	1,13	1,13	0,588744364334636	0,858160137994512	28,1207709623458	27,7796180261822	27,3368848768073	27,8119809890143	27,9775310134962	27,8257406617401	1121578243285,29	1272177211148,65	−0,181769379585402	0,0141657133896098	0,0032960344997593	1,13427415230723	0.181769379585403	0,881621077202457	-0.181769379585403	2996
Q8BHC1	sp|Q8BHC1|RB39B_MOUSE Ras-related protein Rab-39B OS=Mus musculus GN=Rab39b PE=2 SV=1	1,13	1,13	0,569374063242546	0,846785345038636	26,6258838312392	27,1925405628277	26,6234109353197	26,9172029562719	27,1520862359721	26,7516578076357	441721358587,131	501222357355,746	−0,182314175654479	0,0122278019935603	0,0074688165290414	1,13470256217388	0.18231417565448	0,881288218900451	-0.18231417565448	2997

Q924T2	sp|Q924T2|RT02_MOUSE 28S ribosomal protein S2, mitochondrial OS=Mus musculus GN=Mrps2 PE=2 SV=1	1,14	0,465996434590293	0,795722853632858	25,3867743256002	25,6624905516586	25,8861812107947	25,9600094693325	25,6404580141254	25,7149021750404	137260812799,978	155792352866,672	−0,18270461699914	0,00975446403288463	0,0064876677254931	1,13500969205026	0.182704616999144	0,881049745217255	-0.182704616999144	2998
Q9CQ45	sp|Q9CQ45|NENF_MOUSE Neudesin OS=Mus musculus GN=Nenf PE=1 SV=1	1,14	0,395008572187224	0,753881747531964	25,3191904935594	25,0980650300174	24,8802711146083	25,0998804841175	25,2084612346714	25,369136158521	79512139579,4297	90247881401,055	−0,18271792282167	0,00874378508416587	0,0053700973979875	1,1350201601719	0.182717922821673	0,881041619426871	-0.182717922821673	2999
Q9D7X1	sp|Q9D7X1|KCTD4_MOUSE BTB/POZ domain-containing protein KCTD4 OS=Mus musculus GN=Kctd4 PE=2 SV=1	1,14	0,712684393226929	0,917434617372953	26,1321115412	25,8014926834204	26,8620165414284	26,6541371203836	26,1606183781872	26,3614633838226	255172831220,83	289688765384,19	−0,18302900500734	0,0206602183598187	0,0094035494379818	1,13526492612174	0.183029005007346	0,880851664656086	-0.183029005007346	3000
Q69Z98-3	sp|Q69Z98-3|BRSK2_MOUSE Isoform 3 of Serine/threonine-protein kinase BRSK2 OS=Mus musculus GN=Brsk2;sp|Q69Z98|BRSK2_MOUSE Serine/threonine-protein kinase BRSK2 OS=Mus musculus GN=Brsk2 PE=1 SV=2;sp|Q69Z98-4|BRSK2_MOUSE Isoform 4 of Serine/threonine-prot	1,14	0,399065003200069	0,754780017703466	29,1006990223782	28,9577992893341	28,6029701816381	29,0269449893171	29,0558930771154	28,9598110128047	3511822201594,17	3987622720016,95	−0,18330911364737	0,00887155349273115	0,0016988049580923	1,13548536660164	0.183309113647378	0,880680658168996	-0.183309113647378	3001
P28028	sp|P28028|BRAF_MOUSE Serine/threonine-protein kinase B-raf OS=Mus musculus GN=Braf PE=1 SV=3	1,14	0,312345557707382	0,720394556114739	28,9940526281682	29,0477196217778	28,6599523335218	28,963339565092	29,0789957161334	29,0412682162963	3559263869412,24	4042432911669,58	−0,18364493850854	0,00726995144971478	0,0020318766924617	1,13574971117191	0.183644938508544	0,880475680656928	-0.183644938508545	3002
Q8CCB4	sp|Q8CCB4|VPS53_MOUSE Vacuolar protein sorting-associated protein 53 homolog OS=Mus musculus GN=Vps53 PE=2 SV=1;sp|Q8CCB4-2|VPS53_MOUSE Isoform 2 of Vacuolar protein sorting-associated protein 53 homolog OS=Mus musculus GN=Vps53	1,14	0,388335942713222	0,753319093070654	29,6323404854074	29,2839283038169	29,139589158536	29,450672574706	29,5177234927931	29,469468974537	5589733062396,26	6348808969263,9	−0,18370658016539	0,00863045976567833	0,0011732697587586	1,13579823909198	0.183706580165397	0,880438061604545	-0.183706580165397	3003
Q9D554	sp|Q9D554|SF3A3_MOUSE Splicing factor 3A subunit 3 OS=Mus musculus GN=Sf3a3 PE=2 SV=2	1,14	0,573943782797802	0,849998193752035	26,9699393539991	27,6856496210637	27,576491819139	27,5736087892473	27,5855513418751	27,4549509257709	802256001759,736	911207991154,328	−0,18371772181817	0,0140670163610671	0,0026219011299533	1,13580701067441	0.183717721818172	0,880431262179154	-0.183717721818172	3004
Q3TZZ7	sp|Q3TZZ7|ESYT2_MOUSE Extended synaptotagmin-2 OS=Mus musculus GN=Esyt2 PE=1 SV=1	1,14	0,414124592651947	0,766833335315752	27,7510871215627	27,5915719767911	27,343085730949	27,8881308498074	27,5571437909233	27,6226377001941	933227591405,882	1060014935069,63	−0,18378372460191	0,00745997273468428	0,0063300709162494	1,13585897462884	0.183783724601914	0,880390983684188	-0.183783724601914	3005
P21619	sp|P21619|LMNB2_MOUSE Lamin-B2 OS=Mus musculus GN=Lmnb2 PE=1 SV=2;sp|P21619-2|LMNB2_MOUSE Isoform B3 of Lamin-B2 OS=Mus musculus GN=Lmnb2	1,14	0,736530253688858	0,924701393764781	22,4762920876387	22,9075582228068	22,9075582228068	23,1903792338134	22,1923188032946	23,2912926988169	7694740222,57164	8741347975,32821	−0,18398314884268	0,0109380502428977	0,0265367192691904	1,136015995665	0.183983148842681	0,880269295340886	-0.183983148842681	3006
P47758	sp|P47758|SRPRB_MOUSE Signal recognition particle receptor subunit beta OS=Mus musculus GN=Srprb PE=1 SV=1	1,14	0,372758339437521	0,744143356508855	26,9761562920847	27,1437352154627	27,4568631562061	27,3351344193571	27,270293282656	27,3540715475072	644829356120,395	732576114612,779	−0,18406123860434	0,00897308025357586	0,0016081779078235	1,13607748726006	0.184061238604339	0,880221649679686	-0.184061238604339	3007
Q8K0D5	sp|Q8K0D5|EFGM_MOUSE Elongation factor G, mitochondrial OS=Mus musculus GN=Gfm1 PE=2 SV=1	1,14	0,399965411564308	0,755175958024291	28,5819748597617	28,3855524287391	28,0674871197023	28,4283424016431	28,4869189419964	28,5025208578432	2042110203717,02	2320013376124,2	−0,18407239905885	0,00915962189932695	0,0013735657732613	1,13608627580497	0.184072399058854	0,880214840454307	-0.184072399058854	3008
P05480	sp|P05480|SRC_MOUSE Neuronal proto-oncogene tyrosine-protein kinase Src OS=Mus musculus GN=Src PE=1 SV=4	1,14	0,34737368266221	0,732776399267436	27,7954933381178	28,0355976856729	28,2346990589835	28,2415791179015	28,1288847943823	28,0782614029079	1478323849218,91	1679597177111,18	−0,18415292026343	0,00784819682704604	0,0029699080267894	1,13614968601001	0.184152920263446	0,880165714353932	-0.184152920263446	3009
P38060	sp|P38060|HMGCL_MOUSE Hydroxymethylglutaryl-CoA lyase, mitochondrial OS=Mus musculus GN=Hmgcl PE=1 SV=2	1,14	0,662671580992546	0,895998303880002	28,5094418456896	28,1401525448235	29,1293211094485	28,7725746006029	28,7679720830482	28,6215713507648	2616796127531,28	2973337013191,33	−0,18428146537112	0,0174814473919477	0,0029903154593126	1,13625092222848	0.184281465371122	0,880087294484869	-0.184281465371122	3010
Q8VE42	sp|Q8VE42|ANR49_MOUSE Ankyrin repeat domain-containing protein 49 OS=Mus musculus GN=Ankrd49 PE=2 SV=1	1,14	0,61069841110674	0,866610575848175	23,6613231107722	23,8239823554939	24,2686184132209	23,8148854521229	23,936307566344	24,3866384851643	24403066229,5457	27734524184,9216	−0,18462054183743	0,0131436255201267	0,012527334521591	1,13651800655044	0.184620541837439	0,879880471964718	-0.184620541837439	3011
Q922Y1	sp|Q922Y1|UBXN1_MOUSE UBX domain-containing protein 1 OS=Mus musculus GN=Ubxn1 PE=1 SV=1	1,14	0,0219583206762292	0,206781779148142	29,0383137035681	29,0951539669868	29,1339128359005	29,3002269215392	29,2199061542331	29,1319348639858	4297810554078,57	4885808917921,28	−0,18499555077273	0,00165297883461842	0,0028809932851996	1,13681346733273	0.184995550772734	0,879651788737395	-0.184995550772733	3012
Q9JJ43-2	sp|Q9JJ43-2|RFOX1_MOUSE Isoform 2 of RNA binding protein fox-1 homolog 1 OS=Mus musculus GN=Rbfox1;sp|Q9JJ43-4|RFOX1_MOUSE Isoform 4 of RNA binding protein fox-1 homolog 1 OS=Mus musculus GN=Rbfox1;sp|Q9JJ43|RFOX1_MOUSE RNA binding protein fox-1 homolog	1,14	0,323002328296323	0,726515147738855	27,3151166099119	27,436658796006	27,5128434547509	27,7681093764479	27,3712277656425	27,5100018761904	811004646362,964	921971060955,386	−0,18501128783899	0,0036368023081994	0,0073107185102367	1,13682586787889	0.185011287838996	0,879642193457315	-0.185011287838996	3013
Q8VEE4	sp|Q8VEE4|RFA1_MOUSE Replication protein A 70 kDa DNA-binding subunit OS=Mus musculus GN=Rpa1 PE=1 SV=1	1,14	0,819476229023229	0,952523073888973	25,0513945111618	26,1835363389388	26,0346229337524	25,0187798247048	26,2212886157278	26,4148721289321	153431174364,997	174463291016,004	−0,18533186809400	0,0238843337874521	0,02922042976663	1,13707850922769	0.185331868094003	0,87944675049677	-0.185331868094003	3014
P58404-2	sp|P58404-2|STRN4_MOUSE Isoform 2 of Striatin-4 OS=Mus musculus GN=Strn4;sp|P58404|STRN4_MOUSE Striatin-4 OS=Mus musculus GN=Strn4 PE=1 SV=2	1,14	0,35455348125365	0,732843629012038	29,3037700876513	29,0311658380649	28,8266770279669	29,2013315647317	29,1565083052916	29,1893852573842	4148927506325,45	4718010751808,11	−0,18544025691284	0,00823833669981744	0,0007954046959926	1,13716394046776	0.185440256912847	0,879380680668316	-0.185440256912847	3015
Q9D0A3	sp|Q9D0A3|ARPIN_MOUSE Arpin OS=Mus musculus GN=Arpin PE=1 SV=1	1,14	0,769202905289065	0,936218563920244	26,4743118091212	26,8620165414284	27,3177386114909	27,6547108054017	26,9928732615539	26,3924755423625	474102313847,063	539200434898,16	−0,18562322601571	0,0157029993274634	0,023372396421771	1,13730816988186	0.185623226015712	0,879269160709427	-0.185623226015712	3016

Q9JM62-2	sp|Q9JM62-2|REEP6_MOUSE Isoform 2 of Receptor expression-enhancing protein 6 OS=Mus musculus GN=Reep6;sp|Q9JM62|REEP6_MOUSE Receptor expression-enhancing protein 6 OS=Mus musculus GN=Reep6 PE=1 SV=1	1,14	0,654310818034468	0,891901943526937	23,1009237110647	22,4762920876387	22,4525488290568	22,4968102137115	22,7554239515794	23,164049627614	7052078332,43881	8021794051,22287	−0,18587642758760	0,0162139483968302	0,0147516854891192	1,13750779175601	0.185876427587601	0,879114857276069	-0.185876427587601	3017
Q9QYC0	sp|Q9QYC0|ADDA_MOUSE Alpha-adducin OS=Mus musculus GN=Add1 PE=1 SV=2	1,14	0,464292000476139	0,79405478152268	31,7966976397976	31,2915687270389	31,3949762767942	31,6843541136303	31,7308789356197	31,4546926223212	47626679533246,7	54178678273616,2	−0,18595522893527	0,00847268174912598	0,0046758854020050	1,13756992518859	0.185955228935271	0,879066840514635	-0.185955228935271	3018
Q61161	sp|Q61161|M4K2_MOUSE Mitogen-activated protein kinase kinase kinase kinase 2 OS=Mus musculus GN=Map4k2 PE=1 SV=1	1,14	0,433005151907495	0,77653511833588 3	25,8783648256611	25,5763604830779	25,4664846831164	25,9735589231531	25,7383458751693	25,5972870851289	136611003099,856	155471866258,939	−0,18657985032003	0,00831803678580751	0,0073764498873637	1,13806254789958	0.186579850320034	0,878686326903221	-0.186579850320034	3019
P12970	sp|P12970|RL7A_MOUSE 60S ribosomal protein L7a OS=Mus musculus GN=Rpl7a PE=1 SV=2	1,14	0,645951929737497	0,889692946522666	26,4796440426437	26,960898296503	27,2162114865181	27,2381962259367	26,6553553897964	27,1520862359721	474527120138,53	540203964504,601	−0,18701368528310	0,013911348000691 8	0,0116454959005364	1,13840482783555	0.187013685283105	0,878422135560777	-0.187013685283105	3020
Q99LU0	sp|Q99LU0|CH1B1_MOUSE Charged multivesicular body protein 1b-1 OS=Mus musculus GN=Chmp1b1 PE=1 SV=1	1,14	0,52150524700326	0,823916573667872	26,4148721289321	26,7912227971815	26,7982568071736	27,0927002045124	26,7489665802186	26,5520528301124	381782627041,002	434693289735,672	−0,18724637060081	0,00822498638606787	0,010210726222272	1,13858845046133	0.187246370600816	0,878280470520159	-0.187246370600816	3021
P26040	sp|P26040|EZRI_MOUSE Ezrin OS=Mus musculus GN=Ezr PE=1 SV=3;sp|P31977|EZRI_RAT Ezrin OS=Rattus norvegicus GN=Ezr PE=1 SV=3;sp|P15311|EZRI_HUMAN Ezrin OS=Homo sapiens GN=EZR PE=1 SV=4	1,14	0,493765411601519	0,808514142589933	30,2119986671099	30,0547925878358	29,6166517537297	30,1944393020597	30,1096724551239	29,9691108569161	10279242326161,1	11705432950539,2	−0,18744436792839	0,0102974399459006	0,0037821747765815	1,13874472253158	0.187444367928393	0,878159942446861	-0.187444367928393	3022
Q80ZI6	sp|Q80ZI6|LRSM1_MOUSE E3 ubiquitin-protein ligase LRSAM1 OS=Mus musculus GN=Lrsam1 PE=2 SV=1	1,14	0,323409521367432	0,726710800736026	27,1138793678718	26,8522659694571	26,660624355741	27,0220262352562	27,0068439722442	26,9881320866984	469807981273,914	535072148307,495	−0,18766221281391	0,00846589381292424	0,0006286708870282	1,13891668433689	0.187662212813914	0,878027351563672	-0.187662212813914	3023
P81377	sp|P81377|KAP1_RAT cAMP-dependent protein kinase type I-beta regulatory subunit OS=Rattus norvegicus GN=Prkar1b PE=2 SV=2;sp|P12849|KAP1_MOUSE cAMP-dependent protein kinase type I-beta regulatory subunit OS=Mus musculus GN=Prkar1b PE=2 SV=2	1,14	0,0235760582929232	0,21524005202738	27,9732115534468	27,791225622099	27,8832358468707	28,0108060548788	27,9732115534468	28,0545669515707	1285999913059,79	1464978261858,2	−0,18798871218281	0,00326350480941125	0,0014534976833789	1,13917446415106	0.187988712182808	0,877828665818296	-0.187988712182808	3024
Q3UVG3	sp|Q3UVG3|F91A1_MOUSE Protein FAM91A1 OS=Mus musculus GN=Fam91a1 PE=2 SV=1	1,14	0,597654288409818	0,861464850775537	26,0393274349537	25,3890942487746	25,2670557453431	25,8025240721104	25,5969041943702	25,6870742225767	126709023736,988	144349146407,626	−0,18804330496835	0,0162389212578066	0,0040111541417695	1,13921757228004	0.188043304968348	0,87779544867675	-0.188043304968349	3025
Q6A058	sp|Q6A058|ARMX2_MOUSE Armadillo repeat-containing X-linked protein 2 OS=Mus musculus GN=Armcx2 PE=2 SV=2	1,14	0,251086423804411	0,673538059877323	23,6519620919524	23,4411557402849	23,4099585585598	23,7908431155132	23,5004085167368	23,6043336988036	16082948745,7978	18331042607,42	−0,18875690053754	0,00560155883448934	0,0062272356177164	1,13978119915414	0.188756900537544	0,877361374921892	-0.188756900537544	3026
P56212-2	sp|P56212-2|ARP19_MOUSE Isoform ARPP-16 of cAMP-regulated phosphoprotein 19 OS=Mus musculus GN=Arpp19;sp|P56212|ARP19_MOUSE cAMP-regulated phosphoprotein 19 OS=Mus musculus GN=Arpp19 PE=1 SV=2	1,14	0,579509016908514	0,852639036547081	24,0418006571554	23,8568543067188	23,3156309902351	23,6905689153765	23,9922099240447	23,9242273809443	20385614511,3939	23236776940,68	−0,18885852686149	0,015897245731304 7	0,0066287646603142	1,13986149044919	0.188858526861497	0,877299574008707	-0.188858526861497	3027
Q62093	sp|Q62093|SRSF2_MOUSE Serine/arginine-rich splicing factor 2 OS=Mus musculus GN=Srsf2 PE=1 SV=4	1,14	0,493491932861208	0,808357951373881	28,3714205600626	28,8313933089507	28,9989754780626	28,9384359919015	28,8167867144637	28,8395124734223	3012916861274,53	3434566135353,3	−0,18896700139700	0,011308295158705 2	0,0022407764037057	1,13994719850995	0.188967001397005	0,877233613370093	-0.188967001397005	3028
Q08274	sp|Q08274|DMWD_MOUSE Dystrophia myotonica WD repeat-containing protein OS=Mus musculus GN=Dmwd PE=2 SV=3	1,14	0,49805890243247	0,809992604135071	27,039238096365	26,8636371325686	26,4086406469355	26,9742992198464	26,8149454374718	26,9157427213204	422943861458,193	482218158476,294	−0,18921979525892	0,0121570784873231	0,0029962673840105	1,14014696138097	0.189219795258927	0,87707991502146	-0.189219795258927	3029
Q3U2V3	sp|Q3U2V3|NUD18_MOUSE 8-oxo-dGDP phosphatase NUDT18 OS=Mus musculus GN=Nudt18 PE=2 SV=1;sp|Q3U2V3-2|NUD18_MOUSE Isoform 2 of 8-oxo-dGDP phosphatase NUDT18 OS=Mus musculus GN=Nudt18	1,14	0,211054297498075	0,641644645910089	26,4958547362392	26,2537200098112	26,5056129060047	26,6783050470106	26,4656131249857	26,5056129060047	297415469082,14	339195913276,138	−0,18963910167310	0,00540142286322716	0,0042574345474276	1,14047838306103	0.189639101673107	0,876825036627182	-0.189639101673107	3030
Q3TFD2-2	sp|Q3TFD2-2|PCAT1_MOUSE Isoform 2 of Lysophosphatidylcholine acyltransferase 1 OS=Mus musculus GN=Lpcat1;sp|Q3TFD2|PCAT1_MOUSE Lysophosphatidylcholine acyltransferase 1 OS=Mus musculus GN=Lpcat1 PE=1 SV=1;sp|Q1HAQ0|PCAT1_RAT Lysophosphatidylcholine acyl	1,14	0,43873167038676	0,779719284215154	25,1183229982551	25,5677487880404	25,2008916720812	25,6042732888588	25,4142056413629	25,2629129193492	96774995424,0355	110372916211,76	−0,18967996132063	0,00945734614873849	0,0067269417316232	1,1405106838615	0.189679961320638	0,876800203759809	-0.189679961320638	3031
P12382	sp|P12382|PFKAL_MOUSE ATP-dependent 6-phosphofructokinase, liver type OS=Mus musculus GN=Pfkl PE=1 SV=4	1,14	0,336749321173629	0,7295967392874	31,6602286177643	31,3364515285498	31,2250526281598	31,6172183495446	31,5283219649286	31,471637043656	43650866895155,5	49801146380173,4	−0,19016864659530	0,00719771092073906	0,0023267078936387	1,14089707541869	0.190168646595302	0,876503254803258	-0.190168646595302	3032
O35709	sp|O35709|ENC1_MOUSE Ectoderm-neural cortex protein 1 OS=Mus musculus GN=Enc1 PE=2 SV=2	1,14	0,827143605612921	0,955959931044103	20,543586508048	21,6089602302478	22,1997126389534	21,3395865968786	21,1169734730192	22,2911985568517	2069873074,65035	2361555169,10074	−0,19019493530982	0,0391278329831423	0,0288977796720538	1,14091786497569	0.190194935309829	0,876487283351733	-0.190194935309828	3033
P28661-2	sp|P28661-2|SEPT4_MOUSE Isoform 2 of Septin-4 OS=Mus musculus GN=Sept4;sp|P28661|SEPT4_MOUSE Septin-4 OS=Mus musculus GN=Sept4 PE=1 SV=1;sp|P28661-3|SEPT4_MOUSE Isoform 3 of Septin-4 OS=Mus musculus GN=Sept4;sp|P28661-4|SEPT4_MOUSE Isoform 4 of Septin-	1,14	0,204528752626872	0,635648869851434	28,1229950257761	28,415569281999	28,3939473383592	28,399855058049	28,5307522510992	28,3974533755861	1973514768404,63	225165562505 8,21	−0,19021887880555	0,00575874685925428	0,0026817539822174	1,14093680022391	0.190218878805552	0,876472736968208	-0.190218878805552	3034

Q8N7N5	sp|Q8N7N5|DCAF8_MOUSE DDB1- and CUL4-associated factor 8 OS=Mus musculus GN=Dcaf8 PE=1 SV=1;sp|Q8N7N5-2|DCAF8_MOUSE Isoform 2 of DDB1- and CUL4-associated factor 8 OS=Mus musculus GN=Dcaf8	1,14	0,266171620550426	0,685404159524725	25,4761717842147	25,4520364849735	25,1593816694031	25,6146994185905	25,4494598461444	25,4189974157507	103468181734,268	118051352823,441	−0,190227392290521	0,00695297310670906	0,0041304083771919	1,14094353302377	0.190227392290527	0,87646756483186	-0.190227392290527	3035
Q9JHE7	sp|Q9JHE7|TSSC4_MOUSE Protein TSSC4 OS=Mus musculus GN=Tssc4 PE=2 SV=1	1,14	0,753806605401664	0,930268673713472	23,2726265887226	23,7908431155132	22,8052947352916	23,088004659985	24,040363892071	23,1361785433251	13017860635,408	14853712908,6262	−0,190331224956695	0,0211679602571801	0,0229054031493046	1,1410256511907	0.190331224956692	0,876404486574394	-0.190331224956692	3036
P48381	sp|P48381|RFX3_MOUSE Transcription factor RFX3 OS=Mus musculus GN=Rfx3 PE=1 SV=2;sp|P48379-2|RFX2_MOUSE Isoform 2 of DNA-binding protein RFX2 OS=Mus musculus GN=Rfx2;sp|P48379|RFX2_MOUSE DNA-binding protein RFX2 OS=Mus musculus GN=Rfx2 PE=2 SV=2;sp|P48	1,14	0,403151156569517	0,758037738056768	23,3655480943154	23,0529989350732	22,9428230979447	23,4411557402849	23,1560227153882	23,160204023241	10992254321,3806	12543404497,1469	−0,190441685252121	0,0094845672318189	0,0070284157533205	1,1411301744076	0.190441685252118	0,876337387021273	-0.190441685252118	3037
Q8CHP8	sp|Q8CHP8|PGP_MOUSE Phosphoglycolate phosphatase OS=Mus musculus GN=Pgp PE=1 SV=1	1,14	0,377033215769299	0,74727992066734	30,343830029212	30,6071786088968	30,7847908092274	30,8636183629237	30,6125340776466	30,6563201848299	19059748440615,9	21754118426195,1	−0,190759475615561	0,00725557063607291	0,0043672713854553	1,14136440436105	0.190759475615562	0,87614437262901	-0.190759475615562	3038
P97770-2	sp|P97770-2|THUM3_MOUSE Isoform 2 of THUMP domain-containing protein 3 OS=Mus musculus GN=Thumpd3;sp|P97770|THUM3_MOUSE THUMP domain-containing protein 3 OS=Mus musculus GN=Thumpd3 PE=2 SV=1;sp|P97770-3|THUM3_MOUSE Isoform 3 of THUMP domain-containing p	1,14	0,373167396656842	0,744143356508855	25,2008916720812	25,6830722098595	25,5972870851289	25,6245416244004	25,6146994185905	25,6392323739276	117976385954,573	134678703630,461	−0,191023619508906	0,0100897058687025	0,0004817761826041	1,14157339658056	0.191023619508911	0,875983973518807	-0.191023619508911	3039
Q9DB16	sp|Q9DB16|CB39L_MOUSE Calcium-binding protein 39-like OS=Mus musculus GN=Cab39l PE=2 SV=3;sp|Q9DB16-2|CB39L_MOUSE Isoform 2 of Calcium-binding protein 39-like OS=Mus musculus GN=Cab39l	1,14	0,492375819878071	0,80770032675628	26,2900150804149	26,2503112648405	26,5462394020755	26,7585971508416	26,1785692818595	26,5473065949047	281159674751,353	321037681980,843	−0,191352953328724	0,00609297581646313	0,0110796683275503	1,14183402105852	0.191352953328723	0,875784029515047	-0.191352953328723	3040
O70435	sp|O70435|PSA3_MOUSE Proteasome subunit alpha type-3 OS=Mus musculus GN=Psma3 PE=1 SV=3	1,14	0,541571182599812	0,83243873952609	30,2058798248574	30,766243128978	30,8724847418601	30,8605365941129	30,6271401858965	30,7552693729942	19763723602124,5	22570135666643,8	−0,191560305651291	0,0116987757602109	0,0038014182298483	1,14199814372114	0.191560305651293	0,875658165906957	-0.191560305651293	3041
O55126	sp|O55126|NIPS2_MOUSE Protein NipSnap homolog 2 OS=Mus musculus GN=Gbas PE=2 SV=1	1,14	0,574712119554224	0,849998193752035	30,4692900893302	30,9878465239167	31,2206366567217	31,1921742262174	30,9306955015522	30,9542765997972	26090466360630,8	29805541320995,8	−0,192057843220553	0,0124503635758134	0,0046619038500595	1,14239204884321	0.192057843220552	0,875356232575853	-0.192057843220552	3042
Q9R0M5	sp|Q9R0M5|TPK1_MOUSE Thiamin pyrophosphokinase 1 OS=Mus musculus GN=Tpk1 PE=1 SV=1	1,14	0,0434296937449402	0,30674678910092	23,3379615440826	23,3098271341832	23,3964502955791	23,3655480943154	23,5718044898966	23,5068458156028	13802004303,5124	15770382090,9686	−0,19233982680117	0,00189262180021569	0,0044910632092322	1,14261535818789	0.192339826801165	0,875185155559202	-0.192339826801165	3043
Q61696	sp|Q61696|HS71A_MOUSE Heat shock 70 kDa protein 1A OS=Mus musculus GN=Hspa1a PE=1 SV=2;sp|P17879|HS71B_MOUSE Heat shock 70 kDa protein 1B OS=Mus musculus GN=Hspa1b PE=1 SV=3	1,14	0,447233626159473	0,785601172266968	29,5895854758228	30,1229682803372	30,0077005409481	30,1714331301119	29,9997657346318	29,9490951305956	9735026066633,4	11123687933307,4	−0,192378429598919	0,00938434282661537	0,0038790519405078	1,14264593203644	0.192378429598914	0,875161738175344	-0.192378429598914	3044
P08249	sp|P08249|MDHM_MOUSE Malate dehydrogenase, mitochondrial OS=Mus musculus GN=Mdh2 PE=1 SV=3	1,14	0,470661220675898	0,796841208745954	35,4772270626791	35,9416998882443	36,084202935126	35,9416998882443	36,0203651980554	35,9416998882443	3653188083051020	4175129030234590	−0,192664751792486	0,00885745517270522	0,0012627206605643	1,14287272796195	0.192664751792485	0,874988067816854	-0.192664751792485	3045
Q8BIZ1-2	sp|Q8BIZ1-2|ANS1B_MOUSE Isoform 2 of Ankyrin repeat and sterile alpha motif domain-containing protein 1B OS=Mus musculus GN=Anks1b;sp|Q8BIZ1-4|ANS1B_MOUSE Isoform 4 of Ankyrin repeat and sterile alpha motif domain-containing protein 1B OS=Mus musculus GN	1,14	0,309399992259006	0,719081702439134	27,6931390764562	28,1431261777461	27,860378365634	28,0336619814108	28,0173481149224	28,0469322356155	1307164242765,04	1494252867964,82	−0,19298388729326	0,00815269188526821	0,0005286026332796	1,14312556837084	0.192983887293255	0,874794534974127	-0.192983887293255	3046
Q99J99	sp|Q99J99|THTM_MOUSE 3-mercaptopyruvate sulfurtransferase OS=Mus musculus GN=Mpst PE=1 SV=3	1,14	0,457781475551682	0,790957322506963	28,7908543389162	29,209851847461	29,2251086014483	29,4282378281537	29,0635435904717	29,1365569873341	4238673981055,36	4847315368332,71	−0,193572942574219	0,00847561018782636	0,0066062179471161	1,14359240413338	0.19357294257422	0,874437427518421	-0.19357294257422	3047
Q8BTH8	sp|Q8BTH8|KC1G1_MOUSE Casein kinase I isoform gamma-1 OS=Mus musculus GN=Csnk1g1 PE=1 SV=2;sp|Q8BVP5|KC1G2_MOUSE Casein kinase I isoform gamma-2 OS=Mus musculus GN=Csnk1g2 PE=1 SV=1;sp|Q8C4X2|KC1G3_MOUSE Casein kinase I isoform gamma-3 OS=Mus musculus G	1,14	0,794530812475374	0,943719708239887	25,114611226522	23,6075161552656	24,9442410806871	25,0796635391775	24,4443019138267	24,5452995178382	46163540377,5159	52798836439,2719	−0,193752264871188	0,0336115895379041	0,0138286462232211	1,14373455778075	0.193752264871189	0,874328744547472	-0.193752264871189	3048
Q61823	sp|Q61823|PDCD4_MOUSE Programmed cell death protein 4 OS=Mus musculus GN=Pdcd4 PE=1 SV=1	1,14	0,5512855218188	0,838423390503939	24,391301183712	24,534409034057	25,0570831721089	24,9825849920557	24,6425366875427	24,7608480287448	51298693961,5222	58677561655,676	−0,193886825084556	0,0142111470383391	0,0069620248499140	1,14384123891514	0.193886825084557	0,874247199679947	-0.193886825084557	3049

Q3UQN2	sp|Q3UQN2|FCHO2_MOUSE FCH domain only protein 2 OS=Mus musculus GN=Fcho2 PE=1 SV=1;sp|Q3UQN2-3|FCHO2_MOUSE Isoform 3 of FCH domain only protein 2 OS=Mus musculus GN=Fcho2	1,14	0,684627101252052	0,905719152603632	24,8516604772475	23,9723375129221	23,9556768269568	24,3457169607271	24,6781682335195	24,1607946176825	34350815825,4178	39315837742,8976	−0,19476623251237	0,0211276531476994	0,0107468813333822	1,14453868993137	0.194766232512371	0,873714457009714	-0.194766232512371	3050
Q8C0L6	sp|Q8C0L6|PAOX_MOUSE Peroxisomal N(1)-acetyl-spermine/spermidine oxidase OS=Mus musculus GN=Paox PE=1 SV=3	1,15	0,496119627502335	0,809158098897987	25,8014926834204	26,3537858638036	26,4297525807184	26,3722038170175	26,2965204560717	26,3230679595192	237874812891,899	272416344394,363	−0,19561074284274	0,0130905272782142	0,0014583557589394	1,1452088646231	0.195610742842745	0,873203160481223	-0.195610742842745	3051
Q80X80	sp|Q80X80|C2C2L_MOUSE C2 domain-containing protein 2-like OS=Mus musculus GN=C2cd2l PE=1 SV=3	1,15	0,292831017457062	0,711969538636193	29,6166517537297	29,3820077371923	29,1799288907371	29,4785745078136	29,5350096320515	29,5724244467374	5823151895534,9	6670190451305,09	−0,19592770307659	0,00743594446566915	0,0015999742277597	1,14546049475709	0.195927703076595	0,873011338738543	-0.195927703076595	3052
Q8CBE3	sp|Q8CBE3|WDR37_MOUSE WD repeat-containing protein 37 OS=Mus musculus GN=Wdr37 PE=2 SV=1	1,15	0,338655003897848	0,729620681454069	29,7170699033562	30,1695834981336	30,0330859729261	30,1824637696736	30,0218464917338	30,1229682803372	10404364090435,5	11918260637530,8	−0,19598491189103	0,00774409720395473	0,0026969483276834	1,14550591789526	0.19598491189103	0,872976720921171	-0.19598491189103	3053
Q04899	sp|Q04899|CDK18_MOUSE Cyclin-dependent kinase 18 OS=Mus musculus GN=Cdk18 PE=1 SV=1	1,15	0,275027823940047	0,696624605392787	27,5509487648623	27,30041308278	27,7288300481742	27,680487545215	27,6529615702981	27,6547108054017	900963543920,631	1032206617431,91	−0,19619114888350	0,00781904734525142	0,0005571399074060	1,14566968263795	0.196191148883512	0,872851935557431	-0.196191148883512	3054
Q8R1F1	sp|Q8R1F1|NIBL1_MOUSE Niban-like protein 1 OS=Mus musculus GN=Fam129b PE=2 SV=2	1,15	0,53991646195652	0,831584940896045	27,2393633429942	26,7551771799298	26,7506588434913	27,2953748650026	27,0846207130066	26,7734005079762	488725322279,634	559960469867,547	−0,19630112767697	0,0104349892547993	0,0097073073400601	1,14575702207457	0.196301127676975	0,872785399289412	-0.196301127676975	3055
Q9ERI6	sp|Q9ERI6|RDH14_MOUSE Retinol dehydrogenase 14 OS=Mus musculus GN=Rdh14 PE=2 SV=1	1,15	0,536251009507344	0,830981962695391	27,2551673122194	27,7318880781206	27,9465752622869	27,9221362330221	27,6376773679146	27,7823931945621	1013614276577,77	1161502566154,41	−0,19648359171561	0,0128011809000361	0,0051199751788884	1,1459019402094	0.196483591715622	0,872675021230233	-0.196483591715622	3056
Q9CXE7	sp|Q9CXE7|TMED5_MOUSE Transmembrane emp24 domain-containing protein 5 OS=Mus musculus GN=Tmed5 PE=2 SV=1	1,15	0,737071963569284	0,924701393764781	25,1454481767251	25,0561523437009	24,4418972929147	24,3457169607271	25,2593441260161	25,4471000951404	63937120028,3667	73267800138,6123	−0,19652553839658	0,0153943310962748	0,0235520265716916	1,14593525804894	0.196525538396582	0,872649648377687	-0.196525538396582	3057
Q8QZR5	sp|Q8QZR5|ALAT1_MOUSE Alanine aminotransferase 1 OS=Mus musculus GN=Gpt PE=2 SV=3	1,15	0,673360788410821	0,900894908348996	25,4301759017466	25,8115297761429	25,7062486810014	25,4643078754517	25,4993244819944	26,3957362099935	137834306011,2	158094088432,907	−0,19784841280815	0,00767848835823 33	0,0204736303620052	1,14698650145966	0.197848412808145	0,871849841935709	-0.197848412808145	3058
Q84WV1	sp|Q84WV1|TCPG_ARATH T-complex protein 1 subunit gamma OS=Arabidopsis thaliana GN=CCT3 PE=1 SV=1	1,15	0,136245262955371	0,524972197414624	26,6219754182725	26,8911690458988	26,8832357761076	26,9807462280019	26,9359933509139	26,8914057622921	435078979414,128	499088088445,507	−0,19801715637376	0,00571593803964266	0,0016583820164259	1,14712066558024	0.198017156373768	0,871747872743775	-0.198017156373768	3059
Q91YN0	sp|Q91YN0|CL004_MOUSE Protein C12orf4 homolog OS=Mus musculus GN=D6Wsu163e PE=2 SV=1	1,15	0,518994160036273	0,82335612354586	25,32730793297	25,6458690980175	25,7260541994384	26,0446839509163	25,6027289502231	25,4643078754517	126867669805,572	145567673519,927	−0,19836554089035	0,00824967035061273	0,0117930870601799	1,14739770772974	0.198365540890354	0,871537386961157	-0.198365540890354	3060
Q9CR27	sp|Q9CR27|CCD53_MOUSE WASH complex subunit CCDC53 OS=Mus musculus GN=Ccdc53 PE=2 SV=1	1,15	0,775725309056259	0,938619589104496	22,2206266183963	22,7593573011881	23,6813632656051	22,6399360793508	23,4227004245546	23,0112645071695	8704584940,08737	9987834809,77754	−0,19839645290146	0,0322758563235877	0,0170058466054274	1,14742229279427	0.198396452901468	0,871518713101468	-0.198396452901468	3061
Q62167	sp|Q62167|DDX3X_MOUSE ATP-dependent RNA helicase DDX3X OS=Mus musculus GN=Ddx3x PE=1 SV=3;sp|O00571-2|DDX3X_HUMAN Isoform 2 of ATP-dependent RNA helicase DDX3X OS=Homo sapiens GN=DDX3X;sp|O00571|DDX3X_HUMAN ATP-dependent RNA helicase DDX3X OS=Homo sapie	1,15	0,529839554230833	0,829480190327658	28,0336619814108	28,3908102287772	27,6784770577248	28,2841998398392	28,2028134144342	28,0287075649402	1496748826377,44	1717527615380,56	−0,19850115669385	0,0127046806170388	0,0046329859590272	1,14750557014798	0.198501156693863	0,871455464805321	-0.198501156693863	3062
Q8R3I3	sp|Q8R3I3|COG6_MOUSE Conserved oligomeric Golgi complex subunit 6 OS=Mus musculus GN=Cog6 PE=2 SV=2	1,15	0,437031033241129	0,778453531535867	24,6713112340392	25,1266580132717	25,1789507460175	25,2670557453431	25,0158762888834	25,1070055387849	71453066922,7806	81999516758,4603	−0,19861947133620	0,0111721215550798	0,0050599283411801	1,14759968031991	0.198619471336206	0,871384000143006	-0.198619471336205	3063
Q6PGF7	sp|Q6PGF7|EXOC8_MOUSE Exocyst complex component 8 OS=Mus musculus GN=Exoc8 PE=1 SV=1	1,15	0,358426271228751	0,732886916572711	29,383338090787	29,2004756920356	28,9505536434768	29,4637941408889	29,1799288907371	29,3037700876513	4697833098377,12	5391426053338,18	−0,19867146284108	0,00744586640585774	0,0048545935108318	1,14764103799274	0.198671462841084	0,871352597977002	-0.198671462841084	3064
P97760	sp|P97760|RPB3_MOUSE DNA-directed RNA polymerase II subunit RPB3 OS=Mus musculus GN=Polr2c PE=1 SV=1	1,15	0,723046238364086	0,921631098094813	24,333802902532	25,1021464688603	25,3648968031627	25,1555905514942	24,6282909811731	25,4301759017466	67380088712,1307	77330360557,4285	−0,19871261181266	0,0214894008390716	0,0162546209800407	1,14767377181423	0.19871261181266	0,871327745356776	-0.19871261181266	3065
P84089	sp|P84089|ERH_MOUSE Enhancer of rudimentary homolog OS=Mus musculus GN=Erh PE=1 SV=1	1,15	0,750583799278092	0,928854093158237	24,7017312592705	24,7376841280185	24,6044469627772	25,663480608275	24,5525942656835	24,2411722072097	52353647653,0645	60088382705,2525	−0,19879603384668	0,0027928489319656	0,0301245832976486	1,14774013653154	0.198796033846685	0,871277363377732	-0.198796033846685	3066
Q9DB34	sp|Q9DB34|CHM2A_MOUSE Charged multivesicular body protein 2a OS=Mus musculus GN=Chmp2a PE=1 SV=1	1,15	0,590622764779966	0,858967231443853	27,0764572082296	27,6980271242862	27,9056879546371	27,8204849529863	27,6317151295644	27,6419884256892	931495717031,464	1069340090274,45	−0,19909971633655	0,01565631203587	0,0038322183370995	1,14798175742802	0.199099716336557	0,871093981702671	-0.199099716336557	3067
Q9WVM3	sp|Q9WVM3|APC7_MOUSE Anaphase-promoting complex subunit 7 OS=Mus musculus GN=Anapc7 PE=1 SV=3	1,15	0,648643393023384	0,889714023663571	23,5193424313764	23,4391885073479	24,2701951976947	23,5891400062898	23,9878581132619	24,0659982136174	20483975042,1648	23517220517,637	−0,19922185281321	0,0193067658769996	0,0107097246381551	1,14807894801807	0.199221852813215	0,871020239266907	-0.199221852813215	3068
P22315	sp|P22315|HEMH_MOUSE Ferrochelatase, mitochondrial OS=Mus musculus GN=Fech PE=1 SV=2	1,15	0,338533768419364	0,729620681454069	29,6508210992447	29,1702498422972	29,4637941408889	29,497763870862	29,6230833170184	29,57918938533	6033137828012,06	6928599786718,97	−0,19965528362203	0,00823170809589693	0,0021507153715570	1,14842391873583	0.199655283622039	0,870758596791322	-0.199655283622039	3069
Q9CZN8	sp|Q9CZN8|GATA_MOUSE Glutamyl-tRNA(Gln) amidotransferase subunit A, mitochondrial OS=Mus musculus GN=Qrsl1 PE=2 SV=1	1,15	0,569047755369261	0,846700858951536	25,8205849296101	26,6322995250514	26,3498310529515	26,5056129060047	26,3382310811099	26,3744753513748	255777007115,121	293782767806,505	−0,19986319525993	0,0156870984671444	0,0033483466394 81	1,14858943389809	0.199863195259931	0,870633117881115	-0.199863195259931	3070

O55143	sp|O55143|AT2A2_MOUSE Sarcoplasmic/endoplasmic reticulum calcium ATPase 2 OS=Mus musculus GN=Atp2a2 PE=1 SV=2;sp|O55143-2|AT2A2_MOUSE Isoform 2 of Sarcoplasmic/endoplasmic reticulum calcium ATPase 2 OS=Mus musculus GN=Atp2a2	1,15	0,416356553089799	0,767877733621866	32,6728334389707	32,2397875300273	32,1255304366215	32,4866102924521	32,5465656838934	32,420921979523	111612087231841	128211110063616	−0,200028008439734	0,00892569353606453	0,0019345614518022	1,14872065601009	0.200028008439724	0,870533662616766	-0.200028008439724	3071
D3Z7P3	sp|D3Z7P3|GLSK_MOUSE Glutaminase kidney isoform, mitochondrial OS=Mus musculus GN=Gls PE=1 SV=1	1,15	0,550792094123048	0,838423390503939	32,0651775689469	32,7150311669666	32,6569761311727	32,8262003127847	32,5334327252068	32,4938038454955	127490328450805	146465686410248	−0,20017490660715	0,0110719815027631	0,0055660940825975	1,14883762705784	0.200174906607148	0,870445027606716	-0.200174906607147	3072
Q9EQ20	sp|Q9EQ20|MMSA_MOUSE Methylmalonate-semialdehyde dehydrogenase [acylating], mitochondrial OS=Mus musculus GN=Aldh6a1 PE=1 SV=1	1,15	0,219837672071934	0,648792713611515	31,271198233726	31,4973921613793	31,4444699733367	31,6337004171238	31,6337004171238	31,3626131085233	43524863095138,3	50014695137393,8	−0,200512284654997	0,0037674020151951	0,0049618189009995	1,14910631718863	0.200512284654999	0,870241495535912	-0.200512284654999	3073
Q8VBW6	sp|Q8VBW6|ULA1_MOUSE NEDD8-activating enzyme E1 regulatory subunit OS=Mus musculus GN=Nae1 PE=1 SV=1	1,15	0,386473774881113	0,752468171504595	29,1775307111958	29,6000841427096	29,5767627429087	29,7557574407144	29,5272518601179	29,4883704069559	6174562828535,35	7095343945950,36	−0,200535625847512	0,00806464081648307	0,0048821785707331	1,14912490859429	0.200535625847511	0,87022741611553	-0.200535625847512	3074
Q8C4Y3	sp|Q8C4Y3|NELFB_MOUSE Negative elongation factor B OS=Mus musculus GN=Nelfb PE=1 SV=2	1,15	0,75831376338679	0,933040436978216	23,6043336988036	24,8003069736163	23,8568543067188	23,7132586719014	24,615571684966	24,3497113967141	28901663560,2407	33212115949,3047	−0,200557104435805	0,0261704989338822	0,0191390880315737	1,14914201668979	0.200557104435804	0,870214460420302	-0.200557104435804	3075
Q8CGA0	sp|Q8CGA0|PPM1F_MOUSE Protein phosphatase 1F OS=Mus musculus GN=Ppm1f PE=2 SV=1	1,15	0,151696714711937	0,553053323757015	28,1946741447067	28,299685761744	28,2885021532674	28,4314922090982	28,2288491072506	28,5402325045527	1877484603288,94	2157974731844,01	−0,20087689546011	0,00204068779614865	0,0055645367896895	1,14939676632432	0.200876895460114	0,870021588104793	-0.200876895460114	3076
Q9Z2A0	sp|Q9Z2A0|PDPK1_MOUSE 3-phosphoinositide-dependent protein kinase 1 OS=Mus musculus GN=Pdpk1 PE=1 SV=2	1,15	0,575412618374903	0,849998193752035	27,7767708216757	28,5025208578432	28,399855058049	28,5701797962947	28,2874146092639	28,2400659404567	1813685802931	2085201860342,77	−0,201262502483895	0,01391407689416	0,0062927651008047	1,14970402093515	0.201262502483895	0,869789077702464	-0.201262502483895	3077
Q9CQ79	sp|Q9CQ79|TXND9_MOUSE Thioredoxin domain-containing protein 9 OS=Mus musculus GN=Txndc9 PE=2 SV=1	1,15	0,504714329238943	0,814993646406912	26,0995196682378	26,5728087866134	26,7761234567643	26,7414467689503	26,5473065949047	26,5785183382483	317206019613,779	364730258933,549	−0,201409744920918	0,0131089835935029	0,0039159282446366	1,1498213664975	0.201409744920918	0,86970031096754	-0.201409744920918	3078
Q9CZJ0	sp|Q9CZJ0|MPPD2_MOUSE Metallophosphoesterase MPPED2 OS=Mus musculus GN=Mpped2 PE=2 SV=1	1,15	0,21361441610771	0,645012843958033	24,6644462690852	24,8622015383108	24,9975459496661	25,0758097375073	24,8783295174502	24,9892097138287	61444361979,1825	70657939870,6982	−0,201571048439063	0,0067436409384429	0,0039625358029800	1,14994993185278	0.201571048439062	0,869603077752107	-0.201571048439062	3079
Q8K1N4-2	sp|Q8K1N4-2|SPAS2_MOUSE Isoform 2 of Spermatogenesis-associated serine-rich protein 2 OS=Mus musculus GN=Spats2;sp|Q8K1N4|SPAS2_MOUSE Spermatogenesis-associated serine-rich protein 2 OS=Mus musculus GN=Spats2 PE=1 SV=1	1,15	0,271357804745738	0,691573877451378	21,6488223621637	21,9399864331884	22,0365622706126	21,968529841547	22,115881027351	21,9601516514935	3164065817,58955	3638561228,14669	−0,201588477494796	0,00922709537882409	0,0039787704449015	1,14996382436779	0.201588477494798	0,86959257222701	-0.201588477494798	3080
Q921W0	sp|Q921W0|CHM1A_MOUSE Charged multivesicular body protein 1a OS=Mus musculus GN=Chmp1a PE=1 SV=1	1,15	0,636142598005916	0,883588334123886	25,9776537154619	26,9150426236507	26,7284847671728	26,8090056281754	26,6885628808377	26,5444468376233	336005612989,221	386605945370,534	−0,202378490530235	0,0186957923625085	0,0049644854106761	1,15059371160843	0.202378490530233	0,869116517768975	-0.202378490530233	3081
Q8BGB7	sp|Q8BGB7|ENOPH_MOUSE Enolase-phosphatase E1 OS=Mus musculus GN=Enoph1 PE=2 SV=1;sp|Q8BGB7-2|ENOPH_MOUSE Isoform 2 of Enolase-phosphatase E1 OS=Mus musculus GN=Enoph1	1,15	0,210661665099566	0,641644645910089	28,8367004893606	28,8343162844237	29,151901871807	29,0513152204935	29,0359313240441	29,1565083052916	3705994952698,4	4264097279228,08	−0,202379434960209	0,00631194238758245	0,0022566615238848	1,15059446482066	0.202379434960208	0,869115948820306	-0.202379434960208	3082
Q8CHH9	sp|Q8CHH9|SEPT8_MOUSE Septin-8 OS=Mus musculus GN=Sept8 PE=1 SV=4;sp|Q8CHH9-2|SEPT8_MOUSE Isoform 2 of Septin-8 OS=Mus musculus GN=Sept8;sp|Q92599-2|SEPT8_HUMAN Isoform 2 of Septin-8 OS=Homo sapiens GN=SEPT8;sp|Q92599-4|SEPT8_HUMAN Isoform 4 of Septin-	1,15	0,0543956899112204	0,341924788314513	30,1667006838448	30,2457298105003	30,3560902036496	30,4798415039778	30,3961101691837	30,3135389013481	13806678854788,2	15886596117931,1	−0,202443717737244	0,00314401007695202	0,0027355775914182	1,15064573349018	0.202443717737238	0,869077224113773	-0.202443717737238	3083
A2RT62	sp|A2RT62|FXL16_MOUSE F-box/LRR-repeat protein 16 OS=Mus musculus GN=Fbxl16 PE=2 SV=1	1,15	0,23307736736626	0,657279264635888	30,2476801384455	29,8734429990539	30,0602349868501	30,2268071384523	30,2664339291469	30,1096724551239	11352427437299,8	13065171927906,9	−0,202725294231126	0,00622474539924794	0,0026989772335659	1,15087033148344	0.202725294231123	0,868907619428358	-0.202725294231123	3084
P70188-2	sp|P70188-2|KIFA3_MOUSE Isoform KAP3B of Kinesin-associated protein 3 OS=Mus musculus GN=Kifap3;sp|P70188|KIFA3_MOUSE Kinesin-associated protein 3 OS=Mus musculus GN=Kifap3 PE=1 SV=1	1,15	0,494616850792031	0,808741313308728	27,7938677088192	27,5648173464815	27,268187615531	27,6943403199442	27,436658796006	27,918409250953	915092408736,601	1053496894402,9	−0,203196717773658	0,00956939390195133	0,0087082107109917	1,15124645811168	0.203196717773661	0,868623736432803	-0.203196717773661	3085
Q62189	sp|Q62189|SNRPA_MOUSE U1 small nuclear ribonucleoprotein A OS=Mus musculus GN=Snrpa PE=2 SV=3	1,15	0,364571527471885	0,739315231400715	28,4734319680636	28,7281279757244	28,9967840041252	28,934830454227	28,8780890237702	28,808297486709	3009458613751,41	3465018155486,8	−0,203358934751029	0,00910830888239868	0,0021950238545845	1,15137591181808	0.203358934751032	0,868526073661687	-0.203358934751032	3086
Q91W61	sp|Q91W61|FXL15_MOUSE F-box/LRR-repeat protein 15 OS=Mus musculus GN=Fbxl15 PE=1 SV=2	1,15	0,564902828917323	0,845110794140252	25,7918978973668	26,2250524440376	26,5421786038889	26,5398052352229	26,1521497135295	26,2906517440577	235829828239,794	271583522972,994	−0,20364974875638	0,0143827715527747	0,0074614707514042	1,15160802600783	0.203649748756378	0,868351016505684	-0.203649748756378	3087
Q9CQE8	sp|Q9CQE8|CN166_MOUSE UPF0568 protein C14orf166 homolog OS=Mus musculus PE=2 SV=1	1,15	0,372410533870918	0,744143356508855	29,119788155716	29,4242425054228	29,5939809288885	29,6000841427096	29,3677682146643	29,5939809288885	5744920544019,5	6616655160472,99	−0,20381515312652	0,00817801178528838	0,0044850355169678	1,15174006494502	0.203815153126519	0,8682514661394	-0.203815153126519	3088
O88796	sp|O88796|RPP30_MOUSE Ribonuclease P protein subunit p30 OS=Mus musculus GN=Rpp30 PE=2 SV=1	1,15	0,602457623895533	0,863725570076382	23,709903168677	24,1834776243377	24,4117497112943	24,4689312575944	23,8956506099416	24,3651120990611	29325115441,523	33783262422,9593	−0,204171867195219	0,0148535775107733	0,0125996743011119	1,15202487404785	0.204171867195215	0,868036812856578	-0.204171867195215	3089

Q8VC88	sp|Q8VC88|GRAN_MOUSE Grancalcin OS=Mus musculus GN=Gca PE=2 SV=1	1,15	0,272399983795205	0,693189404859215	24,2127444651752	24,5625402578922	24,615571684966	24,5917632179099	24,6569155403246	24,567454870373	42112851935,5785	48526596993,7833	−0,204515112375134	0,0089469841934171	0,0018800040761386	1,15229899575588	0.204515112375133	0,86783031459992	-0.204515112375133	3090
Q3URQ0	sp|Q3URQ0|TEX10_MOUSE Testis-expressed sequence 10 protein OS=Mus musculus GN=Tex10 PE=1 SV=1	1,15	0,714031222004858	0,917934955801873	21,8345749480041	21,7873155101343	20,9771283438076	22,0605658022871	21,8106908372461	21,1542211809417	2247324573,75109	2590610174,42324	−0,205083437057997	0,0223834972351022	0,0215973398391513	1,15275301337499	0.205083437057996	0,867488515230364	-0.205083437057996	3091
Q9DBH5	sp|Q9DBH5|LMAN2_MOUSE Vesicular integral-membrane protein VIP36 OS=Mus musculus GN=Lman2 PE=2 SV=2	1,15	0,38679762093713	0,752468171504595	27,3015176496588	27,3269456084881	27,7242460573172	27,7767708216757	27,5000243142379	27,5035939037474	835171646343,254	963138455142,409	−0,205670472395828	0,0086358950779476	0,0057535095010482	1,15322216619715	0.205670472395828	0,867135604319491	-0.205670472395828	3092
P50396	sp|P50396|GDIA_MOUSE Rab GDP dissociation inhibitor alpha OS=Mus musculus GN=Gdi1 PE=1 SV=3	1,15	0,43675430272502	0,778450481049573	34,0257535658343	34,6005663418216	34,4663738267566	34,6244837935552	34,4527289384383	34,4432127689793	839838934555813	968537676882713	−0,20569549948241	0,0087507052982298	0,0029565414143153	1,15324217183973	0.205695499482409	0,867120561854523	-0.205695499482409	3093
Q3UUF8	sp|Q3UUF8|AN34B_MOUSE Ankyrin repeat domain-containing protein 34B OS=Mus musculus GN=Ankrd34b PE=1 SV=1	1,15	0,597072739902194	0,861464850775537	24,511216945845	24,9865539749528	25,4412526778006	25,1379645009733	25,0703734773173	25,1585755949886	70556139360,2669	81372606548,3589	−0,205771581506046	0,0186173774620609	0,0018365847262353	1,1533029908689	0.205771581506047	0,867074834555485	-0.205771581506047	3094
Q8CIV8	sp|Q8CIV8|TBCE_MOUSE Tubulin-specific chaperone E OS=Mus musculus GN=Tbce PE=1 SV=1	1,15	0,631076886898494	0,87947743003754	26,2503112648405	26,7284847671728	27,2501225437999	26,9938047890829	26,7551771799298	26,9078396230827	411458029774,56	474537839262,655	−0,20577785319059	0,0186988587859811	0,0044952427671349	1,15330800451909	0.205777853190592	0,867071065215559	-0.205777853190592	3095
Q9WUM3	sp|Q9WUM3|COR1B_MOUSE Coronin-1B OS=Mus musculus GN=Coro1b PE=1 SV=1	1,15	0,522461933743113	0,823948365557954	29,709391036643	30,4270212494486	30,2534697710926	30,3129183925351	30,348015515816	30,1584841687995	12169582123451,2	14042918440397,2	−0,206563161962928	0,0124267808112615	0,0033307773651259	1,15393596081956	0.206563161962927	0,866599216900884	-0.206563161962927	3096
Q80X85	sp|Q80X85|RT07_MOUSE 28S ribosomal protein S7, mitochondrial OS=Mus musculus GN=Mrps7 PE=2 SV=1	1,15	0,51013504291898	0,816791532413287	25,7670259217681	26,1671183698062	26,3911397781081	26,4478784720452	26,0393274349537	26,2677834736084	218145481442,896	251740121101,906	−0,206644573705016	0,0121098508739866	0,0077995825104363	1,15400107963182	0.206644573705016	0,866550315809967	-0.206644573705016	3097
Q7TQK5	sp|Q7TQK5|CCD93_MOUSE Coiled-coil domain-containing protein 93 OS=Mus musculus GN=Ccdc93 PE=2 SV=1	1,15	0,433808809674092	0,776748791759647	27,8568791263886	28,0252316570059	27,4194342735147	27,869672887315	27,9743749343137	27,8881308498074	1145865870171,96	1322739689311,6	−0,207090993372676	0,0112615275099361	0,0020024081200759	1,15435822267147	0.207090993372683	0,866282216698517	-0.207090993372683	3098
Q9CZP0	sp|Q9CZP0|UFSP1_MOUSE Ufm1-specific protease 1 OS=Mus musculus GN=Ufsp1 PE=1 SV=1	1,15	0,879094685739497	0,973716798448825	21,4835907437214	22,9507064919501	22,7884248163617	23,7216479337666	20,966955718834	22,9648263864877	5388721619,24627	6220669324,58792	−0,207126758998578	0,0358939513377867	0,0631088587051646	1,15438684053937	0.207126758998578	0,866260741098505	-0.207126758998578	3099
Q91XM9-6	sp|Q91XM9-6|DLG2_MOUSE Isoform 6 of Disks large homolog 2 OS=Mus musculus GN=Dlg2;sp|Q91XM9-3|DLG2_MOUSE Isoform 3 of Disks large homolog 2 OS=Mus musculus GN=Dlg2;sp|Q91XM9|DLG2_MOUSE Disks large homolog 2 OS=Mus musculus GN=Dlg2 PE=1 SV=2;sp|Q91XM9-2	1,15	0,0684088126858576	0,38813450097638	29,8391442128287	29,6207138314607	29,7364644042824	29,7814723531849	29,914375034854	29,9327417629286	8175046190069,26	9442070301649,3	−0,207876342629559	0,0036754996364336	0,0027629300111648	1,15498678320854	0.207876342629559	0,865810773368345	-0.207876342629559	3100
O70551	sp|O70551|SRPK1_MOUSE SRSF protein kinase 1 OS=Mus musculus GN=Srpk1 PE=1 SV=2	1,16	0,310610208102634	0,719371512809526	24,5429385120525	24,1261151702096	24,2030232322995	24,3111380538731	24,4925993676552	24,5010625962898	35425307572,9013	40921987174,3075	−0,208095825048724	0,0091314949470786	0,0043909815407860	1,1551625088955	0.208095825048724	0,865679064460065	-0.208095825048723	3101
Q8CHP5-2	sp|Q8CHP5-2|WIBG_MOUSE Isoform 2 of Partner of Y14 and mago OS=Mus musculus GN=Wibg;sp|Q8CHP5|WIBG_MOUSE Partner of Y14 and mago OS=Mus musculus GN=Wibg PE=2 SV=2	1,16	0,625935737415087	0,876102744788026	24,5294754056694	23,9322127236138	24,5294754056694	24,7545101478558	24,0418006571554	24,6282909811731	36859817757,0946	42589230881,7971	−0,208439738527892	0,014172802030889	0,0155391805990765	1,15543791242971	0.208439738527891	0,86547272617803	-0.208439738527891	3102
P08226	sp|P08226|APOE_MOUSE Apolipoprotein E OS=Mus musculus GN=Apoe PE=1 SV=2	1,16	0,538074109138386	0,83148516922726	30,1944393020597	30,8215053344581	30,8170158313743	30,9631543867588	30,7148429894456	30,5898070211484	19687141496179,2	22757930616749,9	−0,209115726864526	0,0117849098049499	0,0061788216164560	1,15597943059264	0.209115726864529	0,865067295779934	-0.209115726864529	3103
Q91V36	sp|Q91V36|NRBP2_MOUSE Nuclear receptor-binding protein 2 OS=Mus musculus GN=Nrbp2 PE=1 SV=2	1,16	0,507297893434294	0,816124555116335	27,072984791289	27,7863646768548	27,4741157907615	27,7251228271318	27,576491819139	27,4684064394126	829831429095,864	959815608577,453	−0,209938975454673	0,0130303610355829	0,0046716456059168	1,15663925819635	0.209938975454673	0,864573801134325	-0.209938975454673	3104
Q8VE11	sp|Q8VE11|MTMR6_MOUSE Myotubularin-related protein 6 OS=Mus musculus GN=Mtmr6 PE=1 SV=1	1,16	0,418114572830886	0,768519642229897	26,0031584198129	26,5591097026922	26,4349457329772	26,5728087866134	26,5038887636353	26,3577801409492	272909102412,932	315731886643,752	−0,210279455782323	0,0110820038994408	0,0041467789104482	1,15691226072052	0.210279455782324	0,864369783216929	-0.210279455782324	3105
Q78J03	sp|Q78J03|MSRB2_MOUSE Methionine-R-sulfoxide reductase B2, mitochondrial OS=Mus musculus GN=Msrb2 PE=1 SV=1	1,16	0,559366692020632	0,842973036472956	25,9331697089281	25,3346416265303	25,3412575352839	25,3979425844979	25,7318503388321	25,9168429770321	123111500552,799	142443599708,171	−0,210425261229678	0,0134579271187918	0,0102400609071441	1,15702918954416	0.210425261229683	0,864282430414716	-0.210425261229682	3106
Q9CWS0	sp|Q9CWS0|DDAH1_MOUSE N(G),N(G)-dimethylarginine dimethylaminohydrolase 1 OS=Mus musculus GN=Ddah1 PE=1 SV=3	1,16	0,353522599703111	0,73284362901203	31,1972620093183	31,5568375533821	31,7123495077502	31,6995393111186	31,5630720591258	31,6424407003006	47360816981850,8	54816773940403,1	−0,21092345771839	0,0083901184767363	0,0021664539999909	1,15742880789851	0.210923457718389	0,863984024914373	-0.210923457718389	3107
P06745	sp|P06745|G6PI_MOUSE Glucose-6-phosphate isomerase OS=Mus musculus GN=Gpi PE=1 SV=4	1,16	0,254623368046042	0,676705623584201	34,4595513825974	34,7975818162301	34,8145794845827	34,9429327686499	34,8145794845827	34,7531101143992	116392102557041	134729344924608	−0,211070941608207	0,0057724388188763	0,0027802521748459	1,15754713562787	0.211070941608208	0,863895706033246	-0.211070941608208	3108

Q6PEE2-2	sp|Q6PEE2-2|CTIF_MOUSE Isoform 2 of CBP80/20-dependent translation initiation factor OS=Mus musculus GN=Ctif;sp|Q6PEE2|CTIF_MOUSE CBP80/20-dependent translation initiation factor OS=Mus musculus GN=Ctif PE=1 SV=2	1,16	0,428255621314223	0,774134713319498	24,9238571579815	24,7267151368333	24,2991722917027	24,7764875068518	24,7970047172291	24,8152314567564	50736675170,2657	58731451850,8504	−0,211104320809706	0,0129552348271612	0,0007817013685521	1,15757391775782	0.211104320809706	0,863875718569199	-0.211104320809706	3109
P51410	sp|P51410|RL9_MOUSE 60S ribosomal protein L9 OS=Mus musculus GN=Rpl9 PE=1 SV=2	1,16	0,571769793527064	0,848402449434241	26,6674061630996	27,4078116367401	27,3198293289793	27,0553730818285	27,4118429955384	27,3672699965619	606931867022,228	702676199601,569	−0,211325462294431	0,0149078252250643	0,0071201193776101	1,15775136845111	0.21132546229443	0,863743310740239	-0.21132546229443	3110
Q8BYY4	sp|Q8BYY4|TT39B_MOUSE Tetratricopeptide repeat protein 39B OS=Mus musculus GN=Ttc39b PE=1 SV=1;sp|Q8BYY4-2|TT39B_MOUSE Isoform 2 of Tetratricopeptide repeat protein 39B OS=Mus musculus GN=Ttc39b	1,16	0,457545542080512	0,790957322506963	25,9217837339592	26,5351193087617	26,0052225567599	26,2939048107703	26,3810756525348	26,2266208709123	228326364695,616	264348402919,652	−0,211343154365237	0,0127188466497	0,0029444450247937	1,15776556628516	0.211343154365238	0,863732718540445	-0.211343154365238	3111
Q8BYK6	sp|Q8BYK6|YTHD3_MOUSE YTH domain-containing family protein 3 OS=Mus musculus GN=Ythdf3 PE=1 SV=2;sp|Q8BYK6-3|YTHD3_MOUSE Isoform 3 of YTH domain-containing family protein 3 OS=Mus musculus GN=Ythdf3;sp|Q7Z739|YTHD3_HUMAN YTH domain-containing family pro	1,16	0,48875949856844	0,806133489826362	27,2176411250769	27,8507513155764	27,8519734871773	27,763130170804	27,7887330476403	27,8080490843163	1009142398758,64	1168377831915,91	−0,211377125117445	0,0132372664669916	0,0008109172218876	1,15779282820061	0.211377125117446	0,863712380697813	-0.211377125117445	3112
Q8BRK8	sp|Q8BRK8|AAPK2_MOUSE 5-AMP-activated protein kinase catalytic subunit alpha-2 OS=Mus musculus GN=Prkaa2 PE=1 SV=3;sp|Q09137|AAPK2_RAT 5-AMP-activated protein kinase catalytic subunit alpha-2 OS=Rattus norvegicus GN=Prkaa2 PE=1 SV=1	1,16	0,377373226963685	0,74727992066734	25,5908651221833	26,0393274349537	26,0415130488842	26,126952961226	26,0912494688688	25,8942049257386	175440981463,993	203202553419,201	−0,211932742988422	0,0100250046497003	0,0048141446030676	1,15823880899176	0.211932742988421	0,863379807546332	-0.211932742988421	3113
P00493	sp|P00493|HPRT_MOUSE Hypoxanthine-guanine phosphoribosyltransferase OS=Mus musculus GN=Hprt1 PE=1 SV=3;sp|Q64531|HPRT_MUSSP Hypoxanthine-guanine phosphoribosyltransferase (Fragment) OS=Mus spretus GN=Hprt1 PE=1 SV=3;sp|P00494|HPRT_CRIGR Hypoxanthine-gua	1,16	0,416937109797292	0,767877733621866	30,9917314842313	31,4546926223212	31,4973921613793	31,5630720591258	31,3009254761233	31,5209983110802	39788753887297,8	46092219722445,5	−0,212162529965156	0,00895527536088902	0,0044748218491080	1,15842330355463	0.21216252996155	0,86324230264662	-0.212162529965155	3114
Q9CQM5	sp|Q9CQM5|TXD17_MOUSE Thioredoxin domain-containing protein 17 OS=Mus musculus GN=Txndc17 PE=1 SV=1	1,16	0,422775596983994	0,7711031271846	28,6735464306224	28,7693521714792	28,2685915515089	28,6169807290695	28,6169807290695	28,9188433011032	2558639320971,33	2964120824147,67	−0,212227464338905	0,00930363868975345	0,0060687671530479	1,15847544429295	0.212227464338907	0,863203449780784	-0.212227464338907	3115
P29416	sp|P29416|HEXA_MOUSE Beta-hexosaminidase subunit alpha OS=Mus musculus GN=Hexa PE=2 SV=2	1,16	0,549047086441073	0,838092765530185	25,8889079988918	26,377343207	26,6940235846991	26,3947233703869	26,6160829578104	26,3911397781081	269569370025,104	312326630185,89	−0,212399005628072	0,0154102679198966	0,0048682296897581	1,15861319910643	0.212399005628071	0,863100818091183	-0.212399005628071	3116
Q00558	sp|Q00558|F8I2_MOUSE Factor VIII intron 22 protein OS=Mus musculus GN=F8a1 PE=2 SV=1	1,16	0,681706519179619	0,904517472334246	27,4410098735373	27,0158477199576	26,4187355803847	27,3032177671707	26,6995442217379	27,3151166099119	510435978844,923	591518935741,393	−0,212694329739853	0,0190493207344309	0,0129866741182845	1,15885039506806	0.212694329739852	0,862924156781485	-0.212694329739852	3117
Q80UP5	sp|Q80UP5|AN13A_MOUSE Ankyrin repeat domain-containing protein 13A OS=Mus musculus GN=Ankrd13a PE=1 SV=2	1,16	0,875671902203089	0,972992598310849	20,5963942637238	23,5324035752081	22,2997639006898	22,9406662221807	21,4919382859881	22,4425016638024	4135416613,07787	4799137529,31895	−0,214742479362358	0,06658042298372	0,033018676168634	1,16049674756882	0.214742479362358	0,861699959172608	-0.214742479362358	3118
O70194	sp|O70194|EIF3D_MOUSE Eukaryotic translation initiation factor 3 subunit D OS=Mus musculus GN=Eif3d PE=1 SV=2	1,16	0,599217252079426	0,86182550787914	28,2371702788332	28,8887694965435	28,8887694965435	29,1488133739494	28,7923191655361	28,520313058957	2830769665758,99	3285309127401,65	−0,214834760952915	0,013121045932708	0,0109365000389319	1,16057098079747	0.214834760952912	0,86164484253506	-0.214834760952913	3119
A6X8Z5	sp|A6X8Z5|RHG31_MOUSE Rho GTPase-activating protein 31 OS=Mus musculus GN=Arhgap31 PE=1 SV=1	1,16	0,204545507438659	0,635648869851434	25,4119626398757	25,2008916720812	25,0158762888834	25,3545598909119	25,3144985329333	25,4069322364685	88793285198,0872	103068902046,776	−0,215086623263343	0,0078615344762448	0,0018279074181715	1,16077360823897	0.215086623263336	0,86149443173257	-0.215086623263337	3120
Q9JIZ9	sp|Q9JIZ9|PLS3_MOUSE Phospholipid scramblase 3 OS=Mus musculus GN=Plscr3 PE=1 SV=1	1,16	0,37916788707724	0,748785300113128	24,2355146437319	24,6912518557112	24,7487693428392	24,8070307785967	24,656098949068	24,6602962520587	46304822367,821	53760705698,5247	−0,215389626716519	0,011450137742521	0,0034788394461171	1,16101742646755	0.215389626716518	0,861313514511617	-0.215389626716518	3121
Q8R1G2	sp|Q8R1G2|CMBL_MOUSE Carboxymethylenebutenolidase homolog OS=Mus musculus GN=Cmbl PE=2 SV=1	1,16	0,686977126735201	0,907675629195552	26,7131195606615	27,6659486438165	27,7842060823852	27,7483704429287	27,2462324621921	27,6170252356209	784065261562,296	910454152137,603	−0,215612627184666	0,021441642285624	0,0094566728379814	1,16119690129297	0.215612627184666	0,861180389722463	-0.215612627184666	3122
Q91YR5	sp|Q91YR5|MET13_MOUSE Methyltransferase-like protein 13 OS=Mus musculus GN=Mettl13 PE=1 SV=1;sp|Q91YR5-1|MET13_MOUSE Isoform 2 of Methyltransferase-like protein 13 OS=Mus musculus GN=Mettl13;sp|Q91YR5-2|MET13_MOUSE Isoform 3 of Methyltransferase-like pr	1,16	0,637141356595612	0,883588334123886	24,6683213514674	24,0418006571554	23,5871603405659	24,2039362195013	24,34213713151	24,1996408868827	29248499682,3093	33964150649,2803	−0,215650154003871	0,0225258601466111	0,0033428256243979	1,1612271062855	0.215650154003872	0,861157989326286	-0.215650154003872	3123
Q8R086	sp|Q8R086|SUOX_MOUSE Sulfite oxidase, mitochondrial OS=Mus musculus GN=Suox PE=2 SV=2	1,16	0,416273375155588	0,767877733621866	26,4033494281023	26,9425529647263	26,960577018864	26,9340296968593	26,8725182103567	26,9494249747504	422234409943,702	490483574102,171	−0,216160666825312	0,0118287096715044	0,0015116877014223	1,16163809142786	0.21616066825312	0,860853313419519	-0.216160666825312	3124

P63137-2	sp|P63137-2|GBRB2_MOUSE Isoform 2 of Gamma-aminobutyric acid receptor subunit beta-2 OS=Mus musculus GN=Gabrb2;sp|P63137|GBRB2_MOUSE Gamma-aminobutyric acid receptor subunit beta-2 OS=Mus musculus GN=Gabrb2 PE=1 SV=2	1,16	0,409900192173669	0,763224927792945	26,8725182103567	26,886722967848	27,210872020632	27,355199209797	27,1995228618436	26,8651963070805	526774148519,619	611984500346,637	−0,21631056746181	0,00709076215819238	0,0092251553022634	1,1617587956176	0.216310567461808	0,860763872649138	-0.216310567461808	3125
O35350	sp|O35350|CAN1_MOUSE Calpain-1 catalytic subunit OS=Mus musculus GN=Capn1 PE=1 SV=1	1,16	0,257619561517676	0,67968190098123	26,7811512810363	26,7099050263889	26,3722038170175	26,8191926913355	26,6900416088642	26,8042111906981	364242870286,11	423215988576,023	−0,21649339855521	0,0082063845255796	0,0026386211467574	1,16190603331121	0.216493398555215	0,860654795939214	-0.216493398555214	3126
Q9DCL8	sp|Q9DCL8|IPP2_MOUSE Protein phosphatase inhibitor 2 OS=Mus musculus GN=Ppp1r2 PE=1 SV=3	1,16	0,539164174261446	0,831485169227261	25,7281474875069	26,1234980759642	26,238233207829	26,3833279274518	25,8014926834204	26,3554364395044	201682313405,344	234350966026,378	−0,21658616991595	0,010280403221341	0,0125350089128026	1,16198075115975	0.216586169915951	0,860599453994327	-0.216586169915951	3127
Q923E4	sp|Q923E4|SIR1_MOUSE NAD-dependent protein deacetylase sirtuin-1 OS=Mus musculus GN=Sirt1 PE=1 SV=2	1,16	0,340975673812318	0,72969758766342	22,2366110073191	21,9501523629703	21,7145801016844	22,0562287492798	22,0882083383777	22,2077631703379	3468938549,72064	4031482801,29134	−0,21681628311479	0,0119009023022674	0,0036113458059389	1,16216610456129	0.216816283114801	0,860462197335802	-0.216816283114801	3128
Q7TMW6	sp|Q7TMW6|NARFL_MOUSE Cytosolic Fe-S cluster assembly factor NARFL OS=Mus musculus GN=Narfl PE=2 SV=2;sp|Q7TMW6-2|NARFL_MOUSE Isoform 2 of Cytosolic Fe-S cluster assembly factor NARFL OS=Mus musculus GN=Narfl	1,16	0,683770035229217	0,905376951547671	21,602992944783	22,2626581889544	22,6095143587135	22,4379044725108	21,8769368165872	22,6114053377269	4200160171,09526	4881648834,21643	−0,21692417186663	0,0230746845515709	0,017208067249483	1,162253017828	0.21692417186663	0,860397851983026	-0.21692417186663	3129
P16054	sp|P16054|KPCE_MOUSE Protein kinase C epsilon type OS=Mus musculus GN=Prkce PE=1 SV=1	1,16	0,342112427492716	0,729721949431128	31,143072998167	30,871713126719	30,5960927494958	30,9917314842313	31,0545441670067	31,0176093622394	25515135936187,6	29674078548230,4	−0,21784990345522	0,00885941996616615	0,0010176274316699	1,1629990380002	0.217849903455223	0,85984593909856	-0.217849903455223	3130
Q9ES97-2	sp|Q9ES97-2|RTN3_MOUSE Isoform 2 of Reticulon-3 OS=Mus musculus GN=Rtn3;sp|Q9ES97|RTN3_MOUSE Reticulon-3 OS=Mus musculus GN=Rtn3 PE=1 SV=2;sp|Q9ES97-5|RTN3_MOUSE Isoform 5 of Reticulon-3 OS=Mus musculus GN=Rtn3	1,16	0,195785345835732	0,623627848946236	30,4957910271004	30,688685623596	30,6703686802763	30,8043490382255	30,9222771011418	30,5824842469731	19831283409992,2	23073444008206,4	−0,21845531420936	0,0034774883268309	0,0056069911755342	1,16348717988573	0.218455314209368	0,859485190114615	-0.218455314209368	3131
Q9QYE6	sp|Q9QYE6|GOGA5_MOUSE Golgin subfamily A member 5 OS=Mus musculus GN=Golga5 PE=1 SV=2	1,16	0,489437654346998	0,806156092714947	24,6808051491555	24,6103867125833	24,1996408868827	24,9531426386628	24,4117497112943	24,5806585090775	43539928454,5952	50665799457,6939	−0,21867318763177	0,0106082021338289	0,0112380829853276	1,16366290106631	0.218673187631772	0,859355401881128	-0.218673187631772	3132
Q9WUP7-2	sp|Q9WUP7-2|UCHL5_MOUSE Isoform 2 of Ubiquitin carboxyl-terminal hydrolase isozyme L5 OS=Mus musculus GN=Uchl5;sp|Q9WUP7|UCHL5_MOUSE Ubiquitin carboxyl-terminal hydrolase isozyme L5 OS=Mus musculus GN=Uchl5 PE=1 SV=2	1,16	0,597474375932986	0,861464850775537	28,3022437834415	27,4879183321299	27,7318880781206	28,1645775605123	28,130530652555	27,6817373259402	1233261494975,88	1435137596112,12	−0,21871032976865	0,0150111977481086	0,0096268566385109	1,16369285991548	0.218710329768656	0,859333278089059	-0.218710329768656	3133
P63141	sp|P63141|KCNA2_MOUSE Potassium voltage-gated channel subfamily A member 2 OS=Mus musculus GN=Kcna2 PE=1 SV=1	1,16	0,279098274117861	0,699221280539289	27,5185848493879	27,9434810304077	27,8140234660062	27,9932539266564	27,9495817820908	27,7887330476403	1136184060965,51	1322470797032,13	−0,21903929562634	0,00784562409306432	0,0038591095494692	1,1639582374605	0.219039295626347	0,859137353743703	-0.219039295626347	3134
Q3TC72	sp|Q3TC72|FAHD2_MOUSE Fumarylacetoacetate hydrolase domain-containing protein 2A OS=Mus musculus GN=Fahd2 PE=1 SV=1	1,16	0,398524727418565	0,754338506152154	28,8042521530299	29,0919148705394	29,3723329959481	29,3608168934757	29,104176927775	29,2598728637706	4299414671716,16	5005819383786,42	−0,21946597504986	0,00976462711045623	0,0044214278263883	1,16430253092761	0.219465975049864	0,858883300033104	-0.219465975049864	3135
Q8R1Y2	sp|Q8R1Y2|CP045_MOUSE Uncharacterized protein C16orf45 homolog OS=Mus musculus PE=2 SV=1	1,16	0,529045635097097	0,829460904757119	23,3553531113101	24,040363892071	24,0841927204206	23,9952333945482	23,8813417836964	24,0597088731534	22272900998,6241	25932461236,018	−0,21946965973741	0,0171543873326554	0,0037665508237076	1,16430550459592	0.21946965973742	0,858881106421512	-0.21946965973742	3136
O88487	sp|O88487|DC1I2_MOUSE Cytoplasmic dynein 1 intermediate chain 2 OS=Mus musculus GN=Dync1i2 PE=1 SV=1	1,16	0,527368952828035	0,828347679607515	30,161244964118	29,9691108569161	29,3648762637523	30,0077005409481	29,9407135560488	30,0036031524467	9031540151817,98	10516912060874,9	−0,21966723060093	0,0139306477469803	0,0012522700231325	1,1644649621314	0.219667230600937	0,858763494411747	-0.219667230600937	3137
Q8VDS8-3	sp|Q8VDS8-3|STX18_MOUSE Isoform 3 of Syntaxin-18 OS=Mus musculus GN=Stx18;sp|Q8VDS8|STX18_MOUSE Syntaxin-18 OS=Mus musculus GN=Stx18 PE=2 SV=2;sp|Q8VDS8-2|STX18_MOUSE Isoform 2 of Syntaxin-18 OS=Mus musculus GN=Stx18	1,16	0,344896672746379	0,730213633407042	24,656098949068	24,3111380538731	24,5401576917216	24,8485211097813	24,6958846864592	24,4199170205648	43780961821,2564	50983825497,8735	−0,21973597861926	0,00716426706882995	0,0088112469940881	1,16452045311439	0.219735978619261	0,858722573163571	-0.219735978619261	3138
Q91Z31	sp|Q91Z31|PTBP2_MOUSE Polypyrimidine tract-binding protein 2 OS=Mus musculus GN=Ptbp2 PE=1 SV=2;sp|Q91Z31-2|PTBP2_MOUSE Isoform 2 of Polypyrimidine tract-binding protein 2 OS=Mus musculus GN=Ptbp2	1,16	0,242524552968679	0,666355294143363	29,4260099986843	29,6878677659393	29,8475138120678	29,7730411693604	29,8931980979023	29,7525593270964	7559280973190,68	8804342645379,09	−0,21996627868568	0,00717639467431037	0,0025490803831882	1,16470636249718	0.219966278685685	0,858585504638228	-0.219966278685685	3139

Q5SXY1-2	sp|Q5SXY1-2|CYTSB_MOUSE Isoform 2 of Cytospin-B OS=Mus musculus GN=Specc1;sp|Q5SXY1|CYTSB_MOUSE Cytospin-B OS=Mus musculus GN=Specc1 PE=1 SV=2;sp|Q5SXY1-3|CYTSB_MOUSE Isoform 4 of Cytospin-B OS=Mus musculus GN=Specc1	1,17	1,17	0,403470933032687	0,758324871650256	27,2162114865181	26,9195750813523	26,6674061630996	27,0605273992974	27,2584776827915	26,9435946391298	498264838579,619	580719239537,926	−0,220928062193882	0,0101989565064209	0,0058761036660235	1,16548308163457	0.220928062193883	0,858013312898131	-0.220928062193883	3140
P34914	sp|P34914|HYES_MOUSE Bifunctional epoxide hydrolase 2 OS=Mus musculus GN=Ephx2 PE=1 SV=2;sp|P34914-2|HYES_MOUSE Isoform 2 of Bifunctional epoxide hydrolase 2 OS=Mus musculus GN=Ephx2	1,17	1,17	0,490977115419936	0,807151440380692	28,6392790424527	29,3536338319982	29,209851847461	29,2839283038169	29,1293211094485	29,2505827592898	4206231535855,77	4905005797251,73	−0,221726574852853	0,0129982983146183	0,00278437768580519	1,16612833968822	0.221726574852853	0,857538545257687	-0.221726574852853	3141
Q01065	sp|Q01065|PDE1B_MOUSE Calcium/calmodulin-dependent 3,5-cyclic nucleotide phosphodiesterase 1B OS=Mus musculus GN=Pde1b PE=1 SV=2	1,17	1,17	0,245237149226687	0,669351425339707	29,9085130255149	30,2434645802855	30,279068162713	30,392909761289	30,1871752749852	30,312297883722	12337714700889,3	14388652245414,8	−0,22185627354072	0,006782143351341	0,0034216276390039	1,16623317966476	0.221856273540717	0,857461455767751	-0.221856273540717	3142
Q9Z2U0	sp|Q9Z2U0|PSA7_MOUSE Proteasome subunit alpha type-7 OS=Mus musculus GN=Psma7 PE=1 SV=1	1,17	1,17	0,399917718674482	0,755175958024291	31,1921742262174	31,471637043656	31,6726545295256	31,7073332672964	31,3626131085233	31,7285609258969	45352598960976,5	52904125255903,6	−0,222194994692831	0,00767375797613106	0,0065009425743192	1,16650702424848	0.222194994692831	0,857260161501594	-0.222194994692831	3143
Q4VAA2-2	sp|Q4VAA2-2|CDV3_MOUSE Isoform 2 of Protein CDV3 OS=Mus musculus GN=Cdv3;sp|Q4VAA2|CDV3_MOUSE Protein CDV3 OS=Mus musculus GN=Cdv3 PE=1 SV=2	1,17	1,17	0,581449156317854	0,853379506269256	27,1760569863579	27,9171142157975	27,4832478791861	28,0364216970831	27,5285613999933	27,4741157907615	899831199990,46	1049882773184,76	−0,222501954117313	0,0135266965577199	0,0112041491836205	1,16675524609048	0.222501954117315	0,857077783323249	-0.222501954117315	3144
Q9CZP7-2	sp|Q9CZP7-2|CD37L_MOUSE Isoform 2 of Hsp90 co-chaperone Cdc37-like 1 OS=Mus musculus GN=Cdc37l1;sp|Q9CZP7-3|CD37L_MOUSE Isoform 3 of Hsp90 co-chaperone Cdc37-like 1 OS=Mus musculus GN=Cdc37l1;sp|Q9CZP7-4|CD37L_MOUSE Isoform 4 of Hsp90 co-chaperone Cdc37	1,17	1,17	0,368000880832153	0,739685989256097	24,1280009506016	24,2840779865289	24,6282909811731	24,4697558524359	24,3684748551525	24,6655484483854	37469382612,4902	43728229683,052	−0,222852736363001	0,0105136199260825	0,0061638335897657	1,16703896979812	0.222852736362999	0,856869415571434	-0.222852736362999	3145
Q99LD4	sp|Q99LD4|CSN1_MOUSE COP9 signalosome complex subunit 1 OS=Mus musculus GN=Gps1 PE=1 SV=1;sp|Q99LD4-2|CSN1_MOUSE Isoform 2 of COP9 signalosome complex subunit 1 OS=Mus musculus GN=Gps1	1,17	1,17	0,399745530567441	0,755175958024291	29,5072210768158	30,0424084078288	30,0254239818711	30,1173421092281	29,9807891911943	29,9407135560488	9275068923552,93	10825745821355,2	−0,22303651276523	0,0101883095932498	0,0030853902305886	1,16718764147019	0.223036512765236	0,856760270988133	-0.223036512765236	3146
Q9WVG6-2	sp|Q9WVG6-2|CARM1_MOUSE Isoform 2 of Histone-arginine methyltransferase CARM1 OS=Mus musculus GN=Carm1;sp|Q9WVG6|CARM1_MOUSE Histone-arginine methyltransferase CARM1 OS=Mus musculus GN=Carm1 PE=1 SV=2	1,17	1,17	0,630158022315184	0,878466604204121	26,9612195741421	27,6904097335274	27,8785137992401	27,9018726638348	27,3661184166555	27,7259995969464	886057516336,826	1034214750110,7	−0,223063529909238	0,0176123010806825	0,0098715426542171	1,16720949943113	0.223063529909239	0,85674422671112	-0.223063529909239	3147
Q9ER72	sp|Q9ER72|SYCC_MOUSE Cysteine--tRNA ligase, cytoplasmic OS=Mus musculus GN=Cars PE=2 SV=2;sp|Q9ER72-2|SYCC_MOUSE Isoform 2 of Cysteine--tRNA ligase, cytoplasmic OS=Mus musculus GN=Cars	1,17	1,17	0,191546113478852	0,618802084535046	29,8531115187151	29,6347178699823	29,4808389247203	29,9023714560052	29,7364644042824	29,7939275700076	7577638860936,47	8845421915602,32	−0,22318257453999	0,00630780514817838	0,0028261078735322	1,16730581622218	0.223182574539991	0,856673534992195	-0.223182574539991	3148
Q61423	sp|Q61423|KCNA4_MOUSE Potassium voltage-gated channel subfamily A member 4 OS=Mus musculus GN=Kcna4 PE=1 SV=2	1,17	1,17	0,599008254347482	0,86182550787914	23,8277112897959	23,3453837113846	23,3986660676142	24,18201000329	23,4130659507513	23,4411557402849	16455413633,2695	19210904512,2697	−0,223363156030993	0,011241157754753	0,0184159882822643	1,1674519365122	0.223363156030998	0,856566312260815	-0.223363156030998	3149
Q80TA6	sp|Q80TA6|MTMRC_MOUSE Myotubularin-related protein 12 OS=Mus musculus GN=Mtmr12 PE=2 SV=2	1,17	1,17	0,398009733565107	0,754338506152154	26,3040142907628	25,9004504146625	25,7355004334847	26,2484016717649	26,0039735970023	26,1521497135295	191851674089,891	223984269472,326	−0,223406060749978	0,0112580383101464	0,00471132753730401	1,16748665621432	0.223406060749974	0,856540838969921	-0.223406060749975	3150
Q8C1M2	sp|Q8C1M2|ZN428_MOUSE Zinc finger protein 428 OS=Mus musculus GN=Znf428 PE=2 SV=1	1,17	1,17	0,387092571152948	0,752468171504595	24,0448449514442	24,2820927735618	24,4657851778578	24,6781682335195	24,2429818835158	24,3378985272248	34500545333,3062	40302642780,1457	−0,224255278183731	0,00869745574880586	0,00937073131195109	1,16817407930182	0.224255278183729	0,856036799410638	-0.224255278183729	3151
Q8VC30	sp|Q8VC30|DHAK_MOUSE Bifunctional ATP-dependent dihydroxyacetone kinase/FAD-AMP lyase (cyclizing) OS=Mus musculus GN=Dak PE=2 SV=1	1,17	1,17	0,505090963548778	0,814993646406912	26,660624355741	27,4089401757686	27,1570050725175	27,2429325656057	27,3601226677854	27,0900402227433	573786493393,941	670327220363,995	−0,224351511093968	0,0140626647625272	0,0049735148774043	1,16825200328264	0.224351511093967	0,855979700604085	-0.224351511093967	3152
O09167	sp|O09167|RL21_MOUSE 60S ribosomal protein L21 OS=Mus musculus GN=Rpl21 PE=2 SV=3	1,17	1,17	0,676286522039677	0,902046583466969	25,0954650688965	25,6958788171413	24,5072217421758	25,2882777697561	24,9892097138287	25,4876194191801	79539681788,5593	92922870287,9682	−0,224358927721688	0,0236792925592541	0,0099331552504503	1,16825800906503	0.224358927721687	0,855975300182459	-0.224358927721687	3153
P97950	sp|P97950|RB33A_MOUSE Ras-related protein Rab-33A OS=Mus musculus GN=Rab33a PE=2 SV=1	1,17	1,17	0,552805706656529	0,838891344884342	25,5829712431895	26,1971184130789	26,4478784720452	26,133625476344	26,2484016717649	26,3128537120198	211182679633,506	246746406881,339	−0,224537560905861	0,0170653057639529	0,0034608601970083	1,16840267066197	0.224537560905861	0,85586932066275	-0.224537560905861	3154

Q9JMJ4	sp|Q9JMJ4|PRP19_RAT Pre-mRNA-processing factor 19 OS=Rattus norvegicus GN=Prpf19 PE=1 SV=2;sp|Q99KP6|PRP19_MOUSE Pre-mRNA-processing factor 19 OS=Mus musculus GN=Prpf19 PE=1 SV=1;sp|Q99KP6-2|PRP19_MOUSE Isoform 2 of Pre-mRNA-processing factor 19 OS=Mus	1,17	0,53222971367587	0,829883431939119	28,8657160830252	29,6309744420806	29,4440052105859	29,6629732680634	29,3758840404762	29,3699426896834	5379224018557,54	6287596501625,41	−0,225110566057589	0,013609950512724	0,0056835691473281	1,16886682538859	0.22511056605759	0,85552945663211	-0.22511056605759	3155
P50571	sp|P50571|GBRB1_MOUSE Gamma-aminobutyric acid receptor subunit beta-1 OS=Mus musculus GN=Gabrb1 PE=1 SV=1	1,17	0,707352192492049	0,916805961322165	24,5138570468075	23,2726265887226	24,3237320068627	24,2505125478808	23,8988976203711	24,4296234518414	27480391404,9259	32128560499,7632	−0,225453790502703	0,0278129919956887	0,0111600396092691	1,16914493779751	0.225453790502703	0,85532594605751	-0.225453790502703	3156
Q9CPU4	sp|Q9CPU4|MGST3_MOUSE Microsomal glutathione S-transferase 3 OS=Mus musculus GN=Mgst3 PE=1 SV=1	1,17	5,09×10^−05	0,002180300982045	27,5668665706527	27,5020965064079	27,4925739899774	27,6192585353685	27,7242460573172	27,6870999533187	895378486416,443	1046914289999,62	−0,225573775249074	0,00146891507510078	0,0019234971350733	1,16924217622166	0.225573775249074	0,855254814046686	-0.225573775249074	3157
Q91X52	sp|Q91X52|DCXR_MOUSE L-xylulose reductase OS=Mus musculus GN=Dcxr PE=2 SV=2	1,17	0,544538399048812	0,834738194286578	24,7157174558305	25,4023621789469	25,5170328077361	25,4374912672445	25,2415366291153	25,4252755505049	88982374479,0141	104046229209,849	−0,225633178402443	0,0171883179159644	0,0043274084879998	1,16929032090942	0.22563317840244	0,855219599544998	-0.22563317840244	3158
Q8K386	sp|Q8K386|RAB15_MOUSE Ras-related protein Rab-15 OS=Mus musculus GN=Rab15 PE=1 SV=1	1,17	0,441390958772426	0,780917496263522	26,2799304014618	26,6173376487642	26,8968700970283	26,8093381012677	26,5652571906599	26,8888307780833	355946419370,475	416218149114,821	−0,225679786303338	0,0116144603138952	0,0063024640331494	1,16932809677069	0.225679786303337	0,855191971151361	-0.225679786303337	3159
P35293	sp|P35293|RAB18_MOUSE Ras-related protein Rab-18 OS=Mus musculus GN=Rab18 PE=2 SV=2	1,17	0,449212617817153	0,786343703431612	29,2802995314596	29,5120517470896	29,3586082207491	29,2720468450267	29,4282378281537	29,9201519045597	5769769031431,75	6747178449306,48	−0,22577075095964	0,0040120452551526	0,0114499926033876	1,16940182744753	0.225770750959643	0,85513805137684	-0.225770750959643	3160
Q64442	sp|Q64442|DHSO_MOUSE Sorbitol dehydrogenase OS=Mus musculus GN=Sord PE=1 SV=3	1,17	0,317599203137696	0,722907157640623	28,0588742128856	27,5436012651194	27,6547108054017	27,8961549127051	27,9088339896986	27,9221362330221	1129047449148,32	1320513394064,51	−0,22599281710968	0,00977105494028702	0,00046550884101881	1,16958184092318	0.225992817109679	0,855006434787562	-0.225992817109679	3161
P55821	sp|P55821|STMN2_MOUSE Stathmin-2 OS=Mus musculus GN=Stmn2 PE=1 SV=1	1,17	0,65836147368092	0,893187009962568	28,5471743747981	28,0831095688452	27,964154597457	27,745330412391	28,6000440221418	28,7194223068834	1763190698195,5	2062484102277,25	−0,22619448101244	0,0109249535693258	0,018737278440015	1,16974533973441	0.22619448101244	0,854886927976176	-0.22619448101244	3162
Q8VDS4	sp|Q8VDS4|RPR1A_MOUSE Regulation of nuclear pre-mRNA domain-containing protein 1A OS=Mus musculus GN=Rprd1a PE=2 SV=1	1,17	0,331493857906196	0,72953198881961	26,4722972144417	26,4269514236666	26,8725182103567	26,8676217628967	26,7734005079762	26,6015089080294	353301963709,322	413329276937,128	−0,226389788316475	0,0092216221109926	0,0050446691368289	1,16990370672605	0.226389788316473	0,854771204032235	-0.226389788316473	3163
P50427	sp|P50427|STS_MOUSE Steryl-sulfatase OS=Mus musculus GN=Sts PE=2 SV=1	1,17	0,486015237856669	0,804557984450102	21,9541395813542	21,602992944783	21,7767731508675	22,026190417643	22,2295117621056	21,5496008959617	2871121505,92167	3359644578,89892	−0,226694229800197	0,00806210964665446	0,0159100267254557	1,17015060908069	0.226694229800198	0,854590846887334	-0.226694229800199	3164
P54726	sp|P54726|RD23A_MOUSE UV excision repair protein RAD23 homolog A OS=Mus musculus GN=Rad23a PE=1 SV=2	1,17	0,352596118757787	0,732843629012038	25,1215085600284	25,3227647390092	25,3293144456964	25,3025335055744	25,7219669464862	25,2215409768175	93185930000,164	109079972515,726	−0,227202195721481	0,00467701682358314	0,0105688397850614	1,17056268597131	0.227202195721483	0,854290002564214	-0.227202195721483	3165
Q62073	sp|Q62073|M3K7_MOUSE Mitogen-activated protein kinase kinase kinase 7 OS=Mus musculus GN=Map3k7 PE=1 SV=1	1,17	0,664318967306612	0,896648481102019	24,3880879517679	25,4719455493022	25,3944926619741	25,4354333008474	25,0898230655906	25,2026579862367	78380581944,6733	91777969082,0603	−0,227651597865094	0,0241041262983493	0,0069818240319183	1,17092737518642	0.227651597865094	0,85402393110913	-0.227651597865095	3166
P47757-2	sp|P47757-2|CAPZB_MOUSE Isoform 2 of F-actin-capping protein subunit beta OS=Mus musculus GN=Capzb;sp|P47757-4|CAPZB_MOUSE Isoform 3 of F-actin-capping protein subunit beta OS=Mus musculus GN=Capzb	1,17	0,292510615315011	0,711969538636193	31,7691737448374	32,1255304366215	32,26957919978	32,209626068356	32,1814288440177	32,2466587951576	83407648279659,8	97665670429384,6	−0,22767186131614	0,00803623958701559	0,0010155834887497	1,17094382162555	0.22767186131614	0,854011935954161	-0.22767186131614	3167
Q9D483	sp|Q9D483|RPC3_MOUSE DNA-directed RNA polymerase III subunit RPC3 OS=Mus musculus GN=Polr3c PE=2 SV=1	1,17	0,177175397469093	0,595262123695199	22,1958548520544	22,4867335334843	22,1585650899638	22,3388119487222	22,524642240339	22,451234435472	4745129174,06697	5556473834,06479	−0,227722270397768	0,00806420232705086	0,0041712401688451	1,17098473618631	0.227722270397768	0,853982096518884	-0.227722270397768	3168
Q3UMT1	sp|Q3UMT1|PP12C_MOUSE Protein phosphatase 1 regulatory subunit 12C OS=Mus musculus GN=Ppp1r12c PE=1 SV=1	1,17	0,31116959111837	0,719371512809526	27,4854015248497	27,4054318063668	27,1109039589626	27,3661184166555	27,7048655608525	27,4054318063668	742963258366,343	870330267717,945	−0,228272102957166	0,00721545823646506	0,0067391140173950	1,171431100956	0.228272102957165	0,853656693239492	-0.228272102957165	3169
Q9CQV8	sp|Q9CQV8|1433B_MOUSE 14-3-3 protein beta/alpha OS=Mus musculus GN=Ywhab PE=1 SV=3	1,17	0,366793903605434	0,739315231400715	32,8262003127847	33,268184717111	33,3161706276563	33,4446452460359	33,1690884811052	33,2718440784801	246122842222432	288348980939385	−0,22843736577731	0,00815104318550918	0,0041824132911287	1,17156529778244	0.228437365777308	0,853558911221436	-0.228437365777308	3170
Q61739-2	sp|Q61739-2|ITA6_MOUSE Isoform Alpha-6X1A of Integrin alpha-6 OS=Mus musculus GN=Itga6;sp|Q61739|ITA6_MOUSE Integrin alpha-6 OS=Mus musculus GN=Itga6 PE=1 SV=3	1,17	0,38233409584986	0,749797813889279	22,4867335334843	22,8906559375229	22,9902222541556	23,114783803093	22,7826561503887	22,9454843610105	7892698340,94757	9247706743,98164	−0,228577038499181	0,0117000893834247	0,0072370981428065	1,1716787269069	0.228577038499182	0,853476278979553	-0.228577038499182	3171
Q9CXF4	sp|Q9CXF4|TBC15_MOUSE TBC1 domain family member 15 OS=Mus musculus GN=Tbc1d15 PE=1 SV=1	1,17	0,298901379595315	0,71351128730767	26,9015107380824	26,5143677630458	26,4862939995624	26,9395921810098	26,7117428695641	26,7270880304719	368998162105,351	432482497236,416	−0,229028116832929	0,00871238808147732	0,0047531574308569	1,17204512556065	0.229028116832927	0,853209469662396	-0.229028116832927	3172
Q91YQ5	sp|Q91YQ5|RPN1_MOUSE Dolichyl-diphosphooligosaccharide--protein glycosyltransferase subunit 1 OS=Mus musculus GN=Rpn1 PE=1 SV=1	1,17	0,508273535656174	0,816124555116335	28,6599523335218	29,2120692784127	29,2944931553283	29,104176927775	29,0578855856183	29,4808389247203	4155712137379,14	4870903110043,1	−0,229093562527377	0,0118748138259756	0,0079408573529585	1,17209829483401	0.229093562527376	0,853170765973698	-0.229093562527376	3173
Q6GV12	sp|Q6GV12|KDSR_MOUSE 3-ketodihydrosphingosine reductase OS=Mus musculus GN=Kdsr PE=2 SV=1	1,17	0,378822037707966	0,748785300113128	25,3363157103993	25,6922263661063	25,9276015798895	25,9004504146625	25,8243737283388	25,7084262444795	138211075402,744	162035852997,815	−0,229439838304051	0,0116046687778938	0,0037464333113313	1,17237965572329	0.229439838304051	0,852966012433111	-0.229439838304051	3174

Q8BG51-3	sp|Q8BG51-3|MIRO1_MOUSE Isoform 3 of Mitochondrial Rho GTPase 1 OS=Mus musculus GN=Rhot1;sp|Q8BG51-4|MIRO1_MOUSE Isoform 4 of Mitochondrial Rho GTPase 1 OS=Mus musculus GN=Rhot1;sp|Q8BG51|MIRO1_MOUSE Mitochondrial Rho GTPase 1 OS=Mus musculus GN=Rhot1 P	1,17	0,132116196518739	0,516676186281332	27,7242460573172	27,5334727094064	27,4002419470032	27,7954933381178	27,6263820326853	27,7146979344159	924624523252,212	1084555239900,61	−0,23016400408429	0,0059105541396927	0,0030522133008079	1,17296828347778	0.23016400408429	0,852537970621898	-0.23016400408429	3175
Q9CQX5	sp|Q9CQX5|CLDN1_MOUSE Claudin domain-containing protein 1 OS=Mus musculus GN=Cldnd1 PE=1 SV=1	1,17	0,468994104934954	0,796715861328205	23,0429429798347	23,0974215930066	23,6663250302842	23,5344531294744	23,2404230130127	23,5108751221745	12751267742,2822	14959071691,9045	−0,23037996112606	0,0148378061535915	0,0069734421048252	1,17314387825937	0.230379961126074	0,852410363751575	-0.230379961126074	3176
Q6PER3	sp|Q6PER3|MARE3_MOUSE Microtubule-associated protein RP/EB family member 3 OS=Mus musculus GN=Mapre3 PE=1 SV=1	1,17	0,503176273772178	0,814811668445208	30,7513155117469	31,2366860347337	31,4372046772841	31,4973921613793	31,3364515285498	31,0706333494719	33472168148706,7	39270406915828,4	−0,23048054298714	0,0113245179902765	0,0068851841961169	1,17322567039464	0.230480542987147	0,852350937448915	-0.230480542987147	3177
Q6ZPU9	sp|Q6ZPU9|KBP_MOUSE KIF1-binding protein OS=Mus musculus GN=Kbp PE=2 SV=2;sp|Q6ZPU9-2|KBP_MOUSE Isoform 2 of KIF1-binding protein OS=Mus musculus GN=Kbp	1,17	0,271353916299055	0,691573877451378	28,43987847634	28,2742677874623	27,9495817820908	28,4187987708268	28,3770459552226	28,3478835582042	1804388162243,24	2117469292121,71	−0,23083132116940	0,00883774781888691	0,0012558786467333	1,17351096423136	0.2308313211694	0,85214372126041	-0.2308313211694	3178
O70293	sp|O70293|GRK6_MOUSE G protein-coupled receptor kinase 6 OS=Mus musculus GN=Grk6 PE=1 SV=1;sp|O70293-2|GRK6_MOUSE Isoform GRK6B of G protein-coupled receptor kinase 6 OS=Mus musculus GN=Grk6	1,17	0,613075506113433	0,868059454773913	24,1143670029103	25,1428879762118	24,3139660285988	24,7396961746093	24,6308082037023	24,6808051491555	44722400191,8935	52483771395,8951	−0,23087377554188	0,0222399223559556	0,0022081105102622	1,17354549779751	0.230873775541882	0,852118645486505	-0.230873775541882	3179
P14576	sp|P14576|SRP54_MOUSE Signal recognition particle 54 kDa protein OS=Mus musculus GN=Srp54 PE=1 SV=2	1,17	0,533370327599365	0,8300431463043	26,9273907888982	27,541410950126	27,5899208736384	27,7842060823852	27,4606749995793	27,2944083728934	757210813856,851	888763035292,986	−0,23110380001693	0,0135015143565691	0,0090528229371947	1,17373262376705	0.231103800016929	0,851982793824489	-0.231103800016929	3180
Q9D902	sp|Q9D902|T2EB_MOUSE General transcription factor IIE subunit 2 OS=Mus musculus GN=Gtf2e2 PE=1 SV=2	1,17	0,209708720319373	0,641644645910089	22,4365116962145	22,5905470800734	22,8291248292886	22,8392616668271	22,8329525082737	22,6646252533469	6655622802,25691	7812153321,28814	−0,23114659067687	0,00874575309869258	0,0043485473465219	1,1737674374574	0.231146590676879	0,851957524197627	-0.231146590676879	3181
Q9R008	sp|Q9R008|KIME_MOUSE Mevalonate kinase OS=Mus musculus GN=Mvk PE=2 SV=1	1,17	0,791244624243618	0,942055274591853	25,7523846556078	25,5653482547121	25,6958788171413	25,2737175242021	25,1843097620353	27,0364602305821	140884171239,517	165377378036,553	−0,23125237219697	0,00373681649701971	0,0404346259517986	1,17385350377932	0.231252372196968	0,851895059119741	-0.231252372196968	3182
Q9CYZ8-2	sp|Q9CYZ8-2|SSBP2_MOUSE Isoform 2 of Single-stranded DNA-binding protein 2 OS=Mus musculus GN=Ssbp2;sp|Q9CYZ8|SSBP2_MOUSE Single-stranded DNA-binding protein 2 OS=Mus musculus GN=Ssbp2 PE=1 SV=1;sp|Q9D032-2|SSBP3_MOUSE Isoform 2 of Single-stranded DNA-b	1,17	0,462211467079472	0,793595716606975	24,3651120990611	23,7711154655879	24,1454988690372	24,1206114949404	24,5199292078442	24,1228372462825	29097229814,9151	34164718180,1102	−0,23162541755891	0,012465393671696	0,0094789317072397	1,17415707259519	0.231625417558917	0,851674808541367	-0.231625417558917	3183
Q8K4Z3	sp|Q8K4Z3|NNRE_MOUSE NAD(P)H-hydrate epimerase OS=Mus musculus GN=Apoa1bp PE=1 SV=1	1,17	0,358450970432126	0,732886916572711	29,7621840572139	30,0952385083161	30,3514194051073	30,295446665439	30,1584841687995	30,2374046939875	11456908085124,4	13455985964271,7	−0,23203035426468	0,00982560854914096	0,0022740638914927	1,17448668212176	0.232030354264687	0,851435793374395	-0.232030354264687	3184
Q62448	sp|Q62448|IF4G2_MOUSE Eukaryotic translation initiation factor 4 gamma 2 OS=Mus musculus GN=Eif4g2 PE=1 SV=2;sp|Q62448-2|IF4G2_MOUSE Isoform 2 of Eukaryotic translation initiation factor 4 gamma 2 OS=Mus musculus GN=Eif4g2	1,17	0,52581993479445	0,827322301337652	29,6207138314607	28,8605637488314	29,1550985480598	29,5137526899067	29,4808389247203	29,1249791637666	4860319906791,51	5709715191549,83	−0,23236750846638	0,0131202451357104	0,0073395553080895	1,17476118877925	0.232367508466385	0,851236838219988	-0.232367508466385	3185
Q9CRT8	sp|Q9CRT8|XPOT_MOUSE Exportin-T OS=Mus musculus GN=Xpot PE=2 SV=3	1,17	0,434753128219263	0,777519483446506	27,5887500513035	27,0364602305821	27,270293282656	27,7199698313342	27,3216488345134	27,3373962883802	717114378246,992	842527088694,6	−0,23251982803783	0,010155258355902	0,0082143117234403	1,17488522647417	0.232519828037834	0,851146969479734	-0.232519828037834	3186
O88643	sp|O88643|PAK1_MOUSE Serine/threonine-protein kinase PAK 1 OS=Mus musculus GN=Pak1 PE=1 SV=1	1,17	0,475918096442594	0,79947899324683	31,2551943052669	31,9425028325732	31,7966976397976	31,9827023770707	31,8094472578625	31,6858278589972	56473818607876,1	66351832696578,8	−0,23255412888505	0,0114367165742555	0,0046857053356715	1,1749131603317	0.232554128885055	0,851126733245274	-0.232554128885055	3187
O08795-2	sp|O08795-2|GLU2B_MOUSE Isoform 2 of Glucosidase 2 subunit beta OS=Mus musculus GN=Prkcsh;sp|O08795|GLU2B_MOUSE Glucosidase 2 subunit beta OS=Mus musculus GN=Prkcsh PE=1 SV=1	1,18	0,466319187191761	0,795722853632858	29,2199061542331	28,9217714421477	28,5719407915324	29,0218456153508	28,8859884835841	29,2904693642646	3573402941596,39	4199981155406,45	−0,23308425150280	0,0112205973693113	0,0070817895468492	1,17534496502377	0.2330842515028	0,850814041628855	-0.2330842515028	3188
Q91VT4	sp|Q91VT4|CBR4_MOUSE Carbonyl reductase family member 4 OS=Mus musculus GN=Cbr4 PE=1 SV=2	1,18	0,51091503142341	0,817055380454204	26,9068501601645	27,2983095342163	27,6483028203886	27,6870999533187	27,3399629165513	27,311293764426	707135051367,8	831185538382,266	−0,23318478053243	0,0135945207169846	0,0076217497430810	1,17542686757574	0.233184780532437	0,85075475776936	-0.233184780532437	3189
Q9QXY6	sp|Q9QXY6|EHD3_MOUSE EH domain-containing protein 3 OS=Mus musculus GN=Ehd3 PE=1 SV=2	1,18	0,382005134190369	0,749797813889279	31,0176093622394	31,5209983110802	31,4485046525447	31,6858278589972	31,3817725927108	31,4047907964564	40367149621816,8	47454718745035,9	−0,23336983155008	0,00868617227332412	0,0053759602305713	1,17557764641843	0.233369831550088	0,85064564050418	-0.233369831550089	3190
O08672	sp|O08672|KIFC2_MOUSE Kinesin-like protein KIFC2 OS=Mus musculus GN=Kifc2 PE=2 SV=1	1,18	0,492129254216847	0,80770032675628	25,3314691252537	24,8413906379364	24,6958846864592	24,9998552831891	24,9641191392659	25,3909018070988	68922474360,4236	81046757367,0124	−0,23377996936224	0,0133431762141911	0,0094258857996068	1,17591189403889	0.233779969362244	0,850403848340469	-0.233779969362244	3191

P63073	sp|P63073|IF4E_MOUSE Eukaryotic translation initiation factor 4E OS=Mus musculus GN=Eif4e PE=1 SV=1	1,18	0,645826875905594	0,889692946522666	27,4982193752751	28,3063564870261	28,663140619517	28,4367014913091	28,3553953189567	28,1636447074317	1690263405463,44	1988855584149,48	−0,234690433030852	0,0211982502597918	0,0049508710095515	1,1766542289923	0.234690433030851	0,849867340260539	-0.234690433030851	3192
Q9WVQ5	sp|Q9WVQ5|MTNB_MOUSE Methylthioribulose-1-phosphate dehydratase OS=Mus musculus GN=Apip PE=1 SV=1	1,18	0,6072122086467	0,866033363367613	25,3098334236589	25,9504924356993	26,3128537120198	26,2458051503349	25,9760107033085	25,839608189605	169772734511,441	199778418354,467	−0,234795959436239	0,019642256077028	0,0079443845888908	1,17674029890238	0.234795959436241	0,849805178706606	-0.234795959436241	3193
Q8BKZ9	sp|Q8BKZ9|ODPX_MOUSE Pyruvate dehydrogenase protein X component, mitochondrial OS=Mus musculus GN=Pdhx PE=1 SV=1	1,18	0,118278183934081	0,491463415677796	29,5137526899067	29,6699505699435	29,8286939050459	29,8851245258515	29,7312890475709	29,8851245258515	7688901604394,83	9050544459408,13	−0,235227066774277	0,00530731652019174	0,0029770534679877	1,1770919859652	0.235227066774277	0,849551277150199	-0.235227066774277	3194
Q9CQ10	sp|Q9CQ10|CHMP3_MOUSE Charged multivesicular body protein 3 OS=Mus musculus GN=Chmp3 PE=1 SV=3	1,18	0,383857167875195	0,751327160407723	26,4349457329772	26,5785183382483	27,0364602305821	26,8999307211861	26,7527581374521	26,886722967848	387626506404,978	456324770438,234	−0,235393741476929	0,0117732999665015	0,0030330102446317	1,17722798337605	0.235393741476927	0,849453134075362	-0.235393741476927	3195
Q3UGS4	sp|Q3UGS4|F195B_MOUSE Protein FAM195B OS=Mus musculus GN=Fam195b PE=1 SV=1	1,18	0,59305493923309	0,860080030359509	25,4815166293187	25,2026579862367	24,7900819061277	24,8661841819719	25,4876194191801	25,6104311364534	84336915064,7681	99300017000,5823	−0,235629714084899	0,0138271916076228	0,0157571019457376	1,17742055094525	0.235629714084901	0,849314205699215	-0.2356297140849	3196
Q9CQR4	sp|Q9CQR4|ACO13_MOUSE Acyl-coenzyme A thioesterase 13 OS=Mus musculus GN=Acot13 PE=1 SV=1	1,18	0,515859196230095	0,820430016377981	29,8993775648934	30,0580803905391	29,7550162373898	29,6389646388719	30,111798004507	30,4517424809733	9709812269085,77	11432733404173,1	−0,235655064933475	0,00506914861096737	0,0135759703824245	1,17744124060697	0.235655064933481	0,849299281792188	-0.235655064933481	3197
Q9CX30-2	sp|Q9CX30-2|YIF1B_MOUSE Isoform 2 of Protein YIF1B OS=Mus musculus GN=Yif1b;sp|Q9CX30|YIF1B_MOUSE Protein YIF1B OS=Mus musculus GN=Yif1b PE=1 SV=2	1,18	0,544271192353142	0,834610453409091	25,1035425680548	24,9224630227272	25,4312369546124	25,6579078890505	24,8919913695741	25,3979425844979	83859966716,7364	98758891881,2395	−0,235928391298701	0,0102528448502836	0,0153850113859415	1,17766433433999	0.235928391298701	0,849138392698659	-0.235928391298701	3198
Q06890	sp|Q06890|CLUS_MOUSE Clusterin OS=Mus musculus GN=Clu PE=1 SV=1	1,18	0,0453984644974009	0,312598733858675	28,5539886874427	28,3714205600626	28,4835717147051	28,7572558211707	28,5598383543679	28,5833286268197	2313217019446,28	2724958371607,57	−0,236333568555577	0,00323416751824991	0,0037662030442972	1,17799512484127	0.236333568555574	0,848899947811538	-0.236333568555574	3199
Q8CC35-3	sp|Q8CC35-3|SYNPO_MOUSE Isoform 3 of Synaptopodin OS=Mus musculus GN=Synpo;sp|Q8CC35|SYNPO_MOUSE Synaptopodin OS=Mus musculus GN=Synpo PE=1 SV=2;sp|Q8CC35-2|SYNPO_MOUSE Isoform 2 of Synaptopodin OS=Mus musculus GN=Synpo	1,18	0,297178118519415	0,711969538636193	31,625103247153	31,3439939544913	31,138693565225	31,5806609515781	31,625103247153	31,3949762767942	42024066791999	49529033975295,7	−0,237058700028531	0,0077842901474647	0,0038712375414768	1,17858736091495	0.237058700028533	0,848473378522988	-0.237058700028533	3200
Q8BTY8-2	sp|Q8BTY8-2|SCFD2_MOUSE Isoform 2 of Sec1 family domain-containing protein 2 OS=Mus musculus GN=Scfd2;sp|Q8BTY8|SCFD2_MOUSE Sec1 family domain-containing protein 2 OS=Mus musculus GN=Scfd2 PE=2 SV=1;sp|Q8BTY8-3|SCFD2_MOUSE Isoform 3 of Sec1 family domai	1,18	0,436817223007989	0,778450481049573	23,9062094718821	24,6324302907486	24,2905635733946	24,49311460887	24,3979637610652	24,4317373548227	34922638099,289	41168472711,5369	−0,237377381782007	0,0149658712427591	0,0019736613713145	1,17884773179192	0.237377381782007	0,848285977087083	-0.237377381782007	3201
Q6P1Y8	sp|Q6P1Y8|INP4B_MOUSE Type II inositol 3,4-bisphosphate 4-phosphatase OS=Mus musculus GN=Inpp4b PE=2 SV=1	1,18	0,0746838651501144	0,406462906374543	24,4250582171058	24,517555127317	24,4487515518704	24,573149001652	24,511216945845	24,801127570362	42119990504,1409	49661600183,2092	−0,237625637298129	0,00196399775344493	0,0061991204212000	1,17905060254766	0.237625637298128	0,848140018621105	-0.237625637298128	3202
Q91XU3	sp|Q91XU3|PI42C_MOUSE Phosphatidylinositol 5-phosphate 4-kinase type-2 gamma OS=Mus musculus GN=Pip4k2c PE=2 SV=1	1,18	0,11940272662249	0,49404646867774	29,6000841427096	29,3699426896834	29,3002269215392	29,5874435150517	29,6831401315376	29,4939123683025	6003825215763,16	7079081887348,66	−0,237680286294664	0,00533310218026781	0,0031977632137439	1,17909526559207	0.237680286294665	0,848107891857115	-0.237680286294665	3203
Q6PDL0	sp|Q6PDL0|DC1L2_MOUSE Cytoplasmic dynein 1 light intermediate chain 2 OS=Mus musculus GN=Dync1li2 PE=1 SV=2	1,18	0,368907898099296	0,740483034506945	29,5856163544791	30,111798004507	30,1282886884742	30,1853711148321	30,0338596200803	30,1011524211797	10083293041248,9	11890898427274,6	−0,237890846516508	0,0103086880463293	0,0025214625604271	1,17926736618991	0.237890846516507	0,847984120200747	-0.237890846516507	3204
Q8K2C6	sp|Q8K2C6|SIR5_MOUSE NAD-dependent protein deacylase sirtuin-5, mitochondrial OS=Mus musculus GN=Sirt5 PE=1 SV=1	1,18	0,5489495260955	0,838092765530185	26,2439255198213	26,8229841428725	27,0900402227433	27,0846207130066	26,886722967848	26,6810725947884	401704787020,772	473841520854,742	−0,238268968025746	0,0161884384538962	0,0075057814555619	1,17957648543093	0.238268968025748	0,84776189789395	-0.238268968025748	3205
Q922Q1	sp|Q922Q1|MARC2_MOUSE Mitochondrial amidoxime reducing component 2 OS=Mus musculus GN=Marc2 PE=1 SV=1	1,18	0,274436546814379	0,695903623458361	28,3564695698073	27,9148680147052	28,3623185321326	28,4137586698619	28,3160857868948	28,399855058049	1786391368271,88	2107590578319,22	−0,238546450197328	0,0090979369232171	0,0018619997824120	1,17980338225551	0.238546450197326	0,847598858454049	-0.238546450197326	3206
Q9DB20	sp|Q9DB20|ATPO_MOUSE ATP synthase subunit O, mitochondrial OS=Mus musculus GN=Atp5o PE=1 SV=1	1,18	0,337550548818111	0,729620681454069	30,8553810226389	30,9355404205447	30,7896398570902	30,7635181652725	30,9766739283493	31,3364515285498	25258582126287	29800547270620,8	−0,238565168558217	0,00236774073822407	0,0093334914304532	1,17981868980709	0.238565168558214	0,847587861286979	-0.238565168558214	3207
Q9JKY0	sp|Q9JKY0|RCD1_MOUSE Cell differentiation protein RCD1 homolog OS=Mus musculus GN=Rqcd1 PE=1 SV=1	1,18	0,356945289441794	0,732843629012038	26,7926130600652	27,0501953007888	27,4054318063668	27,2015892625047	27,2501225437999	27,2928682782776	577946258202,14	681930348671,907	−0,238689045790804	0,0113616034662879	0,0016760772722594	1,1799199994706	0.238689045790804	0,847515086148782	-0.238689045790804	3208
Q9EP97	sp|Q9EP97|SENP3_MOUSE Sentrin-specific protease 3 OS=Mus musculus GN=Senp3 PE=1 SV=1	1,18	0,694314367812551	0,910309912045398	23,3186569620764	24,6569155403246	23,9175054815775	24,4713782819228	23,9835441013259	23,9369813448864	25561657914,6556	30185713184,9001	−0,239884475787483	0,0279731434903188	0,0122669660244493	1,18089809689509	0.239884475787482	0,84681311844712	-0.239884475787482	3209

Q8BW96	sp|Q8BW96|KCC1D_MOUSE Calcium/calmodulin-dependent protein kinase type 1D OS=Mus musculus GN=Camk1d PE=1 SV=2;sp|Q8BW96-3|KCC1D_MOUSE Isoform 3 of Calcium/calmodulin-dependent protein kinase type 1D OS=Mus musculus GN=Camk1d	1,18	0,249508901274804	0,673464050610029	30,3400376693966	29,8391442128287	30,0893751498851	30,2511880637938	30,2570757598888	30,2598956411556	11687242879699,7	13805016478146,6	−0,2402579840374	0,00832338859200011	0,0001468448538671	1,18120386649322	0.240257984037484	0,846593910134092	-0.240257984037485	3210
Q9WV34	sp|Q9WV34|MPP2_MOUSE MAGUK p55 subfamily member 2 OS=Mus musculus GN=Mpp2 PE=1 SV=1;sp|Q9WV34-2|MPP2_MOUSE Isoform 2 of MAGUK p55 subfamily member 2 OS=Mus musculus GN=Mpp2	1,18	0,322294517281212	0,725294159481728	30,8001322897362	31,2859805099124	31,2665446190202	31,3949762767942	31,2366860347337	31,2206366567217	32672421960912,1	38593294360719,7	−0,2402767952673	0,0088395660755924	0,0030800536062594	1,18121926825293	0.240276795267395	0,846582871509568	-0.240276795267395	3211
Q8BKX1-3	sp|Q8BKX1-3|BAIP2_MOUSE Isoform 3 of Brain-specific angiogenesis inhibitor 1-associated protein 2 OS=Mus musculus GN=Baiap2;sp|Q8BKX1-2|BAIP2_MOUSE Isoform 2 of Brain-specific angiogenesis inhibitor 1-associated protein 2 OS=Mus musculus GN=Baiap2;sp|Q8	1,18	0,400776617702778	0,755487293133664	31,090420390194	31,736695594508	31,5364546439614	31,7285609258969	31,5462290964587	31,5898004571495	45764213769660,1	54082412680066,9	−0,2409396180654	0,0105179138815716	0,0030111491639024	1,18176208493986	0.240939618065428	0,846194012097529	-0.240939618065428	3212
P24668	sp|P24668|MPRD_MOUSE Cation-dependent mannose-6-phosphate receptor OS=Mus musculus GN=M6pr PE=1 SV=1	1,18	0,318960169629879	0,722907157640623	27,7361956183784	27,3309114068393	27,8449372025876	27,924640903582	27,749949888637	27,738528557757	1006347017496,67	1189284660482,67	−0,2409661979562	0,00980174959514409	0,003751618300096	1,18178385766081	0.240966197956248	0,846178422151888	-0.240966197956248	3213
Q9CRY7	sp|Q9CRY7|GDPD1_MOUSE Glycerophosphodiester phosphodiesterase domain-containing protein 1 OS=Mus musculus GN=Gdpd1 PE=2 SV=1	1,18	0,376729064991905	0,74727992066734	28,3607504246018	28,696381711632	28,9559879547382	28,9338137105631	28,6809841596718	28,9014392327936	2829270527787,06	3345862758314,26	−0,2419481394269	0,0104086507333635	0,004770702766506	1,18258848895982	0.241948139426927	0,845602683719319	-0.241948139426927	3214
Q9D2G2	sp|Q9D2G2|ODO2_MOUSE Dihydrolipoyllysine-residue succinyltransferase component of 2-oxoglutarate dehydrogenase complex, mitochondrial OS=Mus musculus GN=Dlst PE=1 SV=1;sp|Q9D2G2-2|ODO2_MOUSE Isoform 2 of Dihydrolipoyllysine-residue succinyltransferase co	1,18	0,215637181856227	0,646799042967852	31,9453672776117	31,5364546439614	31,5898004571495	31,8186731138519	31,9453672776117	31,8110328188897	57946471369280,9	68534455640479,6	−0,2421086727083	0,00701446709641621	0,002368271657280	1,18272008667661	0.242108672708337	0,845508596044864	-0.242108672708337	3215
P16460	sp|P16460|ASSY_MOUSE Argininosuccinate synthase OS=Mus musculus GN=Ass1 PE=1 SV=1	1,18	0,13380177967908	0,518639007654437	30,4506158641339	30,0650549662063	30,2888262852398	30,4300715036396	30,4300715036396	30,447826850692	13973247228802,5	16526560876334,8	−0,2421192095568	0,00639645643666612	0,000336806997775	1,18272872480702	0.242119209556857	0,845502420821956	-0.242119209556857	3216
Q91WQ3	sp|Q91WQ3|SYYC_MOUSE Tyrosine--tRNA ligase, cytoplasmic OS=Mus musculus GN=Yars PE=2 SV=3	1,18	0,325759997548697	0,726927682919497	30,2081872493644	30,7314349400907	30,6260640465842	30,7933119851167	30,5824842469731	30,6939719257556	18009047960673,7	21304143905631,3	−0,2424121629304	0,00906695359087719	0,003436704008939	1,1829689138567	0.242412162930417	0,845330750695567	-0.242412162930416	3217
P61961	sp|P61961|UFM1_MOUSE Ubiquitin-fold modifier 1 OS=Mus musculus GN=Ufm1 PE=1 SV=1	1,18	0,566647295316852	0,845222501953069	27,12666654682	26,8202978882426	27,1802077444987	26,8583139812733	27,0296671524623	27,7433277429496	555087017405,242	656662675601,998	−0,24243850429029	0,00718102701244391	0,0172501112646695	1,18299051322002	0.24243850429029	0,845315316416247	-0.24243850429029	3218
Q8BNW9	sp|Q8BNW9|KBTBB_MOUSE Kelch repeat and BTB domain-containing protein 11 OS=Mus musculus GN=Kbtbd11 PE=1 SV=3	1,18	0,41544022172835	0,767871528551302	29,7971159967527	29,541802057065	29,104176927775	29,5767627429087	29,6534011397899	29,7170699033562	6359886584723,45	7523690779867,91	−0,2424395177056	0,0118870971941545	0,0023694974928252	1,18299134420729	0.24243951770568	0,845314722628075	-0.24243951770568	3219
Q91VZ6	sp|Q91VZ6|SMAP1_MOUSE Stromal membrane-associated protein 1 OS=Mus musculus GN=Smap1 PE=1 SV=1	1,18	0,425684431028006	0,771946317393575	29,5576191780415	30,2207870528903	30,1058851287532	30,1189132769238	30,2236511949269	30,0459928945559	10282149539066,2	12164209665277,7	−0,2425006890620	0,0118284895072991	0,002963961957842	1,18304150499473	0.24250068906204	0,845278881407004	-0.242500689062041	3220
O89017	sp|O89017|LGMN_MOUSE Legumain OS=Mus musculus GN=Lgmn PE=1 SV=1;sp|Q9R0J8|LGMN_RAT Legumain OS=Rattus norvegicus GN=Lgmn PE=1 SV=1	1,18	0,553462726516892	0,839347582913522	24,620547219318	25,1499790121567	25,526381389398	25,404963452821	25,0561523437009	25,3401904290326	79495734808,3138	94050910642,7242	−0,2425644552019	0,0181308781946849	0,007342999798739	1,18309379578045	0.242564455201906	0,84524152148083	-0.242564455201906	3221
Q8VE38-2	sp|Q8VE38-2|OXND1_MOUSE Isoform 2 of Oxidoreductase NAD-binding domain-containing protein 1 OS=Mus musculus GN=Oxnad1;sp|Q8VE38|OXND1_MOUSE Oxidoreductase NAD-binding domain-containing protein 1 OS=Mus musculus GN=Oxnad1 PE=2 SV=2	1,18	0,652223180988206	0,891901943526937	24,3850652312053	25,0817452967474	25,5637107751137	25,1266580132717	24,946752196482	25,462854440618	72741199578,5842	86098246597,9944	−0,24321113970659	0,0236932925797707	0,0104042293556591	1,18362423354017	0.243211139706591	0,844862728949914	-0.243211139706592	3222
Q99LS3	sp|Q99LS3|SERB_MOUSE Phosphoserine phosphatase OS=Mus musculus GN=Psph PE=1 SV=1	1,18	0,46249624544347	0,793595716606975	27,745330412391	28,1788730675729	28,429387167353	28,3414907470162	28,0692982272581	28,4487032309194	1627170201139,5	1926059653817,75	−0,2432872229089	0,012308378097605	0,00691458775557	1,18368665580831	0.243287222908937	0,844818174719664	-0.243287222908937	3223
Q8QZT2	sp|Q8QZT2|CCSAP_MOUSE Centriole, cilia and spindle-associated protein OS=Mus musculus GN=Ccsap PE=2 SV=1	1,18	0,198561280257017	0,628440552344853	27,436658796006	27,0647658758816	27,0428461881834	27,2900373208184	27,3802624477312	27,3802624477312	637884851221,838	755153899203,086	−0,2434746762829	0,00814201985382536	0,001904612225818	1,18384046549565	0.243474676282954	0,844708412278607	-0.243474676282954	3224
Q9JHW2	sp|Q9JHW2|NIT2_MOUSE Omega-amidase NIT2 OS=Mus musculus GN=Nit2 PE=1 SV=1	1,18	0,392399248281674	0,753881747531964	28,1372043839272	28,4660063045051	28,7679720830482	28,7546109971162	28,4918017637118	28,6310783417307	2284254716985,04	2704208468617,19	−0,2434828394690	0,0110861406884044	0,004593165968338	1,18384716402663	0.243482839469094	0,844703632687425	-0.243482839469094	3225

Q99N50-2	sp|Q99N50-2|SYTL2_MOUSE Isoform 2 of Synaptotagmin-like protein 2 OS=Mus musculus GN=Sytl2;sp|Q99N50-7|SYTL2_MOUSE Isoform 7 of Synaptotagmin-like protein 2 OS=Mus musculus GN=Sytl2;sp|Q99N50-8|SYTL2_MOUSE Isoform 8 of Synaptotagmin-like protein 2 OS=Mu	1,18	0,416195917282232	0,767877733621866	24,9908094544841	25,6077300356249	25,4571592515809	25,6922263661063	25,3737724117129	25,4960085354994	102374099251,757	121195296786,839	−0,24348295514946	0,0126873341499646	0,006295009300823	1,18384725895167	0.243482955149469	0,844703564956113	-0.243482955149469	3226
P27048	sp|P27048|RSMB_MOUSE Small nuclear ribonucleoprotein-associated protein B OS=Mus musculus GN=Snrpb PE=1 SV=1;sp|P63163|RSMN_MOUSE Small nuclear ribonucleoprotein-associated protein N OS=Mus musculus GN=Snrpn PE=2 SV=1	1,18	0,409276294157388	0,76291790520755	27,3368848768073	27,7067011958313	28,0072704850482	27,9775310134962	27,7557362941429	27,8242150678396	1054005572001,07	1247913565619,44	−0,24363551830486	0,0121294959894302	0,004077529847903	1,18397245590479	0.243635518304875	0,844614243357377	-0.243635518304875	3227
Q9D8B3	sp|Q9D8B3|CHM4B_MOUSE Charged multivesicular body protein 4b OS=Mus musculus GN=Chmp4b PE=1 SV=2	1,18	0,351684804618565	0,732843629012038	28,4487032309194	28,8597896366355	29,0458241396507	28,8780890237702	29,0254710300426	28,9577992893341	3170062233789,31	3753787505674,23	−0,24383582119440	0,0106148874381312	0,0025479535276841	1,18413684932209	0.243835821194404	0,844496985777016	-0.243835821194404	3228
Q91XD7	sp|Q91XD7|CREL1_MOUSE Cysteine-rich with EGF-like domain protein 1 OS=Mus musculus GN=Creld1 PE=2 SV=1	1,18	0,625731552719003	0,876102744788026	26,5257288518079	25,4184137485655	26,3040142907628	26,3932386786055	26,2285373005935	26,133625476344	212608644674,121	251774702074,916	−0,24393307252918	0,022462710174196	0,005003874219093	1,18421667407187	0.243933072529184	0,844440060585829	-0.243933072529183	3229
Q78PG9	sp|Q78PG9|CCD25_MOUSE Coiled-coil domain-containing protein 25 OS=Mus musculus GN=Ccdc25 PE=1 SV=1	1,18	0,534252828998456	0,8300431463043	24,846359962555	25,5463841594127	25,528533155215	25,7421229489083	25,4434411168015	25,2432823524486	97888256546,4445	115933448013,213	−0,24408916086468	0,01577051314554	0,0098537384280761	1,18434480399808	0.24408916086468	0,84434870370877	-0.24408916086468	3230
Q8BLN5	sp|Q8BLN5|ERG7_MOUSE Lanosterol synthase OS=Mus musculus GN=Lss PE=2 SV=2	1,18	0,00658887899223629	0,0910375021402342	22,3531370911114	22,3263702104077	22,3263702104077	22,4867335334843	22,6191277512212	22,4077897022244	5012960541,56817	5937478165,89017	−0,24418742476065	0,000691903442809242	0,004745245347331	1,18442547405984	0.244187424760653	0,844291195943557	-0.244187424760653	3231
Q9CZ13	sp|Q9CZ13|QCR1_MOUSE Cytochrome b-c1 complex subunit 1, mitochondrial OS=Mus musculus GN=Uqcrc1 PE=1 SV=2	1,18	0,0888056942826122	0,441176105282424	29,9610005225932	29,7389386091483	29,7592429065261	30,1058851287532	29,9980024503029	29,8638697170678	8923660480299,33	10572235963020,6	−0,24457300080938	0,00411696065335719	0,004042939727070	1,18474206704309	0.244573000809379	0,844065580026058	-0.244573000809379	3232
Q9DCZ1	sp|Q9DCZ1|GMPR1_MOUSE GMP reductase 1 OS=Mus musculus GN=Gmpr PE=2 SV=1	1,18	0,547323862787519	0,836745479092484	27,8490417699696	26,960898296503	27,4627343947558	27,5648173464815	27,8064691473181	27,4101106762398	813185282602,84	963462165908,196	−0,24464390972967	0,0162382820123495	0,007239389850157	1,1848002989237	0.244643909729676	0,844024094953745	-0.244643909729676	3233
Q6RHR9-3	sp|Q6RHR9-3|MAGI1_MOUSE Isoform 3 of Membrane-associated guanylate kinase, WW and PDZ domain-containing protein 1 OS=Mus musculus GN=Magi1;sp|Q6RHR9|MAGI1_MOUSE Membrane-associated guanylate kinase, WW and PDZ domain-containing protein 1 OS=Mus musculus	1,18	0,724926035756289	0,921649709826669	23,2006453878741	23,7458020638621	23,9294139405435	24,5253945429987	23,8225765445107	23,0370888286754	18210917808,4759	21579723244,4296	−0,24487272842196	0,0160436846891455	0,0312895482069101	1,18498822911527	0.244872728421958	0,843890239101037	-0.244872728421958	3234
Q3UFF7	sp|Q3UFF7|LYPL1_MOUSE Lysophospholipase-like protein 1 OS=Mus musculus GN=Lyplal1 PE=1 SV=3	1,19	0,411535275226876	0,765243184864724	26,0128955673314	26,2170897415584	26,6455238891739	26,5920726184234	26,2893784167721	26,5038887636353	262059234060,47	310602542359,18	−0,24517669314000	0,012280232297651	0,005883147236237	1,18523792329908	0.245176693140001	0,843712456665681	-0.245176693140001	3235
O35841	sp|O35841|API5_MOUSE Apoptosis inhibitor 5 OS=Mus musculus GN=Api5 PE=1 SV=2	1,19	0,476443970317211	0,799905345588749	29,1030743452272	29,7243921412505	29,8846521691104	29,7503945006217	29,6861147613	29,7887479080092	6956560023034,87	8254275215382,62	−0,24676746331049	0,013960853092264	0,001743659061814	1,18654553228186	0.246767463310497	0,842782660077855	-0.246767463310497	3236
Q8BWU5	sp|Q8BWU5|OSGEP_MOUSE Probable tRNA N6-adenosine threonylcarbamoyltransferase OS=Mus musculus GN=Osgep PE=2 SV=2	1,19	0,667520778076615	0,898352728146089	24,8919913695741	25,7084262444795	26,1449400230198	25,6220992401053	25,5763604830779	26,0601159193524	128833392946,941	152870737378,252	−0,24680569045839	0,0248615129714098	0,0103706404894838	1,18657697264257	0.246805690458391	0,842760329128035	-0.246805690458391	3237
Q8VHE0	sp|Q8VHE0|SEC63_MOUSE Translocation protein SEC63 homolog OS=Mus musculus GN=Sec63 PE=2 SV=4	1,19	0,445925564840996	0,784849692944763	24,5262771526765	24,4846098063473	24,024166766318	24,7207953569625	24,2081894088514	24,6197060572759	37403043252,6137	44387790257,3667	−0,24700723124595	0,0114456754947564	0,0110750019185902	1,18674274597335	0.247007231245953	0,842642605900081	-0.247007231245953	3238
Q3UUJ4	sp|Q3UUJ4|STRAA_MOUSE STE20-related kinase adapter protein alpha OS=Mus musculus GN=Strada PE=2 SV=1;sp|Q7TNZ6-2|STRAA_RAT Isoform 2 of STE20-related kinase adapter protein alpha OS=Rattus norvegicus GN=Strada;sp|Q3UUJ4-2|STRAA_MOUSE Isoform 2 of STE20-	1,19	0,477811383123909	0,799905345588749	26,2041255291928	25,7508401370558	25,7471346094699	26,446728601505	25,9119492395323	25,8577700523384	177227506549,655	210373276685,435	−0,24734891909571	0,010145697512037	0,012485519425570	1,18702384737605	0.247348919095712	0,842443058082221	-0.247348919095712	3239
P18572-2	sp|P18572-2|BASI_MOUSE Isoform 2 of Basigin OS=Mus musculus GN=Bsg;sp|P18572|BASI_MOUSE Basigin OS=Mus musculus GN=Bsg PE=1 SV=2	1,19	0,178951053855132	0,598329724962355	29,8229827561781	29,4859248540224	29,4161611861033	29,7621840572139	29,7759040277786	29,7016909671295	6986654407788,18	8294327948102,24	−0,24752331118787	0,00735600872888655	0,001327438396919	1,18716734276371	0.247523311187875	0,84234123023635	-0.247523311187875	3240
Q9D7V2	sp|Q9D7V2|LYSM2_MOUSE LysM and putative peptidoglycan-binding domain-containing protein 2 OS=Mus musculus GN=Lysmd2 PE=2 SV=2	1,19	0,453645692180865	0,788322280554728	26,5444468376233	26,660624355741	27,1876699138534	26,9044098251012	26,8091718647215	27,1948465255442	434551529289,606	516053392952,505	−0,24799307785909	0,0127916474441776	0,007449221044153	1,18755396810163	0.247993077859091	0,842066993888751	-0.247993077859091	3241

Q8BL65-2	sp|Q8BL65-2|ABLM2_MOUSE Isoform 2 of Actin-binding LIM protein 2 OS=Mus musculus GN=Ablim2;sp|Q8BL65-3|ABLM2_MOUSE Isoform 3 of Actin-binding LIM protein 2 OS=Mus musculus GN=Ablim2;sp|Q8BL65-4|ABLM2_MOUSE Isoform 4 of Actin-binding LIM protein 2 OS=Mus	1,19	0,330048129152093	0,72953198881961	27,6388890090614	27,4813946984528	27,2551673122194	27,5118748975731	27,9035914445976	27,4758239742999	841526779200,603	999409164173,929	−0,248066265099366	0,00702457172987654	0,0085865621747282	1,1876142136835	0.248066265099366	0,842024277310059	-0.248066265099366	3242
Q9Z2M7	sp|Q9Z2M7|PMM2_MOUSE Phosphomannomutase 2 OS=Mus musculus GN=Pmm2 PE=1 SV=1	1,19	0,561424157634787	0,844368539265838	27,3001174003902	27,3379076999531	28,0318362877081	27,9902351106764	27,3902087291243	27,8064691473181	928299693040,467	1102907505753,9	−0,248649259286154	0,0149504003446857	0,0110869123220458	1,18809422649009	0.24864925928615	0,841684083386414	-0.24864925928615	3243
O70166	sp|O70166|STMN3_MOUSE Stathmin-3 OS=Mus musculus GN=Stmn3 PE=1 SV=1	1,19	0,486514579545387	0,804557984450102	26,0333327348677	26,7397710425091	26,7440322826555	26,8289507902745	26,5997264252204	26,6060338018682	324552134113,1	385665791326,101	−0,248900941409812	0,0154343338944029	0,0048938788716641	1,18830151088054	0.248900941409811	0,841537262086784	-0.248900941409811	3244
Q9WUQ2	sp|Q9WUQ2|PREB_MOUSE Prolactin regulatory element-binding protein OS=Mus musculus GN=Preb PE=1 SV=1	1,19	0,156164477637932	0,560783674049504	26,7237904447108	26,4478784720452	26,336120588715	26,7516578076357	26,6541371203836	26,6200366760221	323542559527,199	384525983640,373	−0,24912558885976	0,0075291979234671	0,0025607848883744	1,18848656016782	0.249125588859761	0,841406233368592	-0.249125588859761	3245
Q9R049-2	sp|Q9R049-2|AMFR_MOUSE Isoform 2 of E3 ubiquitin-protein ligase AMFR OS=Mus musculus GN=Amfr;sp|Q9R049|AMFR_MOUSE E3 ubiquitin-protein ligase AMFR OS=Mus musculus GN=Amfr PE=1 SV=2	1,19	0,699675714674108	0,913381670666027	23,8670266020545	23,8343072841257	23,2024655836816	24,3700164161251	23,9989811215525	23,0529989350732	18381302625,9747	21847059168,1627	−0,249200082090491	0,0158495019233761	0,0285249477079919	1,18854792898576	0.249200082090487	0,841362788670496	-0.249200082090488	3246
Q9WUA6-2	sp|Q9WUA6-2|AKT3_MOUSE Isoform 2 of RAC-gamma serine/threonine-protein kinase OS=Mus musculus GN=Akt3;sp|Q9WUA6|AKT3_MOUSE RAC-gamma serine/threonine-protein kinase OS=Mus musculus GN=Akt3 PE=1 SV=1	1,19	0,466902247229798	0,796164546189264	27,6254440730023	28,2304457403943	28,3342637623689	28,410000627506	28,1636447074317	28,1351226080958	1540894989033,6	1831682356598,44	−0,249400791930436	0,0136406703315961	0,0053527488426635	1,1887132930111	0.249400791930438	0,841245745193041	-0.249400791930438	3247
Q5SWW4	sp|Q5SWW4|MED13_MOUSE Mediator of RNA polymerase II transcription subunit 13 OS=Mus musculus GN=Med13 PE=2 SV=1	1,19	0,100393446921316	0,46324407107743	25,9154627291353	25,9984600276786	26,239003526529	26,2361110611353	26,147377349823	26,2893784167721	205965687799,179	244942406066,565	−0,250038548314958	0,00645050115025681	0,0027354378337009	1,18923889063206	0.250038548314962	0,840873947091082	-0.250038548314962	3248
P24270	sp|P24270|CATA_MOUSE Catalase OS=Mus musculus GN=Cat PE=1 SV=4	1,19	0,34533459533055	0,730303241451822	28,415569281999	28,9014392327936	28,8574544305787	29,0627516643017	28,7217355155172	28,9104405271909	2985597415041,73	3551209038538,87	−0,250290643524457	0,00935502016546415	0,0059114682055835	1,18944671530312	0.250290643524455	0,840727026384834	-0.250290643524455	3249
P14602	sp|P14602|HSPB1_MOUSE Heat shock protein beta-1 OS=Mus musculus GN=Hspb1 PE=1 SV=3;sp|P14602-2|HSPB1_MOUSE Isoform B of Heat shock protein beta-1 OS=Mus musculus GN=Hspb1;sp|P14602-3|HSPB1_MOUSE Isoform C of Heat shock protein beta-1 OS=Mus musculus GN=	1,19	0,459283284727888	0,791152496918363	26,3698715068758	26,9594848677855	27,1124388235916	27,0856899088401	26,8756565229089	27,0009567310953	441715449700,364	525404557033,019	−0,250311419753048	0,0146229207832469	0,0039154378107628	1,1894638446299	0.250311419753047	0,840714919175329	-0.250311419753047	3250
O35075	sp|O35075|DSCR3_MOUSE Down syndrome critical region protein 3 homolog OS=Mus musculus GN=Dscr3 PE=2 SV=1	1,19	0,780243943069343	0,940251150142017	23,3825283651281	24,7031955909906	25,3615584974045	24,2905635733946	25,0535866622164	24,6240536772299	42912435149,7148	51049825888,65	−0,250510268683392	0,0411648903735407	0,015514137950415	1,18962780160448	0.250510268683392	0,840599050098927	-0.250510268683392	3251
Q9CX56	sp|Q9CX56|PSMD8_MOUSE 26S proteasome non-ATPase regulatory subunit 8 OS=Mus musculus GN=Psmd8 PE=1 SV=2	1,19	0,373750649999866	0,744143356508855	28,961551696187	29,3206469921288	29,5830171708225	29,5664435234524	29,3158045711717	29,5039457329552	5245571361638,99	6240395072417,32	−0,250537443827417	0,0106521587549263	0,0044281557080396	1,18965021009027	0.250537443827418	0,840583216409571	-0.250537443827418	3252
A2AJL3-2	sp|A2AJL3-2|FGGY_MOUSE Isoform 2 of FGGY carbohydrate kinase domain-containing protein OS=Mus musculus GN=Fggy;sp|A2AJL3|FGGY_MOUSE FGGY carbohydrate kinase domain-containing protein OS=Mus musculus GN=Fggy PE=2 SV=1	1,19	0,38699138180825	0,752468171504595	22,5214498237658	22,4425016638024	22,1623409962791	22,7884248163617	22,2228918150151	22,6368476224383	5218265103,50966	6209759998,77687	−0,250967271504141	0,00843406414116756	0,0129819481045105	1,19000469995293	0.250967271504141	0,840332815525479	-0.250967271504141	3253
Q9CQA9	sp|Q9CQA9|NTPCR_MOUSE Cancer-related nucleoside-triphosphatase homolog OS=Mus musculus GN=Ntpcr PE=2 SV=1	1,19	0,389117951226236	0,753319093070654	24,6585297673407	24,5199292078442	24,3206206984587	24,5452995178382	24,4526054481224	25,0243761876188	43659783293,2398	51978380936,5839	−0,251606726829708	0,00693318845213633	0,012437076709198	1,19053227056746	0.251606726829708	0,839960431751555	-0.251606726829708	3254
P48758	sp|P48758|CBR1_MOUSE Carbonyl reductase [NADPH] 1 OS=Mus musculus GN=Cbr1 PE=1 SV=3	1,19	0,462023631539002	0,793595716606975	31,2799527489482	31,7920975405357	32,0501774277855	32,0149961289679	31,7691737448374	31,8619322988203	58932229028829,6	70176461014176,5	−0,251930359596953	0,0123641108019694	0,0038936635490933	1,19079936684299	0.251930359596953	0,839772028642547	-0.251930359596953	3255
Q9QYI4	sp|Q9QYI4|DJB12_MOUSE DnaJ homolog subfamily B member 12 OS=Mus musculus GN=Dnajb12 PE=2 SV=2	1,19	0,505110593004985	0,814993646406912	25,2114965092376	24,3086666205643	24,8090721097822	24,9050721699232	24,9733867043026	24,974920529059	57578308577,6551	68570377788,7521	−0,25206006189399	0,0182552748460291	0,0015987881160875	1,19090642783075	0.252060061893992	0,839696534194973	-0.252060061893992	3256
Q9DCW4	sp|Q9DCW4|ETFB_MOUSE Electron transfer flavoprotein subunit beta OS=Mus musculus GN=Etfb PE=1 SV=3	1,19	0,424199153492488	0,7711031271846	30,31031921555	30,8605365941129	31,0040688696121	30,9907657784686	30,7933119851167	30,9161912299983	22064142448119,4	26286843019719,1	−0,252637212337358	0,0119187510579766	0,0032267478321116	1,1913829454976	0.252637212337359	0,83936068060999	-0.252637212337359	3257
Q91W82	sp|Q91W82|UB2E2_MOUSE Ubiquitin-conjugating enzyme E2 E2 OS=Mus musculus GN=Ube2e2 PE=2 SV=1	1,19	0,400796078842446	0,755487293133664	26,0372835360317	26,5284979350479	26,7001686947059	26,6864288116258	26,6335512707477	26,4722972144417	298484364626,101	355725700148,206	−0,253109847274002	0,0130209575893987	0,0041937573589645	1,19177331313085	0.253109847274003	0,839085746410067	-0.253109847274003	3258

Q9D2N4-2	sp|Q9D2N4-2|DTNA_MOUSE Isoform 2 of Dystrobrevin alpha OS=Mus musculus GN=Dtna;sp|Q9D2N4|DTNA_MOUSE Dystrobrevin alpha OS=Mus musculus GN=Dtna PE=1 SV=2;sp|Q9D2N4-7|DTNA_MOUSE Isoform 7 of Dystrobrevin alpha OS=Mus musculus GN=Dtna;sp|Q9D2N4-4|DTNA_MOU	1,19	0,13776661736267	0,528144153635909	28,4788737237828	28,2518043492866	28,1910966219225	28,5793494389656	28,5437339219033	28,3251900002394	1966464215621,57	2343713579277,39	−0,253192338213612	0,00535874390273418	0,0048315110437608	1,19184145872524	0.253192338213616	0,839037770232942	-0.253192338213616	3259
P49813	sp|P49813|TMOD1_MOUSE Tropomodulin-1 OS=Mus musculus GN=Tmod1 PE=2 SV=2;sp|P28289|TMOD1_HUMAN Tropomodulin-1 OS=Homo sapiens GN=TMOD1 PE=1 SV=1;sp|P28289-2|TMOD1_HUMAN Isoform 2 of Tropomodulin-1 OS=Homo sapiens GN=TMOD1	1,19	0,105179542126951	0,468689258180758	28,1438826384783	27,7704056984658	27,9843470437695	28,1529200921064	28,1351226080958	28,1372043839272	1398206966614,47	1666503821870,39	−0,253246697664004	0,00670086333431709	0,0003457588791028	1,19188636708452	0.253246697664004	0,839006156640674	-0.253246697664004	3260
P63101	sp|P63101|1433Z_MOUSE 14-3-3 protein zeta/delta OS=Mus musculus GN=Ywhaz PE=1 SV=1	1,19	0,00175192310753663	0,0352789063737934	36,084202935126	36,1442586580311	36,1442586580311	36,3824391571779	36,2026315719732	36,314388636062	4881554375990770	5818505218776100	−0,253307969215384	0,000959831617364902	0,0025009711703635	1,19193698781552	0.253307969215384	0,838970524635464	-0.253307969215384	3261
P00920	sp|P00920|CAH2_MOUSE Carbonic anhydrase 2 OS=Mus musculus GN=Ca2 PE=1 SV=4	1,19	0,438337419532361	0,779323755290791	32,1601216416315	32,704254865978	32,8262003127847	32,5562887960631	32,773886273776	32,8872840950895	138726516918660	165361161438837	−0,253376848530628	0,0108906785563881	0,0051378545043587	1,19199389642136	0.253376848530626	0,838930470199748	-0.253376848530626	3262
Q9D1C8	sp|Q9D1C8|VPS28_MOUSE Vacuolar protein sorting-associated protein 28 homolog OS=Mus musculus GN=Vps28 PE=2 SV=1	1,19	0,286019599499473	0,704836107321858	27,3351344193571	27,8187164630971	27,745330412391	27,9400277759159	27,7219629002009	27,7640796168976	1002041396505	1194429877692,57	−0,253380048252545	0,00943100669624495	0,0041596613197945	1,19199654012159	0.253380048252538	0,838928609556192	-0.253380048252539	3263
Q06185	sp|Q06185|ATP5I_MOUSE ATP synthase subunit e, mitochondrial OS=Mus musculus GN=Atp5i PE=1 SV=2	1,19	0,423522786254311	0,7711031271846	28,3845625088349	28,3251900002394	28,0782614029079	28,0535845608097	28,5454840142889	28,7164765416841	1880711547720,8	2242281609183,06	−0,253688884359986	0,0057474864074163	0,0121014946455955	1,19225173679634	0.253688884359986	0,838749040271533	-0.253688884359987	3264
Q3TDK6	sp|Q3TDK6|ROGDI_MOUSE Protein rogdi homolog OS=Mus musculus GN=Rogdi PE=2 SV=2;sp|Q3TDK6-2|ROGDI_MOUSE Isoform 2 of Protein rogdi homolog OS=Mus musculus GN=Rogdi;sp|Q3TDK6-3|ROGDI_MOUSE Isoform 3 of Protein rogdi homolog OS=Mus musculus GN=Rogdi	1,19	0,31915157523299	0,722907157640623	29,8638697170678	30,2028177448941	30,4385207524286	30,4559240288616	30,2511880637938	30,3275359257424	12646514229254,2	15087424116810,8	−0,254606726563519	0,00957515657742452	0,0034096167210124	1,19301048836922	0.254606726563519	0,838215598059786	-0.254606726563519	3265
Q9JJC6	sp|Q9JJC6|RIPL1_MOUSE RILP-like protein 1 OS=Mus musculus GN=Rilpl1 PE=2 SV=1	1,19	0,146422375519548	0,542097196152716	27,4140252679283	27,3368848768073	27,6889296233201	27,6754662525446	27,7483704429287	27,5472752363342	859783619541,636	1026357569865,04	−0,255487905335635	0,00673396101700352	0,003681030021489	1,19373938574476	0.255487905335633	0,837703783540752	-0.255487905335633	3266
Q9WUD1	sp|Q9WUD1|CHIP_MOUSE STIP1 homology and U box-containing protein 1 OS=Mus musculus GN=Stub1 PE=1 SV=1	1,19	0,372814838129024	0,744143356508855	27,4312593139902	27,9254032200445	28,0692982272581	28,0758284429146	27,9689280949635	27,913295506351	1194386269168,4	1426175280653,7	−0,255881818397453	0,0120343273260907	0,0029515843894471	1,19406536852327	0.255881818397454	0,83747508835025	-0.255881818397454	3267
O35386	sp|O35386|PAHX_MOUSE Phytanoyl-CoA dioxygenase, peroxisomal OS=Mus musculus GN=Phyh PE=1 SV=1	1,19	0,534980703338495	0,8300431463043	22,2752567028884	22,8991140368772	23,1133185411363	23,1361785433251	23,0064630885348	22,6774724465421	7685206573,24762	9177659256,69196	−0,256042204999886	0,0191278164856413	0,0103076229993908	1,19419812196586	0.256042204999886	0,837381990145678	-0.256042204999886	3268
Q8R395	sp|Q8R395|COMD5_MOUSE COMM domain-containing protein 5 OS=Mus musculus GN=Commd5 PE=2 SV=1	1,19	0,589990250892663	0,858561075424286	25,6547165375052	26,3577801409492	26,5982162339486	26,5989713295845	26,0494943656724	26,4947769969841	239919903891,386	286521925054,413	−0,256092690832681	0,0187107691079645	0,0110628466108823	1,19423991260068	0.25609269083268	0,837352687218694	-0.25609269083268	3269
Q80TM9-6	sp|Q80TM9-6|NISCH_MOUSE Isoform 6 of Nischarin OS=Mus musculus GN=Nisch;sp|Q80TM9|NISCH_MOUSE Nischarin OS=Mus musculus GN=Nisch PE=1 SV=2;sp|Q80TM9-4|NISCH_MOUSE Isoform 4 of Nischarin OS=Mus musculus GN=Nisch;sp|Q80TM9-3|NISCH_MOUSE Isoform 3 of Nisc	1,19	0,418376742883912	0,768519642229897	27,5035939037474	28,1810020312617	28,0224070708488	28,2250895728033	27,9479032340272	28,0674871197023	1311685851448,49	1566962229813,93	−0,256548169295444	0,012698117699387	0,0049510771684547	1,19461700992165	0.25654816929544	0,837088365304279	-0.25654816929544	3270
Q9R1P4	sp|Q9R1P4|PSA1_MOUSE Proteasome subunit alpha type-1 OS=Mus musculus GN=Psma1 PE=1 SV=1	1,19	0,382370371631072	0,749797813889279	30,7354901350717	31,138693565225	31,3898117118546	31,4086456757793	31,2024247921078	31,1864065676514	31720950282688,4	37894446180759,3	−0,256550430822061	0,0106180343337794	0,0039642255326111	1,19461888256986	0.256550430822059	0,837087053110029	-0.256550430822059	3271
P12023-2	sp|P12023-2|A4_MOUSE Isoform APP695 of Amyloid beta A4 protein OS=Mus musculus GN=App;sp|P08592-2|A4_RAT Isoform APP695 of Amyloid beta A4 protein OS=Rattus norvegicus GN=App;sp|P12023|A4_MOUSE Amyloid beta A4 protein OS=Mus musculus GN=App PE=1 SV=3;s	1,19	0,414248807709677	0,766833335315752	29,2784329903639	29,002551104323	28,7527330443723	29,194048580810 7	29,4570920219591	28,9160873320475	3975767973892,06	4749573679545,08	−0,256564459767003	0,00906399192427042	0,0092684202114735	1,19463049924805	0.256564459767002	0,837078913211609	-0.256564459767002	3272

Q60676	sp|Q60676|PPP5_MOUSE Serine/threonine-protein phosphatase 5 OS=Mus musculus GN=Ppp5c PE=1 SV=3;sp|P53042|PPP5_RAT Serine/ threonine-protein phosphatase 5 OS=Rattus norvegicus GN=Ppp5c PE=1 SV=1	1,19	0,366576441391561	0,739315231400715	30,2268071384523	30,8315955900058	30,7314349400907	30,8796473041202	30,6668670710889	30,7768742173066	19406177442507,4	23183521548962,5	−0,25658375735627	0,0105944034035966	0,0034577568481316	1,19464647881562	0.256583757356269	0,837067716460697	-0.256583757356269	3273
P62307	sp|P62307|RUXF_MOUSE Small nuclear ribonucleoprotein F OS=Mus musculus GN=Snrpf PE=2 SV=1	1,19	0,487573338262867	0,805347664619779	26,9359933509139	27,1164940541076	26,3976388083925	26,7178490875963	27,1273945621664	27,139097165185	442943800515,27	529278328699,171	−0,256902918801057	0,0139457226366198	0,0088869332065833	1,19491079474071	0.256902918801055	0,836882555920835	-0.256902918801055	3274
Q9D6S7	sp|Q9D6S7|RRFM_MOUSE Ribosome-recycling factor, mitochondrial OS=Mus musculus GN=Mrrf PE=1 SV=1	1,20	0,492378674627589	0,80770032675628	26,3276898796021	26,9530356848158	26,9731145224765	26,6350593510396	27,02680358158	27,12666654682	414890318288,51	495835386475,229	−0,257131245013685	0,0137181166645921	0,0096490473856799	1,19509992067453	0.257131245013685	0,836750118296039	-0.257131245013685	3275
Q9JLJ1	sp|Q9JLJ1|SELK_MOUSE Selenoprotein K OS=Mus musculus GN=Selk PE=1 SV=3	1,20	0,327652012714388	0,726927682919497	22,3211019379397	22,6586817635727	22,0749258890454	22,4030699429728	22,5528552230193	22,6338453533699	5095226018,6943	6090058389,89542	−0,257309916186417	0,0131117487427594	0,0051968327421062	1,19524793749111	0.257309916186415	0,836646497043158	-0.257309916186415	3276
Q6NXK8	sp|Q6NXK8|ASIC1_MOUSE Acid-sensing ion channel 1 OS=Mus musculus GN=Asic1 PE=1 SV=1;sp|P78348|ASIC1_HUMAN Acid-sensing ion channel 1 OS=Homo sapiens GN=ASIC1 PE=1 SV=3;sp|P78348-1|ASIC1_HUMAN Isoform 1 of Acid-sensing ion channel 1 OS=Homo sapiens GN=AS	1,20	0,703379562060295	0,915064618821753	23,4877467946751	24,6395384667734	24,6947800953991	24,9238571579815	24,3880879517679	24,0459906620689	34839644518,3241	41653250980,7375	−0,257699196745767	0,0280750378135277	0,0180953643576287	1,1955704932302	0.257699196745768	0,836420776242307	-0.257699196745768	3277
Q9D394	sp|Q9D394|RUFY3_MOUSE Protein RUFY3 OS=Mus musculus GN=Rufy3 PE=1 SV=1	1,20	0,217598470712034	0,647685277431762	30,4582833926756	30,8636183629237	30,8120815400983	31,0273688047328	30,836402611215	30,8064361728851	21765075618264,8	26024751874878,8	−0,257869376170194	0,00718473242848638	0,0038797339132121	1,19571153031232	0.257869376170188	0,836322118378167	-0.257869376170188	3278
Q6NZA9	sp|Q6NZA9|TAF9B_MOUSE Transcription initiation factor TFIID subunit 9B OS=Mus musculus GN=Taf9b PE=1 SV=2;sp|Q8VI33|TAF9_MOUSE Transcription initiation factor TFIID subunit 9 OS=Mus musculus GN=Taf9 PE=1 SV=1	1,20	0,253311678120523	0,674868697133729	22,3703619013105	22,5214498237658	22,7662261675712	22,9428230979447	22,6062886817837	22,6461403806329	6230231310,50924	7452018111,62139	−0,258345453308502	0,00885798711670513	0,0080889882135994	1,19610617009536	0.258345453308504	0,83604618469636	-0.258345453308504	3279
Q3UUG6-2	sp|Q3UUG6-2|TBC24_MOUSE Isoform 2 of TBC1 domain family member 24 OS=Mus musculus GN=Tbc1d24;sp|Q3UUG6|TBC24_MOUSE TBC1 domain family member 24 OS=Mus musculus GN=Tbc1d24 PE=2 SV=2	1,20	0,457884317817712	0,790957322506963	28,3770459552226	29,0002145179698	29,0238572486725	29,1591429707384	29,0156519772701	28,7636310588687	3219903718513,27	3851467404455,44	−0,258390666071876	0,0127360208625482	0,0069091080671267	1,19614365557296	0.258390666071878	0,836019984172379	-0.258390666071878	3280
Q91VF2	sp|Q91VF2|HNMT_MOUSE Histamine N-methyltransferase OS=Mus musculus GN=Hnmt PE=1 SV=1	1,20	0,429109396768322	0,774752407291731	27,8772178137753	28,1742570180425	28,6215713507648	28,5057267905654	28,2756209716639	28,429387167353	1810001387042,03	2165296262583,08	−0,258573629612738	0,0132756456537301	0,0041263765712229	1,19629536092328	0.258573629612734	0,835913966286903	-0.258573629612734	3281
Q8BUK6	sp|Q8BUK6|HOOK3_MOUSE Protein Hook homolog 3 OS=Mus musculus GN=Hook3 PE=1 SV=2	1,20	0,248690692602385	0,673464050610029	29,441616430095	29,3723329959481	28,9686502897513	29,5194704291796	29,4521853631682	29,3492574870933	5103086006283,91	6106069707953,87	−0,258874102905455	0,00872929484230183	0,0029118647826033	1,19654454195655	0.258874102905455	0,835739886761617	-0.258874102905455	3282
P14152	sp|P14152|MDHC_MOUSE Malate dehydrogenase, cytoplasmic OS=Mus musculus GN=Mdh1 PE=1 SV=3;sp|P40925|MDHC_HUMAN Malate dehydrogenase, cytoplasmic OS=Homo sapiens GN=MDH1 PE=1 SV=4;sp|P40925-3|MDHC_HUMAN Isoform 3 of Malate dehydrogenase, cytoplasmic OS=Ho	1,20	0,349004972191764	0,732843629012038	35,016095172406	35,4772270626791	35,5823748158424	35,6500093773241	35,3817684358028	35,5823748158424	2270021468032890	2716310379714020	−0,258942397393315	0,00851877891706579	0,0039255222626937	1,19660118548036	0.258942397393311	0,835700325333176	-0.258942397393311	3283
P63318	sp|P63318|KPCG_MOUSE Protein kinase C gamma type OS=Mus musculus GN=Prkcg PE=1 SV=1	1,20	0,266416288000339	0,685404159524725	32,4366499180038	32,1724065672547	31,8989697166028	32,4315691732345	32,3364067192113	32,2785243609912	93533620701688,9	111923130617034	−0,258951281285175	0,00835743545427655	0,0023888173291682	1,19660855398719	0.25895128128517	0,83569517923629	-0.25895128128517	3284
Q61120	sp|Q61120|SHC3_MOUSE SHC-transforming protein 3 OS=Mus musculus GN=Shc3 PE=1 SV=2	1,20	0,296232300676047	0,711969538636193	25,8903660723575	26,1988563938474	26,2127010125378	26,5473065949047	26,1944717091464	26,0995196682378	216453408843,024	259087753387,753	−0,25938430234037	0,00698201579434675	0,0089779888347292	1,19696776674766	0.259384302340368	0,835444385204413	-0.259384302340368	3285
P58044	sp|P58044|IDI1_MOUSE Isopentenyl-diphosphate Delta-isomerase 1 OS=Mus musculus GN=Idi1 PE=2 SV=1	1,20	0,330812973068402	0,72953198881961	27,6547108054017	27,9628273575294	28,2856963956054	28,0992599920198	28,1337438378691	28,2102303198389	1400352144694,46	1676525673653,56	−0,259684910764524	0,0112816223938125	0,0020177303369749	1,19721719997748	0.259684910764525	0,835270325233225	-0.259684910764525	3286
Q8R3B1	sp|Q8R3B1|PLCD1_MOUSE 1-phosphatidylinositol 4,5-bisphosphate phosphodiesterase delta-1 OS=Mus musculus GN=Plcd1 PE=2 SV=2	1,20	0,346983380952901	0,732538402858241	27,6333084505179	27,5285613999933	27,04657919394	27,5334727094064	27,7082701854407	27,5067207470531	795961255929,202	952943272806,31	−0,259692127249055	0,0114194241113725	0,0039684962743202	1,19722318857574	0.259692127249056	0,835266147149752	-0.259692127249056	3287
Q9CR57	sp|Q9CR57|RL14_MOUSE 60S ribosomal protein L14 OS=Mus musculus GN=Rpl14 PE=1 SV=3	1,20	0,261381313380139	0,682629333390362	26,4832460049278	26,5713233590086	26,9359933509139	26,8620165414284	26,6952330944468	26,9742992198464	380031851142,431	455130313675,412	−0,260159340874893	0,00900265018212731	0,0052308908563595	1,19761096946802	0.260159340874893	0,83499569183489	-0.260159340874893	3288
Q8R326	sp|Q8R326|PSPC1_MOUSE Paraspeckle component 1 OS=Mus musculus GN=Pspc1 PE=1 SV=1;sp|Q8R326-2|PSPC1_MOUSE Isoform 2 of Paraspeckle component 1 OS=Mus musculus GN=Pspc1	1,20	0,3809359895707 7	0,749797813889279	29,6285973016842	30,3249842800509	29,9739816452622	30,2457298105003	30,1058851287532	30,1173421092281	10431533573657	12494616858508,2	−0,260355393807789	0,0116159246595473	0,0025747051389888	1,19777372811809	0.260355393807789	0,834882229025987	-0.260355393807789	3289

Q9WTL8-3	sp|Q9WTL8-3|BMAL1_MOUSE Isoform 3 of Aryl hydrocarbon receptor nuclear translocator-like protein 1 OS=Mus musculus GN=Arntl;sp|Q9WTL8-2|BMAL1_MOUSE Isoform 2 of Aryl hydrocarbon receptor nuclear translocator-like protein 1 OS=Mus musculus GN=Arntl;sp|Q9	1,20	0,172135479232139	0,586461292712533	24,1940816395417	24,1331519049364	24,2920359350875	24,3792155998351	24,605749863721	24,1764319247229	32562369860,8174	39011896021,5906	−0,26070841514271€	0,00331142575901427	0,00880662929318505	1,1980668541123	0.260708415142715	0,834677961891322	-0,260708415142715	3290
O70152	sp|O70152|DPM1_MOUSE Dolichol-phosphate mannosyltransferase subunit 1 OS=Mus musculus GN=Dpm1 PE=2 SV=1	1,20	0,0952061253865726	0,452029919591687	26,5713233590086	26,4927145297487	26,8480719120113	26,8348651408146	26,8289507902745	26,7919179286233	370222470527,626	443772532200,809	−0,261427911315372	0,00700738564385258	0,00086793817852810	1,19866449912768	0.261427911315374	0,83426179779892	-0,261427911315374	3291
Q8K2T1	sp|Q8K2T1|NMRL1_MOUSE NmrA-like family domain-containing protein 1 OS=Mus musculus GN=Nmral1 PE=1 SV=1;sp|Q8K2T1-2|NMRL1_MOUSE Isoform 2 of NmrA-like family domain-containing protein 1 OS=Mus musculus GN=Nmral1;sp|Q8K2T1-3|NMRL1_MOUSE Isoform 3 of NmrA-	1,20	0,379740132847174	0,749083208602053	27,2596824498093	27,6333084505179	27,9088339896986	27,9512595440036	27,6817373259402	27,7129019603996	970044997628,158	1162932882085,34	−0,261644258524335	0,0118044082423354	0,00530696809030300	1,19884426488339	0.261644258524332	0,834136700897733	-0,261644258524332	3292
Q9D772	sp|Q9D772|F219A_MOUSE Protein FAM219A OS=Mus musculus GN=Fam219a PE=2 SV=2	1,20	0,539327784987788	0,831485169227261	23,8772170718654	24,6730051571631	24,752533055932	24,7672234633243	24,4317373548227	24,64930457552	40894105166,673	49049138767,9375	−0,26233490952852	0,0198099889052884	0,00691384012156656	1,19941831635701	0.262334909528523	0,833737476210382	-0,262334909528523	3293
P60879	sp|P60879|SNP25_MOUSE Synaptosomal-associated protein 25 OS=Mus musculus GN=Snap25 PE=1 SV=1	1,20	0,39027477948009	0,753319093070654	31,7896349241163	32,1601216416315	32,4800692515926	32,4536789640953	32,209626068356	32,3120570949621	91127001690996,5	109300349544884	−0,262347509722392	0,010749536494248	0,00379116219451535	1,19942879186909	0.262347509722397	0,833730194555099	-0,262347509722397	3294
Q9JK42	sp|Q9JK42|PDK2_MOUSE [Pyruvate dehydrogenase (acetyl-transferring)] kinase isozyme 2, mitochondrial OS=Mus musculus GN=Pdk2 PE=1 SV=2	1,20	0,122746281425216	0,49968194743413	28,6927585562291	28,4734319680636	28,6768520225877	28,7785417083211	28,9686502897513	28,6424177181693	2673334045744,55	3207575821847,94	−0,262843248250066	0,00427391648383836	0,0056902341941722	1,19984101012509	0.262843248250063	0,83344375759897	-0,262843248250063	3295
Q8BWR2	sp|Q8BWR2|PITH1_MOUSE PITH domain-containing protein 1 OS=Mus musculus GN=Pithd1 PE=2 SV=1;sp|Q8BWR2-2|PITH1_MOUSE Isoform 2 of PITH domain-containing protein 1 OS=Mus musculus GN=Pithd1	1,20	0,260163810409273	0,682439759823898	28,020951024883	28,4530620211488	28,3824394113629	28,5904583519206	28,2885021532674	28,525661745392	1924109063670,5	2309858577808,05	−0,263613947397855	0,0081949383806801	0,0055839776514067	1,20048214595574	0.263613947397854	0,832998644227125	-0,263613947397854	3296
Q9Z2Y3	sp|Q9Z2Y3|HOME1_MOUSE Homer protein homolog 1 OS=Mus musculus GN=Homer1 PE=1 SV=2;sp|Q9Z2Y3-3|HOME1_MOUSE Isoform 3 of Homer protein homolog 1 OS=Mus musculus GN=Homer1;sp|Q9Z2Y3-2|HOME1_MOUSE Isoform 2 of Homer protein homolog 1 OS=Mus musculus GN=Home	1,20	0,204996181216369	0,636399775345126	30,5960927494958	30,1282886884742	30,4732906815785	30,577500263056	30,6442031737676	30,5244583269409	15929980883995,3	19125696652549,4	−0,263767763229791	0,0079784191276691	0,0019620054928056	1,20061014459627	0.263767763229786	0,83290983713641	-0,263767763229786	3297
P46460	sp|P46460|NSF_MOUSE Vesicle-fusing ATPase OS=Mus musculus GN=Nsf PE=1 SV=2	1,20	0,250861342324151	0,673538059877323	34,8580740958575	34,7308080145928	34,3323405522909	34,8580740958575	34,7975818162301	34,8145794845827	1106975098990200	1329277290549800	−0,264019316208181	0,00791784721999306	0,00089590125078683	1,2008195051202	0.264019316208187	0,832764620941012	-0,264019316208187	3298
Q6KAU4	sp|Q6KAU4|MB12B_MOUSE Multivesicular body subunit 12B OS=Mus musculus GN=Mvb12b PE=2 SV=2	1,20	0,441470433005551	0,780917496263522	25,8761040310522	26,1808314726337	26,6335512707477	26,5548176288872	26,2458051503349	26,438944362597	246385263143,066	295870899938,371	−0,264051841025509	0,0145300512800696	0,0059102258223516	1,2008465772832	0.26405184102551	0,832745846902779	-0,26405184102551	3299
Q812E0	sp|Q812E0|CPEB2_MOUSE Cytoplasmic polyadenylation element-binding protein 2 OS=Mus musculus GN=Cpeb2 PE=1 SV=1	1,20	0,392249528362486	0,753881747531964	23,6248549139189	23,3895979642284	22,9274759558395	23,6104580044563	23,3953782803666	23,4856512423187	13339244984,8603	16020940889,6079	−0,264281867097294	0,0152177524362396	0,00459633982697005	1,20103805783545	0.264281867097294	0,832613082887841	-0,264281867097294	3300
Q8BFU3	sp|Q8BFU3|RN214_MOUSE RING finger protein 214 OS=Mus musculus GN=Rnf214 PE=2 SV=1;sp|Q8BFU3-4|RN214_MOUSE Isoform 4 of RING finger protein 214 OS=Mus musculus GN=Rnf214;sp|Q8BFU3-3|RN214_MOUSE Isoform 3 of RING finger protein 214 OS=Mus musculus GN=Rnf2	1,20	0,356777661494479	0,732843629012038	28,8185754978522	28,496957284198	28,1337438378691	28,6988938908549	28,6238583935333	28,6768520225877	2344497494114,71	2816552594997,52	−0,264651674993465	0,0120291117524976	0,0013453676804627	1,20134596094378	0.264651674993467	0,832399685444814	-0,264651674993467	3301
Q91YL2	sp|Q91YL2|RN126_MOUSE E3 ubiquitin-protein ligase RNF126 OS=Mus musculus GN=Rnf126 PE=1 SV=1	1,20	0,678944362994501	0,903732687282123	22,8884318811075	23,5452376736717	23,5469090051832	22,7429179730565	23,9648611641733	23,8239823554939	13512209485,3965	16237466636,8124	−0,265062961239107	0,0162769614264236	0,0284357942824187	1,20168849175712	0.265062961239115	0,832162417181668	-0,265062961239115	3302
Q8VHV1	sp|Q8VHV1|BAALC_MOUSE Brain and acute leukemia cytoplasmic protein OS=Mus musculus GN=Baalc PE=2 SV=3;sp|Q8VHV1-2|BAALC_MOUSE Isoform 2 of Brain and acute leukemia cytoplasmic protein OS=Mus musculus GN=Baalc;sp|Q8VHV1-3|BAALC_MOUSE Isoform 3 of Brain a	1,20	0,444395049290411	0,783647680158965	24,1600126179529	23,936307566344	24,0578211291045	23,8445866552698	24,2149220663738	24,6467589088478	27885716403,0871	33520483728,4932	−0,265516633203347	0,00465634457776175	0,0165657879308981	1,20206643587548	0.265516633203347	0,831900775327523	-0,265516633203347	3303

Q6RI63	sp|Q6RI63|F120B_MOUSE Constitutive coactivator of peroxisome proliferator-activated receptor gamma OS=Mus musculus GN=Fam120b PE=1 SV=1;sp|Q6RI63-2|F120B_MOUSE Isoform 2 of Constitutive coactivator of peroxisome proliferator-activated receptor gamma OS=M	1,20	0,424545302390664	0,7711031271846	25,3243138240262	25,4643078754517	24,7940826638887	25,5653482547121	25,3815883916897	25,1895497765879	87441408405,1634	105168410071,401	−0,26631287705049	0,01403259472130 1	0,00740437780318 26	1,2027300564979 3	0.266312877050499	0,831441764174219	-0.266312877050499	3304
Q07076	sp|Q07076|ANXA7_MOUSE Annexin A7 OS=Mus musculus GN=Anxa7 PE=2 SV=2	1,20	0,283058441560519	0,704458182186602	31,0315490815031	30,5433566106407	30,6061730946971	30,8724847418601	31,0631349310244	30,8001322897362	2210945060610 1,4	26599600714582,2	−0,26674141333720	0,0086434720590 7307	0,00439517035773 33	1,2030873669579 9	0.266741413337204	0,831194830454006	-0.266741413337204	3305
Q8CI61	sp|Q8CI61|BAG4_MOUSE BAG family molecular chaperone regulator 4 OS=Mus musculus GN=Bag4 PE=1 SV=2	1,20	0,399351763159438	0,754959455635438	25,2310892455359	25,8861812107947	25,879283072998	25,9154627291353	25,809182802241	25,8273556009734	140085367714,271	168578447823,795	−0,26711383411740	0,01465943579771 82	0,00219899969324 37	1,203397974923 7	0.2671138341174	0,830980291506147	-0.2671138341174	3306
P61021	sp|P61021|RAB5B_MOUSE Ras-related protein Rab-5B OS=Mus musculus GN=Rab5b PE=1 SV=1	1,20	0,348393417101993	0,732843629012038	29,1339128359005	29,5484224910215	29,6681665776184	29,7799328650923	29,4120521281494	29,714294747174	6166591077170,61	7421680130357,69	−0,26727264255112	0,00951954347263 213	0,00662086403675 78	1,2035304493974 9	0.267272642551126	0,830888824209325	-0.267272642551126	3307
O88630	sp|O88630|GOSR1_MOUSE Golgi SNAP receptor complex member 1 OS=Mus musculus GN=Gosr1 PE=1 SV=2	1,20	0,389796511556866	0,753319093070654	23,4099585585598	23,4022584280165	23,8870579935421	23,8772170718654	23,9139624756217	23,4643347891491	17169922417,8219	20667703723,0516	−0,26749458706519	0,01178384135000 78	0,01051127596983 03	1,2037156150221 8	0.2674945870652	0,830761009926397	-0.2674945870652	3308
Q99KJ0	sp|Q99KJ0|UBX2A_MOUSE UBX domain-containing protein 2A OS=Mus musculus GN=Ubxn2a PE=2 SV=1	1,20	0,27021232389245	0,690593321029184	22,7607929420049	23,2438889303564	23,2058538104204	23,2219874868566	23,1629885154419	23,3825283651281	10453247795,7995	12585796952,8458	−0,26784531975587	0,01164316399054 2	0,00488549702976 21	1,2040082851478 2	0.267845319755878	0,830559068683838	-0.267845319755878	3309
Q6ZPR5-5	sp|Q6ZPR5-5|NSMA3_MOUSE Isoform 5 of Sphingomyelin phosphodiesterase 4 OS=Mus musculus GN=Smpd4;sp|Q6ZPR5-3|NSMA3_MOUSE Isoform 3 of Sphingomyelin phosphodiesterase 4 OS=Mus musculus GN=Smpd4;sp|Q6ZPR5-2|NSMA3_MOUSE Isoform 2 of Sphingomyelin phosphodie	1,20	0,770600223919625	0,936218563920244	22,2152717627516	21,6695873010279	20,7310314969917	20,7897257103873	22,2381882530085	22,1461242459219	2259998952,92676	2722127016,73745	−0,26841228154726	0,03485532937439 63	0,03733086525606 11	1,204481538901 7	0.268412281547262	0,830232733090991	-0.268412281547262	3310
Q9D787	sp|Q9D787|PPIL2_MOUSE Peptidyl-prolyl cis-trans isomerase-like 2 OS=Mus musculus GN=Ppil2 PE=1 SV=2	1,20	0,412837612628299	0,766530246241387	22,2806686370197	22,9337468186366	22,8379813625276	22,847371833861	23,0551430075932	22,7080917274642	7105480841,69208	8558597666,21776	−0,26844214638680	0,01554722884453 11	0,00763650479033 63	1,2045064728060 9	0.268442146386805	0,83021554684579	-0.268442146386805	3311
P28663	sp|P28663|SNAB_MOUSE Beta-soluble NSF attachment protein OS=Mus musculus GN=Napb PE=1 SV=2	1,21	0,324809616198423	0,726844368394785	32,0369458698543	32,5562887960631	32,5868757685581	32,704254865978	32,4938038454955	32,5416809718635	117020487803162	141018644460163	−0,26912478068961	0,00954056674470 601	0,00338588292931 30	1,2050765392242 1	0.269124780689617	0,829822809963399	-0.269124780689617	3312
Q8VED9	sp|Q8VED9|LEGL_MOUSE Galectin-related protein OS=Mus musculus GN=Lgalsl PE=2 SV=1	1,21	0,0915952367322726	0,445606408925815	30,0107694072549	30,0323123257719	29,8451084763157	29,9691108569161	30,2825025623271	30,1964189422983	10295521077857,1	12407771430752	−0,26922716555267	0,00341862088639 129	0,00537034507888 4	1,2051620638646 2	0.269227165552673	0,829763921371101	-0.269227165552673	3313
Q3UYC0	sp|Q3UYC0|PPM1H_MOUSE Protein phosphatase 1H OS=Mus musculus GN=Ppm1h PE=1 SV=1;sp|Q3UYC0-2|PPM1H_MOUSE Isoform 2 of Protein phosphatase 1H OS=Mus musculus GN=Ppm1h	1,21	0,385912313898629	0,75210648037178	28,8830163528639	29,4939123683025	29,4859248540224	29,6207138314607	29,3880565211407	29,41407607155	5241442477773,79	6317104948595,74	−0,26929963537723	0,01196471298242 15	0,00432509776467 69	1,2052226033927 2	0.269299635377239	0,829722241505412	-0.269299635377239	3314
Q9Z2C4	sp|Q9Z2C4|MTMR1_MOUSE Myotubularin-related protein 1 OS=Mus musculus GN=Mtmr1 PE=1 SV=1	1,21	0,491394667153791	0,807429585179475	27,9268847299193	28,0603919717577	27,6419884256892	28,3076758523563	28,3022437834415	27,5796655021705	1278133222133,59	1540603049041,68	−0,26945696686886	0,00766661283667 5	0,01492195296520 48	1,2053540447606 5	0.269456966868856	0,829631762009456	-0.269456966868856	3315
Q9WV76	sp|Q9WV76|AP4B1_MOUSE AP-4 complex subunit beta-1 OS=Mus musculus GN=Ap4b1 PE=2 SV=2	1,21	0,582044338747047	0,853379506269256	23,0095077948973	22,5052388583284	21,8690502708892	22,579222249095	22,8085879999257	22,5567734350707	5685959010,89047	6854660078,59655	−0,26968142587145	0,02544375638230 53	0,00615314198732 01	1,2055415920986 5	0.269681425871458	0,829502695348044	-0.269681425871458	3316
P61294	sp|P61294|RAB6B_MOUSE Ras-related protein Rab-6B OS=Mus musculus GN=Rab6b PE=1 SV=1	1,21	0,178446701842345	0,597763527426129	29,3143252751957	29,656852932955	29,3941367297357	29,5501594072729	29,5484224910215	29,8286939050459	6197115013588,31	7473804339132,76	−0,27024605125467	0,00608445118111 317	0,00544205503000 06	1,2060134954321 6	0.270246051254679	0,829178117647566	-0.270246051254679	3317
Q3TXX3-2	sp|Q3TXX3-2|ZFY27_MOUSE Isoform 2 of Protrudin OS=Mus musculus GN=Zfyve27;sp|Q3TXX3|ZFY27_MOUSE Protrudin OS=Mus musculus GN=Zfyve27 PE=1 SV=2	1,21	0,125551789619895	0,506559620519737	23,9376551234288	23,6630592406412	23,9566725543437	24,1600126179529	24,0639628296117	23,8956506099416	2285560697 7,6144	27566727373,8852	−0,27037987258751	0,00688831382282 704	0,00556645720652 24	1,2061253678751 6	0.270379872587518	0,829101208410622	-0.270379872587518	3318
Q91XV3	sp|Q91XV3|BASP1_MOUSE Brain acid soluble protein 1 OS=Mus musculus GN=Basp1 PE=1 SV=3	1,21	0,460943304620801	0,792808510675944	31,7308789356197	32,3444630888764	32,390014054121	32,5833119363487	32,3444630888764	32,099937215965	92212673427289,3	111224383197101	−0,27043614898583	0,01144787137033 93	0,00747290369094 75	1,2061724171222 8	0.27043614898584	0,829068867605038	-0.27043614898584	3319
Q9R061	sp|Q9R061|NUBP2_MOUSE Cytosolic Fe-S cluster assembly factor NUBP2 OS=Mus musculus GN=Nubp2 PE=1 SV=1	1,21	0,225736812183009	0,652238880554131	24,9183070413901	25,1800505226162	25,4520364849735	25,3867743256002	25,3909018070988	25,3363157103993	86504708505,5772	104382786065,098	−0,27103324754354	0,01059747355749 96	0,00119796401229 59	1,2066717276825 2	0.27103324754354 1	0,828725805916205	-0.27103324754354 1	3320

Q3TUH1	sp|Q3TUH1|TAM41_MOUSE Phosphatidate cytidylyltransferase, mitochondrial OS=Mus musculus GN=Tamm41 PE=2 SV=2;sp|Q3TUH1-2|TAM41_MOUSE Isoform 2 of Phosphatidate cytidylyltransferase, mitochondrial OS=Mus musculus GN=Tamm41	1,21	0,735972781032228	0,924701393764781	23,7026990686974	22,8039538529234	21,8174060215298	23,1210148714882	23,0480487104139	22,7197042232794	7778943075,13923	9390107718,57185	−0,27156755826602	0,041405058381161	0,0093093333742061	1,20711870852761	0.271567558266023	0,828418939194271	-0.271567558266023	3321
Q7TMQ7	sp|Q7TMQ7|WDR91_MOUSE WD repeat-containing protein 91 OS=Mus musculus GN=Wdr91 PE=1 SV=1	1,21	0,45476775026638	0,788460969617685	26,0782885894433	25,8243737283388	25,272388648783	26,0846949698555	25,8115297761429	25,8459890590547	148673280779,219	179613160768,536	−0,27274767148769	0,0160168829401777	0,0057407256869233	1,20810652611657	0.272747671487699	0,827741576079781	-0.272747671487699	3322
P63321	sp|P63321|RALA_MOUSE Ras-related protein Ral-A OS=Mus musculus GN=Rala PE=1 SV=1	1,21	0,508252978688715	0,816124555116335	29,2505827592898	29,8563790555124	30,192477358805	30,0981104206294	29,8391442128287	29,9295036034965	8460925870668,04	10222232069738,6	−0,27282279976436	0,016036134338284	0,0043877813855922	1,20816943984542	0.272822799764359	0,827698472598304	-0.272822799764359	3323
Q6P5F7-2	sp|Q6P5F7-2|TTYH3_MOUSE Isoform 2 of Protein tweety homolog 3 OS=Mus musculus GN=Ttyh3;sp|Q6P5F7|TTYH3_MOUSE Protein tweety homolog 3 OS=Mus musculus GN=Ttyh3 PE=1 SV=1	1,21	0,359470110200403	0,733319024808822	24,961398664519	25,6245416244004	25,1577695205742	25,5441223434088	25,3243138240262	25,4434411168015	92262468426,7266	111500223026,118	−0,27323079940200	0,0134916451814824	0,0043256640629061	1,20851116307028	0.273230799402004	0,827464429421947	-0.273230799402004	3324
Q8BGZ4	sp|Q8BGZ4|CDC23_MOUSE Cell division cycle protein 23 homolog OS=Mus musculus GN=Cdc23 PE=1 SV=2;sp|Q8BGZ4-2|CDC23_MOUSE Isoform 2 of Cell division cycle protein 23 homolog OS=Mus musculus GN=Cdc23	1,21	0,260593976106723	0,682439759823898	26,5380610554905	27,0207057287637	26,9415112903228	27,1751206599197	26,9215383290468	26,9717991804572	450410859764,556	544328841817,531	−0,27323686838898	0,00964618815452003	0,0049687446782876	1,20851624692635	0.273236868388986	0,827460948533648	-0.273236868388987	3325
Q9QUR8	sp|Q9QUR8|SEM7A_MOUSE Semaphorin-7A OS=Mus musculus GN=Sema7a PE=1 SV=1	1,21	0,477976128251554	0,799905345588749	24,4689312575944	23,8100825694553	24,2617206040686	24,4554828608451	24,6363202435582	24,018150513668	31721001627,46	38348626120,4213	−0,27373656606531	0,0139327090286254	0,0130418641153581	1,20893490599061	0.273736566065311	0,82717439544902	-0.273736566065311	3326
O09172	sp|O09172|GSH0_MOUSE Glutamate--cysteine ligase regulatory subunit OS=Mus musculus GN=Gclm PE=2 SV=1	1,21	0,414686986408584	0,76733152519713	26,8202978882426	27,0220262352562	27,5950102812602	27,3567816774972	27,3032177671707	27,3465665285104	615547612212,084	744160065960,293	−0,27374252029201	0,014805574792145	0,0010403568239874	1,20893989546319	0.27374252029201	0,827170981578751	-0.27374252029201	3327
Q9Z275	sp|Q9Z275|RLBP1_MOUSE Retinaldehyde-binding protein 1 OS=Mus musculus GN=Rlbp1 PE=2 SV=3	1,21	0,278593141552133	0,699221280539289	27,6721550228499	28,0407745775257	28,197075208073	28,3022437834415	28,1529200921064	28,0241091157644	1403515957980,96	1696787144368,88	−0,27376012811778	0,0096360964459548	0,0049429880710421	1,20895465044075	0.273760128117782	0,827160886171729	-0.273760128117782	3328
O08749	sp|O08749|DLDH_MOUSE Dihydrolipoyl dehydrogenase, mitochondrial OS=Mus musculus GN=Dld PE=1 SV=2	1,21	0,394474377847483	0,753881747531964	32,3364067192113	32,9774514497747	32,9992079572545	33,042314781574	32,8687527944652	32,9720957537316	170715926430079	206444854297115	−0,27415880278598	0,0114901841401607	0,0026487686231619	1,20928877940202	0.274158802785984	0,826932339928342	-0.274158802785984	3329
Q99MX7	sp|Q99MX7|CECR6_MOUSE Cat eye syndrome critical region protein 6 homolog OS=Mus musculus GN=Cecr6 PE=2 SV=1	1,21	0,43458749537806	0,777519483446506	21,4673136439321	21,5152196899429	21,711461611698	22,1923188032946	21,4030055681932	21,6695873010279	2319610178,17447	2805844941,57769	−0,27455291024035	0,00599900237202003	0,0184566584181396	1,20961917134968	0.27455291024035	0,82670647397578	-0.27455291024035	3330
Q8VE70	sp|Q8VE70|PDC10_MOUSE Programmed cell death protein 10 OS=Mus musculus GN=Pdcd10 PE=1 SV=1	1,21	0,2964882716702	0,711969538636193	28,5437339219033	28,9794072842181	29,1231989742319	29,1687376261914	29,0665390787366	28,9822197969154	3494157463490,58	4226937425792,38	−0,27466813086212	0,0104471521308202	0,0032127141476273	1,20971578126012	0.274668130862125	0,826640451824424	-0.274668130862125	3331
P21661	sp|P21661|NEC2_MOUSE Neuroendocrine convertase 2 OS=Mus musculus GN=Pcsk2 PE=1 SV=1	1,21	0,436653711125729	0,778450481049573	25,1963348706422	25,8804365348968	25,6555739842145	25,9217837339592	25,4898256936456	25,8919891587445	128275799088,614	155182269529,922	−0,27471471794025	0,0136322501137754	0,0093624567569946	1,20975484567218	0.274714717940255	0,826613758628398	-0.274714717940254	3332
P42125	sp|P42125|ECI1_MOUSE Enoyl-CoA delta isomerase 1, mitochondrial OS=Mus musculus GN=Eci1 PE=1 SV=2	1,21	0,390129662446527	0,753319093070654	28,706063719518	28,7785417083211	29,3758840404762	29,2598728637706	29,0383137035681	29,1339128359005	3752699221702,21	4540385945601,58	−0,27488627281249	0,0126958425596919	0,0038129843671502	1,20989870953262	0.274886272812496	0,826515469535706	-0.274886272812496	3333
P97470	sp|P97470|PP4C_MOUSE Serine/threonine-protein phosphatase 4 catalytic subunit OS=Mus musculus GN=Ppp4c PE=1 SV=2	1,21	0,35382016234942	0,732843629012038	25,8351165872011	25,979047873499	26,4947769969841	26,341020672108	26,1971184130789	26,3439130720978	216960356725,12	262631405409,949	−0,27560798806472	0,0132878933002224	0,0031919592763778	1,21050411869802	0.275607988064728	0,826102104531102	-0.275607988064728	3334
Q80VP9	sp|Q80VP9|ASPH2_MOUSE Aspartate beta-hydroxylase domain-containing protein 2 OS=Mus musculus GN=Asphd2 PE=2 SV=1	1,21	0,0655790172144932	0,382600461615147	25,9984600276786	25,9874283403252	26,1555786458727	26,2819309123858	26,3667324253046	26,0680641034151	205180449690,667	248549820434,638	−0,27664178852763	0,0036110142125087	0,0058657854656889	1,2113718476071	0.276641788527631	0,825510351734992	-0.276641788527631	3335
Q3SXD3	sp|Q3SXD3|HDDC2_MOUSE HD domain-containing protein 2 OS=Mus musculus GN=Hddc2 PE=2 SV=1	1,21	0,0852732360749962	0,433918406439919	25,8344529325275	26,0052225567599	26,0312420351845	26,2965204560717	26,1720458506377	25,9776537154619	187486467825,919	227119013910,54	−0,27666202014736	0,0041183409883942	0,0061458354530584	1,21138883538741	0.27666202014736	0,825498775279854	-0.27666202014736	3336
Q9ER00	sp|Q9ER00|STX12_MOUSE Syntaxin-12 OS=Mus musculus GN=Stx12 PE=1 SV=1	1,21	0,385824470125533	0,75210648037178	28,7823143927038	29,3261521639233	29,4671293506286	29,5156147134624	29,2631523510523	29,3723329959481	4762840273907,64	5770039372916,36	−0,27675899594764	0,0123876302618715	0,0043090028252406	1,21147026586772	0.276758995947644	0,825443288353221	-0.276758995947644	3337
Q9SS47	sp|Q9SS47|PUM4_ARATH Pumilio homolog 4 OS=Arabidopsis thaliana GN=APUM4 PE=1 SV=2	1,21	0,303549623813015	0,716532404521184	27,683736916332	27,3684215764684	27,0441476199735	27,6006069372341	27,5035939037474	27,5687786150749	766756694511,076	929265028474,507	−0,27732125752226	0,0116864614244073	0,0017945296355725	1,2119425042217	0.277321257522264	0,825121650999601	-0.277321257522264	3338

Q5RJI5	sp|Q5RJI5|BRSK1_MOUSE Serine/threonine-protein kinase BRSK1 OS=Mus musculus GN=Brsk1 PE=1 SV=1	1,21	0,0346680321283947	0,272246017008276	29,0169853290817	29,0836947794638	28,8118954040246	29,2120692784127	29,1940485808107	29,0836947794638	3818422534552,49	4628578324643,05	−0,277592379755385	0,00488936745167722	0,0023831847221566	1,21217028308406	0.277592379755384	0,824966602427963	-0.277592379755384	3339
Q9D051	sp|Q9D051|ODPB_MOUSE Pyruvate dehydrogenase E1 component subunit beta, mitochondrial OS=Mus musculus GN=Pdhb PE=1 SV=1	1,21	0,180205626590219	0,598356502628386	32,6953467176655	32,9187789512132	33,1377845162753	33,1717080697328	32,9774514497747	33,1816073307961	197607408915551	239663175176903	−0,278371213398988	0,00672055628066735	0,00347681516573487	1,21282484544557	0.278371213398988	0,824521367413624	-0.278371213398988	3340
Q8C0C7	sp|Q8C0C7|SYFA_MOUSE Phenylalanine--tRNA ligase alpha subunit OS=Mus musculus GN=Farsa PE=2 SV=1	1,21	0,38241505599332	0,749797813889279	29,2410664809566	29,8306347317633	29,6878677659393	30,0419786001201	29,6742040431969	29,6230833170184	7067465131672,13	8573998593928,19	−0,27877532022747	0,0103956911034617	0,0076738699406751	1,21316461194901	0.27877532022747	0,824290446779064	-0.278775320227469	3341
Q8BYN3	sp|Q8BYN3|ITPK1_MOUSE Inositol-tetrakisphosphate 1-kinase OS=Mus musculus GN=Itpk1 PE=2 SV=1	1,21	0,458610753125478	0,790967157530661	24,1307029648063	23,5843529479607	23,779469321038	24,4391865491359	23,8296632028795	23,8072085417944	22381675685,5158	27169280119,1564	−0,279658287260613	0,0116177094834044	0,0149245004011275	1,21390732762422	0.279658287260613	0,823786113851983	-0.279658287260613	3342
Q9Z0P4	sp|Q9Z0P4|PALM_MOUSE Paralemmin-1 OS=Mus musculus GN=Palm PE=1 SV=1	1,21	0,12844108402342	0,512962462222669	29,7669313833442	30,161244964118	30,0707031025332	30,102498361754	30,2374046939875	30,2413049199915	10682483750511	12970984170511,7	−0,280040825416666	0,00688482846051327	0,0026177007973762	1,21422924419531	0.280040825416674	0,82356771160022	-0.280040825416674	3343
Q03059	sp|Q03059|CLAT_MOUSE Choline O-acetyltransferase OS=Mus musculus GN=Chat PE=2 SV=2;sp|P32738|CLAT_RAT Choline O-acetyltransferase OS=Rattus norvegicus GN=Chat PE=1 SV=2	1,21	0,351438983042536	0,732843629012038	24,4150238637808	24,5607808475858	24,9472029184827	25,1021464688603	24,6675378862814	24,7358047995479	50286502761,5322	61062456328,9566	−0,280114402432159	0,0111606495609774	0,0094108642997218	1,21429117110263	0.280114402432159	0,823525710964328	-0.280114402432159	3344
Q91WS0	sp|Q91WS0|CISD1_MOUSE CDGSH iron-sulfur domain-containing protein 1 OS=Mus musculus GN=Cisd1 PE=1 SV=1	1,21	0,422617609516832	0,7711031271846	28,5486023400049	28,5243734839213	28,1083623316671	28,3270681210585	28,9711760736878	28,4660063045051	2144182303624,33	2604035569517,36	−0,28032158248942	0,00871582140965107	0,0118567923501881	1,21446556345313	0.280321582489423	0,823407455997899	-0.280321582489423	3345
Q8VDD8	sp|Q8VDD8|WASH1_MOUSE WAS protein family homolog 1 OS=Mus musculus GN=Wash1 PE=1 SV=1	1,21	0,423477916080966	0,7711031271846	24,295877056704	25,0087957507153	24,9839800051415	24,9190543270454	24,8661841819719	25,0866146564925	56804663936,0998	68993931507,0866	−0,280460085681392	0,0163401966191186	0,0046111039009526	1,21458216150524	0.28046008568139	0,823328410126407	-0.28046008568139	3346
Q80Y98	sp|Q80Y98|DDHD2_MOUSE Phospholipase DDHD2 OS=Mus musculus GN=Ddhd2 PE=2 SV=3;sp|Q80Y98-2|DDHD2_MOUSE Isoform 2 of Phospholipase DDHD2 OS=Mus musculus GN=Ddhd2;sp|Q80Y98-3|DDHD2_MOUSE Isoform 3 of Phospholipase DDHD2 OS=Mus musculus GN=Ddhd2	1,21	0,0748725306436975	0,406514852382467	26,9782391770371	26,8591980973836	26,613068974371	27,0421427414941	27,0158477199576	26,9761562920847	442999915507,652	538138743376,984	−0,280671753952384	0,00694468372248454	0,0012298183547929	1,21476037475156	0.280671753952385	0,823207622494699	-0.280671753952385	3347
P19253	sp|P19253|RL13A_MOUSE 60S ribosomal protein L13a OS=Mus musculus GN=Rpl13a PE=1 SV=4;sp|P40429|RL13A_HUMAN 60S ribosomal protein L13a OS=Homo sapiens GN=RPL13A PE=1 SV=2	1,21	0,287210895622714	0,706202738478603	25,8503583907544	26,3399692238663	26,4269514236666	26,4441071435333	26,3309093895965	26,4261270803001	240445593736,174	292105596145,284	−0,28077950903635	0,0118616696799602	0,0023042210583560	1,2148511087535	0.280779509036346	0,823146139304308	-0.280779509036346	3348
P62911	sp|P62911|RL32_MOUSE 60S ribosomal protein L32 OS=Mus musculus GN=Rpl32 PE=1 SV=2	1,21	0,703384105545075	0,915064618821753	24,4199170205648	22,8730078302888	23,2438889303564	23,3986660676142	23,6149347963874	24,1071874390443	16264835131,1777	19760077164,581	−0,280832382219349	0,0343489709832715	0,015316561914201	1,2148956325234	0.28083238221935	0,82311597245843	-0.28083238221935	3349
Q9CWG8-2	sp|Q9CWG8-2|NDUF7_MOUSE Isoform 2 of NADH dehydrogenase [ubiquinone] complex I, assembly factor 7 OS=Mus musculus GN=Ndufaf7;sp|Q9CWG8|NDUF7_MOUSE NADH dehydrogenase [ubiquinone] complex I, assembly factor 7 OS=Mus musculus GN=Ndufaf7 PE=2 SV=4	1,21	0,0727465326934913	0,402678673043606	26,6568132883569	26,4656131249857	26,5328938470056	26,7069466343535	26,9234814664797	26,6089362359476	339851100904,062	412893190958,776	−0,280865830909949	0,00365279427431999	0,0060176714232593	1,21492380004187	0.28086583090995	0,823096888846475	-0.28086583090995	3350
Q8CHW4	sp|Q8CHW4|EI2BE_MOUSE Translation initiation factor eIF-2B subunit epsilon OS=Mus musculus GN=Eif2b5 PE=1 SV=1	1,22	0,110131193045705	0,475628435047057	27,4384765453677	27,1640538722618	27,173042131448	27,6170252356209	27,3567816774972	27,386252220826	689011764820,749	837220274826,553	−0,281078632484409	0,00571960911923328	0,0051909190175527	1,2151030179352	0.281078632484408	0,822975488695005	-0.281078632484408	3351
P54103	sp|P54103|DNJC2_MOUSE DnaJ homolog subfamily C member 2 OS=Mus musculus GN=Dnajc2 PE=1 SV=2	1,22	0,354620194207361	0,732843629012038	24,752533055932	24,1086932997909	24,1446543239673	24,5032761075892	24,5823748158982	24,5047987626687	37041085775,8185	45009971694,7545	−0,281118268895172	0,0148667648697891	0,0018440374539338	1,21513640197173	0.281118268895172	0,822952878686998	-0.281118268895172	3352
P46638	sp|P46638|RB11B_MOUSE Ras-related protein Rab-11B OS=Mus musculus GN=Rab11b PE=1 SV=3;sp|P62492|RB11A_MOUSE Ras-related protein Rab-11A OS=Mus musculus GN=Rab11a PE=1 SV=3	1,22	0,318837740051339	0,722907157640623	31,2146660379869	31,7107286381385	31,8397012538833	31,8676070981556	31,7211883042871	31,7608767264179	52318194307097,9	63573894803803,7	−0,281121727701809	0,0104467329631171	0,0023825237928474	1,21513931521866	0.28112172770181	0,822950905691052	-0.28112172770181	3353
Q9JI75	sp|Q9JI75|NQO2_MOUSE Ribosyldihydronicotinamide dehydrogenase [quinone] OS=Mus musculus GN=Nqo2 PE=2 SV=3	1,22	0,449838558091949	0,786343703431612	26,5548176288872	26,8535256285277	27,3642301077782	27,3269456084881	27,024059925362	27,0068439722442	493205185282,714	599452855545,628	−0,281458328676231	0,0152022081430355	0,0066390706955726	1,21542285732866	0.281458328676234	0,82275892210705	-0.281458328676234	3354
P56695	sp|P56695|WFS1_MOUSE Wolframin OS=Mus musculus GN=Wfs1 PE=1 SV=1	1,22	0,181585854382076	0,601053022412213	28,1645775605123	27,8449372025876	27,6856496210637	28,1272472497798	28,1372043839272	28,0160964439985	1306519866982,67	1588031041174,98	−0,281510050563449	0,00874233282824849	0,0023931469128487	1,21546643208912	0.28151005056345	0,822729426004154	-0.28151005056345	3355
Q9CY27	sp|Q9CY27|TECR_MOUSE Very-long-chain enoyl-CoA reductase OS=Mus musculus GN=Tecr PE=1 SV=1	1,22	0,188255344563836	0,613397297160539	26,2339091105729	26,0372835360317	26,2070979591574	26,1555786458727	26,6147061508255	26,2965204560717	229559984328,883	279313809219,861	−0,283015720909572	0,00407608895195987	0,0089239720691971	1,21673561721322	0.283015720909574	0,821871231394025	-0.283015720909574	3356
Q9D2R0	sp|Q9D2R0|AACS_MOUSE Acetoacetyl-CoA synthetase OS=Mus musculus GN=Aacs PE=2 SV=1	1,22	0,00726656031581776	0,096724097576237	27,4741157907615	27,5472752363342	27,3497540213979	27,7251228271318	27,6642808277829	27,570534038433	840319782253,856	1022541759329,28	−0,283149409614332	0,00363697347439662	0,0028161496303050	1,2168483723978	0.283149409614331	0,821795075445183	-0.283149409614331	3357

Q9QYK9	sp|Q9QYK9|KCC1B_MOUSE Calcium/calmodulin-dependent protein kinase type 1B OS=Mus musculus GN=Pnck PE=2 SV=1;sp|Q6P2M8|KCC1B_HUMAN Calcium/calmodulin-dependent protein kinase type 1B OS=Homo sapiens GN=PNCK PE=2 SV=2;sp|Q6P2M8-6|KCC1B_HUMAN Isoform 5 of	1,22	0,330714844566213	0,72953198881961	24,1461515325522	23,6005714162421	23,8956506099416	24,086565009139	24,2905635733946	23,8542629495858	23512339049,7699	28613100539,2259	−0,28325776973165	0,011435694105121	0,0090668306926034	1,21693977271504	0.283257769731656	0,821733353137898	-0.283257769731656	3358
Q9WU79	sp|Q9WU79|PROD_MOUSE Proline dehydrogenase 1, mitochondrial OS=Mus musculus GN=Prodh PE=1 SV=2	1,22	0,240975351653368	0,664601444032504	27,3802624477312	27,8762541482515	27,8373225183337	27,9056879546371	27,7989733088988	27,9786139581145	106921557216,58	1301352304402,87	−0,28345884965718	0,0099577824391441	0,0032389404497555	1,21710939914735	0.283458849657176	0,821618829581428	-0.283458849657176	3359
Q8BKX1-4	sp|Q8BKX1-4|BAIP2_MOUSE Isoform 4 of Brain-specific angiogenesis inhibitor 1-associated protein 2 OS=Mus musculus GN=Baiap2	1,22	0,0874878251322806	0,440251927134614	25,1499790121567	25,4761717842147	25,4069322364685	25,4876194191801	25,4779842302653	25,6571152896275	101605246643,219	123672936990,094	−0,28355493261745	0,00678110351121575	0,0039448548483176	1,21719046088599	0.283554932617456	0,821564111891007	-0.283554932617456	3360
Q9R099	sp|Q9R099|TBL2_MOUSE Transducin beta-like protein 2 OS=Mus musculus GN=Tbl2 PE=2 SV=2;sp|Q9Y4P3|TBL2_HUMAN Transducin beta-like protein 2 OS=Homo sapiens GN=TBL2 PE=1 SV=1	1,22	0,174941734579503	0,590462219359101	24,4846098063473	24,2355146437319	24,3684748551525	24,6103867125833	24,3206206984587	24,7472989515349	38076625812,4458	46347604936,4798	−0,2835891490697	0,0051160749725837	0,0088705032618952	1,21721932938003	0.283589149069732	0,821544627055281	-0.283589149069732	3361
P54731	sp|P54731|FAF1_MOUSE FAS-associated factor 1 OS=Mus musculus GN=Faf1 PE=2 SV=2	1,22	0,16502003449942	0,573087939244734	27,0337631233805	26,8427056892879	26,882581127589	27,235518194489	26,8583139812733	27,2551673122194	490986914375,385	597687071819,448	−0,28370576229212	0,00374401589066857	0,0082484250398241	1,21731772134865	0.283705762292125	0,821478224183013	-0.283705762292125	3362
Q9QXN3-2	sp|Q9QXN3-2|TRIP4_MOUSE Isoform 2 of Activating signal cointegrator 1 OS=Mus musculus GN=Trip4;sp|Q9QXN3|TRIP4_MOUSE Activating signal cointegrator 1 OS=Mus musculus GN=Trip4 PE=1 SV=2	1,22	0,601560914038653	0,862899589595385	21,2326685501382	21,3395865968786	22,0134108257316	22,1096485360386	21,1647787321785	21,9012572974974	2237344087,78701	2723621290,46007	−0,28373896603483	0,0196616331939702	0,0228500070434594	1,21734573833658	0.28373896603484	0,821459318012998	-0.28373896603484	3363
P39053-3	sp|P39053-3|DYN1_MOUSE Isoform 3 of Dynamin-1 OS=Mus musculus GN=Dnm1	1,22	0,333360624060573	0,72953198881961	32,033953090713	31,4680190706971	31,42290936091	31,8280562746036	31,9339369527224	31,7533387554372	55180302905315,2	67183177295574,4	−0,28394665039070	0,0107615207178572	0,0028502054316077	1,21752099496183	0.283946650390703	0,821341072669841	-0.283946650390703	3364
P35288	sp|P35288|RAB23_MOUSE Ras-related protein Rab-23 OS=Mus musculus GN=Rab23 PE=1 SV=2	1,22	0,279803399854399	0,699221280539289	27,2481132134366	27,5687786150749	27,8568791263886	27,8507513155764	27,7199698313342	27,6943403199442	929510231551,254	1132015161251,53	−0,28435062977385	0,0110504729110561	0,0030225117754553	1,21786196948291	0.284350629773853	0,821111115264224	-0.284350629773853	3365
P21995	sp|P21995|EMB_MOUSE Embigin OS=Mus musculus GN=Emb PE=1 SV=2	1,22	0,310992229280418	0,719371512809526	24,6655484483854	25,0371457729701	25,3172002676571	25,2995911794629	25,149119368486	25,1630860770961	72483986339,756	88293489332,8767	−0,28464475878186	0,0130723644351644	0,0032985691404806	1,21811028602947	0.284644758781868	0,820943728551524	-0.284644758781869	3366
P48453-2	sp|P48453-2|PP2BB_MOUSE Isoform 1 of Serine/threonine-protein phosphatase 2B catalytic subunit beta isoform OS=Mus musculus GN=Ppp3cb;sp|P48453|PP2BB_MOUSE Serine/threonine-protein phosphatase 2B catalytic subunit beta isoform OS=Mus musculus GN=Ppp3cb P	1,22	0,342435667939915	0,729891123744941	30,1853711148321	30,7896398570902	30,7747464720785	30,8893105450994	30,7710464061561	30,6814880843035	19148633692000,2	23326589634046,7	−0,28473394403762	0,0112694301542081	0,0033865836179077	1,21818559011821	0.28473394403763	0,820892980603196	-0.28473394403763	3367
Q9Z2U1	sp|Q9Z2U1|PSA5_MOUSE Proteasome subunit alpha type-5 OS=Mus musculus GN=Psma5 PE=1 SV=1	1,22	0,272449856415254	0,693189404859215	30,4856864089267	30,6477397566522	31,0273688047328	31,0315490815031	30,7592274156069	30,9631543867588	21960466820409,4	26761274076632,9	−0,28523808035396	0,00905033116210065	0,0045821390850147	1,21861134808673	0.285238080353969	0,820606177326385	-0.285238080353968	3368
Q8BK75-2	sp|Q8BK75-2|ELP6_MOUSE Isoform 2 of Elongator complex protein 6 OS=Mus musculus GN=Elp6;sp|Q8BK75|ELP6_MOUSE Elongator complex protein 6 OS=Mus musculus GN=Elp6 PE=2 SV=1	1,22	0,591993974571139	0,859859408185088	24,4879210503984	25,1593816694031	25,5141697482735	25,5630102279109	24,8413906379364	25,35089230623	75986699552,1756	92619393590,448	−0,28556739494708	0,0208033625949118	0,0146873701575124	1,21888954430573	0.285567394947085	0,820418884280109	-0.285567394947085	3369
Q8CFI2	sp|Q8CFI2|UB2R1_MOUSE Ubiquitin-conjugating enzyme E2 R1 OS=Mus musculus GN=Cdc34 PE=1 SV=1	1,22	0,837659551154903	0,959408706205426	26,3230679595192	26,3230679595192	24,7267151368333	26,7568583343213	24,3708201043853	26,8407111037321	158806187952,772	193678069259,542	−0,28639347374285	0,0357355862937171	0,0539608251056109	1,21958767322808	0.286393473742854	0,819949251662359	-0.286393473742854	3370
Q9D0L8	sp|Q9D0L8|MCES_MOUSE mRNA cap guanine-N7 methyltransferase OS=Mus musculus GN=Rnmt PE=1 SV=1;sp|Q9D0L8-3|MCES_MOUSE Isoform 3 of mRNA cap guanine-N7 methyltransferase OS=Mus musculus GN=Rnmt;sp|Q9D0L8-2|MCES_MOUSE Isoform 2 of mRNA cap guanine-N7 methyl	1,22	0,186059855003117	0,609765835277365	28,4283424016431	28,7725746006029	28,4283424016431	28,5498933163116	28,8639896558021	28,8118954040246	2489458836294,52	3037105760468,64	−0,28686498768668	0,00696289635517286	0,0058566956583266	1,21998633445543	0.28686498768669	0,81968131261599	-0.28686498768669	3371

Q920M5	sp|Q920M5|CORO6_MOUSE Coronin-6 OS=Mus musculus GN=Coro6 PE=2 SV=1;sp|Q920M5-3|CORO6_MOUSE Isoform C of Coronin-6 OS=Mus musculus GN=Coro6;sp|Q920M5-2|CORO6_MOUSE Isoform B of Coronin-6 OS=Mus musculus GN=Coro6;sp|Q920M5-4|CORO6_MOUSE Isoform D of Coro	1,22	0,376719023250151	0,74727992066734	22,3849401581905	23,1009237110647	22,6399360793508	23,0698661387242	22,8052947352916	22,847371833861	7281480055,866	8883939230,51173	−0,28696779751196	0,015980593310051	0,0062062197139148	1,22007327663485	0.286967797511965	0,819622902288423	-0.286967797511965	3372
Q9CQH3	sp|Q9CQH3|NDUB5_MOUSE NADH dehydrogenase [ubiquinone] 1 beta subcomplex subunit 5, mitochondrial OS=Mus musculus GN=Ndufb5 PE=1 SV=1	1,22	0,391122496156128	0,753845014884359	26,5328938470056	26,4967677565846	26,2722099307776	26,2722099307776	26,6033375672616	27,024059925362	302079836338,861	368682794013,331	−0,287450200956606	0,00534301554348538	0,0141482671984976	1,22048130878804	0.287450200956607	0,819348885394251	-0.287450200956607	3373
Q8R104	sp|Q8R104|SIR3_MOUSE NAD-dependent protein deacetylase sirtuin-3 OS=Mus musculus GN=Sirt3 PE=1 SV=2;sp|Q8R104-2|SIR3_MOUSE Isoform S of NAD-dependent protein deacetylase sirtuin-3 OS=Mus musculus GN=Sirt3	1,22	0,218648833550157	0,648658206198798	23,9338055309889	24,1582085472173	24,333802902532	24,3866384851643	24,1261151702096	24,511216945845	30528881092,5299	37265117327,5321	−0,287651087319164	0,0083048169512847	0,0080730761548353	1,22065126509502	0.287651087319162	0,819234804071706	-0.287651087319162	3374
Q8BIP0	sp|Q8BIP0|SYDM_MOUSE Aspartate--tRNA ligase, mitochondrial OS=Mus musculus GN=Dars2 PE=2 SV=1	1,22	0,334098604122588	0,72953198881961	25,7876482760269	26,4159750442456	26,3652422189801	26,4441071435333	26,3000797338373	26,4229344891842	236596464731,902	288811613330,052	−0,287700238410596	0,0133275039020422	0,0029469117745790	1,22069285209869	0.287700238410594	0,819206894085388	-0.287700238410594	3375
P35802	sp|P35802|GPM6A_MOUSE Neuronal membrane glycoprotein M6-a OS=Mus musculus GN=Gpm6a PE=1 SV=1	1,22	0,405105264845927	0,759526281549977	31,8110328188897	32,4866102924521	32,4715364843887	32,5334327252068	32,2785243609912	32,5562887960631	102040733606429	124594049055701	−0,288089986913825	0,0119593977306176	0,0047508343522491	1,22102267057644	0.288089986913826	0,818985612714221	-0.288089986913826	3376
Q9CZR8	sp|Q9CZR8|EFTS_MOUSE Elongation factor Ts, mitochondrial OS=Mus musculus GN=Tsfm PE=1 SV=1	1,22	0,228239371027803	0,652923723647267	28,2936711973808	28,5614572625613	28,7707322599102	28,8042521530299	28,8632336136022	28,5598383543679	2486640138570,65	3038671027225,83	−0,289242755370755	0,00837811644224271	0,0055967814059392	1,22199870423249	0.289242755370756	0,818331473295693	-0.289242755370755	3377
O88737-2	sp|O88737-2|BSN_MOUSE Isoform 2 of Protein bassoon OS=Mus musculus GN=Bsn;sp|O88737|BSN_MOUSE Protein bassoon OS=Mus musculus GN=Bsn PE=1 SV=4	1,22	0,0784688671600484	0,417369905631425	26,2649632160102	26,1388382616424	26,133625476344	26,5758572324515	26,2127010125378	26,3515957296419	234129991665,683	286227081756,562	−0,289850427893114	0,00284076399216 9	0,0069462615829684	1,22251352643991	0.289850427893114	0,817986859345518	-0.289850427893114	3378
Q08122-2	sp|Q08122-2|TLE3_MOUSE Isoform 2 of Transducin-like enhancer protein 3 OS=Mus musculus GN=Tle3;sp|Q08122-1|TLE3_MOUSE Isoform 1 of Transducin-like enhancer protein 3 OS=Mus musculus GN=Tle3;sp|Q08122|TLE3_MOUSE Transducin-like enhancer protein 3 OS=Mus	1,22	0,591452145533624	0,85975361851476	25,3839467459252	24,6240536772299	24,2731664169751	25,2357243145929	24,6180184843432	25,0309060428071	56663094590,8755	69288834644,0667	−0,290213496997639	0,02293185504935 3	0,0126049566951083	1,22282122330862	0.290213496997639	0,817781030406284	-0.290213496997639	3379
Q60823	sp|Q60823|AKT2_MOUSE RAC-beta serine/threonine-protein kinase OS=Mus musculus GN=Akt2 PE=1 SV=1	1,22	0,430022170175892	0,775108167118334	26,0601159193524	26,7056831906351	26,8246682302946	26,9195750813523	26,624681115036	26,6511319794876	332583163013,093	406884849561,489	−0,290905428181828	0,0155065387347899	0,0061035295812330	1,22340784144106	0.290905428181829	0,817388908364435	-0.290905428181829	3380
O70251	sp|O70251|EF1B_MOUSE Elongation factor 1-beta OS=Mus musculus GN=Eef1b PE=1 SV=5	1,22	0,2524677243134	0,67459732861346	29,6861147613	29,9112106332108	30,239302803755	30,2744604699913	30,1444136462248	30,0229121032537	10120080484350,1	12381964737736,7	−0,29101949204844 1	0,00928977910024669	0,0041727959083233	1,22350457161723	0.291019492048435	0,817324285660981	-0.291019492048435	3381
Q6PDM2	sp|Q6PDM2|SRSF1_MOUSE Serine/arginine-rich splicing factor 1 OS=Mus musculus GN=Srsf1 PE=1 SV=3;sp|Q6PDM2-3|SRSF1_MOUSE Isoform 3 of Serine/arginine-rich splicing factor 1 OS=Mus musculus GN=Srsf1;sp|Q6PDM2-2|SRSF1_MOUSE Isoform 2 of Serine/arginine-ric	1,22	0,157484104402417	0,562098346210542	27,7726589193272	28,0782614029079	28,2415791179015	28,2936711973808	28,2458362215109	28,159207652022	1491544306875,17	1825554739525,84	−0,291528094743838	0,00849174572793874	0,0024141360864875	1,22393597770517	0.291528094743844	0,81703619978143	-0.291528094743844	3382
Q61142-2	sp|Q61142-2|SPIN1_MOUSE Isoform 2 of Spindlin-1 OS=Mus musculus GN=Spin1;sp|Q61142|SPIN1_MOUSE Spindlin-1 OS=Mus musculus GN=Spin1 PE=1 SV=2	1,22	0,201229362424696	0,632096938439929	24,5413781637526	24,8891462278101	24,9892097138287	25,1642937576338	24,974920529059	24,8877355888685	59341696158,5303	72654654463,4285	−0,292009060124602	0,00947555813545262	0,0056535935126052	1,22434408125667	0.292009060124601	0,81676386181701	-0.292009060124601	3383
Q9DBS2	sp|Q9DBS2|TPRGL_MOUSE Tumor protein p63-regulated gene 1-like protein OS=Mus musculus GN=Tprg1l PE=1 SV=1	1,22	0,279727359089764	0,699221280539289	29,0701689048778	29,4005511866025	29,6954546184483	29,6661513123182	29,6094844022266	29,497763870862	5799106113895,21	7100122796696,35	−0,292013438852253	0,010643897253621	0,0028954807387163	1,22434779727237	0.292013438852253	0,816761382858548	-0.292013438852253	3384
Q8R4N0	sp|Q8R4N0|CLYBL_MOUSE Citrate lyase subunit beta-like protein, mitochondrial OS=Mus musculus GN=Clybl PE=1 SV=2	1,22	0,189685084024941	0,615865948776258	28,3939473383592	28,6988938908549	28,9032203325384	28,8498970986966	28,801510817816	28,952674136364	2813228448733,62	3445283640471,32	−0,292396049101158	0,0089406822042147	0,0026740765720898	1,22467254375386	0.29239604910116	0,816544802200601	-0.29239604910116	3385
Q9D920	sp|Q9D920|L12R1_MOUSE Loss of heterozygosity 12 chromosomal region 1 protein homolog OS=Mus musculus GN=Loh12cr1 PE=2 SV=1	1,22	0,26474620514438	0,68394025335819	24,8499747977413	25,343368132376	25,3047067573104	25,4898256936456	25,4222737780244	25,1944575563232	85008455288,8415	104124417446,304	−0,292630174192784	0,0109028770427031	0,0061004341822903	1,22487130359575	0.292630174192785	0,816412301491911	-0.292630174192785	3386
O70318	sp|O70318|E41L2_MOUSE Band 4.1-like protein 2 OS=Mus musculus GN=Epb41l2 PE=1 SV=2	1,23	0,293876117110219	0,711969538636193	28,663140619517	28,1083623316671	28,1229950257761	28,4901679344148	28,6215713507648	28,3939473383592	1948665689158,41	2388998941451,97	−0,293919609822171	0,0111725249121435	0,0040090062876605	1,22596654456606	0.293919609822169	0,815682943741307	-0.293919609822169	3387

P58021	sp|P58021|TM9S2_MOUSE Transmembrane 9 superfamily member 2 OS=Mus musculus GN=Tm9sf2 PE=2 SV=1	1,23	0,594140609695659	0,860080030359509	23,6768094780596	23,0785352794043	24,1365945678446	23,4130659507513	23,8870579935421	24,2039362195013	18308777777,5572	22452924754,9241	-0,29436789936963	0,0224512715746645	0,0166995124410092	1,22634754912186	0.294367899369625	0,815429525435965	-0.294367899369625	3388
Q3TKY6	sp|Q3TKY6|CWC27_MOUSE Peptidyl-prolyl cis-trans isomerase CWC27 homolog OS=Mus musculus GN=Cwc27 PE=2 SV=1	1,23	0,299735801154501	0,713946294657213	23,8407893507363	24,4774642448869	24,1834776243377	24,3990475359719	24,4792576365056	24,2371377474287	31311263938,2686	38418859417,3638	-0,295132942015543	0,0131851090246503	0,0050605961745935	1,22699803793	0.295132942015546	0,814997228265375	-0.295132942015546	3389
Q8R0S4	sp|Q8R0S4|CACB4_MOUSE Voltage-dependent L-type calcium channel subunit beta-4 OS=Mus musculus GN=Cacnb4 PE=1 SV=2;sp|O00305-4| CACB4_HUMAN Isoform 4 of Voltage-dependent L-type calcium channel subunit beta-4 OS=Homo sapiens GN=CACNB4;sp|Q8R0S4-2|CACB4_MO	1,23	0,101643841951479	0,46388602391647	26,8636371325686	27,2381962259367	27,1948465255442	27,3412496695829	27,2212697752775	27,3497540213979	587353855064,915	721134249133,536	-0,29603793608521	0,00756073382373312	0,0026315232906821	1,22776796800599	0.296037936085211	0,814486145638815	-0.296037936085211	3390
Q9QUJ7-2	sp|Q9QUJ7-2|ACSL4_MOUSE Isoform Short of Long-chain-fatty-acid--CoA ligase 4 OS=Mus musculus GN=Acsl4;sp|Q9QUJ7|ACSL4_MOUSE Long-chain-fatty-acid--CoA ligase 4 OS=Mus musculus GN=Acsl4 PE=2 SV=2	1,23	0,00232219260498808	0,0431477969467192	26,3063436289981	26,1961885123238	26,2142780507656	26,5421786038889	26,341020672108	26,4493758498685	248556676443,098	305187356517,21	-0,29612033877161	0,00225133464726883	0,0038072299373134	1,22783809666475	0.296120338771621	0,814439625807636	-0.296120338771621	3391
Q9Z2R6	sp|Q9Z2R6|U119A_MOUSE Protein unc-119 homolog A OS=Mus musculus GN=Unc119 PE=1 SV=1	1,23	0,391669806948587	0,753881747531964	23,3054422881137	23,8383590411367	24,018150513668	23,8751848769123	23,7695776688911	24,1331519049364	20033077398,8509	24598996431,3732	-0,29621539989234	0,0156255279226611	0,0078171172814306	1,22791900323733	0.296215399892344	0,814385963050956	-0.296215399892344	3392
Q6PDY2	sp|Q6PDY2|AEDO_MOUSE 2-aminoethanethiol dioxygenase OS=Mus musculus GN=Ado PE=1 SV=2	1,23	0,340319235417521	0,729668875559815	27,3125156226977	27,8204849529863	28,0120626766497	28,0160964439985	27,9171142157975	27,8286564820045	1087628859203,38	1335894713397,28	-0,29661997084495	0,0130435302654895	0,0033584212929531	1,22826339342976	0.296619970844959	0,814157619081715	-0.296619970844959	3393
P34884	sp|P34884|MIF_MOUSE Macrophage migration inhibitory factor OS=Mus musculus GN=Mif PE=1 SV=2	1,23	0,223329522288454	0,652238880554131	32,420921979523	32,8262003127847	32,9074754208366	32,9074754208366	32,799089045904	33,0667878409893	161932263586851	199024842439958	-0,29755806171198	0,00796612225126244	0,0040897966811342	1,22906231304062	0.297558061711979	0,813628397347949	-0.297558061711979	3394
Q9D9S3	sp|Q9D9S3|RWD2A_MOUSE RWD domain-containing protein 2A OS=Mus musculus GN=Rwdd2a PE=2 SV=1	1,23	0,490555767072201	0,807043358731685	23,8870579935421	24,7872986927083	24,517555127317	24,8641690818116	24,4042625278283	24,5429385120525	39410729474,1252	48449605874,4652	-0,29789647638983	0,0189369440338971	0,0095885464682695	1,22935064945383	0.29789647638983	0,813437565957501	-0.29789647638983	3395
P06733	sp|P06733|ENOA_HUMAN Alpha-enolase OS=Homo sapiens GN=ENO1 PE=1 SV=2;sp|P06733-2|ENOA_HUMAN Isoform MBP-1 of Alpha-enolase OS=Homo sapiens GN=ENO1	1,23	0,342887472941437	0,729891123744941	25,0998804841175	24,4468590871809	24,5156871626391	24,8439697910874	25,0338656906186	24,8070307785967	52678623181,8195	64824886668,4824	-0,29933013931345	0,0145339810962686	0,0048889168629981	1,23057291085115	0.299330139313459	0,812629622496998	-0.299330139313459	3396
Q91X58	sp|Q91X58|ZFN2B_MOUSE AN1-type zinc finger protein 2B OS=Mus musculus GN=Zfand2b PE=1 SV=1	1,23	0,31832146505322	0,722907157640623	24,8661841819719	25,0120600285972	25,4178300813803	25,4779842302653	25,3812269421496	25,0594100709954	79473655843,2637	97801630060,8137	-0,29938179986438	0,0113888188368034	0,0086595109201385	1,23061697644432	0.299381799864382	0,812600524079675	-0.299381799864381	3397
Q4PJX1-3	sp|Q4PJX1-3|ODR4_MOUSE Isoform 3 of Protein odr-4 homolog OS=Mus musculus GN=Odr4;sp|Q4PJX1|ODR4_MOUSE Protein odr-4 homolog OS=Mus musculus GN=Odr4 PE=2 SV=2;sp|Q4PJX1-2|ODR4_MOUSE Isoform 2 of Protein odr-4 homolog OS=Mus musculus GN=Odr4	1,23	0,216073013468409	0,646936285048224	24,2064354818055	24,7055174223122	24,305821491108	24,6644462690852	24,7005673453217	24,4753092267965	39751952582,3361	48919452064,9415	-0,29938251857506	0,010824042938512	0,0049152512785179	1,23061758950374	0.299382518575063	0,812600119264718	-0.299382518575063	3398
Q9Z2H2	sp|Q9Z2H2|RGS6_MOUSE Regulator of G-protein signaling 6 OS=Mus musculus GN=Rgs6 PE=1 SV=2	1,23	0,300013086740942	0,713946294657213	29,6117382906239	30,1964189422983	30,1644509291729	30,3198830461946	30,1714331301119	30,1038443023282	10589344624487,6	13031450569212,7	-0,29938437992190	0,0109608691515993	0,0036595570293034	1,23061917723197	0.299384379921903	0,812599070859029	-0.299384379921903	3399
P24472	sp|P24472|GSTA4_MOUSE Glutathione S-transferase A4 OS=Mus musculus GN=Gsta4 PE=1 SV=3	1,23	0,2219059249191	0,650666016284106	30,333769820244	30,7592274156069	30,8893105450994	30,8856809268552	30,8120815400983	30,9084445981046	20692020531584,5	25475432908387,6	-0,30003213439882	0,00947670418919019	0,0016316447389208	1,23117183599841	0.300032134398829	0,812234304555107	-0.300032134398829	3400
Q3UHD9-2	sp|Q3UHD9-2|AGAP2_MOUSE Isoform 2 of Arf-GAP with GTPase, ANK repeat and PH domain-containing protein 2 OS=Mus musculus GN=Agap2;sp|Q3UHD9|AGAP2_MOUSE Arf-GAP with GTPase, ANK repeat and PH domain-containing protein 2 OS=Mus musculus GN=Agap2 PE=2 SV=1	1,23	0,628257168190711	0,877439099287608	22,7080917274642	22,2152717627516	22,5567734350707	22,0698626041024	22,579222249095	23,4559970658618	5871517616,44102	7231367321,57224	-0,30053501444809	0,0112233924754058	0,0308847433392753	1,23160106023074	0.300535014448092	0,811951233472183	-0.300535014448092	3401
Q64332	sp|Q64332|SYN2_MOUSE Synapsin-2 OS=Mus musculus GN=Syn2 PE=1 SV=2	1,23	0,057422093349569	0,354544580715217	34,2755682575707	34,6244837935552	34,4336965995204	34,6457890411816	34,5822675043587	34,7308080145928	910106629226117	1120952150437850	-0,30061720753248	0,00507227289429031	0,0021507277357774	1,23167122888778	0.300617207532482	0,811904976381574	-0.300617207532482	3402

Q7TN99-4	sp|Q7TN99-4|CPEB3_MOUSE Isoform 4 of Cytoplasmic polyadenylation element-binding protein 3 OS=Mus musculus GN=Cpeb3;sp|Q7TN99-3|CPEB3_MOUSE Isoform 3 of Cytoplasmic polyadenylation element-binding protein 3 OS=Mus musculus GN=Cpeb3;sp|Q7TN99-2|CPEB3_MOU	1,23	0,155584868923456	0,559587733790465	26,6820304486377	26,8651963070805	26,6885628808377	27,1365607688038	26,6924412252218	27,0327780523882	412401494167,539	508091142162,532	−0,30103775331534	0,00388541512183392	0,0086192412642449	1,23203031353742	0.301037753315347	0,811668340471909	-0.301037753315347	3403
P35278	sp|P35278|RAB5C_MOUSE Ras-related protein Rab-5C OS=Mus musculus GN=Rab5c PE=1 SV=2	1,23	0,23428439114036	0,657331974177289	29,6792282244892	30,1327617042981	30,2236511949269	30,2703137998586	30,1213977470374	30,2703137998586	10814191060012,3	13325160295443,4	−0,30122713742384	0,00971773786434748	0,0028449636831125	1,23219205407938	0.30122713742386	0,811561798900855	-0.301227137423859	3404
Q9WUA2	sp|Q9WUA2|SYFB_MOUSE Phenylalanine--tRNA ligase beta subunit OS=Mus musculus GN=Farsb PE=2 SV=2	1,23	0,220091094509315	0,648948732547882	29,0726137546512	29,643927476549	29,4282378281537	29,6607221554744	29,5620727820686	29,5484224910215	5757894696736,35	7094960147361,83	−0,30125317622758	0,00981903713303386	0,0020708410362421	1,23221429377362	0.30125317622758	0,811547151378623	-0.30125317622758	3405
P97372	sp|P97372|PSME2_MOUSE Proteasome activator complex subunit 2 OS=Mus musculus GN=Psme2 PE=2 SV=4	1,23	0,0646887451257087	0,38006509013234	27,9562316171464	28,2437154901872	28,2953913503404	28,4258373734941	28,2936711973808	28,4026187469632	1705898076755,38	2102277589311,07	−0,30142172674775	0,00648789738644998	0,0024869585012000	1,23235826217098	0.301421726747751	0,811452343605306	-0.301421726747751	3406
Q6NVG1	sp|Q6NVG1|LPCT4_MOUSE Lysophospholipid acyltransferase LPCAT4 OS=Mus musculus GN=Lpcat4 PE=1 SV=1	1,23	0,0834418812572589	0,429900906954255	27,2725327823275	26,8845106514255	26,9807462280019	27,3163381242936	27,2162114865181	27,232167200089	557055040099,728	686523026611,594	−0,30148822969044	0,00747087872804194	0,0019738447718510	1,23241507067001	0.301488229690448	0,811414939494646	-0.301488229690448	3407
P67871	sp|P67871|CSK2B_MOUSE Casein kinase II subunit beta OS=Mus musculus GN=Csnk2b PE=1 SV=1;sp|P67870|CSK2B_HUMAN Casein kinase II subunit beta OS=Homo sapiens GN=CSNK2B PE=1 SV=1	1,23	0,247140482745759	0,671718952804191	28,6868977896118	29,0383137035681	29,2120692784127	29,3627419253404	29,0412682162963	29,1607446221874	3849997406266,01	4745659790434,78	−0,30175120562641	0,00923242354313901	0,0055670513723348	1,2326397370323	0.301751205626409	0,81126704742425	-0.301751205626409	3408
P70425	sp|P70425|RIT2_MOUSE GTP-binding protein Rit2 OS=Mus musculus GN=Rit2 PE=1 SV=1	1,23	0,593613575212625	0,860080030359509	24,7672234633243	25,7970224427502	25,9419377124712	25,8903660723575	25,4581618858169	25,7852122009926	118960959664,136	146640040948,417	−0,30179090301058	0,0251156089670466	0,0087664297649781	1,2326736549741	0.301790903010595	0,811244724801887	-0.301790903010595	3409
Q60817	sp|Q60817|NACA_MOUSE Nascent polypeptide-associated complex subunit alpha OS=Mus musculus GN=Naca PE=1 SV=1;sp|P70670|NACAM_MOUSE Nascent polypeptide-associated complex subunit alpha, muscle-specific form OS=Mus musculus GN=Naca PE=1 SV=2	1,23	0,295415800701835	0,711969538636193	28,4901679344148	28,8574544305787	28,995304264796	28,8118954040246	29,2467701061378	28,9121400873384	3158048985700,61	3893262194372,71	−0,3019459576652	0,00907224656077092	0,0078550984473088	1,23280614455352	0.301945957665201	0,811157540395103	-0.301945957665201	3410
Q9CQR6	sp|Q9CQR6|PPP6_MOUSE Serine/threonine-protein phosphatase 6 catalytic subunit OS=Mus musculus GN=Ppp6c PE=2 SV=1	1,23	0,340878226390912	0,72969758766342	28,130530652555	28,5775854342843	28,8465312886039	28,8489065316386	28,6090306171416	28,7253092782756	2428307896448,48	2994351537471,4	−0,30229224482305	0,0126821959506556	0,0041756339512640	1,23310208802219	0.302292244823055	0,810962863264572	-0.302292244823056	3411
P53026	sp|P53026|RL10A_MOUSE 60S ribosomal protein L10a OS=Mus musculus GN=Rpl10a PE=1 SV=3	1,23	0,319998054553363	0,723342215945077	26,2872690468727	26,7306224117162	26,9915324948691	26,9699393539991	26,8071250785447	26,8620165414284	382428668211,524	471739922016,019	−0,30280102031895	0,0133502467629254	0,0030816595818746	1,23353702593001	0.30280102031896	0,810676922523716	-0.30280102031896	3412
P56371	sp|P56371|RAB4A_MOUSE Ras-related protein Rab-4A OS=Mus musculus GN=Rab4a PE=1 SV=2	1,23	0,147254077835096	0,544732077663815	26,8651963070805	27,0534472647175	27,3351344193571	27,2944083728934	27,210872020632	27,3782962363329	579014098357,859	714269045893,615	−0,30286912427188	0,0087323492651169	0,0030669948528015	1,23359525773095	0.302869124271879	0,810638654561126	-0.302869124271879	3413
Q922H1-2	sp|Q922H1-2|ANM3_MOUSE Isoform 2 of Protein arginine N-methyltransferase 3 OS=Mus musculus GN=Prmt3;sp|Q922H1|ANM3_MOUSE Protein arginine N-methyltransferase 3 OS=Mus musculus GN=Prmt3 PE=1 SV=2	1,23	0,299201650498586	0,71351128730767	23,8148854521229	24,4657851778578	24,3120591899788	24,4408004316673	24,3111380538731	24,4713782819228	32275575564,3694	39825460502,2662	−0,30324822067099	0,0140599436218318	0,0034854791147912	1,23391945165593	0.303248220670996	0,810425671349935	-0.303248220670997	3414
P28651	sp|P28651|CAH8_MOUSE Carbonic anhydrase-related protein OS=Mus musculus GN=Ca8 PE=1 SV=5;sp|P35219|CAH8_HUMAN Carbonic anhydrase-related protein OS=Homo sapiens GN=CA8 PE=1 SV=3	1,23	0,285192699881282	0,704836107321858	23,3964502955791	23,9032676843376	23,9723375129221	23,8482598894016	23,9460675522639	24,1086932997909	20781974366,861	25646516246,4083	−0,30343014505134	0,0132359895888995	0,0054888177776915	1,23407505916783	0.303430145051343	0,810323482814998	-0.303430145051344	3415
P50518	sp|P50518|VATE1_MOUSE V-type proton ATPase subunit E 1 OS=Mus musculus GN=Atp6v1e1 PE=1 SV=2	1,23	0,349643257053656	0,732843629012038	32,9774514497747	33,6516402612364	33,6516402612364	33,658865904478	33,6093552004065	33,6444146179947	328944663801659	406069417898227	−0,30388146911857	0,0116446026308353	0,0007568964812684	1,23446118020346	0.303881469118577	0,810070025721815	-0.303881469118577	3416
Q9QYX7-2	sp|Q9QYX7-2|PCLO_MOUSE Isoform 2 of Protein piccolo OS=Mus musculus GN=Pclo;sp|Q9QYX7|PCLO_MOUSE Protein piccolo OS=Mus musculus GN=Pclo PE=1 SV=4	1,23	0,748148733157333	0,928334362985549	22,6427415317092	20,9840740472232	22,5448185137444	22,6062886817837	21,5152196899429	22,6823866658236	3795991066,24419	4686561582,72947	−0,30405324323884	0,042192686282643	0,029324907902241	1,23460816976222	0.304053243238841	0,809973580680741	-0.304053243238841	3417
Q80YA7	sp|Q80YA7|DPP8_MOUSE Dipeptidyl peptidase 8 OS=Mus musculus GN=Dpp8 PE=2 SV=1	1,23	0,570543755379902	0,847969255294491	27,4002419470032	26,417345126627	26,3537858638036	26,9247281789472	27,2058942963641	26,6737413947028	403640706613,55	498459389020,337	−0,30440429312039	0,0219536620718132	0,0098838364721420	1,23490862257747	0.304404293120399	0,809776514405432	-0.304404293120399	3418
Q9CPT3	sp|Q9CPT3|NANP_MOUSE N-acylneuraminate-9-phosphatase OS=Mus musculus GN=Nanp PE=1 SV=1	1,23	0,0944006330893956	0,450242157645468	26,0769885460403	26,062710168417	26,3698715068758	26,5234452786041	26,2599380141522	26,35933783101	231966021873,359	286472115783,221	−0,30448122235826	0,00662460361495974	0,0050442236121348	1,23497447371676	0.304481222358261	0,809733335613342	-0.304481222358261	3419
Q8R066	sp|Q8R066|C1QT4_MOUSE Complement C1q tumor necrosis factor-related protein 4 OS=Mus musculus GN=C1qtnf4 PE=1 SV=1	1,23	0,320175891200551	0,723365701688419	25,0338656906186	25,2054719742707	25,0467789403677	25,6958788171413	25,0006538060705	25,2227384535695	79210301896,0033	97822817276,4083	−0,30448293872827	0,0038081745173807	0,0140315017324259	1,2349759429631	0.304482938728271	0,809732372276568	-0.304482938728271	3420

Q9QZB0	sp|Q9QZB0|RGS17_MOUSE Regulator of G-protein signaling 17 OS=Mus musculus GN=Rgs17 PE=1 SV=1;sp|Q9CX84|RGS19_MOUSE Regulator of G-protein signaling 19 OS=Mus musculus GN=Rgs19 PE=2 SV=1;sp|Q9QZB1|RGS20_MOUSE Regulator of G-protein signaling 20 OS=Mus mu	1,24	0,285823394343815	0,704836107321858	25,0561523437009	25,4354333008474	25,6586432911233	25,7670259217681	25,4561566173449	25,5614805926799	105651274592,046	130532452453,379	−0,305098356171502	0,011999803609608	0,0061771813213739	1,2355028650379	0.305098356171498	0,809387034460112	-0.305098356171498	3421
Q9DBJ1	sp|Q9DBJ1|PGAM1_MOUSE Phosphoglycerate mutase 1 OS=Mus musculus GN=Pgam1 PE=1 SV=3	1,24	0,289879405057366	0,709103602076768	34,0837055790221	34,4336965995204	34,7308080145928	34,698424438377	34,486208526548	34,698424438377	884523381933885	1092981634470490	−0,305296973943308	0,00941162416840372	0,00353829344890484	1,23567297009248	0.305296973943305	0,809275612725556	-0.305296973943305	3422
Q9CY64	sp|Q9CY64|BIEA_MOUSE Biliverdin reductase A OS=Mus musculus GN=Blvra PE=2 SV=1	1,24	0,435961378228828	0,778450481049573	28,8118954040246	29,4120521281494	29,7389386091483	29,5542026275958	29,5120517470896	29,531702480219	5419160592249,31	6696809165754,73	−0,305404456366213	0,0160358477187726	0,000714175134852	1,23576503256478	0.305404456366213	0,809215323016984	-0.305404456366213	3423
Q8C7D2-2	sp|Q8C7D2-2|CRBN_MOUSE Isoform 2 of Protein cereblon OS=Mus musculus GN=Crbn;sp|Q8C7D2-3|CRBN_MOUSE Isoform 3 of Protein cereblon OS=Mus musculus GN=Crbn;sp|Q8C7D2|CRBN_MOUSE Protein cereblon OS=Mus musculus GN=Crbn PE=1 SV=1	1,24	0,397580053234206	0,754338506152154	26,9302714663707	27,749949888637	27,5668665706527	27,6776121109565	27,6333084505179	27,5713979478984	806279243349,861	996424793520,257	−0,305481337647601	0,0156932480277273	0,0019310420388026	1,23583088829175	0.305481337647602	0,80917220104627	-0.305481337647602	3424
Q8VCM7	sp|Q8VCM7|FIBG_MOUSE Fibrinogen gamma chain OS=Mus musculus GN=Fgg PE=1 SV=1	1,24	0,373536817613519	0,744143356508855	25,9627882430883	25,1843097620353	25,698996365124	25,7149021750404	25,9504924356993	25,8175097401103	133232930447,929	164740077947,927	−0,30624086700121	0,0154563591563397	0,00457343620763107	1,23648168207418	0.306240867001205	0,808746311811524	-0.306240867001205	3425
Q8CII2	sp|Q8CII2|CD123_MOUSE Cell division cycle protein 123 homolog OS=Mus musculus GN=Cdc123 PE=2 SV=2	1,24	0,567114572524626	0,845527592693687	23,1990977632119	22,2997639006898	23,5004085167368	23,3895979642284	23,1262887967585	23,1210148714882	9742433085,84026	12049639075,0726	−0,30663591115979	0,0271590486077812	0,00661574717314399	1,23682030647823	0.306635911159796	0,808524888184799	-0.306635911159796	3426
P26450	sp|P26450|P85A_MOUSE Phosphatidylinositol 3-kinase regulatory subunit alpha OS=Mus musculus GN=Pik3r1 PE=1 SV=2	1,24	0,217607413104965	0,647685277431762	27,0327780523882	26,9096572194275	26,7611411553313	27,024059925362	27,4140252679283	26,9044098251012	481991416324,163	596392596511,4	−0,307254894373386	0,00505613852087042	0,00982839009922366	1,23735107371766	0.307254894373385	0,808178067842513	-0.307254894373385	3427
O55176	sp|O55176|PJA1_MOUSE E3 ubiquitin-protein ligase Praja-1 OS=Mus musculus GN=Pja1 PE=1 SV=3;sp|O55176-5|PJA1_MOUSE Isoform 4 of E3 ubiquitin-protein ligase Praja-1 OS=Mus musculus GN=Pja1;sp|O55176-4|PJA1_MOUSE Isoform 3 of E3 ubiquitin-protein ligase Pr	1,24	0,384250299357133	0,751739350763028	22,1323356543618	21,9944238728401	21,3460209259128	22,0365622706126	22,1014692905163	21,9746816952802	3007154594,10077	3722164107,72642	−0,307742627271651	0,0192386091294336	0,00287689678858378	1,23776945655814	0.307742627271653	0,807904892709743	-0.307742627271653	3428
Q921L5	sp|Q921L5|COG2_MOUSE Conserved oligomeric Golgi complex subunit 2 OS=Mus musculus GN=Cog2 PE=2 SV=2	1,24	0,207934084518235	0,638546806637671	24,9023550022812	24,9719414710983	24,4526054481224	24,9200121770116	24,9662926902838	25,0817452967474	57533543152,3932	71242120085,009	−0,308327129996171	0,0113783672362024	0,00333323796501866	1,23827103601641	0.308327129996172	0,807577639235635	-0.308327129996172	3429
E9Q6B2	sp|E9Q6B2|CC85C_MOUSE Coiled-coil domain-containing protein 85C OS=Mus musculus GN=Ccdc85c PE=2 SV=1	1,24	0,556600339001098	0,841293423821966	23,8100825694553	23,4130659507513	22,6513182672976	23,6408917707753	23,6274438234	23,2474511581564	13042628290,6683	16151233328,3094	−0,308409710959902	0,02528268159355	0,0095030712286338	1,23834191762294	0.308409710959906	0,807531414199036	-0.308409710959906	3430
Q91VW3	sp|Q91VW3|SH3L3_MOUSE SH3 domain-binding glutamic acid-rich-like protein 3 OS=Mus musculus GN=Sh3bgrl3 PE=1 SV=1	1,24	0,471102717755848	0,79699412444793	29,6831401315376	30,2888262852398	30,1824637696736	29,8078729257526	30,4410482207959	30,5474125413229	11250984240729,9	13935275717311,8	−0,308690332742816	0,0107612551636286	0,0132104933144625	1,2385828136586	0.308690332742814	0,807374354764491	-0.308690332742814	3431
P14148	sp|P14148|RL7_MOUSE 60S ribosomal protein L7 OS=Mus musculus GN=Rpl7 PE=1 SV=2	1,24	0,277130709004138	0,699203439845458	27,3252327797371	27,840803271956	27,8436592712793	28,0716553884336	27,7598510714024	27,8204849529863	1039642937694,28	1287852395926,09	−0,30887912786964	0,0107876302804379	0,00592886266667113	1,23874490869172	0.30887912786964	0,807268706400687	-0.30887912786964	3432
Q9CVB6	sp|Q9CVB6|ARPC2_MOUSE Actin-related protein 2/3 complex subunit 2 OS=Mus musculus GN=Arpc2 PE=1 SV=3	1,24	0,181140850493329	0,600744525039334	32,2182618919671	32,504668068945	32,72254043549	32,773886273776	32,5652501037116	32,7488011970875	127842923417288	158373802207309	−0,308961403961436	0,00778633173474288	0,00348383379885624	1,23881555563593	0.308961403961434	0,807222669630319	-0.308961403961434	3433
Q8QZY1	sp|Q8QZY1|EIF3L_MOUSE Eukaryotic translation initiation factor 3 subunit L OS=Mus musculus GN=Eif3l PE=1 SV=1	1,24	0,169785206951091	0,581421989337334	29,2762930224565	29,7887479080092	29,5915968002301	29,8475138120678	29,7669313833442	29,6861147613	6829090218454,09	8464087415454,26	−0,309661134063929	0,00874681044600881	0,00271105423329879	1,23941654667	0.309661134063928	0,806831248692577	-0.309661134063928	3434
P63300	sp|P63300|SELW_MOUSE Selenoprotein W OS=Mus musculus GN=Sepw1 PE=1 SV=3	1,24	0,340106740544816	0,729620681454069	26,5688885730431	26,2649632160102	26,0872422456991	26,1720458506377	26,7284847671728	26,6649401997547	266071616623,509	329823534966,39	−0,309879729675988	0,0092584875382808	0,0114840568028216	1,23960435596966	0.309879729675988	0,806709007744467	-0.309879729675988	3435
Q3UYG8	sp|Q3UYG8|MACD2_MOUSE O-acetyl-ADP-ribose deacetylase MACROD2 OS=Mus musculus GN=Macrod2 PE=2 SV=1	1,24	0,467724806025735	0,796164546189264	25,8152971897798	26,613068974371	26,4619673766869	26,8215162251178	26,4634183601821	26,2503112648405	263357390776,567	326517249793,679	−0,310137263479789	0,0161146610337588	0,0108877444155384	1,23982565604433	0.310137263479786	0,806565015915632	-0.310137263479786	3436
Q6PHU5	sp|Q6PHU5|SORT_MOUSE Sortilin OS=Mus musculus GN=Sort1 PE=1 SV=1;sp|Q6PHU5-2|SORT_MOUSE Isoform 2 of Sortilin OS=Mus musculus GN=Sort1	1,24	0,333485873195065	0,72953198881961	28,2415791179015	27,763130170804	27,4854015248497	28,0603919717577	28,0805683689805	27,9943201847396	1220201271677,95	1512966650455,13	−0,310261047974132	0,0137442495768946	0,00160860250628433	1,23993203873209	0.310261047974128	0,806495814901729	-0.310261047974128	3437

Q5DTT2-2	sp|Q5DTT2-2|PSD1_MOUSE Isoform 2 of PH and SEC7 domain-containing protein 1 OS=Mus musculus GN=Psd;sp|Q5DTT2|PSD1_MOUSE PH and SEC7 domain-containing protein 1 OS=Mus musculus GN=Psd PE=1 SV=2;sp|Q5DTT2-3|PSD1_MOUSE Isoform 3 of PH and SEC7 domain-conta	1,24	0,219090719402036	0,648792713611515	23,921929541055	23,326766206189	23,5542435875806	23,8091764130591	23,8645191979705	23,775612781693	17773595219,7497	22046862333,3677	−0,310837811471143	0,0127249624599455	0,0018850681384318	1,24042784033191	0.310837811471143	0,806173456839232	-0.310837811471143	3438
Q8CHU3	sp|Q8CHU3|EPN2_MOUSE Epsin-2 OS=Mus musculus GN=Epn2 PE=1 SV=1;sp|Q8CHU3-2|EPN2_MOUSE Isoform 2 of Epsin-2 OS=Mus musculus GN=Epn2	1,24	0,0048436130512657	0,0752916426016952	28,2742677874623	28,2185163002376	28,046173766494	28,4457488486355	28,3308483775731	28,4095407476388	1730882687081,01	2147615611220,91	−0,311227849728691	0,00421969968820472	0,00206881171098822	1,24076324019549	0.311227849728692	0,805955534145615	-0.311227849728692	3439
Q9Z0H4-11	sp|Q9Z0H4-11|CELF2_MOUSE Isoform 11 of CUGBP Elav-like family member 2 OS=Mus musculus GN=Celf2;sp|Q9Z0H4-7|CELF2_MOUSE Isoform 7 of CUGBP Elav-like family member 2 OS=Mus musculus GN=Celf2;sp|Q9Z0H4-5|CELF2_MOUSE Isoform 5 of CUGBP Elav-like family mem	1,24	0,503946971428121	0,814993646406912	27,6294369835375	28,3695269666755	28,6623100861355	28,2559271319953	28,5963004492755	28,4562699090736	1802912770505,62	2237020203955,55	−0,311248689142358	0,0188634578677777	0,0060161037180860	1,24078116287799	0.311248689142354	0,805943892378651	-0.311248689142354	3440
Q9DB05	sp	Q9DB05	SNAA_MOUSE Alpha-soluble NSF attachment protein OS=Mus musculus GN=Napa PE=1 SV=1	1,24	0,288784434143151	0,708193823372149	29,9903371484726	30,392909761289	30,6302391026061	30,7148429894456	30,4798415039778	30,4662615796495	14981371882854,5	18590070146939,5	−0,311362472917652	0,0106628382786199	0,0045743600262802	1,24087902578635	0.311362472917659	0,805880330974485	-0.311362472917659	3441
P68510	sp	P68510	1433F_MOUSE 14-3-3 protein eta OS=Mus musculus GN=Ywhah PE=1 SV=2	1,24	0,462435018809488	0,793595716606975	32,9459102626137	33,5634667763458	33,6211455554601	33,8575362770501	33,2027166374192	33,7181235415771	312880005766614	388297203997405	−0,311551851129903	0,0112146472124261	0,0102680391921314	1,24104192291228	0.311551851129903	0,805774552444897	-0.311551851129903	3442
Q8BGD5	sp	Q8BGD5	CPT1C_MOUSE Carnitine O-palmitoyltransferase 1, brain isoform OS=Mus musculus GN=Cpt1c PE=1 SV=1	1,24	0,15487653243146	0,558367419147258	28,1859618455125	27,924640903582	27,763130170804	28,3456414045074	28,1272472497798	28,0488815319204	1386648739631,28	1720994427831,91	−0,311640050138395	0,00763183523212682	0,0054585057773014	1,241117796198	0.311640050138396	0,805725293008743	-0.311640050138397	3443
Q9WVK8	sp	Q9WVK8	CP46A_MOUSE Cholesterol 24-hydroxylase OS=Mus musculus GN=Cyp46a1 PE=2 SV=1	1,24	7,28×10^−06	0,0003980338172769	29,0763948194066	28,934830454227	29,0088476558689	29,1775307111958	29,2251086014483	29,2657804305218	3957726960423,32	4912512221978,1	−0,31178891094946	0,00244105129064412	0,0015114863539714	1,24124586438188	0.311788910949462	0,805642160586764	-0.311788910949462	3444
Q9JMA1	sp	Q9JMA1	UBP14_MOUSE Ubiquitin carboxyl-terminal hydrolase 14 OS=Mus musculus GN=Usp14 PE=1 SV=3	1,24	0,205611829306613	0,636399775345126	30,1644509291729	30,6680635283742	30,6742077219904	30,7710464061561	30,6923605230542	30,6923605230542	17658541616328,9	21923695426068	−0,312124798758526	0,00959112082497979	0,0014788867928591	1,24153488450004	0.312124798758532	0,805454613063649	-0.312124798758532	3445
Q3U5F4	sp	Q3U5F4	YRDC_MOUSE YrdC domain-containing protein, mitochondrial OS=Mus musculus GN=Yrdc PE=1 SV=1	1,24	0,205211332932548	0,636399775345126	24,9004151407677	25,2652188489382	25,4600043280656	25,506098678661	25,3401904290326	25,4287308522879	88701807694,4295	110138736981,903	−0,312286558286878	0,0112686647540663	0,0032651633504298	1,24167409712012	0.31228655828688	0,805364308009127	-0.31228655828688	3446
P17751	sp	P17751	TPIS_MOUSE Triosephosphate isomerase OS=Mus musculus GN=Tpi1 PE=1 SV=4	1,24	0,202574495785886	0,634128025084232	34,6244837935552	34,8145794845827	35,1168009278487	35,2475710661237	34,9429327686499	35,016095172406	1367765437137800	1699085118848350	−0,312937290205082	0,00712377576400221	0,0045347179736121	1,24223428426724	0.312937290205085	0,805001127939299	-0.312937290205085	3447
Q9DCH4	sp	Q9DCH4	EIF3F_MOUSE Eukaryotic translation initiation factor 3 subunit F OS=Mus musculus GN=Eif3f PE=1 SV=2	1,24	0,130822790114487	0,514825425964414	29,6053749310012	29,1446312403025	29,41407607155	29,7040018754618	29,5252640648693	29,5856163544791	5795062711304,51	7198982157452,04	−0,312968669189317	0,00787646357720121	0,0030712944399162	1,24226130347285	0.312968669189316	0,804983619150346	-0.312968669189316	3448
Q8BWY4	sp	Q8BWY4	MBLC1_MOUSE Metallo-beta-lactamase domain-containing protein 1 OS=Mus musculus GN=Mblac1 PE=2 SV=1	1,24	0,355290680122421	0,732843629012038	22,1014692905163	22,6688521928451	22,6062886817837	22,9780575036453	22,5052388583284	22,5448185137444	5672354107,6159	7048201338,33952	−0,313307538353135	0,0138517603618153	0,0115674486539382	1,24255312778805	0.313307538353137	0,80479456180692	-0.313307538353137	3449
Q9CQD1	sp	Q9CQD1	RAB5A_MOUSE Ras-related protein Rab-5A OS=Mus musculus GN=Rab5a PE=1 SV=1	1,24	0,273739209645904	0,695545109045926	30,7710464061561	31,2428831501822	31,4086456757793	31,4372046772841	31,2551943052669	31,3817725927108	33442826018801,1	41555757339312,8	−0,313351604305156	0,0106232511235515	0,0029751185946776	1,24259108114699	0.313351604305156	0,804769980384002	-0.313351604305156	3450
Q9CZJ2	sp	Q9CZJ2	HS12B_MOUSE Heat shock 70 kDa protein 12B OS=Mus musculus GN=Hspa12b PE=1 SV=1	1,24	0,210086421844269	0,641644645910089	27,1500360627144	26,9993754734062	26,6200366760221	27,2818929651463	27,0927002045124	27,04657919394	492691672233,875	612240360253,25	−0,313413067111072	0,0101436215394787	0,0045944576085949	1,24264402009748	0.313413067111074	0,804735695683433	-0.313413067111074	3451
O08919	sp	O08919	NUMBL_MOUSE Numb-like protein OS=Mus musculus GN=Numbl PE=1 SV=3	1,24	0,226158743447856	0,652238880554131	28,9104405271909	28,7307284512667	28,4931602800957	29,151901871807	28,9475574493059	28,6894779101035	2945922380449,2	3664253487946,17	−0,314799891962495	0,00729003890830755	0,0080101826271573	1,24383911547168	0.314799891962498	0,80396249608277	-0.314799891962498	3452
Q8VD63	sp	Q8VD63	TSYL4_MOUSE Testis-specific Y-encoded-like protein 4 OS=Mus musculus GN=Tspyl4 PE=2 SV=1	1,24	0,221430226333205	0,650415221831493	25,1876434359737	25,7705950123853	25,3445401043115	25,7001815061086	25,6863781120229	25,5721737946229	111162565128,424	138330437919,122	−0,31544760789669	0,0118601564503474	0,0027388856231085	1,24439767793512	0.315447607896684	0,803601628106012	-0.315447607896683	3453
P52196	sp	P52196	THTR_MOUSE Thiosulfate sulfurtransferase OS=Mus musculus GN=Tst PE=1 SV=3	1,24	0,245006886285985	0,669125304965153	30,3514194051073	30,8170158313743	30,9382317000035	31,039393479464	30,8577552537318	30,8665321302372	21567794274683,7	26848389520706,1	−0,315956911400879	0,0100901746899681	0,0033125873073690	1,24483705560103	0.315956911400881	0,803317988889059	-0.315956911400881	3454
Q8CGC4	sp	Q8CGC4	LS14B_MOUSE Protein LSM14 homolog B OS=Mus musculus GN=Lsm14b PE=2 SV=3	1,24	0,233659923622552	0,657279264635888	25,5653482547121	26,0701199334524	25,7758152556667	26,1491158481579	26,1186864653252	25,8007350073515	160853708811,438	200252673498,856	−0,316072302985956	0,00982583771218415	0,0074148058872503	1,24493662582318	0.316072302985954	0,803253739393182	-0.316072302985954	3455

P23819-4	sp|P23819-4|GRIA2_MOUSE Isoform 4 of Glutamate receptor 2 OS=Mus musculus GN=Gria2;sp|P23819|GRIA2_MOUSE Glutamate receptor 2 OS=Mus musculus GN=Gria2 PE=1 SV=3;sp|P23819-3|GRIA2_MOUSE Isoform 3 of Glutamate receptor 2 OS=Mus musculus GN=Gria2;sp|P2381	1,25	0,124730052437116	0,505039882258761	30,0424084078288	29,7678736961036	29,5724244467374	29,9281463515691	30,0523063560686	30,0602349868501	8699055016442,52	10832397896247,9	−0,31642204439490	0,00792429694810305	0,00246817622488897	1,24523846277246	0.316422044394904	0,803059036398181	-0.316422044394904	3456
Q02819	sp|Q02819|NUCB1_MOUSE Nucleobindin-1 OS=Mus musculus GN=Nucb1 PE=1 SV=2	1,25	0,0742428318968757	0,405033911033557	27,9951791545785	28,3357637084218	28,381044130583	28,4815245373331	28,4245313453588	28,4643306249264	1833648837475,74	2283648507611,2	−0,31662323794060	0,00746977526421049	0,00102731941686611	1,24541213177707	0.316623237940608	0,802947052212435	-0.316623237940608	3457
P14069	sp|P14069|S10A6_MOUSE Protein S100-A6 OS=Mus musculus GN=S100a6 PE=1 SV=3	1,25	0,327990283018094	0,726927682919497	27,1210844296353	26,8802456280836	26,6001617794996	26,7663350011133	27,1684490497881	27,3252327797371	465866032455,471	580219476623,101	−0,31668358076952	0,0097035770024578	0,0106429944215528	1,24546422404935	0.316683580769527	0,802913468480632	-0.316683580769527	3458
Q8IWP9	sp|Q8IWP9|CC28A_HUMAN Coiled-coil domain-containing protein 28A OS=Homo sapiens GN=CCDC28A PE=2 SV=1	1,25	0,00298148785406623	0,0530704838023788	21,7873155101343	21,7927037897341	21,8402680937608	22,1674770243792	21,9810470764094	21,9306752260833	2954994021,26862	3680814056,33375	−0,31686965949059	0,00133635289636109	0,00566345093662871	1,24562487431143	0.316869659490596	0,80280991542722	-0.316869659490596	3459
Q99L04	sp|Q99L04|DHRS1_MOUSE Dehydrogenase/reductase SDR family member 1 OS=Mus musculus GN=Dhrs1 PE=2 SV=1	1,25	0,373793093818063	0,744143356508855	28,1401525448235	28,7146432611685	28,9124490208349	28,9598110128047	28,6623100861355	28,8042521530299	2606635978885,57	3247124930536,11	−0,31697377008770	0,0140321662475426	0,00516517306205411	1,24571476678703	0.316973770087701	0,802751983569418	-0.316973770087701	3460
Q8BMB3	sp|Q8BMB3|IF4E2_MOUSE Eukaryotic translation initiation factor 4E type 2 OS=Mus musculus GN=Eif4e2 PE=1 SV=1	1,25	0,65867766825366	0,893259018883586	24,5790747392146	25,0703734773173	25,9760107033085	25,3323809620451	24,9925963663285	25,9600094693325	88696503546,0936	110505257125,479	−0,31716586624166	0,0281109789751429	0,0193009830534992	1,24588064588196	0.317165866241668	0,802645103530038	-0.317165866241668	3461
Q60929-2	sp|Q60929-2|MEF2A_MOUSE Isoform 2 of Myocyte-specific enhancer factor 2A OS=Mus musculus GN=Mef2a;sp|Q60929|MEF2A_MOUSE Myocyte-specific enhancer factor 2A OS=Mus musculus GN=Mef2a PE=1 SV=2;sp|Q60929-3|MEF2A_MOUSE Isoform 3 of Myocyte-specific enhancer	1,25	0,503609191197212	0,814993646406912	22,3912477426894	23,3041913196319	23,0456642223253	23,5022385635319	23,0008472762563	22,8991140368772	8939103083,62317	11142880665,1688	−0,31792025828476	0,0205362070677988	0,0139568293461089	1,24653229310925	0.317920258284764	0,802225506332994	-0.317920258284764	3462
P62812	sp|P62812|GBRA1_MOUSE Gamma-aminobutyric acid receptor subunit alpha-1 OS=Mus musculus GN=Gabra1 PE=1 SV=1	1,25	0,0938399140366359	0,450242157645468	27,5359993615751	27,0965384478712	27,3283963991727	27,4832478791861	27,5359993615751	27,6030325495586	732926604016,126	913692510573,979	−0,31803999701118	0,00804683311584426	0,00217982605037459	1,24663575529573	0.318039997011186	0,802158927138085	-0.318039997011186	3463
Q8BHT6	sp|Q8BHT6|B3GLT_MOUSE Beta-1,3-glucosyltransferase OS=Mus musculus GN=B3galtl PE=2 SV=3	1,25	0,0571246528339762	0,353736424574922	22,5849545096597	22,8462665924133	22,9194574103533	23,1133185411363	22,9717251193296	22,9274759558395	7848274252,62294	9785555487,13088	−0,31827829280766	0,00771815488181678	0,00421999167179291	1,2468416841907	0.318278292807662	0,802026442233588	-0.318278292807663	3464
Q9DB90	sp|Q9DB90|SMG9_MOUSE Protein SMG9 OS=Mus musculus GN=Smg9 PE=2 SV=1;sp|Q9DB90-2|SMG9_MOUSE Isoform 2 of Protein SMG9 OS=Mus musculus GN=Smg9	1,25	0,508839334061668	0,816124555116335	23,80523416359	24,9361022925742	24,4377243018323	24,5806585090775	24,517555127317	24,7428706679691	39242267392,8069	48931870427,7513	−0,31836602909845	0,0232344292281446	0,00472232629053841	1,24691751212929	0.318366029098454	0,801977669150186	-0.318366029098454	3465
Q9DBR3	sp|Q9DBR3|ARMC8_MOUSE Armadillo repeat-containing protein 8 OS=Mus musculus GN=Armc8 PE=2 SV=2;sp|Q9DBR3-2|ARMC8_MOUSE Isoform 2 of Armadillo repeat-containing protein 8 OS=Mus musculus GN=Armc8	1,25	0,309802736297022	0,719371512809526	26,69891974877	27,4170667080726	27,1164940541076	27,2799384547728	27,1975304598764	27,4194342735147	574918140723,597	717449019710,816	−0,31951976715677	0,0133194931566178	0,00410891419649421	1,24791508371579	0.31951976715677	0,801336575740714	-0.31951976715677	3466
P97441	sp|P97441|ZNT3_MOUSE Zinc transporter 3 OS=Mus musculus GN=Slc30a3 PE=1 SV=1	1,25	0,113663202639427	0,482581632051629	29,763614849229	29,3677682146643	29,3536338319982	29,7016909671295	29,7910678192371	29,656852932955	6449383314200,43	8048744558846,04	−0,31960255198772	0,00789047937423935	0,00229932631597839	1,24798669372374	0.319602551987729	0,801290594706746	-0.319602551987729	3467
Q9CQ54	sp|Q9CQ54|NDUC2_MOUSE NADH dehydrogenase [ubiquinone] 1 subunit C2 OS=Mus musculus GN=Ndufc2 PE=1 SV=1	1,25	0,229867880277041	0,654468548759245	25,5700819737257	25,2712628519499	25,0980650300174	25,3674906455573	25,462854440618	25,773705472882	98481703693,3012	122905735405,791	−0,31962461557219	0,00943297801554475	0,0083188745717043	1,24800577971877	0.319624615572193	0,801278340413892	-0.319624615572193	3468
Q64337	sp|Q64337|SQSTM_MOUSE Sequestosome-1 OS=Mus musculus GN=Sqstm1 PE=1 SV=1;sp|Q64337-2|SQSTM_MOUSE Isoform 2 of Sequestosome-1 OS=Mus musculus GN=Sqstm1	1,25	0,328663817144648	0,728064941932433	25,530459038085	25,8337781042129	25,4434411168015	25,7062486810014	26,2029568269526	25,5637107751137	131537710550,463	164192511620,328	−0,31991186606333	0,00800363791260094	0,0129946187492937	1,24825429098021	0.31991186606334	0,801118816274797	-0.31991186606334	3469
Q5M8N4-2	sp|Q5M8N4-2|D39U1_MOUSE Isoform 2 of Epimerase family protein SDR39U1 OS=Mus musculus GN=Sdr39u1;sp|Q5M8N4|D39U1_MOUSE Epimerase family protein SDR39U1 OS=Mus musculus GN=Sdr39u1 PE=2 SV=1	1,25	0,194229479910079	0,621726099655746	27,3749832819008	27,6204662728737	27,572598641113	27,7557362941429	28,0044923642566	27,4732502744015	897323905029,382	1120160369008,8	−0,32000454140002	0,00472814987881347	0,00958025984185866	1,24833447847589	0.320004541400034	0,801067355938864	-0.320004541400034	3470
Q8CCT4	sp|Q8CCT4|TCAL5_MOUSE Transcription elongation factor A protein-like 5 OS=Mus musculus GN=Tceal5 PE=1 SV=1	1,25	0,272360331342004	0,693189404859215	28,1677723504312	28,6513548480099	28,586024909645	28,8605637488314	28,7849374021746	28,4258373734941	2310266579139,21	2884711972934,29	−0,32036794642268	0,00921649145891361	0,00809526459578891	1,2486489650078	0.320367946422685	0,80086559795751	-0.320367946422685	3471
Q9R1C6	sp|Q9R1C6|DGKE_MOUSE Diacylglycerol kinase epsilon OS=Mus musculus GN=Dgke PE=2 SV=1	1,25	0,105354501435087	0,468827531386139	25,5847097988631	26,0066860711259	25,8539662924407	26,1388382616424	25,9308965147294	26,0422096112122	162691355919,817	203171197061,94	−0,32055829019155	0,00827628466950372	0,00399646637912900	1,24881371793394	0.320558290191562	0,800759941726474	-0.320558290191562	3472
Q922S4	sp|Q922S4|PDE2A_MOUSE cGMP-dependent 3,5-cyclic phosphodiesterase OS=Mus musculus GN=Pde2a PE=1 SV=3	1,25	0,419164387612823	0,769072485508496	30,6061730946971	30,1496750736804	29,8638697170678	30,3642246304452	30,7513155117469	30,1714331301119	13138561242161,2	16411297585690,6	−0,32088201254248	0,012394928227984	0,00970510767640644	1,24909396723191	0.320882012542474	0,800580281574872	-0.320882012542474	3473

E9PV24-2	sp|E9PV24-2|FIBA_MOUSE Isoform 2 of Fibrinogen alpha chain OS=Mus musculus GN=Fga;sp|E9PV24|FIBA_MOUSE Fibrinogen alpha chain OS=Mus musculus GN=Fga PE=2 SV=1	1,25	0,256453254403951	0,679533754664059	26,6767161700113	27,1289472953639	27,2058942963641	27,4252531095558	27,0626686517635	27,1916972274608	534101956387,469	667322759369,119	−0,321269539754016	0,0105877069821011	0,0067502593485837	1,24942953566904	0.321269539754016	0,800365263867823	-0.321269539754015	3474
Q8C437	sp|Q8C437|PEX5R_MOUSE PEX5-related protein OS=Mus musculus GN=Pex5l PE=1 SV=2;sp|Q8C437-3|PEX5R_MOUSE Isoform 3 of PEX5-related protein OS=Mus musculus GN=Pex5l;sp|Q8C437-4|PEX5R_MOUSE Isoform 4 of PEX5-related protein OS=Mus musculus GN=Pex5l	1,25	0,286269059495814	0,704836107321858	27,4627343947558	27,8588173640719	28,01920864903	28,2148956435959	28,0241091157644	27,7715323088965	1160942706808,64	1451345765526,56	−0,32209448882032	0,0103107475827694	0,0079417943926580	1,25014417767111	0.322094488820325	0,799907736932309	-0.322094488820325	3475
Q9JKN6	sp|Q9JKN6|NOVA1_MOUSE RNA-binding protein Nova-1 OS=Mus musculus GN=Nova1 PE=1 SV=2	1,25	0,300426839126521	0,713946294657213	27,3070559481143	27,9358995293517	27,9281151032895	28,0545669515707	27,8663186708468	27,9203165194517	1097098620743,58	1371693715148,81	−0,322265159987306	0,0130154691054569	0,0034684597092366	1,2502920787733	0.322265159987302	0,79981311325361	-0.322265159987302	3476
Q9Z2B2-2	sp|Q9Z2B2-2|UCP5_MOUSE Isoform 2 of Brain mitochondrial carrier protein 1 OS=Mus musculus GN=Slc25a14;sp|Q9Z2B2|UCP5_MOUSE Brain mitochondrial carrier protein 1 OS=Mus musculus GN=Slc25a14 PE=2 SV=2;sp|Q9CR58|KMCP1_MOUSE Kidney mitochondrial carrier pro	1,25	0,369977744922981	0,74142560009069	23,2969409758378	23,3825283651281	23,7144489157279	23,9115536096075	23,2593273389152	23,8940402301816	15508272356,103	19395502758,4646	−0,32268419600214	0,0093985331797925	0,0156875086599909	1,25065528339344	0.322684196002142	0,799580838363928	-0.322684196002142	3477
Q8K2K6-3	sp|Q8K2K6-3|AGFG1_MOUSE Isoform 3 of Arf-GAP domain and FG repeat-containing protein 1 OS=Mus musculus GN=Agfg1;sp|Q8K2K6-1|AGFG1_MOUSE Isoform 1 of Arf-GAP domain and FG repeat-containing protein 1 OS=Mus musculus GN=Agfg1;sp|Q8K2K6|AGFG1_MOUSE Arf-GAP	1,25	0,0380098909335334	0,284440831260416	28,5498933163116	28,8842531253939	28,7307284512667	28,9871137767746	28,9989754780626	28,8498970986966	2976070625557,83	3722173115595,58	−0,32273639201198	0,00582716661705755	0,0028625964507396	1,25070053231613	0.322736392011981	0,799551910438651	-0.322736392011981	3478
Q8VBT9	sp|Q8VBT9|ASPC1_MOUSE Tether containing UBX domain for GLUT4 OS=Mus musculus GN=Aspscr1 PE=1 SV=1;sp|Q8VBT9-2|ASPC1_MOUSE Isoform 2 of Tether containing UBX domain for GLUT4 OS=Mus musculus GN=Aspscr1;sp|Q8VBT9-3|ASPC1_MOUSE Isoform 3 of Tether containi	1,25	0,263994268342382	0,68394025335819	27,0441476199735	27,6263820326853	27,6333084505179	27,6401569056551	27,7433277429496	27,5915719767911	821676581206,108	1027708012892,86	−0,322787877786116	0,0123264147028828	0,0028019314133541	1,2507451671366	0.322787877786112	0,799523377163518	-0.322787877786113	3479
Q9QZN4	sp|Q9QZN4|FBX6_MOUSE F-box only protein 6 OS=Mus musculus GN=Fbxo6 PE=1 SV=1	1,25	0,432602727267786	0,776120070778056	24,3939467194112	24,817993141014	25,2191352268015	25,3211500311766	24,7814467480862	24,9998552831891	59566451748,4494	74506386797,5756	−0,322864077574758	0,0166320543592687	0,0108444340250062	1,25081123032505	0.32286407757476	0,799481149317895	-0.32286407757476	3480
Q8BFX3	sp|Q8BFX3|KCTD3_MOUSE BTB/POZ domain-containing protein KCTD3 OS=Mus musculus GN=Kctd3 PE=2 SV=1;sp|Q8BFX3-2|KCTD3_MOUSE Isoform 2 of BTB/POZ domain-containing protein KCTD3 OS=Mus musculus GN=Kctd3	1,25	0,28634452123849	0,704836107321858	22,1831864358735	21,9172465363468	21,5346685350988	22,1461242459219	22,2366110073191	21,9237820258192	3174345064,47258	3970557803,17364	−0,322882734755178	0,0149006736508237	0,0072834124133646	1,25082740613562	0.322882734755179	0,79947081035701	-0.322882734755179	3481
Q9CYT6	sp|Q9CYT6|CAP2_MOUSE Adenylyl cyclase-associated protein 2 OS=Mus musculus GN=Cap2 PE=1 SV=1	1,25	0,23598643037432	0,658933495707757	31,8752640211253	32,4315691732345	32,4315691732345	32,5416809718635	32,4315691732345	32,4366499180038	100999241468770	126336069156483	−0,322922131759045	0,00996035456843775	0,0019143328947013	1,25086156409944	0.322922131759049	0,799448978768445	-0.322922131759049	3482
O09117-2	sp|O09117-2|SYPL1_MOUSE Isoform 2 of Synaptophysin-like protein 1 OS=Mus musculus GN=Sypl1;sp|O09117|SYPL1_MOUSE Synaptophysin-like protein 1 OS=Mus musculus GN=Sypl1 PE=1 SV=2	1,25	0,10367548228741	0,465707963539203	23,8999487036671	23,9154891156968	23,8119599107002	24,3651120990611	24,0031450361206	23,9322127236138	23395259266,9346	29279592822,2376	−0,323679274093772	0,0023383447494979	0,0096340829548006	1,25151820239152	0.323679274093773	0,799029529166338	-0.323679274093773	3483
Q91ZJ5	sp|Q91ZJ5|UGPA_MOUSE UTP--glucose-1-phosphate uridylyltransferase OS=Mus musculus GN=Ugp2 PE=2 SV=3;sp|Q91ZJ5-2|UGPA_MOUSE Isoform 2 of UTP--glucose-1-phosphate uridylyltransferase OS=Mus musculus GN=Ugp2	1,25	0,127485367974189	0,511193951851664	30,7450551800597	31,1864065676514	31,1032923983988	31,3310729947621	31,2460357843299	31,1310784705769	29387329129088,3	36783202995248,3	−0,323852866291725	0,00756273780979345	0,0032132598235714	1,25166880030752	0.323852866291728	0,798933391768101	-0.323852866291728	3484
Q811U3	sp|Q811U3|RB6I2_RAT ELKS/Rab6-interacting/CAST family member 1 OS=Rattus norvegicus GN=Erc1 PE=1 SV=1;sp|Q99MI1-2|RB6I2_MOUSE Isoform 2 of ELKS/Rab6-interacting/CAST family member 1 OS=Mus musculus GN=Erc1;sp|Q99MI1|RB6I2_MOUSE ELKS/Rab6-interacting/CAS	1,25	0,424842452860233	0,7711031271846	26,269865312127	26,308372107962	25,6571152896275	26,0782885894433	26,6724274176718	26,1582354818588	211703151845,162	264996864200,767	−0,323932539461139	0,014011374719121	0,0122584934349588	1,25173792591706	0.323932539461136	0,798889271703877	-0.323932539461136	3485
P21460	sp|P21460|CYTC_MOUSE Cystatin-C OS=Mus musculus GN=Cst3 PE=2 SV=2	1,25	0,371328296218577	0,74351830919698	29,4599545956542	29,1969573151545	29,0558930771154	29,0701689048778	29,4785745078136	29,8391442128287	4985733563313,78	6243919390011,84	−0,324646124483321	0,00701405454277452	0,0130584256396686	1,25235721297987	0.32464612448332	0,798494223242098	-0.32464612448332	3486

Q9CR61	sp|Q9CR61|NDUB7_MOUSE NADH dehydrogenase [ubiquinone] 1 beta subcomplex subunit 7 OS=Mus musculus GN=Ndufb7 PE=1 SV=3	1,25	0,355245194232225	0,732843629012038	25,7970224427502	25,3314691252537	25,3144985329333	25,5763604830779	25,4703197299504	26,072361667895	116481313775,734	145923346037,764	−0,32511218345673	0,0107459622059746	0,0125020280212118	1,2527618491 5562	0.325112183456735	0,79823631336955	-0,325112183456735	3487
Q8C6G1	sp|Q8C6G1|CU002_MOUSE Protein C21orf2 homolog OS=Mus musculus PE=2 SV=1	1,25	0,353276582240447	0,732843629012038	23,2244008906573	23,7679115608697	23,2715183887361	23,9242273809443	23,6787354581344	23,3379615440826	14850166982,6432	18610183994,0076	−0,32561316551915	0,0128565743368001	0,0124506646909899	1,25319695164095	0.325613165519157	0,797959170496374	-0,325613165519157	3488
P16388	sp|P16388|KCNA1_MOUSE Potassium voltage-gated channel subfamily A member 1 OS=Mus musculus GN=Kcna1 PE=1 SV=1	1,25	0,152508592333076	0,554232586549105	23,9566725543437	24,361859950797	24,2064354818055	24,2731664169751	24,6092541358523	24,3206206984587	31554729795,6743	39557207428,72	−0,326084311940852	0,0084556761172342 5	0,0074454292901277	1,25360627978322	0.326084311940854	0,797698620473507	-0,326084311940854	3489
Q9DCC8	sp|Q9DCC8|TOM20_MOUSE Mitochondrial import receptor subunit TOM20 homolog OS=Mus musculus GN=Tomm20 PE=1 SV=1	1,25	0,017391536361827	0,179002683778532	26,2900150804149	26,2580903346101	26,1926661523628	26,3128537120198	26,4796440426437	26,6267436086175	250549879095,58	314132420228,037	−0,32627500330935	0,0018907286602584 9	0,0059323602580773	1,25377198888291	0.32627500330935	0,797593189883742	-0,32627500330935	3490
Q9Z1W9	sp|Q9Z1W9|STK39_MOUSE STE20/SPS1-related proline-alanine-rich protein kinase OS=Mus musculus GN=Stk39 PE=1 SV=1	1,25	0,157041948256315	0,561270396169617	28,7849374021746	29,1461064414382	29,1446312403025	29,4521853631682	29,0951539669868	29,2069428457707	4031763656224,43	5055143683209,88	−0,32634102878521	0,0071695049797573 3	0,0062432963025881	1,25382936953795	0.326341028785214	0,797556688569605	-0,326341028785214	3491
Q9QZ73	sp|Q9QZ73|DCNL1_MOUSE DCN1-like protein 1 OS=Mus musculus GN=Dcun1d1 PE=2 SV=1	1,25	0,205876256721617	0,636561532193065	27,9581743773589	28,344311301084	28,5146694615578	28,6310783417307	28,3918361374394	28,4734319680636	1899069312691,62	2381572617544,49	−0,32662197691993	0,0100852683360041	0,0042675157886214	1,25407356204867	0.326621976919939	0,797401388772116	-0,326621976919939	3492
Q99JR1	sp|Q99JR1|SFXN1_MOUSE Sideroflexin-1 OS=Mus musculus GN=Sfxn1 PE=1 SV=3	1,25	0,1829012877 8858	0,602896837525319	26,6810725947884	26,9928732615539	27,2041523196231	27,039238096365	27,2974019823772	27,2212697752775	510862371236,34	640791489360,6	−0,32692031222238	0,0097607907318941 3	0,0048794498503737	1,2543329190792	0.326920312222387	0,797236510968791	-0,326920312222387	3493
Q8VCD6	sp|Q8VCD6|REEP2_MOUSE Receptor expression-enhancing protein 2 OS=Mus musculus GN=Reep2 PE=2 SV=2	1,25	0,345752688055248	0,730405757239477	28,7755965557594	29,3052903367405	29,383338090787	29,5856163544791	29,0984836692739	29,4599545956542	4589268050191,21	5756504448292,75	−0,32692894822673	0,0113415557994317	0,0086074994581630	1,2543404275662	0.326928948226734	0,79723173870773	-0,326928948226733	3494
Q9DD18	sp|Q9DD18|DTD1_MOUSE D-tyrosyl-tRNA(Tyr) deacylase 1 OS=Mus musculus GN=Dtd1 PE=1 SV=2;sp|Q9DD18-2|DTD1_MOUSE Isoform 2 of D-tyrosyl-tRNA(Tyr) deacylase 1 OS=Mus musculus GN=Dtd1	1,25	0,203760376215914	0,635648869851434	28,8212517944698	29,2873491997899	29,3963002344319	29,441616430095	29,3963002344319	29,3473309859604	4651916539118,58	5836092230382,82	−0,32717746960371	0,01047198122593	0,0016041633898259	1,25455652123299	0.327177469603719	0,797094417888155	-0,327177469603719	3495
O70311	sp|O70311|NMT2_MOUSE Glycylpeptide N-tetradecanoyltransferase 2 OS=Mus musculus GN=Nmt2 PE=2 SV=1	1,25	0,261751721678403	0,682806179310143	26,9717991804572	27,5987969361743	27,4208032405863	27,6263820326853	27,401725558271	27,6440499937839	740407429773,691	929010484585,763	−0,32737550629700	0,0118224522578781	0,0049023125114309	1,2547287442398	0.327375506297006	0,79698500938214	-0,327375506297006	3496
Q91YM4-2	sp|Q91YM4-2|TBRG4_MOUSE Isoform 2 of Protein TBRG4 OS=Mus musculus GN=Tbrg4;sp|Q91YM4|TBRG4_MOUSE Protein TBRG4 OS=Mus musculus GN=Tbrg4 PE=2 SV=1	1,25	0,10103803268708	0,46324407107743	25,7943002713021	25,6315175429121	26,102075650852	26,062710168417	26,0548622267635	26,0912494688688	167229198582,693	209839184880,5	−0,32745734147109	0,0092472430979553 7	0,0007345348466281	1,2547999192661 1	0.327457341471089	0,796939802629939	-0,327457341471089	3497
P70122	sp|P70122|SBDS_MOUSE Ribosome maturation protein SBDS OS=Mus musculus GN=Sbds PE=1 SV=4	1,25	0,264708038383647	0,68394025335819	27,7875488622476	28,2910319082209	28,4428094286615	28,4918017637118	28,3357637084218	28,3747558938646	1720776456422,24	2159232150761,66	−0,32745867254251	0,0121744674969158	0,0028593430064476	1,2548010769807	0.327458672542519	0,796939067350976	-0,327458672542519	3498
Q8BVI4	sp|Q8BVI4|DHPR_MOUSE Dihydropteridine reductase OS=Mus musculus GN=Qdpr PE=1 SV=2	1,25	0,395386954535092	0,753881747531964	30,7768742173066	31,4086456757793	31,6337004171238	31,6424407003006	31,3898117118546	31,4680190706971	38170093671236,5	47897790612617,5	−0,32751638312102	0,014204237131021 1	0,0041057681162580	1,25485127244294	0.327516383121023	0,796907188891955	-0,327516383121023	3499
Q99MK8	sp|Q99MK8|ARBK1_MOUSE Beta-adrenergic receptor kinase 1 OS=Mus musculus GN=Adrbk1 PE=1 SV=2	1,26	0,155239950407891	0,559233440397952	29,5542026275958	29,3002269215392	29,0218456153508	29,5382706477389	29,5856163544791	29,4348108568803	5264944507091,24	6609743426200,29	−0,32817594576919	0,0090902258096887 2	0,0026126098109456	1,255425088963	0.328175945769195	0,796542946920085	-0,328175945769195	3500
Q9DCT1	sp|Q9DCT1|AKCL2_MOUSE 1,5-anhydro-D-fructose reductase OS=Mus musculus GN=Akr1e2 PE=1 SV=1	1,26	0,262354884006858	0,682806179310143	27,3379076999531	27,860378365634	27,9479032340272	28,0831095688452	27,889164479335	27,8568791263886	1088037176335,73	1366195599402,34	−0,32843619850106	0,0119006399617057	0,0043790387360881	1,25565157985078	0.328436198501058	0,796399268751638	-0,328436198501058	3501
Q64444	sp|Q64444|CAH4_MOUSE Carbonic anhydrase 4 OS=Mus musculus GN=Ca4 PE=1 SV=1	1,26	0,377505702895987	0,74727992066734	24,5589890746974	25,2380141227444	25,2978380035958	25,343368132376	25,3783577930722	25,0561523437009	74317607100,0516	93319297516,1302	−0,32847139696921	0,0163951269766625	0,0069991074530098	1,25568221525886	0.328471396969217	0,796379838663121	-0,328471396969218	3502
P60487	sp|P60487|PLPP_MOUSE Pyridoxal phosphate phosphatase OS=Mus musculus GN=Pdxp PE=1 SV=1	1,26	0,276995301460758	0,699203439845458	31,209001882346	31,7285609258969	31,8676070981556	31,8989697166028	31,7073332672964	31,8823889345278	53021748911752,7	66589230274518,3	−0,32870460569727	0,010984837831226 2	0,0033358623811738	1,25588521014927	0.328704605697278	0,79625111588116	-0,328704605697278	3503
Q9DB75-2	sp|Q9DB75-2|CDIP1_MOUSE Isoform 2 of Cell death-inducing p53-target protein 1 OS=Mus musculus GN=Cdip1;sp|Q9DB75|CDIP1_MOUSE Cell death-inducing p53-target protein 1 OS=Mus musculus GN=Cdip1 PE=2 SV=1	1,26	0,2576322087565 3	0,67968190098123	25,1428879762118	25,7876482760269	25,6404580141254	25,8054283782832	25,6522026352665	25,7970224427502	121558899942,858	152671005470,328	−0,32877057432617	0,0132377117836527	0,0033450775485264	1,25594263803058	0.328770574326177	0,796214707359632	-0,328770574326177	3504
Q91VE0	sp|Q91VE0|S27A4_MOUSE Long-chain fatty acid transport protein 4 OS=Mus musculus GN=Slc27a4 PE=1 SV=1	1,26	0,460903570433422	0,792808510675944	26,8602290837401	27,6623996166401	27,6204662728737	27,918409250953	27,3476415937145	27,5612721540117	778808993094,505	978324905699,041	−0,32904412637280	0,0164900773081113	0,0104449207433375	1,25618080219103	0.329044126372805	0,796063749944119	-0,329044126372805	3505
P15208	sp|P15208|INSR_MOUSE Insulin receptor OS=Mus musculus GN=Insr PE=1 SV=2	1,26	0,2508536502797	0,673538059877323	22,7144546071874	22,7337848548684	22,1414207633023	22,6991159284757	22,7369952893588	22,8379813625276	6089834495,03649	7650454234,80434	−0,32914238812942	0,0149383890304589	0,0031541754666354	1,25626636340279	0.32914238812942	0,796009532000323	-0,32914238812942	3506

Q9ESJ4	sp|Q9ESJ4|SPN90_MOUSE NCK-interacting protein with SH3 domain OS=Mus musculus GN=Nckipsd PE=2 SV=2	1,26	0,207984544736336	0,638546806637671	29,5350096320515	29,1339128359005	28,9317927292942	29,5056687085734	29,3922159985882	29,3880565211407	4802887421260,76	6035302395008,83	−0,32952406563088	0,0105146149344665	0,0022676919072372	1,2565987635464	0.329524065630879	0,795798968620483	-0.329524065630879	3507
Q64520	sp|Q64520|KGUA_MOUSE Guanylate kinase OS=Mus musculus GN=Guk1 PE=1 SV=2	1,26	0,0097163652554862	0,120498857635661	29,1855954237581	29,2370422808287	29,183067170165	29,258547537147	29,497763870862	29,5350096320515	4810882354550,24	6046135035296,69	−0,329711680548072	0,00104304660448976	0,0050975751704404	1,2567621882456	0.329711680548071	0,795695485870696	-0.329711680548071	3508
Q9DBN5-2	sp|Q9DBN5-2|LONP2_MOUSE Isoform 2 of Lon protease homolog 2, peroxisomal OS=Mus musculus GN=Lonp2;sp|Q9DBN5|LONP2_MOUSE Lon protease homolog 2, peroxisomal OS=Mus musculus GN=Lonp2 PE=2 SV=1;sp|Q9DBN5-3|LONP2_MOUSE Isoform 3 of Lon protease homolog 2, p	1,26	0,801776407144537	0,945787729921946	23,3607684831536	22,3631795486139	20,4319780326739	22,7620598330115	21,7051251708901	22,376705503092	3776167165,64984	4749460338,84845	−0,330840963192649	0,0675218146832891	0,0240061287313411	1,25774631537826	0.330840963192646	0,795072891705715	-0.330840963192646	3509
Q6ZWV3	sp|Q6ZWV3|RL10_MOUSE 60S ribosomal protein L10 OS=Mus musculus GN=Rpl10 PE=1 SV=3;sp|P86048|RL10L_MOUSE 60S ribosomal protein L10-like OS=Mus musculus GN=Rpl10l PE=2 SV=1	1,26	0,276103886758191	0,698570536229335	24,8315460515407	25,4287308522879	25,1630860770961	25,4079277565894	25,1454481767251	25,5584985256053	82918247846,5711	104309139238,022	−0,331104031632914	0,0119007307196647	0,0082393142515347	1,25797568022701	0.331104031632912	0,794927927239058	-0.331104031632912	3510
P84099	sp|P84099|RL19_MOUSE 60S ribosomal protein L19 OS=Mus musculus GN=Rpl19 PE=1 SV=1	1,26	0,197512394048047	0,626240510020104	25,9119492395323	26,4067100219599	26,1771723436785	26,6055017936493	26,3128537120198	26,2659948811303	230906153201,407	290475032315,682	−0,33110754393823	0,00946272365140269	0,0069705183510290	1,25797874282855	0.331107543938232	0,794925991953977	-0.331107543938232	3511
Q9Z108	sp|Q9Z108|STAU1_MOUSE Double-stranded RNA-binding protein Staufen homolog 1 OS=Mus musculus GN=Stau1 PE=2 SV=1	1,26	0,020792066940716	0,199524718486067	22,1514329050445	22,3810757628557	22,4664435194351	22,5214498237658	22,5407241994077	22,6264019676859	5001401761,77987	6293975000,47799	−0,331638947141088	0,00729498505983209	0,0024760870650973	1,25844219286197	0.331638947141088	0,794633242330966	-0.331638947141088	3512
Q8K215	sp|Q8K215|LYRM4_MOUSE LYR motif-containing protein 4 OS=Mus musculus GN=Lyrm4 PE=1 SV=1	1,26	0,373288018239112	0,744143356508855	26,7618369074011	26,2659948811303	25,9967084867646	26,3533892025814	26,5628276441229	26,7982568071736	275406334784,47	346618718198,72	−0,33178782127461	0,0147339385253155	0,0083759057242820	1,25857206033398	0.33178782127461	0,794551247017701	-0.33178782127461	3513
Q91VM5	sp|Q91VM5|RMXL1_MOUSE RNA binding motif protein, X-linked-like-1 OS=Mus musculus GN=Rbmxl1 PE=1 SV=1	1,26	0,0714432267067884	0,398379368577534	28,046173766494	27,6568502118367	27,749949888637	28,1207709623458	27,9512595440036	28,0716553884336	1205189528609,05	1517211608899,96	−0,332162272403832	0,00730836099012691	0,0031096006773659	1,25889876478683	0.332162272403825	0,794345048205149	-0.332162272403825	3514
P62774	sp|P62774|MTPN_MOUSE Myotrophin OS=Mus musculus GN=Mtpn PE=1 SV=2	1,26	0,0844549927094076	0,432177240031568	30,6742077219904	30,5591116316026	30,4045709486719	30,5824842469731	30,7896398570902	30,9568503354048	18447748271988,1	23226728295255,6	−0,332341219193337	0,00442933770798227	0,0060935764816633	1,25905492382093	0.332341219193342	0,794246526565531	-0.332341219193342	3515
Q15831-2	sp|Q15831-2|STK11_HUMAN Isoform 2 of Serine/threonine-protein kinase STK11 OS=Homo sapiens GN=STK11;sp|Q9WTK7-2|STK11_MOUSE Isoform 2 of Serine/threonine-protein kinase STK11 OS=Mus musculus GN=Stk11;sp|Q15831|STK11_HUMAN Serine/threonine-protein kinase	1,26	0,510810290939886	0,817055380454204	22,9990398942442	23,8690116229415	23,985379748144	24,0597088731534	23,9524385927456	23,5324035752081	18075267815,3787	22758025295,254	−0,332358357724826	0,02282262833262	0,011685735805347	1,25906988088393	0.332358357724825	0,794237091350283	-0.332358357724825	3516
Q9WTP7	sp|Q9WTP7|KAD3_MOUSE GTP:AMP phosphotransferase AK3, mitochondrial OS=Mus musculus GN=Ak3 PE=1 SV=3	1,26	0,333889338194023	0,72953198881961	29,8660763732758	30,4762511723909	30,6173890438023	30,6703686802763	30,4692900893302	30,5115424211656	14715250631090,2	18529782101419,1	−0,332533801717006	0,0131699857839475	0,0034703874859165	1,25922300380461	0.332533801717006	0,794140511234789	-0.332533801717006	3517
Q9D1R2	sp|Q9D1R2|KTI12_MOUSE Protein KTI12 homolog OS=Mus musculus GN=Kti12 PE=1 SV=1	1,26	0,583077353618034	0,853738099378148	23,5758934844069	23,2303027245149	22,2381882530085	23,493812176176	23,0900203571021	23,1523256748072	9890047848,55291	12454976132,5214	−0,332672850998328	0,030172058960477	0,0093518371529961	1,25934437560318	0.332672850998324	0,794063974376381	-0.332672850998324	3518
O08917	sp|O08917|FLOT1_MOUSE Flotillin-1 OS=Mus musculus GN=Flot1 PE=1 SV=1	1,26	0,050550924821293	0,33109761582085	28,8233279788742	28,4585732117269	28,6558298268788	28,9252452807151	28,9338137105631	28,7707322599102	2759057740945,93	3474935674120,32	−0,332810622389216	0,00637367042259506	0,0031784102208477	1,25946464350868	0.332810622389214	0,793988148181875	-0.332810622389214	3519
Q9JJL8	sp|Q9JJL8|SYSM_MOUSE Serine--tRNA ligase, mitochondrial OS=Mus musculus GN=Sars2 PE=1 SV=2	1,26	0,525097587652686	0,826714516252356	25,1686290907268	26,1820526790869	26,1892833237637	26,0089907009257	25,8942049257386	26,3290904224167	167903463840,023	211486855174,687	−0,332936003069567	0,0227185613708077	0,0086425550445324	1,25957410489274	0.332936003069565	0,793919147841768	-0.332936003069566	3520
Q9CQC8	sp|Q9CQC8|SPG21_MOUSE Maspardin OS=Mus musculus GN=Spg21 PE=1 SV=1;sp|Q9CQC8-2|SPG21_MOUSE Isoform 2 of Maspardin OS=Mus musculus GN=Spg21	1,26	0,283765432502412	0,704836107321858	24,1206114949404	24,0965663332202	24,4006943482069	24,6713112340392	24,0558013103755	24,5842883547047	32547206891,1205	41012126697,9744	−0,33351681634272	0,00698485667461055	0,0136307960345166	1,26008129776455	0.333516816342721	0,793599588990054	-0.333516816342721	3521
P13634	sp|P13634|CAH1_MOUSE Carbonic anhydrase 1 OS=Mus musculus GN=Ca1 PE=2 SV=4	1,26	0,278291645616674	0,699221280539289	26,4229344891842	26,8570170371907	27,0832508388608	27,1479973377852	26,8468256315125	27,0626686517635	430293823068,159	542342671411,954	−0,333882555440638	0,0125268511517269	0,0057450915434501	1,26040078276012	0.333882555440638	0,793398428244484	-0.333882555440638	3522
Q3TW96	sp|Q3TW96|UAP1L_MOUSE UDP-N-acetylhexosamine pyrophosphorylase-like protein 1 OS=Mus musculus GN=Uap1l1 PE=2 SV=1;sp|Q3TW96-2|UAP1L_MOUSE Isoform 2 of UDP-N-acetylhexosamine pyrophosphorylase-like protein 1 OS=Mus musculus GN=Uap1l1	1,26	0,286599817837217	0,70508215347595	26,8914057622921	27,6110235759658	27,4305707381541	27,5128434547509	27,5920034587656	27,5237275215483	726133722419,495	915611646503,141	−0,334500559266026	0,0137092488474217	0,0015578484855344	1,26094081328753	0.334500559266025	0,793058634840119	-0.334500559266026	3523
Q8R2E9	sp|Q8R2E9|ERO1B_MOUSE ERO1-like protein beta OS=Mus musculus GN=Ero1lb PE=1 SV=1	1,26	0,349633438468661	0,732843629012038	21,1169734730192	21,2458172816804	21,5318623747088	21,7258576307335	21,0988721291394	21,7674627603317	1777045000,75606	2242216763,61187	−0,335445539975461	0,00997032130477916	0,0173973985341228	1,26176701358598	0.335445539975461	0,792539343026546	-0.335445539975461	3524

O09114	sp|O09114|PTGDS_MOUSE Prostaglandin-H2 D-isomerase OS=Mus musculus GN=Ptgds PE=1 SV=1;sp|O09114-2|PTGDS_MOUSE Isoform 2 of Prostaglandin-H2 D-isomerase OS=Mus musculus GN=Ptgds	1,26	0,313699458552212	0,722049615805523	28,4367014913091	29,0789957161334	29,0120311248533	29,2745375866608	28,9001884639333	29,0513152204935	3358703360348,97	4238993989371,32	−0,335817537927847	0,0122419309844535	0,0064772425994339	1,26209240131611	0.335817537927846	0,792335013630698	-0.335817537927846	3525
P56564	sp|P56564|EAA1_MOUSE Excitatory amino acid transporter 1 OS=Mus musculus GN=Slc1a3 PE=1 SV=2	1,26	0,0225077464444021	0,210337162518039	30,3043185118729	30,2374046939875	30,1784256328546	30,5331676346361	30,59946784483	30,2860766360239	13585845748248,7	17148023558493,9	−0,335937925050127	0,00208293645058102	0,0054198435727737	1,26219772226579	0.335937925050131	0,792268899206127	-0.335937925050131	3526
Q8C878	sp|Q8C878|UBA3_MOUSE NEDD8-activating enzyme E1 catalytic subunit OS=Mus musculus GN=Uba3 PE=1 SV=2;sp|Q8C878-2|UBA3_MOUSE Isoform 2 of NEDD8-activating enzyme E1 catalytic subunit OS=Mus musculus GN=Uba3	1,26	0,0950255672847004	0,451645078434822	28,6442862543706	28,5934970858939	28,7636310588687	28,7908543389162	28,7501446235731	29,1591429707384	2818252514368,65	3557386674673,75	−0,336016916123577	0,00304664871882264	0,0077960001545009	1,26226683256262	0.336016916123577	0,792225521738402	-0.336016916123577	3527
Q8CDN6	sp|Q8CDN6|TXNL1_MOUSE Thioredoxin-like protein 1 OS=Mus musculus GN=Txnl1 PE=2 SV=3	1,26	0,124890687203929	0,505239598234075	30,108137787763	30,2413049199915	30,59946784483	30,620839005307	30,4856864089267	30,5414934586332	14662341464336,3	18510136637159,7	−0,336200035571804	0,00838182917157449	0,0022231925972305	1,26242706065621	0.33620003557181	0,792124971941113	-0.33620003557181	3528
Q9CPX7	sp|Q9CPX7|RT16_MOUSE 28S ribosomal protein S16, mitochondrial OS=Mus musculus GN=Mrps16 PE=2 SV=1	1,26	0,324910161593527	0,726844368394785	25,0309060428071	24,4023850233445	25,0535866622164	24,9340519903573	24,9865539749528	25,266100588418	60684809163,2474	76628546800,9755	−0,336546525272676	0,0148857680373758	0,0071220002923987	1,26273029210388	0.336546525272677	0,79193475142967	-0.336546525272677	3529
O54781	sp|O54781|SRPK2_MOUSE SRSF protein kinase 2 OS=Mus musculus GN=Srpk2 PE=1 SV=2	1,26	0,19983495039148	0,630334148594112	28,5178789364239	28,4562699090736	28,1252116374367	28,468006362801	28,8699406290185	28,4613395369887	2086383904806,08	2634625536056,1	−0,336593278457142	0,00744472188140479	0,0081820661899188	1,26277121386295	0.33659327845714	0,791909087744321	-0.33659327845714	3530
Q9CZX9	sp|Q9CZX9|EMC4_MOUSE ER membrane protein complex subunit 4 OS=Mus musculus GN=Emc4 PE=2 SV=1	1,26	0,534749658008997	0,8300431463043	24,9101751598063	24,6425366875427	23,835941088441	24,3139660285988	24,9261116536089	24,8485211097813	42081931792,1376	53140202997,9293	−0,336602805209732	0,0228589845094031	0,013495582017172	1,26277955252657	0.336602805209731	0,791903858436096	-0.336602805209731	3531
Q920N7	sp|Q920N7|SYT12_MOUSE Synaptotagmin-12 OS=Mus musculus GN=Syt12 PE=2 SV=1	1,26	0,0739468681955582	0,404916706615332	26,7995725862334	27,1705289713067	27,2015892625047	27,2883154174475	27,3269456084881	27,2571389591788	563385679094,646	711612952874,607	−0,336969879183798	0,00826684354492898	0,0012813690179794	1,2631008903495	0.336969879183799	0,791702394987068	-0.336969879183798	3532
Q8K0Z7	sp|Q8K0Z7|TACO1_MOUSE Translational activator of cytochrome c oxidase 1 OS=Mus musculus GN=Taco1 PE=1 SV=1	1,26	0,0741584672489311	0,405033911033557	27,576491819139	27,9268847299193	27,9932539266564	28,0805683689805	28,0252316570059	28,092191112154	1222855921889,55	1544925872320,73	−0,337283183185354	0,0080457519624904	0,0012748155957098	1,26337522243301	0.337283183185352	0,791530482982087	-0.337283183185352	3533
Q9CY18	sp|Q9CY18|SNX7_MOUSE Sorting nexin-7 OS=Mus musculus GN=Snx7 PE=2 SV=1	1,26	0,0198458266720286	0,1954017511157	25,35089230623	25,484150064461	25,6777150180066	25,773705472882	25,6721471841847	25,769120591376	119221919476,649	150664962340,549	−0,337694446162552	0,00644347808215421	0,0022284665263667	1,26373541880492	0.337694446162556	0,791304876890826	-0.337694446162556	3534
Q61074	sp|Q61074|PPM1G_MOUSE Protein phosphatase 1G OS=Mus musculus GN=Ppm1g PE=1 SV=3	1,26	0,0918316858383124	0,445915480582476	27,4684064394126	27,8950448565735	27,8507513155764	28,0347736742627	27,9465752622869	27,9358995293517	1112985919487,74	1406908915512,57	−0,338093589190709	0,00845701083629844	0,0019399954868498	1,26408509836325	0.338093589190708	0,791085980916009	-0.338093589190708	3535
Q8C5L3-3	sp|Q8C5L3-3|CNOT2_MOUSE Isoform 3 of CCR4-NOT transcription complex subunit 2 OS=Mus musculus GN=Cnot2;sp|Q9NZN8-5|CNOT2_HUMAN Isoform 5 of CCR4-NOT transcription complex subunit 2 OS=Homo sapiens GN=CNOT2;sp|Q8C5L3-2|CNOT2_MOUSE Isoform 2 of CCR4-NOT t	1,26	0,417129518790503	0,767877733621866	23,0076169888695	23,775612781693	23,750582547653	23,4769036420011	23,804353255327	23,9556768269568	16248570501,8948	20540093114,498	−0,338129921893141	0,0185594596475392	0,0103061561854431	1,26411693336978	0.338129921893144	0,791066058528524	-0.338129921893144	3536
Q8BQ47	sp|Q8BQ47|CNPY4_MOUSE Protein canopy homolog 4 OS=Mus musculus GN=Cnpy4 PE=1 SV=1	1,26	0,434998389720295	0,777651709704773	23,936307566344	24,6867200139736	24,8859998394022	24,8983311212619	24,6713112340392	24,6425366875427	43804795730,8354	55374941799,219	−0,338144453224609	0,0204379180359754	0,0056641818098914	1,26412966606392	0.338144453224609	0,791058090673302	-0.338144453224609	3537
Q99PT1	sp|Q99PT1|GDIR1_MOUSE Rho GDP-dissociation inhibitor 1 OS=Mus musculus GN=Arhgdia PE=1 SV=3	1,27	0,289535369373986	0,708855938867559	32,4315691732345	32,8606940502048	33,1779674945783	33,1617830500677	32,9992079572545	33,0157551936444	179897838056494	227669333453733	−0,339762127853956	0,0114123835407021	0,0027063612569743	1,26554791271164	0.339762127853958	0,790171584936154	-0.339762127853958	3538
Q80WW9	sp|Q80WW9|DDRGK_MOUSE DDRGK domain-containing protein 1 OS=Mus musculus GN=Ddrgk1 PE=1 SV=2	1,27	0,133760237587256	0,518639007654437	24,6754463944538	24,197055235212	24,5790747392146	24,758319535241	24,7940826638887	24,605749863721	42973899914,738	54386620885,2177	−0,339791083234562	0,0103337487620255	0,0040462762203666	1,26557331294397	0.339791083234563	0,790155726082595	-0.339791083234563	3539
O88441	sp|O88441|MTX2_MOUSE Metaxin-2 OS=Mus musculus GN=Mtx2 PE=1 SV=1	1,27	0,687393234078502	0,907791064731545	23,4130659507513	24,3939467194112	22,8730078302888	24,0742215615509	24,2731664169751	23,0407523644372	17060075993,4932	21601892876,9205	−0,34053366171561	0,0327267782967089	0,0278039678696787	1,26622489168041	0.340533661715615	0,789749124796384	-0.340533661715615	3540
Q9CQJ4	sp|Q9CQJ4|RING2_MOUSE E3 ubiquitin-protein ligase RING2 OS=Mus musculus GN=Rnf2 PE=1 SV=1	1,27	0,341602613286776	0,729721949431128	22,1382342375959	21,5846616401495	21,4141158051472	21,8445984352844	22,1831864358735	21,8174060215298	2688736526,93516	3404612491,10699	−0,340562211343973	0,017435552527682	0,0092848560032633	1,26624994937226	0.340562211343971	0,789733496531035	-0.340562211343971	3541
Q03265	sp|Q03265|ATPA_MOUSE ATP synthase subunit alpha, mitochondrial OS=Mus musculus GN=Atp5a1 PE=1 SV=1	1,27	0,213725339791338	0,645012843958033	33,597564845353	34,1909777177394	34,1391253660482	34,172245247307	34,2388631383902	34,2285718437873	569562312915047	722130324342218	−0,34240553825271	0,00967341186286739	0,0010481939159853	1,26786886696615	0.342405538252711	0,788725100879618	-0.342405538252711	3542
Q7TMF3	sp|Q7TMF3|NDUAC_MOUSE NADH dehydrogenase [ubiquinone] 1 alpha subcomplex subunit 12 OS=Mus musculus GN=Ndufa12 PE=2 SV=2	1,27	0,238152597463106	0,662768019549379	26,1422103225398	25,769120591376	25,7705950123853	25,8337781042129	26,1853542778432	26,3762555589271	176039688387,95	223303122768,547	−0,343102703481517	0,00830228226854314	0,0105303122811219	1,26848169758424	0.343102703481515	0,788344050926751	-0.343102703481515	3543
Q9ERT9	sp|Q9ERT9|PPR1A_MOUSE Protein phosphatase 1 regulatory subunit 1A OS=Mus musculus GN=Ppp1r1a PE=2 SV=1	1,27	0,53367687561832	0,8300431463043	23,6005714162421	23,8072085417944	24,5212483266218	24,5509061141610	23,6978719068565	24,3957976508923	25869817058,5728	32829282823,1898	−0,34371338178098	0,0201481935665025	0,0187527236020049	1,26901874678355	0.343713381780982	0,788010423435111	-0.343713381780982	3544
O35963	sp|O35963|RB33B_MOUSE Ras-related protein Rab-33B OS=Mus musculus GN=Rab33b PE=1 SV=1	1,27	0,369172399954707	0,740483034506945	24,4861190808891	25,0006538060705	25,30131699854	25,4023621789469	25,0503231987898	25,0503231987898	67094190347,1909	85149087320,6758	−0,343803216727779	0,0165366559453771	0,0080758291804866	1,26909776956927	0.343803216727779	0,787961356467754	-0.343803216727779	3545

Q9WV54	sp|Q9WV54|ASAH1_MOUSE Acid ceramidase OS=Mus musculus GN=Asah1 PE=1 SV=1	1,27	0,000316895967959855	0,00958927199046521	29,0578855856183	28,8498970986966	28,9668367465018	29,2449656239036	29,1969573151545	29,1488133739494	3770416078463,71	4786938142770,83	−0,34437942487787	0,0036004465875876	0,0016466173117512	1,26960474471595	0.344379424877872	0,787646709861447	-0.344379424877872	3546
P10833	sp|P10833|RRAS_MOUSE Ras-related protein R-Ras OS=Mus musculus GN=Rras PE=2 SV=1	1,27	0,29372115443407 2	0,711969538636193	26,1288765661872	26,4330141857912	26,8802456280836	26,5989713295845	26,7376285098563	26,8229841428725	316538947137,726	402057651686,262	−0,34501936595523	0,014272601685162 4	0,0042312475012260	1,27016803247067	0.345019365955238	0,787297408245152	-0.345019365955238	3547
Q8C3X4-2	sp|Q8C3X4-2|GUF1_MOUSE Isoform 2 of Translation factor Guf1, mitochondrial OS=Mus musculus GN=Guf1;sp|Q8C3X4|GUF1_MOUSE Translation factor Guf1, mitochondrial OS=Mus musculus GN=Guf1 PE=2 SV=1	1,27	0,39842140665618 8	0,754338506152154	21,9746816952802	22,2381882530085	22,0605658022871	21,7873155101343	22,5552010672857	22,6504567909222	3927014285,07618	4991444195,54168	−0,34602445096159	0,0060833624033044 6	0,0211920588110948	1,27105323107956	0.34602445096159	0,786749111326089	-0.34602445096159	3548
Q8K021	sp|Q8K021|SCAM1_MOUSE Secretory carrier-associated membrane protein 1 OS=Mus musculus GN=Scamp1 PE=1 SV=1	1,27	0,401856366981143	0,757171461074058	29,3699426896834	29,9229385752534	30,1173421092281	30,1964189422983	29,6878677659393	30,2457298105003	8779212186742,21	11159796518200,3	−0,34614733338048	0,0130109977369995	0,0102795740896771	1,27116149841475	0.346147333380493	0,786682102350554	-0.346147333380493	3549
Q9D0R8	sp|Q9D0R8|LSM12_MOUSE Protein LSM12 homolog OS=Mus musculus GN=Lsm12 PE=1 SV=1	1,27	0,2806247568750 9	0,700250561548121	26,8777286255423	27,386252220826	27,5334727094064	27,6999254581679	27,355199209797	27,4646552402382	694055560027,224	883002000159,733	−0,34736554885064	0,0126187899355928	0,0064038494977438	1,27223532381917	0.347365548850641	0,786018106302897	-0.347365548850641	3550
Q9JHW4	sp|Q9JHW4|SELB_MOUSE Selenocysteine-specific elongation factor OS=Mus musculus GN=Eefsec PE=2 SV=2	1,27	0,259718215687864	0,682211215860657	26,0548622267635	26,4042232161596	26,7663350011133	26,7646133507678	26,6173376487642	26,5664270729435	294478985614,826	374725411046,854	−0,34766912844061	0,0134712606505585	0,0038621336695973	1,27250306253428	0.347669128440617	0,785852725578852	-0.347669128440617	3551
Q80TN4	sp|Q80TN4|DJC16_MOUSE DnaJ homolog subfamily C member 16 OS=Mus musculus GN=Dnajc16 PE=1 SV=2	1,27	0,394250179027514	0,753881747531964	25,2247645740049	25,3412575352839	24,6502909577635	25,2687133583084	25,6555739842145	25,0158762888834	77388525382,8356	98506451174,3293	−0,34809853984607	0,0147540711414756	0,0127276641197482	1,27288187346929	0.34809853984608	0,78561885501163	-0.348098539846079	3552
Q62418	sp|Q62418|DBNL_MOUSE Drebrin-like protein OS=Mus musculus GN=Dbnl PE=1 SV=2;sp|Q62418-3|DBNL_MOUSE Isoform 3 of Drebrin-like protein OS=Mus musculus GN=Dbnl;sp|Q62418-2|DBNL_MOUSE Isoform 2 of Drebrin-like protein OS=Mus musculus GN=Dbnl	1,27	0,23400821582982 9	0,657279264635888	29,9807891911943	30,5719607650948	30,5694328473646	30,7077418394737	30,6034495341565	30,5354699767686	15534134348325,4	19777257521905,8	−0,34840053553983	0,0112130434728395	0,0028342274500615	1,27314835049291	0.348400535539832	0,78545442062022	-0.348400535539832	3553
O70503	sp|O70503|DHB12_MOUSE Estradiol 17-beta-dehydrogenase 12 OS=Mus musculus GN=Hsd17b12 PE=2 SV=1;sp|O70503-2|DHB12_MOUSE Isoform 2 of Estradiol 17-beta-dehydrogenase 12 OS=Mus musculus GN=Hsd17b12	1,27	0,248734834049607	0,673464050610029	29,2041525384792	29,5542026275958	29,9201519045597	29,8638697170678	29,7887479080092	29,7503945006217	6879054677300,73	8758134502821,69	−0,34841328334659	0,012112149196957 6	0,0019369106866443	1,2731596002168	0.348413283346596	0,785447480292115	-0.348413283346596	3554
Q9D9V7	sp|Q9D9V7|DEN6B_MOUSE Protein DENND6B OS=Mus musculus GN=Dennd6b PE=2 SV=3	1,27	0,261212546112666	0,682581316525843	21,0220202458726	21,6895799900045	21,3089099562841	21,569596405202	21,4244040100507	21,7524718215974	1853182121,42622	2360542759,26972	−0,34911394724880	0,0156922810037808	0,0076171351766617	1,27377807716655	0.349113947248799	0,785066109965124	-0.349113947248799	3555
Q62048	sp|Q62048|PEA15_MOUSE Astrocytic phosphoprotein PEA-15 OS=Mus musculus GN=Pea15 PE=1 SV=1;sp|Q15121|PEA15_HUMAN Astrocytic phosphoprotein PEA-15 OS=Homo sapiens GN=PEA15 PE=1 SV=2;sp|Q15121-2|PEA15_HUMAN Isoform 2 of Astrocytic phosphoprotein PEA-15 OS=	1,27	0,108429505939901	0,473232237943471	30,7896398570902	30,3786052252511	30,8636183629237	30,9161912299983	30,9161912299983	30,9261836153758	21036661454449,2	26802653912386,7	−0,34947009355232	0,0085176386351794	0,0001865845915358	1,27409256313901	0.349470093552317	0,784872331046562	-0.349470093552317	3556
Q8R5H6	sp|Q8R5H6|WASF1_MOUSE Wiskott-Aldrich syndrome protein family member 1 OS=Mus musculus GN=Wasf1 PE=1 SV=2	1,27	0,0028345395569158 7	0,0508474448240013	30,6783315069509	30,5864926907295	30,4090370110476	30,8120815400983	30,82620630466	30,7635181652725	18670273755744,8	23797508430782,8	−0,35006745162685	0,0044799074755460 7	0,0010676059616070	1,27462021939878	0.350067451626846	0,784547416383903	-0.350067451626846	3557
O88951	sp|O88951|LIN7B_MOUSE Protein lin-7 homolog B OS=Mus musculus GN=Lin7b PE=1 SV=2	1,27	0,311227040179265	0,719371512809526	26,062710168417	26,5380610554905	26,4033494281023	26,9612195741421	26,3439130720978	26,4269514236666	273538135449,237	348659401830,354	−0,35007640431605	0,0093031932970487 6	0,012605228642404	1,27462812911898	0.35007640431606	0,784542547865471	-0.350076404316059	3558
Q8VI63	sp|Q8VI63|MOB2_MOUSE MOB kinase activator 2 OS=Mus musculus GN=Mob2 PE=2 SV=1;sp|Q8VI63-2|MOB2_MOUSE Isoform 2 of MOB kinase activator 2 OS=Mus musculus GN=Mob2	1,28	0,326720951139153	0,726927682919497	25,4794674273275	25,9701667726243	26,2637346192989	26,1582354818588	25,9776537154619	26,3063436289981	177894456983,04	226817358418,103	−0,35050949615992	0,0152962596553784	0,0062955410144792	1,27501082532171	0.350509496159933	0,78430706637152	-0.350509496159933	3559
Q9D1J1	sp|Q9D1J1|NECP2_MOUSE Adaptin ear-binding coat-associated protein 2 OS=Mus musculus GN=Necap2 PE=1 SV=1	1,28	0,101320277006988	0,463601549732581	25,2509320978723	25,3172002676571	24,9575896134171	25,5677487880404	25,2310892455359	25,4561566173449	85796209114,4378	109405997841,391	−0,35070602243792	0,0076019701538840 4	0,0067466073685219	1,27518452121191	0.350706022437922	0,784200234056811	-0.350706022437922	3560
Q8CI51	sp|Q8CI51|PDLI5_MOUSE PDZ and LIM domain protein 5 OS=Mus musculus GN=Pdlim5 PE=1 SV=4	1,28	0,340820743949962	0,7296975876634 2	26,1745346539103	26,8191926913355	26,5284979350479	27,0989232001278	26,587342146856	26,5652571906599	325103173806,936	414569932474,01	−0,35071784550427	0,0121794648699216	0,0112872186134475	1,27519497155141	0.350717845504274	0,784193807464118	-0.350717845504273	3561
P26049	sp|P26049|GBRA3_MOUSE Gamma-aminobutyric acid receptor subunit alpha-3 OS=Mus musculus GN=Gabra3 PE=1 SV=1	1,28	0,462960193548995	0,793595716606975	24,9321216980073	25,9627882430883	25,4540678038503	25,698996365124	25,4374912672445	25,9419377124712	112887687821,476	143961026168,194	−0,35079014498498	0,0202496783995588	0,0098190957171917	1,27525887850461	0.350790144984989	0,784154509218251	-0.350790144984989	3562
Q3TPX4	sp|Q3TPX4|EXOC5_MOUSE Exocyst complex component 5 OS=Mus musculus GN=Exoc5 PE=1 SV=2	1,28	0,166335835425268	0,575385119523278	28,6513548480099	28,3974533755861	28,0588742128856	28,6907897587117	28,5719407915324	28,5757099628488	2092179536231,48	2669236265034,44	−0,35142035107226	0,0104778010776164	0,0023610299344819	1,27581606588237	0.351420351072259	0,783812045279733	-0.351420351072259	3563
Q8K406	sp|Q8K406|LGI3_MOUSE Leucine-rich repeat LGI family member 3 OS=Mus musculus GN=Lgi3 PE=1 SV=1	1,28	0,210915699122904	0,641644645910089	23,6385289981359	24,1280009506016	24,2039362195013	24,389762517735	24,1153516205821	24,197055235212	26229626450,4168	33474723140,1117	−0,35187486189161	0,0127917131950695	0,0058140356609568	1,27621806598697	0.351874861891615	0,783565149758824	-0.351874861891616	3564

Q80VP1	sp|Q80VP1|EPN1_MOUSE Epsin-1 OS=Mus musculus GN=Epn1 PE=1 SV=3;sp|Q80VP1-2|EPN1_MOUSE Isoform 2 of Epsin-1 OS=Mus musculus GN=Epn1	1,28	1,28	0,0609109041563363	0,365707134875146	30,4762511723909	30,3043185118729	30,049400664825	30,5694328473646	30,5524835884731	30,4410482207959	14092400311804,2	17992717684468,3	−0,352495750831502	0,00709343490174687	0,0022852091285728	1,27676742686603	0.352495750831506	0,783228001402426	-0.352495750831507	3565
Q99MR3	sp|Q99MR3|S12A9_MOUSE Solute carrier family 12 member 9 OS=Mus musculus GN=Slc12a9 PE=1 SV=2	1,28	1,28	0,0170891640272666	0,178300688102698	23,9032676843376	24,0935577863108	23,9779430843893	24,3850652312053	24,2384213358069	24,0856454874652	26267270103,0837	33552504638,8421	−0,353154192950487	0,0039962429380542	0,0061775047588092	1,27735027306485	0.353154192950485	0,782870619818807	-0.353154192950485	3566
Q9D7M1	sp|Q9D7M1|GID8_MOUSE Glucose-induced degradation protein 8 homolog OS=Mus musculus GN=Gid8 PE=2 SV=1	1,28	1,28	0,327637759635579	0,726927682919497	26,5997264252204	26,9469309955916	27,2250623653521	27,4170667080726	26,8359983838767	27,2537191581988	493064875376,88	629963730086,065	−0,353491285641176	0,0116366148305173	0,0110298876974058	1,27764876701985	0.353491285641178	0,782687719671604	-0.353491285641178	3567
Q61234	sp|Q61234|SNTA1_MOUSE Alpha-1-syntrophin OS=Mus musculus GN=Snta1 PE=1 SV=1	1,28	1,28	0,0773823590491188	0,415866149908827	27,4482593170704	27,7938677088192	27,8990401696981	28,0120626766497	27,9328192440591	27,9313864300688	1086217288077,32	1387821152334,31	−0,35350893038136	0,00850969475994167	0,0016513750878958	1,27766439327333	0.353508930381367	0,782678147144758	-0.353508930381367	3568
Q8BZA9	sp|Q8BZA9|TIGAR_MOUSE Fructose-2,6-bisphosphatase TIGAR OS=Mus musculus GN=Tigar PE=1 SV=1	1,28	1,28	0,0895617041525701	0,441176105282424	26,8060735616088	27,0764572082296	27,183957488667	27,2761384034076	27,1060915962256	27,4198860408206	543971532698,699	695135073150,532	−0,353762184319031	0,0072053765342951 8	0,0057607602601299	1,27788869704614	0.353762184319031	0,782540766117985	-0.353762184319031	3569
Q9QWI6-2	sp|Q9QWI6-2|SRCN1_MOUSE Isoform 2 of SRC kinase signaling inhibitor 1 OS=Mus musculus GN=Srcin1;sp|Q9QWI6|SRCN1_MOUSE SRC kinase signaling inhibitor 1 OS=Mus musculus GN=Srcin1 PE=1 SV=2;sp|Q9QWI6-3|SRCN1_MOUSE Isoform 3 of SRC kinase signaling inhibito	1,28	1,28	6,71×10^−05	0,0026944568092495	24,8641690818116	24,8284747330514	24,7005673453217	25,0441535538573	25,1063810759423	24,9798946960453	58819369648,4151	75204465060,8213	−0,354526997217117	0,0034689927435262	0,0025254444443221	1,27856632110045	0.354526997217119	0,782126029363352	-0.354526997217119	3570
Q61644	sp|Q61644|PACN1_MOUSE Protein kinase C and casein kinase substrate in neurons protein 1 OS=Mus musculus GN=Pacsin1 PE=1 SV=1	1,28	1,28	0,000196141378126937	0,00626961771350823	33,2547258597017	33,450829164933	33,2755034398491	33,577674493259	33,6093552004065	33,5323458377698	297673834573555	380735177478297	−0,355055457031495	0,0032323453211355 9	0,0011528780504780	1,27903474628106	0.355055457031493	0,781839588727058	-0.355055457031493	3571
Q8VHW2	sp|Q8VHW2|CCG8_MOUSE Voltage-dependent calcium channel gamma-8 subunit OS=Mus musculus GN=Cacng8 PE=1 SV=1	1,28	1,28	0,34199609136653	0,729721949431128	25,7421229489083	26,4583956916079	26,517535165276	26,6200366760221	26,3678330475435	26,4706027605894	248659733965,331	318267319742,693	−0,356066118485136	0,0164495844651825	0,0047881343445343	1,27993107153837	0.356066118485139	0,781292072859893	-0.356066118485139	3572
Q8JZK9	sp|Q8JZK9|HMCS1_MOUSE Hydroxymethylglutaryl-CoA synthase, cytoplasmic OS=Mus musculus GN=Hmgcs1 PE=1 SV=1	1,28	1,28	0,163461245502498	0,569854526371611	28,8395124734223	29,3037700876513	29,2565888136709	29,5685996112172	29,3328195161808	29,2389697441818	4491871105901,31	5749474779441,24	−0,356113640125215	0,00877039827258998	0,0057804792962406	1,27997323251055	0.356113640125216	0,78126633792067	-0.356113640125216	3573
Q9DC23	sp|Q9DC23|DJC10_MOUSE DnaJ homolog subfamily C member 10 OS=Mus musculus GN=Dnajc10 PE=1 SV=2	1,28	1,28	0,185502934316305	0,609238393171755	26,6381041414573	26,3529925413592	25,9923002404933	26,5785183382483	26,5603842110093	26,587342146856	271655070256,75	347981411026,808	−0,357234265986474	0,0122926430075682	0,0005171814189985	1,28096784903709	0.357234265986473	0,780659718158974	-0.357234265986473	3574
Q9JKB1	sp|Q9JKB1|UCHL3_MOUSE Ubiquitin carboxyl-terminal hydrolase isozyme L3 OS=Mus musculus GN=Uchl3 PE=1 SV=2;sp|P58321|UCHL4_MOUSE Ubiquitin carboxyl-terminal hydrolase isozyme L4 OS=Mus musculus GN=Uchl4 PE=2 SV=1	1,28	1,28	0,0821094864535231	0,426912896921582	29,0627516643017	29,3120180582318	29,4780244796227	29,6792282244892	29,4911659736167	29,4260099986843	5223896702425,88	6693344110809,74	−0,357600817204609	0,0071377198208183 3	0,0044523191586096	1,2812933509388 6	0.357600817204611	0,780461398060992	-0.357600817204611	3575
O35678	sp|O35678|MGLL_MOUSE Monoglyceride lipase OS=Mus musculus GN=Mgll PE=1 SV=1	1,28	1,28	0,15945032087735 8	0,565425786298694	29,7550162373898	30,1058851287532	30,3275359257424	30,4253950011429	30,2534697710926	30,2534697710926	11379247647570,4	14581550499229,7	−0,357738958529085	0,0096025612673666 2	0,0032747782738185	1,28141604355917	0.357738958529093	0,780386670688521	-0.357738958529093	3576
P53810	sp|P53810|PIPNA_MOUSE Phosphatidylinositol transfer protein alpha isoform OS=Mus musculus GN=Pitpna PE=1 SV=2	1,28	1,28	0,0831916414628778	0,429460790968485	31,4753765257957	31,7691737448374	31,7786943855435	32,1255304366215	31,7920975405357	31,849738476019	57019525936500,2	73071204397922,2	−0,357846942116273	0,0054440783056857 5	0,0055827129824056	1,28151195924179	0.357846942116272	0,780328262087898	-0.357846942116272	3577
Q5SSM3	sp|Q5SSM3|RHG44_MOUSE Rho GTPase-activating protein 44 OS=Mus musculus GN=Arhgap44 PE=1 SV=1;sp|Q5SSM3-2|RHG44_MOUSE Isoform 2 of Rho GTPase-activating protein 44 OS=Mus musculus GN=Arhgap44;sp|Q5SSM3-3|RHG44_MOUSE Isoform 3 of Rho GTPase-activating pro	1,28	1,28	0,390439015078674	0,753319093070654	29,4637941408889	28,991263159107	28,6266029003322	29,0477196217778	29,5767627429087	29,2013315647317	4039815210079,09	5177126610041,54	−0,357862298205747	0,0144606430964391	0,0092966401379449	1,28152559976628	0.357862298205746	0,780319956294574	-0.357862298205746	3578
Q8VD62	sp|Q8VD62|CK068_MOUSE UPF0696 protein C11orf68 homolog OS=Mus musculus GN=Bles03 PE=2 SV=1	1,28	1,28	0,225174567909231	0,652238880554131	27,9203165194517	28,3498300207127	28,5978831612972	28,6424177181693	28,4788737237828	28,4918017637118	1931548702938,04	2476079888117,49	−0,35829980755331	0,0121179614254739	0,0031859696192165	1,28191429206583	0.358299807553307	0,780083353613665	-0.358299807553306	3579
Q8K0S0	sp|Q8K0S0|PHYIP_MOUSE Phytanoyl-CoA hydroxylase-interacting protein OS=Mus musculus GN=Phyhip PE=1 SV=1	1,28	1,28	0,00157008695368741	0,0327329861064157	30,4582833926756	30,5331676346361	30,6939719257556	30,82620630466	30,8665321302372	30,7404493890883	18742366229609,1	24047751322264	−0,359598890341571	0,0039404133885765 2	0,0020898693249195	1,28306911879	0.359598890341575	0,779381239370059	-0.359598890341575	3580
Q8R1V4	sp|Q8R1V4|TMED4_MOUSE Transmembrane emp24 domain-containing protein 4 OS=Mus musculus GN=Tmed4 PE=2 SV=1	1,28	1,28	0,26855656263573	0,687806963093714	25,839608189605	26,1649342395901	26,5713233590086	26,5575313626245	26,2906517440577	26,4776551762052	237149187398,517	304502792338,528	−0,36066053296106	0,0139968627088881	0,0051802695772908	1,28401364423327	0.360660532961062	0,778807923491449	-0.360660532961062	3581

Q9QZ23	sp|Q9QZ23|NFU1_MOUSE NFU1 iron-sulfur cluster scaffold homolog, mitochondrial OS=Mus musculus GN=Nfu1 PE=1 SV=2;sp|Q9UMS0-3|NFU1_HUMAN Isoform 3 of NFU1 iron-sulfur cluster scaffold homolog, mitochondrial OS=Homo sapiens GN=NFU1;sp|Q9UMS0|NFU1_HUMAN NFU	1,28	0,2708208230551	0,691573877451378	27,067317939297	27,5612721540117	27,7989733088988	27,6908756572043	27,6226377001941	27,8646384205141	856272469048,026	1099691269927,84	−0,360956709126102	0,0135839951117449	0,0045003155324975	1,28427727117098	0.360956709126109	0,778648055562188	-0.360956709126109	3582
Q99MN9	sp|Q99MN9|PCCB_MOUSE Propionyl-CoA carboxylase beta chain, mitochondrial OS=Mus musculus GN=Pccb PE=1 SV=2	1,28	0,127624067214638	0,511284767067292	29,5442070726591	30,0268702489686	29,9652839885513	30,2207870528903	30,0171565154699	30,049400664825	9156212898160,6	11760662725986,7	−0,36114644627116	0,00880195359729832	0,0036367870829028	1,2844461849887	0.361146446271165	0,778545657799434	-0.361146446271165	3583
Q6PHZ8	sp|Q6PHZ8|KCIP4_MOUSE Kv channel-interacting protein 4 OS=Mus musculus GN=Kcnip4 PE=1 SV=1;sp|Q6PIL6-3|KCIP4_HUMAN Isoform 3 of Kv channel-interacting protein 4 OS=Homo sapiens GN=KCNIP4;sp|Q6PHZ8-3|KCIP4_MOUSE Isoform 3 of Kv channel-interacting protei	1,29	0,235897879821738	0,658933495707757	24,6197060572759	24,8266336914146	25,1454481767251	25,4043835260839	24,9200121770116	25,0225487947601	62844503158,7369	80832760008,4648	−0,363153547384663	0,0106518928850595	0,0101631394901652	1,28623437127495	0.363153547384664	0,777463285333271	-0.363153547384664	3584
Q91VR8	sp|Q91VR8|BRK1_MOUSE Protein BRICK1 OS=Mus musculus GN=Brk1 PE=2 SV=1	1,29	0,321397431390365	0,724291969738039	28,6029701816381	28,2591635841223	28,1859618455125	28,1842803916153	28,6927585562291	28,9271249389734	2051034064806,27	2638912357158,96	−0,363591983900712	0,00785404602701004	0,0132775984488756	1,28662531863322	0.363591983900713	0,77722704933422	-0.363591983900713	3585
Q9CWU4	sp|Q9CWU4|CA052_MOUSE UPF0690 protein C1orf52 homolog OS=Mus musculus PE=1 SV=1	1,29	0,00872941765711356	0,110950504365343	22,7884248163617	23,0370888286754	23,0327998585375	23,2165788422459	23,1133185411363	23,2867314959679	9295266860,84904	11968487126,7199	−0,364672610687039	0,0062016158807939	0,0037590856763372	1,28758940500463	0.364672610687034	0,776645098284574	-0.364672610687035	3586
Q9R1V7-2	sp|Q9R1V7-2|ADA23_MOUSE Isoform Beta of Disintegrin and metalloproteinase domain-containing protein 23 OS=Mus musculus GN=Adam23;sp|Q9R1V7|ADA23_MOUSE Disintegrin and metalloproteinase domain-containing protein 23 OS=Mus musculus GN=Adam23 PE=1 SV=1;sp|	1,29	0,00517191967071511	0,078512854131692	26,8037426952769	27,0871885833272	27,024059925362	27,1783471400919	27,219419790962	27,2761384034076	517183579580,243	666053018374,444	−0,364960550842088	0,00551727148186411	0,0018036529283034	1,28784641406254	0.364960550842088	0,776490107112601	-0.364960550842088	3587
Q9CPQ3	sp|Q9CPQ3|TOM22_MOUSE Mitochondrial import receptor subunit TOM22 homolog OS=Mus musculus GN=Tomm22 PE=2 SV=3	1,29	0,262657728411722	0,682806179310143	25,3545598909119	25,1824661258207	25,1917952891573	25,0665939202135	25,6369206672453	25,784338153794	91805719384,601	118236289640,272	−0,365016962559019	0,00383385460115203	0,0148678243773926	1,28789677193145	0.365016962559023	0,776459745683117	-0.365016962559023	3588
Q9WUK2	sp|Q9WUK2|IF4H_MOUSE Eukaryotic translation initiation factor 4H OS=Mus musculus GN=Eif4h PE=1 SV=3;sp|Q9WUK2-2|IF4H_MOUSE Isoform Short of Eukaryotic translation initiation factor 4H OS=Mus musculus GN=Eif4h	1,29	0,100102341913242	0,46324407107743	30,8856809268552	31,1972620093183	31,3626131085233	31,486087748298	31,2748215534313	31,4444699733367	33699990842994,8	43413566123804,2	−0,365397735468603	0,00777480032483442	0,0035638789940093	1,28823673353693	0.365397735468605	0,776254840408438	-0.365397735468605	3589
Q9CQQ7	sp|Q9CQQ7|AT5F1_MOUSE ATP synthase F(0) complex subunit B1, mitochondrial OS=Mus musculus GN=Atp5f1 PE=1 SV=1	1,29	0,00186897442475997	0,03688380397385	30,7747464720785	30,9123179140515	30,9542765997972	31,1488485026459	31,0218212612287	31,2308454760787	25775537400884,3	33208879579939,6	−0,365566542165015	0,00304116829877791	0,0033827314373715	1,28838747621224	0.365566542165013	0,776164017784402	-0.365566542165013	3590
Q9D1E6	sp|Q9D1E6|TBCB_MOUSE Tubulin-folding cofactor B OS=Mus musculus GN=Tbcb PE=1 SV=2	1,29	0,302401787995715	0,715641870477102	28,808297486709	29,3290713947903	29,5685996112172	29,6534011397899	29,3062942508444	29,5072210768158	4974384851257,06	6410588265997,11	−0,365938623174183	0,0132963846897014	0,0059097186212669	1,28871980308824	0.365938623174182	0,775963865538218	-0.365938623174182	3591
Q3UHJ0	sp|Q3UHJ0|AAK1_MOUSE AP2-associated protein kinase 1 OS=Mus musculus GN=Aak1 PE=1 SV=2;sp|Q3UHJ0-2|AAK1_MOUSE Isoform 2 of AP2-associated protein kinase 1 OS=Mus musculus GN=Aak1	1,29	0,139821485725843	0,531977974609894	27,8286564820045	27,9865800133104	27,6281717314036	28,3325463587444	28,0003802943019	27,8721796264246	1201352845427,27	1548594342857,81	−0,366299334453532	0,00645796350060555	0,0084648533242146	1,28904205683805	0.366299334453536	0,775769878643795	-0.366299334453536	3592
Q9DCT8	sp|Q9DCT8|CRIP2_MOUSE Cysteine-rich protein 2 OS=Mus musculus GN=Crip2 PE=1 SV=1	1,29	0,179651015042916	0,598356502628386	30,5719607650948	30,7820965217206	31,1488485026459	31,2206366567217	30,9261836153758	31,1184365708611	24613149038378	31734291015202,4	−0,366613361432295	0,00946885754742289	0,0048089928827252	1,2893226691847	0.366613361432296	0,775601037583824	-0.366613361432296	3593
Q9D1J3	sp|Q9D1J3|SARNP_MOUSE SAP domain-containing ribonucleoprotein OS=Mus musculus GN=Sarnp PE=1 SV=3	1,29	0,142505638434277	0,535014965139111	27,3269456084881	27,6440499937839	27,9203165194517	27,8646384205141	27,8519734871773	27,9380967665236	999416428323,595	1289019401554,54	−0,367116140170314	0,0107461049603804	0,0016675849761730	1,28977207600712	0.367116140170308	0,77533078797597	-0.367116140170308	3594
Q8BHN0	sp|Q8BHN0|PPM1L_MOUSE Protein phosphatase 1L OS=Mus musculus GN=Ppm1l PE=1 SV=1;sp|Q8BHN0-2|PPM1L_MOUSE Isoform 2 of Protein phosphatase 1L OS=Mus musculus GN=Ppm1l	1,29	0,801804623207677	0,945787729921946	24,2234160190066	22,258729843119	24,0670079530992	24,6083461525581	24,5212483266218	22,1831864358735	16331875790,2422	21066016185,5134	−0,367227010003716	0,0464342197348688	0,0578737671766165	1,28987119765506	0.367227010003719	0,775271206782479	-0.367227010003719	3595
O35943	sp|O35943|FRDA_MOUSE Frataxin, mitochondrial OS=Mus musculus GN=Fxn PE=1 SV=1;sp|Q16595|FRDA_HUMAN Frataxin, mitochondrial OS=Homo sapiens GN=FXN PE=1 SV=2	1,29	0,381182834725183	0,749797813889279	24,5156871626391	24,2934651158925	24,4368206009609	24,1071874390443	24,8936242018883	25,0087957507153	40127362419,5918	51759256908,2494	−0,367230574579444	0,00461430645678754	0,0198901505711778	1,2898743846413	0.367230574579447	0,775269291263652	-0.367230574579446	3596
Q3TWL2	sp|Q3TWL2|TM55B_MOUSE Type 1 phosphatidylinositol 4,5-bisphosphate 4-phosphatase OS=Mus musculus GN=Tmem55b PE=1 SV=1	1,29	0,355741638604179	0,732843629012038	23,2912926988169	24,0856454874652	23,7519013740161	24,2663462043832	23,8407893507363	23,7858998078014	19813179544,3872	25561294762,2708	−0,367500498237391	0,0168227654673318	0,0109736621433469	1,29011573861763	0.367500498237391	0,775124254411088	-0.367500498237391	3597
O08989	sp|O08989|RASM_MOUSE Ras-related protein M-Ras OS=Mus musculus GN=Mras PE=1 SV=1	1,29	0,21576472680253	0,646799042967852	27,4170667080726	27,8646384205141	28,1207709623458	28,0948693999158	28,01920864903	28,0563316653839	1185072847896,4	1530787214217,78	−0,369298010069003	0,01281133512308	0,0013484280245781	1,29172414753663	0.369298010069	0,7741590972864	-0.369298010069	3598

Q8BR90	sp|Q8BR90|CE051_MOUSE UPF0600 protein C5orf51 homolog OS=Mus musculus PE=2 SV=1;sp|Q8BR90-2|CE051_MOUSE Isoform 2 of UPF0600 protein C5orf51 homolog OS=Mus musculus	1,29	0,163036578658	0,569625464688209	26,8570170371907	27,0652717104084	27,4684064394126	27,4089401757686	27,311293764426	27,4384765453677	606052061264,993	782873395678,43	−0,369337287515248	0,011456890451432	0,0024304762694163	1,2917593152713	0.369337287515248	0,774138021052299	-0.369337287515248	3599
P46097	sp|P46097|SYT2_MOUSE Synaptotagmin-2 OS=Mus musculus GN=Syt2 PE=1 SV=1	1,29	0,217066282413788	0,64735591278721	26,6200366760221	27,2314097361691	27,1479973377852	27,4078116367401	27,2232737570987	27,1365607688038	531949599131,51	687193709810,775	−0,369427270384176	0,0122790128884909	0,0050827279848232	1,2918398865846	0.369427270384176	0,774089738507629	-0.369427270384176	3600
Q9D6J5	sp|Q9D6J5|NDUB8_MOUSE NADH dehydrogenase [ubiquinone] 1 beta subcomplex subunit 8, mitochondrial OS=Mus musculus GN=Ndufb8 PE=1 SV=1	1,29	0,197458263423491	0,626240510020104	24,4408004316673	24,0084076421851	23,9597534682194	24,1399594220105	24,5413781637526	24,4958624117327	30357836154,4569	39217934786,0704	−0,369444603286757	0,0109713136119485	0,0090110560872927	1,2918554071685	0.369444603286757	0,774080438453876	-0.369444603286757	3601
Q7TQI3	sp|Q7TQI3|OTUB1_MOUSE Ubiquitin thioesterase OTUB1 OS=Mus musculus GN=Otub1 PE=1 SV=2	1,29	0,210780777045243	0,641644645910089	31,6337004171238	32,1137938104902	32,2466587951576	32,420921979523	32,1406176218661	32,20118575714	78809211848491	101821437889144	−0,369605165787299	0,0100773113721214	0,0045727886241426	1,29199919020753	0.369605165787296	0,773994293169312	-0.369605165787296	3602
P70182	sp|P70182|PI51A_MOUSE Phosphatidylinositol 4-phosphate 5-kinase type-1 alpha OS=Mus musculus GN=Pip5k1a PE=1 SV=2;sp|P70182-3|PI51A_MOUSE Isoform 3 of Phosphatidylinositol 4-phosphate 5-kinase type-1 alpha OS=Mus musculus GN=Pip5k1a;sp|P70182-2|PI51A_MO	1,29	0,41374732392408	0,766530246241387	24,7436143644013	25,4301759017466	25,810667197616	25,6452858661328	25,526381389398	25,5829712431895	99971650517,9055	129232571714,208	−0,370378786572758	0,0213514568440067	0,0023246231289845	1,29269218868265	0.370378786572756	0,77357936309577	-0.370378786572756	3603
Q80TN5	sp|Q80TN5|ZDH17_MOUSE Palmitoyltransferase ZDHHC17 OS=Mus musculus GN=Zdhhc17 PE=2 SV=2;sp|Q80TN5-2|ZDH17_MOUSE Isoform 2 of Palmitoyltransferase ZDHHC17 OS=Mus musculus GN=Zdhhc17	1,29	0,768553295305104	0,935997694496879	22,8005593253074	25,2526747161242	24,9101751598063	24,8141487793	25,1516291000811	23,7679115608697	36520383744,6608	47211175948,6591	−0,370426493639546	0,0546005206844449	0,0293547332113628	1,29273493615908	0.370426493639545	0,773553782781766	-0.370426493639545	3604
Q64373-2	sp|Q64373-2|B2CL1_MOUSE Isoform Bcl-X(S) of Bcl-2-like protein 1 OS=Mus musculus GN=Bcl2l1;sp|Q64373-3|B2CL1_MOUSE Isoform Bcl-X(beta) of Bcl-2-like protein 1 OS=Mus musculus GN=Bcl2l1;sp|Q64373-4|B2CL1_MOUSE Isoform Bcl-X(delta-TM) of Bcl-2-like protei	1,29	0,485170598183958	0,804012174208465	25,5382714274755	26,1521497135295	25,4336098486706	26,1835363389388	26,3276898796021	25,3853556236696	146166223753,301	189097252042,592	−0,371518427928828	0,0150995559527505	0,0195484905265061	1,29371374033545	0.371518427928831	0,77296852373285	-0.371518427928831	3605
P48347-2	sp|P48347-2|14310_ARATH Isoform 2 of 14-3-3-like protein GF14 epsilon OS=Arabidopsis thaliana GN=GRF10;sp|P48347|14310_ARATH 14-3-3-like protein GF14 epsilon OS=Arabidopsis thaliana GN=GRF10 PE=1 SV=1;sp|Q9S9Z8-2|14311_ARATH Isoform 2 of 14-3-3-like pro	1,29	0,207208932410672	0,637641589296298	30,9306955015522	31,5115733756014	31,3364515285498	31,7123495077502	31,3310729947621	31,5082584125245	37658261255730	48725663169147,4	−0,371715431206121	0,0095321913445938	0,0060537182712740	1,29389041194071	0.371715431206122	0,77286298033548	-0.371715431206122	3606
Q9CPU0	sp|Q9CPU0|LGUL_MOUSE Lactoylglutathione lyase OS=Mus musculus GN=Glo1 PE=1 SV=3	1,29	0,191950992602683	0,618802084535046	30,8190110775727	31,2551943052669	31,4594116268809	31,4180321721597	31,3439939544913	31,5462290964587	34703870434954,2	44928123553877	−0,372522236313257	0,0104922887704031	0,0032548288776164	1,29461420270359	0.372522236313254	0,772430889381301	-0.372522236313254	3607
Q9UIV1-2	sp|Q9UIV1-2|CNOT7_HUMAN Isoform 2 of CCR4-NOT transcription complex subunit 7 OS=Homo sapiens GN=CNOT7;sp|Q9UIV1|CNOT7_HUMAN CCR4-NOT transcription complex subunit 7 OS=Homo sapiens GN=CNOT7 PE=1 SV=3;sp|Q60809|CNOT7_MOUSE CCR4-NOT transcription complex	1,30	0,109871893236835	0,475080634873879	25,9086542006991	26,1456344965372	26,4451428857265	26,4873971485829	26,336120588715	26,4519728460025	231183405192,828	299435097376,159	−0,37320549040814	0,0102746335311257	0,0029941707334123	1,29522747156701	0.373205490408141	0,772065156084254	-0.37320549040814	3608
O08842	sp|O08842|GFRA2_MOUSE GDNF family receptor alpha-2 OS=Mus musculus GN=Gfra2 PE=2 SV=2;sp|O08842-2|GFRA2_MOUSE Isoform 2 of GDNF family receptor alpha-2 OS=Mus musculus GN=Gfra2	1,30	0,316492632721134	0,722907157640623	24,3639618883844	24,9622562481828	24,939766410663	25,3260413000039	24,9705054088962	24,7462638463834	56377060410,9027	73039789050,6595	−0,373574343150676	0,0136988545479299	0,0116875659091507	1,29555866372441	0.373574343150675	0,771867788005251	-0.373574343150675	3609
Q8K297	sp|Q8K297|GT251_MOUSE Procollagen galactosyltransferase 1 OS=Mus musculus GN=Colgalt1 PE=1 SV=2	1,30	0,139594604260972	0,531560326124625	24,6110501334689	24,3866384851643	24,0418006571554	24,6838243385297	24,6402971622954	24,4925993676552	37458385182,9241	48536096841,0815	−0,373769388132804	0,0117774612212642	0,0040734648076296	1,29573382846219	0.373769388132802	0,771763442486354	-0.373769388132803	3610
Q9WV55	sp|Q9WV55|VAPA_MOUSE Vesicle-associated membrane protein-associated protein A OS=Mus musculus GN=Vapa PE=1 SV=2	1,30	0,101032406692967	0,46324407107743	30,0419786001201	30,2998683979432	30,4692900893302	30,6358451815377	30,333769820244	30,620839005307	14004208802739,9	18158344729599,7	−0,374772218441701	0,0071084890704362	0,0055760224323191	1,29663481781613	0.374772218441703	0,771227169176484	-0.374772218441703	3611
Q922Q8	sp|Q922Q8|LRC59_MOUSE Leucine-rich repeat-containing protein 59 OS=Mus musculus GN=Lrrc59 PE=1 SV=1	1,30	0,148687162806609	0,547137360146565	28,2250895728033	28,6062432168209	28,8326097047498	28,8233279788742	28,7527330443723	28,8699406290185	2518406555290,82	3268439873518,35	−0,376090956257037	0,0107523279365553	0,0020479131414551	1,29782058685156	0.37609095625704	0,770522528407369	-0.37609095625704	3612
Q8BHZ0	sp|Q8BHZ0|FA49A_MOUSE Protein FAM49A OS=Mus musculus GN=Fam49a PE=2 SV=1	1,30	0,233909856178948	0,657279264635888	29,3261521639233	29,7525593270964	30,0459928945559	30,0859791046874	29,8563790555124	29,9652839885513	7982196939416,48	10362493919222,2	−0,376513476086004	0,0121838795784128	0,0038322815679450	1,2982007331906	0.376513476085997	0,770296899726974	-0.376513476085997	3613

P19536	sp|P19536|COX5B_MOUSE Cytochrome c oxidase subunit 5B, mitochondrial OS=Mus musculus GN=Cox5b PE=1 SV=1	1,30	0,278951784483201	0,699221280539289	27,8257406617401	27,5855513418751	27,122691076725	27,5237275215483	27,9148680147052	27,8785137992401	887192388268,479	1151826176655,85	−0,376604121566576	0,0129893231454131	0,00778098377365648	1,29828230256106	0.376604121566581	0,770248502985325	-0.376604121566581	3614
Q9QYF1	sp|Q9QYF1|RDH11_MOUSE Retinol dehydrogenase 11 OS=Mus musculus GN=Rdh11 PE=2 SV=2	1,30	0,27597453631193	0,69857053622935	26,0524841852733	26,4148721289321	26,8380058322934	26,8403763238565	26,5713233590086	26,6783050470106	302431522079,352	392840000807,651	−0,377333321302728	0,01487233962282	0,00507416142789498	1,29893867579246	0.377333321302727	0,769859284842619	-0.377333321302727	3615
Q8K1N1	sp|Q8K1N1|PLPL8_MOUSE Calcium-independent phospholipase A2-gamma OS=Mus musculus GN=Pnpla8 PE=1 SV=1	1,30	0,392707901750466	0,753881747531964	23,8591535596167	23,8591535596167	23,168322265652	24,2934651158925	23,7251359806086	23,6540441959298	18276400266,7126	23750793185,2632	−0,377993750625213	0,0168798391317026	0,0146689748129476	1,29953343320683	0.377993750625216	0,769506943374534	-0.377993750625217	3616
Q8BFS6	sp|Q8BFS6|CPPED_MOUSE Serine/threonine-protein phosphatase CPPED1 OS=Mus musculus GN=Cpped1 PE=2 SV=1;sp|Q8BFS6-2|CPPED_MOUSE Isoform 2 of Serine/threonine-protein phosphatase CPPED1 OS=Mus musculus GN=Cpped1;sp|Q8BFS6-4|CPPED_MOUSE Isoform 4 of Serine/	1,30	0,087250503584134 3	0,440251927134614	27,1876699138534	27,3724149190948	27,6294369835375	27,8242150678396	27,6095679757196	27,5436012651194	790955284015,194	1028505715239,62	−0,37888177012969	0,0080983599679808 3	0,00530475321365834	1,30033357893322	0.378881770129687	0,76903343588219	-0.378881770129687	3617
O55222	sp|O55222|ILK_MOUSE Integrin-linked protein kinase OS=Mus musculus GN=Ilk PE=1 SV=2	1,30	0,351931158083542	0,732843629012038	25,9650637893661	26,1438469895555	26,014799565769	26,4120453633107	26,7207981765655	25,7790605133414	203969596223,556	265257799339,641	−0,379041051517279	0,00354349700548202	0,0182512856418699	1,3004771507657	0.379041051517276	0,768948535090535	-0.379041051517276	3618
Q99LP6	sp|Q99LP6|GRPE1_MOUSE GrpE protein homolog 1, mitochondrial OS=Mus musculus GN=Grpel1 PE=1 SV=1	1,30	0,241062476996109	0,664601444032504	28,0347736742627	28,4788737237828	28,6894779101035	28,8639896558021	28,468006362801	28,6599523335218	2159810860879,48	2809373958944,82	−0,379343697894343	0,011767865576671	0,0069084053342576	1,30074999150659	0.379343697894345	0,768787243151741	-0.379343697894345	3619
Q9CQC7	sp|Q9CQC7|NDUB4_MOUSE NADH dehydrogenase [ubiquinone] 1 beta subcomplex subunit 4 OS=Mus musculus GN=Ndufb4 PE=1 SV=3	1,30	0,103292836011877	0,465125181208242	26,0701199334524	25,879283072998	25,7001815061086	25,9039797918375	26,2659948811303	26,2691378786882	174152090467,568	226581570241,152	−0,379682728882685	0,0071475038055746 4	0,0080287508337045	1,30105570155845	0.379682728882688	0,768606600626066	-0.379682728882688	3620
Q8R429	sp|Q8R429|AT2A1_MOUSE Sarcoplasmic/endoplasmic reticulum calcium ATPase 1 OS=Mus musculus GN=Atp2a1 PE=2 SV=1	1,30	0,332914692119368	0,72953198881961	23,897846537075	23,4577010632101	23,1698545542957	23,5485803366946	24,1071874390443	23,6595996010165	16203083245,314	21084186165,0732	−0,379892969501983	0,0155961468372991	0,0124399571380204	1,3012453152193	0.379892969501985	0,768494601520596	-0.379892969501985	3621
Q9WVL0	sp|Q9WVL0|MAAI_MOUSE Maleylacetoacetate isomerase OS=Mus musculus GN=Gstz1 PE=1 SV=1	1,30	0,201865034748179	0,632779967349437	28,485463756269	28,9124490208349	29,0169853290817	29,1969573151545	28,8140965858009	29,1940485808107	3234728243962,09	4209510533015,2	−0,380007977979602	0,0097757641150822 8	0,00757558430583555	1,3013490517704 6	0.3800079779796	0,768433341261914	-0.3800079779796	3622
Q8VHK9	sp|Q8VHK9|DHX36_MOUSE ATP-dependent RNA helicase DHX36 OS=Mus musculus GN=Dhx36 PE=1 SV=2;sp|Q9H2U1-3|DHX36_HUMAN Isoform 3 of ATP-dependent RNA helicase DHX36 OS=Homo sapiens GN=DHX36;sp|Q9H2U1-2|DHX36_HUMAN Isoform 2 of ATP-dependent RNA helicase DHX3	1,30	0,505182067268648	0,814993646406912	23,7132586719014	24,8919913695741	24,7970047172291	24,8802711146083	24,8090721097822	24,5032761075892	42273161972,4407	55015076932,5678	−0,3800850167860 6	0,0267639956605121	0,00809893920122011	1,30141854466515	0.380085016786061	0,768392308607604	-0.380085016786061	3623
Q9JIA1	sp|Q9JIA1|LGI1_MOUSE Leucine-rich glioma-inactivated protein 1 OS=Mus musculus GN=Lgi1 PE=1 SV=1	1,30	0,108268340673625	0,472983636494304	29,104176927775	29,6681665776184	29,3206469921288	29,6188712147222	29,6259603347948	29,6401923534164	5659350029888,38	7369281304154,49	−0,380887555401708	0,0096893435085660 8	0,00036647860206953	1,30214269575756	0.380887555401708	0,767964988213691	-0.380887555401708	3624
Q8R313	sp|Q8R313|EXOC6_MOUSE Exocyst complex component 6 OS=Mus musculus GN=Exoc6 PE=1 SV=2	1,30	0,00047243893810178 7	0,0131420572635874	26,6267436086175	26,5887417463319	26,4120453633107	26,8149454374718	26,8660517354712	26,7397710425091	336717534548,994	438630293198,023	−0,381466601148738	0,0043165645499902 3	0,00236959171210613	1,30266543376048	0.381466601148737	0,767656816618866	-0.381466601148737	3625
A2AHG0	sp|A2AHG0|LZTS3_MOUSE Leucine zipper putative tumor suppressor 3 OS=Mus musculus GN=Lzts3 PE=2 SV=1;sp|A2AHG0-2|LZTS3_MOUSE Isoform 2 of Leucine zipper putative tumor suppressor 3 OS=Mus musculus GN=Lzts3;sp|A2AHG0-3|LZTS3_MOUSE Isoform 3 of Leucine zip	1,30	0,333533195164726	0,72953198881961	26,2191523428398	26,0872422456991	25,5829712431895	26,377343207	26,4605063694145	25,8448215763115	188642956044,779	245744213868,389	−0,381499217504721	0,012932258244806 2	0,0127368581064053	1,30269488466909	0.381499217504721	0,767639461679488	-0.381499217504721	3626
Q63959-2	sp|Q63959-2|KCNC3_MOUSE Isoform KV3.3A of Potassium voltage-gated channel subfamily C member 3 OS=Mus musculus GN=Kcnc3;sp|Q63959|KCNC3_MOUSE Potassium voltage-gated channel subfamily C member 3 OS=Mus musculus GN=Kcnc3 PE=1 SV=2	1,30	0,495197673728345	0,808788130730939	23,4691216538316	23,2491539067247	23,075311364659	23,3727250633184	24,2355146437319	22,9793957310975	12695697266,2354	16542717516,5368	−0,381856617277698	0,0084830788634352 3	0,0273070941708281	1,30301764208987	0.381856617277694	0,767449317413795	-0.381856617277694	3627
Q9DAW6	sp|Q9DAW6|PRP4_MOUSE U4/U6 small nuclear ribonucleoprotein Prp4 OS=Mus musculus GN=Prpf4 PE=1 SV=1	1,30	0,54136991145563	0,832411256130455	24,3741949927678	25,2169254586012	25,1555905514942	25,3812269421496	25,6392323739276	24,520588767233	66175115755,2752	86235637832,0874	−0,381995388918348	0,0188575541826538	0,0232622437589724	1,30314298430544	0.381995388918348	0,767375500650059	-0.381995388918348	3628

Q5SUC9	sp|Q5SUC9|SCO1_MOUSE Protein SCO1 homolog, mitochondrial OS=Mus musculus GN=Sco1 PE=2 SV=1	1,30	0,160986428183084	0,567767985643373	23,8733808899669	23,9779430843893	23,8790570595803	23,9032676843376	24,5308608808026	24,0912088731561	24212309826,8781	31558616014,1397	−0,38229322076422	0,00245916618249929	0,0133236038576592	1,30341203461334	0.382293220764223	0,76721709900174	-0.382293220764223	3629
Q91VK4	sp|Q91VK4|ITM2C_MOUSE Integral membrane protein 2C OS=Mus musculus GN=Itm2c PE=2 SV=2	1,30	0,235817651997617	0,658933495707757	24,9418514401016	25,3552094720957	25,6889101757867	25,4960085354994	25,6974368156947	25,5883688052889	100022103081,722	130408550050,445	−0,382719616083102	0,0147752210500151	0,0039395876058367	1,30379732111707	0.3827196160831	0,76699037787807	-0.3827196160831	3630
Q9CYW4	sp|Q9CYW4|HDHD3_MOUSE Haloacid dehalogenase-like hydrolase domain-containing protein 3 OS=Mus musculus GN=Hdhd3 PE=1 SV=1	1,30	0,338813519500191	0,729620681454069	26,9524248585844	27,5887500513035	27,7640796168976	27,9533352167808	27,5493288208279	27,5987969361743	822064620172,974	1071935481138,71	−0,382894364202364	0,0155672817909418	0,0079552924408502	1,3039552546528	0.382894364202366	0,766897480900343	-0.382894364202366	3631
Q8K070	sp|Q8K070|SAM14_MOUSE Sterile alpha motif domain-containing protein 14 OS=Mus musculus GN=Samd14 PE=1 SV=1	1,30	0,592883089548211	0,860080030359509	21,7524718215974	23,114783803093	22,6901014055072	23,3634269361434	22,4379044725108	22,5528552230193	6024612567,25713	7857457181,36585	−0,383194038160979	0,0309543670162532	0,0221399246014623	1,30422613797109	0.38319403816098	0,766738198910539	-0.38319403816098	3632
Q9JK91	sp|Q9JK91|MLH1_MOUSE DNA mismatch repair protein Mlh1 OS=Mus musculus GN=Mlh1 PE=2 SV=2	1,30	0,427728148377095	0,773941056137542	22,8379813625276	22,5849545096597	21,9979846941957	22,5552010672857	23,1878192907474	22,4762920876387	5756757325,42489	7512024311,66254	−0,383945334978663	0,0191744489618809	0,0171515402472381	1,30490550270123	0.383945334978663	0,766339016832977	-0.383945334978663	3633
Q9CR25	sp|Q9CR25|DPH2_MOUSE Diphthamide biosynthesis protein 2 OS=Mus musculus GN=Dph2 PE=1 SV=1	1,31	0,432329946781575	0,775937377794215	23,4856512423187	24,1940816395417	24,4180530212713	24,1331519049364	24,0856454874652	24,6781682335195	27366768917,1323	35720426821,5085	−0,384324207615743	0,0202527204104844	0,0135494205653608	1,30524823480884	0.384324207615741	0,7661377915186	-0.384324207615741	3634
Q9Z2Y8	sp|Q9Z2Y8|PROSC_MOUSE Proline synthase co-transcribed bacterial homolog protein OS=Mus musculus GN=Prosc PE=1 SV=1	1,31	0,292937720423198	0,711969538636193	28,0782614029079	28,5598383543679	28,9252452807151	28,8541446687965	28,6599523335218	28,8489065316386	2435358312748,81	3179254478946,59	−0,384554448844941	0,0148948475872985	0,0038431693206128	1,30545655737949	0.384554448844941	0,766015532533193	-0.384554448844941	3635
Q9JKK7	sp|Q9JKK7|TMOD2_MOUSE Tropomodulin-2 OS=Mus musculus GN=Tmod2 PE=1 SV=2;sp|Q9NZR1|TMOD2_HUMAN Tropomodulin-2 OS=Homo sapiens GN=TMOD2 PE=1 SV=1;sp|Q9NZR1-2|TMOD2_HUMAN Isoform 2 of Tropomodulin-2 OS=Homo sapiens GN=TMOD2;sp|Q9JKK7-2|TMOD2_MOUSE Isoform	1,31	0,00126670551754988	0,0286048574336265	31,3626131085233	31,1488485026459	31,2024247921078	31,4047907964564	31,5806609515781	31,5283219649286	36853147119286,4	48113852130164,6	−0,384664484984674	0,00356057201716659	0,0028663980125308	1,30555612996712	0.384664484984674	0,765957109806671	-0.384664484984674	3636
Q8BY35	sp|Q8BY35|FGD2_MOUSE FYVE, RhoGEF and PH domain-containing protein 2 OS=Mus musculus GN=Fgd2 PE=1 SV=1;sp|Q8BY35-2|FGD2_MOUSE Isoform 2 of FYVE, RhoGEF and PH domain-containing protein 2 OS=Mus musculus GN=Fgd2	1,31	0,0644622915358572	0,38006509013234	27,3177386114909	26,894872517499	26,9324340242253	27,3601226677854	27,2136596408025	27,3724149190948	558403906585,917	729335057533,405	−0,385272708277447	0,0086531528492412	0,0032334522919335	1,30610665314389	0.385272708277446	0,765634259340867	-0.385272708277446	3637
P22366-2	sp|P22366-2|MYD88_MOUSE Isoform 2 of Myeloid differentiation primary response protein MyD88 OS=Mus musculus GN=Myd88;sp|P22366|MYD88_MOUSE Myeloid differentiation primary response protein MyD88 OS=Mus musculus GN=Myd88 PE=1 SV=3	1,31	0,0175563804118178	0,179292377689348	22,2077631703379	22,5407241994077	22,4379044725108	22,672615926723	22,7520392588109	22,5636759051768	5323857371,62872	6955350317,67158	−0,38565125894634	0,00761267876542912	0,0041727601142418	1,30644940917035	0.385651258946339	0,765433389904503	-0.385651258946339	3638
P50428	sp|P50428|ARSA_MOUSE Arylsulfatase A OS=Mus musculus GN=Arsa PE=2 SV=2	1,31	0,26060422967023	0,682439759823898	24,7747333955639	25,3648968031627	25,4944981642208	25,6464523299023	25,3552094720957	25,4354333008474	88953190686,9836	116252652801,164	−0,386145377883197	0,0152175671817096	0,0059043354686060	1,30689694100175	0.386145377883202	0,765171276040704	-0.386145377883202	3639
Q8BSF4	sp|Q8BSF4|PISD_MOUSE Phosphatidylserine decarboxylase proenzyme OS=Mus musculus GN=Pisd PE=2 SV=1	1,31	0,373759208933341	0,744143356508855	22,5528552230193	22,6338453533699	23,4824267429013	23,0613359467876	23,2107589666823	23,2006453878741	8727188516,60373	11407993153,2588	−0,386456154667536	0,0224949729334314	0,0036058662878646	1,30717849529144	0.386456154667539	0,765006465147705	-0.386456154667538	3640
Q63810-2	sp|Q63810-2|CANB1_MOUSE Isoform 2 of Calcineurin subunit B type 1 OS=Mus musculus GN=Ppp3r1;sp|Q63810|CANB1_MOUSE Calcineurin subunit B type 1 OS=Mus musculus GN=Ppp3r1 PE=1 SV=3	1,31	0,308331313521571	0,71853230316343	31,2308454760787	31,3817725927108	30,9049840027489	30,9997610762329	31,6098246071065	31,7123495077502	34519103931196,6	45133463135087,7	−0,386802467599724	0,00781727401833248	0,0122530117850957	1,30749231570575	0.386802467599724	0,764822850572721	-0.386802467599724	3641
Q8R191	sp|Q8R191|SNG3_MOUSE Synaptogyrin-3 OS=Mus musculus GN=Syngr3 PE=1 SV=1	1,31	0,257148739904079	0,67968190098123	31,1656015127658	31,63710827341	31,6602286177643	31,7966976397976	31,4372046772841	32,033953090713	47305426719599,6	61863521135082,9	−0,387083256596027	0,00886501012005073	0,0094613302772261	1,30774681521796	0.387083256596026	0,764674009038464	-0.387083256596026	3642
Q8CCJ3	sp|Q8CCJ3|UFL1_MOUSE E3 UFM1-protein ligase 1 OS=Mus musculus GN=Ufl1 PE=1 SV=2;sp|Q8CCJ3-1|UFL1_MOUSE Isoform 2 of E3 UFM1-protein ligase 1 OS=Mus musculus GN=Ufl1	1,31	0,0657270924316224	0,382601734678419	25,7291565919797	25,5351249444596	25,3323809620451	25,9455567246878	25,8217373765899	25,6347612167554	122603429461,705	160359676280,04	−0,387312075576815	0,00777072067599657	0,0060643198806247	1,30795424715365	0.387312075576816	0,764552737357736	-0.387312075576816	3643
P34022	sp|P34022|RANG_MOUSE Ran-specific GTPase-activating protein OS=Mus musculus GN=Ranbp1 PE=1 SV=2	1,31	0,279765641552586	0,699221280539289	28,8875389662165	29,1143759145037	29,5664435234524	29,6912193119391	29,183067170165	29,4999946244056	4751363765961,94	6215664178333,91	−0,387566895333871	0,0118406808067714	0,0087125214023735	1,30818528837173	0.38756689533387	0,764417708170896	-0.38756689533387	3644
P97825	sp|P97825|HN1_MOUSE Hematological and neurological expressed 1 protein OS=Mus musculus GN=Hn1 PE=1 SV=3	1,31	4,56×10^−08	4,22×10^−06	24,6453225574227	24,5072217421758	24,6380315993696	24,8936242018883	24,8266336914146	24,8771377433071	48114943091,2769	62962084292,6289	−0,387998278129049	0,00315948374630116	0,0014037388774922	1,30857650965493	0.387998278129048	0,764189172449455	-0.387998278129048	3645
Q9D735	sp|Q9D735|CS043_MOUSE Uncharacterized protein C19orf43 homolog OS=Mus musculus PE=1 SV=1	1,31	0,551156150353227	0,838423390503939	21,8876980909906	23,0354461367347	22,6823866658236	22,8291248292886	22,2752567028884	23,3098271341832	6122136442,82013	8016247223,91477	−0,388891804170675	0,0260865173054903	0,0227021364118698	1,30938722107639	0.388891804170674	0,763716022200019	-0.388891804170674	3646

P27546-2	sp|P27546-2|MAP4_MOUSE Isoform 2 of Microtubule-associated protein 4 OS=Mus musculus GN=Map4;sp|P27546|MAP4_MOUSE Microtubule-associated protein 4 OS=Mus musculus GN=Map4 PE=1 SV=3;sp|P27546-3|MAP4_MOUSE Isoform 3 of Microtubule-associated protein 4 OS=	1,31	0,105220170541131	0,468689258180758	24,5842883547047	24,9340519903573	24,3990475359719	24,8003069736163	24,9418514401016	24,9839800051415	50192391764,3052	65722870460,6422	−0,388926796745703	0,0110265926455086	0,0038627559809325	1,30941898065479	0.388926796745702	0,763697498488942	-0.388926796745702	3647
Q3UTH8-2	sp|Q3UTH8-2|ARHG9_MOUSE Isoform 2 of Rho guanine nucleotide exchange factor 9 OS=Mus musculus GN=Arhgef9;sp|Q3UTH8-4|ARHG9_MOUSE Isoform 4 of Rho guanine nucleotide exchange factor 9 OS=Mus musculus GN=Arhgef9;sp|Q3UTH8|ARHG9_MOUSE Rho guanine nucleotid	1,31	0,00272730971179294	0,0493197561029011	27,3540715475072	27,235518194489	27,3412496695829	27,5472752363342	27,7361956183784	27,4581127618677	725610933504,701	950760352239,411	−0,389885547994907	0,00238232918502879	0,0051482818545633	1,31028945174136	0.389885547994906	0,763190147544126	-0.389885547994906	3648
Q6ZWZ2	sp|Q6ZWZ2|UB2R2_MOUSE Ubiquitin-conjugating enzyme E2 R2 OS=Mus musculus GN=Ube2r2 PE=2 SV=1	1,31	0,105982918976039	0,469781708234609	25,9967084867646	26,2266208709123	26,5695977401837	26,5658421318017	26,5247335525472	26,513257270011	25494381707 1,593	334068194685,322	−0,38996328593309	0,0109768030811135	0,0010419958409049	1,31036005706116	0.389963285933089	0,763149025041846	-0.389963285933089	3649
P47963	sp|P47963|RL13_MOUSE 60S ribosomal protein L13 OS=Mus musculus GN=Rpl13 PE=2 SV=3	1,31	0,352332765820676	0,732843629012038	26,8832357761076	27,7647974432772	27,6503089516748	27,8617602051029	27,5544638957705	27,6931390764562	820172666291,613	1074762753905,04	−0,390018661267432	0,0174736288116047	0,0055550112067126	1,3104103539131	0.390018661267432	0,763119733458942	-0.390018661267432	3650
P70195	sp|P70195|PSB7_MOUSE Proteasome subunit beta type-7 OS=Mus musculus GN=Psmb7 PE=1 SV=1	1,31	0,397128856219553	0,754338506152154	29,6401923534164	30,2310954123744	30,3400376693966	30,8001322897362	30,0350880404903	30,1871752749852	1146639628687 9,2	15025930658100,8	−0,390042304035644	0,0125228442364693	0,0133482922555267	1,31043182898665	0.390042304035637	0,763107227617706	-0.390042304035637	3651
O88622-2	sp|O88622-2|PARG_MOUSE Isoform 2 of Poly(ADP-ribose) glycohydrolase OS=Mus musculus GN=Parg;sp|O88622|PARG_MOUSE Poly(ADP-ribose) glycohydrolase OS=Mus musculus GN=Parg PE=1 SV=2;sp|Q86W56-5|PARG_HUMAN Isoform 5 of Poly(ADP-ribose) glycohydrolase OS=Hom	1,31	0,381173250350229	0,749797813889279	25,2754018283628	25,2111392176563	24,6324302907486	25,2111392176563	25,7523846556078	24,9676751874106	74917782231,0275	98212534516,1195	−0,390598969784328	0,0141427445179905	0,0158694134787799	1,31093755836574	0.390598969784328	0,762812838505164	-0.390598969784328	3652
Q5SRX1-2	sp|Q5SRX1-2|TM1L2_MOUSE Isoform 2 of TOM1-like protein 2 OS=Mus musculus GN=Tom1l2	1,31	0,0670176076915818	0,384081971353649	30,0323123257719	30,4732906815785	30,4662615796495	30,5524835884731	30,5924988627797	30,6402261423119	14774958380547,3	19376256316121,9	−0,391135788269227	0,00832985994824711	0,0014357779724951	1,31142544141665	0.39113578826923	0,762529053058298	-0.39113578826923	3653
Q9D517	sp|Q9D517|PLCC_MOUSE 1-acyl-sn-glycerol-3-phosphate acyltransferase gamma OS=Mus musculus GN=Agpat3 PE=1 SV=2	1,31	0,44484835036622	0,783837990028056	24,9733867043026	25,6064342261004	25,8273556009734	25,9119492395323	25,2553298211587	26,0532668033667	115099043651,64	150945088406,412	−0,391147967556843	0,017403432442891	0,0165421482660905	1,31143651256794	0.39114796755685	0,762522615785561	-0.39114796755685	3654
Q925I1-2	sp|Q925I1-2|ATAD3_MOUSE Isoform 2 of ATPase family AAA domain-containing protein 3 OS=Mus musculus GN=Atad3;sp|Q925I1|ATAD3_MOUSE ATPase family AAA domain-containing protein 3 OS=Mus musculus GN=Atad3 PE=1 SV=1	1,31	0,174708951799163	0,590462219359101	23,4227004245546	24,0531878454148	23,9091561309391	24,1615766174121	23,9566725543437	24,0804450555623	21579611639,5951	28302936772,9135	−0,3912828565271	0,0138843467749778	0,0042877030546423	1,31155913487258	0.3912828565271	0,76245132484793	-0.3912828565271	3655
Q64362	sp|Q64362|AKTIP_MOUSE AKT-interacting protein OS=Mus musculus GN=Aktip PE=2 SV=1	1,31	0,224891370498199	0,652238880554131	25,8289851792492	25,9104073439626	25,9818743454278	26,147377349823	26,5728087866134	25,8152971897798	178363413755,228	233978353606,979	−0,391555348518689	0,00295280500969144	0,0145047783765732	1,31180688169645	0.391555348518689	0,762307328885777	-0.391555348518689	3656
Q9D1B9	sp|Q9D1B9|RM28_MOUSE 39S ribosomal protein L28, mitochondrial OS=Mus musculus GN=Mrpl28 PE=2 SV=3	1,31	0,177419573456428	0,595358910106914	24,6324302907486	24,9689418598456	25,1861283043439	25,4105254331557	25,1986693862256	24,9925963663285	67081009186,6994	87999508123,4977	−0,391591066375438	0,011910565095268	0,0082923115381086	1,31183935946131	0.391591066375438	0,762288456119079	-0.391591066375438	3657
Q64105	sp|Q64105|SPRE_MOUSE Sepiapterin reductase OS=Mus musculus GN=Spr PE=1 SV=1	1,31	0,185460041078561	0,609238393171755	29,9143750348548	30,3721900067996	30,5656049997843	30,6656706138036	30,4288703183794	30,5719607650948	14197069421648,2	18624528524617,8	−0,391610747172679	0,0110434650116166	0,0039029039121591	1,31185725528808	0.391610747172678	0,76227805728787 1	-0.391610747172678	3658
Q9ERE7	sp|Q9ERE7|MESD_MOUSE LDLR chaperone MESD OS=Mus musculus GN=Mesdc2 PE=1 SV=1	1,31	0,363460985357852	0,738142913887825	26,7143626330946	27,4646552402382	27,6904097335274	27,6529615702981	27,4925739899774	27,5386383573167	710908241088,42	932739046609,774	−0,39181015402513	0,0187240184167697	0,0029961895575840	1,31203859049619	0.39181015402513	0,762172703793583	-0.39181015402513	3659
Q8C5P7	sp|Q8C5P7|TDRP_MOUSE Testis development-related protein OS=Mus musculus GN=Tdrp PE=2 SV=1	1,31	0,250138471723297	0,673538059877323	23,707498296656	24,35916318947	24,3832569119608	24,5308608808026	24,2586916472883	24,4760775927859	30775132035,6619	40391156803,8249	−0,392274419088026	0,0158751101275421	0,0058944081585523	1,31246087773141	0.392274419088028	0,761927473014281	-0.392274419088028	3660
Q3UHL1	sp|Q3UHL1|CAMKV_MOUSE CaM kinase-like vesicle-associated protein OS=Mus musculus GN=Camkv PE=1 SV=2	1,31	0,182425215104576	0,601764572209063	31,9593241419042	32,6101813602848	32,3276858928127	32,7150311669666	32,5562887960631	32,4429895051478	106489105315041	139828318472032	−0,392950730663823	0,0101046661850139	0,0041954536373098	1,31307628191972	0.392950730663826	0,761570377722454	-0.392950730663827	3661
Q8C7M3	sp|Q8C7M3|TRIM9_MOUSE E3 ubiquitin-protein ligase TRIM9 OS=Mus musculus GN=Trim9 PE=1 SV=2;sp|Q8C7M3-2|TRIM9_MOUSE Isoform 2 of E3 ubiquitin-protein ligase TRIM9 OS=Mus musculus GN=Trim9;sp|Q8C7M3-5|TRIM9_MOUSE Isoform 5 of E3 ubiquitin-protein ligase T	1,31	0,0248821503864219	0,219211300821997	28,8443921491069	28,8709870434371	28,5307522510992	29,0383137035681	28,9217714421477	29,104176927775	3057780620157,7	4016454608196,83	−0,393437667493493	0,0065820402386331	0,0031827755187350	1,31351954477025	0.393437667493493	0,761313376707244	-0.393437667493493	3662

Q9WVA2	sp|Q9WVA2|TIM8A_MOUSE Mitochondrial import inner membrane translocase subunit Tim8 A OS=Mus musculus GN=Timm8a1 PE=1 SV=1;sp|Q90YI5|TIM8A_TAKRU Mitochondrial import inner membrane translocase subunit Tim8 A OS=Takifugu rubripes GN=timm8a PE=3 SV=1;sp|Q4	1,31	0,127939140036547	0,511795090773976	24,5625402578922	24,7917929036	24,7055174223122	24,7031955909906	24,903996713159	25,2712628519499	52633406584,679	69145931124,602	−0,393665585633069	0,00469035946228125	0,0115415038007808	1,31372707205179	0.393665585633071	0,761193113298783	-0.393665585633071	3663
Q9JKK1-2	sp|Q9JKK1-2|STX6_MOUSE Isoform 2 of Syntaxin-6 OS=Mus musculus GN=Stx6;sp|Q9JKK1|STX6_MOUSE Syntaxin-6 OS=Mus musculus GN=Stx6 PE=1 SV=1	1,31	0,172636755676165	0,586864499748362	26,8812927262519	27,1210844296353	27,4198860408206	27,6642808277829	27,3015176496588	27,275090612165	612463018083,085	804614965758,318	−0,39367583867627	0,00994205580010107	0,00793298971786698	1,31373640856984	0.39367583867627	0,761187703619038	-0.39367583867627	3664
Q8R238	sp|Q8R238|SDSL_MOUSE Serine dehydratase-like OS=Mus musculus GN=Sdsl PE=2 SV=1	1,31	0,229123800623354	0,653672489647647	24,1615766174121	24,2731664169751	24,882110644788	24,6324302907486	24,752533055932	24,752533055932	41086737852,6378	54013443210,5066	−0,394645729148309	0,015869004707806	0,00280592279913966	1,31461990008143	0.394645729148308	0,760676146723519	-0.394645729148308	3665
O54786-2	sp|O54786-2|DFFA_MOUSE Isoform ICAD-S of DNA fragmentation factor subunit alpha OS=Mus musculus GN=Dffa;sp|O54786|DFFA_MOUSE DNA fragmentation factor subunit alpha OS=Mus musculus GN=Dffa PE=1 SV=2	1,31	0,267275911513277	0,685404159524725	24,4227555609276	24,8358716084403	25,1715068365119	25,2380141227444	25,1612997878741	24,8516604772475	59547769053,3792	78287840120,0565	−0,394740782817599	0,0151165864836482	0,00815449268260179	1,31470651822201	0.394740782817598	0,760626030326818	-0.394740782817599	3666
Q9Z204-2	sp|Q9Z204-2|HNRPC_MOUSE Isoform C1 of Heterogeneous nuclear ribonucleoproteins C1/C2 OS=Mus musculus GN=Hnrnpc;sp|Q9Z204-4|HNRPC_MOUSE Isoform 4 of Heterogeneous nuclear ribonucleoproteins C1/C2 OS=Mus musculus GN=Hnrnpc;sp|Q9Z204-3|HNRPC_MOUSE Isoform	1,32	0,140908526683182	0,532098608288336	28,8422515451511	28,2148956435959	28,6297384787753	28,7823143927038	28,8541446687965	28,8732918283341	2537740470504,03	3338636579828,73	−0,395714525183181	0,0111710010667832	0,00166329324411383	1,31559417467367	0.39571452518318	0,760112821454264	-0.39571452518318	3667
P54728	sp|P54728|RD23B_MOUSE UV excision repair protein RAD23 homolog B OS=Mus musculus GN=Rad23b PE=1 SV=2	1,32	0,00984362820949065	0,121744491669967	30,1096724551239	30,4606427564896	30,2207870528903	30,4899709322161	30,5656049997843	30,5591116316026	13910996450311,5	18305619183813,8	−0,396060808919832	0,00592714467110401	0,00137266620431781	1,31590998884943	0.396060808919836	0,759930396815634	-0.396060808919836	3668
Q2NL51	sp|Q2NL51|GSK3A_MOUSE Glycogen synthase kinase-3 alpha OS=Mus musculus GN=Gsk3a PE=1 SV=2	1,32	0,144657403895577	0,539068536314443	28,6581410656887	29,1855954237581	29,2338980175545	29,3723329959481	29,2449656239036	29,2839283038169	4034397826635,02	5308916994572,47	−0,396064231746541	0,0110034267046605	0,00222739128484607	1,31591311087942	0.396064231746541	0,759928593865671	-0.396064231746541	3669
P70445	sp|P70445|4EBP2_MOUSE Eukaryotic translation initiation factor 4E-binding protein 2 OS=Mus musculus GN=Eif4ebp2 PE=2 SV=1	1,32	0,458461820649776	0,790967157530661	21,7796036535179	22,2860368944435	22,9837162468125	22,4108797662297	22,84403303344	22,6191277512212	5086142889,67607	6695357305,21963	−0,396589055083934	0,0270509198316788	0,0095749423289766	1,3163919005913	0.396589055083934	0,759652197457926	-0.396589055083934	3670
Q8R526	sp|Q8R526|PK1L1_MOUSE Polycystic kidney disease protein 1-like 1 OS=Mus musculus GN=Pkd1l1 PE=1 SV=2	1,32	0,28506087383079	0,704836107321858	25,500765153124	24,9755754828187	25,0120600285972	25,5531568912004	25,0599359090997	25,7001815061086	84735478340,7269	111552157878,797	−0,396680370828043	0,0116542736267651	0,013183548038747	1,31647522458348	0.396680370828044	0,759604116603403	-0.396680370828044	3671
O70456	sp|O70456|1433S_MOUSE 14-3-3 protein sigma OS=Mus musculus GN=Sfn PE=1 SV=2	1,32	0,152009024336899	0,553091666781035	32,3520069298941	32,8872840950895	32,9002084952502	33,0753864301016	32,8606940502048	33,0286081008433	161119349373908	212131933963464	−0,396832055326381	0,00956312556398134	0,00342238134065385	1,31661364564706	0.396832055326376	0,759524256266188	-0.396832055326376	3672
Q8K2T8	sp|Q8K2T8|PAF1_MOUSE RNA polymerase II-associated factor 1 homolog OS=Mus musculus GN=Paf1 PE=2 SV=1	1,32	0,433301488559034	0,7767413243707	24,7970047172291	24,2505125478808	23,9931989064267	24,6522337538154	25,0570831721089	24,1582085472173	37473707493,6934	49365048527,6651	−0,397611226395468	0,016859677562639	0,0182830257454117	1,31732491470114	0.397611226395468	0,759114162982995	-0.397611226395468	3673
Q5SVL6	sp|Q5SVL6|RPGP2_MOUSE Rap1 GTPase-activating protein 2 OS=Mus musculus GN=Rap1gap2 PE=2 SV=1;sp|Q5SVL6-2|RPGP2_MOUSE Isoform 2 of Rap1 GTPase-activating protein 2 OS=Mus musculus GN=Rap1gap2	1,32	0,470111107956569	0,796715861328205	29,8955901225831	29,0701689048778	29,0045580088892	29,9610005225932	29,7220781608845	29,1143759145037	5432600301389,03	7157282835738,9	−0,39776908609952	0,0169346503207019	0,0147468227301764	1,31746906429116	0.397769086099521	0,759031105248781	-0.397769086099521	3674
Q921H9	sp|Q921H9|COA7_MOUSE Cytochrome c oxidase assembly factor 7 OS=Mus musculus GN=Coa7 PE=2 SV=1	1,32	0,19236169669067	0,618802084535046	26,2985172198808	26,6864288116258	26,9273907888982	27,122691076725	26,7811512810363	26,8359983838767	370250487511,191	487853136257,384	−0,397945267826466	0,0119112536756759	0,0068151038855337	1,31762996326274	0.397945267826466	0,758938418130518	-0.397945267826466	3675
Q9CRA5	sp|Q9CRA5|GOLP3_MOUSE Golgi phosphoprotein 3 OS=Mus musculus GN=Golph3 PE=1 SV=1	1,32	0,0708557399952579	0,396415636831615	26,3290904224167	26,1085492621562	26,6322995250514	26,6674061630996	26,6200366760221	26,6107194319358	279605747489,283	368505235848,577	−0,398291101159844	0,00997699888733939	0,00114136089671122	1,31794585468134	0.398291101159843	0,75875651222545	-0.398291101159843	3676
P49722	sp|P49722|PSA2_MOUSE Proteasome subunit alpha type-2 OS=Mus musculus GN=Psma2 PE=1 SV=3	1,32	0,141219985600843	0,532098608288336	30,3862745237618	30,871713126719	30,9907657784686	31,0751300491871	30,9631543867588	31,039393479464	22613868316231,7	29810823210088,8	−0,398628415296041	0,0104137041127982	0,0018434810364569	1,31825403744353	0.398628415296043	0,758579129360592	-0.398628415296044	3677

Q8BH60-2	sp|Q8BH60-2|GOPC_MOUSE Isoform 2 of Golgi-associated PDZ and coiled-coil motif-containing protein OS=Mus musculus GN=Gopc;sp|Q8BH60|GOPC_MOUSE Golgi-associated PDZ and coiled-coil motif-containing protein OS=Mus musculus GN=Gopc PE=1 SV=1	1,32	0,335687189961376	0,72953198881961	24,6071514587832	25,3797969498621	25,5185024275634	25,4520364849735	25,520279157572	25,3625137240185	85218434271,7053	112356548691,512	−0,398846764254439	0,019510513541928	0,0031095196694823	1,31845356760817	0.398846764254438	0,758464328640803	-0.398846764254438	3678
F6SEU4	sp|F6SEU4|SYGP1_MOUSE Ras/Rap GTPase-activating protein SynGAP OS=Mus musculus GN=Syngap1 PE=1 SV=2	1,32	0,00627682773513122	0,0888740760486895	25,0703734773173	24,8936242018883	24,7814467480862	25,2582028351988	25,2380141227444	25,0796635391775	66147183057,1506	87242738282,1112	−0,399355333239194	0,0058462822780866	0,0038811670440860	1,31891842176762	0.399355333239194	0,758197007105104	-0.399355333239194	3679
P50429	sp|P50429|ARSB_MOUSE Arylsulfatase B OS=Mus musculus GN=Arsb PE=2 SV=3;sp|P50429-2|ARSB_MOUSE Isoform 2 of Arylsulfatase B OS=Mus musculus GN=Arsb	1,32	0,0286101410874179	0,238644139389349	27,5899208736384	27,1760569863579	27,4118429955384	27,7067011958313	27,7129019603996	27,5887500513035	787876313440,11	1039177934771,13	−0,399401635175958	0,00757873894016001	0,0025283431291093	1,3189607518898	0.399401635175959	0,758172673877677	-0.399401635175959	3680
P97300	sp|P97300|NPTN_MOUSE Neuroplastin OS=Mus musculus GN=Nptn PE=1 SV=3;sp|P97300-1|NPTN_MOUSE Isoform 1 of Neuroplastin OS=Mus musculus GN=Nptn	1,32	0,208624731734819	0,639143370435791	31,3439939544913	31,9696296459334	31,9339369527224	32,1512238864603	31,8619322988203	32,0651775689469	61446429803930,2	81051936088702,6	−0,399517459100725	0,0110667672423494	0,0046383959503248	1,31906664630202	0.399517459100725	0,758111807923795	-0.399517459100725	3681
O35593	sp|O35593|PSDE_MOUSE 26S proteasome non-ATPase regulatory subunit 14 OS=Mus musculus GN=Psmd14 PE=1 SV=2	1,32	0,563070893468314	0,845110794140252	28,1252116374367	29,1607446221874	29,7312890475709	29,142784813033	29,2505827592898	29,4546675630231	3953998398846,53	5215616314176,76	−0,399525455031471	0,0280696074026972	0,0054093141922177	1,31907395706034	0.399525455031472	0,758107606209264	-0.399525455031472	3682
Q9D020-1	sp|Q9D020-1|5NT3A_MOUSE Isoform 1 of Cytosolic 5-nucleotidase 3A OS=Mus musculus GN=Nt5c3a;sp|Q9D020|5NT3A_MOUSE Cytosolic 5-nucleotidase 3A OS=Mus musculus GN=Nt5c3a PE=1 SV=4	1,32	0,213503388982632	0,645012843958033	27,4194342735147	27,9400277759159	28,159207652022	28,1559691605545	28,0390196313671	28,155149069996	1231872600676,31	1625298521926,28	−0,399851663919934	0,0136490287679127	0,0023930732144401	1,31937224761227	0.399851663919933	0,757936209291763	-0.399851663919933	3683
Q14BB9	sp|Q14BB9|MA6D1_MOUSE MAP6 domain-containing protein 1 OS=Mus musculus GN=Map6d1 PE=1 SV=1	1,32	0,0218847232424627	0,20661982619166	27,4089401757686	27,6361586212546	27,8257406617401	27,9035914445976	27,918409250953	27,8808173603113	992619408320,205	1309857347211,85	−0,400097132053303	0,00755452440255149	0,0006786779888302	1,3195967520205	0.400097132053305	0,757807260793006	-0.400097132053305	3684
Q8BQP9	sp|Q8BQP9|R7BP_MOUSE Regulator of G-protein signaling 7-binding protein OS=Mus musculus GN=Rgs7bp PE=1 SV=1	1,32	0,0536512073566554	0,341068389624452	25,6844554864885	26,1166289768567	26,0532668033667	26,308372107962	26,1356881565921	26,242967719549	186454004740,292	246101361845,108	−0,400432856948013	0,00899312698015855	0,0033239563745009	1,31990386684318	0.400432856948007	0,757630934434418	-0.400432856948007	3685
B7ZMP1	sp|B7ZMP1|XPP3_MOUSE Probable Xaa-Pro aminopeptidase 3 OS=Mus musculus GN=Xpnpep3 PE=2 SV=1;sp|B7ZMP1-2|XPP3_MOUSE Isoform 2 of Probable Xaa-Pro aminopeptidase 3 OS=Mus musculus GN=Xpnpep3	1,32	0,0387296497749722	0,288186689063277	25,4581618858169	25,848124338265	25,7580916626282	26,0967000396117	25,9004504146625	25,9023194601262	143288506433,322	189279381867,475	−0,401594375678193	0,0079483818512666	0,0043428703397679	1,32096695386768	0.401594375678191	0,757021208647259	-0.401594375678191	3686
Q8BX10-2	sp|Q8BX10-2|PGAM5_MOUSE Isoform 2 of Serine/threonine-protein phosphatase PGAM5, mitochondrial OS=Mus musculus GN=Pgam5;sp|Q8BX10|PGAM5_MOUSE Serine/threonine-protein phosphatase PGAM5, mitochondrial OS=Mus musculus GN=Pgam5 PE=2 SV=1	1,32	0,286093490099751	0,704836107321858	26,5451722092463	27,122691076725	27,3684215764684	27,4410098735373	27,3125156226977	27,1190534987677	538522413161,51	711655416059,885	−0,402172465924443	0,0156456208776248	0,0059385083896820	1,32149637353432	0.402172465924444	0,756717929785549	-0.402172465924443	3687
Q9Z1K6	sp|Q9Z1K6|ARI2_MOUSE E3 ubiquitin-protein ligase ARIH2 OS=Mus musculus GN=Arih2 PE=1 SV=1	1,32	0,00197671681001337	0,0380182949180113	23,8464232723357	23,921929541055	23,9449572067503	24,2860801827562	24,2494398701144	24,0144583554802	24074924856,0176	31818895109,8045	−0,40235244484626	0,00215623233096731	0,0060945634818509	1,32166124297793	0.402352444846258	0,75662353368767	-0.402352444846258	3688
Q9CYH5	sp|Q9CYH5|GFOD2_MOUSE Glucose-fructose oxidoreductase domain-containing protein 2 OS=Mus musculus GN=Gfod2 PE=2 SV=1	1,32	0,317186905291324	0,722907157640623	21,616411665443	21,5346685350988	21,4030055681932	21,3656840009079	21,7258576307335	22,2997639006898	2213915688,82228	2926584393,91137	−0,402617600358118	0,00500379264299885	0,0216128631821247	1,32190417579461	0.40261760035812	0,756484485268299	-0.40261760035812	3689
Q8JZY2	sp|Q8JZY2|COMDA_MOUSE COMM domain-containing protein 10 OS=Mus musculus GN=Commd10 PE=2 SV=1	1,32	0,0706517625579729	0,395977259611237	26,2677834736084	26,5920726184234	26,7926130600652	26,7968413620251	26,8215162251178	26,8719382927359	339528270239,906	448914654082,285	−0,402909488431634	0,00997465021884849	0,0014266448727612	1,32217165234897	0.402909488431634	0,7563314477537	-0.402909488431634	3690
Q9QXL2-3	sp|Q9QXL2-3|KI21A_MOUSE Isoform 3 of Kinesin-like protein KIF21A OS=Mus musculus GN=Kif21a;sp|Q9QXL2-2|KI21A_MOUSE Isoform 2 of Kinesin-like protein KIF21A OS=Mus musculus GN=Kif21a;sp|Q9QXL2-4|KI21A_MOUSE Isoform 4 of Kinesin-like protein KIF21A OS=Mus	1,32	0,17379315870166	0,5886919010051	23,2867314959679	23,9597534682194	23,80523416359	24,0144583554802	23,9621918362882	23,9139624756217	19310336818,3645	25540660683,5448	−0,403422516477668	0,0148849448696641	0,0020974137555235	1,3226419054097	0.403422516477668	0,75606254112313	-0.403422516477668	3691
Q6QWF9	sp|Q6QWF9|CK2N1_MOUSE Calcium/calmodulin-dependent protein kinase II inhibitor 1 OS=Mus musculus GN=Camk2n1 PE=1 SV=1	1,32	0,381879396767984	0,749797813889279	26,030206424081	25,2267225649949	25,3954169770848	25,4600043280656	25,8593137304936	26,1720458506377	124900341557,29	165205276302,269	−0,403482341878395	0,0165816744613111	0,013816904659763	1,32269675360729	0.403482341878395	0,756031189517006	-0.403482341878395	3692
Q8K4F5	sp|Q8K4F5|ABHDB_MOUSE Alpha/beta hydrolase domain-containing protein 11 OS=Mus musculus GN=Abhd11 PE=1 SV=1	1,32	0,180003334865227	0,598356502628386	26,3439130720978	26,6900416088642	27,039238096365	27,0652717104084	26,868788726629	26,9782391770371	390644688649,259	516719772309,544	−0,403525090717191	0,013025469378251	0,0036504120312841	1,32273594732906	0.403525090717192	0,756008787709484	-0.403525090717192	3693
Q8BH66	sp|Q8BH66|ATLA1_MOUSE Atlastin-1 OS=Mus musculus GN=Atl1 PE=1 SV=1	1,32	0,170186336732607	0,582121915922611	30,8043490382255	31,3724626453784	31,2915687270389	31,63710827341	31,3267661620555	31,3439939544913	33957362299374,4	44922330698134,4	−0,403708381547444	0,00986392643740312	0,0055482869140616	1,32290400832935	0.403708381547444	0,755912744767373	-0.403708381547444	3694

Q9D0F3	sp|Q9D0F3|LMAN1_MOUSE Protein ERGIC-53 OS=Mus musculus GN=Lman1 PE=2 SV=1	1,32	0,129868479701054	0,513077226998934	25,9585083386998	26,3554364395044	26,3924755423625	26,7026272203015	26,5613075057639	26,2847000887215	247697341387,551	327977749103,043	−0,40501955805871	0,00916974878850092	0,0080170484731202	1,32410686067835	0.405019558058719	0,755226054404472	-0.405019558058719	3695
Q9CXT8	sp|Q9CXT8|MPPB_MOUSE Mitochondrial-processing peptidase subunit beta OS=Mus musculus GN=Pmpcb PE=2 SV=1	1,32	0,00513848405010382	0,0782670439712122	27,3309114068393	27,1109039589626	27,0697573276934	27,5285613999933	27,4902079656322	27,3351344193571	630970048199,374	835504241576,083	−0,40507562949178	0,00516790648003556	0,0037307824333372	1,32415832409224	0.405075629491781	0,75519670254351	-0.405075629491781	3696
Q99PL5	sp|Q99PL5|RRBP1_MOUSE Ribosome-binding protein 1 OS=Mus musculus GN=Rrbp1 PE=1 SV=2	1,32	0,497116648161211	0,809698318764119	22,3016141097192	21,7051251708901	22,61434833139	21,8565573461608	22,847371833861	22,7607929420049	4409509095,51705	5841425563,52918	−0,40570244131306	0,0208011733797015	0,0244022625695796	1,32473375992532	0.405702441313068	0,754868661350019	-0.405702441313068	3697
Q9CQA3	sp|Q9CQA3|SDHB_MOUSE Succinate dehydrogenase [ubiquinone] iron-sulfur subunit, mitochondrial OS=Mus musculus GN=Sdhb PE=1 SV=1	1,32	0,220176481190513	0,648948732547882	28,7636310588687	29,2928257170814	29,4911659736167	29,57918938533	29,3261521639233	29,4859248540224	4718636121791,52	6250955623560,46	−0,40570683849441	0,0128877337432158	0,0043432028440393	1,32473779757935	0.405706838494411	0,754866360593974	-0.405706838494411	3698
P42669	sp|P42669|PURA_MOUSE Transcriptional activator protein Pur-alpha OS=Mus musculus GN=Pura PE=1 SV=1	1,33	0,00119744436873208	0,0271759999483744	31,7786943855435	31,5462290964587	31,736695594508	31,8752640211253	32,043087513796	31,9879882223648	57753574460603,1	76535815149784,2	−0,40622477903063	0,00390954850564728	0,0026758833918137	1,32521347578224	0.40622477903063	0,754595405400421	-0.40622477903063	3699
Q8BYW1-2	sp|Q8BYW1-2|RHG25_MOUSE Isoform 2 of Rho GTPase-activating protein 25 OS=Mus musculus GN=Arhgap25;sp|Q8BYW1-3|RHG25_MOUSE Isoform 3 of Rho GTPase-activating protein 25 OS=Mus musculus GN=Arhgap25;sp|Q8BYW1|RHG25_MOUSE Rho GTPase-activating protein 25 OS	1,33	0,00548138389884406	0,0817809931121212	26,613068974371	26,3667324253046	26,2939048107703	26,7315571743892	26,7434074548887	26,6443531342949	299257049045,682	396697258835,305	−0,40665319807155	0,00632963762838524	0,0020254905867654	1,32560706623405	0.406653198071555	0,754371355941038	-0.406653198071555	3700
Q9ERG0	sp|Q9ERG0|LIMA1_MOUSE LIM domain and actin-binding protein 1 OS=Mus musculus GN=Lima1 PE=1 SV=3;sp|Q9ERG0-2|LIMA1_MOUSE Isoform Alpha of LIM domain and actin-binding protein 1 OS=Mus musculus GN=Lima1	1,33	0,40408503877651	0,759164731376895	23,591698233046	24,5565050782037	24,6071514587832	24,3929941603799	24,5876185868543	24,620547219318	34073470367,2151	45170948681,0452	−0,40674632085886	0,0235946531866772	0,0050126616493851	1,32569263401206	0.406746320858864	0,754322664503018	-0.406746320858864	3701
Q0VBF8	sp|Q0VBF8|STUM_MOUSE Protein stum homolog OS=Mus musculus PE=2 SV=1	1,33	0,232652822602904	0,657279264635888	26,8620165414284	26,1869977779283	26,6553553897964	27,0009567310953	26,5982162339486	26,9524248585844	345453276437,151	458182130965,537	−0,40743059975169	0,0130178847246942	0,0081881293691871	1,32632156710459	0.407430599751695	0,753964969583536	-0.407430599751695	3702
Q9D2E2	sp|Q9D2E2|TOE1_MOUSE Target of EGR1 protein 1 OS=Mus musculus GN=Toe1 PE=1 SV=1;sp|Q9D2E2-2|TOE1_MOUSE Isoform 2 of Target of EGR1 protein 1 OS=Mus musculus GN=Toe1	1,33	0,393876348072078	0,753881747531964	22,9428230979447	23,9284441294148	22,9522447247002	23,6043336988036	23,4485091486631	23,6185544152518	12822970681,4818	17011108706,2899	−0,40774664430996	0,02433423868712	0,0040046831027615	1,32661215008909	0.40774664430996	0,753799820039976	-0.40774664430996	3703
Q8C145	sp|Q8C145|S39A6_MOUSE Zinc transporter ZIP6 OS=Mus musculus GN=Slc39a6 PE=1 SV=1	1,33	0,484652891884692	0,803590359311294	21,7715837835218	21,8174060215298	22,1674770243792	21,8248730561356	21,8085797576661	22,9717251193296	3305396512,1634	4386186368,46942	−0,40814376681872	0,00987994262342103	0,0300376907370844	1,3269773693803	0.408143766818725	0,753592354379787	-0.408143766818725	3704
Q9CXJ1	sp|Q9CXJ1|SYEM_MOUSE Probable glutamate--tRNA ligase, mitochondrial OS=Mus musculus GN=Ears2 PE=1 SV=1	1,33	0,440672703043316	0,780702789085957	24,5360198544844	25,7191474648085	25,506098678661	25,5777956432148	25,4392340632725	25,5940945146071	92803977113,9237	123195875208,085	−0,40869541466118	0,0249720898785595	0,0033322096044591	1,32748486691312	0.408695414661187	0,753304255983996	-0.408695414661187	3705
Q62203	sp|Q62203|SF3A2_MOUSE Splicing factor 3A subunit 2 OS=Mus musculus GN=Sf3a2 PE=1 SV=2	1,33	0,230967744555037	0,654468548759245	24,5775770341929	25,0998804841175	25,2157838114411	25,3648968031627	24,9839800051415	25,3949548195294	69487573612,1652	92266214923,0438	−0,40904746829049	0,0136188206410141	0,0090737257158974	1,32780884590984	0.409047468290493	0,753120453354703	-0.409047468290493	3706
Q8JZW4	sp|Q8JZW4|CPNE5_MOUSE Copine-5 OS=Mus musculus GN=Cpne5 PE=2 SV=1	1,33	0,0757298098167956	0,410188074890734	28,197075208073	28,6894779101035	28,6169807290695	28,8632336136022	28,7823143927038	28,7093526612442	2387285229636,98	3170679040326,87	−0,40942089654344	0,00932745999474167	0,0026741305731349	1,32815258141945	0.409420896543448	0,752925540325542	-0.409420896543448	3707
O35083	sp|O35083|PLCA_MOUSE 1-acyl-sn-glycerol-3-phosphate acyltransferase alpha OS=Mus musculus GN=Agpat1 PE=2 SV=1	1,33	0,117715575073845	0,491140824870552	26,696871523607	26,9837240922818	27,2953748650026	27,3379076999531	27,1640538722618	27,3269456084881	527803641453,336	701370695359,291	−0,41017584900344	0,0110898559204574	0,0035695625927578	1,32884777647238	0.410175849003448	0,752531642604426	-0.410175849003448	3708
Q8VIJ8	sp|Q8VIJ8|NPRL3_MOUSE Nitrogen permease regulator 3-like protein OS=Mus musculus GN=Nprl3 PE=2 SV=1	1,33	0,196875028425457	0,626219869672844	21,8141497464129	22,3849401581905	22,1923188032946	22,6823866658236	22,3703619013105	22,1923188032946	4084518014,94406	5429009038,96604	−0,41052303968166	0,013121188499303	0,0110670259275779	1,32916760780657	0.410523039681661	0,752350564463595	-0.410523039681662	3709
Q8C407	sp|Q8C407|YIPF4_MOUSE Protein YIPF4 OS=Mus musculus GN=Yipf4 PE=2 SV=1;sp|Q8C407-2|YIPF4_MOUSE Isoform 2 of Protein YIPF4 OS=Mus musculus GN=Yipf4	1,33	0,289952915940622	0,709103602076768	23,3797067172341	24,018150513668	24,2820927735618	24,2030232322995	24,2030232322995	24,1280009506016	23808694550,3809	31650374090,3424	−0,41073403296857	0,0194181574671382	0,0017914677069747	1,32936201198969	0.410734032968575	0,752240541689071	-0.410734032968575	3710
P03995-2	sp|P03995-2|GFAP_MOUSE Isoform 2 of Glial fibrillary acidic protein OS=Mus musculus GN=Gfap;sp|P03995|GFAP_MOUSE Glial fibrillary acidic protein OS=Mus musculus GN=Gfap PE=1 SV=4	1,33	0,552977066290802	0,838891344884342	26,1541626625147	24,8371424988238	26,0494943656724	26,4159750442456	25,6757304025179	25,8041604093304	142166751084,349	189052127091,897	−0,41119998419970	0,0285059313388462	0,0152339189757435	1,32979142907845	0.411199984199701	0,751997627697903	-0.411199984199701	3711

P48774	sp|P48774|GSTM5_MOUSE Glutathione S-transferase Mu 5 OS=Mus musculus GN=Gstm5 PE=1 SV=1	1,33	0,1162942672934	0,487484510268456	32,3027531536818	32,773886273776	32,8606940502048	33,0286081008433	32,839500065196	32,9255457365668	150619488086535	200376250479572	−0,41180307682593	0,00919635475409247	0,0028751300485008	1,33034744059448	0.411803076825934	0,751683334357484	-0.411803076825934	3712
P47802	sp|P47802|MTX1_MOUSE Metaxin-1 OS=Mus musculus GN=Mtx1 PE=1 SV=1	1,33	0,026929444412027	0,22890027750223	25,526381389398	25,8942049257386	25,8313075112595	25,9331697089281	26,030206424081	26,1449400230198	152530631552,861	202925628205,905	−0,411852082622596	0,00764004334521378	0,0040715891485374	1,33039263090954	0.411852082622591	0,751657801438914	-0.411852082622591	3713
Q8R180	sp|Q8R180|ERO1A_MOUSE ERO1-like protein alpha OS=Mus musculus GN=Ero1l PE=1 SV=2	1,33	0,307146192203657	0,71837258919956	27,1975304598764	28,0890345798713	27,966093558593	28,0003802943019	28,0758284429146	28,0336619814108	1127344743932,24	1500206037407,79	−0,41223189164249	0,0174101292161355	0,00134863468632514	1,33074292090544	0.412231891642491	0,751459943382298	-0.412231891642491	3714
P58281-2	sp|P58281-2|OPA1_MOUSE Isoform 2 of Dynamin-like 120 kDa protein, mitochondrial OS=Mus musculus GN=Opa1;sp|P58281|OPA1_MOUSE Dynamin-like 120 kDa protein, mitochondrial OS=Mus musculus GN=Opa1 PE=1 SV=1	1,33	0,0336780599105975	0,266896130438124	31,6276420584925	31,4180321721597	31,2460357843299	31,7211883042871	31,849738476019	31,5806609515781	44681023847876,2	59511810859885,6	−0,413513772648512	0,00608044458337542	0,00424322448401681	1,33192585430681	0.413513772648512	0,750792543568756	-0.413513772648512	3715
Q8K354	sp|Q8K354|CBR3_MOUSE Carbonyl reductase [NADPH] 3 OS=Mus musculus GN=Cbr3 PE=2 SV=1	1,33	0,141490929480061	0,532526806724708	28,2047593538113	28,4835717147051	28,8498970986966	28,9104405271909	28,7307284512667	28,7572558211707	2415053901883,07	3217014698583,75	−0,41366713859165	0,0113477902168821	0,00336846553646451	1,33206745243879	0.41366713859165	0,75071273468109	-0.41366713859165	3716
Q6A0A2	sp|Q6A0A2|LAR4B_MOUSE La-related protein 4B OS=Mus musculus GN=Larp4b PE=1 SV=2;sp|Q6A0A2-2|LAR4B_MOUSE Isoform 2 of La-related protein 4B OS=Mus musculus GN=Larp4b;sp|Q6A0A2-3|LAR4B_MOUSE Isoform 3 of La-related protein 4B OS=Mus musculus GN=Larp4b	1,33	0,380637543763231	0,749797813889279	24,4164001122609	24,0448449514442	23,3213560979182	24,167659258453	24,3357757397456	24,1399594220105	24637455926,4061	32825280641,8548	−0,413954055130765	0,023273098147375	0,00437619995142100	1,33233239421742	0.413954055130762	0,750563451237989	-0.413954055130762	3717
P52623	sp|P52623|UCK1_MOUSE Uridine-cytidine kinase 1 OS=Mus musculus GN=Uck1 PE=2 SV=2	1,33	0,00157931790202799	0,0327329861064157	26,325250003904	26,4937458641267	26,4674873848154	26,7434074548887	26,5758572324515	26,8289507902745	300534307046,326	400536936781,077	−0,414405602415974	0,00343022064998793	0,00481889882702063	1,33274946450402	0.414405602415976	0,750328570097869	-0.414405602415976	3718
Q8VH51-3	sp|Q8VH51-3|RBM39_MOUSE Isoform 3 of RNA-binding protein 39 OS=Mus musculus GN=Rbm39;sp|Q8VH51-2|RBM39_MOUSE Isoform 2 of RNA-binding protein 39 OS=Mus musculus GN=Rbm39;sp|Q8VH51|RBM39_MOUSE RNA-binding protein 39 OS=Mus musculus GN=Rbm39 PE=1 SV=2	1,33	0,018034739351192	0,181507055244037	23,8813417836964	23,8751848769123	24,2277572078905	24,2384213358069	24,3517999664098	24,2573141568899	26350716138,5942	35136693481,4633	−0,415136262936283	0,00841033022360651	0,00250156580368312	1,33342461345864	0.415136262936281	0,749948658444363	-0.415136262936281	3719
Q91ZW2	sp|Q91ZW2|OFUT1_MOUSE GDP-fucose protein O-fucosyltransferase 1 OS=Mus musculus GN=Pofut1 PE=1 SV=1	1,33	0,575045251988551	0,849998193752035	23,3432131075201	22,5764449156736	21,6089602302478	22,6225346670564	22,9990398942442	22,7708085059644	5967174789,87798	7958139055,09331	−0,415383071132537	0,0386085259130014	0,00831945137432756	1,33365274779491	0.415383071132537	0,749820372397101	-0.415383071132537	3720
Q8K0U4	sp|Q8K0U4|HS12A_MOUSE Heat shock 70 kDa protein 12A OS=Mus musculus GN=Hspa12a PE=1 SV=1	1,33	0,00556737758793687	0,0817809931121212	32,4536789640953	32,4047952999906	32,1359937740807	32,6843873490794	32,5913113535174	32,5833119363487	109998665084905	146738060662854	−0,415757107593671	0,00529079627764471	0,00172255634898587	1,33399855852424	0.415757107593673	0,749625997427064	-0.415757107593673	3721
O09053	sp|O09053|WRN_MOUSE Werner syndrome ATP-dependent helicase homolog OS=Mus musculus GN=Wrn PE=1 SV=3;sp|Q14191|WRN_HUMAN Werner syndrome ATP-dependent helicase OS=Homo sapiens GN=WRN PE=1 SV=2	1,33	0,745966923533965	0,927700293161169	23,0407523644372	24,0841927204206	22,383010120132	24,1365945678446	24,2549465990353	21,9810470764094	11542692638,059	15398399531,3535	−0,415800598847794	0,0370251541399021	0,0545683976515252	1,33403877363773	0.415800598847792	0,749603399662173	-0.415800598847792	3722
B9EJA2-2	sp|B9EJA2-2|CTTB2_MOUSE Isoform 2 of Cortactin-binding protein 2 OS=Mus musculus GN=Cttnbp2;sp|B9EJA2-3|CTTB2_MOUSE Isoform 3 of Cortactin-binding protein 2 OS=Mus musculus GN=Cttnbp2;sp|B9EJA2-5|CTTB2_MOUSE Isoform 5 of Cortactin-binding protein 2 OS=M	1,34	0,151924915553909	0,553091666781035	29,5991882691668	29,5177234927931	28,9598110128047	29,6715275253764	29,6607221554744	29,6117382906239	5628744838585,2	7515555013449,94	−0,417066399476361	0,0118539718606971	0,00107465917571200	1,33520975438975	0.417066399476362	0,748945996471574	-0.417066399476362	3723
Q8CDM8	sp|Q8CDM8|F16B1_MOUSE Protein FAM160B1 OS=Mus musculus GN=Fam160b1 PE=2 SV=2	1,34	0,0417335490710505	0,300203770576067	24,974920529059	24,6602962520587	24,547663319805	24,9689418598456	25,1410841300026	24,9418514401016	54836765010,7182	73260870506,3402	−0,41789937904439	0,00895530737159626	0,00431937327620863	1,33598089697707	0.417899379044389	0,748513696762209	-0.417899379044389	3724
Q6IRU5	sp|Q6IRU5|CLCB_MOUSE Clathrin light chain B OS=Mus musculus GN=Cltb PE=1 SV=1;sp|Q6IRU5-2|CLCB_MOUSE Isoform 2 of Clathrin light chain B OS=Mus musculus GN=Cltb;sp|Q6IRU5-3|CLCB_MOUSE Isoform 3 of Clathrin light chain B OS=Mus musculus GN=Cltb	1,34	0,106825888271657	0,47075990957772	30,4337492602583	30,7450551800597	31,0449398168704	31,0862260035438	31,039393479464	30,9678402142619	22426133878287,1	29968059060782,2	−0,418244717463345	0,00994146570349946	0,00192134383061874	1,33630072947157	0.418244717463341	0,748334546218081	-0.418244717463341	3725
Q6ZQ82	sp|Q6ZQ82|RHG26_MOUSE Rho GTPase-activating protein 26 OS=Mus musculus GN=Arhgap26 PE=1 SV=3	1,34	0,0832618409456415	0,429460790968485	28,3225587196079	28,0044923642566	27,7726589193272	28,3382640165987	28,4053017035069	28,2272204810265	1495133576681,35	1998854535925,04	−0,418899103668365	0,00984811325574263	0,00317552342871699	1,33690699419765	0.418899103668366	0,747995189149382	-0.418899103668366	3726
Q9CQ26	sp|Q9CQ26|STABP_MOUSE STAM-binding protein OS=Mus musculus GN=Stambp PE=2 SV=1	1,34	0,576446758926369	0,850431678837126	23,6328120342077	23,2373012347919	23,6005714162421	23,3797067172341	23,1798328591074	24,7822925530271	15910231478,1482	21271004895,768	−0,418933365841623	0,00934999799223529	0,0367198092483039	1,33693874441629	0.41893336584163	0,747977425425427	-0.41893336584163	3727

Q3TVA9-2	sp|Q3TVA9-2|CC136_MOUSE Isoform 2 of Coiled-coil domain-containing protein 136 OS=Mus musculus GN=Ccdc136;sp|Q3TVA9|CC136_MOUSE Coiled-coil domain-containing protein 136 OS=Mus musculus GN=Ccdc136 PE=2 SV=2	1,34	0,0308367351751248	0,250389876493545	26,2733618497883	26,4412629368967	26,7131195606615	26,7355071353139	26,7159035528554	26,8480719120113	315024037520,289	421250792560,403	-0,41921748477237	0,00838185459859742	0,0026647151546481	1,33720206202764	0.419217484772378	0,747830136070587	-0.419217484772378	3728
Q6PD26	sp|Q6PD26|PIGS_MOUSE GPI transamidase component PIG-S OS=Mus musculus GN=Pigs PE=1 SV=3	1,34	0,0453638429531988	0,312598733858675	26,1944717091464	26,5257288518079	26,5816409484069	26,6885628808377	26,6065658100871	26,8788895373272	302076813037,621	403996779261,458	-0,41942834237422	0,00791662181267873	0,0052276343394964	1,33739751554895	0.419428342374227	0,747720844680604	-0.419428342374227	3729
P70290	sp|P70290|EM55_MOUSE 55 kDa erythrocyte membrane protein OS=Mus musculus GN=Mpp1 PE=1 SV=1	1,34	0,184121847336568	0,606039931153503	26,8222501839952	27,4252531095558	27,4732502744015	27,6464734713961	27,4252531095558	27,5217488359946	676535791017,054	904961711770,57	-0,41969049453972	0,0133183098066355	0,0040285248029771	1,33764055617829	0.419690494539727	0,747584988643776	-0.419690494539727	3730
Q9WU84	sp|Q9WU84|CCS_MOUSE Copper chaperone for superoxide dismutase OS=Mus musculus GN=Ccs PE=2 SV=1	1,34	0,278141214630092	0,699221280539289	24,4526054481224	24,7396961746093	24,9939508693433	25,4427882169683	24,7119475537275	24,9059090438174	54898443345,1844	73475285738,9291	-0,42049382245765	0,0109523783765891	0,0151314180638172	1,33838559459581	0.42049382245765	0,747168830894356	-0.42049382245765	3731
Q9QZ08	sp|Q9QZ08|NAGK_MOUSE N-acetyl-D-glucosamine kinase OS=Mus musculus GN=Nagk PE=2 SV=3	1,34	0,00148359742532361	0,0320668986359231	28,1771173987933	28,381044130583	28,3308483775731	28,7217355155172	28,496957284198	28,5454840142889	1945104143243,93	2603971610634,62	-0,42086631891934	0,00375502953706179	0,0041374839010374	1,33873120350866	0.420866318919345	0,746975940636267	-0.420866318919345	3732
P01831	sp|P01831|THY1_MOUSE Thy-1 membrane glycoprotein OS=Mus musculus GN=Thy1 PE=1 SV=1	1,34	0,178843214630351	0,598329724962355	31,1636297730172	31,5806609515781	31,8752640211253	31,9593241419042	31,7285609258969	31,8078616968354	49840627564266,3	66746006800917,6	-0,42135929351889	0,0113370224826151	0,0036833157035362	1,33918873141902	0.421359293518893	0,746720739608068	-0.421359293518893	3733
Q8R0J7	sp|Q8R0J7|VP37B_MOUSE Vacuolar protein sorting-associated protein 37B OS=Mus musculus GN=Vps37b PE=2 SV=1	1,34	0,195015557540935	0,622304200947129	26,1262269484152	26,5989713295845	26,8348651408146	26,882581127589	26,6409567083219	26,9128048037016	329229574633,59	440913352370,98	-0,42140122875986	0,0136069178342953	0,0055570063161362	1,33922765857741	0.421401228759868	0,74669903477221	-0.421401228759868	3734
Q8BK03-2	sp|Q8BK03-2|FA73B_MOUSE Isoform 2 of Protein FAM73B OS=Mus musculus GN=Fam73b;sp|Q8BK03|FA73B_MOUSE Protein FAM73B OS=Mus musculus GN=Fam73b PE=1 SV=1	1,34	0,185630468772095	0,609238393171755	23,1903792338134	23,9424348342287	23,6458420059548	23,9449572067503	23,8772170718654	23,8343072841257	17630308962,159	23623170170,9984	-0,42214482645924	0,0160562903538707	0,0023356170149315	1,33991810476505	0.422144826459247	0,746314268345038	-0.422144826459247	3735
Q9CZH7	sp|Q9CZH7|MXRA7_MOUSE Matrix-remodeling-associated protein 7 OS=Mus musculus GN=Mxra7 PE=1 SV=2	1,34	0,392050490605342	0,753881747531964	22,6225346670564	22,0655043166933	22,3959226638979	22,3326153441959	22,3731430345571	23,2567715611815	5145150971,86498	6895788132,87498	-0,42250203945484	0,012527105452718	0,0230533991688743	1,34024991114604	0.422502039454839	0,746129502925994	-0.422502039454839	3736
Q99K01-3	sp|Q99K01-3|PDXD1_MOUSE Isoform 3 of Pyridoxal-dependent decarboxylase domain-containing protein 1 OS=Mus musculus GN=Pdxdc1;sp|Q99K01|PDXD1_MOUSE Pyridoxal-dependent decarboxylase domain-containing protein 1 OS=Mus musculus GN=Pdxdc1 PE=1 SV=2;sp|Q99K0	1,34	0,0357619452596416	0,275959769531894	25,6452858661328	25,3086497347007	25,1986693862256	25,6281918452435	25,7508401370558	25,6522026352665	105734985357,043	141714202199,145	-0,42253153690335	0,00916697946639176	0,00253136468753	1,34027731427406	0.422531536903353	0,746114247663463	-0.422531536903353	3737
Q4V9Z5-3	sp|Q4V9Z5-3|SE6L2_MOUSE Isoform 3 of Seizure 6-like protein 2 OS=Mus musculus GN=Sez6l2;sp|Q4V9Z5|SE6L2_MOUSE Seizure 6-like protein 2 OS=Mus musculus GN=Sez6l2 PE=2 SV=1;sp|Q4V9Z5-2|SE6L2_MOUSE Isoform 2 of Seizure 6-like protein 2 OS=Mus musculus GN=S	1,34	0,22694338261995	0,652517395617958	23,2567715611815	23,5382575829121	23,277183681821	23,7026990686974	23,2373012347919	24,0114271695296	13931304613,4826	18675452993,2846	-0,4228128704181	0,00671974220166119	0,0164773124877977	1,34053870125061	0.422812870418095	0,745968765442642	-0.422812870418095	3738
Q9Z2W8	sp|Q9Z2W8|GRIA4_MOUSE Glutamate receptor 4 OS=Mus musculus GN=Gria4 PE=1 SV=2	1,34	0,467981275442378	0,796164546189264	22,9159360119671	21,6835797836826	22,3477845269731	22,9717251193296	22,5286124376778	22,3263702104077	4916028245,24127	6590549653,15645	-0,42290558650763	0,0276397263397531	0,0145997472492232	1,34062485493978	0.422905586507629	0,745920826631935	-0.422905586507629	3739
O08579	sp|O08579|EMD_MOUSE Emerin OS=Mus musculus GN=Emd PE=1 SV=1	1,34	0,164425860666957	0,572338176048558	27,2620275033788	27,6006069372341	27,8165098051471	28,1132288221562	27,6547108054017	27,791225622099	931176566333,165	1248613789171,19	-0,42320064606666	0,0101413894242217	0,0084525408604284	1,34089906717482	0.423200646066674	0,745768286726407	-0.423200646066675	3740
Q02780-6	sp|Q02780-6|NFIA_MOUSE Isoform 6 of Nuclear factor 1 A-type OS=Mus musculus GN=Nfia;sp|Q02780-2|NFIA_MOUSE Isoform 1 of Nuclear factor 1 A-type OS=Mus musculus GN=Nfia;sp|Q02780-5|NFIA_MOUSE Isoform 5 of Nuclear factor 1 A-type OS=Mus musculus GN=Nfia;	1,34	0,221741077899236	0,650602942847209	23,4404474775681	23,9801195100244	24,0935577863108	24,2341635524642	24,2886600925541	23,871854383327	22528377896,3812	30213640839,6705	-0,42345660447403	0,0146390742578577	0,0093883556442553	1,34113698636615	0.423456604474033	0,745635986603821	-0.423456604474033	3741
Q9Z2L6	sp|Q9Z2L6|MINP1_MOUSE Multiple inositol polyphosphate phosphatase 1 OS=Mus musculus GN=Minpp1 PE=1 SV=3	1,34	0,135814937460901	0,523758709545479	23,7647352552483	23,7382122559989	23,199871575543	23,7444339745312	23,8514133380406	23,9894755268835	17190218416,1911	23069431808,7316	-0,42439459584526	0,0135246514906395	0,0051483556658998	1,34200923165717	0.42439459584526	0,745151356943466	-0.42439459584526	3742
	sp|Q9DB73|NB5R1_MOUSE NADH-cytochrome b5 reductase 1 OS=Mus musculus GN=Cyb5r1 PE=2 SV=1;sp|Q9DB73-2|NB5R1_MOUSE Isoform 2																			

Q9DB73	[illegible] of NADH-cytochrome b5 reductase 1 OS=Mus musculus GN=Cyb5r1	1,34	0,128548443438776	0,512962462222669	26,7315571743892	27,0716328405969	27,3540715475072	27,2638746068392	27,2868283286901	27,4902079656322	560682439574,366	752728393433,505	−0,42494550626045	0,0115221325040869	0,0045554313082856	1,34252179184518	0.424945506260456	0,744866866276775	-0.424945506260456	3743
Q99N96	sp|Q99N96|RM01_MOUSE 39S ribosomal protein L1, mitochondrial OS=Mus musculus GN=Mrpl1 PE=1 SV=2	1,34	0,228429802412836	0,652923723647267	25,4532890416025	25,9023194601262	26,2847000887215	26,1422103225398	26,2677834736084	26,1149474128652	173614448341,558	233157587478,156	−0,42541836393667	0,0160799411843753	0,0031143339447101	1,34296188885994	0.425418363936677	0,74462276874358	-0.425418363936677	3744
Q8CGU1	sp|Q8CGU1|CACO1_MOUSE Calcium-binding and coiled-coil domain-containing protein 1 OS=Mus musculus GN=Calcoco1 PE=1 SV=2	1,34	0,071791969335696	0,399342829429809	27,612789504722	27,6754662525446	27,1665322140943	27,738528557757	27,8477202619897	27,7534331423157	864078383938,89	1160525443885,42	−0,42554405736585	0,0100969917484381	0,002131402245617	1,34307889823048	0.425544057365854	0,744557897021172	-0.425544057365853	3745
Q8CA71	sp|Q8CA71|SHSA4_MOUSE Protein shisa-4 OS=Mus musculus GN=Shisa4 PE=2 SV=1	1,34	0,153634064743244	0,556984840151426	25,5484527128798	26,1217410182589	26,0769885460403	26,3439130720978	26,262865549841	26,0271456176258	179910874892,113	241783390053,695	−0,42643274389398	0,0123035460753203	0,0062780951489184	1,34390647701916	0.426432743893979	0,744099397614363	-0.42643274389398	3746
Q9CXZ1	sp|Q9CXZ1|NDUS4_MOUSE NADH dehydrogenase [ubiquinone] iron-sulfur protein 4, mitochondrial OS=Mus musculus GN=Ndufs4 PE=1 SV=3	1,34	0,0332115159653163	0,265230857800811	25,549542709295	25,4079277565894	25,530459038085	25,528533155215	25,9259085166726	25,9217837339592	118239315058,575	158984910998,394	−0,42718003082677	0,0030140888548785	0,0088493603755459	1,34460277378665	0.42718003082677	0,743714068939344	-0.427180030826769	3747
Q8K274	sp|Q8K274|KT3K_MOUSE Ketosamine-3-kinase OS=Mus musculus GN=Fn3krp PE=2 SV=2	1,34	0,430728591082413	0,775108167118334	27,2849017576652	28,4991310077434	28,0944458379701	28,0971533682127	28,485463756269	28,1842803916153	1388844007520,06	1867520078370,4	−0,42723918653689	0,0221127613027051	0,0072109968148378	1,34465790848972	0.427239186536905	0,743683574600155	-0.427239186536906	3748
Q91VR2	sp|Q91VR2|ATPG_MOUSE ATP synthase subunit gamma, mitochondrial OS=Mus musculus GN=Atp5c1 PE=1 SV=1	1,35	0,114537778683913	0,484066087007709	30,1327617042981	30,7404493890883	30,5614058829116	30,8405947262776	30,6703686802763	30,8129891084665	17239177285488,2	23187879861754,8	−0,42767999046700	0,010245694277064	0,0029686671556321	1,34506882073045	0.427679990467001	0,743456382742514	-0.427679990467001	3749
P28867	sp|P28867|KPCD_MOUSE Protein kinase C delta type OS=Mus musculus GN=Prkcd PE=1 SV=3;sp|P28867-2|KPCD_MOUSE Isoform 2 of Protein kinase C delta type OS=Mus musculus GN=Prkcd	1,35	0,0570939326779506	0,353736424574922	28,6238583935333	28,3747558938646	28,1061634244686	28,7527330443723	28,612083924889	28,6297384787753	2090154781482,73	2811816455882,58	−0,42789264248867	0,0091267011400637	0,0026728754901635	1,34526709734286	0.427892642488667	0,743346805980146	-0.427892642488666	3750
Q8VBT0	sp|Q8VBT0|TMX1_MOUSE Thioredoxin-related transmembrane protein 1 OS=Mus musculus GN=Tmx1 PE=1 SV=1	1,35	0,18711943382352	0,611912903548239	26,7001686947059	27,275090612165	27,3519448291009	27,5836630656943	27,3497540213979	27,2837384201724	593360540521,058	798274613524,34	−0,42797614333216	0,0131393704005135	0,0057506194965546	1,34534496146868	0.427976143332162	0,743303783520564	-0.427976143332162	3751
P08414	sp|P08414|KCC4_MOUSE Calcium/calmodulin-dependent protein kinase type IV OS=Mus musculus GN=Camk4 PE=1 SV=2	1,35	0,0441694871474102	0,307965133889546	29,3158045711717	29,6534011397899	29,7856451223423	29,9631043655379	29,799152623176	29,8841798123692	7056358758280,2	9498410648313,69	−0,42876221808079	0,0081894438163571	0,0027439394589414	1,34607819325624	0.428762218080794	0,742898893251468	-0.428762218080794	3752
P70452	sp|P70452|STX4_MOUSE Syntaxin-4 OS=Mus musculus GN=Stx4 PE=1 SV=1	1,35	0,55127322213736	0,840187701076407	22,6774724465421	23,0095077948973	21,7051251708901	23,1878192907474	22,1414207633023	22,954648045036	5701728078,1398	7675475117,22849	−0,42885681991124	0,030176196327259	0,0241349008995	1,34616646252492	0.428856819911248	0,742850180745373	-0.428856819911248	3753
Q91XE4	sp|Q91XE4|ACY3_MOUSE N-acyl-aromatic-L-amino acid amidohydrolase (carboxylate-forming) OS=Mus musculus GN=Acy3 PE=1 SV=1	1,35	0,25082046184561 5	0,673538059877323	23,3634269361434	23,7871003649615	24,1071874390443	24,2371377474287	23,766323408059	24,1475198498776	20682868379,7248	27856356052,548	−0,42957027034027	0,0157069541679095	0,0103949832209747	1,34683234168116	0.429570270340273	0,742482912722282	-0.429570270340273	3754
Q8K1L5	sp|Q8K1L5|PP1RB_MOUSE Protein phosphatase 1 regulatory subunit 11 OS=Mus musculus GN=Ppp1r11 PE=2 SV=1;sp|O60927|PP1RB_HUMAN Protein phosphatase 1 regulatory subunit 11 OS=Homo sapiens GN=PPP1R11 PE=1 SV=1	1,35	0,304874374677466	0,7166363473128	23,6595996010165	24,4199170205648	23,8315975050895	24,5934480971174	24,2257383334506	23,985379748144	25715798704,4538	34637014354,5443	−0,42965961491731	0,0166331696750585	0,0126193499062234	1,34691575216543	0.429659614917319	0,742436932965036	-0.429659614917319	3755
P16014	sp|P16014|SCG1_MOUSE Secretogranin-1 OS=Mus musculus GN=Chgb PE=1 SV=2	1,35	0,107298732405301	0,471927273631455	21,9237820258192	22,3477845269731	22,4317248569495	22,7260651701659	22,4525488290568	22,4181695734126	4532005845,38546	6104311677,89089	−0,42967890415979	0,0122460325472609	0,0074878028878479	1,34693376093201	0.429678904159799	0,742427006438721	-0.429678904159799	3756
Q3V1U8	sp|Q3V1U8|ELMD1_MOUSE ELMO domain-containing protein 1 OS=Mus musculus GN=Elmod1 PE=2 SV=2	1,35	0,0290089082804848	0,238968121025627	25,6433260264358	25,9776537154619	25,9874283403252	26,2003292010332	26,2799304014618	26,0218395960174	171778099343,233	231389066098,817	−0,42977458051907	0,00757289766391675	0,0050507622147613	1,34702308957601	0.429774580519081	0,742377771946551	-0.429774580519081	3757
Q8BKR5	sp|Q8BKR5|PPR37_MOUSE Protein phosphatase 1 regulatory subunit 37 OS=Mus musculus GN=Ppp1r37 PE=2 SV=1	1,35	0,00051649357810726	0,014122676813427	25,9468628589073	26,1186864653252	25,9308965147294	26,2857612704694	26,4091064430994	26,1971184130789	195497861883,351	263502504303,984	−0,43066384398690	0,0040047207525993	0,0040485617166907	1,34785363770991	0.430663843986903	0,741920318365624	-0.430663843986903	3758
Q8C8N2	sp|Q8C8N2|SCAI_MOUSE Protein SCAI OS=Mus musculus GN=Scai PE=1 SV=2;sp|Q8C8N2-2|SCAI_MOUSE Isoform 2 of Protein SCAI OS=Mus musculus GN=Scai	1,35	0,0953966503459034	0,452461228756589	28,7125758401729	28,9188433011032	29,1461064414382	29,469468974537	29,1687376261914	29,0359313240441	3650341573425,1	4921884697069,59	−0,43117939316242	0,00749675737513058	0,0076005194214599	1,34833538124253	0.431179393162427	0,741655239424535	-0.431179393162427	3759

Q3UFY7	sp|Q3UFY7|5NT3B_MOUSE 7-methylguanosine phosphate-specific 5-nucleotidase OS=Mus musculus GN=Nt5c3b PE=2 SV=3;sp|Q3UFY7-4|5NT3B_MOUSE Isoform 4 of 7-methylguanosine phosphate-specific 5-nucleotidase OS=Mus musculus GN=Nt5c3b;sp|Q3UFY7-2|5NT3B_MOUSE Is	1,35	0,213457440423696	0,645012843958033	26,6724274176718	27,0983319088859	27,4433741140667	27,3684215764684	27,5261939943834	27,2162114865181	571412849101,063	770477034883,465	−0,431218478025111	0,014265231996348	0,0056630621125651	1,34837191024942	0.431218478025112	0,741635147097524	-0.431218478025112	3760
Q9JHS9	sp|Q9JHS9|CWC15_MOUSE Spliceosome-associated protein CWC15 homolog OS=Mus musculus GN=Cwc15 PE=1 SV=1	1,35	0,271119363508613	0,691573877451378	25,3890942487746	26,2113399560879	26,1961885123238	26,2339091105729	26,238233207829	26,2212886157278	182900398731,981	246627181018,287	−0,43127358907098	0,0181400268198911	0,0003356429078255	1,34842341912928	0.431273589070979	0,741606817127019	-0.43127358907098	3761
Q91YE3	sp|Q91YE3|EGLN1_MOUSE Egl nine homolog 1 OS=Mus musculus GN=Egln1 PE=2 SV=2	1,35	0,598755375422066	0,86182550787914	22,6026322326214	21,9012572974974	23,2126920867356	22,4285357176485	22,4664435194351	23,7193060179096	6353005673,57452	8569098901,37824	−0,431704195643637	0,0290732500115428	0,0321155335303009	1,34882594816838	0.431704195643637	0,741385500003123	-0.431704195643637	3762
Q9Z2G9	sp|Q9Z2G9|HTAI2_MOUSE Oxidoreductase HTATIP2 OS=Mus musculus GN=Htatip2 PE=1 SV=3;sp|Q9Z2G9-2|HTAI2_MOUSE Isoform 2 of Oxidoreductase HTATIP2 OS=Mus musculus GN=Htatip2	1,35	0,10408982251416	0,465873103918229	25,1593816694031	25,5447622122442	25,7650431834166	25,8849767619169	25,769120591376	25,7127945872499	117502920047,269	158491052914,409	−0,431704790678444	0,0120267360582001	0,0034041497268462	1,3488265044873	0.431704790678446	0,741385194221184	-0.431704790678446	3763
Q8BTZ4-2	sp|Q8BTZ4-2|APC5_MOUSE Isoform 2 of Anaphase-promoting complex subunit 5 OS=Mus musculus GN=Anapc5;sp|Q8BTZ4|APC5_MOUSE Anaphase-promoting complex subunit 5 OS=Mus musculus GN=Anapc5 PE=2 SV=1	1,35	0,289166427252639	0,708707566576526	26,0912494688688	25,2157838114411	25,2894343129533	25,8919891587445	25,8385679589886	25,7650431834166	122595464370,935	165438732533,187	−0,432391432922985	0,019018669205304	0,0024674074736619	1,34946862334663	0.432391432922986	0,741032420242596	-0.432391432922986	3764
Q8K1E0-2	sp|Q8K1E0-2|STX5_MOUSE Isoform 2 of Syntaxin-5 OS=Mus musculus GN=Stx5;sp|Q8K1E0|STX5_MOUSE Syntaxin-5 OS=Mus musculus GN=Stx5 PE=1 SV=3	1,35	0,010623444607792	0,127904018765963	24,8936242018883	24,9939508693433	25,2615134752874	25,3815883916897	25,2670557453431	25,4007405161479	75673674966,4773	102158859463,816	−0,43295090947081	0,00759188259138145	0,0028517489098469	1,34999204821322	0.432950909470815	0,74074510388676	-0.432950909470815	3765
P35283	sp|P35283|RAB12_MOUSE Ras-related protein Rab-12 OS=Mus musculus GN=Rab12 PE=1 SV=3	1,35	0,224883876599873	0,652238880554131	27,6226377001941	28,1771173987933	28,4120046915943	28,5243734839213	28,2538132214311	28,3342637623689	1552032684177,53	2095507372531,48	−0,433140657761418	0,0144394175727084	0,0048970936808447	1,35016961555932	0.433140657761413	0,74064768491012	-0.433140657761413	3766
Q99LY9	sp|Q99LY9|NDUS5_MOUSE NADH dehydrogenase [ubiquinone] iron-sulfur protein 5 OS=Mus musculus GN=Ndufs5 PE=1 SV=3	1,35	0,0282852666776077	0,237741997546121	25,5141697482735	25,2995911794629	25,0736508555744	25,5077187593753	25,6844554864885	25,5969041943702	96789458719,2049	130724707576,005	−0,433610004825956	0,00870831643031923	0,0034524293484215	1,35060893309931	0.433610004825961	0,740406771710928	-0.433610004825961	3767
Q9CQZ5	sp|Q9CQZ5|NDUA6_MOUSE NADH dehydrogenase [ubiquinone] 1 alpha subcomplex subunit 6 OS=Mus musculus GN=Ndufa6 PE=1 SV=1	1,35	0,155954587900889	0,560473376470415	26,9947363166119	26,9252720505903	26,6409567083219	26,8777286255423	27,0871885833272	27,4982193752751	459615002624,657	620864601056,429	−0,43385278717268	0,00698035320577788	0,0116244611343135	1,35083623796209	0.43385278717268	0,740282183655827	-0.43385278717268	3768
Q8CDJ3	sp|Q8CDJ3|BAKOR_MOUSE Beclin 1-associated autophagy-related key regulator OS=Mus musculus GN=Atg14 PE=1 SV=1	1,35	0,451694809974328	0,786543907075754	22,6513182672976	22,7662261675712	23,3607684831536	23,3706667882325	23,7193060179096	22,5905470800734	9050667112,29709	12226113623,0902	−0,433869839622569	0,0166094302565174	0,024883320369907	1,35085220474838	0.433869839622574	0,740273433677569	-0.433869839622574	3769
P53612	sp|P53612|PGTB2_MOUSE Geranylgeranyl transferase type-2 subunit beta OS=Mus musculus GN=Rabggtb PE=1 SV=2	1,35	0,202311675790352	0,633742371575159	27,6170252356209	28,2272204810265	28,3695269666755	28,4734319680636	28,3382640165987	28,3063564870261	1553074392054,18	2099424041797,48	−0,434866655416992	0,0142409001357677	0,0031261318311043	1,35178588516978	0.434866655416994	0,739762125770682	-0.434866655416994	3770
O08709	sp|O08709|PRDX6_MOUSE Peroxiredoxin-6 OS=Mus musculus GN=Prdx6 PE=1 SV=3	1,35	0,0674657888343717	0,385471493631646	32,9567183360255	33,3509572701444	33,4715004505582	33,6444146179947	33,4570130838302	33,5825229115311	278301271216720	376265779927433	−0,435104588656486	0,00809521131601376	0,0028450473950157	1,35200884380591	0.435104588656489	0,73964013222354	-0.435104588656489	3771
Q9R226	sp|Q9R226|KHDR3_MOUSE KH domain-containing, RNA-binding, signal transduction-associated protein 3 OS=Mus musculus GN=Khdrbs3 PE=1 SV=1	1,35	0,163074497417181	0,569625464688209	25,506098678661	25,1861283043439	25,8617916340518	25,6433260264358	25,9600094693325	25,8555074920393	120872995300,603	163424072210,143	−0,435128544185919	0,0132451185399135	0,0062496672769634	1,35203129370393	0.435128544185911	0,739627850817323	-0.435128544185911	3772
Q9QXT8	sp|Q9QXT8|CSEN_MOUSE Calsenilin OS=Mus musculus GN=Kcnip3 PE=1 SV=2;sp|Q9QXT8-3|CSEN_MOUSE Isoform 3 of Calsenilin OS=Mus musculus GN=Kcnip3;sp|Q9Y2W7-3|CSEN_HUMAN Isoform 3 of Calsenilin OS=Homo sapiens GN=KCNIP3;sp|Q9Y2W7|CSEN_HUMAN Calsenilin OS=Hom	1,35	0,343028295624125	0,729891123744941	22,7383944477728	23,3576826764757	23,7883009221215	23,750582547653	23,6075161552656	23,4318412203999	13085789169,6111	17696746412,1372	−0,435483209697243	0,0226561807873432	0,0067657249337159	1,35236371171515	0.435483209697236	0,739446046457234	-0.435483209697236	3773
Q9Z2G6	sp|Q9Z2G6|SE1L1_MOUSE Protein sel-1 homolog 1 OS=Mus musculus GN=Sel1l PE=2 SV=2;sp|Q9Z2G6-2|SE1L1_MOUSE Isoform 2 of Protein sel-1 homolog 1 OS=Mus musculus GN=Sel1l	1,35	0,124428268842298	0,504307734532748	27,5334727094064	27,2462324621921	26,9028762084041	27,5544638957705	27,6419884256892	27,3917352883225	667982041852,632	903368029023,332	−0,435504538900337	0,0115954018142297	0,0046131021690911	1,35238370558266	0.435504538900336	0,739435114362874	-0.435504538900336	3774
Q8JZX4	sp|Q8JZX4|SPF45_MOUSE Splicing factor 45 OS=Mus musculus GN=Rbm17 PE=1 SV=1	1,35	0,486412493643661	0,804557984450102	23,9931989064267	24,6878806630467	25,4392340632725	24,8284747330514	24,9676751874106	25,2300086282913	53704960629,8947	72635492465,5051	−0,435619322712732	0,0292714346885368	0,0081527283193210	1,35249130831823	0.435619322712731	0,739376285710452	-0.435619322712731	3775
Q91YN9	sp|Q91YN9|BAG2_MOUSE BAG family molecular chaperone regulator 2 OS=Mus musculus GN=Bag2 PE=1 SV=1	1,35	0,208682633836934	0,639143370435791	23,800646758244	24,4516913640593	24,5565050782037	24,595883751326	24,4846098063473	24,6363202435582	34686430660,3795	46946348581,1677	−0,436641560979391	0,0168731624822897	0,0031972298458541	1,35344997128206	0.436641560979388	0,738852577648473	-0.436641560979388	3776

Q8CHT1-2	sp|Q8CHT1-2|NGEF_MOUSE Isoform 2 of Ephexin-1 OS=Mus musculus GN=Ngef;sp|Q8CHT1|NGEF_MOUSE Ephexin-1 OS=Mus musculus GN=Ngef PE=1 SV=1	1,35	0,0486648484158309	0,323205843530114	30,0859791046874	30,038498203068	29,6401923534164	30,2534697710926	30,3135389013481	30,108137787763	9880226511828,57	13383568245211,7	−0,43784678760257	0,00818213854197308	0,00349408180819755	1,35458111503708	0.437846787602569	0,738235598369924	-0.437846787602569	3777
Q810C1	sp|Q810C1|SLIK1_MOUSE SLIT and NTRK-like protein 1 OS=Mus musculus GN=Slitrk1 PE=2 SV=1	1,35	0,0880475562607518	0,441176105282424	24,7119475537275	24,5775770341929	24,6655484483854	25,1183229982551	25,1396857445748	24,6083461525581	50826871189,4046	68867598220,8085	−0,43823393964996	0,00276851867397195	0,0120531529287486	1,35494467019573	0.438233939649964	0,738037516953033	-0.438233939649964	3778
Q8CI43	sp|Q8CI43|MYL6B_MOUSE Myosin light chain 6B OS=Mus musculus GN=Myl6b PE=2 SV=1	1,36	0,174629644617284	0,590462219359101	23,7565991016167	24,4506102071283	24,1615766174121	24,4487515518704	24,5842883547047	24,2472581965185	29953998009,1297	40589125967,3787	−0,43834469912544	0,0144516442225155	0,00694268349134084	1,35504869683865	0.438344699125441	0,737980858055519	-0.438344699125441	3779
Q91V14-2	sp|Q91V14-2|S12A5_MOUSE Isoform 2 of Solute carrier family 12 member 5 OS=Mus musculus GN=Slc12a5;sp|Q91V14|S12A5_MOUSE Solute carrier family 12 member 5 OS=Mus musculus GN=Slc12a5 PE=1 SV=2	1,36	0,000652537817678644	0,0169249665968192	24,0692182003921	23,9912209416627	24,1228372462825	24,2064354818055	24,4199170205648	24,4689312575944	28157853197,4968	38161560895,3029	−0,43858283743364	0,00275063840550139	0,0057283276627057	1,35527238627323	0.438582837433642	0,737859053374376	-0.438582837433642	3780
O35927-2	sp|O35927-2|CTND2_MOUSE Isoform 2 of Catenin delta-2 OS=Mus musculus GN=Ctnnd2;sp|O35927|CTND2_MOUSE Catenin delta-2 OS=Mus musculus GN=Ctnnd2 PE=1 SV=1	1,36	0,115240992924585	0,485230859818824	23,0737103227927	22,6703028947368	22,8505765759992	23,4148105713821	22,8859967988134	23,2058538104204	8513025714,92787	11537714869,8231	−0,43861362236243	0,00883813859243376	0,0114955006528953	1,35530130604344	0.438613622362437	0,737843308746838	-0.438613622362437	3781
Q91W39	sp|Q91W39|NCOA5_MOUSE Nuclear receptor coactivator 5 OS=Mus musculus GN=Ncoa5 PE=1 SV=1	1,36	0,0354672137502712	0,275189202072617	22,672615926723	23,0200748010908	22,6586817635727	23,2438889303564	22,9576116421202	23,0632449784514	7850089975,02878	10643854464,6693	−0,43923959449408	0,00898649809017261	0,0062701699161148	1,35588948642978	0.439239594494084	0,737523234753537	-0.439239594494084	3782
Q9CXR1	sp|Q9CXR1|DHRS7_MOUSE Dehydrogenase/reductase SDR family member 7 OS=Mus musculus GN=Dhrs7 PE=2 SV=2	1,36	0,12111412030047	0,496600715486755	27,4591263032037	27,7361956183784	28,1148719832427	28,1742570180425	28,0287075649402	28,020951024883	1149174109838,49	1558334184112,09	−0,43940725657655	0,0118538513051643	0,0030760596889815	1,35604706960471	0.439407256576562	0,737437528839983	-0.439407256576562	3783
Q9EQG7	sp|Q9EQG7|ENPP5_MOUSE Ectonucleotide pyrophosphatase/phosphodiesterase family member 5 OS=Mus musculus GN=Enpp5 PE=2 SV=3	1,36	0,0160367355499306	0,170071828647511	23,4589281980348	23,6813632656051	23,3727250633184	23,7401078459667	23,7058662910714	23,9835441013259	16136326026,6737	21901904047,286	−0,44074415800374	0,00677540056334825	0,0063588752389812	1,35730425941331	0.440744158003742	0,736754484534108	-0.440744158003742	3784
Q6PDS3-2	sp|Q6PDS3-2|SARM1_MOUSE Isoform 2 of Sterile alpha and TIR motif-containing protein 1 OS=Mus musculus GN=Sarm1;sp|Q6PDS3|SARM1_MOUSE Sterile alpha and TIR motif-containing protein 1 OS=Mus musculus GN=Sarm1 PE=1 SV=1;sp|Q6PDS3-3|SARM1_MOUSE Isoform 3 of	1,36	0,257521657274084	0,67968190098123	26,3515957296419	27,0626686517635	27,1900125023832	27,3081622510207	27,1365607688038	27,0764572082296	466298719465,258	632993976143,738	−0,44093730258212	0,0168157897012569	0,0044248824326149	1,35748598424127	0.44093730258212	0,736655856199448	-0.44093730258212	3785
Q9CZW5	sp|Q9CZW5|TOM70_MOUSE Mitochondrial import receptor subunit TOM70 OS=Mus musculus GN=Tomm70a PE=1 SV=2	1,36	0,0778133329092461	0,416490629392219	29,4546675630231	30,0338596200803	29,8563790555124	30,1096724551239	30,1189132769238	30,0338596200803	8590133669577,05	11663457553190,9	−0,44124304296184	0,00996392585791059	0,0015510585656467	1,35777369734052	0.441243042961839	0,736499758360843	-0.441243042961839	3786
Q60648	sp|Q60648|SAP3_MOUSE Ganglioside GM2 activator OS=Mus musculus GN=Gm2a PE=1 SV=2	1,36	0,293877808784332	0,711969538636193	26,4297525807184	25,9119492395323	25,5969041943702	25,979047873499	26,3515957296419	26,5257288518079	191764777018,644	260392900994,387	−0,44135236405169	0,0161864927777459	0,0106247777838836	1,3578765873624	0.441352364051691	0,736443951760335	-0.441352364051691	3787
Q91YN5-2	sp|Q91YN5-2|UAP1_MOUSE Isoform AGX1 of UDP-N-acetylhexosamine pyrophosphorylase OS=Mus musculus GN=Uap1;sp|Q91YN5-3|UAP1_MOUSE Isoform 3 of UDP-N-acetylhexosamine pyrophosphorylase OS=Mus musculus GN=Uap1;sp|Q91YN5|UAP1_MOUSE UDP-N-acetylhexosamine pyro	1,36	0,0740429315908186	0,404916706615332	25,7624652702272	26,2484016717649	26,0680641034151	26,4947769969841	26,3399692238663	26,1622102512306	200947667110,123	272885689326,304	−0,44147691015749	0,0094383033234932	0,0063198123410928	1,35799381625445	0.441476910157493	0,736380378195059	-0.441476910157493	3788
Q6ZQI3	sp|Q6ZQI3|MLEC_MOUSE Malectin OS=Mus musculus GN=Mlec PE=2 SV=2	1,36	0,048900755822469	0,323205843530114	27,0989232001278	27,2953748650026	27,5687786150749	27,7219629002009	27,4793096785935	27,680487545215	733450215772,397	996239347772,323	−0,44179334950764	0,00863714380330159	0,0046979126837902	1,35829170998768	0.441793349507643	0,736218878939539	-0.441793349507643	3789
O08914	sp|O08914|FAAH1_MOUSE Fatty-acid amide hydrolase 1 OS=Mus musculus GN=Faah PE=2 SV=1	1,36	0,0050237047714218	0,0767764173652645	28,6310783417307	28,6463208267188	28,3200413611814	28,796752241005	28,8367004893606	28,8830163528639	2463194440531,75	3346121492956,13	−0,44195931223732	0,00645352933171297	0,0014969822830354	1,35844797223307	0.441959312237327	0,736134191695365	-0.441959312237327	3790
Q8BVL9	sp|Q8BVL9|JKIP1_MOUSE Janus kinase and microtubule-interacting protein 1 OS=Mus musculus GN=Jakmip1 PE=1 SV=2	1,36	0,314664618239231	0,722907157640623	25,8243737283388	25,6867261672998	24,8622015383108	25,6734309040907	25,8629458516474	25,7580916626282	113806690044,708	154710707404,538	−0,44298768008296	0,020439597954861	0,0036847375757934	1,35941663309741	0.442987680082967	0,735609654651284	-0.442987680082967	3791
Q9EP89	sp|Q9EP89|LACTB_MOUSE Serine beta-lactamase-like protein LACTB, mitochondrial OS=Mus musculus GN=Lactb PE=1 SV=1	1,36	0,12286971216738	0,499736221799049	26,7355071353139	26,7858549456868	26,475991979372	27,3151166099119	26,8602290837401	26,7434074548887	380893134857,365	517832525031,764	−0,44309929839317	0,00623577851716892	0,011198434084512	1,35952181239937	0.44309929839317	0,735552744266114	-0.44309929839317	3792
O55023	sp|O55023|IMPA1_MOUSE Inositol monophosphatase 1 OS=Mus musculus GN=Impa1 PE=1 SV=1	1,36	0,156889388932746	0,561270396169617	30,577500263056	31,1056636057856	31,039393479464	31,42290936091	30,9481182033933	31,2748215534313	26482867121095	36026757393905,2	−0,44400948568291	0,009309031626311	0,0077824257144331	1,36037979683884	0.444009485682909	0,735088834988386	-0.444009485682909	3793

P50172	sp|P50172|DHI1_MOUSE Corticosteroid 11-beta-dehydrogenase isozyme 1 OS=Mus musculus GN=Hsd11b1 PE=1 SV=3	1,36	0,0405999473000852	0,297230904508205	25,1555905514942	25,4434411168015	25,6547165375052	25,7816672426511	25,6452858661328	25,7508401370558	109360551092,48	148809244130,182	−0,444371732273946	0,00985683023845188	0,0027801301055301	1,36072141776556	0.444371732273946	0,734904284553779	-0.444371732273946	3794
Q8C6G8	sp|Q8C6G8|WDR26_MOUSE WD repeat-containing protein 26 OS=Mus musculus GN=Wdr26 PE=2 SV=3	1,36	0,000972488960196868	0,0231106146090763	26,7839028148677	26,7117428695641	27,0068439722442	27,1995228618436	27,1190534987677	27,1079700259264	450743022381,871	613338074396,607	−0,444377353890569	0,00573286936699968	0,0018409276976293	1,36072671997346	0.44437735389057	0,734901420925654	-0.44437735389057	3795
P0C7L0-2	sp|P0C7L0-2|WIPF3_MOUSE Isoform 2 of WAS/WASL-interacting protein family member 3 OS=Mus musculus GN=Wipf3;sp|P0C7L0|WIPF3_MOUSE WAS/WASL-interacting protein family member 3 OS=Mus musculus GN=Wipf3 PE=1 SV=1	1,36	0,0944009940987771	0,450242157645468	27,8646384205141	28,4815245373331	28,3139767888204	28,5325900295205	28,5146694615578	28,5376790926525	1802231224241,85	2452950891526,52	−0,44473423201701	0,0113036479505797	0,00042366382526634	1,36106336330867	0.444734232017007	0,734719651529707	-0.444734232017007	3796
Q8CI70	sp|Q8CI70|LRC20_MOUSE Leucine-rich repeat-containing protein 20 OS=Mus musculus GN=Lrrc20 PE=2 SV=1	1,36	0,105933411091995	0,469781708234609	24,6220110958197	24,7509761402325	24,2310959464558	24,6249971441151	24,939766410663	24,9641191392659	45214984944,7867	61540473316,6907	−0,444734556369818	0,0110339965879536	0,0076140065470717	1,36106366930874	0.444734556369816	0,734719486346942	-0.444734556369816	3797
O09164	sp|O09164|SODE_MOUSE Extracellular superoxide dismutase [Cu-Zn] OS=Mus musculus GN=Sod3 PE=1 SV=1	1,36	0,0985204823495524	0,461490680479482	24,3480812542672	24,7207953569625	24,8661841819719	25,0980650300174	25,0138713240005	24,7487693428392	50488946314,1465	68738037999,4315	−0,445141104042656	0,0108433301402211	0,0073051172519488	1,36144726752144	0.445141104042656	0,734512473494866	-0.445141104042656	3798
B1AZA5	sp|B1AZA5|TM245_MOUSE Transmembrane protein 245 OS=Mus musculus GN=Tmem245 PE=1 SV=1	1,36	0,393357249054944	0,753881747531964	21,829564652429	22,0365622706126	22,2120966659598	22,7708085059644	21,6547649475765	22,579222249095	3679617081,1103	5011153178,36191	−0,445586997788846	0,00869340285760002	0,0267195900780913	1,36186811505121	0.445586997788846	0,73428549280809	-0.445586997788846	3799
Q3TEA8-3	sp|Q3TEA8-3|HP1B3_MOUSE Isoform 3 of Heterochromatin protein 1-binding protein 3 OS=Mus musculus GN=Hp1bp3;sp|Q3TEA8-2|HP1B3_MOUSE Isoform 2 of Heterochromatin protein 1-binding protein 3 OS=Mus musculus GN=Hp1bp3;sp|Q3TEA8|HP1B3_MOUSE Heterochromatin p	1,36	0,179855117873681	0,598356502628386	26,35933783101	26,4873971485829	25,8385679589886	26,6900416088642	26,3073973295377	26,6147061508255	245959890505,724	334995084794,666	−0,445716858141184	0,0131022650760459	0,0076383909909912	1,36199070550029	0.445716858141183	0,734219401029374	-0.445716858141183	3800
Q9EPC1	sp|Q9EPC1|PARVA_MOUSE Alpha-parvin OS=Mus musculus GN=Parva PE=1 SV=1	1,36	0,223429595201285	0,652238880554131	27,0501953007888	26,2819309123858	26,8191926913355	27,0501953007888	27,2082466545883	26,8202978882426	400951496871,003	546197579549,491	−0,445995196556647	0,0147536483388831	0,0072181693375972	1,36225349901915	0.445995196556648	0,734077762119912	-0.445995196556648	3801
Q9DC07	sp|Q9DC07|LNEBL_MOUSE LIM zinc-binding domain-containing Nebulette OS=Mus musculus GN=Nebl PE=2 SV=1	1,36	0,209952999239351	0,641644645910089	29,4774744514319	30,1905445773157	30,0107694072549	30,4410482207959	30,1305251963861	30,0350880404903	9601394638238,62	13081504599576,7	−0,446212602312568	0,0124060436058461	0,007028098155326	1,36245879817065	0.446212602312571	0,733967149203105	-0.446212602312571	3802
E9Q9D5	sp|E9Q9D5|RBL2A_MOUSE Rab-like protein 2A OS=Mus musculus GN=Rabl2 PE=1 SV=1;sp|E9Q9D5-3|RBL2A_MOUSE Isoform 3 of Rab-like protein 2A OS=Mus musculus GN=Rabl2;sp|E9Q9D5-1|RBL2A_MOUSE Isoform 1 of Rab-like protein 2A OS=Mus musculus GN=Rabl2	1,36	0,366106543632402	0,739315231400715	24,2494398701144	23,6838064405286	24,5823748158982	24,7499941271413	24,0448449514442	24,64930457552	31456570237,5202	42867560786,9244	−0,446524948623554	0,0187936683697601	0,0155787349452923	1,36275380511107	0.446524948623555	0,733808261073608	-0.446524948623555	3803
P54830	sp|P54830|PTN5_MOUSE Tyrosine-protein phosphatase non-receptor type 5 OS=Mus musculus GN=Ptpn5 PE=2 SV=2;sp|P54830-2|PTN5_MOUSE Isoform STEP46 of Tyrosine-protein phosphatase non-receptor type 5 OS=Mus musculus GN=Ptpn5;sp|P54830-3|PTN5_MOUSE Isoform ST	1,36	0,125042837850612	0,505290108611383	25,9104073439626	26,3932386786055	26,4605063694145	26,7734005079762	26,4538794096732	26,4674873848154	252510195734,766	344349316540,929	−0,447531172109988	0,0114292681976636	0,0068012797075415	1,36370460423954	0.447531172109986	0,733296636889805	-0.447531172109986	3804
Q9Z2W9	sp|Q9Z2W9|GRIA3_MOUSE Glutamate receptor 3 OS=Mus musculus GN=Gria3 PE=1 SV=2	1,36	0,158112760087153	0,562881425910264	27,6959168630177	27,1009588259087	27,1000546991755	27,6503089516748	27,7510871215627	27,4280292367288	717455524596,585	979010727136,555	−0,448435266476	0,0125924309031839	0,0059867538726906	1,36455946546239	0.448435266476001	0,732837245507027	-0.448435266476001	3805
Q6P2B1	sp|Q6P2B1|TNPO3_MOUSE Transportin-3 OS=Mus musculus GN=Tnpo3 PE=1 SV=1;sp|Q6P2B1-2|TNPO3_MOUSE Isoform 2 of Transportin-3 OS=Mus musculus GN=Tnpo3	1,36	0,0990277467150906	0,462035382641852	28,8755859989111	28,5978831612972	28,2385123208996	28,8313933089507	28,9598110128047	28,8541446687965	2559058398572,64	3493003196436,68	−0,448854892400579	0,0111795834174815	0,002372605081979	1,36495642240324	0.448854892400581	0,732624121610657	-0.448854892400581	3806
Q8VDG3	sp|Q8VDG3|PARN_MOUSE Poly(A)-specific ribonuclease PARN OS=Mus musculus GN=Parn PE=1 SV=1	1,37	0,0632149560323278	0,375566342186827	23,3453837113846	23,3771965789393	23,1523256748072	23,3098271341832	23,8201140153781	23,6787354581344	13044537773,9452	17807618397,9816	−0,449048758451924	0,00522461215335219	0,0111622841122991	1,36513985444161	0.449048758451923	0,732525679875515	-0.449048758451923	3807
Q8CE96	sp|Q8CE96|TRM6_MOUSE tRNA (adenine(58)-N(1))-methyltransferase non-catalytic subunit TRM6 OS=Mus musculus GN=Trmt6 PE=1 SV=1;sp|Q8CE96-3|TRM6_MOUSE Isoform 3 of tRNA (adenine(58)-N(1))-methyltransferase non-catalytic subunit TRM6 OS=Mus musculus GN=Trmt6	1,37	0,0352852831604691	0,274716810060667	24,4697558524359	24,758319535241	24,2636612879042	24,8771377433071	24,7436143644013	24,8090721097822	43553049402,9424	59541620301,6989	−0,451124748210796	0,0101428647599931	0,0026910959945909	1,36710565891343	0.451124748210798	0,731472358028854	-0.451124748210798	3808

Q3UZ39-2	sp|Q3UZ39-2|LRRF1_MOUSE Isoform 2 of Leucine-rich repeat flightless-interacting protein 1 OS=Mus musculus GN=Lrrfip1;sp|Q3UZ39| LRRF1_MOUSE Leucine-rich repeat flightless-interacting protein 1 OS=Mus musculus GN=Lrrfip1 PE=1 SV=2	1,37	0,0870862378218679	0,440251927134614	22,524642240339	22,4253348908122	22,0655043166933	22,8020772128933	22,4181695734126	22,7337848548684	5029034307,00936	6876281618,70322	−0,45134723651404	0,0108142418974526	0,00904147240035	1,36731650629609	0.451347236514043	0,731359561151565	-0.451347236514043	3809
P97765	sp|P97765|WBP2_MOUSE WW domain-binding protein 2 OS=Mus musculus GN=Wbp2 PE=1 SV=1	1,37	0,0155860830446578	0,168843033268978	29,1607446221874	29,2978797219439	29,5874435150517	29,709391036643	29,6699505699435	29,6053749310012	5571521466812,37	7618283358386,64	−0,45139459782384	0,00742247247312827	0,00177036356288	1,36736139378913	0.451394597823844	0,731335552211893	-0.451394597823845	3810
P43006	sp|P43006|EAA2_MOUSE Excitatory amino acid transporter 2 OS=Mus musculus GN=Slc1a2 PE=1 SV=1;sp|P43006-2|EAA2_MOUSE Isoform Glt-1A<br>of Excitatory amino acid transporter 2 OS=Mus musculus GN=Slc1a2;sp|P43006-3|EAA2_MOUSE Isoform Glt-1B of Excitatory amino	1,37	0,0010466186925494	0,0243630678181958	31,6843541136303	31,7786943855435	31,8078616968354	32,2397875300273	32,0248903492098	31,9453672776117	61926546536269,9	84689695575640	−0,45162845024288	0,00203281680094346	0,00474926181509	1,36758305302941	0.451628450242888	0,731217016608126	-0.451628450242888	3811
Q8K3J1	sp|Q8K3J1|NDUS8_MOUSE NADH dehydrogenase [ubiquinone] iron-sulfur protein 8, mitochondrial OS=Mus musculus GN=Ndufs8 PE=1 SV=1	1,37	0,00043560164502768	0,0126743324793632	26,1926661523628	26,2637346192989	26,325250003904	26,6793021818755	26,6267436086175	26,4159750442456	253987339572,998	347491814025,579	−0,45222240698975	0,00252657545701386	0,00524421589728	1,36814620212874	0.452222406989755	0,730916036929439	-0.452222406989755	3812
Q8K2H2	sp|Q8K2H2|OTU6B_MOUSE OTU domain-containing protein 6B OS=Mus musculus GN=Otud6b PE=2 SV=1	1,37	0,0733417760539362	0,40449370778714	26,0255942843937	25,9331697089281	26,438944362597	26,593052849711	26,4316752166706	26,3143352941035	223475908215,999	305848113174,359	−0,45269606560125	0,0103060202317015	0,00529136864831	1,36859545897334	0.452696065601254	0,730676105523656	-0.452696065601254	3813
O55022	sp|O55022|PGRC1_MOUSE Membrane-associated progesterone receptor component 1 OS=Mus musculus GN=Pgrmc1 PE=1 SV=4	1,37	0,129403179853778	0,512962462222669	30,2028177448941	30,7513155117469	30,7005833571881	30,8577552537318	30,6775354171231	31,0631349310244	18551508169400,1	25409439818140,1	−0,45382809236744	0,00992071818541854	0,00625074922042	1,36966976410316	0.453828092367438	0,730102997239473	-0.453828092367438	3814
Q8BNL5	sp|Q8BNL5|KCTD6_MOUSE BTB/POZ domain-containing protein KCTD6 OS=Mus musculus GN=Kctd6 PE=2 SV=1	1,37	0,622359413834283	0,873497025168154	24,4774642448869	22,5936782818681	23,7908431155132	23,4404474775681	24,547663319805	23,8182850170703	18126882253,3516	24833646181,1005	−0,45416529065410	0,0403610148098484	0,0235145731062886	1,36998993174951	0.4541652906541	0,729932371636468	-0.4541652906541	3815
A2A699	sp|A2A699|F1712_MOUSE Protein FAM171A2 OS=Mus musculus GN=Fam171a2 PE=1 SV=1;sp|A2A699-2|F1712_MOUSE Isoform 2 of Protein<br>FAM171A2 OS=Mus musculus GN=Fam171a2	1,37	0,144117097600876	0,538836495889932	26,8127695771732	26,242967719549	26,2087814532622	26,894872517499	26,5828657246869	26,7315571743892	298341980185,39	408775443994,466	−0,45434153721270	0,0128408592250155	0,0058369982242198	1,37015730652606	0.454341537212705	0,729843205037113	-0.454341537212705	3816
Q3UHX2	sp|Q3UHX2|HAP28_MOUSE 28 kDa heat- and acid-stable phosphoprotein OS=Mus musculus GN=Pdap1 PE=1 SV=1	1,37	0,271216008552302	0,691573877451378	27,2900373208184	28,0023313775896	28,2304457403943	28,2385123208996	28,1061634244686	28,1229950257761	1233575706352,38	1690237651299,88	−0,45437984834077	0,0176193167062329	0,00255882604845	1,37019369187954	0.454379848340774	0,729823824125381	-0.454379848340773	3817
Q9JIX8-3	sp|Q9JIX8-3|ACINU_MOUSE Isoform 3 of Apoptotic chromatin condensation inducer in the nucleus OS=Mus musculus GN=Acin1;sp|Q9JIX8-2| ACINU_MOUSE Isoform 2 of Apoptotic chromatin condensation inducer in the nucleus OS=Mus musculus GN=Acin1;sp|Q9JIX8-4|ACINU	1,37	0,349872766108247	0,732843629012038	24,7472989515349	24,5401576917216	23,6580266549454	24,5775770341929	24,7280852384171	24,5863610385453	36302814128,0334	49769811234,4389	−0,45518952756364	0,0237893901022303	0,00342964544809	1,37096289722637	0.455189527563644	0,729414342301406	-0.455189527563644	3818
P10922	sp|P10922|H10_MOUSE Histone H1.0 OS=Mus musculus GN=H1f0 PE=2 SV=4	1,37	0,455243062800493	0,788787570274876	24,0558013103755	25,369136158521	24,9719414710983	24,7822925530271	25,2737175242021	25,2882777697561	58891325791,4798	80761210219,5322	−0,45560737726977	0,0271599506909314	0,0114681371796735	1,37136002856326	0.455607377269776	0,729203111634858	-0.455607377269776	3819
O70252	sp|O70252|HMOX2_MOUSE Heme oxygenase 2 OS=Mus musculus GN=Hmox2 PE=2 SV=1	1,37	0,0509471487892404	0,332732530006276	28,5325900295205	28,8755859989111	29,0597621446406	29,2338980175545	29,1173946133026	29,0665390787366	3292426864927,58	4518843600900,85	−0,45680223150404	0,0092824100158151	0,00294442944926	1,37249627289754	0.45680223150404	0,728599428462455	-0.45680223150404	3820
Q9D850	sp|Q9D850|TMM68_MOUSE Transmembrane protein 68 OS=Mus musculus GN=Tmem68 PE=2 SV=1	1,37	0,355141172383318	0,732843629012038	23,2674949779094	23,5235637626522	24,2775725239655	23,9139624756217	23,7883009221215	24,3163006006741	19419503923,0733	26653545003,2712	−0,45682108145378	0,0221657436329195	0,0114894170329851	1,37251420576211	0.456821081453783	0,72858990879787	-0.456821081453783	3821
Q64133	sp|Q64133|AOFA_MOUSE Amine oxidase [flavin-containing] A OS=Mus musculus GN=Maoa PE=1 SV=3	1,37	0,118439148521393	0,491463415677796	26,9179630935536	27,5118748975731	27,5217488359946	27,6959168630177	27,6401569056551	27,5668665706527	730646509819,977	1003297756186,12	−0,45750433139638	0,0126579884541	0,00234213482682	1,37316437251349	0.457504331396376	0,728244935578662	-0.457504331396376	3822
Q8K3A0	sp|Q8K3A0|HSC20_MOUSE Iron-sulfur cluster co-chaperone protein HscB, mitochondrial OS=Mus musculus GN=Hscb PE=2 SV=2	1,37	0,140620210129061	0,532098608288336	25,9834974475046	26,5575313626245	26,642274809204	26,6568132883569	26,647166109089	26,8316021926563	290373696724,633	398853704029,67	−0,45794890199215	0,0135784495735876	0,00388634277231	1,37358758223859	0.457948901992154	0,728020559395464	-0.457948901992154	3823
P56213	sp|P56213|ALR_MOUSE FAD-linked sulfhydryl oxidase ALR OS=Mus musculus GN=Gfer PE=2 SV=2	1,37	0,214725636559617	0,646613851007495	24,5493168001552	25,3572525299008	25,2247645740049	25,4703197299504	25,3163007018966	25,2978380035958	75227144586,8274	103360234437,998	−0,45835601159147	0,0173020832313972	0,00373415188881	1,37397524531453	0.45835601159147	0,727815150535029	-0.45835601159147	3824
Q99MS8	sp|Q99MS8|TPGS1_MOUSE Tubulin polyglutamylase complex subunit 1 OS=Mus musculus GN=Tpgs1 PE=1 SV=1	1,37	0,0533027117307887	0,340808374718977	22,3731430345571	22,5875559426552	22,9029360555361	22,9959305482374	22,8225685424939	22,9990398942442	6672174645,75538	9169784860,05247	−0,45873083378738	0,0117807660219833	0,0044029542087822	1,37433226000551	0.458730833787386	0,727626083663341	-0.458730833787386	3825
Q6PEV3	sp|Q6PEV3|WIPF2_MOUSE WAS/WASL-interacting protein family member 2 OS=Mus musculus GN=Wipf2 PE=2 SV=1	1,37	0,216431683724635	0,64735591278721	25,9039797918375	26,7489665802186	26,4656131249857	26,8032741998557	26,5703771561402	26,69891974877	284174171108,601	390563746008,168	−0,45878260513805	0,0163070243150509	0,00437069808267	1,37438157903136	0.458782605138055	0,727599973149218	-0.458782605138055	3826
Q640M6	sp|Q640M6|GDPD5_MOUSE Glycerophosphodiester phosphodiesterase domain-containing protein 5 OS=Mus musculus GN=Gdpd5 PE=2 SV=1	1,37	0,339551506133835	0,729620681454069	22,5476991420516	21,7584100724818	22,8020772128933	22,7144546071874	22,484003771526	22,8640191705498	5186865884,03335	7129397148,6829	−0,45891702301261	0,0243279135562603	0,00843799919144	1,37450963801266	0.458917023012612	0,727532184820365	-0.458917023012612	3827
Q8VIM9	sp|Q8VIM9|IRGQ_MOUSE Immunity-related GTPase family Q protein OS=Mus musculus GN=Irgq PE=2 SV=1	1,37	0,0537963422697314	0,341512723863372	28,3251900002394	28,759343091525	28,8605637488314	28,9711760736878	28,9355947047689	28,9933056026423	2765840307831,97	3802546641572,81	−0,45924808241147	0,00992787560315628	0,0010051395339992	1,37482508690224	0.459248082411473	0,727365255061793	-0.459248082411473	3828

Q9D0B6	sp|Q9D0B6|PBDC1_MOUSE Protein PBDC1 OS=Mus musculus GN=Pbdc1 PE=2 SV=1;sp|Q9D0B6-2|PBDC1_MOUSE Isoform 2 of Protein PBDC1 OS=Mus musculus GN=Pbdc1	1,38	0,102113151186498	0,46388602391647	26,6579118643942	26,9807462280019	27,3015176496588	27,3540715475072	27,1813739338955	27,3601226677854	521543531055,492	717129816606,031	−0,45944662929136	0,0119274553350738	0,0037181105690996	1,37501430638918	0.459446629291369	0,727265160335694	-0.459446629291369	3829
Q8BIG7	sp|Q8BIG7|CMTD1_MOUSE Catechol O-methyltransferase domain-containing protein 1 OS=Mus musculus GN=Comtd1 PE=2 SV=1	1,38	0,252857992613894	0,674733937962647	26,0401650290287	26,3128537120198	26,4356893840667	26,9837240922818	26,1149474128652	26,6455238891739	254585535388,805	350069822633,973	−0,45949224830520	0,00770814085542705	0,0164748228767164	1,37505778597886	0.459492248305206	0,727242164072495	-0.459492248305206	3830
Q6P8I4	sp|Q6P8I4|PCNP_MOUSE PEST proteolytic signal-containing nuclear protein OS=Mus musculus GN=Pcnp PE=1 SV=1	1,38	0,0945320062207823	0,450242157645468	25,9677083343354	26,5608458583866	26,1820526790869	26,7001686947059	26,4253543694505	26,5421786038889	248043248561,848	341256610953,384	−0,46026530539697	0,011447410809745	0,0051935755500563	1,37579479760883	0.460265305396973	0,726852581313746	-0.460265305396973	3831
P40630-2	sp|P40630-2|TFAM_MOUSE Isoform Nuclear of Transcription factor A, mitochondrial OS=Mus musculus GN=Tfam;sp|P40630|TFAM_MOUSE Transcription factor A, mitochondrial OS=Mus musculus GN=Tfam PE=1 SV=2	1,38	0,0901985956602972	0,441176105282424	23,8848298274034	24,3700164161251	24,4023850233445	24,4971412211746	24,7055174223122	24,4132109090808	32977029786,0943	45392974539,3102	−0,46100756692592	0,01197067518512	0,0061333359237977	1,37650282131993	0.46100756692592	0,726478714399654	-0.46100756692592	3832
Q9Z268	sp|Q9Z268|RASL1_MOUSE RasGAP-activating-like protein 1 OS=Mus musculus GN=Rasal1 PE=2 SV=2	1,38	0,0172806551537665	0,179002683778532	30,6271401858965	30,4090370110476	30,1905445773157	30,6639825913764	30,8180134544735	30,7034268677074	16084983616906	22141489215487,6	−0,46103779311971	0,00717874639028863	0,0026038363871802	1,37653166100934	0.461037793119712	0,726463493957526	-0.461037793119712	3833
Q8BL86	sp|Q8BL86|MBLC2_MOUSE Metallo-beta-lactamase domain-containing protein 2 OS=Mus musculus GN=Mblac2 PE=2 SV=2	1,38	0,179799920448571	0,598356502628386	26,5794042509612	27,064290140851	27,30041308278	27,5020965064079	27,1289472953639	27,2725327823275	522227502379,87	719046736542,162	−0,46140710872403	0,0136237416131864	0,0068941398598390	1,37688408455196	0.46140710872403	0,726277550317828	-0.46140710872403	3834
P47915	sp|P47915|RL29_MOUSE 60S ribosomal protein L29 OS=Mus musculus GN=Rpl29 PE=2 SV=2	1,38	0,166113109413086	0,575360434338823	24,3302859701029	25,1110816865738	24,6343822529779	25,0309060428071	24,9321216980073	25,0723990419734	52913092293,0382	72860240370,5724	−0,46150702190841	0,0159390081336934	0,0028811263844589	1,37697944333068	0.461507021908417	0,726227254040316	-0.461507021908416	3835
O35621	sp|O35621|PMM1_MOUSE Phosphomannomutase 1 OS=Mus musculus GN=Pmm1 PE=1 SV=1	1,38	0,123050593484747	0,500023853023517	28,3918361374394	28,7923191655361	29,0984836692739	29,1279023668535	29,0088476558689	29,1075726323578	3095218624133,71	4264904414550,3	−0,46247209337460	0,012321295468478	0,0021898516766513	1,37790086338213	0.462472093374604	0,725741616523445	-0.462472093374604	3836
Q9DCM7	sp|Q9DCM7|NACC2_MOUSE Nucleus accumbens-associated protein 2 OS=Mus musculus GN=Nacc2 PE=2 SV=1	1,38	0,863554249161908	0,967731774645151	22,5764449156736	22,6191277512212	17,0744220795957	21,5496008959617	20,5085414095425	21,1739565399968	1033963089,40678	1424898303,0415	−0,46267427082046	0,15363678429808	0,0250130011300251	1,3780939741853	0.46267427082046	0,725639919143525	-0.46267427082046	3837
Q9JKX6	sp|Q9JKX6|NUDT5_MOUSE ADP-sugar pyrophosphatase OS=Mus musculus GN=Nudt5 PE=1 SV=1	1,38	0,150557834715892	0,551142531890274	26,3810756525348	26,7506588434913	27,102742443713	27,2041523196231	26,9273907888982	27,0652717104084	412221080932,176	568123657956,866	−0,46278669532262	0,0134930307905696	0,0051128015274094	1,37820136872219	0.462786695322622	0,725583374603057	-0.462786695322622	3838
Q9ERR1-2	sp|Q9ERR1-2|NDEL1_MOUSE Isoform 2 of Nuclear distribution protein nudE-like 1 OS=Mus musculus GN=Ndel1;sp|Q9ERR1|NDEL1_MOUSE Nuclear distribution protein nudE-like 1 OS=Mus musculus GN=Ndel1 PE=1 SV=2	1,38	0,214825663652633	0,646613851007495	26,5520528301124	26,0769885460403	26,9056299926328	27,0119621691426	26,6820304486377	26,8032741998557	326454729755,938	449958985644,747	−0,46291056014629	0,015683814462973	0,0062195658393965	1,37831970142121	0.462910560146299	0,725521081189568	-0.462910560146299	3839
Q8R5A6	sp|Q8R5A6|TB22A_MOUSE TBC1 domain family member 22A OS=Mus musculus GN=Tbc1d22a PE=1 SV=3;sp|Q8R5A6-2|TB22A_MOUSE Isoform 2 of TBC1 domain family member 22A OS=Mus musculus GN=Tbc1d22a	1,38	0,424703480114562	0,7711031271846	25,3070616187359	26,4430714013401	25,9585083386998	26,6815515217131	26,0460311816041	25,9437232703096	177614348070,284	244814999426,748	−0,46294382196159	0,02200698151326	0,0152433525861312	1,37835147940791	0.462943821961596	0,725504354251908	-0.462943821961596	3840
Q9CR24	sp|Q9CR24|NUDT8_MOUSE Nucleoside diphosphate-linked moiety X motif 8, mitochondrial OS=Mus musculus GN=Nudt8 PE=1 SV=1	1,38	0,228073885324239	0,652923723647267	22,9507064919501	23,3919312518076	22,5407241994077	23,2244008906573	23,1401154868964	23,4824267429013	9373202412,22408	12923515211,4529	−0,46338459532318	0,0185399955679947	0,0076604419534033	1,37877265880855	0.463384595323177	0,72528273142879	-0.463384595323176	3841
Q61414	sp|Q61414|K1C15_MOUSE Keratin, type I cytoskeletal 15 OS=Mus musculus GN=Krt15 PE=1 SV=2	1,38	0,700268960782153	0,913439194888196	22,3631795486139	24,6992019327018	24,2081894088514	24,9004151407677	23,9469638537138	23,3895979642284	20771692586,2415	28666422285,4683	−0,46474308085719	0,0518450755394344	0,0317294399031644	1,38007156453182	0.464743080857193	0,724600104588956	-0.464743080857193	3842
Q9D0K0	sp|Q9D0K0|TBCD7_MOUSE TBC1 domain family member 7 OS=Mus musculus GN=Tbc1d7 PE=1 SV=1;sp|Q9D0K0-2|TBCD7_MOUSE Isoform 2 of TBC1 domain family member 7 OS=Mus musculus GN=Tbc1d7	1,38	0,219781337133052	0,648792713611515	23,9878581132619	24,2198565302694	23,4130659507513	24,021910159197	24,3929941603799	24,1736455229619	23343712933,6102	32230636486,6619	−0,46539863172801	0,017398107085914	0,0077105465585624	1,38069880221395	0.465398631728022	0,724270925995228	-0.465398631728022	3843
Q91YD9	sp|Q91YD9|WASL_MOUSE Neural Wiskott-Aldrich syndrome protein OS=Mus musculus GN=Wasl PE=1 SV=1	1,38	0,145154006946223	0,540044293056479	28,3382640165987	28,9967840041252	28,8294358832546	29,1565083052916	29,1319348639858	28,8443921491069	2975682780545,95	4109319008975,13	−0,46567859446702	0,0119169543253908	0,0059750873098313	1,38096676024761	0.465678594467022	0,724130390959374	-0.465678594467022	3844
Q9CY52	sp|Q9CY52|THG1_MOUSE Probable tRNA(His) guanylyltransferase OS=Mus musculus GN=Thg1l PE=2 SV=1	1,38	0,245726952705478	0,669981621023675	22,5905470800734	22,9239850676747	23,4741435389005	23,5108751221745	23,1467880451938	23,3004795688856	9708088382,73452	13411533894,617	−0,46621510159027	0,0194034386355394	0,0078380294817284	1,38148040745786	0.466215101590273	0,723861152573388	-0.466215101590273	3845
Q61838	sp|Q61838|A2M_MOUSE Alpha-2-macroglobulin OS=Mus musculus GN=A2m PE=1 SV=3	1,38	0,0808518307460988	0,423283113906046	27,8165098051471	28,2841998398392	28,415569281999	28,5614572625613	28,4428094286615	28,4815245373331	1717847166940,46	2373208001307,32	−0,46623686318560	0,011176714623163	0,0021233487844971	1,38150124584952	0.466236863185597	0,723850233942476	-0.466236863185597	3846
P06797	sp|P06797|CATL1_MOUSE Cathepsin L1 OS=Mus musculus GN=Ctsl PE=1 SV=2	1,38	0,613167287833843	0,868059454773913	23,6996447819213	25,3025335055744	25,3493149492024	25,4252755505049	24,4296234518414	25,4664846831164	58007089854,8735	80146968716,3773	−0,46641871354605	0,0378966505457747	0,0233837075305312	1,38167539376471	0.466418713546051	0,723758999047712	-0.466418713546051	3847

Q9D7I5-2	sp|Q9D7I5-2|LHPP_MOUSE Isoform 2 of Phospholysine phosphohistidine inorganic pyrophosphate phosphatase OS=Mus musculus GN=Lhpp;sp|Q9D7I5|LHPP_MOUSE Phospholysine phosphohistidine inorganic pyrophosphate phosphatase OS=Mus musculus GN=Lhpp PE=2 SV=1	1,38	0,246639001512906	0,67153016550665	25,956784137851	26,5218729579552	26,9102965508651	26,8535256285277	26,7839028148677	26,7226120902923	310976924582,956	429840556440,686	−0,466994078723688	0,0181187474928275	0,0024452804754739	1,38222653342249	0.466994078723687	0,723470412280345	-0.466994078723686	3848
Q9DCU6	sp|Q9DCU6|RM04_MOUSE 39S ribosomal protein L4, mitochondrial OS=Mus musculus GN=Mrpl4 PE=2 SV=1	1,38	0,243901485570234	0,667713415562903	24,0670079530992	24,6402971622954	24,9798946960453	24,9908094544841	24,7304936940114	24,9376594065974	46485205477,7462	64267561344,7708	−0,46731909706296	0,0187848788202458	0,0055268124146872	1,38253796416018	0.467319097062961	0,723307443211839	-0.467319097062961	3849
Q921M7	sp|Q921M7|FA49B_MOUSE Protein FAM49B OS=Mus musculus GN=Fam49b PE=2 SV=1	1,38	0,114518714451456	0,484066087007709	31,3230324235924	31,6995393111186	32,0093513947963	32,099937215965	31,9210716119724	31,9827023770707	57184712227165,6	79060703205379,1	−0,467331279106687	0,0108500118627149	0,0028393232849031	1,38254963828989	0.467331279106686	0,723301335666274	-0.467331279106686	3850
Q8BX70-2	sp|Q8BX70-2|VP13C_MOUSE Isoform 2 of Vacuolar protein sorting-associated protein 13C OS=Mus musculus GN=Vps13c;sp|Q8BX70-3|VP13C_MOUSE Isoform 3 of Vacuolar protein sorting-associated protein 13C OS=Mus musculus GN=Vps13c;sp|Q8BX70|VP13C_MOUSE Vacuolar	1,38	0,156848292864756	0,561270396169617	24,0997473766248	24,801127570362	24,5452995178382	24,9510957123413	24,6071514587832	24,8622015383108	42896589245,0223	59355833114,2009	−0,468526873721688	0,0144980068385477	0,0071970494004051	1,3836958639104	0.468526873721688	0,722702167493615	-0.468526873721688	3851
Q8VBV3	sp|Q8VBV3|EXOS2_MOUSE Exosome complex component RRP4 OS=Mus musculus GN=Exosc2 PE=2 SV=1	1,38	0,0114897859358394	0,136523922415642	24,3563980610013	24,2573141568899	24,5429385120525	24,558208524557	24,6808051491555	24,8919913695741	38950219446,9205	53896696007,9595	−0,468565378642897	0,00594723376531083	0,0068327459033275	1,38373279466133	0.468565378642896	0,722682879135454	-0.468565378642896	3852
Q99PJ0	sp|Q99PJ0|NTRI_MOUSE Neurotrimin OS=Mus musculus GN=Ntm PE=2 SV=2	1,38	0,0257358019547234	0,222504390614265	29,9877542613183	30,3560902036496	30,4410482207959	30,6563201848299	30,5960927494958	30,507269860691	13882232420062,5	19212090910267,7	−0,468774952175611	0,00796249332355914	0,0024514341677942	1,38393381762594	0.468774952175609	0,722577906012473	-0.468774952175609	3853
Q8BGY4-2	sp|Q8BGY4-2|KLH26_MOUSE Isoform 2 of Kelch-like protein 26 OS=Mus musculus GN=Klhl26;sp|Q8BGY4|KLH26_MOUSE Kelch-like protein 26 OS=Mus musculus GN=Klhl26 PE=2 SV=1;sp|Q8BGY4-3|KLH26_MOUSE Isoform 3 of Kelch-like protein 26 OS=Mus musculus GN=Klhl26	1,38	0,16062515461787	0,567767985643373	25,4354333008474	25,9818743454278	25,956784137851	26,4033494281023	25,9086542006991	26,0372835360317	158871879977,62	219897871474,446	−0,468969846547842	0,0119613737275143	0,0098278657667529	1,38412078654462	0.46896984654784	0,722480299205999	-0.46896984654784	3854
P61982	sp|P61982|1433G_MOUSE 14-3-3 protein gamma OS=Mus musculus GN=Ywhag PE=1 SV=2	1,38	0,0878226519298429	0,440959089722961	34,0600793369985	34,6457890411816	34,6005663418216	34,7873562853203	34,698424438377	34,7975818162301	901858039988732	1249001524515810	−0,469802973704485	0,00946379648615505	0,0015689042138779	1,38492031909081	0.469802973704479	0,722063201915104	-0.469802973704479	3855
O55100	sp|O55100|SNG1_MOUSE Synaptogyrin-1 OS=Mus musculus GN=Syngr1 PE=1 SV=2;sp|O55100-2|SNG1_MOUSE Isoform 1B of Synaptogyrin-1 OS=Mus musculus GN=Syngr1	1,39	0,0679945624268039	0,386751026134415	30,5824842469731	31,0545441670067	31,1184365708611	31,3230324235924	31,1310784705769	31,2799527489482	26774962408407,5	37101847458945,7	−0,470606477105423	0,00946800586750271	0,0032235513956862	1,38569185991819	0.470606477105424	0,721661163585851	-0.470606477105424	3856
Q9CZ28	sp|Q9CZ28|SNF8_MOUSE Vacuolar-sorting protein SNF8 OS=Mus musculus GN=Snf8 PE=2 SV=1	1,39	0,110259095052598	0,47572816772219	25,0187798247048	25,3191904935594	25,6844554864885	25,8041604093304	25,6064342261004	25,5908651221833	101244943564,692	140314652081,228	−0,470815809551728	0,013155221817471 4	0,00463264348282 4	1,38589293589335	0.47081580955173	0,721556459450018	-0.47081580955173	3857
Q63912	sp|Q63912|OMGP_MOUSE Oligodendrocyte-myelin glycoprotein OS=Mus musculus GN=Omg PE=1 SV=1	1,39	0,0918552488091242	0,445915480582476	28,0890345798713	28,0738882662073	27,6440499937839	28,3939473383592	28,0072704850482	28,3855524287391	1356131955852,12	1879932054164,63	−0,471182955925985	0,0090441090132084	0,0078148208347496	1,38624567178154	0.471182955925984	0,721372856453971	-0.471182955925984	3858
Q8BXK9	sp|Q8BXK9|CLIC5_MOUSE Chloride intracellular channel protein 5 OS=Mus musculus GN=Clic5 PE=1 SV=1	1,39	0,284031729941347	0,704836107321858	21,4438630713332	22,3236330895835	22,2752567028884	22,2077631703379	22,5521406244179	22,2626581889544	3636367041,23698	5040917744,27039	−0,47118858610162	0,0224655498683818	0,0082819906227599	1,38625108167178	0.471188586101619	0,721370041272772	-0.471188586101619	3859
P62918	sp|P62918|RL8_MOUSE 60S ribosomal protein L8 OS=Mus musculus GN=Rpl8 PE=1 SV=2	1,39	0,0759847201253301	0,41058886267723	25,7758152556667	26,1377623120407	26,341020672108	26,5380610554905	26,2569962681467	26,4430714013401	213065630570,33	295729072047,204	−0,472978184502046	0,010975385407109	0,0054129658577445	1,38797173084933	0.472978184502045	0,720475768903507	-0.472978184502045	3860
Q3TP92	sp|Q3TP92|CNEP1_MOUSE CTD nuclear envelope phosphatase 1 OS=Mus musculus GN=Ctdnep1 PE=2 SV=2	1,39	0,332502122745085	0,72953198881961	22,343926520924	22,8146495088482	23,5022385635319	23,2829667656251	23,2126920867356	23,1493509179357	8703039746,81579	12082249835,4177	−0,473297833704555	0,0254524991174286	0,0028790820672509	1,38827928941015	0.473297833704555	0,720316155133943	-0.473297833704554	3861
Q9DB72	sp|Q9DB72|BTBDH_MOUSE BTB/POZ domain-containing protein 17 OS=Mus musculus GN=Btbd17 PE=2 SV=1	1,39	0,166519621704987	0,575385119523278	28,299685761744	28,9711760736878	28,952674136364	29,2410664809566	28,952674136364	29,0156519772701	3034836468970,36	4215534597682,48	−0,474096820244635	0,0133068990230035	0,0052160308679395	1,38904835261608	0.474096820244636	0,719917343493939	-0.474096820244636	3862
O89051	sp|O89051|ITM2B_MOUSE Integral membrane protein 2B OS=Mus musculus GN=Itm2b PE=2 SV=1	1,39	0,45153648246749	0,786543907075754	24,567454870373	25,0441535538573	25,9945560886311	25,6812465519977	25,6220992401053	25,2894343129533	88127885219,3356	122444867167,769	−0,47446180737445	0,0288284299488945	0,0082730704444627	1,38939981213692	0.474461807374449	0,719735234786007	-0.474461807374449	3863
Q91YX5	sp|Q91YX5|LGAT1_MOUSE Acyl-CoA:lysophosphatidylglycerol acyltransferase 1 OS=Mus musculus GN=Lpgat1 PE=2 SV=1	1,39	0,0893387748542616	0,441176105282424	25,8742929382005	25,839608189605	26,4159750442456	26,4148721289321	26,2691378786882	26,4330141857912	204389241091,177	284028777103,592	−0,474717851680004	0,0124108167141344	0,0034064479372217	1,38964641968061	0.474717851680005	0,719607510110257	-0.474717851680004	3864
Q9Z1E4	sp|Q9Z1E4|GYS1_MOUSE Glycogen [starch] synthase, muscle OS=Mus musculus GN=Gys1 PE=1 SV=2	1,39	8,26×10^−09	8,52×10^−07	25,9187584767025	25,8152971897798	25,8459890590547	26,0949756141312	26,2191523428398	26,2537200098112	170161684707,02	236516226990,925	−0,475032945839498	0,00205481644126728	0,0031876055355458	1,38994996081611	0.475032945839497	0,719450360222214	-0.475032945839497	3865
P21981	sp|P21981|TGM2_MOUSE Protein-glutamine gamma-glutamyltransferase 2 OS=Mus musculus GN=Tgm2 PE=1 SV=4	1,39	0,000632113656932536	0,0165847623631028	27,3684215764684	27,3917352883225	27,1210844296353	27,7082701854407	27,570534038433	27,5924349407401	713713242982,379	992751669059,211	−0,476088339270169	0,0054951755912038	0,0026793722677013	1,39096714096381	0.47608833927017	0,718924243822964	-0.47608833927017	3866
P11531	sp|P11531|DMD_MOUSE Dystrophin OS=Mus musculus GN=Dmd PE=1 SV=3	1,39	0,10049456577899 8	0,46324407107743	25,3737724117129	26,0475252294948	25,8344529325275	26,0548622267635	26,1422103225398	26,0494943656724	152726848354,73	212495993636,653	−0,476481940646487	0,0133728319085589	0,0019955863159856	1,39134668151536	0.47648194064648	0,718728131015391	-0.47648194064648	3867

Q9Z2E1	sp|Q9Z2E1|MBD2_MOUSE Methyl-CpG-binding domain protein 2 OS=Mus musculus GN=Mbd2 PE=2 SV=2;sp|Q9Z2E1-2|MBD2_MOUSE Isoform 2 of Methyl-CpG-binding domain protein 2 OS=Mus musculus GN=Mbd2	1,39	0,659584675289909	0,89341619202324	21,2714023663618	22,9293534018626	23,0354461367347	21,7715837835218	23,2912926988169	23,1651192385014	5412989157,18595	7533798800,16298	−0,47695200658529	0,0441399734976248	0,0370820274321753	1,39180009074313	0.476952006585298	0,718493989654841	-0.476952006585298	3868
Q9ERS2	sp|Q9ERS2|NDUAD_MOUSE NADH dehydrogenase [ubiquinone] 1 alpha subcomplex subunit 13 OS=Mus musculus GN=Ndufa13 PE=1 SV=3	1,39	0,159088057100323	0,565024015006545	26,3833279274518	26,5421786038889	26,1377623120407	26,3000797338373	26,7646133507678	26,9915324948691	278984747215,212	388441542816,382	−0,47751125299280	0,00773121484475182	0,0132081655575771	1,39233971281137	0.477511252992799	0,718215526569179	-0.477511252992799	3869
F8VPU2	sp|F8VPU2|FARP1_MOUSE FERM, RhoGEF and pleckstrin domain-containing protein 1 OS=Mus musculus GN=Farp1 PE=1 SV=1	1,39	0,263870167187813	0,68394025335819	22,9029360555361	23,3634269361434	22,7898514546523	23,8315975050895	23,1051187112	23,1166209487376	9929124547,96701	13843925597,3224	−0,47951466713973	0,0131986841015883	0,0178215089603137	1,39427454358571	0.47951466713974	0,717218860948475	-0.47951466713974	3870
Q9WUN2	sp|Q9WUN2|TBK1_MOUSE Serine/threonine-protein kinase TBK1 OS=Mus musculus GN=Tbk1 PE=1 SV=1	1,39	0,0369110856023221	0,279698526792889	25,9168429770321	25,6889101757867	25,4127860971458	26,1149474128652	25,8919891587445	26,0089907009257	141115764692,499	196771519055,507	−0,47964225133501	0,00983187041573682	0,0042885363114856	1,3943978511847	0.479642251335014	0,717155436771781	-0.479642251335014	3871
Q61024	sp|Q61024|ASNS_MOUSE Asparagine synthetase [glutamine-hydrolyzing] OS=Mus musculus GN=Asns PE=2 SV=3	1,39	0,11272982940993	0,481076973512557	28,0649683586343	28,6488796693929	28,696381711632	28,7755965557594	28,7281279757244	28,9044917431211	2314180117631	3227531624924,83	−0,47993007494666	0,0123511100495183	0,0031684831953643	1,39467606705947	0.479930074946665	0,717012375575064	-0.479930074946665	3872
P06151	sp|P06151|LDHA_MOUSE L-lactate dehydrogenase A chain OS=Mus musculus GN=Ldha PE=1 SV=3	1,40	0,00720074460753804	0,0961299405106329	34,1977817973996	34,2755682575707	34,5639686668958	34,7873562853203	34,6244837935552	34,6244837935552	824479098662667	1150271761991990	−0,48041992551400	0,00561711465590931	0,0027115859191500	1,39514969373726	0.480419925513995	0,716768963566375	-0.480419925513995	3873
Q8VBY2	sp|Q8VBY2|KKCC1_MOUSE Calcium/calmodulin-dependent protein kinase kinase 1 OS=Mus musculus GN=Camkk1 PE=1 SV=1	1,40	0,188789606550622	0,613397297160539	27,8808173603113	28,5948343218379	28,4754028990078	28,9001884639333	28,520313058957	28,5307522510992	1985750796800,96	2771522501503,26	−0,48099413846684	0,0135060742596862	0,0075520922269508	1,39570509349319	0.480994138466842	0,716483736186121	-0.480994138466842	3874
Q76LS9-2	sp|Q76LS9-2|FA63A_MOUSE Isoform 2 of Protein FAM63A OS=Mus musculus GN=Fam63a;sp|Q76LS9|FA63A_MOUSE Protein FAM63A OS=Mus musculus GN=Fam63a PE=1 SV=1;sp|Q76LS9-3|FA63A_MOUSE Isoform 3 of Protein FAM63A OS=Mus musculus GN=Fam63a	1,40	0,623865291595197	0,874528893931624	24,7292894662142	25,4815166293187	24,4506102071283	25,2204546504586	26,2722099307776	24,1698893423587	64320148305,1505	89800044505,8253	−0,48144542698946	0,0214276247377309	0,0416782263813376	1,39614175141189	0.481445426989463	0,716259648412291	-0.481445426989463	3875
Q91VD9	sp|Q91VD9|NDUS1_MOUSE NADH-ubiquinone oxidoreductase 75 kDa subunit, mitochondrial OS=Mus musculus GN=Ndufs1 PE=1 SV=2	1,40	0,122415029521427	0,499228947886575	29,5664435234524	29,323260678621	28,8489065316386	29,6815604610594	29,5382706477389	29,5228299333287	5028805405531,85	7027745083523,72	−0,48284613358442	0,012477590699957	0,0029589010120574	1,397497917854	0.482846133584429	0,715564572386339	-0.482846133584429	3876
O88998	sp|O88998|NOE1_MOUSE Noelin OS=Mus musculus GN=Olfm1 PE=1 SV=1;sp|O88998-3|NOE1_MOUSE Isoform 3 of Noelin OS=Mus musculus GN=Olfm1	1,40	0,0132167992667406	0,14987902972529	26,513257270011	26,596578656695	26,3554364395044	26,7334809843027	27,0515185236898	26,6843310444171	318989531337,891	445788376468,597	−0,48284992199782	0,00462368404609978	0,0074312089821057	1,39750158758782	0.48284992199782	0,715562693367717	-0.48284992199782	3877
Q9CYU6	sp|Q9CYU6|DPH7_MOUSE Diphthamide biosynthesis protein 7 OS=Mus musculus GN=Dph7 PE=2 SV=1	1,40	0,45537890236683	0,788787570274876	21,6228591796111	22,9454843610105	22,8619222612621	22,9604916551048	22,9337468186366	22,5407241994077	5774718036,38879	8071895907,26972	−0,48315706459048	0,0329529354491637	0,0103023235719101	1,39779914039188	0.483157064590487	0,715410369847306	-0.483157064590487	3878
Q8QZV4	sp|Q8QZV4|ST32C_MOUSE Serine/threonine-protein kinase 32C OS=Mus musculus GN=Stk32c PE=2 SV=1	1,40	0,175877329107924	0,592216021380467	27,6317151295644	28,3794989601019	28,3097572274689	28,5719407915324	28,3974533755861	28,3564695698073	1609573638498,09	2250007305380,25	−0,48325110355295	0,0146965213016454	0,0040229753285208	1,39789025588152	0.483251103552958	0,715363738886205	-0.483251103552958	3879
Q9CXJ4	sp|Q9CXJ4|ABCB8_MOUSE ATP-binding cassette sub-family B member 8, mitochondrial OS=Mus musculus GN=Abcb8 PE=2 SV=1	1,40	0,0101318782159626	0,124583502622858	29,0412682162963	29,465248371337	29,2839283038169	29,6230833170184	29,6593636472282	29,5137526899067	5116448339012,45	7154289465894,16	−0,48366580283406	0,00726941290424745	0,0025608520389157	1,39829213388971	0.483665802834063	0,715158138820566	-0.483665802834063	3880
O08576-2	sp|O08576-2|RUN3A_MOUSE Isoform 2 of RUN domain-containing protein 3A OS=Mus musculus GN=Rundc3a;sp|O08576-3|RUN3A_MOUSE Isoform 3 of RUN domain-containing protein 3A OS=Mus musculus GN=Rundc3a;sp|O08576-4|RUN3A_MOUSE Isoform 4 of RUN domain-containing	1,40	0,393229263088713	0,753881747531964	25,1944575563232	25,9613397477305	26,3183357187085	26,4937458641267	26,2158504682045	25,7705950123853	164259118308,714	229705474948,83	−0,48381178397622	0,0222368933059565	0,0139444938706832	1,39843362921938	0.483811783976221	0,715085778191856	-0.483811783976221	3881
Q923D2	sp|Q923D2|BLVRB_MOUSE Flavin reductase (NADPH) OS=Mus musculus GN=Blvrb PE=2 SV=3	1,40	0,0306804049844508	0,249959154423884	28,2346990589835	28,7164765416841	28,6735464306224	28,8343162844237	28,9104405271909	28,8887694965435	2485696481442,58	3479264729342,09	−0,48513230915502	0,00934170415927015	0,0013581667860601	1,39971422710583	0.485132309155028	0,71443154655053	-0.485132309155028	3882
Q8CAA7	sp|Q8CAA7|PGM2L_MOUSE Glucose 1,6-bisphosphate synthase OS=Mus musculus GN=Pgm2l1 PE=1 SV=1	1,40	0,0713951012035168	0,398379368577534	31,6098246071065	32,1814288440177	32,1512238864603	32,3520069298941	32,3027531536818	32,2966368508442	77463330013433,7	108430693093358	−0,48518776633680	0,0100576261172738	0,0009393388559217	1,39976803313973	0.485187766336798	0,714404084337434	-0.485187766336798	3883
Q9JM13	sp|Q9JM13|RABX5_MOUSE Rab5 GDP/GTP exchange factor OS=Mus musculus GN=Rabgef1 PE=1 SV=1	1,40	0,131376318103909	0,515845249025643	26,8149454374718	27,5185848493879	26,9868342577459	27,570534038433	27,3283963991727	27,4312593139902	592009300218,29	828925883901,949	−0,48562327276318	0,0135330021027516	0,0044281782930011	1,4001906449718	0.485623272763187	0,714188459686604	-0.485623272763187	3884
Q9QUP5	sp|Q9QUP5|HPLN1_MOUSE Hyaluronan and proteoglycan link protein 1 OS=Mus musculus GN=Hapln1 PE=2 SV=1	1,40	0,0380382429114503	0,284440831260416	31,0273688047328	30,4899709322161	30,6783315069509	31,1238583488395	31,090420390194	30,9917314842313	22217256321445,7	31113722459010,5	−0,48587034504890	0,00887263825589102	0,0022111123194790	1,40043045859705	0.485870345048904	0,714066160058955	-0.485870345048904	3885

Q9CPV4	sp|Q9CPV4|GLOD4_MOUSE Glyoxalase domain-containing protein 4 OS=Mus musculus GN=Glod4 PE=1 SV=1;sp|Q9CPV4-3|GLOD4_MOUSE Isoform 3 of Glyoxalase domain-containing protein 4 OS=Mus musculus GN=Glod4;sp|Q9CPV4-2|GLOD4_MOUSE Isoform 2 of Glyoxalase domain-c	1,40	0,0671807427140883	0,384531388624523	30,2860766360239	30,6402261423119	30,8796473041202	31,0680797664268	30,8636183629237	30,8856809268552	19510684622888,2	27333286252907,1	−0,48639452154677	0,00975843796045399	0,0036271365828379	1,4009393714889	0.486394521546773	0,713806764483473	-0.486394521546773	3886
Q9Z127	sp|Q9Z127|LAT1_MOUSE Large neutral amino acids transporter small subunit 1 OS=Mus musculus GN=Slc7a5 PE=1 SV=2	1,40	0,284269222156336	0,704836107321858	25,1516291000811	25,5297811363375	25,8966554254401	26,1869977779283	25,4159343606792	25,9896298932137	121845768255,716	170873156601,883	−0,48786962731493	0,0145940244958224	0,0154864848240261	1,40237251607519	0.487869627314932	0,713077294753817	-0.487869627314932	3887
Q8C6B2-2	sp|Q8C6B2-2|RTKN_MOUSE Isoform 2 of Rhotekin OS=Mus musculus GN=Rtkn;sp|Q8C6B2|RTKN_MOUSE Rhotekin OS=Mus musculus GN=Rtkn PE=1 SV=3	1,40	0,330734395929628	0,72953198881961	22,7520392588109	23,7308311655272	23,7805294570105	23,6813632656051	23,6506356046121	23,9460675522639	14848033865,2473	20823655772,165	−0,48795146234928	0,0247633192294821	0,0068362825494329	1,40245206612197	0.487951462349284	0,713036847501805	-0.487951462349284	3888
Q9CQ62	sp|Q9CQ62|DECR_MOUSE 2,4-dienoyl-CoA reductase, mitochondrial OS=Mus musculus GN=Decr1 PE=1 SV=1	1,40	0,0452378028092268	0,312598733858675	29,1870270397349	29,3941367297357	29,6508210992447	29,9092926391212	29,5576191780415	29,7799328650923	5927725059705,99	8313885855045,39	−0,48804440673039	0,00789979488288094	0,0059791156756880	1,40254242079469	0.488044406730396	0,712990912198857	-0.488044406730396	3889
O54724	sp|O54724|PTRF_MOUSE Polymerase I and transcript release factor OS=Mus musculus GN=Ptrf PE=1 SV=1	1,40	0,144532027014929	0,539068536314443	26,1456344965372	26,6783050470106	26,7618369074011	27,072984791289	26,9028762084041	26,6277756439543	332063532436,621	466199215697,359	−0,48948728410816	0,0126003887312515	0,0083615986766420	1,40394584216001	0.489487284108161	0,712278187641108	-0.489487284108161	3890
Q9JKC6	sp|Q9JKC6|CEND_MOUSE Cell cycle exit and neuronal differentiation protein 1 OS=Mus musculus GN=Cend1 PE=1 SV=1	1,40	0,335856712860588	0,72953198881961	30,1038443023282	29,8880330606952	28,9822197969154	29,9571855137959	29,8851245258515	30,1496750736804	7591363888492	10657962391247,5	−0,48950063417833	0,0200665690766066	0,0045593209606565	1,40395883372213	0.489500634178343	0,71227159656016	-0.489500634178342	3891
Q63918	sp|Q63918|SDPR_MOUSE Serum deprivation-response protein OS=Mus musculus GN=Sdpr PE=1 SV=3	1,40	0,249458592799252	0,673464050610029	22,8687955606518	22,0882083383777	22,1710415493333	22,5083970764616	23,0297539046255	22,6079015202486	5221315139,0433	7330802539,17402	−0,48955790897135	0,019161828364293	0,0121851159148768	1,40401457179948	0.489557908971357	0,712243320037863	-0.489557908971357	3892
Q9Z1R4	sp|Q9Z1R4|CF047_MOUSE Uncharacterized protein C6orf47 homolog OS=Mus musculus GN=D17h6s53e PE=2 SV=1	1,40	0,0446400744327706	0,310768861733659	23,2829667656251	23,6723843197498	23,8201140153781	24,0390384667258	23,8680352044495	23,8870579935421	17611566317,08	24732332225,4491	−0,48987506671688	0,011762038552325	0,0039162501276764	1,40432326007615	0.48987506671689	0,71208675981467	-0.48987506671689	3893
Q8BTV1	sp|Q8BTV1|TUSC3_MOUSE Tumor suppressor candidate 3 OS=Mus musculus GN=Tusc3 PE=2 SV=1	1,40	0,0874336017190966	0,440251927134614	25,5584985256053	25,9437232703096	26,0654090296045	26,4297525807184	25,9923002404933	26,1649342395901	169459016138,955	238029952514,507	−0,49020672851371	0,0102346833848677	0,0084112802520220	1,40464613767924	0.490206728513715	0,711923076691901	-0.490206728513715	3894
Q8BMS4	sp|Q8BMS4|COQ3_MOUSE Hexaprenyldihydroxybenzoate methyltransferase, mitochondrial OS=Mus musculus GN=Coq3 PE=1 SV=1	1,41	0,104277087914904	0,465873103918229	25,7725191212397	25,4651569064471	26,1606183781872	26,2409001964166	26,1388382616424	26,0393274349537	160158769353,119	225072549861,127	−0,49088732079195	0,0135084733761634	0,0038557869869977	1,40530893668948	0.490887320791957	0,711587305746255	-0.490887320791957	3895
Q8BIL5	sp|Q8BIL5|HOOK1_MOUSE Protein Hook homolog 1 OS=Mus musculus GN=Hook1 PE=1 SV=2;sp|Q8BIL5-2|HOOK1_MOUSE Isoform 2 of Protein Hook homolog 1 OS=Mus musculus GN=Hook1	1,41	0,297104137927602	0,711969538636193	25,3445401043115	25,6433260264358	24,9510957123413	25,3944926619741	26,1684627487745	25,3969460609751	98466997776,5505	138384307775,216	−0,49096817975962	0,0137159689551532	0,017391340707837	1,40538770247925	0.49096817975962	0,711547424412419	-0.490968179759621	3896
Q9CWD3	sp|Q9CWD3|NUD17_MOUSE Nucleoside diphosphate-linked moiety X motif 17 OS=Mus musculus GN=Nudt17 PE=2 SV=1;sp|Q9CWD3-2|NUD17_MOUSE Isoform 2 of Nucleoside diphosphate-linked moiety X motif 17 OS=Mus musculus GN=Nudt17	1,41	0,0533331502281378	0,340808374718977	22,7197042232794	23,0310308799512	23,0672620680928	23,47931822413	23,0151791532857	23,3482940068699	9171185457,08435	12905642572,8725	−0,49282184299074	0,00832911414908944	0,0102783648273511	1,40719459150217	0.492821842990742	0,71063377164668	-0.492821842990742	3897
Q99L43	sp|Q99L43|CDS2_MOUSE Phosphatidate cytidylyltransferase 2 OS=Mus musculus GN=Cds2 PE=1 SV=1	1,41	0,00161244249006614	0,0329679119928388	27,8519734871773	27,5493288208279	27,6642808277829	27,9254032200445	28,1431261777461	28,0224070708488	1059192257064,7	1490767428852,97	−0,49309072282117	0,00551753139708311	0,0038913776349179	1,40745687943781	0.493090722821175	0,710501340829311	-0.493090722821175	3898
Q99J85	sp|Q99J85|NPTXR_MOUSE Neuronal pentraxin receptor OS=Mus musculus GN=Nptxr PE=1 SV=1	1,41	0,0535136542383152	0,340882645254604	27,6943403199442	27,9960381244174	28,2599624963169	28,1896196889447	28,381044130583	28,4053017035069	1422514192750,6	2002308420222,87	−0,49322116625962	0,0101138800397351	0,0041710286135204	1,40758414251823	0.493221166259627	0,710437102687839	-0.493221166259627	3899
Q6PDY0	sp|Q6PDY0|CC85B_MOUSE Coiled-coil domain-containing protein 85B OS=Mus musculus GN=Ccdc85b PE=1 SV=1	1,41	0,526802867763084	0,82805770353786	22,0223540321112	23,508629348876	23,3886660809348	23,7193060179096	23,3797067172341	22,8462665924133	9487267631,57216	13354150991,9084	−0,49322370697974	0,0359397946496979	0,0188758258128821	1,40758662140697	0.493223706979742	0,710435851543144	-0.493223706979742	3900
Q91YT7	sp|Q91YT7|YTHD2_MOUSE YTH domain-containing family protein 2 OS=Mus musculus GN=Ythdf2 PE=2 SV=1;sp|Q9Y5A9-2|YTHD2_HUMAN Isoform 2 of YTH domain-containing family protein 2 OS=Homo sapiens GN=YTHDF2;sp|Q9Y5A9|YTHD2_HUMAN YTH domain-containing family pro	1,41	0,181939900630495	0,601053022412213	24,573149001652	25,404963452821	25,003648975935	25,5041690285268	25,3025335055744	25,2008916720812	71568471861,2437	100745637379,766	−0,49332128613034	0,0166437495102107	0,0060926139613129	1,40768182915922	0.493321286130356	0,710387801622244	-0.493321286130356	3901
P62259	sp|P62259|1433E_MOUSE 14-3-3 protein epsilon OS=Mus musculus GN=Ywhae PE=1 SV=1	1,41	0,111405885662558	0,477950203234738	35,2475710661237	35,8547745671773	35,8547745671773	36,1142307965785	35,8547745671773	36,0203651980554	3045292651006820	4295983253450640	−0,49640749241696	0,00983298084678394	0,0036495272865011	1,41069635853563	0.496407492416963	0,70886976772099	-0.496407492416963	3902

P23506	sp|P23506|PIMT_MOUSE Protein-L-isoaspartate(D-aspartate) O-methyltransferase OS=Mus musculus GN=Pcmt1 PE=1 SV=3;sp|P23506-2|PIMT_MOUSE Isoform 2 of Protein-L-isoaspartate(D-aspartate) O-methyltransferase OS=Mus musculus GN=Pcmt1	1,41	0,0946778673547673	0,450464192791707	30,6477397566522	30,9123179140515	31,1921742262174	31,4753765257957	31,0176093622394	31,2915687270389	26746122682254,4	37731567907250,5	−0,496442288692057	0,00880581379177412	0,0073687512066618	1,41073038344675	0.496442288692057	0,708852670739794	-0.496442288692057	3903
Q80TL4	sp|Q80TL4|K1045_MOUSE Protein KIAA1045 OS=Mus musculus GN=Kiaa1045 PE=2 SV=2	1,41	8,27×10^−05	0,00323598342950993	31,2915687270389	31,0706333494719	31,0545441670067	31,5568375533821	31,486087748298	31,4086456757793	33377909821313,6	47126518900444,3	−0,497645503949055	0,00425339736708733	0,0023542579132186	1,41190743077481	0.497645503949052	0,708261730339665	-0.497645503949052	3904
Q8JZL3	sp|Q8JZL3|THTPA_MOUSE Thiamine-triphosphatase OS=Mus musculus GN=Thtpa PE=1 SV=3	1,41	0,0628465431173693	0,375342709486499	26,1491158481579	26,5548176288872	26,7655997645261	26,8888307780833	26,7516578076357	26,8660517354712	319442911439,159	451352036363,171	−0,498694990377709	0,0118285206892265	0,0027392365618597	1,41293489446904	0.498694990377711	0,707746693718528	-0.498694990377711	3905
Q8CC21	sp|Q8CC21|TTC19_MOUSE Tetratricopeptide repeat protein 19, mitochondrial OS=Mus musculus GN=Ttc19 PE=1 SV=1;sp|Q8CC21-2|TTC19_MOUSE Isoform 2 of Tetratricopeptide repeat protein 19, mitochondrial OS=Mus musculus GN=Ttc19	1,41	0,221430205789588	0,650415221831493	26,4605063694145	26,8505074822295	27,3802624477312	27,4129341317333	27,2381962259367	27,0796340180657	480019276679,882	678797118458,549	−0,499888097609538	0,0171633513367763	0,0061194385648610	1,41410387339763	0.499888097609537	0,707161629928449	-0.499888097609537	3906
Q9Z2D8-2	sp|Q9Z2D8-2|MBD3_MOUSE Isoform 2 of Methyl-CpG-binding domain protein 3 OS=Mus musculus GN=Mbd3;sp|Q9Z2D8|MBD3_MOUSE Methyl-CpG-binding domain protein 3 OS=Mus musculus GN=Mbd3 PE=1 SV=1	1,41	0,118453619024346	0,491463415677796	25,0817452967474	25,7001815061086	25,7816672426511	25,8263359153978	25,8804365348968	25,8966554254401	121259415234,645	171493172439,507	−0,500054370072649	0,0149974103006684	0,0014234334746687	1,41426686008387	0.500054370072649	0,707080133335445	-0.500054370072649	3907
Q8C0I1	sp|Q8C0I1|ADAS_MOUSE Alkyldihydroxyacetonephosphate synthase, peroxisomal OS=Mus musculus GN=Agps PE=1 SV=1	1,42	0,194061158968554	0,621726099655746	24,8983311212619	25,7318503388321	24,9733867043026	25,5940945146071	25,5091258895633	25,5419778283143	88051647980,6676	124603110992,882	−0,5009181778905	0,0182965794310664	0,0016770817453779	1,41511390019911	0.500918177890505	0,706656898684477	-0.500918177890505	3908
Q9DC71	sp|Q9DC71|RT15_MOUSE 28S ribosomal protein S15, mitochondrial OS=Mus musculus GN=Mrps15 PE=2 SV=2	1,42	0,217276659338549	0,647550070083832	25,0665939202135	25,8188439996991	25,9613397477305	26,0592651454691	25,9154627291353	25,9147147066523	133263279808,859	188647782192,488	−0,501415833393144	0,0187680818082633	0,0032061266618316	1,41560212582992	0.501415833393143	0,706413180478756	-0.501415833393143	3909
Q9R0P4	sp|Q9R0P4|SMAP_MOUSE Small acidic protein OS=Mus musculus GN=Smap PE=1 SV=1	1,42	0,48199141708825	0,802303567138125	23,9139624756217	24,9067459177116	25,3070616187359	24,7729139695752	25,6042732888588	24,7970047172291	53838608168,2139	76309554628,6507	−0,503222592518147	0,029027166773093	0,0188835205046285	1,41737606570787	0.503222592518147	0,705529057667963	-0.503222592518147	3910
Q8BPB0	sp|Q8BPB0|MOB1B_MOUSE MOB kinase activator 1B OS=Mus musculus GN=Mob1b PE=1 SV=3;sp|Q921Y0|MOB1A_MOUSE MOB kinase activator 1A OS=Mus musculus GN=Mob1a PE=2 SV=3;sp|Q921Y0-2|MOB1A_MOUSE Isoform 2 of MOB kinase activator 1A OS=Mus musculus GN=Mob1a	1,42	0,129100598157861	0,512962462222669	24,8955478776018	25,482818253974	25,4079277565894	25,3363157103993	25,7538649352021	25,7441964423723	93581444917,9179	132713569406,739	−0,504021478267553	0,0126530491245272	0,0093056056330963	1,41816114853906	0.50402147826755	0,705138482343961	-0.50402147826755	3911
Q8K2D3	sp|Q8K2D3|EDC3_MOUSE Enhancer of mRNA-decapping protein 3 OS=Mus musculus GN=Edc3 PE=1 SV=1	1,42	0,112336451411531	0,480127262671319	25,2798116120726	25,6844554864885	25,6116604995013	26,2285373005935	25,7758152556667	25,6220992401053	121758961183,95	172814384264,245	−0,505195350408622	0,00845044418263722	0,0121839355356985	1,41931552785804	0.505195350408626	0,704564968375388	-0.505195350408626	3912
Q8JZN7	sp|Q8JZN7|MIRO2_MOUSE Mitochondrial Rho GTPase 2 OS=Mus musculus GN=Rhot2 PE=2 SV=1;sp|Q5E9M9|MIRO2_BOVIN Mitochondrial Rho GTPase 2 OS=Bos taurus GN=RHOT2 PE=2 SV=1;sp|Q7TSA0|MIRO2_RAT Mitochondrial Rho GTPase 2 OS=Rattus norvegicus GN=Rhot2 PE=2 SV=1;	1,42	0,237513077684577	0,661799791043766	24,6730051571631	24,5842883547047	23,8277112897959	24,5323306407483	24,6467589088478	24,9575896134171	38031030943,9407	53998731239,5642	−0,505748461916273	0,0190686042033105	0,0089048475799749	1,41985978027155	0.505748461916273	0,704294898619319	-0.505748461916273	3913
Q9D338	sp|Q9D338|RM19_MOUSE 39S ribosomal protein L19, mitochondrial OS=Mus musculus GN=Mrpl19 PE=2 SV=1	1,42	0,0134716959272341	0,150982784725224	24,6092541358523	24,8485211097813	25,0061922723973	25,2247645740049	25,0100063220039	25,2820160355893	60223140314,6165	85541058203,7102	−0,506299115635021	0,00805202613757691	0,0056969026911144	1,42040182157271	0.506299115635019	0,704026131769369	-0.506299115635019	3914
Q9QVP9	sp|Q9QVP9|FAK2_MOUSE Protein-tyrosine kinase 2-beta OS=Mus musculus GN=Ptk2b PE=1 SV=2	1,42	0,648333131879941	0,889714023663571	24,7462638463834	24,4408004316673	22,5971910117934	23,5434255149496	24,4989816470079	24,7957915615014	24651044175,8917	35027509467,1161	−0,506839655017859	0,0485899074101197	0,0269537727625117	1,42093410799095	0.50683965501786	0,70376240135012	-0.506839655017861	3915
P60853	sp|P60853|LZTS1_MOUSE Leucine zipper putative tumor suppressor 1 OS=Mus musculus GN=Lzts1 PE=2 SV=3	1,42	0,095769915050383	0,452812129597592	25,0392995070299	24,5452995178382	24,4132109090808	25,1986693862256	24,9274050418198	24,9261116536089	51556114050,283	73268408258,2766	−0,507047746508713	0,01337949425761	0,0062752033536596	1,4211390755094	0.507047746508714	0,703660899368031	-0.507047746508714	3916

Q60770	sp|Q60770|STXB3_MOUSE Syntaxin-binding protein 3 OS=Mus musculus GN=Stxbp3 PE=1 SV=1;sp|Q60770-2|STXB3_MOUSE Isoform 2 of Syntaxin-binding protein 3 OS=Mus musculus GN=Stxbp3	1,42	2,04×10^−07	1,72×10^−05	24,3139660285988	24,4391865491359	24,4263753482029	24,8473603223002	24,6602962520587	24,7267151368333	39248378778,3884	55786099946,3284	−0,507272632633089	0,00282440677261201	0,0038324397593856	1,42136061877405	0.507272632633089	0,703551221830333	-0.507272632633089	3917
P10605	sp|P10605|CATB_MOUSE Cathepsin B OS=Mus musculus GN=Ctsb PE=1 SV=2	1,42	0,182405544960901	0,601764572209063	29,4120521281494	30,1201555119806	30,2119986671099	30,3249842800509	30,1150222657986	30,3606214007766	9813061651286,19	13955237227134,4	−0,508031420076723	0,014633180654652	0,0043846887401735	1,42210838197529	0.508031420076721	0,7031812853891	-0.508031420076721	3918
Q62210	sp|Q62210|BIRC2_MOUSE Baculoviral IAP repeat-containing protein 2 OS=Mus musculus GN=Birc2 PE=1 SV=1	1,42	0,0809983202195954	0,423561492484728	22,2607396283641	22,1585650899638	21,7460236150581	22,242612719966	22,3236330895835	22,6586817635727	3788020577,19824	5392684722,24371	−0,50955952283239	0,0123557627600489	0,0098437061197793	1,42361547735634	0.509559522832391	0,702436870001585	-0.509559522832391	3919
A2ASS6-2	sp|A2ASS6-2|TITIN_MOUSE Isoform 2 of Titin OS=Mus musculus GN=Ttn;sp|A2ASS6|TITIN_MOUSE Titin OS=Mus musculus GN=Ttn PE=1 SV=1	1,42	0,00753807348319469	0,0991748276528137	24,7396961746093	24,5984115810658	24,4554828608451	24,9493555858095	25,1330874985912	24,7729139695752	48163318381,3304	68615595442,9107	−0,510601724633209	0,00577722732865502	0,0072178826522752	1,42464426764889	0.51060172463321	0,701929613383638	-0.51060172463321	3920
Q99L48	sp|Q99L48|NMD3_MOUSE 60S ribosomal export protein NMD3 OS=Mus musculus GN=Nmd3 PE=2 SV=1;sp|Q99L48-2|NMD3_MOUSE Isoform 2 of 60S ribosomal export protein NMD3 OS=Mus musculus GN=Nmd3	1,42	0,41204140370883	0,765870569792949	21,9399864331884	23,1722651329508	23,0651904909605	23,3432131075201	23,1493509179357	22,7467657754392	7407908769,13771	10554061512,7797	−0,510660060651351	0,0300384947148548	0,013182517509527	1,42470187494064	0.510660060651356	0,701901231120135	-0.510660060651356	3921
P18653	sp|P18653|KS6A1_MOUSE Ribosomal protein S6 kinase alpha-1 OS=Mus musculus GN=Rps6ka1 PE=1 SV=1	1,42	0,029891063396917	0,244901690900012	25,2553298211587	24,9510957123413	24,8100392117994	25,5454020810795	25,2215409768175	25,3115808292264	72401166525,0797	103155971104,52	−0,510742485680107	0,00910095479180308	0,0065918223377996	1,42478327429693	0.510742485680108	0,701861130769843	-0.510742485680108	3922
P15327	sp|P15327|PMGE_MOUSE Bisphosphoglycerate mutase OS=Mus musculus GN=Bpgm PE=2 SV=2	1,43	0,326687526022154	0,726927682919497	26,275921090209	27,2501225437999	27,3379076999531	27,5509487648623	27,1783471400919	27,1975304598764	508459012595,317	724640945551,384	−0,511134845372956	0,021867595070104	0,0076825849776712	1,42517081534776	0.511134845372957	0,701670276454537	-0.511134845372957	3923
Q3V038	sp|Q3V038|TTC9A_MOUSE Tetratricopeptide repeat protein 9A OS=Mus musculus GN=Ttc9 PE=2 SV=1	1,43	0,0528968686311436	0,339122721351357	26,6147061508255	26,9494249747504	27,1149582581361	27,4305707381541	27,0534472647175	27,2584776827915	478073247224,999	681456708273	−0,511390332758293	0,00947642096403066	0,0069291244781715	1,42542322171038	0.511390332758293	0,701546028413997	-0.511390332758293	3924
Q8VHN7	sp|Q8VHN7|GPR98_MOUSE G-protein coupled receptor 98 OS=Mus musculus GN=Gpr98 PE=2 SV=1;sp|Q8VHN7-5|GPR98_MOUSE Isoform 5 of G-protein coupled receptor 98 OS=Mus musculus GN=Gpr98;sp|Q8VHN7-4|GPR98_MOUSE Isoform 4 of G-protein coupled receptor 98 OS=Mus	1,43	0,664337704768912	0,896648481102019	24,040363892071	24,1861387683333	24,6754463944538	24,3957976508923	23,4277205768759	26,1422103225398	35779812868,4305	51007720986,417	−0,511569800895021	0,0136896139442978	0,0557981560080849	1,42560055229977	0.511569800895023	0,701458763036255	-0.511569800895023	3925
P28076	sp|P28076|PSB9_MOUSE Proteasome subunit beta type-9 OS=Mus musculus GN=Psmb9 PE=1 SV=1;sp|O35524|PSB9_MUSSI Proteasome subunit beta type-9 OS=Mus spicilegus GN=Psmb9 PE=1 SV=1;sp|O35522|PSB9_MUSMB Proteasome subunit beta type-9 OS=Mus musculus bactrianu	1,43	0,0803538913136004	0,422536951051524	21,9012572974974	22,2366110073191	22,6026322326214	22,6264019676859	22,5936782818681	22,5849545096597	4588566544,08391	6543108877,97799	−0,511932747537532	0,015768499957304	0,0009667930915308	1,42595924350582	0.511932747537532	0,701282315433808	-0.511932747537531	3926
Q5SSH8	sp|Q5SSH8|NEUFC_MOUSE Neuferricin OS=Mus musculus GN=Cyb5d2 PE=1 SV=1	1,43	1,19×10^−05	0,00059852716146845	22,7116660906337	22,7467657754392	22,9604916551048	23,1467880451938	23,2058538104204	23,1309610408383	8028855609,71842	11449374830,322	−0,512002551615186	0,00590518534278257	0,0017042251247711	1,42602823949944	0.512002551615187	0,701248385060743	-0.512002551615187	3927
Q9WTR1	sp|Q9WTR1|TRPV2_MOUSE Transient receptor potential cation channel subfamily V member 2 OS=Mus musculus GN=Trpv2 PE=1 SV=2	1,43	0,101854377399634	0,46388602391647	24,0770833167058	24,615571684966	24,132235856752	24,8222977128199	24,3731216083975	24,6947800953991	34872473132,2817	49739562134,9291	−0,51230512464038	0,0122044030864592	0,0093980670380467	1,42632734840033	0.512305124640377	0,701101329313731	-0.512305124640377	3928
Q8BP40	sp|Q8BP40|PPA6_MOUSE Lysophosphatidic acid phosphatase type 6 OS=Mus musculus GN=Acp6 PE=2 SV=1	1,43	0,0888172035310061	0,441176105282424	26,9993754734062	26,3698715068758	26,9302714663707	27,04657919394	27,0716328405969	27,2501225437999	421255826042,062	601551623196,435	−0,513991910934236	0,0128978015281709	0,0040922285891186	1,42799597301325	0.513991910934236	0,700282086853421	-0.513991910934236	3929
Q8BLY2	sp|Q8BLY2|SYTC2_MOUSE Probable threonine--tRNA ligase 2, cytoplasmic OS=Mus musculus GN=Tarsl2 PE=2 SV=1	1,43	0,0367991919259395	0,279698526792889	28,736881630549	28,6120839248889	28,2538132214311	28,8212517944698	29,0701689048778	28,7807657090108	2467581389450,13	3524391110935,65	−0,514276362212857	0,00878807890629031	0,0054243219441438	1,42827755388487	0.514276362212854	0,70014402822479	-0.514276362212854	3930
Q99P30-5	sp|Q99P30-5|NUDT7_MOUSE Isoform 5 of Peroxisomal coenzyme A diphosphatase NUDT7 OS=Mus musculus GN=Nudt7;sp|Q99P30-3| NUDT7_MOUSE Isoform 3 of Peroxisomal coenzyme A diphosphatase NUDT7 OS=Mus musculus GN=Nudt7;sp|Q99P30|NUDT7_MOUSE Peroxisomal coenzyme	1,43	0,254330664694537	0,676674611400062	22,5875559426552	22,6901014055072	23,5394665768213	23,1401154868964	23,3213560979182	23,4260786804776	9168516277,53465	13099633483,859	−0,514766257600016	0,0227779912641957	0,0062104520781922	1,42876263588653	0.514766257600018	0,699906320954081	-0.514766257600018	3931

Q8R555	sp|Q8R555|CRAC1_MOUSE Cartilage acidic protein 1 OS=Mus musculus GN=Crtac1 PE=2 SV=1	1,43	0,520662494428307	0,823916573667872	23,0384874615479	23,8790570595803	22,3912477426894	22,7662261675712	23,9037358086288	23,709903168677	10801282938,3106	15435795747,1142	−0,51507717797838	0,0322900679533292	0,02594010467091	1,42907058682498	0.515077177978384	0,699755497887432	-0.515077177978384	3932
Q9WTS2	sp|Q9WTS2|FUT8_MOUSE Alpha-(1,6)-fucosyltransferase OS=Mus musculus GN=Fut8 PE=2 SV=2	1,43	0,102608849529001	0,46388602391647	23,5278189761452	24,2149220663738	23,7427329093668	24,2210527725757	24,0297670368369	24,305821491108	22314249832,9119	31889642266,5291	−0,51512260727918	0,0147507288215637	0,0058469120573296	1,42911558781126	0.515122607279185	0,699733463499295	-0.515122607279185	3933
Q91WE2	sp|Q91WE2|F192A_MOUSE Protein FAM192A OS=Mus musculus GN=Fam192a PE=2 SV=1	1,43	0,172230205592819	0,586461292712533	24,7137513679622	24,7436143644013	25,4554077650705	25,1359541100947	25,3839467459252	25,4651569064471	69941463898,8985	99991656803,5145	−0,51565973052879	0,0168131707398369	0,0067709993860040	1,42964775441438	0.515659730528794	0,699472997395521	-0.515659730528794	3934
Q9JJY3	sp|Q9JJY3|NSMA2_MOUSE Sphingomyelin phosphodiesterase 3 OS=Mus musculus GN=Smpd3 PE=1 SV=1	1,43	0,00775403274681685	0,100846861426366	24,7764875068518	25,2227384535695	25,0392995070299	25,3552094720957	25,4412526778006	25,3172002676571	72935536220,0303	104371324575,67	−0,51703158206295	0,00896734856795583	0,0025050917448031	1,4310078458984	0.517031582062957	0,69880818813554	-0.517031582062957	3935
Q3UHB8	sp|Q3UHB8|CC177_MOUSE Coiled-coil domain-containing protein 177 OS=Mus musculus GN=Ccdc177 PE=1 SV=1	1,43	0,0181925948627553	0,182690681597447	25,4079277565894	25,1215085600284	25,2111392176563	25,8593137304936	25,5141697482735	25,4494598461444	92166126772,8752	132208726890,215	−0,52050888132354	0,00580319419208893	0,0086043669998645	1,43446113577081	0.520508881323543	0,697125892827099	-0.520508881323543	3936
P0C605-2	sp|P0C605-2|KGP1_MOUSE Isoform Beta of cGMP-dependent protein kinase 1 OS=Mus musculus GN=Prkg1;sp|P0C605|KGP1_MOUSE cGMP-dependent protein kinase 1 OS=Mus musculus GN=Prkg1 PE=1 SV=1	1,43	0,112895773132248	0,481076973512557	25,1651628272617	24,4478937746508	24,8599080669657	25,3615584974045	25,0452755773747	25,149119368486	60404023644,109	86665161724,1553	−0,52080751138227	0,0145001457075506	0,0064012967494512	1,4347580921889	0.520807511382271	0,69698160647721	-0.520807511382271	3937
Q91XF0	sp|Q91XF0|PNPO_MOUSE Pyridoxine-5-phosphate oxidase OS=Mus musculus GN=Pnpo PE=1 SV=1	1,43	0,24845367418465	0,673464050610029	26,8071250785447	27,6075461660519	27,8242150678396	27,8539827882304	27,7767708216757	27,6913415808811	804077990190,484	1153742047804,29	−0,52091335901462	0,0195432372871056	0,0029291819973893	1,43486336136495	0.520913359014625	0,696930472214947	-0.520913359014625	3938
Q8BR92	sp|Q8BR92|PALM2_MOUSE Paralemmin-2 OS=Mus musculus GN=Palm2 PE=1 SV=1	1,44	0,19418419553031	0,621726099655746	25,3493149492024	25,2931187432148	25,6042732888588	25,9701667726243	26,1042219466073	25,2593441260161	109104174679,349	156748892088,087	−0,52274894108997	0,00652437275838335	0,0176145565534737	1,43669014085632	0.522748941089972	0,696044311547903	-0.522748941089972	3939
P49615	sp|P49615|CDK5_MOUSE Cyclin-dependent-like kinase 5 OS=Mus musculus GN=Cdk5 PE=1 SV=1	1,44	0,0321548594956331	0,260162045010123	30,0952385083161	30,4210659683686	30,6656706138036	30,7552693729942	30,6939719257556	30,8202582060154	15846847440718,3	22770793295971,1	−0,52298869310762	0,00941561730776798	0,0020532938247650	1,43692891479865	0.522988693107623	0,695928650124022	-0.522988693107623	3940
Q8BFY6	sp|Q8BFY6|PEF1_MOUSE Peflin OS=Mus musculus GN=Pef1 PE=2 SV=1	1,44	0,128894707095869	0,512962462222669	27,8935880061131	28,3225587196079	28,5757099628488	28,8685229978762	28,4137586698619	28,5978831612972	1883122142722,09	2706619647523,31	−0,52336558573623	0,0121998794002346	0,0079907726547701	1,4373043501102	0.523365585736241	0,69574686803343	-0.523365585736241	3941
Q99J47-2	sp|Q99J47-2|DRS7B_MOUSE Isoform 2 of Dehydrogenase/reductase SDR family member 7B OS=Mus musculus GN=Dhrs7b;sp|Q99J47|DRS7B_MOUSE Dehydrogenase/reductase SDR family member 7B OS=Mus musculus GN=Dhrs7b PE=2 SV=1	1,44	0,406900779709595	0,761303643487985	25,0467789403677	24,6180184843432	25,8525777820242	25,1009475178655	25,5531568912004	25,9516347704544	85557833741,0078	122974935259,29	−0,52339243540645	0,0249003065414427	0,0166681904505005	1,43733109970441	0.523392435406457	0,695733919766747	-0.523392435406457	3942
Q9DAU1	sp|Q9DAU1|CNPY3_MOUSE Protein canopy homolog 3 OS=Mus musculus GN=Cnpy3 PE=1 SV=1	1,44	0,0607417104873076	0,365467698475045	25,7036960429525	26,2312364204502	26,2659948811303	26,3465902358295	26,5664270729435	26,3810756525348	209287717613,479	301297600864,087	−0,52570153806372	0,012087675195161	0,0044731650126662	1,43963345914324	0.525701538063717	0,69462125490965	-0.525701538063717	3943
Q9D1G5	sp|Q9D1G5|LRC57_MOUSE Leucine-rich repeat-containing protein 57 OS=Mus musculus GN=Lrrc57 PE=2 SV=1	1,44	0,0211210268416215	0,202253883616287	28,4643306249264	28,5402325045527	28,9384359919015	29,1075726323578	28,9070949594495	29,0218456153508	2763906998380,9	3979475207236,67	−0,52586911623115	0,00888929708167729	0,0034671096710812	1,43980069140092	0.525869116231155	0,6945405749368	-0.525869116231155	3944
P00397	sp|P00397|COX1_MOUSE Cytochrome c oxidase subunit 1 OS=Mus musculus GN=Mtco1 PE=3 SV=1	1,44	0,257637985002813	0,67968190098123	24,1908952126927	24,3990475359719	24,9321216980073	25,3243138240262	24,758319535241	24,534409034057	43995571389,3617	63375770998,5531	−0,52657308450599	0,0155993312776252	0,0163680070981622	1,44050341880268	0.526573084505993	0,694201754016786	-0.526573084505993	3945
Q60829	sp|Q60829|PPR1B_MOUSE Protein phosphatase 1 regulatory subunit 1B OS=Mus musculus GN=Ppp1r1b PE=2 SV=2;sp|Q60829-2|PPR1B_MOUSE Isoform 2 of Protein phosphatase 1 regulatory subunit 1B OS=Mus musculus GN=Ppp1r1b	1,44	0,0042982233763253	0,0698611208659772	26,4553495397667	26,6649401997547	26,8788895373272	27,097740617644	26,9675427036421	27,0296671524623	381124942841,934	549156970259,862	−0,52695455723878	0,00794159659940587	0,0024090872959203	1,44088436239561	0.526954557238788	0,694018219711543	-0.526954557238788	3946
Q60634-3	sp|Q60634-3|FLOT2_MOUSE Isoform 3 of Flotillin-2 OS=Mus musculus GN=Flot2;sp|Q60634-2|FLOT2_MOUSE Isoform 2 of Flotillin-2 OS=Mus musculus GN=Flot2;sp|Q60634|FLOT2_MOUSE Flotillin-2 OS=Mus musculus GN=Flot2 PE=1 SV=2	1,44	0,0460763741952036	0,313885648890293	28,5793494389656	28,1229950257761	28,0944458379701	28,808297486709	28,6029701816381	28,4835717147051	1886221556587,02	2719891559699,24	−0,52804998761996	0,00962627759429186	0,0057365551602858	1,44197883339892	0.528049987619972	0,693491455518026	-0.528049987619971	3947
Q8R2U6	sp|Q8R2U6|NUDT4_MOUSE Diphosphoinositol polyphosphate phosphohydrolase 2 OS=Mus musculus GN=Nudt4 PE=1 SV=1	1,44	0,121694370781812	0,498080026130429	24,4377243018323	25,10451441028	25,1478806807997	25,3346416265303	25,3086497347007	25,1478806807997	64938497350,132	93733736930,7604	−0,52949439888041	0,0159893109781979	0,0040042204108149	1,44342325054692	0.529494398880411	0,69279748654533	-0.529494398880411	3948
Q9DB77	sp|Q9DB77|QCR2_MOUSE Cytochrome b-c1 complex subunit 2, mitochondrial OS=Mus musculus GN=Uqcrc2 PE=1 SV=1	1,44	0,0221014939177097	0,207699132282576	30,1824637696736	29,8358818720028	29,7341360948367	30,239302803755	30,4506158641339	30,1644509291729	9840168119591	14207482319766,1	−0,52989605067646	0,00785634541319833	0,0049003539475868	1,44382516102343	0.529896050676465	0,692604635931935	-0.529896050676465	3949

Q9QZS3-4	sp|Q9QZS3-4|NUMB_MOUSE Isoform 4 of Protein numb homolog OS=Mus musculus GN=Numb;sp|Q9QZS3-2|NUMB_MOUSE Isoform 2 of Protein numb homolog OS=Mus musculus GN=Numb;sp|Q9QZS3-3|NUMB_MOUSE Isoform 3 of Protein numb homolog OS=Mus musculus GN=Numb;sp|Q9QZS3	1,45	7,07×10^−05	0,00278869643469304	25,810667197616	25,6268016812449	25,5327595104657	25,9585083386998	26,0089907009257	26,1085492621562	138861489721,771	200754666318,145	−0,531786967938444	0,00550936962295309	0,0029335406159253	1,44571880022594	0.531786967938445	0,691697444789208	-0.531786967938445	3950
Q8BL97-3	sp|Q8BL97-3|SRSF7_MOUSE Isoform 3 of Serine/arginine-rich splicing factor 7 OS=Mus musculus GN=Srsf7;sp|Q8BL97-4|SRSF7_MOUSE Isoform 4 of Serine/arginine-rich splicing factor 7 OS=Mus musculus GN=Srsf7;sp|Q8BL97-2|SRSF7_MOUSE Isoform 2 of Serine/arginin	1,45	0,115207031947584	0,485230859818824	26,4583956916079	27,0036061767615	27,2136596408025	27,3724149190948	27,2620275033788	27,1479973377852	477527300819,543	690590767787,572	−0,532247831402282	0,0144961616274587	0,00411630118789337	1,44618070339092	0.532247831402282	0,691476519950281	-0.532247831402282	3951
Q8R071	sp|Q8R071|IP3KA_MOUSE Inositol-trisphosphate 3-kinase A OS=Mus musculus GN=Itpka PE=2 SV=1	1,45	0,000469010633559332	0,0131409831217642	30,620839005307	30,3606214007766	30,4506158641339	30,8064361728851	30,9878465239167	30,7450551800597	17224583571898,9	24913874148775,8	−0,532480276290669	0,00433613782618485	0,00409265293107389	1,44641372865592	0.532480276290668	0,691365119252048	-0.532480276290668	3952
P97384	sp|P97384|ANX11_MOUSE Annexin A11 OS=Mus musculus GN=Anxa11 PE=1 SV=2	1,45	0,02167851767771	0,205425452482517	28,5025208578432	28,9505536434768	28,9188433011032	29,1644059935391	29,2802995314596	29,034879459619	3188715437555,51	4612825359900,26	−0,532675316902477	0,00868411071550851	0,00421034674732274	1,44660928522254	0.532675316902481	0,691271658640131	-0.532675316902481	3953
Q9EPB5	sp|Q9EPB5|SERHL_MOUSE Serine hydrolase-like protein OS=Mus musculus GN=Serhl PE=2 SV=1	1,45	0,970958877854745	0,993055959121831	20,7818141292922	0	21,7584100724818	22,080652069264	0	21,569596405202	1439886,33430268	2084590,15348739	−0,533808837826403	0,86670975747338	0,86620345306388	1,44774632818286	0.533808837826396	0,690728741999399	-0.533808837826396	3954
O55242-2	sp|O55242-2|SGMR1_MOUSE Isoform 2 of Sigma non-opioid intracellular receptor 1 OS=Mus musculus GN=Sigmar1;sp|O55242|SGMR1_MOUSE Sigma non-opioid intracellular receptor 1 OS=Mus musculus GN=Sigmar1 PE=1 SV=1;sp|Q9R0C9-2|SGMR1_RAT Isoform 2 of Sigma non-o	1,45	0,0573825383273633	0,354544580715217	24,2840779865289	24,6453225574227	24,9531426386628	25,0452755773747	24,9476536404833	25,003648975935	49612784734,3559	71922817220,6403	−0,535737595334751	0,0135981092258564	0,00195956512250626	1,44968313320327	0.535737595334751	0,689805914890082	-0.535737595334751	3955
Q8BMQ8	sp|Q8BMQ8|MON1B_MOUSE Vacuolar fusion protein MON1 homolog B OS=Mus musculus GN=Mon1b PE=2 SV=1	1,45	0,325326293401955	0,726927682919497	25,347826740016	25,0272681171071	25,0954650688965	26,2158504682045	25,3954169770848	24,9733867043026	84233059238,5763	122113655426,43	−0,535766070476913	0,0067125387036198	0,0247486155323374	1,44971174655503	0.535766070476915	0,689792300004681	-0.535766070476916	3956
Q8BH44	sp|Q8BH44|COR2B_MOUSE Coronin-2B OS=Mus musculus GN=Coro2b PE=2 SV=2	1,45	2,32×10^−07	1,91×10^−05	29,1733008248421	29,3780373232367	29,3362258860121	29,6285973016842	29,7389386091483	29,6357657449474	5284793586620,99	7665625415476,97	−0,536556377914603	0,00369247095197617	0,00208105845150486	1,45050611529716	0.536556377914602	0,689414535694758	-0.536556377914602	3957
Q3TVP5	sp|Q3TVP5|F105A_MOUSE Inactive ubiquitin thioesterase FAM105A OS=Mus musculus GN=Fam105a PE=2 SV=1;sp|Q3TVP5-2|F105A_MOUSE Isoform 2 of Inactive ubiquitin thioesterase FAM105A OS=Mus musculus GN=Fam105a	1,45	0,137708182587684	0,528144153635909	22,4725237041215	23,0737103227927	23,1166209487376	23,3393648146177	22,9874166300028	23,4522005639565	8708960936,39268	12634040935,5246	−0,536743645134486	0,0157343669759195	0,0104226138648482	1,45069440864408	0.536743645134482	0,689325052913568	-0.536743645134482	3958
Q8CI04	sp|Q8CI04|COG3_MOUSE Conserved oligomeric Golgi complex subunit 3 OS=Mus musculus GN=Cog3 PE=2 SV=3	1,45	0,241154126089785	0,664601444032504	24,3017734131296	23,2058538104204	23,8296632028795	24,1252534040277	24,1461515325522	24,1834776243377	21238827063,7885	30826098447,1473	−0,537448210054164	0,0231172316442971	0,00122127767850039	1,45140305321779	0.537448210054163	0,688988491365633	-0.537448210054163	3959
Q9DC16	sp|Q9DC16|ERGI1_MOUSE Endoplasmic reticulum-Golgi intermediate compartment protein 1 OS=Mus musculus GN=Ergic1 PE=1 SV=1	1,45	0,0783513631426177	0,417369905631425	27,6060239625494	28,0971533682127	28,2936711973808	28,4835717147051	28,3251900002394	28,3076758523563	1444738582527,8	2098294222884,17	−0,538408518208889	0,0126499723628258	0,00341511560646682	1,45236947933713	0.538408518208888	0,688530029188167	-0.538408518208888	3960
P12787	sp|P12787|COX5A_MOUSE Cytochrome c oxidase subunit 5A, mitochondrial OS=Mus musculus GN=Cox5a PE=1 SV=2	1,45	0,0593824455699975	0,361309544828711	27,8242150678396	27,6483028203886	27,3929021595667	27,6908756572043	28,1132288221562	28,1810020312617	990827889467,256	1439094919148,71	−0,538455369090514	0,00785154248351879	0,00948671394237914	1,45241664515769	0.53845536909051	0,688507669843887	-0.53845536909051	3961
Q6PA06	sp|Q6PA06|ATLA2_MOUSE Atlastin-2 OS=Mus musculus GN=Atl2 PE=1 SV=1;sp|Q6PA06-2|ATLA2_MOUSE Isoform 2 of Atlastin-2 OS=Mus musculus GN=Atl2	1,45	0,0996151837631851	0,46324407107743	27,6980271242862	28,2008611250849	28,470179165499	28,5146694615578	28,5161702425418	28,4598031167191	1635586336559,7	2377049052197,99	−0,539363758714983	0,0139358020216238	0,00112710945348393	1,45333144393826	0.539363758714981	0,688074289021221	-0.539363758714981	3962
Q6PDG8	sp|Q6PDG8|MON1A_MOUSE Vacuolar fusion protein MON1 homolog A OS=Mus musculus GN=Mon1a PE=1 SV=3;sp|Q6PDG8-2|MON1A_MOUSE Isoform 2 of Vacuolar fusion protein MON1 homolog A OS=Mus musculus GN=Mon1a	1,45	0,0144782725447159	0,15989508292084	23,775612781693	24,2181219065247	24,2198565302694	24,5156871626391	24,4408004316673	24,3792155998351	28443829161,9054	41345705596,7574	−0,539621794199578	0,0106344929003065	0,00279578995603316	1,45359140506059	0.539621794199577	0,687951233419902	-0.539621794199577	3963
Q8VEK0	sp|Q8VEK0|CC50A_MOUSE Cell cycle control protein 50A OS=Mus musculus GN=Tmem30a PE=1 SV=1	1,45	0,197430387862201	0,626240510020104	26,8991884585141	27,745330412391	27,7229066479733	27,9943201847396	27,7640796168976	27,7318880781206	839278563089,382	1220273292605,2	−0,539982653213874	0,0175619476614348	0,00514300268250455	1,45395503504031	0.53998265321387	0,687779178791646	-0.53998265321387	3964
P10649	sp|P10649|GSTM1_MOUSE Glutathione S-transferase Mu 1 OS=Mus musculus GN=Gstm1 PE=1 SV=2	1,45	0,0198345942036924	0,1954017511157	32,8095502245621	32,9377590356243	33,2755034398491	33,5323458377698	33,3596098324373	33,2547258597017	216282001227085	314569821895809	−0,540466662489319	0,00729228253101717	0,00419939422598616	1,45444290376029	0.540466662489316	0,687548474687192	-0.540466662489316	3965

P69566	sp|P69566|RANB9_MOUSE Ran-binding protein 9 OS=Mus musculus GN=Ranbp9 PE=1 SV=1;sp|P69566-2|RANB9_MOUSE Isoform 2 of Ran-binding protein 9 OS=Mus musculus GN=Ranbp9	1,45	0,0469232058132931	0,315151075529124	27,2761384034076	26,7315571743892	26,6924412252218	27,2571389591788	27,2725327823275	27,2953748650026	481439109903,993	700468711310,835	−0,54096726498093	0,012129815901099	0,00070535180780	1,4549476702267 1	0.540967264980931	0,687309942799648	-0.540967264980931	3966
Q8C015	sp|Q8C015|PAK7_MOUSE Serine/threonine-protein kinase PAK 7 OS=Mus musculus GN=Pak7 PE=1 SV=1	1,46	0,332977831162202	0,72953198881961	26,1684627487745	25,0651396225333	25,0286278335686	25,9984600276786	25,6830722098595	25,7062486810014	109670187306,315	159598475968,427	−0,54127547762360	0,025483174429659	0,00681429899306	1,45525853368573	0.541275477623611	0,687163123838418	-0.541275477623611	3967
Q7M6Z0	sp|Q7M6Z0|R4RL2_MOUSE Reticulon-4 receptor-like 2 OS=Mus musculus GN=Rtn4rl2 PE=2 SV=1	1,46	0,102466197317303	0,46388602391647	22,6114053377269	23,2024655836816	22,5224468316406	23,1840498105516	22,993646883691	23,2844462621002	7810794969,99545	11367766098,6791	−0,54140747923320	0,016226289781153	0,00637914243817	1,4553916909031	0.541407479233198	0,687100253664002	-0.541407479233198	3968
P51655	sp|P51655|GPC4_MOUSE Glypican-4 OS=Mus musculus GN=Gpc4 PE=2 SV=2	1,46	0,219439939355286	0,648792713611515	25,5562998340598	26,2070979591574	26,387321076432	26,6455238891739	26,0840270683502	26,5491925988097	205814193048,044	299759958237,271	−0,54246520716009	0,016781139143722	0,0113621982503636	1,45645911877077	0.542465207160094	0,68659668308712	-0.542465207160094	3969
Q8C419	sp|Q8C419|GP158_MOUSE Probable G-protein coupled receptor 158 OS=Mus musculus GN=Gpr158 PE=1 SV=2	1,46	0,105828081541191	0,469781708234609	24,4925993676552	24,6585297673407	25,0836030464032	25,3572525299008	25,1686290907268	24,8371424988238	55792803424,7165	81267176575,291	−0,54259372790110	0,012318648876994	0,010481459638473	1,45658887144734	0.542593727901106	0,686535521177195	-0.542593727901106	3970
Q9CRD2	sp|Q9CRD2|EMC2_MOUSE ER membrane protein complex subunit 2 OS=Mus musculus GN=Emc2 PE=2 SV=1	1,46	0,297201899419761	0,711969538636193	25,8716928723719	26,8329937693396	26,7075038532864	27,1097993894113	26,9731145224765	26,4583956916079	313394985054,376	456613529314,667	−0,54299151280092	0,01974113048228 8	0,0127945997219345	1,45699054257502	0.54299151280092	0,686346253306931	-0.54299151280092	3971
Q62351	sp|Q62351|TFR1_MOUSE Transferrin receptor protein 1 OS=Mus musculus GN=Tfrc PE=1 SV=1	1,46	0,0463554518660469	0,314182682726068	28,8709870434371	28,6351446174494	28,2288491072506	28,9271249389734	29,0045580088892	28,9338137105631	2578752633886,32	3758967944504,76	−0,54366322285515	0,011365871627036	0,0014817965158752	1,45766906647412	0.543663222855157	0,686026769038082	-0.543663222855157	3972
Q9WUU7	sp|Q9WUU7|CATZ_MOUSE Cathepsin Z OS=Mus musculus GN=Ctsz PE=2 SV=1	1,46	0,107534135243005	0,472047814185685	25,1021464688603	25,698996365124	25,7441964423723	26,0516722330455	25,9437232703096	25,6812465519977	120523801370,467	175729904279,503	−0,54404163633389	0,014044732907674	0,00735777369217	1,4580514577311	0.544041636333885	0,685846850395882	-0.544041636333885	3973
Q9JJZ4	sp|Q9JJZ4|UB2J1_MOUSE Ubiquitin-conjugating enzyme E2 J1 OS=Mus musculus GN=Ube2j1 PE=1 SV=2	1,46	0,171460044564509	0,585155746073914	23,8464232723357	22,9604916551048	23,7113493878119	23,9062094718821	23,8848298274034	23,8591535596167	16164577403,2882	23573702330,2294	−0,54434256552134	0,020305492049847	0,00098648555585	1,4583556218076	0.544342565521342	0,685703805742884	-0.544342565521342	3974
Q8C078	sp|Q8C078|KKCC2_MOUSE Calcium/calmodulin-dependent protein kinase kinase 2 OS=Mus musculus GN=Camkk2 PE=1 SV=2;sp|Q8C078-5|KKCC2_MOUSE Isoform 5 of Calcium/calmodulin-dependent protein kinase kinase 2 OS=Mus musculus GN=Camkk2	1,46	0,0171662041641065	0,178300688102698	28,3908102287772	28,7125758401729	28,9014392327936	29,0984836692739	28,9384359919015	29,1006990223782	2821458598249,39	4115876359758,77	−0,54475846476299	0,00900580749364239	0,00320354222131	1,45877609627606	0.544758464762993	0,685506159960246	-0.544758464762993	3975
O35425	sp|O35425|BOK_MOUSE Bcl-2-related ovarian killer protein OS=Mus musculus GN=Bok PE=1 SV=1	1,46	0,0740188132348288	0,404916706615332	25,2380141227444	24,9952475903761	24,6363202435582	25,1715068365119	25,2652188489382	25,5677487880404	68941718065,3428	100640925348,626	−0,54576793531544	0,012129490839203	0,00817406653051	1,45979717611967	0.545767935315448	0,685026671073668	-0.545767935315448	3976
Q62813	sp|Q62813|LSAMP_RAT Limbic system-associated membrane protein OS=Rattus norvegicus GN=Lsamp PE=1 SV=1;sp|Q8BLK3|LSAMP_MOUSE Limbic system-associated membrane protein OS=Mus musculus GN=Lsamp PE=1 SV=1;sp|Q62813-2|LSAMP_RAT Isoform 2 of Limbic system-ass	1,46	0,0056549868806747	0,0827999530689757	28,9338137105631	29,2199061542331	29,3699426896834	29,6401923534164	29,472404298724	29,5469002992119	4681099737163,68	6835601946311,77	−0,54622088387983	0,0075947446436313	0,00284468034523	1,46025556602507	0.546220883879837	0,684811633844394	-0.546220883879837	3977
P97429	sp|P97429|ANXA4_MOUSE Annexin A4 OS=Mus musculus GN=Anxa4 PE=2 SV=4	1,46	0,0177019774337523	0,180065108755229	25,528533155215	25,8984378098441	25,9259085166726	26,3143352941035	25,9864544591337	26,1892833237637	157752495973,292	230463351788,832	−0,54687452014186	0,0086067952717133	0,00632452215425	1,46091730826148	0.546874520141866	0,684501439160864	-0.546874520141866	3978
O89116	sp|O89116|VTI1A_MOUSE Vesicle transport through interaction with t-SNAREs homolog 1A OS=Mus musculus GN=Vti1a PE=1 SV=1	1,46	0,00149326071181996	0,0321227979665915	25,1824661258207	25,530459038085	25,3937401987218	25,8761040310522	25,6812465519977	25,6889101757867	104128177607,761	152244495157,061	−0,54802954224359	0,0069109249718448	0,00428584883208	1,46208738743656	0.548029542243594	0,683953646405005	-0.548029542243594	3979
Q9JHG6	sp|Q9JHG6|RCAN1_MOUSE Calcipressin-1 OS=Mus musculus GN=Rcan1 PE=1 SV=1;sp|Q9JHG6-2|RCAN1_MOUSE Isoform B of Calcipressin-1 OS=Mus musculus GN=Rcan1	1,46	0,111275659874654	0,4778431600483	26,2550580787104	26,6533800549077	27,0804277839634	27,0475341194994	27,0605273992974	27,0207057287637	379816966248,479	553822229725,008	−0,54817666528785	0,015480960476446 3	0,00075093372450	1,4622364956748	0.548176665287859	0,683883901788758	-0.548176665287859	3980
Q80TJ1-4	sp|Q80TJ1-4|CAPS1_MOUSE Isoform 4 of Calcium-dependent secretion activator 1 OS=Mus musculus GN=Cadps;sp|Q80TJ1|CAPS1_MOUSE Calcium-dependent secretion activator 1 OS=Mus musculus GN=Cadps PE=1 SV=3;sp|Q80TJ1-2|CAPS1_MOUSE Isoform 2 of Calcium-dependent	1,46	0,00033724679276253	0,0100708104759812	25,7136461877525	25,4794674273275	25,4651569064471	25,8007350073515	25,9650637893661	26,0333327348677	125147244938,783	183053417090,149	−0,54863817385152	0,0054599926176703	0,00461030034248	1,46270433024468	0.548638173851518	0,683665166857559	-0.548638173851518	3981

Q3V3V9-2	sp|Q3V3V9-2|LR16C_MOUSE Isoform 2 of Leucine-rich repeat-containing protein 16C OS=Mus musculus GN=Rltpr;sp|Q3V3V9|LR16C_MOUSE Leucine-rich repeat-containing protein 16C OS=Mus musculus GN=Rltpr PE=2 SV=2	1,46	0,017577684087191	0,179292377689348	27,2250623653521	27,7767708216757	27,4982193752751	27,913295506351	27,8740286712693	27,8539827882304	877214620657,667	1283273890355,14	−0,54882735613036	0,0100312162836816	0,0010821551903055	1,46289614894134	0.548827356130365	0,683575522926678	-0.548827356130365	3982
Q80UW8	sp|Q80UW8|RPAB1_MOUSE DNA-directed RNA polymerases I, II, and III subunit RPABC1 OS=Mus musculus GN=Polr2e PE=2 SV=1	1,46	0,122177165652397	0,498706973827545	25,2157838114411	26,0548622267635	25,7605581739283	26,0312420351845	26,0422096112122	26,102075650852	141712765912,535	207523225211,33	−0,550303080023813	0,016579938144842	0,0014630988796182	1,4643933020079	0.550303080023819	0,68287665521882	-0.550303080023819	3983
P54729	sp|P54729|NUB1_MOUSE NEDD8 ultimate buster 1 OS=Mus musculus GN=Nub1 PE=1 SV=2	1,46	0,35991109563154	0,733888797426577	25,2961103046975	25,4252755505049	25,048560475253	25,5847097988631	26,35933783101	24,9705054088962	93072889093,4816	136308201500,552	−0,550439473950914	0,0075794964752245	0,0271453975047431	1,46453175385633	0.550439473950917	0,682812098383564	-0.550439473950917	3984
Q5PR73	sp|Q5PR73|DIRA2_MOUSE GTP-binding protein Di-Ras2 OS=Mus musculus GN=Diras2 PE=2 SV=1	1,46	0,0571234920295862	0,353736424574922	30,0196926870257	30,507269860691	30,6034495341565	30,8944000450301	30,7847908092274	30,5960927494958	15576804416697	22814738487067,2	−0,550566822357117	0,0103033896484008	0,0049056766530318	1,46466103552098	0.55056682235712	0,682751828408066	-0.55056682235712	3985
P12658	sp|P12658|CALB1_MOUSE Calbindin OS=Mus musculus GN=Calb1 PE=1 SV=2	1,47	0,101731144443148	0,46388602391647	31,4444699733367	31,7608767264179	32,2397875300273	32,2182618919671	32,1255304366215	32,2512806916565	65629390880720,5	96287312221013,8	−0,553003663442355	0,0125857500709488	0,0020247991617404	1,46713706967071	0.553003663442356	0,68159957285003	-0.553003663442356	3986
Q6QD59	sp|Q6QD59|SEC20_MOUSE Vesicle transport protein SEC20 OS=Mus musculus GN=Bnip1 PE=2 SV=1	1,47	0,11959710871771	0,494141117083746	24,7074766283013	25,2300086282913	25,4860362621515	25,6624905516586	25,5584985256053	25,3532998395674	82922629855,0873	121692471303,424	−0,553402139479113	0,0157832382371701	0,0061638880899702	1,46754235262545	0.553402139479114	0,68141133931228	-0.553402139479114	3987
P97952	sp|P97952|SCN1B_MOUSE Sodium channel subunit beta-1 OS=Mus musculus GN=Scn1b PE=2 SV=1	1,47	0,338885267620122	0,729620681454069	26,0460311816041	24,8516604772475	26,0460311816041	26,1207629911894	26,0234593655428	25,9504924356993	137640026923,696	202007688898,499	−0,553510127072896	0,0268860235331606	0,0032815788728315	1,46765220418394	0.5535101270729	0,681360336699138	-0.5535101270729	3988
Q8VCM8	sp|Q8VCM8|NCLN_MOUSE Nicalin OS=Mus musculus GN=Ncln PE=2 SV=2	1,47	0,0720185158043773	0,400112660019668	24,8903374302592	25,4471000951404	25,5584985256053	25,6255477551962	25,7876482760269	25,6338439500692	97064869565,232	142462787247,785	−0,553563977257776	0,0141495707172694	0,0035545103415591	1,46770698694489	0.553563977257772	0,681334904647116	-0.553563977257772	3989
P19783	sp|P19783|COX41_MOUSE Cytochrome c oxidase subunit 4 isoform 1, mitochondrial OS=Mus musculus GN=Cox4i1 PE=1 SV=2	1,47	0,0899793456315197	0,441176105282424	28,9855437086712	28,6927585562291	28,5486023400049	28,7807657090108	29,1775307111958	29,4203370103753	3038246133133,86	4460184030384,37	−0,553864488417484	0,00774621095099846	0,0110848373401783	1,46801273989735	0.553864488417486	0,681192998413572	-0.553864488417486	3990
Q8CHY3	sp|Q8CHY3|DYM_MOUSE Dymeclin OS=Mus musculus GN=Dym PE=2 SV=1	1,47	0,0288247744121751	0,238644139389349	25,5614805926799	26,0995196682378	26,0234593655428	26,1926661523628	26,2733618497883	26,3722038170175	176188428451,744	258823087753,857	−0,554847140245168	0,0112445083761392	0,0034217488395121	1,4690129767787	0.554847140245169	0,680729180618155	-0.554847140245169	3991
Q9D1H7	sp|Q9D1H7|GET4_MOUSE Golgi to ER traffic protein 4 homolog OS=Mus musculus GN=Get4 PE=2 SV=2;sp|Q9D1H7-2|GET4_MOUSE Isoform 2 of Golgi to ER traffic protein 4 homolog OS=Mus musculus GN=Get4	1,47	0,0322656844246858	0,260594202141724	25,9516347704544	25,9923002404933	26,3577801409492	26,2614070104605	26,6940235846991	26,501323798867	216438378346,266	318085101278,889	−0,55545646221755	0,00856973945406857	0,0081832178950455	1,46963354516547	0.555456462217551	0,68044173548543	-0.555456462217551	3992
Q91WG2	sp|Q91WG2|RABE2_MOUSE Rab GTPase-binding effector protein 2 OS=Mus musculus GN=Rabep2 PE=2 SV=3;sp|Q91WG2-2|RABE2_MOUSE Isoform 3 of Rab GTPase-binding effector protein 2 OS=Mus musculus GN=Rabep2;sp|Q91WG2-1|RABE2_MOUSE Isoform 2 of Rab GTPase-binding	1,47	0,0415210316480834	0,299624741892926	23,9894755268835	24,6092541358523	24,2371377474287	24,567454870373	24,6585297673407	24,7655337624833	35000300007,9512	51448105003,0747	−0,555750650772738	0,0128493669940731	0,0040198987471095	1,46993325746885	0.555750650772738	0,680302996696564	-0.555750650772738	3993
P63328	sp|P63328|PP2BA_MOUSE Serine/threonine-protein phosphatase 2B catalytic subunit alpha isoform OS=Mus musculus GN=Ppp3ca PE=1 SV=1;sp|P63328-2|PP2BA_MOUSE Isoform 2 of Serine/threonine-protein phosphatase 2B catalytic subunit alpha isoform OS=Mus musculus	1,47	0,133044363617336	0,518639007654437	33,3079535850214	34,1068660507989	34,0257535658343	34,2755682575707	34,1391253660482	34,1841736380791	484202537272279	712372753516363	−0,557021698771927	0,0130040124330266	0,0020327991440627	1,47122887362273	0.557021698771926	0,679703897829042	-0.557021698771926	3994
Q9JJA2	sp|Q9JJA2|COG8_MOUSE Conserved oligomeric Golgi complex subunit 8 OS=Mus musculus GN=Cog8 PE=2 SV=3	1,47	0,421585610971409	0,770981904995658	24,9865539749528	24,946752196482	23,7647352552483	24,7376841280185	25,4794674273275	24,6395384667734	46653500964,5001	68646089684,6754	−0,557192194256305	0,028259077552765	0,0184043854971498	1,47140275146575	0.557192194256305	0,679623576212456	-0.557192194256305	3995
Q8BYN5-2	sp|Q8BYN5-2|FSD1L_MOUSE Isoform 2 of FSD1-like protein OS=Mus musculus GN=Fsd1l;sp|Q8BYN5|FSD1L_MOUSE FSD1-like protein OS=Mus musculus GN=Fsd1l PE=2 SV=2	1,47	0,00326165150478358	0,0556565269932807	26,6526229894318	26,8756565229089	26,9761562920847	27,3974954083987	27,1665322140943	27,1000546991755	451035521418,856	663875702513,253	−0,557672093203639	0,0061706721782078	0,0057350745623293	1,47189228117743	0.557672093203638	0,679397543412657	-0.557672093203638	3996
Q8BM65-2	sp|Q8BM65-2|NYAP2_MOUSE Isoform 2 of Neuronal tyrosine-phosphorylated phosphoinositide-3-kinase adapter 2 OS=Mus musculus GN=Nyap2;sp|Q8BM65|NYAP2_MOUSE Neuronal tyrosine-phosphorylated phosphoinositide-3-kinase adapter 2 OS=Mus musculus GN=Nyap2 PE=1 SV	1,47	0,0556571414053766	0,347972127877416	25,5441223434088	25,4540678038503	24,9641191392659	25,7790605133414	25,8188439996991	25,5256101773492	99236305006,8223	146141070745,456	−0,558421759202978	0,0123271652489487	0,0061873274904785	1,47265731765617	0.558421759202973	0,679044600539906	-0.558421759202973	3997
Q9R257	sp|Q9R257|HEBP1_MOUSE Heme-binding protein 1 OS=Mus musculus GN=Hebp1 PE=1 SV=2	1,47	0,096093604687816	0,453869793629549	27,1537780055346	27,5359993615751	27,8119809890143	28,1463259904393	27,8140234660062	27,7031011328236	877713544914,442	1292781045592,06	−0,55865587459923	0,0120189221849884	0,0082703836336433	1,4728963146149	0.558655874599231	0,678934416548838	-0.558655874599231	3998
Q7TQ95	sp|Q7TQ95|LNP_MOUSE Protein lunapark OS=Mus musculus GN=Lnp PE=1 SV=1	1,47	0,0824640612448838	0,427288098162703	28,2591635841223	28,9363589553108	28,7457012936542	29,0836947794638	28,8498970986966	29,1702498422972	2762271904976,28	4069795656275,17	−0,559101020186032	0,0121898847635421	0,0057076754519444	1,47335084896724	0.559101020186031	0,678724962693684	-0.559101020186031	3999
Q8BGN3	sp|Q8BGN3|ENPP6_MOUSE Ectonucleotide pyrophosphatase/phosphodiesterase family member 6 OS=Mus musculus GN=Enpp6 PE=2 SV=1	1,47	1,46×10^−06	0,00010142846166440	25,663480608275	25,5700819737257	25,4119626398757	25,8804365348968	25,9331697089281	25,9945560886311	124616693031,556	183605286964,045	−0,559110264595555	0,00497638599196637	0,0022021248284567	1,47336028984136	0.559110264595561	0,678720613616967	-0.559110264595561	4000

Q61481	sp|Q61481|PDE1A_MOUSE Calcium/calmodulin-dependent 3,5-cyclic nucleotide phosphodiesterase 1A OS=Mus musculus GN=Pde1a PE=2 SV=2;sp|Q61481-2|PDE1A_MOUSE Isoform PDE1A1 of Calcium/calmodulin-dependent 3,5-cyclic nucleotide phosphodiesterase 1A OS=Mus	1,47	0,0393384708974831	0,289869612893965	29,5767627429087	30,1150222657986	30,1189132769238	30,4090370110476	30,3453333642794	30,2207870528903	10032986482264,1	14791184481227,3	−0,559986476775645	0,010418366376243	0,0031574441852250	1,47425539816829	0.55998647677564	0,678308521876512	-0.559986476775639	4001
Q9QZD8	sp|Q9QZD8|DIC_MOUSE Mitochondrial dicarboxylate carrier OS=Mus musculus GN=Slc25a10 PE=1 SV=2	1,47	0,25350322287133 9	0,674868697133729	21,4030055681932	21,6429764340917	22,502151721827	22,3355549008786	22,2687401280658	22,1096485360386	3083644174,391	4548126324,69639	−0,560636025348083	0,0264497935883664	0,0052186273647248	1,47491930569279	0.560636025348083	0,678003193896961	-0.560636025348083	4002
Q8C0P5	sp|Q8C0P5|COR2A_MOUSE Coronin-2A OS=Mus musculus GN=Coro2a PE=2 SV=1	1,47	0,00644227832694847	0,0899738502339049	26,6924412252218	26,9970599843299	27,1813739338955	27,3497540213979	27,3351344193571	27,3519448291009	509633858920,601	751705972146,512	−0,560707335617988	0,0091598183576871 1	0,00033420165556 64	1,47499221056195	0.560707335617987	0,677969682035824	-0.560707335617987	4003
Q03717	sp|Q03717|KCNB1_MOUSE Potassium voltage-gated channel subfamily B member 1 OS=Mus musculus GN=Kcnb1 PE=1 SV=2	1,48	0,0133149657290392	0,1498790297252 9	23,9801195100244	24,2663462043832	24,143043453479	24,7872986927083	24,3700164161251	24,4023850233445	30161628961,7442	44550982480,7187	−0,562742900358692	0,0059498816052356	0,0094673664937823	1,47707481373852	0.562742900358693	0,677013777974434	-0.562742900358693	4004
Q8K1Z0	sp|Q8K1Z0|COQ9_MOUSE Ubiquinone biosynthesis protein COQ9, mitochondrial OS=Mus musculus GN=Coq9 PE=1 SV=1	1,48	0,0647036815613592	0,38006509013234	28,0992599920198	28,6768520225877	28,6988938908549	29,031165838064 9	28,8465312886039	28,7679720830482	2364691381209,46	3493376058524,95	−0,562970047866239	0,0119338223530735	0,0046781065299439	1,47730739253518	0.562970047866237	0,676907192811052	-0.562970047866236	4005
O35526	sp|O35526|STX1A_MOUSE Syntaxin-1A OS=Mus musculus GN=Stx1a PE=1 SV=3	1,48	0,0393259772498501	0,289869612893965	31,1726430469682	31,7308789356197	31,7285609258969	32,0093513947963	31,8850101738384	31,9095496846577	50049199930709,4	73966772234323,4	−0,563530313942384	0,0101962431933382	0,0020622610631032	1,4778812116223	0.563530313942383	0,676644369070963	-0.563530313942383	4006
Q9CZ00-2	sp|Q9CZ00-2|DBND1_MOUSE Isoform 2 of Dysbindin domain-containing protein 1 OS=Mus musculus GN=Dbndd1;sp|Q9CZ00|DBND1_MOUSE Dysbindin domain-containing protein 1 OS=Mus musculus GN=Dbndd1 PE=2 SV=1	1,48	0,258041160515824	0,67968190098123	22,7620598330115	23,4721841914717	23,5193424313764	24,1261151702096	23,4577010632101	23,3432131075201	12527542850,2415	18524186872,5677	−0,564306743690547	0,0182467880988575	0,0178853482562116	1,47867679193056	0.564306743690543	0,67628031051627	-0.564306743690543	4007
P58158	sp|P58158|B3GA3_MOUSE Galactosylgalactosylxylosylprotein 3-beta-glucuronosyltransferase 3 OS=Mus musculus GN=B3gat3 PE=2 SV=1	1,48	0,675436238890321	0,901266728168293	24,9780625663119	21,8876980909906	24,2411722072097	23,9621918362882	24,1861387683333	24,13223585675 2	19669026073,7005	29085981329,3891	−0,564398456670818	0,0680994713387423	0,0048513069956601	1,47877079528	0.564398456670814	0,676237320341896	-0.564398456670814	4008
Q9QYH6	sp|Q9QYH6|MAGD1_MOUSE Melanoma-associated antigen D1 OS=Mus musculus GN=Maged1 PE=1 SV=1	1,48	4,73×10^−06	0,00028596752760378	24,8532287371371	24,9079666938932	24,8141487793	25,1746957465022	25,1630860770961	25,4119626398757	62500979784,0146	92448245905,329	−0,564767140409685	0,0018957831850787 7	0,0055626932257617	1,47914874654451	0.564767140409684	0,676064528558155	-0.564767140409684	4009
O88531	sp|O88531|PPT1_MOUSE Palmitoyl-protein thioesterase 1 OS=Mus musculus GN=Ppt1 PE=2 SV=2	1,48	0,0736092324240467	0,404916706615332	28,2731322206399	27,6913415808811	28,3845625088349	28,5524438127231	28,5471743747981	28,4245313453588	162470184810 2,48	2403746900231,55	−0,565110006206149	0,0132398839117186	0,0025388307315587	1,47950031757453	0.565110006206153	0,675903876546226	-0.565110006206153	4010
Q8CJ61	sp|Q8CJ61|CKLF4_MOUSE CKLF-like MARVEL transmembrane domain-containing protein 4 OS=Mus musculus GN=Cmtm4 PE=2 SV=1	1,48	0,235081019009525	0,658255857670718	26,3614633838226	25,3098334236589	25,9119492395323	26,0039735970023	26,4201728012214	26,3346641672483	170343362376,481	252060973911,705	−0,56532703367483	0,0204035192521067	0,0083726496011038	1,47972289847501	0.565327033674832	0,675802206636522	-0.565327033674832	4011
Q9WUR9	sp|Q9WUR9|KAD4_MOUSE Adenylate kinase 4, mitochondrial OS=Mus musculus GN=Ak4 PE=1 SV=1	1,48	0,053984802306009	0,34156181191547	29,5830171708225	29,9490951305956	30,153305359808	30,5053526695314	30,108137787763	30,2476801384455	9622634699626,94	14239727211693,1	−0,565417642644587	0,0096653630423742 3	0,0066534625096509	1,47981583591084	0.565417642644585	0,675759763973934	-0.565417642644585	4012
Q99NF2-4	sp|Q99NF2-4|NSMF_MOUSE Isoform 4 of NMDA receptor synaptonuclear signaling and neuronal migration factor OS=Mus musculus GN=Nsmf;sp|Q99NF2-3|NSMF_MOUSE Isoform 3 of NMDA receptor synaptonuclear signaling and neuronal migration factor OS=Mus musculus GN=N	1,48	0,17497711120895	0,590462219359101	24,3832569119608	23,9578712584091	24,9079666938932	24,9939508693433	24,7207953569625	24,7137513679622	40169143160,4143	59515296357,0563	−0,567172823263011	0,0194915263076489	0,0064402209052656	1,4816172732234 7	0.567172823263014	0,674938135558015	-0.567172823263014	4013
[truncated: 241,623 more chars]
